# Transcriptome Analysis of Zebrafish Embryogenesis Using Microarrays

**DOI:** 10.1371/journal.pgen.0010029

**Published:** 2005-08-26

**Authors:** Sinnakaruppan Mathavan, Serene G. P Lee, Alicia Mak, Lance D Miller, Karuturi Radha Krishna Murthy, Kunde R Govindarajan, Yan Tong, Yi Lian Wu, Siew Hong Lam, Henry Yang, Yijun Ruan, Vladimir Korzh, Zhiyuan Gong, Edison T Liu, Thomas Lufkin

**Affiliations:** 1 Genome Institute of Singapore, Singapore; 2 Department of Biological Sciences, National University of Singapore, Singapore; 3 Institute of Molecular and Cell Biology, Singapore; 4 Bioinformatics Institute, Singapore; University of Pennsylvania School of Medicine, United States of America

## Abstract

Zebrafish *(Danio rerio)* is a well-recognized model for the study of vertebrate developmental genetics, yet at the same time little is known about the transcriptional events that underlie zebrafish embryogenesis. Here we have employed microarray analysis to study the temporal activity of developmentally regulated genes during zebrafish embryogenesis. Transcriptome analysis at 12 different embryonic time points covering five different developmental stages (maternal, blastula, gastrula, segmentation, and pharyngula) revealed a highly dynamic transcriptional profile. Hierarchical clustering, stage-specific clustering, and algorithms to detect onset and peak of gene expression revealed clearly demarcated transcript clusters with maximum gene activity at distinct developmental stages as well as co-regulated expression of gene groups involved in dedicated functions such as organogenesis. Our study also revealed a previously unidentified cohort of genes that are transcribed prior to the mid-blastula transition, a time point earlier than when the zygotic genome was traditionally thought to become active. Here we provide, for the first time to our knowledge, a comprehensive list of developmentally regulated zebrafish genes and their expression profiles during embryogenesis, including novel information on the temporal expression of several thousand previously uncharacterized genes. The expression data generated from this study are accessible to all interested scientists from our institute resource database (http://giscompute.gis.a-star.edu.sg/~govind/zebrafish/data_download.html).

## Synopsis

In an effort to shed light on the mysterious molecular process of embryogenesis, scientists have employed the powerful technological approach of high-density microarrays to analyze for the first time (to our knowledge) the expression patterns of over 16,000 zebrafish genes during the major stages of embryonic development. They determined that developmentally regulated genes in zebrafish could be grouped based upon their time of transcript accumulation onset (activation) and their peak of activity (peak accumulation). For example, genes encoding transcriptional regulators are activated en masse earlier than the target genes that they control. Thus, the developmental gene expression profile in zebrafish can be grouped into successive waves of gene activation followed by peak activity. One very novel group of genes that was discovered in this study is a set of about 100 genes that are activated even earlier than what was previously thought to be the earliest time of embryonic transcriptional activation. These genes are likely to be important for preparing the embryonic cells for subsequent high-level genome activation and normal development.

## Introduction

Zebrafish is an important vertebrate model for the analysis of developmentally regulated genes. Its advantages are rapidly developing transparent embryos, a short generation time, and amenability to genetic manipulation. In recent years, large-scale mutagenesis has been undertaken by both chemical mutagens [1–3] and proviral insertions [4–6]. Genetic tools such as transgenesis and morpholino knock-down assays have also been established for zebrafish [7,8]. In effect, a unique combination of these features makes zebrafish a strong model to study vertebrate developmental disorders and human hereditary disease [1,9–11]. It is believed that the zebrafish genome may contain about 30% more genes than the human genome, owing to an additional round of genome duplication about 450 million years ago, followed by extensive gene loss [12]. Several expressed sequence tag (EST) projects were launched to characterize genes expressed during different developmental stages [13–15] and in the various tissues of zebrafish [16]. The zebrafish database contains more than 400,000 ESTs grouped into about 18,000 clusters, but only a few thousand ESTs have been analyzed by whole embryo in-situ hybridization (WISH) (http://zfin.org). Systematic analysis of the temporal expression of the ESTs by WISH may take several more years to complete. A high-throughput expression genomics approach would provide complementary information to the single-gene approaches currently under way. Microarrays are currently the strongest technology platform for large-scale analysis of gene expression profiles during embryogenesis. They provide an opportunity to simultaneously monitor the expression of thousands of genes at various developmental stages, thus providing an opportunity to analyze temporal and spatial patterns of gene expression [17–23]. Preliminary analysis of the expression of a subset of zebrafish genes has been undertaken using cDNA microarrays [14,24,25]. Global evaluation of gene expression during the life cycle and organogenesis of *Drosophila* and mouse has been reported [20,23,26–29]. In contrast, a high-density microarray for genome-wide gene expression (transcriptome) analysis has not previously been attempted for zebrafish. In the present study, we used high-density oligonucleotide microarrays containing 16,416 genes to map the transcriptome profiles at 12 different developmental time points (from unfertilized eggs to the hatching stage). RNA was collected at closely spaced time points during blastula and gastrula stages—the dynamic development stages—and thus provides a dense coverage and a comprehensive analysis of the expression patterns of developmentally regulated genes during embryogenesis.

Traditionally, it is believed that the zygotic genome becomes active at the mid-blastula transition (MBT) [30]. However, recent evidence in *Xenopus* has shown the commencement of zygotic genome transcription to occur prior to MBT [31]. We speculated that there might be a subset of genes activated with regards to transcript accumulation prior to MBT that in turn could play a subsequent role in genome-wide transcriptional activation but had escaped detection owing to their low numbers relative to the entire genome. To test our speculation, we made a systematic analysis of gene expression profiles preceding MBT, which revealed for the first time to our knowledge a striking cohort of genes that actively increase transcript levels prior to MBT. These new data form the starting point for determining the role of these genes in the control of zebrafish genome activation and embryogenesis. To make the data we have generated here easily available to the public, we have created a GIS-sponsored Web site (http://giscompute.gis.a-star.edu.sg/~govind/zebrafish/index.html) that includes annotation data and all expression data for all the genes in the zebrafish microarray.

## Results/Discussion

### Validation of Zebrafish Microarray Data

The microarray data obtained in this work were validated using the following criteria: Firstly, 172 copies of the beta-actin gene were printed on the oligonucleotide array as calibration spots. Biological and experimental replicates of array hybridizations showed that, at the selected time points, the signals of all the beta-actin calibration spots were identical (Figure S1), indicating the reproducibility and reliability of the array data. Secondly, candidate genes with known expression patterns were compared with the array-derived expression patterns, and the patterns were found to be similar to one another (Table S1). In addition, real-time PCR analysis was performed for two genes *(beta-actin* and *myosin light chain, mlc2f)* at all selected time points. The quantity of transcripts detected could not be compared because of different methods of estimations. However, the pattern of transcript abundance detected for these genes in the array and in real-time PCR showed nearly identical expression profiles (Figure S2). Further, expression data of ribosomal protein (RP) genes of zebrafish obtained from the arrays showed temporally coordinated expression (see results section of RP gene expression). In order to confirm the array data, a Northern blot analysis was performed for selected genes *(rpl7a-, rps3a-, rps12-,* and *rps10-)* using the RNA samples collected at 14 different time points during embryogenesis. The pattern of expression of RP genes obtained in the array and Northern blot were essentially identical, validating the array data (Figure S3A). Intensity of hybridization of RP genes in the Northern blot were digitized and measured. Using the digitized data, the pattern of transcript accumulation was compared with array data, and the pattern of expression was similar in both the experiments (Figure S3B). These analyses demonstrated the validity and reliability of the array data presented in this work.

### Annotation of the Zebrafish Oligoarray

The annotation given by Compugen (oligonucleotide supplier) is based on Entrez Nucleotide, BLAST, and Gene Ontology (GO) (http://www.labonweb.com/oligo) and is outdated. For example, the company database gives a description based on GO terms for about 1,152 probes, and the remaining are indicated as “GO unknown.” From our recent annotation, we have found GO terms for more than twice those reported by the company. We made critical analysis of the clones and identified the duplicates to determine the actual number of non-redundant clones in the array. The array represented 16,416 GenBank entries. Of this, 16,177 probes had non-redundant GenBank entries and the rest of the 239 GenBank entries were duplicated (including 171 copies of beta-actin) in the array. We mapped the non-redundant GenBank entries to the zebrafish UniGene database (UniGene build 79) and found that 2,751 GenBank entries did not have UniGene IDs and the rest of the 13,426 entries matched to UniGenes. Of these clones with UniGene entries, 12,153 clones were non-redundant, while the rest (1,273 UniGenes) were duplicates. On the whole, the probes in the array represented 14,904 non-redundant genes (12,153 non-redundant UniGene IDs plus 2,751 non-redundant GenBank entries). We have included here the most recent putative annotations for the clones in the array by obtaining the annotations from various database sources. The annotations can be obtained from our institute resource Web site (http://giscompute.gis.a-star.edu.sg/~govind/zebrafish/index.html). We have also included examples of annotations in the Datasets. The resources used to build the zebrafish annotation database, strategies followed, and the query methods for annotation retrieval are described in the Materials and Methods section.

### Transcriptome Analysis Identified Developmentally Regulated Genes

The gene expression pattern of the zebrafish genome during embryogenesis was investigated using high-density oligonucleotide arrays representing 16,416 zebrafish genes. High-resolution time-course expression data were generated during embryogenesis. Considering the sensitivity of the array data and the number of replicates of each sample, it is very likely that most of the polyadenylated transcripts were detected for the genes represented in the array for each time point. Total RNA was extracted at 12 different developmental time points (see Materials and Methods for details), and for each time point analysis two to three independent biological replicates were made. Reference RNA was prepared by pooling equal concentration of total RNA from various embryonic stages and also adult male and female fish. The intensity of value of each spot and each side was normalized using the intensity-based log ratio median method [32]. Differentially expressed genes were selected using modified t-statistic (SAM) [33] with 15% of the standard deviation percentile as the fudge constant and log2 ratios. Stage-specific genes were selected following paired *t*-test (see Materials and Methods for details). By this procedure, we have identified 3,657 genes that showed significant levels of differential expression with a single peak during the course of development. This subset of developmentally regulated genes was used for further analysis of gene activity during embryogenesis. For the benefit of other scientists, the entire dataset generated from this study has been placed in our institute resource database, which can be accessed at http://giscompute.gis.a-star.edu.sg/~govind/zebrafish/data_download.html.

**Figure 1:**
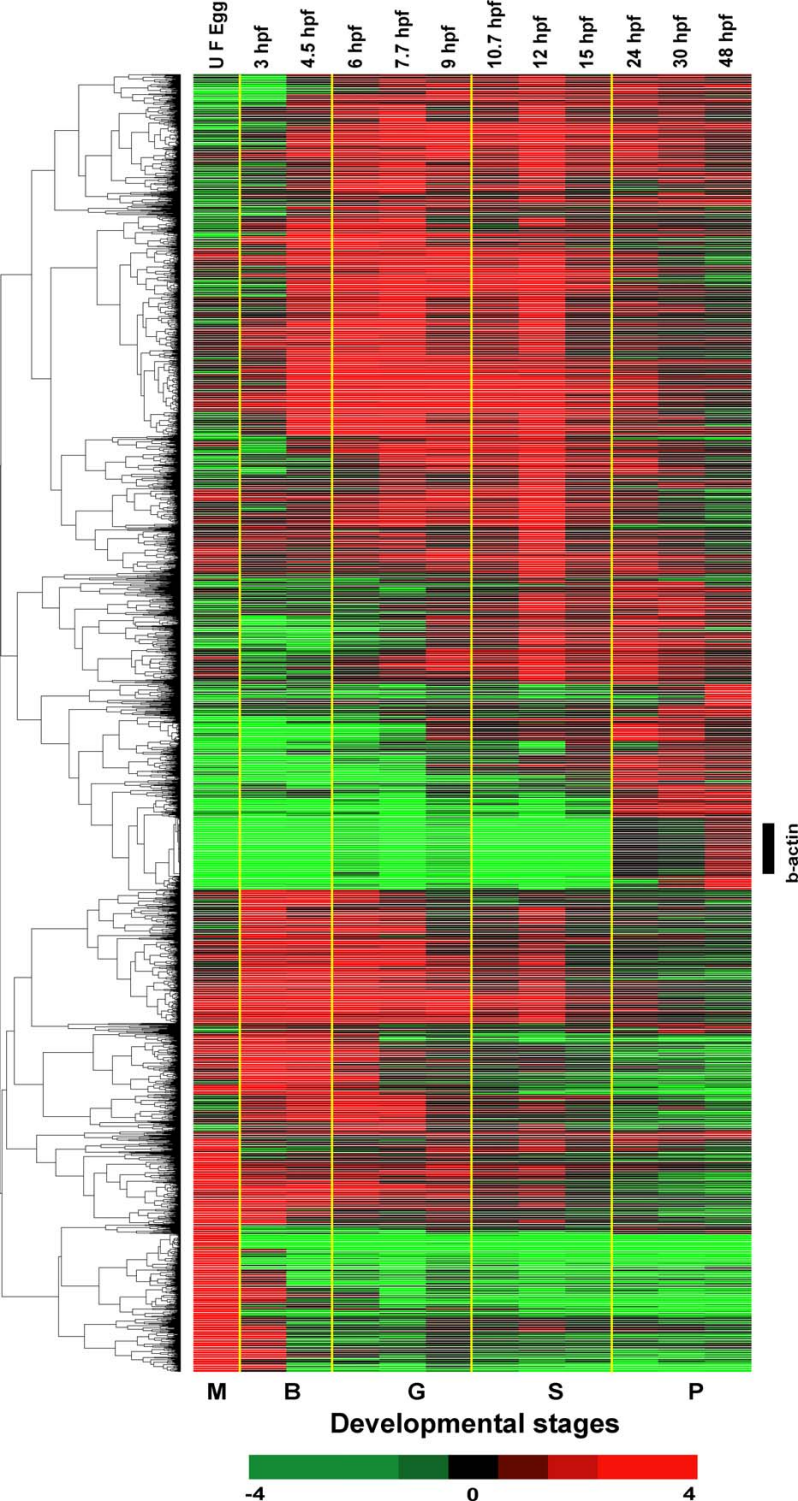
Hierarchical Clustering Analysis of Differentially Expressed Zebrafish Genes Of the 16,416 genes in the array, 3,657 genes, which showed a significant level of differential expression at least at one time point, were clustered hierarchically into groups on the basis of similarity of their expression profile, following Eisen's clustering method. The horizontal lines indicate the expression pattern of each gene, and the vertical rows indicate the embryonic developmental stages. For each gene, the ratio of transcript abundance in the developing stages of the embryo to its abundance in the reference RNA is represented by color intensities (red color indicates the higher expression, and green color indicates the lower expression of the gene in the embryos). Coordinated expression of the beta-actin gene (control) is indicated.

**Figure 2:**
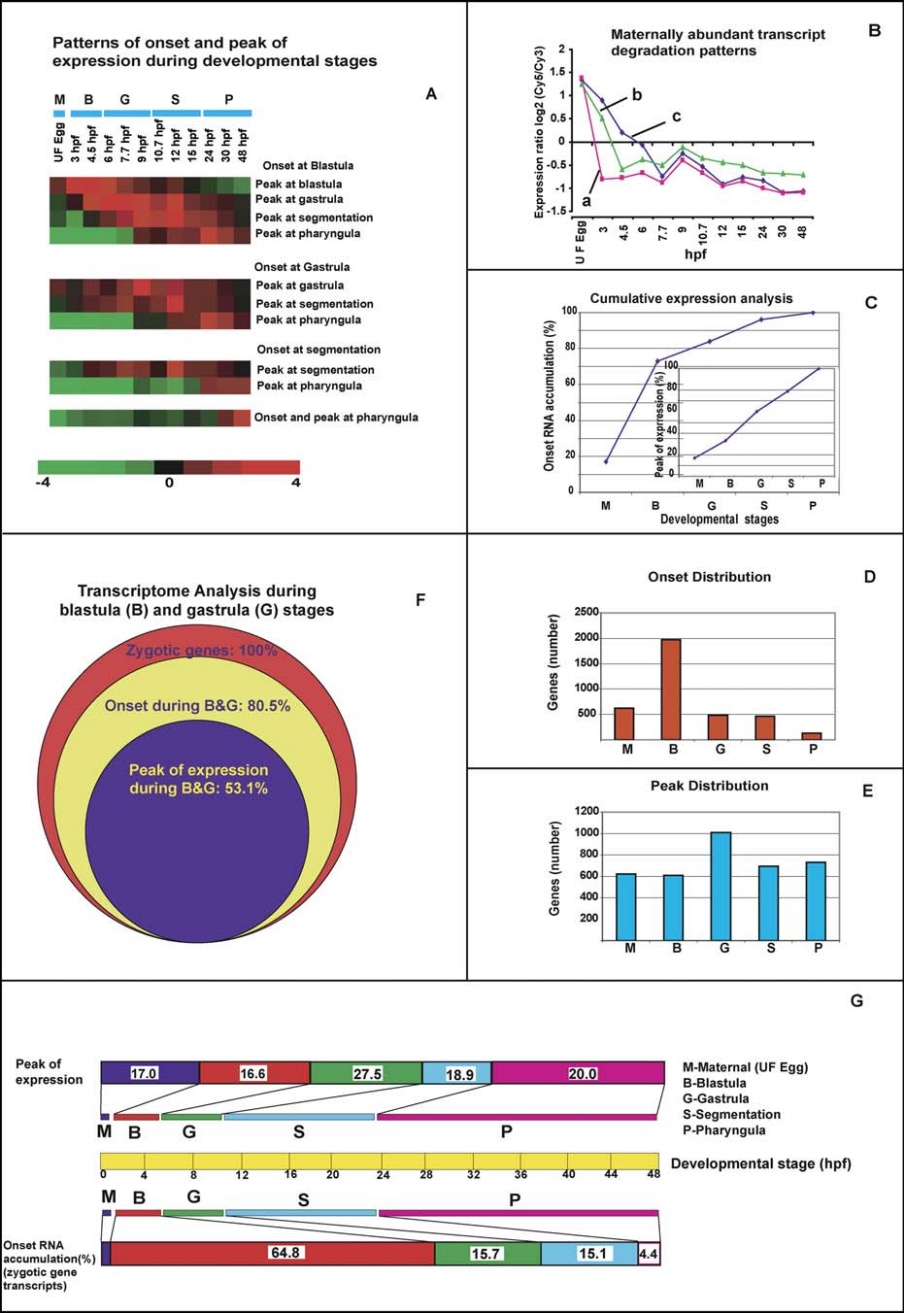
Analysis of Expression Patterns of the Zebrafish Embryonic Transcriptome (A) The onset and peak of transcript accumulation were calculated using an algorithm for each of the differentially expressed genes. An average performance of the gene in each group is presented in the clustergram. Of the set of 1,972 genes commencing at the onset of the blastula stage, subsets of genes displayed peak activity at blastula, gastrula, segmentation, and pharyngula stages. Average performance of these genes in each subset is represented in the clustergram. Similar analysis was made for the genes commencing transcript accumulation at gastrula, segmentation, and pharyngula stages, and the data are presented. (B) Patterns of differential degradation of maternally loaded transcripts. (a) Transcripts level of the genes in this group degraded drastically prior to blastula. Groups of genes whose transcripts persisted till blastula (b) and subsequent stages (c) of development are represented graphically. (C) Cumulative analysis of onset and peak of expression of zygotic genes. A number of zygotic genes commenced their transcript accumulation steadily during early development. About 99% of the genes initiated transcript accumulation by the end of the segmentation stage. The figure inserted in Figure 2C indicates that the percentage of genes showing peak of activity also increased steadily and about 90% of the zygotic genes displayed their peak of expression within 24 hpf (prior to the pharyngula stage). The dynamics of zygotic genome activation during early development is evident from the cumulative expression analysis. (D and E) Frequency distribution of onset of transcript accumulation (D) and peak of expression (E). Of the 3,657 genes analyzed, about 54% (> 2,000 genes) commenced transcript accumulation during the blastula stage. Considering the peak of expression, the majority of genes (> 1,000 genes) displayed a peak of expression during the gastrula stage. (F) Transcriptome analysis during blastula and gastrula stages. Of the total zygotic genes (3,035 genes; 100%; red circle), percentage of genes commencing transcript accumulation (80.5%; yellow circle) and showing peak of expression (53%; light-blue circle) during blastula and gastrula stages are indicated pictorially. (G) Total period of embryonic development of zebrafish (48 h; yellow bar) and the time required for each stage of development is indicated (color-coded for each stage). Considering all the developmentally regulated genes (3,657), the percentage of genes showing peak of activity at the designated developmental stages are indicated in the top bar. The bottom bar indicates the percentage of zygotic genes (3,035 genes) commencing transcript accumulation at the designated developmental stages.

Hierarchical clustering of 3,657 genes demonstrated diverse temporal expression profiles during zebrafish embryogenesis (Figure 1 and Dataset S1). The complexity of the gene cluster clearly displayed the significant modulations in the temporal activity of developmentally regulated genes. The cluster also revealed the identical expression profile of all the copies of beta-actin genes. Of the 3,657 genes extracted for the analysis, 622 genes showed a maximum level of transcript accumulation at the maternal stage (unfertilized egg), and the rest of the 3,035 genes showed an onset and peak of transcript abundance during one or more stages of embryonic development (Figure 2).

### Differential Degradation of Maternally Loaded Gene Transcripts

The dataset of 622 gene transcripts displaying maximum abundance at the unfertilized egg stage (maternally loaded transcripts; (Figure 2B and Dataset S2) was divided into three groups based on the pattern of transcript degradation. The first group represented 221 genes, and the transcripts of these genes persisted only at the unfertilized egg stage and were degraded prior to the commencement of the blastula stage (Figure 2B, line a). This group contained the known maternal genes (examples are those encoding proteins of the zona pellucida, *zp2, zp2.4,* and *zp3* [34]) and the chorion protein component. A number of genes with putative annotations also displayed an expression pattern identical to previously characterized maternal genes, suggesting a possible maternal function. The clones with putative annotation of rhamnose-binding lectins (STL) of other fish are included in this group of genes. This family of lectins is expressed in the ovary and egg of the steelhead trout *(Oncorhynchus mykiss).* Furthermore, a few members of the lectin family (STL2 and STL3) were found to be abundant in the ovary and the levels dramatically decreased after fertilization [35,36], indicating the maternal specificity of this gene. We have identified here, for the first time to our knowledge, a large number of previously uncharacterized genes and ESTs that appear to have maternal-specific activity. The maternal function of these clones and their control of zygotic genome activation will be an important area for future investigations.

The second group of transcripts (259 genes) persisted to a greater extent during maternal and blastula stages and subsequently degraded (Figure 2B, line b). These genes may be involved in cleavage and other early embryonic functions. One example is the maternally derived gene *cyclin B1,* which is expressed up to the early blastula stage. It is known that zebrafish embryos undergo synchronous cell divisions during the cleavage stage prior to their entry into the MBT [30]. *cyclin B1* has been shown to be involved in the synchronous cell division phase [37], consistent with its maximum activity during these stages. Similar to *cyclin B1,* there are a number of genes that display activity at maternal and early zygotic stages. The gene transcripts that persisted during the blastula stage are involved, in one way or the other, in early developmental functions. Some of the genes functionally relevant during maternal and blastula stages are given here as examples: *calreticulin* (a chaperone involved in folding of newly synthesized glycoproteins), *catalase* (catalytic functions in cell growth), *birc5b/survivin2* (cell death regulator), *mcl1a* (cellular apoptosis function), *claudin-like gene* (tight junction function during growth), *lmnl3 (maternal lamin L3),* oocyte and blastula nucleoskeletal proteins of the nuclear membrane during cell division. These gene transcripts are degraded slowly (by the end of the blastula stage). The third group (Figure 2B, line c) had 142 genes with a maximum level of transcripts at the maternal stage; this group of genes underwent a very slow rate of transcript degradation. Some of the transcripts persisted up to the segmentation stage, and the average performance of this group is presented.

These observations revealed that the maternally deposited gene transcripts are not degraded at the same rate and there is a definite gene-specific differential degradation. The differential degradation and the extended stability of specific maternal genes implicated a potential relevance of these genes in later zygotic stages. It is known that after fertilization the earliest developmental processes in the egg are programmed by maternally deposited gene transcripts [38]. Subsequently, embryos initiate zygotic genome activation for the continuation of embryonic development. The maternal control of zebrafish development before and after MBT has recently been reported following extensive analysis of maternal mutants [2,3]. These authors showed that mutations in some of the maternal genes have generated embryos with defects in zygotic gene functions and developmental alterations during pre-MBT, MBT, and also beyond these stages. In this study, we have identified maternal stage-specific expression for a number of previously uncharacterized ESTs, which may play a vital role in modulating normal development. It has been reported in *Drosophila* that maternal RNA is degraded during the course of early development either by maternally derived factors (to degrade transient transcripts) or zygotically derived factors (to degrade moderately stable transcripts), and that the combined action of maternal and zygotic factors is required to degrade the highly stable maternal transcripts [39–42]. Zygotic regulation of maternal cyclin A1 and cyclin B2 mRNAs has been demonstrated in *Xenopus laevis* [43,44]. We presume that similar mechanisms of maternal RNA degradation reported for *Drosophila* and *Xenopus* may be acting in zebrafish to differentially degrade the maternally loaded messages.

### Zygotic Transcriptome Analysis and an Overview of Stage-Specific Clusters

The time point at which the gene transcripts commenced accumulation from their basal expression level is considered as the time of onset, and it is determined using an algorithm (see Materials and Methods and Protocol S1). In the present study, stage-wise developmental onset of gene transcription was determined for 3,052 zygotic genes expressed during MBT and beyond (Figure 2A). Of these, transcript accumulation of 1,967 genes began during the blastula stage, which represents 64.8% of the zygotic genes. Though these genes commenced transcript accumulation at the blastula stage, a subset of these genes showed expression peaks at subsequent stages of development. It was also observed that 15.7% (477genes), 15.1% (460 genes), and 4.4 % (131 genes) of the zygotic genes began their onset of transcript accumulation at gastrula, segmentation, and pharyngula stages, respectively. Average performance of the genes in these groups is presented as a clustergram (Figure 2A and Datasets S3–S12). Onset of temporally expressing genes aligns with their required developmental functions. For example, some of the genes exhibiting early transcript accumulation are those involved in early developmental functions such as the cell cycle *(cyclins),* cell regulation and cell adhesion *(claudins, connexins),* embryonic apoptotic functions *(survivins),* transcriptional regulation *(smad, pou, sox, wnt),* and other early developmental processes. The genes that began transcription during later stages of development (segmentation and pharyngula) are related more to organogenesis and encode, to a large extent, structural proteins (e.g., collagens, pro-collagens, skeletal muscle proteins, selenoproteins, ceruloplasmin).

Embryonic development in zebrafish (from fertilization to hatching) extends for 48 h. Within the first 5 h post-fertilization (hpf), cleavage and blastula are completed, and the process of gastrulation takes another 5 h. Thus, within 10 hpf (which is equal to about 21% of the total embryonic period), the early developmental processes are completed. Segmentation and pharyngula stages are completed in about 14 and 24 h, respectively (Figure 2G). About 80.5% of the zygotic genes initiated their transcript accumulation during the blastula (64.8%) and gastrula (15.7%) stages. Considering the peak of expression of zygotic genes, about 53% (Figure 2F) of the genes attained their peak during blastula and gastrula stages. Such high level of activity occurred within about 21% of the developmental period. Cumulative analysis of onset and peak of expression showed a sigmoid pattern: the onset of transcript accumulation sharply increased, displaying about 99% of the activity by 12 hpf (Figure 2C). Similarly, most of the developmentally regulated genes attained their peak by about 24 hpf (Figure 2C insert). Analysis of the frequency of distribution indicated the dominance of transcriptome dynamics during blastula and gastrula stages (Figure 2D and 2E). It is interesting that such a high percentage of genes would be activated within the relatively short window of the developmental period.

**Figure 3:**
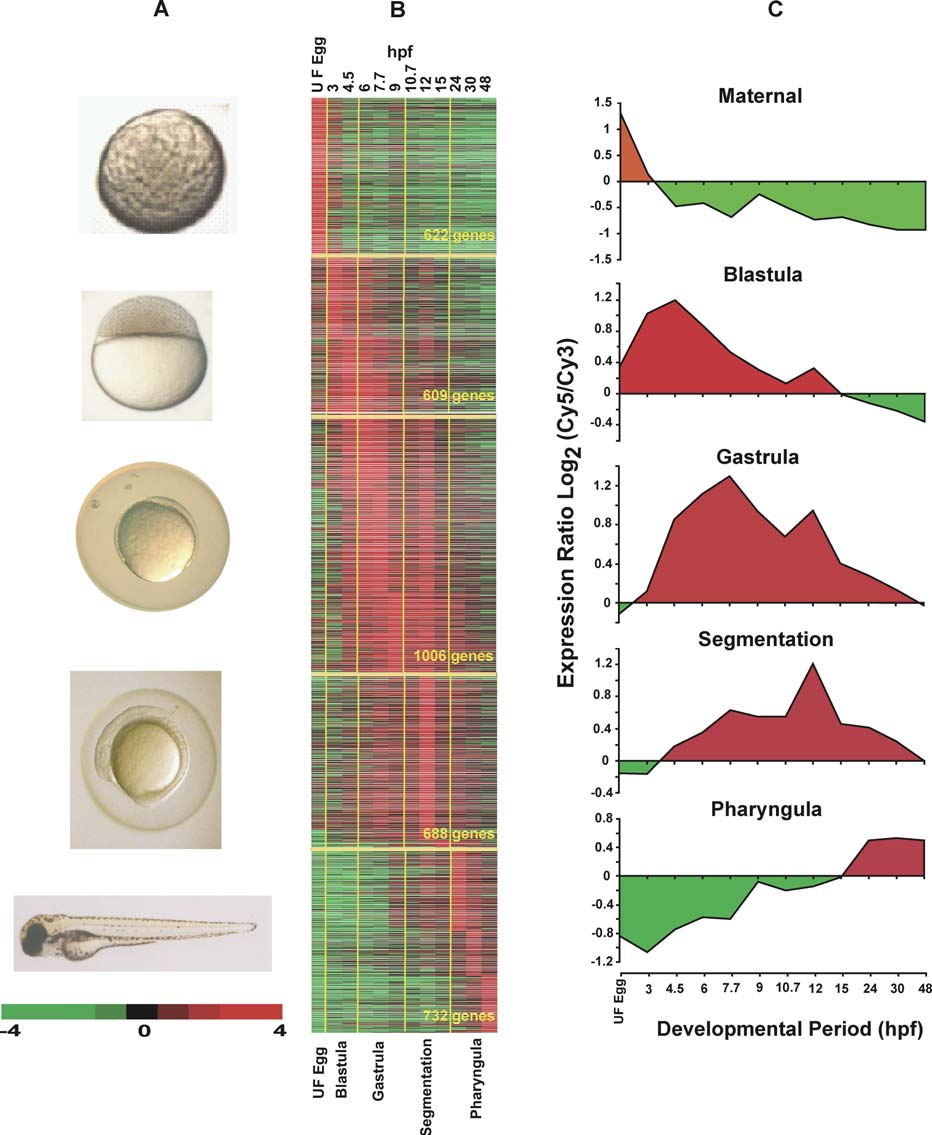
Overview of the Expression Patterns of Genes Peaking at Selected Developmental Stages (A,B) Differentially expressed genes were clustered based on the peak of expression at the selected developmental stages and presented in the clustergram. Of the total number of 3,657 genes analyzed, 622, 609, 1,006, 688, and 732 genes showed peak expression at maternal, blastula, gastrula, segmentation, and pharyngula stages, respectively. (C) General trend and average performance of the genes at each developmental stage are graphically represented (red, high expression; green, low expression). It is clear from this analysis that different sets of genes displayed their maximum at different stages of development, indicating the temporal/stage-specific maximum activity of the developmentally regulated genes.

To obtain an overview of embryonic gene expression, we sought to specifically address the temporal expression patterns in the following developmental stages: (1) maternal (unfertilized eggs); (2) blastula; (3) gastrula; (4) segmentation; and (5) pharyngula/hatching stages (Figure 3A). From the expression data, we selected the genes that showed peak of activity at each stage and presented the data in clustergrams (Figure 3B). The average performance of the genes in each developmental stage is also graphically presented (Figure 3C). This analysis illustrates that different set of genes have peaks of expression at different developmental stages suggestive of their relevance for stage-specific developmental functions. Intriguingly, the clusters of the developmentally regulated genes grouped into specific or adjacent clusters, forming a wave of gene activity moving from maternal to blastula and through each subsequent stage of embryogenesis. The clustering of developmentally related genes indicated that the process of embryogenesis is continuously regulated and displayed dynamic gene activity that, in turn, directs the evolving program of development.

The following observations can be made from this analysis. Of the total number of 3,657 genes analyzed, 622 genes (17%; Dataset S13) showed maximum activity at the maternal stage. Of the remaining 3,035 (zygotic) genes, 609 (16.6%), 1,006 (27.5%), 688 (18.9%), and 732 (20.0%) genes showed maximum transcript levels at blastula (Dataset S14), gastrula (Dataset S15), segmentation (Dataset S16), and pharyngula (Dataset S17) stages, respectively (Figure 3B). Analysis of our results indicated that some of the genes have short spans of activity while others have extended periods of expression, suggestive of functional requirements. As an example, we analyzed the array data and found abundant transcripts for *birc5b (baculoviral IAP repeat-containing 5B,* also called *survivin2)* and cell-death regulator *mcl1a (myeloid cell leukemia sequence 1a)* up to the blastula stage. These gene families have been implicated in anti-apoptotic functions [45–47]. It has also been shown that *survivin* is abundantly expressed in *Xenopus* during oogenesis and early embryogenesis functioning as an inhibitor of apoptosis and a positive regulator of the cell cycle [48]. The transcript abundance of *birc5b* and *mcl1a,* negative regulators of apoptosis, during maternal/blastula stages, indicated the importance of regulating apoptotic process during early embryonic development. It has been reported that embryonic apoptosis is activated in the zebrafish during the gastrula stage, and it is most likely that down-regulation of the maternally derived negative regulators may activate the embryonic apoptotic process in zebrafish during the gastrula stage [49].

Genes displaying a peak of expression during the pharyngula stage continued their expression beyond the pharyngula stage, suggesting a functional requirement for these genes during embryonic and post-embryonic stages of development. For example, *ceruloplasmin (cp),* which has been implicated in liver development [50], displayed maximum expression during the pharyngula stage. Expression of this gene did not terminate at the end of the pharyngula stage, suggesting that its continued expression during post-embryonic stages could be playing a role in liver function. The genes that displayed similar expression are involved in parallel embryonic and post-embryonic developmental processes. For example, muscle-specific genes and collagens also show similar patterns of expression, as they are involved in the progressive formation of muscles and skeletal development in zebrafish. It is known that almost all the primordial processes of organogenesis commence at gastrulation and continue during segmentation and post-segmentation stages. Thus, an overview of genome-wide expression analysis demonstrated a temporally demarked expression pattern of developmentally regulated genes and showed that the gene activities were well correlated with the developmental events at the respective stages. Prior to our analysis, only 7% of the differentially expressed genes were fully annotated (with GO), and temporal expression patterns for the majority of the clones in the array were not established. Our analysis, for the first time to our knowledge, identified the temporal patterns of expression for about 80%–90% of the clones, and for most of them the patterns of expression are presented here for the first time, to our knowledge.

### Transcription Analysis of Selected Clusters of Functionally Related Genes

From the array data, expression patterns of a number of functionally related genes could be identified based on their GO and/or putative functions. However, we selected three groups of genes involved in (1) cell cycle, (2) ubiquitin functions (ubiquitins and proteasomes), and (3) somitogenesis as examples and discuss here the expression patterns of these genes in relation to their biological significance.

#### Cell cycle.

**Figure 4:**
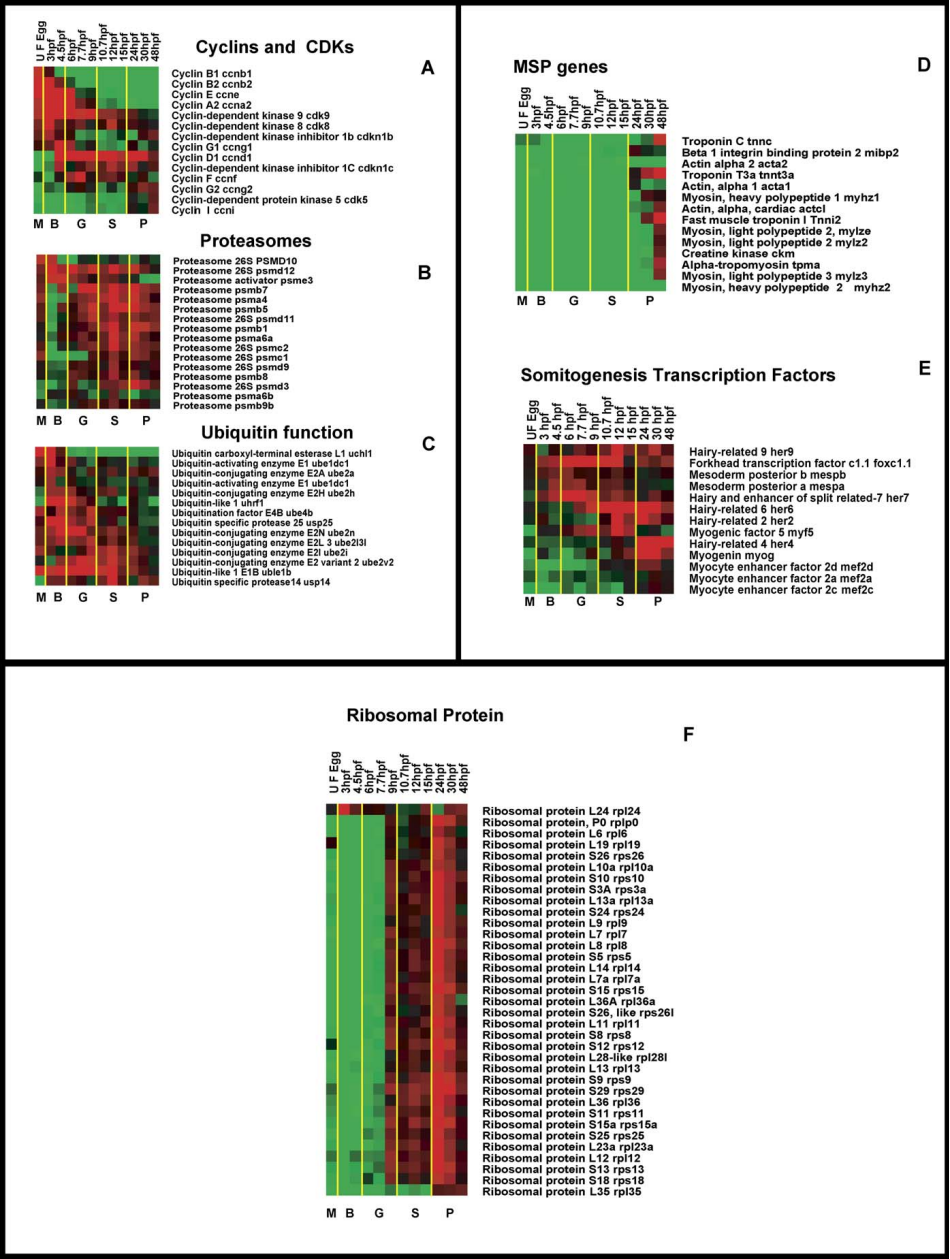
Expression of Genes Involved in Specific Functions (A) Expression patterns of genes involved in the cell cycle. Genes involved in the cell cycle, namely cyclin and cyclin-dependent kinase genes, were identified based on GO and clustered separately. Most of the cyclin genes commenced their expression maternally, and the cyclin-dependent kinase genes showed expression throughout embryogenesis. (B and C) Genes involved in ubiquitin function (proteasomes and ubiquitins) are active during most of the developmental stages, and the peak of activity is between the gastrula and segmentation stages. (D) Gene expression during somitogenesis. Genes involved in somitogenesis were selected based on the GO list. Expression of MSP genes (acta 1, actc1, tpma, ckm, tnnt1, mibp2, myhz1, myhz2, mylz2, mylz3, tnnc, and tnnt3a) are clustered. Most of the MSP genes commenced their expression around 12 hpf and displayed their maximum during the pharyngula stage. (E) Expression pattern of somitogenic (myotome-specific) transcription factors (mespa, mespb, mef2a, mef2c, med2d, myf5, myog, foxc1a, her4, her6, her7, and her9). Most of the transcription factors commenced transcript accumulation from the blastula stage onwards well in advance of commencement of MSP gene transcription. (F) Coordinated expression of RP genes. From the array data, the expression profile of RP genes was clustered. Most of the genes showed an identical pattern of expression.

Expression of cell cycle related genes encoding cyclins and cyclin-dependent kinases were analyzed (Figure 4A; Dataset S18). Most of the *cyclin* genes involved in the cell cycle commenced their expression maternally, maintained expression through the blastula stage, and were subsequently down-regulated. The down-regulation of maternally derived cyclin transcripts was shown to be regulated by zygotically derived factors [43,44,51]. From the array data, it is clear that *cyclinD1* alone commenced expression zygotically (at the blastula stage) and exhibited its peak after MBT, an observation that is in agreement with the earlier analysis of *cyclinD1* gene transcription [52]. Thus, *cyclinD1* is a non-maternally supplied G1 *cyclin,* and the onset of expression indicates the G1 phase in the developing embryo [52]. In contrast to *cyclinD1,* the maximum level of *cyclinB1* and *cyclinE* transcripts were observed during the phase of synchronous cell divisions consisting of S and M phases (prior to MBT) [37,53]. However, the continued presence of transcripts of *cyclinE* during asynchronous cell divisions and post-MBT development indicated that the expression profiles differ from the transcripts of *cyclinB1*. Cyclin-dependent kinases, their regulatory subunits, activators, and associated proteins showed almost continuous transcript abundance with a maximum during blastula, gastrula, and segmentation stages (Figure 4A), indicating their continuous involvement in the regulation of one or other cyclin genes during the cell cycle.

#### Ubiquitins and proteasomes.

Genes involved in protein degradation by ubiquitin-mediated proteolytic function displayed low levels of transcripts during early development (up to the blastula stage). From the beginning of the early gastrula stage, these genes increased their transcript levels (Figure 4B; Dataset S19). Most of the genes encoding the proteasome subunits are involved in ubiquitin-mediated proteolysis (Figure 4C). Ubiquitin-mediated proteolysis and regulation of cell differentiation through the *notch* signaling pathway has been described previously [54]. In zebrafish, an involvement of the ubiquitin pathway in the degradation of paired-like homeobox gene *Vsx1* has been reported [55]. From the present array data, significant expression of proteasome subunits could be detected from the late blastula stage. The expression patterns of proteasome subunits and ubiquitin conjugating enzymes present a global view of transcriptional regulation of genes behind the ubiquitin-dependent proteolytic pathway during zebrafish development.

### Analysis of Gene Expression during Somitogenesis

Initiation of organogenesis in the embryo is accomplished by a set of structural genes and the transcription factors that modulate their expression. We have taken somitogenesis as an example and analyzed a subset of genes involved in this process. Broadly, somitogenesis can be divided into an early phase where the paraxial mesoderm is subdivided in a rostrocaudal pattern into blocks of cells called somites and a subsequent phase of cell differentiation within the somites where somatic cells acquire a variety of distinct dorsoventral and mediolateral fates. In zebrafish, the dorsolateral somitic mesodermal cells contribute to muscle development. Consistent with this, most of the muscle-specific protein (MSP) genes commenced expression during early somitogenesis, and they steadily increased their transcript abundance in positive correlation with somite formation. From the array expression data, the transcripts for MSP genes were first detected around 11 hpf (Figure 4D; Dataset S20), and, subsequently, their transcript levels increased in accordance with the progress of somitogenesis. We compared the expression of selected MSP genes from arrays with Northern blot analysis of the same genes reported earlier [56], and the results were identical. Therefore, array analysis faithfully recapitulated the data obtained from single gene analysis (Northern or WISH).

Distinct classes of genes encoding transcriptional regulators are expressed dynamically during somitogenesis in all vertebrates and precede the onset of MSP gene expression. The activities of members of the bHLH transcription factors, namely *hairy (h), Enhancer of split (E spl),* and *hairy-related,* have been shown to play critical roles during somitogenesis in vertebrates. Analysis of the zebrafish bHLH family of *h/E (spl)-*related genes has shown the involvement of *hairy related 9 (her9-)* [57], *her1-, her4-, her6-,* and *her7-* [58,59] in somitogenesis. Our array data clearly revealed that most of these transcription factors (*mespa-, mespb-, mef2a-, mef2c-, mef2d-, foxc1a,* and a number of *her* genes) commenced their expression prior to the onset of muscle-specific gene expression (Figure 4E). These results revealed that global analysis of gene expression using microarrays could detect a cascade of gene activity involved in somitogenesis in the zebrafish. Similar analyses could be extended to explore the expression patterns of genes involved in other organ systems.

### RP Genes Are Coordinately Expressed during Zebrafish Embryogenesis

From the array expression data, 35 RP genes of zebrafish (similar to known RP genes) showed temporally coordinated expression (Figure 4F; Dataset S21). From the clustergram, it is evident that the level of transcripts of almost all the RP genes changed in concert except for a few, which differed slightly in their expression profile. On average, the onset of RP gene transcript accumulation commenced at the blastula stage and the expression increased continuously. Assembly of ribosomes requires coordinated expression of genes coding for their structural components, and are represented by several rRNA molecules and about 80 RPs. In eukaryotes, genes encoding rRNA and 5S RNA are amplified and presented by multiple copies, whereas the genes for RPs are present in only one or two copies per haploid genome. It has been reported that after fertilization and during the period of synchronous cell division, no transcripts were detected for zygotic genes [60]. This transcriptional block is terminated at MBT in concert with desynchronization of the cell cycle. It was shown that transcripts for some of the RPs *(rps3, rpl17,* and *rpl31)* [61] and *rps24* [62] were detected at the early blastula stage prior to the detection of new zygotic transcripts in the sea urchin, suggesting their maternal origin. Published data on the expression of fish RP genes have not presented a comprehensive view [63,64]. However, our results presented here indicate that almost all the RP genes were coordinately expressed in a global and continuous fashion from the onset (beginning of MBT) to hatching.

### Accumulation of Gene Transcripts Do Occur prior to the MBT in Zebrafish

**Figure 5:**
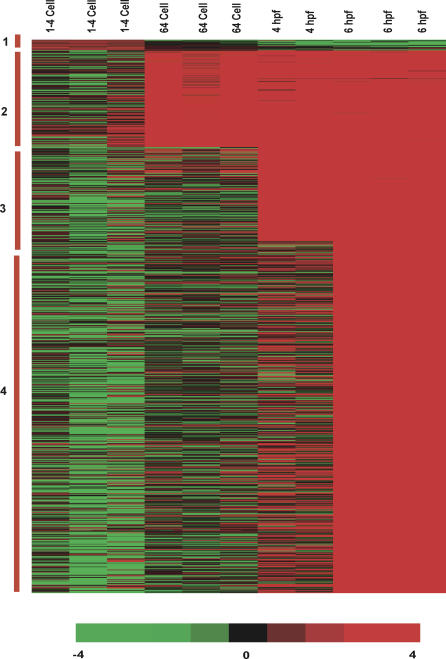
Analysis of the Zebrafish Transcriptome during Pre-MBT and Post-MBT Stages of Embryonic Development Transcriptional profiles of the genes during pre-MBT: one- to four-cell, 64-/128-cell, and post-MBT: blastula (4 hpf) and gastrula (6 hpf) stages were analyzed. This analysis revealed four different gene clusters. Clusters 1, 3, and 4 represent gene expression patterns, which have been identified earlier. Cluster 2 represents a novel pattern of gene expression commencing transcript accumulation at the 64-/128-cell stage onwards that represents the pre-MBT stage.

Gene expression patterns during early development (pre-MBT and post-MBT stages) were studied by looking at the profiles of expression at selected time points during early cleavage and post-cleavage stages. The expression data obtained during these stages were selected following the statistical method described in the Materials and Methods section. Groups of genes showing distinct expression patterns were clustered based on the onset of transcript accumulation (Figure 5; Dataset S23–S25). Four distinct expression patterns were recorded. The first cluster represented maternally loaded transcripts (Dataset S22), and almost all the transcripts in this cluster degraded rapidly prior to blastula (4 hpf). Cluster 3 (Dataset S24) and Cluster 4 (Dataset S25) represented the expression of zygotic genes commencing the transcript accumulation after MBT stage (blastula and gastrula). This pattern of expression is in accordance with the pattern described earlier [30]. Cluster 2 contained a cohort of genes (Dataset S23) that commenced transcript accumulation prior to the onset of MBT. This expression pattern is a new discovery, demonstrating pre-MBT accumulation of zygotic transcripts. As transcript accumulation can take place only in the presence of gene transcription, these genes represent a group of pre-MBT transcribed genes. From a total of 16,416 genes analyzed in the array, 125 genes showed evidence for pre-MBT transcript accumulation. These genes did not show transcript accumulation during the one-cell, four-cell, and eight-cell stages; however, transcripts for this subset of 125 genes increased from the 64-/128-cell stage onwards. This pattern of pre-MBT zygotic gene expression has not been previously reported in zebrafish. We were the first to detect transcript accumulation at these early cleavage stages, owing to the unbiased and global nature of high-density microarrays.

Since these novel gene transcripts were the earliest activated in zebrafish development, they are likely to be involved in specific early embryonic functions and possibly in subsequent genome-wide activation. We analyzed several of the genes in this cluster based on the predicted putative functions, which are as follows. Several proteasome subunits in this cluster are known to be involved in protein degradation by the ubiquitin-mediated proteolytic pathway [54,55]. The proteasomes may be required at this stage for the proteolytic degradation of maternal gene products. A number of proteasome subunits have been shown to be functional in the proliferating ARPE19 retinal pigment epithelium cells. Since the cleavage stage is similar to the proliferating cells stage, it is reasonable to find the proteasome transcripts at this stage. This cluster also contains the putative gene for ubiquitin conjugating enzyme, and it is known that ubiquitin conjugating enzymes mediate ubiquitination and degradation of specific substrates by the ubiquitin-dependent degradation pathway [65]. This enzyme has also been shown to promote cyclin proteolysis during mitosis [66]. We have reported in the present study that transcript levels of cyclins involved in cell cycle regulation *(cyclin A2, cyclin B, cyclin B1, cyclin E)* are highly abundant in unfertilized eggs and during cleavage stages [37,53]. For the degradation of these gene products, proteasomes and ubiquitin conjugating enzyme may be required at the synchronous cell cycles stage (pre-MBT) of zebrafish development.

Another gene in this cluster is a putative RNA helicase. RNA helicases are implicated in a number of cellular processes involving RNA secondary structures such as translation initiation and ribosome and spliceosome assembly. Some of the members of this family have been active in embryogenesis and in embryonic stem cells. RNA helicase has been shown to be essential for normal gastrulation in the mouse [67]. It has been shown that RNA helicase II is necessary for cell growth and cell cycle progression [68], and a role for RNA helicases in ribosomal RNA biogenesis in *Xenopus* oocytes has been observed [69]. In addition, it has been shown that down-regulation of RNA helicase II results in depletion of ribosomal RNA production in *Xenopus* oocytes and cell culture [69,70]. Thus, the expression of putative RNA helicase at the pre-MBT stage may be essential for cell growth and/or cell cycling during the cleavage stages of embryogenesis and also early commitment to normal gastrulation. Furthermore, it appears that the presence of RNA helicase II during early embryogenesis may facilitate ribosomal RNA production and the positioning of the translational machinery so as to meet the demand at MBT.

Other interesting genes in this cluster are alanyl-tRNA synthetase and nascent-polypeptide associated complex alpha (DNA primase small subunit). Transcripts for these genes have also been found in ES cells. DNA primase is involved in nascent polypeptide production resulting in protein biosynthesis, and tRNA synthetase is involved in protein synthesis related processes such as tRNA binding and tRNA ligase activity. Thus, all the genes described above are related to cell growth, cell proliferation, cell adhesion, nascent RNA synthesis and stability, and other cellular functions. It may be that these genes need to commence their function during pre-MBT stages in order to facilitate normal progression through MBT and subsequent embryonic development. GO analysis of the genes in this group reveals that more than 50% of the genes show putative functions such as DNA/RNA binding, development, protein folding, and protein biosynthesis (Figure S4). Human and mouse orthologs of some of the known genes in this group are given in Table S2. Clusters 3 and 4 displayed the cohort of zygotic genes displaying maximum transcript accumulation at blastula (4 hpf) and gastrula (6 hpf) stages, respectively. The genes that have been shown to be expressed in blastula- (for example, *cyclin D1*) and gastrula- (for example, *cathepsin L*) stage embryos were identified with Clusters 3 and 4, respectively, as expected. Thus, the microarray detected the expression of known genes at the expected time points, once again confirming the reliability of the array detection.

It has been shown that transcription can occur prior to MBT in *Xenopus* embryos [31]; namely, these authors showed that *beta-catenin* and *tcf* regulated pre-MBT transcription of *nodal* genes *xnr5* and *xnr6* does take place. However, in this study, they restricted the investigation to single gene expression analyses. Most of the earlier studies on the functional analysis of zebrafish genes were restricted to a handful of genes using WISH or Northern blot analysis with embryos at MBT or beyond. Parallel analysis of the expression of thousands of genes at selected closely spaced time points and early cleavage stages has not been previously attempted, to our knowledge. Thus, we are the first to our knowledge to undertake parallel analysis of expression of early genes using high-density zebrafish arrays. This approach revealed a cohort of genes that actively accumulate zygotic transcripts prior to the MBT stage, and the data presented here will serve as a starting point for the functional investigations of the roles of these genes during zebrafish embryogenesis.

**Figure 6:**
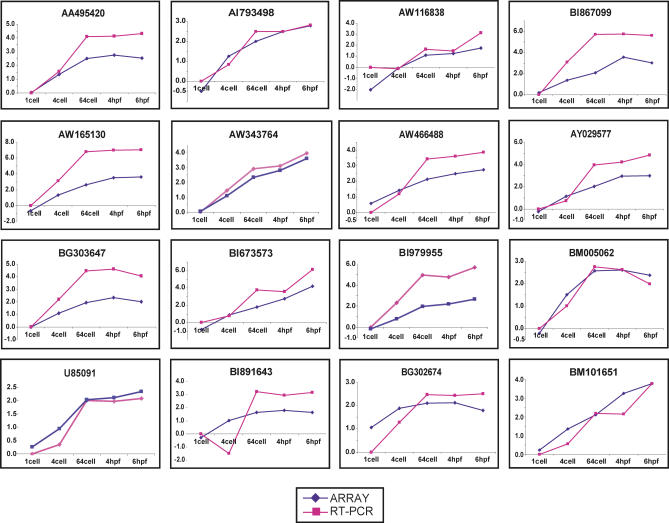
Validation of Zebrafish Transcriptome Pre-MBT and Post-MBT Results Comparison between RT-PCR and microarray results for selected genes. Real-time PCR was done for 16 clones from Cluster 2 using the same RNA used for array hybridization. The RT-PCR profile closely parallels the microarray data, cross-validating both techniques as quantitative estimates.

**Figure 7:**
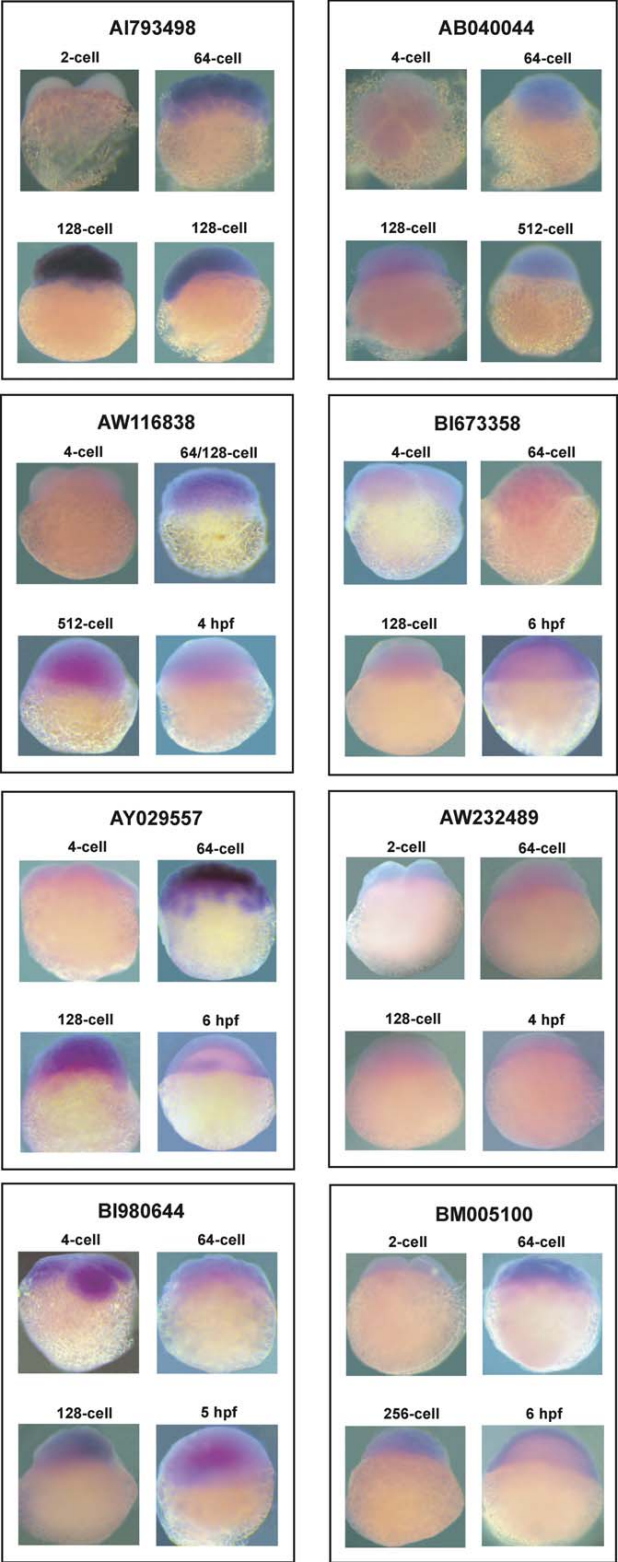
WISH Was Performed for Selected Clones All the clones subjected for in situ analysis showed a lower intensity of hybridization signal at one- to four-cell stages and increased intensity at 64-/128-cell and 256-/512-cell stages. These observations support the data obtained in the real-time PCR and microarray data, indicating the pre-MBT accumulation of transcripts.

To validate the expression profiles, real-time PCR was done for selected genes from each cluster. Real-time PCR was done for 16 samples from Cluster 2 (Figures 5 and 6) using the same total RNA used for array hybridization. The pattern of expression was similar in both analyses. Subsequently, we extracted new batches of RNA from one-cell, two-/four-cell, 64-/128-cell, 256-/512-cell, and 4-hpf embryos and performed real-time PCR for eight genes from Cluster 2 and compared the array data with RT-PCR data (Figure S5). Of these eight genes, real-time PCR was done for four genes using independently isolated batches of RNA, and the results are comparable. Quantity of transcripts for the selected genes at 64-/128-cell and 256-/512-cell (pre-MBT stages) is more than that at one- to four-cell stages indicating the accumulation of new transcripts prior to MBT. Similarly, real-time PCR was also done for selected genes from Clusters 3 and 4 (unpublished data). The results show that the pattern of expression obtained via real-time PCR analysis parallels the observations made in the array. Furthermore, whole-embryo RNA in situ hybridization was performed for eight different clones from the early genes of which real-time PCR was also done. Hybridization intensity was significantly higher at 64-/128-cell and 256-/512-cell compared to one- to four-cell stages, indicating transcript accumulation during the pre-MBT stages of development (Figure 7). The pattern of in situ data is similar to the real-time PCR profile. All these observations clearly support the presence of a cohort of early genes in zebrafish exhibiting pre-MBT transcript accumulation.

In summary, we described here the first genome-wide microarray analysis of the embryonic zebrafish transcriptome. This study revealed a remarkable undulating developmental expression program and temporal clustering of gene groups never before characterized (to our knowledge) during embryogenesis. Furthermore, a previously unknown cohort of very early genes transcribed *prior* to MBT was discovered in this study, which now represents the earliest known (to our knowledge) transcribed and accumulating RNAs in zebrafish and revised our earlier thinking that MBT marked the onset of all zygotic gene transcription. We are the first to create a comprehensive integrated database by incorporating resources from different databases for the annotation of the clones in the zebrafish oligoarray (Compugen) that will serve as a significant resource for the zebrafish community.

## Materials and Methods

### Embryo collection.

Wild-type zebrafish (Singapore local stock) embryos were obtained from the zebrafish facility of the Institute of Molecular and Cell Biology. Embryos were collected immediately after fertilization, maintained at 28.5 °C, and staged by hpf using standard morphological criteria [71]. Unfertilized eggs were collected by squeezing the abdomen of spawning females. Embryos were collected at 12 time points (unfertilized egg, 3, 4.5, 6, 7.7, 9, 10.7, 12, 15, 24, 30, and 48 hpf) (Table S3), snap-frozen in liquid nitrogen, and stored at −80 °C. The 12 time points were assigned to the following developmental stages: (1) maternal (unfertilized egg), (2) blastula (3.0 and 4.5 hpf), (3) gastrula (6.0, 7.7, and 9.0 hpf), (4) segmentation (10.7, 12.0, and 15.0 hpf), and (5) pharyngula (24.0, 30.0, and 48.0 hpf). In order to maintain a uniform genetic background, all embryos were collected from the same batches of fish stock.

### RNA isolation and reference RNA.

Total RNA was extracted from all the frozen embryos using Trizol reagent (Gibco BRL, Gaithersburg, Maryland, United States). RNA quality was evaluated by gel electrophoresis, and the concentration was measured with a UV spectrophotometer. To prepare reference RNA, total RNA was collected from the following stages (embryos/adults) and mixed in equal concentration: 1 hpf, 4 hpf, 24 hpf, 48 hpf, 3-wk-old fry, adult of male and female. Sufficient amount of reference RNA required for the entire project was prepared at one time, and the aliquots were stored at −80 °C.

### Zebrafish oligonucleotide probe design and microarray construction.

Zebrafish oligonucleotide probes for this array were designed by Compugen and synthesized by Sigma-Genosys (The Woodlands, Texas, United States). For each gene, one 65-mer oligonucleotide probe was designed from the 3′ region of the sequence. Each probe was selected from a sequence segment that is common to a maximum number of splice variants predicted for each gene. The arrays contained 16,416 probes representing oligonucleotides of selected genes. Oligonucleotide probes were re-suspended in 3XSSC at 20 μM concentration and spotted onto poly-L-lysine-coated microscope slides using a custom-built DNA microarrayer. Printed arrays were post-processed following the standard procedure described for cDNA arrays [72].

### Target labeling and hybridization strategy.

For fluorescence labeling of target cDNAs, 20 μg of total RNA from the reference and experimental samples was reverse transcribed in the presence of Cy3-dUTP and Cy5-dUTP (Amersham Biosciences, Little Chalfont, United Kingdom), respectively. Labeled target cDNAs were pooled, concentrated, and resuspended in DIG EasyHyb (Roche, Basel, Switzerland) buffer for hybridization. Hybridization strategies were as described by [72] with the following modifications: (1) usage of DIG EasyHyb buffer (Roche) and (2) hybridization at 42 °C. For each developmental stage, two to four independent replicate hybridizations were performed using a minimum of two biological replicates. For dye bias control, we employed a “dye-swapping” strategy for microarray hybridization whereby the Cy dye labeling scheme was reversed for alternating replicate hybridizations [73].

### Data acquisition and statistical analysis.

The arrays were scanned using the GenePix 4000B microarray scanner (Axon Instruments, USA) to generate 16-bit TIFF images of Cy3 and Cy5 signal intensities. GenePix Pro 4.0 image analysis software (Axon Instruments, Union City, California, United States) was employed to measure the fluorescence signal intensities of the array features and local background.

Normalization of the two channels (sample and reference) was done for each slide using the intensity-based log ratio median method [32]. For selection of differentially expressed genes, which are supposed to be specific for a developmental stage, a two-step filtering process was used. First, only genes that differentially expressed at least at one time point (in all the arrays tested) were retained. Modified *t*-statistic (SAM) [33] with 15% of the standard deviation percentile as the fudge constant was used for identification of differentially expressed genes, and log2 ratios were used in the modified *t*-statistic. Predicted false discovery rate of 0.05 was used as the threshold for differential expression. At the next step, those differentially expressed genes were further filtered to determine whether they were specific for a particular stage. Paired *t*-test was used to check whether the means of log2 ratios of their expression levels (over the replicates) in a stage was significantly (at 95% confidence level) larger than the means at other stage(s). The above methods of analysis have been used for all the experimental data that are presented in this paper. The log ratios from replicate arrays were averaged, and the average expression values were used in subsequent analyses. The extracted datasets were hierarchically clustered and visualized (Cluster and Tree View; [74]).

### Onset and offset finding algorithm.

The objective of the algorithm was to detect the onset (beginning) and peak time of transcript accumulation for a given gene using its time-course expression profile. Peak of the expression is defined as the time at which the mRNA abundance attains its highest level. The onset of expression is defined as the earliest time of the peak in the profile. This algorithm was used for the single peak genes selected as differentially expressed for this study. Prior to applying the algorithm, the dataset was selected based on statistical methods (SAM and FDR) described above, which removed the noisy profiles. Apart from that, the algorithm contains a Gaussian smoothing step that also removes the noisy profiles. Both onset and peak of expression are the onset and peak of relative abundance rather than absolute abundance. Manual verification of the profiles derived by using the algorithm showed that less than 0.1% of the data deviated from onset time by just one time point. The prediction of peak of expression did not vary from the observed data. Details of the algorithm are given in Protocol S1.

### Annotation of the zebrafish oligoarray.

In order to obtain the most recent annotation, we tried to obtain the full-length sequences or assembled sequences for the clones in the array from different sources: Zebrafish gene collection (ZGC: http://zgc.nci.nih.gov/ZGC; ftp://ftp1.nci.nih.gov/pub/MGC/fasta); Zebrafish UniGene (UniGene build 79: ftp://ftp.ncbi.nih.gov/repository/UniGene/) and Zebrafish Gene Index ([ZGI] release 16: http://www.tigr.org/tigr-scripts/tgi/tc_ann.pl?db=zest ftp://ftp.tigr.org/pub/data/tgi/Danio_rerio/). If the full-length sequence was not available, we obtained the assembled sequences from the zebrafish UniGene assembly (unique, longest sequences in the UniGene assembly) and from the ZGI (release 16), which provides current tentative consensus sequences generating assemblies of ESTs of particular gene. However, some of the clones in the array are represented as ESTs. Thus, we downloaded the sequences for the full-length clones, assembled sequences, and ESTs for all the probes in the array.

Putative annotations available for the clones in the array were downloaded from the following databases: (1) Compugen description (provided by oligo supplier: http://www.labonweb.com/oligo (2) Entrez Gene (for gene ID, gene description, and gene symbol: ftp://ftp.ncbi.nlm.nih.gov/gene/; http://www.ncbi.nlm.nih.gov/entrez/query.fcgi?db=gene); (3) Zebrafish UniGene cluster ( for UniGene ID, UniGene description: ftp://ftp.ncbi.nlm.nih.gov/repository/UniGene/; http://www.ncbi.nlm.nih.gov/UniGene/clust); (4) GO http://www.geneontology.org release 21 November 2004; ftp://ftp.geneontology.org/pub/go/); (5) Locus link (ftp://ftp.ncbi.nih.gov/refseq/LocusLink/; release 21 November 2004); (6) UniGene protein similarity and description (for mouse and human protein similarity ID, percentage of similarity and description: ftp://ftp.ncbi.nlm.nih.gov/repository/UniGene/; http://www.ncbi.nlm.nih.gov/entrez/query.fcgi?cmd=Retrieve&db=protein&dopt=GenPept&list); (7) ZGI (build 16) (for gene description and tentative consensus sequence number; ftp://ftp.tigr.org/pub/data/tgi/Danio_rerio/; http://www.tigr.org/tigr-scripts/tgi/tc_ann.pl?db=zest); (8) HomoloGene (mouse and human homolog gene ID and description; http://www.ncbi.nlm.nih.gov/entrez/query.fcgi?DB=homologene; ftp://ftp.ncbi.nih.gov/pub/HomoloGene/); (9) Chromosomal location (ftp://ftp.ncbi.nih.gov/repository/UniGene/).

All the sequence data and annotation information are stored in a MySqL database and can be accessed at http://giscompute.gis.a-star.edu.sg/~govind/zebrafish/index.html details of retrieval are given in the help file. The database can be queried using the GenBank accession number of the probes in Compugen Zebrafish array and the following information can be obtained: (1) Compugen description, (2) Zebrafish UniGene ID (build 79), (3) Zebrafish UniGene description (build 79), (4) Entrez Gene description, (5) Entrez Gene ID and Gene symbol, (6) GO term, (7) Locus link, (8) UniGene protein similarity and description (mouse and human), (9) full-length or assembled sequences, (10) HomoloGene (human and mouse, build 38.1), and (11) chromosomal location of the gene. The annotation for all the GenBank entries in the Compugen array is available from the GIS-sponsored Web site designed for zebrafish microarray annotation (http://giscompute.gis.a-star.edu.sg/~govind/zebrafish/index.html). To our knowledge, we are the first to create such an integrated database and search facility for the zebrafish genes present in the array. This GIS zebrafish database will be updated periodically by integrating the latest release from the resource database.

From our expression analysis during embryogenesis, 3,657 differentially expressed genes were identified and annotated. There were no duplicates in the differentially expressed genes selected for the analysis. To obtain the annotation of these genes, the following procedures were adopted. Annotations/putative gene descriptions were obtained from the following databases by using GenBank accession number as query information: Entrez Gene, UniGene descriptions, protein similarity to human/mouse, and ZGI (build 16). From the above sources, we obtained annotation for about 75% of the clones. (Entrez Gene: 31%; UniGene, protein similarity, and ZGI: 44%). To annotate the rest of the clones, we BLASTed the assembled DNA sequence of the clones against the non-redundant database (ftp://ftp.ncbi.nlm.nih.gov/blast/db/ protein; ftp://www.expasy.ch/databases/sp_tr_nrdb/). Putative annotations were obtained for about 20% of clones from the non-redundant database. For the rest of the clones (5%), we got the description obtained from BLAST search (ftp://ftp.ncbi.nlm.nih.gov/blast/db/). By this process, we have obtained putative annotation for most of the differentially expressed genes. All of the resource data used in the annotations are available via our database (http://giscompute.gis.a-star.edu.sg/~govind/zebrafish/index.html http://giscompute.gis.a-star.edu.sg/~govind/zebrafish/data_download.html) (Datasets S26 and S27). Based on the gene description and GO, the genes involved in selected functions were identified using appropriate keywords, such as “cell cycle,” “proteasomes,” “RPs,” and so on.

### Northern blot hybridization and WISH analysis.

Total RNA isolated from embryos at various stages of development were fractionated on 1.2% formaldehyde-agarose gels and transferred to GeneScreen membranes following standard procedures. The blotted membranes were prehybridized and hybridized following standard protocols. Selected RP cDNA clones (l7a, s3a, s12, and s10) were used as templates for probe preparation and labeled with 32P using Random Primer DNA labeling system (Gibco BRL). Whole mount in situ hybridization using RNA probes labeled with digoxigenin (Roche) was carried out as previously reported [75].

### Quantification by real-time PCR.

In order to verify the microarray results, two randomly chosen differentially expressed genes were tested in real-time RT-PCR analysis for all the time points. The SYBR Green I (Roche) RNA amplification kit was used on the LightCycler according to the manufacturer's instructions. The beta-actin and myosin light chain 2 (mlc2f) transcripts were amplified using the following primers (beta-actin: LC1–5′ CCGTGACATCAAGGAGAAGCT-3′; LC2 5′-TCGTGGATACCGCAAGATTCC-3′; mlc2f: LC1 5′-TCTCACTCATTACCCACAA-3′; LC2 5′-ACTCCATCGTGCTTCTTTC-3′). Prior to quantification, the optimal concentrations of template, primers, and magnesium were determined. Serially diluted plasmid DNA samples were used to construct a standard curve to quantify the test samples as well as the amplification efficiency. The products from real-time RT-PCR were also analyzed by agarose gel electrophoresis for single bands of the predicted size (unpublished data). The same protocol was followed for all real-time PCR analysis.

### Analysis of early gene expression.

For the analysis of gene expression during early developmental time points, embryos were collected at the following cleavage and post-cleavage stages: one-cell, four-/eight-cell (mostly four-cell), 64-/128-cell (mostly 64-cell), 4 hpf, and 6 hpf. Total RNA extracted from unfertilized eggs was used as reference RNA in this analysis of gene expression. To perform the real-time PCR using the new set of RNA, total RNA was extracted from one-cell, two-/four-cell, 64-/128-cell, 256-/512-cell, and 4-hpf embryos and used for the experiment. To calculate ratio of expression for the real-time PCR data, real-time data of one-cell stage was used as reference. Normalization statistical analyses were done as described in the Materials and Methods.

## Supporting Information

Dataset S1 Developmentally Regulated Genes with Expression Data This dataset contains all the genes selected for subsequent analysis.
(688 KB DOC)

Dataset S2 Maternal Genes Genes showing maximum transcript levels in unfertilized eggs and different patterns of degradation.
(137 KB DOC)

Dataset S3 List of Genes with Onset of Transcript Accumulation at the Blastula Stage and Peak of Expression at the Blastula Stage (130 KB DOC)

Dataset S4 List of Genes with Onset of Transcript Accumulation at the Blastula Stage and Peak of Expression at the Gastrula Stage (191 KB DOC)

Dataset S5 List of Genes with Onset of Transcript Accumulation at the Blastula Stage and Peak of Expression at the Segmentation Stage (66 KB DOC)

Dataset S6 List of Genes with Onset of Transcript Accumulation at the Blastula Stage and Peak of Expression at the Pharyngula Stage (44 KB DOC)

Dataset S7 List of Genes with Onset of Transcript Accumulation at Gastrula and Peak of Expression at Gastrula Stages (27 KB DOC)

Dataset S8 List of Genes with Onset of Transcript Accumulation at Gastrula and Peak of Expression at Segmentation Stages (51 KB DOC)

Dataset S9 List of Genes with Onset of Transcript Accumulation at Gastrula and Peak of Expression at Pharyngula Stages (29 KB DOC)

Dataset S10 List of Genes with Onset of Transcript and Peak of Expression at the Segmentation Stage (36 KB DOC)

Dataset S11 List of Genes with Onset of Transcript Accumulation at the Segmentation Stage and Peak of Expression at the Pharyngula Stage (88 KB DOC)

Dataset S12 List of Genes with Onset of Transcript Accumulation and Peak of Expression at the Pharyngula Stage (43 KB DOC)

Dataset S13 Genes Exhibiting Peak of Expression in the Unfertilized Egg (Maternally Loaded RNA) (137 KB DOC)

Dataset S14 Genes Exhibiting Peak of Expression at the Blastula Stage (130 KB DOC)

Dataset S15 Genes Exhibiting Peak of Expression at the Gastrula Stage (199 KB DOC)

Dataset S16 Genes Exhibiting Peak of Expression at the Segmentation Stage (142 KB DOC)

Dataset S17 Genes Exhibiting Peak of Expression at the Pharyngula Stage (156 KB DOC)

Dataset S18 Cell-Cycle-Related Genes (21 KB DOC)

Dataset S19 Proteasomes, Ubiquitins (24 KB DOC)

Dataset S20 Somitogenesis-Related Genes (24 KB DOC)

Dataset S21 RP Genes (25 KB DOC)

Dataset S22 Gene Expression Dataset of Pre-MBT and Post-MBT Stages—Maternal Dominant Genes (22 KB DOC)

Dataset S23 Gene Expression Dataset of Pre-MBT and Post-MBT Stages with Onset of Expression from 64 Cells Onwards (39 KB DOC)

Dataset S24 Gene Expression Dataset of Pre-MBT and Post-MBT Stages with Onset of Expression from 4 hpf Onwards (40 KB DOC)

Dataset S25 Gene Expression Dataset of Pre-MBT and Post-MBT Stages with Onset of Expression from 6 hpf Onwards (100 KB DOC)

Dataset S26 Putative Annotation Obtained for the Selected 3,675 Genes Using the Zebrafish Annotation Database Generated in the Work also from the NR DB Blast Search (3.5 MB XLS)

Dataset S27 Putative Annotation Obtained for the Selected Pre-MBT and Post-MBT Genes using the Zebrafish Annotation Database Generated in this Work (525 MB XLS)

Figure S1 Expression Profile of the Beta-Actin Gene in the Array All the copies of the oligos in the array showed an almost identical expression pattern, indicating the reproducibility and homogeneity of the array data.
(410 KB DOC)

Figure S2 Comparison of Expression Profiles Obtained for Myosin Light Chain (mlc2f) and Beta-Actin Genes Using Microarray Analysis and Real-Time PCR The patterns of expression detected in both methods are almost identical.
(204 KB DOC)

Figure S3 Northern Blot Analysis of RP Genes (A) Northern blot analysis to detect the transcript abundance for selected RP genes *(l7a, s3a, s12,* and *s10)* during embryogenesis. The expression pattern obtained by Northern blot analysis is similar to the data obtained from the array analysis.
(B) (I). Intensity of hybridization in the Northern blots for the selected RP gene was digitized and plotted. (II). Expression data obtained from the arrays for the selected RP genes used in Northern blotting are presented. Both analyses show similarity in the pattern of transcript accumulation.
(463 KB DOC)

Figure S4 Distribution of Early Gene Expression at Later Stages of Embryogenesis Based upon Peak Expression and Classification of Early Genes into Functional Groups Based on GO Terminology (227 KB PDF)

Figure S5 Real-Time PCR Analysis of Early Genes Real-time PCR was done using total RNA extracted from early stages of embryonic development (one-cell, two-/four-cell, 64-/128-cell, 256-/512-cell, and 4-hpf) for eight genes from Cluster 2 (pre-MBT transcribed genes). The expression pattern of these genes by real-time PCR and microarray are compared. The expression pattern is similar in both types of analysis.
(182 KB PDF)

Table S1 Comparison of the Expression Profile of Selected Genes in the Microarray Analysis and Other Methods of Transcript Detection (11 KB PDF)

Table S2 Zebrafish Pre-MBT Transcribed (Early Gene) Orthologs in Mouse and Human (9 KB PDF)

Table S3 Characteristics of Developmental Stages of Embryos That Were Collected for the Expression Profile Analysis in this Work (24 KB PDF)

Protocol S1 Onset and Offset Finding Algorithm (71 KB PDF)

### Accession Numbers

The GenBank (http://www.ncbi.nlm.nih.gov/Genbank) accession numbers for the genes and gene products discussed in this paper are alanyl-tRNA synthetase (AI793498), beta-actin (AF025305), *birc5b survivin2* (AY057058), *calreticulin* (BM101539), *catalase* (AF170069), *cathepsin L* (BI865754), *ceruloplasmin* (BC048037), *cyclin B1* (BC055553), *cyclin D1* (BI888360 and BC075743), *cyclinE* (BC045842), *foxc1a* (AF219949), *her1-* (X97329)*, her4-* (AI794276), *her6-* (X97333), *her7-* (AF240772), *her9-* (AF301264), *lmnl3* (AF397015), *mcl1a* (AF302805), *mef2a-* (BI880399, *mef2c-* (U66569), *mef2d-* (U66570), *mespa-* (AB037939), *mespb-* (AB037940), *mlc2f* (AF081462), nascent-polypeptide associated complex alpha (AI626587), RNA helicase (AW154620), *rpl7a-* (AI964218), *rps10-* (BI842921), *rps12-* (BM070699), *rps3a-* (AI558833), *Vsx1* (AF025348), *zp2* (AW170860), *zp2.4* (AF331967), *zp3* (AF095457), and BM184007.

## Abbreviations

EST: expressed sequence tag
GO: Gene Ontology
hpf: hours post-fertilization
MBT: mid-blastula transition
MSP: muscle-specific protein
RP: ribosomal protein
WISH: whole embryo in-situ hybridization
ZGI: Zebrafish Gene Index
