# Supplementary material for: Transcriptome Analysis of Zebrafish Embryogenesis Using Microarrays
Source: PLoS Genet. 2005 Aug 26;1(2):e29. doi: 10.1371/journal.pgen.0010029 (PMC1193535; doi:10.1371/journal.pgen.0010029)
Supplement: Dataset S1 — This dataset contains all the genes selected for subsequent analysis. (688 KB DOC) [file pgen.0010029.sd001.doc]

Dataset S1. Developmentally regulated genes with expression data. This dataset contain all the

genes selected for subsequent analysis.

Genbank ID UF egg 3hpf 4.5hpf 6hpf 7.7hpf 9hpf 10.7hpf 12hpf 15hpf 24hpf 30hpf 48hpf

AA494728 0.905 1.376 1.165 0.795 0.872 0.814 0.468 0.475 0.212 -0.146 -0.509 -0.597

AA494746 -0.547 -0.364 -0.35 0.21 -0.024 0.01 -0.004 -0.209 -0.105 0.118 0.506 0.689

AA494762 0.505 -0.047 0.173 0.56 0.624 0.742 0.684 0.446 0.475 0.064 -0.043 -0.186

AA494787 -0.261 -0.292 -0.099 -0.305 0.354 0.132 0.673 0.8 0.255 0.604 0.833 0.586

AA494837 0.897 0.492 0.584 1.001 0.972 0.092 0.701 1.004 -0.08 0.271 0.165 0.194

AA494842 1.776 1.231 -1.928 -1.956 -1.46 -0.562 -1.724 -1.113 -1.675 -2.426 -2.345 -2.101

AA494997 0.093 1.166 1.246 1.116 2.289 1.522 0.841 1.659 0.223 -0.069 -0.325 -0.485

AA495032 -0.181 -0.012 0.363 0.807 1.041 1.164 0.697 0.495 0.142 0.202 -0.017 0.121

AA495154 0.016 -0.528 0.061 0.713 0.944 0.488 0.231 0.589 0.071 0.145 0.559 1.024

AA495157 0.133 -0.022 -0.092 0.069 -0.001 0.063 0.162 -0.271 -0.23 0.195 0.533 0.274

AA495267 -2.947 -2.885 -0.284 -0.332 0.144 -0.21 -0.444 -0.942 0.653 -0.326 0.395 0.116

AA495418 -0.524 -0.54 -0.283 -1.292 -1.088 -0.06 -0.467 -0.934 -0.543 0.116 0.698 0.689

AA495427 -0.788 -0.757 -0.145 0.004 0.325 1.278 0.596 1.715 0.429 -0.017 -0.293 0.206

AA497144 -0.845 -0.103 -0.057 0.032 -0.285 -0.131 -0.579 -0.558 -0.31 0.891 0.921 0.352

AA497147 -0.129 0.013 0.253 0.51 0.286 0.097 0.106 0.586 0.115 0.175 0.448 0.024

AA497153 0.898 1.285 1.849 1.78 1.427 1.846 0.946 1.186 0.549 0.465 -0.015 -0.013

AA497159 -0.012 0.621 0.133 0.342 0.563 0.228 0.192 1.263 0.121 0.358 0.413 -0.124

AA497205 -0.508 -0.123 0.87 1.577 1.563 0.152 0.435 1.465 0.39 0.438 0.601 0.215

AA497336 -0.276 -0.342 -0.592 -0.083 -1.184 -0.383 -0.12 -0.661 -0.015 0.288 0.33 0.528

AA542593 0.025 -0.185 0.313 -0.335 -0.032 -0.229 -0.043 -0.127 -0.192 -0.112 0.095 0.828

AA605655 -0.728 -0.005 0.143 -0.51 -0.244 -0.416 -0.111 -0.33 -0.406 -0.236 0.008 1.482

AA605677 -3.724 -3.317 -0.595 -0.783 -0.731 1.127 0.383 0.401 0.608 1.526 1.438 1.089

AA605765 0.749 1.146 2.046 1.648 1.7 2.385 1.314 1.738 0.828 0.704 0.182 0.011

AA605842 0.952 1.336 2.382 1.403 1.262 0.922 0.479 0.003 0.408 0.044 0.14 -0.034

AA606010 1.15 -0.39 -0.253 -0.035 -0.155 0.535 0.69 0.879 0.934 1.298 0.572 0.18

AA606013 -0.728 -0.187 0.145 0.5 0.594 0.433 -0.04 1.119 -0.378 -0.552 -0.543 -0.913

AA606054 -2.172 -2.391 -1.001 -1.194 -0.848 -0.495 -0.896 -0.977 -0.583 -0.175 -0.007 0.401

AA606080 -0.476 -0.352 -0.474 -0.089 -0.325 -0.001 -0.225 -0.467 -0.452 0.398 0.713 1.42

AA606085 -0.434 -0.477 0.323 0.384 0.229 0.079 0.359 0.872 0.364 0.994 0.275 0.351

AA606173 -0.869 -3.327 -1.503 -1.121 -0.847 0.487 0.23 0.29 0.372 1.104 0.84 0.393

AA658720 0.137 0.936 0.821 0.012 -0.733 -0.332 -0.568 -0.189 -0.628 -0.729 -0.624 -0.844

AA658743 -0.777 -0.882 -0.138 0.241 0.702 0.328 0.579 1.535 0.358 0.785 0.581 0.128

AA658750 0.041 0.208 0.33 0.477 -0.055 0.158 0.495 0.662 0.061 0.534 0.217 0.036

AA658756 0.667 -0.161 0.094 -0.023 0.183 0.755 0.449 0.226 0.372 0.317 0.132 0.105

AA658759 -2.666 -4.094 -2.319 -1.724 -1.067 -0.272 -0.21 0.079 0.109 0.924 0.728 0.332

AA658796 0.349 -0.742 -0.667 0.168 0.518 1.107 1.077 1.529 0.863 1.476 0.724 0.412

AA658804 1.001 0.081 -0.566 -0.156 -0.958 0.19 0.452 0.54 0.431 0.75 0.171 0.111

AB006104 -0.392 -0.055 -0.35 -0.068 -0.099 0.188 -0.234 -0.017 0.366 0.107 1.15 0.477

AB011826 0.083 0.58 0.583 0.899 0.865 0.125 0.202 0.154 -0.064 0.099 0.182 -0.196

AB017117 0.082 0.723 1.193 1.264 0.747 1.196 0.673 0.649 0.267 0.523 0.127 -0.002

AB017118 -0.545 -1.512 -0.861 -0.704 -0.642 0.443 0.135 0.316 0.294 0.566 0.429 0.616

AB030897 0.787 0.594 0.344 0.056 0.553 0.926 0.276 0.644 0.045 -0.187 -0.358 -0.378

AB030899 -0.131 -1.333 0.113 0.429 0.994 1.176 0.857 0.967 0.45 0.391 0.494 0.16

AB032263 -0.416 0.361 0.039 0.596 0.933 0.617 0.188 1.09 -0.117 0.689 0.233 0.008

AB032265 -1.131 -0.903 -1.384 -1.116 -0.593 -0.015 -0.367 0.571 0.364 0.573 0.419 0.51

AB032415 -0.434 0.201 0.524 0.563 0.55 0.183 -0.264 -0.184 0.234 0.203 0.128 0.109

AB032726 0.896 0.238 0.501 0.47 0.7 1.306 0.689 1.327 0.915 0.619 0.162 -0.406

AB032727 1.857 -0.302 -1.492 -1.571 -3.025 -1.173 -0.679 -2.807 -0.758 -2.328 -1.868 -2.263

AB034245 -0.279 0.263 2.344 1.875 0.737 -0.218 0.546 0.743 0.302 -0.137 0.447 -0.088

AB038320 1.086 -0.008 0.222 0.173 -0.107 0.358 -0.247 -0.446 -0.172 -0.175 -0.475 -0.622

AB040435 0.917 0.251 -0.593 -1.18 -2.03 -1.511 -2.238 -2.456 -3.02 -3.079 -3.34 -3.966

AB045624 -1.486 -1.495 0.571 1.069 1.612 0.87 0.508 0.171 -0.063 -0.449 -0.531 -0.726

AB046866 0.68 1.272 1.588 1.545 1.532 1.395 0.652 1.077 0.266 0.22 0.171 0.586

AB055662 -1.395 -1.305 -0.421 0.387 0.599 1.262 0.688 1.629 0.793 1.006 0.85 -0.02

AB055663 -0.925 -0.955 -1.396 -1.318 -1.08 -0.399 -0.805 -0.881 -0.579 0.124 0.827 0.529

AB055666 -1.046 0.105 -0.64 0.31 -0.316 0.81 0.659 1.662 0.732 0.991 0.796 1.208

AB055671 1.011 1.176 -0.136 0.145 -0.51 -0.15 0.044 0.203 -0.021 -0.249 0.163 -0.28

AB055677 -1.444 -2.002 2.149 3.533 4.187 4.457 3.943 3.753 3.494 1.27 0.464 -0.705

AB055679 -0.016 0.107 0.131 0.493 0.366 0.515 -0.018 0.903 0.448 0.038 0.14 0.085

AB055681 1.126 0.02 -0.07 0.495 0.035 0.188 0.448 0.524 0.158 0.594 0.267 0.035

AF001299 1.224 -1.043 -1.387 -0.922 -0.98 -0.687 -0.607 -0.766 -1.082 -1.304 -1.17 -0.25

AF006831 -0.796 -0.238 -0.671 -0.2 -0.276 -0.376 -0.572 -0.094 -0.303 0.251 0.416 0.668

AF007414 0.524 -1.877 1.262 0.47 0.477 1.456 0.91 0.807 0.949 0.972 0.879 0.077

AF012747 -0.886 -2.264 -1.705 -2.215 -0.854 -0.339 -0.772 -0.733 -0.48 0.647 0.486 0.219

AF014370 -0.64 0.102 -0.449 0.187 -0.097 -0.209 -0.259 0.01 0.055 0.556 0.741 0.083

AF025330 -1.404 -1.682 -0.85 -0.588 -0.842 -0.235 -0.487 0.207 0.375 1.035 0.821 0.383

AF029250 -0.548 -0.766 -1.42 -1.819 -2.053 -0.817 -1.346 -0.756 -0.544 1.261 1.026 1.119

AF030031 -1.495 -1.641 -1.553 -1.041 -0.655 -0.159 0.379 0.113 -0.139 0.331 0.749 0.613

AF030281 -0.668 -0.228 -0.204 -0.567 -0.091 1.184 0.982 2.012 0.803 0.797 0.888 0.781

AF030283 -0.49 -0.014 0.074 0.132 0.328 0.359 0.424 1.649 0.341 0.292 1.011 0.076

AF030284 -0.087 -0.162 0.244 0.055 0.547 -0.212 0.109 0.9 0.052 0.24 0.989 0.358

AF030519 0.123 0.83 1.112 0.655 0.152 0.208 -0.124 0.333 0.2 0.314 0.121 0.009

AF030560 -0.237 0.088 1.435 1.235 1.537 0.666 1.262 2.145 0.688 0.231 0.204 -0.04

AF031378 -0.268 -0.348 -0.122 0.849 1.075 -0.02 0.253 0.442 0.161 -0.056 0.767 0.447

AF032392 0.235 -0.672 -0.855 0.019 0.458 0.342 0.308 1.282 0.319 0.938 0.257 0.16

AF034606 -0.728 0.076 2.492 3.041 2.51 1.905 0.929 2.251 0.723 0.101 0.023 -0.316

AF036148 -0.192 -0.239 -0.24 -0.039 -0.375 -0.167 -0.177 -0.42 -0.463 -0.101 0.063 1.317

AF036149 -0.543 -0.131 -0.273 -0.669 -0.066 0.044 0.297 1.182 0.359 0.991 1.196 0.855

AF038425 -0.638 0.587 1.993 1.417 1.369 0.893 -0.376 0.952 0.489 0.445 0.027 -0.079

AF039410 -0.695 0.411 0.385 0.84 1.276 0.016 0.636 1.182 0.158 -0.113 0.276 -0.315

AF039411 -0.705 -0.496 -0.617 -0.148 0.25 -0.008 0.596 1.573 0.25 0.922 0.662 0.158

AF041440 0.472 0.361 2.184 1.274 0.959 0.785 0.036 0.352 -0.076 -0.072 0.003 -0.223

AF042191 -0.184 -0.353 0.534 1.385 1.698 0.571 0.871 2.281 0.729 -0.179 -0.105 -0.226

AF047837 -4.534 -3.707 -3.381 -3.489 -3.716 -2.618 -3.104 -3.535 -2.718 -1.352 -0.351 0.577

AF052245 -0.116 -0.212 1.348 1.297 2.57 0.785 0.514 1.204 0.119 0.163 0.294 0.106

AF052249 -0.818 -0.338 0.077 -0.194 0.561 0.13 -0.094 0.814 0.194 0.541 0.081 0.074

AF052251 -0.728 0.214 2.368 2.087 3.867 1.415 0.804 1.812 -0.021 -0.416 -0.083 -0.266

AF060118 -0.761 -0.797 -0.555 -0.972 -0.633 0.103 -0.464 -0.201 0.066 -0.24 0.834 0.256

AF060499 0.643 1.249 1.105 0.866 0.787 0.341 0.162 0.811 -0.439 -0.103 0.074 -0.39

AF061252 -0.637 0.223 -0.483 -0.334 -0.429 0.25 0.412 1.095 0.387 0.447 0.773 0.787

AF062643 -0.284 0.202 0.658 0.743 0.637 0.505 0.166 0.346 -0.022 0.087 0.51 0.013

AF064830 0.399 1.211 0.842 0.69 -0.231 -0.448 -0.093 -0.306 -0.126 -0.548 -0.767 -1.147

AF064835 -3.391 -2.993 -0.805 -0.603 -0.7 -1.293 -1.726 -0.682 0.318 0.534 0.119 -0.17

AF064838 0.643 0.903 1.055 0.823 0.39 0.21 0.12 -0.068 -0.079 -0.229 -0.464 -0.483

AF067532 -0.332 -0.29 -0.013 -0.33 -0.07 -0.09 0.358 1.309 0.122 0.128 0.457 0.812

AF068772 -1.603 -1.025 -0.605 0.007 -0.206 -0.05 -0.123 0.162 0.047 0.486 0.004 -0.163

AF068773 -1.865 -1.741 -1.225 -0.055 -0.325 -0.073 0.072 0.165 0.198 1.167 0.202 -0.258

AF071245 -0.798 -0.426 -0.383 -0.262 -0.305 -0.04 -0.512 0.077 0.075 0.114 1.072 0.666

AF071246 -0.837 -0.615 -1.049 -0.62 -0.727 -0.323 -0.558 0.051 0.525 0.432 0.788 0.489

AF071248 -0.341 0.06 -0.074 0.346 0.185 -0.23 0.293 1.46 0.299 0.384 0.365 0.006

AF071255 -0.905 0.093 -0.778 0.244 -0.325 0.26 0.318 1.383 0.516 0.658 0.965 0.502

AF071267 -0.355 0.309 0.512 -0.422 -0.138 0.04 0.257 0.86 0.29 -0.05 1.139 0.185

AF071496 -0.786 -0.035 -0.135 -0.628 0.091 -0.221 -0.181 0.069 -0.019 -0.204 0.966 1.528

AF072456 -0.561 0.019 0.927 0.681 1.537 -0.189 0.353 1.302 -0.153 0.304 0.188 0.199

AF075384 -1.18 0.235 0.404 0.464 0.425 0.839 0.444 0.389 0.634 0.617 0.503 0.253

AF077225 -0.605 0.807 4.683 4.967 4.68 2.241 1.615 3.264 0.636 -0.037 -0.017 -0.295

AF082662 -4.932 -5.147 -4.876 -4.098 -4.939 -3.778 -3.013 -4.62 -4.127 0.208 0.905 2.008

AF083557 -0.778 -2.324 -0.694 -1.19 -1.518 -0.846 -0.526 -1.55 -0.692 0.32 0.35 -0.026

AF084948 -0.226 -0.469 -0.34 -0.118 -0.031 -0.113 -0.198 0.694 -0.035 0.194 -0.126 -0.093

AF093129 -1.54 -1.446 0.378 0.752 0.834 1.562 1.1 0.85 0.377 0.503 0.231 0.039

AF095457 1.221 -2.998 -2.489 -3.206 -4.066 -3.554 -4.045 -5.04 -3.945 -4.241 -6.51 -5.517

AF095639 -0.623 -0.384 -0.403 -0.285 -0.165 -0.238 -0.213 0.233 -0.09 0.92 1.242 -0.005

AF097478 -0.025 0.727 0.721 0.679 0.298 -0.42 0.015 0.217 -0.098 0.04 -0.31 -0.311

AF097483 -0.384 1.324 1.059 1.136 -0.466 -0.171 -0.297 -0.008 -0.26 -0.662 -0.522 -0.44

AF097875 1.095 0.213 -1.754 -1.652 -2.092 -1.451 -1.613 -1.79 -1.245 -0.967 -1.191 -1.683

AF101266 0.902 -0.047 -0.45 -0.333 -0.682 0.249 -0.224 0.984 0.732 0.848 1.17 0.109

AF102865 -2.487 -2.252 -1.72 -1.622 -1.635 -1.096 -0.397 -0.975 -1.315 1.083 0.289 0.515

AF108819 -0.383 -0.261 0.243 0.665 0.508 0.7 0.375 0.695 0.281 0.493 0.818 0.623

AF114262 -1.583 -1.935 -1.168 -0.925 -1.669 -0.838 -0.821 -0.889 -0.348 -0.797 -0.31 0.649

AF115774 -0.801 -0.447 -0.72 -0.429 0.379 -0.146 0.224 0.524 0.27 0.723 0.705 0.117

AF121796 -2.395 -1.666 -2.489 -2.316 -1.773 -1.2 -1.064 -1.928 -2.096 -0.057 0.326 1.466

AF124095 -1.012 -0.48 -0.755 -0.016 -0.305 -0.028 -0.306 0.271 0.352 0.77 0.74 0.359

AF124332 -0.139 0.113 0.599 0.453 0.896 0.038 0.249 0.739 0.04 0.088 -0.151 -0.161

AF124396 -0.369 -0.12 0.494 0.799 0.75 0.38 0.329 0.678 0.139 0.258 0.455 0.172

AF124435 0.869 1.072 0.046 -0.31 -0.811 -0.108 -0.436 -0.414 -0.913 -0.625 -0.417 -0.507

AF127920 0.788 1.662 1.632 1.342 0.499 0.893 -0.042 0.46 -0.549 -0.313 -0.883 -1.224

AF127981 -0.958 -0.046 -0.805 -0.918 -0.366 0.211 -0.069 0.373 -0.399 1.024 1.016 0.868

AF130459 0.289 0.842 1.003 0.424 0.263 0.788 0.144 0.03 0.001 -0.242 -0.248 -0.013

AF132445 -0.272 0.398 0.602 0.77 1.093 0.46 0.076 0.642 -0.003 0.186 0.342 -0.019

AF134850 -6.382 -5.38 -1.241 -0.434 -0.066 0.822 0.451 0.66 0.58 1.266 0.871 0.136

AF134852 -1.041 -4.135 -2.07 -1.404 -1.052 0.269 -0.205 0.105 0.178 1.069 0.826 0.299

AF135438 -0.29 0.726 1.255 1.48 1.326 0.28 -0.144 1.137 0.438 0.387 -0.051 -0.181

AF137535 0.459 0.735 1.14 0.771 0.545 0.089 -0.298 0.223 -0.055 -0.398 -0.352 -0.747

AF139990 1.639 1.578 1.918 1.939 1.601 1.011 0.602 0.876 0.101 -0.02 -0.364 -0.807

AF140608 -0.054 0.063 0.275 1.049 1.012 0.39 0.447 0.915 0.135 0.612 0.499 0.233

AF143493 -0.013 -0.285 0.104 -0.093 0.029 0.255 0.447 1.058 0.43 0.858 0.295 0.039

AF149720 2.107 -1.396 -1.147 -1.066 -1.496 -0.845 -0.921 -1.455 -0.778 -1.451 -1.122 -1.356

AF149802 -2.361 -2.012 -2.327 -1.351 -2.433 -0.921 -1.253 -1.481 -0.171 1.679 1.269 0.639

AF150107 0.675 0.144 0.212 0.564 0.589 0.839 0.138 0.926 0.075 0.919 0.297 -0.073

AF151535 -1.002 -0.486 0.271 0.802 1.512 2.165 1.236 1.618 0.843 0.878 1.336 0.856

AF155578 0.313 -0.514 0.39 0.13 0.558 0.931 0.461 1.235 0.989 1.446 0.664 0.47

AF155581 0.429 -0.426 0.606 -0.316 1.309 1.134 0.563 1.773 0.732 1.564 0.632 0.468

AF157109 -1.102 1.007 2.918 1.854 2.338 0.978 0.095 1.098 -0.088 0.816 0.294 0.49

AF157110 -0.522 -0.377 0.855 1.995 2.574 1.429 1.305 2.254 0.628 -0.123 -0.133 -0.387

AF157560 -0.912 -0.376 -0.576 -0.04 -0.673 -0.25 0.118 -0.272 -0.12 0.869 0.852 0.419

AF159135 -0.161 0.308 0.812 0.545 1.188 -0.241 0.101 0.851 -0.073 -0.039 -0.018 -0.006

AF160635 -0.537 -0.251 0.316 0.554 0.621 0.788 1.004 0.862 0.694 0.609 0.365 0.266

AF160646 -0.299 -0.131 0.385 0.697 1.293 1.662 0.914 1.302 1.022 0.924 0.603 0.087

AF160659 -1.165 -0.489 0.023 0.785 0.753 0.326 0.069 0.624 0.318 0.147 0.352 0.161

AF160683 -0.938 -0.639 -0.579 0.327 0.29 0.437 0.116 0.877 0.4 0.205 -0.148 -0.155

AF161270 0.001 0.522 0.667 0.309 0.305 0.057 0.093 0.477 0.541 -0.004 -0.006 -0.468

AF162696 -0.76 -0.417 0.218 0.299 0.412 0.356 0.468 0.214 0.738 0.699 0.679 0.363

AF164477 1.413 1.219 0.458 0.341 0.651 0.351 0.221 0.551 0.243 0.195 -0.049 -0.639

AF164483 0.265 0.294 1.3 0.84 1.41 1.083 0.877 1.17 0.339 0.345 -0.269 -0.122

AF164726 0.022 0.02 0.534 0.502 0.825 0.049 0.251 0.177 -0.179 -0.262 -0.622 -0.651

AF168007 -0.304 0.263 1.977 2.021 2.285 1.291 0.849 2.239 0.503 0.632 0.527 0.299

AF168008 -0.308 0.116 0.876 1.313 1.504 1.804 0.889 1.415 0.377 0.404 0.515 0.261

AF168614 -0.314 0.874 0.981 1.126 1.105 0.882 0.512 0.686 0.375 0.042 -0.362 -0.728

AF169146 0.215 0.419 0.692 0.782 0.635 0.85 0.182 0.211 0.034 0.058 0.223 0.002

AF170069 1.109 -0.667 -1.106 -1.293 -2.115 -1.924 -1.105 -3.109 -2.77 -2.591 -2.384 -1.833

AF172089 -0.155 0.202 0.631 1.057 1.514 -0.151 0.447 1.453 -0.137 -0.169 0.66 -0.259

AF173984 -2.697 -2.181 -3.052 -1.907 -2.252 -1.324 -1.223 -1.795 -1.332 0.041 -0.576 1.144

AF175294 -1.885 -0.783 -1.672 -0.669 -0.841 -0.191 0.083 -0.462 0.345 0.961 0.941 -0.081

AF176316 1.163 0.289 -0.082 0.253 0.082 -0.356 -0.439 -0.709 -0.541 -1.129 -1.096 -0.92

AF177868 -0.136 0.466 0.868 1.318 0.615 1.058 0.538 0.727 0.537 0.238 0.179 0.272

AF177869 -0.261 0.375 0.536 0.329 0.056 0.543 0.376 0.783 0.577 0.051 0.253 -0.04

AF180889 -2.938 -2.542 -1.676 -0.694 -3.392 -2.131 -1.994 -2.811 -2.393 -0.201 0.778 1.283

AF180890 -2.656 -6.312 -1.701 -4.795 -5.838 -2.243 -2.459 -5.819 -2.443 -0.401 -0.283 0.891

AF180892 -6.37 -6.683 -5.006 -5.412 -6.568 -5.087 -6.354 -6.553 -4.62 -1.118 -0.464 0.707

AF180921 -0.519 -2.559 -1.147 -1.693 -2.319 0.183 0.319 0.716 0.576 0.779 -0.157 -1.305

AF184244 -0.061 0.885 2.183 1.788 1.437 1.317 1.156 1.633 0.8 0.815 0.223 0.068

AF184245 -0.307 -0.157 -0.004 0.143 0.742 0.353 0.475 1.435 0.402 0.764 0.143 0.369

AF190144 -0.978 -0.948 -1.346 -0.545 -1.109 -0.306 -0.448 -0.474 -0.263 0.269 1.169 0.445

AF191559 -1.181 -2.258 -1.867 -1.843 -0.516 -0.426 -0.489 -0.469 -0.786 0.572 0.508 0.358

AF191561 0.892 0.25 0.319 0.337 0.068 0.524 0.141 0.611 0.226 0.519 -0.133 -0.092

AF191578 -0.572 0.032 1.052 2.112 2.705 0.244 0.468 1.513 0.46 0.187 0.22 0.015

AF193837 -0.796 1.757 4.28 3.725 3.714 2.698 2.596 2.427 1.815 -0.419 0.163 -0.243

AF195050 0.33 0.929 0.93 0.579 0.403 0.088 0.438 0.365 -0.034 -0.059 -0.527 -0.631

AF195881 -0.784 -0.034 0.315 0.422 0.145 -0.023 0.055 0.351 0.652 1.341 0.448 0.214

AF196346 -0.133 0.74 0.778 0.712 0.175 0.224 -0.353 0.334 0.057 0.001 -0.172 -0.187

AF197880 -4.428 -3.512 -0.999 0.126 0.276 0.993 0.961 0.606 0.849 1.512 0.519 -0.012

AF200950 -0.381 -0.19 0.329 0.669 0.266 0.25 0.189 0.783 0.699 0.353 0.556 -0.118

AF201379 -0.728 0.249 2.5 1.948 1.345 0.289 -0.219 0.772 -0.314 -0.66 -0.286 -0.666

AF202054 0.13 -0.536 -0.569 -0.264 -0.092 0.16 0.284 1.028 0.177 0.464 0.083 -0.08

AF202639 -0.613 -0.003 -0.32 -0.664 -0.238 0.185 -0.287 0.175 0.472 1.872 1.163 0.51

AF204240 -0.779 -0.236 -0.154 -0.964 -0.678 -0.392 -0.489 -0.447 -0.36 0.017 0.903 1.621

AF204241 -0.27 0.966 0.228 0.109 0.135 -0.215 0.129 0.623 0.242 -0.124 0.64 -0.123

AF207751 -0.462 0.804 4.331 4.013 3.902 2.737 1.996 3.268 1.29 1.233 1.19 0.352

AF210320 -0.766 -0.533 -0.787 -1.15 -1.077 -0.153 -0.352 -0.318 0.103 0.486 0.903 0.702

AF210637 -0.817 -0.619 0.045 0.632 0.741 0.103 0.219 -0.06 0.481 0.328 -0.015 -0.148

AF210638 -0.371 0.095 0.697 0.318 0.362 0.792 0.313 0.554 0.287 0.691 0.33 0.086

AF210641 -0.601 -1.358 -0.484 -0.13 -0.566 0.468 0.042 0.312 0.07 0.852 0.697 0.087

AF210645 0.457 0.801 0.59 0.761 1.076 0.24 0.548 1.339 0.29 0.429 0.346 -0.131

AF210646 -0.043 0.54 -0.018 0.754 0.563 -0.3 0.069 0.333 -0.092 0.036 -0.026 -0.144

AF211852 0.535 0.542 2.347 2.444 2.201 1.979 1.774 1.813 0.951 0.925 0.525 0.287

AF212941 0.145 1.433 1.728 1.306 0.464 -0.28 -0.282 -0.132 -0.343 -0.436 -0.464 -0.881

AF219949 -0.195 0.389 0.407 0.433 2.351 1.238 1.071 2.132 0.41 -0.469 1.171 0.451

AF222995 -0.711 -0.268 0.179 -0.978 -0.193 0.351 0.619 1.84 0.337 0.551 0.739 0.861

AF222996 -1.021 -0.438 0.325 0.806 1.402 1.757 1.288 1.089 0.955 0.837 0.542 0.704

AF231015 -0.31 0.895 0.459 0.552 0.208 0.326 0.056 0.866 0.206 0.049 0.178 -0.182

AF234784 0.81 1.692 2.084 1.562 0.464 0.17 -0.465 -0.333 -0.806 -0.907 -1.084 -1.411

AF237712 -1.784 -1.984 -1.481 -0.544 -1.188 -0.671 -0.61 -1.588 -1.102 0.066 0.255 1.869

AF239925 -1.014 -0.124 2.182 1.543 1.62 1.576 0.569 0.975 0.234 0.629 0.412 0.579

AF240772 -0.382 0.015 0.807 1.661 1.515 0.753 0.684 1.179 0.527 -0.004 0.594 0.138

AF246162 -0.685 -0.707 1.11 2.543 3.85 0.879 1.507 1.991 1.235 -0.041 -0.063 -0.295

AF246184 -0.459 -0.408 -0.091 0.486 0.46 -0.09 0.235 0.061 0.036 -0.157 -0.106 -0.118

AF250368 -0.451 -1.192 -1.136 -0.614 -0.451 0.162 -0.055 0.562 0 0.793 0.522 0.24

AF252546 -0.053 -0.333 -0.309 -0.183 0.212 0.56 0.714 1.074 0.621 0.233 0.198 -0.191

AF254955 -0.186 0.453 0.48 0.347 1.183 -0.05 0.637 1.061 0.239 -0.682 -0.514 -0.608

AF255044 -0.371 0.262 0.783 1.146 1.548 -0.188 0.28 1.75 0.25 -0.007 0.072 -0.027

AF257519 -0.154 -0.169 -0.168 0.507 0.914 0.658 0.413 0.961 0.053 0.316 -0.035 -0.274

AF257743 -0.082 -0.921 -1.051 -0.775 -0.778 0.705 0.394 1.372 0.901 1.828 0.974 0.173

AF258786 0.62 0.941 0.474 0.551 0.983 1.272 0.298 0.896 0.239 -0.318 -0.409 -0.388

AF259079 0.545 0.932 1.834 1.395 1.757 1.042 1.132 1.541 0.489 0.889 0.62 -0.202

AF260240 -0.447 0.631 1.263 0.615 -0.477 -0.543 -0.138 -0.135 -0.077 -0.698 -0.651 -0.884

AF262047 0.807 -0.201 -0.234 -0.248 -0.158 0.231 0.065 0.27 0.128 -0.263 0.09 -0.422

AF262978 -0.293 0.998 2.602 2.105 1.805 0.57 0.658 1.481 0.2 0.314 0.186 -0.193

AF263000 0.112 1.685 2.116 2.205 0.315 -0.371 -0.151 0.49 -0.277 -0.59 0.13 -0.201

AF270789 -0.768 0.025 0.009 0.201 0.555 0.715 0.369 2.49 0.706 -0.086 0.024 -0.31

AF272962 -0.451 -0.033 -0.153 -0.166 0.03 0.159 -0.578 0.657 0.541 0.215 0.862 0.415

AF274877 0.095 0.558 0.122 0.587 0.677 1.137 0.273 0.804 0.228 0.401 0.338 0.127

AF277097 -0.608 -0.04 -0.143 -0.226 0.158 0.191 0.145 0.117 0.111 0.083 0.727 0.551

AF277172 0.802 0.433 0.164 0.264 0.188 0.836 0.387 0.146 0.528 0.437 0.458 0.405

AF280090 -0.907 -0.473 -0.864 -0.522 -0.757 -0.287 -0.747 -0.727 -0.202 0.24 1.04 1.187

AF281003 -2.203 -2.458 -2.203 -1.488 -2.283 -1.568 -1.738 -1.873 -1.876 -0.125 0.271 0.602

AF286364 -0.596 -0.075 0.967 1.782 1.228 0.245 0.227 0.516 0.605 -0.278 0.827 -0.003

AF286374 -0.968 0.166 -0.691 -0.765 -0.405 -0.017 -0.389 -0.167 -0.868 0.674 0.845 0.986

AF286375 -1.659 -2.38 0.139 0.622 0.526 0.043 -0.247 -0.318 -0.421 -0.229 -0.353 -0.213

AF288217 -1.691 -2.012 -0.517 -0.286 -0.333 0.553 0.171 0.485 0.175 0.563 0.244 -0.125

AF288409 2.002 -0.708 -1.194 -0.813 -0.018 -0.431 -0.948 -0.336 -0.74 -1.505 -0.865 -0.964

AF290981 -0.434 0.509 1.624 1.355 1.547 1.303 0.73 0.827 0.36 0.706 -0.262 -0.43

AF295373 0.546 -0.135 0.76 0.722 0.509 1.227 0.575 0.477 0.186 0.288 -0.117 -0.515

AF295377 -1.073 -3.867 -1.78 -2.067 -1.968 -0.506 -0.701 -0.981 -0.004 0.731 0.807 0.134

AF295804 -0.341 0.332 -0.702 0 -0.059 0.071 -0.166 0.728 -0.108 0.454 0.236 0.048

AF302805 1.821 -0.08 -0.639 -0.524 -1.126 -0.395 -1.195 -1.136 -1.623 -1.82 -1.693 -1.706

AF315945 -0.717 -1.069 -1.43 -1.13 -0.521 -0.314 0.202 0.504 0.169 0.919 0.605 0.569

AF321194 -0.879 -0.611 -0.648 -0.711 -0.925 -0.125 -0.312 -0.412 -0.204 0.332 0.861 1.31

AF331967 1.749 -3.198 -2.422 -2.993 -3.42 -2.84 -3.009 -3.908 -3.619 -4.677 -4.381 -4.744

AF332623 -0.353 0.164 -0.041 0.224 0.169 0.177 0.117 0.456 0.21 0.397 0.19 0.071

AF332983 0.046 0.352 1.238 1.049 1.295 1.073 0.448 0.412 -0.261 0.192 -0.049 -0.624

AF336123 -1.57 -0.396 0.263 1.712 2.381 1.641 1.859 2.302 1.093 1.564 1.231 0.662

AF337035 -0.722 -0.503 0.686 0.739 0.448 1.055 0.587 0.151 0.274 0.449 0.274 0.834

AF348959 -0.604 0.237 -0.677 -0.256 -0.354 0.338 0.029 0.537 0.491 0.317 -0.113 -0.066

AF354750 -0.023 -0.284 0.184 0.206 0.451 0.198 0.372 0.968 0.506 0.8 0.341 0.12

AF354754 -0.329 0.69 1.428 1.844 1.929 0.808 0.183 1.064 0.376 0.205 0.769 0.076

AF359242 0.709 0.588 1.74 0.887 0.648 1.013 0.309 0.346 -0.073 -0.134 -0.174 -0.126

AF359424 -0.764 -0.125 1.203 1.285 1.086 0.495 0.352 0.713 0.075 0.048 0.386 0.002

AF359425 -0.992 0.095 1.926 1.36 1.619 1.774 1.296 1.245 0.265 0.406 0.362 -0.302

AF359429 -0.643 0.096 -0.074 0.006 -0.031 0.259 -0.257 0.716 0.699 0.642 0.552 0.787

AF361478 -0.037 0.524 0.601 0.764 0.631 0.857 0.246 0.954 0.331 0.758 0.567 0.04

AF364083 -0.811 -0.549 -0.023 -0.339 -0.923 -0.125 -0.656 -0.812 -0.121 -0.138 0.515 0.927

AF364084 1.062 -0.065 -0.386 -0.193 -0.412 0.158 0.382 0.507 0.271 0.529 0.146 -0.428

AF364085 0.055 0.302 0.883 1.221 1.755 1.378 0.816 1.584 0.556 0.82 0.21 -0.512

AF369382 -0.763 -0.006 -0.131 0.629 0.595 0.736 0.552 1.467 -0.304 0.657 0.708 0.574

AF371368 -0.696 -0.669 0.619 0.478 0.593 0.421 0.748 1.447 0.733 0.497 0.91 0.167

AF375226 -0.185 0.188 0.395 1.056 1.037 -0.039 0.364 0.808 0.131 0.035 -0.108 0.005

AF375227 -1.038 -0.328 -0.643 -0.715 -0.69 -0.152 -0.372 -0.496 0.257 0.406 0.661 0.59

AF375871 -0.508 -1.287 -1.201 -0.967 -1.404 0.093 0.171 0.264 0.427 0.633 0.754 0.457

AF384863 -0.487 0.225 -0.084 0.125 0.457 0.09 0.264 0.336 -0.137 -0.064 0.729 -0.092

AF387341 0.965 0.333 -0.478 -0.31 -0.384 -0.186 -0.053 -0.055 -0.202 0.003 0.106 0.119

AF387342 -0.103 0.646 0.609 0.746 0.591 0.147 0.347 0.341 0.181 -0.206 -0.221 -0.887

AF387820 1.449 1.061 -0.839 -0.632 -0.901 -0.232 -0.724 -0.561 -1.061 -1.166 -0.812 -1.02

AF388363 -0.588 -0.037 0.536 1.212 1.899 0.826 0.534 1.494 0.15 0.044 0.729 -0.073

AF389401 -1.942 -0.713 -1.278 -0.59 -0.489 -0.051 -0.602 0.246 0.154 0.519 0.208 0.69

AF395113 -0.6 -0.524 -0.397 0.002 0.366 0.461 0.413 0.778 0.283 0.033 0.597 0.003

AF395831 -0.942 -0.211 -0.385 -0.621 -0.059 -0.037 -0.402 -0.283 -0.291 0.076 0.238 0.848

AF397015 0.863 0.741 -0.132 -0.122 -0.33 -0.263 -0.677 -0.463 -0.457 -0.632 -0.612 -0.698

AF398433 -0.455 0.236 0.643 0.601 1.95 2.425 1.344 1.659 1.19 0.167 0.637 0.096

AF414110 1.6 1.424 1.844 1.89 1.164 1.489 1.107 1.006 0.102 0.158 -0.195 -0.099

AF423762 -0.142 -0.795 0.677 0.921 1.334 1.573 1.501 1.662 1.391 1.102 0.797 0.828

AF426384 -0.75 0.026 0.392 1.604 1.737 1.203 0.569 1.317 0.163 0.606 0.979 0.166

AF428249 -0.922 0.003 0.306 0.538 1.021 -0.032 0.067 0.877 0.416 0.348 0.045 0.181

AF430840 -0.819 -0.481 0.028 0.266 0.057 0.05 -0.044 0.143 -0.21 -0.587 -0.029 0.913

AF448057 -0.219 -0.907 -0.76 -0.383 -2.167 -0.98 -1.017 -2.255 -0.785 0.807 0.832 0.182

AI106133 -0.516 -1.723 -0.777 -0.373 -0.788 0.497 0.273 0.7 0.296 1.187 0.581 -0.44

AI106421 -0.405 -2.679 -0.973 -0.895 -0.423 0.048 0.4 0.622 0.317 1.361 0.739 0.218

AI322210 0.442 -0.646 1.248 0.241 0.894 1.7 1.392 0.795 0.879 0.557 0.334 -0.499

AI330707 0.118 -0.108 -0.728 -0.276 0.377 0.156 0.211 1.068 0.26 0.647 0.376 0.078

AI330805 -1.018 -1.527 -1.655 -1.051 -1.536 -0.675 -0.455 -0.947 -0.037 0.043 1.056 0.06

AI330980 0.04 -0.956 0.079 0.091 0.229 0.475 0.678 1.175 0.362 0.939 0.873 0.564

AI331043 -1.565 -1.236 -0.708 -0.075 -0.076 0.568 0.586 0.307 0.324 0.26 0.335 -0.232

AI331055 -0.388 -0.482 -0.354 -0.336 -0.383 0.581 -0.022 0.659 0.334 0.907 0.43 0.36

AI331515 -1.071 -1.318 -1.511 -0.609 -0.23 0.267 0.346 0.899 0.579 0.513 -0.479 -0.35

AI331606 -0.26 -1.349 -0.509 -0.978 -1.974 0.076 -0.714 -1.826 -0.757 -0.416 -0.337 1.483

AI331812 -0.575 -0.348 1.008 0.453 1.013 0.733 0.209 0.254 0.187 0.126 -0.036 0.227

AI331953 0.488 0.324 -0.25 -0.25 0.06 0.346 0.303 1.086 0.233 0.88 0.162 0.246

AI353083 -4.956 -4.135 -4.296 -2.727 -4.102 -3.193 -3.663 -4.553 -3.654 0.35 1.008 2.248

AI353168 -1.17 -3.123 -1.685 -0.944 -0.667 0.354 0.063 0.253 0.299 0.877 0.585 -0.313

AI353394 0.508 -0.983 -0.048 0.133 0.391 1.198 0.557 1.057 0.416 1.295 0.863 1.017

AI353412 -1.573 -0.006 3.87 3.542 3.997 4.521 4.403 4.471 3.266 1.757 1.5 0.192

AI353581 -0.529 -0.212 -0.448 -0.527 -0.435 -0.091 -0.588 -0.592 -0.122 -0.102 0.039 1.413

AI354033 -0.406 -0.046 -0.066 0.025 -0.083 -0.252 0.121 0.233 0.192 0.09 0.428 0.112

AI354177 -0.336 0.27 0.211 0.358 0.664 1.093 0.646 0.947 0.325 0.216 -0.043 0.038

AI384140 -0.669 -0.801 -0.083 0.388 0.933 0.919 0.6 1.437 0.506 0.587 0.49 0.15

AI384221 -0.222 -0.472 1.555 0.331 0.7 0.25 0.13 0.828 -0.031 0.082 -0.067 0.24

AI384355 -1.279 -3.67 -1.778 -1.335 -0.982 0.264 -0.028 0.217 0.234 1.146 0.997 0.597

AI384393 -1.225 0.136 -0.716 -0.388 -1.029 0.07 -0.496 -0.771 -0.101 0.493 0.426 0.739

AI384655 -1.691 -1.564 -1.034 -0.488 -0.433 -0.418 -0.532 -1.088 0.038 -0.538 0.253 0.463

AI384860 1.806 0.742 -0.612 -0.065 0.251 0.832 0.114 0.018 -0.389 -0.719 -0.762 -0.891

AI385123 -0.453 -0.376 0.432 0.406 1.204 1.823 1.231 1.413 1.259 0.395 0.439 0.247

AI396677 0.165 -0.381 0.503 0.469 1.603 1.706 1.653 2.001 0.752 0.435 0.279 0.081

AI396688 0.536 1.344 1.175 1.173 0.422 0.086 0.129 0.228 0.242 -0.085 -0.16 -0.643

AI396886 -0.605 0.756 0.957 0.94 0.726 -0.098 -0.164 0.544 0.081 0.034 -0.198 -0.367

AI397023 -0.387 -0.111 -0.141 0.058 0.484 0.303 0.265 0.886 0.336 0.445 0.629 0.44

AI397274 -0.373 -0.746 -1.164 -0.981 -0.778 -0.371 -0.683 -0.687 -0.447 0.54 0.617 1.327

AI397333 1.109 0.61 0.607 0.501 0.011 0.307 0.04 -0.062 -0.204 -0.036 -0.261 -0.342

AI397462 -0.728 -0.201 -0.552 -0.216 -0.161 -0.137 -0.366 0.039 0.646 1.536 0.641 0.034

AI415831 -0.826 0.079 0.066 0.566 0.945 0.294 0.144 0.885 0.293 0.123 0.076 -0.243

AI415871 0.282 0.921 0.922 0.662 0.063 -0.216 0.033 0.008 -0.212 0.015 -0.081 -0.203

AI415997 0.073 -0.259 -0.303 0.179 0.277 0.174 0.069 0.648 0.116 0.228 0.081 0.238

AI416043 0.756 0.493 0.026 0.069 -0.016 0.21 0.294 0.277 0.049 -0.082 -0.275 0.052

AI416128 -0.248 -0.285 0.115 0.198 0.524 0.178 0.413 0.819 0.445 0.495 0.036 -0.106

AI416261 -1.02 -0.431 0.521 1.226 1.196 0.436 0.383 0.878 0.338 -0.039 0.046 -0.145

AI416338 0.308 0.37 1.191 1.312 1.297 1.639 1.251 1.06 0.708 0.346 0.058 -0.289

AI436892 1.185 1.345 1.037 1.056 0.13 0.09 -0.16 -0.641 -0.551 -0.398 -1.064 -0.507

AI437156 0.241 1.11 1.123 0.715 0.416 0.806 0.066 0.232 -0.235 0.094 -0.211 -0.225

AI437239 -0.021 -1.183 -0.31 -0.336 0.16 0.495 0.137 0.794 0.148 0.536 0.326 0.187

AI437329 -0.952 -1.467 -1.101 -0.624 -0.446 -0.484 -0.239 -1.054 -0.121 -0.015 0.469 0.3

AI437364 0.286 0.159 0.361 0.833 0.938 0.625 0.576 1.125 0.41 0.32 0.606 0.1

AI437388 -0.494 -0.04 0.003 0.336 0.806 0.104 0.17 0.668 0.127 0.204 0.178 -0.033

AI437402 -0.165 -0.364 0.127 0.385 0.528 0.418 0.115 0.732 0.042 0.15 0.12 -0.016

AI437428 -0.247 0.539 1.858 1.632 2.081 1.957 1.738 1.096 0.661 0.466 -0.105 -0.208

AI437466 -0.387 -0.621 -0.058 -0.887 0.112 0.216 0.328 1.015 0.692 0.34 0.449 0.426

AI444184 -0.169 -0.222 -0.361 0.482 0.13 -0.029 -0.183 0.654 0.085 0.587 0.325 0.246

AI444200 1.457 0.583 0.551 0.869 0.095 -0.151 -0.037 -0.166 -0.193 -0.585 -0.122 -0.654

AI444293 -0.397 -0.351 0.39 0.397 -0.069 0.651 0.29 0.393 0.312 0.742 0.345 0.453

AI444335 -0.528 0.96 0.194 0.439 0.535 0.09 -0.022 0.335 -0.182 -0.059 -0.182 -0.109

AI444338 0.784 0.757 0.782 0.902 0.445 1.413 0.53 1.087 0.92 1.205 0.696 0.236

AI444340 0.335 0.841 -0.512 -0.384 0.819 1.035 0.68 1.139 0.347 -0.348 -0.06 -0.146

AI444432 -0.728 0.545 0.124 -0.339 -0.055 -0.125 -0.428 0.193 -0.332 0.005 0.05 1.006

AI444433 0.263 0.045 0.322 0.243 0.228 0.022 0.328 -0.113 0.181 0.143 0.484 0.892

AI461323 -0.974 -0.223 -0.573 -0.678 -0.395 -0.034 -0.307 -0.07 0.076 0.576 0.698 0.728

AI461367 -0.352 -0.114 0.283 0.108 1.076 0.493 0.188 1.131 0.361 0.796 0.867 0.709

AI461372 -2.624 -1.519 -0.695 -0.453 0.061 -0.085 -0.633 -0.366 0.678 -0.228 0.483 0.375

AI476925 0.247 0.27 0.639 1.368 1.639 0.291 0.774 0.845 0.045 0.135 -0.19 -0.17

AI476945 -0.153 -0.091 0.317 0.17 0.98 -0.018 -0.117 0.544 -0.005 -0.103 0.598 -0.21

AI477017 1.506 -0.515 0.621 1.004 1.389 1.994 1.16 1.452 0.888 0.757 -0.007 -0.564

AI477020 -0.543 -0.211 -0.702 -0.88 -0.565 0.149 -0.243 0.84 0.548 0.428 0.446 1.106

AI477041 -0.327 -2.059 -0.292 0.216 0.284 0.507 0.696 1.021 0.699 1.109 0.578 -0.317

AI477049 1.156 1.11 0.066 -0.2 -1.625 -0.487 -0.529 -0.626 -0.669 -0.818 -0.966 -1.244

AI477247 0.545 0.955 0.938 1.03 0.438 0.031 0.137 0.404 0.128 0.049 -0.452 -0.538

AI477274 -2.032 -1.834 -2.202 -0.937 -2.806 -0.883 -0.898 -1.154 -0.095 1.615 0.624 0.823

AI477285 0.628 0.641 1.044 0.961 0.821 0.41 0.789 0.53 0.612 0.025 -0.661 -0.588

AI477286 -0.158 -0.096 0.154 0.555 0.687 1.258 1.211 1.411 1.169 1.064 0.472 0.312

AI477305 -0.535 -2.694 -0.681 -0.215 0.28 0.859 1.005 1.103 0.507 0.807 0.362 0.08

AI477343 -0.045 0.01 -0.442 -1.177 -1.693 -0.553 -0.404 -0.747 -0.651 0.065 0.082 -0.226

AI477417 -0.728 0.173 1.274 1.391 1.552 0.749 0.812 1.279 0.198 -0.272 0.142 -0.143

AI477419 -0.495 -0.332 0.882 1.233 1.83 0.344 0.614 1.522 0.73 0.902 0.466 0.056

AI477424 0.691 -0.221 -0.071 0.057 0.64 0.92 0.401 1.207 0.367 0.707 0.506 0.251

AI477432 0.338 0.127 0.755 0.766 0.774 1.349 0.993 1.105 0.715 0.406 0.307 0.257

AI477493 0.18 -0.213 0.028 0.063 0.196 0.419 0.802 1.331 0.691 1.281 0.609 0.005

AI477656 -2.837 -2.541 -2.726 -2.208 -2.339 -1.241 -1.199 -2.91 -1.675 -0.838 0.67 1.67

AI477935 -0.542 -0.004 1.268 2.229 3.722 4.366 4.045 4.581 3.747 1.287 -0.149 -0.687

AI477949 0.08 1.131 1.806 1.987 2.348 1.944 1.782 2.498 0.84 0.783 0.082 -0.156

AI477962 0.623 1.065 1.93 1.953 2.226 1.983 1.718 1.384 0.266 0.754 0.195 0.338

AI477963 -0.728 -0.059 0.342 1.852 2.316 0.605 0.823 0.786 0.205 0.459 1.126 0.408

AI477969 -0.728 1.013 2.31 2.843 1.995 0.108 -0.083 0.553 -0.378 -0.851 -0.652 -0.636

AI478002 -0.677 -1.323 -1.58 -0.586 -0.107 -0.392 -0.357 1.118 0.836 1.606 0.535 -0.04

AI496761 -0.621 -0.01 -0.239 -0.082 0.45 0.052 0.022 0.919 0.08 0.036 0.055 -0.229

AI496943 -1.23 -0.709 -0.039 0.199 0.472 1.339 1.013 1.592 1.061 1.124 1.151 1.58

AI497075 -0.088 0.586 0.724 0.535 0.26 0.087 0.019 0.274 -0.072 -0.101 -0.299 -0.511

AI497192 -0.397 0.109 0.183 0.478 0.585 -0.141 -0.116 0.277 0.055 0.214 0.152 -0.2

AI497232 2.107 -2.202 -1.519 -1.5 -2.699 -1.905 -1.516 -3.002 -3.018 -2.578 -3.75 -2.593

AI497234 -0.419 -0.043 0.332 0.753 1.343 1.136 1.301 2.252 0.944 0.02 -0.356 0.086

AI497265 -0.109 0.2 1.042 1.416 1.874 0.296 0.801 1.496 0.234 0.474 0.151 0.056

AI497414 0.699 0.869 0.698 0.746 1.026 1.083 0.675 1.145 0.543 0.079 0.345 -0.185

AI522349 -0.728 -0.428 -0.969 -1.548 -0.881 -0.25 -0.274 0.85 0.518 0.233 -0.098 -0.006

AI522421 -0.979 -0.025 0.021 -0.384 0.01 -0.129 -0.526 -0.562 -0.337 1.421 0.496 -0.591

AI522447 -1.414 -0.532 -1.15 -0.611 -0.559 0.454 0.529 1.046 0.344 1.242 1.29 1.055

AI522514 -0.312 -0.032 0.782 0.659 1.398 0.212 0.444 0.849 0.334 0.479 0.67 0.509

AI522700 -0.056 -0.832 -0.606 -0.434 -0.675 0.069 0.099 -0.085 0.2 0.637 -0.195 -0.006

AI522706 -0.503 -0.335 -0.576 0.09 -0.618 -0.441 -0.364 -0.156 0.76 0.744 0.408 -0.411

AI544512 -0.264 0.091 1.096 1.692 2.037 0.167 0.746 1.734 0.205 0.631 0.259 0.263

AI544535 0.64 1.491 2.202 2.273 1.915 0.284 0.465 0.467 -0.113 0.054 0.084 -0.036

AI544649 -0.454 0.248 0.878 1.482 3.674 4.326 4.566 3.167 3.601 1.261 0.632 -0.616

AI544688 -0.728 0.757 1.734 2.33 1.529 0.394 0.62 0.543 0.217 -0.471 0.453 -0.173

AI544976 1.357 -0.236 0.392 0.318 -0.324 -0.133 -0.225 -0.905 -1.081 -0.779 -0.933 -0.613

AI545002 -0.728 0.07 0.001 -0.012 0.12 0.018 -0.294 0.264 -0.253 -0.445 0.079 0.694

AI545040 -0.029 0.338 -0.164 0.209 0.508 -0.019 0.34 0.66 -0.159 0.143 0.153 0.511

AI545065 -0.663 -0.527 2.102 2.511 2.801 2.428 2.265 2.853 1.872 0.184 0.039 -0.54

AI545141 0.982 1.541 0.809 0.946 0.075 0.175 -0.115 0.387 -0.153 -0.233 -0.647 -0.889

AI545142 -0.853 0.334 -0.436 0.341 1.054 0.905 0.97 2.212 0.996 1.545 0.791 -0.38

AI545168 0.937 0.541 0.466 0.31 -0.092 -0.243 -0.519 -0.385 -0.601 -0.119 -0.389 -0.08

AI545274 0.249 0.629 0.637 0.432 0.592 0.058 0.579 0.752 0.014 0.21 0.074 0.214

AI545284 -0.187 -0.769 0.34 0.057 1.041 1.21 0.552 0.771 0.562 0.41 0.504 0.377

AI545291 -0.977 -2.79 -0.623 -1.127 -2.039 -0.943 -0.852 -2.154 -1.263 0.641 0.65 0.223

AI545318 -0.295 -0.544 -0.651 0.093 0.443 0.915 0.425 0.979 0.319 0.901 0.372 0.478

AI545320 0.348 0.042 0.842 0.593 0.79 1.042 0.978 0.831 0.605 0.494 0.524 0.264

AI545321 -0.405 -0.149 -0.362 -0.273 -0.473 0.142 0.261 1.085 0.862 0.797 0.94 -0.03

AI545424 -0.092 -0.308 0.425 0.758 1.135 0.753 0.961 1.091 0.771 0.771 1.032 -0.053

AI545442 1.216 -0.788 -0.52 -0.865 -0.807 -0.226 -0.822 -0.762 -0.574 -0.092 -0.134 0.313

AI545450 -0.645 -0.367 -0.76 0.09 0.233 0.523 0.243 0.647 0.533 0.741 0.492 0.019

AI545455 -0.45 -0.795 -0.018 -0.136 -0.437 -0.158 0.02 -0.447 -0.057 0.195 0.351 0.126

AI545576 0.576 -0.05 -0.632 0.102 0.507 0.163 0.454 0.953 0.767 0.956 0.692 -0.193

AI545947 0.164 -0.293 -0.11 0.073 0.438 0.215 0.468 0.779 0.231 0.233 0.068 -0.42

AI558273 0.903 0.356 0.337 0.377 0.359 0.829 0.062 0.929 0.474 0.349 0.273 -0.046

AI558282 -0.201 0.225 0.372 0.829 0.701 -0.136 0.126 0.435 0.166 -0.009 0.4 0.559

AI558301 0.074 0.849 0.845 0.861 1.366 0.63 0.907 0.975 0.515 -0.046 -0.196 0.013

AI558311 -0.445 -0.226 -0.538 -0.23 0.206 0.221 0.047 0.532 0.148 -0.038 0.681 0.137

AI558351 -0.774 -0.705 0.49 0.338 1.178 1.902 1.068 1.295 1.039 0.704 1.115 0.679

AI558352 -0.453 0.095 -0.352 -0.623 0.407 0.066 0.149 0.172 -0.026 0.425 0.511 -0.126

AI558398 1.751 0.584 -0.479 -0.748 -0.722 0.031 -0.11 -0.423 -0.225 -0.192 -0.523 -0.502

AI558431 -0.044 -0.079 0.01 0.708 0.633 0.555 0.363 1.151 0.241 0.472 0.246 -0.199

AI558451 -0.775 -2.631 -1.15 -0.979 -0.572 0.505 0.165 0.356 0.32 1.172 0.919 0.344

AI558502 -0.434 -1.186 -0.728 0.067 0.023 0.696 0.934 0.884 0.698 0.948 0.424 0.6

AI558512 -0.54 0.545 0.407 -0.128 0.105 0.056 -0.212 0.215 -0.097 -0.04 0.701 -0.153

AI558547 -0.651 -1.56 -0.297 -0.069 0.232 -0.157 -0.193 -0.387 0.216 0.03 0.336 0.156

AI558559 0.987 0.519 -0.936 -0.401 -0.429 -0.055 -0.212 -0.099 -0.137 -0.995 -0.271 -0.721

AI558578 0.226 0.556 1.976 1.461 1.677 0.812 0.577 0.898 0.458 -0.123 0.091 -0.098

AI558661 -0.107 -0.48 0.162 0.061 0.61 -0.053 0.27 -0.127 -0.333 -0.094 -0.605 -0.836

AI558833 -0.853 -3.19 -1.354 -1.176 -0.748 0.48 0.001 0.246 0.239 1.075 0.661 0.196

AI558845 -0.504 0.514 0.444 0.392 0.199 -0.248 0.242 0.741 -0.195 0.168 -0.217 -0.234

AI584223 -0.486 0.151 0.326 0.339 0.614 0.346 -0.02 0.933 0.381 0.524 0.328 0.23

AI584292 0.196 -0.045 0.188 0.406 0.206 0.356 0.495 0.752 0.131 0.798 0.459 0.165

AI584307 -0.236 -0.373 -0.223 -0.095 0.772 0.641 0.91 0.734 0.419 0.776 0.734 0.566

AI584322 -0.728 -0.129 0.191 0.191 1.77 0.432 0.263 1.276 0.308 -0.171 -0.056 0.134

AI584348 -0.017 0.242 0.541 0.778 0.689 -0.188 0.175 0.752 -0.031 -0.2 -0.164 0.086

AI584349 0.493 0.878 0.516 0.687 0.646 0.325 0.134 0.762 0.169 -0.005 0.19 -0.149

AI584352 -1.853 -2.896 -1.132 -0.809 -0.347 -0.2 -0.326 -1.229 0.245 -0.334 0.315 0.532

AI584354 0.156 -0.621 0.073 0.146 0.107 0.304 0.809 1.042 0.467 0.575 0.557 -0.04

AI584379 0.305 0.545 0.513 1.477 0.34 0.42 0.693 0.49 0.33 -0.158 0.567 0.773

AI584394 0.083 0.4 1.163 0.993 1.231 0.412 0.756 1.165 1.409 0.743 0.703 -0.931

AI584395 0.621 0.697 0.736 0.986 0.218 0.173 -0.126 0.014 -0.01 0.19 0.158 0.417

AI584401 0.668 0.665 1.474 1.55 1.52 1.443 1.105 1.153 0.259 0.261 -0.294 -0.32

AI584406 0.025 0.305 0.401 0.837 1.408 0.032 0.294 0.923 0.027 0.074 0.141 -0.123

AI584407 -0.537 -0.92 1.275 0.508 1.497 1.178 0.732 0.919 0.626 0.308 0.232 0.119

AI584415 0.934 1.18 -0.673 -0.737 -0.232 -0.123 0.004 -0.096 -0.212 0.067 -0.3 0.167

AI584421 0.379 -0.258 -0.486 0.06 0.091 0.279 0.204 0.574 0.31 0.306 -0.139 -0.251

AI584429 0.567 0.574 0.576 0.352 0.212 0.024 -0.08 -0.094 -0.112 -0.29 -0.261 -0.546

AI584440 -0.704 -0.12 -0.491 0.252 0.611 0.684 0.356 0.99 0.288 0.438 0.377 0.237

AI584446 -0.878 -1.711 -0.404 -0.379 0.365 0.351 0.291 0.907 0.283 0.9 0.152 0.439

AI584487 -1 -1.245 -0.437 -0.42 0.349 0.114 -0.035 0.329 0.571 0.046 0.602 0.421

AI584501 -0.134 -0.118 0.923 0.971 1.757 1.225 0.958 1.632 0.756 0.453 0.714 0.16

AI584515 -0.679 -1.22 -0.3 -0.396 -0.491 0.068 -0.242 -0.349 -0.108 0.07 0.086 0.433

AI584532 0.304 1.364 1.227 1.395 0.764 -0.28 -0.163 1.055 -0.022 0.084 0.038 -0.295

AI584541 0.293 1.216 0.6 1.118 0.843 0.59 0.44 0.994 0.097 -0.102 0.136 -0.291

AI584554 -0.494 -0.907 -0.138 -0.466 0.161 -0.489 -0.052 -0.31 0.014 -0.079 0.365 0.339

AI584555 -1.235 -1.715 -1.292 -2.098 -2.281 -0.677 -1.338 -2.143 -0.862 0.47 0.826 -0.032

AI584556 -0.399 0.289 0.283 0.759 -0.031 -0.01 0.17 0.987 0.238 0.344 0.3 0.338

AI584569 0.67 0.386 0.318 0.372 0.148 0.933 0.234 0.756 0.465 0.609 0.349 -0.106

AI584575 -0.271 0.764 0.417 0.899 0.988 0.239 0.327 0.869 -0.142 -0.026 -0.068 0.292

AI584590 1.42 -0.394 -0.833 -0.745 -0.227 0.122 -0.213 -0.127 -0.088 0.026 -0.062 -0.197

AI584659 -0.743 0.455 1.246 0.759 1.286 -0.028 -0.023 0.183 0.183 -0.132 0.281 0.14

AI584734 -3.322 -3.041 -0.694 -0.67 0.139 -0.288 -0.813 -1.15 0.762 -0.179 0.518 0.339

AI584977 -0.858 -0.978 -0.466 -1.324 -1.717 -0.394 -0.399 -1.434 -0.84 0.367 0.714 0.421

AI584986 -0.36 0.029 -0.226 0.105 1.225 -0.155 0.253 1.454 0.486 0.322 0.519 0.264

AI585020 -0.046 -0.362 -0.165 -0.035 0.212 0.324 0.453 0.303 0.227 0.232 0.523 0.114

AI585084 0.522 1.271 1.215 0.896 -0.205 -0.137 -0.285 0.447 -0.365 0.137 -0.391 -0.22

AI585168 -0.114 0.263 0.484 0.714 0.254 0.052 0.151 0.436 0.164 0.548 0.229 -0.24

AI588095 -0.27 0.29 1.117 1.12 1.265 -0.232 0.538 0.639 0.24 0.111 0.461 0.043

AI588114 0.516 1.384 0.958 0.223 0.39 0.374 0.027 0.578 -0.073 -0.21 -0.482 -0.325

AI588119 0.559 0.213 0.123 0.379 0.972 1.73 0.689 1.441 1.033 0.977 0.541 0.036

AI588156 -0.327 -0.326 -0.248 0.008 -0.062 -0.252 0.116 1.492 0.267 0.761 0.222 -0.304

AI588171 -0.184 1.299 0.44 0.592 0.761 0.115 0.124 0.532 0.068 -0.646 -0.077 -0.251

AI588172 0.325 -0.006 -0.097 0.163 -0.616 0.172 -0.283 0.27 0.291 1.015 0.589 0.091

AI588173 1.106 0.632 -0.496 -0.203 0.366 0.343 -0.003 0.398 -0.106 0.339 0.163 -0.059

AI588190 -0.728 -0.001 0.259 0.339 0.604 1.124 0.593 1.295 0.832 0.802 0.267 -0.169

AI588196 -0.441 -0.957 0.16 2.314 2.446 -0.124 1.381 1.012 0.812 0.189 0.355 -0.019

AI588214 -1.46 -0.374 2.555 2.093 1.883 1.455 1.76 1.256 1.42 1.021 0.888 0.227

AI588225 -0.567 0.041 0.126 -0.254 -0.134 -0.255 0.399 0.78 0.111 0.432 0.646 0.074

AI588286 0.133 0.279 2.754 2.045 1.857 0.516 0.404 1.659 0.485 0.696 0.235 -0.125

AI588301 -0.394 -0.098 -0.49 -0.059 -0.337 -0.236 0.004 0.458 0.122 0.867 0.261 -0.124

AI588304 -1 0.029 0.025 -0.225 -0.148 0.099 0.169 1.064 -0.005 0.687 0.515 -0.214

AI588305 0.832 0.944 1.161 0.551 0.677 0.444 0.146 0.465 0.083 -0.158 -0.39 -0.77

AI588306 0.836 1.115 0.767 0.327 0.154 0.501 0.488 0.592 0.741 -0.166 0.384 -0.191

AI588319 -0.127 0.464 0.595 0.909 1.387 0.643 0.384 0.929 0.097 0.099 0.056 0.077

AI588340 -2.249 -1.739 -2.15 -0.657 -1.332 -0.6 -0.582 0.446 1.137 1.017 0.799 0.13

AI588357 -1.767 -1.984 -1.2 -0.954 -0.742 0.548 -0.315 0.22 -0.266 0.611 0.085 0.65

AI588384 0.745 0.789 1.707 1.099 1.384 0.936 0.505 0.649 0.076 0.073 -0.164 -0.475

AI588395 -0.6 0.093 0.028 0.635 0.989 0.549 0.12 0.754 0.137 0.42 0.621 0.385

AI588447 0.306 1.383 1.037 0.678 0.932 0.046 0.112 0.625 -0.174 -0.25 -0.216 -0.257

AI588468 1.281 0.736 -0.186 -0.474 -0.071 -0.059 -0.138 -0.375 0.225 -0.552 -0.134 -0.216

AI588475 -0.433 0.267 0.245 0.072 0.889 0.409 0.553 1.35 -0.078 -0.604 0.486 0.234

AI588476 0.272 0.142 0.253 0.585 0.849 0.213 0.525 0.972 -0.053 0.44 0.232 0.263

AI588480 0.727 0.917 1.964 1.464 1.467 1.137 1.192 1.179 0.775 0.765 0.32 -0.01

AI588515 -0.859 -0.582 -0.687 -0.003 -0.214 0.456 0.087 0.188 0.084 0.741 0.391 0.265

AI588589 -0.379 -0.404 -0.413 -1.15 -1.002 -0.033 -0.06 -0.853 -0.094 0.473 0.913 0.602

AI588696 0.306 -0.119 0.624 0.771 0.964 0.264 0.599 1.087 0.228 0.103 0.045 -0.072

AI588708 -0.728 -0.544 0.996 0.962 1.027 0.593 0.424 0.634 1.445 0.812 0.558 0.162

AI588743 -1.066 -0.039 0.733 1.169 0.949 0.389 0.062 0.22 0.937 0.386 0.074 -0.843

AI588758 0.45 -0.119 -0.517 -0.112 -0.197 0.256 0.173 0.919 0.538 0.994 0.268 -0.119

AI588792 -0.958 0.621 3.218 3.383 3.336 1.811 1.388 2.132 2.479 1.48 0.833 0.44

AI601297 -0.613 0.279 -0.266 -0.337 0.239 0.318 0.221 1.004 0.45 0.447 0.833 0.567

AI601325 -0.327 0.791 1.425 1.251 1.055 -0.006 0.25 0.323 -0.077 0.261 0.116 -0.282

AI601355 -0.728 -0.045 0.277 -0.754 -0.902 -0.532 0.118 0.815 0.399 -0.08 0.932 0.559

AI601365 -2.036 -1.302 -1.991 -2.172 -1.735 -0.879 -1.403 -1.276 -0.216 1.467 1.012 0.26

AI601390 -1.251 -1.719 -0.529 0.223 0.208 0.239 0.559 0.558 0.138 0.709 0.501 0.545

AI601396 -0.23 0.406 0.524 0.341 0.175 0.176 0.26 0.193 0.117 -0.324 0.486 0.186

AI601443 -0.528 -0.162 0.577 1.422 1.582 0.275 0.553 1.269 0.512 0.703 0.754 0.551

AI601467 0.295 0.45 -0.291 0.007 0.289 0.643 0.498 1.266 0.734 0.573 0.454 0.084

AI601488 -0.921 -1.14 -0.198 0.256 0.441 0.266 -0.006 0.479 0.122 0.613 0.471 0.957

AI601514 -0.476 0.548 0.519 0.332 0.123 0.106 -0.191 0.108 0.128 -0.514 0.496 -0.1

AI601527 -0.085 0.633 0.897 0.732 0.478 0.135 0.222 0.801 0.188 0.198 -0.05 -0.044

AI601529 -0.672 -0.08 0.451 0.677 0.831 -0.089 -0.27 0.284 -0.122 -0.539 0.453 0.031

AI601541 0.11 0.089 0.064 0.372 0.69 0.569 0.766 1.31 0.282 0.331 0.032 -0.286

AI601556 0.745 0.475 0.136 0.293 0.081 0.734 0.083 0.119 0.234 0.019 -0.319 -0.058

AI601567 -3.082 -2.648 -0.939 -1.165 -0.426 -0.621 -0.905 -1.129 0.364 -0.312 0.473 0.26

AI601583 0.744 -0.652 1.139 0.976 1.491 1.387 0.721 0.909 0.538 0.269 0.163 -0.135

AI601596 -0.367 0.031 0.253 0.313 1.167 -0.029 0.014 0.644 0.003 0.116 0.174 0.299

AI601641 0.384 1.425 -0.648 -0.115 1.136 1.503 0.612 1.368 0.765 -0.259 0.074 0.041

AI601664 -3.851 -5.142 -4.7 -4.147 -4.55 -2.514 -2.505 -3.943 -3.246 -1.096 0.785 1.543

AI601691 -0.186 0.459 0.586 0.521 0.445 -0.308 0.147 0.135 -0.017 0.114 0.554 0.204

AI601692 0.341 0.179 0.308 0.105 -0.163 0.039 0.522 0.564 0.155 0.241 0.217 0.294

AI601696 0.227 0.631 0.089 -0.002 0.318 -0.127 -0.037 0.706 0.08 -0.462 -0.14 -0.307

AI601714 -2.571 -1.432 -1.49 -0.935 -1.685 -0.957 -0.92 -1.683 -0.705 0.631 0.65 0.957

AI601765 -0.175 -0.603 -0.322 0.188 0.907 0.45 0.841 1.248 -0.378 0.586 -0.543 -0.525

AI601781 -2.101 -1.46 -2.149 -0.736 -2.274 -0.679 -1.625 -1.358 -0.714 1.746 1.367 0.135

AI601782 0.255 0.129 -0.327 -0.542 0.077 0.166 0.243 0.311 0.126 0.44 0.869 0.615

AI601793 -1.177 -1.514 -1.009 -1.502 -2.075 -0.573 -0.092 -0.039 0.422 1.235 0.755 -0.048

AI601848 -0.914 -1.341 -0.284 -0.322 0.17 0.262 0.102 0.542 0.578 0.753 0.338 0.101

AI601856 0.392 0.307 0.127 0.694 0.928 1.055 0.77 0.237 0.212 0.058 0.034 0.285

AI616791 -0.498 -0.311 0.902 1.224 1.794 1.573 0.828 1.025 0.399 0.892 1.055 1.106

AI617012 0.837 -0.386 -0.877 -0.816 -0.061 0.18 -0.206 0.218 -0.052 0.557 -0.018 -0.002

AI618133 -4.183 -6.661 -3.612 -4.65 -5.34 -3.359 -3.884 -5.997 -3.872 -3.063 -2.339 0.703

AI618715 -0.966 -1.265 0.052 0.887 1.519 0.915 0.659 1.409 0.644 0.487 0.298 0.486

AI626336 0.843 0.172 -0.569 -0.205 -0.528 0.269 -0.191 0.064 -0.03 0.204 0.015 0.3

AI626348 -0.35 -0.718 -0.735 -0.837 -1.027 -0.29 -0.182 -1.088 -0.446 -0.223 0.291 0.518

AI626420 1.582 1.488 1.703 0.916 0.988 0.973 0.694 1.035 0.251 0.243 0.015 -0.01

AI626435 0.045 1.336 1.606 2.652 2.284 0.922 0.828 1.492 0.242 0.02 0.464 -0.196

AI626451 -0.32 -0.687 0.08 -0.842 0.26 -0.195 -0.607 -0.378 0.51 -0.343 0.468 0.429

AI626587 0.697 0.594 0.294 0.758 0.27 0.517 0.607 1 0.308 0.552 0.458 0.021

AI626598 -0.879 -0.234 0.05 0.065 0.115 -0.039 -0.164 -0.062 -0.096 -0.175 0.595 0.384

AI626599 -0.023 -0.314 1.163 1.64 1.403 1.238 0.924 1.662 0.66 0.414 0.029 0.048

AI626603 -3.014 -3.394 -1.296 -1.237 -0.6 -0.834 -0.941 -1.553 0.368 -0.767 0.39 0.412

AI626604 -0.051 -0.282 0.118 0.525 0.611 0.504 0.403 0.275 0.063 0.2 0.052 0.739

AI626645 -1.82 -1.646 -2.22 -1.588 -1.941 -0.231 -1.139 -1.811 -0.93 0.483 0.18 -0.159

AI626681 0.847 0.235 -0.815 -0.482 -0.202 -0.066 0.042 0.032 -0.474 -0.406 -0.491 -0.495

AI626686 -0.387 -0.136 -0.525 0.087 0.303 0.121 -0.039 0.424 0.236 0.565 -0.05 0.033

AI626799 -0.679 0.35 0.212 -0.308 -1.107 -0.284 -0.286 -0.373 -0.331 -0.14 1.113 0.552

AI629101 0.284 -0.129 0.553 1.176 1.561 0.353 0.855 1.596 0.29 0.095 0.299 -0.119

AI641018 0.337 0.065 -0.137 0.267 0.476 0.91 0.678 1.033 0.409 0.528 0.424 0.129

AI641022 -0.327 -0.016 1.234 0.821 1.636 1.909 1.426 1.858 1.155 1.089 0.366 0.319

AI641028 0.111 0.253 0.23 0.248 0.103 0.205 0.108 0.848 -0.047 0.141 -0.01 -0.058

AI641036 1.026 1.511 0.746 1.006 0.988 0.845 0.344 0.514 0.027 -0.007 -0.128 -0.526

AI641041 -0.561 -0.429 0.645 0.737 1.158 0.984 0.489 0.562 0.468 0.393 0.662 0.601

AI641051 0.208 -0.129 0.006 0.333 0.011 0.176 0.389 0.722 0.391 0.411 0.488 0.355

AI641052 0.185 0.342 1.664 1.511 1.641 2.115 1.561 1.352 1.171 1.08 0.581 0.538

AI641066 -0.024 0.629 1.026 1.216 0.744 0.113 0.125 0.613 0.116 0.402 0.32 -0.095

AI641069 1.506 1.293 0.691 0.133 0.316 0.196 0.049 0.789 0.038 -0.138 -0.08 -0.38

AI641092 -0.824 -1.091 0.25 0.571 1.685 2.137 1.515 0.862 1.178 0.875 1.039 0.792

AI641124 -0.24 -0.89 -0.023 -0.238 0.248 0.41 0.271 0.988 0.134 0.485 0.265 0.01

AI641129 0.016 -0.176 1.639 1.64 1.296 0.248 0.447 0.561 -0.086 -0.352 -0.555 -0.583

AI641141 -0.559 -0.699 -0.4 -0.305 -0.516 -0.172 -0.133 -0.03 0.351 0.478 0.419 0.265

AI641146 0.398 -0.079 0.539 0.171 -0.065 0.086 0.092 0.738 0.16 0.295 0.112 0.075

AI641272 0.257 -0.569 0.765 0.707 0.884 0.081 -0.03 0.524 -0.356 -0.046 -0.053 -0.259

AI641401 -0.728 0.141 0.218 0.819 1.336 0.255 0.075 1.694 -0.378 0.006 0.349 0.119

AI641409 -0.197 -0.576 1.189 0.473 1.036 1.642 0.824 1.416 0.585 0.159 0.072 0.021

AI641414 0.025 -0.377 -0.269 -0.221 -0.269 0.144 -0.03 -0.048 0.388 0.2 0.423 0.905

AI641454 0.093 0.655 1.033 1.221 1.018 0.028 0.224 0.41 -0.091 -0.049 -0.455 -0.218

AI641460 -0.482 -0.355 0.391 0.474 0.71 0.197 0.291 0.544 0.411 -0.105 0.68 0.707

AI641473 1.063 0.762 0.759 0.827 0.605 0.904 0.406 0.505 -0.025 -0.104 -0.451 -0.785

AI641480 -0.682 0.079 0.893 2.363 2.239 0.221 0.022 0.248 -0.235 -0.799 0.107 0.018

AI641491 0.839 0.841 0.761 -0.142 0.494 0.66 0.404 -0.005 0.196 0.261 0.252 0.557

AI641523 -0.172 0.17 -0.164 0.213 1.067 -0.067 0.444 1.602 0.26 0.11 0.033 -0.017

AI641534 -1.09 -1.603 -0.709 -0.182 -0.053 0.813 0.509 0.667 0.37 0.645 0.348 0.135

AI641561 -0.145 -0.866 -0.151 0.448 0.492 0.302 -0.19 -0.212 0.35 -0.318 0.316 0.02

AI641585 1.057 -0.297 -0.188 0.023 -0.006 0.16 0.355 0.661 0.053 0.245 0.126 -0.002

AI641624 -0.047 -0.483 -0.436 -0.194 0.009 0.179 0.435 0.536 0.209 0.647 0.122 0.405

AI641626 -0.248 -0.233 -0.185 -0.638 -0.208 -0.238 0.251 0.668 0.133 0.478 0.807 0.214

AI641634 -1.56 -2.449 -0.737 -0.679 -0.167 -0.28 -0.223 -0.933 0.154 -0.354 0.311 0.292

AI641664 -1.452 -1.617 -2.646 -2.268 -2.03 -1.118 -1.655 -1.529 -1.812 0.849 0.719 0.356

AI641680 -0.825 0 -0.263 0.341 -0.199 -0.148 -0.144 0.789 1.02 0.682 0.456 0.044

AI641708 0.365 0.545 0.748 0.344 0.619 0.135 0.036 0.212 -0.012 -0.112 0.201 -0.307

AI641716 0.317 0.322 0.714 0.954 1.067 0.457 0.371 0.875 0.204 0.291 0.529 0.078

AI641717 0.464 0.648 1.092 0.632 0.841 1.251 0.993 0.793 0.319 0.6 0.119 0.056

AI641772 -0.666 -0.093 -0.096 0.135 0.015 -0.378 0.026 -0.379 -0.113 0.283 0.348 1.126

AI641775 0.52 -0.523 -0.217 0.085 0.244 0.851 0.986 0.91 0.598 0.813 0.477 0.325

AI641779 1.285 0.512 0.058 0.414 0.694 0.916 0.331 0.771 0.518 0.497 0.304 0.256

AI657598 0.453 1.31 1.029 0.937 -0.38 -0.452 -0.456 0.031 -0.472 -0.066 -0.235 -0.347

AI657601 -0.706 -1.033 -1.125 -0.355 0.11 0.124 0.199 1.333 0.738 0.992 0.306 -0.36

AI657632 -0.458 0.206 0.665 1.115 1.281 0.464 0.086 0.526 -0.232 0.041 0.138 -0.321

AI657670 -0.458 -0.325 -0.061 -0.169 -0.727 -0.039 -0.808 -0.158 -0.169 1.113 0.607 -0.049

AI657697 0.163 0.479 0.868 1.062 1.009 1.234 0.817 1.075 0.605 0.449 0.072 -0.113

AI657777 -0.662 -0.891 -0.183 -0.73 -0.205 0.076 0.02 0.622 0.124 0.746 1.055 0.396

AI657832 0.158 0.189 0.207 0.398 0.443 0.094 0.144 0.691 0.185 -0.221 -0.206 -0.444

AI657922 -0.116 -0.578 -0.066 0.346 0.471 0.231 0.436 0.797 0.129 0.511 0.483 0.49

AI658011 -2.105 -2.831 -2.338 -2.415 -2.821 -1.4 -1.731 -2.48 -1.406 1.029 0.832 1.501

AI658017 0.117 -0.431 -0.108 -0.129 0.069 0.093 -0.129 0.543 0.42 0.86 0.51 0.251

AI658223 0.744 1.552 1.589 1.29 0.405 0.043 -0.028 -0.021 -0.013 -0.857 -0.449 -0.818

AI658234 -1.346 -1.949 -1.498 -0.678 -1.042 -0.726 -0.529 0.2 0.974 0.74 0.588 -0.372

AI658249 -0.525 -0.047 0.149 -0.51 -1.217 -0.349 0.073 -0.742 -0.407 -0.375 0.31 1.46

AI666873 0.674 1.331 1.17 0.901 0.662 -0.098 0.06 0.464 0.228 0.022 -0.02 -0.28

AI666879 0.858 0.603 -0.342 -0.485 -0.247 -0.258 -0.448 -0.532 -0.784 -0.331 -0.405 -0.304

AI666881 -0.071 0.767 0.65 0.647 -0.301 0.005 0.029 0.182 -0.215 0.158 -0.236 -0.209

AI666882 0.26 0.045 1.103 1.61 1.632 1.736 1.657 1.429 0.796 0.682 0.01 -0.07

AI666886 0.778 0.36 -0.201 -0.142 -0.289 0.314 0.254 0.173 0.034 -0.255 -0.269 -0.216

AI666897 -0.281 1.675 0.623 0.978 1.02 0.032 0.351 0.347 -0.084 -0.524 -0.304 -0.244

AI666911 1.069 0.898 -0.216 -0.129 -0.15 0.387 -0.371 -0.244 -0.053 0.096 -0.139 0.266

AI666922 -0.128 -0.953 -0.889 -0.499 -0.16 0.379 0.619 1.418 0.867 0.702 0.608 0.758

AI666923 0.55 0.252 -0.219 0.375 0.395 0.063 0.429 0.674 0.13 0.324 -0.05 -0.087

AI666975 -0.728 -0.309 0.323 1.066 1.581 0.041 0.272 1.619 1.134 0.562 0.345 -0.237

AI666989 -0.332 -0.525 -0.538 0.242 0.673 1.069 0.462 1.38 0.822 1.018 1.017 0.511

AI667069 0.171 0.513 0.453 0.338 0.632 0.161 0.214 0.095 0.025 0.136 -0.058 0.065

AI667071 -0.681 -0.216 -0.191 -1.076 -0.968 -0.206 -0.061 -0.242 -0.248 0.303 0.625 1.088

AI667083 0.266 0.081 0.14 0.262 0.801 0.441 0.135 0.761 0.142 0.157 -0.005 0.037

AI667141 1.264 0.117 0.466 0.026 -0.06 0.565 0.388 0.005 0.229 0.338 0.336 0.53

AI667165 0.534 0.388 1.383 0.915 0.941 1.114 0.683 0.614 0.251 0.279 0.014 -0.179

AI667167 -0.613 -0.133 1.09 0.248 0.846 1.755 0.972 1.149 1.245 0.806 0.397 0.227

AI667202 0.468 0.044 0.816 0.466 0.418 0.256 0.336 0.709 0.435 0.681 -0.054 0.096

AI667214 0.991 0.459 -0.978 -1.179 -0.859 -0.102 -0.545 -0.647 -0.308 -0.409 -0.497 -0.498

AI667241 -0.614 -0.087 -0.483 -0.448 -0.193 -0.499 -0.308 -0.667 -0.26 -0.289 0.585 1.009

AI667252 0.168 1.377 0.815 0.948 0.604 -0.027 0.068 0.193 -0.113 -0.733 -0.47 -0.384

AI667275 -2.328 0.627 3.249 2.456 1.78 1.655 1.485 1.596 1.414 1.142 0.612 0.355

AI667279 0.844 1.161 0.77 0.299 -0.02 -0.137 -0.143 0.204 -0.039 0.164 0.095 -0.488

AI667289 1.039 -1.263 -0.838 -1.613 -0.966 -0.011 -0.625 -0.657 -1.122 -1.124 -1.648 -1.65

AI667290 -1.257 0.384 -0.379 0.072 0.206 0.906 0.276 0.588 0.71 1.038 1.216 0.817

AI667319 -0.359 -0.349 -0.775 -0.18 -0.332 -0.091 0.024 -0.306 -0.087 0.102 1.13 0.728

AI667323 1.995 -0.778 -0.642 -0.7 -0.31 0.442 -0.109 0.334 -0.08 0.727 0.251 0.019

AI667324 0.467 -0.606 0.223 0.693 0.376 0.021 0.458 0.789 0.113 0.431 0.04 -0.264

AI667325 -0.598 -0.28 1.121 0.333 0.682 1.574 0.927 1.124 0.786 0.921 0.682 1.106

AI667326 0.028 0.772 1.289 1.623 0.868 1.361 0.476 0.787 0.16 0.148 0.043 -0.155

AI667333 0.392 -0.088 0.227 -0.058 0.493 0.402 0.058 0.707 0.183 0.133 -0.005 0.018

AI667396 -3.384 -3.319 -3.531 -2.728 -2.813 -1.5 -1.265 -0.582 0.796 0.417 0.672 0.143

AI667403 0.116 0.165 2.419 2.743 1.961 1.197 0.527 0.779 0.42 0.85 0.166 0.691

AI667411 0.802 0.79 -0.212 0.033 -0.122 0.015 -0.41 -0.099 -0.194 -0.524 -0.433 -0.636

AI667513 -0.629 -1.308 -1.288 -0.48 -0.708 -0.255 -0.211 0.224 -0.08 0.537 0.757 0.367

AI667526 0.307 0.986 0.644 0.706 0.42 0.135 0.213 0.714 0.012 0.129 0.089 -0.219

AI667530 -0.08 0.15 1.032 0.963 0.85 0.448 0.312 0.498 0.03 -0.121 -0.711 -0.293

AI667546 -2.11 -0.978 -1.102 -0.883 -0.985 -0.513 -0.573 -0.122 0.356 0.803 0.768 0.873

AI667581 0.894 0.927 0.458 -0.276 -0.69 -0.149 -0.436 -1.147 -0.888 -0.393 -0.511 -0.428

AI667622 1.891 1.044 -0.791 -0.091 -0.537 -1.192 -0.739 -1.111 -1.715 -1.513 -1.977 -2.14

AI667625 0.769 0.677 1.273 1.029 1.067 0.934 0.639 1.062 0.404 0.446 -0.33 -0.482

AI667626 1.113 1.152 1.181 0.907 0.256 0.17 0.007 -0.2 -0.401 -0.412 -0.673 -0.742

AI667659 0.997 -1.111 -1.388 -1.616 -1.386 -0.137 -0.477 -0.17 -0.357 -0.235 -0.246 0.089

AI667671 1.183 1.232 0.721 0.68 0.547 0.379 0.529 0.827 0.101 0.087 -0.065 -0.215

AI667676 1.65 0.668 -0.701 -0.531 -0.73 -0.68 -0.395 -1.042 -1.179 -1.687 -1.613 -1.855

AI667677 0.596 1.117 1.655 1.416 0.896 0.19 0.335 0.726 0.542 0.318 0.246 -0.066

AI667682 0.836 1.832 0.835 0.363 0.557 -0.088 0.112 0.561 -0.059 -0.644 -0.264 -0.104

AI721290 1.106 0.677 0.457 0.154 0.115 0.6 0.309 0.751 0.166 0.345 0.116 -0.022

AI721333 0.565 0.614 0.097 0.184 0.683 0.681 0.327 0.74 0.198 0.36 -0.057 -0.101

AI721335 -0.232 -0.286 -0.267 0.164 0.387 0.568 0.379 0.529 0.173 0.546 0.132 -0.223

AI721340 0.433 -0.438 0.269 0.028 0.757 1.373 0.738 1.051 0.793 0.768 0.457 0.267

AI721358 1.077 -0.315 -0.308 -0.451 -1.379 -0.502 -0.39 -1.494 -0.575 -0.125 -0.97 -0.339

AI721361 -0.202 -0.509 1.223 1.74 1.965 1.81 0.826 0.961 0.726 0.29 0.067 0.244

AI721398 1.611 -0.299 -3.023 -1.153 -2.283 -1.413 -1.155 -2.316 -1.451 -2.221 -1.308 -1.124

AI721419 -0.464 -1.437 -1.735 -1.2 -0.273 1.44 0.916 1.195 0.501 0.619 -0.011 -0.364

AI721420 1.257 0.872 1.082 0.817 0.063 0.007 0.375 -0.285 0.145 0.274 -0.225 -0.173

AI721423 0.92 1.255 1.642 1.212 0.644 1.226 0.4 0.439 0.202 0.078 -0.187 -0.113

AI721428 0.46 -0.218 -0.226 0.381 0.788 0.166 0.595 1.062 0.383 0.305 -0.027 -0.413

AI721479 -0.112 -0.631 -0.518 0.092 1.091 0.905 1.256 1.958 1.054 1.017 1.155 0.074

AI721501 -0.452 0.545 -0.058 0.341 0.569 0.14 -0.001 0.836 -0.254 -0.357 -0.059 -0.503

AI721504 -0.653 -0.005 -0.296 -0.503 -0.058 0.011 -0.45 -0.221 0.107 0.829 1.237 0.601

AI721511 0.333 0.605 0.804 0.736 0.406 0.837 0.256 0.794 0.453 0.675 0.234 -0.151

AI721531 -0.209 -1.324 -0.461 -0.812 -1.284 -0.48 -0.043 -0.98 -0.255 0.389 0.465 1.205

AI721534 -0.236 -0.683 -1.504 -1.349 -1.148 -0.39 -0.268 0.692 0.392 0.767 0.299 0.284

AI721548 -1.777 -1.616 -0.273 -0.415 0.22 0.126 -0.172 -0.319 0.709 0.074 0.554 0.312

AI721573 -0.708 0 0.34 0.464 0.534 -0.276 0.066 0.809 0.279 0.438 0.588 -0.01

AI721587 -0.033 -0.06 0.542 1.116 1.282 0.449 0.566 1.408 0.635 0.466 0.139 0.169

AI721611 0.311 0.496 0.9 1.112 1.47 0.691 0.54 1.651 0.301 0.529 0.026 -0.248

AI721615 -0.543 -0.642 0.421 0.496 1.182 0.597 0.455 1.008 0.23 0.301 -0.059 0.112

AI721616 0.691 0.11 0.198 -0.016 -0.117 0.319 0.501 0.952 0.286 0.653 0.175 -0.063

AI721634 0.177 -0.407 0.097 0.219 -0.094 0.312 0.459 0.857 0.812 1.055 0.212 -0.623

AI721654 0.128 1.127 0.585 1.04 0.697 0.505 0.335 1.177 0.109 0.014 0.309 -0.097

AI721655 -0.68 0.012 0.889 1.683 1.731 0.89 0.362 1.019 0.822 0.66 0.475 0.509

AI721701 -0.609 0.226 2.295 1.464 1.557 1.793 1.413 1.64 0.911 0.79 0.274 0.192

AI721710 -0.463 0.089 1.633 1.161 1.462 0.609 0.517 1.037 0.547 0.386 0.367 0.123

AI721741 -0.271 -0.258 -0.276 -0.824 -0.76 0.033 -0.221 0.458 0.36 0.122 1.137 0.62

AI721869 -0.129 1.07 0.806 0.52 0.507 0.706 0.182 0.327 0.808 0.214 0.421 -0.067

AI722307 -0.287 0.545 0.407 -0.438 -0.025 -0.163 -0.193 -0.121 0.158 0.097 0.705 0.044

AI722328 0.381 0.015 1.576 1.122 2.013 1.245 1.017 1.432 0.778 0.383 0.162 -0.086

AI722334 -1.989 -3.035 -1.474 -2.454 -3.394 -1.038 -1.001 -1.328 -0.558 0.73 0.718 0.134

AI722349 -0.764 -0.031 -0.62 -0.738 -0.003 -0.013 0.06 0.554 0.57 0.867 1.085 0.237

AI722354 -0.247 0.545 0.504 0.341 0.064 -0.205 0.051 0.193 0.048 -0.378 0.606 -0.082

AI722369 -1.634 -4.058 -1.963 -2.107 -2.634 -1.155 -1.471 -3.924 -1.597 -0.916 0.582 1.915

AI722402 0.497 0.816 0.284 0.611 0.811 0.596 0.465 1.401 0.527 0.218 -0.107 -0.516

AI722421 -0.653 0.171 0.763 0.981 0.783 0.17 -0.085 0.665 0.125 0.346 -0.166 0.213

AI722432 -1.451 -0.382 -1.165 -0.655 -0.826 -0.443 -0.396 -0.681 -0.318 0.792 0.012 -0.404

AI722464 -0.906 -1.086 -1.397 -1.71 -1.401 -0.259 -0.757 -1.252 -0.156 1.04 0.971 0.994

AI722482 1.179 0.576 -0.232 -0.237 -0.339 0.026 -0.005 -0.466 -0.17 -0.316 -0.077 -0.383

AI722485 0.386 0.819 0.383 0.65 0.593 -0.235 0.086 0.376 0.115 -0.095 0.248 -0.064

AI722493 0.616 1.444 1.059 0.723 1.018 0.724 0.319 0.677 -0.073 -0.753 -0.31 -0.418

AI722496 -0.011 0.319 0.407 0.644 0.704 0.181 0.406 0.834 -0.024 0.324 -0.195 -0.03

AI722499 1.196 1.737 2.443 0.999 0.448 0.499 -0.107 -0.099 -0.04 -0.372 -0.597 -0.942

AI722515 -0.114 -0.675 0.045 0.037 0.398 0.461 0.374 1.338 0.517 0.515 -0.171 -0.885

AI722538 -0.034 0.225 0.152 0.562 0.77 0.131 0.309 1.052 0.611 0.717 0.239 -0.322

AI722566 0.999 1.258 0.202 0.073 -0.034 0.154 0.303 0.425 -0.213 -0.282 -0.462 -0.408

AI722567 -0.036 -0.045 1.117 1.003 0.878 0.75 0.565 0.507 0.532 0.076 0.232 -0.438

AI722592 -0.538 -1.219 -0.539 -0.498 -0.266 0.573 0.348 0.444 0.251 1.137 0.826 0.239

AI722645 -0.351 -1.571 -0.358 -0.759 -0.684 -0.154 -0.295 -0.463 -0.036 0.063 0.524 0.778

AI722650 -0.292 0.327 -0.009 0.211 0.895 1.289 0.768 1.201 0.816 0.342 0.206 0.164

AI722733 -0.106 0.903 0.723 0.043 -0.064 -0.189 0.07 -0.191 0.045 -0.282 0.059 -0.411

AI722745 -1.013 0.196 0.92 0.761 1.912 1.332 0.137 1.489 1.304 0.77 0.493 1.446

AI722764 -0.272 0.001 0.826 0.677 0.834 0.166 0.153 0.782 0.222 0.041 0.152 -0.086

AI722818 -0.093 -0.373 -0.099 -0.085 0.614 0.004 -0.028 -0.114 0.148 0.018 0.491 0.43

AI722829 -0.224 -0.395 -0.176 0.306 0.288 0.022 0.43 1.018 0.1 0.542 0.578 0.324

AI723092 -0.676 0.067 0.391 0.868 1.008 0.306 0.499 0.884 0.216 -0.055 0.73 0.317

AI723170 0.006 0.093 1.063 0.931 0.887 1.412 1 1.195 1.034 0.901 0.516 0.273

AI723212 -0.539 0.104 2.374 1.695 1.807 0.869 1.168 1.458 0.72 0.742 0.47 0.293

AI723219 -1.339 -1.305 0.957 0.726 0.466 0.607 0.838 0.602 0.502 0.778 0.449 0.174

AI723236 -0.343 0.006 0.612 0.858 1.039 0.361 -0.031 0.469 -0.096 0.06 0.393 -0.191

AI723269 0.601 1.203 1.872 1.617 1.461 1.903 1.756 1.564 0.847 0.7 0.159 -0.096

AI723286 0.779 0.534 0.436 0.42 0.723 0.844 0.796 0.943 0.478 0.8 0.099 -0.285

AI793350 -0.234 -0.906 -0.018 -0.168 0.372 -0.106 0.28 1.278 0.1 0.523 0.352 0.264

AI793353 0.843 0.181 -0.168 -0.007 -0.776 -0.007 -0.356 -0.742 -0.52 -0.166 -0.051 -0.18

AI793372 0.432 0.756 0.352 0.117 0.601 -0.04 0.484 1.118 0.3 0.272 0.27 0.282

AI793374 -0.007 -0.394 -0.129 -0.012 0.594 0.085 0.383 0.131 0.191 0.183 0.542 0.484

AI793391 0.709 1.509 0.629 0.478 0.571 0.23 0.399 0.37 0.223 -0.159 -0.594 -0.423

AI793424 -1.891 -1.113 -1.885 0.01 -1.501 -0.571 -0.502 -1.886 -1.307 0.388 0.528 1.185

AI793428 -0.228 -0.384 -0.578 -0.249 0.072 0.3 0.371 0.732 0.447 0.628 0.208 0.355

AI793454 0.05 -0.188 -0.35 0.145 -0.07 -0.274 -0.045 0.15 0.174 0.647 0.337 -0.202

AI793467 -0.764 -1.463 -1.138 -1.435 -2.131 -0.337 -1.379 -1.269 -0.665 1.122 0.881 0.275

AI793485 0.155 -0.238 -0.144 -0.184 0.233 0.506 0.2 0.494 -0.076 0.199 0.01 0.096

AI793487 -0.114 -0.2 -0.067 -0.477 0.332 -0.035 0.451 1.478 0.196 0.884 0.973 0.308

AI793490 0.348 0.044 0.244 0.586 0.333 0.752 0.537 0.206 0.156 -0.007 -0.123 -0.17

AI793509 -0.395 0.147 -0.373 -0.013 0.204 0.007 -0.127 1.169 0.592 0.453 0.704 -0.18

AI793533 -0.659 -1.727 -0.722 -1.231 -1.88 -0.436 -0.498 -1.366 -0.506 0.715 0.805 -0.056

AI793540 0.485 0.636 0.7 0.38 0.223 0.231 0.045 0.241 0.083 0.222 0.061 -0.107

AI793555 0.5 0.112 -0.263 -0.146 0.56 1.143 1.034 2.216 1.185 0.7 0.21 0.005

AI793558 0.87 0.349 -1.034 -0.618 -0.591 -0.193 -0.36 -0.769 -0.83 -1.25 -0.893 -0.68

AI793560 -0.718 -0.403 -0.25 -0.418 -0.504 -0.255 -0.192 -0.246 0.027 0.608 0.514 0.546

AI793574 0.573 0.999 1.116 1.66 1.612 1.636 0.847 1.562 0.293 0.25 -0.118 -0.415

AI793600 -0.057 -1.383 -1.53 -0.068 -0.102 0.679 0.144 0.543 0.674 0.759 0.545 0.256

AI793622 1.287 1.022 -0.552 -0.413 -0.504 -0.133 -0.3 -0.225 -0.562 -0.976 -0.627 -0.913

AI793637 -1.564 -1.937 0.593 1.126 1.301 1.884 1.595 1.335 0.873 1.228 0.708 0.565

AI793666 0.02 0.489 0.931 1.336 0.951 0.277 0.546 0.361 0.269 -0.137 -0.309 -0.304

AI793673 0.273 -0.065 1.228 1.295 1.448 1.053 0.67 0.708 0.406 0.382 0.169 -0.354

AI793690 -0.058 0.081 -0.27 -0.071 0.038 0.456 0.551 1.453 0.487 0.102 0.722 0.704

AI793701 0.068 0.575 0.466 1.301 0.962 0.516 0.435 0.978 0.321 0.376 0.286 0.09

AI793714 0.553 1.052 1.352 0.917 0.528 0.093 0.099 0.843 -0.128 0.234 0.134 -0.087

AI793728 0.877 1.196 0.6 0.703 0.545 0.422 0.147 0.403 -0.209 -0.246 -0.428 -0.676

AI793733 0.126 0.386 0.381 0.51 0.765 0.367 0.154 0.595 0.352 0.303 0.085 -0.078

AI793745 0.352 -0.605 -0.104 0.502 1.037 0.949 0.922 1.215 0.649 0.322 0.05 -0.325

AI793761 -0.488 -0.489 -1.011 -0.203 -0.361 -0.341 -0.191 -0.448 -0.184 0.469 -0.059 0.175

AI793769 -0.569 -0.448 -0.277 0.011 0.334 0.862 0.444 0.736 0.27 0.771 0.218 0.231

AI793772 -0.158 -0.506 0.017 0.336 1.171 0.833 0.443 0.745 0.195 0.145 0.889 0.421

AI793793 0.507 0.592 0.87 0.266 0.418 0.385 0.169 0.645 0.284 0.063 0.006 -0.457

AI793794 0.903 -2.113 -2.251 -2.077 -1.759 -0.609 -1.292 -1.631 -1.825 -2.031 -2.409 -2.292

AI793802 0.768 1.114 0.897 0.498 -0.195 -0.214 0.036 0.281 -0.384 -0.397 -0.11 -0.727

AI793807 -0.603 -0.248 -0.274 0.38 0.596 -0.077 0.128 0.391 -0.148 0.696 0.817 0.62

AI793812 0.025 0.862 0.594 0.401 -0.039 0.319 0.364 0.879 0.244 -0.245 -0.358 -0.23

AI793830 -0.348 -0.007 0.559 1.02 1.138 1.832 0.884 1.421 0.943 1.125 0.448 0.123

AI793838 0.412 0.933 0.779 0.781 0.921 0.269 0.382 1.032 0.084 0.329 0.063 -0.091

AI793839 -1.442 -0.725 0.824 0.78 1.624 2.048 1.371 1.703 1.114 0.755 0.401 1.15

AI793850 0.579 1.202 0.767 0.003 -0.133 -0.285 0.266 -0.009 -0.184 -0.477 -0.415 -0.093

AI793853 -0.231 0.266 0.239 0.595 1.231 0.052 0.301 1.25 0.584 0.53 0.156 0.758

AI793866 -1.399 -0.942 -1.183 -1.287 -0.542 0.175 -0.075 0.146 0.265 0.538 0.964 1.008

AI793880 0.294 0.613 0.98 0.732 1.227 0.492 0.541 1.367 0.465 0.118 -0.092 -0.511

AI793897 -0.2 -0.537 -0.48 0.064 -0.934 -0.058 -0.043 0.046 0.15 0.026 0.281 0.757

AI793901 -2.554 -1.255 -2.495 -1.142 -2.193 -1.29 -1.81 -1.537 -0.729 0.894 0.707 0.66

AI793925 -0.348 0.651 0.648 0.857 0.685 0.131 -0.152 0.096 -0.384 -0.216 0.318 -0.309

AI793927 -0.529 0.067 -0.017 0.454 0.945 0.033 0.364 1.141 0.392 0.461 0.037 -0.36

AI793934 -0.071 0.136 -0.165 0.114 1.356 0.772 0.551 2.449 1.206 0.85 0.444 -0.18

AI793969 -0.451 -0.395 0.004 0.268 0.757 0.045 0.596 0.738 0.192 0.422 0.551 0.25

AI793974 -0.055 -0.485 0.167 -0.184 0.429 0.185 0.228 1.131 0.153 0.149 0.022 0.038

AI794024 -0.839 -0.043 2.469 2.163 2.17 1.952 1.496 1.687 0.706 1.009 1.093 0.425

AI794059 -0.196 -0.539 -0.123 0.075 0.925 1.626 1.021 1.183 0.916 0.755 0.559 0.532

AI794204 0.202 0.395 0.867 0.801 0.909 0.978 0.899 0.76 0.123 0.603 0.258 0.015

AI794483 -0.643 -0.774 -0.534 -0.417 -0.028 0.101 0.194 0.935 0.181 0.428 0.182 0.611

AI794518 -1.509 -1.481 -3.206 -1.149 -2.079 -0.902 -1.031 -1.601 -0.638 0.222 0.241 1.5

AI794547 -0.113 0.352 0.066 0.584 0.47 0.178 0.255 0.087 0.022 0.047 -0.093 -0.139

AI794634 -0.361 0.948 0.696 1.214 1.183 0.241 0.386 0.883 0.14 -0.056 -0.31 -0.107

AI816663 -0.45 0.383 0.801 0.659 0.999 0.49 0.347 0.361 0.393 -0.105 -0.104 -0.463

AI816701 -0.073 0.766 0.798 0.771 -0.881 -0.454 -0.688 -1.313 -0.605 -0.299 -0.106 0.452

AI877506 1.136 1.232 1.428 1.545 1.507 1.297 1.195 1.471 0.714 0.654 -0.292 -0.84

AI877518 -0.497 0.024 0.383 0.727 1.587 1.335 0.651 1.519 0.862 0.411 0.47 0.357

AI877538 0.349 0.379 0.269 1.25 1.241 -0.143 0.647 1.385 0.304 0.303 0.281 -0.362

AI877596 0.027 0.181 1.012 2.523 3.165 1.089 1.485 0.711 0.657 -0.586 -0.282 0.002

AI877609 -0.145 0.023 0.261 0.489 0.494 0.029 0.22 0.615 0.173 0.307 -0.154 0.025

AI877678 0.233 0.399 0.186 0.598 0.928 -0.074 0.416 1.391 0.687 0.393 0.459 -0.097

AI877743 -0.093 -0.196 -0.001 -0.246 0.906 0.173 0.273 0.575 0.23 0.309 0.471 0.103

AI877869 -0.097 -0.094 0.089 0.54 1.04 0.286 0.058 0.962 0.017 0.312 0.337 0.186

AI877922 -4.777 -4.532 -4.199 -3.744 -4.242 -3.224 -3.122 -1.581 -0.243 1.774 1.229 0.918

AI877924 -0.373 0.03 -0.176 0.038 0.486 -0.21 0.298 0.143 0.042 -0.159 -0.015 0.572

AI877955 -0.401 -0.152 1.463 1.831 1.44 1.581 1.491 1.916 0.626 0.595 0.17 0.067

AI878021 -0.333 -0.343 -0.879 -0.108 0.35 0.374 0.223 1.184 0.342 0.948 0.392 0.385

AI878053 -0.255 -0.547 -0.895 -0.449 -0.651 0.165 -0.67 0.302 0.253 0.475 -0.17 0.126

AI878068 -1.035 -2.87 -1.539 -1.093 -0.572 0.381 0.111 0.427 0.27 1.034 0.561 0.176

AI878080 0.046 0.113 0.796 0.813 0.584 0.409 0.225 0.889 0.133 0.341 0.168 0.293

AI878204 0.568 1.196 1.234 1.047 0.988 0.785 0.292 0.781 0.313 0.236 -0.124 -0.291

AI878235 -1.376 -2.631 -0.993 -0.448 -0.506 -0.381 -0.162 -1.336 0.286 -0.643 0.145 -0.122

AI878244 -0.515 -0.507 0.333 0.497 0.373 0.126 0.204 0.459 0.406 0.492 0.003 0.051

AI878344 0.25 0.321 0.613 0.718 0.794 0.435 0.437 0.418 0.093 0.315 -0.174 -0.539

AI878386 -0.662 0.18 0.463 0.782 1.305 0.18 0.431 1.531 0.258 0.763 0.271 0.937

AI878392 -0.689 -0.979 -0.56 -0.257 -0.584 -0.101 -0.597 -0.446 -0.289 0.065 0.252 0.787

AI878397 1.028 1.153 -0.206 0.255 -0.115 -0.131 -0.263 -0.12 -0.563 -0.417 -0.587 -0.544

AI878403 -1.743 -1.781 -0.48 -1.104 -1.099 -0.43 -0.448 -1.237 -0.066 0.603 0.814 0.576

AI878421 0.928 0.298 0.001 0.359 0.66 1.096 1.008 0.962 0.632 0.727 0.55 0.736

AI878437 0.036 0.769 0.151 0.259 0.422 -0.15 0.275 0.61 0.381 -0.046 0.465 -0.179

AI878452 1.379 1.165 0.538 0.081 -0.065 0.184 -0.133 -0.377 -0.604 -0.421 -0.686 -1.008

AI878458 -0.804 -0.282 -0.936 -0.193 -0.797 -0.23 -0.015 0.145 0.269 0.854 -0.033 0.251

AI878489 -0.756 -1.637 -2.01 -2.019 -0.727 -0.525 -0.8 -0.341 -1.445 0.644 0.245 -0.564

AI878520 -0.436 -0.275 0.469 -0.804 -1.431 -0.219 -0.138 -0.155 -0.108 0.469 0.384 0.74

AI878608 -0.227 0.793 0.34 0.742 0.825 0.065 -0.217 0.399 0.093 -0.063 0.127 0.011

AI878617 -0.017 0.094 -0.005 0.749 0.763 0.925 0.644 1.087 0.151 0.502 0.051 0.004

AI878627 0.013 0.331 0.228 0.187 0.59 0.194 0.116 1.024 0.251 0.168 -0.156 -0.45

AI878632 0.403 1.073 0.516 0.777 0.679 0.337 0.012 0.443 -0.297 -0.066 0.023 -0.226

AI878677 -0.852 -1.265 0.82 1.121 1.023 1.064 1.344 2.301 1.447 1.729 0.933 -0.264

AI878687 1.206 0.298 0.352 0.27 1.091 0.508 0.359 0.296 0.412 0.34 0.13 0.344

AI878713 -0.205 -0.058 0.224 0.667 1.124 0.782 0.633 0.831 0.122 -0.048 -0.457 -0.234

AI878755 0.516 0.607 0.799 0.811 1.377 1.699 0.833 1.014 0.508 0.453 0.307 0.312

AI878758 -0.266 0.514 0.209 0.341 0.214 0.219 0.203 0.953 0.368 -0.117 -0.32 -0.079

AI878761 -0.626 -0.932 -0.926 -0.819 -0.266 -0.094 -0.332 -0.207 -0.195 1.017 0.692 0.806

AI878772 1.016 1.282 1.535 1.406 0.877 1.055 0.345 0.265 -0.29 -0.558 -0.646 -0.771

AI878787 0.332 0.429 0.124 0.468 0.447 0.014 0.114 0.669 -0.163 0.005 0.107 -0.207

AI878793 0.123 0.627 0.815 0.532 -0.078 -0.226 -0.576 0.121 0.15 -0.001 -0.078 -0.019

AI878796 -0.451 0.013 -0.399 -0.194 0.083 -0.049 0.017 0.745 0.162 0.188 0.034 -0.057

AI882728 -0.213 -0.435 -0.348 -0.24 0.387 0.466 0.478 0.625 0.45 0.631 0.081 0.371

AI882781 0.351 0.068 0.312 0.157 0.244 0.141 0.224 0.528 0.112 0.237 0.376 0.064

AI882824 1.786 0.943 -0.267 -0.413 -1.726 -0.571 -0.415 -1.532 -0.66 -0.357 -0.834 -2.305

AI882827 0.673 1.117 1.17 1.181 0.977 0.445 0.318 0.693 0.092 0.071 -0.057 -0.12

AI882829 1.136 1.169 -0.2 -0.203 -0.451 0.02 -0.105 0.024 -0.294 0.157 -0.126 -0.232

AI882884 -0.177 -0.613 -0.234 -0.156 0.273 0.05 0.281 1.255 0.692 0.836 0.55 -0.114

AI883253 -0.135 0.175 0.806 0.79 1.567 0.171 0.339 1.262 0.518 0.308 0.183 -0.259

AI883262 -0.925 -0.179 -0.666 -0.121 -0.646 -0.25 -0.426 -0.169 0.01 1.172 0.89 0.371

AI883323 0.079 -0.096 0.409 0.909 1.259 0.696 0.144 0.613 0.082 0.244 0.338 0.149

AI883326 -0.163 -1.796 -0.008 -0.222 0.779 0.658 0.464 1.484 0.917 0.872 0.61 0.264

AI883327 0.04 1.476 1.581 0.821 0.531 0.568 -0.086 0.609 0.537 0.142 -0.053 -0.402

AI883356 0.544 1.301 1.268 0.853 0.186 0.601 0.024 0.142 -0.189 -0.112 -0.475 -0.411

AI883430 -1.335 -0.905 -1.353 -2.157 -1.205 -0.307 -0.838 -0.856 -0.694 2.693 1.963 -0.741

AI883443 -0.609 -0.381 0.454 0.59 0.807 0.921 0.411 0.795 0.354 0.664 0.392 0.433

AI883679 -0.337 -0.382 -0.552 -0.278 -0.355 0.422 -0.127 0.393 0.132 0.905 0.493 0.389

AI883714 0.06 0.446 0.3 0.724 1.024 0.295 0.269 0.558 -0.238 -0.49 -0.202 0.076

AI883716 0.416 0.213 0.588 0.6 0.986 0.698 0.643 1.108 0.218 0.344 0.192 0.114

AI883799 -0.621 0.076 0.167 0.296 0.132 -0.19 0.019 -0.251 0.356 -0.045 0.406 0.234

AI883908 -0.4 0.346 0.176 0.363 1.212 0.103 0.551 1.099 0.227 -0.148 0.396 -0.112

AI883911 1.482 -0.406 0.501 -0.124 -0.571 -0.269 -0.462 -1.218 -1.004 -1.892 -1.223 -1.281

AI883922 0.847 0.799 0.3 0.258 0.106 0.101 -0.093 0.31 0.141 0.832 0.497 0.142

AI883925 0.534 0.306 0.09 0.48 0.422 0.824 0.191 0.835 0.559 0.68 0.343 -0.08

AI883929 -0.099 -0.297 0.153 0.398 0.549 0.643 0.133 0.184 0.088 0.001 -0.073 0.079

AI883967 0.084 0.349 0.644 0.591 1.085 0.535 0.326 1.413 0.054 0.163 -0.2 -0.493

AI883980 -0.404 0.422 0.543 0.65 0.609 0.128 0.452 0.363 0.254 -0.305 0.261 -0.311

AI884026 0.565 0.023 -0.148 0.177 0.318 0.5 0.446 1.012 0.384 0.222 0.032 -0.619

AI884029 -0.462 -0.524 -1.05 -0.45 -0.463 0.08 -0.152 0.969 0.272 1.447 0.631 -0.498

AI884045 -0.728 -0.616 -0.806 -0.849 -0.985 -0.137 0.065 0.805 0.496 0.724 0.735 0.453

AI884046 -0.793 -0.679 -0.35 -1.676 -0.971 -0.436 -0.219 -0.209 -0.368 0.279 0.854 0.686

AI884048 0.556 0.316 1.127 1.467 1.463 1.244 1.074 1.301 0.544 0.085 0.003 0.68

AI884050 -0.403 -0.444 0.494 0.806 1.05 0.714 0.356 1.399 0.134 0.716 0.025 0.258

AI884082 -0.675 -0.73 -1.096 -0.71 -0.271 1.196 0.913 1.137 0.977 1.132 0.594 0.125

AI884085 0.725 0.28 -0.807 -0.379 -0.578 0.084 -0.282 -0.445 -0.408 -0.519 -0.869 -0.79

AI884099 -0.216 -1.39 -1.02 -0.618 -0.681 -0.266 -0.312 -0.125 -0.004 0.531 0.062 -0.107

AI884112 1.236 0.976 1.369 1.842 1.645 2.304 1.567 1.26 0.598 0.487 0.071 -0.335

AI884148 -0.074 -0.159 0.254 0.351 0.335 0.363 0.253 0.715 0.446 -0.091 0.54 0.226

AI884177 -0.723 0.173 1.481 1.658 1.686 0.137 -0.12 0.193 -0.299 -0.609 -0.086 -0.222

AI884185 -0.668 0.38 0.407 1.306 1.437 0.179 0.086 0.451 -0.293 -0.61 0.223 -0.232

AI884211 0.299 1.061 1.446 1.511 0.686 0.695 0.184 0.675 0.208 0.447 -0.169 -0.439

AI884227 0.196 0.985 1.325 1.286 1.446 1.292 1.278 1.883 1.39 0.926 -0.087 -0.546

AI884279 -0.233 -0.38 -0.17 -0.291 -0.034 -0.278 -0.008 -0.608 0.064 -0.054 0.528 -0.049

AI931096 0.099 0.244 0.837 0.742 1.172 0.295 0.481 1.511 0.302 0.834 0.223 0.106

AI942585 -0.843 -0.683 -0.746 -0.875 -0.652 -0.014 0.347 1.265 0.563 0.823 0.447 0.11

AI942594 0.072 0.816 1.031 0.466 0.589 0.605 -0.045 0.57 0.202 0.146 -0.293 -0.247

AI942627 -0.07 0.135 -0.116 0.096 0.152 -0.001 0.248 0.638 0.116 0.154 0.271 0.1

AI942818 -0.031 1.327 0.329 0.662 0.924 0.595 0.354 1.212 0.079 -0.21 -0.155 -0.065

AI942839 -0.522 0.018 0.094 -0.188 0.934 0.067 0.11 0.738 0.456 0.153 0.543 -0.027

AI942866 -0.505 -1.596 -1.538 -1.226 0.022 0.716 1.01 0.835 1.822 0.661 0.255 -0.874

AI942929 1.086 0.56 -0.916 -0.494 -0.306 0.101 -0.145 -0.088 -0.061 -0.083 -0.06 0.104

AI942944 1.099 0.025 -0.503 -0.119 0.17 0.228 -0.445 -0.134 0.135 -0.294 -0.469 -0.367

AI942952 0.683 0.694 1.03 1.305 2.06 2.016 2.097 1.57 1.59 0.455 -0.444 -0.519

AI942960 -1.507 -0.377 -1.227 -0.116 -0.445 0.181 0.129 1.352 1.273 0.969 0.552 -0.224

AI942982 -0.587 0.545 0.407 0.341 -0.048 0.161 0.179 0.571 0.304 0.268 0.582 0.237

AI942983 0.846 0.802 -0.384 -0.157 -0.159 -0.056 0.389 0.714 0.069 0.179 0.064 -0.11

AI942987 0.299 -0.455 0.096 -0.044 1.566 1.277 0.66 1.213 0.678 0.352 0.823 0.414

AI942990 -0.119 -0.542 -0.384 -0.432 -0.891 -0.189 0.046 -0.898 -0.38 -0.144 -0.28 1.279

AI943036 -0.842 -0.064 0.05 -0.098 0.133 0.113 0.072 0.662 0.558 0.382 1.113 0.706

AI943044 0.472 1.073 1.229 1.11 0.778 0.289 0.271 0.858 0.177 0.009 -0.107 -0.251

AI943062 1.088 1.142 1.202 1.477 1.633 1.909 0.91 1.149 0.18 0.328 0.066 -0.134

AI943082 -0.317 -0.636 -0.741 -0.255 0.879 0.382 0.386 1.002 0.374 0.663 0.355 0.272

AI943086 -0.645 0.012 0.158 0.652 0.742 0.24 0.328 0.563 -0.21 -0.081 0.632 0.37

AI943105 -0.236 0.113 1.302 1.596 1.952 0.19 0.788 1.089 0.434 0.455 0.246 -0.079

AI943108 0.094 -0.52 0.018 0.035 0.251 0.975 0.464 0.526 0.725 0.696 0.817 0.23

AI943112 -0.436 0.018 0.181 -0.314 0.187 -0.013 -0.357 -0.306 0.096 0.21 0.847 0.784

AI943132 -0.19 -0.16 -0.053 -0.101 -0.547 -0.228 -0.233 -0.734 -0.286 0.11 0.31 0.873

AI943154 0.451 -0.596 0.032 0.268 0.563 0.622 0.625 1.268 0.635 0.812 0.564 0.295

AI943183 -0.816 -0.564 -0.752 -0.732 -0.457 -0.094 -0.342 0.091 0.558 0.486 1.172 0.844

AI943195 -0.468 -0.635 0 0.199 0.517 0.359 0.043 0.679 0.126 0.256 0.17 0.102

AI943216 0.146 -2.595 -1.038 -1.915 -0.375 1.056 -0.103 0.646 -0.881 0.596 -1.298 -0.064

AI943227 -0.093 -0.64 -0.638 -0.264 0.303 0.759 0.731 1.59 1.07 0.737 0.384 -0.072

AI943392 0.068 -0.144 0.122 0.099 0.474 0.345 0.1 0.991 0.258 0.024 -0.168 -0.295

AI957401 -1.283 -1.883 -1.559 -1.393 -1.536 -0.303 -0.016 -0.081 -0.097 0.431 0.799 1.195

AI957409 -0.502 -1.417 -0.925 -0.707 -0.655 -0.092 0.264 1.075 0.989 0.915 0.962 0.066

AI957415 -2.72 -2.669 -2.664 -2.652 -0.691 0.493 0.659 0.985 0.942 0.983 0.551 0.254

AI957426 1.179 0.865 1.032 0.998 0.961 0.727 0.165 -0.115 -0.208 0.01 -0.533 -0.366

AI957464 0.752 1.585 1.036 0.628 -0.552 -0.075 -0.111 0.381 0.159 -0.229 0.403 0.196

AI957472 0.583 0.498 0.649 0.819 1.125 1.292 0.762 0.624 0.48 0.306 -0.025 -0.078

AI957504 -0.998 -1.175 -1.491 -0.812 -1.036 -0.75 -0.903 -1.276 -0.798 0.965 0.435 -0.245

AI957519 -0.695 -0.203 1.35 1.334 1.455 1.772 1.255 1.657 1.828 1.182 0.752 0.673

AI957527 0.53 0.673 0.477 0.273 0.556 0.64 0.805 0.794 0.2 0.059 0.153 0.107

AI957572 0.906 1.041 1.139 0.797 -0.13 0.405 -0.149 -0.001 -0.566 0.059 -0.302 -0.32

AI957575 -0.666 -2.018 -1.048 -1.215 -0.306 0.448 0.679 1.135 0.512 0.698 0.369 -0.538

AI957592 -0.202 -1.759 -0.142 -0.501 -0.423 0.089 0.23 0.605 0.365 0.217 0.494 0.581

AI957596 0.25 -0.803 -1.084 -1.247 -1.395 -0.251 -0.16 -0.226 -0.268 0.104 0.209 0.808

AI957604 -0.095 -0.072 0.101 0.142 0.85 1.088 0.627 0.896 0.596 0.455 0.671 0.669

AI957628 -1.834 -1.738 -1.437 -1.382 -1.926 -0.757 -0.716 0.819 0.934 1.133 0.968 0.351

AI957698 -0.786 -0.26 1.664 1.773 2.383 1.099 0.856 1.987 0.641 0.375 -0.041 -0.912

AI957711 -0.701 -0.494 -0.405 0.512 0.84 1.262 0.538 0.731 0.537 0.295 0.158 -0.161

AI957739 -0.583 -0.103 0.01 -0.311 0.009 0.114 -0.054 0.299 -0.168 0.278 0.778 0.128

AI957746 -0.868 0.135 -0.267 0.302 0.006 -0.158 -0.472 -0.381 -0.08 1.375 0.733 -0.41

AI957752 0.07 1.2 0.654 0.841 0.377 0.239 0.201 0.82 0.249 0.3 0.339 0.285

AI957777 0.857 1.104 1.306 1.211 0.884 0.487 0.395 0.591 -0.057 -0.605 -0.571 -0.535

AI957786 0.09 0.306 0.756 0.555 0.519 0.159 0.672 1.005 0.211 0.858 0.381 0.241

AI957812 -0.627 -1.427 -0.245 -0.173 -1.454 -0.484 -0.255 -1.491 -0.641 -0.043 -0.064 1.004

AI957815 0.153 0.465 0.694 0.433 0.691 0.478 0.448 1.005 0.234 0.203 0.013 0.066

AI957816 -0.729 -1.567 -1.601 -1.015 0.029 0.34 0.542 0.886 0.552 0.466 -0.205 -0.112

AI957821 0.43 0.677 0.225 0.565 0.522 0.376 0.123 0.378 0.327 0.1 0.069 0.104

AI957831 1.475 1.006 -0.264 -0.299 -0.408 0.056 -0.183 -0.108 -0.253 -0.257 -0.337 -0.676

AI957837 0.904 0.405 0.023 0.486 -0.209 0.715 0.305 0.324 0.179 0.058 -0.411 -0.291

AI957847 -0.228 0.331 0.561 1.037 0.834 0.433 0.431 0.776 0.008 0.103 -0.09 -0.066

AI957850 0.06 0.696 0.927 0.997 0.872 0.221 -0.097 0.402 0.122 -0.399 0.011 -0.42

AI957865 0.738 1.227 0.833 0.182 0.288 0.31 0.275 0.262 -0.251 -0.097 -0.47 -0.941

AI957869 0.875 0.925 1.238 0.883 0.665 1.637 0.751 1.474 0.948 0.696 0.059 -0.429

AI957875 -0.454 -0.784 -0.343 -0.241 -0.533 0.186 0.434 0.898 0.81 0.852 0.611 -0.295

AI957893 -0.668 0.142 0.229 0.22 0.243 -0.022 -0.082 1.21 0.063 -0.186 0.148 0.129

AI957909 -0.01 -0.919 0.124 -0.129 -1.073 -0.205 -0.171 -0.804 -0.431 0.169 0.423 0.232

AI957914 -0.311 0.217 -0.501 0.353 0.68 0.081 0.092 0.778 -0.034 -0.317 0.002 0.043

AI957915 0.164 1.527 2.586 1.4 1.227 0.909 0.357 0.107 0.249 0.014 0.116 -0.187

AI958084 -0.777 0.517 1.364 0.935 0.905 0.754 -0.134 0.939 0.535 0.686 0.476 0.392

AI958147 0.45 0.798 0.476 0.577 0.253 -0.088 0.163 0.48 -0.026 -0.186 -0.317 -0.314

AI958191 -0.528 -1.156 -0.045 0.112 0.461 1.236 0.808 1.392 1.153 0.898 0.231 -0.143

AI958208 -0.514 0.139 0.939 0.894 1.072 1.263 0.683 0.912 0.67 0.711 0.442 0.594

AI958373 -0.53 0.308 0.559 0.889 0.912 0.221 0.122 0.998 0.228 0.022 -0.37 -0.95

AI958505 -0.562 -1.321 -1.543 -0.584 -0.434 -0.16 0.123 0.973 0.444 0.536 -0.071 0.151

AI958567 -2.103 -1.031 -2.17 -0.444 -1.285 -0.708 -1.107 -1.642 -1.779 0.84 0.382 1.396

AI958585 0.108 -0.186 -0.773 -0.413 -0.211 0.974 0.61 0.998 0.358 1.049 0.399 0.229

AI958627 0.025 0.469 1.697 1.959 1.904 1.67 1.284 1.064 0.465 0.561 0.327 0.581

AI958665 0.066 1.398 0.203 -0.049 0.496 0.276 0.509 0.939 0.027 0.026 0.306 -0.269

AI958820 -0.209 0.294 1.041 1.007 1.413 0.8 0.515 0.997 0.339 0.396 0.333 0.255

AI958945 -0.364 -0.189 1.392 1.866 2.146 0.542 0.536 1.185 0.249 0.449 0.321 0.322

AI959106 -0.626 -0.271 -0.568 -0.182 0.529 1.267 0.413 1.391 0.362 0.571 0.146 -0.026

AI959157 0.003 -0.26 0.567 0.817 0.936 0.266 0.611 0.3 -0.123 0.033 -0.186 -0.068

AI959222 -0.022 0.029 0.034 0.083 0.471 0.182 0.096 0.798 0.064 0.065 0.074 0.374

AI959257 -0.859 -1.429 -1.687 -0.912 -0.976 -0.477 -0.514 -0.062 0.001 0.544 0.187 0.513

AI959515 -0.553 -0.111 -0.309 -0.501 -0.131 0.301 -0.197 -0.734 -0.382 0.148 0.675 -0.098

AI959558 1.039 0.931 -0.212 -0.298 -1.983 -0.598 -0.162 -1.755 -0.545 -0.678 -1.556 -1.448

AI959620 0.036 0.096 0.341 0.928 1.303 -0.135 0.088 0.67 0.05 -0.074 0.667 -0.074

AI959637 0.624 0.113 1.226 0.719 0.877 0.761 0.639 0.432 -0.179 -0.17 -0.24 -0.14

AI959644 -0.881 0.013 2.18 3.173 4.202 3.96 3.578 3.8 2.558 -0.271 0.566 -0.431

AI959657 0.55 0.686 0.626 0.72 0.729 0.374 0.577 0.821 0.202 0.402 -0.09 -0.25

AI959659 1.564 0.438 0.078 0.374 0.414 0.629 0.135 0.396 0.071 -0.112 -0.524 -0.659

AI959670 0.186 0.643 0.592 0.517 -0.191 0.204 0.199 1.025 0.221 -0.353 0.053 0.037

AI959694 0.345 -0.045 1.047 1.182 0.737 0.699 0.446 0.258 0.091 0.604 0.368 -0.139

AI959722 0.539 0.234 -0.142 0.134 0.114 0.409 -0.036 0.148 0.35 0.508 0.67 0.71

AI959735 -0.678 0.385 2.405 2.732 3.303 1.507 1.295 2.131 0.26 -0.104 0.133 -0.36

AI959750 -0.863 -2.434 -1.026 -0.776 -0.33 0.681 0.351 0.673 0.311 1.163 0.796 0.462

AI964108 -0.001 0.685 0.155 0.717 0.533 0.354 0.258 0.732 0.236 -0.167 0.394 -0.194

AI964116 2.099 -3.467 -2.332 -2.284 -3.585 -2.832 -2.932 -4.446 -4.13 -3.956 -6.182 -5.295

AI964130 0.869 1.036 2.211 2.089 2.08 2.145 1.808 1.322 0.708 0.57 0.145 0.097

AI964174 1.216 -0.219 -0.56 -0.287 -0.35 -0.269 -0.329 -0.717 -0.496 -0.682 -0.565 -0.473

AI964178 1.023 0.223 -0.817 -0.044 -0.354 -0.182 -0.266 -0.239 -0.373 -0.214 -0.284 0.361

AI964189 0.267 1.099 1.988 1.98 1.928 1.729 1.553 1.695 0.454 0.816 0.278 -0.108

AI964204 0.243 -0.413 0.616 0.969 1.214 1.337 0.509 1.149 0.682 0.282 0.141 0.238

AI964207 0.285 0.825 0.812 0.869 0.825 -0.043 0.347 0.461 -0.125 0.009 -0.226 -0.162

AI964212 1.338 0.64 -0.055 -0.051 0.489 0.345 -0.01 0.323 0.071 -0.012 -0.165 0.058

AI964216 -1.265 -2.28 -1.221 -0.904 -0.57 0.389 0.046 0.422 0.469 1.115 0.904 0.395

AI964218 -1.063 -2.966 -1.49 -1.1 -0.802 0.384 0.038 0.24 0.18 0.958 0.635 0.085

AI964231 -0.279 -0.069 0.737 0.893 1.368 1.505 1.154 1.719 0.749 0.598 0.188 -0.337

AI964232 -0.299 -0.054 0.428 0.958 1.247 1.649 0.884 1.518 0.848 0.471 0.094 -0.369

AI964239 -1.077 -3.921 -2.01 -1.342 -0.951 0.276 0.098 0.198 0.217 0.835 0.545 -0.094

AI964258 -0.804 -1.302 -0.598 -0.063 0.155 0.718 0.651 0.896 0.61 0.683 0.138 -0.006

AI964264 -1.151 -2.091 -1.282 -1.84 -1.095 0.798 0.824 0.897 0.45 1.169 0.719 0.311

AI964274 -1.162 -0.725 -1.032 -0.951 -0.804 -0.254 -0.058 -0.741 -0.329 0.677 0.475 0.684

AI964276 -4.704 -4.884 -3.941 -3.54 -3.911 -2.741 -3.768 -5.221 -3.738 -1.064 0.367 2.267

AI964285 -0.015 0.487 0.512 0.578 0.741 0.184 0.069 0.117 0.467 0.07 0.503 0.083

AI964289 -0.623 -1.55 -0.759 -0.474 -0.499 -0.02 0.035 0.196 0.07 0.655 0.337 0.107

AI964300 0.537 0.311 1.499 1.12 1.213 1.572 0.928 0.812 0.672 0.209 0.062 -0.117

AI964306 0.949 0.638 0.411 0.347 0.223 0.166 0.72 1.223 0.563 0.415 -0.091 -0.231

AI964310 -0.405 -0.15 2.21 1.926 2.429 0.911 0.881 1.219 0.53 0.481 0.578 0.355

AI964318 0.019 -0.1 0.453 0.368 0.921 1.184 0.796 1.044 0.18 0.108 -0.351 -0.859

AI964322 -0.514 -1.284 -1.037 -0.286 -0.249 0.4 0.268 0.878 0.348 0.928 0.213 0.253

AI964328 1.112 3.178 4.891 2.989 0.824 -0.252 -0.121 -0.286 -0.435 -0.831 -0.734 -0.426

AI964352 -0.118 -0.94 -0.222 0.035 0.062 0.362 0.365 0.761 0.318 0.668 0.401 0.209

AI964367 0.249 -0.308 0.948 1.216 0.423 0.927 0.368 0.679 0.749 0.902 0.618 0.411

AI964375 0.125 0.381 0.552 0.69 0.988 0.228 0.564 1.078 0.452 0.2 -0.005 -0.485

AI964421 0.367 1.023 0.95 1.078 0.204 -0.3 -0.085 -0.407 -0.47 -0.107 -0.426 0.011

AI964971 -0.306 -1.124 -0.387 -0.436 -0.022 0.591 0.447 0.853 0.598 0.703 0.323 -0.139

AI965037 0.371 1.138 1.113 0.658 0.328 0.242 0.154 -0.161 -0.27 0.257 -0.013 -0.117

AI965042 -0.077 -0.682 -0.696 -0.065 0.064 0.84 0.516 0.696 0.432 0.873 0.289 0.191

AI965047 -2.765 -3.779 -2.306 -1.728 -3.807 -2.588 -1.639 -3.865 -1.881 -0.582 0.743 1.421

AI965120 0.153 1.223 1.455 1.373 1.001 0.581 0.282 0.843 0.025 0.048 -0.008 -0.246

AI965130 -0.282 0.467 0.761 0.863 0.579 0.127 -0.046 0.271 0.01 0.397 0.201 0.107

AI965182 0.826 -0.049 0.467 0.266 0.173 0.157 0.168 0.668 0.005 0.195 -0.039 0.042

AI965224 -0.778 0.545 0.045 0.341 0.173 -0.046 -0.563 -0.008 0.011 0.853 1.139 1.169

AI965225 0.385 0.1 0.366 0.443 0.177 0.728 0.128 0.679 0.557 0.91 0.227 0.078

AI965247 0.582 -0.198 -0.252 -0.683 -1.504 0.115 -0.041 0.044 -0.2 0.384 0.695 -0.128

AI965249 -0.916 0.059 1.887 2.029 2.632 2.428 1.793 1.964 1.421 1.408 1.236 1.455

AI965251 -0.702 -0.455 -0.115 0.356 1.284 1.235 1.701 2.703 0.916 0.719 0.669 -0.582

AI965312 -0.653 -0.311 0.166 -1.399 -0.769 -0.381 -0.562 -0.425 -0.181 0.539 0.838 0.764

AI965321 0.176 0.248 -0.321 -0.089 0.751 0.921 0.814 1.139 0.081 0.532 0.399 -0.422

AI974137 2.313 -2.212 -2.718 -2.913 -2.6 -1.181 -2.179 -1.366 -2.621 -2.786 -1.62 -2.028

AI974139 -0.093 -1.046 0.212 0.858 0.2 0.406 0.516 0.949 0.342 0.337 0.576 0.043

AI974149 -0.031 -0.04 1.084 0.61 0.767 -0.095 0.322 0.448 0.166 0.441 0.157 0.069

AI974174 -0.753 -0.031 -0.555 0.341 -1.775 -0.228 -0.379 -0.965 -0.494 -0.763 -0.059 1.16

AI974191 -0.413 -0.907 1.633 1.901 2.286 2.464 2.089 2.321 0.974 0.744 0.218 -0.394

AI974195 0.266 0.98 0.663 1.144 0.905 0.396 0.456 0.809 -0.223 0.42 0.598 0.389

AI974205 -0.396 -0.224 -0.26 -0.646 0.161 0.056 -0.21 -0.174 0.156 -0.19 0.462 0.587

AI974209 -0.775 -0.388 -0.578 -0.255 0.382 0.071 0.53 1.333 0.019 0.258 0.194 -0.291

AI979356 -0.382 -0.561 0.783 0.569 1.152 1.043 0.456 1.207 0.648 0.57 0.167 -0.054

AJ005026 -0.342 0.254 -0.072 0.343 0.378 0.355 0.217 0.708 0.468 0.094 0.385 0.193

AJ005936 -1.251 -0.65 0.418 0.774 0.996 0.108 0.469 0.665 0.688 0.731 0.434 0.266

AJ006310 -0.664 0.042 -0.077 0.124 0.057 -0.003 0.237 2.107 0.619 0.539 0.451 -0.207

AJ011112 -0.569 0.11 -0.195 -0.071 0.098 0.01 0.079 0.49 -0.141 0.357 0.723 -0.1

AJ011788 0.619 1.141 1.292 0.869 -0.37 -0.287 -0.066 -0.183 -0.485 -0.679 -1.158 -1.508

AJ011789 2.436 0.195 -2.58 -3.21 -3.959 -3.696 -4.103 -4.251 -4.599 -5.055 -4.566 -5.09

AJ011790 2.088 0.415 -1.765 -0.938 -3.901 -1.892 -1.495 -3.68 -1.577 -2.617 -3.243 -3.236

AJ132931 0.169 0.456 0.171 0.132 0.26 0.227 0.358 1.171 0.025 0.341 0.032 0.226

AJ236882 -5.67 -3.087 1.093 1.801 2.022 2.735 2.118 1.986 1.444 1.6 1.121 0.061

AJ242515 -0.633 -0.261 1.215 1.369 1.127 0.905 0.96 1.837 0.972 0.157 0.575 0.199

AJ243250 0.046 0.866 1.561 1.149 1.143 0.88 0.381 0.223 -0.523 -0.44 -0.716 -0.957

AJ243959 0.616 1.45 0.76 0.62 0.063 0.283 -0.269 -0.172 -0.385 -0.596 -0.902 -0.607

AJ245490 -0.638 -0.866 -1.139 -1.351 -0.244 -0.328 -0.398 0.7 0.443 1.163 0.828 0.197

AJ245491 -5.149 -5.056 -3.874 -3.209 -2.408 -0.59 -0.413 -0.294 0.033 0.759 0.643 0.604

AJ245492 1.179 0.721 -0.139 -0.565 -1.051 -0.923 -1.24 -1.464 -1.131 -1.083 -1.054 -0.907

AJ245493 -1.13 -1.228 -0.674 -0.241 -0.605 -0.424 -0.373 -0.311 -0.157 -0.119 0.355 0.446

AJ245962 -0.519 -0.315 -0.206 -0.361 -0.723 -0.113 -0.543 -0.681 -0.25 -0.084 0.272 1.063

AJ245964 -0.812 -0.402 -0.403 -0.893 -0.244 -0.147 -0.451 -0.013 0.141 -0.728 0.425 1.917

AJ245965 0.024 0.446 0.157 0.569 1.049 1.102 0.557 1.021 0.386 0.698 0.639 0.072

AJ249490 1.399 1.02 -0.788 -1.466 -3.217 -2.073 -3.416 -3.605 -3.504 -3.908 -3.409 -3.471

AJ249795 0.231 0.867 1.278 0.793 0.97 1.013 0.245 1.386 0.646 0.805 0.503 0.624

AJ250201 -0.465 0.997 1.175 0.866 -0.002 -0.156 -0.47 0.115 -0.253 0.246 -0.141 -0.323

AJ278244 0.02 -0.043 0.438 0.458 0.774 0.423 -0.084 0.201 0.756 0.786 0.87 0.863

AJ278268 -0.13 -0.951 -0.309 -1.138 -0.687 0.184 -0.251 0.066 -0.223 0.425 -0.219 -0.561

AJ286835 -0.492 0.227 0.486 0.832 0.48 0.209 0.063 1.143 0.247 0.35 0.796 -0.004

AJ290391 -0.311 0.368 0.397 0.625 0.558 -0.267 0.337 0.764 -0.007 -0.37 0.082 -0.231

AJ293862 -0.734 -0.999 -0.188 0.916 1.259 -0.079 0.066 0.086 0.055 -0.247 0.682 0.344

AJ299411 -0.186 0.183 0.007 0.103 -0.685 -0.563 -0.478 -0.525 -0.5 0.896 0.641 -0.304

AJ309314 -0.588 0.005 0.308 0.974 2.329 2.346 1.78 2.696 1.5 0.096 0.168 -0.141

AJ311846 -3.017 -1.827 -2.235 -1.521 -1.92 -1.408 -2.209 -1.976 -2.409 0.424 1.34 1.516

AJ315468 -0.367 -0.446 0.193 1.11 1.673 0.82 0.069 0.42 -0.145 -0.31 0.701 0.272

AJ317957 -2.203 -2.176 -1.523 -1.589 -2.157 -1.172 -1.53 -2.458 -1.631 -0.743 -0.589 1.57

AJ404970 -0.461 0.545 0.15 0.341 -0.173 0.253 -0.098 0.604 -0.008 0.108 0.582 0.946

AL591442 -1.908 -0.526 1.339 1.685 1.353 1.079 0.71 0.85 0.871 0.333 0.797 0.273

AW018514 1.112 1.204 -0.301 -0.336 -0.365 -0.185 -0.368 -0.364 -0.237 -0.616 0.118 -0.241

AW018635 -2.344 -2.123 -1.911 -2.042 -2.15 -1.245 -1.531 -0.845 -0.3 0.248 0.501 1.415

AW018718 0.013 1.052 1.283 1.191 -0.186 -0.227 -0.701 -0.599 -0.388 -0.254 -0.366 -0.507

AW018770 0.309 1.425 1.461 1.457 0.993 1.033 0.741 0.727 0.288 0.214 0.184 -0.129

AW018939 1.326 0.11 -1.478 -1.138 -0.635 -0.527 -0.444 -1.038 -0.69 -1.255 -0.477 -0.833

AW018941 -0.728 0.121 0.11 0.437 0.581 -0.125 -0.029 0.215 -0.378 -0.557 -0.543 -0.913

AW018949 -4.499 -3.927 -4.361 -3.394 -4.318 -1.436 -0.688 -0.633 0.188 0.714 0.229 -0.113

AW018967 -0.576 -1.951 -0.202 -0.545 -0.219 0.28 0.529 0.381 0.485 0.29 0.612 0.248

AW018972 -1.257 0.026 -0.563 0.341 0.305 -0.061 -0.407 -0.535 -1.048 -1.696 -1.781 -2.074

AW018979 1.316 1.397 1.538 1.077 0.986 1.217 0.358 0.425 -0.201 -0.103 -0.565 -0.503

AW018983 0.996 0.135 -0.616 -1.432 -1.763 -0.094 -0.783 -0.985 -0.968 -0.501 -1.357 -0.675

AW018998 -0.728 0.148 0.766 0.85 0.813 0.046 -0.219 0.193 -0.167 -0.36 0.147 -0.194

AW019011 0.996 0.168 0.484 0.346 0.103 0.414 0.517 0.134 0.12 0.123 -0.335 -0.327

AW019116 0.571 0.185 0.621 0.97 1.212 1.627 0.977 0.958 0.407 0.364 0.003 0.004

AW019142 -0.724 0.159 -0.25 0.046 -0.109 0.053 0.466 1.115 0.87 1.555 0.56 -0.099

AW019187 -0.874 -0.461 0.833 1.219 1.198 1.379 1.178 0.686 0.438 0.06 -0.141 -0.054

AW019238 -0.216 -0.052 0.308 0.259 0.529 0.395 0.511 1.461 0.615 0.015 0.247 -0.15

AW019266 -0.253 0.726 0.354 0.75 1.036 -0.332 0.482 1.434 0.373 -0.17 -0.141 -0.236

AW019275 -1.221 -2.211 -0.596 -0.737 -0.188 -0.437 -0.242 -0.974 0.297 -0.293 0.013 0.428

AW019276 -1.098 -0.992 -1.846 -1.07 -1.045 -0.521 -0.504 -0.084 -0.277 0.594 0.084 0.02

AW019294 -0.155 0.132 1.911 1.669 1.898 1.171 1.136 1.087 0.865 0.413 0.521 0.331

AW019321 -0.917 -0.62 -0.537 -0.049 0.294 0.836 0.931 1.233 0.546 -0.448 -0.683 -0.729

AW019421 -0.211 0.709 1.595 1.739 1.058 0.417 0.061 0.046 0.095 -0.43 -0.882 -1.063

AW019428 0.858 -0.408 -0.085 -0.179 0.143 0.441 0.384 1.001 0.571 1.033 0.444 0.054

AW019436 -1.357 -0.408 2.255 1.612 0.906 0.977 0.436 0.189 -0.404 -0.342 -0.48 -0.512

AW019444 1.178 0.296 -0.093 0.527 0.216 0.817 0.562 0.62 0.317 0.436 -0.006 0.025

AW019450 -0.462 -0.461 0.982 0.437 0.053 0.283 0.307 0.902 0.588 0.868 0.439 0.425

AW019482 -0.72 0.137 0.945 1.542 1.862 2.158 1.461 1.758 0.798 0.659 0.443 0.291

AW019523 -0.573 -0.073 0.747 0.856 1.286 0.354 0.241 1.115 0.44 0.269 0.261 0.17

AW019528 0.035 -0.242 -0.086 -0.026 0.45 0.944 0.965 0.978 0.559 0.383 0.23 0.085

AW019579 0.906 0.853 0.742 0.673 0.84 0.675 0.283 1.079 0.481 0.085 0.233 0.453

AW019613 -0.08 -0.118 -0.361 0.092 0.006 0.044 0.174 0.594 0.079 0.024 -0.144 -0.242

AW019629 0.552 1.003 0.951 0.653 -0.485 -0.455 -0.044 -0.804 -0.602 -0.966 -1.066 -0.81

AW019690 0.431 -0.371 1.104 1.592 1.316 1.465 1.042 1.728 0.764 0.509 -0.001 0.073

AW019691 0.071 -0.249 1.142 1.576 1.157 1.004 1.297 1.874 0.555 0.291 0.019 0.123

AW019720 -0.091 -0.326 -0.009 0.19 0.806 0.838 0.667 1.236 0.766 0.27 0.156 0.006

AW019723 1.518 0.81 0.182 0.215 0.294 0.507 0.335 0.668 -0.009 0.158 -0.011 -0.085

AW019725 0.578 -0.764 2.338 2.888 2.418 2.453 1.632 1.454 0.519 1.512 1.026 0.657

AW019740 -0.814 -0.001 0.605 1.051 0.851 0.495 0.202 0.344 -0.231 -0.289 -0.208 -0.379

AW019758 1.146 -1.189 -0.63 -0.49 -0.137 -0.093 -0.26 -0.313 -0.227 -0.629 -0.842 -0.534

AW019847 0.804 -1.903 -1.283 -1.385 -1.313 -0.793 -1.041 -1.684 -1.312 -1.547 -1.094 -0.316

AW058706 -0.473 1.331 1.138 0.922 0.508 0.207 -0.298 -0.106 -0.554 0.097 -0.173 -1.137

AW058759 -0.133 -0.173 0.809 1.078 1.562 0.345 0.234 0.663 0.397 0.104 0.373 0.014

AW058763 -0.035 -0.601 0.757 1.392 2.078 1.945 1.238 1.241 0.892 0.653 0.722 0.989

AW058797 0.257 -0.004 0.647 0.722 1.008 0.826 0.423 1.33 0.672 1.259 0.33 0.363

AW058802 -0.62 0.929 0.639 0.638 0.951 -0.043 -0.223 0.153 -0.166 -0.038 0.002 -0.004

AW058803 0.16 1.284 0.897 0.253 0.225 0.447 0.15 0.588 0.183 0.002 -0.066 -0.514

AW058804 -0.376 -0.412 0.82 0.917 1.528 0.88 0.502 0.927 0.398 0.406 0.861 0.736

AW058811 -0.754 -0.511 1.928 2.388 2.487 0.996 0.579 0.318 -0.344 0.012 -0.306 -0.693

AW058816 0.159 0.187 0.357 0.631 0.419 0.019 0.333 0.955 -0.057 0.549 0.158 -0.125

AW058828 -0.596 -0.16 0.431 1.085 1.181 0.457 0.275 0.518 -0.083 -0.478 0.537 0.131

AW058839 -0.698 -0.825 0.386 0.664 1.145 1.679 1.379 2.02 1.258 0.405 0.173 -0.151

AW058848 -1.052 0.186 3.297 2.22 2.094 1.557 1.237 1.749 0.962 1.064 0.499 0.263

AW058867 -0.019 0.25 0.637 1.037 0.693 0.169 0.39 0.537 0.218 0.375 0.227 0.029

AW058875 0.214 0.637 0.516 0.708 0.6 0.046 0.389 1.093 0.217 -0.139 0.384 -0.029

AW058902 1.172 0.241 0.639 0.704 0.437 1.285 0.666 1.256 1.021 1.039 0.401 -0.436

AW058967 -0.916 -0.32 -0.062 1.133 1.297 0.985 1.037 0.835 0.727 0.616 1.1 0.576

AW058992 -0.039 -0.188 -0.093 0.244 0.086 -0.093 0.467 0.636 -0.128 0.402 -0.015 -0.666

AW059007 -0.846 -0.118 1.549 1.923 1.99 0.899 0.536 1.01 0.102 0.318 0.281 -0.107

AW059030 0.104 -0.347 0.134 0.258 0.526 0.516 0.216 1.421 -0.122 -0.024 0.64 0.033

AW059046 -0.538 -0.237 0.072 0.478 0.647 0.491 0.194 0.961 0.279 0.83 0.348 0.21

AW059048 0.216 0.168 0.698 1.075 1.389 0.868 0.804 1.318 0.402 0.833 0.403 0.068

AW059069 0.311 1.188 1.583 1.983 1.633 2.62 2.126 2.5 1.704 0.55 -0.009 0.039

AW059073 0.966 0.634 0.312 0.25 0.454 1.015 0.87 1.402 0.45 0.118 -0.152 -0.638

AW059098 -0.006 -0.227 0.079 -0.481 -0.439 0.027 0.281 0.421 0.079 0.687 0.256 0.362

AW059102 -0.023 -0.968 0.309 -0.216 1.174 0.282 0.602 1.757 0.383 0.283 0.178 -0.06

AW059104 -0.452 -0.557 -0.719 -0.07 -0.082 0.753 0.438 1.768 0.401 0.533 0.671 0.06

AW059137 0.038 -0.282 0.947 1.012 1.947 1.203 0.678 1.243 0.535 0.249 0.289 0.012

AW059146 -0.048 0.489 1.663 1.671 1.844 1.612 1.307 1.608 0.424 0.593 0.344 0.388

AW059156 -0.661 -0.711 -0.209 0.756 1.612 1.994 1.079 1.573 0.915 0.75 0.686 0.608

AW059158 -0.081 0.08 0.469 0.673 0.771 -0.128 0.414 0.705 0.228 0.159 -0.186 -0.087

AW059217 -0.014 -0.236 0.313 -0.342 0.351 -0.25 0.435 1.328 0.246 0.191 0.87 -0.109

AW059220 0.235 -0.046 0.575 0.652 1.364 0.493 0.261 1.408 0.062 0.507 -0.006 -0.279

AW059234 -1.056 -0.709 -0.091 0.332 1.507 2.547 2.236 2.037 1.279 0.884 -0.048 -0.151

AW059266 0.158 0.215 0.188 0.286 0.696 0.945 0.241 1.054 0.253 0.621 0.474 0.113

AW059316 -0.006 -0.363 0.395 0.734 1.632 0.782 0.638 1.336 0.562 0.174 -0.001 -0.154

AW059366 -0.03 0.142 1.531 1.689 3.508 3.088 2.077 1.357 1.046 0.042 0.932 0.157

AW059389 0.865 -0.556 -0.368 0.033 0.041 0.781 0.49 0.744 0.57 0.722 0.288 -0.513

AW059478 -0.246 -0.09 0.507 0.402 0.485 -0.222 0.056 0.4 0.134 0.1 -0.138 -0.256

AW076546 1.451 1.47 0.702 0.14 -0.213 0.437 -0.097 -0.236 -0.667 -0.789 -1.396 -1.537

AW076647 -0.447 0.496 0.695 0.942 1.116 -0.095 0.636 1.256 -0.05 -0.183 0.158 -0.28

AW076659 0.034 -0.111 0.243 0.034 0.511 0.546 0.451 0.871 0.656 0.781 0.076 0.414

AW076666 0.053 -1.078 0.156 0.276 0.48 0.37 0.324 1.105 0.231 0.925 0.675 0.261

AW076688 -0.571 -0.687 -0.539 -1.223 -1.573 -0.367 -0.2 -1.827 -0.571 0.379 0.994 0.633

AW076692 1.202 1.157 0.038 -0.015 -1.312 -0.671 -0.926 -0.785 -0.88 -1.108 -1.44 -1.304

AW076798 -0.235 0.403 0.88 0.938 0.935 0.673 0.1 0.756 0.402 0.116 -0.485 -0.856

AW076882 0.499 0.884 0.72 0.945 1.007 0.535 0.291 0.62 -0.026 0.031 -0.557 -0.383

AW076961 1.075 0.522 0.16 0.213 -0.226 -0.523 -0.506 -1.517 -1.53 -1.377 -1.658 -0.503

AW076964 1.428 -2.313 -3.401 -3.23 -3.732 -1.666 -2.508 -3.539 -3.797 -4.05 -3.9 -3.539

AW077011 0.997 0.131 -0.048 -0.006 -0.025 -0.106 -0.256 -0.188 -0.127 -0.249 -0.212 -0.23

AW077025 0.89 -0.077 0.522 0.279 0.416 0.745 0.647 0.09 0.478 0.257 0.31 0.546

AW077063 -0.056 -1.141 -1.24 -0.326 -0.3 0.329 0.276 0.482 0.571 1.29 0.937 0.332

AW077142 -1.016 -2.732 -0.928 -0.065 -0.137 0.746 0.33 0.531 0.387 0.943 0.497 -0.203

AW077184 -0.201 -0.029 1.361 1.523 1.613 1.728 1.488 1.846 0.959 0.593 0.192 0.22

AW077190 0.074 -0.456 0.536 1.077 0.994 1.385 0.868 1.307 0.559 0.486 0.045 0.071

AW077193 -0.081 0.14 1.186 1.099 1.192 1.396 1.008 1.37 0.666 0.424 0.108 0.137

AW077237 -0.75 -0.075 -0.461 -0.083 -0.205 0.045 -0.234 0.189 0.167 0.051 0.636 0.364

AW077286 -0.713 -2.383 -1.037 -0.819 -0.578 0.427 0.207 0.088 0.387 1.103 0.776 0.126

AW077337 1.341 0.587 -0.82 -0.475 -0.489 0.067 -0.271 -0.258 -0.427 -0.683 -0.76 -0.452

AW077420 -0.42 -0.177 -0.273 -0.683 -0.373 -0.149 -0.491 -0.506 -0.078 0.239 0.413 0.808

AW077448 0.099 -0.101 0.247 0.454 0.277 0.451 0.318 0.55 -0.153 0.469 0.641 0.472

AW077480 1.112 -1.164 -2.006 -1.794 -1.841 -1.243 -1.727 -1.471 -3.097 -2.986 -2.648 -2.122

AW077743 0.49 0.492 -0.105 0.647 0.83 0.485 0.301 0.704 0.445 -0.082 0.031 -0.179

AW077755 -0.041 -0.315 -0.148 0.497 0.526 1.095 0.565 0.665 0.183 0.324 0.034 0.404

AW077778 0.372 1.039 1.834 1.258 0.908 0.016 -0.302 0.494 0.044 0.284 0.026 -0.031

AW077854 1.834 0.508 -1.713 -1.325 -3.11 -2.077 -2.814 -3.555 -3.362 -2.32 -2.268 -2.525

AW077940 -0.856 -0.226 0.373 0.554 0.555 0.273 0.446 1.243 0.733 0.174 0.514 0.69

AW077961 -2.047 -1.221 -1.763 -0.814 -0.336 0.25 0.242 1.024 0.522 0.434 -0.41 -0.603

AW077976 -0.703 -0.171 -0.006 0.488 0.64 0.46 -0.123 -0.024 -0.216 -0.065 -0.383 -0.408

AW077980 -0.685 0.621 0.55 0.739 1.647 -0.13 0.43 1.327 -0.28 -0.422 0.008 -0.117

AW078048 -0.932 -1.798 -0.404 -0.089 0.267 0.92 0.835 1.043 0.721 1.55 1.148 1.269

AW078116 0.282 0.095 0.469 0.446 0.505 0.278 0.346 0.749 0.143 0.447 0.009 0.162

AW078150 -0.798 0.206 0.541 0.831 1.182 -0.206 0.074 0.979 0.205 0.588 0.569 0.82

AW078163 1.376 0.135 -0.126 0.175 0.567 0.452 0.427 0.688 -0.125 0.042 -0.359 -0.21

AW078193 -0.438 0.926 1.48 0.658 0.052 -0.17 -1.025 0.036 -0.247 -0.447 0.041 -0.742

AW078222 0.119 1.179 0.953 0.446 0.678 0.269 0.203 0.864 -0.008 -0.011 -0.178 -0.307

AW078266 -0.078 -0.497 0.216 0.744 1.043 -0.03 0.47 1.069 0.415 0.355 0.564 0.132

AW078302 -0.465 0.127 1.287 1.056 0.895 0.3 0.306 1.026 0.363 -0.601 0.806 0.141

AW078306 0.154 0.721 1.511 1.672 1.524 1.603 1.117 1.374 0.991 1.262 0.454 0.458

AW078394 0.28 0.389 0.75 1.017 0.814 0.722 0.18 0.624 0.426 0.583 0.836 0.781

AW078404 -0.288 1.457 1.089 0.8 0.591 0.049 0.026 1.003 0.13 0.211 -0.074 -0.414

AW078445 -0.426 0.271 0.407 0.896 1.286 -0.01 0.14 1.529 -0.233 -0.064 0.999 0.102

AW115523 1.088 0.534 -0.01 -0.053 -0.389 -0.21 -0.878 -0.58 -0.639 -0.778 -1.035 -1.285

AW115560 0.891 1.376 1.756 2.224 1.903 1.805 0.949 1.514 0.489 0.359 -0.054 -0.395

AW115580 0.062 -0.657 -0.085 -0.53 -0.497 0.517 -0.027 0.157 0.026 0.581 0.514 0.591

AW115598 1.063 1.09 0.165 -0.035 0.123 0.669 0.136 0.334 0.176 0.035 -0.123 0.075

AW115602 0.466 -2.087 -0.839 0.005 0.057 0.807 0.364 0.572 0.579 1.171 0.673 0.673

AW115606 1.344 1.274 -0.467 0.341 0.086 -0.111 -0.313 0.174 -0.411 -0.683 -0.506 -0.369

AW115626 0.923 -0.746 -0.58 -0.225 -0.494 0.863 0.555 0.985 0.835 1.029 0.351 -0.409

AW115633 0.286 0.317 0.44 0.746 0.576 0.451 0.227 0.792 0.258 0.583 0.078 0.153

AW115638 0.745 0.585 0.262 -0.263 -1.417 -0.388 -0.35 -1.33 -0.996 -0.49 -1.111 -1.234

AW115640 -0.658 -0.291 0.162 -0.037 0.458 0.309 -0.187 -0.117 0.224 0.144 0.418 -0.09

AW115660 1.579 0.154 -1.622 -0.503 -0.817 -0.59 -0.384 -1.055 -0.48 -1.513 -0.474 -0.808

AW115702 0.163 0.232 0.522 0.899 0.828 0.165 0.161 -0.161 -0.216 -0.292 -0.356 -0.463

AW115718 0.813 0.414 0.976 0.468 0.673 0.611 0.202 0.169 -0.049 -0.185 -0.412 -0.486

AW115729 -0.093 0.141 0.011 0.38 0.662 0.779 0.735 1.331 0.41 0.561 0.726 0.167

AW115770 0.128 0.243 0.713 0.754 0.716 0.547 0.835 1.097 0.439 0.937 0.302 -0.027

AW115793 0.134 0.517 0.827 0.234 0.305 0.887 0.3 0.422 0.213 0.02 -0.22 -0.379

AW115799 0.94 0.937 0.824 0.933 0.551 1.09 1.025 0.933 0.419 0.363 0.018 0.005

AW115804 1.087 0.63 -0.508 -0.594 -0.546 -0.335 -0.417 -0.596 -0.232 -0.488 -0.566 -0.567

AW115809 -0.118 -0.698 -0.013 -0.078 0.132 0.904 0.536 0.751 0.46 0.608 0.349 0.205

AW115821 1.557 0.763 -0.552 -0.754 -0.739 0.042 -0.431 0.028 -0.523 -0.659 -0.611 -0.705

AW115824 -0.079 0.695 0.123 0.202 0.553 0.388 0.674 1.318 0.504 0.293 0.695 0.429

AW115841 -0.354 -0.589 0.284 0.956 0.847 1.033 0.96 1.085 0.658 0.7 0.346 1.139

AW115859 1.645 0.666 -0.914 -0.53 -0.261 -0.343 -0.834 -0.6 -0.724 -0.977 -0.959 -1.381

AW115873 1.082 1.112 1.131 1 0.896 1.318 1.024 0.44 0.244 0.394 0.136 0.08

AW115897 0.181 -0.533 -0.133 -0.214 -0.004 -0.083 0.168 0.65 -0.03 0.017 0.118 0.078

AW115906 0.708 1.252 0.985 0.565 -0.262 -0.253 -0.426 -0.368 -0.523 -0.599 -0.398 -0.666

AW115947 0.378 0.819 0.109 0.652 0.733 0.105 0.07 0.291 -0.082 -0.216 -0.628 -0.516

AW115956 0.119 -0.422 0.763 1.929 3.313 1.68 1.617 1.777 1.345 -0.159 -0.373 -0.527

AW115964 -0.019 0.878 0.487 0.878 0.676 0.162 0.211 0.729 -0.003 -0.08 -0.039 -0.071

AW115973 1.339 0.88 -0.879 -0.589 -1.264 -0.712 -0.754 -1.233 -0.455 -0.894 -1.323 -1.188

AW116023 2.409 0.207 -0.941 0.075 -1.074 -0.385 -0.204 -0.933 -0.739 -1.949 -0.75 -1.119

AW116032 0.548 0.744 1.127 0.645 -0.145 0.144 -0.366 0.008 0.413 0.042 -0.122 0.009

AW116039 -0.055 -0.295 0.189 0.181 0.877 0.581 0.187 1.28 0.467 -0.106 -0.044 -0.391

AW116075 1.269 1.205 1.827 1.985 1.811 1.349 0.586 -0.015 -0.933 -1.598 -1.821 -2.886

AW116090 0.746 1.291 1.488 1.336 0.826 1.496 1.09 1.48 0.806 0.486 -0.019 -0.023

AW116127 0.687 0.337 0.601 1.006 1.419 0.685 0.944 1.657 0.472 0.37 0.274 0.099

AW116147 0.117 -0.114 0.345 0.843 0.92 0.395 0.829 1.245 0.365 0.85 0.252 -0.072

AW116159 1.488 1.578 0.779 0.535 0.19 0.538 0.499 0.98 0.446 0.249 -0.429 -0.713

AW116199 0.814 1.747 1.383 0.467 -0.075 0.063 -0.46 -0.632 -0.567 -0.856 -0.766 -0.65

AW116206 1.449 -3.773 -3.24 -2.841 -3.433 -2.796 -3.238 -4.262 -4.089 -5.011 -4.462 -5.078

AW116214 1.19 0.268 -0.624 -0.099 -0.478 -0.118 -0.237 -0.288 -0.267 -0.065 -0.33 0.003

AW116226 0.287 0.543 0.858 0.704 0.877 1.48 0.697 0.744 0.338 0.342 -0.107 0.018

AW116228 1.262 -1.761 -2.119 -1.705 -2.334 -2.987 -3.65 -3.64 -4.47 -5.033 -5.052 -4.477

AW116232 0.592 0.462 0.205 0.64 0.944 0.457 0.336 0.769 -0.024 0.119 -0.222 -0.01

AW116237 1.084 0.276 -0.074 0.056 -0.535 -0.232 -0.379 -0.638 -0.694 0.079 -0.28 -0.543

AW116246 1.129 0.992 0.856 0.432 0.022 -0.206 -0.124 -0.239 -0.397 -0.95 -0.647 -0.967

AW116271 1.325 0.535 0.116 1.121 -0.11 -0.113 -0.387 -0.295 -0.208 -0.273 -0.13 -0.339

AW116281 0.3 0.737 0.558 0.762 0.916 1.116 0.826 1.739 0.978 0.305 0.012 -0.902

AW116284 1.688 -0.206 -0.422 -0.291 -0.975 -0.954 -1.167 -1.626 -1.205 -1.255 -1.011 -0.786

AW116302 0.901 0.588 0.197 0.67 -0.341 -0.258 -0.527 -1.119 -0.751 -0.897 -1.146 -1.234

AW116326 -0.573 -0.367 -0.313 -0.325 -0.426 -0.54 -0.152 -0.831 -0.1 -0.1 -0.04 0.742

AW116327 0.577 -0.348 -0.368 -0.407 -0.079 0.832 0.432 0.605 0.189 0.403 0.328 -0.458

AW116335 0.275 1.49 0.448 0.784 0.719 0.18 0.111 0.504 -0.135 -0.104 -0.282 -0.171

AW116347 0.761 1.234 1.281 0.733 0.639 0.759 0.361 0.583 0.238 0.03 0.087 0.002

AW116371 0.388 1.274 1.625 1.818 1.451 1.261 0.844 1.121 0.235 0.08 -0.079 -0.26

AW116374 0.124 0.481 0.866 0.714 0.828 0.871 0.782 0.763 -0.148 0.353 -0.116 -0.233

AW116386 -0.049 0.603 0.558 0.815 0.705 0.072 0.464 0.967 0.141 0.109 0.195 -0.153

AW116392 0.729 0.985 0.716 0.259 -0.448 -0.326 -0.468 -0.526 -0.248 -0.373 0.012 0.003

AW116402 1.066 0.444 0.065 -0.069 -1.057 -0.803 -0.762 -2.496 -1.213 -1.497 -1.206 -1.198

AW116409 -0.907 -0.61 -0.04 -0.153 0.134 0.655 0.251 0.482 0.36 0.512 0.101 -0.014

AW116414 1.933 1.471 0.758 -0.084 -1.78 -2.103 -2.741 -3.332 -2.848 -2.794 -2.393 -2.236

AW116420 1.455 1.152 -0.605 -0.822 -0.585 -0.442 -0.575 -0.622 -1.138 -1.092 -1.181 -1.063

AW116425 0.321 -0.637 0.278 0.85 0.885 1.145 0.691 0.932 0.193 0.474 0.027 -0.093

AW116431 0.683 1.004 0.947 0.98 0.637 0.449 0.288 0.407 -0.165 0.003 -0.072 -0.471

AW116453 -0.075 -0.592 -0.241 0.553 0.9 1.251 1.161 1.112 1.093 0.557 0.041 -0.288

AW116461 0.129 0.9 1.408 1.805 1.712 1.554 0.77 1.263 0.443 0.256 0.028 -0.315

AW116474 1.282 -0.968 -1.094 -0.857 -1.632 -0.531 -0.826 -1.72 -1.166 -1.616 -1.419 -1.74

AW116486 -0.473 0.045 0.73 0.476 1.028 -0.438 0.178 0.858 0.436 0.202 0.072 0.196

AW116489 1.35 -0.636 -0.185 -1.132 -3.283 -1.373 -1.47 -2.895 -1.823 -0.946 -3.801 -3.894

AW116490 1.432 1.407 -0.549 -0.79 -0.695 -0.141 -0.374 -0.423 -0.602 -1.04 -0.747 -1.05

AW116504 0.933 0.393 0.234 0.407 0.43 0.961 0.521 0.63 0.37 0.194 -0.359 -0.438

AW116521 0.679 1.152 1.518 1.212 0.109 0.304 -0.144 0.29 0.167 -0.271 0.01 0.013

AW116556 -0.129 0.516 0.423 0.458 0.499 0.636 0.239 0.317 -0.037 -0.086 -0.192 -0.404

AW116567 1.859 0.877 -0.153 -0.337 -1.023 -0.115 -0.513 -0.708 -1.15 -1.072 -0.972 -1.428

AW116573 1.854 -0.201 -2.067 -2.401 -1.422 -0.899 -1.579 -1.339 -1.817 -1.704 -1.46 -1.436

AW116597 0.856 1.257 1.225 0.748 -0.2 -0.11 -0.205 0.046 -0.102 -0.491 -0.451 -0.643

AW116601 0.186 0.207 1.063 1.001 0.955 0.32 0.297 0.793 0.121 0.583 0.617 0.508

AW116617 1.164 0.874 1.055 1.172 1.148 2.02 0.9 1.451 0.382 0.559 0.27 -0.002

AW116620 -0.701 -0.673 0.002 0.486 0.49 0.1 -0.162 0.179 0.049 -0.088 -0.088 -0.337

AW116628 1.2 0.397 -0.749 -0.745 -1.591 -0.707 -0.858 -1.647 -1.073 -1.173 -1.751 -2.269

AW116649 0.865 0.105 -1.39 -0.912 -1.147 -0.394 -0.646 -0.539 -0.381 -0.357 -0.151 0.05

AW116650 1.008 1.289 2.383 1.871 1.733 2.319 1.484 1.497 1.007 0.734 0.153 -0.116

AW116654 0.295 -0.367 -0.503 -0.621 0.42 -0.053 -0.171 0.67 -0.059 0.173 -0.543 -0.408

AW116681 1.529 0.742 0.269 0.265 0.231 0.512 -0.048 0.115 -0.373 -0.211 -0.604 -1.166

AW116685 0.972 0.716 -0.982 -0.33 -0.993 -0.331 0.013 -1.21 -0.48 -0.704 -0.554 -0.515

AW116722 0.525 0.542 0.007 0.417 0.614 0.377 0.554 1.037 0.342 -0.026 -0.199 -0.31

AW116726 -0.351 -0.017 0.796 0.813 0.59 -0.145 -0.177 0.122 0.005 -0.451 -0.668 -0.484

AW116727 1.263 0.576 -0.38 -0.248 -0.373 -0.043 -0.377 -0.467 -0.275 -0.813 -0.645 -0.474

AW116767 0.963 0.787 1.125 0.896 -0.053 0.344 0.164 -0.216 -0.173 -0.418 -0.775 -0.327

AW116777 0.483 0.564 0.783 0.465 0.718 0.643 0.46 0.704 0.443 0.209 -0.155 -0.311

AW116780 -1.142 -0.48 -0.991 -0.811 -0.835 -0.716 -1.04 -1.141 -0.994 1.119 0.401 -0.205

AW116809 1.244 1.555 0.081 -0.554 -0.574 0.029 -0.208 0.396 -0.005 0.12 -0.09 -0.773

AW116863 -3.941 -2.372 -2.632 -1.828 -3.077 -2.975 -2.703 -2.872 -1.808 -0.528 -0.293 0.644

AW116868 0.237 0.591 0.304 0.268 0.542 0.48 0.096 0.829 0.232 0.368 -0.072 0.055

AW116874 0.26 1.203 0.89 0.105 -0.179 -0.102 0.012 -0.321 -0.53 -0.22 -0.675 -0.509

AW116894 -0.225 0.64 1.084 0.928 1.076 -0.261 0.228 0.055 -0.089 -0.21 -0.461 -0.579

AW116915 0.468 0.606 0.639 0.707 0.557 0.248 0.419 0.443 0.041 0.092 -0.134 -0.255

AW116947 1.236 0.505 0.956 1.217 1.221 1.419 0.829 1.037 0.177 0.471 -0.094 -0.018

AW116961 -0.429 0.252 3.512 1.77 2.755 0.844 0.361 0.941 -0.002 -0.316 0.176 -0.174

AW116978 -0.312 -0.771 -0.522 -0.709 -0.091 0.037 0.257 0.961 0.14 0.23 0.677 0.208

AW116980 -0.046 -0.416 -0.388 -0.107 0.398 0.269 0.369 0.736 0.075 0.735 0.108 0.187

AW116994 1.53 0.934 0.34 1.073 0.471 0.341 0.128 0.099 -0.19 -0.755 -0.781 -0.933

AW117015 1.925 -1.043 -1.454 -0.865 -1.215 -0.517 -0.527 -1.445 -0.961 -1.169 -1.091 -0.89

AW117034 0.892 0.851 0.3 -0.015 0.122 0.204 -0.168 0.153 -0.292 -0.115 -0.406 -0.004

AW117039 0.662 0.307 0.668 0.286 0.739 0.316 0.406 -0.015 0.353 0.789 0.791 0.787

AW117050 0.237 -0.371 0.559 0.731 1.188 1.392 1.424 1.456 0.683 0.767 0.072 -0.531

AW117056 -1.271 -1.706 -1.154 -1.412 -1.451 0.151 0.257 0.997 1.223 1.241 0.872 -1.13

AW117076 -0.788 -0.032 0.368 1.124 1.434 0.635 0.431 1.525 0.822 0.522 0.35 0.111

AW117083 -0.975 0.212 0.938 1.226 1.508 0.754 1.013 1.41 0.439 0.558 0.499 -0.201

AW117094 -1.936 -2.566 -1.102 -0.582 -0.472 -0.16 -0.121 -0.03 -0.515 0.28 0.393 -0.334

AW117104 1.161 1.396 -0.141 -0.434 -0.464 -0.388 -0.283 -0.585 -0.833 -0.849 -1.308 -0.768

AW117105 0.204 -0.309 0.113 0.221 0.367 0.624 0.522 0.667 0.272 0.16 0.261 0.011

AW117140 0.48 0.797 0.683 0.669 0.741 0.556 0.183 0.48 -0.089 -0.235 -0.428 -0.648

AW117141 -0.08 -0.119 1.599 1.511 1.376 1.157 0.727 1.056 1.265 0.589 0.735 0.823

AW117146 -0.692 -0.108 -0.788 0.008 0.531 0.898 0.607 1.055 0.269 0.712 0.194 -0.069

AW117160 0.388 0.004 0.836 0.906 1.178 0.912 0.593 0.46 0.574 0.142 0.705 0.535

AW117161 1.127 0.774 0.597 0.32 0.622 0.434 0.446 0.731 0.127 -0.149 -0.316 -0.303

AW127725 -0.249 0.588 0.489 0.724 1.008 0.096 0.025 0.78 0.048 -0.173 -0.21 -0.073

AW127801 0.314 -0.316 -0.121 0.056 -0.103 0.978 0.378 0.669 0.864 0.894 0.486 -0.162

AW127926 0.003 1.013 0.873 0.574 -0.316 -0.32 0.004 -0.396 -0.046 -0.787 -0.061 -0.219

AW128002 -0.302 0.68 1.494 1.649 1.253 -0.149 0.084 0.553 -0.099 -0.015 0.238 -0.187

AW128153 1.168 1.028 0.75 1.014 0.746 1.113 0.461 0.406 0.008 0.193 -0.029 -0.559

AW128192 1.242 0.501 -1.287 -0.321 -0.44 0.08 -0.236 -0.274 -0.034 -0.548 -0.333 -0.653

AW128211 -0.266 -0.234 0.154 -0.416 0.363 -0.163 0.204 1.017 0.087 0.403 -0.314 -0.426

AW128244 -0.854 -0.754 -0.244 0.072 -0.318 0.052 0.358 0.277 0.225 0.888 0.697 0.756

AW128246 2.113 -1.006 -2.169 -2.199 -1.964 -0.922 -0.401 -1.425 -0.922 -1.679 -1.187 -1.656

AW128293 0.898 1.158 1.219 0.862 0.92 1.376 0.501 0.876 0.483 0.6 0.213 -0.585

AW128316 0.533 0.757 0.634 0.456 0.461 0.841 0.351 0.817 0.291 0.084 -0.097 -0.417

AW128332 1.415 -0.534 -0.297 0.083 0.315 0.786 0.029 0.273 -0.104 -0.07 -0.259 -0.418

AW128354 0.458 0.223 0.798 0.66 0.751 1.138 0.383 0.799 0.625 0.669 0.174 0.147

AW128372 -0.413 -0.823 -0.677 -1.099 -0.88 0.196 0.171 1.339 0.938 0.645 0.184 -0.491

AW128377 -0.088 -0.053 0.602 1.234 1.809 0.524 0.898 1.65 0.318 0.378 0.153 0.345

AW128379 -1.503 -0.336 -1.991 -0.74 -2.033 -0.576 -0.909 -1.605 -1.566 0.848 0.675 0.909

AW128382 -0.528 -0.464 -1.765 -0.396 -0.415 -0.084 0.178 0.665 0.51 1.028 0.294 0.28

AW128384 0.108 0.352 0.364 -0.174 0.091 -0.059 0.06 0.767 -0.057 0.067 -0.231 0.071

AW128413 0.744 0.198 0.701 0.257 0.598 0.514 0.451 1.35 0.585 0.37 0.263 -0.412

AW128428 -1.238 -2.825 0.046 0.605 1.181 1.452 1.416 1.442 0.173 -1.017 -1.803 -1.647

AW128619 0.543 0.606 1.385 1.183 1.088 1.038 0.815 1.049 0.201 0.303 -0.34 -0.783

AW128744 -1.007 -2.859 -1.569 -1.22 -0.937 0.313 0.035 0.282 0.202 1.024 0.813 0.298

AW133635 -0.73 -0.255 0.201 0.544 0.39 0.478 -0.221 0.878 0.458 0.709 0.123 -0.013

AW133729 0.401 0.685 0.655 0.481 0.331 0.471 -0.093 0.538 0.125 0.051 -0.233 -0.363

AW133803 0.254 -0.058 0.032 0.345 0.447 0.014 0.35 0.624 0.378 0.373 0.186 0.211

AW133873 1.159 -0.566 -0.843 -0.503 -2.152 -0.755 -0.415 -1.82 -0.562 -0.688 -1.255 -0.78

AW134000 1.869 -3.232 -2.957 -3.488 -3.722 -2.825 -4.167 -5.218 -4.834 -5.32 -7.339 -5.879

AW134011 -0.693 0.281 0.709 1.327 1.489 0.36 0.183 0.813 0.07 0.333 0.302 -0.091

AW134106 -0.512 -3.369 -3.237 -3.602 -4.37 -4.024 -4.196 -5.231 -4.961 -7.317 -6.735 -7.685

AW153364 0.194 1.017 1.793 1.419 0.771 0.601 0.061 0.09 -0.212 -0.285 -0.181 -0.359

AW153863 0.015 1.062 0.888 1.001 1.326 -0.055 0.098 1.013 0.141 0.155 -0.007 -0.238

AW154075 -0.155 -0.652 -0.842 -1.192 -1.06 -0.234 -0.248 -0.893 -1.195 -0.608 -0.198 1.188

AW154108 1.053 0.927 1.547 0.639 0.404 -0.142 -0.853 -0.406 -0.395 0.044 -0.054 -0.037

AW154137 1.096 1.221 -1.177 -0.207 -0.341 -0.237 -0.367 -0.256 -0.384 -0.364 -0.53 -0.435

AW154153 1.695 0.377 -1.018 -0.291 -0.087 0.312 0.188 0.265 -0.198 -0.333 -0.441 -0.674

AW154162 -0.196 1.067 0.372 0.935 0.654 -0.236 -0.219 -0.021 -0.195 -0.672 -0.306 -0.389

AW154176 1.711 0.948 -0.213 -0.455 -1.206 -0.534 -0.977 -1.441 -0.857 -0.953 -1.339 -1.037

AW154231 1.428 1.243 0.222 0.537 0.152 0.701 0.189 0.348 -0.029 0.108 -0.347 -0.233

AW154269 1.398 0.611 1.195 0.958 1.186 1.401 0.508 0.686 0.409 0.151 -0.036 -0.256

AW154283 0.729 1.14 1.173 1.22 1.287 0.854 0.571 1.29 -0.049 -0.021 -0.31 -0.657

AW154285 0.283 0.755 0.772 0.995 0.463 0.346 0.399 0.391 -0.088 0.259 -0.431 -0.233

AW154286 0.921 1.14 0.913 0.269 0.126 0.28 -0.026 0.46 0.33 0.231 0.037 -0.045

AW154302 0.175 1.214 0.817 0.174 0.167 -0.04 -0.034 0.666 0.074 -0.17 -0.221 -0.379

AW154320 0.952 0.134 -0.518 -0.031 -0.399 0.173 0.075 0.11 -0.011 -0.2 -0.576 -0.793

AW154324 1.144 0.506 0.111 0.113 0.177 0.715 0.447 0.97 -0.071 0.16 0.522 0.077

AW154375 -2.034 -1.766 -0.663 -0.291 0.245 1.085 0.658 1.755 0.705 0.023 -0.35 -0.686

AW154399 1.185 1.504 0.974 0.307 -1.874 -0.669 -0.233 -2.166 -0.646 -1.495 -1.532 -2.07

AW154402 0.362 1.142 0.838 0.698 0.78 -0.089 -0.09 0.18 -0.119 -0.495 -0.272 -0.427

AW154413 0.862 0.47 1.101 1.396 1.43 1.529 1.239 1.41 0.399 0.271 -0.08 0.057

AW154414 -0.098 0.387 0.774 0.743 1.154 0.213 0.457 1.152 0.089 0.296 0.058 0.102

AW154454 0.893 0.899 0.401 0.344 0.31 0.152 -0.201 -0.009 0.054 0.01 0.033 -0.244

AW154457 1.319 1.001 -0.087 -0.245 -0.236 0.229 -0.062 0.48 0.125 -0.106 0.137 -0.379

AW154468 -0.342 -0.932 -0.562 -0.633 -0.581 -0.276 0.304 1.138 0.262 0.285 0.593 0.406

AW154475 0.265 1.451 0.938 0.853 0.755 0.114 -0.039 0.026 -0.14 -0.563 -0.249 -0.546

AW154476 0.452 1.243 1.149 1.014 0.389 0.789 0.435 0.528 0.542 0.305 0.328 0.48

AW154483 -0.008 0.592 0.621 0.996 0.542 0.797 0.594 0.295 -0.143 0.018 -0.268 0.262

AW154496 1.143 0.71 -0.615 -0.55 -0.602 -0.582 -0.744 -0.655 -0.841 -1.259 -0.464 -1.182

AW154506 0.911 0.729 0.818 1.033 0.305 -0.072 -0.388 -0.177 -0.137 0.204 0.005 -0.171

AW154507 0.028 0.573 0.343 0.265 0.538 -0.254 0.317 0.818 -0.045 -0.506 -0.399 -0.431

AW154514 -0.395 0.101 1.26 0.7 1.042 0.418 0.337 0.624 0.178 -0.524 -0.034 -0.23

AW154516 1.194 1.318 0.046 0.03 -0.267 -0.012 0.149 -0.107 0.021 -0.134 -0.105 -0.305

AW154517 1.385 0.479 -0.996 -0.472 -1.375 -0.303 -0.611 -1.297 -0.949 -1.202 -0.958 -0.943

AW154540 0.896 0.853 1.53 1.265 1.643 1.562 0.912 1.645 0.873 1.022 0.293 0.163

AW154574 0.949 -0.009 -0.064 0.174 0.312 1.366 1.139 1.302 0.353 -0.188 -0.874 -0.985

AW154587 1.792 0.269 -0.58 -0.202 -0.273 -0.045 -0.524 -0.563 -0.515 -1.046 -0.648 -0.679

AW154642 -0.033 0.08 0.401 0.609 1.588 0.957 0.955 1.947 0.855 0.468 0.174 -0.414

AW154647 1.185 1.14 0.501 -0.021 -0.476 -0.125 -0.429 -0.355 -0.2 -0.015 -0.234 -0.405

AW154701 1.04 -0.012 0.411 0.414 -0.011 0.179 0.125 -0.405 -0.334 -0.4 -0.504 -0.391

AW154707 0.88 -0.493 -0.297 0.474 0.264 0.764 0.688 1.051 0.408 0.708 0.055 0.091

AW154716 -1.197 -0.184 -1.041 -0.576 -0.364 0.245 0.27 1.144 1.325 1.127 0.896 0.699

AW154725 0.945 1.027 1.404 1.534 1.442 1.922 1.227 0.995 0.371 -0.098 -0.397 -0.116

AW154726 -0.988 -0.38 -0.295 0.149 -0.383 -0.174 -0.205 -0.497 -0.316 -0.058 0.01 0.584

AW154782 -1.778 -0.775 -1.734 -0.781 -0.494 -0.023 0.258 0.696 0.657 1.141 0.584 0.313

AW154792 0.565 -0.707 -0.073 0.311 0.759 0.616 0.688 1.214 0.469 0.314 0.007 -0.307

AW165108 0.566 0.342 1.388 1.127 1.614 2.253 1.434 1.999 1.012 1.015 0.523 0.814

AW165150 -1.089 -0.023 1.427 1.321 2.062 2.211 1.078 1.628 0.703 0.591 0.312 0.285

AW165160 0.941 -2.896 -1.247 -2.895 -0.329 0.183 0.063 -0.081 -0.552 -0.412 -0.574 -0.396

AW165221 0.961 0.715 0.915 1.065 1.049 0.346 0.69 0.683 0.102 0.169 -0.199 -0.028

AW165240 0.257 1.152 1.488 1.474 1.365 0.905 0.371 0.847 0.003 -0.287 -0.314 -0.108

AW165251 1.006 -0.65 -0.471 0.19 0.599 0.474 0.35 0.667 0.137 -0.447 -0.354 -0.339

AW165272 0.336 0.06 0.401 0.887 1.288 0.791 1.006 1.17 0.401 0.304 -0.103 -0.241

AW165301 -0.464 0.498 0.736 0.873 0.711 -0.261 0.379 0.268 -0.18 -0.267 -0.249 0.042

AW165310 1.087 0.328 0.017 -0.446 -1.489 0.082 -0.503 -1.317 -0.851 -1.073 -1.548 -1.22

AW165313 0.211 1.173 0.711 0.702 0.604 -0.09 0.089 0.57 -0.355 -0.06 -0.61 -0.444

AW165362 0.304 -1.184 0.459 1.312 1.702 0.84 0.794 1.936 0.357 0.654 -0.258 -0.57

AW165417 0.12 0.884 1.787 2.045 2.015 1.164 0.775 1.768 0.595 0.505 -0.232 -0.578

AW170850 0.012 0.141 0.224 0.473 0.655 0.091 0.146 0.207 -0.029 -0.296 -0.531 -0.269

AW170860 1.591 -3.303 -3.424 -2.933 -3.497 -1.938 -2.953 -4.659 -3.741 -5.338 -4.994 -4.085

AW170898 0.34 0.706 0.471 1.125 1.136 0.249 0.446 0.729 0.321 0.162 -0.147 0.127

AW170915 0.542 1.247 1.481 0.801 0.56 0.447 0.03 0.038 -0.388 -0.534 -0.634 -0.889

AW170941 0.961 0.321 -0.961 -0.817 -0.575 -0.359 -0.434 -0.691 -0.638 -1.117 -1.162 -1.218

AW170954 -0.644 -0.246 -0.246 0.059 0.283 0.68 0.297 0.515 0.468 0.51 0.678 0.487

AW170975 0.296 0.239 0.463 0.615 0.485 0.596 0.405 0.28 0.154 0.018 0.087 0.004

AW170991 0.414 0.933 0.658 0.872 0.389 0.39 0.053 0.06 -0.344 -0.464 -0.747 -0.449

AW171011 0.32 -0.569 0.953 1.037 1.08 1.518 1.208 0.945 0.948 0.336 -0.114 -0.163

AW171012 0.84 0.668 0.155 0.405 0.521 1.242 0.457 0.98 0.344 0.205 -0.083 -0.264

AW171049 1.619 -2.866 -1.865 -2.807 -3.899 -1.919 -1.944 -4.515 -3.745 -3.747 -5.879 -3.391

AW171072 -0.984 -0.475 0.038 -0.319 -0.201 -0.067 0.097 -0.46 0.334 -0.065 0.411 0.312

AW171085 1.304 0.445 0.225 0.238 0.512 0.61 0.372 0.662 0.236 0.01 -0.392 -0.626

AW171089 -0.18 -0.155 0.499 0.466 0.679 0.668 0.54 0.699 0.298 0.511 0.168 0.035

AW171143 0.458 0.757 0.761 0.674 0.676 0.37 0.324 0.523 -0.009 0.303 -0.138 -0.036

AW171172 -0.189 0.162 0.197 0.306 0.651 0.221 0.075 0.558 -0.161 0.09 0.531 0.169

AW171190 0.132 0.125 0.215 0.671 0.921 0.488 0.495 0.787 0.046 0.377 0.034 0.166

AW171194 0.852 1.014 0.941 0.331 -0.192 0.168 -0.008 0.37 -0.051 -0.173 -0.376 -0.271

AW171204 1.829 -0.057 -0.544 -0.195 -1.712 -0.703 -0.395 -1.05 -0.884 -0.878 -1.242 -1.261

AW171207 0.371 0.868 1.165 0.654 0.235 -0.288 -0.328 0.218 0.026 -0.188 -0.463 -0.389

AW171211 0.778 1.126 1.244 1.056 0.574 0.792 0.369 0.675 0.047 -0.119 -0.669 -0.612

AW171224 0.312 0.739 0.796 0.543 0.163 0.238 0.332 0.284 -0.191 -0.181 -0.542 -0.277

AW171228 -0.034 -0.208 1.343 1.244 1.08 1.389 0.999 1.205 0.906 0.933 0.543 0.409

AW171254 0.464 -0.553 0.21 0.504 0.612 0.955 0.476 0.665 0.413 0.486 0.221 0.226

AW171255 -0.01 0.855 1.727 1.58 1.637 -0.06 0.169 -0.129 -0.179 -0.628 -0.674 -0.362

AW171260 0.381 0.077 -0.427 -0.154 0.043 0.557 0.499 0.498 0.136 -0.414 -0.58 -0.524

AW171268 0.31 0.526 1.07 0.747 0.454 1.221 0.524 0.88 0.478 0.744 0.351 0.1

AW171271 1.664 1.07 0.343 -0.052 -0.402 -0.243 -0.687 -0.567 -1.024 -0.674 -0.969 -0.797

AW171290 1.199 1.645 1.675 1.097 0.251 0.338 0.086 0.663 0.216 0.002 -0.348 -0.192

AW171303 0.494 1.253 1.126 1.175 0.608 -0.193 0.205 0.349 -0.096 0.213 0.04 -0.417

AW171308 0.045 -0.321 0.018 -0.013 0.474 0.715 0.408 0.345 0.274 0.258 0.158 -0.086

AW171350 1.248 1.013 0.57 0.506 0.028 0.403 -0.153 -0.214 -0.144 -0.16 -0.602 -0.568

AW171363 1.096 0.017 -0.221 0.298 0.173 0.018 -0.156 -0.396 -0.569 -0.203 -0.261 -0.123

AW171367 0.452 0.272 0.154 0.507 0.808 0.712 0.217 1.368 0.436 0.289 -0.271 -0.79

AW171388 0.997 0.937 -0.221 -0.69 -0.308 -0.032 -0.383 -0.445 -0.523 -0.818 -0.374 -0.115

AW171390 -0.351 0.054 -0.449 -0.241 -0.264 -0.116 -0.16 -0.477 -0.418 -0.196 0.462 0.403

AW171396 1.116 0.564 0.37 0.324 0.166 0.259 -0.079 -0.26 -0.1 -0.146 -0.033 0.213

AW171447 0.667 0.164 0.611 0.125 0.573 0.439 0.197 1.374 0.402 0.225 0.222 -0.33

AW171454 -0.283 -0.063 0.018 0.295 0.679 1.254 0.515 1.25 0.621 0.857 0.376 0.249

AW171467 1.311 0.937 -1.581 -1.835 -2.169 -0.61 -1.341 -1.494 -0.83 -1.456 -1.773 -1.317

AW171471 -0.482 -1.335 -0.506 -0.657 -0.258 0.694 0.218 0.416 -0.065 0.03 -0.32 -0.548

AW171479 -1.391 -1.518 -1.069 -0.516 -1.096 -0.507 -1.242 -0.989 -0.469 0.222 0.383 0.646

AW171480 1.179 0.433 -0.128 -0.329 -0.287 0.068 -0.117 -0.167 -0.088 0.058 -0.116 -0.26

AW171484 1.155 0.056 -0.178 -0.797 -1.04 0.52 -0.201 0.268 -0.014 -0.303 -0.543 -0.349

AW171505 -0.723 -0.158 0.297 -0.701 -0.146 -0.134 -0.556 -0.285 -0.155 -0.422 -0.298 0.812

AW171522 1.28 1.091 0.7 0.663 0.44 0.3 0.092 0.137 -0.206 -0.308 -0.285 -0.879

AW171527 1.087 0.596 0.162 -0.191 -0.872 -0.524 -0.283 -1.113 -0.945 -1.678 -0.891 -0.938

AW171529 -0.112 1.018 0.655 0.55 0.508 -0.162 0.444 0.561 0.164 -0.219 -0.338 -0.307

AW171553 0.482 0.639 0.954 0.763 0.75 0.478 0.117 0.528 -0.143 -0.024 -0.33 -0.256

AW171554 0.818 0.41 0.443 0.859 0.981 0.872 0.841 1.563 0.585 0.477 -0.311 -0.518

AW171576 0.862 0.887 0.644 0.048 -0.2 0.491 0.061 -0.104 -0.117 0.015 -0.222 -0.374

AW171595 1.281 0.594 0.761 0.442 0.754 1.172 0.575 0.807 0.383 0.138 0.339 0.056

AW171604 0.608 1.401 1.186 1.595 1.835 0.656 0.699 0.496 -0.433 -0.71 -0.69 -0.316

AW171610 0.648 1.157 0.717 0.865 0.995 0.76 0.391 0.733 0.118 -0.498 -1.472 -1.665

AW173869 1.828 1.359 1.466 0.412 -0.882 -0.886 -1.453 -1.676 -1.255 -1.13 -0.905 -1.002

AW174275 1.48 -1.004 -0.824 0.967 0.171 -0.543 0.557 -0.923 -0.811 -2.882 -2.11 -2.221

AW174328 -0.615 0.976 1.027 0.676 -0.363 -0.612 -0.658 -0.895 -0.616 -1.02 -0.849 -0.973

AW174421 -0.562 0.348 1.362 0.922 0.607 0.061 -0.033 0.14 -0.229 -0.146 -0.15 0.484

AW174486 -0.087 1.139 0.834 1.085 0.775 -0.043 0.278 0.353 0.252 -0.568 -1.792 -1.291

AW174544 0.217 -0.882 -0.416 -0.291 -0.411 0.522 0.033 0.571 0.138 0.804 0.596 0.276

AW174595 1.272 -1.568 -1.208 -1.05 -0.746 0.028 -0.299 -0.103 -0.397 0.239 -0.168 0.219

AW174685 0.107 0.579 1.26 1.003 1.497 1.407 0.709 0.962 0.541 0.565 0.205 0.07

AW174733 1.082 0.901 1.574 1.111 0.473 0.167 -0.074 -0.244 -0.323 -0.803 -0.456 -0.008

AW174734 0.651 1.306 2.28 1.029 0.474 0.051 -0.012 -0.138 -0.155 -0.572 -0.39 -1.09

AW174857 0.29 -0.631 -0.05 -0.074 0.383 0.505 0.36 1.174 0.454 0.642 0.457 0.329

AW174887 -0.282 0.635 0.886 0.909 1.184 0.015 0.339 1.237 0.293 0.34 0.276 -0.102

AW175139 -0.872 -0.355 -0.974 -0.231 -0.24 -0.104 -0.144 0.478 0.343 0.677 0.19 0.196

AW175150 -0.145 0.731 0.608 0.698 0.311 -0.326 0.07 0.363 -0.054 -0.042 -0.032 -0.333

AW175474 0.663 0.741 0.367 0.421 1.28 0.87 0.419 0.896 0.12 -0.376 -0.352 -0.251

AW175480 0.345 -0.109 0.185 0.187 -0.407 0.096 0.067 0.76 0.487 0.601 0.414 0.037

AW175486 0.154 -0.374 -0.066 0.469 0.707 0.462 0.211 0.439 -0.101 0.203 -0.188 -0.299

AW175518 0.196 1.263 1.285 1.23 1.027 0.29 0.262 1.011 0.028 0.112 0.026 0.043

AW175546 -0.601 -0.1 0.308 0.322 0.043 0.238 -0.071 0.579 0.133 0.223 0.705 0.747

AW175553 1.061 0.694 0.602 0.879 0.533 0.743 -0.022 -0.054 0.213 0.183 -0.218 0.046

AW184197 -0.121 0.059 0.254 0.378 0.313 -0.003 -0.033 0.53 -0.219 -0.272 -0.368 -0.109

AW184237 -2.052 -1.684 0.479 0.079 0.66 0.798 0.595 0.363 1.142 0.284 0.738 0.428

AW184334 -0.319 -0.02 -0.087 0.633 0.903 -0.025 0.138 0.522 0.323 0.419 0.268 0.171

AW184672 0.086 1.248 1.289 0.945 0.827 -0.024 0.165 0.605 -0.04 0.094 -0.158 -0.144

AW202603 -1.511 -1.898 -0.93 -0.636 -0.409 0.233 -0.064 0.081 0.152 0.726 0.583 0.455

AW202620 1.051 -0.048 0.121 0.362 0.474 0.286 0.284 0.671 0.248 0.667 0.384 0.23

AW202926 -0.028 0.784 1.333 1.064 1.092 0.494 0.392 0.487 0.19 0.019 -0.283 -0.564

AW202947 -1.019 -0.323 -1.246 -0.985 -1.012 -0.166 -0.531 -0.875 -0.295 0.927 0.532 0.569

AW203020 -0.119 0.073 0.2 0.359 0.478 0.52 0.101 0.371 0.217 0.18 0.603 0.296

AW203026 0.741 -0.768 -1.06 -0.546 -1.031 -0.172 -0.164 -0.84 -0.449 0.136 -0.032 0.053

AW203038 -0.059 0.351 -0.203 -0.148 0.441 0.361 0.34 0.717 -0.241 -0.36 -0.183 -0.433

AW203061 -1.304 -0.998 -0.442 0.148 0.713 1.429 0.707 1.075 0.895 1.033 0.507 0.258

AW203129 0.196 -0.695 -0.284 -0.071 -0.154 0.054 0.226 0.279 0.314 0.088 0.675 0.444

AW203148 -0.973 -2.026 -1.075 -0.416 -0.234 0.492 0.46 0.634 0.523 1.189 0.95 0.528

AW203151 0.578 0.55 1.365 1.15 1.147 2.004 1.441 1.395 0.912 0.887 0.548 0.191

AW203156 0.495 2.18 1.15 0.953 0.1 -0.298 -0.353 0.201 -0.54 -1.056 -1.188 -0.982

AW231998 0.369 0.955 1.376 1.182 1.357 1.014 0.866 1.464 0.295 0.183 -0.341 -0.268

AW232003 -0.38 -0.255 1.567 1.959 1.653 2.21 2.064 1.948 1.125 0.661 0.161 0.267

AW232020 -2.569 -2.335 -3.261 -2.127 -2.903 -0.649 -1.642 -2.548 -0.871 1.088 0.764 0.95

AW232047 0.162 0.368 1.299 0.968 0.63 -0.195 0.116 -0.195 -0.147 -0.225 -0.261 -0.238

AW232071 -0.086 -1.143 -0.733 -0.705 -0.77 -0.405 -0.111 0.256 -0.212 0.572 0.274 -0.082

AW232088 0.841 0.341 -0.611 -0.374 -0.792 -0.018 -0.189 -0.586 -0.567 -0.163 -0.295 0.275

AW232241 -0.089 0.129 0.047 0.222 0.233 1.098 0.441 1.182 0.638 0.768 0.663 0.428

AW232262 0.244 0.868 1.114 0.746 0.138 -0.289 0.461 0.101 0.109 -0.107 -0.425 -0.531

AW232264 0.443 -0.58 -0.003 -0.223 -0.559 -0.255 0.023 -0.476 -0.22 0.017 -0.023 0.851

AW232289 0.433 -0.016 0.114 0.429 0.775 0.566 0.705 0.886 0.349 0.242 0.091 -0.012

AW232296 0.447 1.06 1.453 1.484 1.437 1.446 1.522 1.477 0.718 1.064 0.46 -0.095

AW232317 0.78 0.297 -0.24 0.194 0.682 0.687 0.832 1.388 0.678 0.631 0.29 0.14

AW232425 -0.013 0.166 0.371 0.071 0.097 -0.448 -0.176 -0.23 -0.195 0.307 0.496 0.069

AW232435 0.644 0.616 0.426 0.362 0.253 0.831 0.497 0.966 0.474 0.834 0.413 0.335

AW232464 1.587 0.399 -0.349 0.011 0.296 0.954 0.103 0.041 -0.594 -0.834 -1.607 -1.477

AW232471 -0.509 -0.271 0.135 1.05 1.079 0.311 0.073 0.323 0.045 -0.114 -0.287 -0.218

AW232489 0.933 1.208 0.651 0.714 0.884 0.607 0.559 0.737 0.368 0.363 -0.13 0.04

AW232589 1.127 -0.885 -0.917 -0.851 -0.865 -0.419 -0.473 -0.676 -1.088 -0.888 -1.277 -0.915

AW232627 1.073 0.833 2.196 2.255 1.786 2.237 1.956 1.376 0.474 0.525 0.195 0.267

AW232641 0.629 -0.723 0.217 0.707 1.293 1.914 1.536 1.577 1.116 1.052 0.429 -0.164

AW232642 1.07 0.213 0.535 0.572 0.597 0.663 0.405 0.49 -0.013 -0.093 -0.342 -0.429

AW232844 -3.529 -2.511 -1.069 -1.098 -0.558 -0.873 -1.008 -1.582 0.108 -0.785 0.187 -0.003

AW232853 -0.71 0.545 0.407 -0.404 0.12 0.004 -0.161 -0.002 -0.029 -0.697 0.425 -0.062

AW232866 -1.023 -0.546 -0.786 -0.074 0.631 2.158 1.541 1.99 1.424 0.46 -0.095 -0.226

AW232975 -0.373 -2.874 -0.874 -0.479 -0.155 0.745 0.639 0.615 0.359 0.734 0.424 -0.085

AW233059 -0.728 -0.245 -0.831 -0.522 -0.579 -0.517 -0.375 -0.565 -0.322 0.241 0.096 1.3

AW233144 -0.01 0.402 0.551 0.852 0.818 0.541 0.637 0.773 0.181 0.319 0.356 0.094

AW233556 -0.737 -0.039 0.017 0.097 -0.296 -0.403 -0.43 -0.285 -0.021 -0.225 0.043 0.807

AW233564 0.852 -3.294 -3.229 -3.365 -2.646 -2.309 -2.979 -2.878 -3.898 -4.108 -3.754 -3.53

AW233578 0.066 -0.61 0.567 -0.08 1.002 1.086 0.199 -0.178 -0.092 -0.188 -0.551 -0.135

AW233586 -0.114 0.677 0.161 0.532 0.429 -0.106 0.25 0.911 -0.139 0.129 -0.032 -0.018

AW233684 -0.2 0.016 0.098 -0.351 -0.607 -0.335 -0.233 -0.323 -0.092 -0.478 -0.269 1.442

AW233688 -0.357 -1.439 -1.201 -1.372 -1.137 -0.106 -0.315 -0.264 -0.241 0.754 0.344 0.581

AW233689 0.36 0.274 0.995 1.173 0.84 0.594 0.389 0.708 0.256 0.293 -0.177 -0.289

AW233726 0.106 1.328 0.791 0.194 -0.037 -0.274 -0.389 -0.066 -0.045 -0.389 -0.039 -0.347

AW233729 0.635 0.924 1.224 0.826 0.592 0.689 0.217 0.669 0.443 0.682 0.468 0.344

AW279630 0.099 -0.042 -0.632 0.081 0.128 -0.113 0.085 0.482 0.172 0.317 0.544 0.203

AW279837 -2.401 -2.601 -0.686 -0.867 -0.009 -0.09 -0.67 -1.003 0.346 -0.393 0.46 0.3

AW279908 0.672 1.295 0.741 -0.269 -1.085 0.024 -0.351 0.093 0.054 -0.023 -0.459 -0.014

AW279985 1.203 -0.098 0.258 0.185 0.795 0.619 0.512 1.388 0.367 0.61 0.473 0.196

AW280006 0.09 0.319 0.487 0.602 1.234 1.633 1.37 1.66 0.867 1.026 0.626 0.284

AW280046 0.294 0.76 0.787 0.698 -0.069 -0.109 -0.003 -0.354 -0.288 0.06 -0.389 -0.067

AW280152 -0.138 -0.035 -0.291 1.18 0.712 0.174 -0.05 0.237 0.054 0.027 -0.049 0.533

AW280158 0.916 0.204 -0.382 -0.345 -0.14 -0.04 -0.094 0.388 0.294 0.389 0.001 -0.256

AW280171 0.079 0.878 1.108 1.232 1.475 1.04 0.945 1.458 0.332 0.711 0.375 -0.171

AW280174 0.87 -0.103 0.08 0.687 0.604 1.356 1.242 1.233 0.643 0.884 0.415 0.257

AW280659 -0.503 1.147 1.155 0.693 0.525 -0.231 -0.043 0.659 -0.147 -0.426 -0.533 -0.182

AW281219 0.341 -0.868 -0.066 0.243 0.54 0.726 0.378 1.288 0.465 0.608 0.631 0.27

AW281249 -0.963 -0.636 -0.621 -1.172 -1.456 -0.465 -0.548 -1.574 -0.562 0.528 0.576 0.804

AW281293 -0.893 -1.451 -0.856 -0.712 -0.959 -0.325 -0.183 -1.134 -0.451 -0.204 -0.102 0.77

AW281340 0.434 1.061 1.596 1.22 0.675 0.527 0.231 0.478 0.318 -0.051 -0.157 -0.299

AW281416 1.293 0.085 0.329 0.566 0.412 0.192 -0.211 -0.18 -0.316 0.234 -0.306 -0.308

AW281444 -0.849 -0.423 -0.301 -0.229 -0.044 0.496 0.035 0.745 0.381 0.76 0.22 -0.334

AW281574 -0.364 -0.491 0.221 0.648 0.66 -0.027 0.135 0.515 -0.128 0.514 0.255 0.479

AW281646 0.03 0.884 1.149 1.438 0.841 0.748 0.273 0.726 0.004 0.233 0.127 0.164

AW281650 0.395 -0.017 0.485 -0.173 0.206 0.245 0.398 0.257 0.312 0.321 0.208 1.137

AW281931 0.195 1.091 1.751 0.99 1.3 1.683 1.808 2.73 1.257 0.765 0.301 -0.103

AW282001 -0.074 0.996 0.622 0.959 0.842 0.143 -0.316 0.004 -0.163 0.256 0.227 0.652

AW282014 -1.081 -0.44 -0.941 0.066 -0.202 -0.052 -0.596 -0.253 -0.868 -0.098 0.113 1.25

AW282022 -0.345 0.448 0.881 0.756 0.711 -0.211 0.061 0.04 0.195 0.286 0.099 -0.236

AW282035 0.377 -0.11 -0.097 -0.109 -0.349 0.184 0.499 -0.244 0.109 0.191 0.372 0.922

AW282065 1.206 -1.398 -2.554 -2.628 -2.351 -1.725 -2.649 -2.895 -2.678 -3.401 -3.268 -2.962

AW282142 0.317 -0.822 1.506 1.124 2.397 1.256 0.986 2.554 0.568 0.586 -0.098 -0.414

AW305388 0.553 0.168 0.443 0.643 0.877 1.888 1.101 1.388 0.946 1.128 0.938 0.51

AW305456 -0.233 -0.214 -0.336 0.472 0.491 0.742 0.653 0.266 0.646 0.303 0.325 0.104

AW305460 0.065 -0.668 -0.675 -0.483 -0.637 0.302 0.467 0.294 0.448 0.788 0.699 0.468

AW305462 0.421 0 0.585 0.565 0.322 0.704 0.492 0.879 0.265 0.389 -0.042 -0.058

AW305598 1.364 0.137 -0.001 -0.248 -0.219 0.115 -0.202 0.149 -0.216 -0.211 -0.11 -0.507

AW305840 -0.491 -0.356 -0.932 -1.162 -0.869 -0.409 -0.393 -0.64 -0.391 0.036 0.335 1.135

AW306050 0.124 -0.909 -0.997 -1.829 0.367 0.405 -0.212 0.804 0.226 0.141 -0.63 -0.598

AW306102 0.74 1.437 0.892 0.385 0.102 0.104 -0.078 0.437 -0.044 -0.191 0.03 -0.07

AW306107 1.057 1.458 2.09 2.065 1.75 1.318 0.388 0.803 -0.001 -0.345 -0.923 -1.164

AW342711 0.508 -0.05 -0.042 1.87 1.368 -0.159 -0.377 -0.285 -0.515 -0.595 -0.612 -0.251

AW342745 1.109 0.724 0.163 0.303 0.834 1.161 0.392 0.599 0.164 0.292 -0.009 0.133

AW342762 -0.174 0.54 1.771 1.505 1.597 2.172 1.592 1.808 1.451 0.82 0.543 0.356

AW342840 0.739 -0.073 0.092 0.139 0.384 1.054 0.764 0.81 0.294 0.063 -0.137 -0.572

AW342850 0.844 1.331 1.112 0.808 0.552 0.714 0.569 1.262 0.275 0.295 -0.059 0.033

AW343324 -0.004 0.199 0.592 0.629 0.609 0.435 0.42 1.155 0.283 0.495 0.512 0.554

AW343508 0.249 0.318 0.471 0.047 0.663 0.561 0.214 0.925 0.137 0.332 -0.055 0.053

AW343567 -0.282 0.234 0.786 0.616 1.126 0.024 0.758 1.342 0.09 -0.096 0.237 -0.222

AW343655 0.768 0.707 1.229 0.911 0.739 1.115 0.482 0.397 0.096 0.011 -0.133 0.002

AW343764 1.17 0.703 0.983 1.197 1.298 1.631 1.137 1.283 0.636 0.37 -0.515 -1.036

AW343865 0.456 0.599 0.999 0.622 0.511 0.838 0.116 0.639 0.137 0.202 -0.164 -0.233

AW343882 0.664 0.685 1.047 1.037 0.717 0.438 0.363 0.509 -0.128 0.064 -0.449 -0.467

AW343883 0.373 0.24 0.551 0.566 0.89 1.023 0.477 1.217 0.45 0.045 -0.234 -0.461

AW343911 -0.114 -1.189 -0.055 0.133 0.529 0.566 0.399 1.256 0.611 0.682 0.188 -0.321

AW343922 1.156 -1.703 -1.979 -1.476 -1.214 -0.594 -1.065 -0.931 -0.878 -0.449 -0.759 -0.508

AW343985 0.676 0.906 1.24 2.366 3.043 2.724 2.107 1.825 1.317 -0.153 -0.26 -0.711

AW343989 -1.334 -1.42 -0.581 -0.238 0.284 0.058 0.042 0.834 1.027 1.354 0.637 0.215

AW344020 -0.509 1.088 0.972 1.342 1.013 -0.309 0.379 0.502 -0.125 -0.528 0.064 -0.242

AW344022 1.271 0.287 -0.499 -0.647 -0.285 0.01 -0.114 -0.486 -0.384 -0.192 -0.629 -0.336

AW344023 -0.728 -0.036 0.097 1.118 0.956 -0.451 -0.139 -0.636 -0.105 -1.127 -1.415 -1.233

AW344030 1.055 0.334 -0.406 -0.003 0.097 0.652 -0.055 0.488 0.058 0.092 -0.232 -0.089

AW344043 1.545 1.541 0.894 0.82 0.832 1.091 0.412 0.715 0.071 0.102 -0.204 -0.563

AW344046 0.873 0.133 -0.311 -0.636 -0.747 -0.226 -0.738 -1.097 -1.121 -0.229 -0.4 0.214

AW344056 1.308 1.104 -0.298 -0.561 -0.7 -0.394 -0.882 -1.052 -0.839 -1.23 -1.038 -0.797

AW344075 1.428 -2.213 -2.493 -1.675 -2.414 -1.312 -1.389 -2.697 -1.922 -3.31 -2.504 -2.583

AW344113 0.091 0.172 1 0.771 0.897 0.909 0.586 1.296 0.317 0.281 -0.148 -0.02

AW344134 1.057 1.184 0.578 -0.034 -0.308 -0.152 0.372 0.267 -0.135 -0.009 -0.36 -0.067

AW344143 0.27 0.507 0.371 0.702 0.512 0.155 0.4 0.572 0.111 0.242 0.142 -0.029

AW344187 0.185 -0.179 -0.122 0.272 1.148 1.294 1.173 1.682 0.792 1.046 0.255 0.113

AW344188 0.894 1.143 1.469 1.2 0.399 0.758 0.238 0.51 -0.226 -0.103 -0.242 -0.533

AW344202 0.972 1.505 1.089 0.725 -0.046 0.367 -0.389 -0.475 -0.6 -0.632 -0.517 -1.921

AW344255 0.409 0.639 0.828 0.945 0.859 0.828 0.619 0.692 0.465 0.237 -0.002 -0.322

AW344260 1.089 0.706 -0.525 -0.217 -0.29 0.218 0.256 0.584 0.324 0.265 0.271 -0.409

AW419481 -0.558 0.018 -0.305 0.14 0.531 0.517 0.27 0.809 0.191 0.518 0.573 0.774

AW419638 -0.539 -0.446 -0.264 -0.202 0.038 -0.018 0.053 -0.281 0.373 -0.17 0.34 0.384

AW420284 -0.707 0.086 1.145 1.406 1.771 0.826 0.378 0.515 -0.194 -0.102 0.206 -0.077

AW420304 -0.403 0.384 0.391 1.028 1.635 -0.048 0.159 0.749 -0.017 -0.076 -0.226 -0.206

AW420314 0.385 0.698 1.157 1.016 1.558 1.498 1.127 1.542 0.539 0.385 0.235 0.004

AW420405 -0.071 -0.023 0.186 0.047 0.649 0.501 0.328 0.902 0.125 0.178 -0.003 0.148

AW420476 -0.207 0.017 1.64 1.499 1.477 1.001 1.065 1.827 0.769 0.771 0.652 0.532

AW420509 0.611 1.387 1.783 1.157 0.963 0.988 0.545 0.148 0.066 -0.548 -0.167 -0.199

AW420705 -0.372 -0.078 0.27 0.464 0.984 -0.113 0.442 0.959 0.266 0.249 0.412 0.311

AW420717 1.25 1.2 1.056 1.301 0.287 0.397 0.228 0.172 0.058 -0.424 -0.866 -0.685

AW420720 -1.617 -1.541 -1.361 -1.208 -1.811 -0.647 -0.423 -0.63 -0.126 0.734 0.584 -0.002

AW420722 0.435 1.226 0.829 0.418 -0.139 -0.295 -0.38 -0.363 -0.352 -0.043 -0.113 -0.226

AW420737 0.303 0.139 0.179 0.448 0.063 0.297 0.425 0.282 0.482 0.174 0.499 0.482

AW420829 -0.25 -0.035 -0.435 -0.482 1.248 0.651 1.046 0.975 0.34 0.058 0.331 0.207

AW420849 -0.32 -0.158 -0.423 0.024 0.067 0.432 0.146 0.048 0.444 0.381 0.334 0.595

AW420883 -0.084 0.051 0.154 0.313 -0.145 0.392 -0.194 0.254 0.283 0.661 0.289 -0.389

AW421022 -1.389 -1.786 -1.083 -0.061 0.489 1.613 1.407 1.551 1.189 0.905 0.751 0.069

AW421050 0.621 2.134 0.453 0.601 0.623 0.553 -0.206 -0.01 -0.65 -1.242 -1.216 -0.864

AW421096 -0.622 -0.134 0.036 -0.583 -1.01 -0.265 -0.247 -0.267 -0.284 0.034 0.753 0.321

AW421172 -0.081 0.036 -0.268 0.755 0.219 0.32 0.166 1.056 0.125 0.761 0.275 0.124

AW421191 -1.226 -0.49 -0.224 0.924 1.42 1.404 1.054 0.995 0.458 -0.003 -0.19 -0.238

AW421309 -0.122 0.398 0.321 0.5 0.544 0.231 0.19 1.307 0.613 0.281 0.215 -0.162

AW421436 1.679 0.731 -0.409 -0.955 -1.93 -1.966 -1.806 -3.551 -2.246 -3.567 -4.003 -3.458

AW421939 -5.597 -6.185 -5.674 -4.972 -4.629 -3.232 -2.978 -2.079 -0.6 0.315 0.385 -0.22

AW421941 1.955 -0.689 -1.343 -0.921 -0.96 -0.216 -0.806 -1.365 -1.052 -1.471 -1.637 -1.156

AW422010 1.794 1.917 0.438 -0.188 -1.51 -1.231 -1.644 -2.43 -2.169 -2.328 -2.958 -2.831

AW422167 0.731 -0.04 0.759 1.17 1.098 1.125 0.506 0.501 -0.146 -0.48 -0.834 -0.624

AW422278 -0.024 0.724 1.243 0.39 0.071 -0.097 -0.523 0 0.135 -0.254 -0.149 -0.652

AW422582 -0.076 0.744 0.563 1.024 0.804 0.204 -0.127 -0.137 0.069 -0.332 -0.581 -0.557

AW422922 -0.19 0.227 0.55 0.56 0.542 0.724 0.091 0.686 0.929 0.86 0.764 0.417

AW422929 1.644 -0.883 -1.754 -1.58 -1.564 -0.59 -1.229 -1.146 -1.477 -1.924 -2.023 -1.726

AW423211 -1.426 -1.134 1.503 1.609 1.428 1.211 0.814 0.129 0.303 0.299 -0.244 -0.272

AW454605 0.279 -0.635 0.1 0.587 0.981 0.837 0.464 0.239 0.509 0.579 0.7 0.553

AW454617 -0.549 0.479 1.151 1.328 1.002 0.716 0.574 0.596 0.144 0.368 -0.085 -0.039

AW455045 -2.069 -1.071 -2.143 0.05 -1.339 -0.448 -0.834 0.347 0.521 1.114 0.45 0.282

AW466488 0.751 1 1.801 1.346 1.138 0.967 0.84 0.983 0.187 0.19 0.099 -0.305

AW466509 0.628 0.29 -0.333 0.205 0.295 0.679 0.251 0.769 0.262 0.518 0.539 -0.117

AW466547 0 0.844 0.585 0.399 0.388 0.523 0.13 0.355 0.133 -0.032 -0.045 0.149

AW466584 0.658 -0.589 -0.562 -0.169 -0.126 0.56 0.654 0.906 0.203 0.193 -0.023 -0.104

AW466697 0.163 -0.143 -0.06 0.627 1.025 0.778 0.86 0.591 0.428 -0.105 -0.251 -0.303

AW466751 0.084 0.827 1.088 0.973 1.271 0.781 0.704 0.886 0.336 0.657 0.268 0.079

AW466858 1.178 1.585 1.46 1.015 -0.308 -0.377 -0.48 -0.672 -1.026 -0.656 -0.969 -1.381

AW510270 -0.052 -0.371 -0.433 -0.383 -0.395 -0.115 -0.338 -0.402 0.01 -0.119 0.237 0.697

AW566530 -0.34 1.288 0.814 0.503 0.042 -0.141 -0.218 -0.001 -0.359 -0.816 -0.628 -0.914

AW566851 1.424 1.352 2.148 2.218 1.897 2.049 1.666 1.407 0.71 0.566 0.012 -0.275

AW567098 -0.6 -0.207 -0.045 0.895 1.161 1.016 0.767 1.139 0.346 1.237 0.84 0.235

AW567130 -0.533 -0.575 1.898 2.066 2.706 1.28 2.222 2.362 1.631 0.057 0.043 -0.127

AW567292 -0.627 0.157 -0.008 0.259 0.294 0.015 -0.209 0.375 0.101 0.795 0.96 0.636

AW567345 0.624 0.916 1.29 1.332 0.75 0.073 0.493 0.541 -0.015 -0.075 -0.27 -0.27

AW567517 -0.749 -0.53 -1.515 -0.462 0.14 0.264 0.136 0.788 0.319 0.167 0.183 0.876

AW567528 0.356 1.032 2.052 1.919 1.707 1.959 1.026 1.204 0.782 0.636 -0.105 -0.307

AW594981 -1.293 1.091 1.113 0.773 0.379 -0.096 -1.062 -0.226 -0.082 0.236 0 -0.507

AW595094 -0.055 -0.22 -0.715 -1.119 -0.556 0.063 -0.391 -0.507 -0.307 -0.253 0.253 1.34

AW595163 -0.281 0.604 1.283 1.406 1.503 2.125 1.462 1.608 0.654 1.041 0.477 0.301

AW595789 -0.21 -1.286 -0.297 -0.382 -0.204 0.914 0.48 0.762 0.761 1.357 0.84 -0.115

AW777320 -1.1 -1.251 -2.176 -1.766 -2.285 -0.829 -1.21 -1.728 -0.461 1.47 0.849 1.216

AW777326 -0.408 0.122 -0.085 0.353 0.157 0.304 -0.126 0.36 0.004 0.298 0.443 0.049

AW777332 1.016 0.832 0.545 0.641 0.034 0.457 0.007 0.423 -0.043 0.173 -0.087 -0.298

AW777355 0.753 0.203 0.736 0.664 0.679 1.237 0.919 0.885 0.721 0.543 0.231 -0.008

AW777364 -0.072 -0.774 -0.934 -0.29 -0.193 0.462 0.318 0.754 0.335 0.843 0.082 0.333

AW777378 0.036 -0.307 -1.174 -0.382 -0.065 0.42 -0.079 0.796 0.208 0.238 0.037 0.365

AW777430 0.613 -0.148 0.717 0.675 0.637 1.325 0.884 0.479 0.697 0.712 0.467 0.014

AW777445 0.893 1.011 0.417 0.803 0.299 0.522 0.495 0.322 -0.187 -0.231 -0.553 -0.922

AW777460 -0.406 -0.023 0.729 1.145 1.211 1.027 1.171 1.208 0.383 0.053 -0.333 -0.038

AW777467 0.815 0.361 0.312 0.39 0.555 0.759 0.559 0.284 0.047 -0.044 -0.192 -0.074

AW777479 0.049 -0.186 0.12 0.715 0.884 -0.048 0.359 1.185 0.444 0.262 0.262 -0.189

AW777535 -0.408 -0.081 0.782 0.864 1.022 1.426 1.239 1.671 1.012 1.29 0.861 0.564

AW777539 0.462 0.017 0.315 0.164 0.389 0.693 0.783 1.47 0.955 1.126 1.004 0.234

AW777561 -0.751 0.344 -0.508 0.027 -0.076 -0.055 -0.357 0.476 0.626 1.305 0.412 -0.147

AW777691 1.231 0.159 0.326 -0.046 0.253 0.883 0.285 0.991 0.431 0.972 0.573 0.061

AW777717 -0.26 0.367 0.522 -0.229 -0.13 0.487 0.178 0.824 -0.37 -0.414 0.754 0.271

AW777769 -0.243 -0.136 1.301 2.744 3.642 1.661 1.352 1.63 1.275 -0.515 -0.495 -0.193

AW777836 -1.107 -1.465 -1.334 -0.982 -1.213 -0.543 -0.044 0.416 0.685 0.914 1.129 0.5

AW777843 1.079 1.597 0.454 0.074 -0.843 0.024 -0.426 -0.597 -0.495 -0.801 -0.979 -0.774

AW777876 1.003 0.842 0.983 0.806 0.403 0.547 0.241 0.682 0.255 0.644 0.173 -0.164

AW777903 0.152 0.285 0.528 0.388 0.451 0.658 0.689 1.402 0.47 0.273 0.748 0.449

AW777906 0.28 -0.091 -0.353 -0.119 -0.147 0.704 -0.072 0.399 0.319 0.425 0.375 0.065

AW778179 0.308 -0.115 -0.143 -0.24 -0.255 -0.109 0.15 1.203 0.796 0.798 0.824 0.035

AW826304 -1.031 -0.077 -1.014 -0.413 -1.015 -0.183 -0.518 -0.906 -0.616 -0.684 -0.261 1.5

AW826449 -0.167 -0.481 0.095 0.485 0.748 0.221 0.705 1.194 0.337 0.515 0.751 -0.065

AW826482 -0.755 0.545 -0.445 0.312 -0.027 0.115 0.054 0.6 0.385 0.604 0.757 0.937

AW826487 1.992 0.014 -1.667 -1.659 -2.124 -0.611 -0.701 -2.197 -1.354 -2.326 -1.403 -1.156

AW826500 -0.471 0.19 1.579 1.403 0.996 1.471 0.904 1.046 0.549 0.847 0.475 0.573

AW826550 -0.447 -0.205 -0.089 -0.308 0.31 0.615 0.654 1.052 0.672 0.244 0.636 0.817

AW826646 -0.423 -1.077 -0.193 -0.166 0.365 -0.258 -0.061 -0.347 0.277 0.253 0.522 0.262

AW826653 -0.593 -0.604 -0.399 -0.24 -0.109 -0.415 0.02 -0.455 0.282 -0.254 0.367 0.351

AW826723 -0.195 -1.229 -0.851 -0.38 -0.188 0.518 0.107 0.264 -0.079 0.545 0.275 0.48

AW826726 -0.395 -0.4 0.189 1.233 0.056 -0.444 0.256 -0.672 -0.643 -0.406 -0.823 1.03

AW826907 -0.448 -1.272 -0.307 -0.741 -0.22 -0.241 -0.183 -1.127 0.158 -0.453 0.422 0.22

AW827044 -0.213 -0.031 0.221 0.013 -0.746 -0.221 -0.145 -0.707 -0.084 0.186 0.261 0.754

AY007304 0.939 0.191 0.161 0.524 0.421 0.979 0.664 1.472 1 1.444 0.823 -0.052

AY007434 -3.23 -1.089 0.313 0.389 0.479 1.025 0.255 0.584 0.6 0.98 0.986 0.608

AY007990 -0.608 -0.548 0.527 1.177 1.086 0.759 0.708 1.865 0.567 1.075 1.531 0.413

AY008836 -0.811 -0.735 -1.049 -0.212 0.15 0.323 0.34 1.645 0.148 0.727 0.314 0.86

AY017308 -0.808 0.235 0.33 0.476 1.175 0.352 0.469 1.762 0.836 0.169 0.921 0.336

AY017309 -1.071 -1.343 -0.731 -0.465 0.184 -0.169 -0.201 -0.096 0.057 -0.183 0.656 0.583

AY026507 1.134 1.436 1.375 0.976 1.575 1.622 0.943 1.301 0.496 0.569 0.2 -0.434

AY029402 -0.471 -0.761 0.617 0.669 -0.418 -0.265 0.076 -0.945 -0.242 0.281 0.747 0.537

AY029527 -0.174 -0.863 0.398 0.28 -0.097 0.795 0.341 -0.083 1.029 0.829 0.22 -0.052

AY029529 0.687 1.573 1.064 0.867 0.031 0.512 -0.089 0.891 0.673 0.317 -0.421 -1.255

AY029577 0.977 -0.366 0.613 0.622 0.359 0.308 0.094 -0.228 -0.391 -0.086 -0.67 -0.748

AY029808 -0.242 -0.338 0.414 1.08 1.201 0.212 0.686 1.673 0.498 0.414 0.818 0.4

AY034614 0.129 0.971 0.334 1.19 1.263 0.68 0.912 1.229 0.561 -0.025 -1.001 -1

AY045753 -0.49 -0.086 -0.084 0.658 1.002 0.45 0.158 0.502 -0.258 -0.101 -0.254 -0.024

AY050507 -0.589 0.13 1.223 1.089 1.576 1.483 0.492 1.223 0.505 0.334 0.325 0.202

AY057057 0.411 0.189 0.399 0.644 1.005 0.528 0.108 0.682 0.09 -0.074 -0.144 -0.402

AY057058 1.451 -0.277 -1.309 -0.527 -1.699 -0.864 -0.23 -1.978 -0.616 -1.085 -1.244 -0.57

AY057095 -0.437 0.676 2.997 2.712 2.818 1.017 1.3 2.482 0.545 0.658 0.487 0.149

BE015668 -0.182 1.074 1.08 0.947 0.827 0.062 0.468 1.818 0.313 0.286 -0.556 0.062

BE016083 -0.035 -0.098 0.035 0.456 0.474 0.431 0.197 0.631 0.356 0.278 0.189 0.181

BE016113 -0.567 -0.727 0.356 0.707 0.922 0.386 0.314 0.631 0.093 0.257 0.161 -0.05

BE016123 0.806 0.394 0.197 0.285 0.404 0.955 0.567 0.504 0.155 -0.318 -0.791 -0.664

BE016164 1.607 1.31 -0.3 -0.383 -0.42 -0.169 -0.106 0.608 -0.002 -0.451 -0.525 -0.439

BE016292 -0.647 0.336 0.408 0.931 1.567 0.287 -0.142 1.182 0.769 0.137 0.056 -0.326

BE016354 -0.281 0.824 0.741 0.73 0.835 -0.01 -0.049 0.944 0.295 0.328 -0.048 -0.483

BE016510 0.394 0.231 1.453 0.653 0.719 1.141 0.573 0.59 0.002 0.517 -0.087 0.095

BE016522 -0.557 -1.183 -1.225 -0.75 -0.324 0.292 0.181 0.683 0.274 0.702 0.552 -0.021

BE016756 0.305 0.23 0.875 1.128 1.19 0.695 0.763 0.628 0.536 0.434 0.148 -0.11

BE017363 0.96 -0.295 -0.053 0.634 0.925 -0.187 -0.127 0.363 -0.019 -0.011 0.227 -0.311

BE017477 1.274 1.173 1.633 1.043 0.793 1.059 0.866 0.967 0.727 1.326 0.815 0.075

BE017542 0.198 0.658 1.045 1.212 0.215 0.092 0.608 0.938 0.095 0.397 -0.065 -0.581

BE017549 -0.464 0.275 0.391 0.543 0.726 -0.141 -0.215 0.495 0.232 0.046 0.099 -0.224

BE017551 1.268 -2.541 -2.35 -2.217 -2.396 -1.927 -2.569 -2.748 -1.702 -2.977 -2.953 -2.908

BE017652 -1.536 -0.098 0.239 0.418 1.231 1.86 1.504 1.459 0.999 0.557 0.271 -0.957

BE017795 1.779 -1.516 -0.513 -2.2 -2.122 0.03 -1.015 -1.44 -0.559 -0.314 -0.427 -0.46

BE017831 0.538 0.104 0.372 0.222 0.359 0.432 0.344 0.809 0.055 0.45 0.032 -0.251

BE017895 -1.018 -2.981 -1.196 -1.049 -0.586 0.427 0.113 0.434 0.204 1.092 0.592 0.25

BE200552 -0.674 -1.039 0.077 -0.394 0.096 0.382 0.538 1.188 0.455 0.794 0.822 0.709

BE200802 -0.085 -0.108 0.348 3.046 3.583 0.269 1 1.22 0.131 -0.595 -0.369 -0.231

BE200811 1.244 1.355 0.051 -0.396 -1.075 -0.163 -0.715 -0.216 -0.532 -0.969 -1.025 -0.872

BE200900 -0.459 0.059 -0.275 -0.145 0.133 -0.177 -0.02 0.661 0.33 0.589 0.56 -0.103

BE201102 -0.578 -0.002 0.328 -0.584 -0.775 -0.235 0.285 1.231 0.15 0.147 -0.153 -0.326

BE201151 -0.659 -0.26 -0.538 -0.086 0.038 0.066 -0.003 0.817 0.371 1.208 0.578 -0.458

BE201182 0.008 0.333 1.443 1.519 0.831 1.214 1.411 1.714 0.522 0.295 0.474 0.436

BE201395 -2.225 -4.034 -2.193 -3.84 -4.285 -1.064 -1.929 -4.284 -2.203 -0.365 0.497 2.327

BE201398 -0.619 0.545 0.508 0.58 0.563 0.361 0.566 1.091 0.799 0.096 0.286 -0.376

BE201470 -3.167 -1.166 0.12 0.73 0.907 1.468 0.849 0.831 0.873 0.891 0.373 0.364

BE201475 0.179 0.423 2.574 2.595 2.318 0.907 0.997 1.851 0.906 0.184 -0.171 -0.336

BE201596 -0.281 0.592 0.524 0.277 0.73 1.772 1.511 1.459 0.765 0.759 0.341 0.42

BE201677 -0.662 1.211 3.703 2.578 0.621 0.317 0.03 -0.09 -0.047 0.186 0.675 0.159

BE201733 0.476 0.19 0.174 0.612 1.345 2.044 1.315 2.221 1.258 1.26 0.594 -0.144

BE201769 -0.324 -1.017 -0.783 0.033 1.123 1.183 1.279 1.592 0.877 0.607 0.656 0.942

BE201771 1.544 0.786 -0.991 -0.963 -0.675 -1.069 -1.526 -1.314 -1.67 -0.595 -0.448 -1.417

BE201992 0.373 0.877 1.143 0.556 -0.1 -0.183 -0.275 -0.781 -0.407 -0.724 -0.661 -0.563

BE202178 -0.455 -0.141 0.193 0.763 0.568 0.544 0.551 0.972 0.549 1.096 0.624 0.693

BE202229 0.522 -0.773 0.196 0.605 0.882 1.213 1.009 1.1 0.681 0.48 -0.081 -0.179

BE556841 -0.368 0.253 0.912 0.831 1.004 0.228 0.107 0.662 0.213 -0.258 0.647 0.161

BE556846 0.061 -1.876 0.188 0.374 0.121 0.574 0.708 1.133 0.569 0.447 0.391 -0.112

BE556860 0.1 0.477 -0.408 -0.266 -0.365 -0.281 -0.19 0.71 0.141 0.227 0.003 0.032

BE557106 1.834 -0.668 -0.134 -0.944 -1.026 -0.384 -0.554 -0.75 -0.529 -1.095 -1.168 -0.941

BE557115 -0.035 1.025 2.097 2.263 1.617 0.521 0.387 0.596 0.108 -0.347 -0.253 -0.564

BE557308 0.017 -0.636 0.212 0.433 0.339 1.201 0.781 0.964 0.625 1.055 0.615 0.198

BE557659 0.146 0.119 0.209 0.521 0.239 0.274 0.172 0.952 0.346 0.857 0.353 0.145

BE558061 -0.099 0.286 0.105 -0.236 0.339 -0.172 0.295 0.679 0.012 -0.174 -0.116 0.425

BE558184 0.257 -0.282 0.299 -0.122 0.33 0.72 0.446 0.675 0.458 0.463 0.055 -0.018

BE605273 -0.283 -0.062 -0.172 0.329 0.25 -0.038 0.23 0.66 0.254 0.527 0.28 0.449

BE605276 -0.11 0.188 0.016 0.019 -0.366 -0.212 0.014 0.648 -0.062 0.448 0.734 0.191

BE605308 0.811 0.531 1.466 1.511 1.174 1.37 0.579 0.402 0.015 -0.711 -1.254 -1.466

BE605522 0.614 0.081 -0.54 -0.227 -0.479 0.131 -0.091 -0.086 -0.272 0.638 0.193 0.04

BE605606 0.276 1.497 1.379 1.407 1.026 0.933 0.439 0.973 0.135 0.144 0.124 -0.05

BE605707 0.613 0.707 0.642 0.029 -0.631 -0.793 -0.346 -0.976 -1.318 -0.673 -0.651 -0.218

BE605721 1.023 1 0.816 0.814 0.596 1.134 0.562 0.986 0.575 0.396 0.148 0.009

BE605766 -0.173 0.309 0.738 1.079 0.856 0.166 0.349 0.623 0.224 0.666 0.222 0.121

BE605810 0.634 0.683 0.696 0.387 0.316 0.451 0.111 0.321 0.192 0.058 -0.108 -0.231

BE605880 1.461 0.71 0.952 0.299 -0.406 0.441 0.075 0.116 -0.562 -0.299 -0.958 -0.984

BE605975 0.907 0.582 0.67 0.275 -0.479 0.072 -0.463 -0.58 -0.289 -0.16 -0.398 -0.466

BE605983 -0.36 -0.136 0.467 1.178 1.275 0.096 0.412 1.169 0.213 0.249 0.26 -0.065

BE606048 -0.153 -0.514 0.6 0.466 0.701 0.515 0.998 0.58 0.779 0.838 0.155 0.385

BE606074 0.053 0.259 0.728 0.368 0.538 0.454 0.765 1.043 0.531 0.125 -0.146 -0.346

BE606087 -0.052 -0.401 -0.179 0.094 0.115 0.046 0.345 1.013 0.465 0.889 0.18 -0.148

BE606152 -0.55 -0.017 -0.217 0.158 1.158 0.562 0.227 1.464 0.395 0.658 0.113 0.532

BE693119 -1.16 -0.795 0.469 0.5 0.61 0.464 -0.272 -0.563 -0.082 0.502 0.33 0.078

BE693122 1.142 0.854 1.336 1.318 0.73 0.97 0.412 0.819 0.545 0.824 0.347 -0.22

BE693123 0.246 -0.376 -0.212 -0.541 -0.829 0.232 0.067 -0.412 -0.151 0.642 0.647 0.791

BE693132 -0.792 -0.118 -0.14 -0.855 -0.627 -0.163 -0.362 -0.469 -0.434 0.19 0.884 0.174

BE693133 -0.053 -0.372 0.235 0.366 0.509 0.623 0.589 1.078 0.597 0.997 0.23 0.319

BE693134 -0.123 0.248 0.159 0.495 0.962 0.351 0.447 0.74 0.327 0.171 0.392 -0.009

BE693143 -0.197 -0.392 0.544 0.01 0.49 0.246 0.29 0.071 -0.237 0.127 0.492 0.277

BE693152 -0.338 0.173 0.705 0.821 0.851 1.33 0.865 1.176 0.501 0.69 0.416 0.357

BE693153 0.499 -0.348 0.179 0.296 0.453 0.958 0.557 1.242 0.772 1.309 0.608 0.031

BE693173 0.389 0.159 1.046 1.356 0.934 1.353 0.592 0.566 0.136 -0.153 -0.532 -0.696

BE693186 -2.7 -4.376 -4.107 -3.132 -4.54 -2.778 -3.48 -5.365 -3.512 0.504 1.16 2.194

BE693194 -0.486 0.044 -0.373 0.538 0.416 0.777 0.297 1.228 0.48 0.959 0.416 1.229

BE693204 -1.552 -2.278 -2.064 -1.637 -2.103 -1.823 -1.123 -2.236 -1.454 -0.46 0.754 1.422

BF156074 0.736 1.244 0.723 0.788 -0.43 -0.025 -0.289 -0.654 -0.653 -0.487 -1.046 -0.549

BF156211 -1.505 -1.153 2.498 1.714 1.457 0.687 1.094 0.794 1.45 0.947 0.843 0.094

BF156220 -0.23 0.1 0.252 0.703 0.807 -0.046 0.121 0.845 0.22 0.081 0.073 0.195

BF156260 0.618 1.642 1.423 1.133 0.058 0.173 -0.548 -0.188 -0.424 -0.424 -0.692 -0.688

BF157011 -0.728 -0.037 0.277 -0.24 0.05 -0.332 -0.219 0.193 -0.182 -0.749 -0.125 0.942

BF157279 -0.142 0.679 0.397 1.203 0.927 -0.017 0.039 0.474 -0.151 -0.223 0.243 -0.343

BF157344 -0.779 -0.292 -0.093 0.931 1.069 -0.161 0.16 0.912 0.065 0.588 0.479 0.293

BF157346 1.237 0.327 -0.802 -0.334 -0.543 0.206 0.148 -0.177 -0.159 -0.318 -0.502 -0.404

BF157490 -1.959 -0.387 0.678 0.703 0.25 0.005 -0.188 0.268 0.789 0.857 0.313 0.217

BF157620 0.231 0.475 1.886 1.999 2.439 0.894 0.118 1.468 0.726 0.478 -0.218 -0.266

BF157841 -0.09 -1.141 0.017 0.652 1.155 0.007 0.338 1.013 0.101 0.148 0.062 0.118

BF713861 0.139 0.824 1.033 1.192 0.748 -0.046 0.013 -0.355 0.024 -0.061 -0.231 -0.51

BF713867 -0.4 0.541 1.484 1.659 1.362 0.546 0.344 0.536 0.561 0.003 -0.298 -0.571

BF717296 -0.574 -2.459 -1.426 -1.099 -0.962 0.169 -0.069 0.034 0.165 0.868 0.488 -0.02

BF717383 -0.933 -1.16 -0.635 -0.832 -2.045 -0.734 -0.438 -1.136 -0.477 0.084 0.285 1.197

BF717503 -2.046 -1.241 -1.596 -0.88 -1.301 -0.747 -1.135 -0.731 -0.539 0.243 -0.099 -0.587

BF717510 0.094 0.139 -0.286 -0.342 -0.775 0.046 -0.441 0.081 0.309 0.45 0.341 1.199

BF717548 -2.75 -1.849 -2.551 -0.542 -0.94 -0.673 -1.024 -1.216 -0.918 0.662 0.408 0.872

BF717971 0.984 0.323 0.021 0.159 -0.782 -0.452 -1.076 -1.578 -0.769 -1.273 -1.054 -0.526

BF718003 0.266 1.065 1.568 0.967 -0.125 -0.266 -0.172 -0.391 -0.601 -0.756 -0.455 -0.537

BF718175 -0.286 -0.042 0.059 0.483 -0.026 0.257 0.192 0.661 0.306 0.286 0.363 0.357

BF718182 -0.725 -0.545 -0.939 0.616 0.749 0.866 0.312 0.676 0.215 0.777 0.423 0.815

BF718229 -1.24 -2.836 -1.569 -1.154 -0.781 0.263 0.076 0.29 0.402 1.01 0.687 0.174

BF937404 0.212 0.156 0.651 0.435 0.607 0.324 0.4 0.773 0.24 0.602 -0.049 0.081

BF938407 0.2 -0.041 -0.115 0.341 -0.108 0.442 0.432 -0.245 0.295 0.049 0.16 1.651

BF938808 -0.731 -0.028 1.161 1.773 1.582 1.521 0.905 1.267 0.223 0.566 0.226 -0.574

BF938837 -0.15 0.631 0.229 0.472 0.262 0.044 0.308 0.665 -0.017 0.075 0.017 0.087

BG302504 2.067 -4.968 -4.639 -4.352 -5.23 -4.092 -4.583 -5.691 -5.38 -5.537 -5.57 -6.063

BG302583 0.4 0.208 0.14 0.019 -0.576 -0.224 -0.613 -0.456 -0.083 0.17 0.4 0.658

BG302584 0.225 0.565 0.689 0.461 -0.393 -0.469 -0.628 -0.448 -0.483 -0.078 -0.239 -0.258

BG302610 -0.093 1.203 0.808 0.796 -0.078 -0.254 -0.065 0.103 -0.223 -0.315 -0.464 -0.6

BG302674 1.074 1.094 1.272 0.939 0.838 1.098 0.326 0.411 -0.021 0.023 -0.195 -0.316

BG302711 0.091 -0.646 -1.555 -0.311 -1.013 0.072 -0.163 0.217 0.36 1.07 0.67 0.596

BG302802 1.067 -0.086 -0.944 -0.162 -0.196 0.407 -0.197 -0.039 -0.137 -0.487 -0.29 -0.417

BG302807 0.549 0.227 0.123 0.95 0.915 0.701 0.488 1.128 0.562 0.239 0.2 -0.414

BG302813 0.888 1.348 1.144 0.562 0.081 0.42 0.123 0.321 -0.106 -0.078 -0.317 -0.706

BG302868 0.489 -0.089 0.111 0.492 0.677 0.755 0.438 1.127 0.455 0.336 0.142 -0.371

BG302910 0.326 1.726 1.644 1.624 1.256 0.615 0.215 0.702 -0.241 -0.687 -0.628 -0.703

BG302931 1.113 0.993 -0.198 -0.157 -0.079 -0.205 0.009 -0.169 -0.312 -0.204 0.055 -0.238

BG302933 -0.13 0.263 0.743 0.865 0.62 -0.284 0.01 0.291 0.278 0.192 0.105 0.3

BG302934 -0.955 -1.085 -1.254 -0.422 -1.665 -0.474 -0.545 -1.982 -0.751 -0.079 0.684 0.728

BG302974 0.167 0.846 1.555 1.451 1.208 -0.137 0.014 0.313 -0.095 0.143 -0.057 -0.495

BG302989 0.915 0.752 -0.909 -0.304 -0.821 -0.317 -0.345 -0.734 -0.543 -0.958 -0.298 -0.586

BG303079 1.451 0.132 -0.304 -0.283 -0.2 -0.344 0.169 -0.447 -0.291 -0.379 -0.432 -0.422

BG303177 1.036 1.072 0.352 -0.219 -0.262 0.158 -0.503 -0.678 -0.674 -0.716 -0.973 -0.807

BG303217 -0.767 -0.118 -0.081 -0.202 -0.221 0.123 -0.049 -0.236 -0.015 0.081 0.727 -0.099

BG303237 -2.013 -2.195 -2.393 -1.643 -1.457 -1.21 -1.649 -1.447 -1.254 0.818 0.75 0.479

BG303239 0.086 0.414 1.064 1.213 1.279 0.449 0.126 0.698 -0.045 -0.039 -0.035 -0.279

BG303243 -0.457 0.57 1.462 1.313 0.822 0.859 0.719 0.475 0.284 0.089 -0.004 -0.023

BG303246 1.177 0.711 -0.114 -1.185 -0.762 -0.678 -0.912 -1.133 -0.828 -1.039 -0.635 -0.639

BG303289 -0.632 0.013 0.411 0.523 0.544 0.1 0.496 1.044 0.188 0.146 0.331 0.149

BG303377 1.89 1.564 -0.514 -0.296 -0.231 0.634 0.366 0.884 0.271 0.237 -0.429 -0.675

BG303391 0.065 1.113 1.439 1.436 1.456 -0.087 0.349 0.716 -0.202 -0.574 -0.476 -0.442

BG303445 0.419 1.221 1.287 1.125 0.572 0.673 0.484 0.957 0.131 0.013 -0.4 -0.467

BG303457 1.513 1.158 0.949 0.057 -0.933 -0.145 0.04 -1.041 -0.421 -0.812 -1.004 -0.23

BG303472 1.056 0.571 -1.243 -1.14 -0.925 -0.167 -0.391 -0.56 -0.707 -0.738 -0.773 -0.302

BG303491 0.157 1.119 1.183 0.674 0.061 0.035 -0.037 -0.072 -0.156 -0.449 -0.618 -0.417

BG303497 0.129 -0.702 -0.418 0.001 0.112 -0.077 0.426 0.797 0.452 0.905 0.359 -0.293

BG303518 0.513 0.98 0.97 0.984 0.067 -0.409 -1.253 -0.966 -0.535 -0.281 -0.755 -0.224

BG303560 0.085 -2.986 -0.314 -0.492 0.083 0.825 0.377 0.9 0.29 0.912 0.545 0.263

BG303575 0.919 -0.894 -0.521 -0.212 0.017 1.337 0.968 0.801 0.187 0.225 -0.309 -0.509

BG303594 -0.103 0.89 0.926 1.015 0.401 0.814 0.463 0.441 -0.046 -0.418 -0.094 -0.303

BG303602 0.27 -0.154 0.072 -0.094 -0.309 0.718 0.213 0.65 0.471 1.063 -0.012 -0.461

BG303611 -1.053 0.619 0.949 -2.695 -3.259 1.368 1.218 -0.13 1.09 0.652 -1.626 -1.486

BG303613 0.671 0.767 0.7 0.937 0.708 0.574 0.426 1.408 0 0.325 -0.119 -0.285

BG303641 1.079 0.945 -0.62 -0.05 -0.278 -0.074 -0.5 -0.488 -0.455 -1.022 -0.49 -0.252

BG303647 0.794 1.098 1.247 1.363 0.91 1.352 0.62 0.589 0.348 0.344 -0.077 -0.175

BG303695 1.277 0.948 -0.099 0.144 -0.031 0.236 -0.422 -0.547 -0.902 -0.686 -0.488 -0.301

BG303721 0.628 0.26 0.85 0.344 0.959 1.681 0.808 0.823 0.573 0.586 0.183 -0.196

BG303725 0.835 0.957 -0.289 -0.107 -0.642 0.069 -0.486 -0.267 -0.244 -0.426 -0.252 -0.247

BG303768 0.868 0.85 0.981 1.233 0.713 0.203 0.541 0.267 -0.02 -0.057 -0.838 -0.632

BG303781 1.776 1.794 0.651 0.088 -1.185 -0.466 -0.841 -1.053 -0.629 -0.975 -1.447 -1.003

BG303824 1.079 0.185 0.236 0.091 -0.199 0.037 -0.079 -0.154 -0.226 -0.284 -0.07 -0.239

BG303835 1.474 1.151 1.873 1.368 1.147 0.512 0.089 0.311 -0.337 -0.704 -0.797 -0.567

BG303872 0.514 0.455 0.622 1.08 1.536 0.19 0.185 0.528 0.206 0.019 -0.432 -0.167

BG303890 1.146 1.225 -0.304 -0.368 -1.054 -0.749 -0.556 -1.412 -1.004 -0.688 -1.091 -0.974

BG303935 1.903 0.346 -0.101 0.571 0.239 0.228 -0.429 -1.102 -1.105 -2.289 -2.04 -2.077

BG303979 0.789 -1.53 -2.205 -2.106 -2.349 -1.738 -1.208 -2.789 -1.585 -2.387 -1.834 -2.223

BG303999 0.185 0.386 1.173 1.145 0.879 0.708 0.519 0.838 0.81 0.809 0.41 0.726

BG304082 0.927 1.462 0.771 0.257 -0.462 -0.145 -0.518 0.158 0.023 -0.219 0.102 -0.572

BG304114 0.293 0.605 1.356 0.916 0.944 1.453 0.592 0.857 0.711 0.695 0.1 0.293

BG304135 0.873 1.727 1.304 0.634 -0.977 -0.543 -0.462 -1.498 -1.05 -0.867 -1.482 -1.358

BG304149 0.807 1.426 1.581 1.624 1.508 1.442 0.85 1.103 0.028 0.215 -0.289 -0.133

BG304158 -0.024 0.384 1.589 1.826 1.952 1.364 1.475 1.67 0.969 0.929 0.227 -0.325

BG304162 0.108 1.173 1.14 1.117 -0.213 -0.043 -0.307 -0.834 -0.521 -0.628 -0.659 -0.366

BG304168 -0.146 -0.082 0.527 0.36 0.496 0.909 0.403 0.829 0.291 0.401 0.088 0.051

BG304211 0.93 0.215 -0.243 0.082 -0.29 0.201 0.083 -0.134 0.014 -0.429 -0.315 -0.569

BG304219 -0.361 -0.025 -0.153 -0.098 -0.032 0.003 0.239 0.952 0.087 -0.122 0.053 -0.176

BG304232 1.547 -0.823 -0.57 -0.698 -2.327 -1.269 -1.655 -2.199 -1.904 -1.568 -2.441 -1.71

BG304233 1.136 0.49 -0.442 -0.158 -0.86 -0.349 -0.07 -0.012 -0.237 -0.369 -0.719 -0.367

BG304234 0.568 0.008 0.163 0.06 0.227 0.673 0.158 1.032 0.392 0.569 0.254 0.199

BG304255 -0.066 0.101 0.286 0.641 1.051 0.373 0.082 1.34 0.684 0.338 0.093 -0.46

BG304274 1.224 0.883 0.255 -0.153 -0.538 -0.022 -0.494 -0.336 -0.462 -0.269 -0.651 -0.594

BG304285 0.438 0.763 0.892 0.795 0.981 1.086 0.514 0.64 0.223 0.083 -0.139 -0.085

BG304294 0.996 0.419 -0.036 0.021 -0.182 0.179 0.105 0.646 0.387 0.76 0.472 0.415

BG304333 -0.224 -1.104 -0.552 -0.631 -0.127 0.743 0.279 0.902 0.659 0.979 0.675 0.276

BG305033 -0.458 0.132 -0.08 0.328 -0.083 0.015 -0.397 -0.14 0.039 0.284 0.601 0.997

BG305295 0.508 0.013 -0.603 -0.323 -0.219 -0.084 -0.264 -0.278 -0.321 0.045 0.124 0.721

BG305296 0 -0.569 -0.178 0.062 0.31 1.359 0.498 1.102 0.566 0.596 0.488 0.068

BG305301 -1.243 -1.257 -0.695 -0.462 0.228 0.177 -0.279 -0.201 0.526 0.008 0.61 0.39

BG305364 0.688 -0.196 -0.593 -0.88 -1.094 -0.133 -0.065 0.291 0.258 0.354 0.498 -0.144

BG305366 0.322 0.513 0.245 0.638 0.53 0.491 0.506 0.911 0.162 0.815 0.398 0.204

BG305367 0.283 0.13 0.038 0.648 1.153 0.534 0.63 1.037 0.078 0.1 0.002 0.404

BG305441 0.915 0.361 -0.677 -0.291 0.119 -0.009 -0.982 -0.365 -1.198 -0.445 -0.344 -0.178

BG305445 -0.541 -0.382 -0.352 0.079 0.808 1.121 0.606 1.303 0.84 0.728 0.477 0.562

BG305492 1.541 1.562 1.072 0.726 0.65 0.61 0.34 0.248 -0.272 0.141 -0.226 0.124

BG305533 -0.885 0.091 -0.468 -0.192 -0.645 -0.199 -0.48 -0.708 -0.689 -0.606 -0.02 1.254

BG305537 1.302 0.918 -0.099 0.136 -0.179 -0.028 -0.019 -0.06 -0.241 -0.675 -0.678 -0.408

BG305568 -0.649 -1.432 -0.958 -1.096 -1.292 -0.365 -0.196 -1.338 -0.662 -0.262 -0.27 0.898

BG305572 -0.611 -0.194 1.141 1.774 1.391 0.352 0.622 1.448 0.955 0.751 0.348 -0.521

BG305622 0.981 0.262 -0.213 -0.011 -0.772 -0.167 -0.411 -0.337 -0.342 -0.018 -0.214 -0.437

BG305654 0.902 1.754 1.566 0.455 0.298 0.496 0.073 0.035 -0.164 -0.308 -0.291 -0.378

BG305705 0.949 0.204 0.495 0.174 0.267 0.554 0.636 -0.184 0.429 0.271 0.512 0.523

BG305790 -0.653 0.033 1.14 1.154 1.736 1.791 1.188 1.785 0.532 0.399 0.296 0.083

BG305800 1.202 0.155 0.404 0.038 -0.26 0.047 -0.249 0.072 -0.113 0.259 -0.023 -1.261

BG305857 -0.118 -0.062 -0.031 0.128 0.113 0.368 -0.059 0.083 0.378 0.128 0.384 0.65

BG305892 1.007 1.177 1.098 1.248 1.54 1.95 1.4 0.77 0.532 -0.09 0.422 -0.189

BG305988 -0.94 -3.359 -1.396 -1.333 -0.816 0.51 0.114 0.176 0.032 0.85 0.589 0.252

BG305992 -0.157 0.144 0.072 0.335 0.219 -0.068 0.211 0.688 0.035 0.378 0.111 0.128

BG306038 0.708 -0.509 -0.081 0.377 0.645 1.127 0.853 1.424 0.731 0.974 0.33 0.143

BG306096 1.229 -0.06 -0.559 0.29 -0.562 -0.162 -0.231 -0.546 -0.276 -0.682 -0.476 -0.614

BG306111 -0.962 -2.195 -1.69 -1.341 -0.632 -0.114 0.142 0.428 -0.117 0.846 0.483 0.263

BG306138 -0.777 -0.608 -0.966 -0.466 0.111 0.454 0.42 0.834 0.532 0.871 0.952 1.011

BG306139 -0.728 -0.321 -0.53 0.092 0.905 0.195 0.82 1.87 0.962 0.423 0.449 0.021

BG306148 0.321 1.378 0.969 1.107 1.166 0.904 0.39 1.26 0.489 0.198 -0.374 -0.692

BG306150 -1.028 -1.311 -0.669 -0.556 -0.985 -0.761 -0.453 -1.686 -1.026 -0.434 0.147 1.506

BG306178 0.08 0.793 0.318 0.59 0.873 0.06 0.486 1 0.686 -0.001 -0.122 0.064

BG306295 0.177 0.583 1.178 1.241 1.784 1.326 1.411 1.961 0.76 0.748 0.282 0.231

BG306308 1.104 -1.842 -1.875 -1.296 -1.045 0.128 -0.423 -0.104 -0.41 0.41 0.759 0.158

BG306318 -0.519 -0.806 0.173 -0.688 0.186 0.344 0.725 1.374 0.494 0.71 0.621 -0.02

BG306390 1.019 0.343 1.777 1.25 1.505 2.005 1.641 1.624 0.915 0.879 0.434 0.201

BG306405 0.144 0.368 1.268 1.287 1.34 2.351 1.345 1.481 0.771 0.889 0.638 0.563

BG306420 -0.449 -0.369 -0.812 -0.335 -0.064 0.092 0.17 0.465 0.047 0.54 0.34 0.164

BG306459 -0.931 -2.668 -0.832 -0.844 -0.338 0.485 0.303 0.473 0.265 0.598 0.283 -0.037

BG306468 -0.159 -0.385 0.407 0.904 1.022 0.464 0.596 0.978 0.163 -0.26 0.111 0.195

BG306779 -0.147 -0.472 0.314 0.221 0.985 1.349 1.342 1.555 0.949 0.759 0.727 0.716

BG307024 0.822 1.055 1.072 1.249 0.53 0.555 -0.024 0.024 0.024 -0.044 -0.158 -0.304

BG307330 -0.184 0.053 0.096 -0.829 0.086 0.314 0.233 -0.219 -0.061 -0.314 0.655 0.207

BG307383 1.305 -0.699 -0.659 -0.465 -0.572 -0.136 -0.137 -0.139 -0.352 0.212 -0.155 0.063

BG307533 0.852 1.217 1.086 -0.058 -0.572 -0.038 -0.147 -0.791 -0.603 -0.61 -0.964 -0.539

BG307539 1.425 0.326 1.024 1.475 1.177 0.592 0.892 0.811 0.131 0.112 -0.297 -0.428

BG307572 -0.468 -0.085 0.294 0.809 1.666 -0.494 0.437 1.08 0.424 0.043 -0.081 -0.267

BG308220 -0.434 0.425 0.687 1.041 0.981 -0.019 0.489 0.61 0.129 -0.038 -0.235 -0.276

BG308318 -0.728 0.366 0.439 0.524 0.67 -0.058 -0.331 0.229 -0.306 -0.589 -0.18 -0.265

BG308501 -0.356 -0.736 -0.215 -0.078 -0.298 0.516 0.567 0.398 0.5 0.369 0.434 0.875

BG308524 -0.524 -0.841 -0.08 -0.221 -1.358 -0.907 -0.189 -0.763 -0.325 -0.058 0.348 1.366

BG308557 -0.345 -0.143 0.306 0.335 0.328 0.222 0.184 0.227 0.24 -0.013 0.409 0.757

BG308628 -1.186 -1.349 -1.625 -0.659 -1.519 -0.454 -0.863 -1.605 -0.731 0.386 -0.061 -0.218

BG308652 1.027 0.622 -1.17 -0.822 -1.329 -0.175 -0.58 -0.298 -0.345 -0.169 -0.672 -0.377

BG308713 1.515 0.399 -0.526 -0.15 0.333 0.182 0.09 0.365 0.129 -0.217 -0.261 -0.193

BG308784 0.444 0.503 0.804 0.768 0.886 0.339 0.189 1.159 0.033 0.518 0.085 0.168

BG727181 0.935 0.325 0.173 -0.461 -0.714 0.096 -0.273 -0.717 -0.254 -0.71 -0.318 -0.46

BG727207 0.322 0.214 0.745 1.111 1.024 1.162 0.746 1.093 0.447 0.529 0 -0.036

BG727249 0.443 0.606 2.247 2.163 1.354 0.72 0.979 0.502 0.3 -0.084 -0.675 -0.313

BG727402 0.952 1.341 0.504 0.152 -0.465 -0.216 -0.745 -1.006 -0.743 -0.81 -0.942 -0.882

BG727413 1.582 0.28 -0.401 0.019 0.218 0.581 0.159 0.63 -0.264 -0.426 -0.207 -0.394

BG727431 -0.814 0.123 0.488 0.704 1.38 1.51 0.744 0.93 0.526 0.27 0.277 0.253

BG727480 0.071 1.015 0.825 0.171 0.176 -0.222 -0.098 -0.189 0.021 -0.201 0.043 -0.245

BG727484 -1.565 -0.908 -0.623 -0.482 0.297 -0.261 -0.487 -0.618 0.426 -0.158 0.582 0.571

BG727557 -0.434 0.252 1.633 1.795 2.593 2.382 1.807 2.117 1.253 1.097 0.471 0.078

BG727595 1.3 0.468 0.71 0.626 0.562 1.117 0.672 0.293 0.492 0.469 0.32 -0.093

BG727615 -2.472 -2.218 -2.066 -1.095 -1.503 -0.435 0.193 0.359 0.435 1.371 0.848 0.289

BG727890 -0.012 -0.366 0.39 0.278 0.043 0.527 0.378 0.565 0.079 0.365 -0.012 -0.1

BG728382 1.042 0.349 -1.281 -0.641 -0.764 -0.402 -0.407 -0.199 -0.688 -0.735 -0.653 -0.981

BG728404 0.409 0.156 0.225 0.591 0.877 0.25 0.508 0.736 -0.124 0.163 0.137 0.06

BG728452 -0.464 -2.34 -1.934 -1.535 -0.892 0.171 -0.212 0.31 0.022 0.797 0.316 -0.067

BG728552 -1.205 -0.451 -1.385 -0.15 -0.187 -0.316 -0.047 0.299 1.104 1.242 0.601 0.369

BG728568 -0.065 0.141 0.596 0.895 1.13 0.366 1.155 1.498 0.35 -0.132 -0.068 -0.095

BG728569 -0.114 0.877 0.963 1.032 -0.237 -0.493 -1.027 -1.652 -0.96 -0.011 -0.03 -0.144

BG728626 -0.497 0.217 0.21 -0.233 -0.117 0.035 0.289 0.951 -0.057 -0.26 -0.216 -0.117

BG728726 -0.137 0.742 0.724 0.481 0.394 0.199 -0.062 -0.087 0.174 -0.003 -0.238 -0.348

BG728817 0.377 -0.05 0.755 1.343 3.093 1.549 1.395 1.308 0.925 -0.066 -0.224 -0.427

BG728956 -0.929 -0.46 -1.681 -0.726 -1.024 -0.008 0.322 1.004 0.57 0.774 -0.277 -0.505

BG729054 1.156 -1.105 -0.747 -0.933 -1.147 -0.78 -1.798 -1.343 -0.962 -1.052 -1.207 -1.229

BG729144 0.893 0.835 -0.359 -0.318 -0.401 -0.049 0.085 -0.449 -0.386 -0.66 -0.498 -0.718

BG729177 1.127 1.144 0.153 -0.177 -0.166 0.302 -0.021 0.608 0.441 -0.047 -0.356 -0.285

BG729232 0.163 1.026 0.501 0.653 0.231 -0.039 0.042 0.846 -0.173 0.04 -0.149 -0.14

BG729245 -0.412 -0.172 -0.486 -0.946 -0.063 0.099 0.305 0.898 0.999 0.289 0.1 0.362

BG729372 0.87 0.593 -0.231 -0.147 0.212 0.188 -0.297 0.616 0.128 -0.007 0.311 0.038

BG737927 -0.728 0.545 0.666 0.969 1.115 -0.29 -0.053 0.618 0.146 -0.717 0.122 -0.25

BG737957 -0.869 0.267 -0.12 -0.52 0.285 0.691 0.642 1.6 0.856 0.988 0.801 1.199

BG738252 -1.155 -0.118 0.654 0.679 0.991 1.174 0.57 1.256 0.719 0.749 0.604 0.506

BG738534 -0.876 -1.376 -1.552 -1.342 -0.396 -0.284 -0.322 -0.664 -0.949 -0.244 -0.024 1.461

BG738899 0.523 1.205 0.837 0.627 -0.152 -0.265 -0.114 0.076 -0.193 -0.265 -0.228 -0.372

BG799157 1.249 -0.824 -0.565 -0.401 0.04 0.294 0.02 0.182 -0.228 -0.238 -0.171 -0.138

BG799259 0.268 0.853 1.432 1.605 1.458 1.78 0.648 1.409 0.451 0.701 0.3 -0.144

BG799326 -1.094 -0.934 -0.305 0.084 -0.084 0.686 0.655 0.419 0.634 0.918 0.918 0.623

BG799357 -0.163 0.138 0.89 1.221 1.239 -0.105 0.059 0.31 -0.052 -0.222 -0.117 -0.171

BG799399 -0.777 -0.694 1.608 3 4.15 4.333 3.419 3.418 2.052 0.008 0.025 -0.241

BG799577 -0.631 0.045 0.034 0.119 0.914 0.084 0.059 1.057 0.763 0.388 0.565 0.135

BG799622 0.351 1.413 0.998 1.025 0.549 -0.043 -0.037 -0.067 -0.184 -0.297 -0.565 -0.654

BG883207 0.706 0.484 2.427 1.969 2.813 1.712 1.247 1.881 0.663 0.963 0.383 0.135

BG883210 0.995 1.26 0.206 0.631 -0.133 0.405 0.199 0.51 -0.079 0.228 -0.046 -0.289

BG883236 -0.138 -0.138 0.509 0.198 1.61 0.805 0.639 1.027 0.563 0.32 0.273 0.516

BG883304 -0.547 0.453 1.935 1.845 1.97 0.81 0.118 0.299 -0.127 -0.022 -0.641 -0.355

BG883325 -1.649 -0.661 -1.583 -1.699 -1.948 -0.215 -1.175 -1.512 -1.097 -0.482 0.275 1.626

BG883345 -0.691 0.545 -0.168 0.341 0.441 0.398 0.25 -0.057 0.046 0.005 0.794 0.512

BG883671 0.005 0.215 0.765 0.679 1.193 1.241 0.565 0.953 0.681 0.489 0.242 0.349

BG883685 -0.128 0.513 0.885 1.296 1.307 0.222 0.424 1.052 0.23 0.001 0.096 -0.074

BG883692 0.988 0.356 0.005 -0.154 0.32 0.694 -0.01 0.665 0.306 0.359 0.132 0.246

BG883703 2.524 -1.172 -1.223 -0.883 -0.962 -0.401 -0.515 -0.986 -0.994 -0.703 -1.134 -0.364

BG883878 0.555 1.441 1.97 1.813 1.187 0.53 0.081 0.576 0.296 -0.326 -0.326 -0.101

BG884096 -0.008 -0.169 0.677 0.468 0.454 0.852 0.77 0.894 0.532 0.602 0.348 0.094

BG884107 -1.586 -0.292 -0.458 -0.057 0.501 1.295 0.719 0.735 0.428 1.33 0.827 1.131

BG884388 -0.723 -0.117 2.244 1.73 1.501 0.554 0.479 0.513 0.402 0.381 0.476 0.558

BG884401 -0.278 -0.612 -0.505 -0.627 -0.266 -0.268 -0.125 -0.734 -0.212 -0.022 0.176 0.979

BG884507 1.699 -0.863 -1.373 -1.433 -1.086 -0.062 -0.953 -0.568 -1.132 -1.665 -0.413 -1.148

BG891864 -0.132 -1.478 -0.474 -0.068 0.363 0.3 0.399 0.859 0.068 0.103 -0.12 0.195

BG891888 0.237 0.63 0.372 0.591 -0.076 0.396 0.163 0.737 0.082 0.35 0.254 -0.278

BG891893 -0.21 0.27 -0.006 0.373 0.649 0.117 0.462 0.588 0.186 0.069 0.149 0.247

BG891955 0.241 -0.182 0.119 0.842 1.412 1.218 0.752 1.013 0.366 0.218 0.276 0.388

BG892019 0.159 0.083 0.669 0.665 0.672 0.278 0.495 0.822 0.211 0.453 -0.01 0.037

BG892077 1.609 0.095 0.409 0.701 0.962 0.71 0.869 0.444 0.095 0.176 -0.252 -0.527

BG985441 0.1 -1.247 -0.382 -0.574 -0.825 0.298 0.332 0.345 0.725 0.82 0.969 0.606

BG985449 0.908 -0.539 -0.955 -0.986 -1.351 0.252 0.322 0.346 1.054 0.678 0.291 0.228

BG985455 -2.468 -2.178 -2.284 -1.861 -1.904 0.224 0.138 -0.069 0.355 0.721 1.045 1.76

BG985460 -1.109 -1.649 1.801 2.578 3.241 2.613 1.623 1.109 1.668 0.504 0.229 -0.073

BG985468 -1.635 -1.49 -0.657 -0.15 0.419 1.35 0.973 1.159 0.906 0.68 0.195 -0.218

BG985470 0.483 0.016 0.212 0.576 1.37 1.464 0.449 1.033 0.217 0.473 0.062 0.408

BG985472 -0.181 0.283 2.434 1.823 1.871 1.142 0.834 0.845 1.207 0.27 0.454 0.165

BG985475 -0.031 1.133 1.756 1.674 1.365 0.812 0.632 0.845 0.1 0.063 -0.092 0.515

BG985485 -0.443 -0.841 -1.389 -0.255 -0.177 -0.452 -0.386 -0.073 -0.252 0.287 -0.165 -0.094

BG985493 0.379 -0.001 0.841 0.602 0.78 1.435 1.206 0.955 0.589 0.43 0.071 -0.034

BG985498 -1.013 -1.923 2.883 2.542 3.052 1.85 1.369 2.023 0.953 0.744 0.504 0.282

BG985501 -0.387 0.668 1.113 1.281 1.266 1.107 0.647 1.369 0.022 0.801 0.656 0.337

BG985503 -0.612 -0.164 1.419 1.396 1.722 1.968 1.606 1.962 1.2 1.045 0.958 -0.26

BG985504 -0.057 0.376 0.675 0.424 0.681 0.794 0.101 0.587 0.292 0.338 0.325 0.074

BG985507 -1.117 -1.324 -1.357 -0.779 -2.544 -0.533 -0.769 -1.615 -0.253 0.663 0.774 1.403

BG985512 0.745 1.387 1.522 0.518 0.423 0.711 0.376 0.762 0.14 0.15 -0.095 -0.274

BG985516 -0.567 0.222 0.467 0.401 0.104 -0.334 0.148 0.04 0.321 0.154 0.464 0.482

BG985522 -0.791 -0.142 1.535 1.639 1.122 0.92 0.337 0.759 0.492 0.211 0.549 0.071

BG985575 -0.436 -0.403 0.252 0.179 0.04 0.305 -0.019 0.687 0.395 0.525 0.853 0.462

BG985598 0.142 -0.126 0.182 0.685 1.191 0.686 0.513 1.026 0.272 0.732 0.469 0.286

BG985620 -0.42 -0.472 -0.314 0.207 1.201 0.569 0.465 1.132 0.333 0.189 0.155 0.207

BG985671 0.174 0.674 1.022 0.646 0.603 1.245 0.619 1.324 0.561 0.888 0.371 -0.011

BG985673 -1.717 -3.699 -2.59 -1.413 -2.097 -1.158 -0.793 -1.653 -0.601 0.891 0.598 0.537

BG985680 -0.446 -0.434 1.023 1.189 0.533 0.861 0.561 0.786 0.649 1.24 1.329 0.321

BG985688 1.008 1.091 0.733 0.953 1.034 1.173 0.695 0.847 0.215 0.169 -0.382 -0.827

BG985698 -0.739 -0.431 0.482 0.859 1.807 1.733 0.706 1.382 0.308 0.398 0.828 0.355

BG985703 0.921 -0.067 0.225 0.309 -0.374 0.09 0.085 -0.07 -0.124 0.401 -0.231 -0.695

BG985722 -1.335 -0.4 -1.024 -1.283 -1.091 -0.341 -0.809 -0.505 -0.018 1.117 1.059 1.44

BG985738 0.078 -0.628 0.151 -0.304 0.21 0.055 0.237 1.621 0.634 0.365 0.529 0.32

BG985742 -0.728 0.42 0.27 -0.369 -0.645 -0.317 -0.478 -0.054 -0.406 0.157 0.724 0.08

BG985763 -0.728 -0.149 0.465 0.903 1.606 -0.125 0.132 0.929 -0.378 -0.766 -0.543 -0.913

BG985777 0.309 0.374 0.744 0.386 0.72 0.534 0.437 1.592 0.551 0.72 -0.118 -0.579

BG985787 0.125 1.156 1.189 1.268 1.38 0.354 0.424 0.699 0.187 -0.337 -0.627 -1.127

BG985833 0.033 1.04 0.049 0.022 0.531 0.566 0.271 0.889 0.179 0.197 -0.003 -0.115

BG985846 -0.622 -0.493 1.243 1.426 1.57 2.372 2.141 1.812 1.375 0.889 0.503 0.223

BI318080 -0.443 -0.284 0.142 1.68 1.805 0.36 0.372 0.227 -0.029 -0.271 -0.444 -0.239

BI318094 -0.654 -1.773 -1.007 -0.343 -0.466 0.407 0.197 0.292 0.131 0.953 0.497 0.204

BI318628 0.088 0.256 0.334 0.489 0.756 0.102 0.454 1.29 0.105 0.162 0.433 0.088

BI325077 -1.956 -1.575 -2.353 -1.153 -1.71 -1.106 -1.498 -1.321 -0.062 0.313 0.604 1.321

BI325085 -1.339 -1.656 -0.812 -0.741 -1.008 -0.257 -0.282 -0.012 0.552 1.131 1.106 0.503

BI325086 -0.137 0.481 0.156 0.675 0.786 0.25 0.436 0.084 0.413 0.386 -0.283 -0.374

BI325513 -0.091 1.452 0.759 0.826 0.153 -0.178 0.093 -0.04 -0.095 -0.04 -0.328 -0.219

BI325685 1.853 1.552 0.338 0.256 0.034 0.266 -0.342 -0.018 -0.405 -0.571 -0.787 -0.621

BI325825 -0.91 -2.913 -1.058 -0.825 -0.396 0.65 0.253 0.413 0.465 1.3 0.914 0.265

BI325924 0.53 1.193 1.161 1.4 0.687 0.088 -0.207 -0.025 -0.154 -0.924 -0.464 -0.766

BI326643 -0.901 -1.378 -1.043 -0.745 -0.008 -0.345 -0.328 -0.76 0.061 -0.278 0.357 0.379

BI326788 -0.119 0.556 0.843 0.787 0.688 0.244 0.284 0.587 0.376 -0.201 -0.495 -1.002

BI350696 -0.239 0.062 0.04 0.252 -0.668 -0.392 0.024 -0.057 0.112 -0.613 0.078 1.37

BI350885 0.28 1.382 1.62 1.457 1.615 0.407 0.726 1.427 0.193 -0.434 0.035 -0.303

BI427744 0.083 -0.766 -0.593 -0.754 -0.166 0.09 0.7 1.512 0.578 1.069 1.138 0.023

BI427758 0.092 -0.435 1.28 1.538 1.374 1.614 1.376 1.878 0.726 0.639 -0.108 0.028

BI427792 -0.625 -3.292 -1.079 -1.225 -1.044 0.025 -0.026 0.018 -0.017 0.713 0.472 -0.033

BI428149 -0.685 -0.785 -0.169 -0.271 -0.255 -0.187 -0.02 -0.893 -0.486 -0.058 0.475 0.393

BI428520 1.043 0.002 -0.326 -0.258 -0.922 -0.175 -0.45 -0.766 -0.505 0.249 -0.034 -0.335

BI428543 0.045 -0.195 0.882 1.007 0.864 0.855 0.739 0.559 0.127 0.461 0.017 0.457

BI428973 -0.406 -0.284 1.891 2.453 2.338 2.51 1.666 1.07 0.827 0.821 0.44 0.616

BI428991 -0.483 0.232 1.546 1.819 1.482 0.424 0.317 0.747 -0.049 0.313 0.469 0.58

BI429006 -0.542 0.214 0.32 -0.367 -0.119 -0.042 -0.424 -0.131 -0.468 -0.708 -0.039 0.745

BI429020 -0.22 -0.742 -0.084 0.032 0.357 0.415 0.154 1.037 0.637 0.835 0.106 -0.164

BI429605 -0.416 -0.616 0.092 0.241 0.459 0.947 0.506 0.703 0.344 0.109 0.327 0.006

BI429638 -0.186 -0.525 0.122 -0.168 0.107 -0.289 -0.305 0.611 0.509 0.259 0.619 0.119

BI429706 1.237 -0.997 -0.921 -0.722 -0.802 -0.358 -0.193 -0.61 -0.556 -0.546 -0.517 -0.168

BI430050 -0.865 -0.895 -0.432 -0.234 -1.125 -0.483 -0.184 -1.048 -0.102 0.079 0.253 0.535

BI430095 0.891 0.511 1.464 0.963 0.994 0.552 0.045 0.457 -0.202 -0.246 -0.464 -0.503

BI430207 1.052 1.775 1.071 -0.03 0.13 0.495 0.192 0.65 0.095 -0.424 -0.473 -0.833

BI430221 0.115 0.723 0.828 0.789 0.476 -0.02 -0.164 -0.255 -0.039 -0.456 -0.711 -0.872

BI430251 -0.274 -0.238 -0.341 -0.236 0.341 0.221 0.393 1.176 0.249 0.38 0.78 0.069

BI430332 0.57 1.856 1.886 1.5 1.138 0.01 -0.471 -0.5 -0.364 -0.948 -0.607 -0.001

BI430334 0.361 -0.492 -0.661 -0.096 -0.719 -0.109 -0.02 -0.694 -0.443 -0.131 0.05 0.753

BI430340 -0.091 -0.961 -0.802 -0.365 -0.002 0.605 0.326 0.69 0.242 0.893 0.566 0.626

BI472540 0.047 -0.037 0.244 0.57 0.483 0.067 0.624 1.821 0.393 0.061 0.056 -0.179

BI472743 -0.839 -3.492 -1.865 -1.411 -1.021 0.326 0.016 0.27 0.204 0.885 0.611 -0.089

BI473004 1.165 -0.66 -0.799 -0.479 -0.048 0.898 0.675 1.362 0.411 0.856 0.725 -0.004

BI473383 -0.328 -0.371 0.195 0.444 0.471 0.353 0.681 0.189 0.213 0.266 -0.29 -0.145

BI473591 -0.17 -1.132 -1.181 -1.591 -0.562 -0.276 -0.413 0.286 0.584 0.666 0.364 -0.243

BI473712 0.827 0.918 0.382 0.501 -0.195 0.355 0.233 0.156 -0.205 0.021 -0.282 -0.338

BI474299 -0.368 -0.823 1.011 1.127 1.853 2.506 2.282 2.019 1.33 0.804 0.309 -0.186

BI474897 0.498 0.828 0.965 0.952 0.15 0.344 0.368 0.845 0.094 0.356 -0.101 0.021

BI474928 -0.704 -0.286 0.407 0.571 0.562 -0.065 0.371 0.294 -0.225 -0.502 0.161 -0.139

BI474952 1.413 1.022 -0.627 -0.905 -1.733 -0.858 -1.057 -1.117 -1.67 -1.91 -0.923 -1.522

BI474953 0.452 0.064 -0.158 -0.053 -0.034 0.154 -0.036 -0.068 0.262 0.683 0.532 0.699

BI474957 1.387 0.698 -0.497 0.015 -0.211 -0.231 0.015 -0.453 -0.525 -0.572 -0.64 -0.572

BI475104 0.664 0.749 0.097 0.236 0.101 0.355 0.327 0.426 -0.069 -0.203 -0.147 -0.197

BI475215 0.073 -0.05 -0.24 -0.019 -0.095 0.119 -0.127 -0.163 0.079 0.1 0.134 1.345

BI475648 1.855 0.641 -0.452 -0.044 0.217 0.479 0.011 0.131 -0.218 0.018 -0.078 -0.34

BI475794 1.108 0.485 1.92 1.892 2.347 2.82 2.145 2.367 1.589 1.619 1.017 0.375

BI475856 0.36 0.483 1.043 1.407 1.648 0.711 1.143 1.31 0.41 0.055 0.037 -0.124

BI475873 -0.375 0.95 2.319 2.254 1.475 0.507 0.473 0.399 0.077 -0.174 -0.522 -0.365

BI476025 0.38 0.717 0.928 0.912 1.282 0.61 0.637 1.305 0.391 0.263 0.068 0.364

BI476031 -0.211 0.045 -0.309 0.466 0.539 0.025 -0.057 0.504 0.281 0.479 0.121 0.072

BI476292 1.284 0.631 -0.449 -0.39 -0.141 -0.149 -0.564 -0.14 -0.312 -0.579 -0.503 -0.354

BI476547 0.147 0.468 0.546 0.176 0.55 0.333 0.265 1.093 0.116 0.204 -0.104 -0.039

BI476729 0.063 -0.106 0.142 0.163 0.568 0.101 0.344 0.921 -0.099 0.38 0.234 0.358

BI476846 -0.546 -1.171 -1.035 0.201 0.715 1.199 0.479 0.38 0.234 0.752 0.489 1.006

BI476854 -1.031 -0.864 0.567 1.176 1.276 1.893 1.312 1.323 0.636 0.836 0.459 0.749

BI533160 0.686 -0.172 0.495 0.916 1.614 2.26 1.59 2.254 1.444 1.623 1.002 0.405

BI533161 -0.951 -2.53 -1.153 -1.991 -2.07 -0.193 -1.051 -2.653 -1.072 -0.066 0.536 1.081

BI533326 0.613 0.902 0.66 0.477 0.132 0.165 -0.208 0.491 0.069 0.306 0.177 0.05

BI533952 1.515 1.093 1.14 1.623 1.902 2.304 1.646 1.891 0.953 0.857 0.419 -0.161

BI534059 -0.754 -1.263 -0.584 -0.022 0.18 0.589 0.284 0.751 0.354 0.814 0.498 0.194

BI534261 0.322 0.599 0.563 0.331 -0.068 0.22 0.171 0.243 -0.165 0.276 -0.202 -0.345

BI534277 0.08 -0.005 0.001 0.202 -0.018 0.09 -0.01 -0.325 -0.048 0.061 0.449 0.306

BI534295 -0.302 -0.16 0.223 -0.355 -0.043 0.146 -0.087 -0.47 -0.143 -0.268 0.555 0.56

BI534308 0.736 0.363 -0.25 -0.004 -0.205 0.303 -0.383 -0.051 -0.214 0.263 0.238 0.122

BI562940 -0.06 -0.044 0.106 0.389 0.862 1.158 0.364 1.418 0.699 1.054 0.773 0.669

BI563035 0.012 1.03 0.775 0.162 0.003 -0.074 -0.24 -0.052 0.212 -0.255 0.043 -0.148

BI563231 -3.401 -3.266 -2.836 -1.927 -2.733 -0.643 -0.943 -0.335 -0.309 0.219 0.32 1.05

BI670841 0.672 1.113 0.774 0.849 -0.207 0.027 -0.254 0.022 -0.064 0.105 0.16 -0.305

BI670852 0.539 -0.255 0.195 0.538 0.909 0.766 0.682 1.346 0.534 1.099 0.76 0.529

BI670932 -1.039 -1.085 -1.016 -0.629 -0.621 -0.544 -0.571 -0.696 -0.721 -0.136 -0.13 0.518

BI670969 0.465 0.996 0.739 1.126 0.581 0.955 0.51 0.999 0.635 0.901 0.381 0.071

BI671110 0.693 0.865 1.258 1.018 0.889 1.353 0.389 0.852 0.213 0.235 0.119 -0.031

BI671227 -0.691 -0.282 1.073 0.852 1.665 1.088 0.985 0.875 0.376 0.108 0.038 0.274

BI671843 0.981 0.709 0.699 0.495 1.081 1.446 0.977 1.355 0.8 -0.191 -0.577 -0.349

BI671845 1.553 1.658 1.55 0.31 0.295 0.444 0.245 0.507 0.332 0.257 0.058 0.031

BI672022 -1.198 -1.693 -2.077 -2.443 -2.278 -1.256 -1.318 -1.925 -1.458 0.387 0.402 1.391

BI672025 1.221 1.519 1.11 1.252 0.744 1.022 0.599 0.918 0.149 0.187 -0.2 -0.306

BI672045 0.49 -0.19 0.056 0.397 0.617 0.367 0.343 0.595 0.088 0.091 0.087 0.105

BI672058 -0.224 -0.816 0.346 0.071 0.371 0.61 0.381 0.825 0.307 0.334 0.672 0.194

BI672089 0.881 1.08 -0.11 0.126 0.26 0.096 0.133 0.334 0.088 -0.407 0.183 -0.41

BI672095 1.143 0.546 0.615 0.413 0.273 0.266 0.184 0.135 0.089 -0.037 0.164 -0.252

BI672139 0.087 0.39 0.466 1.169 1.179 0.633 0.694 0.772 0.179 0.516 0.228 0.259

BI672150 -0.368 -0.524 0.397 0.384 0.26 0.432 0.402 0.652 0.362 0.936 0.291 0.416

BI672201 -0.34 -0.173 0.293 0.353 0.562 1.222 0.619 1.451 0.698 0.91 0.307 0.035

BI672214 -0.075 0.335 0.66 0.786 0.62 0.046 0.149 0.499 0.06 0.154 0.19 0.183

BI672216 -0.045 1.33 0.642 1.018 0.624 -0.203 0.235 0.527 -0.114 -0.397 -0.742 -0.106

BI672219 0.247 -0.101 -0.38 0.034 0.498 0.312 0.19 0.671 0.086 -0.432 0.067 -0.231

BI672273 0.872 -0.147 -1.459 -0.593 -0.147 -0.57 -0.614 -0.622 -0.659 -0.302 -0.42 -0.507

BI672289 -0.225 -0.651 -1.036 -0.329 -0.207 0.385 0.344 0.714 0.346 0.67 0.566 0.753

BI672301 -0.881 -0.728 0.197 0.229 0.258 0.355 0.448 0.755 0.374 0.944 0.543 0.096

BI672308 -0.728 0.037 -0.058 0.184 0.32 -0.059 -0.229 0.147 0.141 0.434 0.518 -0.261

BI672337 -1.251 -1.507 -1.458 -0.47 0.104 1.041 1.026 1.491 0.245 0.77 -0.285 -0.201

BI672347 -0.501 -1.061 0.898 1.444 1.845 1.542 0.77 0.734 -0.149 0.027 -0.178 -0.484

BI672378 0.741 -0.952 0.516 0.615 0.235 0.259 0.224 0.001 -0.25 0.56 0.177 -0.188

BI672391 -0.006 0.094 1.156 1.734 1.935 0.353 0.341 0.781 0.358 0.445 0.093 0.042

BI672394 0.8 0.316 0.466 0.734 1.169 1.874 1.096 0.768 0.222 0.236 0.065 -0.173

BI672395 -1.685 -0.208 1.617 1.404 1.689 1.504 0.723 0.741 0.986 0.604 0.78 -0.446

BI672450 0.428 1.467 0.897 0.866 0.429 -0.325 -0.219 0.198 -0.058 -0.221 -0.211 -0.3

BI672464 1.088 -0.034 -1.072 -0.582 -0.596 -0.45 -0.764 -1.128 -0.762 -1.046 -1.188 -0.731

BI672468 0.751 -0.519 0.038 -0.274 -0.745 -0.132 -0.255 -1.037 -0.677 -0.222 -1.157 -0.932

BI672475 -0.308 1.059 0.67 0.706 -0.155 -0.314 0.009 0.206 0.036 -0.14 -0.106 -0.172

BI672508 -0.783 -3.033 -1.339 -1.012 -0.677 0.484 0.127 0.473 0.401 1.198 0.868 0.25

BI672555 1.229 0.013 -0.428 0.017 0.15 0.6 0.249 -0.084 -0.158 0.039 0.068 0.328

BI672616 1.559 0.042 -0.228 -0.011 -1.415 -0.724 -1.478 -2.638 -1.262 -1.386 -1.721 -0.943

BI672630 1.316 0.721 0.035 0.044 -0.526 0.307 0.115 -0.289 -0.227 0.004 -0.26 -0.29

BI672656 -0.605 -1.062 -1.097 -0.743 -1.02 -0.012 -0.72 0.01 0.204 0.562 0.487 0.55

BI672765 0.059 0.622 0.077 -0.276 1.064 0.033 0.593 1.155 -0.073 -0.205 0.002 -0.093

BI672781 0.394 0.915 1.893 0.84 1.125 0.726 0.389 0.795 0.427 0.396 0.268 0.276

BI672829 2.547 0.527 -0.903 -1.725 -1.4 -1.287 -1.131 -1.923 -2.474 -1.877 -4.203 -3.296

BI673276 1.27 0.148 -1.438 -0.774 -0.769 0.159 0.158 0.634 -0.096 0.027 -0.466 -0.756

BI673308 1.098 0.669 0.132 0.394 0.31 0.488 0.139 0.591 0.186 -0.012 -0.323 -0.655

BI673379 0.753 0.688 0.925 0.464 0.553 0.563 0.375 1.037 0.089 0.173 -0.032 -0.124

BI673408 0.042 0.574 0.613 0.072 -0.179 0.075 0.052 -0.248 -0.023 0.202 -0.239 -0.049

BI673416 0.415 0.891 2.481 2.38 2.425 2.695 2.027 1.668 0.991 0.813 0.404 -0.074

BI673444 0.943 1.002 0.207 0.268 0.012 -0.086 0.058 -0.224 -0.281 -0.241 -0.759 -0.892

BI673452 2.379 -0.165 -0.093 -0.248 -1.924 -1.703 -1.938 -3.082 -1.782 -2.238 -2.128 -2.426

BI673470 -0.578 -0.147 0.521 -0.063 -0.252 -0.032 0.345 -0.344 0.094 -0.129 0.063 1.458

BI673483 2.623 0.277 -2.229 -2.813 -2.008 -1.696 -2.105 -2.001 -3.12 -3.62 -3.256 -3.394

BI673487 0.207 0.846 1.328 1.305 1.15 0.35 0.874 1.165 0.426 0.401 -0.047 -0.586

BI673488 1.353 1.427 0.244 0.221 0.223 0.774 0.194 0.144 -0.381 -0.167 -0.3 -0.367

BI673509 1.629 0.492 0.081 0.501 0.305 0.317 -0.061 -0.095 0.044 -0.754 -0.045 -0.369

BI673511 0.491 1.015 0.671 0.949 0.362 -0.158 -0.11 -0.945 -0.842 -0.72 -1.533 -0.89

BI673527 0.227 1.081 0.595 0.709 0.983 0.602 0.299 0.944 0.335 0.41 0.475 0.027

BI673573 -0.009 -0.327 0.074 0.113 0.82 1.942 1.719 1.875 1.404 1.17 0.307 0.115

BI673579 1.293 1.008 -0.777 -0.432 -0.539 0.052 -0.619 -0.22 -0.475 -0.3 -0.539 -0.567

BI673606 -0.078 0.373 0.325 0.767 0.994 0.313 0.526 1.359 0.067 0.529 0.105 -0.048

BI673663 0.599 1.362 1.025 0.838 0.373 0.054 0.156 0.839 0.786 0.08 0.416 -0.413

BI673712 -0.449 0.344 0.783 2.888 3.442 0.682 0.488 0.994 0.043 -0.2 0.474 0.084

BI673727 0.895 1.288 1.008 0.978 0.59 0.466 0.177 0.758 -0.088 -0.368 -0.479 -0.768

BI673772 0.224 0.062 0.624 0.665 0.526 0.323 0.399 0.605 0.165 0.634 0.272 -0.05

BI704177 -0.32 0.568 0.185 1.051 1.353 0.698 0.587 0.816 0.419 -0.114 -0.058 0.144

BI704180 0.319 0.358 0.727 0.517 0.876 0.657 0.255 1.283 0.477 0.025 -0.332 -0.762

BI704181 0.044 0.77 0.39 0.605 0.324 -0.226 -0.049 0.842 -0.02 -0.041 -0.433 -0.452

BI704199 0.322 -0.087 0.861 1.916 1.706 -0.142 0.45 1.02 0.116 0.357 0.209 -0.02

BI704236 0.017 0.672 1.448 1.486 1.461 1.704 0.918 2.217 0.828 0.741 0.236 -0.293

BI704240 0.701 1.191 1.142 1.114 1.191 0.801 0.948 0.971 0.007 0.175 -0.387 -0.856

BI704244 0.209 -0.035 0.77 0.402 0.5 1.056 0.685 1.569 1.168 0.698 0.738 -0.351

BI704249 -1.19 -2.038 1.18 2.333 3.317 3.151 2.768 3.828 2.173 1.371 1.122 0.344

BI704267 0.288 1.392 1.552 1.613 1.286 0.773 0.23 1.092 0.071 0.522 0.147 0.173

BI704278 0.235 0.826 1.924 2.043 1.917 1.005 1.357 0.819 0.016 0.76 0.369 -0.452

BI704280 -0.279 -0.691 0.123 0.992 0.841 0.25 0.523 1.311 0.568 0.54 0.595 0.417

BI704281 -4.509 -4.245 0.937 1.408 1.815 2.267 1.964 1.85 1.03 0.217 0.049 -0.251

BI704288 -0.894 -0.719 1.727 2.316 2.584 3.313 2.896 2.643 1.91 1.66 1.137 0.856

BI704293 -0.728 -0.233 0.216 0.152 0.847 1.076 0.945 1.281 0.536 0.69 0.641 0.112

BI704306 0.651 0.732 1.348 0.204 0.337 0.384 -0.095 0.007 0.092 -0.205 -0.412 -0.536

BI704310 -0.505 0 4.183 4.459 5.947 4.662 2.738 3.76 1.964 0.446 0.184 0.359

BI704311 0.132 0.78 1.303 1.045 0.71 0.186 -0.725 -0.163 0.388 -0.338 0.088 -0.469

BI704324 -2.499 -3.043 -2.506 -2.699 -2.57 -1.229 -1.429 -3.134 -1.655 -0.749 0.615 1.315

BI704334 -0.37 -1.092 0.676 1.06 0.673 -0.031 0.064 -0.185 -0.454 -0.508 -0.564 -0.576

BI704340 0.287 0.393 0.872 0.953 1.269 1.434 1.216 1.16 0.764 0.705 0.359 0.344

BI704344 -0.438 -0.08 2.02 2.297 2.045 2.031 1.203 1.416 0.997 0.779 0.709 0.558

BI704353 0.195 0.77 0.576 0.695 0.664 0.052 0.13 0.133 -0.04 -0.005 0.111 0.301

BI704359 -0.304 -1.06 0.096 0.645 1.735 0.303 0.528 1.749 0.437 0.283 0.529 0.44

BI704370 1.248 0.597 0.979 1.223 0.677 0.732 0.574 0.119 -0.05 -0.299 -0.838 -0.621

BI704373 0.171 0.492 0.906 1.354 1.233 0.689 0.306 0.779 0.173 0.215 0.042 -0.147

BI704393 0.291 -0.534 -0.097 0.648 0.603 0.552 0.684 1.307 0.47 0.112 -0.117 -0.67

BI704401 0.369 0.973 0.851 0.954 0.276 0.068 0.3 -0.106 -0.351 -0.268 -1.035 -0.819

BI704416 0.519 0.628 1.282 1.21 1.071 0.25 0.443 0.904 0.105 0.3 0.008 0.143

BI704420 -0.745 0.815 3.803 2.866 1.771 1.245 1.156 1.671 1.334 0.652 0.593 0.217

BI704422 -0.373 -0.027 1.213 1.078 1.615 1.338 0.931 1.354 0.694 0.973 0.218 -0.146

BI705182 -0.728 -0.185 0.155 0.106 0.832 0.507 0.536 1.471 0.908 0.234 0.708 0.082

BI705282 1.687 1.074 -0.022 0.135 0.004 0.45 0.207 0.517 0.255 0.151 0.173 -0.393

BI705495 0.542 0.451 0.935 0.459 0.38 0.703 0.184 0.313 0.036 0.184 -0.08 -0.037

BI705506 -0.592 -0.513 0.105 0.828 0.714 0.566 0.508 0.633 0.291 0.431 0.032 0.169

BI705512 -0.279 1.584 1.209 0.988 0.115 -0.431 -0.232 -0.161 -0.43 -0.749 -1.225 -0.843

BI705519 1.161 1.77 1.023 0.858 0.341 0.405 0.027 0.178 0.154 -0.261 0.317 -0.246

BI705525 0.913 0.826 0.36 0.629 0.734 1.295 0.191 0.768 0.267 0.271 -0.195 -0.177

BI705531 0.451 -0.448 -0.185 0.026 -0.271 -0.12 -0.293 -0.11 -0.002 0.402 0.639 -0.018

BI705594 1.886 0.066 -1.1 -1.604 -1.423 -0.48 -1.489 -0.951 -0.804 -0.878 -0.797 -0.467

BI705646 -0.174 1.013 0.355 0.439 0.149 -0.225 0.006 0.625 0.032 -0.252 -0.111 0.004

BI705720 1.174 0.015 0.078 -0.426 0.11 0.276 -0.408 0.04 0.262 0.447 0.622 0.502

BI705721 0.513 0.615 -0.446 0.172 0.805 0.625 0.769 0.905 0.443 0.188 -0.196 -0.405

BI705734 -0.614 -0.092 0.379 0.511 0.832 0.082 0.036 0.305 -0.171 -0.707 0.05 0.033

BI705843 -1.068 -0.873 -1.079 -0.68 -0.974 -0.614 -0.302 -1.079 -0.707 0.545 0.944 0.482

BI705891 0.076 -0.753 0.636 0.627 0.696 1.263 0.992 1.09 0.645 0.533 0.268 0.382

BI705930 -0.413 0.053 0.891 1.043 1.799 0.857 0.903 2.107 0.961 0.614 0.353 -0.268

BI706099 -0.055 0.244 -0.038 0.179 0.294 0.177 0.38 0.61 0.124 0.205 -0.161 0.187

BI706176 1.306 0.974 0.416 0.283 0.195 0.546 0.235 0.712 0.209 0.414 0.109 -0.244

BI706321 -0.484 0.142 0.338 0.991 1.014 0.312 0.059 0.287 -0.069 -0.554 -0.128 -0.341

BI706468 -0.031 -0.679 -0.415 -0.615 0.198 0.437 0.464 0.791 0.357 0.786 0.29 0.084

BI706873 0.174 0.771 0.55 0.745 0.323 0.275 0.243 0.802 0.11 0.42 -0.024 -0.039

BI706908 -0.16 -1.223 0.45 1.101 0.917 0.536 0.776 1.256 0.046 0.171 0.276 -0.005

BI706999 -0.365 0.989 0.135 0.789 0.906 0.159 0.296 0.841 -0.04 0.104 0.392 -0.182

BI707410 -0.587 -3.041 -1.196 -0.901 -0.492 0.486 0.33 0.498 0.231 1.061 0.627 0.072

BI707482 -0.38 -1.249 -0.961 -0.459 0.531 0.528 0.432 0.733 0.787 0.623 0.403 0.525

BI707547 -0.244 0.363 1.932 1.169 0.996 0.469 0.229 0.353 0.051 -0.055 -0.442 -0.228

BI707695 0.19 0.603 0.692 0.423 0.45 -0.166 -0.337 0.05 -0.088 0.326 0.11 -0.003

BI708092 -0.788 -0.604 -0.383 -0.128 0.335 0.018 -0.092 0.278 0.515 0.155 0.813 0.763

BI708259 0.791 0.14 0.616 1.035 1.09 1.226 0.64 0.976 0.436 0.828 0.341 0.321

BI708320 2.478 0.515 -0.776 -0.754 -1.52 -1.034 -1.279 -1.834 -1.67 -1.795 -1.709 -1.215

BI708386 0.701 0.832 1.589 1.486 1.719 1.861 1.322 1.448 0.979 0.428 0.281 -0.1

BI708455 1.921 -0.034 -2.471 -0.983 -1.432 -1.185 -0.889 -1.566 -1.36 -2.74 -2.636 -2.164

BI708483 -0.421 2.093 1.921 1.611 0.45 0.286 -0.407 -0.455 0.044 -0.296 -0.033 -0.83

BI708766 -1.55 -0.564 0.724 0.98 0.452 0.329 -0.011 0.701 0.229 0.753 0.055 0.106

BI708781 0.073 0.201 0.265 0.527 0.675 0.398 0.281 0.786 0.106 0.247 -0.006 -0.206

BI708877 -0.344 -0.764 -0.158 0.165 0.076 -0.091 -0.005 -0.069 0.111 0.174 0.388 0.273

BI709110 -0.53 0.104 0.649 0.521 1.185 0.827 0.238 1.217 0.463 0.623 0.325 0.623

BI709146 1.556 0.341 -0.024 0.17 0.381 1.13 0.013 1.059 0.47 0.49 0.232 -0.223

BI709409 0.544 0.386 0.13 0.31 0.498 0.805 0.602 1.003 0.812 0.477 0.515 0.138

BI709411 0.918 1.315 1.12 0.687 0.221 0.014 -0.143 0.03 -0.07 -0.068 -0.006 0.085

BI709417 1.272 -1.539 -0.584 -1.482 -1.516 0.243 -0.398 -1.047 -0.546 -0.056 -0.219 0.072

BI709620 0.032 -0.252 -0.603 -0.133 0.059 0.526 -0.067 0.751 0.044 0.553 0.28 0.403

BI709715 0.317 0.536 0.873 0.52 0.635 0.295 0.323 0.717 0.04 0.362 0.157 0.014

BI709743 1.497 0.407 -0.121 -0.195 0.056 0.361 -0.001 0.316 -0.01 0.191 0.074 -0.369

BI709770 0.998 -0.123 -0.119 0.08 0.237 0.416 -0.08 -0.077 -0.159 -0.152 -0.425 -0.499

BI709791 0.401 0.147 0.542 0.974 0.961 0.605 0.91 1.311 0.118 0.368 0.124 -0.011

BI709805 -0.728 0.639 0.421 0.89 1.042 1.081 0.116 1.154 -0.378 -0.468 -0.543 -0.913

BI709862 1.034 0.046 -0.458 0.024 -0.276 -0.386 -0.302 -0.601 -0.212 -0.366 -0.881 -0.498

BI709863 2.316 0.367 -0.115 0.817 0.283 0.077 -0.373 0.039 -0.261 -0.673 -1.012 -0.903

BI710035 0.837 1.271 0.675 0.1 -0.476 -0.174 -0.2 -0.413 -0.364 -0.406 -0.235 -0.549

BI710046 1.126 -0.3 0.149 0.049 0.245 0.423 0.048 0.419 0.013 0.31 0.075 -0.179

BI710051 -0.037 0.579 0.505 0.256 -0.097 0.487 0.388 0.65 0.534 0.469 -0.082 -0.392

BI710147 -0.552 -2.654 -0.952 -1.001 -0.428 0.616 0.315 0.443 0.249 1.234 0.854 0.227

BI710295 -2.211 -3.349 -0.592 -0.752 -0.206 -0.391 -0.467 -0.939 0.43 -0.376 0.427 0.18

BI710499 -0.685 0.793 1.127 0.78 0.957 -0.202 -0.232 0.294 0.062 -0.165 -0.027 -0.076

BI710508 0.044 0.492 -0.192 -0.095 -0.21 -0.245 0.115 -0.069 0.138 -0.024 0.293 0.587

BI710730 -0.712 0.204 1.652 1.848 1.684 0.801 0.598 1.066 0.859 0.378 -0.424 -1.049

BI839625 1.403 0.177 -1.091 -0.235 -0.799 -0.886 -0.59 -0.954 -0.6 -1.145 -0.786 -0.943

BI839727 0.324 -0.324 0.422 0.321 0.342 0.881 0.988 0.524 0.239 0.253 -0.064 -0.138

BI839826 -0.27 -0.1 0.991 -0.667 0.257 0.081 0.769 1.683 0.311 -0.042 0.515 0.173

BI839927 -1.594 -1.453 -2.144 -1.338 -1.4 -0.594 -1.089 -1.931 -1.114 -0.407 0.065 1.837

BI839952 -0.362 -0.75 -0.205 0.339 -0.353 -0.163 0.399 0.406 0.558 1.287 0.729 0.287

BI840104 -0.486 0.069 0.581 0.927 1.169 -0.032 0.011 0.363 0.209 -0.034 0.059 -0.008

BI840279 -0.202 1.538 1.277 1.279 0.686 0.192 0.012 0.373 -0.125 -0.371 -0.234 -0.348

BI840365 0.955 -0.92 -0.672 -0.228 -0.218 0.258 0.04 0.043 -0.425 0.008 -0.18 -0.134

BI840456 0.264 0.572 1.064 0.536 1.259 1.61 1.193 1.848 0.682 0.792 0.294 0.161

BI840738 1.678 1.31 2.136 1.531 1.004 1.276 0.808 0.763 0.403 0.23 0.007 -0.502

BI840762 -1.311 0.002 -0.776 -0.45 -0.265 -0.282 -0.613 -0.856 -0.746 -0.251 0.275 2.068

BI840839 0.129 -0.408 0.438 0.397 0.514 1.425 0.553 0.831 0.749 0.567 0.566 0.323

BI840845 -0.559 -0.773 0.458 0.821 0.14 0.708 0.722 0.705 0.223 0.158 -0.147 -0.093

BI840935 0.209 -0.514 0.441 0.929 0.445 1.432 0.696 0.232 0.261 0.718 0.275 -0.303

BI840953 -0.082 -0.409 0.192 0.251 0.13 0.851 0.409 0.148 0.179 0.174 0.301 0.65

BI840999 -0.503 -0.803 -0.342 -1.094 -1.692 -0.519 -0.076 -0.556 -0.559 0.136 0.074 1.717

BI841076 -0.681 0.197 0.33 0.745 0.871 0.254 0.059 1.281 0.29 0.453 -0.033 -0.511

BI841477 -0.549 0.388 0.506 0.828 1.125 0.551 0.293 1.081 0.034 0.184 0.296 0.043

BI841627 0.641 0.732 1.335 1.354 1.279 1.143 0.951 1.659 0.752 0.76 0.279 -0.092

BI841667 -0.607 -0.063 0.016 -0.243 0.103 -0.396 -0.216 -0.166 -0.398 -0.36 0.246 0.812

BI842047 -0.425 0.053 -0.061 0.36 0.344 -0.238 -0.184 0.412 -0.038 0.036 0.576 0.146

BI842162 -0.211 -0.151 1.002 1.192 1.394 0.868 0.376 0.775 0.995 -0.123 0.512 0.356

BI842428 1.108 1.221 1.687 1.107 0.482 0.888 0.57 1.164 0.268 0.736 0.544 0.065

BI842822 -1.016 -0.271 -0.224 0.258 1.266 0.882 0.371 1.271 1.214 0.726 -0.008 -0.18

BI842851 -0.886 -1.071 -1.46 -0.259 -0.862 -0.323 0.065 0.128 -0.004 0.713 0.211 0.311

BI842900 -0.559 -3.581 -1.423 -1.184 -0.556 0.15 0.087 0.471 0.181 1.112 0.699 0.002

BI842921 -0.811 -2.677 -1.475 -1.214 -0.899 0.445 0.084 0.342 0.212 1.18 0.723 0.355

BI843105 0.52 1.179 0.885 0.737 0.522 0.425 0.296 0.822 0.068 0.245 0.109 -0.201

BI843117 0.359 0.803 0.531 1.109 0.83 0.267 0.244 0.644 -0.102 0.063 -0.24 -0.069

BI843129 0.032 0.708 0.535 0.885 0.934 -0.018 0.376 1.124 0.078 0.23 0.099 -0.296

BI843130 1.551 1.101 0.266 0.242 -0.313 -0.207 -0.467 -0.374 -0.531 -0.653 -1.025 -0.656

BI843185 0.931 -0.005 -0.052 -0.719 0.026 -0.192 -0.034 -0.417 -0.192 -0.685 -0.274 -0.124

BI843229 -0.197 -0.159 0.272 0.295 0.645 1.666 0.914 1.167 0.626 1.01 0.149 0.162

BI843230 0.316 -0.747 0.132 0.234 0.402 0.536 0.324 0.727 0.139 0.246 -0.305 -0.682

BI843250 1.607 1.163 1.105 0.888 -0.183 0.037 -0.568 -0.396 -0.341 -0.232 -0.554 -0.528

BI843286 2.118 -1.165 -1.262 -2.332 -2.77 -1.576 -2.302 -3.616 -2.02 -1.951 -3.558 -2.277

BI843291 0.457 1.247 0.854 0.549 -0.49 -0.346 -0.399 -0.614 -0.641 -0.334 -0.793 -1.003

BI843298 0.861 1.404 1.052 0.612 -0.001 -0.148 -0.277 0.02 -0.176 -0.37 -0.473 -0.553

BI843480 1.493 -0.026 -0.108 -0.187 -0.166 -0.125 -0.414 -0.405 -0.626 -1.171 -0.591 -0.637

BI843519 -0.63 -0.289 -0.402 -0.171 0.878 -0.011 0.19 1.519 0.458 -0.708 -0.575 -0.416

BI844046 0.564 1.457 1.152 0.778 0.651 1.195 0.776 1.011 0.308 0.335 0.051 -0.1

BI844059 1.075 0.423 0.204 0.057 -0.251 0.514 -0.1 -0.126 -0.041 -0.245 -0.703 -0.775

BI845254 -0.043 0.062 0.657 0.481 0.547 0.322 0.324 0.238 0.41 -0.118 -0.263 -0.038

BI845363 0.911 0.056 0.01 0.144 0.078 -0.135 0.108 0.28 -0.349 -0.031 -0.189 0.028

BI845367 -0.734 -0.238 -0.375 -0.398 -0.243 -0.157 -0.183 -0.676 -0.332 -0.137 0.32 1.005

BI845510 -0.728 -0.12 0.041 0.182 0.888 0.869 0.996 0.957 1.059 0.31 1.139 0.931

BI845638 -0.136 0.287 1.001 0.478 0.798 0.45 0.07 0.436 0.189 0.168 0.062 -0.149

BI845763 0.767 0.324 -0.576 -0.119 -0.711 -0.372 -0.084 -0.433 -0.322 -0.352 -0.279 -0.472

BI845781 -0.008 0.347 0.11 0.182 0.395 -0.03 0.183 0.903 0.18 0.137 0.487 0.039

BI845814 0.069 0.095 -0.177 0.134 -0.256 0.089 0.047 0.549 0.251 0.205 0.046 0.269

BI845927 0.803 0.913 0.868 0.394 -0.153 0.232 -0.094 0.241 -0.061 -0.225 -0.416 -0.332

BI846219 -0.828 0.556 1.608 0.45 1.403 0.097 0.279 0.744 0.729 0.36 0.645 -0.455

BI846231 -1.586 -2.519 -1.313 -2.333 -2.094 -0.479 -1.142 -2.751 -1.55 -0.96 -0.386 0.686

BI846235 1.147 0.449 -0.409 -0.154 -0.693 -0.286 -0.745 -0.817 -0.348 -0.334 -0.291 -0.315

BI846592 -0.52 -0.291 -0.575 0.186 1.094 1.081 0.554 1.892 0.678 1.301 0.118 -0.436

BI846833 -2.254 -2.063 -0.124 0.504 0.823 1.352 1.334 0.954 1.512 1.114 1.139 0.442

BI846965 -0.173 0.548 0.688 0.639 1.441 -0.004 0.252 0.862 0.036 0.144 0.164 -0.004

BI847093 -1.038 -1.035 -1.448 -0.391 -0.717 -0.215 -0.219 0.086 -0.162 0.584 0.681 0.28

BI850015 -0.511 -0.138 2.214 1.239 1.222 0.389 0.339 1.019 0.236 1.155 0.701 0.261

BI850028 -1.04 -1.585 0.542 1.324 2.291 2.079 1.427 2.39 1.744 0.82 0.474 0.098

BI850032 0.378 -0.529 -0.862 -0.542 0.142 0.891 1.139 1.014 0.839 0.64 0.142 0.3

BI850036 -0.381 0.056 0.543 -0.309 -0.03 0.063 0.425 0.655 0.134 -0.065 0.434 0.083

BI850039 0.1 0.126 0.228 0.623 0.88 0.045 0.356 1.09 0.158 0.709 0.145 0.304

BI863932 -1.902 -1.064 -1.738 -0.537 -1.776 -0.427 -1.045 -1.593 -1.029 -0.317 -0.176 1.566

BI863967 0.131 0.228 0.98 0.564 0.477 0.53 0.502 0.953 0.076 0.077 -0.141 -0.179

BI863998 -0.506 -0.04 -0.259 -0.149 -0.883 -0.452 -0.477 -0.69 -0.065 0.18 0.163 -0.643

BI864018 0.576 0.546 1.606 1.329 0.796 0.934 0.57 0.862 0.35 0.018 0.081 0.004

BI864033 0.205 0.897 0.792 0.574 -0.042 -0.224 -0.342 -0.022 0.102 -0.199 -0.059 -0.348

BI864067 0.606 -0.813 -0.41 -0.222 -0.563 -0.282 -0.398 -0.699 -0.573 0.204 0.169 0.115

BI864190 -1.084 -0.7 -1.045 -0.167 -0.089 1.079 0.966 1.528 1.245 1.569 1.186 -0.003

BI864451 0.26 0.605 0.67 0.508 0.044 0.342 0.226 0.95 0.162 0.759 0.497 0.026

BI864920 -0.704 -1.545 -1.307 -0.933 -1.6 -0.64 -0.762 -1.528 -0.211 -0.748 -0.089 0.896

BI864988 0.02 0.033 0.816 1.309 0.955 0.46 0.297 0.755 0.245 -0.151 0.057 -0.095

BI865356 1.128 1.204 -0.144 0.115 0.162 0.123 0.129 -0.038 -0.045 -0.238 -0.155 -0.448

BI865412 0.194 -0.362 0.465 0.368 0.402 1.24 0.365 0.484 0.31 0.609 0.398 0.415

BI865459 -0.46 0.092 -0.209 0.762 1.432 0.648 0.419 0.758 -0.128 0.157 0.128 0.875

BI865477 -0.766 -0.882 -1.301 -1.439 -0.642 -1.073 -0.96 -1.381 -0.722 0.236 0.8 1.444

BI865609 -1.249 -0.931 -0.229 -0.817 -0.646 -0.108 -0.153 -0.203 -0.24 -0.527 0.081 1.259

BI865754 -0.415 -1.431 0.346 0.38 0.672 0.692 0.566 0.398 0.439 0.871 0.826 1.202

BI865765 -0.074 0.963 1.134 0.364 0.134 -0.041 -0.401 -0.391 -0.389 -0.642 -0.538 -0.568

BI865912 -0.728 -0.334 -0.036 -0.89 -0.972 -0.522 -0.312 -0.932 -0.284 -0.075 0.796 1.537

BI865976 -1.248 -0.042 0.828 1.11 0.892 0.673 0.411 -0.5 0.548 -0.104 0.274 -0.466

BI865978 -1.666 -2.161 -0.524 -0.348 -0.393 -0.478 -0.242 0.078 0.241 0.604 0.163 -0.147

BI866264 0.513 1.244 1.441 0.723 0.437 0.48 0.361 0.871 0.004 0.207 -0.072 -0.031

BI866308 0.261 -0.022 0.261 0.563 1.093 0.858 0.592 1.13 0.412 0.121 0.797 0.847

BI866326 -1.072 -1.154 -0.76 -1.47 -2.553 -1.016 -0.852 -2.017 -1.135 -1.357 0.19 1.484

BI866335 -0.701 -0.154 -0.025 -0.621 -0.763 -0.016 -0.555 -0.55 -0.13 -0.692 0.666 0.729

BI866342 0.452 0.068 -0.178 0.055 0.278 0.625 0.425 0.881 0.291 0.397 0.016 0.007

BI866448 0.095 0.187 0.309 0.309 0.682 0.279 0.065 0.91 0.125 0.223 0.033 0.225

BI866527 0.729 0.424 0.127 0.178 0.309 0.523 -0.042 0.104 0.095 -0.014 0.023 -0.04

BI866724 1.389 1.367 0.559 0.235 -0.348 0.337 -0.099 0.118 -0.277 -0.685 -0.861 -0.739

BI866770 1.308 1.313 0.356 0.219 0.283 0.233 -0.025 0.298 -0.118 -0.009 -0.113 -0.275

BI866874 1.109 -0.22 -0.653 -0.521 0.407 0.169 -0.324 -0.467 -0.429 -0.005 -0.188 -0.564

BI866879 0.177 -0.048 1.255 1.347 1.475 0.306 0.033 0.297 -0.343 -0.067 0.019 -0.558

BI866952 0.454 0.427 0.565 0.435 -0.452 -0.457 -0.39 -0.476 -0.127 0.738 0.87 -0.239

BI866963 2.231 -2.741 -2.877 -2.768 -2.901 -1.046 -2.767 -2.836 -2.166 -3.318 -3.14 -2.11

BI866976 0.714 -0.115 0.081 0.285 0.572 0.336 0.768 1.078 0.118 0.656 0.839 0.791

BI866992 0.81 0.445 0.665 1.102 1.446 0.983 0.903 1.332 0.517 0.672 0.047 -0.166

BI867066 2.461 1.229 0.932 1.043 0.177 0.817 0.618 0.272 -0.129 -0.187 -0.988 -1.367

BI867089 1.554 0.931 -0.418 0.05 -2.49 -1.309 -0.972 -2.543 -2.112 -1.616 -3.469 -2.513

BI867169 0.964 -0.489 -0.236 -0.392 -0.307 -0.111 -0.467 -0.419 -0.775 -0.141 -0.297 0.087

BI867171 -0.005 -0.894 -0.899 -0.407 -0.091 0.917 0.689 0.719 0.394 0.643 0.307 0.051

BI867235 1.097 0.62 -0.222 -0.299 -0.33 0.116 -0.256 -0.309 -0.321 -0.051 -0.309 0.144

BI867240 1.03 0.68 0.306 0.422 0.241 0.178 0.343 0.761 0.144 0.031 0.106 -0.008

BI867248 1.401 -1.282 -1.543 -0.915 -1.181 -0.56 -1.162 -1.374 -1.005 -1.512 -1.009 -1.492

BI867261 0.561 0.41 0.716 0.75 1.328 0.767 0.466 0.976 0.279 0.435 0.345 0.069

BI867264 1.498 0.429 -0.878 -0.609 -1.718 -1.304 -0.9 -2.424 -1.789 -1.439 -1.574 -1.503

BI867308 0.302 1.156 0.323 0.782 0.769 -0.087 0.081 0.45 0.299 -0.246 -0.357 -0.411

BI867396 0.764 0.498 0.132 0.03 -0.24 0.268 -0.2 -0.588 -0.374 -0.515 -0.814 -0.469

BI867407 1.485 2.125 1.904 1.13 0.111 0.305 0.019 -0.021 -0.328 -0.584 -0.572 -0.713

BI867449 1.058 0.427 -0.421 -0.167 -0.332 -0.115 -0.233 -0.728 -0.15 -1.368 -0.275 -0.558

BI867483 0.802 1.196 0.731 0.589 -0.863 -0.264 0.247 -0.807 -0.372 -0.36 -1.038 -1.039

BI867489 -0.16 -0.564 -0.125 0.387 0.646 0.258 0.174 0.87 0.14 0.63 0.463 -0.099

BI867531 0.341 0.565 0.41 0.94 0.951 0.204 0.402 0.781 0.256 0.512 0.358 0.259

BI867642 1.397 0.773 0.699 0.394 0.289 0.494 0.209 0.967 0.213 0.256 -0.045 -0.055

BI867717 0.822 0.522 0.389 0.738 1.247 1.36 0.987 1.463 0.307 0.276 0.334 0.046

BI867819 -0.281 -0.249 1.511 1.684 1.521 1.821 1.231 1.866 0.62 0.598 0.141 0.291

BI867929 0.542 0.533 0.161 0.643 0.646 -0.096 0.283 0.364 -0.045 -0.081 -0.071 -0.08

BI867946 0.179 -0.387 0.061 0.059 0.296 -0.118 0.092 1.01 0.206 0.468 0.4 -0.2

BI868116 -1.267 -3.251 -0.071 1.089 3.043 2.44 2.587 1.719 1.335 -0.465 0.072 0.078

BI875704 -0.124 0.598 0.496 0.893 0.865 0.517 0.335 0.647 -0.175 0.01 -0.064 -0.166

BI876163 -2.743 -1.634 0.339 0.339 0.379 0.164 0.043 -0.046 0.84 0.796 0.493 0.366

BI876181 1.701 1.078 3.005 2.817 2.091 2.166 1.413 1.558 0.437 0.058 -0.408 -0.907

BI876201 -0.562 0.025 0.774 0.737 0.7 0.026 0.41 0.216 0.72 -0.006 -0.442 -1.233

BI876262 -0.662 -3.149 -3.22 -3.553 -4.23 -4.031 -4.485 -5.109 -5.114 -7.37 -7.359 -8.24

BI876503 -0.316 0.557 1.475 1.189 1.421 0.106 -0.032 0.812 0.375 0.379 -0.014 -0.209

BI876589 -0.502 0.18 0.686 0.427 0.503 0.261 0.254 0.029 0.217 -0.004 -0.393 -0.958

BI876694 0.454 1.229 0.492 0.363 -0.882 -0.775 -1.486 -1.907 -1.89 -1.892 -2.598 -3.032

BI876830 0.795 0.666 0.735 0.684 -0.407 -0.326 -0.802 -1.161 -0.855 -1.196 -1.478 -1.345

BI877132 0.959 -3.513 -3.198 -3.268 -4.126 -3.181 -4.511 -5.157 -4.499 -6.549 -5.749 -6.206

BI877263 0.119 0.345 0.164 0.219 0.266 0.125 0.448 0.684 0.17 0.009 -0.103 -0.219

BI877285 0.966 0.493 0.022 -0.034 -0.152 -0.016 -0.213 -0.145 -0.229 -0.377 -0.335 -0.56

BI877328 1.4 1.403 1.243 1.146 0.507 -0.001 0.304 0.614 0.024 0.699 0.173 -0.589

BI877478 0.987 -0.154 -0.161 -0.421 -0.789 -0.562 -0.564 -0.91 -0.67 -0.733 -1.029 -0.966

BI877500 1.468 1.803 -0.483 -0.277 -0.508 -0.201 -0.393 -0.203 -0.699 -0.828 -0.63 -1.058

BI877523 1.02 0.786 0.839 0.448 0.347 0.521 -0.083 0.038 0.358 -0.003 0.119 0.062

BI877594 -0.296 -0.075 1.134 0.982 1.267 0.518 0.349 0.675 0.615 0.541 0.477 0.414

BI877608 0.977 0.017 0.058 -0.038 -0.168 0.102 0.19 -0.224 -0.33 0.087 -0.488 0.163

BI877620 1.054 1.548 0.789 0.166 -0.004 0.482 0.274 0.524 0.207 0.195 -0.253 -0.141

BI877622 0.153 -0.125 0.64 0.42 0.789 0.275 0.272 0.846 0.019 0.412 0.136 0.265

BI877634 0.485 1.149 1.15 1.01 0.049 0.03 -0.356 -0.122 -0.182 -0.839 -0.416 -0.408

BI877644 0.315 1.161 0.948 0.75 0.51 -0.24 -0.245 -0.056 -0.274 -0.884 -0.52 -0.609

BI877645 1.224 1.214 -0.086 -0.352 -0.771 -0.209 -0.649 -1.154 -0.782 -0.395 -0.935 -0.632

BI877686 0.543 0.566 0.951 0.539 0.427 0.373 0.095 0.113 0.269 -0.071 0.014 -0.117

BI877691 1.088 0.736 -0.25 -0.294 -0.532 -0.047 -0.265 -0.075 0.348 0.616 0.458 0.077

BI877718 0.434 -1.025 -0.108 -0.218 0.383 1.094 0.271 0.619 0.491 0.435 0.581 0.49

BI877725 1.249 0.651 -0.789 -1.121 -0.229 -0.323 -0.449 -0.494 -0.585 -0.353 -0.316 0.032

BI877730 -0.589 0.447 1.153 1.37 1.943 0.872 0.52 0.739 0.477 0.344 0.381 0.025

BI877740 0.669 -0.11 -0.387 -0.156 -0.955 -0.327 -0.236 -0.097 0.079 0.92 0.146 -0.792

BI877751 0.64 1.163 0.751 0.765 -0.375 -0.102 -0.315 0.05 0.055 0.594 0.16 -0.481

BI877821 -0.747 -0.558 0.37 0.813 1.342 1.011 0.867 1.189 0.414 0.694 0.048 0.135

BI877866 0.366 0.664 1.018 1.156 1.034 0.352 0.335 0.594 0.302 0.265 -0.122 0.041

BI877872 0.082 0.756 2.55 2.114 2.863 2.766 1.911 1.865 0.263 0.296 -0.062 0.131

BI877878 1.289 -1.86 -1.365 -1.085 -1.594 -1.061 -0.794 -2.322 -0.952 -1.433 -2.008 -2.033

BI877887 1.191 0.367 0.154 0.5 0.286 0.514 -0.054 0.46 0.051 0.11 0.125 0.05

BI877898 1.337 1.099 -0.564 -0.701 -0.735 -0.164 -0.733 -0.769 -0.69 -1.268 -0.952 -0.867

BI877917 0.211 0.446 0.36 0.563 1.09 0.332 0.28 0.396 0.063 -0.022 -0.497 -0.595

BI877921 0.281 -0.439 0.729 0.519 0.675 1.277 1.193 0.899 0.417 0.414 -0.02 0.047

BI877938 0.425 0.528 0.486 0.398 0.229 0.773 0.803 0.843 0.427 0.407 0.028 0.035

BI877974 0.446 1.149 0.908 0.372 -0.457 -0.365 0.017 -0.28 -0.439 -0.581 -0.877 -0.623

BI877985 0.758 1.496 1.147 1.963 1.511 0.629 0.279 0.806 -0.129 -0.47 -0.379 -0.399

BI877998 0.245 -0.314 -0.33 0.244 -0.039 0.256 0.258 0.645 0.31 0.766 0.316 0.381

BI877999 1.023 1.094 0.349 0.703 0.366 0.001 0.097 -0.29 -0.3 -0.249 -0.722 -0.776

BI878013 0.457 1.604 1.021 0.812 0.795 0.325 0.266 0.587 0.517 -0.256 0.311 0.045

BI878018 1.485 0.662 -1.518 -0.268 -1.178 -0.453 -0.698 -1.072 -0.831 -1.354 -1.006 -1.608

BI878039 0.97 0.931 1.915 1.486 1.435 1.6 0.525 0.667 0.446 0.372 0.176 0.17

BI878043 -0.369 -0.754 0.427 0.8 0.621 0.772 0.387 0.374 0.361 0.452 0.319 0.258

BI878059 -0.728 0.579 0.735 1.232 0.758 -0.045 0.161 0.699 0.149 -0.099 0.369 -0.178

BI878078 -0.082 0.041 0.61 1.364 1.461 2.308 1.46 1.825 1.186 1.38 0.699 0.535

BI878085 -0.526 0.415 0.407 0.288 0.202 -0.151 0.43 1.141 0.264 -0.131 0.569 0.335

BI878102 0.5 0.884 1.149 1.288 1.07 1.511 0.962 1.088 0.688 0.711 0.114 -0.147

BI878117 -0.586 -1.903 -0.987 -1.094 -1.077 -0.233 -0.14 0.299 -0.074 0.713 0.413 0.474

BI878123 1.271 0.863 0.149 -0.003 -1.054 -0.352 -0.319 -0.488 -0.325 -0.246 -0.706 -0.533

BI878184 1.909 0.576 -0.756 -1.595 -2.638 -0.905 -1.678 -2.749 -1.701 -1.636 -2.416 -2.149

BI878195 2.144 -2.395 -3.302 -2.216 -3.259 -3.066 -4.083 -4.539 -4.078 -5.2 -5.376 -5.064

BI878269 1.497 0.389 0.075 0.354 0.134 -0.018 -0.312 0.171 -0.067 -0.72 -0.417 -0.717

BI878279 0.3 0.394 0.667 0.684 1.069 0.373 0.383 1.175 0.197 0.486 0.1 -0.11

BI878280 1.446 1.704 0.4 -0.341 -2.037 -0.408 -1.154 -1.416 -0.872 -1.841 -2.539 -1.641

BI878304 -0.728 0.321 0.001 -0.122 -0.107 -0.135 -0.243 0.193 -0.336 0.27 0.107 0.707

BI878322 -0.023 0.069 0.994 1.21 1.378 0.954 0.976 1.657 0.455 0.788 0.478 0.377

BI878403 0.068 0.411 1.062 0.902 0.763 0.626 0.399 0.418 0.249 0.454 0.514 0.635

BI878416 1.005 0.662 -0.301 -0.234 -1.042 0.019 -0.472 -0.827 -0.415 -0.506 -0.617 -0.717

BI878456 -0.193 -0.718 -0.733 -0.473 -0.744 -0.133 0.192 0.166 0.158 0.964 0.56 0.556

BI878459 1.253 0.58 -0.488 -0.582 -1.302 -0.363 -1.204 -1.011 -1.376 -1.319 -1.473 -0.963

BI878464 -0.075 0.635 0.266 0.656 0.628 -0.246 0.374 1.176 0.277 -0.514 -0.365 -0.245

BI878475 0.2 0.725 1.388 1.372 0.973 0.497 0.327 0.758 0.358 0.094 0.006 0.421

BI878477 0.022 -0.058 0.234 0.272 0.519 0.699 0.542 0.952 0.331 0.742 0.306 0.314

BI878480 1.737 0.964 -1.527 -1.73 -2.973 -0.797 -1.838 -3.785 -2.046 -3.77 -3.31 -2.247

BI878520 0.1 0.287 0.144 0.035 0.19 0.463 0.361 1.139 0.462 0.548 0.454 0.218

BI878536 0.402 0.452 0.466 0.59 0.975 0.426 0.221 0.509 0.167 -0.242 -0.229 -0.183

BI878578 0.208 0.671 1.271 0.793 0.675 1.223 0.673 0.731 0.79 0.446 0.072 -0.51

BI878583 -0.481 0.053 0.104 0.547 0.369 -0.154 0.139 0.851 0.031 -0.047 0.409 0.215

BI878609 1.478 1.189 -0.064 -0.433 -1.371 -0.445 -1.174 -1.83 -0.873 -1.011 -1.253 -0.952

BI878611 1.154 1.838 1.662 1.131 0.078 0.278 -0.547 -0.208 -0.615 -0.91 -1.139 -1.231

BI878642 1.092 0.82 -0.491 -0.327 -0.84 0.253 -0.324 0.11 -0.177 0.073 -0.042 0.03

BI878714 -0.337 0.867 0.639 0.643 0.181 0.053 -0.08 -0.364 -0.565 -0.784 -0.478 -0.756

BI878720 -0.785 -1.941 -0.526 -0.582 -0.483 0.006 -0.061 -0.322 0.189 0.449 0.251 0.059

BI878741 0.682 0.791 1.219 0.85 0.856 0.365 0.033 0.414 -0.141 -0.295 -0.466 -0.336

BI878743 0.242 0.682 0.711 0.989 0.636 0.184 0.506 0.77 0.238 -0.29 -0.141 -0.012

BI878817 0.983 0.986 -0.182 -0.274 -0.404 -0.137 -0.015 -0.44 -0.256 -0.464 -0.775 -0.483

BI878819 0.824 0.696 0.724 0.957 0.015 0.426 0.027 0.157 -0.369 -0.176 -0.423 -0.262

BI878820 0.534 0.874 1.336 1.253 1.384 1.809 0.923 1.11 0.333 0.481 0.045 0.521

BI878842 1.92 -1.273 -1.207 -1.088 -0.849 -0.355 -0.419 -0.988 -0.933 -1.854 -1.01 -0.915

BI878849 -0.106 1.278 1.121 0.985 0.12 -0.319 -0.075 -0.419 0.123 -0.474 -0.41 -0.413

BI878854 -0.1 0.766 1.531 1.757 1.699 1.258 0.966 1.081 0.572 0.723 0.392 -0.175

BI878876 0.16 0.81 0.722 0.818 0.596 0.29 0.338 0.715 0.328 0.182 -0.077 -0.057

BI878903 0.185 0.704 0.537 1.096 0.767 0.332 0.123 0.04 -0.197 -0.152 -0.45 -0.526

BI878904 0.698 0.553 0.861 0.661 0.627 1.082 0.368 0.459 0.357 0.222 0.231 0.306

BI878907 -0.15 0.253 0.353 0.5 0.598 -0.046 -0.136 0.049 0.035 -0.18 -0.089 -0.135

BI878923 0.3 0.254 1.012 0.878 1.016 0.996 0.472 1.171 0.444 0.739 0.144 0.102

BI878928 0.829 1.19 0.789 0.632 -0.151 -0.078 -0.758 -1.287 -1.112 -0.756 -1.003 -1.079

BI878929 0.402 0.814 0.957 1.401 1.457 1.156 1.063 1.216 0.202 0.43 0.285 0.244

BI878952 -0.015 0.674 0.466 1.036 1.152 0.949 0.259 0.428 -0.048 0.084 0.107 -0.262

BI878961 0.401 0.722 0.766 1.204 1.039 0.418 0.324 0.807 0.396 -0.111 -0.337 -0.117

BI878962 0.344 0.698 0.808 0.805 0.909 0.359 0.282 0.562 0.02 -0.034 -0.201 -0.128

BI878967 0.797 0.543 0.289 0.531 0.754 0.724 0.668 1.211 0.129 0.124 0.402 0.027

BI878979 0.9 0.04 0.851 0.496 0.951 0.962 0.767 1.062 0.34 0.417 -0.4 -0.658

BI879035 -0.256 -0.929 -0.814 -1.53 -1.314 -0.235 -0.583 -0.26 -0.465 0.596 0.47 -0.035

BI879038 -0.813 -0.971 -0.863 -0.06 0.584 1.263 0.756 1.253 0.583 1.237 0.858 0.725

BI879231 1.259 0.694 -0.082 0.337 0.88 1.045 1.014 1.425 0.535 0.027 -0.03 -0.558

BI879262 -0.437 -0.354 0.563 0.638 0.566 1.416 0.814 0.716 0.233 0.507 0.405 0.487

BI879432 0.94 1.574 0.178 0.341 0.129 0.209 0.506 0.986 0.693 0.742 0.514 0.205

BI879444 1.036 0.868 2.755 3.127 2.955 1.676 2.028 1.635 0.746 1.122 0.848 0.509

BI879454 -0.515 0.545 0.751 1.136 1.98 1.181 0.722 2.033 0.617 0.686 0.073 0.408

BI879489 0.118 -0.063 -0.06 0.04 0.258 0.617 0.239 0.842 0.35 0.73 0.333 0.425

BI879523 0.861 0.475 0.677 0.997 0.076 -0.157 0.222 0.134 0.091 0.461 -0.132 -0.18

BI879533 -0.541 -0.068 0.729 -0.562 0.829 0.627 0.044 1.092 0.327 0.182 0.366 1.044

BI879550 -0.275 0.606 0.918 1.501 1.805 0.612 0.879 1.233 -0.004 0.396 0.037 -0.037

BI879566 0.982 0.453 0.48 -0.024 0.146 0.767 0.267 0.22 0.343 0.044 -0.07 -0.064

BI879576 1.33 -0.051 -0.634 -0.676 -0.801 -0.39 -0.562 -1.157 -0.943 -0.91 -1.701 -0.939

BI879589 -0.925 -0.847 -1.692 -1.046 -1.503 -0.277 -0.797 -0.765 0.367 -0.177 0.659 1.18

BI879615 0.708 -2.781 -2.476 -2.08 -3.584 -2.575 -2.187 -3.87 -2.607 -3.835 -4.309 -4.2

BI879616 0.967 -0.501 -0.03 0.569 -0.285 0.397 -0.073 0.102 -0.396 0.172 -0.017 0.063

BI879661 -0.879 -3.098 -1.445 -1.712 -3.558 -0.932 -0.982 -2.772 -0.837 0.568 0.61 1.063

BI879686 1.409 0.874 0.826 1.221 0.912 1.52 0.617 0.815 -0.009 0.457 -0.157 -0.264

BI879735 0.526 0.463 0.792 0.759 1.127 1.658 0.705 1.407 0.481 0.899 0.308 0.166

BI879868 0.52 -0.239 0.246 0.186 -0.077 0.27 0.467 -0.127 0.363 0.193 0.479 0.746

BI879889 0.705 0.291 0.82 0.328 0.393 0.187 0.491 1.122 0.447 0.905 0.363 0.28

BI879928 0.615 0.789 0.906 1.547 1.447 0.64 0.681 0.998 -0.275 -0.249 -0.383 -0.363

BI879932 -0.023 0.003 0.152 -0.029 0.11 -0.317 0.619 2.253 0.598 0.552 0.177 -0.129

BI879969 1.143 0.9 0.269 0.012 -0.233 -0.249 -0.249 -0.053 -0.129 -0.153 -0.221 -0.396

BI880002 -1.299 -1.334 -1.435 -1.556 -1.263 -0.527 -0.236 0.019 0.093 0.483 0.676 0.476

BI880006 0.132 -0.488 -0.067 0.19 0.263 0.493 0.309 0.357 0.184 0.578 0.305 0.293

BI880007 -2.635 -2.384 -0.794 -0.805 -0.067 -0.291 -0.774 -1.134 0.475 -0.469 0.472 0.251

BI880060 -0.347 -0.03 1.283 1.413 1.261 1.186 0.2 0.803 0.172 0.399 0.175 0.138

BI880061 0.262 0.555 1.904 1.459 2.139 2.208 1.57 2.18 1.066 1.246 0.71 0.75

BI880074 -2.049 -1.205 -1.944 -0.5 -1.529 -0.49 -1.399 -1.693 -1.043 0.456 0.495 1.076

BI880092 -0.443 0.051 0.572 0.84 1.219 0.056 0.147 0.62 0.612 0.133 0.566 0.615

BI880125 0.321 -0.05 0.512 0.455 0.866 1.326 0.593 0.526 0.448 0.692 0.312 0.338

BI880136 -0.5 -1.189 -0.529 -0.753 -0.095 -0.173 -0.258 -0.479 0.155 -0.247 0.406 0.312

BI880170 -0.349 -0.054 -0.231 0.03 -0.252 -0.102 0.029 -0.421 -0.213 -0.25 0.109 0.823

BI880201 -0.353 -0.24 0.21 0.463 0.042 0.184 -0.097 -0.192 -0.066 -0.453 -0.015 0.968

BI880302 -0.238 0.096 0.725 1.278 1.214 0.003 0.119 0.406 0.273 0.174 -0.395 -0.596

BI880304 -0.323 0.093 -0.191 0.341 0.123 -0.264 -0.1 0.91 -0.118 0.113 -0.039 -0.355

BI880342 -0.179 0.19 0.185 0.228 0.162 0.147 0.309 -0.158 0.164 -0.419 0.086 1.778

BI880357 0.822 -0.027 -0.364 0.062 0.405 0.398 0.107 -1.164 -0.805 -0.896 -0.84 -0.831

BI880392 -1.024 -1.346 -0.976 -0.708 -0.872 0.022 0.498 0.34 0.667 0.97 0.838 0.348

BI880444 0.459 -0.006 0.499 0.756 0.799 0.305 0.546 0.177 0.024 -0.128 -0.197 -0.035

BI880518 0.96 1.131 -0.688 -0.996 -0.494 -0.196 -0.444 -0.678 -0.573 -0.66 -0.731 -0.544

BI880524 -3.095 -1.639 1.033 1.548 1.109 0.924 0.341 0.306 -0.034 0.784 0.765 0.84

BI880563 1.175 0.947 0.267 0.183 0.682 0.976 0.32 0.682 0.25 0.393 0.181 0.265

BI880658 0.864 -0.897 -0.45 -0.358 -0.455 0.244 -0.327 0.004 0.067 0.412 0.153 0.17

BI880694 -0.27 -0.141 0.065 -0.013 -0.093 -0.083 0.098 0.623 -0.017 -0.078 0.239 0.178

BI880767 0.995 1.284 0.645 0.452 0.091 0.401 0.137 0.465 -0.037 0.346 0.381 0.368

BI880780 0.921 0.921 0.089 0.079 -0.166 0.356 0.317 -0.129 -0.11 0.008 -0.316 -0.283

BI880833 0.039 0.038 1.447 1.085 1.579 0.222 0.145 0.506 0.072 -0.035 0.368 0.422

BI881430 -0.305 -1.265 -0.28 -0.387 -0.357 0.654 0.221 0.364 0.285 1.066 0.611 0.208

BI881679 -0.39 -0.395 1.395 1.655 1.644 2.129 1.82 1.645 1.028 0.607 0.174 0.125

BI882056 0.126 1.122 1.111 1.06 1.326 0.623 0.503 0.688 0.378 0.175 0.076 -0.48

BI882169 0.11 -0.045 0.026 0.393 0.693 0.66 0.272 1.09 0.126 0.275 -0.025 -0.012

BI882313 -0.917 -1.832 -0.762 -1.084 -2.069 -0.466 -0.489 -1.611 -0.627 0.776 0.757 0.428

BI882464 0.828 0.735 0.208 0.082 -0.414 0.286 -0.135 -0.148 -0.261 -0.234 -0.329 -0.366

BI882529 -0.27 0.573 1.164 0.863 0.944 0.107 0.255 0.188 0.202 0.065 0.201 0.136

BI882568 -0.029 0.307 0.641 0.672 0.793 1.09 0.373 0.719 0.303 0.151 -0.184 -0.406

BI882649 -0.302 -0.942 0.173 -1.098 -0.869 -0.356 0.048 -1.126 -0.669 -0.114 0.618 1.476

BI882727 1.069 -0.892 -0.251 0.019 0.249 1.391 0.829 1.396 1.114 1.204 0.613 0.004

BI882791 -2.34 -3.06 -0.442 -0.742 -0.084 -0.461 -0.358 -0.776 0.336 -0.218 0.432 0.079

BI882972 -1.1 -2.183 -1.072 -0.797 -0.575 0.589 0.221 0.361 0.406 1.287 1.086 0.645

BI883018 -0.337 -0.472 0.065 -0.359 0.905 0.68 0.321 0.851 0.161 0.512 0.402 -0.028

BI883215 0.839 1.63 0.609 0.559 0.412 0.144 -0.06 0.121 -0.071 -0.736 -0.564 -0.665

BI883229 0.041 0.819 1.021 0.497 -0.079 -0.103 -0.219 0.012 0.236 -0.28 0.297 0.1

BI883230 -0.817 0.011 -0.315 -0.783 -0.326 -0.12 -0.079 0.128 -0.102 0.368 0.704 -0.102

BI883231 -0.227 0.812 0.461 0.657 1.12 0.465 0.493 1.022 0.075 0.067 0.061 -0.263

BI883233 -0.728 0.203 0.467 0.841 1.59 0.116 0.378 0.717 -0.414 -0.453 -0.178 -0.287

BI883242 0.488 0.994 0.915 0.85 0.673 1.098 0.285 0.758 0.185 0.22 0.175 0.342

BI883251 0.101 -0.434 0.163 0.344 1.281 1.334 0.744 0.944 0.563 0.687 0.49 0.421

BI883252 0.018 -0.384 0.256 1.169 2.185 2.142 1.616 2.184 0.891 0.477 0.538 -0.261

BI883421 -0.406 1.008 2.374 2.075 1.027 0.457 0.204 0.221 0.151 0.05 0.053 -0.336

BI883638 -0.728 0.378 0.775 1.612 1.456 0.707 0.503 1.887 -0.378 -0.449 -0.543 -0.913

BI883674 -0.248 -0.459 0.97 1.757 1.453 1.901 1.769 1.394 0.92 0.538 0.154 0.076

BI883698 -0.382 -0.403 0.133 0.325 0.695 0.815 0.401 0.892 0.622 0.853 -0.11 0.124

BI883903 0.092 -0.412 0.496 0.613 0.845 0.185 -0.085 -0.255 -0.084 0.036 -0.061 0.38

BI883910 0.607 0.336 -0.259 -0.077 0.421 0.596 0.387 0.75 0.407 0.354 0.28 -0.016

BI883928 0.262 1.22 4.936 2.901 2.733 0.544 0.439 0.549 0.36 -0.082 0.242 -0.103

BI883935 0.336 0.332 1.274 1.116 1.622 1.538 1.239 0.803 0.795 0.759 0.529 0.481

BI883953 0.114 0.064 0.262 0.655 1.233 2.162 1.285 2.023 1.472 1.516 0.964 0.473

BI884186 0.749 -0.589 -0.155 -0.244 0.011 0.492 0.077 0.901 -0.09 0.164 0.316 -0.217

BI884413 -0.552 0.689 0.753 0.098 -0.059 -0.11 0.064 -0.03 -0.128 -0.055 -0.045 0.15

BI884752 -0.201 0.837 1.809 1.89 1.095 -0.149 0.152 0.159 0.067 0.326 -0.21 -0.341

BI884788 2.06 0.251 -0.329 -0.087 -0.6 0.014 -0.264 -0.154 -0.311 -0.427 -0.324 -0.587

BI884839 -0.697 0.391 0.826 1.374 1.683 1.598 0.711 0.905 0.607 0.594 0.281 0.221

BI885026 -0.948 -0.04 -0.415 -0.659 -0.003 0.263 0.433 1.042 0.447 1.046 -0.079 -0.682

BI885228 0.984 1.037 -1.269 -1.23 -1.536 -0.47 -1.038 -1.459 -1.155 -0.802 -0.793 -0.22

BI885240 -0.594 -0.621 -0.103 0.693 0.916 0.956 0.923 1.376 0.338 0.622 0.247 -0.002

BI885253 0.145 -0.137 0.177 0.218 0.499 0.154 0.289 0.887 0.481 0.391 0.375 0.019

BI885257 0.317 1.114 0.837 0.746 1.016 1.001 0.52 0.908 0.143 0.009 -0.066 -0.598

BI885399 1.125 0.377 -0.079 -0.171 -0.432 0.069 -0.207 -0.069 -0.06 0.069 -0.269 -0.237

BI885469 1.024 0.804 -0.305 -0.021 -0.985 -0.608 -0.042 -0.935 0.109 -0.371 -0.663 -0.612

BI885492 -1.278 -0.993 -0.203 0.33 0.324 1.172 0.583 0.626 0.414 0.852 0.67 0.465

BI885503 2.031 -0.301 -1.706 -0.795 -1.945 -0.385 -0.189 -1.856 -0.714 -1.651 -1.519 -1.094

BI885564 1.303 0.082 -0.11 -0.28 -0.393 -0.28 -0.594 -0.534 -0.458 -1.129 -0.2 -0.486

BI885653 -3.548 -1.131 3.457 3.722 2.8 1.754 1.059 1.023 0.56 0.958 0.877 1.316

BI885759 0.323 1.415 -0.552 -0.584 -0.899 -0.057 0.104 1.002 0.483 0.523 0.053 -0.811

BI885768 0.066 -0.015 1.031 0.788 0.932 1.66 0.815 0.939 0.3 0.218 0.184 0.096

BI885777 -0.784 -0.029 0.569 0.662 0.821 1.282 0.758 0.736 0.451 0.552 0.249 0.475

BI885798 -0.064 0.696 0.89 1.303 1.831 0.559 0.346 1.304 0.395 0.157 0.272 -0.182

BI885813 -0.035 -0.048 0.906 1.149 1.29 0.617 0.358 0.491 0.33 -0.055 0.123 -0.013

BI885824 0.109 -0.025 0.496 0.772 0.62 0.893 0.551 0.797 0.218 0.394 0.223 -0.137

BI885851 1.397 0.958 -0.445 0.062 -0.132 0.217 0.372 -0.302 -0.176 -0.247 -0.208 -0.199

BI885886 -0.008 -0.184 0.859 1.769 1.423 0.701 1.15 0.785 0.503 0.353 0.162 0.115

BI885889 -0.728 0.21 1.711 1.772 1.218 0.307 -0.219 0.34 -0.255 -0.143 0.55 -0.112

BI885890 -0.815 0.844 0.274 0.54 0.669 0.651 0.212 0.819 -0.07 0.194 0.325 0.406

BI885896 0.967 -0.681 -0.805 -0.356 -0.591 0.084 -0.653 -0.783 -0.827 -0.99 -1.213 -0.538

BI885898 -0.052 0.316 0.661 0.626 1.241 0.28 0.326 0.7 0.224 0.14 -0.209 -0.18

BI885907 0.416 0.36 0.02 -0.174 0.313 0.857 0.281 0.893 0.136 0.273 -0.049 -0.2

BI885914 -0.16 0.843 0.778 0.367 -0.141 -0.027 0.26 0.633 0.149 0.777 0.301 -0.085

BI885924 0.742 -0.077 0.319 0.41 0.544 1.176 0.799 1.157 0.458 0.409 0.171 -0.184

BI885932 1.19 0.812 1.26 0.612 -0.24 -0.411 -0.307 -1.046 -0.455 -0.863 -1.182 -1.145

BI885944 -0.85 0.45 1.191 1.649 2.567 1.012 0.739 1.695 1.016 -0.504 -0.684 -0.65

BI885968 0.785 -0.222 -0.224 -0.131 -0.018 0.596 0.634 0.926 0.656 0.791 0.447 -0.173

BI885973 -0.077 1.298 1.376 1.529 1.834 1.341 0.743 1.56 0.503 0.187 0.126 -0.082

BI885983 -0.191 0.308 0.552 0.516 0.512 -0.218 0.059 0.49 0.466 -0.079 0.383 -0.2

BI885984 0.925 1.511 2.227 1.52 1.497 1.244 0.75 1.031 0.33 0.22 -0.048 -0.43

BI885991 0.748 0.839 0.752 1.797 1.704 0.996 0.776 0.998 0.124 0.141 -0.125 -0.179

BI885993 0.458 0.852 1.015 1.554 1.256 -0.038 0.449 1.062 0.038 0.385 0.235 -0.062

BI885994 0.18 0.544 0.592 0.542 0.726 0.318 0.095 0.315 0.108 0.11 -0.095 -0.174

BI886006 -0.164 -0.351 1.008 1.247 1.213 -0.029 0.205 0.741 0.111 0.221 0.325 -0.037

BI886016 -0.365 0.292 0.635 1.121 0.791 0.647 0.738 0.923 0.476 0.376 0.054 0.002

BI886020 0.569 1.643 1.901 1.967 1.581 1.595 1.053 0.74 0.327 0.175 0.308 0.078

BI886025 -2.881 -1.279 -0.295 -0.28 0.686 1.605 1.052 1.385 0.65 1.423 1.05 0.933

BI886029 -1.442 -1.297 -0.919 -0.611 -0.257 0.461 0.146 0.532 0.394 1.176 0.762 0.686

BI886069 -0.589 -0.668 -1.333 -0.536 -0.784 -0.517 0.308 -0.237 0.129 0.271 0.069 -0.366

BI886089 -0.177 0.793 1.158 0.865 0.539 -0.064 0.052 -0.192 -0.007 0.048 -0.642 -0.33

BI886095 -1.066 -0.774 0.889 0.964 1.302 2.394 1.851 1.67 1.44 1.299 1.194 1.203

BI886106 -0.253 0.481 2.597 0.911 1.567 0.313 0.327 0.961 0.616 0.517 0.525 -0.014

BI886119 1.333 1.009 -0.132 0.026 -0.106 0.203 -0.207 -0.459 -0.399 -0.717 -0.865 -0.788

BI886127 0.255 -0.085 0.734 1.066 1.133 0.715 0.573 1.338 0.404 0.211 -0.053 -0.321

BI886133 -0.728 0.545 0.407 1.341 1.131 0.022 -0.089 0.193 -0.328 -0.749 -0.129 -0.358

BI886160 0.253 -0.349 0.761 1.303 1.205 2.184 1.784 3.178 1.374 0.71 -0.338 -0.093

BI886163 0.361 -0.104 -0.048 -0.308 0.044 0.455 0.325 0.939 0.197 0.443 -0.016 -0.08

BI886167 0.108 -2.879 -1.573 -1.301 -1.091 0.126 -0.17 0.157 0.12 0.956 0.595 0.143

BI886184 0.353 0.196 0.826 1.147 1.372 1.249 0.804 0.868 0.672 0.228 -0.035 -0.316

BI886187 -0.127 0.156 0.777 0.348 0.603 0.188 0.232 0.835 0.332 0.304 0.002 -0.029

BI886200 0.606 0.698 1.303 1.248 1.493 1.745 1.215 1.865 0.96 0.653 0.367 -0.164

BI886229 0.375 -0.317 -0.203 -0.213 0.042 0.496 0.352 0.69 0.702 0.435 0.591 0.527

BI886248 -0.283 -0.412 0.353 0.181 0.364 0.826 0.359 0.442 0.298 0.611 0.342 0.234

BI886249 0.853 1.247 1 1.227 0.995 0.901 0.742 0.975 -0.098 0.114 -0.054 -0.274

BI886251 -0.942 0.35 1.821 1.613 1.83 1.545 0.726 2.09 1.646 0.922 0.061 -0.45

BI886259 -0.472 -0.698 0.159 0.519 0.573 -0.188 0.259 0.554 0.059 0.363 0.775 0.847

BI886268 0.298 1.464 0.925 0.817 0.375 -0.342 0.011 -0.139 -0.304 0.185 -0.156 -0.667

BI886271 -0.491 1.412 4.23 3.43 3.297 1.383 0.952 1.988 0.327 -0.141 0.873 -0.03

BI886272 -0.195 0.334 1.563 2.079 2.216 1.158 1.427 1.986 0.671 0.598 0.336 0.148

BI886276 1.328 1.234 1.582 1.32 1.54 1.056 0.602 1.624 0.492 0.483 0.083 -0.102

BI886290 0.084 -0.391 -0.381 0.344 0.325 0.611 0.379 0.925 0.429 1.142 0.638 0.25

BI886303 0.009 0.631 2.1 1.063 1.253 0.871 0.588 0.796 0.299 0.312 -0.202 0.271

BI886316 -0.135 -0.195 2.099 1.21 1.556 1.077 1.115 0.818 0.435 0.921 1.179 0.837

BI886329 -0.325 0.242 1.073 1.125 1.314 1.193 0.745 1.851 0.857 1.47 0.753 0.31

BI886353 -0.033 1.17 0.83 2.112 1.837 0.88 0.609 1.248 0.004 0.094 -0.214 -0.289

BI886373 -0.546 0.568 0.741 0.912 1.168 0.261 0.354 0.906 0.84 0.65 0.218 -0.218

BI886374 0.256 0.559 1.199 0.986 1.248 1.173 0.978 0.967 0.623 0.448 0.072 -0.367

BI886376 1.216 0.636 1.462 1.908 1.428 0.963 0.373 0.512 0.126 0.223 -0.225 -0.918

BI886387 0.233 0.051 0.434 0.114 0.265 0.056 0.027 0.89 0.439 0.296 0.077 -0.426

BI886388 -1.219 -0.599 -0.253 0.177 0.745 1.142 1.019 1.107 0.36 1.232 0.98 1.37

BI886392 -0.413 0.217 0.457 0.627 0.378 0.555 0.448 1.431 1.164 1.438 0.746 0.246

BI886431 -0.691 -0.474 1.99 3.04 4.379 3.376 2.483 3.455 2.321 -0.149 0.078 -0.148

BI886435 -0.26 0.005 -0.052 0.54 0.788 0.514 0.205 0.51 -0.026 0.477 0.36 1.364

BI886461 0.232 0.019 0.634 0.432 1.182 0.974 0.677 1.114 0.361 0.605 0.141 0.306

BI886468 0.184 1.203 0.593 0.573 0.232 0.027 -0.049 0.223 -0.207 -0.08 -0.118 -0.692

BI886470 -0.728 -0.501 0.201 0.707 1.821 0.121 0.208 0.672 0.272 -0.045 -0.025 -0.278

BI886473 0.026 1.105 0.665 0.647 -0.01 -0.29 -0.032 0.571 0.089 0.361 0.238 -0.213

BI886477 0.255 0.374 0.554 1.213 0.652 -0.071 0.353 0.833 0.133 0.267 0.194 0.087

BI886511 0.476 0.444 0.739 0.547 0.473 0.574 -0.198 0.138 -0.266 -0.063 -0.418 -0.05

BI886513 0.89 0.971 1.078 0.808 0.842 0.735 0.247 0.614 -0.311 0.197 -0.343 0.067

BI886522 -0.132 0.632 0.835 0.948 0.892 -0.14 0.048 0.332 0.006 0.041 -0.199 -0.183

BI886549 -0.524 -0.475 -0.366 0.237 0.645 0.918 0.877 1.172 0.287 0.782 0.176 0.13

BI886552 -0.289 0.728 0.666 0.52 0.693 0.072 0.261 0.546 0.122 -0.072 -0.118 -0.293

BI886564 -0.411 -0.067 0.52 0.568 0.631 -0.11 0.306 0.395 0.259 0.09 -0.117 -0.405

BI886597 -0.023 0.552 0.737 0.601 0.473 0.301 0.449 0.334 0.234 0.058 -0.435 -0.453

BI886648 -0.401 -0.331 0.122 0.294 0.713 -0.234 0.319 0.946 0.28 0.825 1.742 0.166

BI886653 -0.245 0.381 1.379 1.607 2.417 1.66 1.173 1.97 0.486 1.143 0.718 0.622

BI886664 0.833 1 0.686 0.654 -0.214 -0.29 -0.09 -0.672 -0.673 -1.103 -1.022 -1.205

BI886677 -0.5 0.454 1.876 2.443 1.798 0.792 0.009 1.232 0.844 0.558 0.482 0.224

BI886691 -0.079 0.215 0.428 0.943 0.605 0.185 0.293 0.32 0.099 0.086 -0.16 -0.301

BI886699 1.327 1.71 0.138 0.237 -0.291 -0.06 -0.367 -0.827 -0.419 -1.118 -1.372 -1.72

BI886702 -0.107 0.2 0.151 2.293 1.7 0.482 0.074 -0.028 -0.148 -0.805 -1.051 -1.286

BI886711 -0.356 1.031 1.28 0.687 0.413 -0.125 0.111 0.184 -0.015 -0.462 -0.012 0.134

BI886717 1.01 0.515 1.119 0.634 0.676 0.434 0.067 0.116 -0.027 -0.076 -0.458 -0.204

BI886728 -0.14 0.992 0.781 0.796 0.411 0.277 0.011 0.661 0.24 0.286 0.158 -0.001

BI886736 0.753 1.382 0.423 0.145 0.192 0.188 -0.188 -0.291 -0.589 -1.112 -0.844 -0.859

BI886745 0.003 0.053 1.196 1.123 1.289 0.521 0.499 0.742 0.202 0.045 -0.31 -0.376

BI886755 -0.254 -0.202 1.927 1.059 1.618 1.11 0.431 1.159 0.175 0.069 -0.066 -0.056

BI886766 -0.098 0.955 1.181 1.193 1.04 0.896 0.096 0.531 0.359 -0.058 -0.329 -0.505

BI886767 -0.125 -0.486 -0.32 -0.277 -0.25 0.138 -0.091 0.181 0.127 0.648 0.232 0.32

BI886779 -0.012 -0.02 0.709 1.092 0.747 0.35 0.34 0.279 0.009 -0.055 -0.199 -0.026

BI886789 -2.881 -1.165 3.378 3.291 2.527 1.698 0.991 0.476 0.471 0.962 0.918 1.344

BI886791 -0.772 -0.611 3.814 3.471 3.747 2.49 2.088 2.781 1.013 0.485 0.309 0.923

BI886794 -0.224 -0.301 -0.043 0.329 0.084 0.351 -0.012 0.964 0.67 0.821 0.625 0.304

BI886811 -0.502 -0.102 0.448 1.216 1.217 -0.204 0.306 0.264 -0.254 -0.61 0.036 -0.293

BI886819 0.74 1.782 3.097 1.887 1.388 0.604 0.322 0.156 0.086 0.076 0.058 -0.246

BI886825 -0.096 -1.845 -0.856 -0.698 -0.475 0.628 0.142 0.326 0.307 1.037 0.726 0.2

BI886847 -0.112 -0.872 -0.194 0.081 0.248 0.046 0.333 0.473 0.368 0.339 0.526 0.324

BI886872 -0.53 0.192 0.258 0.196 1.105 -0.065 0.565 0.918 0.25 0.115 0.302 0.882

BI886921 1.041 0.636 0.399 0.472 0.501 0.409 0.321 0.213 0.3 0.126 0.044 -0.13

BI886934 -0.01 0.714 1.342 1.359 1.415 1.014 0.758 0.875 0.581 0.559 0.255 0.024

BI886935 0.432 -0.64 -0.077 0.103 0.119 0.882 0.33 0.565 0.288 0.341 0.085 -0.052

BI886936 -1.375 -0.076 2.026 1.845 1.802 1.209 0.3 0.923 1.111 0.66 0.508 0.539

BI887057 -0.33 -0.483 0.191 0.546 1.222 0.337 0.423 0.596 0.3 0.667 0.509 0.109

BI887101 0.42 0.935 0.738 0.545 1.094 0.201 0.032 0.862 -0.054 -0.096 -0.116 -0.37

BI887133 -0.089 0.527 1.49 1.59 1.409 1.602 1.022 0.723 0.755 0.582 0.162 -0.018

BI887138 0.77 0.896 0.734 0.496 0.536 0.227 0.133 -0.329 -0.505 -0.048 -0.258 -0.271

BI887141 -0.247 0.57 0.318 0.327 0.354 0.368 -0.157 0.501 -0.223 0.406 0.103 0.141

BI887157 0.172 1.249 0.998 1.117 0.266 -0.208 -0.496 0.015 -0.199 -0.33 -0.148 -0.444

BI887166 0.133 0.242 0.172 0.361 0.636 0.122 0.45 0.833 0.156 0.035 -0.074 -0.18

BI887172 0.36 0.238 0.86 0.975 1.743 0.948 1.018 1.916 0.273 0.395 0.093 0.011

BI887199 0.07 0.481 1.145 1.263 1.608 0.569 0.438 1.467 0.188 0.509 0.215 0.371

BI887226 0.631 0.774 1.577 1.514 1.704 2.587 2.379 2.332 1.737 1.325 0.847 0.672

BI887231 -0.545 -2.001 -0.8 -0.67 -0.408 0.642 0.297 0.504 0.377 1.288 1.08 0.462

BI887270 -0.852 -0.655 0.027 0.392 0.578 0.678 0.374 0.395 0.221 0.161 0.25 0.603

BI887309 -0.035 -0.251 0.865 0.795 0.768 0.277 0.087 0.769 0.231 0.333 -0.219 -0.197

BI887324 -0.18 0.3 0.749 0.54 0.922 0.868 0.448 0.641 0.309 0.131 -0.244 -0.486

BI887326 0.236 0.58 1.552 1.209 0.682 0.12 -0.22 0.207 0.243 -0.121 -0.314 -0.548

BI887338 0.599 0.324 1.356 2.578 1.4 1.321 1.149 1.225 0.969 0.494 0.025 -0.086

BI887346 -0.167 -0.595 -0.09 0.205 0.414 0.462 0.227 1.161 0.579 0.595 0.144 -0.045

BI887350 -0.22 0.716 0.636 0.243 0.771 0.839 0.511 0.631 0.119 0.082 -0.056 -0.379

BI887366 -0.872 -2.986 -1.682 -1.209 -0.679 0.346 0.225 0.54 0.379 1.067 0.699 0.098

BI887368 -0.627 0.484 4.191 4.239 4.366 2.479 2.782 4.767 3.248 0.972 1.059 -0.289

BI887377 -0.723 0.469 0.808 1.198 1.521 0.287 0.151 0.862 -0.156 -0.522 0.568 -0.501

BI887401 0.286 0.613 0.693 0.672 1.098 0.47 0.913 1.565 0.245 0.458 0.157 0.179

BI887446 -0.728 0.843 4.999 4.723 5.164 3.541 2.85 2.104 0.391 -0.38 -0.059 -0.19

BI887468 0.077 -0.517 0.377 0.148 0.222 0.756 0.239 0.708 0.655 0.582 0.138 0.187

BI887477 -0.438 0.623 0.467 0.566 1.039 -0.04 0.248 0.833 0.102 0.354 -0.022 0.143

BI887480 -0.005 0.618 0.788 1.018 1.11 0.193 0.346 0.912 0.019 0.28 0.385 -0.104

BI887500 1.487 1.142 -1.149 -0.761 -0.946 0.08 -0.736 -0.703 -0.491 -1.492 -0.415 -0.309

BI887512 -0.01 -0.297 0.309 0.401 0.896 1.086 0.374 1.037 0.313 0.662 0.113 0.246

BI887522 0.688 -0.261 0.566 -1.41 0.226 1.058 0.419 0.797 -0.78 -0.078 -0.32 -0.85

BI887526 0.21 -0.275 -0.29 -0.067 -0.098 0.431 -0.089 0.197 0.166 0.671 0.265 0.398

BI887534 0.352 1.231 0.862 0.256 0.132 0.057 -0.31 -0.067 -0.24 -0.111 -0.169 -0.13

BI887535 -0.001 0.101 1.259 1.209 1.598 0.166 0.326 0.967 0.188 0.489 0.099 0.012

BI887540 -0.95 -0.795 1.422 1.292 1.359 2.048 1.594 1.583 1.454 1.34 0.943 0.261

BI887548 -0.725 0.523 2.469 2.135 2.426 3.137 2.288 2.011 1.243 1.528 0.989 0.844

BI887559 -0.43 -0.904 0.027 0.295 0.577 0.494 0.226 0.983 0.265 0.658 0.243 0.282

BI887570 -0.205 0.791 1.502 1.342 1.096 0.689 0.564 0.339 -0.255 -0.242 -0.816 -0.913

BI887574 -0.626 -0.374 0.745 -0.085 0.26 0.537 0.343 0.787 0.467 0.654 -0.021 0.28

BI887620 0.319 -0.033 0.124 0.502 0.854 0.872 1.086 1.372 0.723 0.845 0.638 0.722

BI887627 -0.163 0.397 0.635 0.786 0.887 0.068 0.09 0.536 0.076 -0.163 -0.627 -0.594

BI887628 0.023 0.859 0.995 0.643 0.016 0.654 0.308 0.293 0.279 -0.389 -0.182 -0.302

BI887651 0.047 -0.561 0.071 0.169 0.882 1.121 0.733 0.842 0.241 -0.02 -0.135 -0.136

BI887656 -0.191 0.343 0.481 1.538 2.004 0.676 0.747 1.808 0.42 0.01 0.271 0.553

BI887658 0.211 1.057 0.978 0.54 0.273 0.223 0.043 0.193 0.263 -0.362 -0.374 -0.889

BI887659 -0.701 -0.062 0.214 0.668 1.419 0.944 0.525 1.618 0.846 0.473 0.175 0.002

BI887704 0.016 0.222 0.854 0.523 0.784 1.005 0.552 0.881 0.441 0.495 0.257 0.411

BI887709 -1.484 -0.61 0.814 1.017 1.389 2.209 1.917 1.841 1.374 1.642 0.772 1.117

BI887718 -0.791 -0.525 2.215 3.674 4.74 4.448 5.293 4.54 3.835 0.612 0.468 -0.196

BI887737 0.197 -0.312 -0.937 -0.397 -0.238 0.681 0.148 0.548 0.421 0.765 0.179 0.198

BI887742 -1.164 -0.028 3.072 2.684 1.29 1.018 1.191 1.77 1.082 1.139 0.474 0.31

BI887764 -0.166 -0.148 1.247 1.069 1.686 0.61 0.671 1.161 0.635 0.345 0.567 0.02

BI887770 1.303 0.148 -0.837 -0.403 -0.648 -0.9 -0.36 -1.077 -1.286 -1.244 -2.087 -1.571

BI887789 0.319 -0.817 0.325 0.712 1.465 0.392 0.683 0.855 0.818 0.644 1.033 0.303

BI887791 0.738 1.293 1.156 1.081 0.836 0.71 0.63 0.767 -0.072 0.001 -0.011 -0.246

BI887812 -0.491 0.115 1.565 1.179 1.569 1.326 1.203 1.885 1.133 0.684 0.174 0.361

BI887817 0.118 -0.177 0.402 0.546 1.46 0.666 0.661 1.592 0.38 0.214 -0.029 -0.165

BI887847 0.61 0.965 1.268 1.177 0.788 0.474 0.541 0.807 0.043 0.088 -0.388 -0.576

BI887861 0.136 0.37 1.073 0.819 1.835 0.96 0.277 0.014 -0.527 -0.237 -1.15 -1.157

BI887931 -0.629 -0.056 0.427 0.792 1.069 1.367 0.726 1.058 0.374 0.412 0.642 0.431

BI887935 0.21 1.13 0.655 1.352 1.556 0.186 0.66 1.079 0.325 0.075 -0.176 -0.291

BI887957 -0.319 1.279 1.837 1.362 0.52 0.039 0.191 0.589 0.113 0.094 -0.003 -0.114

BI887958 0.464 1.133 0.872 0.211 -0.447 -0.21 -0.37 0.231 -0.064 -0.189 -0.211 -0.42

BI887990 -0.482 -0.195 0.108 0.092 -0.027 0.436 0.393 0.522 0.342 0.505 0.41 0.139

BI888000 -0.728 -0.05 0.9 1.579 1.472 0.508 0.378 1.501 0.62 0.144 1.035 0.675

BI888008 0.323 0.553 0.041 0.039 -0.011 0.901 0.124 0.589 0.138 0.512 0.238 0.684

BI888024 0.143 -0.936 0.281 0.619 0.761 0.774 0.684 0.575 0.476 0.383 -0.002 0.245

BI888033 -0.728 0.119 -0.124 0.55 1.038 -0.063 0.225 1.296 0.491 0.525 0.516 0.404

BI888090 -0.024 -0.167 0.765 0.549 2.432 2.316 1.942 2.327 1.465 0.27 0.057 -0.109

BI888131 1.944 0.378 0.512 0.566 0.25 0.489 0.087 0.47 -0.166 -0.216 -0.681 -1.159

BI888149 -0.111 -0.326 0.14 0.55 0.956 0.967 0.753 0.723 0.279 0.106 -0.016 0.11

BI888150 1.062 -2.111 0.455 1.152 1.217 1.738 1.336 0.92 0.655 1.051 0.639 0.2

BI888158 -0.567 0.055 2.182 2.398 3.208 1.226 0.67 2.981 1.848 0.477 0.048 0.042

BI888165 0.092 -0.167 0.22 -0.05 0.901 0.478 0.216 0.6 0.18 0.207 0.103 0.04

BI888166 1.014 -0.557 0.099 0.848 1.435 1.862 1.414 1.931 0.426 0.984 0.608 0.344

BI888169 -0.002 -1.276 -0.706 -0.244 0.144 1.06 0.839 0.81 0.32 0.892 0.418 0.145

BI888172 -0.069 0.004 0.057 1.149 1.929 0.219 0.186 0.406 0.436 -0.029 -0.132 -0.204

BI888177 -0.01 0.348 -0.054 0.445 0.422 0.422 0.189 0.886 0.49 0.258 0.348 0.211

BI888179 -0.168 1.003 2.581 2.108 1.087 -0.176 0.143 0.165 -0.032 -0.319 0.321 0.211

BI888186 0.454 -0.156 0.032 0.179 0.473 0.856 0.506 1.096 0.781 1.052 0.528 0.468

BI888206 1.461 1.396 2.219 1.609 1.414 1.25 1.101 0.779 0.194 0.034 -0.116 -0.013

BI888210 -0.991 -0.05 1.499 1.138 1.236 0.979 0.842 1.017 1.206 0.714 0.384 0.015

BI888232 -0.195 0.697 1.866 3.261 3.045 1.671 1.983 2.777 1.522 -0.301 0.721 -0.392

BI888241 -0.728 0.04 0.618 1.503 2.68 1.586 1.825 1.617 0.395 -0.762 -0.274 -0.505

BI888258 -0.51 0.062 0.597 0.844 1.021 -0.054 0.195 1.114 0.218 0.218 0.296 0.178

BI888263 -0.033 1.661 2.115 1.385 -0.152 -0.173 -0.463 -1.141 -0.82 -1.362 -1.642 -1.449

BI888265 1.281 0.802 -0.819 -0.359 -0.627 -0.423 -0.717 -0.718 -0.433 -1.092 -0.757 -1.067

BI888267 -0.026 -0.34 0.953 1.409 2.243 0.337 0.844 1.119 0.524 0.489 0.753 -0.474

BI888338 -1.058 -0.729 1.79 1.534 2.663 2.163 1.734 2.502 1.936 0.469 1.079 0.39

BI888344 -1.041 -1.027 0.084 0.256 0.891 0.619 0.313 0.673 1.001 0.263 0.657 0.388

BI888349 -0.675 0.23 0.969 1.43 1.394 0.231 0.09 0.247 -0.36 -0.622 0.019 -0.297

BI888350 2.148 1.536 0.863 0.877 0.35 0.466 0.282 0.266 -0.118 -0.257 -0.522 -0.675

BI888359 0.647 0.467 0.066 0.602 0.469 0.61 0.202 -0.151 0.111 -0.095 -0.332 0.021

BI888360 -0.07 -1.288 1.24 2.033 1.955 0.744 0.721 1.108 0.372 0.75 0.753 0.319

BI888372 0.725 1.168 1.007 0.511 -0.158 -0.295 -0.046 -0.368 -0.158 -0.236 -0.314 -0.55

BI888425 0.52 0.694 1.459 1.477 1.235 1.428 1.205 1.043 0.423 0.747 0.575 0.342

BI888431 0.308 0.018 1.028 0.665 1.805 0.693 0.305 1.212 0.172 -0.17 -0.02 -0.049

BI888432 -0.199 -0.176 0.34 0.769 1.178 0.894 0.579 1.215 1.048 0.645 0.172 -0.297

BI888449 0.026 1.131 1.103 0.729 0.821 0.593 0.282 0.397 -0.184 -0.046 -0.428 -0.615

BI888458 0.149 0.751 0.733 0.911 0.834 0.156 0.657 0.946 -0.241 -0.076 -0.076 -0.346

BI888483 -0.438 0.993 5.111 4.773 5.126 2.01 1.304 2.75 1.361 -0.117 -0.144 0.092

BI888493 -0.11 -0.057 1.792 2.345 2.239 0.066 0.389 1.186 0.032 0.181 0.311 -0.199

BI888505 0.288 0.726 0.419 0.644 0.852 -0.256 0.384 1.143 -0.022 0.092 0.002 -0.229

BI888520 0.902 0.755 1.51 1.736 1.142 0.16 -0.063 0.257 0.298 -0.006 0.104 -0.062

BI888534 0.469 1.019 1.081 0.698 0.916 0.602 0.338 1.171 0.151 0.342 0.074 -0.112

BI888545 -0.535 0.102 1.261 1.244 1.79 0.441 0.632 1.7 1.06 0.096 0.597 0.087

BI888550 -0.756 -0.802 -0.356 0.293 0.844 1.217 0.629 0.812 -0.286 -0.878 -1.165 -1.088

BI888564 0.077 0.476 1.388 1.506 2.022 0.556 0.356 1.225 -0.114 -0.185 -0.11 0.016

BI888567 0.283 0.689 1.137 1.271 1.497 0.968 0.598 1.318 0.559 0.247 0.251 0.333

BI888569 0.782 0.097 -0.415 -0.232 -0.575 -0.032 -0.043 -0.289 -0.112 0.112 -0.221 -0.17

BI888571 -0.493 1.216 1.862 1.432 1.118 0.348 0.206 0.317 0.379 -0.085 -0.108 -0.324

BI888576 -0.269 0.899 0.889 1.035 0.693 -0.039 0.349 0.999 0.281 0.397 0.075 -0.436

BI888606 0.139 0.639 1.428 0.292 -0.006 0.468 0.819 2.067 0.641 0.975 1.109 0.157

BI888620 1.143 1.28 2.635 2.463 2.685 1.633 0.894 -0.73 -1.085 -0.832 -1.243 -0.986

BI888634 0.106 1.083 0.656 0.915 0.425 0.456 0.118 0.59 0.062 0.055 -0.159 -0.111

BI888683 -0.632 0.797 0.037 0.421 0.149 -0.322 -0.142 0.831 0.103 -0.39 -0.365 -0.323

BI888703 0.684 1.38 1.363 0.573 0.692 0.864 0.564 0.782 -0.027 -0.156 -0.171 -0.188

BI888721 -0.525 0.77 1.886 1.942 2.106 2.55 1.879 2.022 1.506 0.724 -0.212 -1.139

BI888729 -1.749 -1.295 -1.547 -0.988 -0.625 -0.261 -0.795 -0.395 0.143 0.386 0.058 -0.013

BI888732 1.092 0.083 0.784 0.188 0.832 1.41 0.606 0.289 0.584 0.479 0.388 0.356

BI888742 0.814 0.583 0.608 0.519 0.187 0.041 0.116 -0.115 -0.204 -0.376 -0.719 -0.635

BI888748 -0.018 0.078 1.517 1.524 1.81 1.091 1.067 1.429 0.711 0.487 -0.333 -0.774

BI888750 -0.189 -0.684 1.319 1.747 1.055 0.381 0.067 0.759 0.025 -0.231 0.036 0.097

BI888755 -1.44 -1.054 0.551 0.444 0.276 0.614 0.472 0.447 0.43 0.34 0.164 0.875

BI888760 0.672 1.167 0.563 0.433 0.043 0.322 -0.382 -0.638 -0.443 -0.346 -0.706 -0.358

BI888784 -0.681 -0.498 0.363 1.113 1.238 0.373 0.304 0.306 0.474 -0.017 0.118 0.618

BI888791 -0.38 0.148 0.243 1.236 1.632 0.735 1.209 1.865 0.663 -0.543 -1.019 -0.862

BI888801 0.314 0.814 0.626 0.715 0.243 -0.121 0.302 0.97 0.136 0.63 0.515 -0.061

BI888812 -0.992 -2.583 -1.804 -1.041 -0.646 0.337 0.228 0.293 0.263 1.001 0.661 0.341

BI888816 0.368 0.368 1.371 1.172 1.188 0.97 0.997 0.938 0.889 0.516 0.109 -0.597

BI888818 -0.666 0.335 1.273 1.023 0.845 0.774 -0.621 0.361 0.431 0.262 0.013 0.188

BI888822 0.077 1.03 1.05 1.025 0.409 -0.277 0.234 0.19 -0.291 -0.095 -0.565 -0.638

BI888826 0.564 1.014 1.78 1.049 1.154 0.216 -0.05 0.13 -0.128 -0.378 -0.47 -0.525

BI888828 0.773 1.088 0.918 1.233 1.101 1.198 0.506 0.807 0.087 0.554 0.185 -0.007

BI888859 -0.029 0.081 0.701 0.675 1.043 1.688 1.078 1.331 0.88 0.758 0.141 0.417

BI888863 -0.06 0.227 0.663 0.729 0.864 0.746 0.249 0.931 0.276 0.352 0.047 -0.012

BI888884 -0.18 0.823 0.584 -0.025 -0.046 -0.143 0.001 -0.181 -0.327 -0.068 -0.136 -0.417

BI888888 0.045 0.742 1.514 1.41 1.144 0.886 0.166 0.394 0.024 -0.195 -0.286 -0.242

BI888891 -0.279 -0.312 2.211 1.804 1.677 0.503 0.306 1.008 0.27 -0.243 0.248 -0.088

BI888897 -1.229 -2.824 -1.785 -1.181 -0.974 0.193 -0.091 0.092 0.039 0.735 0.348 -0.092

BI888899 1.112 0.462 -0.578 -0.7 -0.177 0.03 0.004 -0.216 -0.165 -0.39 -0.362 -0.098

BI888908 0.105 0.683 0.673 0.622 0.727 0.433 0.245 0.346 -0.056 -0.186 -0.391 -0.403

BI888914 1 0.771 0.739 1.068 1.076 0.646 0.608 0.696 -0.097 0.043 -0.485 -0.677

BI888920 -0.018 -0.318 0.553 0.721 1.323 0.897 0.612 0.937 0.519 0.467 0.201 -0.105

BI888926 -0.608 -1.851 -0.112 0.11 0.638 1.155 0.613 0.55 -0.029 0.704 0.383 0.495

BI888928 0.855 -0.448 -0.357 0.299 0.5 0.69 0.685 1.439 0.458 0.467 0.585 0.283

BI888934 -0.783 -0.551 -1.144 -0.24 0.533 0.361 0.283 1.302 0.769 0.944 0.612 1.509

BI888943 1.481 0.606 0.323 0.403 -0.357 -0.634 -1.182 -1.478 -1.297 -1.547 -1.683 -2.113

BI888977 0.578 1.082 2.224 2.134 1.942 1.339 1.073 1.517 0.761 0.789 0.519 0.16

BI888982 0.147 1.702 1.479 0.853 0.32 0.529 -0.012 0.06 -0.379 -0.337 0.084 -0.989

BI888986 0.279 1.188 1.047 1.284 1.238 0.321 0.606 0.688 0.031 -0.044 -0.487 -0.671

BI889005 0.607 1.205 1.788 1.673 1.66 1.733 0.775 1.253 0.53 0.265 -0.535 -0.968

BI889009 1.34 1.756 0.641 0.268 -0.145 -0.155 -0.339 -0.34 -0.597 -0.845 -0.85 -1.089

BI889064 -0.087 1.037 1.942 1.998 1.267 0.843 0.462 0.96 0.012 0.172 -0.035 0.143

BI889074 0.307 0.591 0.797 0.406 0.934 0.802 0.935 1.107 1.11 0.749 0.181 -0.666

BI889101 1.659 1.234 1.209 1.308 1.215 0.594 0.293 0.671 -0.054 -0.595 -0.57 -0.333

BI889113 0.98 1.194 1.865 2.179 1.371 1.229 1.238 1.15 0.53 0.475 -0.01 -0.448

BI889119 0.746 1.614 1.708 1.8 1.376 1.006 0.559 1.102 0.4 -0.222 0.108 -0.465

BI889129 1.37 1.333 2.155 2.24 2.077 2.091 1.058 1.625 0.767 0.399 -0.686 -0.411

BI889130 0.02 -0.615 0.622 1.87 4.055 2.217 1.535 2.181 1.275 0.022 -0.163 -0.363

BI889131 -0.601 -0.014 0.006 0.433 0.771 -0.04 0.234 0.902 0.287 0.335 0.091 -0.054

BI889140 0.176 0.384 0.309 1.308 1.748 1.262 1.216 1.84 0.403 0.463 0.435 0.317

BI889160 0.123 0.816 1.683 1.64 1.441 0.211 0.838 0.637 0.364 -0.649 -0.199 -0.107

BI889166 -0.93 -0.964 1.667 2.222 2.232 2.397 2.12 2.434 1.542 1.662 1.003 0.224

BI889170 0.095 -0.055 0.51 0.822 1.318 0.965 0.926 1.127 0.155 0.232 -0.204 -0.169

BI889190 -0.643 -1.184 0.411 1.186 1.448 1.606 0.88 1.167 0.5 0.29 0.528 0.24

BI889194 -0.577 0.037 0.587 1.239 1.632 -0.116 0.264 0.754 0.322 0.275 0.392 -0.039

BI889202 -0.289 -0.363 0.182 0.284 0.772 0.344 0.466 1.021 -0.036 0.026 0.164 0.177

BI889241 -0.661 -0.451 0.839 1.163 1.29 1.395 0.633 0.888 0.379 -0.068 0.596 -0.063

BI889280 0.42 -0.441 0.591 1.001 1.383 0.305 0.755 1.604 0.627 0.528 0.329 -0.446

BI889290 0.365 0.665 1.253 1.263 1.493 1.047 0.491 1.075 0.129 0.248 -0.115 -0.234

BI889298 -0.963 -0.727 1.212 1.632 1.616 1.92 1.521 1.855 1.465 1.092 0.472 0.53

BI889302 -0.309 0.105 0.437 0.073 0.165 0.014 0.428 1.442 0.082 0.122 -0.083 0.527

BI889310 -0.124 0.214 0.238 0.664 0.692 0.334 0.432 0.748 0.197 0.206 -0.427 -0.472

BI889332 0.355 0.464 0.409 0.466 0.754 0.317 0.243 0.145 0.009 0.091 -0.199 -0.126

BI889335 0.207 0.789 1.229 1.095 0.593 0.04 0.038 -0.088 0.004 -0.413 -0.048 -0.346

BI889370 -1.505 -1.791 -1.555 -0.444 0.061 0.712 0.594 1.074 0.752 1.344 0.869 -0.617

BI889396 -0.505 -0.015 0.201 0.612 0.896 -0.013 0.181 0.681 0.179 0.03 0.289 -0.01

BI889398 0.024 0.807 1.253 1.286 2.089 1.138 2.005 3.676 1.857 0.508 -0.139 -0.246

BI889402 0.723 1.149 1.236 0.916 0.551 0.062 -0.026 0.532 -0.219 0.337 0.303 -0.187

BI889409 1.681 -0.141 -0.417 -0.023 0.277 1.071 0.437 0.979 0.332 0.376 0.058 -0.354

BI889410 -0.529 0.073 1.206 1.115 0.994 1.008 0.815 0.351 0.332 0.453 -0.005 0.043

BI889437 1.23 1.628 2.012 1.855 0.916 1.126 0.843 0.626 0.221 0.06 -0.038 -0.731

BI889445 -0.734 -2.851 -1.29 -0.81 -0.641 0.539 0.088 0.326 0.15 0.918 0.621 0.236

BI889456 -0.566 -0.272 0.348 0.576 0.838 1.186 0.624 1.047 0.575 0.367 0.239 -0.019

BI889458 0.105 1.181 1.389 1.255 1.171 0.306 0.09 0.293 -0.124 -0.038 -0.035 -0.127

BI889465 0.95 0.277 -0.067 -0.038 -0.341 0.211 -0.523 -0.126 -0.36 0.324 0.125 0.087

BI889526 -0.542 -0.235 0.916 1.028 1.015 1.218 0.563 1.338 0.373 0.122 -0.048 0.2

BI889529 -4.057 -4.534 -2.887 -2.368 -2.081 -0.716 -1.026 -0.761 -0.06 0.867 0.685 0.452

BI889533 0.048 -0.127 0.298 0.074 -0.053 -0.279 -0.022 0.926 -0.041 0.295 -0.405 -0.383

BI889553 0.197 0.476 0.267 0.678 0.813 0.172 0.18 0.381 -0.37 -0.026 -0.292 -0.117

BI889566 -0.419 -0.85 -0.027 -0.362 -0.602 -0.247 0.121 1.031 0.616 1.344 1.312 0.118

BI889609 -0.372 -0.97 0.969 0.685 1.429 1.069 0.879 0.86 0.868 1.146 1.01 0.412

BI889621 -0.953 -0.862 0.165 0.352 0.615 0.428 0.005 0.555 0.081 0.935 0.438 0.832

BI889627 -0.039 0.941 1.118 0.996 0.469 0.126 0.096 -0.092 -0.151 -0.09 -0.459 -0.041

BI889637 -0.998 -0.213 0.805 1.805 1.697 -0.11 0.185 -1.339 -1.211 -0.444 -0.659 0.034

BI889650 0.438 1.067 1.281 0.763 0.803 0.438 0.238 0.582 0.162 0.323 0.054 0.07

BI889676 -0.771 1.396 1.979 1.773 1.542 1.467 1.292 0.314 0.546 -0.379 -0.011 -0.824

BI889686 0.057 1.039 0.966 0.785 0.26 -0.118 0.136 0.375 -0.104 -0.234 -0.601 -0.657

BI889700 1.168 0.491 0.378 0.794 1.082 1.364 0.895 0.817 0.051 -0.233 -0.257 -0.623

BI889705 0.052 1.062 0.61 0.708 0.016 -0.176 0.046 0.124 -0.422 0.199 0.012 -0.153

BI889720 0.607 0.707 1.975 1.545 1.711 1.667 1.362 1.708 0.544 0.681 0.159 0.032

BI889786 -0.592 -0.224 0.329 -0.058 0.719 0.242 0.43 1.88 0.831 0.626 0.618 -0.513

BI889810 0.369 0.846 1.635 1.631 1.244 1.05 0.737 1.276 0.636 0.377 -0.12 -0.12

BI889835 0.873 0.175 1.498 1.008 0.741 0.017 -0.131 -0.728 -0.733 -1.715 -1.066 -1.089

BI889901 0.255 0.192 1.275 1.641 1.234 0.66 0.757 0.506 0.138 0.122 -0.097 0.048

BI889917 0.131 0.098 -0.6 -0.28 1.228 1.231 0.794 1.542 -0.07 0.32 0.222 -0.158

BI889922 -2.288 -1.316 1.667 2.018 2.774 2.812 2.82 2.879 2.829 -0.87 -1.154 -1.457

BI889925 1.904 -1.509 -1.511 -1.852 -2.242 -1.102 -1.552 -2.284 -1.875 -1.043 -1.95 -1.002

BI889933 0.021 0.406 0.306 0.636 0.852 0.102 0.439 1.003 0.256 0.303 0.443 -0.062

BI889937 -0.892 0.267 0.861 0.938 1.22 0.35 -0.08 -0.734 0.148 0.076 0.003 -1.026

BI889943 1.191 1.845 1.753 0.194 -2.17 -1.242 -1.987 -2.778 -2.313 -3.369 -2.553 -2.505

BI889944 -0.395 0.587 0.875 0.856 0.786 0.111 0.099 -0.353 0.295 -0.425 -0.366 -0.463

BI889946 0.555 0.334 0.59 0.121 0.48 1.18 0.877 1.201 0.699 0.919 0.444 0.017

BI889958 1.049 1.784 1.048 1.518 0.719 0.31 0.376 0.186 -0.524 -0.163 -1.109 -1.515

BI889959 1.303 -1.154 -0.915 -0.964 -1.9 -0.604 -1.089 -2.161 -1.307 -0.875 -1.636 -0.975

BI889982 -0.03 -0.499 -0.452 -0.136 0.357 0.113 0.151 0.884 0.027 0.77 0.34 0.448

BI889995 0.019 1.405 1.454 1.386 0.922 0.001 -0.053 0.021 -0.254 -0.756 -0.389 -0.927

BI890013 0.559 -0.731 0.232 -0.039 0.617 0.482 0.456 0.94 0.489 0.809 0.704 -0.197

BI890023 -0.393 -0.456 -0.051 -0.108 0.025 0.613 0.505 0.776 0.54 0.901 0.515 -0.18

BI890034 -0.645 -0.75 1.688 1.398 1.372 0.551 0.855 1.619 1.225 0.972 0.716 -0.164

BI890038 0.182 1.28 0.64 1.501 0.992 0.426 -0.061 0.244 -0.221 -0.24 -0.535 -0.035

BI890050 -0.134 -0.668 -0.111 0.347 0.955 0.453 0.761 1.217 0.542 0.512 0.45 0.276

BI890056 1.867 -0.331 0.554 0.994 0.513 0.619 -0.001 -0.506 -0.485 -1.176 -1.098 -1.316

BI890073 -0.275 0.415 1.398 1.833 1.712 0.781 1.057 1.716 0.153 0.481 0.419 0.573

BI890108 -0.502 -1.355 0.259 0.369 0.902 0.873 0.558 1.819 0.902 1.173 0.543 0.044

BI890113 -1.149 -2.935 -1.558 -1.169 -0.93 0.223 0.063 0.176 0.192 0.871 0.486 -0.044

BI890141 0.218 0.137 0.246 0.792 1.365 0.386 0.601 1.469 0.275 0.257 0.174 0.295

BI890158 1.432 -0.082 -1.082 -1.156 -1.313 -0.408 -0.786 -1.409 -1.237 -0.946 -1.734 -1.891

BI890191 -0.438 -0.363 1.143 1.333 2.239 0.83 0.461 1.514 0.662 0.347 0.456 0.151

BI890193 0.552 1.677 2.238 1.779 1.911 1.577 0.694 0.75 -0.155 -0.609 -0.415 -0.67

BI890195 0.522 0.652 0.794 0.979 0.543 0.289 0.103 0.547 0.177 0.092 -0.282 -0.349

BI890196 -0.791 0.19 -0.295 0.365 0.663 1.497 1.113 1.521 1.318 0.584 0.33 0.128

BI890214 -0.682 0.938 2.635 1.725 1.109 0.925 -0.2 0.332 0.255 -0.229 -0.164 -0.526

BI890218 -0.725 -2.578 -1.236 -1.145 -0.79 0.059 0.127 0.301 0.015 0.992 0.613 0.291

BI890235 -0.289 -0.044 0.036 0.616 0.252 0.778 0.265 0.721 0.236 0.623 0.148 0.385

BI890247 0.321 0.482 0.614 0.81 1.18 0.528 0.473 1.237 0.183 0.411 0.32 0.043

BI890250 0.058 1.066 1.703 1.222 1.595 0.58 0.751 1.809 1.011 0.619 0.259 0.273

BI890257 0.049 0.658 0.491 0.495 0.747 0.835 0.401 0.806 0.181 0.194 -0.003 0.026

BI890262 -0.728 0.17 1.09 2.848 2.359 0.268 -0.219 0.193 -0.378 -0.68 -0.117 -0.384

BI890279 -0.199 -0.483 1.692 2.054 2.906 1.847 1.443 1.88 0.563 0.387 0.147 0.222

BI890287 -1.467 -2.301 -0.023 0.517 1.214 1.119 0.978 0.835 0.073 -0.503 -0.828 -1.662

BI890294 -0.144 -0.11 0.704 1.974 2.251 0.889 0.575 1.213 0.487 0.208 0.155 0.125

BI890305 -0.728 -0.028 0.201 1.267 1.079 -0.03 0.245 0.844 0.264 0.262 0.872 0.652

BI890314 -0.248 -0.196 0.655 2.21 1.637 0.228 1.112 1.317 0.234 -0.021 0.39 0.84

BI890315 -0.462 0.29 0.766 0.725 1.19 0.098 -0.153 0.807 0.305 0.681 0.843 0.203

BI890317 0.907 1.334 0.661 0.684 0.887 0.319 -0.008 0.726 0.108 -0.027 0.191 -0.091

BI890323 -0.93 0.55 2.61 2.294 1.78 1.489 0.673 0.948 1.209 0.698 0.121 -0.114

BI890334 -0.503 -0.096 1.285 1.668 1.927 0.674 1 0.976 0.919 0.241 -0.208 -0.512

BI890367 -0.771 0.247 0.862 1.822 1.886 1.07 0.339 1.694 1.73 1.5 0.567 0.091

BI890375 1.333 1.22 1.52 1.497 0.974 1.578 1.288 0.649 0.621 0.55 0.182 0.037

BI890420 -0.058 0.13 0.475 0.769 1.007 0.828 0.715 0.763 0.446 0.192 0.262 -0.161

BI890439 -0.514 0.081 1.452 2.519 3.822 3.403 2.873 3.828 1.495 0.191 0.148 -0.304

BI890444 -0.087 -0.021 0.71 0.652 1.47 1.837 0.662 1.028 0.674 0.644 0.37 0.228

BI890446 -5.187 -4.116 0.084 0.595 1.476 2.73 2.164 2.261 1.733 1.813 1.288 1.067

BI890453 -0.405 1.09 2.373 1.776 1.967 1.465 0.729 1.175 0.975 0.795 0.33 0.16

BI890462 -0.09 -0.339 0.336 0.513 0.607 0.16 0.243 0.814 0.206 0.632 0.614 1.065

BI890477 -0.378 -0.06 1.048 0.248 1.597 1.577 0.376 1.105 0.574 0.249 0.156 -0.009

BI890491 1.196 1.385 2.264 2.165 1.656 2.236 1.508 1.57 0.692 0.555 -0.196 -0.43

BI890492 0.124 0.734 0.752 0.897 1.272 0.159 0.704 0.868 0.303 -0.058 0.185 -0.142

BI890505 -0.285 -0.09 4.319 3.893 4.671 3.049 1.938 1.746 0.989 -0.311 0.149 -0.077

BI890508 0.304 -0.413 0.111 0.245 0.847 0.552 0.47 0.83 0.356 0.268 0.406 0.471

BI890512 -0.619 0.788 1.553 1.003 1.474 1.431 0.711 1.432 0.675 0.506 0.367 0.215

BI890519 0.387 0.479 1.25 1.042 1.153 0.319 0.2 0.694 0.08 0.005 -0.356 -0.542

BI890587 -0.126 0.229 3.047 3.361 3.914 2.207 3.129 3.702 3.213 0.388 0.772 -0.033

BI890592 1.382 0.223 0.294 0.176 0.465 0.424 0.137 0.877 0.204 -0.016 0.246 -0.177

BI890607 -0.68 -1.293 -0.427 -0.061 -0.044 0.549 0.192 0.444 0.158 0.333 0.051 -0.329

BI890609 0.567 -0.045 0.172 0.224 0.632 1.762 1.62 0.936 0.869 0.138 -0.419 -0.864

BI890612 0.882 1.243 2.185 1.494 1.417 1.814 1.16 0.908 0.319 0.473 0.16 0.787

BI890616 -0.683 0.169 1.564 1.324 1.028 0.294 -0.358 0.549 0.601 0.422 0.407 0.192

BI890618 -0.728 0.138 0.238 1.14 1.921 0.883 0.79 1.711 0.755 0.868 0.824 -0.057

BI890643 -0.72 0.323 0.494 0.966 1.192 -0.085 0.256 0.991 -0.128 -0.061 -0.128 0.124

BI890669 -0.011 0.059 1.836 0.835 2.062 2.269 1.703 1.855 1.04 0.883 0.553 0.23

BI890682 -0.009 0.164 0.389 0.747 0.938 0.005 0.177 -0.021 0.182 0.033 -0.18 -0.266

BI890693 0.424 1.442 0.663 0.326 0.037 0.448 -0.22 0.309 -0.411 -0.177 0.318 -0.251

BI890704 0.302 0.594 0.268 0.378 0.358 0.844 0.498 0.95 0.372 0.328 0.004 -0.126

BI890730 0.135 -0.08 0.171 0.591 1.052 0.243 0.432 1.53 0.565 0.062 -0.264 -0.052

BI890739 -0.044 0.123 1.074 1.599 1.94 0.867 0.742 1.875 0.39 0.417 0.688 -0.076

BI890749 -0.107 0.06 0.61 0.57 0.754 0.105 0.328 0.714 0.561 0.256 0.059 -0.092

BI890755 -0.269 -0.589 0.586 0.912 1.268 0.861 0.645 0.329 0.759 0.494 0.265 0.033

BI890771 0.276 0.736 0.531 0.582 0.446 0.285 0.081 0.893 0.244 0.626 0.545 0.164

BI890772 0.065 -1.307 -0.353 -0.191 0.144 0.522 -0.002 0.096 0.398 0.587 -0.093 0.038

BI890789 -0.617 0.786 0.794 1.114 0.631 0.513 0.004 0.503 0.134 -0.03 -0.038 -0.164

BI890821 0.599 0.75 1.667 1.795 1.393 1.971 1.662 1.526 0.871 1.079 0.876 0.525

BI890823 -0.244 -0.159 0.691 1.14 1.6 1.535 0.912 1.284 0.972 0.822 0.837 0.175

BI890830 1.213 0.815 1.713 1.184 1.153 0.908 0.319 0.227 -0.472 -0.43 -0.377 -0.767

BI890848 1.3 -0.596 1.444 1.07 1.136 1.949 0.975 1.323 1.069 0.578 -0.068 -0.389

BI890854 0.371 1.144 0.514 0.741 0.824 0.367 0.688 0.528 -0.058 0.093 -0.129 0.053

BI890861 -1.181 -1.99 -0.937 -1.081 -0.49 0.335 0.101 0.423 0.198 1.064 0.826 0.374

BI890871 0.533 0.783 1.165 0.696 0.783 0.988 0.318 0.706 0.342 0.199 -0.254 -0.375

BI890893 0.874 0.005 0.708 0.413 1.103 0.537 0.375 1.064 -0.033 -0.3 -0.119 -0.031

BI890894 -0.014 0.729 0.873 0.761 0.552 -0.064 0.219 0.811 0.071 -0.115 -0.042 -0.342

BI890906 -0.854 -2.499 -0.93 -0.976 -0.607 -0.024 0.213 0.32 0.139 1.214 0.734 0.286

BI890907 0.984 0.878 0.485 0.094 -0.591 -0.08 -0.224 -0.317 -0.38 -0.46 -0.567 -0.366

BI890925 -0.205 1.093 0.887 0.727 0.203 -0.215 -0.207 0.28 -0.155 -0.507 -0.381 -0.322

BI890940 -0.477 0.503 3.17 1.253 1.054 0.067 0.369 0.45 0.307 -0.539 0.341 -0.005

BI890954 -0.337 -0.396 -0.422 -0.401 -0.88 -0.252 -0.229 -0.076 0.085 0.9 0.815 0.653

BI891001 -1.158 -1.384 -0.627 0.24 0.119 0.592 0.412 1.011 0.16 0.531 0.659 0.802

BI891040 -0.575 -2.54 -1.146 -0.869 -0.684 -0.021 -0.001 0.187 0.044 1.09 0.722 0.339

BI891041 -0.203 -0.189 0.415 0.27 0.586 0.249 0.218 0.673 0.345 0.768 -0.054 -0.001

BI891043 0.816 0.083 -0.195 -0.392 -0.346 -0.083 -0.154 -0.238 -0.197 0.145 0.045 0.039

BI891054 -0.189 0.929 1.88 1.628 1.095 -0.023 0.943 0.516 0.177 -0.081 0.095 -0.26

BI891068 -0.046 0.847 0.558 0.753 1.104 0.043 0.411 1.189 0.138 0.193 0.119 0.113

BI891069 0.147 0.413 0.608 0.691 0.576 0.129 0.181 0.765 0.004 0.14 -0.093 -0.365

BI891076 0.3 0.27 -0.037 0.211 0.031 0.105 -0.019 0.816 -0.143 -0.192 -0.315 -0.166

BI891090 -0.208 0.555 0.246 0.929 0.957 0.626 0.304 0.409 -0.005 -0.439 -0.367 -0.173

BI891108 0.618 0.51 1.673 -0.115 0.771 1.507 0.535 0.738 0.229 0.12 -0.073 -0.144

BI891112 0.9 0.906 1.087 0.958 0.055 -0.17 -0.394 0.098 -0.244 -0.056 -0.244 -0.38

BI891116 0.296 0.932 1.414 1.07 1.516 1.417 1.039 1.569 0.944 0.432 0.218 -0.052

BI891122 -0.222 -0.152 0.366 0.649 0.468 0.617 0.326 0.919 0.295 0.652 0.158 0.151

BI891136 0.025 0.361 0.758 0.955 1.059 0.578 0.688 0.847 0.435 0.313 -0.035 0.226

BI891138 -0.667 -3.238 -1.973 -0.918 -0.846 0.381 0.072 0.294 0.392 1.085 0.723 0.102

BI891158 -1.998 -2.154 -0.18 -0.886 0.478 3.195 1.919 1.072 1.235 -1.221 -0.852 -0.985

BI891176 0.195 0.258 0.646 0.741 1.15 0.669 0.816 1.073 0.309 0.395 -0.032 0.092

BI891235 -0.06 0.346 0.655 1.159 0.755 0.002 0.027 -0.096 -0.048 0.118 0.036 -0.129

BI891245 -1.868 -2.408 0.065 1.288 1.382 1.273 0.992 1.096 0.201 0.466 0.709 0.641

BI891276 0.109 -0.73 0.448 0.585 0.612 0.694 0.348 0.725 0.214 0.344 0.417 -0.147

BI891278 -0.916 -2.831 -0.923 -0.106 0.79 1.201 1.326 1.401 0.528 0.875 0.707 0.733

BI891279 -1.118 -1.771 0.474 0.973 1.074 0.88 0.705 0.954 0.789 0.743 0.262 -0.45

BI891290 0.356 -0.442 0.277 0.339 0.95 0.191 0.632 1.737 0.417 0.578 0.559 0.384

BI891293 0.318 1.373 0.3 0.389 0.516 0.134 0.364 0.996 0.263 -0.115 -0.015 0.116

BI891320 0.594 0.645 1.328 1.705 1.746 0.994 1.596 1.706 0.751 1.04 0.457 0.173

BI891327 0.905 0.598 0.675 0.161 0.088 0.756 0.144 0.231 -0.043 -0.224 -0.65 -0.506

BI891329 0.151 0.973 1.346 1.332 0.983 0.745 0.545 0.757 0.33 0.217 -0.373 -0.441

BI891331 -0.696 -0.05 0.418 0.563 0.666 -0.15 -0.051 0.304 -0.048 0.321 0.174 0.217

BI891332 1.035 -0.436 -0.261 -0.212 0.092 -0.123 0.085 0.134 -0.38 0.18 0.235 0.164

BI891338 -0.493 -0.665 1.322 2.059 2.403 0.907 0.367 0.462 0.418 -0.137 -0.194 0.224

BI891349 0.915 1.122 1.248 0.596 0.443 0.446 0.045 0.191 -0.052 -0.714 -0.454 -0.534

BI891355 -0.346 -0.222 -0.061 0.035 0.123 0.947 0.515 0.775 0.616 0.633 0.151 0.421

BI891411 0.446 0.646 0.753 0.547 0.646 0.515 0.382 0.295 0.054 -0.218 -0.245 -0.296

BI891423 -0.526 -0.081 0.793 0.609 0.976 1.246 0.653 1.158 0.406 0.7 0.264 0.424

BI891429 -0.367 0.558 0.659 0.98 1.101 0.506 0.479 0.83 0.23 -0.048 -0.066 -0.223

BI891434 -0.854 -1.878 -0.893 -0.836 -0.581 0.393 0.187 0.493 0.308 1.204 0.915 0.388

BI891455 0.758 0.232 0.409 0.789 0.978 0.951 1.101 1.28 0.727 1.173 0.598 0.392

BI891472 -0.293 -0.688 0.925 0.692 0.935 1.083 0.425 0.733 0.658 0.261 0.471 0.146

BI891474 -0.476 0.126 1.735 0.384 1.426 0.708 0.321 1.024 0.427 0.358 0.085 -0.124

BI891492 -0.321 -1.729 0.917 1.394 1.885 1.036 1.3 1.67 0.634 0.804 0.708 0.639

BI891493 -0.326 0.14 0.183 0.539 0.653 0.157 0.159 0.54 0.319 0.132 -0.248 -0.185

BI891521 -0.555 -0.554 0.597 1.466 1.253 -0.131 0.318 0.694 0.135 0.155 0.6 0.481

BI891527 0.57 -0.035 0.509 0.757 1.159 0.208 0.544 0.963 0.325 -0.003 0.006 -0.434

BI891552 -0.658 -0.202 -0.013 -0.591 0.062 0.095 0.034 0.153 0.638 0.058 1.013 0.678

BI891570 0.407 0.303 0.967 0.997 0.924 0.221 0.556 1.14 0.415 0.553 0.134 -0.183

BI891591 0.216 0.04 0.051 0.33 0.239 1.002 0.41 0.968 0.413 0.35 0.725 0.198

BI891592 -0.541 0.615 0.074 0.469 0.594 0.098 -0.19 0.222 -0.068 -0.203 -0.352 0.084

BI891596 -0.679 0.306 0.506 1.402 1.64 0.831 1.028 2.391 0.936 0.054 -0.149 -0.387

BI891608 0.458 1.304 1.193 1.447 1.035 0.793 0.284 0.46 -0.135 -0.102 -0.063 -0.526

BI891613 0.668 0.117 1.86 0.887 1.113 1.343 0.571 1.131 0.586 0.333 0.101 -0.096

BI891643 1.248 0.456 -0.609 -0.271 -0.207 0.426 0.166 -0.193 -0.534 -0.862 -1.31 -2.089

BI891665 0.384 0.727 0.759 1.22 1.061 0.744 0.895 1.434 0.198 0.234 -0.023 -0.017

BI891666 -0.728 -0.147 0.656 1.377 1.101 -0.147 0.31 0.848 0.298 -0.251 -0.038 0.133

BI891674 0.229 -0.449 -0.141 0.33 1.577 1.452 1.086 2.633 1.606 0.478 0.247 0.044

BI891681 -1.3 -1.649 -1.081 -0.119 0.28 -0.09 0.571 0.844 0.594 0.77 0.299 0.542

BI891684 0.64 0.509 1.25 1.796 1.729 1.798 1.338 1.521 1.137 0.57 0.149 -0.447

BI891689 0.903 0.318 0.182 0.491 0.362 1.008 0.757 1.416 0.441 0.385 0.053 -0.408

BI891699 0.075 0.496 1.583 1.297 1.078 1.085 0.883 0.29 0.295 0.342 0.044 0.396

BI891704 -0.049 -0.406 -0.1 0.906 1.283 1.188 1.284 1.609 1.022 1.259 0.302 0.145

BI891709 -0.956 -0.348 0.556 1.394 1.888 2.097 2.11 2.502 1.283 1.252 0.511 0.421

BI891752 -0.503 0.089 1.557 1.099 0.615 0.683 -0.149 -0.307 -0.536 -0.482 -0.378 0.322

BI891754 -0.405 -0.111 0.374 0.708 1.411 -0.042 0.046 0.409 0.044 -0.133 0.297 0.275

BI891768 -0.24 0.184 1.556 1.377 1.567 0.938 0.89 1.853 0.48 0.635 0.29 -0.294

BI891769 -0.88 -2.729 -1.832 -1.288 -0.963 0.421 0.146 0.206 0.299 0.784 0.576 0.042

BI891773 -0.828 -0.697 1.44 1.516 3.962 3.15 1.954 2.415 1.74 0.11 -0.042 -0.386

BI891793 -0.728 -0.335 2.14 1.682 2.526 2.648 2.007 2.011 1.134 0.097 0.566 0.387

BI891804 -2.112 -1.02 -0.426 -0.228 -0.004 -0.159 -0.345 -0.585 0.409 -0.315 0.343 0.283

BI891821 1.695 0.27 -0.176 -0.463 -1.209 -0.806 -1.325 -2.47 -1.858 -2.457 -1.788 -2.243

BI891827 0.898 0.768 -1.067 -1.019 -0.5 0.111 -0.361 -0.488 -0.962 -0.929 -0.778 -0.124

BI891855 -1.777 -2.561 -0.856 -0.943 -0.315 -0.441 -0.043 -1.211 0.222 -0.253 0.396 0.13

BI891858 0.236 0.094 0.466 0.746 1.215 0.408 0.331 0.499 0.147 0.291 -0.044 -0.101

BI891859 -0.106 0.101 0.33 0.542 0.575 0.437 0.065 0.011 0.306 -0.574 -0.437 -0.347

BI891860 0.245 -0.126 1.259 1.444 1.464 0.812 1.385 1.597 1.146 0.707 0.751 0.587

BI891871 0.828 0.401 -0.383 -0.227 0.364 0.415 -0.194 -0.043 -0.659 -0.762 -0.75 -0.56

BI891877 0.443 0.341 1.468 1.753 1.852 1.587 1.372 1.382 0.841 0.683 -0.064 -0.819

BI891905 0.152 0.667 0.387 0.552 1.389 0.656 0.551 1.309 0.268 0.125 -0.352 -0.454

BI891913 0.947 0.334 1.316 0.761 0.966 1.184 0.791 1.014 0.328 0.406 -0.556 -0.818

BI891936 1.309 0.49 0.515 -0.145 -0.266 -0.111 -0.672 -1.057 -1.035 -0.098 0.051 -0.861

BI891948 -0.474 -0.088 3.268 3.516 3.885 2.822 2.563 4.233 2.387 1.651 0.537 -0.247

BI891978 1.221 0.845 0.38 0.241 0.11 0.28 -0.204 -0.165 -0.284 -0.536 -0.849 -0.824

BI891984 -0.299 -0.03 2.326 2.165 2.287 0.248 0.46 1.084 0.32 0.164 0.053 -0.208

BI891993 -0.097 0.711 1.056 1.39 1.062 0.819 0.453 0.938 0.231 0.142 -0.414 -0.255

BI892022 0.249 1.378 0.928 0.99 0.237 0.599 0.047 -0.022 0.009 -0.298 -0.148 -0.509

BI892036 -0.808 -0.22 0.73 1.577 1.897 1.264 0.113 -0.091 -0.274 0.572 0.902 -0.134

BI892038 -0.43 0.435 0.69 0.799 0.791 0.562 0.175 0.695 0.193 0.045 -0.05 -0.187

BI892041 0.439 0.116 0.777 1.193 1.561 1.592 0.479 0.72 0.077 0.495 0.274 0.565

BI892054 -0.039 0.818 1.396 1.119 0.747 0.339 -0.016 0.745 0.134 0.479 0.371 0.578

BI892060 0.16 -0.133 0.757 1.039 1.941 0.74 0.677 1.356 0.398 0.897 0.392 -0.254

BI892066 -0.363 -0.414 0.556 0.342 0.895 0.883 0.554 0.57 0.332 0.306 0.023 0.023

BI892068 -0.234 0.46 1.047 2.446 3.409 0.9 1.05 0.813 0.134 -0.122 0.064 0.12

BI892074 -1.096 -1.388 -1.078 -0.567 -0.569 -0.198 -0.292 -0.064 0.108 0.743 0.574 0.799

BI892082 0.211 0.072 0.36 0.99 0.697 0.558 0.798 1.212 0.3 0.407 -0.164 -0.109

BI892084 0.71 1.288 1.955 1.846 1.246 1.206 0.762 0.843 0.27 0.466 0.12 0.079

BI892100 -0.454 -0.31 -0.4 -1.317 -0.335 0.121 0.202 1.495 0.386 0.402 0.552 0.297

BI892110 -0.496 -0.892 1.503 2.679 2.476 0.098 1.275 1.143 1.483 0.757 -0.012 -0.173

BI892128 -0.5 0.16 1.673 1.781 2.075 1.717 0.788 2.028 0.449 -0.276 0.102 0.198

BI892134 -0.265 0.443 0.555 0.226 0.768 0.073 0.184 1.085 -0.059 0.162 -0.417 -0.216

BI892135 -0.199 0.747 2.318 1.786 1.74 2.348 1.313 1.145 0.584 0.461 -0.082 -0.135

BI892136 -0.429 1.513 4.304 2.872 2.969 1.261 0.449 0.97 -0.184 -0.333 0.03 -0.542

BI892151 -0.482 0.739 0.814 1.05 1.093 0.584 0.325 1.247 0.461 0.128 0.047 -0.341

BI892155 -0.319 -0.307 1.614 2.161 2.241 2.704 2.01 2.167 1.038 0.79 0.355 0.414

BI892167 -0.592 0.33 1.084 2 1.776 1.112 0.94 3.149 2.268 1.455 0.915 -0.497

BI892172 0.962 0.185 0.573 0.837 1.536 1.242 1.606 2.258 0.978 0.816 0.33 -0.142

BI892176 0.513 0.527 0.17 0.424 0.305 0.068 0.198 0.792 0.181 0.567 0.08 -0.256

BI892197 0.278 0.613 0.72 0.518 0.117 -0.137 -0.066 -0.056 0.085 -0.022 -0.383 -0.513

BI892200 -0.823 -0.459 0.243 1.066 1.667 1.094 1.022 1.034 0.141 0.534 0.644 0.035

BI892201 0.341 0.247 0.338 0.293 0.783 1.176 0.894 1.006 0.663 0.099 0.161 -0.127

BI892210 -2.439 -2.367 -0.129 0.23 1.147 1.659 1.434 1.532 1.332 1.308 0.993 1.009

BI892229 0.4 0.507 0.376 0.239 0.312 0.934 0.358 0.741 0.351 0.312 -0.172 -0.462

BI892237 0.553 0.465 1.344 0.675 1.218 1.726 0.913 0.896 0.359 0.355 -0.2 -0.032

BI892244 -0.513 -0.056 2.232 3.232 3.987 2.062 1.974 1.669 1.208 0.137 0.572 0.044

BI892250 -0.427 0.369 0.261 0.554 0.512 -0.113 -0.075 -0.083 0.185 0.044 -0.147 -0.282

BI892254 1.518 -0.237 -1.226 -1.026 -1.213 -0.7 -0.777 -1.399 -1.383 -1.871 -1.389 -1.663

BI892263 -0.754 -0.429 1.008 1.416 1.222 1.511 1.547 1.361 1.575 1.399 0.875 0.259

BI892272 -1.568 -1.744 0.544 1.253 1.566 1.288 1.045 0.142 1.384 1.615 0.99 1.425

BI892293 1.201 -3.872 -3.16 -2.827 -4.032 -2.986 -2.674 -4.719 -3.465 -4.145 -4.836 -5.033

BI892299 -0.595 0.144 0.455 0.485 0.705 0.341 -0.026 0.397 0.506 0.429 0.67 0.686

BI892323 -0.688 0.023 -0.257 0.037 0.455 0.16 0.82 1.502 0.553 0.138 -0.229 -0.086

BI892349 -0.493 0.065 3.322 3.139 2.871 2.644 2.198 1.907 0.968 0.944 0.976 0.056

BI892353 1.811 -2.305 -2.942 -2.859 -3.706 -2.955 -3.06 -3.945 -3.735 -3.958 -4.792 -4.513

BI892384 0.18 0.329 1.129 1.039 1.26 1.013 1.474 1.665 1.04 0.749 0.208 0.06

BI892406 -0.149 0.355 1.499 0.908 1.684 1.464 0.716 1.096 0.401 0.362 -0.048 0.014

BI892407 -0.244 -0.259 -0.318 0.121 0.103 0.125 -0.058 0.435 0.101 0.469 0.588 0.103

BI892410 -0.12 1.219 0.658 1.727 2.12 0.401 0.43 1.208 -0.043 -0.321 0.133 -0.039

BI892430 -0.446 -2.368 -0.921 -0.77 -0.351 0.508 0.28 0.356 0.181 0.988 0.587 0.065

BI892431 0.001 0.348 0.383 0.528 0.428 -0.117 0.255 0.784 0.191 -0.035 -0.11 0.045

BI892444 0.454 0.01 0.712 0.792 1.718 0.442 0.731 1.949 0.543 0.718 -0.148 -0.313

BI896231 -1.151 -1.418 -0.402 -0.082 -0.078 1.014 0.593 0.812 0.682 0.824 0.501 0.029

BI896233 -1.099 -1.381 -1.669 -1.194 -1.558 -0.638 -1.261 -1.456 -0.61 1.129 1.034 0.809

BI896246 -1.362 -2.14 -2.257 -1.651 -1.44 -0.442 0.131 0.4 0.193 0.946 0.858 0.117

BI896301 -0.041 0.453 0.4 0.072 -0.517 0.063 -0.132 -0.453 0.299 0.193 1.019 0.067

BI896347 -0.793 -0.996 -0.574 -0.292 -0.132 1.003 0.675 1.373 1.15 0.863 0.847 0.248

BI896418 -1.978 -1.482 -2.546 -1.598 -1.638 0.279 0.624 1.209 1.117 1.881 0.983 1.089

BI896473 0.104 -0.597 0.284 0.22 0.101 -0.224 0.19 0.461 0.32 0.775 0.596 -0.011

BI896491 0.739 0.452 -0.195 0.376 0.056 0.699 0.789 0.08 -0.16 -0.439 -0.877 -1.314

BI896504 -0.896 -2.379 -1.221 -0.962 -0.551 0.671 0.328 0.516 0.381 1.259 0.912 0.405

BI896532 0.907 -0.978 -0.959 -0.592 -1.232 -0.706 -0.743 -1.317 -0.789 -0.818 -0.737 -0.389

BI896959 0.985 -0.166 -0.147 -0.329 0.171 0.2 0.134 0.68 0.297 0.448 0.598 0.387

BI897147 -0.668 0.222 1.372 1.041 0.628 0.21 0.296 -0.368 0.868 0.151 0.477 -0.879

BI897366 0.309 1.495 1.65 1.644 1.004 0.332 0.296 0.971 0.309 0.426 -0.05 -0.125

BI897414 -0.304 0.048 -1.529 -0.56 -1.089 -0.538 -0.372 -0.447 -0.335 0.513 0.123 0.238

BI897419 1.41 0.658 -1.211 -1.051 -1.615 -0.601 -1.016 -0.902 -0.962 -0.872 -0.097 -0.79

BI897492 0.265 0.189 0.567 0.529 0.333 0.648 0.396 0.855 0.599 1.038 0.562 0.416

BI979064 -0.218 -0.288 -0.129 -0.046 -0.31 0.021 0.185 0.941 0.209 0.108 0.337 0.199

BI979115 -0.03 0.334 0.46 0.33 0.519 0.616 0.227 0.613 0.145 -0.044 -0.233 -0.049

BI979228 1.411 -0.003 -0.13 0.045 -0.379 -0.326 -0.09 -0.698 -0.198 -0.962 -0.248 -0.574

BI979286 2.197 -1.095 -1.643 -0.716 -0.504 -0.391 -0.732 -0.751 -0.786 -1.752 -0.744 -1.55

BI979304 1.243 1.111 0.957 1.055 0.715 0.484 0.235 0.77 0.15 0.232 -0.186 -0.215

BI979438 1.405 -0.673 -0.988 -0.898 -0.959 -0.348 -0.849 -1.193 -0.584 -1.163 -1.127 -0.981

BI979451 0.071 0.36 0.395 0.587 0.775 0.232 0.498 0.795 0.091 0.041 -0.14 -0.056

BI979549 1.259 -0.418 -0.356 -0.525 -0.183 -0.375 -0.029 0.462 0.078 -0.1 0.063 -0.251

BI979556 1.458 -0.941 -0.303 0.789 0.95 0.12 -0.079 -0.214 -0.294 -0.544 -0.658 -0.748

BI979581 -0.817 -1.189 1.126 1.87 2.524 0.791 1.075 2.093 1.796 -0.808 -0.988 -1.127

BI979759 1.308 1.806 2.13 1.881 1.702 1.409 1.129 1.755 0.542 0.785 0.287 -0.199

BI979865 1.697 1.652 0.287 0.353 -0.658 -0.43 -0.53 -0.587 -0.578 -0.951 -0.981 -0.682

BI979883 0.493 0.208 0.8 0.961 0.405 0.757 0.58 1.203 0.784 1.162 0.668 0.254

BI979955 1.272 1.184 0.859 1.2 1.062 1.67 0.316 1.025 0.172 0.38 0.043 0.215

BI979957 1.103 0.937 -0.257 -0.242 -0.151 0.451 0.362 0.827 0.042 0.166 -0.437 -0.27

BI979961 1.574 0.613 -0.487 -0.685 -0.075 -0.045 -0.37 -0.494 -0.29 0.006 -0.507 -0.308

BI979975 2.343 -2.442 -1.813 -1.49 -1.68 -0.991 -0.545 -1.417 -1.164 -0.867 -1.937 -2.565

BI979995 -0.058 1.458 0.824 0.292 0.302 -0.05 -0.293 0.046 -0.375 -0.32 -0.47 -0.592

BI980026 1.287 -2.131 -2.184 -2.514 -3.578 -2.029 -2.416 -4.168 -3.162 -3.11 -4.399 -3.453

BI980047 0.476 1.218 0.803 0.856 0.721 -0.168 0.24 0.581 -0.108 0.235 -0.371 -0.312

BI980084 1.171 0.766 -0.344 -0.072 0.09 0.474 -0.002 0.132 -0.293 0.002 -0.35 -0.898

BI980132 0.124 0.716 0.717 1.007 0.796 -0.056 -0.122 0.154 0.12 -0.187 -0.348 -0.708

BI980138 0.342 -0.22 0.086 0.407 0.679 1.142 0.413 0.762 0.211 0.268 -0.013 -0.171

BI980148 0.523 0.29 0.583 1.658 2.178 1.664 1.765 1.579 0.88 -0.028 0.109 -0.191

BI980160 1.132 0.971 0.026 -0.382 -0.848 -0.236 -0.409 -0.34 -0.393 0.324 -0.146 -0.402

BI980180 -0.167 0.156 0.041 -0.825 -0.706 -0.247 -0.331 -0.407 -0.323 0.325 0.845 0.402

BI980199 1.088 -0.02 -0.277 -0.002 0.032 0.042 0.224 0.874 0.194 0.586 0.07 0.153

BI980217 -0.122 0.711 1.383 1.513 1.35 1.022 1.272 1.236 0.595 0.919 0.372 0.591

BI980223 1.081 0.771 -0.267 0.057 -1.244 -0.174 -0.213 -0.973 -0.673 -0.379 -1.099 -0.473

BI980224 1.625 1.308 2.334 1.236 1.695 2.59 2.08 2.035 1.458 0.827 0.402 -0.5

BI980311 0.263 1.871 1.294 0.481 0.13 -0.209 -0.392 -0.045 0.032 -0.494 -0.14 -0.497

BI980382 2.253 2.013 0.884 -0.582 -1.17 -0.77 -0.566 -1.708 -0.784 -1.789 -1.242 -1.611

BI980396 1.328 -0.851 -0.998 -0.453 -1.056 -0.845 -0.171 -1.374 -0.31 -1.038 -0.479 -0.437

BI980436 2.555 -2.223 -3.135 -2.456 -3.163 -1.873 -2.613 -4.349 -2.714 -3.946 -4.395 -3.267

BI980448 -0.396 0.227 0.521 0.71 0.554 1.321 0.647 1.247 0.77 1.1 0.713 0.427

BI980457 0.65 1.106 0.657 -0.158 -0.941 0.068 0.236 -0.876 -0.279 -0.306 -0.834 -0.506

BI980640 0.891 -0.66 -1.584 -1.172 -1.825 -1.098 -0.914 -1.637 -1.174 -2.28 -2.366 -1.978

BI980644 1.837 0.896 2.001 0.889 0.66 0.612 0.411 0.571 -0.091 -0.731 -0.628 -1.054

BI980715 0.776 -0.191 -0.155 -0.845 -0.413 -0.052 -0.063 0.18 -0.198 -0.354 -0.38 -0.005

BI980725 0.123 -0.522 0.042 0.58 0.671 0.29 0.204 0.63 -0.117 0.308 0.025 0.45

BI980747 1.866 0.554 -0.143 0.386 0.682 1.054 0.498 0.917 0.325 0.183 -0.054 -0.393

BI980758 0.345 -0.078 -0.066 0.271 0.216 0.69 0.392 0.048 0.427 -0.018 0.351 0.606

BI980764 -0.155 0.177 0.512 1.124 1.499 0.547 0.487 1.113 0.153 -0.273 0.054 -0.031

BI980786 0.329 0.453 0.882 0.881 0.463 0.468 0.202 0.52 0.189 0.243 0.001 -0.178

BI980800 -0.433 -0.441 -0.095 1.71 0.735 0.844 0.375 0.905 0.537 0.931 0.911 0.806

BI980805 0.181 -0.02 0.519 0.747 1.561 0.442 0.294 1.039 0.064 0.033 -0.231 0.03

BI980843 -0.107 0.185 2.232 1.995 2.032 1.509 0.835 1.257 0.229 0.532 0.493 0.504

BI981066 -0.838 -0.802 -0.963 -0.398 0.004 -0.106 -0.461 -0.511 -0.206 -0.247 0.519 0.597

BI981133 1.357 0.695 -1.484 -0.479 -0.896 -0.171 -0.47 -0.936 -0.467 -0.535 -0.78 -0.757

BI981134 0.03 0.417 0.278 0.54 0.694 -0.077 0.225 0.14 0.037 0.077 -0.111 -0.037

BI981135 -0.642 0.545 0.362 0.61 0.599 0.225 -0.07 1.06 -0.157 0.07 -0.329 0.32

BI981429 -0.165 -1.342 -1.079 0.012 -0.126 0.05 0.472 0.7 0.524 0.901 0.585 0.451

BI982030 0.113 0.211 -0.6 -0.241 0.034 0.877 0.399 0.846 0.739 1.2 0.643 0.558

BI982117 -1.596 -1.039 -0.707 -0.044 -0.099 -0.255 -0.648 -0.977 0.426 -0.397 0.661 0.576

BI982141 -0.121 -1.062 1.356 1.03 1.666 1.508 0.761 -0.324 0.066 0.517 0.629 -0.309

BI982718 -0.198 -1.867 -0.564 -0.64 -0.364 0.475 0.295 0.742 0.324 1.369 1.053 0.211

BI982770 -0.661 -0.359 -0.402 -0.829 -0.266 0.203 0.145 1.147 0.423 0.501 0.769 0.463

BI982778 -1.391 -0.567 0.017 -1.212 -1.817 0.09 -1.219 -1.575 -0.847 0.299 0.768 1.895

BI982877 0.718 0.142 0.147 0.316 -0.103 0.168 0.114 0.544 -0.035 -0.054 1.038 0.06

BI982951 -0.739 -1.111 -0.897 -0.904 -1.172 -0.62 -0.674 -1.239 -0.474 -0.269 -0.019 0.623

BI983014 0.041 0.509 1.625 1.424 1.195 0.767 1.079 0.691 0.039 0.53 0.065 0.009

BI983027 1.071 0.487 0.071 -0.152 0.026 -0.063 -0.14 0.719 0.173 0.525 0.433 -0.267

BI983044 0.213 0.09 0.031 0.264 0.432 1.123 0.591 0.591 0.466 0.271 0.013 -0.191

BI983233 0.869 0.101 -1.359 -0.739 -0.343 -0.33 -0.272 -0.271 -0.368 -0.063 -0.307 0.136

BI983256 -0.301 1.148 0.74 1.059 0.034 -0.249 -0.324 -0.597 -0.124 -0.315 -0.601 -0.454

BI983343 1.452 1.414 2.103 1.472 0.111 -0.271 -1.308 -1.384 -1.904 -2.021 -2.775 -2.796

BI983370 1.076 0.901 1.388 1.398 1.529 1.391 0.869 0.903 0.07 0.323 -0.203 -0.235

BI983375 0.644 1.354 1.386 0.665 0.79 0.12 0.316 1.031 0.216 0.305 0.432 -0.162

BI983378 0.832 0.685 0.463 0.348 -0.026 -0.009 0.041 -0.348 -0.343 -0.041 -0.616 -0.137

BI983379 0.159 -1.579 -0.742 -0.456 -0.52 0.342 0.193 0.212 0.116 1.037 0.615 0.161

BI983442 0.958 0.372 -0.113 0.165 -0.143 -0.078 -0.313 -0.066 -0.142 -0.305 -0.203 -0.245

BI983488 0.226 1.02 1.233 0.908 0.801 0.687 0.156 0.807 0.111 -0.197 -0.209 -0.223

BI983566 0.912 0.675 -0.152 -0.093 -0.204 -0.244 -0.241 -0.09 -0.256 -0.014 -0.164 -0.282

BI983579 -0.048 -0.111 1.708 1.68 2.141 2.049 1.264 1.447 0.999 0.845 0.337 -0.126

BI983582 1.387 0.333 0.101 0.085 -0.004 0.112 -0.192 0.652 0.015 0.846 0.516 0.379

BI983593 0.341 0.295 0.603 0.994 0.822 0.859 0.729 0.56 0.073 0.398 -0.06 0.489

BI983596 1.209 -0.214 0.044 0.184 0.138 0.381 -0.137 -0.03 -0.045 -0.163 -0.228 -0.299

BI983651 1.396 1.544 1.019 0.72 0.348 -0.089 -0.302 0.143 -0.147 -0.334 -0.395 -0.823

BI983753 1.028 0.47 1.03 0.53 0.09 0.127 -0.233 -0.351 -0.255 -0.268 -0.236 -0.368

BI983762 1.172 0.551 -0.422 -0.479 -0.084 0.061 -0.326 0.53 -0.178 0.088 -0.116 0

BI983850 1.134 0.815 0.121 0.695 0.6 1.244 0.303 0.323 0.403 0.249 0.202 0.357

BI983855 1.338 0.214 -1.19 -0.601 -0.205 -0.182 -0.5 -0.547 -0.939 -0.894 -0.71 -1.228

BI983894 1.685 1.184 0.172 0.077 0.215 0.279 -0.029 0.123 0.032 0.458 0.01 -0.371

BI984060 0.317 0.207 0.532 0.329 0.704 0.337 0.24 0.606 0.07 0.349 0.107 0.023

BI984272 0.076 0.185 0.275 0.575 0.859 0.136 -0.309 0.849 0.715 0.339 -0.075 0.034

BI984291 0.522 0.821 1.08 1.053 0.955 0.724 0.656 0.919 0.212 0.367 -0.221 -0.528

BI984380 0.336 0.393 0.042 0.314 0.728 0.079 0.524 0.916 0.076 0.334 -0.021 -0.151

BI984492 0.101 0.831 1.606 1.407 0.746 0.945 0.546 0.591 0.112 -0.313 -0.364 -0.55

BI984734 1.395 -0.794 -0.827 -0.576 -1.421 -0.517 -0.911 -1.439 -0.483 -1.081 -0.857 -0.721

BI984759 0.009 0.177 1.007 0.867 0.806 0.65 0.257 0.317 0.259 0.253 0.069 0.12

BI984775 0.018 1.323 0.712 0.508 -0.903 -0.548 -0.922 -1.145 -0.459 -0.642 -1.024 -0.775

BI984810 -0.074 0.201 0.082 0.499 0.998 0.011 0.125 0.966 0.239 0.298 0.209 0.187

BM004967 0.139 0.479 0.187 0.056 0.033 0.512 0.366 1.108 0.384 0.724 0.381 0.963

BM005035 0.437 0.544 0.524 0.834 0.893 -0.123 0.279 0.555 -0.015 0.059 -0.14 -0.296

BM005077 0.548 0.077 -0.136 0.011 0.43 0.759 0.226 0.135 0.439 0.344 0.374 0.441

BM005197 0.677 0.44 0.449 0.79 0.746 -0.02 0.187 0.588 -0.066 0.021 -0.436 -0.322

BM005412 1.63 1.116 -0.649 -0.522 0.022 0.276 -0.551 -0.089 -0.296 -0.241 -0.574 -0.584

BM005437 1.682 -1.012 -1.051 -1.244 -1.034 -0.516 -0.917 -0.567 -0.622 -0.602 -0.728 -0.291

BM005469 -0.258 -0.587 -1.047 -0.043 0.128 0.295 0.038 0.637 0.098 0.766 0.496 0.333

BM023676 0.178 1.272 1.636 1.504 1.584 0.229 0.367 0.833 0.066 0.089 -0.107 -0.045

BM023680 -0.011 -0.236 0.462 1.358 1.445 0.885 0.87 0.526 -0.024 -0.026 -0.283 0.058

BM023720 -0.237 -0.23 0.489 0.625 1.111 0.593 0.643 0.786 0.472 0.056 -0.344 -0.077

BM023784 0.268 0.468 -0.161 0.068 0.262 0.487 0.363 1.108 0.378 0.4 0.293 0.112

BM023958 -0.612 -1.373 -1.264 -1.406 -0.673 -0.091 -0.37 -0.08 -0.425 0.561 0.106 0.318

BM024150 -0.728 -0.193 -0.153 -0.574 -0.83 -0.137 -0.193 0.637 0.338 0.794 1.079 0.533

BM024193 0.444 0.525 0.596 0.558 0.637 0.147 0.214 0.254 -0.121 0.145 -0.212 -0.007

BM024216 -0.087 -0.775 -0.118 0.3 0.266 0.288 0.618 0.901 0.804 0.946 0.452 0.033

BM024326 -0.33 0.224 0.359 0.343 0.412 0.2 0.211 0.61 0.319 0.167 0.916 0.537

BM024634 1.69 0.61 0.759 0.533 0.077 0.728 -0.066 0.093 -0.329 -0.294 -0.446 -0.242

BM024640 0.374 -0.03 0.378 0.634 0.651 0.312 0.585 0.874 0.09 0.459 0.351 0.272

BM024812 0.947 -0.091 -0.823 -0.698 -0.627 -0.244 -0.709 -0.673 -0.527 -0.388 -0.179 0.873

BM024816 0.873 -0.395 -0.282 -0.484 -0.141 0.15 -0.105 0.016 -0.152 0.054 0.224 0.166

BM025184 -0.295 -1.02 0.009 1.676 2.839 0.877 1.28 1.111 0.781 -0.187 0.102 0.009

BM025541 -0.387 -1.141 0.066 0.303 -1.97 -0.468 0.01 0.63 0.599 0.793 0.499 0.075

BM025866 0.014 0.147 0.154 0.431 0.999 0.036 0.455 1.55 0.297 0.729 0.231 0.056

BM025872 0.401 0.677 0.816 0.557 0.67 0.339 0.062 0.843 -0.133 0.221 -0.081 -0.067

BM026015 -1.111 -0.52 0.31 0.312 0.711 -0.183 -0.431 -0.171 0.773 0.401 1.282 0.924

BM026032 0.384 0.164 0.846 0.284 0.554 0.438 0.193 0.89 0.207 0.249 -0.031 -0.132

BM026053 1.182 -0.528 -1.49 -1.045 -0.603 -0.526 -0.765 -1.58 -1.021 -1.254 -1.334 -1.487

BM026057 0.237 0.232 0.688 0.415 0.461 0.258 0.263 0.55 -0.209 -0.097 -0.291 0.042

BM026121 -0.106 0.414 1.115 1.333 1.537 0.359 0.605 1.261 0.315 0.177 0.198 -0.005

BM026308 -0.463 0.508 0.31 1.229 1.628 1.304 0.913 1.87 0.154 0.33 0.135 -0.153

BM026316 0.367 0.518 0.407 -0.014 0.108 0.602 0.459 0.746 0.102 0.154 -0.219 0.032

BM026429 0.237 0.112 0.195 0.334 0.646 0.694 0.352 1.212 0.319 0.463 0.054 0.085

BM026462 0.725 1.134 1.362 1.084 0.783 0.764 0.214 0.712 0.349 -0.148 -0.062 -0.662

BM026470 -0.16 -0.107 0.154 0.305 0.001 0.227 0.061 0.657 0.405 0.54 0.594 0.256

BM026491 -0.836 -0.599 -0.53 -0.134 0.832 -0.044 -0.411 -0.411 -0.091 -0.231 0.777 0.575

BM026607 -0.296 -0.288 -0.976 -1.154 -1.704 -0.211 0.151 -0.756 -0.819 0.199 0.097 1.465

BM026830 -0.583 0.145 0.397 2.439 3.626 0.064 1.179 0.172 0.121 -0.674 0.009 -0.156

BM026841 -0.407 -0.573 -0.384 -0.572 0.089 -0.034 -0.002 -0.297 0.392 0.518 0.694 0.324

BM026879 1.688 -0.811 -0.595 -0.936 -0.767 0.01 -0.175 -0.672 -0.48 -0.315 -0.621 -0.358

BM034949 0.873 1.219 0.564 0.576 0.562 0.032 -0.179 0.077 -0.201 -0.177 -0.249 -0.373

BM034969 0.851 1.274 1.297 1.516 0.881 1.472 0.591 0.867 0.534 0.253 0.044 -0.088

BM034983 0.245 0.51 0.16 0.251 0.304 0.635 0.495 1.312 0.202 0.273 -0.363 -0.348

BM035036 -0.873 -1.719 -1.64 -0.908 -0.768 -0.007 -0.236 0.114 0.056 0.59 0.245 -0.382

BM035055 1.027 0.839 -0.566 -0.225 -0.46 -0.043 -0.043 -0.062 0.03 -0.083 -0.462 -0.281

BM035348 0.116 -1.108 0.077 -0.329 -1.14 -0.031 -0.115 -1.005 -0.209 -0.023 0.057 1.258

BM035368 2.385 0.61 -0.55 -2.382 -2.723 -1.072 -1.127 -2.802 -1.128 -1.942 -2.409 -2.345

BM035394 1.498 0.055 -0.212 -0.726 -0.173 0.041 -0.357 -0.166 -0.284 -0.585 -0.443 -0.286

BM035545 1.044 1.33 1.427 1.049 0.285 0.425 0.043 0.491 -0.087 -0.826 -0.336 -0.474

BM035598 2.406 1.217 -0.192 -0.935 -1.439 -0.536 -0.677 -1.384 -0.632 -0.288 -1.305 -0.818

BM035613 1.047 0.674 0.238 -0.357 -0.087 0.058 -0.345 -0.044 0.099 -0.827 -0.645 -0.139

BM036098 -0.328 0.981 0.712 0.495 -0.475 -0.324 -0.324 -0.399 -0.472 -0.731 -0.686 -0.621

BM036121 0.889 1.196 0.639 0.802 0.167 0.13 -0.109 -0.308 -0.345 -0.628 -0.326 -0.236

BM036281 1.025 1.646 0.694 0.33 0.148 0.183 0.035 0.286 -0.323 -0.357 -0.312 -0.374

BM036297 -0.107 -0.63 0.247 0.194 0.572 0.37 0.29 0.74 0.261 0.232 -0.027 0.016

BM036347 0.817 -0.151 -0.072 0.742 0.196 0.062 -0.087 0.327 0.067 -0.233 0.003 -0.243

BM036392 0.016 -0.904 -0.603 -0.225 -0.23 0.338 -0.104 0.197 -0.07 0.565 0.267 0.228

BM036406 0.86 0.689 0.485 0.64 0.556 1.038 0.526 1.01 0.436 0.146 -0.294 -0.736

BM036413 0.545 1.391 1.094 0.395 0.601 0.753 0.745 1.372 0.845 0.579 0.5 -0.044

BM036431 0.826 -0.097 0.425 0.509 0.622 0.783 0.59 0.455 0.345 0.511 0.249 0.147

BM036445 1.236 0.187 -1.429 -0.687 -0.757 -0.161 -0.706 -0.469 -0.989 -0.617 -0.952 -1.336

BM036471 0.053 0.175 1.121 0.406 0.437 0.356 0.291 0.768 0.113 -0.333 -0.129 -0.089

BM036484 -0.542 0.214 0.61 0.705 1.008 -0.159 0.198 0.847 0.126 0.236 0.328 -0.111

BM036509 -0.832 -1.089 1.921 3.063 4.217 3.439 3.199 3.112 2.503 0.124 0.31 -0.36

BM036521 0.598 -0.516 -0.007 0.168 0.566 0.322 0.39 1.266 0.366 0.566 0.331 0.037

BM036771 1.151 1.204 1.008 1.409 0.206 -0.229 -0.516 -0.709 -0.598 -0.519 -0.562 -0.728

BM036795 0.201 0.782 1.001 1.14 1.233 0.437 0.368 0.76 0.021 0.178 0.082 0.236

BM036802 -0.049 0.341 1.187 1.578 1.378 1.27 1.273 1.853 0.782 0.559 -0.021 0.222

BM036823 0.737 1.155 -0.268 1.286 1.339 0.465 0.654 1.258 0.492 -0.003 -0.326 -0.453

BM036885 2.085 -0.061 -1.04 -0.072 -1.353 -0.358 -0.943 -0.564 -0.917 -1.82 -0.748 -0.862

BM036903 0.787 1.148 1.16 0.691 -0.365 -0.076 -0.177 -0.114 -0.264 0.1 -0.149 -0.672

BM036938 1.763 1.075 0.201 0.46 0.179 0.148 -0.287 -0.309 -0.504 -0.77 -0.513 -0.763

BM036954 -0.079 0.525 0.89 0.22 1.117 0.466 0.19 1.613 0.165 0.167 0.228 0.004

BM036958 1.564 1.427 0.243 0.102 -0.265 0.144 -0.232 -0.346 -0.454 -0.222 -0.985 -0.699

BM037178 2.43 0.07 -1.643 -0.831 -2.107 -1.446 -1.065 -2.789 -1.413 -2.629 -2.144 -2.326

BM037305 1.587 -0.089 0.256 0.269 0.452 0.476 0.162 0.584 0.257 0.499 0.272 -0.013

BM037469 1.409 1.968 2.456 1.735 1.479 1.078 0.706 0.696 -0.095 -0.237 -0.577 -0.98

BM037539 1.647 0.312 -0.54 0.438 -0.291 -0.135 -0.275 -0.73 -0.626 -0.684 -1.085 -0.88

BM070534 -0.495 0.627 0.749 1.081 0.86 -0.033 -0.123 0.473 0.198 0.16 0.089 0.069

BM070558 0.149 0.833 1.028 1.453 1.928 1.144 1.037 1.28 0.415 0.242 0.402 0.339

BM070561 1.404 -1.209 -0.984 -0.914 -0.989 -0.174 -0.536 -1.155 -1.098 -0.639 -0.884 -0.118

BM070584 0.472 0.084 0.808 1.137 1.148 0.909 1.101 1.174 0.728 0.618 0.28 0.301

BM070699 -0.087 -2.361 -1.068 -0.649 -0.569 0.561 0.153 0.48 0.383 1.152 0.897 0.251

BM070919 -0.585 -0.933 -0.588 -0.311 -0.646 -0.466 -0.338 -0.795 -0.292 0.041 0.098 0.752

BM070949 1.63 1.204 -0.386 -0.246 0.011 -0.179 -0.327 -0.443 -0.546 -0.523 -0.554 -0.562

BM071061 0.736 -0.437 -0.517 -0.418 -0.621 0.208 -0.226 -0.134 -0.285 0.31 0.065 0.118

BM071213 0.968 0.658 0.014 0.08 -0.03 0.114 -0.054 -0.266 -0.353 -0.494 -0.544 0.054

BM071225 -5.191 -4.481 -4.423 -3.382 -3.575 -3.213 -4.224 -4.817 -3.741 0.638 0.744 0.749

BM071290 -0.511 0.27 -0.122 -0.244 -0.725 -0.299 -0.344 0.321 0.262 0.939 0.862 0.359

BM071353 0.631 0.304 -0.085 -0.112 0.182 0.73 0.684 0.874 0.422 0.583 0.286 0.026

BM071664 1.219 -0.711 -0.3 -0.062 -0.018 0.763 0.333 0.808 0.287 0.709 0.259 -0.172

BM071679 0.378 1.01 1.705 1.556 1.776 0.869 0.902 1.281 0.464 0.71 0.131 0.122

BM071697 1.203 -0.592 -0.256 -0.185 0.196 0.501 -0.009 -0.028 -0.081 -0.385 -0.114 -0.368

BM071732 1.755 0.041 0.683 0.286 0.181 0.873 0.344 0.409 0.171 0.128 -0.02 -0.109

BM071784 0.269 1.345 0.706 0.336 -0.131 -0.115 0.073 0.035 -0.178 -0.157 -0.422 -0.355

BM071795 1.468 1.794 0.222 -0.353 -0.881 0.116 -0.711 -1.126 -0.948 -1.227 -2.103 -1.228

BM071805 2.427 0.296 -2.048 -3.703 -4.472 -0.848 -1.633 -4.037 -1.943 -2.593 -3.908 -2.017

BM071837 1.843 -0.709 -1.339 -0.549 -1.267 -0.661 -0.694 -1.425 -1.151 -1.484 -1.039 -1.029

BM071872 0.781 0.279 -0.563 -0.158 -0.91 -0.289 -0.611 -0.535 -0.591 -0.188 -0.198 -0.435

BM071941 0.111 0.051 -0.476 0.014 0.091 1.016 0.491 0.75 0.388 0.882 0.348 -0.128

BM072263 -0.124 -1.727 0.92 0.719 3.206 3.052 2.56 2.146 1.799 -0.001 -0.074 0.119

BM072333 -0.131 0.357 0.284 1.288 1.328 0.864 0.616 0.968 0.402 0.056 0.334 0.332

BM072353 0.473 0.54 0.742 0.924 1.4 0.864 0.862 1.348 0.505 0.48 -0.197 -0.567

BM072375 1.085 1.561 0 -0.003 -0.675 -0.276 -0.785 -0.536 -0.549 -0.604 -0.782 -0.626

BM080877 0.566 1.184 1.327 1.168 0.501 -0.22 0.378 0.086 -0.236 -0.665 -0.876 -0.668

BM080950 0.821 -0.045 0.31 0.987 1.3 1.168 1.092 1.536 0.418 0.954 0.699 0.347

BM081047 -0.507 -1.418 -1.065 -0.7 -0.595 -0.076 0.04 0.114 0.274 0.978 0.551 -0.143

BM081091 2.827 0.293 -1.323 -0.651 -1.403 -0.535 -0.272 -1.384 -0.942 -1.909 -1.218 -1.157

BM082387 1.16 0.723 -0.095 0.181 0.42 -0.105 0.295 -0.015 -0.04 -0.369 -0.186 -0.469

BM082431 0.336 0.063 0.503 0.535 0.481 -0.187 0.107 -0.123 0.079 0.096 -0.375 -0.016

BM082504 1.306 1.248 -0.539 -0.99 -0.905 -0.119 -0.551 -0.968 -0.516 -1.017 -0.687 -0.69

BM082684 -0.287 0.758 0.818 0.888 0.833 0.068 0.288 0.193 -0.191 -0.092 -0.069 0.045

BM082789 1.604 0.555 -0.173 -0.079 -1.14 -0.584 -0.727 -2.146 -1.103 -2.367 -1.677 -1.621

BM083940 0.243 -0.894 0.175 0.614 1.082 1.178 0.693 0.842 0.969 0.103 0.334 0.181

BM083944 0.127 0.415 0.645 0.595 1.022 0.341 0.286 0.556 0.076 0.378 0.251 0.158

BM083945 -0.678 0.306 0.34 0.776 1.097 0.217 0.422 1.076 0.525 -0.129 -0.106 -0.469

BM083952 0.007 0.279 0.091 0.712 0.771 0.315 0.659 0.954 0.399 0.31 0.298 -0.062

BM083966 -0.477 0.181 0.802 2.011 2.5 1.293 1.129 1.728 0.337 -0.074 0.132 0.9

BM095156 1.508 0.515 -0.459 -0.079 0.117 -0.039 -0.456 -0.531 -0.631 -1.408 -0.72 -0.992

BM095178 -0.112 0.016 -0.015 -0.14 0.352 0.171 0.092 0.885 0.154 -0.005 -0.271 -0.003

BM095242 2.041 -2.393 -1.749 -1.447 -3.338 -1.81 -0.938 -3.798 -1.378 -2.945 -3.005 -3.586

BM095259 1.481 1.427 0.047 -0.125 -0.249 0.168 0.048 0.959 0.358 0.059 0.155 -0.698

BM095268 1.114 0.207 -0.001 0.175 -0.405 0.317 -0.117 0.546 0.31 0.591 0.26 -0.174

BM095301 -0.154 0.759 0.557 0.237 1.036 0.136 0.004 0.493 0.182 0.344 0.137 -0.012

BM095334 0.884 1.033 -0.351 -0.224 -0.117 0.026 -0.021 0.506 0.134 -0.311 -0.323 -0.349

BM095365 1.303 0.251 -0.163 -0.389 -1.419 -0.022 -0.187 -0.909 -0.636 0.023 -0.224 -0.036

BM095366 0.922 0.83 0.77 -0.083 0.101 0.207 -0.121 0.264 -0.278 -0.265 -0.475 -0.456

BM095386 1.276 -0.616 -2.389 -1.217 -1.503 -0.652 -1.426 -1.984 -1.812 -2.887 -2.301 -2.227

BM095389 1.332 0.206 0.067 0.586 0.618 1.414 1.229 1.707 0.79 0.644 0.609 0.184

BM095390 1.815 -1.135 -1.669 -0.91 -0.334 -0.546 -0.63 -0.822 -0.752 -1.429 -0.538 -1.078

BM095392 0.094 0.029 -0.896 -0.481 -0.764 -0.064 -0.424 -0.148 -0.296 0.764 0.453 0.3

BM095404 1.65 -2.744 -2.535 -2.842 -3.273 -2.764 -3.46 -4.215 -4.317 -5.652 -5.952 -5.761

BM095417 1.59 1.44 0.048 0.473 0.168 0.242 -0.151 -0.094 -0.353 -0.111 0.199 -0.563

BM095484 1.631 -1.449 -2.099 -0.921 -2.802 -1.826 -1.071 -3.305 -1.783 -3.723 -2.218 -2.922

BM095607 2.51 -3.655 -4.336 -4.056 -3.689 -2.586 -4.015 -4.684 -3.918 -5.755 -3.732 -4.504

BM095657 1.823 -2.53 -2.688 -1.408 -2.738 -2.2 -2.089 -3.419 -2.033 -2.199 -2.313 -2.133

BM095658 0.051 0.468 0.681 1.006 1.262 0.988 1.074 1.444 0.576 1.005 0.601 -0.289

BM095746 -0.173 0.343 0.492 1.085 0.822 0.258 0.015 0.23 0.418 -0.009 0.918 0.043

BM095814 0.308 1.275 1.199 0.801 -0.11 -0.233 0.264 0.039 -0.162 -0.254 -0.44 -0.493

BM095815 1.551 -0.483 -2.051 -1.333 -1.737 -1.281 -1.167 -2.007 -2.196 -3.061 -2.458 -2.331

BM095845 2.021 0.705 -1.569 -1.068 -1.902 -0.949 -1.824 -1.418 -1.417 -2.774 -2.028 -1.939

BM095865 -0.281 0.525 3.001 1.331 0.999 0.209 0.606 0.222 -0.225 -0.328 0.032 -0.155

BM095868 -0.75 -0.797 -0.393 -0.154 0.532 -0.016 0.337 1.427 0.743 0.591 1.118 0.455

BM095877 1.301 2.068 2.347 1.552 0.302 -0.641 -0.914 -1.311 -1.177 -2.19 -1.225 -1.367

BM095884 -0.163 0.068 1.236 1.132 0.592 0.81 0.689 0.528 0.159 0.476 0.464 -0.018

BM095897 1.801 -0.725 -1.563 -1.432 -3.851 -1.629 -1.147 -3.404 -1.282 -2.887 -3.118 -3.259

BM095922 0.032 1.012 0.948 1.141 0.959 -0.115 0.101 0.668 -0.03 -0.191 -0.319 0.179

BM095989 1.346 1.088 0.119 0.621 0.395 1.02 0.646 0.763 0.435 0.216 0.033 -0.222

BM096012 1.686 1.358 -0.272 0.341 -0.233 -0.229 -0.231 0.07 -0.271 -0.687 -0.213 -0.409

BM096050 1.195 1.243 1.35 0.426 0.405 0.469 0.205 0.777 0.305 0.371 0.031 -0.245

BM096052 0.338 0.893 0.545 0.781 1.119 0.756 0.268 1.033 0.254 0.441 -0.164 -0.131

BM096064 0.957 0.912 1.315 1.776 0.976 0.866 0.77 0.945 0.435 0.23 0.035 -0.578

BM096076 -0.099 0.897 0.234 0.828 0.669 -0.269 -0.253 0.285 0.26 0.344 0.434 -0.119

BM096092 1.183 -0.95 -1.172 -1.49 -1.063 -0.299 -0.83 -1.005 -0.859 -0.751 -0.614 -0.636

BM096095 1.254 -0.568 -0.169 -0.211 -0.018 0.268 0.191 0.553 0.273 0.46 0.152 -0.431

BM096116 0.039 0.766 0.817 0.509 -0.707 -0.366 -0.167 -0.481 -0.091 -0.394 -0.326 -0.382

BM096428 1.663 -0.106 -0.15 0.369 0.187 0.411 0.225 0.713 0.12 0.39 -0.177 -0.287

BM096465 0.034 0.111 2.124 1.906 1.663 1.33 0.691 0.911 0.251 0.343 0.075 -0.037

BM101507 1.001 0.286 -0.512 0.066 -0.434 -0.289 -0.482 -0.278 0.025 0.04 -0.078 -0.106

BM101524 0.961 0.937 1.8 1.434 1.779 2.168 1.556 1.693 0.887 0.791 -0.197 -0.616

BM101527 2.104 -2.532 -3.325 -3.098 -3.153 -2.984 -3.368 -3.428 -3.074 -4.723 -4.244 -3.918

BM101531 -0.318 0.397 0.127 0.201 0.949 -0.039 0.046 1.014 0.273 0.331 0.236 0.204

BM101541 2.3 0.991 -0.337 -1.032 -1.346 -0.554 -0.865 -1.584 -1.161 -1.343 -1.559 -1.437

BM101574 0.822 -0.104 -1.582 -1.365 -0.929 -0.202 -0.73 -0.384 -0.348 -0.099 -0.133 -0.526

BM101576 0.215 1.364 1.021 0.846 0.56 -0.304 0.269 0.372 0.233 -0.376 -0.17 0.008

BM101584 1.151 -0.177 -0.146 0.184 0.186 -0.058 0.133 -0.083 -0.191 -0.308 0.055 -0.369

BM101590 1.507 -2.58 -1.698 -1.758 -2.787 -1.392 -1.451 -3.585 -2.01 -2.302 -3.691 -3.217

BM101600 1.385 1.222 0.363 0.667 0.584 0.217 0.263 0.312 -0.151 -0.196 -0.54 -0.204

BM101604 1.647 -0.051 0.031 -0.019 -0.477 -0.126 -0.356 -1.171 -0.381 -0.98 -0.467 -1.157

BM101621 1.379 1.582 1.018 0.03 -0.247 0.095 -0.171 -0.274 -0.155 -0.522 -0.36 -0.396

BM101651 1.225 1.091 1.942 1.807 1.705 2.256 1.988 2.148 1.337 1.087 0.439 -0.15

BM101666 1.005 0.751 -0.972 -0.462 -0.201 -0.419 -0.267 -0.756 -0.836 -1.141 -0.419 -0.707

BM101681 1.161 1.613 1.433 0.71 0.327 0.549 0.335 0.639 0.459 0.397 0.146 -0.319

BM101689 0.514 -0.129 0.086 0.194 0.879 1.226 0.403 0.597 0.725 0.483 0.677 0.466

BM101748 -0.418 0.236 0.359 1.428 1.839 0.325 0.58 1.186 0.205 -0.256 0.444 -0.262

BM101834 0.213 1 0.637 0.75 0.959 0.286 -0.143 0.359 0.459 -0.218 -0.204 -0.47

BM102022 -0.728 0.439 0.143 -0.535 -0.029 -0.161 -0.395 -0.114 -0.237 -0.545 0.402 1.501

BM102070 -0.146 -0.157 0.071 0.101 0.68 0.341 0.395 0.958 0.272 0.225 0.264 0.127

BM102093 -0.623 0.783 0.696 0.179 -0.452 -0.219 -0.473 -1.031 -0.08 0.353 0.721 0.5

BM102116 0.877 0.83 0.798 0.588 0.242 0.187 0.117 0.531 -0.06 -0.189 -0.125 -0.199

BM102195 -0.505 0.222 0.397 0.105 -0.343 0.336 -0.151 -0.203 -0.138 0.246 0.318 0.789

BM102223 -0.385 -0.25 0.431 0.075 -0.049 -0.136 0.124 -0.03 0.455 0.093 0.617 0.484

BM102339 0.093 0.778 0.866 1.235 0.683 0.285 -0.138 0.22 -0.323 -0.345 -0.437 0.27

BM102580 1.581 1.936 1.209 0.605 0.016 0.087 -0.022 0.012 -0.3 -0.197 -0.931 -1.174

BM102623 0.191 0.548 0.08 0.494 0.914 0.222 0.45 1.166 0.294 0.126 0.137 0.102

BM102635 0.275 -0.176 0.534 0.587 0.553 0.442 0.88 0.576 0.635 0.552 0.47 0.118

BM102647 -0.504 0.538 0.496 0.894 0.735 -0.175 0.137 0.848 0.131 0.199 0.062 0.147

BM102865 0.15 -0.587 0.36 0.586 1.163 0.423 0.676 1.277 0.374 0.305 -0.055 0.017

BM102872 0.207 0.21 0.893 0.293 0.483 0.693 0.095 1.446 0.681 0.428 0.236 0.04

BM103051 0.571 1.334 1.552 0.538 -0.805 -0.495 -0.388 0.262 -0.162 -0.167 -0.031 -0.656

BM103127 1.85 -0.53 -0.66 -0.796 -1.866 -0.903 -1.345 -2.272 -1.575 -0.732 -2.171 -1.894

BM103179 -0.068 0.604 1.426 1.578 1.132 0.69 -0.049 0.704 0.251 0.21 0.044 -0.12

BM103255 -0.553 -0.795 0.761 0.706 0.836 0.138 0.404 -0.153 0.458 0.168 -0.255 -0.722

BM103291 0.314 0.581 0.257 0.677 0.721 -0.127 0.151 0.644 0.142 0.09 -0.262 -0.221

BM103340 -1.107 -0.902 0.261 0.425 0.277 0.638 0.337 0.481 0.69 0.539 0.115 -0.329

BM103396 1.283 -0.743 -1.615 -0.851 -1.172 -0.452 -0.767 -0.8 -0.738 -0.36 -0.51 -0.081

BM103785 0.72 1.241 1.618 0.363 0.935 1.205 0.479 1.173 0.605 -0.015 -0.042 -0.262

BM103793 0.58 0.907 0.65 0.887 0.559 0.533 0.451 0.491 -0.09 -0.115 -0.592 -0.883

BM103822 -0.228 0.162 0.826 0.553 0.806 1.076 0.597 0.976 0.529 0.659 0.236 -0.036

BM103840 1.987 -3.33 -3.321 -2.826 -3.824 -3.224 -3.424 -4.271 -3.633 -3.647 -4.548 -4.319

BM103866 0.382 0.981 0.9 0.375 0.377 -0.145 0.22 -0.334 -0.035 -0.948 -0.25 -0.352

BM103889 0.791 1.039 0.57 1.029 0.742 0.855 0.667 0.948 0.073 0.206 -0.077 -0.07

BM103906 0.845 1.867 0.809 0.495 0.151 -0.023 -0.089 0.38 0.213 -0.634 -0.259 -0.361

BM103911 0.819 0.465 -0.428 -0.587 -0.752 -0.038 -0.384 -0.863 -0.487 -0.155 -0.506 -0.535

BM103922 1.221 -0.124 -0.014 -0.478 -0.883 -0.732 -1.229 -0.983 -1.033 0.42 0.17 0.521

BM103927 0.584 0.02 0.592 0.322 0.489 1.07 0.808 1.485 0.46 0.296 -0.061 -0.129

BM103941 -0.032 1.017 0.364 0.602 0.522 -0.315 0.281 0.862 0.117 0.175 0.063 0.188

BM103943 -0.252 -0.35 0.106 0.388 1.142 0.929 0.436 1.385 0.36 0.752 0.646 0.303

BM103957 0.216 0.664 0.663 0.461 0.316 0.853 0.241 0.593 0 0.08 -0.25 -0.096

BM103974 1.257 0.9 -1.044 -1.302 -0.601 0.132 -0.154 -0.47 -0.763 -0.068 -0.279 -0.534

BM103978 1.052 0.92 1.456 1.856 1.293 0.258 0.286 0.503 -0.511 -0.329 -0.781 -0.947

BM103996 1.084 0.635 -0.119 0.214 0.174 0.234 0.099 -0.149 -0.406 -0.474 -0.447 -0.924

BM104000 2.197 -2.156 -1.677 -2.405 -3.089 -1.079 -1.529 -4.164 -2.167 -3.145 -3.527 -3.405

BM104005 2.588 -1.245 -1.378 -1.131 -1.885 -0.822 -0.933 -2.395 -0.908 -2.577 -1.87 -2.093

BM104033 1.39 1.531 -0.563 -0.536 -0.418 -0.093 -0.195 -0.779 -0.599 -0.593 -0.55 -0.86

BM104048 1.083 1.044 0.581 0.284 0.596 0.309 -0.057 0.111 -0.664 -0.49 -0.678 -1.118

BM104076 0.621 1.25 0.655 0.787 0.839 0.508 0.386 1.001 0.34 0.292 0.227 -0.02

BM104100 1.213 -0.102 -0.221 -0.297 -0.526 -0.051 -0.423 -0.689 -0.081 -0.142 -0.255 -0.169

BM104103 0.365 1.387 0.996 0.847 0.078 -0.245 -0.181 -0.594 -0.543 -0.498 -1.069 -0.702

BM104112 0.011 0.637 0.42 0.421 0.435 0.05 0.095 0.206 0.001 0.05 0.021 0.121

BM104124 1.508 1.031 2.577 1.373 0.732 -0.067 -0.024 -0.504 -0.55 -1.014 -0.348 -0.489

BM104267 -0.293 0.947 0.345 0.691 0.832 -0.483 0.09 0.505 -0.096 -0.504 -0.076 -0.295

BM104287 0.505 0.404 1.326 0.726 1.196 1.249 0.66 1.156 0.394 0.824 0.086 0.115

BM104296 1.094 1.434 1.586 0.389 -0.952 -0.637 -0.767 -1.236 -1.305 -1.353 -1.537 -1.398

BM104302 0.861 0.725 -0.393 -0.432 -0.775 -0.077 -0.339 -0.521 -0.208 -0.119 -0.257 -0.33

BM104310 0.246 0.068 1.656 1.399 1.958 1.705 0.66 1.532 1.546 1.585 0.985 0.111

BM104313 0.464 0.981 1.419 1.209 0.845 0.242 0.46 0.32 -0.402 -0.415 -0.321 -0.396

BM104315 0.805 1.23 1.685 1.591 1.23 1.872 0.578 0.888 0.277 -0.07 -0.424 -0.574

BM104348 0.427 0.841 0.692 0.965 0.271 0.324 0.372 0.308 -0.038 0.177 -0.245 -0.537

BM104357 1.225 1.297 0.688 0.342 0.189 -0.025 -0.108 -0.129 -0.165 -0.412 -0.404 -0.388

BM104369 0.492 1.089 1.022 0.394 0.308 0.304 0.041 0.026 -0.125 -0.199 -0.282 -0.609

BM104379 1.13 0.64 0.111 -0.368 -1.189 -0.148 -0.576 -1.185 -0.499 -0.487 -0.864 -0.666

BM104403 0.159 0.683 1.03 1.567 0.446 -0.061 0.128 -0.076 -0.068 -0.601 -0.296 -0.533

BM104515 2.322 1.2 1.154 0.776 0.538 0.693 -0.183 -0.586 -1.334 -2.04 -2.601 -2.214

BM104526 1.596 -3.1 -3.537 -2.565 -3.504 -3.155 -2.908 -4.425 -3.431 -4.981 -3.16 -4.345

BM104683 0.807 1.779 2.227 2.012 2.262 1.8 1.856 2.086 0.958 1.073 0.376 0.265

BM104738 -0.511 -2.579 -1.188 -1.129 -0.491 0.62 0.2 0.667 0.375 1.291 0.891 0.217

BM104753 0.88 1.93 1.629 1.228 0.164 0.483 -0.399 -0.526 -0.381 -0.913 -0.882 -0.736

BM153935 -0.464 -0.304 0.108 -0.332 -0.877 -0.406 -0.656 -0.335 -0.042 0.558 0.516 0.811

BM153976 -0.481 -3.354 -1.545 -0.985 -0.782 0.432 0.108 0.438 0.26 1.035 0.651 0.172

BM154004 0.531 0.71 0.359 0.425 0.795 0.171 0.464 0.92 -0.072 0.22 0.05 0.197

BM154014 1.654 0.824 -1.168 -0.12 -0.566 -0.351 -0.686 -0.922 -0.979 -1.713 -1.699 -2.088

BM154123 -0.416 0.073 0.541 0.764 1.158 -0.164 0.048 0.531 -0.044 -0.241 0.01 -0.224

BM154193 0.602 0.75 0.008 0.315 0.187 0.22 -0.263 0.829 -0.177 -0.06 0.078 -0.173

BM154199 -4.446 -4.19 -4.237 -4.168 -4.457 -2.868 -2.792 -4.815 -3.254 -3.974 -2.759 2.525

BM154327 1.02 -0.068 -0.104 0.253 -0.04 0.488 -0.303 0.133 -0.538 -1.246 -1.188 -1.295

BM154370 -0.63 -0.668 -0.843 -0.447 0.092 -0.026 0.029 0.191 -0.185 -0.359 0.333 0.644

BM154625 -0.344 -0.156 0.402 1.139 1.182 1.408 0.515 1.313 0.265 0.059 0.091 -0.106

BM154820 -0.595 -0.851 -0.908 -0.556 0.055 -0.347 -0.008 -0.676 0.069 -0.104 0.518 0.206

BM154897 1.317 1.16 1.704 1.689 0.704 0.488 0.115 0.3 0.231 -0.342 -0.38 -0.765

BM155225 1.698 0.842 1.388 1.632 1.652 1.903 1.532 1.095 0.275 -0.065 -0.472 -0.704

BM155230 -0.102 -0.069 -0.161 -0.579 -0.022 -0.031 0.076 -0.401 0.083 0.139 0.542 0.137

BM155251 1.163 -0.217 -0.373 -0.071 -0.191 -0.296 0.282 -0.217 -0.312 -0.143 -0.454 -0.181

BM155353 0.68 0.262 -0.449 -0.269 -0.541 0.166 -0.164 -0.598 -0.111 -0.21 -0.312 -0.412

BM155459 1.208 0.432 -0.038 0.485 0.819 1.113 0.583 0.594 -0.257 -0.344 -0.788 -1.277

BM155568 -0.257 -1.979 -0.899 -0.654 -0.319 0.668 0.379 0.63 0.512 1.372 1.123 0.449

BM155767 0.593 0.913 0.7 1.344 1.155 0.212 0.291 0.435 -0.044 -0.03 -0.059 -0.092

BM155853 -2.209 -1.565 -2.752 -1.168 -1.723 -2.121 -1.227 -2.024 -1.665 -2.593 -1.368 2.272

BM156012 0.639 0.484 0.461 0.389 0.779 1.541 0.849 1.777 0.917 0.615 0.465 -0.028

BM156045 -0.618 0.02 -0.055 0.239 0.589 0.056 -0.056 0.624 -0.119 0.085 0.118 0.126

BM156079 -0.702 -0.431 -0.73 -0.212 -0.927 -0.595 -0.442 -1.094 -0.42 -0.665 -0.335 1.745

BM156086 -0.746 -0.413 -0.608 -0.318 0.086 -0.145 -0.273 -0.264 -0.023 -0.007 0.72 0.277

BM156110 -0.319 0.211 0.928 1.469 1.767 1.709 1.163 1.454 0.577 0.456 0.411 0.462

BM156154 -1.247 -2.881 -1.119 -0.826 -0.929 0.237 -0.092 0.007 0.132 0.892 0.55 0.054

BM156550 0.04 1.385 1.374 0.444 0.013 0.008 -0.153 0.032 -0.096 -0.445 -0.103 -0.316

BM156717 0.249 0.103 -0.327 -0.045 0.042 -0.217 0.252 0.733 0.051 0.586 0.119 0.344

BM156822 0.62 0.758 0.904 0.486 0.893 0.621 0.054 0.558 0.03 -0.436 -0.148 -0.064

BM156854 0.497 1.025 1.353 0.592 0.404 0.436 0.342 0.769 0.12 0.185 -0.018 -0.059

BM156878 0.476 1.001 2.003 1.591 1.392 0.984 0.556 0.252 0.74 -0.014 0.186 -0.061

BM156904 1.519 1.754 1.823 1.037 0.593 0.343 0.315 0.029 -0.749 -0.686 -1.323 -1.466

BM156937 -0.349 0.079 0.298 -0.598 -0.048 0.052 0.002 0.181 0.076 0.319 1.45 0.077

BM156973 0.954 0.69 0.607 0.429 -1.245 -0.258 -0.408 -0.48 -0.455 -0.185 -0.228 -0.28

BM156982 0.166 -0.269 -0.822 -0.454 -0.753 -0.33 -0.241 0.559 0.399 0.92 0.891 0.36

BM157063 -0.563 -1.812 -1.162 -0.545 -0.27 0.346 0.277 0.886 0.282 1.551 1.254 0.958

BM157095 -0.138 0.607 0.684 0.72 0.69 0.491 0.261 1.014 0.605 0.342 0.09 -0.38

BM157248 0.826 0.935 0.283 0.433 0.388 -0.127 0.306 1.135 0.211 0.498 0.277 0.144

BM157299 0.174 0.724 0.195 0.197 0.596 0.066 0.153 0.762 0.043 -0.24 -0.138 0.03

BM157381 -0.33 -1.806 -0.878 -0.646 -0.581 0.332 0.145 0.288 0.073 0.668 0.68 0.167

BM159128 -0.54 -2.462 -0.958 -1.048 -0.505 0.555 0.163 0.451 0.153 1.202 0.83 0.313

BM171814 0.738 1.744 1.43 1.095 -0.119 0.05 -0.143 -0.261 -0.643 -0.602 -0.81 -0.571

BM172681 -0.187 0.546 0.896 1.198 0.992 0.397 0.05 0.331 -0.07 -0.114 -0.321 -0.305

BM181246 -5.052 -4.362 -1.702 -0.922 0.042 1.376 1.551 1.722 1.819 2.433 1.635 0.163

BM181598 -0.078 0.544 0.669 0.675 0.568 -0.173 0.295 0.674 0.158 0.203 -0.15 -0.429

BM181627 1.124 1.28 -0.289 -0.611 -1.28 -0.455 -0.926 -0.996 -0.861 -0.854 -1.024 -0.734

BM181646 0.413 0.477 0.041 0.066 -0.753 -0.066 -0.375 -0.337 0.003 0.853 0.697 -0.422

BM181650 1.313 1.287 1.875 1.825 1.332 1.162 0.354 0.532 -0.12 -0.026 -0.615 -0.973

BM181653 1.692 1.169 -1.147 -1.191 -1.975 -1.303 -2.028 -2.454 -2.436 -2.278 -3.029 -3.227

BM181685 0.927 1.05 1.375 0.875 0.685 0.52 0.389 1.035 0.439 0.19 -0.154 -0.166

BM181708 1.848 -0.241 -0.72 -0.896 -2.613 -2.091 -2.234 -3.924 -2.403 -2.319 -3.433 -2.627

BM181720 1.427 -1.269 -0.747 -0.241 -0.987 -0.471 -0.76 -2.045 -1.17 -1.455 -1.973 -1.183

BM181726 0.572 1.063 1.125 0.822 0.899 0.676 0.159 0.758 0.422 0.151 0.359 0.179

BM181733 1.029 0.385 -0.312 0.041 -0.377 0.4 -0.01 -0.082 -0.045 -0.045 -0.504 -0.263

BM181739 -0.461 -0.193 0.016 -0.78 -0.472 -0.525 -0.172 0.968 0.418 1.007 0.62 -0.323

BM181750 -1.133 -2.179 -0.998 -0.665 -1.136 -0.425 -0.232 -0.612 -0.346 0.801 0.876 0.184

BM181759 0.865 0.14 0.681 0.522 0.321 0.845 0.486 0.557 0.376 0.385 0.001 -0.153

BM181762 0.883 0.168 -0.007 0.188 -0.31 0.252 0.048 0.545 -0.28 0.182 -0.226 -0.224

BM181792 1.903 0.429 -1.442 -1.394 -2.733 -2.416 -3.231 -3.422 -2.991 -2.4 -2.041 -2.725

BM181817 -0.262 -0.242 0.426 0.53 0.386 1.329 0.468 0.735 0.642 1.276 0.831 0.189

BM181821 0.83 1.029 1.35 1.496 0.827 0.816 0.258 0.533 0.122 0.099 -0.058 -0.401

BM181828 0.774 0.223 0.413 1.023 0.693 0.821 0.752 0.358 0.316 0.231 -0.125 -0.165

BM181840 0.45 0.729 0.807 1.245 1.359 0.631 0.805 1.087 0.079 0.245 0.001 0.08

BM181897 1.535 -0.067 -2.066 -3.148 -2.698 -1.025 -1.697 -1.682 -2.225 -2.663 -2.114 -2.25

BM181944 2.815 -0.694 -2.599 -0.429 -1.221 -0.721 -1.215 -1.553 -1.69 -2.598 -1.645 -1.903

BM182031 1.182 1.341 1.14 -0.144 -0.286 -0.12 -0.103 -0.228 -0.372 -0.696 -0.715 -1.094

BM182238 1.988 1.253 -0.441 -0.544 -2.303 -1.088 -0.497 -2.209 -1.117 -2.457 -1.286 -1.482

BM182245 1.419 1.422 1.112 0.622 0.003 -0.331 -0.21 -1.164 -0.94 -0.984 -1.492 -1.459

BM182248 0.2 0.884 0.332 0.627 0.838 0.006 0.552 1.2 0.205 0.412 0.443 -0.129

BM182275 0.121 0.366 0.235 0.671 0.456 -0.113 0.207 0.762 0.153 0.356 0.512 -0.089

BM182302 -0.191 0.224 0.419 0.228 0.811 0.059 0.376 0.998 -0.039 0.183 0.541 0.186

BM182319 -0.338 0.264 -0.049 0.629 1.057 0.616 0.858 1.489 0.309 -0.358 -0.722 -1.107

BM182325 0.303 0.52 -0.04 0.252 0.254 0.508 0.629 0.743 0.374 0.311 0.003 -0.015

BM182327 0.91 0.888 1.003 1.504 1.698 1.609 1.339 1.971 1.017 0.705 0.128 0.256

BM182332 0.832 1.071 -0.498 -0.978 -1.239 -0.735 -0.555 -0.551 -0.588 -0.627 -0.683 -0.672

BM182386 1.214 1.141 1.787 1.62 0.95 1.159 0.293 0.258 0.095 0.273 -0.205 -0.229

BM182430 -0.031 1.432 1.176 0.875 0.267 0.261 -0.219 0.353 -0.296 -0.29 0.198 -0.207

BM182440 1.367 0.785 -0.757 -0.531 -0.361 -0.062 0.492 -0.131 -0.396 -0.103 -0.329 -0.666

BM182450 -0.395 -0.015 0.683 0.778 1.024 1.195 0.83 1.046 0.152 0.287 -0.085 -0.129

BM182563 -2.762 -5.406 -1.611 -0.382 -0.439 0.344 -0.06 -0.263 -0.409 0.509 -0.033 -0.381

BM182574 -0.728 0.153 0.303 0.514 0.875 0.116 -0.087 0.629 -0.099 -0.203 0.076 -0.218

BM182575 1.091 -0.162 -0.259 0.306 0.387 0.152 0.006 -0.078 0.05 -0.684 -0.465 -0.476

BM182673 0.092 0.47 0.523 0.623 0.634 -0.026 -0.045 0.125 0.066 -0.168 -0.295 -0.327

BM182680 -0.587 0.545 -0.02 0.341 0.097 -0.045 -0.003 -0.207 0.394 -0.632 0.423 0.913

BM182720 1.721 1.553 0.166 -0.077 -0.855 -0.419 -1.185 -1.52 -1.158 -2.395 -1.831 -1.489

BM182738 0.263 0.735 0.72 0.561 0.698 0.61 0.147 0.489 0.141 -0.154 -0.158 -0.207

BM182739 0.157 -0.057 0.003 -0.12 -0.103 0.009 0.251 0.442 0.288 0.817 -0.2 -0.078

BM182742 1.555 -0.768 -1.027 -0.799 -0.853 -0.293 -0.062 -0.834 -0.127 -0.621 -1.154 -0.896

BM182744 -0.595 -1.724 -0.008 -0.143 -1.112 -0.347 -0.046 -0.802 -0.112 0.943 0.97 0.147

BM182750 0.234 0.431 0.012 0.411 0.521 0.563 0.43 0.877 0.183 0.157 -0.044 -0.157

BM182761 1.01 0.879 -0.482 -0.028 0.401 -0.115 0.048 -0.143 -0.384 -0.65 -0.73 -0.44

BM182782 1.983 -0.854 -1.131 -1.34 -1.648 -1.27 -2.014 -1.667 -1.625 -1.334 -2.039 -1.133

BM182849 0.933 0.427 -0.285 -0.298 -0.87 -0.223 -0.27 -0.464 -0.494 -0.209 -0.658 -0.233

BM182911 -2.587 -1.889 -2.749 -1.759 -1.38 -0.522 -0.083 0.379 0.235 1.343 0.634 0.19

BM183007 0.444 0.612 0.397 0.323 0.502 0.597 0.151 1.044 0.517 0.875 0.719 0.593

BM183152 -0.646 -0.811 0.948 1.471 1.276 1.907 1.164 1.278 0.508 1.604 1.859 0.663

BM183157 0.641 0.921 1.471 1.574 0.735 1.038 0.197 1.293 0.655 0.975 0.283 0.306

BM183234 0.662 1.152 1.105 0.824 0.726 0.346 0.25 0.649 0.09 0.308 -0.163 -0.077

BM183249 -0.239 -0.69 -0.482 -0.151 0.087 0.486 -0.127 0.966 -0.378 0.361 -0.543 -0.913

BM183276 0.88 1.012 0.628 0.216 -0.168 0.196 -0.034 0.362 0.021 0.26 0.186 0.242

BM183279 -0.225 1.068 1.525 2.298 2.119 0.371 0.698 1.401 0.039 -0.237 0.086 0.3

BM183288 0.019 1.423 0.911 0.264 0.518 0.08 0.029 0.303 -0.083 -0.232 -0.093 -0.34

BM183338 0.752 -1.383 -2.007 -1.548 -2.026 -0.962 -1.411 -2.394 -1.651 -1.135 -1.02 0.558

BM183382 1.078 -0.785 -1.002 -1.209 -1.782 -0.937 -0.487 -2.098 -1.086 -1.537 -1.511 -1.298

BM183399 0.906 0.573 -0.156 0.126 0.136 0.289 0.155 -0.071 -0.196 -0.273 -0.372 -0.327

BM183463 -0.43 1.337 1.725 1.397 0.758 -0.136 -0.032 0.284 0.166 0.232 0.136 -0.263

BM183474 -1.163 -2.775 -1.341 -0.84 -0.581 0.496 0.215 0.389 0.337 1.15 0.855 0.296

BM183518 0.1 0.243 -0.014 -0.125 0.167 -0.146 0.046 0.563 -0.139 0.129 0.01 -0.021

BM183574 -0.293 0.406 1.344 2.067 1.196 0.141 0.256 0.17 0.021 -0.268 0.128 -0.194

BM183623 -0.533 -3.251 -1.514 -1.026 -0.687 0.337 0.142 0.162 0.273 1.032 0.632 -0.118

BM183654 1.663 0.375 0.678 0.341 -0.103 0.013 -0.124 -0.24 -0.289 -1.018 -0.48 -0.671

BM183759 0.401 1.259 1.157 1.472 1.126 0.743 0.496 1.392 0.421 0.396 0.295 0.032

BM183764 1.021 1.289 0.838 0.21 -0.035 0.475 -0.058 0.004 -0.175 -0.14 -0.499 -0.808

BM183770 1.297 1.094 -0.433 -0.961 -1.126 -0.138 -0.753 -1.359 -0.924 -0.665 -1.248 -0.656

BM183794 0.222 0.859 1.593 1.649 1.52 1.251 0.959 0.681 0.443 0.118 0.341 0.359

BM183795 1.595 0.601 -0.282 -0.342 -1.09 -0.357 -0.743 -1.472 -0.817 -1.783 -1.746 -1.28

BM183857 -1.962 -2.772 -2.052 -1.539 -1.674 -1.625 -1.748 -1.647 -1.685 -0.79 -0.158 1.103

BM183873 1.345 1.011 0.43 0.097 -0.913 -0.762 -0.561 -1.652 -1.323 -1.921 -1.736 -2.218

BM183903 0.596 -1.367 -0.441 0.295 0.508 0.692 0.773 1.294 0.622 0.942 0.541 0.124

BM183908 0.667 0.867 0.604 0.601 -0.31 -0.14 0.01 -0.062 -0.042 -0.038 -0.358 -0.362

BM183973 1.164 1.241 1.479 1.526 1.023 1.063 0.601 1.079 0.582 0.651 0.275 -0.466

BM183980 -0.762 -1.606 -0.741 -0.245 -0.617 0.327 0.201 0.274 0.219 0.927 0.759 0.144

BM184003 1.279 0.656 -0.309 -0.472 -0.516 -0.191 -0.255 -0.509 -0.246 -0.549 -0.651 -0.699

BM184007 2.08 -3.212 -3.123 -2.097 -2.584 -2.071 -1.975 -3.349 -3.033 -2.774 -3.499 -3.265

BM184017 1.101 0.143 0.075 -0.051 -0.745 -0.074 0.134 0.233 -0.04 -0.044 -0.219 -0.429

BM184035 0.587 0.685 1.359 1.391 1.538 1.314 1.225 1.163 0.467 0.168 -0.384 -0.183

BM184045 0.084 0.626 0.251 0.688 0.601 0.427 0.387 0.698 0.085 0.216 0.081 0.009

BM184046 1.71 1.329 0.293 -0.118 -0.62 -0.375 -1.044 -1.032 -2.037 -2.516 -1.971 -2.401

BM184075 1.329 1.528 0.479 -0.295 -0.763 -0.463 -1.033 -1.399 -1.231 -2.388 -2.028 -2.502

BM184100 0.398 -0.112 0.133 0.409 1.143 0.212 0.37 1.15 0.223 0.379 0.104 0.201

BM184104 0.21 1.465 0.746 0.44 -0.75 -0.1 -0.279 -1.407 -0.786 -0.713 -1.656 -0.97

BM184105 1.863 -3.329 -3.261 -2.873 -3.313 -2.25 -3.042 -4.128 -2.319 -3.802 -3.808 -3.464

BM184122 1.122 0.332 0.165 -0.11 0.029 0.881 0.262 0.156 -0.029 0.409 0.267 0.281

BM184129 1.172 1.088 0.396 -0.374 -0.497 0.114 -0.311 -0.474 -0.267 -0.488 -0.482 -0.425

BM184148 0.245 0.076 -0.151 -0.005 -0.435 0.494 -0.119 0.462 -0.244 0.502 -0.037 0.405

BM184161 -0.492 0.147 2.595 2.185 1.085 0.126 0.616 0.97 0.65 0.18 0.36 -0.013

BM184209 0.312 1.747 0.753 0.697 0.222 0.287 0.072 0.194 -0.029 -0.262 -0.873 -0.711

BM184227 1.168 0.895 -0.798 -0.007 -0.975 -0.463 -0.346 -1.532 -0.414 -0.773 -1.159 -1.13

BM184237 0.094 0.504 0.321 0.699 0.965 -0.177 0.499 1.073 -0.014 0.031 -0.002 -0.173

BM184239 1.222 0.473 -0.083 -0.315 -0.187 0.253 0.231 0.454 -0.036 0.395 -0.155 -0.632

BM184248 0.067 -0.07 -0.375 0.895 1.088 -0.219 -0.367 -0.422 -0.579 -1.084 -0.88 -0.899

BM184281 1.568 0.863 -1.31 -1.657 -1.503 -1.042 -1.194 -1.397 -1.998 -2.06 -2.549 -2.474

BM184284 1.128 0.231 0.501 0.662 0.171 0.787 0.548 0.81 0.521 0.6 0.244 0.238

BM184367 -1.181 0.405 1.523 2.016 2.175 0.921 -0.064 1.287 0.516 0.565 0.258 0.215

BM184381 -0.688 -1.837 -1.252 -0.738 -1.163 -0.957 -0.642 -0.323 1.195 0.975 1.079 -0.445

BM184447 1.721 1.887 1.047 0.476 -0.509 -0.273 -0.312 -0.947 -0.896 -1.472 -1.26 -1.557

BM184509 0.564 0.656 0.638 0.63 0.418 0.275 0.244 0.901 0.298 0.514 0.666 0.433

BM184694 0.908 0.46 0.925 1.089 1.531 1.81 1.978 2.494 1.003 1.383 0.796 0.409

BM184696 -0.635 0.218 0.733 0.782 0.688 -0.061 -0.077 0.299 0.36 0.158 0.26 -0.303

BM184757 1.62 1.236 0.734 0.619 0.326 0.243 -0.424 -0.487 -0.62 -0.938 -1.328 -1.145

BM184838 0.555 0.055 0.702 0.275 0.941 0.633 0.254 1.262 0.75 0.825 0.183 -0.06

BM184886 0.512 0.378 0.148 0.219 0.709 0.051 0.368 0.842 -0.089 0.038 0.019 -0.12

BM185057 0 0.699 0.79 1.342 0.701 0.223 0.233 0.235 -0.245 -0.404 -0.382 0.231

BM185124 0.687 -0.161 0.633 0.808 0.835 0.966 0.773 0.667 -0.006 -0.212 -0.475 -0.644

BM185145 0.608 0.993 1.629 1.291 1.26 0.869 0.732 0.872 0.007 -0.043 -0.508 -0.28

BM185158 -0.132 0.013 1.945 3.112 3.293 2.218 1.281 1.514 0.715 -0.062 0.191 0.333

BM185168 0.14 -0.359 -0.911 -0.641 -0.545 0.387 0.293 1.006 0.334 0.439 0.159 0.371

BM185181 1.101 1.021 0.293 -0.484 -2.334 -0.481 -0.637 -2.931 -1.535 -0.703 -2.689 -1.968

BM185198 -0.061 -0.147 0.116 -0.103 0.653 0.341 0.236 0.891 0.133 0.162 -0.018 0.275

BM185202 0.992 0.74 0.623 0.35 0.404 1.003 0.191 0.225 0.062 0.154 -0.023 0.068

BM185211 0.901 0.278 -1.278 -1.089 -0.353 -0.079 0.131 -0.259 -0.38 -0.336 -0.442 -0.856

BM185226 1.466 0.626 -0.27 -0.635 -0.804 0.04 -0.485 -0.558 -0.41 -0.516 -0.307 -0.391

BM185237 -0.285 -0.45 0.693 0.618 0.411 0.812 0.782 0.796 0.323 0.165 -0.137 -0.248

BM185239 1.381 0.244 0.236 0.099 0.884 1.303 1.032 1.351 0.593 0.523 0.119 -0.256

BM185242 0.828 -1.934 -3.851 -3.261 -3.715 -3.058 -3.714 -3.418 -3.859 -4.908 -3.251 -3.132

BM185251 -2.041 -1.986 -0.391 -0.447 0.317 -0.132 -0.396 -1.013 0.494 -0.344 0.437 0.42

BM185255 0.974 0.407 0.178 0.025 -0.16 0.101 0.03 -0.005 0.08 -0.01 0.025 0.113

BM185263 0.388 0.893 1.376 1.016 0.286 0.821 0.467 0.512 0.073 0.261 0.025 -0.217

BM185291 1.825 0.297 -1.947 -1.845 -4.31 -1.753 -1.864 -4.659 -2.307 -3.591 -4.063 -3.301

BM185294 1.408 1.023 -0.126 -0.159 -0.133 0.047 0.133 0.523 0.181 0.272 0.249 -0.139

BM185338 -0.028 1.311 0.723 0.405 -0.441 -0.397 -0.24 -0.564 -0.562 -0.535 -0.359 -0.567

BM185350 1.299 -0.792 -0.462 -0.329 -0.3 0.616 0.406 0.406 -0.071 0.886 0.451 0.037

BM185367 0.436 0.298 0.508 0.38 0.577 0.591 0.344 0.805 0.36 0.485 0.212 0.158

BM185382 0.36 0.904 1.23 0.615 0.976 0.927 0.768 0.93 0.367 0.137 -0.029 0.053

BM185394 0.23 -0.364 0.004 -0.002 0.525 1.176 0.482 0.857 0.403 0.551 0.666 0.159

BM185759 -0.645 0.099 0.461 1.394 1.207 -0.134 0.597 1.076 0.426 0.657 0.772 0.353

BM185901 0.05 -0.114 -0.085 0.066 -0.146 0.386 -0.313 -0.312 -0.166 -0.041 0.095 1.595

BM186050 0.493 0.712 0.711 0.554 0.836 0.758 0.688 0.863 0.456 0.41 -0.112 -0.533

BM186051 0.612 1.491 1.718 1.152 1.618 1.923 1.76 1.734 1.531 1.431 0.688 0.517

BM186095 0.177 0.138 -0.56 -0.163 -0.294 -0.133 -0.008 0.603 0.135 0.063 -0.064 -0.058

BM186124 -0.343 -0.094 0.164 -0.309 0.013 -0.168 0.181 0.864 0.348 0.497 0.628 0.32

BM186246 -1.733 -2.543 -1.219 -1.008 -0.969 -0.102 -1.35 -0.932 -0.876 -0.02 -0.173 0.497

BM186588 -0.012 0.043 -0.04 0.494 0.478 0.087 0.179 0.258 -0.09 -0.083 0.092 0.016

BM186665 -1.055 -0.089 -0.201 -0.421 -0.421 -0.178 -0.489 -0.323 -0.705 -0.025 0.356 1.458

BM186976 -1.623 -1.194 -1.556 -1.064 -1.519 -0.459 -0.539 0.105 0.376 1.103 0.567 0.221

BM187382 -1.106 -0.907 -1.01 -0.681 -0.467 -0.343 -0.513 -0.617 0.03 -0.186 0.445 0.311

D13045 -0.895 -1.574 -0.982 -0.75 -1.018 -0.474 -0.353 -1.007 -0.421 0.185 0.882 0.48

D26174 -0.284 0.052 -0.004 1.143 1.975 0.321 0.575 1.242 0.297 -0.121 1 0.015

D32214 -0.111 -0.152 -0.016 0.203 0.091 0.131 -0.169 0.655 0.409 0.152 0.563 0.177

D38454 0.876 1.359 2.007 2.087 1.386 1.501 1.006 1.561 0.718 0.588 -0.164 -0.882

H56788 -1.223 -1.502 -1.697 -0.61 -1.168 -0.418 -0.971 -2.071 -0.922 -1.369 -0.32 1.883

L03395 0.157 0.961 1.465 1.267 0.916 0.385 0.02 0.76 -0.145 -0.02 -0.157 -0.142

L03399 0.281 1.193 0.377 0.678 0.944 0.306 -0.039 1.158 0.231 -0.021 -0.492 -0.395

L11711 0.308 0.8 0.914 0.738 0.563 0.475 0.093 0.358 0.382 0.052 -0.212 -0.515

L25273 -0.733 -0.728 -0.523 -0.321 -0.617 -0.451 0.37 0.866 0.162 0.341 0.249 0.65

L27585 -0.148 -0.173 -0.071 -0.033 -0.116 -0.32 0.016 0.667 -0.069 0.26 0.183 0.359

L35586 -1.849 -1.375 -2.687 -1.058 -1.502 -1.008 -1.039 -0.424 0.173 1.539 0.226 -0.745

L46801 0.534 0.449 1.877 2.076 2.922 2.188 1.918 2.095 1.126 0.414 0.188 -0.149

L47669 -2.241 -1.942 0.378 0.997 1.079 1.453 1.127 0.92 0.463 0.72 0.449 0.124

L48017 -0.663 0.613 2.432 1.413 1.983 0.003 0.132 1.409 0.154 -0.116 0.493 -0.27

L77146 -0.894 -0.78 0.531 0.89 0.56 0.832 0.434 0.456 0.289 0.684 0.369 0.071

S68799 -0.221 0.37 1.468 0.803 1.194 1.231 0.403 0.965 -0.091 -0.024 -0.2 -0.096

S76875 -0.04 0.633 0.589 0.869 1.157 0.376 0.227 0.69 0.086 -0.241 0.077 -0.353

S76877 -0.351 -0.322 0.419 0.56 0.98 0.838 0.691 1.088 0.602 0.22 0.535 0.04

S80425 -0.432 -0.018 1.244 1.182 1.073 0.885 1.207 1.964 0.254 0.059 0.42 0.804

S80986 -2.376 -1.161 -1.912 -1.309 -2.06 -0.93 -1.115 -0.798 -0.318 0.82 0.636 0.982

U00931 2.166 1.372 0.777 0.047 -1.133 -1.523 -2.244 -1.924 -2.301 -3.19 -2.85 -3.045

U10869 0.055 0.572 0.685 0.983 2.823 0.173 0.76 1.707 0.163 -0.011 -0.134 -0.074

U14587 0.126 -0.451 -0.485 -0.458 -0.64 -0.269 0.183 -0.499 -0.163 0.086 0.982 0.524

U14590 0.002 -0.289 0.077 -0.093 -0.087 -0.197 -0.069 -0.101 -0.167 0.452 0.902 0.797

U16310 -0.92 0.663 1.142 1.317 0.939 -0.315 -0.03 0.432 -0.096 -0.327 -0.469 -0.194

U16311 -0.739 0.472 0.042 0.341 1.947 0.759 0.612 2.266 0.562 0.657 0.574 0.197

U18312 -0.744 0.451 1.602 1.94 1.952 1.665 1.098 2.151 0.744 -0.18 0.224 0.742

U23822 -3.984 -3.574 -3.624 -2.803 -3.981 -2.283 -2.654 -3.118 -1.063 0.375 0.669 0.858

U23839 -0.393 -0.134 0.838 1.611 2.002 0.903 1.071 1.917 0.833 0.502 0.71 0.194

U27121 -0.338 0.099 3.223 1.031 1.12 0.289 0.106 0.544 0.114 0.063 -0.148 -0.386

U31079 -0.428 -0.692 -0.58 -0.093 0.363 0.471 0.617 1.884 0.669 0.94 0.956 0.639

U40995 -0.741 -0.14 -0.448 -0.49 1.136 1.203 0.984 2.288 0.767 0.683 0.995 -0.093

U41081 -0.488 -0.143 1.492 1.729 1.731 1.694 1.771 1.534 1.014 0.996 0.69 0.283

U42392 -0.102 -0.654 0.545 2.58 2.552 0.339 1.061 0.924 0.872 -0.34 0.174 -0.068

U43658 -0.259 0.254 0.344 0.34 0.377 0.065 -0.043 0.021 0.216 -0.27 0.987 0.036

U49407 -0.582 -0.107 0.132 -0.301 -0.272 0.045 -0.1 -0.114 0.117 0.185 0.522 0.23

U49408 -0.328 -0.358 -0.316 -0.29 -0.057 0.499 0.038 1.437 1.11 0.717 1.1 0.23

U49412 -0.546 0.095 -0.166 0.391 0.748 -0.258 0.534 1.412 0.258 0.578 0.592 0.171

U49417 0.27 1.065 2.324 1.579 2.096 2.487 1.62 1.71 0.906 0.25 0.294 0.013

U57390 2.024 -3.715 -3.445 -3.736 -3.603 -2.936 -4.124 -4.222 -3.421 -5.514 -3.691 -5.232

U57964 0.878 -0.245 -0.017 0.493 0.026 0.417 0.15 0.17 -0.118 0.507 0.16 -0.448

U57965 0.779 1.136 1.253 1.004 0.342 0.234 -0.378 -0.452 -0.465 -0.486 -0.528 -1.331

U57973 -0.84 -0.793 -0.944 -0.276 -0.396 -0.097 -0.458 -0.596 0.532 -0.018 0.611 0.074

U60804 0.164 0.584 1.205 0.629 0.585 0.206 0.392 0.877 0.047 0.287 -0.026 0.004

U61395 0.595 -0.562 0.804 1.183 1.228 2.068 1.489 1.456 0.899 1.16 0.815 0.148

U62018 -1.289 -1.084 -1.565 -1.417 -1.375 -0.435 -0.259 -1.036 -0.228 0.554 0.629 1.284

U62134 -0.479 -0.42 0.806 0.673 0.789 0.725 0.85 1.002 0.598 0.461 0.05 0.009

U62619 0.205 0.983 0.749 0.285 -0.198 0.441 -0.195 0.074 -0.548 -0.425 -0.619 -0.217

U66570 -0.401 -0.682 -0.692 -1.034 -0.838 -0.392 0.065 -0.039 0.065 0.45 0.457 -0.045

U66872 -0.714 0.442 -0.494 0.358 1.101 0.399 0.536 0.965 0.444 1.535 0.5 -0.017

U68234 -0.236 0.873 0.433 1.208 2.995 1.007 1.277 1.841 0.342 -0.21 0.368 -0.324

U77595 0.054 -0.069 0.181 0.283 0.299 0.545 0.463 1.15 0.403 0.323 0.356 0.031

U77627 0.101 0.147 0.3 0.64 0.963 1.236 0.407 1.358 0.766 0.434 0.17 -0.376

U84616 0.033 -0.002 0.935 1.018 0.881 0.137 0.408 0.671 0.101 0.241 0.118 -0.067

U85090 -1.479 -1.014 1.227 0.659 0.539 -0.125 -0.049 0.738 0.21 0.719 0.742 1.204

U85091 0.308 1.041 1.604 1.169 0.639 -0.014 -0.209 0.011 -0.417 0.375 0.455 0.939

U89380 -0.604 0.194 -0.116 0.148 -0.026 -0.007 -0.121 0.629 0.317 0.261 0.328 0.011

U89710 -0.728 -0.019 -0.578 -0.259 -0.268 -0.125 -0.484 0.042 -0.785 0.013 0.625 1.005

U93458 -1.236 -1.746 -1.256 -1.114 -0.746 -0.326 -0.024 0.816 1.083 1.308 0.914 0.596

U93459 -1.054 -1.831 -1.001 -0.746 -0.356 -0.204 0.327 0.336 0.917 1.073 0.784 0.513

U93478 -0.025 0.146 0.291 0.471 0.529 0.455 -0.1 -0.072 0.301 0.144 0.905 0.184

X12802 -0.67 -2.849 -2.012 -2.563 -0.37 -0.278 -0.387 -0.018 -1.55 -1.091 -1.142 -0.236

X60095 -0.572 -0.111 0.277 -0.178 -0.074 0.016 0.639 1.558 0.587 0.221 0.639 0.196

X65060 -0.646 -0.467 -0.024 0.006 0.701 0.742 0.508 1.287 0.144 -0.121 -0.148 -0.276

X65061 -0.674 0.211 -0.605 -0.467 -0.133 -0.001 0.161 0.573 0.296 0.552 0.747 0.085

X66958 -0.423 0.138 0.256 2.309 3.55 1.587 1.615 4.469 1.539 0.262 0.251 0.201

X67648 -1.484 -0.865 -0.497 0.518 1.062 0.712 1.023 1.535 0.88 0.98 0.555 0.628

X68324 -0.717 0.343 0.063 0.962 0.149 0.38 -0.128 0.269 0.559 0.412 0.658 0.48

X70299 -0.468 -0.75 -0.195 -0.379 -0.561 -0.024 -0.424 -0.514 0.069 0.37 0.697 0.937

X70300 -0.97 -0.821 -0.583 -0.154 -0.295 -0.247 0.227 1.08 0.979 1.143 0.954 0.102

X70322 -0.733 -0.057 -0.061 -0.137 -0.059 -0.259 -0.16 1.412 0.16 -0.317 -0.045 -0.371

X79821 -2.071 -0.96 2.076 2.461 1.681 2.096 1.317 1.66 1.403 1.531 1.042 0.382

X83594 1.515 1.564 2.156 1.635 -0.206 -0.108 -0.701 -0.762 -1.16 -1.984 -1.912 -1.837

X84224 1.632 1.718 3.393 3.219 2.202 1.232 -0.26 -1.81 -1.918 -2.715 -2.928 -2.551

X85977 -0.448 -0.042 0.179 -0.156 -0.138 -0.182 0.04 0.281 0.021 0.29 0.46 0.583

X87581 -1.892 -1.153 2.857 2.754 2.173 1.424 1 1.17 0.804 1.252 1.018 0.398

X87750 -0.43 0.015 0.059 0.059 0.348 -0.012 -0.17 0.551 0.128 0.349 0.559 0.397

X96422 -0.427 -1.564 -0.962 -1.61 -1.825 -0.285 -0.648 -1.446 -0.39 0.075 0.886 0.743

X97330 -0.077 0.171 -0.234 -0.088 -0.21 -0.334 0.121 1.176 0.519 1.096 0.642 0.21

X97332 -1.003 -1.133 -0.652 -0.4 -1.48 -0.429 -0.069 0.154 -0.08 1.053 1.263 1.022

X97333 -1.144 -0.011 0.164 -0.138 0.672 1.826 1.256 2.191 1.625 1.272 1.44 0.741

Y07905 -0.702 -0.446 -0.365 -0.273 -0.697 -0.017 -0.574 -0.409 0.247 0.377 0.922 0.652

Y08321 -6.291 -6.731 -4.656 -4.12 -4.311 -1.57 -0.998 -0.069 0.394 1.843 1.12 0.034

Y12236 0.66 0.248 -0.083 -0.349 -0.664 -0.148 -0.557 -0.625 -0.313 -0.162 -0.503 -0.373

Y12819 -0.767 0.864 1.209 1.133 0.514 -0.065 -0.619 0.024 -0.082 -0.209 -0.888 -0.586

Y13653 -5.37 -5.897 -4.748 -3.724 -2.815 -0.626 -0.576 -0.466 -0.078 1.198 1.081 0.949

Y13944 -0.95 -1.046 -0.46 -0.402 -1.318 -0.378 -0.214 0.11 0.289 0.292 0.601 0.804

Y13948 -0.383 0.021 -0.107 -0.369 -0.325 -0.009 -0.075 0.071 0.276 0.128 1.097 0.553

Y14530 -0.653 -0.433 -0.675 -0.807 -0.245 0.079 0.036 0.759 0.387 0.687 0.876 0.851

Y14531 -0.339 0.288 0.407 0.237 -0.081 0.1 0.471 1.725 0.067 -0.194 0.5 0.227

Y14533 -0.755 -0.185 -0.048 -0.962 -0.1 0.132 0.324 1.793 0.594 -0.331 0.967 -0.072

Y14534 -0.728 0.545 0.407 0.05 0.071 -0.125 -0.012 0.603 -0.31 -0.34 0.527 -0.274

Y14548 -0.667 -0.321 0.461 0.024 -0.234 -0.369 -0.098 0.428 0.244 0.224 0.572 0.203

Y16043 0.579 0.601 1.043 0.84 -0.021 -0.046 -0.762 -0.818 -1.057 -0.672 -0.412 -0.478

Z22762 -0.869 -0.077 1.045 0.736 1.212 1.485 0.813 0.983 0.378 0.556 0.534 0.034
