# Supplementary material for: Transcriptome Analysis of Zebrafish Embryogenesis Using Microarrays
Source: PLoS Genet. 2005 Aug 26;1(2):e29. doi: 10.1371/journal.pgen.0010029 (PMC1193535; doi:10.1371/journal.pgen.0010029)
Supplement: Dataset S2 — Genes showing maximum transcript levels in unfertilized eggs and different patterns of degradation. (137 KB DOC) [file pgen.0010029.sd002.doc]

Dataset S2. Maternal genes. Genes showing maximum transcript levels in unfertilized eggs and

different patterns of degradation

Genbank IDUF egg 3hpf 4.5hpf 6hpf 7.7hpf 9hpf 10.7hpf 12hpf 15hpf 24hpf 30hpf 48hpf

AB038320 1.086 -0.008 0.222 0.173 -0.107 0.358 -0.247 -0.446 -0.172 -0.175 -0.475 -0.622

AB055681 1.126 0.02 -0.07 0.495 0.035 0.188 0.448 0.524 0.158 0.594 0.267 0.035

AF001299 1.224 -1.043 -1.387 -0.922 -0.98 -0.687 -0.607 -0.766 -1.082 -1.304 -1.17 -0.25

AF095457 1.221 -2.998 -2.489 -3.206 -4.066 -3.554 -4.045 -5.04 -3.945 -4.241 -6.51 -5.517

AF149720 2.107 -1.396 -1.147 -1.066 -1.496 -0.845 -0.921 -1.455 -0.778 -1.451 -1.122 -1.356

AF191561 0.892 0.25 0.319 0.337 0.068 0.524 0.141 0.611 0.226 0.519 -0.133 -0.092

AF262047 0.807 -0.201 -0.234 -0.248 -0.158 0.231 0.065 0.27 0.128 -0.263 0.09 -0.422

AF288409 2.002 -0.708 -1.194 -0.813 -0.018 -0.431 -0.948 -0.336 -0.74 -1.505 -0.865 -0.964

AF302805 1.821 -0.08 -0.639 -0.524 -1.126 -0.395 -1.195 -1.136 -1.623 -1.82 -1.693 -1.706

AF331967 1.749 -3.198 -2.422 -2.993 -3.42 -2.84 -3.009 -3.908 -3.619 -4.677 -4.381 -4.744

AF364084 1.062 -0.065 -0.386 -0.193 -0.412 0.158 0.382 0.507 0.271 0.529 0.146 -0.428

AI444200 1.457 0.583 0.551 0.869 0.095 -0.151 -0.037 -0.166 -0.193 -0.585 -0.122 -0.654

AI497232 2.107 -2.202 -1.519 -1.5 -2.699 -1.905 -1.516 -3.002 -3.018 -2.578 -3.75 -2.593

AI544976 1.357 -0.236 0.392 0.318 -0.324 -0.133 -0.225 -0.905 -1.081 -0.779 -0.933 -0.613

AI545442 1.216 -0.788 -0.52 -0.865 -0.807 -0.226 -0.822 -0.762 -0.574 -0.092 -0.134 0.313

AI558273 0.903 0.356 0.337 0.377 0.359 0.829 0.062 0.929 0.474 0.349 0.273 -0.046

AI584590 1.42 -0.394 -0.833 -0.745 -0.227 0.122 -0.213 -0.127 -0.088 0.026 -0.062 -0.197

AI617012 0.837 -0.386 -0.877 -0.816 -0.061 0.18 -0.206 0.218 -0.052 0.557 -0.018 -0.002

AI641585 1.057 -0.297 -0.188 0.023 -0.006 0.16 0.355 0.661 0.053 0.245 0.126 -0.002

AI667141 1.264 0.117 0.466 0.026 -0.06 0.565 0.388 0.005 0.229 0.338 0.336 0.53

AI667289 1.039 -1.263 -0.838 -1.613 -0.966 -0.011 -0.625 -0.657 -1.122 -1.124 -1.648 -1.65

AI667323 1.995 -0.778 -0.642 -0.7 -0.31 0.442 -0.109 0.334 -0.08 0.727 0.251 0.019

AI667659 0.997 -1.111 -1.388 -1.616 -1.386 -0.137 -0.477 -0.17 -0.357 -0.235 -0.246 0.089

AI721358 1.077 -0.315 -0.308 -0.451 -1.379 -0.502 -0.39 -1.494 -0.575 -0.125 -0.97 -0.339

AI793794 0.903 -2.113 -2.251 -2.077 -1.759 -0.609 -1.292 -1.631 -1.825 -2.031 -2.409 -2.292

AI878687 1.206 0.298 0.352 0.27 1.091 0.508 0.359 0.296 0.412 0.34 0.13 0.344

AI883911 1.482 -0.406 0.501 -0.124 -0.571 -0.269 -0.462 -1.218 -1.004 -1.892 -1.223 -1.281

AI942944 1.099 0.025 -0.503 -0.119 0.17 0.228 -0.445 -0.134 0.135 -0.294 -0.469 -0.367

AI964116 2.099 -3.467 -2.332 -2.284 -3.585 -2.832 -2.932 -4.446 -4.13 -3.956 -6.182 -5.295

AI964174 1.216 -0.219 -0.56 -0.287 -0.35 -0.269 -0.329 -0.717 -0.496 -0.682 -0.565 -0.473

AI965182 0.826 -0.049 0.467 0.266 0.173 0.157 0.168 0.668 0.005 0.195 -0.039 0.042

AI974137 2.313 -2.212 -2.718 -2.913 -2.6 -1.181 -2.179 -1.366 -2.621 -2.786 -1.62 -2.028

AW019011 0.996 0.168 0.484 0.346 0.103 0.414 0.517 0.134 0.12 0.123 -0.335 -0.327

AW019444 1.178 0.296 -0.093 0.527 0.216 0.817 0.562 0.62 0.317 0.436 -0.006 0.025

AW019758 1.146 -1.189 -0.63 -0.49 -0.137 -0.093 -0.26 -0.313 -0.227 -0.629 -0.842 -0.534

AW019847 0.804 -1.903 -1.283 -1.385 -1.313 -0.793 -1.041 -1.684 -1.312 -1.547 -1.094 -0.316

AW058902 1.172 0.241 0.639 0.704 0.437 1.285 0.666 1.256 1.021 1.039 0.401 -0.436

AW059389 0.865 -0.556 -0.368 0.033 0.041 0.781 0.49 0.744 0.57 0.722 0.288 -0.513

AW076964 1.428 -2.313 -3.401 -3.23 -3.732 -1.666 -2.508 -3.539 -3.797 -4.05 -3.9 -3.539

AW077011 0.997 0.131 -0.048 -0.006 -0.025 -0.106 -0.256 -0.188 -0.127 -0.249 -0.212 -0.23

AW077025 0.89 -0.077 0.522 0.279 0.416 0.745 0.647 0.09 0.478 0.257 0.31 0.546

AW078163 1.376 0.135 -0.126 0.175 0.567 0.452 0.427 0.688 -0.125 0.042 -0.359 -0.21

AW116206 1.449 -3.773 -3.24 -2.841 -3.433 -2.796 -3.238 -4.262 -4.089 -5.011 -4.462 -5.078

AW116228 1.262 -1.761 -2.119 -1.705 -2.334 -2.987 -3.65 -3.64 -4.47 -5.033 -5.052 -4.477

AW116284 1.688 -0.206 -0.422 -0.291 -0.975 -0.954 -1.167 -1.626 -1.205 -1.255 -1.011 -0.786

AW116474 1.282 -0.968 -1.094 -0.857 -1.632 -0.531 -0.826 -1.72 -1.166 -1.616 -1.419 -1.74

AW117015 1.925 -1.043 -1.454 -0.865 -1.215 -0.517 -0.527 -1.445 -0.961 -1.169 -1.091 -0.89

AW128246 2.113 -1.006 -2.169 -2.199 -1.964 -0.922 -0.401 -1.425 -0.922 -1.679 -1.187 -1.656

AW128332 1.415 -0.534 -0.297 0.083 0.315 0.786 0.029 0.273 -0.104 -0.07 -0.259 -0.418

AW133873 1.159 -0.566 -0.843 -0.503 -2.152 -0.755 -0.415 -1.82 -0.562 -0.688 -1.255 -0.78

AW134000 1.869 -3.232 -2.957 -3.488 -3.722 -2.825 -4.167 -5.218 -4.834 -5.32 -7.339 -5.879

AW154269 1.398 0.611 1.195 0.958 1.186 1.401 0.508 0.686 0.409 0.151 -0.036 -0.256

AW154701 1.04 -0.012 0.411 0.414 -0.011 0.179 0.125 -0.405 -0.334 -0.4 -0.504 -0.391

AW165160 0.941 -2.896 -1.247 -2.895 -0.329 0.183 0.063 -0.081 -0.552 -0.412 -0.574 -0.396

AW165251 1.006 -0.65 -0.471 0.19 0.599 0.474 0.35 0.667 0.137 -0.447 -0.354 -0.339

AW170860 1.591 -3.303 -3.424 -2.933 -3.497 -1.938 -2.953 -4.659 -3.741 -5.338 -4.994 -4.085

AW171049 1.619 -2.866 -1.865 -2.807 -3.899 -1.919 -1.944 -4.515 -3.745 -3.747 -5.879 -3.391

AW171204 1.829 -0.057 -0.544 -0.195 -1.712 -0.703 -0.395 -1.05 -0.884 -0.878 -1.242 -1.261

AW171363 1.096 0.017 -0.221 0.298 0.173 0.018 -0.156 -0.396 -0.569 -0.203 -0.261 -0.123

AW171595 1.281 0.594 0.761 0.442 0.754 1.172 0.575 0.807 0.383 0.138 0.339 0.056

AW174275 1.48 -1.004 -0.824 0.967 0.171 -0.543 0.557 -0.923 -0.811 -2.882 -2.11 -2.221

AW174595 1.272 -1.568 -1.208 -1.05 -0.746 0.028 -0.299 -0.103 -0.397 0.239 -0.168 0.219

AW202620 1.051 -0.048 0.121 0.362 0.474 0.286 0.284 0.671 0.248 0.667 0.384 0.23

AW203026 0.741 -0.768 -1.06 -0.546 -1.031 -0.172 -0.164 -0.84 -0.449 0.136 -0.032 0.053

AW232589 1.127 -0.885 -0.917 -0.851 -0.865 -0.419 -0.473 -0.676 -1.088 -0.888 -1.277 -0.915

AW232642 1.07 0.213 0.535 0.572 0.597 0.663 0.405 0.49 -0.013 -0.093 -0.342 -0.429

AW233564 0.852 -3.294 -3.229 -3.365 -2.646 -2.309 -2.979 -2.878 -3.898 -4.108 -3.754 -3.53

AW281416 1.293 0.085 0.329 0.566 0.412 0.192 -0.211 -0.18 -0.316 0.234 -0.306 -0.308

AW305598 1.364 0.137 -0.001 -0.248 -0.219 0.115 -0.202 0.149 -0.216 -0.211 -0.11 -0.507

AW343922 1.156 -1.703 -1.979 -1.476 -1.214 -0.594 -1.065 -0.931 -0.878 -0.449 -0.759 -0.508

AW344075 1.428 -2.213 -2.493 -1.675 -2.414 -1.312 -1.389 -2.697 -1.922 -3.31 -2.504 -2.583

AW421941 1.955 -0.689 -1.343 -0.921 -0.96 -0.216 -0.806 -1.365 -1.052 -1.471 -1.637 -1.156

AW422929 1.644 -0.883 -1.754 -1.58 -1.564 -0.59 -1.229 -1.146 -1.477 -1.924 -2.023 -1.726

AW777467 0.815 0.361 0.312 0.39 0.555 0.759 0.559 0.284 0.047 -0.044 -0.192 -0.074

AW777691 1.231 0.159 0.326 -0.046 0.253 0.883 0.285 0.991 0.431 0.972 0.573 0.061

AY029577 0.977 -0.366 0.613 0.622 0.359 0.308 0.094 -0.228 -0.391 -0.086 -0.67 -0.748

BE017363 0.96 -0.295 -0.053 0.634 0.925 -0.187 -0.127 0.363 -0.019 -0.011 0.227 -0.311

BE017551 1.268 -2.541 -2.35 -2.217 -2.396 -1.927 -2.569 -2.748 -1.702 -2.977 -2.953 -2.908

BE017795 1.779 -1.516 -0.513 -2.2 -2.122 0.03 -1.015 -1.44 -0.559 -0.314 -0.427 -0.46

BE557106 1.834 -0.668 -0.134 -0.944 -1.026 -0.384 -0.554 -0.75 -0.529 -1.095 -1.168 -0.941

BG302504 2.067 -4.968 -4.639 -4.352 -5.23 -4.092 -4.583 -5.691 -5.38 -5.537 -5.57 -6.063

BG303079 1.451 0.132 -0.304 -0.283 -0.2 -0.344 0.169 -0.447 -0.291 -0.379 -0.432 -0.422

BG303824 1.079 0.185 0.236 0.091 -0.199 0.037 -0.079 -0.154 -0.226 -0.284 -0.07 -0.239

BG303979 0.789 -1.53 -2.205 -2.106 -2.349 -1.738 -1.208 -2.789 -1.585 -2.387 -1.834 -2.223

BG304232 1.547 -0.823 -0.57 -0.698 -2.327 -1.269 -1.655 -2.199 -1.904 -1.568 -2.441 -1.71

BG305705 0.949 0.204 0.495 0.174 0.267 0.554 0.636 -0.184 0.429 0.271 0.512 0.523

BG305800 1.202 0.155 0.404 0.038 -0.26 0.047 -0.249 0.072 -0.113 0.259 -0.023 -1.261

BG306096 1.229 -0.06 -0.559 0.29 -0.562 -0.162 -0.231 -0.546 -0.276 -0.682 -0.476 -0.614

BG306308 1.104 -1.842 -1.875 -1.296 -1.045 0.128 -0.423 -0.104 -0.41 0.41 0.759 0.158

BG307383 1.305 -0.699 -0.659 -0.465 -0.572 -0.136 -0.137 -0.139 -0.352 0.212 -0.155 0.063

BG307539 1.425 0.326 1.024 1.475 1.177 0.592 0.892 0.811 0.131 0.112 -0.297 -0.428

BG727413 1.582 0.28 -0.401 0.019 0.218 0.581 0.159 0.63 -0.264 -0.426 -0.207 -0.394

BG727595 1.3 0.468 0.71 0.626 0.562 1.117 0.672 0.293 0.492 0.469 0.32 -0.093

BG729054 1.156 -1.105 -0.747 -0.933 -1.147 -0.78 -1.798 -1.343 -0.962 -1.052 -1.207 -1.229

BG799157 1.249 -0.824 -0.565 -0.401 0.04 0.294 0.02 0.182 -0.228 -0.238 -0.171 -0.138

BG883703 2.524 -1.172 -1.223 -0.883 -0.962 -0.401 -0.515 -0.986 -0.994 -0.703 -1.134 -0.364

BG884507 1.699 -0.863 -1.373 -1.433 -1.086 -0.062 -0.953 -0.568 -1.132 -1.665 -0.413 -1.148

BG892077 1.609 0.095 0.409 0.701 0.962 0.71 0.869 0.444 0.095 0.176 -0.252 -0.527

BG985703 0.921 -0.067 0.225 0.309 -0.374 0.09 0.085 -0.07 -0.124 0.401 -0.231 -0.695

BI428520 1.043 0.002 -0.326 -0.258 -0.922 -0.175 -0.45 -0.766 -0.505 0.249 -0.034 -0.335

BI429706 1.237 -0.997 -0.921 -0.722 -0.802 -0.358 -0.193 -0.61 -0.556 -0.546 -0.517 -0.168

BI672095 1.143 0.546 0.615 0.413 0.273 0.266 0.184 0.135 0.089 -0.037 0.164 -0.252

BI672378 0.741 -0.952 0.516 0.615 0.235 0.259 0.224 0.001 -0.25 0.56 0.177 -0.188

BI672468 0.751 -0.519 0.038 -0.274 -0.745 -0.132 -0.255 -1.037 -0.677 -0.222 -1.157 -0.932

BI672555 1.229 0.013 -0.428 0.017 0.15 0.6 0.249 -0.084 -0.158 0.039 0.068 0.328

BI673452 2.379 -0.165 -0.093 -0.248 -1.924 -1.703 -1.938 -3.082 -1.782 -2.238 -2.128 -2.426

BI704370 1.248 0.597 0.979 1.223 0.677 0.732 0.574 0.119 -0.05 -0.299 -0.838 -0.621

BI705720 1.174 0.015 0.078 -0.426 0.11 0.276 -0.408 0.04 0.262 0.447 0.622 0.502

BI709146 1.556 0.341 -0.024 0.17 0.381 1.13 0.013 1.059 0.47 0.49 0.232 -0.223

BI709417 1.272 -1.539 -0.584 -1.482 -1.516 0.243 -0.398 -1.047 -0.546 -0.056 -0.219 0.072

BI709770 0.998 -0.123 -0.119 0.08 0.237 0.416 -0.08 -0.077 -0.159 -0.152 -0.425 -0.499

BI709863 2.316 0.367 -0.115 0.817 0.283 0.077 -0.373 0.039 -0.261 -0.673 -1.012 -0.903

BI710046 1.126 -0.3 0.149 0.049 0.245 0.423 0.048 0.419 0.013 0.31 0.075 -0.179

BI840365 0.955 -0.92 -0.672 -0.228 -0.218 0.258 0.04 0.043 -0.425 0.008 -0.18 -0.134

BI843185 0.931 -0.005 -0.052 -0.719 0.026 -0.192 -0.034 -0.417 -0.192 -0.685 -0.274 -0.124

BI843286 2.118 -1.165 -1.262 -2.332 -2.77 -1.576 -2.302 -3.616 -2.02 -1.951 -3.558 -2.277

BI843480 1.493 -0.026 -0.108 -0.187 -0.166 -0.125 -0.414 -0.405 -0.626 -1.171 -0.591 -0.637

BI845363 0.911 0.056 0.01 0.144 0.078 -0.135 0.108 0.28 -0.349 -0.031 -0.189 0.028

BI864067 0.606 -0.813 -0.41 -0.222 -0.563 -0.282 -0.398 -0.699 -0.573 0.204 0.169 0.115

BI866874 1.109 -0.22 -0.653 -0.521 0.407 0.169 -0.324 -0.467 -0.429 -0.005 -0.188 -0.564

BI866963 2.231 -2.741 -2.877 -2.768 -2.901 -1.046 -2.767 -2.836 -2.166 -3.318 -3.14 -2.11

BI867169 0.964 -0.489 -0.236 -0.392 -0.307 -0.111 -0.467 -0.419 -0.775 -0.141 -0.297 0.087

BI867248 1.401 -1.282 -1.543 -0.915 -1.181 -0.56 -1.162 -1.374 -1.005 -1.512 -1.009 -1.492

BI877132 0.959 -3.513 -3.198 -3.268 -4.126 -3.181 -4.511 -5.157 -4.499 -6.549 -5.749 -6.206

BI877478 0.987 -0.154 -0.161 -0.421 -0.789 -0.562 -0.564 -0.91 -0.67 -0.733 -1.029 -0.966

BI877608 0.977 0.017 0.058 -0.038 -0.168 0.102 0.19 -0.224 -0.33 0.087 -0.488 0.163

BI877878 1.289 -1.86 -1.365 -1.085 -1.594 -1.061 -0.794 -2.322 -0.952 -1.433 -2.008 -2.033

BI877887 1.191 0.367 0.154 0.5 0.286 0.514 -0.054 0.46 0.051 0.11 0.125 0.05

BI878195 2.144 -2.395 -3.302 -2.216 -3.259 -3.066 -4.083 -4.539 -4.078 -5.2 -5.376 -5.064

BI878269 1.497 0.389 0.075 0.354 0.134 -0.018 -0.312 0.171 -0.067 -0.72 -0.417 -0.717

BI878842 1.92 -1.273 -1.207 -1.088 -0.849 -0.355 -0.419 -0.988 -0.933 -1.854 -1.01 -0.915

BI879615 0.708 -2.781 -2.476 -2.08 -3.584 -2.575 -2.187 -3.87 -2.607 -3.835 -4.309 -4.2

BI879616 0.967 -0.501 -0.03 0.569 -0.285 0.397 -0.073 0.102 -0.396 0.172 -0.017 0.063

BI880658 0.864 -0.897 -0.45 -0.358 -0.455 0.244 -0.327 0.004 0.067 0.412 0.153 0.17

BI884788 2.06 0.251 -0.329 -0.087 -0.6 0.014 -0.264 -0.154 -0.311 -0.427 -0.324 -0.587

BI885564 1.303 0.082 -0.11 -0.28 -0.393 -0.28 -0.594 -0.534 -0.458 -1.129 -0.2 -0.486

BI885896 0.967 -0.681 -0.805 -0.356 -0.591 0.084 -0.653 -0.783 -0.827 -0.99 -1.213 -0.538

BI888131 1.944 0.378 0.512 0.566 0.25 0.489 0.087 0.47 -0.166 -0.216 -0.681 -1.159

BI889409 1.681 -0.141 -0.417 -0.023 0.277 1.071 0.437 0.979 0.332 0.376 0.058 -0.354

BI889925 1.904 -1.509 -1.511 -1.852 -2.242 -1.102 -1.552 -2.284 -1.875 -1.043 -1.95 -1.002

BI889959 1.303 -1.154 -0.915 -0.964 -1.9 -0.604 -1.089 -2.161 -1.307 -0.875 -1.636 -0.975

BI890056 1.867 -0.331 0.554 0.994 0.513 0.619 -0.001 -0.506 -0.485 -1.176 -1.098 -1.316

BI890592 1.382 0.223 0.294 0.176 0.465 0.424 0.137 0.877 0.204 -0.016 0.246 -0.177

BI891043 0.816 0.083 -0.195 -0.392 -0.346 -0.083 -0.154 -0.238 -0.197 0.145 0.045 0.039

BI891332 1.035 -0.436 -0.261 -0.212 0.092 -0.123 0.085 0.134 -0.38 0.18 0.235 0.164

BI892293 1.201 -3.872 -3.16 -2.827 -4.032 -2.986 -2.674 -4.719 -3.465 -4.145 -4.836 -5.033

BI892353 1.811 -2.305 -2.942 -2.859 -3.706 -2.955 -3.06 -3.945 -3.735 -3.958 -4.792 -4.513

BI896532 0.907 -0.978 -0.959 -0.592 -1.232 -0.706 -0.743 -1.317 -0.789 -0.818 -0.737 -0.389

BI896959 0.985 -0.166 -0.147 -0.329 0.171 0.2 0.134 0.68 0.297 0.448 0.598 0.387

BI979228 1.411 -0.003 -0.13 0.045 -0.379 -0.326 -0.09 -0.698 -0.198 -0.962 -0.248 -0.574

BI979286 2.197 -1.095 -1.643 -0.716 -0.504 -0.391 -0.732 -0.751 -0.786 -1.752 -0.744 -1.55

BI979438 1.405 -0.673 -0.988 -0.898 -0.959 -0.348 -0.849 -1.193 -0.584 -1.163 -1.127 -0.981

BI979549 1.259 -0.418 -0.356 -0.525 -0.183 -0.375 -0.029 0.462 0.078 -0.1 0.063 -0.251

BI979556 1.458 -0.941 -0.303 0.789 0.95 0.12 -0.079 -0.214 -0.294 -0.544 -0.658 -0.748

BI979975 2.343 -2.442 -1.813 -1.49 -1.68 -0.991 -0.545 -1.417 -1.164 -0.867 -1.937 -2.565

BI980026 1.287 -2.131 -2.184 -2.514 -3.578 -2.029 -2.416 -4.168 -3.162 -3.11 -4.399 -3.453

BI980199 1.088 -0.02 -0.277 -0.002 0.032 0.042 0.224 0.874 0.194 0.586 0.07 0.153

BI980396 1.328 -0.851 -0.998 -0.453 -1.056 -0.845 -0.171 -1.374 -0.31 -1.038 -0.479 -0.437

BI980436 2.555 -2.223 -3.135 -2.456 -3.163 -1.873 -2.613 -4.349 -2.714 -3.946 -4.395 -3.267

BI980715 0.776 -0.191 -0.155 -0.845 -0.413 -0.052 -0.063 0.18 -0.198 -0.354 -0.38 -0.005

BI983582 1.387 0.333 0.101 0.085 -0.004 0.112 -0.192 0.652 0.015 0.846 0.516 0.379

BI983596 1.209 -0.214 0.044 0.184 0.138 0.381 -0.137 -0.03 -0.045 -0.163 -0.228 -0.299

BI983753 1.028 0.47 1.03 0.53 0.09 0.127 -0.233 -0.351 -0.255 -0.268 -0.236 -0.368

BI984734 1.395 -0.794 -0.827 -0.576 -1.421 -0.517 -0.911 -1.439 -0.483 -1.081 -0.857 -0.721

BM005437 1.682 -1.012 -1.051 -1.244 -1.034 -0.516 -0.917 -0.567 -0.622 -0.602 -0.728 -0.291

BM024634 1.69 0.61 0.759 0.533 0.077 0.728 -0.066 0.093 -0.329 -0.294 -0.446 -0.242

BM024816 0.873 -0.395 -0.282 -0.484 -0.141 0.15 -0.105 0.016 -0.152 0.054 0.224 0.166

BM026879 1.688 -0.811 -0.595 -0.936 -0.767 0.01 -0.175 -0.672 -0.48 -0.315 -0.621 -0.358

BM035394 1.498 0.055 -0.212 -0.726 -0.173 0.041 -0.357 -0.166 -0.284 -0.585 -0.443 -0.286

BM036347 0.817 -0.151 -0.072 0.742 0.196 0.062 -0.087 0.327 0.067 -0.233 0.003 -0.243

BM036431 0.826 -0.097 0.425 0.509 0.622 0.783 0.59 0.455 0.345 0.511 0.249 0.147

BM037305 1.587 -0.089 0.256 0.269 0.452 0.476 0.162 0.584 0.257 0.499 0.272 -0.013

BM070561 1.404 -1.209 -0.984 -0.914 -0.989 -0.174 -0.536 -1.155 -1.098 -0.639 -0.884 -0.118

BM071061 0.736 -0.437 -0.517 -0.418 -0.621 0.208 -0.226 -0.134 -0.285 0.31 0.065 0.118

BM071664 1.219 -0.711 -0.3 -0.062 -0.018 0.763 0.333 0.808 0.287 0.709 0.259 -0.172

BM071697 1.203 -0.592 -0.256 -0.185 0.196 0.501 -0.009 -0.028 -0.081 -0.385 -0.114 -0.368

BM071732 1.755 0.041 0.683 0.286 0.181 0.873 0.344 0.409 0.171 0.128 -0.02 -0.109

BM071837 1.843 -0.709 -1.339 -0.549 -1.267 -0.661 -0.694 -1.425 -1.151 -1.484 -1.039 -1.029

BM095242 2.041 -2.393 -1.749 -1.447 -3.338 -1.81 -0.938 -3.798 -1.378 -2.945 -3.005 -3.586

BM095268 1.114 0.207 -0.001 0.175 -0.405 0.317 -0.117 0.546 0.31 0.591 0.26 -0.174

BM095390 1.815 -1.135 -1.669 -0.91 -0.334 -0.546 -0.63 -0.822 -0.752 -1.429 -0.538 -1.078

BM095404 1.65 -2.744 -2.535 -2.842 -3.273 -2.764 -3.46 -4.215 -4.317 -5.652 -5.952 -5.761

BM095484 1.631 -1.449 -2.099 -0.921 -2.802 -1.826 -1.071 -3.305 -1.783 -3.723 -2.218 -2.922

BM095607 2.51 -3.655 -4.336 -4.056 -3.689 -2.586 -4.015 -4.684 -3.918 -5.755 -3.732 -4.504

BM095657 1.823 -2.53 -2.688 -1.408 -2.738 -2.2 -2.089 -3.419 -2.033 -2.199 -2.313 -2.133

BM096092 1.183 -0.95 -1.172 -1.49 -1.063 -0.299 -0.83 -1.005 -0.859 -0.751 -0.614 -0.636

BM096095 1.254 -0.568 -0.169 -0.211 -0.018 0.268 0.191 0.553 0.273 0.46 0.152 -0.431

BM096428 1.663 -0.106 -0.15 0.369 0.187 0.411 0.225 0.713 0.12 0.39 -0.177 -0.287

BM101527 2.104 -2.532 -3.325 -3.098 -3.153 -2.984 -3.368 -3.428 -3.074 -4.723 -4.244 -3.918

BM101584 1.151 -0.177 -0.146 0.184 0.186 -0.058 0.133 -0.083 -0.191 -0.308 0.055 -0.369

BM101590 1.507 -2.58 -1.698 -1.758 -2.787 -1.392 -1.451 -3.585 -2.01 -2.302 -3.691 -3.217

BM101604 1.647 -0.051 0.031 -0.019 -0.477 -0.126 -0.356 -1.171 -0.381 -0.98 -0.467 -1.157

BM103127 1.85 -0.53 -0.66 -0.796 -1.866 -0.903 -1.345 -2.272 -1.575 -0.732 -2.171 -1.894

BM103396 1.283 -0.743 -1.615 -0.851 -1.172 -0.452 -0.767 -0.8 -0.738 -0.36 -0.51 -0.081

BM103840 1.987 -3.33 -3.321 -2.826 -3.824 -3.224 -3.424 -4.271 -3.633 -3.647 -4.548 -4.319

BM103922 1.221 -0.124 -0.014 -0.478 -0.883 -0.732 -1.229 -0.983 -1.033 0.42 0.17 0.521

BM104000 2.197 -2.156 -1.677 -2.405 -3.089 -1.079 -1.529 -4.164 -2.167 -3.145 -3.527 -3.405

BM104005 2.588 -1.245 -1.378 -1.131 -1.885 -0.822 -0.933 -2.395 -0.908 -2.577 -1.87 -2.093

BM104100 1.213 -0.102 -0.221 -0.297 -0.526 -0.051 -0.423 -0.689 -0.081 -0.142 -0.255 -0.169

BM104526 1.596 -3.1 -3.537 -2.565 -3.504 -3.155 -2.908 -4.425 -3.431 -4.981 -3.16 -4.345

BM154327 1.02 -0.068 -0.104 0.253 -0.04 0.488 -0.303 0.133 -0.538 -1.246 -1.188 -1.295

BM155251 1.163 -0.217 -0.373 -0.071 -0.191 -0.296 0.282 -0.217 -0.312 -0.143 -0.454 -0.181

BM181720 1.427 -1.269 -0.747 -0.241 -0.987 -0.471 -0.76 -2.045 -1.17 -1.455 -1.973 -1.183

BM181759 0.865 0.14 0.681 0.522 0.321 0.845 0.486 0.557 0.376 0.385 0.001 -0.153

BM181762 0.883 0.168 -0.007 0.188 -0.31 0.252 0.048 0.545 -0.28 0.182 -0.226 -0.224

BM181944 2.815 -0.694 -2.599 -0.429 -1.221 -0.721 -1.215 -1.553 -1.69 -2.598 -1.645 -1.903

BM182575 1.091 -0.162 -0.259 0.306 0.387 0.152 0.006 -0.078 0.05 -0.684 -0.465 -0.476

BM182742 1.555 -0.768 -1.027 -0.799 -0.853 -0.293 -0.062 -0.834 -0.127 -0.621 -1.154 -0.896

BM182782 1.983 -0.854 -1.131 -1.34 -1.648 -1.27 -2.014 -1.667 -1.625 -1.334 -2.039 -1.133

BM183338 0.752 -1.383 -2.007 -1.548 -2.026 -0.962 -1.411 -2.394 -1.651 -1.135 -1.02 0.558

BM183382 1.078 -0.785 -1.002 -1.209 -1.782 -0.937 -0.487 -2.098 -1.086 -1.537 -1.511 -1.298

BM183654 1.663 0.375 0.678 0.341 -0.103 0.013 -0.124 -0.24 -0.289 -1.018 -0.48 -0.671

BM184007 2.08 -3.212 -3.123 -2.097 -2.584 -2.071 -1.975 -3.349 -3.033 -2.774 -3.499 -3.265

BM184017 1.101 0.143 0.075 -0.051 -0.745 -0.074 0.134 0.233 -0.04 -0.044 -0.219 -0.429

BM184105 1.863 -3.329 -3.261 -2.873 -3.313 -2.25 -3.042 -4.128 -2.319 -3.802 -3.808 -3.464

BM184122 1.122 0.332 0.165 -0.11 0.029 0.881 0.262 0.156 -0.029 0.409 0.267 0.281

BM184284 1.128 0.231 0.501 0.662 0.171 0.787 0.548 0.81 0.521 0.6 0.244 0.238

BM185239 1.381 0.244 0.236 0.099 0.884 1.303 1.032 1.351 0.593 0.523 0.119 -0.256

BM185350 1.299 -0.792 -0.462 -0.329 -0.3 0.616 0.406 0.406 -0.071 0.886 0.451 0.037

U57390 2.024 -3.715 -3.445 -3.736 -3.603 -2.936 -4.124 -4.222 -3.421 -5.514 -3.691 -5.232

U57964 0.878 -0.245 -0.017 0.493 0.026 0.417 0.15 0.17 -0.118 0.507 0.16 -0.448

Mean: degradation prior to blastula 1.376 -0.808 -0.77 -0.662 -0.875 -0.392 -0.659 -0.953 -0.854 -0.997 -1.101 -1.081

AA494842 1.776 1.231 -1.928 -1.956 -1.46 -0.562 -1.724 -1.113 -1.675 -2.426 -2.345 -2.101

AA658804 1.001 0.081 -0.566 -0.156 -0.958 0.19 0.452 0.54 0.431 0.75 0.171 0.111

AB032727 1.857 -0.302 -1.492 -1.571 -3.025 -1.173 -0.679 -2.807 -0.758 -2.328 -1.868 -2.263

AF097875 1.095 0.213 -1.754 -1.652 -2.092 -1.451 -1.613 -1.79 -1.245 -0.967 -1.191 -1.683

AF170069 1.109 -0.667 -1.106 -1.293 -2.115 -1.924 -1.105 -3.109 -2.77 -2.591 -2.384 -1.833

AF176316 1.163 0.289 -0.082 0.253 0.082 -0.356 -0.439 -0.709 -0.541 -1.129 -1.096 -0.92

AF277172 0.802 0.433 0.164 0.264 0.188 0.836 0.387 0.146 0.528 0.437 0.458 0.405

AF387341 0.965 0.333 -0.478 -0.31 -0.384 -0.186 -0.053 -0.055 -0.202 0.003 0.106 0.119

AF387820 1.449 1.061 -0.839 -0.632 -0.901 -0.232 -0.724 -0.561 -1.061 -1.166 -0.812 -1.02

AI384860 1.806 0.742 -0.612 -0.065 0.251 0.832 0.114 0.018 -0.389 -0.719 -0.762 -0.891

AI558559 0.987 0.519 -0.936 -0.401 -0.429 -0.055 -0.212 -0.099 -0.137 -0.995 -0.271 -0.721

AI588173 1.106 0.632 -0.496 -0.203 0.366 0.343 -0.003 0.398 -0.106 0.339 0.163 -0.059

AI601556 0.745 0.475 0.136 0.293 0.081 0.734 0.083 0.119 0.234 0.019 -0.319 -0.058

AI626336 0.843 0.172 -0.569 -0.205 -0.528 0.269 -0.191 0.064 -0.03 0.204 0.015 0.3

AI626681 0.847 0.235 -0.815 -0.482 -0.202 -0.066 0.042 0.032 -0.474 -0.406 -0.491 -0.495

AI641779 1.285 0.512 0.058 0.414 0.694 0.916 0.331 0.771 0.518 0.497 0.304 0.256

AI666886 0.778 0.36 -0.201 -0.142 -0.289 0.314 0.254 0.173 0.034 -0.255 -0.269 -0.216

AI666911 1.069 0.898 -0.216 -0.129 -0.15 0.387 -0.371 -0.244 -0.053 0.096 -0.139 0.266

AI667411 0.802 0.79 -0.212 0.033 -0.122 0.015 -0.41 -0.099 -0.194 -0.524 -0.433 -0.636

AI667622 1.891 1.044 -0.791 -0.091 -0.537 -1.192 -0.739 -1.111 -1.715 -1.513 -1.977 -2.14

AI721398 1.611 -0.299 -3.023 -1.153 -2.283 -1.413 -1.155 -2.316 -1.451 -2.221 -1.308 -1.124

AI722482 1.179 0.576 -0.232 -0.237 -0.339 0.026 -0.005 -0.466 -0.17 -0.316 -0.077 -0.383

AI793353 0.843 0.181 -0.168 -0.007 -0.776 -0.007 -0.356 -0.742 -0.52 -0.166 -0.051 -0.18

AI793558 0.87 0.349 -1.034 -0.618 -0.591 -0.193 -0.36 -0.769 -0.83 -1.25 -0.893 -0.68

AI793622 1.287 1.022 -0.552 -0.413 -0.504 -0.133 -0.3 -0.225 -0.562 -0.976 -0.627 -0.913

AI878397 1.028 1.153 -0.206 0.255 -0.115 -0.131 -0.263 -0.12 -0.563 -0.417 -0.587 -0.544

AI884085 0.725 0.28 -0.807 -0.379 -0.578 0.084 -0.282 -0.445 -0.408 -0.519 -0.869 -0.79

AI942929 1.086 0.56 -0.916 -0.494 -0.306 0.101 -0.145 -0.088 -0.061 -0.083 -0.06 0.104

AI942983 0.846 0.802 -0.384 -0.157 -0.159 -0.056 0.389 0.714 0.069 0.179 0.064 -0.11

AI957837 0.904 0.405 0.023 0.486 -0.209 0.715 0.305 0.324 0.179 0.058 -0.411 -0.291

AI959659 1.564 0.438 0.078 0.374 0.414 0.629 0.135 0.396 0.071 -0.112 -0.524 -0.659

AI964178 1.023 0.223 -0.817 -0.044 -0.354 -0.182 -0.266 -0.239 -0.373 -0.214 -0.284 0.361

AI964212 1.338 0.64 -0.055 -0.051 0.489 0.345 -0.01 0.323 0.071 -0.012 -0.165 0.058

AW018939 1.326 0.11 -1.478 -1.138 -0.635 -0.527 -0.444 -1.038 -0.69 -1.255 -0.477 -0.833

AW019723 1.518 0.81 0.182 0.215 0.294 0.507 0.335 0.668 -0.009 0.158 -0.011 -0.085

AW077337 1.341 0.587 -0.82 -0.475 -0.489 0.067 -0.271 -0.258 -0.427 -0.683 -0.76 -0.452

AW077480 1.112 -1.164 -2.006 -1.794 -1.841 -1.243 -1.727 -1.471 -3.097 -2.986 -2.648 -2.122

AW115606 1.344 1.274 -0.467 0.341 0.086 -0.111 -0.313 0.174 -0.411 -0.683 -0.506 -0.369

AW115660 1.579 0.154 -1.622 -0.503 -0.817 -0.59 -0.384 -1.055 -0.48 -1.513 -0.474 -0.808

AW115804 1.087 0.63 -0.508 -0.594 -0.546 -0.335 -0.417 -0.596 -0.232 -0.488 -0.566 -0.567

AW115859 1.645 0.666 -0.914 -0.53 -0.261 -0.343 -0.834 -0.6 -0.724 -0.977 -0.959 -1.381

AW115973 1.339 0.88 -0.879 -0.589 -1.264 -0.712 -0.754 -1.233 -0.455 -0.894 -1.323 -1.188

AW116023 2.409 0.207 -0.941 0.075 -1.074 -0.385 -0.204 -0.933 -0.739 -1.949 -0.75 -1.119

AW116214 1.19 0.268 -0.624 -0.099 -0.478 -0.118 -0.237 -0.288 -0.267 -0.065 -0.33 0.003

AW116237 1.084 0.276 -0.074 0.056 -0.535 -0.232 -0.379 -0.638 -0.694 0.079 -0.28 -0.543

AW116271 1.325 0.535 0.116 1.121 -0.11 -0.113 -0.387 -0.295 -0.208 -0.273 -0.13 -0.339

AW116504 0.933 0.393 0.234 0.407 0.43 0.961 0.521 0.63 0.37 0.194 -0.359 -0.438

AW116573 1.854 -0.201 -2.067 -2.401 -1.422 -0.899 -1.579 -1.339 -1.817 -1.704 -1.46 -1.436

AW116649 0.865 0.105 -1.39 -0.912 -1.147 -0.394 -0.646 -0.539 -0.381 -0.357 -0.151 0.05

AW116685 0.972 0.716 -0.982 -0.33 -0.993 -0.331 0.013 -1.21 -0.48 -0.704 -0.554 -0.515

AW116727 1.263 0.576 -0.38 -0.248 -0.373 -0.043 -0.377 -0.467 -0.275 -0.813 -0.645 -0.474

AW116994 1.53 0.934 0.34 1.073 0.471 0.341 0.128 0.099 -0.19 -0.755 -0.781 -0.933

AW128192 1.242 0.501 -1.287 -0.321 -0.44 0.08 -0.236 -0.274 -0.034 -0.548 -0.333 -0.653

AW134106 -0.512 -3.369 -3.237 -3.602 -4.37 -4.024 -4.196 -5.231 -4.961 -7.317 -6.735 -7.685

AW154137 1.096 1.221 -1.177 -0.207 -0.341 -0.237 -0.367 -0.256 -0.384 -0.364 -0.53 -0.435

AW154153 1.695 0.377 -1.018 -0.291 -0.087 0.312 0.188 0.265 -0.198 -0.333 -0.441 -0.674

AW154231 1.428 1.243 0.222 0.537 0.152 0.701 0.189 0.348 -0.029 0.108 -0.347 -0.233

AW154320 0.952 0.134 -0.518 -0.031 -0.399 0.173 0.075 0.11 -0.011 -0.2 -0.576 -0.793

AW154324 1.144 0.506 0.111 0.113 0.177 0.715 0.447 0.97 -0.071 0.16 0.522 0.077

AW154517 1.385 0.479 -0.996 -0.472 -1.375 -0.303 -0.611 -1.297 -0.949 -1.202 -0.958 -0.943

AW154587 1.792 0.269 -0.58 -0.202 -0.273 -0.045 -0.524 -0.563 -0.515 -1.046 -0.648 -0.679

AW170941 0.961 0.321 -0.961 -0.817 -0.575 -0.359 -0.434 -0.691 -0.638 -1.117 -1.162 -1.218

AW171085 1.304 0.445 0.225 0.238 0.512 0.61 0.372 0.662 0.236 0.01 -0.392 -0.626

AW171396 1.116 0.564 0.37 0.324 0.166 0.259 -0.079 -0.26 -0.1 -0.146 -0.033 0.213

AW171480 1.179 0.433 -0.128 -0.329 -0.287 0.068 -0.117 -0.167 -0.088 0.058 -0.116 -0.26

AW171484 1.155 0.056 -0.178 -0.797 -1.04 0.52 -0.201 0.268 -0.014 -0.303 -0.543 -0.349

AW175553 1.061 0.694 0.602 0.879 0.533 0.743 -0.022 -0.054 0.213 0.183 -0.218 0.046

AW232088 0.841 0.341 -0.611 -0.374 -0.792 -0.018 -0.189 -0.586 -0.567 -0.163 -0.295 0.275

AW232464 1.587 0.399 -0.349 0.011 0.296 0.954 0.103 0.041 -0.594 -0.834 -1.607 -1.477

AW280158 0.916 0.204 -0.382 -0.345 -0.14 -0.04 -0.094 0.388 0.294 0.389 0.001 -0.256

AW282065 1.206 -1.398 -2.554 -2.628 -2.351 -1.725 -2.649 -2.895 -2.678 -3.401 -3.268 -2.962

AW342745 1.109 0.724 0.163 0.303 0.834 1.161 0.392 0.599 0.164 0.292 -0.009 0.133

AW344022 1.271 0.287 -0.499 -0.647 -0.285 0.01 -0.114 -0.486 -0.384 -0.192 -0.629 -0.336

AW344030 1.055 0.334 -0.406 -0.003 0.097 0.652 -0.055 0.488 0.058 0.092 -0.232 -0.089

AW344260 1.089 0.706 -0.525 -0.217 -0.29 0.218 0.256 0.584 0.324 0.265 0.271 -0.409

AW826487 1.992 0.014 -1.667 -1.659 -2.124 -0.611 -0.701 -2.197 -1.354 -2.326 -1.403 -1.156

AY057058 1.451 -0.277 -1.309 -0.527 -1.699 -0.864 -0.23 -1.978 -0.616 -1.085 -1.244 -0.57

BE201771 1.544 0.786 -0.991 -0.963 -0.675 -1.069 -1.526 -1.314 -1.67 -0.595 -0.448 -1.417

BF157346 1.237 0.327 -0.802 -0.334 -0.543 0.206 0.148 -0.177 -0.159 -0.318 -0.502 -0.404

BG302802 1.067 -0.086 -0.944 -0.162 -0.196 0.407 -0.197 -0.039 -0.137 -0.487 -0.29 -0.417

BG302931 1.113 0.993 -0.198 -0.157 -0.079 -0.205 0.009 -0.169 -0.312 -0.204 0.055 -0.238

BG302989 0.915 0.752 -0.909 -0.304 -0.821 -0.317 -0.345 -0.734 -0.543 -0.958 -0.298 -0.586

BG303377 1.89 1.564 -0.514 -0.296 -0.231 0.634 0.366 0.884 0.271 0.237 -0.429 -0.675

BG303472 1.056 0.571 -1.243 -1.14 -0.925 -0.167 -0.391 -0.56 -0.707 -0.738 -0.773 -0.302

BG303641 1.079 0.945 -0.62 -0.05 -0.278 -0.074 -0.5 -0.488 -0.455 -1.022 -0.49 -0.252

BG303695 1.277 0.948 -0.099 0.144 -0.031 0.236 -0.422 -0.547 -0.902 -0.686 -0.488 -0.301

BG303935 1.903 0.346 -0.101 0.571 0.239 0.228 -0.429 -1.102 -1.105 -2.289 -2.04 -2.077

BG304211 0.93 0.215 -0.243 0.082 -0.29 0.201 0.083 -0.134 0.014 -0.429 -0.315 -0.569

BG304233 1.136 0.49 -0.442 -0.158 -0.86 -0.349 -0.07 -0.012 -0.237 -0.369 -0.719 -0.367

BG304294 0.996 0.419 -0.036 0.021 -0.182 0.179 0.105 0.646 0.387 0.76 0.472 0.415

BG305364 0.688 -0.196 -0.593 -0.88 -1.094 -0.133 -0.065 0.291 0.258 0.354 0.498 -0.144

BG305441 0.915 0.361 -0.677 -0.291 0.119 -0.009 -0.982 -0.365 -1.198 -0.445 -0.344 -0.178

BG305537 1.302 0.918 -0.099 0.136 -0.179 -0.028 -0.019 -0.06 -0.241 -0.675 -0.678 -0.408

BG305622 0.981 0.262 -0.213 -0.011 -0.772 -0.167 -0.411 -0.337 -0.342 -0.018 -0.214 -0.437

BG308652 1.027 0.622 -1.17 -0.822 -1.329 -0.175 -0.58 -0.298 -0.345 -0.169 -0.672 -0.377

BG308713 1.515 0.399 -0.526 -0.15 0.333 0.182 0.09 0.365 0.129 -0.217 -0.261 -0.193

BG728382 1.042 0.349 -1.281 -0.641 -0.764 -0.402 -0.407 -0.199 -0.688 -0.735 -0.653 -0.981

BG729372 0.87 0.593 -0.231 -0.147 0.212 0.188 -0.297 0.616 0.128 -0.007 0.311 0.038

BG883692 0.988 0.356 0.005 -0.154 0.32 0.694 -0.01 0.665 0.306 0.359 0.132 0.246

BI474957 1.387 0.698 -0.497 0.015 -0.211 -0.231 0.015 -0.453 -0.525 -0.572 -0.64 -0.572

BI475648 1.855 0.641 -0.452 -0.044 0.217 0.479 0.011 0.131 -0.218 0.018 -0.078 -0.34

BI476292 1.284 0.631 -0.449 -0.39 -0.141 -0.149 -0.564 -0.14 -0.312 -0.579 -0.503 -0.354

BI534308 0.736 0.363 -0.25 -0.004 -0.205 0.303 -0.383 -0.051 -0.214 0.263 0.238 0.122

BI672273 0.872 -0.147 -1.459 -0.593 -0.147 -0.57 -0.614 -0.622 -0.659 -0.302 -0.42 -0.507

BI672464 1.088 -0.034 -1.072 -0.582 -0.596 -0.45 -0.764 -1.128 -0.762 -1.046 -1.188 -0.731

BI672616 1.559 0.042 -0.228 -0.011 -1.415 -0.724 -1.478 -2.638 -1.262 -1.386 -1.721 -0.943

BI673276 1.27 0.148 -1.438 -0.774 -0.769 0.159 0.158 0.634 -0.096 0.027 -0.466 -0.756

BI673308 1.098 0.669 0.132 0.394 0.31 0.488 0.139 0.591 0.186 -0.012 -0.323 -0.655

BI673509 1.629 0.492 0.081 0.501 0.305 0.317 -0.061 -0.095 0.044 -0.754 -0.045 -0.369

BI673579 1.293 1.008 -0.777 -0.432 -0.539 0.052 -0.619 -0.22 -0.475 -0.3 -0.539 -0.567

BI705282 1.687 1.074 -0.022 0.135 0.004 0.45 0.207 0.517 0.255 0.151 0.173 -0.393

BI705594 1.886 0.066 -1.1 -1.604 -1.423 -0.48 -1.489 -0.951 -0.804 -0.878 -0.797 -0.467

BI708320 2.478 0.515 -0.776 -0.754 -1.52 -1.034 -1.279 -1.834 -1.67 -1.795 -1.709 -1.215

BI708455 1.921 -0.034 -2.471 -0.983 -1.432 -1.185 -0.889 -1.566 -1.36 -2.74 -2.636 -2.164

BI709743 1.497 0.407 -0.121 -0.195 0.056 0.361 -0.001 0.316 -0.01 0.191 0.074 -0.369

BI709862 1.034 0.046 -0.458 0.024 -0.276 -0.386 -0.302 -0.601 -0.212 -0.366 -0.881 -0.498

BI839625 1.403 0.177 -1.091 -0.235 -0.799 -0.886 -0.59 -0.954 -0.6 -1.145 -0.786 -0.943

BI845763 0.767 0.324 -0.576 -0.119 -0.711 -0.372 -0.084 -0.433 -0.322 -0.352 -0.279 -0.472

BI846235 1.147 0.449 -0.409 -0.154 -0.693 -0.286 -0.745 -0.817 -0.348 -0.334 -0.291 -0.315

BI865356 1.128 1.204 -0.144 0.115 0.162 0.123 0.129 -0.038 -0.045 -0.238 -0.155 -0.448

BI866527 0.729 0.424 0.127 0.178 0.309 0.523 -0.042 0.104 0.095 -0.014 0.023 -0.04

BI867235 1.097 0.62 -0.222 -0.299 -0.33 0.116 -0.256 -0.309 -0.321 -0.051 -0.309 0.144

BI867240 1.03 0.68 0.306 0.422 0.241 0.178 0.343 0.761 0.144 0.031 0.106 -0.008

BI867449 1.058 0.427 -0.421 -0.167 -0.332 -0.115 -0.233 -0.728 -0.15 -1.368 -0.275 -0.558

BI867642 1.397 0.773 0.699 0.394 0.289 0.494 0.209 0.967 0.213 0.256 -0.045 -0.055

BI878018 1.485 0.662 -1.518 -0.268 -1.178 -0.453 -0.698 -1.072 -0.831 -1.354 -1.006 -1.608

BI878642 1.092 0.82 -0.491 -0.327 -0.84 0.253 -0.324 0.11 -0.177 0.073 -0.042 0.03

BI879576 1.33 -0.051 -0.634 -0.676 -0.801 -0.39 -0.562 -1.157 -0.943 -0.91 -1.701 -0.939

BI879686 1.409 0.874 0.826 1.221 0.912 1.52 0.617 0.815 -0.009 0.457 -0.157 -0.264

BI880357 0.822 -0.027 -0.364 0.062 0.405 0.398 0.107 -1.164 -0.805 -0.896 -0.84 -0.831

BI880563 1.175 0.947 0.267 0.183 0.682 0.976 0.32 0.682 0.25 0.393 0.181 0.265

BI885399 1.125 0.377 -0.079 -0.171 -0.432 0.069 -0.207 -0.069 -0.06 0.069 -0.269 -0.237

BI885503 2.031 -0.301 -1.706 -0.795 -1.945 -0.385 -0.189 -1.856 -0.714 -1.651 -1.519 -1.094

BI885851 1.397 0.958 -0.445 0.062 -0.132 0.217 0.372 -0.302 -0.176 -0.247 -0.208 -0.199

BI886921 1.041 0.636 0.399 0.472 0.501 0.409 0.321 0.213 0.3 0.126 0.044 -0.13

BI887500 1.487 1.142 -1.149 -0.761 -0.946 0.08 -0.736 -0.703 -0.491 -1.492 -0.415 -0.309

BI887770 1.303 0.148 -0.837 -0.403 -0.648 -0.9 -0.36 -1.077 -1.286 -1.244 -2.087 -1.571

BI888265 1.281 0.802 -0.819 -0.359 -0.627 -0.423 -0.717 -0.718 -0.433 -1.092 -0.757 -1.067

BI888359 0.647 0.467 0.066 0.602 0.469 0.61 0.202 -0.151 0.111 -0.095 -0.332 0.021

BI888569 0.782 0.097 -0.415 -0.232 -0.575 -0.032 -0.043 -0.289 -0.112 0.112 -0.221 -0.17

BI888899 1.112 0.462 -0.578 -0.7 -0.177 0.03 0.004 -0.216 -0.165 -0.39 -0.362 -0.098

BI889465 0.95 0.277 -0.067 -0.038 -0.341 0.211 -0.523 -0.126 -0.36 0.324 0.125 0.087

BI890158 1.432 -0.082 -1.082 -1.156 -1.313 -0.408 -0.786 -1.409 -1.237 -0.946 -1.734 -1.891

BI891827 0.898 0.768 -1.067 -1.019 -0.5 0.111 -0.361 -0.488 -0.962 -0.929 -0.778 -0.124

BI891871 0.828 0.401 -0.383 -0.227 0.364 0.415 -0.194 -0.043 -0.659 -0.762 -0.75 -0.56

BI892254 1.518 -0.237 -1.226 -1.026 -1.213 -0.7 -0.777 -1.399 -1.383 -1.871 -1.389 -1.663

BI896491 0.739 0.452 -0.195 0.376 0.056 0.699 0.789 0.08 -0.16 -0.439 -0.877 -1.314

BI897419 1.41 0.658 -1.211 -1.051 -1.615 -0.601 -1.016 -0.902 -0.962 -0.872 -0.097 -0.79

BI979957 1.103 0.937 -0.257 -0.242 -0.151 0.451 0.362 0.827 0.042 0.166 -0.437 -0.27

BI979961 1.574 0.613 -0.487 -0.685 -0.075 -0.045 -0.37 -0.494 -0.29 0.006 -0.507 -0.308

BI980084 1.171 0.766 -0.344 -0.072 0.09 0.474 -0.002 0.132 -0.293 0.002 -0.35 -0.898

BI980640 0.891 -0.66 -1.584 -1.172 -1.825 -1.098 -0.914 -1.637 -1.174 -2.28 -2.366 -1.978

BI980747 1.866 0.554 -0.143 0.386 0.682 1.054 0.498 0.917 0.325 0.183 -0.054 -0.393

BI981133 1.357 0.695 -1.484 -0.479 -0.896 -0.171 -0.47 -0.936 -0.467 -0.535 -0.78 -0.757

BI983233 0.869 0.101 -1.359 -0.739 -0.343 -0.33 -0.272 -0.271 -0.368 -0.063 -0.307 0.136

BI983442 0.958 0.372 -0.113 0.165 -0.143 -0.078 -0.313 -0.066 -0.142 -0.305 -0.203 -0.245

BI983566 0.912 0.675 -0.152 -0.093 -0.204 -0.244 -0.241 -0.09 -0.256 -0.014 -0.164 -0.282

BI983762 1.172 0.551 -0.422 -0.479 -0.084 0.061 -0.326 0.53 -0.178 0.088 -0.116 0

BI983850 1.134 0.815 0.121 0.695 0.6 1.244 0.303 0.323 0.403 0.249 0.202 0.357

BI983855 1.338 0.214 -1.19 -0.601 -0.205 -0.182 -0.5 -0.547 -0.939 -0.894 -0.71 -1.228

BM005412 1.63 1.116 -0.649 -0.522 0.022 0.276 -0.551 -0.089 -0.296 -0.241 -0.574 -0.584

BM024812 0.947 -0.091 -0.823 -0.698 -0.627 -0.244 -0.709 -0.673 -0.527 -0.388 -0.179 0.873

BM026053 1.182 -0.528 -1.49 -1.045 -0.603 -0.526 -0.765 -1.58 -1.021 -1.254 -1.334 -1.487

BM035055 1.027 0.839 -0.566 -0.225 -0.46 -0.043 -0.043 -0.062 0.03 -0.083 -0.462 -0.281

BM036445 1.236 0.187 -1.429 -0.687 -0.757 -0.161 -0.706 -0.469 -0.989 -0.617 -0.952 -1.336

BM036885 2.085 -0.061 -1.04 -0.072 -1.353 -0.358 -0.943 -0.564 -0.917 -1.82 -0.748 -0.862

BM036938 1.763 1.075 0.201 0.46 0.179 0.148 -0.287 -0.309 -0.504 -0.77 -0.513 -0.763

BM037178 2.43 0.07 -1.643 -0.831 -2.107 -1.446 -1.065 -2.789 -1.413 -2.629 -2.144 -2.326

BM037539 1.647 0.312 -0.54 0.438 -0.291 -0.135 -0.275 -0.73 -0.626 -0.684 -1.085 -0.88

BM070949 1.63 1.204 -0.386 -0.246 0.011 -0.179 -0.327 -0.443 -0.546 -0.523 -0.554 -0.562

BM071872 0.781 0.279 -0.563 -0.158 -0.91 -0.289 -0.611 -0.535 -0.591 -0.188 -0.198 -0.435

BM081091 2.827 0.293 -1.323 -0.651 -1.403 -0.535 -0.272 -1.384 -0.942 -1.909 -1.218 -1.157

BM082387 1.16 0.723 -0.095 0.181 0.42 -0.105 0.295 -0.015 -0.04 -0.369 -0.186 -0.469

BM095156 1.508 0.515 -0.459 -0.079 0.117 -0.039 -0.456 -0.531 -0.631 -1.408 -0.72 -0.992

BM095334 0.884 1.033 -0.351 -0.224 -0.117 0.026 -0.021 0.506 0.134 -0.311 -0.323 -0.349

BM095386 1.276 -0.616 -2.389 -1.217 -1.503 -0.652 -1.426 -1.984 -1.812 -2.887 -2.301 -2.227

BM095417 1.59 1.44 0.048 0.473 0.168 0.242 -0.151 -0.094 -0.353 -0.111 0.199 -0.563

BM095815 1.551 -0.483 -2.051 -1.333 -1.737 -1.281 -1.167 -2.007 -2.196 -3.061 -2.458 -2.331

BM095845 2.021 0.705 -1.569 -1.068 -1.902 -0.949 -1.824 -1.418 -1.417 -2.774 -2.028 -1.939

BM095897 1.801 -0.725 -1.563 -1.432 -3.851 -1.629 -1.147 -3.404 -1.282 -2.887 -3.118 -3.259

BM095989 1.346 1.088 0.119 0.621 0.395 1.02 0.646 0.763 0.435 0.216 0.033 -0.222

BM096012 1.686 1.358 -0.272 0.341 -0.233 -0.229 -0.231 0.07 -0.271 -0.687 -0.213 -0.409

BM101507 1.001 0.286 -0.512 0.066 -0.434 -0.289 -0.482 -0.278 0.025 0.04 -0.078 -0.106

BM101574 0.822 -0.104 -1.582 -1.365 -0.929 -0.202 -0.73 -0.384 -0.348 -0.099 -0.133 -0.526

BM101600 1.385 1.222 0.363 0.667 0.584 0.217 0.263 0.312 -0.151 -0.196 -0.54 -0.204

BM101666 1.005 0.751 -0.972 -0.462 -0.201 -0.419 -0.267 -0.756 -0.836 -1.141 -0.419 -0.707

BM103996 1.084 0.635 -0.119 0.214 0.174 0.234 0.099 -0.149 -0.406 -0.474 -0.447 -0.924

BM154014 1.654 0.824 -1.168 -0.12 -0.566 -0.351 -0.686 -0.922 -0.979 -1.713 -1.699 -2.088

BM155353 0.68 0.262 -0.449 -0.269 -0.541 0.166 -0.164 -0.598 -0.111 -0.21 -0.312 -0.412

BM155459 1.208 0.432 -0.038 0.485 0.819 1.113 0.583 0.594 -0.257 -0.344 -0.788 -1.277

BM181733 1.029 0.385 -0.312 0.041 -0.377 0.4 -0.01 -0.082 -0.045 -0.045 -0.504 -0.263

BM182440 1.367 0.785 -0.757 -0.531 -0.361 -0.062 0.492 -0.131 -0.396 -0.103 -0.329 -0.666

BM182761 1.01 0.879 -0.482 -0.028 0.401 -0.115 0.048 -0.143 -0.384 -0.65 -0.73 -0.44

BM183399 0.906 0.573 -0.156 0.126 0.136 0.289 0.155 -0.071 -0.196 -0.273 -0.372 -0.327

BM184227 1.168 0.895 -0.798 -0.007 -0.975 -0.463 -0.346 -1.532 -0.414 -0.773 -1.159 -1.13

BM185211 0.901 0.278 -1.278 -1.089 -0.353 -0.079 0.131 -0.259 -0.38 -0.336 -0.442 -0.856

BM185242 0.828 -1.934 -3.851 -3.261 -3.715 -3.058 -3.714 -3.418 -3.859 -4.908 -3.251 -3.132

BM185255 0.974 0.407 0.178 0.025 -0.16 0.101 0.03 -0.005 0.08 -0.01 0.025 0.113

BM185294 1.408 1.023 -0.126 -0.159 -0.133 0.047 0.133 0.523 0.181 0.272 0.249 -0.139

AF164477 1.413 1.219 0.458 0.341 0.651 0.351 0.221 0.551 0.243 0.195 -0.049 -0.639

AF397015 0.863 0.741 -0.132 -0.122 -0.33 -0.263 -0.677 -0.463 -0.457 -0.632 -0.612 -0.698

AI416043 0.756 0.493 0.026 0.069 -0.016 0.21 0.294 0.277 0.049 -0.082 -0.275 0.052

AI558398 1.751 0.584 -0.479 -0.748 -0.722 0.031 -0.11 -0.423 -0.225 -0.192 -0.523 -0.502

AI588468 1.281 0.736 -0.186 -0.474 -0.071 -0.059 -0.138 -0.375 0.225 -0.552 -0.134 -0.216

AI641491 0.839 0.841 0.761 -0.142 0.494 0.66 0.404 -0.005 0.196 0.261 0.252 0.557

AI666879 0.858 0.603 -0.342 -0.485 -0.247 -0.258 -0.448 -0.532 -0.784 -0.331 -0.405 -0.304

AI667214 0.991 0.459 -0.978 -1.179 -0.859 -0.102 -0.545 -0.647 -0.308 -0.409 -0.497 -0.498

AI667676 1.65 0.668 -0.701 -0.531 -0.73 -0.68 -0.395 -1.042 -1.179 -1.687 -1.613 -1.855

AI882829 1.136 1.169 -0.2 -0.203 -0.451 0.02 -0.105 0.024 -0.294 0.157 -0.126 -0.232

AI957831 1.475 1.006 -0.264 -0.299 -0.408 0.056 -0.183 -0.108 -0.253 -0.257 -0.337 -0.676

AJ011789 2.436 0.195 -2.58 -3.21 -3.959 -3.696 -4.103 -4.251 -4.599 -5.055 -4.566 -5.09

AW018514 1.112 1.204 -0.301 -0.336 -0.365 -0.185 -0.368 -0.364 -0.237 -0.616 0.118 -0.241

AW115598 1.063 1.09 0.165 -0.035 0.123 0.669 0.136 0.334 0.176 0.035 -0.123 0.075

AW115821 1.557 0.763 -0.552 -0.754 -0.739 0.042 -0.431 0.028 -0.523 -0.659 -0.611 -0.705

AW116420 1.455 1.152 -0.605 -0.822 -0.585 -0.442 -0.575 -0.622 -1.138 -1.092 -1.181 -1.063

AW116490 1.432 1.407 -0.549 -0.79 -0.695 -0.141 -0.374 -0.423 -0.602 -1.04 -0.747 -1.05

AW116681 1.529 0.742 0.269 0.265 0.231 0.512 -0.048 0.115 -0.373 -0.211 -0.604 -1.166

AW117034 0.892 0.851 0.3 -0.015 0.122 0.204 -0.168 0.153 -0.292 -0.115 -0.406 -0.004

AW117161 1.127 0.774 0.597 0.32 0.622 0.434 0.446 0.731 0.127 -0.149 -0.316 -0.303

AW154457 1.319 1.001 -0.087 -0.245 -0.236 0.229 -0.062 0.48 0.125 -0.106 0.137 -0.379

AW154496 1.143 0.71 -0.615 -0.55 -0.602 -0.582 -0.744 -0.655 -0.841 -1.259 -0.464 -1.182

AW171388 0.997 0.937 -0.221 -0.69 -0.308 -0.032 -0.383 -0.445 -0.523 -0.818 -0.374 -0.115

AW171467 1.311 0.937 -1.581 -1.835 -2.169 -0.61 -1.341 -1.494 -0.83 -1.456 -1.773 -1.317

BE016164 1.607 1.31 -0.3 -0.383 -0.42 -0.169 -0.106 0.608 -0.002 -0.451 -0.525 -0.439

BG303246 1.177 0.711 -0.114 -1.185 -0.762 -0.678 -0.912 -1.133 -0.828 -1.039 -0.635 -0.639

BG729144 0.893 0.835 -0.359 -0.318 -0.401 -0.049 0.085 -0.449 -0.386 -0.66 -0.498 -0.718

BG729177 1.127 1.144 0.153 -0.177 -0.166 0.302 -0.021 0.608 0.441 -0.047 -0.356 -0.285

BI672829 2.547 0.527 -0.903 -1.725 -1.4 -1.287 -1.131 -1.923 -2.474 -1.877 -4.203 -3.296

BI673483 2.623 0.277 -2.229 -2.813 -2.008 -1.696 -2.105 -2.001 -3.12 -3.62 -3.256 -3.394

BI673488 1.353 1.427 0.244 0.221 0.223 0.774 0.194 0.144 -0.381 -0.167 -0.3 -0.367

BI866770 1.308 1.313 0.356 0.219 0.283 0.233 -0.025 0.298 -0.118 -0.009 -0.113 -0.275

BI876262 -0.662 -3.149 -3.22 -3.553 -4.23 -4.031 -4.485 -5.109 -5.114 -7.37 -7.359 -8.24

BI877285 0.966 0.493 0.022 -0.034 -0.152 -0.016 -0.213 -0.145 -0.229 -0.377 -0.335 -0.56

BI877691 1.088 0.736 -0.25 -0.294 -0.532 -0.047 -0.265 -0.075 0.348 0.616 0.458 0.077

BI877725 1.249 0.651 -0.789 -1.121 -0.229 -0.323 -0.449 -0.494 -0.585 -0.353 -0.316 0.032

BI877898 1.337 1.099 -0.564 -0.701 -0.735 -0.164 -0.733 -0.769 -0.69 -1.268 -0.952 -0.867

BI878817 0.983 0.986 -0.182 -0.274 -0.404 -0.137 -0.015 -0.44 -0.256 -0.464 -0.775 -0.483

BI879566 0.982 0.453 0.48 -0.024 0.146 0.767 0.267 0.22 0.343 0.044 -0.07 -0.064

BI880518 0.96 1.131 -0.688 -0.996 -0.494 -0.196 -0.444 -0.678 -0.573 -0.66 -0.731 -0.544

BI885228 0.984 1.037 -1.269 -1.23 -1.536 -0.47 -1.038 -1.459 -1.155 -0.802 -0.793 -0.22

BI886119 1.333 1.009 -0.132 0.026 -0.106 0.203 -0.207 -0.459 -0.399 -0.717 -0.865 -0.788

BI891643 1.248 0.456 -0.609 -0.271 -0.207 0.426 0.166 -0.193 -0.534 -0.862 -1.31 -2.089

BI983027 1.071 0.487 0.071 -0.152 0.026 -0.063 -0.14 0.719 0.173 0.525 0.433 -0.267

BI983894 1.685 1.184 0.172 0.077 0.215 0.279 -0.029 0.123 0.032 0.458 0.01 -0.371

BM035613 1.047 0.674 0.238 -0.357 -0.087 0.058 -0.345 -0.044 0.099 -0.827 -0.645 -0.139

BM071213 0.968 0.658 0.014 0.08 -0.03 0.114 -0.054 -0.266 -0.353 -0.494 -0.544 0.054

BM082504 1.306 1.248 -0.539 -0.99 -0.905 -0.119 -0.551 -0.968 -0.516 -1.017 -0.687 -0.69

BM095259 1.481 1.427 0.047 -0.125 -0.249 0.168 0.048 0.959 0.358 0.059 0.155 -0.698

BM101541 2.3 0.991 -0.337 -1.032 -1.346 -0.554 -0.865 -1.584 -1.161 -1.343 -1.559 -1.437

BM103911 0.819 0.465 -0.428 -0.587 -0.752 -0.038 -0.384 -0.863 -0.487 -0.155 -0.506 -0.535

BM103974 1.257 0.9 -1.044 -1.302 -0.601 0.132 -0.154 -0.47 -0.763 -0.068 -0.279 -0.534

BM104033 1.39 1.531 -0.563 -0.536 -0.418 -0.093 -0.195 -0.779 -0.599 -0.593 -0.55 -0.86

BM104302 0.861 0.725 -0.393 -0.432 -0.775 -0.077 -0.339 -0.521 -0.208 -0.119 -0.257 -0.33

BM181897 1.535 -0.067 -2.066 -3.148 -2.698 -1.025 -1.697 -1.682 -2.225 -2.663 -2.114 -2.25

BM184003 1.279 0.656 -0.309 -0.472 -0.516 -0.191 -0.255 -0.509 -0.246 -0.549 -0.651 -0.699

BM184239 1.222 0.473 -0.083 -0.315 -0.187 0.253 0.231 0.454 -0.036 0.395 -0.155 -0.632

BM184281 1.568 0.863 -1.31 -1.657 -1.503 -1.042 -1.194 -1.397 -1.998 -2.06 -2.549 -2.474

BM185202 0.992 0.74 0.623 0.35 0.404 1.003 0.191 0.225 0.062 0.154 -0.023 0.068

BM185226 1.466 0.626 -0.27 -0.635 -0.804 0.04 -0.485 -0.558 -0.41 -0.516 -0.307 -0.391

Mean: degradation during to blastula 1.245 0.508 -0.587 -0.387 -0.5 -0.112 -0.354 -0.447 -0.503 -0.671 -0.68 -0.715

AI397333 1.109 0.61 0.607 0.501 0.011 0.307 0.04 -0.062 -0.204 -0.036 -0.261 -0.342

AI477049 1.156 1.11 0.066 -0.2 -1.625 -0.487 -0.529 -0.626 -0.669 -0.818 -0.966 -1.244

AI641069 1.506 1.293 0.691 0.133 0.316 0.196 0.049 0.789 0.038 -0.138 -0.08 -0.38

AI667581 0.894 0.927 0.458 -0.276 -0.69 -0.149 -0.436 -1.147 -0.888 -0.393 -0.511 -0.428

AI721290 1.106 0.677 0.457 0.154 0.115 0.6 0.309 0.751 0.166 0.345 0.116 -0.022

AI878452 1.379 1.165 0.538 0.081 -0.065 0.184 -0.133 -0.377 -0.604 -0.421 -0.686 -1.008

AI882824 1.786 0.943 -0.267 -0.413 -1.726 -0.571 -0.415 -1.532 -0.66 -0.357 -0.834 -2.305

AI883922 0.847 0.799 0.3 0.258 0.106 0.101 -0.093 0.31 0.141 0.832 0.497 0.142

AI959558 1.039 0.931 -0.212 -0.298 -1.983 -0.598 -0.162 -1.755 -0.545 -0.678 -1.556 -1.448

AJ011790 2.088 0.415 -1.765 -0.938 -3.901 -1.892 -1.495 -3.68 -1.577 -2.617 -3.243 -3.236

AJ249490 1.399 1.02 -0.788 -1.466 -3.217 -2.073 -3.416 -3.605 -3.504 -3.908 -3.409 -3.471

AW018983 0.996 0.135 -0.616 -1.432 -1.763 -0.094 -0.783 -0.985 -0.968 -0.501 -1.357 -0.675

AW076692 1.202 1.157 0.038 -0.015 -1.312 -0.671 -0.926 -0.785 -0.88 -1.108 -1.44 -1.304

AW077854 1.834 0.508 -1.713 -1.325 -3.11 -2.077 -2.814 -3.555 -3.362 -2.32 -2.268 -2.525

AW115638 0.745 0.585 0.262 -0.263 -1.417 -0.388 -0.35 -1.33 -0.996 -0.49 -1.111 -1.234

AW116489 1.35 -0.636 -0.185 -1.132 -3.283 -1.373 -1.47 -2.895 -1.823 -0.946 -3.801 -3.894

AW116567 1.859 0.877 -0.153 -0.337 -1.023 -0.115 -0.513 -0.708 -1.15 -1.072 -0.972 -1.428

AW116628 1.2 0.397 -0.749 -0.745 -1.591 -0.707 -0.858 -1.647 -1.073 -1.173 -1.751 -2.269

AW154176 1.711 0.948 -0.213 -0.455 -1.206 -0.534 -0.977 -1.441 -0.857 -0.953 -1.339 -1.037

AW154516 1.194 1.318 0.046 0.03 -0.267 -0.012 0.149 -0.107 0.021 -0.134 -0.105 -0.305

AW154647 1.185 1.14 0.501 -0.021 -0.476 -0.125 -0.429 -0.355 -0.2 -0.015 -0.234 -0.405

AW165310 1.087 0.328 0.017 -0.446 -1.489 0.082 -0.503 -1.317 -0.851 -1.073 -1.548 -1.22

AW171576 0.862 0.887 0.644 0.048 -0.2 0.491 0.061 -0.104 -0.117 0.015 -0.222 -0.374

AW344046 0.873 0.133 -0.311 -0.636 -0.747 -0.226 -0.738 -1.097 -1.121 -0.229 -0.4 0.214

AW344056 1.308 1.104 -0.298 -0.561 -0.7 -0.394 -0.882 -1.052 -0.839 -1.23 -1.038 -0.797

AW777332 1.016 0.832 0.545 0.641 0.034 0.457 0.007 0.423 -0.043 0.173 -0.087 -0.298

BE200811 1.244 1.355 0.051 -0.396 -1.075 -0.163 -0.715 -0.216 -0.532 -0.969 -1.025 -0.872

BE605880 1.461 0.71 0.952 0.299 -0.406 0.441 0.075 0.116 -0.562 -0.299 -0.958 -0.984

BE605975 0.907 0.582 0.67 0.275 -0.479 0.072 -0.463 -0.58 -0.289 -0.16 -0.398 -0.466

BG303177 1.036 1.072 0.352 -0.219 -0.262 0.158 -0.503 -0.678 -0.674 -0.716 -0.973 -0.807

BG303457 1.513 1.158 0.949 0.057 -0.933 -0.145 0.04 -1.041 -0.421 -0.812 -1.004 -0.23

BG303725 0.835 0.957 -0.289 -0.107 -0.642 0.069 -0.486 -0.267 -0.244 -0.426 -0.252 -0.247

BG303781 1.776 1.794 0.651 0.088 -1.185 -0.466 -0.841 -1.053 -0.629 -0.975 -1.447 -1.003

BG303890 1.146 1.225 -0.304 -0.368 -1.054 -0.749 -0.556 -1.412 -1.004 -0.688 -1.091 -0.974

BG304274 1.224 0.883 0.255 -0.153 -0.538 -0.022 -0.494 -0.336 -0.462 -0.269 -0.651 -0.594

BG727181 0.935 0.325 0.173 -0.461 -0.714 0.096 -0.273 -0.717 -0.254 -0.71 -0.318 -0.46

BI325685 1.853 1.552 0.338 0.256 0.034 0.266 -0.342 -0.018 -0.405 -0.571 -0.787 -0.621

BI474952 1.413 1.022 -0.627 -0.905 -1.733 -0.858 -1.057 -1.117 -1.67 -1.91 -0.923 -1.522

BI672630 1.316 0.721 0.035 0.044 -0.526 0.307 0.115 -0.289 -0.227 0.004 -0.26 -0.29

BI706176 1.306 0.974 0.416 0.283 0.195 0.546 0.235 0.712 0.209 0.414 0.109 -0.244

BI844059 1.075 0.423 0.204 0.057 -0.251 0.514 -0.1 -0.126 -0.041 -0.245 -0.703 -0.775

BI866724 1.389 1.367 0.559 0.235 -0.348 0.337 -0.099 0.118 -0.277 -0.685 -0.861 -0.739

BI867066 2.461 1.229 0.932 1.043 0.177 0.817 0.618 0.272 -0.129 -0.187 -0.988 -1.367

BI867089 1.554 0.931 -0.418 0.05 -2.49 -1.309 -0.972 -2.543 -2.112 -1.616 -3.469 -2.513

BI867264 1.498 0.429 -0.878 -0.609 -1.718 -1.304 -0.9 -2.424 -1.789 -1.439 -1.574 -1.503

BI877645 1.224 1.214 -0.086 -0.352 -0.771 -0.209 -0.649 -1.154 -0.782 -0.395 -0.935 -0.632

BI878123 1.271 0.863 0.149 -0.003 -1.054 -0.352 -0.319 -0.488 -0.325 -0.246 -0.706 -0.533

BI878184 1.909 0.576 -0.756 -1.595 -2.638 -0.905 -1.678 -2.749 -1.701 -1.636 -2.416 -2.149

BI878416 1.005 0.662 -0.301 -0.234 -1.042 0.019 -0.472 -0.827 -0.415 -0.506 -0.617 -0.717

BI878459 1.253 0.58 -0.488 -0.582 -1.302 -0.363 -1.204 -1.011 -1.376 -1.319 -1.473 -0.963

BI878480 1.737 0.964 -1.527 -1.73 -2.973 -0.797 -1.838 -3.785 -2.046 -3.77 -3.31 -2.247

BI878609 1.478 1.189 -0.064 -0.433 -1.371 -0.445 -1.174 -1.83 -0.873 -1.011 -1.253 -0.952

BI880780 0.921 0.921 0.089 0.079 -0.166 0.356 0.317 -0.129 -0.11 0.008 -0.316 -0.283

BI882464 0.828 0.735 0.208 0.082 -0.414 0.286 -0.135 -0.148 -0.261 -0.234 -0.329 -0.366

BI885469 1.024 0.804 -0.305 -0.021 -0.985 -0.608 -0.042 -0.935 0.109 -0.371 -0.663 -0.612

BI890907 0.984 0.878 0.485 0.094 -0.591 -0.08 -0.224 -0.317 -0.38 -0.46 -0.567 -0.366

BI891327 0.905 0.598 0.675 0.161 0.088 0.756 0.144 0.231 -0.043 -0.224 -0.65 -0.506

BI891821 1.695 0.27 -0.176 -0.463 -1.209 -0.806 -1.325 -2.47 -1.858 -2.457 -1.788 -2.243

BI891936 1.309 0.49 0.515 -0.145 -0.266 -0.111 -0.672 -1.057 -1.035 -0.098 0.051 -0.861

BI980160 1.132 0.971 0.026 -0.382 -0.848 -0.236 -0.409 -0.34 -0.393 0.324 -0.146 -0.402

BI980223 1.081 0.771 -0.267 0.057 -1.244 -0.174 -0.213 -0.973 -0.673 -0.379 -1.099 -0.473

BI980382 2.253 2.013 0.884 -0.582 -1.17 -0.77 -0.566 -1.708 -0.784 -1.789 -1.242 -1.611

BM035368 2.385 0.61 -0.55 -2.382 -2.723 -1.072 -1.127 -2.802 -1.128 -1.942 -2.409 -2.345

BM035598 2.406 1.217 -0.192 -0.935 -1.439 -0.536 -0.677 -1.384 -0.632 -0.288 -1.305 -0.818

BM036958 1.564 1.427 0.243 0.102 -0.265 0.144 -0.232 -0.346 -0.454 -0.222 -0.985 -0.699

BM071805 2.427 0.296 -2.048 -3.703 -4.472 -0.848 -1.633 -4.037 -1.943 -2.593 -3.908 -2.017

BM095365 1.303 0.251 -0.163 -0.389 -1.419 -0.022 -0.187 -0.909 -0.636 0.023 -0.224 -0.036

BM095366 0.922 0.83 0.77 -0.083 0.101 0.207 -0.121 0.264 -0.278 -0.265 -0.475 -0.456

BM104379 1.13 0.64 0.111 -0.368 -1.189 -0.148 -0.576 -1.185 -0.499 -0.487 -0.864 -0.666

BM156973 0.954 0.69 0.607 0.429 -1.245 -0.258 -0.408 -0.48 -0.455 -0.185 -0.228 -0.28

BM181627 1.124 1.28 -0.289 -0.611 -1.28 -0.455 -0.926 -0.996 -0.861 -0.854 -1.024 -0.734

BM181653 1.692 1.169 -1.147 -1.191 -1.975 -1.303 -2.028 -2.454 -2.436 -2.278 -3.029 -3.227

BM181708 1.848 -0.241 -0.72 -0.896 -2.613 -2.091 -2.234 -3.924 -2.403 -2.319 -3.433 -2.627

BM181792 1.903 0.429 -1.442 -1.394 -2.733 -2.416 -3.231 -3.422 -2.991 -2.4 -2.041 -2.725

BM182238 1.988 1.253 -0.441 -0.544 -2.303 -1.088 -0.497 -2.209 -1.117 -2.457 -1.286 -1.482

BM182849 0.933 0.427 -0.285 -0.298 -0.87 -0.223 -0.27 -0.464 -0.494 -0.209 -0.658 -0.233

BM183770 1.297 1.094 -0.433 -0.961 -1.126 -0.138 -0.753 -1.359 -0.924 -0.665 -1.248 -0.656

BM183795 1.595 0.601 -0.282 -0.342 -1.09 -0.357 -0.743 -1.472 -0.817 -1.783 -1.746 -1.28

BM184129 1.172 1.088 0.396 -0.374 -0.497 0.114 -0.311 -0.474 -0.267 -0.488 -0.482 -0.425

BM185181 1.101 1.021 0.293 -0.484 -2.334 -0.481 -0.637 -2.931 -1.535 -0.703 -2.689 -1.968

BM185291 1.825 0.297 -1.947 -1.845 -4.31 -1.753 -1.864 -4.659 -2.307 -3.591 -4.063 -3.301

Y12236 0.66 0.248 -0.083 -0.349 -0.664 -0.148 -0.557 -0.625 -0.313 -0.162 -0.503 -0.373

AJ245492 1.179 0.721 -0.139 -0.565 -1.051 -0.923 -1.24 -1.464 -1.131 -1.083 -1.054 -0.907

AW116159 1.488 1.578 0.779 0.535 0.19 0.538 0.499 0.98 0.446 0.249 -0.429 -0.713

AW171271 1.664 1.07 0.343 -0.052 -0.402 -0.243 -0.687 -0.567 -1.024 -0.674 -0.969 -0.797

AW171527 1.087 0.596 0.162 -0.191 -0.872 -0.524 -0.283 -1.113 -0.945 -1.678 -0.891 -0.938

BI843130 1.551 1.101 0.266 0.242 -0.313 -0.207 -0.467 -0.374 -0.531 -0.653 -1.025 -0.656

BI877328 1.4 1.403 1.243 1.146 0.507 -0.001 0.304 0.614 0.024 0.699 0.173 -0.589

BI879969 1.143 0.9 0.269 0.012 -0.233 -0.249 -0.249 -0.053 -0.129 -0.153 -0.221 -0.396

BI888350 2.148 1.536 0.863 0.877 0.35 0.466 0.282 0.266 -0.118 -0.257 -0.522 -0.675

BI979865 1.697 1.652 0.287 0.353 -0.658 -0.43 -0.53 -0.587 -0.578 -0.951 -0.981 -0.682

AW115523 1.088 0.534 -0.01 -0.053 -0.389 -0.21 -0.878 -0.58 -0.639 -0.778 -1.035 -1.285

AW154454 0.893 0.899 0.401 0.344 0.31 0.152 -0.201 -0.009 0.054 0.01 0.033 -0.244

BI843250 1.607 1.163 1.105 0.888 -0.183 0.037 -0.568 -0.396 -0.341 -0.232 -0.554 -0.528

AI721420 1.257 0.872 1.082 0.817 0.063 0.007 0.375 -0.285 0.145 0.274 -0.225 -0.173

AW116302 0.901 0.588 0.197 0.67 -0.341 -0.258 -0.527 -1.119 -0.751 -0.897 -1.146 -1.234

AW116402 1.066 0.444 0.065 -0.069 -1.057 -0.803 -0.762 -2.496 -1.213 -1.497 -1.206 -1.198

AW116414 1.933 1.471 0.758 -0.084 -1.78 -2.103 -2.741 -3.332 -2.848 -2.794 -2.393 -2.236

AW171350 1.248 1.013 0.57 0.506 0.028 0.403 -0.153 -0.214 -0.144 -0.16 -0.602 -0.568

AW173869 1.828 1.359 1.466 0.412 -0.882 -0.886 -1.453 -1.676 -1.255 -1.13 -0.905 -1.002

AW421436 1.679 0.731 -0.409 -0.955 -1.93 -1.966 -1.806 -3.551 -2.246 -3.567 -4.003 -3.458

AW422010 1.794 1.917 0.438 -0.188 -1.51 -1.231 -1.644 -2.43 -2.169 -2.328 -2.958 -2.831

BF717971 0.984 0.323 0.021 0.159 -0.782 -0.452 -1.076 -1.578 -0.769 -1.273 -1.054 -0.526

BI867396 0.764 0.498 0.132 0.03 -0.24 0.268 -0.2 -0.588 -0.374 -0.515 -0.814 -0.469

BI877523 1.02 0.786 0.839 0.448 0.347 0.521 -0.083 0.038 0.358 -0.003 0.119 0.062

BI885932 1.19 0.812 1.26 0.612 -0.24 -0.411 -0.307 -1.046 -0.455 -0.863 -1.182 -1.145

BI891978 1.221 0.845 0.38 0.241 0.11 0.28 -0.204 -0.165 -0.284 -0.536 -0.849 -0.824

BM082789 1.604 0.555 -0.173 -0.079 -1.14 -0.584 -0.727 -2.146 -1.103 -2.367 -1.677 -1.621

AB040435 0.917 0.251 -0.593 -1.18 -2.03 -1.511 -2.238 -2.456 -3.02 -3.079 -3.34 -3.966

AI545168 0.937 0.541 0.466 0.31 -0.092 -0.243 -0.519 -0.385 -0.601 -0.119 -0.389 -0.08

BG305492 1.541 1.562 1.072 0.726 0.65 0.61 0.34 0.248 -0.272 0.141 -0.226 0.124

BM104357 1.225 1.297 0.688 0.342 0.189 -0.025 -0.108 -0.129 -0.165 -0.412 -0.404 -0.388

BM182720 1.721 1.553 0.166 -0.077 -0.855 -0.419 -1.185 -1.52 -1.158 -2.395 -1.831 -1.489

BM183873 1.345 1.011 0.43 0.097 -0.913 -0.762 -0.561 -1.652 -1.323 -1.921 -1.736 -2.218

BM184046 1.71 1.329 0.293 -0.118 -0.62 -0.375 -1.044 -1.032 -2.037 -2.516 -1.971 -2.401

U00931 2.166 1.372 0.777 0.047 -1.133 -1.523 -2.244 -1.924 -2.301 -3.19 -2.85 -3.045

AW076961 1.075 0.522 0.16 0.213 -0.226 -0.523 -0.506 -1.517 -1.53 -1.377 -1.658 -0.503

BI876830 0.795 0.666 0.735 0.684 -0.407 -0.326 -0.802 -1.161 -0.855 -1.196 -1.478 -1.345

BI886717 1.01 0.515 1.119 0.634 0.676 0.434 0.067 0.116 -0.027 -0.076 -0.458 -0.204

BI889101 1.659 1.234 1.209 1.308 1.215 0.594 0.293 0.671 -0.054 -0.595 -0.57 -0.333

BI983378 0.832 0.685 0.463 0.348 -0.026 -0.009 0.041 -0.348 -0.343 -0.041 -0.616 -0.137

AI641473 1.063 0.762 0.759 0.827 0.605 0.904 0.406 0.505 -0.025 -0.104 -0.451 -0.785

AI667671 1.183 1.232 0.721 0.68 0.547 0.379 0.529 0.827 0.101 0.087 -0.065 -0.215

AI957426 1.179 0.865 1.032 0.998 0.961 0.727 0.165 -0.115 -0.208 0.01 -0.533 -0.366

AW076546 1.451 1.47 0.702 0.14 -0.213 0.437 -0.097 -0.236 -0.667 -0.789 -1.396 -1.537

AW116246 1.129 0.992 0.856 0.432 0.022 -0.206 -0.124 -0.239 -0.397 -0.95 -0.647 -0.967

AW128153 1.168 1.028 0.75 1.014 0.746 1.113 0.461 0.406 0.008 0.193 -0.029 -0.559

AW171522 1.28 1.091 0.7 0.663 0.44 0.3 0.092 0.137 -0.206 -0.308 -0.285 -0.879

AW344043 1.545 1.541 0.894 0.82 0.832 1.091 0.412 0.715 0.071 0.102 -0.204 -0.563

AW420717 1.25 1.2 1.056 1.301 0.287 0.397 0.228 0.172 0.058 -0.424 -0.866 -0.685

AW777876 1.003 0.842 0.983 0.806 0.403 0.547 0.241 0.682 0.255 0.644 0.173 -0.164

BI673444 0.943 1.002 0.207 0.268 0.012 -0.086 0.058 -0.224 -0.281 -0.241 -0.759 -0.892

BI877999 1.023 1.094 0.349 0.703 0.366 0.001 0.097 -0.29 -0.3 -0.249 -0.722 -0.776

BI888742 0.814 0.583 0.608 0.519 0.187 0.041 0.116 -0.115 -0.204 -0.376 -0.719 -0.635

BI888943 1.481 0.606 0.323 0.403 -0.357 -0.634 -1.182 -1.478 -1.297 -1.547 -1.683 -2.113

BI979304 1.243 1.111 0.957 1.055 0.715 0.484 0.235 0.77 0.15 0.232 -0.186 -0.215

BI980644 1.837 0.896 2.001 0.889 0.66 0.612 0.411 0.571 -0.091 -0.731 -0.628 -1.054

BM102116 0.877 0.83 0.798 0.588 0.242 0.187 0.117 0.531 -0.06 -0.189 -0.125 -0.199

BM104048 1.083 1.044 0.581 0.284 0.596 0.309 -0.057 0.111 -0.664 -0.49 -0.678 -1.118

BM104515 2.322 1.2 1.154 0.776 0.538 0.693 -0.183 -0.586 -1.334 -2.04 -2.601 -2.214

BM182245 1.419 1.422 1.112 0.622 0.003 -0.331 -0.21 -1.164 -0.94 -0.984 -1.492 -1.459

BM184757 1.62 1.236 0.734 0.619 0.326 0.243 -0.424 -0.487 -0.62 -0.938 -1.328 -1.145

Mean: degradation during subsequent stages 1.339 0.897 0.21 -0.06 -0.742 -0.243 -0.535 -0.912 -0.76 -0.838 -1.094 -1.056
