# Supplementary material for: Transcriptome Analysis of Zebrafish Embryogenesis Using Microarrays
Source: PLoS Genet. 2005 Aug 26;1(2):e29. doi: 10.1371/journal.pgen.0010029 (PMC1193535; doi:10.1371/journal.pgen.0010029)
Supplement: Dataset S4 — (191 KB DOC) [file pgen.0010029.sd004.doc]

Dataset S4. List of genes with onset of transcript accumulation at the blastula stage and peak

of expression at gastrula stages.

Genbank IDUF egg 3hpf 4.5hpf 6hpf 7.7hpf 9hpf 10.7hpf 12hpf 15hpf 24hpf 30hpf 48hpf

AA494837 0.897 0.492 0.584 1.001 0.972 0.092 0.701 1.004 -0.08 0.271 0.165 0.194

AA497205 -0.508 -0.123 0.87 1.577 1.563 0.152 0.435 1.465 0.39 0.438 0.601 0.215

AB011826 0.083 0.58 0.583 0.899 0.865 0.125 0.202 0.154 -0.064 0.099 0.182 -0.196

AB017117 0.082 0.723 1.193 1.264 0.747 1.196 0.673 0.649 0.267 0.523 0.127 -0.002

AB032415 -0.434 0.201 0.524 0.563 0.55 0.183 -0.264 -0.184 0.234 0.203 0.128 0.109

AF034606 -0.728 0.076 2.492 3.041 2.51 1.905 0.929 2.251 0.723 0.101 0.023 -0.316

AF062643 -0.284 0.202 0.658 0.743 0.637 0.505 0.166 0.346 -0.022 0.087 0.51 0.013

AF077225 -0.605 0.807 4.683 4.967 4.68 2.241 1.615 3.264 0.636 -0.037 -0.017 -0.295

AF124396 -0.369 -0.12 0.494 0.799 0.75 0.38 0.329 0.678 0.139 0.258 0.455 0.172

AF135438 -0.29 0.726 1.255 1.48 1.326 0.28 -0.144 1.137 0.438 0.387 -0.051 -0.181

AF139990 1.639 1.578 1.918 1.939 1.601 1.011 0.602 0.876 0.101 -0.02 -0.364 -0.807

AF140608 -0.054 0.063 0.275 1.049 1.012 0.39 0.447 0.915 0.135 0.612 0.499 0.233

AF168614 -0.314 0.874 0.981 1.126 1.105 0.882 0.512 0.686 0.375 0.042 -0.362 -0.728

AF177868 -0.136 0.466 0.868 1.318 0.615 1.058 0.538 0.727 0.537 0.238 0.179 0.272

AF207751 -0.462 0.804 4.331 4.013 3.902 2.737 1.996 3.268 1.29 1.233 1.19 0.352

AF210646 -0.043 0.54 -0.018 0.754 0.563 -0.3 0.069 0.333 -0.092 0.036 -0.026 -0.144

AF211852 0.535 0.542 2.347 2.444 2.201 1.979 1.774 1.813 0.951 0.925 0.525 0.287

AF240772 -0.382 0.015 0.807 1.661 1.515 0.753 0.684 1.179 0.527 -0.004 0.594 0.138

AF246184 -0.459 -0.408 -0.091 0.486 0.46 -0.09 0.235 0.061 0.036 -0.157 -0.106 -0.118

AF286364 -0.596 -0.075 0.967 1.782 1.228 0.245 0.227 0.516 0.605 -0.278 0.827 -0.003

AF286375 -1.659 -2.38 0.139 0.622 0.526 0.043 -0.247 -0.318 -0.421 -0.229 -0.353 -0.213

AF354754 -0.329 0.69 1.428 1.844 1.929 0.808 0.183 1.064 0.376 0.205 0.769 0.076

AF359424 -0.764 -0.125 1.203 1.285 1.086 0.495 0.352 0.713 0.075 0.048 0.386 0.002

AF375226 -0.185 0.188 0.395 1.056 1.037 -0.039 0.364 0.808 0.131 0.035 -0.108 0.005

AF387342 -0.103 0.646 0.609 0.746 0.591 0.147 0.347 0.341 0.181 -0.206 -0.221 -0.887

AF414110 1.6 1.424 1.844 1.89 1.164 1.489 1.107 1.006 0.102 0.158 -0.195 -0.099

AI416261 -1.02 -0.431 0.521 1.226 1.196 0.436 0.383 0.878 0.338 -0.039 0.046 -0.145

AI477247 0.545 0.955 0.938 1.03 0.438 0.031 0.137 0.404 0.128 0.049 -0.452 -0.538

AI477969 -0.728 1.013 2.31 2.843 1.995 0.108 -0.083 0.553 -0.378 -0.851 -0.652 -0.636

AI544535 0.64 1.491 2.202 2.273 1.915 0.284 0.465 0.467 -0.113 0.054 0.084 -0.036

AI544688 -0.728 0.757 1.734 2.33 1.529 0.394 0.62 0.543 0.217 -0.471 0.453 -0.173

AI558282 -0.201 0.225 0.372 0.829 0.701 -0.136 0.126 0.435 0.166 -0.009 0.4 0.559

AI584348 -0.017 0.242 0.541 0.778 0.689 -0.188 0.175 0.752 -0.031 -0.2 -0.164 0.086

AI584379 0.305 0.545 0.513 1.477 0.34 0.42 0.693 0.49 0.33 -0.158 0.567 0.773

AI584395 0.621 0.697 0.736 0.986 0.218 0.173 -0.126 0.014 -0.01 0.19 0.158 0.417

AI584401 0.668 0.665 1.474 1.55 1.52 1.443 1.105 1.153 0.259 0.261 -0.294 -0.32

AI585168 -0.114 0.263 0.484 0.714 0.254 0.052 0.151 0.436 0.164 0.548 0.229 -0.24

AI588095 -0.27 0.29 1.117 1.12 1.265 -0.232 0.538 0.639 0.24 0.111 0.461 0.043

AI588743 -1.066 -0.039 0.733 1.169 0.949 0.389 0.062 0.22 0.937 0.386 0.074 -0.843

AI588792 -0.958 0.621 3.218 3.383 3.336 1.811 1.388 2.132 2.479 1.48 0.833 0.44

AI626435 0.045 1.336 1.606 2.652 2.284 0.922 0.828 1.492 0.242 0.02 0.464 -0.196

AI626599 -0.023 -0.314 1.163 1.64 1.403 1.238 0.924 1.662 0.66 0.414 0.029 0.048

AI641066 -0.024 0.629 1.026 1.216 0.744 0.113 0.125 0.613 0.116 0.402 0.32 -0.095

AI641129 0.016 -0.176 1.639 1.64 1.296 0.248 0.447 0.561 -0.086 -0.352 -0.555 -0.583

AI641454 0.093 0.655 1.033 1.221 1.018 0.028 0.224 0.41 -0.091 -0.049 -0.455 -0.218

AI641480 -0.682 0.079 0.893 2.363 2.239 0.221 0.022 0.248 -0.235 -0.799 0.107 0.018

AI667326 0.028 0.772 1.289 1.623 0.868 1.361 0.476 0.787 0.16 0.148 0.043 -0.155

AI667403 0.116 0.165 2.419 2.743 1.961 1.197 0.527 0.779 0.42 0.85 0.166 0.691

AI667530 -0.08 0.15 1.032 0.963 0.85 0.448 0.312 0.498 0.03 -0.121 -0.711 -0.293

AI722421 -0.653 0.171 0.763 0.981 0.783 0.17 -0.085 0.665 0.125 0.346 -0.166 0.213

AI722567 -0.036 -0.045 1.117 1.003 0.878 0.75 0.565 0.507 0.532 0.076 0.232 -0.438

AI793666 0.02 0.489 0.931 1.336 0.951 0.277 0.546 0.361 0.269 -0.137 -0.309 -0.304

AI793701 0.068 0.575 0.466 1.301 0.962 0.516 0.435 0.978 0.321 0.376 0.286 0.09

AI793925 -0.348 0.651 0.648 0.857 0.685 0.131 -0.152 0.096 -0.384 -0.216 0.318 -0.309

AI794024 -0.839 -0.043 2.469 2.163 2.17 1.952 1.496 1.687 0.706 1.009 1.093 0.425

AI794547 -0.113 0.352 0.066 0.584 0.47 0.178 0.255 0.087 0.022 0.047 -0.093 -0.139

AI794634 -0.361 0.948 0.696 1.214 1.183 0.241 0.386 0.883 0.14 -0.056 -0.31 -0.107

AI877506 1.136 1.232 1.428 1.545 1.507 1.297 1.195 1.471 0.714 0.654 -0.292 -0.84

AI878080 0.046 0.113 0.796 0.813 0.584 0.409 0.225 0.889 0.133 0.341 0.168 0.293

AI883980 -0.404 0.422 0.543 0.65 0.609 0.128 0.452 0.363 0.254 -0.305 0.261 -0.311

AI884177 -0.723 0.173 1.481 1.658 1.686 0.137 -0.12 0.193 -0.299 -0.609 -0.086 -0.222

AI884211 0.299 1.061 1.446 1.511 0.686 0.695 0.184 0.675 0.208 0.447 -0.169 -0.439

AI957847 -0.228 0.331 0.561 1.037 0.834 0.433 0.431 0.776 0.008 0.103 -0.09 -0.066

AI957850 0.06 0.696 0.927 0.997 0.872 0.221 -0.097 0.402 0.122 -0.399 0.011 -0.42

AI958373 -0.53 0.308 0.559 0.889 0.912 0.221 0.122 0.998 0.228 0.022 -0.37 -0.95

AI958627 0.025 0.469 1.697 1.959 1.904 1.67 1.284 1.064 0.465 0.561 0.327 0.581

AI959694 0.345 -0.045 1.047 1.182 0.737 0.699 0.446 0.258 0.091 0.604 0.368 -0.139

AI964189 0.267 1.099 1.988 1.98 1.928 1.729 1.553 1.695 0.454 0.816 0.278 -0.108

AI964207 0.285 0.825 0.812 0.869 0.825 -0.043 0.347 0.461 -0.125 0.009 -0.226 -0.162

AI964367 0.249 -0.308 0.948 1.216 0.423 0.927 0.368 0.679 0.749 0.902 0.618 0.411

AI965130 -0.282 0.467 0.761 0.863 0.579 0.127 -0.046 0.271 0.01 0.397 0.201 0.107

AI974195 0.266 0.98 0.663 1.144 0.905 0.396 0.456 0.809 -0.223 0.42 0.598 0.389

AL591442 -1.908 -0.526 1.339 1.685 1.353 1.079 0.71 0.85 0.871 0.333 0.797 0.273

AW018998 -0.728 0.148 0.766 0.85 0.813 0.046 -0.219 0.193 -0.167 -0.36 0.147 -0.194

AW019421 -0.211 0.709 1.595 1.739 1.058 0.417 0.061 0.046 0.095 -0.43 -0.882 -1.063

AW019725 0.578 -0.764 2.338 2.888 2.418 2.453 1.632 1.454 0.519 1.512 1.026 0.657

AW019740 -0.814 -0.001 0.605 1.051 0.851 0.495 0.202 0.344 -0.231 -0.289 -0.208 -0.379

AW058811 -0.754 -0.511 1.928 2.388 2.487 0.996 0.579 0.318 -0.344 0.012 -0.306 -0.693

AW058867 -0.019 0.25 0.637 1.037 0.693 0.169 0.39 0.537 0.218 0.375 0.227 0.029

AW059007 -0.846 -0.118 1.549 1.923 1.99 0.899 0.536 1.01 0.102 0.318 0.281 -0.107

AW076798 -0.235 0.403 0.88 0.938 0.935 0.673 0.1 0.756 0.402 0.116 -0.485 -0.856

AW078306 0.154 0.721 1.511 1.672 1.524 1.603 1.117 1.374 0.991 1.262 0.454 0.458

AW078394 0.28 0.389 0.75 1.017 0.814 0.722 0.18 0.624 0.426 0.583 0.836 0.781

AW115560 0.891 1.376 1.756 2.224 1.903 1.805 0.949 1.514 0.489 0.359 -0.054 -0.395

AW115633 0.286 0.317 0.44 0.746 0.576 0.451 0.227 0.792 0.258 0.583 0.078 0.153

AW115702 0.163 0.232 0.522 0.899 0.828 0.165 0.161 -0.161 -0.216 -0.292 -0.356 -0.463

AW116075 1.269 1.205 1.827 1.985 1.811 1.349 0.586 -0.015 -0.933 -1.598 -1.821 -2.886

AW116371 0.388 1.274 1.625 1.818 1.451 1.261 0.844 1.121 0.235 0.08 -0.079 -0.26

AW116461 0.129 0.9 1.408 1.805 1.712 1.554 0.77 1.263 0.443 0.256 0.028 -0.315

AW116601 0.186 0.207 1.063 1.001 0.955 0.32 0.297 0.793 0.121 0.583 0.617 0.508

AW116726 -0.351 -0.017 0.796 0.813 0.59 -0.145 -0.177 0.122 0.005 -0.451 -0.668 -0.484

AW116915 0.468 0.606 0.639 0.707 0.557 0.248 0.419 0.443 0.041 0.092 -0.134 -0.255

AW117141 -0.08 -0.119 1.599 1.511 1.376 1.157 0.727 1.056 1.265 0.589 0.735 0.823

AW128002 -0.302 0.68 1.494 1.649 1.253 -0.149 0.084 0.553 -0.099 -0.015 0.238 -0.187

AW154285 0.283 0.755 0.772 0.995 0.463 0.346 0.399 0.391 -0.088 0.259 -0.431 -0.233

AW154483 -0.008 0.592 0.621 0.996 0.542 0.797 0.594 0.295 -0.143 0.018 -0.268 0.262

AW154506 0.911 0.729 0.818 1.033 0.305 -0.072 -0.388 -0.177 -0.137 0.204 0.005 -0.171

AW165221 0.961 0.715 0.915 1.065 1.049 0.346 0.69 0.683 0.102 0.169 -0.199 -0.028

AW165240 0.257 1.152 1.488 1.474 1.365 0.905 0.371 0.847 0.003 -0.287 -0.314 -0.108

AW165301 -0.464 0.498 0.736 0.873 0.711 -0.261 0.379 0.268 -0.18 -0.267 -0.249 0.042

AW165417 0.12 0.884 1.787 2.045 2.015 1.164 0.775 1.768 0.595 0.505 -0.232 -0.578

AW170898 0.34 0.706 0.471 1.125 1.136 0.249 0.446 0.729 0.321 0.162 -0.147 0.127

AW170975 0.296 0.239 0.463 0.615 0.485 0.596 0.405 0.28 0.154 0.018 0.087 0.004

AW232627 1.073 0.833 2.196 2.255 1.786 2.237 1.956 1.376 0.474 0.525 0.195 0.267

AW233144 -0.01 0.402 0.551 0.852 0.818 0.541 0.637 0.773 0.181 0.319 0.356 0.094

AW233689 0.36 0.274 0.995 1.173 0.84 0.594 0.389 0.708 0.256 0.293 -0.177 -0.289

AW280152 -0.138 -0.035 -0.291 1.18 0.712 0.174 -0.05 0.237 0.054 0.027 -0.049 0.533

AW281574 -0.364 -0.491 0.221 0.648 0.66 -0.027 0.135 0.515 -0.128 0.514 0.255 0.479

AW281646 0.03 0.884 1.149 1.438 0.841 0.748 0.273 0.726 0.004 0.233 0.127 0.164

AW306107 1.057 1.458 2.09 2.065 1.75 1.318 0.388 0.803 -0.001 -0.345 -0.923 -1.164

AW342711 0.508 -0.05 -0.042 1.87 1.368 -0.159 -0.377 -0.285 -0.515 -0.595 -0.612 -0.251

AW344020 -0.509 1.088 0.972 1.342 1.013 -0.309 0.379 0.502 -0.125 -0.528 0.064 -0.242

AW344023 -0.728 -0.036 0.097 1.118 0.956 -0.451 -0.139 -0.636 -0.105 -1.127 -1.415 -1.233

AW344143 0.27 0.507 0.371 0.702 0.512 0.155 0.4 0.572 0.111 0.242 0.142 -0.029

AW344255 0.409 0.639 0.828 0.945 0.859 0.828 0.619 0.692 0.465 0.237 -0.002 -0.322

AW422167 0.731 -0.04 0.759 1.17 1.098 1.125 0.506 0.501 -0.146 -0.48 -0.834 -0.624

AW422582 -0.076 0.744 0.563 1.024 0.804 0.204 -0.127 -0.137 0.069 -0.332 -0.581 -0.557

AW423211 -1.426 -1.134 1.503 1.609 1.428 1.211 0.814 0.129 0.303 0.299 -0.244 -0.272

AW454617 -0.549 0.479 1.151 1.328 1.002 0.716 0.574 0.596 0.144 0.368 -0.085 -0.039

AW566851 1.424 1.352 2.148 2.218 1.897 2.049 1.666 1.407 0.71 0.566 0.012 -0.275

AW567345 0.624 0.916 1.29 1.332 0.75 0.073 0.493 0.541 -0.015 -0.075 -0.27 -0.27

AY057095 -0.437 0.676 2.997 2.712 2.818 1.017 1.3 2.482 0.545 0.658 0.487 0.149

BE017542 0.198 0.658 1.045 1.212 0.215 0.092 0.608 0.938 0.095 0.397 -0.065 -0.581

BE201475 0.179 0.423 2.574 2.595 2.318 0.907 0.997 1.851 0.906 0.184 -0.171 -0.336

BE557115 -0.035 1.025 2.097 2.263 1.617 0.521 0.387 0.596 0.108 -0.347 -0.253 -0.564

BE605308 0.811 0.531 1.466 1.511 1.174 1.37 0.579 0.402 0.015 -0.711 -1.254 -1.466

BE605766 -0.173 0.309 0.738 1.079 0.856 0.166 0.349 0.623 0.224 0.666 0.222 0.121

BE693173 0.389 0.159 1.046 1.356 0.934 1.353 0.592 0.566 0.136 -0.153 -0.532 -0.696

BF157279 -0.142 0.679 0.397 1.203 0.927 -0.017 0.039 0.474 -0.151 -0.223 0.243 -0.343

BF713861 0.139 0.824 1.033 1.192 0.748 -0.046 0.013 -0.355 0.024 -0.061 -0.231 -0.51

BF713867 -0.4 0.541 1.484 1.659 1.362 0.546 0.344 0.536 0.561 0.003 -0.298 -0.571

BF938808 -0.731 -0.028 1.161 1.773 1.582 1.521 0.905 1.267 0.223 0.566 0.226 -0.574

BG302933 -0.13 0.263 0.743 0.865 0.62 -0.284 0.01 0.291 0.278 0.192 0.105 0.3

BG303239 0.086 0.414 1.064 1.213 1.279 0.449 0.126 0.698 -0.045 -0.039 -0.035 -0.279

BG303391 0.065 1.113 1.439 1.436 1.456 -0.087 0.349 0.716 -0.202 -0.574 -0.476 -0.442

BG303594 -0.103 0.89 0.926 1.015 0.401 0.814 0.463 0.441 -0.046 -0.418 -0.094 -0.303

BG303647 0.794 1.098 1.247 1.363 0.91 1.352 0.62 0.589 0.348 0.344 -0.077 -0.175

BG303768 0.868 0.85 0.981 1.233 0.713 0.203 0.541 0.267 -0.02 -0.057 -0.838 -0.632

BG303999 0.185 0.386 1.173 1.145 0.879 0.708 0.519 0.838 0.81 0.809 0.41 0.726

BG304149 0.807 1.426 1.581 1.624 1.508 1.442 0.85 1.103 0.028 0.215 -0.289 -0.133

BG305572 -0.611 -0.194 1.141 1.774 1.391 0.352 0.622 1.448 0.955 0.751 0.348 -0.521

BG307024 0.822 1.055 1.072 1.249 0.53 0.555 -0.024 0.024 0.024 -0.044 -0.158 -0.304

BG308220 -0.434 0.425 0.687 1.041 0.981 -0.019 0.489 0.61 0.129 -0.038 -0.235 -0.276

BG727249 0.443 0.606 2.247 2.163 1.354 0.72 0.979 0.502 0.3 -0.084 -0.675 -0.313

BG737927 -0.728 0.545 0.666 0.969 1.115 -0.29 -0.053 0.618 0.146 -0.717 0.122 -0.25

BG799357 -0.163 0.138 0.89 1.221 1.239 -0.105 0.059 0.31 -0.052 -0.222 -0.117 -0.171

BG883304 -0.547 0.453 1.935 1.845 1.97 0.81 0.118 0.299 -0.127 -0.022 -0.641 -0.355

BG883685 -0.128 0.513 0.885 1.296 1.307 0.222 0.424 1.052 0.23 0.001 0.096 -0.074

BG985501 -0.387 0.668 1.113 1.281 1.266 1.107 0.647 1.369 0.022 0.801 0.656 0.337

BG985522 -0.791 -0.142 1.535 1.639 1.122 0.92 0.337 0.759 0.492 0.211 0.549 0.071

BG985787 0.125 1.156 1.189 1.268 1.38 0.354 0.424 0.699 0.187 -0.337 -0.627 -1.127

BI325924 0.53 1.193 1.161 1.4 0.687 0.088 -0.207 -0.025 -0.154 -0.924 -0.464 -0.766

BI428543 0.045 -0.195 0.882 1.007 0.864 0.855 0.739 0.559 0.127 0.461 0.017 0.457

BI428991 -0.483 0.232 1.546 1.819 1.482 0.424 0.317 0.747 -0.049 0.313 0.469 0.58

BI474928 -0.704 -0.286 0.407 0.571 0.562 -0.065 0.371 0.294 -0.225 -0.502 0.161 -0.139

BI475873 -0.375 0.95 2.319 2.254 1.475 0.507 0.473 0.399 0.077 -0.174 -0.522 -0.365

BI670969 0.465 0.996 0.739 1.126 0.581 0.955 0.51 0.999 0.635 0.901 0.381 0.071

BI672214 -0.075 0.335 0.66 0.786 0.62 0.046 0.149 0.499 0.06 0.154 0.19 0.183

BI673487 0.207 0.846 1.328 1.305 1.15 0.35 0.874 1.165 0.426 0.401 -0.047 -0.586

BI673772 0.224 0.062 0.624 0.665 0.526 0.323 0.399 0.605 0.165 0.634 0.272 -0.05

BI704199 0.322 -0.087 0.861 1.916 1.706 -0.142 0.45 1.02 0.116 0.357 0.209 -0.02

BI704267 0.288 1.392 1.552 1.613 1.286 0.773 0.23 1.092 0.071 0.522 0.147 0.173

BI704278 0.235 0.826 1.924 2.043 1.917 1.005 1.357 0.819 0.016 0.76 0.369 -0.452

BI704334 -0.37 -1.092 0.676 1.06 0.673 -0.031 0.064 -0.185 -0.454 -0.508 -0.564 -0.576

BI704344 -0.438 -0.08 2.02 2.297 2.045 2.031 1.203 1.416 0.997 0.779 0.709 0.558

BI704373 0.171 0.492 0.906 1.354 1.233 0.689 0.306 0.779 0.173 0.215 0.042 -0.147

BI705506 -0.592 -0.513 0.105 0.828 0.714 0.566 0.508 0.633 0.291 0.431 0.032 0.169

BI708766 -1.55 -0.564 0.724 0.98 0.452 0.329 -0.011 0.701 0.229 0.753 0.055 0.106

BI710730 -0.712 0.204 1.652 1.848 1.684 0.801 0.598 1.066 0.859 0.378 -0.424 -1.049

BI843117 0.359 0.803 0.531 1.109 0.83 0.267 0.244 0.644 -0.102 0.063 -0.24 -0.069

BI864988 0.02 0.033 0.816 1.309 0.955 0.46 0.297 0.755 0.245 -0.151 0.057 -0.095

BI865976 -1.248 -0.042 0.828 1.11 0.892 0.673 0.411 -0.5 0.548 -0.104 0.274 -0.466

BI866879 0.177 -0.048 1.255 1.347 1.475 0.306 0.033 0.297 -0.343 -0.067 0.019 -0.558

BI867531 0.341 0.565 0.41 0.94 0.951 0.204 0.402 0.781 0.256 0.512 0.358 0.259

BI867929 0.542 0.533 0.161 0.643 0.646 -0.096 0.283 0.364 -0.045 -0.081 -0.071 -0.08

BI875704 -0.124 0.598 0.496 0.893 0.865 0.517 0.335 0.647 -0.175 0.01 -0.064 -0.166

BI876201 -0.562 0.025 0.774 0.737 0.7 0.026 0.41 0.216 0.72 -0.006 -0.442 -1.233

BI877866 0.366 0.664 1.018 1.156 1.034 0.352 0.335 0.594 0.302 0.265 -0.122 0.041

BI877985 0.758 1.496 1.147 1.963 1.511 0.629 0.279 0.806 -0.129 -0.47 -0.379 -0.399

BI878043 -0.369 -0.754 0.427 0.8 0.621 0.772 0.387 0.374 0.361 0.452 0.319 0.258

BI878059 -0.728 0.579 0.735 1.232 0.758 -0.045 0.161 0.699 0.149 -0.099 0.369 -0.178

BI878475 0.2 0.725 1.388 1.372 0.973 0.497 0.327 0.758 0.358 0.094 0.006 0.421

BI878743 0.242 0.682 0.711 0.989 0.636 0.184 0.506 0.77 0.238 -0.29 -0.141 -0.012

BI878819 0.824 0.696 0.724 0.957 0.015 0.426 0.027 0.157 -0.369 -0.176 -0.423 -0.262

BI878854 -0.1 0.766 1.531 1.757 1.699 1.258 0.966 1.081 0.572 0.723 0.392 -0.175

BI878903 0.185 0.704 0.537 1.096 0.767 0.332 0.123 0.04 -0.197 -0.152 -0.45 -0.526

BI878961 0.401 0.722 0.766 1.204 1.039 0.418 0.324 0.807 0.396 -0.111 -0.337 -0.117

BI879444 1.036 0.868 2.755 3.127 2.955 1.676 2.028 1.635 0.746 1.122 0.848 0.509

BI879523 0.861 0.475 0.677 0.997 0.076 -0.157 0.222 0.134 0.091 0.461 -0.132 -0.18

BI879928 0.615 0.789 0.906 1.547 1.447 0.64 0.681 0.998 -0.275 -0.249 -0.383 -0.363

BI880060 -0.347 -0.03 1.283 1.413 1.261 1.186 0.2 0.803 0.172 0.399 0.175 0.138

BI880302 -0.238 0.096 0.725 1.278 1.214 0.003 0.119 0.406 0.273 0.174 -0.395 -0.596

BI880524 -3.095 -1.639 1.033 1.548 1.109 0.924 0.341 0.306 -0.034 0.784 0.765 0.84

BI884752 -0.201 0.837 1.809 1.89 1.095 -0.149 0.152 0.159 0.067 0.326 -0.21 -0.341

BI885653 -3.548 -1.131 3.457 3.722 2.8 1.754 1.059 1.023 0.56 0.958 0.877 1.316

BI885886 -0.008 -0.184 0.859 1.769 1.423 0.701 1.15 0.785 0.503 0.353 0.162 0.115

BI885889 -0.728 0.21 1.711 1.772 1.218 0.307 -0.219 0.34 -0.255 -0.143 0.55 -0.112

BI885991 0.748 0.839 0.752 1.797 1.704 0.996 0.776 0.998 0.124 0.141 -0.125 -0.179

BI885993 0.458 0.852 1.015 1.554 1.256 -0.038 0.449 1.062 0.038 0.385 0.235 -0.062

BI886006 -0.164 -0.351 1.008 1.247 1.213 -0.029 0.205 0.741 0.111 0.221 0.325 -0.037

BI886016 -0.365 0.292 0.635 1.121 0.791 0.647 0.738 0.923 0.476 0.376 0.054 0.002

BI886020 0.569 1.643 1.901 1.967 1.581 1.595 1.053 0.74 0.327 0.175 0.308 0.078

BI886133 -0.728 0.545 0.407 1.341 1.131 0.022 -0.089 0.193 -0.328 -0.749 -0.129 -0.358

BI886353 -0.033 1.17 0.83 2.112 1.837 0.88 0.609 1.248 0.004 0.094 -0.214 -0.289

BI886376 1.216 0.636 1.462 1.908 1.428 0.963 0.373 0.512 0.126 0.223 -0.225 -0.918

BI886477 0.255 0.374 0.554 1.213 0.652 -0.071 0.353 0.833 0.133 0.267 0.194 0.087

BI886522 -0.132 0.632 0.835 0.948 0.892 -0.14 0.048 0.332 0.006 0.041 -0.199 -0.183

BI886564 -0.411 -0.067 0.52 0.568 0.631 -0.11 0.306 0.395 0.259 0.09 -0.117 -0.405

BI886677 -0.5 0.454 1.876 2.443 1.798 0.792 0.009 1.232 0.844 0.558 0.482 0.224

BI886691 -0.079 0.215 0.428 0.943 0.605 0.185 0.293 0.32 0.099 0.086 -0.16 -0.301

BI886702 -0.107 0.2 0.151 2.293 1.7 0.482 0.074 -0.028 -0.148 -0.805 -1.051 -1.286

BI886766 -0.098 0.955 1.181 1.193 1.04 0.896 0.096 0.531 0.359 -0.058 -0.329 -0.505

BI886779 -0.012 -0.02 0.709 1.092 0.747 0.35 0.34 0.279 0.009 -0.055 -0.199 -0.026

BI886789 -2.881 -1.165 3.378 3.291 2.527 1.698 0.991 0.476 0.471 0.962 0.918 1.344

BI886811 -0.502 -0.102 0.448 1.216 1.217 -0.204 0.306 0.264 -0.254 -0.61 0.036 -0.293

BI886936 -1.375 -0.076 2.026 1.845 1.802 1.209 0.3 0.923 1.111 0.66 0.508 0.539

BI887133 -0.089 0.527 1.49 1.59 1.409 1.602 1.022 0.723 0.755 0.582 0.162 -0.018

BI887309 -0.035 -0.251 0.865 0.795 0.768 0.277 0.087 0.769 0.231 0.333 -0.219 -0.197

BI887338 0.599 0.324 1.356 2.578 1.4 1.321 1.149 1.225 0.969 0.494 0.025 -0.086

BI887480 -0.005 0.618 0.788 1.018 1.11 0.193 0.346 0.912 0.019 0.28 0.385 -0.104

BI888000 -0.728 -0.05 0.9 1.579 1.472 0.508 0.378 1.501 0.62 0.144 1.035 0.675

BI888232 -0.195 0.697 1.866 3.261 3.045 1.671 1.983 2.777 1.522 -0.301 0.721 -0.392

BI888349 -0.675 0.23 0.969 1.43 1.394 0.231 0.09 0.247 -0.36 -0.622 0.019 -0.297

BI888360 -0.07 -1.288 1.24 2.033 1.955 0.744 0.721 1.108 0.372 0.75 0.753 0.319

BI888425 0.52 0.694 1.459 1.477 1.235 1.428 1.205 1.043 0.423 0.747 0.575 0.342

BI888483 -0.438 0.993 5.111 4.773 5.126 2.01 1.304 2.75 1.361 -0.117 -0.144 0.092

BI888493 -0.11 -0.057 1.792 2.345 2.239 0.066 0.389 1.186 0.032 0.181 0.311 -0.199

BI888520 0.902 0.755 1.51 1.736 1.142 0.16 -0.063 0.257 0.298 -0.006 0.104 -0.062

BI888576 -0.269 0.899 0.889 1.035 0.693 -0.039 0.349 0.999 0.281 0.397 0.075 -0.436

BI888750 -0.189 -0.684 1.319 1.747 1.055 0.381 0.067 0.759 0.025 -0.231 0.036 0.097

BI888828 0.773 1.088 0.918 1.233 1.101 1.198 0.506 0.807 0.087 0.554 0.185 -0.007

BI888914 1 0.771 0.739 1.068 1.076 0.646 0.608 0.696 -0.097 0.043 -0.485 -0.677

BI888977 0.578 1.082 2.224 2.134 1.942 1.339 1.073 1.517 0.761 0.789 0.519 0.16

BI888986 0.279 1.188 1.047 1.284 1.238 0.321 0.606 0.688 0.031 -0.044 -0.487 -0.671

BI889064 -0.087 1.037 1.942 1.998 1.267 0.843 0.462 0.96 0.012 0.172 -0.035 0.143

BI889113 0.98 1.194 1.865 2.179 1.371 1.229 1.238 1.15 0.53 0.475 -0.01 -0.448

BI889119 0.746 1.614 1.708 1.8 1.376 1.006 0.559 1.102 0.4 -0.222 0.108 -0.465

BI889129 1.37 1.333 2.155 2.24 2.077 2.091 1.058 1.625 0.767 0.399 -0.686 -0.411

BI889160 0.123 0.816 1.683 1.64 1.441 0.211 0.838 0.637 0.364 -0.649 -0.199 -0.107

BI889410 -0.529 0.073 1.206 1.115 0.994 1.008 0.815 0.351 0.332 0.453 -0.005 0.043

BI889637 -0.998 -0.213 0.805 1.805 1.697 -0.11 0.185 -1.339 -1.211 -0.444 -0.659 0.034

BI889810 0.369 0.846 1.635 1.631 1.244 1.05 0.737 1.276 0.636 0.377 -0.12 -0.12

BI889901 0.255 0.192 1.275 1.641 1.234 0.66 0.757 0.506 0.138 0.122 -0.097 0.048

BI889944 -0.395 0.587 0.875 0.856 0.786 0.111 0.099 -0.353 0.295 -0.425 -0.366 -0.463

BI890038 0.182 1.28 0.64 1.501 0.992 0.426 -0.061 0.244 -0.221 -0.24 -0.535 -0.035

BI890073 -0.275 0.415 1.398 1.833 1.712 0.781 1.057 1.716 0.153 0.481 0.419 0.573

BI890195 0.522 0.652 0.794 0.979 0.543 0.289 0.103 0.547 0.177 0.092 -0.282 -0.349

BI890262 -0.728 0.17 1.09 2.848 2.359 0.268 -0.219 0.193 -0.378 -0.68 -0.117 -0.384

BI890305 -0.728 -0.028 0.201 1.267 1.079 -0.03 0.245 0.844 0.264 0.262 0.872 0.652

BI890314 -0.248 -0.196 0.655 2.21 1.637 0.228 1.112 1.317 0.234 -0.021 0.39 0.84

BI890789 -0.617 0.786 0.794 1.114 0.631 0.513 0.004 0.503 0.134 -0.03 -0.038 -0.164

BI891069 0.147 0.413 0.608 0.691 0.576 0.129 0.181 0.765 0.004 0.14 -0.093 -0.365

BI891235 -0.06 0.346 0.655 1.159 0.755 0.002 0.027 -0.096 -0.048 0.118 0.036 -0.129

BI891521 -0.555 -0.554 0.597 1.466 1.253 -0.131 0.318 0.694 0.135 0.155 0.6 0.481

BI891608 0.458 1.304 1.193 1.447 1.035 0.793 0.284 0.46 -0.135 -0.102 -0.063 -0.526

BI891666 -0.728 -0.147 0.656 1.377 1.101 -0.147 0.31 0.848 0.298 -0.251 -0.038 0.133

BI891984 -0.299 -0.03 2.326 2.165 2.287 0.248 0.46 1.084 0.32 0.164 0.053 -0.208

BI891993 -0.097 0.711 1.056 1.39 1.062 0.819 0.453 0.938 0.231 0.142 -0.414 -0.255

BI892038 -0.43 0.435 0.69 0.799 0.791 0.562 0.175 0.695 0.193 0.045 -0.05 -0.187

BI892110 -0.496 -0.892 1.503 2.679 2.476 0.098 1.275 1.143 1.483 0.757 -0.012 -0.173

BI892250 -0.427 0.369 0.261 0.554 0.512 -0.113 -0.075 -0.083 0.185 0.044 -0.147 -0.282

BI892349 -0.493 0.065 3.322 3.139 2.871 2.644 2.198 1.907 0.968 0.944 0.976 0.056

BI980132 0.124 0.716 0.717 1.007 0.796 -0.056 -0.122 0.154 0.12 -0.187 -0.348 -0.708

BI980217 -0.122 0.711 1.383 1.513 1.35 1.022 1.272 1.236 0.595 0.919 0.372 0.591

BI980786 0.329 0.453 0.882 0.881 0.463 0.468 0.202 0.52 0.189 0.243 0.001 -0.178

BI980800 -0.433 -0.441 -0.095 1.71 0.735 0.844 0.375 0.905 0.537 0.931 0.911 0.806

BI980843 -0.107 0.185 2.232 1.995 2.032 1.509 0.835 1.257 0.229 0.532 0.493 0.504

BI983593 0.341 0.295 0.603 0.994 0.822 0.859 0.729 0.56 0.073 0.398 -0.06 0.489

BM005035 0.437 0.544 0.524 0.834 0.893 -0.123 0.279 0.555 -0.015 0.059 -0.14 -0.296

BM005197 0.677 0.44 0.449 0.79 0.746 -0.02 0.187 0.588 -0.066 0.021 -0.436 -0.322

BM034969 0.851 1.274 1.297 1.516 0.881 1.472 0.591 0.867 0.534 0.253 0.044 -0.088

BM036771 1.151 1.204 1.008 1.409 0.206 -0.229 -0.516 -0.709 -0.598 -0.519 -0.562 -0.728

BM070534 -0.495 0.627 0.749 1.081 0.86 -0.033 -0.123 0.473 0.198 0.16 0.089 0.069

BM082431 0.336 0.063 0.503 0.535 0.481 -0.187 0.107 -0.123 0.079 0.096 -0.375 -0.016

BM082684 -0.287 0.758 0.818 0.888 0.833 0.068 0.288 0.193 -0.191 -0.092 -0.069 0.045

BM095746 -0.173 0.343 0.492 1.085 0.822 0.258 0.015 0.23 0.418 -0.009 0.918 0.043

BM095922 0.032 1.012 0.948 1.141 0.959 -0.115 0.101 0.668 -0.03 -0.191 -0.319 0.179

BM096064 0.957 0.912 1.315 1.776 0.976 0.866 0.77 0.945 0.435 0.23 0.035 -0.578

BM096465 0.034 0.111 2.124 1.906 1.663 1.33 0.691 0.911 0.251 0.343 0.075 -0.037

BM102339 0.093 0.778 0.866 1.235 0.683 0.285 -0.138 0.22 -0.323 -0.345 -0.437 0.27

BM102647 -0.504 0.538 0.496 0.894 0.735 -0.175 0.137 0.848 0.131 0.199 0.062 0.147

BM103179 -0.068 0.604 1.426 1.578 1.132 0.69 -0.049 0.704 0.251 0.21 0.044 -0.12

BM103291 0.314 0.581 0.257 0.677 0.721 -0.127 0.151 0.644 0.142 0.09 -0.262 -0.221

BM103978 1.052 0.92 1.456 1.856 1.293 0.258 0.286 0.503 -0.511 -0.329 -0.781 -0.947

BM104348 0.427 0.841 0.692 0.965 0.271 0.324 0.372 0.308 -0.038 0.177 -0.245 -0.537

BM104403 0.159 0.683 1.03 1.567 0.446 -0.061 0.128 -0.076 -0.068 -0.601 -0.296 -0.533

BM155767 0.593 0.913 0.7 1.344 1.155 0.212 0.291 0.435 -0.044 -0.03 -0.059 -0.092

BM172681 -0.187 0.546 0.896 1.198 0.992 0.397 0.05 0.331 -0.07 -0.114 -0.321 -0.305

BM181598 -0.078 0.544 0.669 0.675 0.568 -0.173 0.295 0.674 0.158 0.203 -0.15 -0.429

BM181821 0.83 1.029 1.35 1.496 0.827 0.816 0.258 0.533 0.122 0.099 -0.058 -0.401

BM181828 0.774 0.223 0.413 1.023 0.693 0.821 0.752 0.358 0.316 0.231 -0.125 -0.165

BM182673 0.092 0.47 0.523 0.623 0.634 -0.026 -0.045 0.125 0.066 -0.168 -0.295 -0.327

BM183157 0.641 0.921 1.471 1.574 0.735 1.038 0.197 1.293 0.655 0.975 0.283 0.306

BM183279 -0.225 1.068 1.525 2.298 2.119 0.371 0.698 1.401 0.039 -0.237 0.086 0.3

BM183574 -0.293 0.406 1.344 2.067 1.196 0.141 0.256 0.17 0.021 -0.268 0.128 -0.194

BM183759 0.401 1.259 1.157 1.472 1.126 0.743 0.496 1.392 0.421 0.396 0.295 0.032

BM183794 0.222 0.859 1.593 1.649 1.52 1.251 0.959 0.681 0.443 0.118 0.341 0.359

BM183973 1.164 1.241 1.479 1.526 1.023 1.063 0.601 1.079 0.582 0.651 0.275 -0.466

BM184696 -0.635 0.218 0.733 0.782 0.688 -0.061 -0.077 0.299 0.36 0.158 0.26 -0.303

BM185057 0 0.699 0.79 1.342 0.701 0.223 0.233 0.235 -0.245 -0.404 -0.382 0.231

BM185759 -0.645 0.099 0.461 1.394 1.207 -0.134 0.597 1.076 0.426 0.657 0.772 0.353

D38454 0.876 1.359 2.007 2.087 1.386 1.501 1.006 1.561 0.718 0.588 -0.164 -0.882

L77146 -0.894 -0.78 0.531 0.89 0.56 0.832 0.434 0.456 0.289 0.684 0.369 0.071

U16310 -0.92 0.663 1.142 1.317 0.939 -0.315 -0.03 0.432 -0.096 -0.327 -0.469 -0.194

U42392 -0.102 -0.654 0.545 2.58 2.552 0.339 1.061 0.924 0.872 -0.34 0.174 -0.068

U84616 0.033 -0.002 0.935 1.018 0.881 0.137 0.408 0.671 0.101 0.241 0.118 -0.067

X68324 -0.717 0.343 0.063 0.962 0.149 0.38 -0.128 0.269 0.559 0.412 0.658 0.48

X79821 -2.071 -0.96 2.076 2.461 1.681 2.096 1.317 1.66 1.403 1.531 1.042 0.382

X87581 -1.892 -1.153 2.857 2.754 2.173 1.424 1 1.17 0.804 1.252 1.018 0.398

AA494997 0.093 1.166 1.246 1.116 2.289 1.522 0.841 1.659 0.223 -0.069 -0.325 -0.485

AB045624 -1.486 -1.495 0.571 1.069 1.612 0.87 0.508 0.171 -0.063 -0.449 -0.531 -0.726

AF031378 -0.268 -0.348 -0.122 0.849 1.075 -0.02 0.253 0.442 0.161 -0.056 0.767 0.447

AF039410 -0.695 0.411 0.385 0.84 1.276 0.016 0.636 1.182 0.158 -0.113 0.276 -0.315

AF052245 -0.116 -0.212 1.348 1.297 2.57 0.785 0.514 1.204 0.119 0.163 0.294 0.106

AF052251 -0.728 0.214 2.368 2.087 3.867 1.415 0.804 1.812 -0.021 -0.416 -0.083 -0.266

AF072456 -0.561 0.019 0.927 0.681 1.537 -0.189 0.353 1.302 -0.153 0.304 0.188 0.199

AF124332 -0.139 0.113 0.599 0.453 0.896 0.038 0.249 0.739 0.04 0.088 -0.151 -0.161

AF132445 -0.272 0.398 0.602 0.77 1.093 0.46 0.076 0.642 -0.003 0.186 0.342 -0.019

AF157110 -0.522 -0.377 0.855 1.995 2.574 1.429 1.305 2.254 0.628 -0.123 -0.133 -0.387

AF159135 -0.161 0.308 0.812 0.545 1.188 -0.241 0.101 0.851 -0.073 -0.039 -0.018 -0.006

AF160659 -1.165 -0.489 0.023 0.785 0.753 0.326 0.069 0.624 0.318 0.147 0.352 0.161

AF164483 0.265 0.294 1.3 0.84 1.41 1.083 0.877 1.17 0.339 0.345 -0.269 -0.122

AF164726 0.022 0.02 0.534 0.502 0.825 0.049 0.251 0.177 -0.179 -0.262 -0.622 -0.651

AF168007 -0.304 0.263 1.977 2.021 2.285 1.291 0.849 2.239 0.503 0.632 0.527 0.299

AF172089 -0.155 0.202 0.631 1.057 1.514 -0.151 0.447 1.453 -0.137 -0.169 0.66 -0.259

AF191578 -0.572 0.032 1.052 2.112 2.705 0.244 0.468 1.513 0.46 0.187 0.22 0.015

AF210637 -0.817 -0.619 0.045 0.632 0.741 0.103 0.219 -0.06 0.481 0.328 -0.015 -0.148

AF219949 -0.195 0.389 0.407 0.433 2.351 1.238 1.071 2.132 0.41 -0.469 1.171 0.451

AF246162 -0.685 -0.707 1.11 2.543 3.85 0.879 1.507 1.991 1.235 -0.041 -0.063 -0.295

AF290981 -0.434 0.509 1.624 1.355 1.547 1.303 0.73 0.827 0.36 0.706 -0.262 -0.43

AF332983 0.046 0.352 1.238 1.049 1.295 1.073 0.448 0.412 -0.261 0.192 -0.049 -0.624

AF336123 -1.57 -0.396 0.263 1.712 2.381 1.641 1.859 2.302 1.093 1.564 1.231 0.662

AF364085 0.055 0.302 0.883 1.221 1.755 1.378 0.816 1.584 0.556 0.82 0.21 -0.512

AF388363 -0.588 -0.037 0.536 1.212 1.899 0.826 0.534 1.494 0.15 0.044 0.729 -0.073

AF426384 -0.75 0.026 0.392 1.604 1.737 1.203 0.569 1.317 0.163 0.606 0.979 0.166

AF428249 -0.922 0.003 0.306 0.538 1.021 -0.032 0.067 0.877 0.416 0.348 0.045 0.181

AI331812 -0.575 -0.348 1.008 0.453 1.013 0.733 0.209 0.254 0.187 0.126 -0.036 0.227

AI415831 -0.826 0.079 0.066 0.566 0.945 0.294 0.144 0.885 0.293 0.123 0.076 -0.243

AI437388 -0.494 -0.04 0.003 0.336 0.806 0.104 0.17 0.668 0.127 0.204 0.178 -0.033

AI437428 -0.247 0.539 1.858 1.632 2.081 1.957 1.738 1.096 0.661 0.466 -0.105 -0.208

AI476925 0.247 0.27 0.639 1.368 1.639 0.291 0.774 0.845 0.045 0.135 -0.19 -0.17

AI476945 -0.153 -0.091 0.317 0.17 0.98 -0.018 -0.117 0.544 -0.005 -0.103 0.598 -0.21

AI477417 -0.728 0.173 1.274 1.391 1.552 0.749 0.812 1.279 0.198 -0.272 0.142 -0.143

AI477419 -0.495 -0.332 0.882 1.233 1.83 0.344 0.614 1.522 0.73 0.902 0.466 0.056

AI477962 0.623 1.065 1.93 1.953 2.226 1.983 1.718 1.384 0.266 0.754 0.195 0.338

AI477963 -0.728 -0.059 0.342 1.852 2.316 0.605 0.823 0.786 0.205 0.459 1.126 0.408

AI497192 -0.397 0.109 0.183 0.478 0.585 -0.141 -0.116 0.277 0.055 0.214 0.152 -0.2

AI497265 -0.109 0.2 1.042 1.416 1.874 0.296 0.801 1.496 0.234 0.474 0.151 0.056

AI522514 -0.312 -0.032 0.782 0.659 1.398 0.212 0.444 0.849 0.334 0.479 0.67 0.509

AI544512 -0.264 0.091 1.096 1.692 2.037 0.167 0.746 1.734 0.205 0.631 0.259 0.263

AI558301 0.074 0.849 0.845 0.861 1.366 0.63 0.907 0.975 0.515 -0.046 -0.196 0.013

AI558661 -0.107 -0.48 0.162 0.061 0.61 -0.053 0.27 -0.127 -0.333 -0.094 -0.605 -0.836

AI584322 -0.728 -0.129 0.191 0.191 1.77 0.432 0.263 1.276 0.308 -0.171 -0.056 0.134

AI584406 0.025 0.305 0.401 0.837 1.408 0.032 0.294 0.923 0.027 0.074 0.141 -0.123

AI584407 -0.537 -0.92 1.275 0.508 1.497 1.178 0.732 0.919 0.626 0.308 0.232 0.119

AI584501 -0.134 -0.118 0.923 0.971 1.757 1.225 0.958 1.632 0.756 0.453 0.714 0.16

AI584575 -0.271 0.764 0.417 0.899 0.988 0.239 0.327 0.869 -0.142 -0.026 -0.068 0.292

AI588196 -0.441 -0.957 0.16 2.314 2.446 -0.124 1.381 1.012 0.812 0.189 0.355 -0.019

AI588319 -0.127 0.464 0.595 0.909 1.387 0.643 0.384 0.929 0.097 0.099 0.056 0.077

AI588395 -0.6 0.093 0.028 0.635 0.989 0.549 0.12 0.754 0.137 0.42 0.621 0.385

AI601443 -0.528 -0.162 0.577 1.422 1.582 0.275 0.553 1.269 0.512 0.703 0.754 0.551

AI601529 -0.672 -0.08 0.451 0.677 0.831 -0.089 -0.27 0.284 -0.122 -0.539 0.453 0.031

AI601583 0.744 -0.652 1.139 0.976 1.491 1.387 0.721 0.909 0.538 0.269 0.163 -0.135

AI601596 -0.367 0.031 0.253 0.313 1.167 -0.029 0.014 0.644 0.003 0.116 0.174 0.299

AI616791 -0.498 -0.311 0.902 1.224 1.794 1.573 0.828 1.025 0.399 0.892 1.055 1.106

AI618715 -0.966 -1.265 0.052 0.887 1.519 0.915 0.659 1.409 0.644 0.487 0.298 0.486

AI641041 -0.561 -0.429 0.645 0.737 1.158 0.984 0.489 0.562 0.468 0.393 0.662 0.601

AI641272 0.257 -0.569 0.765 0.707 0.884 0.081 -0.03 0.524 -0.356 -0.046 -0.053 -0.259

AI641561 -0.145 -0.866 -0.151 0.448 0.492 0.302 -0.19 -0.212 0.35 -0.318 0.316 0.02

AI641716 0.317 0.322 0.714 0.954 1.067 0.457 0.371 0.875 0.204 0.291 0.529 0.078

AI657632 -0.458 0.206 0.665 1.115 1.281 0.464 0.086 0.526 -0.232 0.041 0.138 -0.321

AI667069 0.171 0.513 0.453 0.338 0.632 0.161 0.214 0.095 0.025 0.136 -0.058 0.065

AI667083 0.266 0.081 0.14 0.262 0.801 0.441 0.135 0.761 0.142 0.157 -0.005 0.037

AI721361 -0.202 -0.509 1.223 1.74 1.965 1.81 0.826 0.961 0.726 0.29 0.067 0.244

AI721615 -0.543 -0.642 0.421 0.496 1.182 0.597 0.455 1.008 0.23 0.301 -0.059 0.112

AI721655 -0.68 0.012 0.889 1.683 1.731 0.89 0.362 1.019 0.822 0.66 0.475 0.509

AI722328 0.381 0.015 1.576 1.122 2.013 1.245 1.017 1.432 0.778 0.383 0.162 -0.086

AI722745 -1.013 0.196 0.92 0.761 1.912 1.332 0.137 1.489 1.304 0.77 0.493 1.446

AI722764 -0.272 0.001 0.826 0.677 0.834 0.166 0.153 0.782 0.222 0.041 0.152 -0.086

AI723092 -0.676 0.067 0.391 0.868 1.008 0.306 0.499 0.884 0.216 -0.055 0.73 0.317

AI723236 -0.343 0.006 0.612 0.858 1.039 0.361 -0.031 0.469 -0.096 0.06 0.393 -0.191

AI793574 0.573 0.999 1.116 1.66 1.612 1.636 0.847 1.562 0.293 0.25 -0.118 -0.415

AI793673 0.273 -0.065 1.228 1.295 1.448 1.053 0.67 0.708 0.406 0.382 0.169 -0.354

AI793733 0.126 0.386 0.381 0.51 0.765 0.367 0.154 0.595 0.352 0.303 0.085 -0.078

AI793772 -0.158 -0.506 0.017 0.336 1.171 0.833 0.443 0.745 0.195 0.145 0.889 0.421

AI816663 -0.45 0.383 0.801 0.659 0.999 0.49 0.347 0.361 0.393 -0.105 -0.104 -0.463

AI877518 -0.497 0.024 0.383 0.727 1.587 1.335 0.651 1.519 0.862 0.411 0.47 0.357

AI877596 0.027 0.181 1.012 2.523 3.165 1.089 1.485 0.711 0.657 -0.586 -0.282 0.002

AI877869 -0.097 -0.094 0.089 0.54 1.04 0.286 0.058 0.962 0.017 0.312 0.337 0.186

AI878344 0.25 0.321 0.613 0.718 0.794 0.435 0.437 0.418 0.093 0.315 -0.174 -0.539

AI878713 -0.205 -0.058 0.224 0.667 1.124 0.782 0.633 0.831 0.122 -0.048 -0.457 -0.234

AI883253 -0.135 0.175 0.806 0.79 1.567 0.171 0.339 1.262 0.518 0.308 0.183 -0.259

AI883323 0.079 -0.096 0.409 0.909 1.259 0.696 0.144 0.613 0.082 0.244 0.338 0.149

AI883714 0.06 0.446 0.3 0.724 1.024 0.295 0.269 0.558 -0.238 -0.49 -0.202 0.076

AI883908 -0.4 0.346 0.176 0.363 1.212 0.103 0.551 1.099 0.227 -0.148 0.396 -0.112

AI884048 0.556 0.316 1.127 1.467 1.463 1.244 1.074 1.301 0.544 0.085 0.003 0.68

AI884185 -0.668 0.38 0.407 1.306 1.437 0.179 0.086 0.451 -0.293 -0.61 0.223 -0.232

AI943086 -0.645 0.012 0.158 0.652 0.742 0.24 0.328 0.563 -0.21 -0.081 0.632 0.37

AI943105 -0.236 0.113 1.302 1.596 1.952 0.19 0.788 1.089 0.434 0.455 0.246 -0.079

AI957698 -0.786 -0.26 1.664 1.773 2.383 1.099 0.856 1.987 0.641 0.375 -0.041 -0.912

AI958820 -0.209 0.294 1.041 1.007 1.413 0.8 0.515 0.997 0.339 0.396 0.333 0.255

AI958945 -0.364 -0.189 1.392 1.866 2.146 0.542 0.536 1.185 0.249 0.449 0.321 0.322

AI959157 0.003 -0.26 0.567 0.817 0.936 0.266 0.611 0.3 -0.123 0.033 -0.186 -0.068

AI959620 0.036 0.096 0.341 0.928 1.303 -0.135 0.088 0.67 0.05 -0.074 0.667 -0.074

AI959644 -0.881 0.013 2.18 3.173 4.202 3.96 3.578 3.8 2.558 -0.271 0.566 -0.431

AI959735 -0.678 0.385 2.405 2.732 3.303 1.507 1.295 2.131 0.26 -0.104 0.133 -0.36

AI964285 -0.015 0.487 0.512 0.578 0.741 0.184 0.069 0.117 0.467 0.07 0.503 0.083

AI964310 -0.405 -0.15 2.21 1.926 2.429 0.911 0.881 1.219 0.53 0.481 0.578 0.355

AI965249 -0.916 0.059 1.887 2.029 2.632 2.428 1.793 1.964 1.421 1.408 1.236 1.455

AI979356 -0.382 -0.561 0.783 0.569 1.152 1.043 0.456 1.207 0.648 0.57 0.167 -0.054

AJ005936 -1.251 -0.65 0.418 0.774 0.996 0.108 0.469 0.665 0.688 0.731 0.434 0.266

AJ293862 -0.734 -0.999 -0.188 0.916 1.259 -0.079 0.066 0.086 0.055 -0.247 0.682 0.344

AJ315468 -0.367 -0.446 0.193 1.11 1.673 0.82 0.069 0.42 -0.145 -0.31 0.701 0.272

AW018941 -0.728 0.121 0.11 0.437 0.581 -0.125 -0.029 0.215 -0.378 -0.557 -0.543 -0.913

AW018972 -1.257 0.026 -0.563 0.341 0.305 -0.061 -0.407 -0.535 -1.048 -1.696 -1.781 -2.074

AW019294 -0.155 0.132 1.911 1.669 1.898 1.171 1.136 1.087 0.865 0.413 0.521 0.331

AW019523 -0.573 -0.073 0.747 0.856 1.286 0.354 0.241 1.115 0.44 0.269 0.261 0.17

AW058759 -0.133 -0.173 0.809 1.078 1.562 0.345 0.234 0.663 0.397 0.104 0.373 0.014

AW058763 -0.035 -0.601 0.757 1.392 2.078 1.945 1.238 1.241 0.892 0.653 0.722 0.989

AW058804 -0.376 -0.412 0.82 0.917 1.528 0.88 0.502 0.927 0.398 0.406 0.861 0.736

AW058828 -0.596 -0.16 0.431 1.085 1.181 0.457 0.275 0.518 -0.083 -0.478 0.537 0.131

AW058967 -0.916 -0.32 -0.062 1.133 1.297 0.985 1.037 0.835 0.727 0.616 1.1 0.576

AW059048 0.216 0.168 0.698 1.075 1.389 0.868 0.804 1.318 0.402 0.833 0.403 0.068

AW059137 0.038 -0.282 0.947 1.012 1.947 1.203 0.678 1.243 0.535 0.249 0.289 0.012

AW059146 -0.048 0.489 1.663 1.671 1.844 1.612 1.307 1.608 0.424 0.593 0.344 0.388

AW059158 -0.081 0.08 0.469 0.673 0.771 -0.128 0.414 0.705 0.228 0.159 -0.186 -0.087

AW059316 -0.006 -0.363 0.395 0.734 1.632 0.782 0.638 1.336 0.562 0.174 -0.001 -0.154

AW059366 -0.03 0.142 1.531 1.689 3.508 3.088 2.077 1.357 1.046 0.042 0.932 0.157

AW076882 0.499 0.884 0.72 0.945 1.007 0.535 0.291 0.62 -0.026 0.031 -0.557 -0.383

AW077743 0.49 0.492 -0.105 0.647 0.83 0.485 0.301 0.704 0.445 -0.082 0.031 -0.179

AW077976 -0.703 -0.171 -0.006 0.488 0.64 0.46 -0.123 -0.024 -0.216 -0.065 -0.383 -0.408

AW077980 -0.685 0.621 0.55 0.739 1.647 -0.13 0.43 1.327 -0.28 -0.422 0.008 -0.117

AW078150 -0.798 0.206 0.541 0.831 1.182 -0.206 0.074 0.979 0.205 0.588 0.569 0.82

AW115956 0.119 -0.422 0.763 1.929 3.313 1.68 1.617 1.777 1.345 -0.159 -0.373 -0.527

AW116232 0.592 0.462 0.205 0.64 0.944 0.457 0.336 0.769 -0.024 0.119 -0.222 -0.01

AW116486 -0.473 0.045 0.73 0.476 1.028 -0.438 0.178 0.858 0.436 0.202 0.072 0.196

AW117083 -0.975 0.212 0.938 1.226 1.508 0.754 1.013 1.41 0.439 0.558 0.499 -0.201

AW117160 0.388 0.004 0.836 0.906 1.178 0.912 0.593 0.46 0.574 0.142 0.705 0.535

AW127725 -0.249 0.588 0.489 0.724 1.008 0.096 0.025 0.78 0.048 -0.173 -0.21 -0.073

AW128377 -0.088 -0.053 0.602 1.234 1.809 0.524 0.898 1.65 0.318 0.378 0.153 0.345

AW134011 -0.693 0.281 0.709 1.327 1.489 0.36 0.183 0.813 0.07 0.333 0.302 -0.091

AW153863 0.015 1.062 0.888 1.001 1.326 -0.055 0.098 1.013 0.141 0.155 -0.007 -0.238

AW154283 0.729 1.14 1.173 1.22 1.287 0.854 0.571 1.29 -0.049 -0.021 -0.31 -0.657

AW154540 0.896 0.853 1.53 1.265 1.643 1.562 0.912 1.645 0.873 1.022 0.293 0.163

AW165272 0.336 0.06 0.401 0.887 1.288 0.791 1.006 1.17 0.401 0.304 -0.103 -0.241

AW170850 0.012 0.141 0.224 0.473 0.655 0.091 0.146 0.207 -0.029 -0.296 -0.531 -0.269

AW171172 -0.189 0.162 0.197 0.306 0.651 0.221 0.075 0.558 -0.161 0.09 0.531 0.169

AW171190 0.132 0.125 0.215 0.671 0.921 0.488 0.495 0.787 0.046 0.377 0.034 0.166

AW171604 0.608 1.401 1.186 1.595 1.835 0.656 0.699 0.496 -0.433 -0.71 -0.69 -0.316

AW174685 0.107 0.579 1.26 1.003 1.497 1.407 0.709 0.962 0.541 0.565 0.205 0.07

AW175474 0.663 0.741 0.367 0.421 1.28 0.87 0.419 0.896 0.12 -0.376 -0.352 -0.251

AW175486 0.154 -0.374 -0.066 0.469 0.707 0.462 0.211 0.439 -0.101 0.203 -0.188 -0.299

AW184334 -0.319 -0.02 -0.087 0.633 0.903 -0.025 0.138 0.522 0.323 0.419 0.268 0.171

AW232471 -0.509 -0.271 0.135 1.05 1.079 0.311 0.073 0.323 0.045 -0.114 -0.287 -0.218

AW280171 0.079 0.878 1.108 1.232 1.475 1.04 0.945 1.458 0.332 0.711 0.375 -0.171

AW343985 0.676 0.906 1.24 2.366 3.043 2.724 2.107 1.825 1.317 -0.153 -0.26 -0.711

AW420284 -0.707 0.086 1.145 1.406 1.771 0.826 0.378 0.515 -0.194 -0.102 0.206 -0.077

AW420304 -0.403 0.384 0.391 1.028 1.635 -0.048 0.159 0.749 -0.017 -0.076 -0.226 -0.206

AW420314 0.385 0.698 1.157 1.016 1.558 1.498 1.127 1.542 0.539 0.385 0.235 0.004

AW454605 0.279 -0.635 0.1 0.587 0.981 0.837 0.464 0.239 0.509 0.579 0.7 0.553

AW466697 0.163 -0.143 -0.06 0.627 1.025 0.778 0.86 0.591 0.428 -0.105 -0.251 -0.303

AW466751 0.084 0.827 1.088 0.973 1.271 0.781 0.704 0.886 0.336 0.657 0.268 0.079

AW567130 -0.533 -0.575 1.898 2.066 2.706 1.28 2.222 2.362 1.631 0.057 0.043 -0.127

AW777769 -0.243 -0.136 1.301 2.744 3.642 1.661 1.352 1.63 1.275 -0.515 -0.495 -0.193

AY034614 0.129 0.971 0.334 1.19 1.263 0.68 0.912 1.229 0.561 -0.025 -1.001 -1

AY045753 -0.49 -0.086 -0.084 0.658 1.002 0.45 0.158 0.502 -0.258 -0.101 -0.254 -0.024

AY050507 -0.589 0.13 1.223 1.089 1.576 1.483 0.492 1.223 0.505 0.334 0.325 0.202

AY057057 0.411 0.189 0.399 0.644 1.005 0.528 0.108 0.682 0.09 -0.074 -0.144 -0.402

BE016113 -0.567 -0.727 0.356 0.707 0.922 0.386 0.314 0.631 0.093 0.257 0.161 -0.05

BE016292 -0.647 0.336 0.408 0.931 1.567 0.287 -0.142 1.182 0.769 0.137 0.056 -0.326

BE016756 0.305 0.23 0.875 1.128 1.19 0.695 0.763 0.628 0.536 0.434 0.148 -0.11

BE017549 -0.464 0.275 0.391 0.543 0.726 -0.141 -0.215 0.495 0.232 0.046 0.099 -0.224

BE200802 -0.085 -0.108 0.348 3.046 3.583 0.269 1 1.22 0.131 -0.595 -0.369 -0.231

BE556841 -0.368 0.253 0.912 0.831 1.004 0.228 0.107 0.662 0.213 -0.258 0.647 0.161

BE605983 -0.36 -0.136 0.467 1.178 1.275 0.096 0.412 1.169 0.213 0.249 0.26 -0.065

BE693119 -1.16 -0.795 0.469 0.5 0.61 0.464 -0.272 -0.563 -0.082 0.502 0.33 0.078

BE693134 -0.123 0.248 0.159 0.495 0.962 0.351 0.447 0.74 0.327 0.171 0.392 -0.009

BF157344 -0.779 -0.292 -0.093 0.931 1.069 -0.161 0.16 0.912 0.065 0.588 0.479 0.293

BF157620 0.231 0.475 1.886 1.999 2.439 0.894 0.118 1.468 0.726 0.478 -0.218 -0.266

BF157841 -0.09 -1.141 0.017 0.652 1.155 0.007 0.338 1.013 0.101 0.148 0.062 0.118

BG303872 0.514 0.455 0.622 1.08 1.536 0.19 0.185 0.528 0.206 0.019 -0.432 -0.167

BG304158 -0.024 0.384 1.589 1.826 1.952 1.364 1.475 1.67 0.969 0.929 0.227 -0.325

BG305367 0.283 0.13 0.038 0.648 1.153 0.534 0.63 1.037 0.078 0.1 0.002 0.404

BG306468 -0.159 -0.385 0.407 0.904 1.022 0.464 0.596 0.978 0.163 -0.26 0.111 0.195

BG307572 -0.468 -0.085 0.294 0.809 1.666 -0.494 0.437 1.08 0.424 0.043 -0.081 -0.267

BG308318 -0.728 0.366 0.439 0.524 0.67 -0.058 -0.331 0.229 -0.306 -0.589 -0.18 -0.265

BG727557 -0.434 0.252 1.633 1.795 2.593 2.382 1.807 2.117 1.253 1.097 0.471 0.078

BG728404 0.409 0.156 0.225 0.591 0.877 0.25 0.508 0.736 -0.124 0.163 0.137 0.06

BG728817 0.377 -0.05 0.755 1.343 3.093 1.549 1.395 1.308 0.925 -0.066 -0.224 -0.427

BG883207 0.706 0.484 2.427 1.969 2.813 1.712 1.247 1.881 0.663 0.963 0.383 0.135

BG891955 0.241 -0.182 0.119 0.842 1.412 1.218 0.752 1.013 0.366 0.218 0.276 0.388

BG985460 -1.109 -1.649 1.801 2.578 3.241 2.613 1.623 1.109 1.668 0.504 0.229 -0.073

BG985498 -1.013 -1.923 2.883 2.542 3.052 1.85 1.369 2.023 0.953 0.744 0.504 0.282

BG985598 0.142 -0.126 0.182 0.685 1.191 0.686 0.513 1.026 0.272 0.732 0.469 0.286

BG985620 -0.42 -0.472 -0.314 0.207 1.201 0.569 0.465 1.132 0.333 0.189 0.155 0.207

BG985698 -0.739 -0.431 0.482 0.859 1.807 1.733 0.706 1.382 0.308 0.398 0.828 0.355

BG985763 -0.728 -0.149 0.465 0.903 1.606 -0.125 0.132 0.929 -0.378 -0.766 -0.543 -0.913

BI318080 -0.443 -0.284 0.142 1.68 1.805 0.36 0.372 0.227 -0.029 -0.271 -0.444 -0.239

BI325086 -0.137 0.481 0.156 0.675 0.786 0.25 0.436 0.084 0.413 0.386 -0.283 -0.374

BI475856 0.36 0.483 1.043 1.407 1.648 0.711 1.143 1.31 0.41 0.055 0.037 -0.124

BI671227 -0.691 -0.282 1.073 0.852 1.665 1.088 0.985 0.875 0.376 0.108 0.038 0.274

BI672045 0.49 -0.19 0.056 0.397 0.617 0.367 0.343 0.595 0.088 0.091 0.087 0.105

BI672139 0.087 0.39 0.466 1.169 1.179 0.633 0.694 0.772 0.179 0.516 0.228 0.259

BI672347 -0.501 -1.061 0.898 1.444 1.845 1.542 0.77 0.734 -0.149 0.027 -0.178 -0.484

BI672391 -0.006 0.094 1.156 1.734 1.935 0.353 0.341 0.781 0.358 0.445 0.093 0.042

BI672395 -1.685 -0.208 1.617 1.404 1.689 1.504 0.723 0.741 0.986 0.604 0.78 -0.446

BI673712 -0.449 0.344 0.783 2.888 3.442 0.682 0.488 0.994 0.043 -0.2 0.474 0.084

BI704177 -0.32 0.568 0.185 1.051 1.353 0.698 0.587 0.816 0.419 -0.114 -0.058 0.144

BI704310 -0.505 0 4.183 4.459 5.947 4.662 2.738 3.76 1.964 0.446 0.184 0.359

BI704422 -0.373 -0.027 1.213 1.078 1.615 1.338 0.931 1.354 0.694 0.973 0.218 -0.146

BI705734 -0.614 -0.092 0.379 0.511 0.832 0.082 0.036 0.305 -0.171 -0.707 0.05 0.033

BI706321 -0.484 0.142 0.338 0.991 1.014 0.312 0.059 0.287 -0.069 -0.554 -0.128 -0.341

BI709110 -0.53 0.104 0.649 0.521 1.185 0.827 0.238 1.217 0.463 0.623 0.325 0.623

BI709805 -0.728 0.639 0.421 0.89 1.042 1.081 0.116 1.154 -0.378 -0.468 -0.543 -0.913

BI840104 -0.486 0.069 0.581 0.927 1.169 -0.032 0.011 0.363 0.209 -0.034 0.059 -0.008

BI841477 -0.549 0.388 0.506 0.828 1.125 0.551 0.293 1.081 0.034 0.184 0.296 0.043

BI842162 -0.211 -0.151 1.002 1.192 1.394 0.868 0.376 0.775 0.995 -0.123 0.512 0.356

BI846965 -0.173 0.548 0.688 0.639 1.441 -0.004 0.252 0.862 0.036 0.144 0.164 -0.004

BI865459 -0.46 0.092 -0.209 0.762 1.432 0.648 0.419 0.758 -0.128 0.157 0.128 0.875

BI866992 0.81 0.445 0.665 1.102 1.446 0.983 0.903 1.332 0.517 0.672 0.047 -0.166

BI867261 0.561 0.41 0.716 0.75 1.328 0.767 0.466 0.976 0.279 0.435 0.345 0.069

BI868116 -1.267 -3.251 -0.071 1.089 3.043 2.44 2.587 1.719 1.335 -0.465 0.072 0.078

BI877594 -0.296 -0.075 1.134 0.982 1.267 0.518 0.349 0.675 0.615 0.541 0.477 0.414

BI877730 -0.589 0.447 1.153 1.37 1.943 0.872 0.52 0.739 0.477 0.344 0.381 0.025

BI877821 -0.747 -0.558 0.37 0.813 1.342 1.011 0.867 1.189 0.414 0.694 0.048 0.135

BI877872 0.082 0.756 2.55 2.114 2.863 2.766 1.911 1.865 0.263 0.296 -0.062 0.131

BI877917 0.211 0.446 0.36 0.563 1.09 0.332 0.28 0.396 0.063 -0.022 -0.497 -0.595

BI878536 0.402 0.452 0.466 0.59 0.975 0.426 0.221 0.509 0.167 -0.242 -0.229 -0.183

BI878907 -0.15 0.253 0.353 0.5 0.598 -0.046 -0.136 0.049 0.035 -0.18 -0.089 -0.135

BI878929 0.402 0.814 0.957 1.401 1.457 1.156 1.063 1.216 0.202 0.43 0.285 0.244

BI878952 -0.015 0.674 0.466 1.036 1.152 0.949 0.259 0.428 -0.048 0.084 0.107 -0.262

BI878962 0.344 0.698 0.808 0.805 0.909 0.359 0.282 0.562 0.02 -0.034 -0.201 -0.128

BI879454 -0.515 0.545 0.751 1.136 1.98 1.181 0.722 2.033 0.617 0.686 0.073 0.408

BI879550 -0.275 0.606 0.918 1.501 1.805 0.612 0.879 1.233 -0.004 0.396 0.037 -0.037

BI880092 -0.443 0.051 0.572 0.84 1.219 0.056 0.147 0.62 0.612 0.133 0.566 0.615

BI880444 0.459 -0.006 0.499 0.756 0.799 0.305 0.546 0.177 0.024 -0.128 -0.197 -0.035

BI880833 0.039 0.038 1.447 1.085 1.579 0.222 0.145 0.506 0.072 -0.035 0.368 0.422

BI882056 0.126 1.122 1.111 1.06 1.326 0.623 0.503 0.688 0.378 0.175 0.076 -0.48

BI883231 -0.227 0.812 0.461 0.657 1.12 0.465 0.493 1.022 0.075 0.067 0.061 -0.263

BI883233 -0.728 0.203 0.467 0.841 1.59 0.116 0.378 0.717 -0.414 -0.453 -0.178 -0.287

BI883903 0.092 -0.412 0.496 0.613 0.845 0.185 -0.085 -0.255 -0.084 0.036 -0.061 0.38

BI883935 0.336 0.332 1.274 1.116 1.622 1.538 1.239 0.803 0.795 0.759 0.529 0.481

BI884839 -0.697 0.391 0.826 1.374 1.683 1.598 0.711 0.905 0.607 0.594 0.281 0.221

BI885798 -0.064 0.696 0.89 1.303 1.831 0.559 0.346 1.304 0.395 0.157 0.272 -0.182

BI885813 -0.035 -0.048 0.906 1.149 1.29 0.617 0.358 0.491 0.33 -0.055 0.123 -0.013

BI885898 -0.052 0.316 0.661 0.626 1.241 0.28 0.326 0.7 0.224 0.14 -0.209 -0.18

BI885944 -0.85 0.45 1.191 1.649 2.567 1.012 0.739 1.695 1.016 -0.504 -0.684 -0.65

BI885973 -0.077 1.298 1.376 1.529 1.834 1.341 0.743 1.56 0.503 0.187 0.126 -0.082

BI885994 0.18 0.544 0.592 0.542 0.726 0.318 0.095 0.315 0.108 0.11 -0.095 -0.174

BI886184 0.353 0.196 0.826 1.147 1.372 1.249 0.804 0.868 0.672 0.228 -0.035 -0.316

BI886272 -0.195 0.334 1.563 2.079 2.216 1.158 1.427 1.986 0.671 0.598 0.336 0.148

BI886373 -0.546 0.568 0.741 0.912 1.168 0.261 0.354 0.906 0.84 0.65 0.218 -0.218

BI886374 0.256 0.559 1.199 0.986 1.248 1.173 0.978 0.967 0.623 0.448 0.072 -0.367

BI886431 -0.691 -0.474 1.99 3.04 4.379 3.376 2.483 3.455 2.321 -0.149 0.078 -0.148

BI886461 0.232 0.019 0.634 0.432 1.182 0.974 0.677 1.114 0.361 0.605 0.141 0.306

BI886470 -0.728 -0.501 0.201 0.707 1.821 0.121 0.208 0.672 0.272 -0.045 -0.025 -0.278

BI886653 -0.245 0.381 1.379 1.607 2.417 1.66 1.173 1.97 0.486 1.143 0.718 0.622

BI886745 0.003 0.053 1.196 1.123 1.289 0.521 0.499 0.742 0.202 0.045 -0.31 -0.376

BI886791 -0.772 -0.611 3.814 3.471 3.747 2.49 2.088 2.781 1.013 0.485 0.309 0.923

BI886872 -0.53 0.192 0.258 0.196 1.105 -0.065 0.565 0.918 0.25 0.115 0.302 0.882

BI886934 -0.01 0.714 1.342 1.359 1.415 1.014 0.758 0.875 0.581 0.559 0.255 0.024

BI887057 -0.33 -0.483 0.191 0.546 1.222 0.337 0.423 0.596 0.3 0.667 0.509 0.109

BI887101 0.42 0.935 0.738 0.545 1.094 0.201 0.032 0.862 -0.054 -0.096 -0.116 -0.37

BI887199 0.07 0.481 1.145 1.263 1.608 0.569 0.438 1.467 0.188 0.509 0.215 0.371

BI887324 -0.18 0.3 0.749 0.54 0.922 0.868 0.448 0.641 0.309 0.131 -0.244 -0.486

BI887377 -0.723 0.469 0.808 1.198 1.521 0.287 0.151 0.862 -0.156 -0.522 0.568 -0.501

BI887446 -0.728 0.843 4.999 4.723 5.164 3.541 2.85 2.104 0.391 -0.38 -0.059 -0.19

BI887477 -0.438 0.623 0.467 0.566 1.039 -0.04 0.248 0.833 0.102 0.354 -0.022 0.143

BI887535 -0.001 0.101 1.259 1.209 1.598 0.166 0.326 0.967 0.188 0.489 0.099 0.012

BI887627 -0.163 0.397 0.635 0.786 0.887 0.068 0.09 0.536 0.076 -0.163 -0.627 -0.594

BI887656 -0.191 0.343 0.481 1.538 2.004 0.676 0.747 1.808 0.42 0.01 0.271 0.553

BI887764 -0.166 -0.148 1.247 1.069 1.686 0.61 0.671 1.161 0.635 0.345 0.567 0.02

BI887789 0.319 -0.817 0.325 0.712 1.465 0.392 0.683 0.855 0.818 0.644 1.033 0.303

BI887861 0.136 0.37 1.073 0.819 1.835 0.96 0.277 0.014 -0.527 -0.237 -1.15 -1.157

BI887935 0.21 1.13 0.655 1.352 1.556 0.186 0.66 1.079 0.325 0.075 -0.176 -0.291

BI888158 -0.567 0.055 2.182 2.398 3.208 1.226 0.67 2.981 1.848 0.477 0.048 0.042

BI888172 -0.069 0.004 0.057 1.149 1.929 0.219 0.186 0.406 0.436 -0.029 -0.132 -0.204

BI888241 -0.728 0.04 0.618 1.503 2.68 1.586 1.825 1.617 0.395 -0.762 -0.274 -0.505

BI888267 -0.026 -0.34 0.953 1.409 2.243 0.337 0.844 1.119 0.524 0.489 0.753 -0.474

BI888338 -1.058 -0.729 1.79 1.534 2.663 2.163 1.734 2.502 1.936 0.469 1.079 0.39

BI888431 0.308 0.018 1.028 0.665 1.805 0.693 0.305 1.212 0.172 -0.17 -0.02 -0.049

BI888432 -0.199 -0.176 0.34 0.769 1.178 0.894 0.579 1.215 1.048 0.645 0.172 -0.297

BI888545 -0.535 0.102 1.261 1.244 1.79 0.441 0.632 1.7 1.06 0.096 0.597 0.087

BI888564 0.077 0.476 1.388 1.506 2.022 0.556 0.356 1.225 -0.114 -0.185 -0.11 0.016

BI888567 0.283 0.689 1.137 1.271 1.497 0.968 0.598 1.318 0.559 0.247 0.251 0.333

BI888620 1.143 1.28 2.635 2.463 2.685 1.633 0.894 -0.73 -1.085 -0.832 -1.243 -0.986

BI888748 -0.018 0.078 1.517 1.524 1.81 1.091 1.067 1.429 0.711 0.487 -0.333 -0.774

BI888784 -0.681 -0.498 0.363 1.113 1.238 0.373 0.304 0.306 0.474 -0.017 0.118 0.618

BI888863 -0.06 0.227 0.663 0.729 0.864 0.746 0.249 0.931 0.276 0.352 0.047 -0.012

BI888908 0.105 0.683 0.673 0.622 0.727 0.433 0.245 0.346 -0.056 -0.186 -0.391 -0.403

BI888920 -0.018 -0.318 0.553 0.721 1.323 0.897 0.612 0.937 0.519 0.467 0.201 -0.105

BI889130 0.02 -0.615 0.622 1.87 4.055 2.217 1.535 2.181 1.275 0.022 -0.163 -0.363

BI889170 0.095 -0.055 0.51 0.822 1.318 0.965 0.926 1.127 0.155 0.232 -0.204 -0.169

BI889194 -0.577 0.037 0.587 1.239 1.632 -0.116 0.264 0.754 0.322 0.275 0.392 -0.039

BI889290 0.365 0.665 1.253 1.263 1.493 1.047 0.491 1.075 0.129 0.248 -0.115 -0.234

BI889332 0.355 0.464 0.409 0.466 0.754 0.317 0.243 0.145 0.009 0.091 -0.199 -0.126

BI889396 -0.505 -0.015 0.201 0.612 0.896 -0.013 0.181 0.681 0.179 0.03 0.289 -0.01

BI889553 0.197 0.476 0.267 0.678 0.813 0.172 0.18 0.381 -0.37 -0.026 -0.292 -0.117

BI889609 -0.372 -0.97 0.969 0.685 1.429 1.069 0.879 0.86 0.868 1.146 1.01 0.412

BI889937 -0.892 0.267 0.861 0.938 1.22 0.35 -0.08 -0.734 0.148 0.076 0.003 -1.026

BI890191 -0.438 -0.363 1.143 1.333 2.239 0.83 0.461 1.514 0.662 0.347 0.456 0.151

BI890279 -0.199 -0.483 1.692 2.054 2.906 1.847 1.443 1.88 0.563 0.387 0.147 0.222

BI890287 -1.467 -2.301 -0.023 0.517 1.214 1.119 0.978 0.835 0.073 -0.503 -0.828 -1.662

BI890294 -0.144 -0.11 0.704 1.974 2.251 0.889 0.575 1.213 0.487 0.208 0.155 0.125

BI890315 -0.462 0.29 0.766 0.725 1.19 0.098 -0.153 0.807 0.305 0.681 0.843 0.203

BI890334 -0.503 -0.096 1.285 1.668 1.927 0.674 1 0.976 0.919 0.241 -0.208 -0.512

BI890367 -0.771 0.247 0.862 1.822 1.886 1.07 0.339 1.694 1.73 1.5 0.567 0.091

BI890420 -0.058 0.13 0.475 0.769 1.007 0.828 0.715 0.763 0.446 0.192 0.262 -0.161

BI890492 0.124 0.734 0.752 0.897 1.272 0.159 0.704 0.868 0.303 -0.058 0.185 -0.142

BI890505 -0.285 -0.09 4.319 3.893 4.671 3.049 1.938 1.746 0.989 -0.311 0.149 -0.077

BI890512 -0.619 0.788 1.553 1.003 1.474 1.431 0.711 1.432 0.675 0.506 0.367 0.215

BI890587 -0.126 0.229 3.047 3.361 3.914 2.207 3.129 3.702 3.213 0.388 0.772 -0.033

BI890618 -0.728 0.138 0.238 1.14 1.921 0.883 0.79 1.711 0.755 0.868 0.824 -0.057

BI890643 -0.72 0.323 0.494 0.966 1.192 -0.085 0.256 0.991 -0.128 -0.061 -0.128 0.124

BI890682 -0.009 0.164 0.389 0.747 0.938 0.005 0.177 -0.021 0.182 0.033 -0.18 -0.266

BI890739 -0.044 0.123 1.074 1.599 1.94 0.867 0.742 1.875 0.39 0.417 0.688 -0.076

BI890749 -0.107 0.06 0.61 0.57 0.754 0.105 0.328 0.714 0.561 0.256 0.059 -0.092

BI890755 -0.269 -0.589 0.586 0.912 1.268 0.861 0.645 0.329 0.759 0.494 0.265 0.033

BI890823 -0.244 -0.159 0.691 1.14 1.6 1.535 0.912 1.284 0.972 0.822 0.837 0.175

BI890893 0.874 0.005 0.708 0.413 1.103 0.537 0.375 1.064 -0.033 -0.3 -0.119 -0.031

BI891090 -0.208 0.555 0.246 0.929 0.957 0.626 0.304 0.409 -0.005 -0.439 -0.367 -0.173

BI891136 0.025 0.361 0.758 0.955 1.059 0.578 0.688 0.847 0.435 0.313 -0.035 0.226

BI891176 0.195 0.258 0.646 0.741 1.15 0.669 0.816 1.073 0.309 0.395 -0.032 0.092

BI891245 -1.868 -2.408 0.065 1.288 1.382 1.273 0.992 1.096 0.201 0.466 0.709 0.641

BI891279 -1.118 -1.771 0.474 0.973 1.074 0.88 0.705 0.954 0.789 0.743 0.262 -0.45

BI891331 -0.696 -0.05 0.418 0.563 0.666 -0.15 -0.051 0.304 -0.048 0.321 0.174 0.217

BI891338 -0.493 -0.665 1.322 2.059 2.403 0.907 0.367 0.462 0.418 -0.137 -0.194 0.224

BI891429 -0.367 0.558 0.659 0.98 1.101 0.506 0.479 0.83 0.23 -0.048 -0.066 -0.223

BI891492 -0.321 -1.729 0.917 1.394 1.885 1.036 1.3 1.67 0.634 0.804 0.708 0.639

BI891493 -0.326 0.14 0.183 0.539 0.653 0.157 0.159 0.54 0.319 0.132 -0.248 -0.185

BI891527 0.57 -0.035 0.509 0.757 1.159 0.208 0.544 0.963 0.325 -0.003 0.006 -0.434

BI891754 -0.405 -0.111 0.374 0.708 1.411 -0.042 0.046 0.409 0.044 -0.133 0.297 0.275

BI891773 -0.828 -0.697 1.44 1.516 3.962 3.15 1.954 2.415 1.74 0.11 -0.042 -0.386

BI891858 0.236 0.094 0.466 0.746 1.215 0.408 0.331 0.499 0.147 0.291 -0.044 -0.101

BI891859 -0.106 0.101 0.33 0.542 0.575 0.437 0.065 0.011 0.306 -0.574 -0.437 -0.347

BI891877 0.443 0.341 1.468 1.753 1.852 1.587 1.372 1.382 0.841 0.683 -0.064 -0.819

BI891905 0.152 0.667 0.387 0.552 1.389 0.656 0.551 1.309 0.268 0.125 -0.352 -0.454

BI892036 -0.808 -0.22 0.73 1.577 1.897 1.264 0.113 -0.091 -0.274 0.572 0.902 -0.134

BI892041 0.439 0.116 0.777 1.193 1.561 1.592 0.479 0.72 0.077 0.495 0.274 0.565

BI892060 0.16 -0.133 0.757 1.039 1.941 0.74 0.677 1.356 0.398 0.897 0.392 -0.254

BI892068 -0.234 0.46 1.047 2.446 3.409 0.9 1.05 0.813 0.134 -0.122 0.064 0.12

BI892128 -0.5 0.16 1.673 1.781 2.075 1.717 0.788 2.028 0.449 -0.276 0.102 0.198

BI892200 -0.823 -0.459 0.243 1.066 1.667 1.094 1.022 1.034 0.141 0.534 0.644 0.035

BI892244 -0.513 -0.056 2.232 3.232 3.987 2.062 1.974 1.669 1.208 0.137 0.572 0.044

BI892406 -0.149 0.355 1.499 0.908 1.684 1.464 0.716 1.096 0.401 0.362 -0.048 0.014

BI892410 -0.12 1.219 0.658 1.727 2.12 0.401 0.43 1.208 -0.043 -0.321 0.133 -0.039

BI979581 -0.817 -1.189 1.126 1.87 2.524 0.791 1.075 2.093 1.796 -0.808 -0.988 -1.127

BI980148 0.523 0.29 0.583 1.658 2.178 1.664 1.765 1.579 0.88 -0.028 0.109 -0.191

BI980725 0.123 -0.522 0.042 0.58 0.671 0.29 0.204 0.63 -0.117 0.308 0.025 0.45

BI980764 -0.155 0.177 0.512 1.124 1.499 0.547 0.487 1.113 0.153 -0.273 0.054 -0.031

BI980805 0.181 -0.02 0.519 0.747 1.561 0.442 0.294 1.039 0.064 0.033 -0.231 0.03

BI981134 0.03 0.417 0.278 0.54 0.694 -0.077 0.225 0.14 0.037 0.077 -0.111 -0.037

BI982141 -0.121 -1.062 1.356 1.03 1.666 1.508 0.761 -0.324 0.066 0.517 0.629 -0.309

BI983370 1.076 0.901 1.388 1.398 1.529 1.391 0.869 0.903 0.07 0.323 -0.203 -0.235

BI983579 -0.048 -0.111 1.708 1.68 2.141 2.049 1.264 1.447 0.999 0.845 0.337 -0.126

BI984060 0.317 0.207 0.532 0.329 0.704 0.337 0.24 0.606 0.07 0.349 0.107 0.023

BI984272 0.076 0.185 0.275 0.575 0.859 0.136 -0.309 0.849 0.715 0.339 -0.075 0.034

BI984810 -0.074 0.201 0.082 0.499 0.998 0.011 0.125 0.966 0.239 0.298 0.209 0.187

BM023680 -0.011 -0.236 0.462 1.358 1.445 0.885 0.87 0.526 -0.024 -0.026 -0.283 0.058

BM023720 -0.237 -0.23 0.489 0.625 1.111 0.593 0.643 0.786 0.472 0.056 -0.344 -0.077

BM025184 -0.295 -1.02 0.009 1.676 2.839 0.877 1.28 1.111 0.781 -0.187 0.102 0.009

BM026121 -0.106 0.414 1.115 1.333 1.537 0.359 0.605 1.261 0.315 0.177 0.198 -0.005

BM026830 -0.583 0.145 0.397 2.439 3.626 0.064 1.179 0.172 0.121 -0.674 0.009 -0.156

BM036484 -0.542 0.214 0.61 0.705 1.008 -0.159 0.198 0.847 0.126 0.236 0.328 -0.111

BM036509 -0.832 -1.089 1.921 3.063 4.217 3.439 3.199 3.112 2.503 0.124 0.31 -0.36

BM036795 0.201 0.782 1.001 1.14 1.233 0.437 0.368 0.76 0.021 0.178 0.082 0.236

BM036823 0.737 1.155 -0.268 1.286 1.339 0.465 0.654 1.258 0.492 -0.003 -0.326 -0.453

BM070558 0.149 0.833 1.028 1.453 1.928 1.144 1.037 1.28 0.415 0.242 0.402 0.339

BM071679 0.378 1.01 1.705 1.556 1.776 0.869 0.902 1.281 0.464 0.71 0.131 0.122

BM072333 -0.131 0.357 0.284 1.288 1.328 0.864 0.616 0.968 0.402 0.056 0.334 0.332

BM072353 0.473 0.54 0.742 0.924 1.4 0.864 0.862 1.348 0.505 0.48 -0.197 -0.567

BM083944 0.127 0.415 0.645 0.595 1.022 0.341 0.286 0.556 0.076 0.378 0.251 0.158

BM083945 -0.678 0.306 0.34 0.776 1.097 0.217 0.422 1.076 0.525 -0.129 -0.106 -0.469

BM083966 -0.477 0.181 0.802 2.011 2.5 1.293 1.129 1.728 0.337 -0.074 0.132 0.9

BM096052 0.338 0.893 0.545 0.781 1.119 0.756 0.268 1.033 0.254 0.441 -0.164 -0.131

BM101748 -0.418 0.236 0.359 1.428 1.839 0.325 0.58 1.186 0.205 -0.256 0.444 -0.262

BM103255 -0.553 -0.795 0.761 0.706 0.836 0.138 0.404 -0.153 0.458 0.168 -0.255 -0.722

BM104310 0.246 0.068 1.656 1.399 1.958 1.705 0.66 1.532 1.546 1.585 0.985 0.111

BM104683 0.807 1.779 2.227 2.012 2.262 1.8 1.856 2.086 0.958 1.073 0.376 0.265

BM154123 -0.416 0.073 0.541 0.764 1.158 -0.164 0.048 0.531 -0.044 -0.241 0.01 -0.224

BM156110 -0.319 0.211 0.928 1.469 1.767 1.709 1.163 1.454 0.577 0.456 0.411 0.462

BM181840 0.45 0.729 0.807 1.245 1.359 0.631 0.805 1.087 0.079 0.245 0.001 0.08

BM182574 -0.728 0.153 0.303 0.514 0.875 0.116 -0.087 0.629 -0.099 -0.203 0.076 -0.218

BM184035 0.587 0.685 1.359 1.391 1.538 1.314 1.225 1.163 0.467 0.168 -0.384 -0.183

BM184248 0.067 -0.07 -0.375 0.895 1.088 -0.219 -0.367 -0.422 -0.579 -1.084 -0.88 -0.899

BM184367 -1.181 0.405 1.523 2.016 2.175 0.921 -0.064 1.287 0.516 0.565 0.258 0.215

BM185158 -0.132 0.013 1.945 3.112 3.293 2.218 1.281 1.514 0.715 -0.062 0.191 0.333

BM186588 -0.012 0.043 -0.04 0.494 0.478 0.087 0.179 0.258 -0.09 -0.083 0.092 0.016

D26174 -0.284 0.052 -0.004 1.143 1.975 0.321 0.575 1.242 0.297 -0.121 1 0.015

L46801 0.534 0.449 1.877 2.076 2.922 2.188 1.918 2.095 1.126 0.414 0.188 -0.149

S76875 -0.04 0.633 0.589 0.869 1.157 0.376 0.227 0.69 0.086 -0.241 0.077 -0.353

U10869 0.055 0.572 0.685 0.983 2.823 0.173 0.76 1.707 0.163 -0.011 -0.134 -0.074

U23839 -0.393 -0.134 0.838 1.611 2.002 0.903 1.071 1.917 0.833 0.502 0.71 0.194

U41081 -0.488 -0.143 1.492 1.729 1.731 1.694 1.771 1.534 1.014 0.996 0.69 0.283

U68234 -0.236 0.873 0.433 1.208 2.995 1.007 1.277 1.841 0.342 -0.21 0.368 -0.324

AA494762 0.505 -0.047 0.173 0.56 0.624 0.742 0.684 0.446 0.475 0.064 -0.043 -0.186

AA495032 -0.181 -0.012 0.363 0.807 1.041 1.164 0.697 0.495 0.142 0.202 -0.017 0.121

AA605765 0.749 1.146 2.046 1.648 1.7 2.385 1.314 1.738 0.828 0.704 0.182 0.011

AB030899 -0.131 -1.333 0.113 0.429 0.994 1.176 0.857 0.967 0.45 0.391 0.494 0.16

AB055677 -1.444 -2.002 2.149 3.533 4.187 4.457 3.943 3.753 3.494 1.27 0.464 -0.705

AF007414 0.524 -1.877 1.262 0.47 0.477 1.456 0.91 0.807 0.949 0.972 0.879 0.077

AF075384 -1.18 0.235 0.404 0.464 0.425 0.839 0.444 0.389 0.634 0.617 0.503 0.253

AF093129 -1.54 -1.446 0.378 0.752 0.834 1.562 1.1 0.85 0.377 0.503 0.231 0.039

AF151535 -1.002 -0.486 0.271 0.802 1.512 2.165 1.236 1.618 0.843 0.878 1.336 0.856

AF160646 -0.299 -0.131 0.385 0.697 1.293 1.662 0.914 1.302 1.022 0.924 0.603 0.087

AF168008 -0.308 0.116 0.876 1.313 1.504 1.804 0.889 1.415 0.377 0.404 0.515 0.261

AF169146 0.215 0.419 0.692 0.782 0.635 0.85 0.182 0.211 0.034 0.058 0.223 0.002

AF210638 -0.371 0.095 0.697 0.318 0.362 0.792 0.313 0.554 0.287 0.691 0.33 0.086

AF222996 -1.021 -0.438 0.325 0.806 1.402 1.757 1.288 1.089 0.955 0.837 0.542 0.704

AF258786 0.62 0.941 0.474 0.551 0.983 1.272 0.298 0.896 0.239 -0.318 -0.409 -0.388

AF274877 0.095 0.558 0.122 0.587 0.677 1.137 0.273 0.804 0.228 0.401 0.338 0.127

AF295373 0.546 -0.135 0.76 0.722 0.509 1.227 0.575 0.477 0.186 0.288 -0.117 -0.515

AF337035 -0.722 -0.503 0.686 0.739 0.448 1.055 0.587 0.151 0.274 0.449 0.274 0.834

AF359425 -0.992 0.095 1.926 1.36 1.619 1.774 1.296 1.245 0.265 0.406 0.362 -0.302

AF398433 -0.455 0.236 0.643 0.601 1.95 2.425 1.344 1.659 1.19 0.167 0.637 0.096

AI322210 0.442 -0.646 1.248 0.241 0.894 1.7 1.392 0.795 0.879 0.557 0.334 -0.499

AI354177 -0.336 0.27 0.211 0.358 0.664 1.093 0.646 0.947 0.325 0.216 -0.043 0.038

AI385123 -0.453 -0.376 0.432 0.406 1.204 1.823 1.231 1.413 1.259 0.395 0.439 0.247

AI416338 0.308 0.37 1.191 1.312 1.297 1.639 1.251 1.06 0.708 0.346 0.058 -0.289

AI477017 1.506 -0.515 0.621 1.004 1.389 1.994 1.16 1.452 0.888 0.757 -0.007 -0.564

AI477432 0.338 0.127 0.755 0.766 0.774 1.349 0.993 1.105 0.715 0.406 0.307 0.257

AI545284 -0.187 -0.769 0.34 0.057 1.041 1.21 0.552 0.771 0.562 0.41 0.504 0.377

AI545320 0.348 0.042 0.842 0.593 0.79 1.042 0.978 0.831 0.605 0.494 0.524 0.264

AI558351 -0.774 -0.705 0.49 0.338 1.178 1.902 1.068 1.295 1.039 0.704 1.115 0.679

AI588119 0.559 0.213 0.123 0.379 0.972 1.73 0.689 1.441 1.033 0.977 0.541 0.036

AI601641 0.384 1.425 -0.648 -0.115 1.136 1.503 0.612 1.368 0.765 -0.259 0.074 0.041

AI601856 0.392 0.307 0.127 0.694 0.928 1.055 0.77 0.237 0.212 0.058 0.034 0.285

AI641022 -0.327 -0.016 1.234 0.821 1.636 1.909 1.426 1.858 1.155 1.089 0.366 0.319

AI641052 0.185 0.342 1.664 1.511 1.641 2.115 1.561 1.352 1.171 1.08 0.581 0.538

AI641092 -0.824 -1.091 0.25 0.571 1.685 2.137 1.515 0.862 1.178 0.875 1.039 0.792

AI641409 -0.197 -0.576 1.189 0.473 1.036 1.642 0.824 1.416 0.585 0.159 0.072 0.021

AI641534 -1.09 -1.603 -0.709 -0.182 -0.053 0.813 0.509 0.667 0.37 0.645 0.348 0.135

AI657697 0.163 0.479 0.868 1.062 1.009 1.234 0.817 1.075 0.605 0.449 0.072 -0.113

AI666882 0.26 0.045 1.103 1.61 1.632 1.736 1.657 1.429 0.796 0.682 0.01 -0.07

AI667167 -0.613 -0.133 1.09 0.248 0.846 1.755 0.972 1.149 1.245 0.806 0.397 0.227

AI667325 -0.598 -0.28 1.121 0.333 0.682 1.574 0.927 1.124 0.786 0.921 0.682 1.106

AI721340 0.433 -0.438 0.269 0.028 0.757 1.373 0.738 1.051 0.793 0.768 0.457 0.267

AI721419 -0.464 -1.437 -1.735 -1.2 -0.273 1.44 0.916 1.195 0.501 0.619 -0.011 -0.364

AI722650 -0.292 0.327 -0.009 0.211 0.895 1.289 0.768 1.201 0.816 0.342 0.206 0.164

AI723170 0.006 0.093 1.063 0.931 0.887 1.412 1 1.195 1.034 0.901 0.516 0.273

AI723269 0.601 1.203 1.872 1.617 1.461 1.903 1.756 1.564 0.847 0.7 0.159 -0.096

AI793490 0.348 0.044 0.244 0.586 0.333 0.752 0.537 0.206 0.156 -0.007 -0.123 -0.17

AI793637 -1.564 -1.937 0.593 1.126 1.301 1.884 1.595 1.335 0.873 1.228 0.708 0.565

AI793769 -0.569 -0.448 -0.277 0.011 0.334 0.862 0.444 0.736 0.27 0.771 0.218 0.231

AI793830 -0.348 -0.007 0.559 1.02 1.138 1.832 0.884 1.421 0.943 1.125 0.448 0.123

AI793839 -1.442 -0.725 0.824 0.78 1.624 2.048 1.371 1.703 1.114 0.755 0.401 1.15

AI794059 -0.196 -0.539 -0.123 0.075 0.925 1.626 1.021 1.183 0.916 0.755 0.559 0.532

AI794204 0.202 0.395 0.867 0.801 0.909 0.978 0.899 0.76 0.123 0.603 0.258 0.015

AI878421 0.928 0.298 0.001 0.359 0.66 1.096 1.008 0.962 0.632 0.727 0.55 0.736

AI878755 0.516 0.607 0.799 0.811 1.377 1.699 0.833 1.014 0.508 0.453 0.307 0.312

AI883443 -0.609 -0.381 0.454 0.59 0.807 0.921 0.411 0.795 0.354 0.664 0.392 0.433

AI883925 0.534 0.306 0.09 0.48 0.422 0.824 0.191 0.835 0.559 0.68 0.343 -0.08

AI883929 -0.099 -0.297 0.153 0.398 0.549 0.643 0.133 0.184 0.088 0.001 -0.073 0.079

AI884112 1.236 0.976 1.369 1.842 1.645 2.304 1.567 1.26 0.598 0.487 0.071 -0.335

AI942952 0.683 0.694 1.03 1.305 2.06 2.016 2.097 1.57 1.59 0.455 -0.444 -0.519

AI943062 1.088 1.142 1.202 1.477 1.633 1.909 0.91 1.149 0.18 0.328 0.066 -0.134

AI943108 0.094 -0.52 0.018 0.035 0.251 0.975 0.464 0.526 0.725 0.696 0.817 0.23

AI957472 0.583 0.498 0.649 0.819 1.125 1.292 0.762 0.624 0.48 0.306 -0.025 -0.078

AI957711 -0.701 -0.494 -0.405 0.512 0.84 1.262 0.538 0.731 0.537 0.295 0.158 -0.161

AI958208 -0.514 0.139 0.939 0.894 1.072 1.263 0.683 0.912 0.67 0.711 0.442 0.594

AI964130 0.869 1.036 2.211 2.089 2.08 2.145 1.808 1.322 0.708 0.57 0.145 0.097

AI964204 0.243 -0.413 0.616 0.969 1.214 1.337 0.509 1.149 0.682 0.282 0.141 0.238

AI964232 -0.299 -0.054 0.428 0.958 1.247 1.649 0.884 1.518 0.848 0.471 0.094 -0.369

AI964300 0.537 0.311 1.499 1.12 1.213 1.572 0.928 0.812 0.672 0.209 0.062 -0.117

AI964318 0.019 -0.1 0.453 0.368 0.921 1.184 0.796 1.044 0.18 0.108 -0.351 -0.859

AI974191 -0.413 -0.907 1.633 1.901 2.286 2.464 2.089 2.321 0.974 0.744 0.218 -0.394

AJ236882 -5.67 -3.087 1.093 1.801 2.022 2.735 2.118 1.986 1.444 1.6 1.121 0.061

AJ245965 0.024 0.446 0.157 0.569 1.049 1.102 0.557 1.021 0.386 0.698 0.639 0.072

AW019116 0.571 0.185 0.621 0.97 1.212 1.627 0.977 0.958 0.407 0.364 0.003 0.004

AW019187 -0.874 -0.461 0.833 1.219 1.198 1.379 1.178 0.686 0.438 0.06 -0.141 -0.054

AW019482 -0.72 0.137 0.945 1.542 1.862 2.158 1.461 1.758 0.798 0.659 0.443 0.291

AW059069 0.311 1.188 1.583 1.983 1.633 2.62 2.126 2.5 1.704 0.55 -0.009 0.039

AW059156 -0.661 -0.711 -0.209 0.756 1.612 1.994 1.079 1.573 0.915 0.75 0.686 0.608

AW059234 -1.056 -0.709 -0.091 0.332 1.507 2.547 2.236 2.037 1.279 0.884 -0.048 -0.151

AW077190 0.074 -0.456 0.536 1.077 0.994 1.385 0.868 1.307 0.559 0.486 0.045 0.071

AW077193 -0.081 0.14 1.186 1.099 1.192 1.396 1.008 1.37 0.666 0.424 0.108 0.137

AW077755 -0.041 -0.315 -0.148 0.497 0.526 1.095 0.565 0.665 0.183 0.324 0.034 0.404

AW115809 -0.118 -0.698 -0.013 -0.078 0.132 0.904 0.536 0.751 0.46 0.608 0.349 0.205

AW116226 0.287 0.543 0.858 0.704 0.877 1.48 0.697 0.744 0.338 0.342 -0.107 0.018

AW116374 0.124 0.481 0.866 0.714 0.828 0.871 0.782 0.763 -0.148 0.353 -0.116 -0.233

AW116425 0.321 -0.637 0.278 0.85 0.885 1.145 0.691 0.932 0.193 0.474 0.027 -0.093

AW116453 -0.075 -0.592 -0.241 0.553 0.9 1.251 1.161 1.112 1.093 0.557 0.041 -0.288

AW116556 -0.129 0.516 0.423 0.458 0.499 0.636 0.239 0.317 -0.037 -0.086 -0.192 -0.404

AW116617 1.164 0.874 1.055 1.172 1.148 2.02 0.9 1.451 0.382 0.559 0.27 -0.002

AW116947 1.236 0.505 0.956 1.217 1.221 1.419 0.829 1.037 0.177 0.471 -0.094 -0.018

AW128293 0.898 1.158 1.219 0.862 0.92 1.376 0.501 0.876 0.483 0.6 0.213 -0.585

AW128316 0.533 0.757 0.634 0.456 0.461 0.841 0.351 0.817 0.291 0.084 -0.097 -0.417

AW128354 0.458 0.223 0.798 0.66 0.751 1.138 0.383 0.799 0.625 0.669 0.174 0.147

AW154413 0.862 0.47 1.101 1.396 1.43 1.529 1.239 1.41 0.399 0.271 -0.08 0.057

AW154725 0.945 1.027 1.404 1.534 1.442 1.922 1.227 0.995 0.371 -0.098 -0.397 -0.116

AW165108 0.566 0.342 1.388 1.127 1.614 2.253 1.434 1.999 1.012 1.015 0.523 0.814

AW165150 -1.089 -0.023 1.427 1.321 2.062 2.211 1.078 1.628 0.703 0.591 0.312 0.285

AW171011 0.32 -0.569 0.953 1.037 1.08 1.518 1.208 0.945 0.948 0.336 -0.114 -0.163

AW171012 0.84 0.668 0.155 0.405 0.521 1.242 0.457 0.98 0.344 0.205 -0.083 -0.264

AW171228 -0.034 -0.208 1.343 1.244 1.08 1.389 0.999 1.205 0.906 0.933 0.543 0.409

AW171254 0.464 -0.553 0.21 0.504 0.612 0.955 0.476 0.665 0.413 0.486 0.221 0.226

AW171454 -0.283 -0.063 0.018 0.295 0.679 1.254 0.515 1.25 0.621 0.857 0.376 0.249

AW203061 -1.304 -0.998 -0.442 0.148 0.713 1.429 0.707 1.075 0.895 1.033 0.507 0.258

AW203151 0.578 0.55 1.365 1.15 1.147 2.004 1.441 1.395 0.912 0.887 0.548 0.191

AW232003 -0.38 -0.255 1.567 1.959 1.653 2.21 2.064 1.948 1.125 0.661 0.161 0.267

AW232641 0.629 -0.723 0.217 0.707 1.293 1.914 1.536 1.577 1.116 1.052 0.429 -0.164

AW232866 -1.023 -0.546 -0.786 -0.074 0.631 2.158 1.541 1.99 1.424 0.46 -0.095 -0.226

AW233578 0.066 -0.61 0.567 -0.08 1.002 1.086 0.199 -0.178 -0.092 -0.188 -0.551 -0.135

AW280174 0.87 -0.103 0.08 0.687 0.604 1.356 1.242 1.233 0.643 0.884 0.415 0.257

AW305388 0.553 0.168 0.443 0.643 0.877 1.888 1.101 1.388 0.946 1.128 0.938 0.51

AW305456 -0.233 -0.214 -0.336 0.472 0.491 0.742 0.653 0.266 0.646 0.303 0.325 0.104

AW342762 -0.174 0.54 1.771 1.505 1.597 2.172 1.592 1.808 1.451 0.82 0.543 0.356

AW342840 0.739 -0.073 0.092 0.139 0.384 1.054 0.764 0.81 0.294 0.063 -0.137 -0.572

AW343764 1.17 0.703 0.983 1.197 1.298 1.631 1.137 1.283 0.636 0.37 -0.515 -1.036

AW421191 -1.226 -0.49 -0.224 0.924 1.42 1.404 1.054 0.995 0.458 -0.003 -0.19 -0.238

AW595163 -0.281 0.604 1.283 1.406 1.503 2.125 1.462 1.608 0.654 1.041 0.477 0.301

AW777355 0.753 0.203 0.736 0.664 0.679 1.237 0.919 0.885 0.721 0.543 0.231 -0.008

AW777430 0.613 -0.148 0.717 0.675 0.637 1.325 0.884 0.479 0.697 0.712 0.467 0.014

AY026507 1.134 1.436 1.375 0.976 1.575 1.622 0.943 1.301 0.496 0.569 0.2 -0.434

BE016123 0.806 0.394 0.197 0.285 0.404 0.955 0.567 0.504 0.155 -0.318 -0.791 -0.664

BE017652 -1.536 -0.098 0.239 0.418 1.231 1.86 1.504 1.459 0.999 0.557 0.271 -0.957

BE201470 -3.167 -1.166 0.12 0.73 0.907 1.468 0.849 0.831 0.873 0.891 0.373 0.364

BE202229 0.522 -0.773 0.196 0.605 0.882 1.213 1.009 1.1 0.681 0.48 -0.081 -0.179

BE557308 0.017 -0.636 0.212 0.433 0.339 1.201 0.781 0.964 0.625 1.055 0.615 0.198

BE693152 -0.338 0.173 0.705 0.821 0.851 1.33 0.865 1.176 0.501 0.69 0.416 0.357

BF718182 -0.725 -0.545 -0.939 0.616 0.749 0.866 0.312 0.676 0.215 0.777 0.423 0.815

BG304114 0.293 0.605 1.356 0.916 0.944 1.453 0.592 0.857 0.711 0.695 0.1 0.293

BG304168 -0.146 -0.082 0.527 0.36 0.496 0.909 0.403 0.829 0.291 0.401 0.088 0.051

BG304285 0.438 0.763 0.892 0.795 0.981 1.086 0.514 0.64 0.223 0.083 -0.139 -0.085

BG305296 0 -0.569 -0.178 0.062 0.31 1.359 0.498 1.102 0.566 0.596 0.488 0.068

BG305790 -0.653 0.033 1.14 1.154 1.736 1.791 1.188 1.785 0.532 0.399 0.296 0.083

BG305892 1.007 1.177 1.098 1.248 1.54 1.95 1.4 0.77 0.532 -0.09 0.422 -0.189

BG306390 1.019 0.343 1.777 1.25 1.505 2.005 1.641 1.624 0.915 0.879 0.434 0.201

BG306405 0.144 0.368 1.268 1.287 1.34 2.351 1.345 1.481 0.771 0.889 0.638 0.563

BG727207 0.322 0.214 0.745 1.111 1.024 1.162 0.746 1.093 0.447 0.529 0 -0.036

BG727431 -0.814 0.123 0.488 0.704 1.38 1.51 0.744 0.93 0.526 0.27 0.277 0.253

BG799259 0.268 0.853 1.432 1.605 1.458 1.78 0.648 1.409 0.451 0.701 0.3 -0.144

BG799399 -0.777 -0.694 1.608 3 4.15 4.333 3.419 3.418 2.052 0.008 0.025 -0.241

BG883671 0.005 0.215 0.765 0.679 1.193 1.241 0.565 0.953 0.681 0.489 0.242 0.349

BG985468 -1.635 -1.49 -0.657 -0.15 0.419 1.35 0.973 1.159 0.906 0.68 0.195 -0.218

BG985470 0.483 0.016 0.212 0.576 1.37 1.464 0.449 1.033 0.217 0.473 0.062 0.408

BG985493 0.379 -0.001 0.841 0.602 0.78 1.435 1.206 0.955 0.589 0.43 0.071 -0.034

BG985503 -0.612 -0.164 1.419 1.396 1.722 1.968 1.606 1.962 1.2 1.045 0.958 -0.26

BG985504 -0.057 0.376 0.675 0.424 0.681 0.794 0.101 0.587 0.292 0.338 0.325 0.074

BG985688 1.008 1.091 0.733 0.953 1.034 1.173 0.695 0.847 0.215 0.169 -0.382 -0.827

BG985846 -0.622 -0.493 1.243 1.426 1.57 2.372 2.141 1.812 1.375 0.889 0.503 0.223

BI428973 -0.406 -0.284 1.891 2.453 2.338 2.51 1.666 1.07 0.827 0.821 0.44 0.616

BI429605 -0.416 -0.616 0.092 0.241 0.459 0.947 0.506 0.703 0.344 0.109 0.327 0.006

BI474299 -0.368 -0.823 1.011 1.127 1.853 2.506 2.282 2.019 1.33 0.804 0.309 -0.186

BI475794 1.108 0.485 1.92 1.892 2.347 2.82 2.145 2.367 1.589 1.619 1.017 0.375

BI476846 -0.546 -1.171 -1.035 0.201 0.715 1.199 0.479 0.38 0.234 0.752 0.489 1.006

BI476854 -1.031 -0.864 0.567 1.176 1.276 1.893 1.312 1.323 0.636 0.836 0.459 0.749

BI533160 0.686 -0.172 0.495 0.916 1.614 2.26 1.59 2.254 1.444 1.623 1.002 0.405

BI533952 1.515 1.093 1.14 1.623 1.902 2.304 1.646 1.891 0.953 0.857 0.419 -0.161

BI671110 0.693 0.865 1.258 1.018 0.889 1.353 0.389 0.852 0.213 0.235 0.119 -0.031

BI671843 0.981 0.709 0.699 0.495 1.081 1.446 0.977 1.355 0.8 -0.191 -0.577 -0.349

BI672394 0.8 0.316 0.466 0.734 1.169 1.874 1.096 0.768 0.222 0.236 0.065 -0.173

BI673416 0.415 0.891 2.481 2.38 2.425 2.695 2.027 1.668 0.991 0.813 0.404 -0.074

BI704281 -4.509 -4.245 0.937 1.408 1.815 2.267 1.964 1.85 1.03 0.217 0.049 -0.251

BI704288 -0.894 -0.719 1.727 2.316 2.584 3.313 2.896 2.643 1.91 1.66 1.137 0.856

BI704340 0.287 0.393 0.872 0.953 1.269 1.434 1.216 1.16 0.764 0.705 0.359 0.344

BI705525 0.913 0.826 0.36 0.629 0.734 1.295 0.191 0.768 0.267 0.271 -0.195 -0.177

BI705891 0.076 -0.753 0.636 0.627 0.696 1.263 0.992 1.09 0.645 0.533 0.268 0.382

BI708259 0.791 0.14 0.616 1.035 1.09 1.226 0.64 0.976 0.436 0.828 0.341 0.321

BI708386 0.701 0.832 1.589 1.486 1.719 1.861 1.322 1.448 0.979 0.428 0.281 -0.1

BI840839 0.129 -0.408 0.438 0.397 0.514 1.425 0.553 0.831 0.749 0.567 0.566 0.323

BI840935 0.209 -0.514 0.441 0.929 0.445 1.432 0.696 0.232 0.261 0.718 0.275 -0.303

BI843229 -0.197 -0.159 0.272 0.295 0.645 1.666 0.914 1.167 0.626 1.01 0.149 0.162

BI865412 0.194 -0.362 0.465 0.368 0.402 1.24 0.365 0.484 0.31 0.609 0.398 0.415

BI867819 -0.281 -0.249 1.511 1.684 1.521 1.821 1.231 1.866 0.62 0.598 0.141 0.291

BI877921 0.281 -0.439 0.729 0.519 0.675 1.277 1.193 0.899 0.417 0.414 -0.02 0.047

BI878078 -0.082 0.041 0.61 1.364 1.461 2.308 1.46 1.825 1.186 1.38 0.699 0.535

BI878102 0.5 0.884 1.149 1.288 1.07 1.511 0.962 1.088 0.688 0.711 0.114 -0.147

BI878820 0.534 0.874 1.336 1.253 1.384 1.809 0.923 1.11 0.333 0.481 0.045 0.521

BI879262 -0.437 -0.354 0.563 0.638 0.566 1.416 0.814 0.716 0.233 0.507 0.405 0.487

BI879735 0.526 0.463 0.792 0.759 1.127 1.658 0.705 1.407 0.481 0.899 0.308 0.166

BI880061 0.262 0.555 1.904 1.459 2.139 2.208 1.57 2.18 1.066 1.246 0.71 0.75

BI880125 0.321 -0.05 0.512 0.455 0.866 1.326 0.593 0.526 0.448 0.692 0.312 0.338

BI881679 -0.39 -0.395 1.395 1.655 1.644 2.129 1.82 1.645 1.028 0.607 0.174 0.125

BI882568 -0.029 0.307 0.641 0.672 0.793 1.09 0.373 0.719 0.303 0.151 -0.184 -0.406

BI883251 0.101 -0.434 0.163 0.344 1.281 1.334 0.744 0.944 0.563 0.687 0.49 0.421

BI883252 0.018 -0.384 0.256 1.169 2.185 2.142 1.616 2.184 0.891 0.477 0.538 -0.261

BI883674 -0.248 -0.459 0.97 1.757 1.453 1.901 1.769 1.394 0.92 0.538 0.154 0.076

BI883953 0.114 0.064 0.262 0.655 1.233 2.162 1.285 2.023 1.472 1.516 0.964 0.473

BI885492 -1.278 -0.993 -0.203 0.33 0.324 1.172 0.583 0.626 0.414 0.852 0.67 0.465

BI885768 0.066 -0.015 1.031 0.788 0.932 1.66 0.815 0.939 0.3 0.218 0.184 0.096

BI885777 -0.784 -0.029 0.569 0.662 0.821 1.282 0.758 0.736 0.451 0.552 0.249 0.475

BI885824 0.109 -0.025 0.496 0.772 0.62 0.893 0.551 0.797 0.218 0.394 0.223 -0.137

BI886025 -2.881 -1.279 -0.295 -0.28 0.686 1.605 1.052 1.385 0.65 1.423 1.05 0.933

BI886095 -1.066 -0.774 0.889 0.964 1.302 2.394 1.851 1.67 1.44 1.299 1.194 1.203

BI886248 -0.283 -0.412 0.353 0.181 0.364 0.826 0.359 0.442 0.298 0.611 0.342 0.234

BI886935 0.432 -0.64 -0.077 0.103 0.119 0.882 0.33 0.565 0.288 0.341 0.085 -0.052

BI887226 0.631 0.774 1.577 1.514 1.704 2.587 2.379 2.332 1.737 1.325 0.847 0.672

BI887270 -0.852 -0.655 0.027 0.392 0.578 0.678 0.374 0.395 0.221 0.161 0.25 0.603

BI887468 0.077 -0.517 0.377 0.148 0.222 0.756 0.239 0.708 0.655 0.582 0.138 0.187

BI887512 -0.01 -0.297 0.309 0.401 0.896 1.086 0.374 1.037 0.313 0.662 0.113 0.246

BI887540 -0.95 -0.795 1.422 1.292 1.359 2.048 1.594 1.583 1.454 1.34 0.943 0.261

BI887548 -0.725 0.523 2.469 2.135 2.426 3.137 2.288 2.011 1.243 1.528 0.989 0.844

BI887651 0.047 -0.561 0.071 0.169 0.882 1.121 0.733 0.842 0.241 -0.02 -0.135 -0.136

BI887704 0.016 0.222 0.854 0.523 0.784 1.005 0.552 0.881 0.441 0.495 0.257 0.411

BI887709 -1.484 -0.61 0.814 1.017 1.389 2.209 1.917 1.841 1.374 1.642 0.772 1.117

BI887931 -0.629 -0.056 0.427 0.792 1.069 1.367 0.726 1.058 0.374 0.412 0.642 0.431

BI888024 0.143 -0.936 0.281 0.619 0.761 0.774 0.684 0.575 0.476 0.383 -0.002 0.245

BI888090 -0.024 -0.167 0.765 0.549 2.432 2.316 1.942 2.327 1.465 0.27 0.057 -0.109

BI888149 -0.111 -0.326 0.14 0.55 0.956 0.967 0.753 0.723 0.279 0.106 -0.016 0.11

BI888150 1.062 -2.111 0.455 1.152 1.217 1.738 1.336 0.92 0.655 1.051 0.639 0.2

BI888169 -0.002 -1.276 -0.706 -0.244 0.144 1.06 0.839 0.81 0.32 0.892 0.418 0.145

BI888550 -0.756 -0.802 -0.356 0.293 0.844 1.217 0.629 0.812 -0.286 -0.878 -1.165 -1.088

BI888721 -0.525 0.77 1.886 1.942 2.106 2.55 1.879 2.022 1.506 0.724 -0.212 -1.139

BI888859 -0.029 0.081 0.701 0.675 1.043 1.688 1.078 1.331 0.88 0.758 0.141 0.417

BI888926 -0.608 -1.851 -0.112 0.11 0.638 1.155 0.613 0.55 -0.029 0.704 0.383 0.495

BI889190 -0.643 -1.184 0.411 1.186 1.448 1.606 0.88 1.167 0.5 0.29 0.528 0.24

BI889241 -0.661 -0.451 0.839 1.163 1.29 1.395 0.633 0.888 0.379 -0.068 0.596 -0.063

BI889298 -0.963 -0.727 1.212 1.632 1.616 1.92 1.521 1.855 1.465 1.092 0.472 0.53

BI889456 -0.566 -0.272 0.348 0.576 0.838 1.186 0.624 1.047 0.575 0.367 0.239 -0.019

BI889700 1.168 0.491 0.378 0.794 1.082 1.364 0.895 0.817 0.051 -0.233 -0.257 -0.623

BI890235 -0.289 -0.044 0.036 0.616 0.252 0.778 0.265 0.721 0.236 0.623 0.148 0.385

BI890257 0.049 0.658 0.491 0.495 0.747 0.835 0.401 0.806 0.181 0.194 -0.003 0.026

BI890444 -0.087 -0.021 0.71 0.652 1.47 1.837 0.662 1.028 0.674 0.644 0.37 0.228

BI890446 -5.187 -4.116 0.084 0.595 1.476 2.73 2.164 2.261 1.733 1.813 1.288 1.067

BI890477 -0.378 -0.06 1.048 0.248 1.597 1.577 0.376 1.105 0.574 0.249 0.156 -0.009

BI890607 -0.68 -1.293 -0.427 -0.061 -0.044 0.549 0.192 0.444 0.158 0.333 0.051 -0.329

BI890609 0.567 -0.045 0.172 0.224 0.632 1.762 1.62 0.936 0.869 0.138 -0.419 -0.864

BI890669 -0.011 0.059 1.836 0.835 2.062 2.269 1.703 1.855 1.04 0.883 0.553 0.23

BI890821 0.599 0.75 1.667 1.795 1.393 1.971 1.662 1.526 0.871 1.079 0.876 0.525

BI890848 1.3 -0.596 1.444 1.07 1.136 1.949 0.975 1.323 1.069 0.578 -0.068 -0.389

BI891276 0.109 -0.73 0.448 0.585 0.612 0.694 0.348 0.725 0.214 0.344 0.417 -0.147

BI891423 -0.526 -0.081 0.793 0.609 0.976 1.246 0.653 1.158 0.406 0.7 0.264 0.424

BI891472 -0.293 -0.688 0.925 0.692 0.935 1.083 0.425 0.733 0.658 0.261 0.471 0.146

BI891684 0.64 0.509 1.25 1.796 1.729 1.798 1.338 1.521 1.137 0.57 0.149 -0.447

BI891793 -0.728 -0.335 2.14 1.682 2.526 2.648 2.007 2.011 1.134 0.097 0.566 0.387

BI892066 -0.363 -0.414 0.556 0.342 0.895 0.883 0.554 0.57 0.332 0.306 0.023 0.023

BI892135 -0.199 0.747 2.318 1.786 1.74 2.348 1.313 1.145 0.584 0.461 -0.082 -0.135

BI892155 -0.319 -0.307 1.614 2.161 2.241 2.704 2.01 2.167 1.038 0.79 0.355 0.414

BI892201 0.341 0.247 0.338 0.293 0.783 1.176 0.894 1.006 0.663 0.099 0.161 -0.127

BI892210 -2.439 -2.367 -0.129 0.23 1.147 1.659 1.434 1.532 1.332 1.308 0.993 1.009

BI892237 0.553 0.465 1.344 0.675 1.218 1.726 0.913 0.896 0.359 0.355 -0.2 -0.032

BI896231 -1.151 -1.418 -0.402 -0.082 -0.078 1.014 0.593 0.812 0.682 0.824 0.501 0.029

BI979115 -0.03 0.334 0.46 0.33 0.519 0.616 0.227 0.613 0.145 -0.044 -0.233 -0.049

BI979955 1.272 1.184 0.859 1.2 1.062 1.67 0.316 1.025 0.172 0.38 0.043 0.215

BI980138 0.342 -0.22 0.086 0.407 0.679 1.142 0.413 0.762 0.211 0.268 -0.013 -0.171

BI980224 1.625 1.308 2.334 1.236 1.695 2.59 2.08 2.035 1.458 0.827 0.402 -0.5

BI980448 -0.396 0.227 0.521 0.71 0.554 1.321 0.647 1.247 0.77 1.1 0.713 0.427

BI980758 0.345 -0.078 -0.066 0.271 0.216 0.69 0.392 0.048 0.427 -0.018 0.351 0.606

BI983044 0.213 0.09 0.031 0.264 0.432 1.123 0.591 0.591 0.466 0.271 0.013 -0.191

BM005077 0.548 0.077 -0.136 0.011 0.43 0.759 0.226 0.135 0.439 0.344 0.374 0.441

BM036406 0.86 0.689 0.485 0.64 0.556 1.038 0.526 1.01 0.436 0.146 -0.294 -0.736

BM072263 -0.124 -1.727 0.92 0.719 3.206 3.052 2.56 2.146 1.799 -0.001 -0.074 0.119

BM083940 0.243 -0.894 0.175 0.614 1.082 1.178 0.693 0.842 0.969 0.103 0.334 0.181

BM101524 0.961 0.937 1.8 1.434 1.779 2.168 1.556 1.693 0.887 0.791 -0.197 -0.616

BM101651 1.225 1.091 1.942 1.807 1.705 2.256 1.988 2.148 1.337 1.087 0.439 -0.15

BM101689 0.514 -0.129 0.086 0.194 0.879 1.226 0.403 0.597 0.725 0.483 0.677 0.466

BM103822 -0.228 0.162 0.826 0.553 0.806 1.076 0.597 0.976 0.529 0.659 0.236 -0.036

BM104287 0.505 0.404 1.326 0.726 1.196 1.249 0.66 1.156 0.394 0.824 0.086 0.115

BM104315 0.805 1.23 1.685 1.591 1.23 1.872 0.578 0.888 0.277 -0.07 -0.424 -0.574

BM154625 -0.344 -0.156 0.402 1.139 1.182 1.408 0.515 1.313 0.265 0.059 0.091 -0.106

BM155225 1.698 0.842 1.388 1.632 1.652 1.903 1.532 1.095 0.275 -0.065 -0.472 -0.704

BM182450 -0.395 -0.015 0.683 0.778 1.024 1.195 0.83 1.046 0.152 0.287 -0.085 -0.129

BM185124 0.687 -0.161 0.633 0.808 0.835 0.966 0.773 0.667 -0.006 -0.212 -0.475 -0.644

BM185394 0.23 -0.364 0.004 -0.002 0.525 1.176 0.482 0.857 0.403 0.551 0.666 0.159

L47669 -2.241 -1.942 0.378 0.997 1.079 1.453 1.127 0.92 0.463 0.72 0.449 0.124

U49417 0.27 1.065 2.324 1.579 2.096 2.487 1.62 1.71 0.906 0.25 0.294 0.013

U61395 0.595 -0.562 0.804 1.183 1.228 2.068 1.489 1.456 0.899 1.16 0.815 0.148

Z22762 -0.869 -0.077 1.045 0.736 1.212 1.485 0.813 0.983 0.378 0.556 0.534 0.034

Mean -0.125 0.132 0.877 1.164 1.34 0.93 0.682 0.953 0.407 0.275 0.14 -0.025
