# Supplementary material for: Transcriptome Analysis of Zebrafish Embryogenesis Using Microarrays
Source: PLoS Genet. 2005 Aug 26;1(2):e29. doi: 10.1371/journal.pgen.0010029 (PMC1193535; doi:10.1371/journal.pgen.0010029)
Supplement: Dataset S5 — (66 KB DOC) [file pgen.0010029.sd005.doc]

Dataset S5. List of genes with onset of transcript accumulation at the blastula stage and peak

of expression at segmentation stages.

Genbank IDUF egg 3hpf 4.5hpf 6hpf 7.7hpf 9hpf 10.7hpf 12hpf 15hpf 24hpf 30hpf 48hpf

AF160635 -0.537 -0.251 0.316 0.554 0.621 0.788 1.004 0.862 0.694 0.609 0.365 0.266

AI331043 -1.565 -1.236 -0.708 -0.075 -0.076 0.568 0.586 0.307 0.324 0.26 0.335 -0.232

AI544649 -0.454 0.248 0.878 1.482 3.674 4.326 4.566 3.167 3.601 1.261 0.632 -0.616

AI584307 -0.236 -0.373 -0.223 -0.095 0.772 0.641 0.91 0.734 0.419 0.776 0.734 0.566

AI641775 0.52 -0.523 -0.217 0.085 0.244 0.851 0.986 0.91 0.598 0.813 0.477 0.325

AI723219 -1.339 -1.305 0.957 0.726 0.466 0.607 0.838 0.602 0.502 0.778 0.449 0.174

AW171260 0.381 0.077 -0.427 -0.154 0.043 0.557 0.499 0.498 0.136 -0.414 -0.58 -0.524

AW232296 0.447 1.06 1.453 1.484 1.437 1.446 1.522 1.477 0.718 1.064 0.46 -0.095

AW420829 -0.25 -0.035 -0.435 -0.482 1.248 0.651 1.046 0.975 0.34 0.058 0.331 0.207

BE606048 -0.153 -0.514 0.6 0.466 0.701 0.515 0.998 0.58 0.779 0.838 0.155 0.385

BI473383 -0.328 -0.371 0.195 0.444 0.471 0.353 0.681 0.189 0.213 0.266 -0.29 -0.145

BI839727 0.324 -0.324 0.422 0.321 0.342 0.881 0.988 0.524 0.239 0.253 -0.064 -0.138

BI840845 -0.559 -0.773 0.458 0.821 0.14 0.708 0.722 0.705 0.223 0.158 -0.147 -0.093

BI850032 0.378 -0.529 -0.862 -0.542 0.142 0.891 1.139 1.014 0.839 0.64 0.142 0.3

BI887718 -0.791 -0.525 2.215 3.674 4.74 4.448 5.293 4.54 3.835 0.612 0.468 -0.196

AA495427 -0.788 -0.757 -0.145 0.004 0.325 1.278 0.596 1.715 0.429 -0.017 -0.293 0.206

AA658743 -0.777 -0.882 -0.138 0.241 0.702 0.328 0.579 1.535 0.358 0.785 0.581 0.128

AB032726 0.896 0.238 0.501 0.47 0.7 1.306 0.689 1.327 0.915 0.619 0.162 -0.406

AB055662 -1.395 -1.305 -0.421 0.387 0.599 1.262 0.688 1.629 0.793 1.006 0.85 -0.02

AB055666 -1.046 0.105 -0.64 0.31 -0.316 0.81 0.659 1.662 0.732 0.991 0.796 1.208

AF032392 0.235 -0.672 -0.855 0.019 0.458 0.342 0.308 1.282 0.319 0.938 0.257 0.16

AF071255 -0.905 0.093 -0.778 0.244 -0.325 0.26 0.318 1.383 0.516 0.658 0.965 0.502

AF160683 -0.938 -0.639 -0.579 0.327 0.29 0.437 0.116 0.877 0.4 0.205 -0.148 -0.155

AF202054 0.13 -0.536 -0.569 -0.264 -0.092 0.16 0.284 1.028 0.177 0.464 0.083 -0.08

AF252546 -0.053 -0.333 -0.309 -0.183 0.212 0.56 0.714 1.074 0.621 0.233 0.198 -0.191

AF257519 -0.154 -0.169 -0.168 0.507 0.914 0.658 0.413 0.961 0.053 0.316 -0.035 -0.274

AF369382 -0.763 -0.006 -0.131 0.629 0.595 0.736 0.552 1.467 -0.304 0.657 0.708 0.574

AF395113 -0.6 -0.524 -0.397 0.002 0.366 0.461 0.413 0.778 0.283 0.033 0.597 0.003

AF423762 -0.142 -0.795 0.677 0.921 1.334 1.573 1.501 1.662 1.391 1.102 0.797 0.828

AI330707 0.118 -0.108 -0.728 -0.276 0.377 0.156 0.211 1.068 0.26 0.647 0.376 0.078

AI330980 0.04 -0.956 0.079 0.091 0.229 0.475 0.678 1.175 0.362 0.939 0.873 0.564

AI331515 -1.071 -1.318 -1.511 -0.609 -0.23 0.267 0.346 0.899 0.579 0.513 -0.479 -0.35

AI353412 -1.573 -0.006 3.87 3.542 3.997 4.521 4.403 4.471 3.266 1.757 1.5 0.192

AI384140 -0.669 -0.801 -0.083 0.388 0.933 0.919 0.6 1.437 0.506 0.587 0.49 0.15

AI396677 0.165 -0.381 0.503 0.469 1.603 1.706 1.653 2.001 0.752 0.435 0.279 0.081

AI397023 -0.387 -0.111 -0.141 0.058 0.484 0.303 0.265 0.886 0.336 0.445 0.629 0.44

AI437239 -0.021 -1.183 -0.31 -0.336 0.16 0.495 0.137 0.794 0.148 0.536 0.326 0.187

AI477286 -0.158 -0.096 0.154 0.555 0.687 1.258 1.211 1.411 1.169 1.064 0.472 0.312

AI477305 -0.535 -2.694 -0.681 -0.215 0.28 0.859 1.005 1.103 0.507 0.807 0.362 0.08

AI477935 -0.542 -0.004 1.268 2.229 3.722 4.366 4.045 4.581 3.747 1.287 -0.149 -0.687

AI477949 0.08 1.131 1.806 1.987 2.348 1.944 1.782 2.498 0.84 0.783 0.082 -0.156

AI497234 -0.419 -0.043 0.332 0.753 1.343 1.136 1.301 2.252 0.944 0.02 -0.356 0.086

AI497414 0.699 0.869 0.698 0.746 1.026 1.083 0.675 1.145 0.543 0.079 0.345 -0.185

AI545065 -0.663 -0.527 2.102 2.511 2.801 2.428 2.265 2.853 1.872 0.184 0.039 -0.54

AI545142 -0.853 0.334 -0.436 0.341 1.054 0.905 0.97 2.212 0.996 1.545 0.791 -0.38

AI545424 -0.092 -0.308 0.425 0.758 1.135 0.753 0.961 1.091 0.771 0.771 1.032 -0.053

AI545947 0.164 -0.293 -0.11 0.073 0.438 0.215 0.468 0.779 0.231 0.233 0.068 -0.42

AI584354 0.156 -0.621 0.073 0.146 0.107 0.304 0.809 1.042 0.467 0.575 0.557 -0.04

AI584421 0.379 -0.258 -0.486 0.06 0.091 0.279 0.204 0.574 0.31 0.306 -0.139 -0.251

AI584440 -0.704 -0.12 -0.491 0.252 0.611 0.684 0.356 0.99 0.288 0.438 0.377 0.237

AI588190 -0.728 -0.001 0.259 0.339 0.604 1.124 0.593 1.295 0.832 0.802 0.267 -0.169

AI588475 -0.433 0.267 0.245 0.072 0.889 0.409 0.553 1.35 -0.078 -0.604 0.486 0.234

AI601297 -0.613 0.279 -0.266 -0.337 0.239 0.318 0.221 1.004 0.45 0.447 0.833 0.567

AI601467 0.295 0.45 -0.291 0.007 0.289 0.643 0.498 1.266 0.734 0.573 0.454 0.084

AI601541 0.11 0.089 0.064 0.372 0.69 0.569 0.766 1.31 0.282 0.331 0.032 -0.286

AI601765 -0.175 -0.603 -0.322 0.188 0.907 0.45 0.841 1.248 -0.378 0.586 -0.543 -0.525

AI641018 0.337 0.065 -0.137 0.267 0.476 0.91 0.678 1.033 0.409 0.528 0.424 0.129

AI641124 -0.24 -0.89 -0.023 -0.238 0.248 0.41 0.271 0.988 0.134 0.485 0.265 0.01

AI657601 -0.706 -1.033 -1.125 -0.355 0.11 0.124 0.199 1.333 0.738 0.992 0.306 -0.36

AI657922 -0.116 -0.578 -0.066 0.346 0.471 0.231 0.436 0.797 0.129 0.511 0.483 0.49

AI666922 -0.128 -0.953 -0.889 -0.499 -0.16 0.379 0.619 1.418 0.867 0.702 0.608 0.758

AI666989 -0.332 -0.525 -0.538 0.242 0.673 1.069 0.462 1.38 0.822 1.018 1.017 0.511

AI721479 -0.112 -0.631 -0.518 0.092 1.091 0.905 1.256 1.958 1.054 1.017 1.155 0.074

AI722515 -0.114 -0.675 0.045 0.037 0.398 0.461 0.374 1.338 0.517 0.515 -0.171 -0.885

AI723286 0.779 0.534 0.436 0.42 0.723 0.844 0.796 0.943 0.478 0.8 0.099 -0.285

AI793428 -0.228 -0.384 -0.578 -0.249 0.072 0.3 0.371 0.732 0.447 0.628 0.208 0.355

AI793690 -0.058 0.081 -0.27 -0.071 0.038 0.456 0.551 1.453 0.487 0.102 0.722 0.704

AI793745 0.352 -0.605 -0.104 0.502 1.037 0.949 0.922 1.215 0.649 0.322 0.05 -0.325

AI794483 -0.643 -0.774 -0.534 -0.417 -0.028 0.101 0.194 0.935 0.181 0.428 0.182 0.611

AI877955 -0.401 -0.152 1.463 1.831 1.44 1.581 1.491 1.916 0.626 0.595 0.17 0.067

AI878021 -0.333 -0.343 -0.879 -0.108 0.35 0.374 0.223 1.184 0.342 0.948 0.392 0.385

AI878617 -0.017 0.094 -0.005 0.749 0.763 0.925 0.644 1.087 0.151 0.502 0.051 0.004

AI878677 -0.852 -1.265 0.82 1.121 1.023 1.064 1.344 2.301 1.447 1.729 0.933 -0.264

AI883326 -0.163 -1.796 -0.008 -0.222 0.779 0.658 0.464 1.484 0.917 0.872 0.61 0.264

AI884026 0.565 0.023 -0.148 0.177 0.318 0.5 0.446 1.012 0.384 0.222 0.032 -0.619

AI884227 0.196 0.985 1.325 1.286 1.446 1.292 1.278 1.883 1.39 0.926 -0.087 -0.546

AI943082 -0.317 -0.636 -0.741 -0.255 0.879 0.382 0.386 1.002 0.374 0.663 0.355 0.272

AI943154 0.451 -0.596 0.032 0.268 0.563 0.622 0.625 1.268 0.635 0.812 0.564 0.295

AI943227 -0.093 -0.64 -0.638 -0.264 0.303 0.759 0.731 1.59 1.07 0.737 0.384 -0.072

AI957816 -0.729 -1.567 -1.601 -1.015 0.029 0.34 0.542 0.886 0.552 0.466 -0.205 -0.112

AI958191 -0.528 -1.156 -0.045 0.112 0.461 1.236 0.808 1.392 1.153 0.898 0.231 -0.143

AI958505 -0.562 -1.321 -1.543 -0.584 -0.434 -0.16 0.123 0.973 0.444 0.536 -0.071 0.151

AI959106 -0.626 -0.271 -0.568 -0.182 0.529 1.267 0.413 1.391 0.362 0.571 0.146 -0.026

AI964231 -0.279 -0.069 0.737 0.893 1.368 1.505 1.154 1.719 0.749 0.598 0.188 -0.337

AI964258 -0.804 -1.302 -0.598 -0.063 0.155 0.718 0.651 0.896 0.61 0.683 0.138 -0.006

AI964352 -0.118 -0.94 -0.222 0.035 0.062 0.362 0.365 0.761 0.318 0.668 0.401 0.209

AI964971 -0.306 -1.124 -0.387 -0.436 -0.022 0.591 0.447 0.853 0.598 0.703 0.323 -0.139

AI965251 -0.702 -0.455 -0.115 0.356 1.284 1.235 1.701 2.703 0.916 0.719 0.669 -0.582

AI965321 0.176 0.248 -0.321 -0.089 0.751 0.921 0.814 1.139 0.081 0.532 0.399 -0.422

AI974139 -0.093 -1.046 0.212 0.858 0.2 0.406 0.516 0.949 0.342 0.337 0.576 0.043

AI974209 -0.775 -0.388 -0.578 -0.255 0.382 0.071 0.53 1.333 0.019 0.258 0.194 -0.291

AJ309314 -0.588 0.005 0.308 0.974 2.329 2.346 1.78 2.696 1.5 0.096 0.168 -0.141

AW019321 -0.917 -0.62 -0.537 -0.049 0.294 0.836 0.931 1.233 0.546 -0.448 -0.683 -0.729

AW019528 0.035 -0.242 -0.086 -0.026 0.45 0.944 0.965 0.978 0.559 0.383 0.23 0.085

AW019613 -0.08 -0.118 -0.361 0.092 0.006 0.044 0.174 0.594 0.079 0.024 -0.144 -0.242

AW019690 0.431 -0.371 1.104 1.592 1.316 1.465 1.042 1.728 0.764 0.509 -0.001 0.073

AW019720 -0.091 -0.326 -0.009 0.19 0.806 0.838 0.667 1.236 0.766 0.27 0.156 0.006

AW058839 -0.698 -0.825 0.386 0.664 1.145 1.679 1.379 2.02 1.258 0.405 0.173 -0.151

AW059073 0.966 0.634 0.312 0.25 0.454 1.015 0.87 1.402 0.45 0.118 -0.152 -0.638

AW076659 0.034 -0.111 0.243 0.034 0.511 0.546 0.451 0.871 0.656 0.781 0.076 0.414

AW076666 0.053 -1.078 0.156 0.276 0.48 0.37 0.324 1.105 0.231 0.925 0.675 0.261

AW077184 -0.201 -0.029 1.361 1.523 1.613 1.728 1.488 1.846 0.959 0.593 0.192 0.22

AW077961 -2.047 -1.221 -1.763 -0.814 -0.336 0.25 0.242 1.024 0.522 0.434 -0.41 -0.603

AW115729 -0.093 0.141 0.011 0.38 0.662 0.779 0.735 1.331 0.41 0.561 0.726 0.167

AW116281 0.3 0.737 0.558 0.762 0.916 1.116 0.826 1.739 0.978 0.305 0.012 -0.902

AW116722 0.525 0.542 0.007 0.417 0.614 0.377 0.554 1.037 0.342 -0.026 -0.199 -0.31

AW116978 -0.312 -0.771 -0.522 -0.709 -0.091 0.037 0.257 0.961 0.14 0.23 0.677 0.208

AW117050 0.237 -0.371 0.559 0.731 1.188 1.392 1.424 1.456 0.683 0.767 0.072 -0.531

AW117105 0.204 -0.309 0.113 0.221 0.367 0.624 0.522 0.667 0.272 0.16 0.261 0.011

AW117146 -0.692 -0.108 -0.788 0.008 0.531 0.898 0.607 1.055 0.269 0.712 0.194 -0.069

AW128428 -1.238 -2.825 0.046 0.605 1.181 1.452 1.416 1.442 0.173 -1.017 -1.803 -1.647

AW154375 -2.034 -1.766 -0.663 -0.291 0.245 1.085 0.658 1.755 0.705 0.023 -0.35 -0.686

AW154574 0.949 -0.009 -0.064 0.174 0.312 1.366 1.139 1.302 0.353 -0.188 -0.874 -0.985

AW154642 -0.033 0.08 0.401 0.609 1.588 0.957 0.955 1.947 0.855 0.468 0.174 -0.414

AW154707 0.88 -0.493 -0.297 0.474 0.264 0.764 0.688 1.051 0.408 0.708 0.055 0.091

AW154792 0.565 -0.707 -0.073 0.311 0.759 0.616 0.688 1.214 0.469 0.314 0.007 -0.307

AW165362 0.304 -1.184 0.459 1.312 1.702 0.84 0.794 1.936 0.357 0.654 -0.258 -0.57

AW171089 -0.18 -0.155 0.499 0.466 0.679 0.668 0.54 0.699 0.298 0.511 0.168 0.035

AW171554 0.818 0.41 0.443 0.859 0.981 0.872 0.841 1.563 0.585 0.477 -0.311 -0.518

AW203038 -0.059 0.351 -0.203 -0.148 0.441 0.361 0.34 0.717 -0.241 -0.36 -0.183 -0.433

AW231998 0.369 0.955 1.376 1.182 1.357 1.014 0.866 1.464 0.295 0.183 -0.341 -0.268

AW232241 -0.089 0.129 0.047 0.222 0.233 1.098 0.441 1.182 0.638 0.768 0.663 0.428

AW232289 0.433 -0.016 0.114 0.429 0.775 0.566 0.705 0.886 0.349 0.242 0.091 -0.012

AW232317 0.78 0.297 -0.24 0.194 0.682 0.687 0.832 1.388 0.678 0.631 0.29 0.14

AW280006 0.09 0.319 0.487 0.602 1.234 1.633 1.37 1.66 0.867 1.026 0.626 0.284

AW281219 0.341 -0.868 -0.066 0.243 0.54 0.726 0.378 1.288 0.465 0.608 0.631 0.27

AW281931 0.195 1.091 1.751 0.99 1.3 1.683 1.808 2.73 1.257 0.765 0.301 -0.103

AW343911 -0.114 -1.189 -0.055 0.133 0.529 0.566 0.399 1.256 0.611 0.682 0.188 -0.321

AW344187 0.185 -0.179 -0.122 0.272 1.148 1.294 1.173 1.682 0.792 1.046 0.255 0.113

AW421022 -1.389 -1.786 -1.083 -0.061 0.489 1.613 1.407 1.551 1.189 0.905 0.751 0.069

AW466509 0.628 0.29 -0.333 0.205 0.295 0.679 0.251 0.769 0.262 0.518 0.539 -0.117

AW466584 0.658 -0.589 -0.562 -0.169 -0.126 0.56 0.654 0.906 0.203 0.193 -0.023 -0.104

AW777378 0.036 -0.307 -1.174 -0.382 -0.065 0.42 -0.079 0.796 0.208 0.238 0.037 0.365

AW777460 -0.406 -0.023 0.729 1.145 1.211 1.027 1.171 1.208 0.383 0.053 -0.333 -0.038

AW777535 -0.408 -0.081 0.782 0.864 1.022 1.426 1.239 1.671 1.012 1.29 0.861 0.564

AY008836 -0.811 -0.735 -1.049 -0.212 0.15 0.323 0.34 1.645 0.148 0.727 0.314 0.86

BE017831 0.538 0.104 0.372 0.222 0.359 0.432 0.344 0.809 0.055 0.45 0.032 -0.251

BE201733 0.476 0.19 0.174 0.612 1.345 2.044 1.315 2.221 1.258 1.26 0.594 -0.144

BE201769 -0.324 -1.017 -0.783 0.033 1.123 1.183 1.279 1.592 0.877 0.607 0.656 0.942

BE556846 0.061 -1.876 0.188 0.374 0.121 0.574 0.708 1.133 0.569 0.447 0.391 -0.112

BG302868 0.489 -0.089 0.111 0.492 0.677 0.755 0.438 1.127 0.455 0.336 0.142 -0.371

BG305445 -0.541 -0.382 -0.352 0.079 0.808 1.121 0.606 1.303 0.84 0.728 0.477 0.562

BG306038 0.708 -0.509 -0.081 0.377 0.645 1.127 0.853 1.424 0.731 0.974 0.33 0.143

BG306295 0.177 0.583 1.178 1.241 1.784 1.326 1.411 1.961 0.76 0.748 0.282 0.231

BG306779 -0.147 -0.472 0.314 0.221 0.985 1.349 1.342 1.555 0.949 0.759 0.727 0.716

BG738252 -1.155 -0.118 0.654 0.679 0.991 1.174 0.57 1.256 0.719 0.749 0.604 0.506

BG884096 -0.008 -0.169 0.677 0.468 0.454 0.852 0.77 0.894 0.532 0.602 0.348 0.094

BG891864 -0.132 -1.478 -0.474 -0.068 0.363 0.3 0.399 0.859 0.068 0.103 -0.12 0.195

BI427758 0.092 -0.435 1.28 1.538 1.374 1.614 1.376 1.878 0.726 0.639 -0.108 0.028

BI429020 -0.22 -0.742 -0.084 0.032 0.357 0.415 0.154 1.037 0.637 0.835 0.106 -0.164

BI430251 -0.274 -0.238 -0.341 -0.236 0.341 0.221 0.393 1.176 0.249 0.38 0.78 0.069

BI473004 1.165 -0.66 -0.799 -0.479 -0.048 0.898 0.675 1.362 0.411 0.856 0.725 -0.004

BI670852 0.539 -0.255 0.195 0.538 0.909 0.766 0.682 1.346 0.534 1.099 0.76 0.529

BI672058 -0.224 -0.816 0.346 0.071 0.371 0.61 0.381 0.825 0.307 0.334 0.672 0.194

BI672201 -0.34 -0.173 0.293 0.353 0.562 1.222 0.619 1.451 0.698 0.91 0.307 0.035

BI672219 0.247 -0.101 -0.38 0.034 0.498 0.312 0.19 0.671 0.086 -0.432 0.067 -0.231

BI672337 -1.251 -1.507 -1.458 -0.47 0.104 1.041 1.026 1.491 0.245 0.77 -0.285 -0.201

BI673573 -0.009 -0.327 0.074 0.113 0.82 1.942 1.719 1.875 1.404 1.17 0.307 0.115

BI704244 0.209 -0.035 0.77 0.402 0.5 1.056 0.685 1.569 1.168 0.698 0.738 -0.351

BI704249 -1.19 -2.038 1.18 2.333 3.317 3.151 2.768 3.828 2.173 1.371 1.122 0.344

BI704293 -0.728 -0.233 0.216 0.152 0.847 1.076 0.945 1.281 0.536 0.69 0.641 0.112

BI704393 0.291 -0.534 -0.097 0.648 0.603 0.552 0.684 1.307 0.47 0.112 -0.117 -0.67

BI705182 -0.728 -0.185 0.155 0.106 0.832 0.507 0.536 1.471 0.908 0.234 0.708 0.082

BI705721 0.513 0.615 -0.446 0.172 0.805 0.625 0.769 0.905 0.443 0.188 -0.196 -0.405

BI706908 -0.16 -1.223 0.45 1.101 0.917 0.536 0.776 1.256 0.046 0.171 0.276 -0.005

BI709409 0.544 0.386 0.13 0.31 0.498 0.805 0.602 1.003 0.812 0.477 0.515 0.138

BI840456 0.264 0.572 1.064 0.536 1.259 1.61 1.193 1.848 0.682 0.792 0.294 0.161

BI843230 0.316 -0.747 0.132 0.234 0.402 0.536 0.324 0.727 0.139 0.246 -0.305 -0.682

BI850028 -1.04 -1.585 0.542 1.324 2.291 2.079 1.427 2.39 1.744 0.82 0.474 0.098

BI863967 0.131 0.228 0.98 0.564 0.477 0.53 0.502 0.953 0.076 0.077 -0.141 -0.179

BI866342 0.452 0.068 -0.178 0.055 0.278 0.625 0.425 0.881 0.291 0.397 0.016 0.007

BI867717 0.822 0.522 0.389 0.738 1.247 1.36 0.987 1.463 0.307 0.276 0.334 0.046

BI878477 0.022 -0.058 0.234 0.272 0.519 0.699 0.542 0.952 0.331 0.742 0.306 0.314

BI878967 0.797 0.543 0.289 0.531 0.754 0.724 0.668 1.211 0.129 0.124 0.402 0.027

BI878979 0.9 0.04 0.851 0.496 0.951 0.962 0.767 1.062 0.34 0.417 -0.4 -0.658

BI879231 1.259 0.694 -0.082 0.337 0.88 1.045 1.014 1.425 0.535 0.027 -0.03 -0.558

BI882727 1.069 -0.892 -0.251 0.019 0.249 1.391 0.829 1.396 1.114 1.204 0.613 0.004

BI883910 0.607 0.336 -0.259 -0.077 0.421 0.596 0.387 0.75 0.407 0.354 0.28 -0.016

BI884186 0.749 -0.589 -0.155 -0.244 0.011 0.492 0.077 0.901 -0.09 0.164 0.316 -0.217

BI885240 -0.594 -0.621 -0.103 0.693 0.916 0.956 0.923 1.376 0.338 0.622 0.247 -0.002

BI885924 0.742 -0.077 0.319 0.41 0.544 1.176 0.799 1.157 0.458 0.409 0.171 -0.184

BI885968 0.785 -0.222 -0.224 -0.131 -0.018 0.596 0.634 0.926 0.656 0.791 0.447 -0.173

BI886160 0.253 -0.349 0.761 1.303 1.205 2.184 1.784 3.178 1.374 0.71 -0.338 -0.093

BI886200 0.606 0.698 1.303 1.248 1.493 1.745 1.215 1.865 0.96 0.653 0.367 -0.164

BI886549 -0.524 -0.475 -0.366 0.237 0.645 0.918 0.877 1.172 0.287 0.782 0.176 0.13

BI887559 -0.43 -0.904 0.027 0.295 0.577 0.494 0.226 0.983 0.265 0.658 0.243 0.282

BI887574 -0.626 -0.374 0.745 -0.085 0.26 0.537 0.343 0.787 0.467 0.654 -0.021 0.28

BI887620 0.319 -0.033 0.124 0.502 0.854 0.872 1.086 1.372 0.723 0.845 0.638 0.722

BI887812 -0.491 0.115 1.565 1.179 1.569 1.326 1.203 1.885 1.133 0.684 0.174 0.361

BI888166 1.014 -0.557 0.099 0.848 1.435 1.862 1.414 1.931 0.426 0.984 0.608 0.344

BI888928 0.855 -0.448 -0.357 0.299 0.5 0.69 0.685 1.439 0.458 0.467 0.585 0.283

BI889140 0.176 0.384 0.309 1.308 1.748 1.262 1.216 1.84 0.403 0.463 0.435 0.317

BI889166 -0.93 -0.964 1.667 2.222 2.232 2.397 2.12 2.434 1.542 1.662 1.003 0.224

BI889202 -0.289 -0.363 0.182 0.284 0.772 0.344 0.466 1.021 -0.036 0.026 0.164 0.177

BI889917 0.131 0.098 -0.6 -0.28 1.228 1.231 0.794 1.542 -0.07 0.32 0.222 -0.158

BI889922 -2.288 -1.316 1.667 2.018 2.774 2.812 2.82 2.879 2.829 -0.87 -1.154 -1.457

BI890013 0.559 -0.731 0.232 -0.039 0.617 0.482 0.456 0.94 0.489 0.809 0.704 -0.197

BI890050 -0.134 -0.668 -0.111 0.347 0.955 0.453 0.761 1.217 0.542 0.512 0.45 0.276

BI890108 -0.502 -1.355 0.259 0.369 0.902 0.873 0.558 1.819 0.902 1.173 0.543 0.044

BI890196 -0.791 0.19 -0.295 0.365 0.663 1.497 1.113 1.521 1.318 0.584 0.33 0.128

BI890439 -0.514 0.081 1.452 2.519 3.822 3.403 2.873 3.828 1.495 0.191 0.148 -0.304

BI890508 0.304 -0.413 0.111 0.245 0.847 0.552 0.47 0.83 0.356 0.268 0.406 0.471

BI890704 0.302 0.594 0.268 0.378 0.358 0.844 0.498 0.95 0.372 0.328 0.004 -0.126

BI891001 -1.158 -1.384 -0.627 0.24 0.119 0.592 0.412 1.011 0.16 0.531 0.659 0.802

BI891116 0.296 0.932 1.414 1.07 1.516 1.417 1.039 1.569 0.944 0.432 0.218 -0.052

BI891278 -0.916 -2.831 -0.923 -0.106 0.79 1.201 1.326 1.401 0.528 0.875 0.707 0.733

BI891455 0.758 0.232 0.409 0.789 0.978 0.951 1.101 1.28 0.727 1.173 0.598 0.392

BI891674 0.229 -0.449 -0.141 0.33 1.577 1.452 1.086 2.633 1.606 0.478 0.247 0.044

BI891681 -1.3 -1.649 -1.081 -0.119 0.28 -0.09 0.571 0.844 0.594 0.77 0.299 0.542

BI891689 0.903 0.318 0.182 0.491 0.362 1.008 0.757 1.416 0.441 0.385 0.053 -0.408

BI891704 -0.049 -0.406 -0.1 0.906 1.283 1.188 1.284 1.609 1.022 1.259 0.302 0.145

BI891709 -0.956 -0.348 0.556 1.394 1.888 2.097 2.11 2.502 1.283 1.252 0.511 0.421

BI892082 0.211 0.072 0.36 0.99 0.697 0.558 0.798 1.212 0.3 0.407 -0.164 -0.109

BI892172 0.962 0.185 0.573 0.837 1.536 1.242 1.606 2.258 0.978 0.816 0.33 -0.142

BI892384 0.18 0.329 1.129 1.039 1.26 1.013 1.474 1.665 1.04 0.749 0.208 0.06

BI896347 -0.793 -0.996 -0.574 -0.292 -0.132 1.003 0.675 1.373 1.15 0.863 0.847 0.248

BM026308 -0.463 0.508 0.31 1.229 1.628 1.304 0.913 1.87 0.154 0.33 0.135 -0.153

BM034983 0.245 0.51 0.16 0.251 0.304 0.635 0.495 1.312 0.202 0.273 -0.363 -0.348

BM036297 -0.107 -0.63 0.247 0.194 0.572 0.37 0.29 0.74 0.261 0.232 -0.027 0.016

BM036521 0.598 -0.516 -0.007 0.168 0.566 0.322 0.39 1.266 0.366 0.566 0.331 0.037

BM036802 -0.049 0.341 1.187 1.578 1.378 1.27 1.273 1.853 0.782 0.559 -0.021 0.222

BM070584 0.472 0.084 0.808 1.137 1.148 0.909 1.101 1.174 0.728 0.618 0.28 0.301

BM080950 0.821 -0.045 0.31 0.987 1.3 1.168 1.092 1.536 0.418 0.954 0.699 0.347

BM095389 1.332 0.206 0.067 0.586 0.618 1.414 1.229 1.707 0.79 0.644 0.609 0.184

BM095658 0.051 0.468 0.681 1.006 1.262 0.988 1.074 1.444 0.576 1.005 0.601 -0.289

BM102070 -0.146 -0.157 0.071 0.101 0.68 0.341 0.395 0.958 0.272 0.225 0.264 0.127

BM102865 0.15 -0.587 0.36 0.586 1.163 0.423 0.676 1.277 0.374 0.305 -0.055 0.017

BM103927 0.584 0.02 0.592 0.322 0.489 1.07 0.808 1.485 0.46 0.296 -0.061 -0.129

BM156012 0.639 0.484 0.461 0.389 0.779 1.541 0.849 1.777 0.917 0.615 0.465 -0.028

BM182319 -0.338 0.264 -0.049 0.629 1.057 0.616 0.858 1.489 0.309 -0.358 -0.722 -1.107

BM182325 0.303 0.52 -0.04 0.252 0.254 0.508 0.629 0.743 0.374 0.311 0.003 -0.015

BM182327 0.91 0.888 1.003 1.504 1.698 1.609 1.339 1.971 1.017 0.705 0.128 0.256

BM182750 0.234 0.431 0.012 0.411 0.521 0.563 0.43 0.877 0.183 0.157 -0.044 -0.157

BM183903 0.596 -1.367 -0.441 0.295 0.508 0.692 0.773 1.294 0.622 0.942 0.541 0.124

BM184694 0.908 0.46 0.925 1.089 1.531 1.81 1.978 2.494 1.003 1.383 0.796 0.409

BM185168 0.14 -0.359 -0.911 -0.641 -0.545 0.387 0.293 1.006 0.334 0.439 0.159 0.371

BM185237 -0.285 -0.45 0.693 0.618 0.411 0.812 0.782 0.796 0.323 0.165 -0.137 -0.248

BM186050 0.493 0.712 0.711 0.554 0.836 0.758 0.688 0.863 0.456 0.41 -0.112 -0.533

S76877 -0.351 -0.322 0.419 0.56 0.98 0.838 0.691 1.088 0.602 0.22 0.535 0.04

U31079 -0.428 -0.692 -0.58 -0.093 0.363 0.471 0.617 1.884 0.669 0.94 0.956 0.639

U40995 -0.741 -0.14 -0.448 -0.49 1.136 1.203 0.984 2.288 0.767 0.683 0.995 -0.093

U62134 -0.479 -0.42 0.806 0.673 0.789 0.725 0.85 1.002 0.598 0.461 0.05 0.009

X65060 -0.646 -0.467 -0.024 0.006 0.701 0.742 0.508 1.287 0.144 -0.121 -0.148 -0.276

X67648 -1.484 -0.865 -0.497 0.518 1.062 0.712 1.023 1.535 0.88 0.98 0.555 0.628

AI658234 -1.346 -1.949 -1.498 -0.678 -1.042 -0.726 -0.529 0.2 0.974 0.74 0.588 -0.372

AI667396 -3.384 -3.319 -3.531 -2.728 -2.813 -1.5 -1.265 -0.582 0.796 0.417 0.672 0.143

AI942866 -0.505 -1.596 -1.538 -1.226 0.022 0.716 1.01 0.835 1.822 0.661 0.255 -0.874

AI957409 -0.502 -1.417 -0.925 -0.707 -0.655 -0.092 0.264 1.075 0.989 0.915 0.962 0.066

AI957519 -0.695 -0.203 1.35 1.334 1.455 1.772 1.255 1.657 1.828 1.182 0.752 0.673

AW154716 -1.197 -0.184 -1.041 -0.576 -0.364 0.245 0.27 1.144 1.325 1.127 0.896 0.699

AW184237 -2.052 -1.684 0.479 0.079 0.66 0.798 0.595 0.363 1.142 0.284 0.738 0.428

BI707482 -0.38 -1.249 -0.961 -0.459 0.531 0.528 0.432 0.733 0.787 0.623 0.403 0.525

BI842822 -1.016 -0.271 -0.224 0.258 1.266 0.882 0.371 1.271 1.214 0.726 -0.008 -0.18

BI846833 -2.254 -2.063 -0.124 0.504 0.823 1.352 1.334 0.954 1.512 1.114 1.139 0.442

BI886229 0.375 -0.317 -0.203 -0.213 0.042 0.496 0.352 0.69 0.702 0.435 0.591 0.527

BI888344 -1.041 -1.027 0.084 0.256 0.891 0.619 0.313 0.673 1.001 0.263 0.657 0.388

BI889074 0.307 0.591 0.797 0.406 0.934 0.802 0.935 1.107 1.11 0.749 0.181 -0.666

BI892263 -0.754 -0.429 1.008 1.416 1.222 1.511 1.547 1.361 1.575 1.399 0.875 0.259

BM103340 -1.107 -0.902 0.261 0.425 0.277 0.638 0.337 0.481 0.69 0.539 0.115 -0.329

Mean -0.163 -0.403 0.07 0.385 0.751 0.923 0.849 1.356 0.656 0.562 0.28 0.003
