# Supplementary material for: Transcriptome Analysis of Zebrafish Embryogenesis Using Microarrays
Source: PLoS Genet. 2005 Aug 26;1(2):e29. doi: 10.1371/journal.pgen.0010029 (PMC1193535; doi:10.1371/journal.pgen.0010029)
Supplement: Dataset S6 — (44 KB DOC) [file pgen.0010029.sd006.doc]

Dataset S6. List of genes with onset of transcript accumulation at the blastula stage and peak

of expression at pharyngula stages.

Genbank IDUF egg 3hpf 4.5hpf 6hpf 7.7hpf 9hpf 10.7hpf 12hpf 15hpf 24hpf 30hpf 48hpf

AA605677 -3.724 -3.317 -0.595 -0.783 -0.731 1.127 0.383 0.401 0.608 1.526 1.438 1.089

AA606010 1.15 -0.39 -0.253 -0.035 -0.155 0.535 0.69 0.879 0.934 1.298 0.572 0.18

AA606173 -0.869 -3.327 -1.503 -1.121 -0.847 0.487 0.23 0.29 0.372 1.104 0.84 0.393

AA658759 -2.666 -4.094 -2.319 -1.724 -1.067 -0.272 -0.21 0.079 0.109 0.924 0.728 0.332

AB032265 -1.131 -0.903 -1.384 -1.116 -0.593 -0.015 -0.367 0.571 0.364 0.573 0.419 0.51

AF068772 -1.603 -1.025 -0.605 0.007 -0.206 -0.05 -0.123 0.162 0.047 0.486 0.004 -0.163

AF115774 -0.801 -0.447 -0.72 -0.429 0.379 -0.146 0.224 0.524 0.27 0.723 0.705 0.117

AF124095 -1.012 -0.48 -0.755 -0.016 -0.305 -0.028 -0.306 0.271 0.352 0.77 0.74 0.359

AF134850 -6.382 -5.38 -1.241 -0.434 -0.066 0.822 0.451 0.66 0.58 1.266 0.871 0.136

AF134852 -1.041 -4.135 -2.07 -1.404 -1.052 0.269 -0.205 0.105 0.178 1.069 0.826 0.299

AF155578 0.313 -0.514 0.39 0.13 0.558 0.931 0.461 1.235 0.989 1.446 0.664 0.47

AF197880 -4.428 -3.512 -0.999 0.126 0.276 0.993 0.961 0.606 0.849 1.512 0.519 -0.012

AF288217 -1.691 -2.012 -0.517 -0.286 -0.333 0.553 0.171 0.485 0.175 0.563 0.244 -0.125

AF332623 -0.353 0.164 -0.041 0.224 0.169 0.177 0.117 0.456 0.21 0.397 0.19 0.071

AI106421 -0.405 -2.679 -0.973 -0.895 -0.423 0.048 0.4 0.622 0.317 1.361 0.739 0.218

AI353168 -1.17 -3.123 -1.685 -0.944 -0.667 0.354 0.063 0.253 0.299 0.877 0.585 -0.313

AI384355 -1.279 -3.67 -1.778 -1.335 -0.982 0.264 -0.028 0.217 0.234 1.146 0.997 0.597

AI477041 -0.327 -2.059 -0.292 0.216 0.284 0.507 0.696 1.021 0.699 1.109 0.578 -0.317

AI478002 -0.677 -1.323 -1.58 -0.586 -0.107 -0.392 -0.357 1.118 0.836 1.606 0.535 -0.04

AI545450 -0.645 -0.367 -0.76 0.09 0.233 0.523 0.243 0.647 0.533 0.741 0.492 0.019

AI545576 0.576 -0.05 -0.632 0.102 0.507 0.163 0.454 0.953 0.767 0.956 0.692 -0.193

AI558451 -0.775 -2.631 -1.15 -0.979 -0.572 0.505 0.165 0.356 0.32 1.172 0.919 0.344

AI558502 -0.434 -1.186 -0.728 0.067 0.023 0.696 0.934 0.884 0.698 0.948 0.424 0.6

AI558833 -0.853 -3.19 -1.354 -1.176 -0.748 0.48 0.001 0.246 0.239 1.075 0.661 0.196

AI584446 -0.878 -1.711 -0.404 -0.379 0.365 0.351 0.291 0.907 0.283 0.9 0.152 0.439

AI588758 0.45 -0.119 -0.517 -0.112 -0.197 0.256 0.173 0.919 0.538 0.994 0.268 -0.119

AI601848 -0.914 -1.341 -0.284 -0.322 0.17 0.262 0.102 0.542 0.578 0.753 0.338 0.101

AI721534 -0.236 -0.683 -1.504 -1.349 -1.148 -0.39 -0.268 0.692 0.392 0.767 0.299 0.284

AI793600 -0.057 -1.383 -1.53 -0.068 -0.102 0.679 0.144 0.543 0.674 0.759 0.545 0.256

AI878068 -1.035 -2.87 -1.539 -1.093 -0.572 0.381 0.111 0.427 0.27 1.034 0.561 0.176

AI878458 -0.804 -0.282 -0.936 -0.193 -0.797 -0.23 -0.015 0.145 0.269 0.854 -0.033 0.251

AI882728 -0.213 -0.435 -0.348 -0.24 0.387 0.466 0.478 0.625 0.45 0.631 0.081 0.371

AI884082 -0.675 -0.73 -1.096 -0.71 -0.271 1.196 0.913 1.137 0.977 1.132 0.594 0.125

AI884099 -0.216 -1.39 -1.02 -0.618 -0.681 -0.266 -0.312 -0.125 -0.004 0.531 0.062 -0.107

AI957415 -2.72 -2.669 -2.664 -2.652 -0.691 0.493 0.659 0.985 0.942 0.983 0.551 0.254

AI959257 -0.859 -1.429 -1.687 -0.912 -0.976 -0.477 -0.514 -0.062 0.001 0.544 0.187 0.513

AI959750 -0.863 -2.434 -1.026 -0.776 -0.33 0.681 0.351 0.673 0.311 1.163 0.796 0.462

AI964216 -1.265 -2.28 -1.221 -0.904 -0.57 0.389 0.046 0.422 0.469 1.115 0.904 0.395

AI964218 -1.063 -2.966 -1.49 -1.1 -0.802 0.384 0.038 0.24 0.18 0.958 0.635 0.085

AI964239 -1.077 -3.921 -2.01 -1.342 -0.951 0.276 0.098 0.198 0.217 0.835 0.545 -0.094

AI964289 -0.623 -1.55 -0.759 -0.474 -0.499 -0.02 0.035 0.196 0.07 0.655 0.337 0.107

AI964322 -0.514 -1.284 -1.037 -0.286 -0.249 0.4 0.268 0.878 0.348 0.928 0.213 0.253

AJ245491 -5.149 -5.056 -3.874 -3.209 -2.408 -0.59 -0.413 -0.294 0.033 0.759 0.643 0.604

AW019428 0.858 -0.408 -0.085 -0.179 0.143 0.441 0.384 1.001 0.571 1.033 0.444 0.054

AW077063 -0.056 -1.141 -1.24 -0.326 -0.3 0.329 0.276 0.482 0.571 1.29 0.937 0.332

AW077142 -1.016 -2.732 -0.928 -0.065 -0.137 0.746 0.33 0.531 0.387 0.943 0.497 -0.203

AW077286 -0.713 -2.383 -1.037 -0.819 -0.578 0.427 0.207 0.088 0.387 1.103 0.776 0.126

AW078048 -0.932 -1.798 -0.404 -0.089 0.267 0.92 0.835 1.043 0.721 1.55 1.148 1.269

AW115626 0.923 -0.746 -0.58 -0.225 -0.494 0.863 0.555 0.985 0.835 1.029 0.351 -0.409

AW127801 0.314 -0.316 -0.121 0.056 -0.103 0.978 0.378 0.669 0.864 0.894 0.486 -0.162

AW128382 -0.528 -0.464 -1.765 -0.396 -0.415 -0.084 0.178 0.665 0.51 1.028 0.294 0.28

AW128744 -1.007 -2.859 -1.569 -1.22 -0.937 0.313 0.035 0.282 0.202 1.024 0.813 0.298

AW154782 -1.778 -0.775 -1.734 -0.781 -0.494 -0.023 0.258 0.696 0.657 1.141 0.584 0.313

AW175139 -0.872 -0.355 -0.974 -0.231 -0.24 -0.104 -0.144 0.478 0.343 0.677 0.19 0.196

AW202603 -1.511 -1.898 -0.93 -0.636 -0.409 0.233 -0.064 0.081 0.152 0.726 0.583 0.455

AW203148 -0.973 -2.026 -1.075 -0.416 -0.234 0.492 0.46 0.634 0.523 1.189 0.95 0.528

AW232975 -0.373 -2.874 -0.874 -0.479 -0.155 0.745 0.639 0.615 0.359 0.734 0.424 -0.085

AW281444 -0.849 -0.423 -0.301 -0.229 -0.044 0.496 0.035 0.745 0.381 0.76 0.22 -0.334

AW305460 0.065 -0.668 -0.675 -0.483 -0.637 0.302 0.467 0.294 0.448 0.788 0.699 0.468

AW343989 -1.334 -1.42 -0.581 -0.238 0.284 0.058 0.042 0.834 1.027 1.354 0.637 0.215

AW455045 -2.069 -1.071 -2.143 0.05 -1.339 -0.448 -0.834 0.347 0.521 1.114 0.45 0.282

AW595789 -0.21 -1.286 -0.297 -0.382 -0.204 0.914 0.48 0.762 0.761 1.357 0.84 -0.115

AY007304 0.939 0.191 0.161 0.524 0.421 0.979 0.664 1.472 1 1.444 0.823 -0.052

BE016522 -0.557 -1.183 -1.225 -0.75 -0.324 0.292 0.181 0.683 0.274 0.702 0.552 -0.021

BE017895 -1.018 -2.981 -1.196 -1.049 -0.586 0.427 0.113 0.434 0.204 1.092 0.592 0.25

BF717296 -0.574 -2.459 -1.426 -1.099 -0.962 0.169 -0.069 0.034 0.165 0.868 0.488 -0.02

BF718229 -1.24 -2.836 -1.569 -1.154 -0.781 0.263 0.076 0.29 0.402 1.01 0.687 0.174

BG302711 0.091 -0.646 -1.555 -0.311 -1.013 0.072 -0.163 0.217 0.36 1.07 0.67 0.596

BG303497 0.129 -0.702 -0.418 0.001 0.112 -0.077 0.426 0.797 0.452 0.905 0.359 -0.293

BG303560 0.085 -2.986 -0.314 -0.492 0.083 0.825 0.377 0.9 0.29 0.912 0.545 0.263

BG305988 -0.94 -3.359 -1.396 -1.333 -0.816 0.51 0.114 0.176 0.032 0.85 0.589 0.252

BG306459 -0.931 -2.668 -0.832 -0.844 -0.338 0.485 0.303 0.473 0.265 0.598 0.283 -0.037

BG728552 -1.205 -0.451 -1.385 -0.15 -0.187 -0.316 -0.047 0.299 1.104 1.242 0.601 0.369

BI472743 -0.839 -3.492 -1.865 -1.411 -1.021 0.326 0.016 0.27 0.204 0.885 0.611 -0.089

BI534059 -0.754 -1.263 -0.584 -0.022 0.18 0.589 0.284 0.751 0.354 0.814 0.498 0.194

BI842900 -0.559 -3.581 -1.423 -1.184 -0.556 0.15 0.087 0.471 0.181 1.112 0.699 0.002

BI842921 -0.811 -2.677 -1.475 -1.214 -0.899 0.445 0.084 0.342 0.212 1.18 0.723 0.355

BI864190 -1.084 -0.7 -1.045 -0.167 -0.089 1.079 0.966 1.528 1.245 1.569 1.186 -0.003

BI882972 -1.1 -2.183 -1.072 -0.797 -0.575 0.589 0.221 0.361 0.406 1.287 1.086 0.645

BI883698 -0.382 -0.403 0.133 0.325 0.695 0.815 0.401 0.892 0.622 0.853 -0.11 0.124

BI886029 -1.442 -1.297 -0.919 -0.611 -0.257 0.461 0.146 0.532 0.394 1.176 0.762 0.686

BI886069 -0.589 -0.668 -1.333 -0.536 -0.784 -0.517 0.308 -0.237 0.129 0.271 0.069 -0.366

BI886167 0.108 -2.879 -1.573 -1.301 -1.091 0.126 -0.17 0.157 0.12 0.956 0.595 0.143

BI886825 -0.096 -1.845 -0.856 -0.698 -0.475 0.628 0.142 0.326 0.307 1.037 0.726 0.2

BI887231 -0.545 -2.001 -0.8 -0.67 -0.408 0.642 0.297 0.504 0.377 1.288 1.08 0.462

BI887366 -0.872 -2.986 -1.682 -1.209 -0.679 0.346 0.225 0.54 0.379 1.067 0.699 0.098

BI887737 0.197 -0.312 -0.937 -0.397 -0.238 0.681 0.148 0.548 0.421 0.765 0.179 0.198

BI887990 -0.482 -0.195 0.108 0.092 -0.027 0.436 0.393 0.522 0.342 0.505 0.41 0.139

BI888186 0.454 -0.156 0.032 0.179 0.473 0.856 0.506 1.096 0.781 1.052 0.528 0.468

BI888812 -0.992 -2.583 -1.804 -1.041 -0.646 0.337 0.228 0.293 0.263 1.001 0.661 0.341

BI889445 -0.734 -2.851 -1.29 -0.81 -0.641 0.539 0.088 0.326 0.15 0.918 0.621 0.236

BI889529 -4.057 -4.534 -2.887 -2.368 -2.081 -0.716 -1.026 -0.761 -0.06 0.867 0.685 0.452

BI890023 -0.393 -0.456 -0.051 -0.108 0.025 0.613 0.505 0.776 0.54 0.901 0.515 -0.18

BI890113 -1.149 -2.935 -1.558 -1.169 -0.93 0.223 0.063 0.176 0.192 0.871 0.486 -0.044

BI890218 -0.725 -2.578 -1.236 -1.145 -0.79 0.059 0.127 0.301 0.015 0.992 0.613 0.291

BI890861 -1.181 -1.99 -0.937 -1.081 -0.49 0.335 0.101 0.423 0.198 1.064 0.826 0.374

BI890906 -0.854 -2.499 -0.93 -0.976 -0.607 -0.024 0.213 0.32 0.139 1.214 0.734 0.286

BI891040 -0.575 -2.54 -1.146 -0.869 -0.684 -0.021 -0.001 0.187 0.044 1.09 0.722 0.339

BI891138 -0.667 -3.238 -1.973 -0.918 -0.846 0.381 0.072 0.294 0.392 1.085 0.723 0.102

BI891434 -0.854 -1.878 -0.893 -0.836 -0.581 0.393 0.187 0.493 0.308 1.204 0.915 0.388

BI892430 -0.446 -2.368 -0.921 -0.77 -0.351 0.508 0.28 0.356 0.181 0.988 0.587 0.065

BI896246 -1.362 -2.14 -2.257 -1.651 -1.44 -0.442 0.131 0.4 0.193 0.946 0.858 0.117

BI896418 -1.978 -1.482 -2.546 -1.598 -1.638 0.279 0.624 1.209 1.117 1.881 0.983 1.089

BI896504 -0.896 -2.379 -1.221 -0.962 -0.551 0.671 0.328 0.516 0.381 1.259 0.912 0.405

BI981429 -0.165 -1.342 -1.079 0.012 -0.126 0.05 0.472 0.7 0.524 0.901 0.585 0.451

BI982030 0.113 0.211 -0.6 -0.241 0.034 0.877 0.399 0.846 0.739 1.2 0.643 0.558

BM024216 -0.087 -0.775 -0.118 0.3 0.266 0.288 0.618 0.901 0.804 0.946 0.452 0.033

BM104738 -0.511 -2.579 -1.188 -1.129 -0.491 0.62 0.2 0.667 0.375 1.291 0.891 0.217

BM153976 -0.481 -3.354 -1.545 -0.985 -0.782 0.432 0.108 0.438 0.26 1.035 0.651 0.172

BM159128 -0.54 -2.462 -0.958 -1.048 -0.505 0.555 0.163 0.451 0.153 1.202 0.83 0.313

BM182911 -2.587 -1.889 -2.749 -1.759 -1.38 -0.522 -0.083 0.379 0.235 1.343 0.634 0.19

BM183980 -0.762 -1.606 -0.741 -0.245 -0.617 0.327 0.201 0.274 0.219 0.927 0.759 0.144

U93458 -1.236 -1.746 -1.256 -1.114 -0.746 -0.326 -0.024 0.816 1.083 1.308 0.914 0.596

U93459 -1.054 -1.831 -1.001 -0.746 -0.356 -0.204 0.327 0.336 0.917 1.073 0.784 0.513

X70300 -0.97 -0.821 -0.583 -0.154 -0.295 -0.247 0.227 1.08 0.979 1.143 0.954 0.102

AF071246 -0.837 -0.615 -1.049 -0.62 -0.727 -0.323 -0.558 0.051 0.525 0.432 0.788 0.489

AF295377 -1.073 -3.867 -1.78 -2.067 -1.968 -0.506 -0.701 -0.981 -0.004 0.731 0.807 0.134

AI667290 -1.257 0.384 -0.379 0.072 0.206 0.906 0.276 0.588 0.71 1.038 1.216 0.817

AI667513 -0.629 -1.308 -1.288 -0.48 -0.708 -0.255 -0.211 0.224 -0.08 0.537 0.757 0.367

AI722349 -0.764 -0.031 -0.62 -0.738 -0.003 -0.013 0.06 0.554 0.57 0.867 1.085 0.237

AW018967 -0.576 -1.951 -0.202 -0.545 -0.219 0.28 0.529 0.381 0.485 0.29 0.612 0.248

AW170954 -0.644 -0.246 -0.246 0.059 0.283 0.68 0.297 0.515 0.468 0.51 0.678 0.487

AY007434 -3.23 -1.089 0.313 0.389 0.479 1.025 0.255 0.584 0.6 0.98 0.986 0.608

BG799326 -1.094 -0.934 -0.305 0.084 -0.084 0.686 0.655 0.419 0.634 0.918 0.918 0.623

BG985441 0.1 -1.247 -0.382 -0.574 -0.825 0.298 0.332 0.345 0.725 0.82 0.969 0.606

BI847093 -1.038 -1.035 -1.448 -0.391 -0.717 -0.215 -0.219 0.086 -0.162 0.584 0.681 0.28

BI880002 -1.299 -1.334 -1.435 -1.556 -1.263 -0.527 -0.236 0.019 0.093 0.483 0.676 0.476

BI882791 -2.34 -3.06 -0.442 -0.742 -0.084 -0.461 -0.358 -0.776 0.336 -0.218 0.432 0.079

BI886847 -0.112 -0.872 -0.194 0.081 0.248 0.046 0.333 0.473 0.368 0.339 0.526 0.324

BM157381 -0.33 -1.806 -0.878 -0.646 -0.581 0.332 0.145 0.288 0.073 0.668 0.68 0.167

AB017118 -0.545 -1.512 -0.861 -0.704 -0.642 0.443 0.135 0.316 0.294 0.566 0.429 0.616

AF389401 -1.942 -0.713 -1.278 -0.59 -0.489 -0.051 -0.602 0.246 0.154 0.519 0.208 0.69

AI496943 -1.23 -0.709 -0.039 0.199 0.472 1.339 1.013 1.592 1.061 1.124 1.151 1.58

AI588357 -1.767 -1.984 -1.2 -0.954 -0.742 0.548 -0.315 0.22 -0.266 0.611 0.085 0.65

AI667546 -2.11 -0.978 -1.102 -0.883 -0.985 -0.513 -0.573 -0.122 0.356 0.803 0.768 0.873

AI722645 -0.351 -1.571 -0.358 -0.759 -0.684 -0.154 -0.295 -0.463 -0.036 0.063 0.524 0.778

AJ245493 -1.13 -1.228 -0.674 -0.241 -0.605 -0.424 -0.373 -0.311 -0.157 -0.119 0.355 0.446

BG306138 -0.777 -0.608 -0.966 -0.466 0.111 0.454 0.42 0.834 0.532 0.871 0.952 1.011

BI865754 -0.415 -1.431 0.346 0.38 0.672 0.692 0.566 0.398 0.439 0.871 0.826 1.202

BI892074 -1.096 -1.388 -1.078 -0.567 -0.569 -0.198 -0.292 -0.064 0.108 0.743 0.574 0.799

Mean -0.905 -1.731 -1.023 -0.629 -0.426 0.291 0.161 0.466 0.41 0.935 0.621 0.282
