# Supplementary material for: Transcriptome Analysis of Zebrafish Embryogenesis Using Microarrays
Source: PLoS Genet. 2005 Aug 26;1(2):e29. doi: 10.1371/journal.pgen.0010029 (PMC1193535; doi:10.1371/journal.pgen.0010029)
Supplement: Dataset S7 — (27 KB DOC) [file pgen.0010029.sd007.doc]

Dataset S7. List of genes with onset of transcript accumulation at gastrula and peak of expression at gastrula and subsequent stages.

Genbank IDUF egg 3hpf 4.5hpf 6hpf 7.7hpf 9hpf 10.7hpf 12hpf 15hpf 24hpf 30hpf 48hpf

AI877743 -0.093 -0.196 -0.001 -0.246 0.906 0.173 0.273 0.575 0.23 0.309 0.471 0.103

AI942839 -0.522 0.018 0.094 -0.188 0.934 0.067 0.11 0.738 0.456 0.153 0.543 -0.027

AI942987 0.299 -0.455 0.096 -0.044 1.566 1.277 0.66 1.213 0.678 0.352 0.823 0.414

BG883236 -0.138 -0.138 0.509 0.198 1.61 0.805 0.639 1.027 0.563 0.32 0.273 0.516

BI888165 0.092 -0.167 0.22 -0.05 0.901 0.478 0.216 0.6 0.18 0.207 0.103 0.04

BM095301 -0.154 0.759 0.557 0.237 1.036 0.136 0.004 0.493 0.182 0.344 0.137 -0.012

AA658756 0.667 -0.161 0.094 -0.023 0.183 0.755 0.449 0.226 0.372 0.317 0.132 0.105

AB030897 0.787 0.594 0.344 0.056 0.553 0.926 0.276 0.644 0.045 -0.187 -0.358 -0.378

AI641717 0.464 0.648 1.092 0.632 0.841 1.251 0.993 0.793 0.319 0.6 0.119 0.056

AI793485 0.155 -0.238 -0.144 -0.184 0.233 0.506 0.2 0.494 -0.076 0.199 0.01 0.096

AI943216 0.146 -2.595 -1.038 -1.915 -0.375 1.056 -0.103 0.646 -0.881 0.596 -1.298 -0.064

AI957869 0.875 0.925 1.238 0.883 0.665 1.637 0.751 1.474 0.948 0.696 0.059 -0.429

AW115793 0.134 0.517 0.827 0.234 0.305 0.887 0.3 0.422 0.213 0.02 -0.22 -0.379

AW115873 1.082 1.112 1.131 1 0.896 1.318 1.024 0.44 0.244 0.394 0.136 0.08

AW116327 0.577 -0.348 -0.368 -0.407 -0.079 0.832 0.432 0.605 0.189 0.403 0.328 -0.458

BE201596 -0.281 0.592 0.524 0.277 0.73 1.772 1.511 1.459 0.765 0.759 0.341 0.42

BE558184 0.257 -0.282 0.299 -0.122 0.33 0.72 0.446 0.675 0.458 0.463 0.055 -0.018

BG303575 0.919 -0.894 -0.521 -0.212 0.017 1.337 0.968 0.801 0.187 0.225 -0.309 -0.509

BG303721 0.628 0.26 0.85 0.344 0.959 1.681 0.808 0.823 0.573 0.586 0.183 -0.196

BI878904 0.698 0.553 0.861 0.661 0.627 1.082 0.368 0.459 0.357 0.222 0.231 0.306

BI883242 0.488 0.994 0.915 0.85 0.673 1.098 0.285 0.758 0.185 0.22 0.175 0.342

BI887350 -0.22 0.716 0.636 0.243 0.771 0.839 0.511 0.631 0.119 0.082 -0.056 -0.379

BI887522 0.688 -0.261 0.566 -1.41 0.226 1.058 0.419 0.797 -0.78 -0.078 -0.32 -0.85

BI888732 1.092 0.083 0.784 0.188 0.832 1.41 0.606 0.289 0.584 0.479 0.388 0.356

BI891108 0.618 0.51 1.673 -0.115 0.771 1.507 0.535 0.738 0.229 0.12 -0.073 -0.144

BI891158 -1.998 -2.154 -0.18 -0.886 0.478 3.195 1.919 1.072 1.235 -1.221 -0.852 -0.985

BI891355 -0.346 -0.222 -0.061 0.035 0.123 0.947 0.515 0.775 0.616 0.633 0.151 0.421

BI892229 0.4 0.507 0.376 0.239 0.312 0.934 0.358 0.741 0.351 0.312 -0.172 -0.462

BM103957 0.216 0.664 0.663 0.461 0.316 0.853 0.241 0.593 0 0.08 -0.25 -0.096

BM186051 0.612 1.491 1.718 1.152 1.618 1.923 1.76 1.734 1.531 1.431 0.688 0.517

AW116409 -0.907 -0.61 -0.04 -0.153 0.134 0.655 0.251 0.482 0.36 0.512 0.101 -0.014

AW171308 0.045 -0.321 0.018 -0.013 0.474 0.715 0.408 0.345 0.274 0.258 0.158 -0.086

AW171471 -0.482 -1.335 -0.506 -0.657 -0.258 0.694 0.218 0.416 -0.065 0.03 -0.32 -0.548

BI877718 0.434 -1.025 -0.108 -0.218 0.383 1.094 0.271 0.619 0.491 0.435 0.581 0.49

AI957604 -0.095 -0.072 0.101 0.142 0.85 1.088 0.627 0.896 0.596 0.455 0.671 0.669

AI444338 0.784 0.757 0.782 0.902 0.445 1.413 0.53 1.087 0.92 1.205 0.696 0.236

AI584569 0.67 0.386 0.318 0.372 0.148 0.933 0.234 0.756 0.465 0.609 0.349 -0.106

AW115799 0.94 0.937 0.824 0.933 0.551 1.09 1.025 0.933 0.419 0.363 0.018 0.005

AW171268 0.31 0.526 1.07 0.747 0.454 1.221 0.524 0.88 0.478 0.744 0.351 0.1

AW777906 0.28 -0.091 -0.353 -0.119 -0.147 0.704 -0.072 0.399 0.319 0.425 0.375 0.065

BE605721 1.023 1 0.816 0.814 0.596 1.134 0.562 0.986 0.575 0.396 0.148 0.009

BI840953 -0.082 -0.409 0.192 0.251 0.13 0.851 0.409 0.148 0.179 0.174 0.301 0.65

BI888008 0.323 0.553 0.041 0.039 -0.011 0.901 0.124 0.589 0.138 0.512 0.238 0.684

BI890375 1.333 1.22 1.52 1.497 0.974 1.578 1.288 0.649 0.621 0.55 0.182 0.037

BI891591 0.216 0.04 0.051 0.33 0.239 1.002 0.41 0.968 0.413 0.35 0.725 0.198

BI867171 -0.005 -0.894 -0.899 -0.407 -0.091 0.917 0.689 0.719 0.394 0.643 0.307 0.051

Mean 0.281 0.076 0.382 0.138 0.518 1.053 0.543 0.731 0.362 0.363 0.137 0.018
