# Supplementary material for: Transcriptome Analysis of Zebrafish Embryogenesis Using Microarrays
Source: PLoS Genet. 2005 Aug 26;1(2):e29. doi: 10.1371/journal.pgen.0010029 (PMC1193535; doi:10.1371/journal.pgen.0010029)
Supplement: Dataset S8 — (51 KB DOC) [file pgen.0010029.sd008.doc]

Dataset S8. List of genes with onset of transcript accumulation at gastrula and peak of expression at segmentation stages.

Genbank IDUF egg 3hpf 4.5hpf 6hpf 7.7hpf 9hpf 10.7hpf 12hpf 15hpf 24hpf 30hpf 48hpf

BM102635 0.275 -0.176 0.534 0.587 0.553 0.442 0.88 0.576 0.635 0.552 0.47 0.118

AA497147 -0.129 0.013 0.253 0.51 0.286 0.097 0.106 0.586 0.115 0.175 0.448 0.024

AA497159 -0.012 0.621 0.133 0.342 0.563 0.228 0.192 1.263 0.121 0.358 0.413 -0.124

AA658750 0.041 0.208 0.33 0.477 -0.055 0.158 0.495 0.662 0.061 0.534 0.217 0.036

AF030281 -0.668 -0.228 -0.204 -0.567 -0.091 1.184 0.982 2.012 0.803 0.797 0.888 0.781

AF030283 -0.49 -0.014 0.074 0.132 0.328 0.359 0.424 1.649 0.341 0.292 1.011 0.076

AF030560 -0.237 0.088 1.435 1.235 1.537 0.666 1.262 2.145 0.688 0.231 0.204 -0.04

AF039411 -0.705 -0.496 -0.617 -0.148 0.25 -0.008 0.596 1.573 0.25 0.922 0.662 0.158

AF042191 -0.184 -0.353 0.534 1.385 1.698 0.571 0.871 2.281 0.729 -0.179 -0.105 -0.226

AF061252 -0.637 0.223 -0.483 -0.334 -0.429 0.25 0.412 1.095 0.387 0.447 0.773 0.787

AF067532 -0.332 -0.29 -0.013 -0.33 -0.07 -0.09 0.358 1.309 0.122 0.128 0.457 0.812

AF071248 -0.341 0.06 -0.074 0.346 0.185 -0.23 0.293 1.46 0.299 0.384 0.365 0.006

AF143493 -0.013 -0.285 0.104 -0.093 0.029 0.255 0.447 1.058 0.43 0.858 0.295 0.039

AF177869 -0.261 0.375 0.536 0.329 0.056 0.543 0.376 0.783 0.577 0.051 0.253 -0.04

AF184245 -0.307 -0.157 -0.004 0.143 0.742 0.353 0.475 1.435 0.402 0.764 0.143 0.369

AF210645 0.457 0.801 0.59 0.761 1.076 0.24 0.548 1.339 0.29 0.429 0.346 -0.131

AF222995 -0.711 -0.268 0.179 -0.978 -0.193 0.351 0.619 1.84 0.337 0.551 0.739 0.861

AF254955 -0.186 0.453 0.48 0.347 1.183 -0.05 0.637 1.061 0.239 -0.682 -0.514 -0.608

AF255044 -0.371 0.262 0.783 1.146 1.548 -0.188 0.28 1.75 0.25 -0.007 0.072 -0.027

AF270789 -0.768 0.025 0.009 0.201 0.555 0.715 0.369 2.49 0.706 -0.086 0.024 -0.31

AF354750 -0.023 -0.284 0.184 0.206 0.451 0.198 0.372 0.968 0.506 0.8 0.341 0.12

AF371368 -0.696 -0.669 0.619 0.478 0.593 0.421 0.748 1.447 0.733 0.497 0.91 0.167

AI331953 0.488 0.324 -0.25 -0.25 0.06 0.346 0.303 1.086 0.233 0.88 0.162 0.246

AI415997 0.073 -0.259 -0.303 0.179 0.277 0.174 0.069 0.648 0.116 0.228 0.081 0.238

AI416128 -0.248 -0.285 0.115 0.198 0.524 0.178 0.413 0.819 0.445 0.495 0.036 -0.106

AI437364 0.286 0.159 0.361 0.833 0.938 0.625 0.576 1.125 0.41 0.32 0.606 0.1

AI437466 -0.387 -0.621 -0.058 -0.887 0.112 0.216 0.328 1.015 0.692 0.34 0.449 0.426

AI444340 0.335 0.841 -0.512 -0.384 0.819 1.035 0.68 1.139 0.347 -0.348 -0.06 -0.146

AI496761 -0.621 -0.01 -0.239 -0.082 0.45 0.052 0.022 0.919 0.08 0.036 0.055 -0.229

AI522349 -0.728 -0.428 -0.969 -1.548 -0.881 -0.25 -0.274 0.85 0.518 0.233 -0.098 -0.006

AI545040 -0.029 0.338 -0.164 0.209 0.508 -0.019 0.34 0.66 -0.159 0.143 0.153 0.511

AI545274 0.249 0.629 0.637 0.432 0.592 0.058 0.579 0.752 0.014 0.21 0.074 0.214

AI558431 -0.044 -0.079 0.01 0.708 0.633 0.555 0.363 1.151 0.241 0.472 0.246 -0.199

AI558845 -0.504 0.514 0.444 0.392 0.199 -0.248 0.242 0.741 -0.195 0.168 -0.217 -0.234

AI584556 -0.399 0.289 0.283 0.759 -0.031 -0.01 0.17 0.987 0.238 0.344 0.3 0.338

AI584986 -0.36 0.029 -0.226 0.105 1.225 -0.155 0.253 1.454 0.486 0.322 0.519 0.264

AI588156 -0.327 -0.326 -0.248 0.008 -0.062 -0.252 0.116 1.492 0.267 0.761 0.222 -0.304

AI588225 -0.567 0.041 0.126 -0.254 -0.134 -0.255 0.399 0.78 0.111 0.432 0.646 0.074

AI588304 -1 0.029 0.025 -0.225 -0.148 0.099 0.169 1.064 -0.005 0.687 0.515 -0.214

AI588476 0.272 0.142 0.253 0.585 0.849 0.213 0.525 0.972 -0.053 0.44 0.232 0.263

AI588696 0.306 -0.119 0.624 0.771 0.964 0.264 0.599 1.087 0.228 0.103 0.045 -0.072

AI601692 0.341 0.179 0.308 0.105 -0.163 0.039 0.522 0.564 0.155 0.241 0.217 0.294

AI601696 0.227 0.631 0.089 -0.002 0.318 -0.127 -0.037 0.706 0.08 -0.462 -0.14 -0.307

AI626587 0.697 0.594 0.294 0.758 0.27 0.517 0.607 1 0.308 0.552 0.458 0.021

AI629101 0.284 -0.129 0.553 1.176 1.561 0.353 0.855 1.596 0.29 0.095 0.299 -0.119

AI641051 0.208 -0.129 0.006 0.333 0.011 0.176 0.389 0.722 0.391 0.411 0.488 0.355

AI641146 0.398 -0.079 0.539 0.171 -0.065 0.086 0.092 0.738 0.16 0.295 0.112 0.075

AI641523 -0.172 0.17 -0.164 0.213 1.067 -0.067 0.444 1.602 0.26 0.11 0.033 -0.017

AI657832 0.158 0.189 0.207 0.398 0.443 0.094 0.144 0.691 0.185 -0.221 -0.206 -0.444

AI666923 0.55 0.252 -0.219 0.375 0.395 0.063 0.429 0.674 0.13 0.324 -0.05 -0.087

AI666975 -0.728 -0.309 0.323 1.066 1.581 0.041 0.272 1.619 1.134 0.562 0.345 -0.237

AI667324 0.467 -0.606 0.223 0.693 0.376 0.021 0.458 0.789 0.113 0.431 0.04 -0.264

AI721428 0.46 -0.218 -0.226 0.381 0.788 0.166 0.595 1.062 0.383 0.305 -0.027 -0.413

AI721573 -0.708 0 0.34 0.464 0.534 -0.276 0.066 0.809 0.279 0.438 0.588 -0.01

AI721587 -0.033 -0.06 0.542 1.116 1.282 0.449 0.566 1.408 0.635 0.466 0.139 0.169

AI721611 0.311 0.496 0.9 1.112 1.47 0.691 0.54 1.651 0.301 0.529 0.026 -0.248

AI721616 0.691 0.11 0.198 -0.016 -0.117 0.319 0.501 0.952 0.286 0.653 0.175 -0.063

AI722402 0.497 0.816 0.284 0.611 0.811 0.596 0.465 1.401 0.527 0.218 -0.107 -0.516

AI722496 -0.011 0.319 0.407 0.644 0.704 0.181 0.406 0.834 -0.024 0.324 -0.195 -0.03

AI722538 -0.034 0.225 0.152 0.562 0.77 0.131 0.309 1.052 0.611 0.717 0.239 -0.322

AI722829 -0.224 -0.395 -0.176 0.306 0.288 0.022 0.43 1.018 0.1 0.542 0.578 0.324

AI793350 -0.234 -0.906 -0.018 -0.168 0.372 -0.106 0.28 1.278 0.1 0.523 0.352 0.264

AI793372 0.432 0.756 0.352 0.117 0.601 -0.04 0.484 1.118 0.3 0.272 0.27 0.282

AI793487 -0.114 -0.2 -0.067 -0.477 0.332 -0.035 0.451 1.478 0.196 0.884 0.973 0.308

AI793555 0.5 0.112 -0.263 -0.146 0.56 1.143 1.034 2.216 1.185 0.7 0.21 0.005

AI793853 -0.231 0.266 0.239 0.595 1.231 0.052 0.301 1.25 0.584 0.53 0.156 0.758

AI793880 0.294 0.613 0.98 0.732 1.227 0.492 0.541 1.367 0.465 0.118 -0.092 -0.511

AI793927 -0.529 0.067 -0.017 0.454 0.945 0.033 0.364 1.141 0.392 0.461 0.037 -0.36

AI793969 -0.451 -0.395 0.004 0.268 0.757 0.045 0.596 0.738 0.192 0.422 0.551 0.25

AI793974 -0.055 -0.485 0.167 -0.184 0.429 0.185 0.228 1.131 0.153 0.149 0.022 0.038

AI877538 0.349 0.379 0.269 1.25 1.241 -0.143 0.647 1.385 0.304 0.303 0.281 -0.362

AI877609 -0.145 0.023 0.261 0.489 0.494 0.029 0.22 0.615 0.173 0.307 -0.154 0.025

AI877678 0.233 0.399 0.186 0.598 0.928 -0.074 0.416 1.391 0.687 0.393 0.459 -0.097

AI878386 -0.662 0.18 0.463 0.782 1.305 0.18 0.431 1.531 0.258 0.763 0.271 0.937

AI878627 0.013 0.331 0.228 0.187 0.59 0.194 0.116 1.024 0.251 0.168 -0.156 -0.45

AI878758 -0.266 0.514 0.209 0.341 0.214 0.219 0.203 0.953 0.368 -0.117 -0.32 -0.079

AI878787 0.332 0.429 0.124 0.468 0.447 0.014 0.114 0.669 -0.163 0.005 0.107 -0.207

AI878796 -0.451 0.013 -0.399 -0.194 0.083 -0.049 0.017 0.745 0.162 0.188 0.034 -0.057

AI882781 0.351 0.068 0.312 0.157 0.244 0.141 0.224 0.528 0.112 0.237 0.376 0.064

AI882884 -0.177 -0.613 -0.234 -0.156 0.273 0.05 0.281 1.255 0.692 0.836 0.55 -0.114

AI883716 0.416 0.213 0.588 0.6 0.986 0.698 0.643 1.108 0.218 0.344 0.192 0.114

AI883967 0.084 0.349 0.644 0.591 1.085 0.535 0.326 1.413 0.054 0.163 -0.2 -0.493

AI884148 -0.074 -0.159 0.254 0.351 0.335 0.363 0.253 0.715 0.446 -0.091 0.54 0.226

AI931096 0.099 0.244 0.837 0.742 1.172 0.295 0.481 1.511 0.302 0.834 0.223 0.106

AI942585 -0.843 -0.683 -0.746 -0.875 -0.652 -0.014 0.347 1.265 0.563 0.823 0.447 0.11

AI942627 -0.07 0.135 -0.116 0.096 0.152 -0.001 0.248 0.638 0.116 0.154 0.271 0.1

AI957527 0.53 0.673 0.477 0.273 0.556 0.64 0.805 0.794 0.2 0.059 0.153 0.107

AI957575 -0.666 -2.018 -1.048 -1.215 -0.306 0.448 0.679 1.135 0.512 0.698 0.369 -0.538

AI957786 0.09 0.306 0.756 0.555 0.519 0.159 0.672 1.005 0.211 0.858 0.381 0.241

AI957815 0.153 0.465 0.694 0.433 0.691 0.478 0.448 1.005 0.234 0.203 0.013 0.066

AI957893 -0.668 0.142 0.229 0.22 0.243 -0.022 -0.082 1.21 0.063 -0.186 0.148 0.129

AI957914 -0.311 0.217 -0.501 0.353 0.68 0.081 0.092 0.778 -0.034 -0.317 0.002 0.043

AI959657 0.55 0.686 0.626 0.72 0.729 0.374 0.577 0.821 0.202 0.402 -0.09 -0.25

AI959670 0.186 0.643 0.592 0.517 -0.191 0.204 0.199 1.025 0.221 -0.353 0.053 0.037

AI964108 -0.001 0.685 0.155 0.717 0.533 0.354 0.258 0.732 0.236 -0.167 0.394 -0.194

AI964306 0.949 0.638 0.411 0.347 0.223 0.166 0.72 1.223 0.563 0.415 -0.091 -0.231

AI964375 0.125 0.381 0.552 0.69 0.988 0.228 0.564 1.078 0.452 0.2 -0.005 -0.485

AJ005026 -0.342 0.254 -0.072 0.343 0.378 0.355 0.217 0.708 0.468 0.094 0.385 0.193

AJ006310 -0.664 0.042 -0.077 0.124 0.057 -0.003 0.237 2.107 0.619 0.539 0.451 -0.207

AJ132931 0.169 0.456 0.171 0.132 0.26 0.227 0.358 1.171 0.025 0.341 0.032 0.226

AJ242515 -0.633 -0.261 1.215 1.369 1.127 0.905 0.96 1.837 0.972 0.157 0.575 0.199

AJ286835 -0.492 0.227 0.486 0.832 0.48 0.209 0.063 1.143 0.247 0.35 0.796 -0.004

AJ290391 -0.311 0.368 0.397 0.625 0.558 -0.267 0.337 0.764 -0.007 -0.37 0.082 -0.231

AW019238 -0.216 -0.052 0.308 0.259 0.529 0.395 0.511 1.461 0.615 0.015 0.247 -0.15

AW019266 -0.253 0.726 0.354 0.75 1.036 -0.332 0.482 1.434 0.373 -0.17 -0.141 -0.236

AW019691 0.071 -0.249 1.142 1.576 1.157 1.004 1.297 1.874 0.555 0.291 0.019 0.123

AW058816 0.159 0.187 0.357 0.631 0.419 0.019 0.333 0.955 -0.057 0.549 0.158 -0.125

AW058875 0.214 0.637 0.516 0.708 0.6 0.046 0.389 1.093 0.217 -0.139 0.384 -0.029

AW058992 -0.039 -0.188 -0.093 0.244 0.086 -0.093 0.467 0.636 -0.128 0.402 -0.015 -0.666

AW059102 -0.023 -0.968 0.309 -0.216 1.174 0.282 0.602 1.757 0.383 0.283 0.178 -0.06

AW059104 -0.452 -0.557 -0.719 -0.07 -0.082 0.753 0.438 1.768 0.401 0.533 0.671 0.06

AW059217 -0.014 -0.236 0.313 -0.342 0.351 -0.25 0.435 1.328 0.246 0.191 0.87 -0.109

AW076647 -0.447 0.496 0.695 0.942 1.116 -0.095 0.636 1.256 -0.05 -0.183 0.158 -0.28

AW077940 -0.856 -0.226 0.373 0.554 0.555 0.273 0.446 1.243 0.733 0.174 0.514 0.69

AW078116 0.282 0.095 0.469 0.446 0.505 0.278 0.346 0.749 0.143 0.447 0.009 0.162

AW078266 -0.078 -0.497 0.216 0.744 1.043 -0.03 0.47 1.069 0.415 0.355 0.564 0.132

AW078445 -0.426 0.271 0.407 0.896 1.286 -0.01 0.14 1.529 -0.233 -0.064 0.999 0.102

AW115770 0.128 0.243 0.713 0.754 0.716 0.547 0.835 1.097 0.439 0.937 0.302 -0.027

AW115824 -0.079 0.695 0.123 0.202 0.553 0.388 0.674 1.318 0.504 0.293 0.695 0.429

AW115897 0.181 -0.533 -0.133 -0.214 -0.004 -0.083 0.168 0.65 -0.03 0.017 0.118 0.078

AW116127 0.687 0.337 0.601 1.006 1.419 0.685 0.944 1.657 0.472 0.37 0.274 0.099

AW116147 0.117 -0.114 0.345 0.843 0.92 0.395 0.829 1.245 0.365 0.85 0.252 -0.072

AW116386 -0.049 0.603 0.558 0.815 0.705 0.072 0.464 0.967 0.141 0.109 0.195 -0.153

AW117076 -0.788 -0.032 0.368 1.124 1.434 0.635 0.431 1.525 0.822 0.522 0.35 0.111

AW128211 -0.266 -0.234 0.154 -0.416 0.363 -0.163 0.204 1.017 0.087 0.403 -0.314 -0.426

AW128372 -0.413 -0.823 -0.677 -1.099 -0.88 0.196 0.171 1.339 0.938 0.645 0.184 -0.491

AW128384 0.108 0.352 0.364 -0.174 0.091 -0.059 0.06 0.767 -0.057 0.067 -0.231 0.071

AW128413 0.744 0.198 0.701 0.257 0.598 0.514 0.451 1.35 0.585 0.37 0.263 -0.412

AW133803 0.254 -0.058 0.032 0.345 0.447 0.014 0.35 0.624 0.378 0.373 0.186 0.211

AW154414 -0.098 0.387 0.774 0.743 1.154 0.213 0.457 1.152 0.089 0.296 0.058 0.102

AW154468 -0.342 -0.932 -0.562 -0.633 -0.581 -0.276 0.304 1.138 0.262 0.285 0.593 0.406

AW154507 0.028 0.573 0.343 0.265 0.538 -0.254 0.317 0.818 -0.045 -0.506 -0.399 -0.431

AW174857 0.29 -0.631 -0.05 -0.074 0.383 0.505 0.36 1.174 0.454 0.642 0.457 0.329

AW174887 -0.282 0.635 0.886 0.909 1.184 0.015 0.339 1.237 0.293 0.34 0.276 -0.102

AW175480 0.345 -0.109 0.185 0.187 -0.407 0.096 0.067 0.76 0.487 0.601 0.414 0.037

AW184197 -0.121 0.059 0.254 0.378 0.313 -0.003 -0.033 0.53 -0.219 -0.272 -0.368 -0.109

AW232435 0.644 0.616 0.426 0.362 0.253 0.831 0.497 0.966 0.474 0.834 0.413 0.335

AW233586 -0.114 0.677 0.161 0.532 0.429 -0.106 0.25 0.911 -0.139 0.129 -0.032 -0.018

AW279985 1.203 -0.098 0.258 0.185 0.795 0.619 0.512 1.388 0.367 0.61 0.473 0.196

AW282142 0.317 -0.822 1.506 1.124 2.397 1.256 0.986 2.554 0.568 0.586 -0.098 -0.414

AW305462 0.421 0 0.585 0.565 0.322 0.704 0.492 0.879 0.265 0.389 -0.042 -0.058

AW306050 0.124 -0.909 -0.997 -1.829 0.367 0.405 -0.212 0.804 0.226 0.141 -0.63 -0.598

AW343324 -0.004 0.199 0.592 0.629 0.609 0.435 0.42 1.155 0.283 0.495 0.512 0.554

AW343567 -0.282 0.234 0.786 0.616 1.126 0.024 0.758 1.342 0.09 -0.096 0.237 -0.222

AW420476 -0.207 0.017 1.64 1.499 1.477 1.001 1.065 1.827 0.769 0.771 0.652 0.532

AW420705 -0.372 -0.078 0.27 0.464 0.984 -0.113 0.442 0.959 0.266 0.249 0.412 0.311

AW421172 -0.081 0.036 -0.268 0.755 0.219 0.32 0.166 1.056 0.125 0.761 0.275 0.124

AW421309 -0.122 0.398 0.321 0.5 0.544 0.231 0.19 1.307 0.613 0.281 0.215 -0.162

AW777479 0.049 -0.186 0.12 0.715 0.884 -0.048 0.359 1.185 0.444 0.262 0.262 -0.189

AW777539 0.462 0.017 0.315 0.164 0.389 0.693 0.783 1.47 0.955 1.126 1.004 0.234

AW777903 0.152 0.285 0.528 0.388 0.451 0.658 0.689 1.402 0.47 0.273 0.748 0.449

AW778179 0.308 -0.115 -0.143 -0.24 -0.255 -0.109 0.15 1.203 0.796 0.798 0.824 0.035

AW826449 -0.167 -0.481 0.095 0.485 0.748 0.221 0.705 1.194 0.337 0.515 0.751 -0.065

AW826550 -0.447 -0.205 -0.089 -0.308 0.31 0.615 0.654 1.052 0.672 0.244 0.636 0.817

AY007990 -0.608 -0.548 0.527 1.177 1.086 0.759 0.708 1.865 0.567 1.075 1.531 0.413

AY017308 -0.808 0.235 0.33 0.476 1.175 0.352 0.469 1.762 0.836 0.169 0.921 0.336

AY029808 -0.242 -0.338 0.414 1.08 1.201 0.212 0.686 1.673 0.498 0.414 0.818 0.4

BE015668 -0.182 1.074 1.08 0.947 0.827 0.062 0.468 1.818 0.313 0.286 -0.556 0.062

BE200552 -0.674 -1.039 0.077 -0.394 0.096 0.382 0.538 1.188 0.455 0.794 0.822 0.709

BE201102 -0.578 -0.002 0.328 -0.584 -0.775 -0.235 0.285 1.231 0.15 0.147 -0.153 -0.326

BE201182 0.008 0.333 1.443 1.519 0.831 1.214 1.411 1.714 0.522 0.295 0.474 0.436

BE201398 -0.619 0.545 0.508 0.58 0.563 0.361 0.566 1.091 0.799 0.096 0.286 -0.376

BE556860 0.1 0.477 -0.408 -0.266 -0.365 -0.281 -0.19 0.71 0.141 0.227 0.003 0.032

BE558061 -0.099 0.286 0.105 -0.236 0.339 -0.172 0.295 0.679 0.012 -0.174 -0.116 0.425

BE605273 -0.283 -0.062 -0.172 0.329 0.25 -0.038 0.23 0.66 0.254 0.527 0.28 0.449

BE606074 0.053 0.259 0.728 0.368 0.538 0.454 0.765 1.043 0.531 0.125 -0.146 -0.346

BE606087 -0.052 -0.401 -0.179 0.094 0.115 0.046 0.345 1.013 0.465 0.889 0.18 -0.148

BF156220 -0.23 0.1 0.252 0.703 0.807 -0.046 0.121 0.845 0.22 0.081 0.073 0.195

BF718175 -0.286 -0.042 0.059 0.483 -0.026 0.257 0.192 0.661 0.306 0.286 0.363 0.357

BF937404 0.212 0.156 0.651 0.435 0.607 0.324 0.4 0.773 0.24 0.602 -0.049 0.081

BF938837 -0.15 0.631 0.229 0.472 0.262 0.044 0.308 0.665 -0.017 0.075 0.017 0.087

BG302807 0.549 0.227 0.123 0.95 0.915 0.701 0.488 1.128 0.562 0.239 0.2 -0.414

BG303289 -0.632 0.013 0.411 0.523 0.544 0.1 0.496 1.044 0.188 0.146 0.331 0.149

BG303613 0.671 0.767 0.7 0.937 0.708 0.574 0.426 1.408 0 0.325 -0.119 -0.285

BG304219 -0.361 -0.025 -0.153 -0.098 -0.032 0.003 0.239 0.952 0.087 -0.122 0.053 -0.176

BG305366 0.322 0.513 0.245 0.638 0.53 0.491 0.506 0.911 0.162 0.815 0.398 0.204

BG305992 -0.157 0.144 0.072 0.335 0.219 -0.068 0.211 0.688 0.035 0.378 0.111 0.128

BG306139 -0.728 -0.321 -0.53 0.092 0.905 0.195 0.82 1.87 0.962 0.423 0.449 0.021

BG306178 0.08 0.793 0.318 0.59 0.873 0.06 0.486 1 0.686 -0.001 -0.122 0.064

BG306318 -0.519 -0.806 0.173 -0.688 0.186 0.344 0.725 1.374 0.494 0.71 0.621 -0.02

BG727890 -0.012 -0.366 0.39 0.278 0.043 0.527 0.378 0.565 0.079 0.365 -0.012 -0.1

BG728568 -0.065 0.141 0.596 0.895 1.13 0.366 1.155 1.498 0.35 -0.132 -0.068 -0.095

BG728626 -0.497 0.217 0.21 -0.233 -0.117 0.035 0.289 0.951 -0.057 -0.26 -0.216 -0.117

BG728956 -0.929 -0.46 -1.681 -0.726 -1.024 -0.008 0.322 1.004 0.57 0.774 -0.277 -0.505

BG737957 -0.869 0.267 -0.12 -0.52 0.285 0.691 0.642 1.6 0.856 0.988 0.801 1.199

BG799577 -0.631 0.045 0.034 0.119 0.914 0.084 0.059 1.057 0.763 0.388 0.565 0.135

BG891888 0.237 0.63 0.372 0.591 -0.076 0.396 0.163 0.737 0.082 0.35 0.254 -0.278

BG891893 -0.21 0.27 -0.006 0.373 0.649 0.117 0.462 0.588 0.186 0.069 0.149 0.247

BG892019 0.159 0.083 0.669 0.665 0.672 0.278 0.495 0.822 0.211 0.453 -0.01 0.037

BG985671 0.174 0.674 1.022 0.646 0.603 1.245 0.619 1.324 0.561 0.888 0.371 -0.011

BG985738 0.078 -0.628 0.151 -0.304 0.21 0.055 0.237 1.621 0.634 0.365 0.529 0.32

BG985777 0.309 0.374 0.744 0.386 0.72 0.534 0.437 1.592 0.551 0.72 -0.118 -0.579

BI318628 0.088 0.256 0.334 0.489 0.756 0.102 0.454 1.29 0.105 0.162 0.433 0.088

BI427744 0.083 -0.766 -0.593 -0.754 -0.166 0.09 0.7 1.512 0.578 1.069 1.138 0.023

BI472540 0.047 -0.037 0.244 0.57 0.483 0.067 0.624 1.821 0.393 0.061 0.056 -0.179

BI476025 0.38 0.717 0.928 0.912 1.282 0.61 0.637 1.305 0.391 0.263 0.068 0.364

BI476547 0.147 0.468 0.546 0.176 0.55 0.333 0.265 1.093 0.116 0.204 -0.104 -0.039

BI476729 0.063 -0.106 0.142 0.163 0.568 0.101 0.344 0.921 -0.099 0.38 0.234 0.358

BI672765 0.059 0.622 0.077 -0.276 1.064 0.033 0.593 1.155 -0.073 -0.205 0.002 -0.093

BI673379 0.753 0.688 0.925 0.464 0.553 0.563 0.375 1.037 0.089 0.173 -0.032 -0.124

BI673606 -0.078 0.373 0.325 0.767 0.994 0.313 0.526 1.359 0.067 0.529 0.105 -0.048

BI704280 -0.279 -0.691 0.123 0.992 0.841 0.25 0.523 1.311 0.568 0.54 0.595 0.417

BI704359 -0.304 -1.06 0.096 0.645 1.735 0.303 0.528 1.749 0.437 0.283 0.529 0.44

BI705930 -0.413 0.053 0.891 1.043 1.799 0.857 0.903 2.107 0.961 0.614 0.353 -0.268

BI706099 -0.055 0.244 -0.038 0.179 0.294 0.177 0.38 0.61 0.124 0.205 -0.161 0.187

BI708781 0.073 0.201 0.265 0.527 0.675 0.398 0.281 0.786 0.106 0.247 -0.006 -0.206

BI709791 0.401 0.147 0.542 0.974 0.961 0.605 0.91 1.311 0.118 0.368 0.124 -0.011

BI710051 -0.037 0.579 0.505 0.256 -0.097 0.487 0.388 0.65 0.534 0.469 -0.082 -0.392

BI839826 -0.27 -0.1 0.991 -0.667 0.257 0.081 0.769 1.683 0.311 -0.042 0.515 0.173

BI841627 0.641 0.732 1.335 1.354 1.279 1.143 0.951 1.659 0.752 0.76 0.279 -0.092

BI843129 0.032 0.708 0.535 0.885 0.934 -0.018 0.376 1.124 0.078 0.23 0.099 -0.296

BI843519 -0.63 -0.289 -0.402 -0.171 0.878 -0.011 0.19 1.519 0.458 -0.708 -0.575 -0.416

BI845781 -0.008 0.347 0.11 0.182 0.395 -0.03 0.183 0.903 0.18 0.137 0.487 0.039

BI845814 0.069 0.095 -0.177 0.134 -0.256 0.089 0.047 0.549 0.251 0.205 0.046 0.269

BI850036 -0.381 0.056 0.543 -0.309 -0.03 0.063 0.425 0.655 0.134 -0.065 0.434 0.083

BI850039 0.1 0.126 0.228 0.623 0.88 0.045 0.356 1.09 0.158 0.709 0.145 0.304

BI866976 0.714 -0.115 0.081 0.285 0.572 0.336 0.768 1.078 0.118 0.656 0.839 0.791

BI867489 -0.16 -0.564 -0.125 0.387 0.646 0.258 0.174 0.87 0.14 0.63 0.463 -0.099

BI867946 0.179 -0.387 0.061 0.059 0.296 -0.118 0.092 1.01 0.206 0.468 0.4 -0.2

BI877263 0.119 0.345 0.164 0.219 0.266 0.125 0.448 0.684 0.17 0.009 -0.103 -0.219

BI877622 0.153 -0.125 0.64 0.42 0.789 0.275 0.272 0.846 0.019 0.412 0.136 0.265

BI877938 0.425 0.528 0.486 0.398 0.229 0.773 0.803 0.843 0.427 0.407 0.028 0.035

BI878085 -0.526 0.415 0.407 0.288 0.202 -0.151 0.43 1.141 0.264 -0.131 0.569 0.335

BI878279 0.3 0.394 0.667 0.684 1.069 0.373 0.383 1.175 0.197 0.486 0.1 -0.11

BI878322 -0.023 0.069 0.994 1.21 1.378 0.954 0.976 1.657 0.455 0.788 0.478 0.377

BI878464 -0.075 0.635 0.266 0.656 0.628 -0.246 0.374 1.176 0.277 -0.514 -0.365 -0.245

BI878520 0.1 0.287 0.144 0.035 0.19 0.463 0.361 1.139 0.462 0.548 0.454 0.218

BI878583 -0.481 0.053 0.104 0.547 0.369 -0.154 0.139 0.851 0.031 -0.047 0.409 0.215

BI879889 0.705 0.291 0.82 0.328 0.393 0.187 0.491 1.122 0.447 0.905 0.363 0.28

BI879932 -0.023 0.003 0.152 -0.029 0.11 -0.317 0.619 2.253 0.598 0.552 0.177 -0.129

BI880304 -0.323 0.093 -0.191 0.341 0.123 -0.264 -0.1 0.91 -0.118 0.113 -0.039 -0.355

BI880694 -0.27 -0.141 0.065 -0.013 -0.093 -0.083 0.098 0.623 -0.017 -0.078 0.239 0.178

BI883638 -0.728 0.378 0.775 1.612 1.456 0.707 0.503 1.887 -0.378 -0.449 -0.543 -0.913

BI885253 0.145 -0.137 0.177 0.218 0.499 0.154 0.289 0.887 0.481 0.391 0.375 0.019

BI885907 0.416 0.36 0.02 -0.174 0.313 0.857 0.281 0.893 0.136 0.273 -0.049 -0.2

BI886127 0.255 -0.085 0.734 1.066 1.133 0.715 0.573 1.338 0.404 0.211 -0.053 -0.321

BI886163 0.361 -0.104 -0.048 -0.308 0.044 0.455 0.325 0.939 0.197 0.443 -0.016 -0.08

BI886187 -0.127 0.156 0.777 0.348 0.603 0.188 0.232 0.835 0.332 0.304 0.002 -0.029

BI886387 0.233 0.051 0.434 0.114 0.265 0.056 0.027 0.89 0.439 0.296 0.077 -0.426

BI887166 0.133 0.242 0.172 0.361 0.636 0.122 0.45 0.833 0.156 0.035 -0.074 -0.18

BI887172 0.36 0.238 0.86 0.975 1.743 0.948 1.018 1.916 0.273 0.395 0.093 0.011

BI887346 -0.167 -0.595 -0.09 0.205 0.414 0.462 0.227 1.161 0.579 0.595 0.144 -0.045

BI887368 -0.627 0.484 4.191 4.239 4.366 2.479 2.782 4.767 3.248 0.972 1.059 -0.289

BI887401 0.286 0.613 0.693 0.672 1.098 0.47 0.913 1.565 0.245 0.458 0.157 0.179

BI887659 -0.701 -0.062 0.214 0.668 1.419 0.944 0.525 1.618 0.846 0.473 0.175 0.002

BI887817 0.118 -0.177 0.402 0.546 1.46 0.666 0.661 1.592 0.38 0.214 -0.029 -0.165

BI888033 -0.728 0.119 -0.124 0.55 1.038 -0.063 0.225 1.296 0.491 0.525 0.516 0.404

BI888258 -0.51 0.062 0.597 0.844 1.021 -0.054 0.195 1.114 0.218 0.218 0.296 0.178

BI888458 0.149 0.751 0.733 0.911 0.834 0.156 0.657 0.946 -0.241 -0.076 -0.076 -0.346

BI888505 0.288 0.726 0.419 0.644 0.852 -0.256 0.384 1.143 -0.022 0.092 0.002 -0.229

BI888606 0.139 0.639 1.428 0.292 -0.006 0.468 0.819 2.067 0.641 0.975 1.109 0.157

BI888791 -0.38 0.148 0.243 1.236 1.632 0.735 1.209 1.865 0.663 -0.543 -1.019 -0.862

BI888801 0.314 0.814 0.626 0.715 0.243 -0.121 0.302 0.97 0.136 0.63 0.515 -0.061

BI889131 -0.601 -0.014 0.006 0.433 0.771 -0.04 0.234 0.902 0.287 0.335 0.091 -0.054

BI889280 0.42 -0.441 0.591 1.001 1.383 0.305 0.755 1.604 0.627 0.528 0.329 -0.446

BI889302 -0.309 0.105 0.437 0.073 0.165 0.014 0.428 1.442 0.082 0.122 -0.083 0.527

BI889310 -0.124 0.214 0.238 0.664 0.692 0.334 0.432 0.748 0.197 0.206 -0.427 -0.472

BI889398 0.024 0.807 1.253 1.286 2.089 1.138 2.005 3.676 1.857 0.508 -0.139 -0.246

BI889533 0.048 -0.127 0.298 0.074 -0.053 -0.279 -0.022 0.926 -0.041 0.295 -0.405 -0.383

BI889786 -0.592 -0.224 0.329 -0.058 0.719 0.242 0.43 1.88 0.831 0.626 0.618 -0.513

BI889933 0.021 0.406 0.306 0.636 0.852 0.102 0.439 1.003 0.256 0.303 0.443 -0.062

BI889946 0.555 0.334 0.59 0.121 0.48 1.18 0.877 1.201 0.699 0.919 0.444 0.017

BI890034 -0.645 -0.75 1.688 1.398 1.372 0.551 0.855 1.619 1.225 0.972 0.716 -0.164

BI890141 0.218 0.137 0.246 0.792 1.365 0.386 0.601 1.469 0.275 0.257 0.174 0.295

BI890247 0.321 0.482 0.614 0.81 1.18 0.528 0.473 1.237 0.183 0.411 0.32 0.043

BI890250 0.058 1.066 1.703 1.222 1.595 0.58 0.751 1.809 1.011 0.619 0.259 0.273

BI890730 0.135 -0.08 0.171 0.591 1.052 0.243 0.432 1.53 0.565 0.062 -0.264 -0.052

BI891068 -0.046 0.847 0.558 0.753 1.104 0.043 0.411 1.189 0.138 0.193 0.119 0.113

BI891290 0.356 -0.442 0.277 0.339 0.95 0.191 0.632 1.737 0.417 0.578 0.559 0.384

BI891320 0.594 0.645 1.328 1.705 1.746 0.994 1.596 1.706 0.751 1.04 0.457 0.173

BI891570 0.407 0.303 0.967 0.997 0.924 0.221 0.556 1.14 0.415 0.553 0.134 -0.183

BI891596 -0.679 0.306 0.506 1.402 1.64 0.831 1.028 2.391 0.936 0.054 -0.149 -0.387

BI891665 0.384 0.727 0.759 1.22 1.061 0.744 0.895 1.434 0.198 0.234 -0.023 -0.017

BI891768 -0.24 0.184 1.556 1.377 1.567 0.938 0.89 1.853 0.48 0.635 0.29 -0.294

BI891860 0.245 -0.126 1.259 1.444 1.464 0.812 1.385 1.597 1.146 0.707 0.751 0.587

BI891948 -0.474 -0.088 3.268 3.516 3.885 2.822 2.563 4.233 2.387 1.651 0.537 -0.247

BI892100 -0.454 -0.31 -0.4 -1.317 -0.335 0.121 0.202 1.495 0.386 0.402 0.552 0.297

BI892134 -0.265 0.443 0.555 0.226 0.768 0.073 0.184 1.085 -0.059 0.162 -0.417 -0.216

BI892167 -0.592 0.33 1.084 2 1.776 1.112 0.94 3.149 2.268 1.455 0.915 -0.497

BI892176 0.513 0.527 0.17 0.424 0.305 0.068 0.198 0.792 0.181 0.567 0.08 -0.256

BI892323 -0.688 0.023 -0.257 0.037 0.455 0.16 0.82 1.502 0.553 0.138 -0.229 -0.086

BI892431 0.001 0.348 0.383 0.528 0.428 -0.117 0.255 0.784 0.191 -0.035 -0.11 0.045

BI892444 0.454 0.01 0.712 0.792 1.718 0.442 0.731 1.949 0.543 0.718 -0.148 -0.313

BI979064 -0.218 -0.288 -0.129 -0.046 -0.31 0.021 0.185 0.941 0.209 0.108 0.337 0.199

BI979451 0.071 0.36 0.395 0.587 0.775 0.232 0.498 0.795 0.091 0.041 -0.14 -0.056

BI982770 -0.661 -0.359 -0.402 -0.829 -0.266 0.203 0.145 1.147 0.423 0.501 0.769 0.463

BI984380 0.336 0.393 0.042 0.314 0.728 0.079 0.524 0.916 0.076 0.334 -0.021 -0.151

BM004967 0.139 0.479 0.187 0.056 0.033 0.512 0.366 1.108 0.384 0.724 0.381 0.963

BM023784 0.268 0.468 -0.161 0.068 0.262 0.487 0.363 1.108 0.378 0.4 0.293 0.112

BM024640 0.374 -0.03 0.378 0.634 0.651 0.312 0.585 0.874 0.09 0.459 0.351 0.272

BM025866 0.014 0.147 0.154 0.431 0.999 0.036 0.455 1.55 0.297 0.729 0.231 0.056

BM026316 0.367 0.518 0.407 -0.014 0.108 0.602 0.459 0.746 0.102 0.154 -0.219 0.032

BM071353 0.631 0.304 -0.085 -0.112 0.182 0.73 0.684 0.874 0.422 0.583 0.286 0.026

BM083952 0.007 0.279 0.091 0.712 0.771 0.315 0.659 0.954 0.399 0.31 0.298 -0.062

BM095178 -0.112 0.016 -0.015 -0.14 0.352 0.171 0.092 0.885 0.154 -0.005 -0.271 -0.003

BM095868 -0.75 -0.797 -0.393 -0.154 0.532 -0.016 0.337 1.427 0.743 0.591 1.118 0.455

BM101531 -0.318 0.397 0.127 0.201 0.949 -0.039 0.046 1.014 0.273 0.331 0.236 0.204

BM102623 0.191 0.548 0.08 0.494 0.914 0.222 0.45 1.166 0.294 0.126 0.137 0.102

BM154004 0.531 0.71 0.359 0.425 0.795 0.171 0.464 0.92 -0.072 0.22 0.05 0.197

BM156045 -0.618 0.02 -0.055 0.239 0.589 0.056 -0.056 0.624 -0.119 0.085 0.118 0.126

BM156717 0.249 0.103 -0.327 -0.045 0.042 -0.217 0.252 0.733 0.051 0.586 0.119 0.344

BM157248 0.826 0.935 0.283 0.433 0.388 -0.127 0.306 1.135 0.211 0.498 0.277 0.144

BM182248 0.2 0.884 0.332 0.627 0.838 0.006 0.552 1.2 0.205 0.412 0.443 -0.129

BM182275 0.121 0.366 0.235 0.671 0.456 -0.113 0.207 0.762 0.153 0.356 0.512 -0.089

BM182302 -0.191 0.224 0.419 0.228 0.811 0.059 0.376 0.998 -0.039 0.183 0.541 0.186

BM183518 0.1 0.243 -0.014 -0.125 0.167 -0.146 0.046 0.563 -0.139 0.129 0.01 -0.021

BM184045 0.084 0.626 0.251 0.688 0.601 0.427 0.387 0.698 0.085 0.216 0.081 0.009

BM184100 0.398 -0.112 0.133 0.409 1.143 0.212 0.37 1.15 0.223 0.379 0.104 0.201

BM184237 0.094 0.504 0.321 0.699 0.965 -0.177 0.499 1.073 -0.014 0.031 -0.002 -0.173

BM184886 0.512 0.378 0.148 0.219 0.709 0.051 0.368 0.842 -0.089 0.038 0.019 -0.12

BM186095 0.177 0.138 -0.56 -0.163 -0.294 -0.133 -0.008 0.603 0.135 0.063 -0.064 -0.058

BM186124 -0.343 -0.094 0.164 -0.309 0.013 -0.168 0.181 0.864 0.348 0.497 0.628 0.32

L25273 -0.733 -0.728 -0.523 -0.321 -0.617 -0.451 0.37 0.866 0.162 0.341 0.249 0.65

L27585 -0.148 -0.173 -0.071 -0.033 -0.116 -0.32 0.016 0.667 -0.069 0.26 0.183 0.359

S80425 -0.432 -0.018 1.244 1.182 1.073 0.885 1.207 1.964 0.254 0.059 0.42 0.804

U16311 -0.739 0.472 0.042 0.341 1.947 0.759 0.612 2.266 0.562 0.657 0.574 0.197

U18312 -0.744 0.451 1.602 1.94 1.952 1.665 1.098 2.151 0.744 -0.18 0.224 0.742

U49412 -0.546 0.095 -0.166 0.391 0.748 -0.258 0.534 1.412 0.258 0.578 0.592 0.171

U77595 0.054 -0.069 0.181 0.283 0.299 0.545 0.463 1.15 0.403 0.323 0.356 0.031

U89380 -0.604 0.194 -0.116 0.148 -0.026 -0.007 -0.121 0.629 0.317 0.261 0.328 0.011

X12802 -0.67 -2.849 -2.012 -2.563 -0.37 -0.278 -0.387 -0.018 -1.55 -1.091 -1.142 -0.236

X60095 -0.572 -0.111 0.277 -0.178 -0.074 0.016 0.639 1.558 0.587 0.221 0.639 0.196

X66958 -0.423 0.138 0.256 2.309 3.55 1.587 1.615 4.469 1.539 0.262 0.251 0.201

X70322 -0.733 -0.057 -0.061 -0.137 -0.059 -0.259 -0.16 1.412 0.16 -0.317 -0.045 -0.371

X97333 -1.144 -0.011 0.164 -0.138 0.672 1.826 1.256 2.191 1.625 1.272 1.44 0.741

Y14531 -0.339 0.288 0.407 0.237 -0.081 0.1 0.471 1.725 0.067 -0.194 0.5 0.227

Y14533 -0.755 -0.185 -0.048 -0.962 -0.1 0.132 0.324 1.793 0.594 -0.331 0.967 -0.072

AF200950 -0.381 -0.19 0.329 0.669 0.266 0.25 0.189 0.783 0.699 0.353 0.556 -0.118

AF348959 -0.604 0.237 -0.677 -0.256 -0.354 0.338 0.029 0.537 0.491 0.317 -0.113 -0.066

AI584394 0.083 0.4 1.163 0.993 1.231 0.412 0.756 1.165 1.409 0.743 0.703 -0.931

AI588340 -2.249 -1.739 -2.15 -0.657 -1.332 -0.6 -0.582 0.446 1.137 1.017 0.799 0.13

AI641680 -0.825 0 -0.263 0.341 -0.199 -0.148 -0.144 0.789 1.02 0.682 0.456 0.044

AI942960 -1.507 -0.377 -1.227 -0.116 -0.445 0.181 0.129 1.352 1.273 0.969 0.552 -0.224

BG303611 -1.053 0.619 0.949 -2.695 -3.259 1.368 1.218 -0.13 1.09 0.652 -1.626 -1.486

BG729245 -0.412 -0.172 -0.486 -0.946 -0.063 0.099 0.305 0.898 0.999 0.289 0.1 0.362

BG985449 0.908 -0.539 -0.955 -0.986 -1.351 0.252 0.322 0.346 1.054 0.678 0.291 0.228

BI876163 -2.743 -1.634 0.339 0.339 0.379 0.164 0.043 -0.046 0.84 0.796 0.493 0.366

Mean -0.096 0.08 0.272 0.365 0.576 0.27 0.457 1.203 0.364 0.341 0.232 0.02
