# Supplementary material for: Transcriptome Analysis of Zebrafish Embryogenesis Using Microarrays
Source: PLoS Genet. 2005 Aug 26;1(2):e29. doi: 10.1371/journal.pgen.0010029 (PMC1193535; doi:10.1371/journal.pgen.0010029)
Supplement: Dataset S9 — (29 KB DOC) [file pgen.0010029.sd009.doc]

Dataset S09. List of genes with onset of transcript accumulation at gastrula and peak of expression at pharyngula stages.

Genbank ID UF egg 3hpf 4.5hpf 6hpf 7.7hpf 9hpf 10.7hpf 12hpf 15hpf 24hpf 30hpf 48hpf

AW018949 -4.499 -3.927 -4.361 -3.394 -4.318 -1.436 -0.688 -0.633 0.188 0.714 0.229 -0.113

AW019142 -0.724 0.159 -0.25 0.046 -0.109 0.053 0.466 1.115 0.87 1.555 0.56 -0.099

AW117056 -1.271 -1.706 -1.154 -1.412 -1.451 0.151 0.257 0.997 1.223 1.241 0.872 -1.13

BI473591 -0.17 -1.132 -1.181 -1.591 -0.562 -0.276 -0.413 0.286 0.584 0.666 0.364 -0.243

AI964264 -1.151 -2.091 -1.282 -1.84 -1.095 0.798 0.824 0.897 0.45 1.169 0.719 0.311

BG304333 -0.224 -1.104 -0.552 -0.631 -0.127 0.743 0.279 0.902 0.659 0.979 0.675 0.276

BG728452 -0.464 -2.34 -1.934 -1.535 -0.892 0.171 -0.212 0.31 0.022 0.797 0.316 -0.067

BI325085 -1.339 -1.656 -0.812 -0.741 -1.008 -0.257 -0.282 -0.012 0.552 1.131 1.106 0.503

BI325825 -0.91 -2.913 -1.058 -0.825 -0.396 0.65 0.253 0.413 0.465 1.3 0.914 0.265

BI889370 -1.505 -1.791 -1.555 -0.444 0.061 0.712 0.594 1.074 0.752 1.344 0.869 -0.617

BI890772 0.065 -1.307 -0.353 -0.191 0.144 0.522 -0.002 0.096 0.398 0.587 -0.093 0.038

BI891769 -0.88 -2.729 -1.832 -1.288 -0.963 0.421 0.146 0.206 0.299 0.784 0.576 0.042

BM155568 -0.257 -1.979 -0.899 -0.654 -0.319 0.668 0.379 0.63 0.512 1.372 1.123 0.449

BM182563 -2.762 -5.406 -1.611 -0.382 -0.439 0.344 -0.06 -0.263 -0.409 0.509 -0.033 -0.381

BM183623 -0.533 -3.251 -1.514 -1.026 -0.687 0.337 0.142 0.162 0.273 1.032 0.632 -0.118

AF025330 -1.404 -1.682 -0.85 -0.588 -0.842 -0.235 -0.487 0.207 0.375 1.035 0.821 0.383

AF180921 -0.519 -2.559 -1.147 -1.693 -2.319 0.183 0.319 0.716 0.576 0.779 -0.157 -1.305

AI601793 -1.177 -1.514 -1.009 -1.502 -2.075 -0.573 -0.092 -0.039 0.422 1.235 0.755 -0.048

AI641141 -0.559 -0.699 -0.4 -0.305 -0.516 -0.172 -0.133 -0.03 0.351 0.478 0.419 0.265

AI721634 0.177 -0.407 0.097 0.219 -0.094 0.312 0.459 0.857 0.812 1.055 0.212 -0.623

AI722334 -1.989 -3.035 -1.474 -2.454 -3.394 -1.038 -1.001 -1.328 -0.558 0.73 0.718 0.134

AI877922 -4.777 -4.532 -4.199 -3.744 -4.242 -3.224 -3.122 -1.581 -0.243 1.774 1.229 0.918

AI957628 -1.834 -1.738 -1.437 -1.382 -1.926 -0.757 -0.716 0.819 0.934 1.133 0.968 0.351

AW019450 -0.462 -0.461 0.982 0.437 0.053 0.283 0.307 0.902 0.588 0.868 0.439 0.425

BI672656 -0.605 -1.062 -1.097 -0.743 -1.02 -0.012 -0.72 0.01 0.204 0.562 0.487 0.55

BI886392 -0.413 0.217 0.457 0.627 0.378 0.555 0.448 1.431 1.164 1.438 0.746 0.246

BI886794 -0.224 -0.301 -0.043 0.329 0.084 0.351 -0.012 0.964 0.67 0.821 0.625 0.304

BI889566 -0.419 -0.85 -0.027 -0.362 -0.602 -0.247 0.121 1.031 0.616 1.344 1.312 0.118

BM025541 -0.387 -1.141 0.066 0.303 -1.97 -0.468 0.01 0.63 0.599 0.793 0.499 0.075

BM156982 0.166 -0.269 -0.822 -0.454 -0.753 -0.33 -0.241 0.559 0.399 0.92 0.891 0.36

BM181739 -0.461 -0.193 0.016 -0.78 -0.472 -0.525 -0.172 0.968 0.418 1.007 0.62 -0.323

BM186976 -1.623 -1.194 -1.556 -1.064 -1.519 -0.459 -0.539 0.105 0.376 1.103 0.567 0.221

AI957875 -0.454 -0.784 -0.343 -0.241 -0.533 0.186 0.434 0.898 0.81 0.852 0.611 -0.295

AW115602 0.466 -2.087 -0.839 0.005 0.057 0.807 0.364 0.572 0.579 1.171 0.673 0.673

BG727615 -2.472 -2.218 -2.066 -1.095 -1.503 -0.435 0.193 0.359 0.435 1.371 0.848 0.289

BI427792 -0.625 -3.292 -1.079 -1.225 -1.044 0.025 -0.026 0.018 -0.017 0.713 0.472 -0.033

BI672508 -0.783 -3.033 -1.339 -1.012 -0.677 0.484 0.127 0.473 0.401 1.198 0.868 0.25

BI707410 -0.587 -3.041 -1.196 -0.901 -0.492 0.486 0.33 0.498 0.231 1.061 0.627 0.072

BI710147 -0.552 -2.654 -0.952 -1.001 -0.428 0.616 0.315 0.443 0.249 1.234 0.854 0.227

BI878117 -0.586 -1.903 -0.987 -1.094 -1.077 -0.233 -0.14 0.299 -0.074 0.713 0.413 0.474

BI878720 -0.785 -1.941 -0.526 -0.582 -0.483 0.006 -0.061 -0.322 0.189 0.449 0.251 0.059

BI880392 -1.024 -1.346 -0.976 -0.708 -0.872 0.022 0.498 0.34 0.667 0.97 0.838 0.348

BI888897 -1.229 -2.824 -1.785 -1.181 -0.974 0.193 -0.091 0.092 0.039 0.735 0.348 -0.092

BM035036 -0.873 -1.719 -1.64 -0.908 -0.768 -0.007 -0.236 0.114 0.056 0.59 0.245 -0.382

BM070699 -0.087 -2.361 -1.068 -0.649 -0.569 0.561 0.153 0.48 0.383 1.152 0.897 0.251

BM081047 -0.507 -1.418 -1.065 -0.7 -0.595 -0.076 0.04 0.114 0.274 0.978 0.551 -0.143

BM156154 -1.247 -2.881 -1.119 -0.826 -0.929 0.237 -0.092 0.007 0.132 0.892 0.55 0.054

BM181246 -5.052 -4.362 -1.702 -0.922 0.042 1.376 1.551 1.722 1.819 2.433 1.635 0.163

BM183474 -1.163 -2.775 -1.341 -0.84 -0.581 0.496 0.215 0.389 0.337 1.15 0.855 0.296

Y08321 -6.291 -6.731 -4.656 -4.12 -4.311 -1.57 -0.998 -0.069 0.394 1.843 1.12 0.034

Y13653 -5.37 -5.897 -4.748 -3.724 -2.815 -0.626 -0.576 -0.466 -0.078 1.198 1.081 0.949

AF257743 -0.082 -0.921 -1.051 -0.775 -0.778 0.705 0.394 1.372 0.901 1.828 0.974 0.173

AF195881 -0.784 -0.034 0.315 0.422 0.145 -0.023 0.055 0.351 0.652 1.341 0.448 0.214

AI878244 -0.515 -0.507 0.333 0.497 0.373 0.126 0.204 0.459 0.406 0.492 0.003 0.051

BE200900 -0.459 0.059 -0.275 -0.145 0.133 -0.177 -0.02 0.661 0.33 0.589 0.56 -0.103

BF157490 -1.959 -0.387 0.678 0.703 0.25 0.005 -0.188 0.268 0.789 0.857 0.313 0.217

BI476031 -0.211 0.045 -0.309 0.466 0.539 0.025 -0.057 0.504 0.281 0.479 0.121 0.072

BI839952 -0.362 -0.75 -0.205 0.339 -0.353 -0.163 0.399 0.406 0.558 1.287 0.729 0.287

BI896473 0.104 -0.597 0.284 0.22 0.101 -0.224 0.19 0.461 0.32 0.775 0.596 -0.011

L35586 -1.849 -1.375 -2.687 -1.058 -1.502 -1.008 -1.039 -0.424 0.173 1.539 0.226 -0.745

X97330 -0.077 0.171 -0.234 -0.088 -0.21 -0.334 0.121 1.176 0.519 1.096 0.642 0.21

BI865978 -1.666 -2.161 -0.524 -0.348 -0.393 -0.478 -0.242 0.078 0.241 0.604 0.163 -0.147

AF210320 -0.766 -0.533 -0.787 -1.15 -1.077 -0.153 -0.352 -0.318 0.103 0.486 0.903 0.702

AF375871 -0.508 -1.287 -1.201 -0.967 -1.404 0.093 0.171 0.264 0.427 0.633 0.754 0.457

AI545321 -0.405 -0.149 -0.362 -0.273 -0.473 0.142 0.261 1.085 0.862 0.797 0.94 -0.03

AI884045 -0.728 -0.616 -0.806 -0.849 -0.985 -0.137 0.065 0.805 0.496 0.724 0.735 0.453

U66570 -0.401 -0.682 -0.692 -1.034 -0.838 -0.392 0.065 -0.039 0.065 0.45 0.457 -0.045

X65061 -0.674 0.211 -0.605 -0.467 -0.133 -0.001 0.161 0.573 0.296 0.552 0.747 0.085

Y14530 -0.653 -0.433 -0.675 -0.807 -0.245 0.079 0.036 0.759 0.387 0.687 0.876 0.851

AF101266 0.902 -0.047 -0.45 -0.333 -0.682 0.249 -0.224 0.984 0.732 0.848 1.17 0.109

AI942982 -0.587 0.545 0.407 0.341 -0.048 0.161 0.179 0.571 0.304 0.268 0.582 0.237

BG985575 -0.436 -0.403 0.252 0.179 0.04 0.305 -0.019 0.687 0.395 0.525 0.853 0.462

BM024150 -0.728 -0.193 -0.153 -0.574 -0.83 -0.137 -0.193 0.637 0.338 0.794 1.079 0.533

BM026470 -0.16 -0.107 0.154 0.305 0.001 0.227 0.061 0.657 0.405 0.54 0.594 0.256

Y14548 -0.667 -0.321 0.461 0.024 -0.234 -0.369 -0.098 0.428 0.244 0.224 0.572 0.203

AW777836 -1.107 -1.465 -1.334 -0.982 -1.213 -0.543 -0.044 0.416 0.685 0.914 1.129 0.5

AW279630 0.099 -0.042 -0.632 0.081 0.128 -0.113 0.085 0.482 0.172 0.317 0.544 0.203

BI672308 -0.728 0.037 -0.058 0.184 0.32 -0.059 -0.229 0.147 0.141 0.434 0.518 -0.261

BI708877 -0.344 -0.764 -0.158 0.165 0.076 -0.091 -0.005 -0.069 0.111 0.174 0.388 0.273

AW421939 -5.597 -6.185 -5.674 -4.972 -4.629 -3.232 -2.978 -2.079 -0.6 0.315 0.385 -0.22

AI957592 -0.202 -1.759 -0.142 -0.501 -0.423 0.089 0.23 0.605 0.365 0.217 0.494 0.581

AI793866 -1.399 -0.942 -1.183 -1.287 -0.542 0.175 -0.075 0.146 0.265 0.538 0.964 1.008

AI584515 -0.679 -1.22 -0.3 -0.396 -0.491 0.068 -0.242 -0.349 -0.108 0.07 0.086 0.433

AI878520 -0.436 -0.275 0.469 -0.804 -1.431 -0.219 -0.138 -0.155 -0.108 0.469 0.384 0.74

BM153935 -0.464 -0.304 0.108 -0.332 -0.877 -0.406 -0.656 -0.335 -0.042 0.558 0.516 0.811

Y13944 -0.95 -1.046 -0.46 -0.402 -1.318 -0.378 -0.214 0.11 0.289 0.292 0.601 0.804

AI957401 -1.283 -1.883 -1.559 -1.393 -1.536 -0.303 -0.016 -0.081 -0.097 0.431 0.799 1.195

AW115580 0.062 -0.657 -0.085 -0.53 -0.497 0.517 -0.027 0.157 0.026 0.581 0.514 0.591

BG985455 -2.468 -2.178 -2.284 -1.861 -1.904 0.224 0.138 -0.069 0.355 0.721 1.045 1.76

BI563231 -3.401 -3.266 -2.836 -1.927 -2.733 -0.643 -0.943 -0.335 -0.309 0.219 0.32 1.05

AW826482 -0.755 0.545 -0.445 0.312 -0.027 0.115 0.054 0.6 0.385 0.604 0.757 0.937

BI672289 -0.225 -0.651 -1.036 -0.329 -0.207 0.385 0.344 0.714 0.346 0.67 0.566 0.753

BG985516 -0.567 0.222 0.467 0.401 0.104 -0.334 0.148 0.04 0.321 0.154 0.464 0.482

U85090 -1.479 -1.014 1.227 0.659 0.539 -0.125 -0.049 0.738 0.21 0.719 0.742 1.204

Mean -1.042 -1.502 -0.918 -0.746 -0.821 -0.07 -0.07 0.335 0.362 0.849 0.638 0.231
