# Supplementary material for: Transcriptome Analysis of Zebrafish Embryogenesis Using Microarrays
Source: PLoS Genet. 2005 Aug 26;1(2):e29. doi: 10.1371/journal.pgen.0010029 (PMC1193535; doi:10.1371/journal.pgen.0010029)
Supplement: Dataset S10 — (36 KB DOC) [file pgen.0010029.sd010.doc]

Dataset S10. List of genes with onset of transcript accumulation at the segmentation stage and peak of expression at segmentation stages.

Genbank IDUF egg 3hpf 4.5hpf 6hpf 7.7hpf 9hpf 10.7hpf 12hpf 15hpf 24hpf 30hpf 48hpf

AA606013 -0.728 -0.187 0.145 0.5 0.594 0.433 -0.04 1.119 -0.378 -0.552 -0.543 -0.913

AAC84044 -0.61 -0.061 0.08 0.128 0.163 0.789 -0.425 1.045 -0.031 0.414 -0.381 -0.845

AB032263 -0.416 0.361 0.039 0.596 0.933 0.617 0.188 1.09 -0.117 0.689 0.233 0.008

AB055679 -0.016 0.107 0.131 0.493 0.366 0.515 -0.018 0.903 0.448 0.038 0.14 0.085

AF052249 -0.818 -0.338 0.077 -0.194 0.561 0.13 -0.094 0.814 0.194 0.541 0.081 0.074

AF084948 -0.226 -0.469 -0.34 -0.118 -0.031 -0.113 -0.198 0.694 -0.035 0.194 -0.126 -0.093

AF295804 -0.341 0.332 -0.702 0 -0.059 0.071 -0.166 0.728 -0.108 0.454 0.236 0.048

AF361478 -0.037 0.524 0.601 0.764 0.631 0.857 0.246 0.954 0.331 0.758 0.567 0.04

AI437402 -0.165 -0.364 0.127 0.385 0.528 0.418 0.115 0.732 0.042 0.15 0.12 -0.016

AI461367 -0.352 -0.114 0.283 0.108 1.076 0.493 0.188 1.131 0.361 0.796 0.867 0.709

AI477424 0.691 -0.221 -0.071 0.057 0.64 0.92 0.401 1.207 0.367 0.707 0.506 0.251

AI584223 -0.486 0.151 0.326 0.339 0.614 0.346 -0.02 0.933 0.381 0.524 0.328 0.23

AI641028 0.111 0.253 0.23 0.248 0.103 0.205 0.108 0.848 -0.047 0.141 -0.01 -0.058

AI641401 -0.728 0.141 0.218 0.819 1.336 0.255 0.075 1.694 -0.378 0.006 0.349 0.119

AI667333 0.392 -0.088 0.227 -0.058 0.493 0.402 0.058 0.707 0.183 0.133 -0.005 0.018

AI721333 0.565 0.614 0.097 0.184 0.683 0.681 0.327 0.74 0.198 0.36 -0.057 -0.101

AI721501 -0.452 0.545 -0.058 0.341 0.569 0.14 -0.001 0.836 -0.254 -0.357 -0.059 -0.503

AI793509 -0.395 0.147 -0.373 -0.013 0.204 0.007 -0.127 1.169 0.592 0.453 0.704 -0.18

AI793934 -0.071 0.136 -0.165 0.114 1.356 0.772 0.551 2.449 1.206 0.85 0.444 -0.18

AI884050 -0.403 -0.444 0.494 0.806 1.05 0.714 0.356 1.399 0.134 0.716 0.025 0.258

AI943195 -0.468 -0.635 0 0.199 0.517 0.359 0.043 0.679 0.126 0.256 0.17 0.102

AI943392 0.068 -0.144 0.122 0.099 0.474 0.345 0.1 0.991 0.258 0.024 -0.168 -0.295

AI959222 -0.022 0.029 0.034 0.083 0.471 0.182 0.096 0.798 0.064 0.065 0.074 0.374

AW019579 0.906 0.853 0.742 0.673 0.84 0.675 0.283 1.079 0.481 0.085 0.233 0.453

AW059030 0.104 -0.347 0.134 0.258 0.526 0.516 0.216 1.421 -0.122 -0.024 0.64 0.033

AW059220 0.235 -0.046 0.575 0.652 1.364 0.493 0.261 1.408 0.062 0.507 -0.006 -0.279

AW059266 0.158 0.215 0.188 0.286 0.696 0.945 0.241 1.054 0.253 0.621 0.474 0.113

AW116039 -0.055 -0.295 0.189 0.181 0.877 0.581 0.187 1.28 0.467 -0.106 -0.044 -0.391

AW116654 0.295 -0.367 -0.503 -0.621 0.42 -0.053 -0.171 0.67 -0.059 0.173 -0.543 -0.408

AW116868 0.237 0.591 0.304 0.268 0.542 0.48 0.096 0.829 0.232 0.368 -0.072 0.055

AW171367 0.452 0.272 0.154 0.507 0.808 0.712 0.217 1.368 0.436 0.289 -0.271 -0.79

AW171447 0.667 0.164 0.611 0.125 0.573 0.439 0.197 1.374 0.402 0.225 0.222 -0.33

AW343508 0.249 0.318 0.471 0.047 0.663 0.561 0.214 0.925 0.137 0.332 -0.055 0.053

AW343883 0.373 0.24 0.551 0.566 0.89 1.023 0.477 1.217 0.45 0.045 -0.234 -0.461

AW344113 0.091 0.172 1 0.771 0.897 0.909 0.586 1.296 0.317 0.281 -0.148 -0.02

AW420405 -0.071 -0.023 0.186 0.047 0.649 0.501 0.328 0.902 0.125 0.178 -0.003 0.148

BE016083 -0.035 -0.098 0.035 0.456 0.474 0.431 0.197 0.631 0.356 0.278 0.189 0.181

BE606152 -0.55 -0.017 -0.217 0.158 1.158 0.562 0.227 1.464 0.395 0.658 0.113 0.532

BG304234 0.568 0.008 0.163 0.06 0.227 0.673 0.158 1.032 0.392 0.569 0.254 0.199

BG304255 -0.066 0.101 0.286 0.641 1.051 0.373 0.082 1.34 0.684 0.338 0.093 -0.46

BG308784 0.444 0.503 0.804 0.768 0.886 0.339 0.189 1.159 0.033 0.518 0.085 0.168

BI562940 -0.06 -0.044 0.106 0.389 0.862 1.158 0.364 1.418 0.699 1.054 0.773 0.669

BI704180 0.319 0.358 0.727 0.517 0.876 0.657 0.255 1.283 0.477 0.025 -0.332 -0.762

BI704236 0.017 0.672 1.448 1.486 1.461 1.704 0.918 2.217 0.828 0.741 0.236 -0.293

BI709620 0.032 -0.252 -0.603 -0.133 0.059 0.526 -0.067 0.751 0.044 0.553 0.28 0.403

BI841076 -0.681 0.197 0.33 0.745 0.871 0.254 0.059 1.281 0.29 0.453 -0.033 -0.511

BI846592 -0.52 -0.291 -0.575 0.186 1.094 1.081 0.554 1.892 0.678 1.301 0.118 -0.436

BI864451 0.26 0.605 0.67 0.508 0.044 0.342 0.226 0.95 0.162 0.759 0.497 0.026

BI866308 0.261 -0.022 0.261 0.563 1.093 0.858 0.592 1.13 0.412 0.121 0.797 0.847

BI866448 0.095 0.187 0.309 0.309 0.682 0.279 0.065 0.91 0.125 0.223 0.033 0.225

BI878923 0.3 0.254 1.012 0.878 1.016 0.996 0.472 1.171 0.444 0.739 0.144 0.102

BI879489 0.118 -0.063 -0.06 0.04 0.258 0.617 0.239 0.842 0.35 0.73 0.333 0.425

BI882169 0.11 -0.045 0.026 0.393 0.693 0.66 0.272 1.09 0.126 0.275 -0.025 -0.012

BI883018 -0.337 -0.472 0.065 -0.359 0.905 0.68 0.321 0.851 0.161 0.512 0.402 -0.028

BI886251 -0.942 0.35 1.821 1.613 1.83 1.545 0.726 2.09 1.646 0.922 0.061 -0.45

BI886329 -0.325 0.242 1.073 1.125 1.314 1.193 0.745 1.851 0.857 1.47 0.753 0.31

BI888177 -0.01 0.348 -0.054 0.445 0.422 0.422 0.189 0.886 0.49 0.258 0.348 0.211

BI889526 -0.542 -0.235 0.916 1.028 1.015 1.218 0.563 1.338 0.373 0.122 -0.048 0.2

BI890771 0.276 0.736 0.531 0.582 0.446 0.285 0.081 0.893 0.244 0.626 0.545 0.164

BI891076 0.3 0.27 -0.037 0.211 0.031 0.105 -0.019 0.816 -0.143 -0.192 -0.315 -0.166

BI891122 -0.222 -0.152 0.366 0.649 0.468 0.617 0.326 0.919 0.295 0.652 0.158 0.151

BI892151 -0.482 0.739 0.814 1.05 1.093 0.584 0.325 1.247 0.461 0.128 0.047 -0.341

BI981135 -0.642 0.545 0.362 0.61 0.599 0.225 -0.07 1.06 -0.157 0.07 -0.329 0.32

BM026032 0.384 0.164 0.846 0.284 0.554 0.438 0.193 0.89 0.207 0.249 -0.031 -0.132

BM026429 0.237 0.112 0.195 0.334 0.646 0.694 0.352 1.212 0.319 0.463 0.054 0.085

BM036954 -0.079 0.525 0.89 0.22 1.117 0.466 0.19 1.613 0.165 0.167 0.228 0.004

BM102872 0.207 0.21 0.893 0.293 0.483 0.693 0.095 1.446 0.681 0.428 0.236 0.04

BM103943 -0.252 -0.35 0.106 0.388 1.142 0.929 0.436 1.385 0.36 0.752 0.646 0.303

BM157095 -0.138 0.607 0.684 0.72 0.69 0.491 0.261 1.014 0.605 0.342 0.09 -0.38

BM183007 0.444 0.612 0.397 0.323 0.502 0.597 0.151 1.044 0.517 0.875 0.719 0.593

BM183249 -0.239 -0.69 -0.482 -0.151 0.087 0.486 -0.127 0.966 -0.378 0.361 -0.543 -0.913

BM184509 0.564 0.656 0.638 0.63 0.418 0.275 0.244 0.901 0.298 0.514 0.666 0.433

BM184838 0.555 0.055 0.702 0.275 0.941 0.633 0.254 1.262 0.75 0.825 0.183 -0.06

BM185198 -0.061 -0.147 0.116 -0.103 0.653 0.341 0.236 0.891 0.133 0.162 -0.018 0.275

BM185367 0.436 0.298 0.508 0.38 0.577 0.591 0.344 0.805 0.36 0.485 0.212 0.158

U49408 -0.328 -0.358 -0.316 -0.29 -0.057 0.499 0.038 1.437 1.11 0.717 1.1 0.23

U77627 0.101 0.147 0.3 0.64 0.963 1.236 0.407 1.358 0.766 0.434 0.17 -0.376

AA495267 -2.947 -2.885 -0.284 -0.332 0.144 -0.21 -0.444 -0.942 0.653 -0.326 0.395 0.116

AF162696 -0.76 -0.417 0.218 0.299 0.412 0.356 0.468 0.214 0.738 0.699 0.679 0.363

AI461372 -2.624 -1.519 -0.695 -0.453 0.061 -0.085 -0.633 -0.366 0.678 -0.228 0.483 0.375

AI522706 -0.503 -0.335 -0.576 0.09 -0.618 -0.441 -0.364 -0.156 0.76 0.744 0.408 -0.411

AI584734 -3.322 -3.041 -0.694 -0.67 0.139 -0.288 -0.813 -1.15 0.762 -0.179 0.518 0.339

AI588708 -0.728 -0.544 0.996 0.962 1.027 0.593 0.424 0.634 1.445 0.812 0.558 0.162

AI626451 -0.32 -0.687 0.08 -0.842 0.26 -0.195 -0.607 -0.378 0.51 -0.343 0.468 0.429

AI721548 -1.777 -1.616 -0.273 -0.415 0.22 0.126 -0.172 -0.319 0.709 0.074 0.554 0.312

AI878235 -1.376 -2.631 -0.993 -0.448 -0.506 -0.381 -0.162 -1.336 0.286 -0.643 0.145 -0.122

AW422922 -0.19 0.227 0.55 0.56 0.542 0.724 0.091 0.686 0.929 0.86 0.764 0.417

AY029527 -0.174 -0.863 0.398 0.28 -0.097 0.795 0.341 -0.083 1.029 0.829 0.22 -0.052

BI710295 -2.211 -3.349 -0.592 -0.752 -0.206 -0.391 -0.467 -0.939 0.43 -0.376 0.427 0.18

BI880007 -2.635 -2.384 -0.794 -0.805 -0.067 -0.291 -0.774 -1.134 0.475 -0.469 0.472 0.251

BI891804 -2.112 -1.02 -0.426 -0.228 -0.004 -0.159 -0.345 -0.585 0.409 -0.315 0.343 0.283

BM184381 -0.688 -1.837 -1.252 -0.738 -1.163 -0.957 -0.642 -0.323 1.195 0.975 1.079 -0.445

BM185251 -2.041 -1.986 -0.391 -0.447 0.317 -0.132 -0.396 -1.013 0.494 -0.344 0.437 0.42

Mean -0.281 -0.178 0.188 0.264 0.571 0.464 0.123 0.861 0.375 0.352 0.221 0.02
