# Supplementary material for: Transcriptome Analysis of Zebrafish Embryogenesis Using Microarrays
Source: PLoS Genet. 2005 Aug 26;1(2):e29. doi: 10.1371/journal.pgen.0010029 (PMC1193535; doi:10.1371/journal.pgen.0010029)
Supplement: Dataset S11 — (88 KB DOC) [file pgen.0010029.sd011.doc]

Dataset S11. List of genes with onset of transcript accumulation at the segmentation stage and peak of expression at pharyngula stages.

Genbank IDUF egg 3hpf 4.5hpf 6hpf 7.7hpf 9hpf 10.7hpf 12hpf 15hpf 24hpf 30hpf 48hpf

AA606085 -0.434 -0.477 0.323 0.384 0.229 0.079 0.359 0.872 0.364 0.994 0.275 0.351

AA658796 0.349 -0.742 -0.667 0.168 0.518 1.107 1.077 1.529 0.863 1.476 0.724 0.412

AF012747 -0.886 -2.264 -1.705 -2.215 -0.854 -0.339 -0.772 -0.733 -0.48 0.647 0.486 0.219

AF029250 -0.548 -0.766 -1.42 -1.819 -2.053 -0.817 -1.346 -0.756 -0.544 1.261 1.026 1.119

AF064835 -3.391 -2.993 -0.805 -0.603 -0.7 -1.293 -1.726 -0.682 0.318 0.534 0.119 -0.17

AF068773 -1.865 -1.741 -1.225 -0.055 -0.325 -0.073 0.072 0.165 0.198 1.167 0.202 -0.258

AF102865 -2.487 -2.252 -1.72 -1.622 -1.635 -1.096 -0.397 -0.975 -1.315 1.083 0.289 0.515

AF127981 -0.958 -0.046 -0.805 -0.918 -0.366 0.211 -0.069 0.373 -0.399 1.024 1.016 0.868

AF149802 -2.361 -2.012 -2.327 -1.351 -2.433 -0.921 -1.253 -1.481 -0.171 1.679 1.269 0.639

AF150107 0.675 0.144 0.212 0.564 0.589 0.839 0.138 0.926 0.075 0.919 0.297 -0.073

AF155581 0.429 -0.426 0.606 -0.316 1.309 1.134 0.563 1.773 0.732 1.564 0.632 0.468

AF157560 -0.912 -0.376 -0.576 -0.04 -0.673 -0.25 0.118 -0.272 -0.12 0.869 0.852 0.419

AF175294 -1.885 -0.783 -1.672 -0.669 -0.841 -0.191 0.083 -0.462 0.345 0.961 0.941 -0.081

AF191559 -1.181 -2.258 -1.867 -1.843 -0.516 -0.426 -0.489 -0.469 -0.786 0.572 0.508 0.358

AF202639 -0.613 -0.003 -0.32 -0.664 -0.238 0.185 -0.287 0.175 0.472 1.872 1.163 0.51

AF210641 -0.601 -1.358 -0.484 -0.13 -0.566 0.468 0.042 0.312 0.07 0.852 0.697 0.087

AF250368 -0.451 -1.192 -1.136 -0.614 -0.451 0.162 -0.055 0.562 0 0.793 0.522 0.24

AF315945 -0.717 -1.069 -1.43 -1.13 -0.521 -0.314 0.202 0.504 0.169 0.919 0.605 0.569

AI106133 -0.516 -1.723 -0.777 -0.373 -0.788 0.497 0.273 0.7 0.296 1.187 0.581 -0.44

AI331055 -0.388 -0.482 -0.354 -0.336 -0.383 0.581 -0.022 0.659 0.334 0.907 0.43 0.36

AI353394 0.508 -0.983 -0.048 0.133 0.391 1.198 0.557 1.057 0.416 1.295 0.863 1.017

AI397462 -0.728 -0.201 -0.552 -0.216 -0.161 -0.137 -0.366 0.039 0.646 1.536 0.641 0.034

AI444184 -0.169 -0.222 -0.361 0.482 0.13 -0.029 -0.183 0.654 0.085 0.587 0.325 0.246

AI444293 -0.397 -0.351 0.39 0.397 -0.069 0.651 0.29 0.393 0.312 0.742 0.345 0.453

AI477274 -2.032 -1.834 -2.202 -0.937 -2.806 -0.883 -0.898 -1.154 -0.095 1.615 0.624 0.823

AI477493 0.18 -0.213 0.028 0.063 0.196 0.419 0.802 1.331 0.691 1.281 0.609 0.005

AI522421 -0.979 -0.025 0.021 -0.384 0.01 -0.129 -0.526 -0.562 -0.337 1.421 0.496 -0.591

AI522700 -0.056 -0.832 -0.606 -0.434 -0.675 0.069 0.099 -0.085 0.2 0.637 -0.195 -0.006

AI545318 -0.295 -0.544 -0.651 0.093 0.443 0.915 0.425 0.979 0.319 0.901 0.372 0.478

AI584292 0.196 -0.045 0.188 0.406 0.206 0.356 0.495 0.752 0.131 0.798 0.459 0.165

AI588172 0.325 -0.006 -0.097 0.163 -0.616 0.172 -0.283 0.27 0.291 1.015 0.589 0.091

AI588301 -0.394 -0.098 -0.49 -0.059 -0.337 -0.236 0.004 0.458 0.122 0.867 0.261 -0.124

AI588515 -0.859 -0.582 -0.687 -0.003 -0.214 0.456 0.087 0.188 0.084 0.741 0.391 0.265

AI601365 -2.036 -1.302 -1.991 -2.172 -1.735 -0.879 -1.403 -1.276 -0.216 1.467 1.012 0.26

AI601390 -1.251 -1.719 -0.529 0.223 0.208 0.239 0.559 0.558 0.138 0.709 0.501 0.545

AI601781 -2.101 -1.46 -2.149 -0.736 -2.274 -0.679 -1.625 -1.358 -0.714 1.746 1.367 0.135

AI626645 -1.82 -1.646 -2.22 -1.588 -1.941 -0.231 -1.139 -1.811 -0.93 0.483 0.18 -0.159

AI626686 -0.387 -0.136 -0.525 0.087 0.303 0.121 -0.039 0.424 0.236 0.565 -0.05 0.033

AI641624 -0.047 -0.483 -0.436 -0.194 0.009 0.179 0.435 0.536 0.209 0.647 0.122 0.405

AI641664 -1.452 -1.617 -2.646 -2.268 -2.03 -1.118 -1.655 -1.529 -1.812 0.849 0.719 0.356

AI657670 -0.458 -0.325 -0.061 -0.169 -0.727 -0.039 -0.808 -0.158 -0.169 1.113 0.607 -0.049

AI658017 0.117 -0.431 -0.108 -0.129 0.069 0.093 -0.129 0.543 0.42 0.86 0.51 0.251

AI721335 -0.232 -0.286 -0.267 0.164 0.387 0.568 0.379 0.529 0.173 0.546 0.132 -0.223

AI722432 -1.451 -0.382 -1.165 -0.655 -0.826 -0.443 -0.396 -0.681 -0.318 0.792 0.012 -0.404

AI722464 -0.906 -1.086 -1.397 -1.71 -1.401 -0.259 -0.757 -1.252 -0.156 1.04 0.971 0.994

AI722592 -0.538 -1.219 -0.539 -0.498 -0.266 0.573 0.348 0.444 0.251 1.137 0.826 0.239

AI793454 0.05 -0.188 -0.35 0.145 -0.07 -0.274 -0.045 0.15 0.174 0.647 0.337 -0.202

AI793467 -0.764 -1.463 -1.138 -1.435 -2.131 -0.337 -1.379 -1.269 -0.665 1.122 0.881 0.275

AI793560 -0.718 -0.403 -0.25 -0.418 -0.504 -0.255 -0.192 -0.246 0.027 0.608 0.514 0.546

AI793761 -0.488 -0.489 -1.011 -0.203 -0.361 -0.341 -0.191 -0.448 -0.184 0.469 -0.059 0.175

AI793901 -2.554 -1.255 -2.495 -1.142 -2.193 -1.29 -1.81 -1.537 -0.729 0.894 0.707 0.66

AI878053 -0.255 -0.547 -0.895 -0.449 -0.651 0.165 -0.67 0.302 0.253 0.475 -0.17 0.126

AI878489 -0.756 -1.637 -2.01 -2.019 -0.727 -0.525 -0.8 -0.341 -1.445 0.644 0.245 -0.564

AI878761 -0.626 -0.932 -0.926 -0.819 -0.266 -0.094 -0.332 -0.207 -0.195 1.017 0.692 0.806

AI883262 -0.925 -0.179 -0.666 -0.121 -0.646 -0.25 -0.426 -0.169 0.01 1.172 0.89 0.371

AI883430 -1.335 -0.905 -1.353 -2.157 -1.205 -0.307 -0.838 -0.856 -0.694 2.693 1.963 -0.741

AI883679 -0.337 -0.382 -0.552 -0.278 -0.355 0.422 -0.127 0.393 0.132 0.905 0.493 0.389

AI884029 -0.462 -0.524 -1.05 -0.45 -0.463 0.08 -0.152 0.969 0.272 1.447 0.631 -0.498

AI957504 -0.998 -1.175 -1.491 -0.812 -1.036 -0.75 -0.903 -1.276 -0.798 0.965 0.435 -0.245

AI957746 -0.868 0.135 -0.267 0.302 0.006 -0.158 -0.472 -0.381 -0.08 1.375 0.733 -0.41

AI958585 0.108 -0.186 -0.773 -0.413 -0.211 0.974 0.61 0.998 0.358 1.049 0.399 0.229

AI965042 -0.077 -0.682 -0.696 -0.065 0.064 0.84 0.516 0.696 0.432 0.873 0.289 0.191

AI965225 0.385 0.1 0.366 0.443 0.177 0.728 0.128 0.679 0.557 0.91 0.227 0.078

AJ245490 -0.638 -0.866 -1.139 -1.351 -0.244 -0.328 -0.398 0.7 0.443 1.163 0.828 0.197

AJ278268 -0.13 -0.951 -0.309 -1.138 -0.687 0.184 -0.251 0.066 -0.223 0.425 -0.219 -0.561

AJ299411 -0.186 0.183 0.007 0.103 -0.685 -0.563 -0.478 -0.525 -0.5 0.896 0.641 -0.304

AW019276 -1.098 -0.992 -1.846 -1.07 -1.045 -0.521 -0.504 -0.084 -0.277 0.594 0.084 0.02

AW058797 0.257 -0.004 0.647 0.722 1.008 0.826 0.423 1.33 0.672 1.259 0.33 0.363

AW059046 -0.538 -0.237 0.072 0.478 0.647 0.491 0.194 0.961 0.279 0.83 0.348 0.21

AW059098 -0.006 -0.227 0.079 -0.481 -0.439 0.027 0.281 0.421 0.079 0.687 0.256 0.362

AW116780 -1.142 -0.48 -0.991 -0.811 -0.835 -0.716 -1.04 -1.141 -0.994 1.119 0.401 -0.205

AW116980 -0.046 -0.416 -0.388 -0.107 0.398 0.269 0.369 0.736 0.075 0.735 0.108 0.187

AW128244 -0.854 -0.754 -0.244 0.072 -0.318 0.052 0.358 0.277 0.225 0.888 0.697 0.756

AW133635 -0.73 -0.255 0.201 0.544 0.39 0.478 -0.221 0.878 0.458 0.709 0.123 -0.013

AW174544 0.217 -0.882 -0.416 -0.291 -0.411 0.522 0.033 0.571 0.138 0.804 0.596 0.276

AW202947 -1.019 -0.323 -1.246 -0.985 -1.012 -0.166 -0.531 -0.875 -0.295 0.927 0.532 0.569

AW232020 -2.569 -2.335 -3.261 -2.127 -2.903 -0.649 -1.642 -2.548 -0.871 1.088 0.764 0.95

AW232071 -0.086 -1.143 -0.733 -0.705 -0.77 -0.405 -0.111 0.256 -0.212 0.572 0.274 -0.082

AW233688 -0.357 -1.439 -1.201 -1.372 -1.137 -0.106 -0.315 -0.264 -0.241 0.754 0.344 0.581

AW420720 -1.617 -1.541 -1.361 -1.208 -1.811 -0.647 -0.423 -0.63 -0.126 0.734 0.584 -0.002

AW420883 -0.084 0.051 0.154 0.313 -0.145 0.392 -0.194 0.254 0.283 0.661 0.289 -0.389

AW567098 -0.6 -0.207 -0.045 0.895 1.161 1.016 0.767 1.139 0.346 1.237 0.84 0.235

AW777320 -1.1 -1.251 -2.176 -1.766 -2.285 -0.829 -1.21 -1.728 -0.461 1.47 0.849 1.216

AW777364 -0.072 -0.774 -0.934 -0.29 -0.193 0.462 0.318 0.754 0.335 0.843 0.082 0.333

AW777561 -0.751 0.344 -0.508 0.027 -0.076 -0.055 -0.357 0.476 0.626 1.305 0.412 -0.147

AW826723 -0.195 -1.229 -0.851 -0.38 -0.188 0.518 0.107 0.264 -0.079 0.545 0.275 0.48

BE201151 -0.659 -0.26 -0.538 -0.086 0.038 0.066 -0.003 0.817 0.371 1.208 0.578 -0.458

BE202178 -0.455 -0.141 0.193 0.763 0.568 0.544 0.551 0.972 0.549 1.096 0.624 0.693

BE557659 0.146 0.119 0.209 0.521 0.239 0.274 0.172 0.952 0.346 0.857 0.353 0.145

BE605522 0.614 0.081 -0.54 -0.227 -0.479 0.131 -0.091 -0.086 -0.272 0.638 0.193 0.04

BE693133 -0.053 -0.372 0.235 0.366 0.509 0.623 0.589 1.078 0.597 0.997 0.23 0.319

BE693153 0.499 -0.348 0.179 0.296 0.453 0.958 0.557 1.242 0.772 1.309 0.608 0.031

BF717503 -2.046 -1.241 -1.596 -0.88 -1.301 -0.747 -1.135 -0.731 -0.539 0.243 -0.099 -0.587

BG303237 -2.013 -2.195 -2.393 -1.643 -1.457 -1.21 -1.649 -1.447 -1.254 0.818 0.75 0.479

BG303602 0.27 -0.154 0.072 -0.094 -0.309 0.718 0.213 0.65 0.471 1.063 -0.012 -0.461

BG306111 -0.962 -2.195 -1.69 -1.341 -0.632 -0.114 0.142 0.428 -0.117 0.846 0.483 0.263

BG306420 -0.449 -0.369 -0.812 -0.335 -0.064 0.092 0.17 0.465 0.047 0.54 0.34 0.164

BG308628 -1.186 -1.349 -1.625 -0.659 -1.519 -0.454 -0.863 -1.605 -0.731 0.386 -0.061 -0.218

BG884107 -1.586 -0.292 -0.458 -0.057 0.501 1.295 0.719 0.735 0.428 1.33 0.827 1.131

BG985485 -0.443 -0.841 -1.389 -0.255 -0.177 -0.452 -0.386 -0.073 -0.252 0.287 -0.165 -0.094

BG985673 -1.717 -3.699 -2.59 -1.413 -2.097 -1.158 -0.793 -1.653 -0.601 0.891 0.598 0.537

BI318094 -0.654 -1.773 -1.007 -0.343 -0.466 0.407 0.197 0.292 0.131 0.953 0.497 0.204

BI430340 -0.091 -0.961 -0.802 -0.365 -0.002 0.605 0.326 0.69 0.242 0.893 0.566 0.626

BI672150 -0.368 -0.524 0.397 0.384 0.26 0.432 0.402 0.652 0.362 0.936 0.291 0.416

BI672301 -0.881 -0.728 0.197 0.229 0.258 0.355 0.448 0.755 0.374 0.944 0.543 0.096

BI706468 -0.031 -0.679 -0.415 -0.615 0.198 0.437 0.464 0.791 0.357 0.786 0.29 0.084

BI842851 -0.886 -1.071 -1.46 -0.259 -0.862 -0.323 0.065 0.128 -0.004 0.713 0.211 0.311

BI863998 -0.506 -0.04 -0.259 -0.149 -0.883 -0.452 -0.477 -0.69 -0.065 0.18 0.163 -0.643

BI877740 0.669 -0.11 -0.387 -0.156 -0.955 -0.327 -0.236 -0.097 0.079 0.92 0.146 -0.792

BI877998 0.245 -0.314 -0.33 0.244 -0.039 0.256 0.258 0.645 0.31 0.766 0.316 0.381

BI878456 -0.193 -0.718 -0.733 -0.473 -0.744 -0.133 0.192 0.166 0.158 0.964 0.56 0.556

BI879035 -0.256 -0.929 -0.814 -1.53 -1.314 -0.235 -0.583 -0.26 -0.465 0.596 0.47 -0.035

BI879038 -0.813 -0.971 -0.863 -0.06 0.584 1.263 0.756 1.253 0.583 1.237 0.858 0.725

BI880006 0.132 -0.488 -0.067 0.19 0.263 0.493 0.309 0.357 0.184 0.578 0.305 0.293

BI881430 -0.305 -1.265 -0.28 -0.387 -0.357 0.654 0.221 0.364 0.285 1.066 0.611 0.208

BI882313 -0.917 -1.832 -0.762 -1.084 -2.069 -0.466 -0.489 -1.611 -0.627 0.776 0.757 0.428

BI885026 -0.948 -0.04 -0.415 -0.659 -0.003 0.263 0.433 1.042 0.447 1.046 -0.079 -0.682

BI886290 0.084 -0.391 -0.381 0.344 0.325 0.611 0.379 0.925 0.429 1.142 0.638 0.25

BI886767 -0.125 -0.486 -0.32 -0.277 -0.25 0.138 -0.091 0.181 0.127 0.648 0.232 0.32

BI887526 0.21 -0.275 -0.29 -0.067 -0.098 0.431 -0.089 0.197 0.166 0.671 0.265 0.398

BI888729 -1.749 -1.295 -1.547 -0.988 -0.625 -0.261 -0.795 -0.395 0.143 0.386 0.058 -0.013

BI889621 -0.953 -0.862 0.165 0.352 0.615 0.428 0.005 0.555 0.081 0.935 0.438 0.832

BI889982 -0.03 -0.499 -0.452 -0.136 0.357 0.113 0.151 0.884 0.027 0.77 0.34 0.448

BI890954 -0.337 -0.396 -0.422 -0.401 -0.88 -0.252 -0.229 -0.076 0.085 0.9 0.815 0.653

BI891041 -0.203 -0.189 0.415 0.27 0.586 0.249 0.218 0.673 0.345 0.768 -0.054 -0.001

BI892272 -1.568 -1.744 0.544 1.253 1.566 1.288 1.045 0.142 1.384 1.615 0.99 1.425

BI896233 -1.099 -1.381 -1.669 -1.194 -1.558 -0.638 -1.261 -1.456 -0.61 1.129 1.034 0.809

BI897414 -0.304 0.048 -1.529 -0.56 -1.089 -0.538 -0.372 -0.447 -0.335 0.513 0.123 0.238

BI897492 0.265 0.189 0.567 0.529 0.333 0.648 0.396 0.855 0.599 1.038 0.562 0.416

BI979883 0.493 0.208 0.8 0.961 0.405 0.757 0.58 1.203 0.784 1.162 0.668 0.254

BI982718 -0.198 -1.867 -0.564 -0.64 -0.364 0.475 0.295 0.742 0.324 1.369 1.053 0.211

BI983379 0.159 -1.579 -0.742 -0.456 -0.52 0.342 0.193 0.212 0.116 1.037 0.615 0.161

BM005469 -0.258 -0.587 -1.047 -0.043 0.128 0.295 0.038 0.637 0.098 0.766 0.496 0.333

BM023958 -0.612 -1.373 -1.264 -1.406 -0.673 -0.091 -0.37 -0.08 -0.425 0.561 0.106 0.318

BM036392 0.016 -0.904 -0.603 -0.225 -0.23 0.338 -0.104 0.197 -0.07 0.565 0.267 0.228

BM071290 -0.511 0.27 -0.122 -0.244 -0.725 -0.299 -0.344 0.321 0.262 0.939 0.862 0.359

BM071941 0.111 0.051 -0.476 0.014 0.091 1.016 0.491 0.75 0.388 0.882 0.348 -0.128

BM095392 0.094 0.029 -0.896 -0.481 -0.764 -0.064 -0.424 -0.148 -0.296 0.764 0.453 0.3

BM157063 -0.563 -1.812 -1.162 -0.545 -0.27 0.346 0.277 0.886 0.282 1.551 1.254 0.958

BM181646 0.413 0.477 0.041 0.066 -0.753 -0.066 -0.375 -0.337 0.003 0.853 0.697 -0.422

BM181817 -0.262 -0.242 0.426 0.53 0.386 1.329 0.468 0.735 0.642 1.276 0.831 0.189

BM182739 0.157 -0.057 0.003 -0.12 -0.103 0.009 0.251 0.442 0.288 0.817 -0.2 -0.078

BM184148 0.245 0.076 -0.151 -0.005 -0.435 0.494 -0.119 0.462 -0.244 0.502 -0.037 0.405

U66872 -0.714 0.442 -0.494 0.358 1.101 0.399 0.536 0.965 0.444 1.535 0.5 -0.017

AA494787 -0.261 -0.292 -0.099 -0.305 0.354 0.132 0.673 0.8 0.255 0.604 0.833 0.586

AA495157 0.133 -0.022 -0.092 0.069 -0.001 0.063 0.162 -0.271 -0.23 0.195 0.533 0.274

AA495418 -0.524 -0.54 -0.283 -1.292 -1.088 -0.06 -0.467 -0.934 -0.543 0.116 0.698 0.689

AA497144 -0.845 -0.103 -0.057 0.032 -0.285 -0.131 -0.579 -0.558 -0.31 0.891 0.921 0.352

AB055663 -0.925 -0.955 -1.396 -1.318 -1.08 -0.399 -0.805 -0.881 -0.579 0.124 0.827 0.529

AF014370 -0.64 0.102 -0.449 0.187 -0.097 -0.209 -0.259 0.01 0.055 0.556 0.741 0.083

AF030031 -1.495 -1.641 -1.553 -1.041 -0.655 -0.159 0.379 0.113 -0.139 0.331 0.749 0.613

AF036149 -0.543 -0.131 -0.273 -0.669 -0.066 0.044 0.297 1.182 0.359 0.991 1.196 0.855

AF083557 -0.778 -2.324 -0.694 -1.19 -1.518 -0.846 -0.526 -1.55 -0.692 0.32 0.35 -0.026

AF095639 -0.623 -0.384 -0.403 -0.285 -0.165 -0.238 -0.213 0.233 -0.09 0.92 1.242 -0.005

AF108819 -0.383 -0.261 0.243 0.665 0.508 0.7 0.375 0.695 0.281 0.493 0.818 0.623

AF190144 -0.978 -0.948 -1.346 -0.545 -1.109 -0.306 -0.448 -0.474 -0.263 0.269 1.169 0.445

AF375227 -1.038 -0.328 -0.643 -0.715 -0.69 -0.152 -0.372 -0.496 0.257 0.406 0.661 0.59

AF448057 -0.219 -0.907 -0.76 -0.383 -2.167 -0.98 -1.017 -2.255 -0.785 0.807 0.832 0.182

AI330805 -1.018 -1.527 -1.655 -1.051 -1.536 -0.675 -0.455 -0.947 -0.037 0.043 1.056 0.06

AI437329 -0.952 -1.467 -1.101 -0.624 -0.446 -0.484 -0.239 -1.054 -0.121 -0.015 0.469 0.3

AI477343 -0.045 0.01 -0.442 -1.177 -1.693 -0.553 -0.404 -0.747 -0.651 0.065 0.082 -0.226

AI522447 -1.414 -0.532 -1.15 -0.611 -0.559 0.454 0.529 1.046 0.344 1.242 1.29 1.055

AI545291 -0.977 -2.79 -0.623 -1.127 -2.039 -0.943 -0.852 -2.154 -1.263 0.641 0.65 0.223

AI545455 -0.45 -0.795 -0.018 -0.136 -0.437 -0.158 0.02 -0.447 -0.057 0.195 0.351 0.126

AI558352 -0.453 0.095 -0.352 -0.623 0.407 0.066 0.149 0.172 -0.026 0.425 0.511 -0.126

AI558547 -0.651 -1.56 -0.297 -0.069 0.232 -0.157 -0.193 -0.387 0.216 0.03 0.336 0.156

AI584554 -0.494 -0.907 -0.138 -0.466 0.161 -0.489 -0.052 -0.31 0.014 -0.079 0.365 0.339

AI584555 -1.235 -1.715 -1.292 -2.098 -2.281 -0.677 -1.338 -2.143 -0.862 0.47 0.826 -0.032

AI584977 -0.858 -0.978 -0.466 -1.324 -1.717 -0.394 -0.399 -1.434 -0.84 0.367 0.714 0.421

AI585020 -0.046 -0.362 -0.165 -0.035 0.212 0.324 0.453 0.303 0.227 0.232 0.523 0.114

AI588589 -0.379 -0.404 -0.413 -1.15 -1.002 -0.033 -0.06 -0.853 -0.094 0.473 0.913 0.602

AI601567 -3.082 -2.648 -0.939 -1.165 -0.426 -0.621 -0.905 -1.129 0.364 -0.312 0.473 0.26

AI601782 0.255 0.129 -0.327 -0.542 0.077 0.166 0.243 0.311 0.126 0.44 0.869 0.615

AI641626 -0.248 -0.233 -0.185 -0.638 -0.208 -0.238 0.251 0.668 0.133 0.478 0.807 0.214

AI641634 -1.56 -2.449 -0.737 -0.679 -0.167 -0.28 -0.223 -0.933 0.154 -0.354 0.311 0.292

AI657777 -0.662 -0.891 -0.183 -0.73 -0.205 0.076 0.02 0.622 0.124 0.746 1.055 0.396

AI721504 -0.653 -0.005 -0.296 -0.503 -0.058 0.011 -0.45 -0.221 0.107 0.829 1.237 0.601

AI793374 -0.007 -0.394 -0.129 -0.012 0.594 0.085 0.383 0.131 0.191 0.183 0.542 0.484

AI793533 -0.659 -1.727 -0.722 -1.231 -1.88 -0.436 -0.498 -1.366 -0.506 0.715 0.805 -0.056

AI793807 -0.603 -0.248 -0.274 0.38 0.596 -0.077 0.128 0.391 -0.148 0.696 0.817 0.62

AI878403 -1.743 -1.781 -0.48 -1.104 -1.099 -0.43 -0.448 -1.237 -0.066 0.603 0.814 0.576

AI884046 -0.793 -0.679 -0.35 -1.676 -0.971 -0.436 -0.219 -0.209 -0.368 0.279 0.854 0.686

AI943112 -0.436 0.018 0.181 -0.314 0.187 -0.013 -0.357 -0.306 0.096 0.21 0.847 0.784

AI943183 -0.816 -0.564 -0.752 -0.732 -0.457 -0.094 -0.342 0.091 0.558 0.486 1.172 0.844

AI957739 -0.583 -0.103 0.01 -0.311 0.009 0.114 -0.054 0.299 -0.168 0.278 0.778 0.128

AI957909 -0.01 -0.919 0.124 -0.129 -1.073 -0.205 -0.171 -0.804 -0.431 0.169 0.423 0.232

AI959515 -0.553 -0.111 -0.309 -0.501 -0.131 0.301 -0.197 -0.734 -0.382 0.148 0.675 -0.098

AI965247 0.582 -0.198 -0.252 -0.683 -1.504 0.115 -0.041 0.044 -0.2 0.384 0.695 -0.128

AI965312 -0.653 -0.311 0.166 -1.399 -0.769 -0.381 -0.562 -0.425 -0.181 0.539 0.838 0.764

AJ011112 -0.569 0.11 -0.195 -0.071 0.098 0.01 0.079 0.49 -0.141 0.357 0.723 -0.1

AJ278244 0.02 -0.043 0.438 0.458 0.774 0.423 -0.084 0.201 0.756 0.786 0.87 0.863

AW076688 -0.571 -0.687 -0.539 -1.223 -1.573 -0.367 -0.2 -1.827 -0.571 0.379 0.994 0.633

AW077448 0.099 -0.101 0.247 0.454 0.277 0.451 0.318 0.55 -0.153 0.469 0.641 0.472

AW115640 -0.658 -0.291 0.162 -0.037 0.458 0.309 -0.187 -0.117 0.224 0.144 0.418 -0.09

AW117039 0.662 0.307 0.668 0.286 0.739 0.316 0.406 -0.015 0.353 0.789 0.791 0.787

AW117094 -1.936 -2.566 -1.102 -0.582 -0.472 -0.16 -0.121 -0.03 -0.515 0.28 0.393 -0.334

AW171072 -0.984 -0.475 0.038 -0.319 -0.201 -0.067 0.097 -0.46 0.334 -0.065 0.411 0.312

AW232425 -0.013 0.166 0.371 0.071 0.097 -0.448 -0.176 -0.23 -0.195 0.307 0.496 0.069

AW232844 -3.529 -2.511 -1.069 -1.098 -0.558 -0.873 -1.008 -1.582 0.108 -0.785 0.187 -0.003

AW279837 -2.401 -2.601 -0.686 -0.867 -0.009 -0.09 -0.67 -1.003 0.346 -0.393 0.46 0.3

AW421096 -0.622 -0.134 0.036 -0.583 -1.01 -0.265 -0.247 -0.267 -0.284 0.034 0.753 0.321

AW567292 -0.627 0.157 -0.008 0.259 0.294 0.015 -0.209 0.375 0.101 0.795 0.96 0.636

AW777326 -0.408 0.122 -0.085 0.353 0.157 0.304 -0.126 0.36 0.004 0.298 0.443 0.049

AW826646 -0.423 -1.077 -0.193 -0.166 0.365 -0.258 -0.061 -0.347 0.277 0.253 0.522 0.262

AY029402 -0.471 -0.761 0.617 0.669 -0.418 -0.265 0.076 -0.945 -0.242 0.281 0.747 0.537

BE605276 -0.11 0.188 0.016 0.019 -0.366 -0.212 0.014 0.648 -0.062 0.448 0.734 0.191

BE693132 -0.792 -0.118 -0.14 -0.855 -0.627 -0.163 -0.362 -0.469 -0.434 0.19 0.884 0.174

BE693143 -0.197 -0.392 0.544 0.01 0.49 0.246 0.29 0.071 -0.237 0.127 0.492 0.277

BG303217 -0.767 -0.118 -0.081 -0.202 -0.221 0.123 -0.049 -0.236 -0.015 0.081 0.727 -0.099

BG305301 -1.243 -1.257 -0.695 -0.462 0.228 0.177 -0.279 -0.201 0.526 0.008 0.61 0.39

BG727484 -1.565 -0.908 -0.623 -0.482 0.297 -0.261 -0.487 -0.618 0.426 -0.158 0.582 0.571

BG985680 -0.446 -0.434 1.023 1.189 0.533 0.861 0.561 0.786 0.649 1.24 1.329 0.321

BG985742 -0.728 0.42 0.27 -0.369 -0.645 -0.317 -0.478 -0.054 -0.406 0.157 0.724 0.08

BI428149 -0.685 -0.785 -0.169 -0.271 -0.255 -0.187 -0.02 -0.893 -0.486 -0.058 0.475 0.393

BI534277 0.08 -0.005 0.001 0.202 -0.018 0.09 -0.01 -0.325 -0.048 0.061 0.449 0.306

BI705531 0.451 -0.448 -0.185 0.026 -0.271 -0.12 -0.293 -0.11 -0.002 0.402 0.639 -0.018

BI705843 -1.068 -0.873 -1.079 -0.68 -0.974 -0.614 -0.302 -1.079 -0.707 0.545 0.944 0.482

BI866952 0.454 0.427 0.565 0.435 -0.452 -0.457 -0.39 -0.476 -0.127 0.738 0.87 -0.239

BI883230 -0.817 0.011 -0.315 -0.783 -0.326 -0.12 -0.079 0.128 -0.102 0.368 0.704 -0.102

BI886648 -0.401 -0.331 0.122 0.294 0.713 -0.234 0.319 0.946 0.28 0.825 1.742 0.166

BI891855 -1.777 -2.561 -0.856 -0.943 -0.315 -0.441 -0.043 -1.211 0.222 -0.253 0.396 0.13

BI892407 -0.244 -0.259 -0.318 0.121 0.103 0.125 -0.058 0.435 0.101 0.469 0.588 0.103

BI980180 -0.167 0.156 0.041 -0.825 -0.706 -0.247 -0.331 -0.407 -0.323 0.325 0.845 0.402

BM026015 -1.111 -0.52 0.31 0.312 0.711 -0.183 -0.431 -0.171 0.773 0.401 1.282 0.924

BM026841 -0.407 -0.573 -0.384 -0.572 0.089 -0.034 -0.002 -0.297 0.392 0.518 0.694 0.324

BM154820 -0.595 -0.851 -0.908 -0.556 0.055 -0.347 -0.008 -0.676 0.069 -0.104 0.518 0.206

BM155230 -0.102 -0.069 -0.161 -0.579 -0.022 -0.031 0.076 -0.401 0.083 0.139 0.542 0.137

BM181750 -1.133 -2.179 -0.998 -0.665 -1.136 -0.425 -0.232 -0.612 -0.346 0.801 0.876 0.184

BM182744 -0.595 -1.724 -0.008 -0.143 -1.112 -0.347 -0.046 -0.802 -0.112 0.943 0.97 0.147

BM183152 -0.646 -0.811 0.948 1.471 1.276 1.907 1.164 1.278 0.508 1.604 1.859 0.663

BM187382 -1.106 -0.907 -1.01 -0.681 -0.467 -0.343 -0.513 -0.617 0.03 -0.186 0.445 0.311

D13045 -0.895 -1.574 -0.982 -0.75 -1.018 -0.474 -0.353 -1.007 -0.421 0.185 0.882 0.48

U14587 0.126 -0.451 -0.485 -0.458 -0.64 -0.269 0.183 -0.499 -0.163 0.086 0.982 0.524

U14590 0.002 -0.289 0.077 -0.093 -0.087 -0.197 -0.069 -0.101 -0.167 0.452 0.902 0.797

U49407 -0.582 -0.107 0.132 -0.301 -0.272 0.045 -0.1 -0.114 0.117 0.185 0.522 0.23

U57973 -0.84 -0.793 -0.944 -0.276 -0.396 -0.097 -0.458 -0.596 0.532 -0.018 0.611 0.074

X87750 -0.43 0.015 0.059 0.059 0.348 -0.012 -0.17 0.551 0.128 0.349 0.559 0.397

X96422 -0.427 -1.564 -0.962 -1.61 -1.825 -0.285 -0.648 -1.446 -0.39 0.075 0.886 0.743

X97332 -1.003 -1.133 -0.652 -0.4 -1.48 -0.429 -0.069 0.154 -0.08 1.053 1.263 1.022

Y07905 -0.702 -0.446 -0.365 -0.273 -0.697 -0.017 -0.574 -0.409 0.247 0.377 0.922 0.652

AA494746 -0.547 -0.364 -0.35 0.21 -0.024 0.01 -0.004 -0.209 -0.105 0.118 0.506 0.689

AA495154 0.016 -0.528 0.061 0.713 0.944 0.488 0.231 0.589 0.071 0.145 0.559 1.024

AA497336 -0.276 -0.342 -0.592 -0.083 -1.184 -0.383 -0.12 -0.661 -0.015 0.288 0.33 0.528

AA606054 -2.172 -2.391 -1.001 -1.194 -0.848 -0.495 -0.896 -0.977 -0.583 -0.175 -0.007 0.401

AA606080 -0.476 -0.352 -0.474 -0.089 -0.325 -0.001 -0.225 -0.467 -0.452 0.398 0.713 1.42

AF006831 -0.796 -0.238 -0.671 -0.2 -0.276 -0.376 -0.572 -0.094 -0.303 0.251 0.416 0.668

AF047837 -4.534 -3.707 -3.381 -3.489 -3.716 -2.618 -3.104 -3.535 -2.718 -1.352 -0.351 0.577

AF082662 -4.932 -5.147 -4.876 -4.098 -4.939 -3.778 -3.013 -4.62 -4.127 0.208 0.905 2.008

AF121796 -2.395 -1.666 -2.489 -2.316 -1.773 -1.2 -1.064 -1.928 -2.096 -0.057 0.326 1.466

AF180889 -2.938 -2.542 -1.676 -0.694 -3.392 -2.131 -1.994 -2.811 -2.393 -0.201 0.778 1.283

AF180890 -2.656 -6.312 -1.701 -4.795 -5.838 -2.243 -2.459 -5.819 -2.443 -0.401 -0.283 0.891

AF180892 -6.37 -6.683 -5.006 -5.412 -6.568 -5.087 -6.354 -6.553 -4.62 -1.118 -0.464 0.707

AF204240 -0.779 -0.236 -0.154 -0.964 -0.678 -0.392 -0.489 -0.447 -0.36 0.017 0.903 1.621

AF237712 -1.784 -1.984 -1.481 -0.544 -1.188 -0.671 -0.61 -1.588 -1.102 0.066 0.255 1.869

AF280090 -0.907 -0.473 -0.864 -0.522 -0.757 -0.287 -0.747 -0.727 -0.202 0.24 1.04 1.187

AF281003 -2.203 -2.458 -2.203 -1.488 -2.283 -1.568 -1.738 -1.873 -1.876 -0.125 0.271 0.602

AF286374 -0.968 0.166 -0.691 -0.765 -0.405 -0.017 -0.389 -0.167 -0.868 0.674 0.845 0.986

AF321194 -0.879 -0.611 -0.648 -0.711 -0.925 -0.125 -0.312 -0.412 -0.204 0.332 0.861 1.31

AF359429 -0.643 0.096 -0.074 0.006 -0.031 0.259 -0.257 0.716 0.699 0.642 0.552 0.787

AF364083 -0.811 -0.549 -0.023 -0.339 -0.923 -0.125 -0.656 -0.812 -0.121 -0.138 0.515 0.927

AF395831 -0.942 -0.211 -0.385 -0.621 -0.059 -0.037 -0.402 -0.283 -0.291 0.076 0.238 0.848

AI353083 -4.956 -4.135 -4.296 -2.727 -4.102 -3.193 -3.663 -4.553 -3.654 0.35 1.008 2.248

AI384393 -1.225 0.136 -0.716 -0.388 -1.029 0.07 -0.496 -0.771 -0.101 0.493 0.426 0.739

AI384655 -1.691 -1.564 -1.034 -0.488 -0.433 -0.418 -0.532 -1.088 0.038 -0.538 0.253 0.463

AI397274 -0.373 -0.746 -1.164 -0.981 -0.778 -0.371 -0.683 -0.687 -0.447 0.54 0.617 1.327

AI461323 -0.974 -0.223 -0.573 -0.678 -0.395 -0.034 -0.307 -0.07 0.076 0.576 0.698 0.728

AI477656 -2.837 -2.541 -2.726 -2.208 -2.339 -1.241 -1.199 -2.91 -1.675 -0.838 0.67 1.67

AI584352 -1.853 -2.896 -1.132 -0.809 -0.347 -0.2 -0.326 -1.229 0.245 -0.334 0.315 0.532

AI601488 -0.921 -1.14 -0.198 0.256 0.441 0.266 -0.006 0.479 0.122 0.613 0.471 0.957

AI601664 -3.851 -5.142 -4.7 -4.147 -4.55 -2.514 -2.505 -3.943 -3.246 -1.096 0.785 1.543

AI601714 -2.571 -1.432 -1.49 -0.935 -1.685 -0.957 -0.92 -1.683 -0.705 0.631 0.65 0.957

AI618133 -4.183 -6.661 -3.612 -4.65 -5.34 -3.359 -3.884 -5.997 -3.872 -3.063 -2.339 0.703

AI626348 -0.35 -0.718 -0.735 -0.837 -1.027 -0.29 -0.182 -1.088 -0.446 -0.223 0.291 0.518

AI626603 -3.014 -3.394 -1.296 -1.237 -0.6 -0.834 -0.941 -1.553 0.368 -0.767 0.39 0.412

AI641414 0.025 -0.377 -0.269 -0.221 -0.269 0.144 -0.03 -0.048 0.388 0.2 0.423 0.905

AI641772 -0.666 -0.093 -0.096 0.135 0.015 -0.378 0.026 -0.379 -0.113 0.283 0.348 1.126

AI658011 -2.105 -2.831 -2.338 -2.415 -2.821 -1.4 -1.731 -2.48 -1.406 1.029 0.832 1.501

AI667071 -0.681 -0.216 -0.191 -1.076 -0.968 -0.206 -0.061 -0.242 -0.248 0.303 0.625 1.088

AI721531 -0.209 -1.324 -0.461 -0.812 -1.284 -0.48 -0.043 -0.98 -0.255 0.389 0.465 1.205

AI722369 -1.634 -4.058 -1.963 -2.107 -2.634 -1.155 -1.471 -3.924 -1.597 -0.916 0.582 1.915

AI793424 -1.891 -1.113 -1.885 0.01 -1.501 -0.571 -0.502 -1.886 -1.307 0.388 0.528 1.185

AI793897 -0.2 -0.537 -0.48 0.064 -0.934 -0.058 -0.043 0.046 0.15 0.026 0.281 0.757

AI794518 -1.509 -1.481 -3.206 -1.149 -2.079 -0.902 -1.031 -1.601 -0.638 0.222 0.241 1.5

AI878392 -0.689 -0.979 -0.56 -0.257 -0.584 -0.101 -0.597 -0.446 -0.289 0.065 0.252 0.787

AI943132 -0.19 -0.16 -0.053 -0.101 -0.547 -0.228 -0.233 -0.734 -0.286 0.11 0.31 0.873

AI957596 0.25 -0.803 -1.084 -1.247 -1.395 -0.251 -0.16 -0.226 -0.268 0.104 0.209 0.808

AI958567 -2.103 -1.031 -2.17 -0.444 -1.285 -0.708 -1.107 -1.642 -1.779 0.84 0.382 1.396

AI959722 0.539 0.234 -0.142 0.134 0.114 0.409 -0.036 0.148 0.35 0.508 0.67 0.71

AI964274 -1.162 -0.725 -1.032 -0.951 -0.804 -0.254 -0.058 -0.741 -0.329 0.677 0.475 0.684

AI964276 -4.704 -4.884 -3.941 -3.54 -3.911 -2.741 -3.768 -5.221 -3.738 -1.064 0.367 2.267

AI965047 -2.765 -3.779 -2.306 -1.728 -3.807 -2.588 -1.639 -3.865 -1.881 -0.582 0.743 1.421

AI965224 -0.778 0.545 0.045 0.341 0.173 -0.046 -0.563 -0.008 0.011 0.853 1.139 1.169

AJ245962 -0.519 -0.315 -0.206 -0.361 -0.723 -0.113 -0.543 -0.681 -0.25 -0.084 0.272 1.063

AJ311846 -3.017 -1.827 -2.235 -1.521 -1.92 -1.408 -2.209 -1.976 -2.409 0.424 1.34 1.516

AJ404970 -0.461 0.545 0.15 0.341 -0.173 0.253 -0.098 0.604 -0.008 0.108 0.582 0.946

AW018635 -2.344 -2.123 -1.911 -2.042 -2.15 -1.245 -1.531 -0.845 -0.3 0.248 0.501 1.415

AW019275 -1.221 -2.211 -0.596 -0.737 -0.188 -0.437 -0.242 -0.974 0.297 -0.293 0.013 0.428

AW077420 -0.42 -0.177 -0.273 -0.683 -0.373 -0.149 -0.491 -0.506 -0.078 0.239 0.413 0.808

AW116863 -3.941 -2.372 -2.632 -1.828 -3.077 -2.975 -2.703 -2.872 -1.808 -0.528 -0.293 0.644

AW128379 -1.503 -0.336 -1.991 -0.74 -2.033 -0.576 -0.909 -1.605 -1.566 0.848 0.675 0.909

AW154726 -0.988 -0.38 -0.295 0.149 -0.383 -0.174 -0.205 -0.497 -0.316 -0.058 0.01 0.584

AW171479 -1.391 -1.518 -1.069 -0.516 -1.096 -0.507 -1.242 -0.989 -0.469 0.222 0.383 0.646

AW175546 -0.601 -0.1 0.308 0.322 0.043 0.238 -0.071 0.579 0.133 0.223 0.705 0.747

AW281249 -0.963 -0.636 -0.621 -1.172 -1.456 -0.465 -0.548 -1.574 -0.562 0.528 0.576 0.804

AW281293 -0.893 -1.451 -0.856 -0.712 -0.959 -0.325 -0.183 -1.134 -0.451 -0.204 -0.102 0.77

AW282014 -1.081 -0.44 -0.941 0.066 -0.202 -0.052 -0.596 -0.253 -0.868 -0.098 0.113 1.25

AW305840 -0.491 -0.356 -0.932 -1.162 -0.869 -0.409 -0.393 -0.64 -0.391 0.036 0.335 1.135

AW419481 -0.558 0.018 -0.305 0.14 0.531 0.517 0.27 0.809 0.191 0.518 0.573 0.774

AW420849 -0.32 -0.158 -0.423 0.024 0.067 0.432 0.146 0.048 0.444 0.381 0.334 0.595

AW827044 -0.213 -0.031 0.221 0.013 -0.746 -0.221 -0.145 -0.707 -0.084 0.186 0.261 0.754

BE201395 -2.225 -4.034 -2.193 -3.84 -4.285 -1.064 -1.929 -4.284 -2.203 -0.365 0.497 2.327

BE693123 0.246 -0.376 -0.212 -0.541 -0.829 0.232 0.067 -0.412 -0.151 0.642 0.647 0.791

BE693186 -2.7 -4.376 -4.107 -3.132 -4.54 -2.778 -3.48 -5.365 -3.512 0.504 1.16 2.194

BE693204 -1.552 -2.278 -2.064 -1.637 -2.103 -1.823 -1.123 -2.236 -1.454 -0.46 0.754 1.422

BF717383 -0.933 -1.16 -0.635 -0.832 -2.045 -0.734 -0.438 -1.136 -0.477 0.084 0.285 1.197

BF717548 -2.75 -1.849 -2.551 -0.542 -0.94 -0.673 -1.024 -1.216 -0.918 0.662 0.408 0.872

BG302583 0.4 0.208 0.14 0.019 -0.576 -0.224 -0.613 -0.456 -0.083 0.17 0.4 0.658

BG302934 -0.955 -1.085 -1.254 -0.422 -1.665 -0.474 -0.545 -1.982 -0.751 -0.079 0.684 0.728

BG305033 -0.458 0.132 -0.08 0.328 -0.083 0.015 -0.397 -0.14 0.039 0.284 0.601 0.997

BG305295 0.508 0.013 -0.603 -0.323 -0.219 -0.084 -0.264 -0.278 -0.321 0.045 0.124 0.721

BG306150 -1.028 -1.311 -0.669 -0.556 -0.985 -0.761 -0.453 -1.686 -1.026 -0.434 0.147 1.506

BG308501 -0.356 -0.736 -0.215 -0.078 -0.298 0.516 0.567 0.398 0.5 0.369 0.434 0.875

BG308524 -0.524 -0.841 -0.08 -0.221 -1.358 -0.907 -0.189 -0.763 -0.325 -0.058 0.348 1.366

BG883325 -1.649 -0.661 -1.583 -1.699 -1.948 -0.215 -1.175 -1.512 -1.097 -0.482 0.275 1.626

BG985507 -1.117 -1.324 -1.357 -0.779 -2.544 -0.533 -0.769 -1.615 -0.253 0.663 0.774 1.403

BG985722 -1.335 -0.4 -1.024 -1.283 -1.091 -0.341 -0.809 -0.505 -0.018 1.117 1.059 1.44

BI325077 -1.956 -1.575 -2.353 -1.153 -1.71 -1.106 -1.498 -1.321 -0.062 0.313 0.604 1.321

BI326643 -0.901 -1.378 -1.043 -0.745 -0.008 -0.345 -0.328 -0.76 0.061 -0.278 0.357 0.379

BI430050 -0.865 -0.895 -0.432 -0.234 -1.125 -0.483 -0.184 -1.048 -0.102 0.079 0.253 0.535

BI430334 0.361 -0.492 -0.661 -0.096 -0.719 -0.109 -0.02 -0.694 -0.443 -0.131 0.05 0.753

BI474953 0.452 0.064 -0.158 -0.053 -0.034 0.154 -0.036 -0.068 0.262 0.683 0.532 0.699

BI533161 -0.951 -2.53 -1.153 -1.991 -2.07 -0.193 -1.051 -2.653 -1.072 -0.066 0.536 1.081

BI670932 -1.039 -1.085 -1.016 -0.629 -0.621 -0.544 -0.571 -0.696 -0.721 -0.136 -0.13 0.518

BI672022 -1.198 -1.693 -2.077 -2.443 -2.278 -1.256 -1.318 -1.925 -1.458 0.387 0.402 1.391

BI704324 -2.499 -3.043 -2.506 -2.699 -2.57 -1.229 -1.429 -3.134 -1.655 -0.749 0.615 1.315

BI839927 -1.594 -1.453 -2.144 -1.338 -1.4 -0.594 -1.089 -1.931 -1.114 -0.407 0.065 1.837

BI845367 -0.734 -0.238 -0.375 -0.398 -0.243 -0.157 -0.183 -0.676 -0.332 -0.137 0.32 1.005

BI846231 -1.586 -2.519 -1.313 -2.333 -2.094 -0.479 -1.142 -2.751 -1.55 -0.96 -0.386 0.686

BI864920 -0.704 -1.545 -1.307 -0.933 -1.6 -0.64 -0.762 -1.528 -0.211 -0.748 -0.089 0.896

BI865477 -0.766 -0.882 -1.301 -1.439 -0.642 -1.073 -0.96 -1.381 -0.722 0.236 0.8 1.444

BI865912 -0.728 -0.334 -0.036 -0.89 -0.972 -0.522 -0.312 -0.932 -0.284 -0.075 0.796 1.537

BI878304 -0.728 0.321 0.001 -0.122 -0.107 -0.135 -0.243 0.193 -0.336 0.27 0.107 0.707

BI879589 -0.925 -0.847 -1.692 -1.046 -1.503 -0.277 -0.797 -0.765 0.367 -0.177 0.659 1.18

BI879661 -0.879 -3.098 -1.445 -1.712 -3.558 -0.932 -0.982 -2.772 -0.837 0.568 0.61 1.063

BI879868 0.52 -0.239 0.246 0.186 -0.077 0.27 0.467 -0.127 0.363 0.193 0.479 0.746

BI880074 -2.049 -1.205 -1.944 -0.5 -1.529 -0.49 -1.399 -1.693 -1.043 0.456 0.495 1.076

BI882649 -0.302 -0.942 0.173 -1.098 -0.869 -0.356 0.048 -1.126 -0.669 -0.114 0.618 1.476

BI886259 -0.472 -0.698 0.159 0.519 0.573 -0.188 0.259 0.554 0.059 0.363 0.775 0.847

BI886388 -1.219 -0.599 -0.253 0.177 0.745 1.142 1.019 1.107 0.36 1.232 0.98 1.37

BI890462 -0.09 -0.339 0.336 0.513 0.607 0.16 0.243 0.814 0.206 0.632 0.614 1.065

BI892299 -0.595 0.144 0.455 0.485 0.705 0.341 -0.026 0.397 0.506 0.429 0.67 0.686

BI981066 -0.838 -0.802 -0.963 -0.398 0.004 -0.106 -0.461 -0.511 -0.206 -0.247 0.519 0.597

BI982778 -1.391 -0.567 0.017 -1.212 -1.817 0.09 -1.219 -1.575 -0.847 0.299 0.768 1.895

BI982951 -0.739 -1.111 -0.897 -0.904 -1.172 -0.62 -0.674 -1.239 -0.474 -0.269 -0.019 0.623

BM026607 -0.296 -0.288 -0.976 -1.154 -1.704 -0.211 0.151 -0.756 -0.819 0.199 0.097 1.465

BM070919 -0.585 -0.933 -0.588 -0.311 -0.646 -0.466 -0.338 -0.795 -0.292 0.041 0.098 0.752

BM071225 -5.191 -4.481 -4.423 -3.382 -3.575 -3.213 -4.224 -4.817 -3.741 0.638 0.744 0.749

BM102195 -0.505 0.222 0.397 0.105 -0.343 0.336 -0.151 -0.203 -0.138 0.246 0.318 0.789

BM183857 -1.962 -2.772 -2.052 -1.539 -1.674 -1.625 -1.748 -1.647 -1.685 -0.79 -0.158 1.103

BM186246 -1.733 -2.543 -1.219 -1.008 -0.969 -0.102 -1.35 -0.932 -0.876 -0.02 -0.173 0.497

BM186665 -1.055 -0.089 -0.201 -0.421 -0.421 -0.178 -0.489 -0.323 -0.705 -0.025 0.356 1.458

S80986 -2.376 -1.161 -1.912 -1.309 -2.06 -0.93 -1.115 -0.798 -0.318 0.82 0.636 0.982

U23822 -3.984 -3.574 -3.624 -2.803 -3.981 -2.283 -2.654 -3.118 -1.063 0.375 0.669 0.858

U62018 -1.289 -1.084 -1.565 -1.417 -1.375 -0.435 -0.259 -1.036 -0.228 0.554 0.629 1.284

U89710 -0.728 -0.019 -0.578 -0.259 -0.268 -0.125 -0.484 0.042 -0.785 0.013 0.625 1.005

X70299 -0.468 -0.75 -0.195 -0.379 -0.561 -0.024 -0.424 -0.514 0.069 0.37 0.697 0.937

X85977 -0.448 -0.042 0.179 -0.156 -0.138 -0.182 0.04 0.281 0.021 0.29 0.46 0.583

Mean -0.861 -0.941 -0.763 -0.609 -0.723 -0.229 -0.385 -0.482 -0.257 0.486 0.532 0.533
