# Supplementary material for: Transcriptome Analysis of Zebrafish Embryogenesis Using Microarrays
Source: PLoS Genet. 2005 Aug 26;1(2):e29. doi: 10.1371/journal.pgen.0010029 (PMC1193535; doi:10.1371/journal.pgen.0010029)
Supplement: Dataset S12 — (43 KB DOC) [file pgen.0010029.sd012.doc]

Dataset S12: List of genes with onset of transcript accumulation and peak of expression at the pharyngula stage.

Genbank IDUF egg 3hpf 4.5hpf 6hpf 7.7hpf 9hpf 10.7hpf 12hpf 15hpf 24hpf 30hpf 48hpf

AB006104 -0.392 -0.055 -0.35 -0.068 -0.099 0.188 -0.234 -0.017 0.366 0.107 1.15 0.477

AF030284 -0.087 -0.162 0.244 0.055 0.547 -0.212 0.109 0.9 0.052 0.24 0.989 0.358

AF060118 -0.761 -0.797 -0.555 -0.972 -0.633 0.103 -0.464 -0.201 0.066 -0.24 0.834 0.256

AF071245 -0.798 -0.426 -0.383 -0.262 -0.305 -0.04 -0.512 0.077 0.075 0.114 1.072 0.666

AF071267 -0.355 0.309 0.512 -0.422 -0.138 0.04 0.257 0.86 0.29 -0.05 1.139 0.185

AF272962 -0.451 -0.033 -0.153 -0.166 0.03 0.159 -0.578 0.657 0.541 0.215 0.862 0.415

AF277097 -0.608 -0.04 -0.143 -0.226 0.158 0.191 0.145 0.117 0.111 0.083 0.727 0.551

AF384863 -0.487 0.225 -0.084 0.125 0.457 0.09 0.264 0.336 -0.137 -0.064 0.729 -0.092

AI354033 -0.406 -0.046 -0.066 0.025 -0.083 -0.252 0.121 0.233 0.192 0.09 0.428 0.112

AI558311 -0.445 -0.226 -0.538 -0.23 0.206 0.221 0.047 0.532 0.148 -0.038 0.681 0.137

AI558512 -0.54 0.545 0.407 -0.128 0.105 0.056 -0.212 0.215 -0.097 -0.04 0.701 -0.153

AI584487 -1 -1.245 -0.437 -0.42 0.349 0.114 -0.035 0.329 0.571 0.046 0.602 0.421

AI601355 -0.728 -0.045 0.277 -0.754 -0.902 -0.532 0.118 0.815 0.399 -0.08 0.932 0.559

AI626598 -0.879 -0.234 0.05 0.065 0.115 -0.039 -0.164 -0.062 -0.096 -0.175 0.595 0.384

AI626799 -0.679 0.35 0.212 -0.308 -1.107 -0.284 -0.286 -0.373 -0.331 -0.14 1.113 0.552

AI667319 -0.359 -0.349 -0.775 -0.18 -0.332 -0.091 0.024 -0.306 -0.087 0.102 1.13 0.728

AI721741 -0.271 -0.258 -0.276 -0.824 -0.76 0.033 -0.221 0.458 0.36 0.122 1.137 0.62

AI722307 -0.287 0.545 0.407 -0.438 -0.025 -0.163 -0.193 -0.121 0.158 0.097 0.705 0.044

AI722354 -0.247 0.545 0.504 0.341 0.064 -0.205 0.051 0.193 0.048 -0.378 0.606 -0.082

AI722818 -0.093 -0.373 -0.099 -0.085 0.614 0.004 -0.028 -0.114 0.148 0.018 0.491 0.43

AI883799 -0.621 0.076 0.167 0.296 0.132 -0.19 0.019 -0.251 0.356 -0.045 0.406 0.234

AI884279 -0.233 -0.38 -0.17 -0.291 -0.034 -0.278 -0.008 -0.608 0.064 -0.054 0.528 -0.049

AI943036 -0.842 -0.064 0.05 -0.098 0.133 0.113 0.072 0.662 0.558 0.382 1.113 0.706

AW077237 -0.75 -0.075 -0.461 -0.083 -0.205 0.045 -0.234 0.189 0.167 0.051 0.636 0.364

AW171390 -0.351 0.054 -0.449 -0.241 -0.264 -0.116 -0.16 -0.477 -0.418 -0.196 0.462 0.403

AW203020 -0.119 0.073 0.2 0.359 0.478 0.52 0.101 0.371 0.217 0.18 0.603 0.296

AW203129 0.196 -0.695 -0.284 -0.071 -0.154 0.054 0.226 0.279 0.314 0.088 0.675 0.444

AW420737 0.303 0.139 0.179 0.448 0.063 0.297 0.425 0.282 0.482 0.174 0.499 0.482

AW777717 -0.26 0.367 0.522 -0.229 -0.13 0.487 0.178 0.824 -0.37 -0.414 0.754 0.271

AW826653 -0.593 -0.604 -0.399 -0.24 -0.109 -0.415 0.02 -0.455 0.282 -0.254 0.367 0.351

AW826907 -0.448 -1.272 -0.307 -0.741 -0.22 -0.241 -0.183 -1.127 0.158 -0.453 0.422 0.22

AY017309 -1.071 -1.343 -0.731 -0.465 0.184 -0.169 -0.201 -0.096 0.057 -0.183 0.656 0.583

BG307330 -0.184 0.053 0.096 -0.829 0.086 0.314 0.233 -0.219 -0.061 -0.314 0.655 0.207

BG883345 -0.691 0.545 -0.168 0.341 0.441 0.398 0.25 -0.057 0.046 0.005 0.794 0.512

BI429638 -0.186 -0.525 0.122 -0.168 0.107 -0.289 -0.305 0.611 0.509 0.259 0.619 0.119

BI708092 -0.788 -0.604 -0.383 -0.128 0.335 0.018 -0.092 0.278 0.515 0.155 0.813 0.763

BI842047 -0.425 0.053 -0.061 0.36 0.344 -0.238 -0.184 0.412 -0.038 0.036 0.576 0.146

BI845510 -0.728 -0.12 0.041 0.182 0.888 0.869 0.996 0.957 1.059 0.31 1.139 0.931

BI880136 -0.5 -1.189 -0.529 -0.753 -0.095 -0.173 -0.258 -0.479 0.155 -0.247 0.406 0.312

BI891552 -0.658 -0.202 -0.013 -0.591 0.062 0.095 0.034 0.153 0.638 0.058 1.013 0.678

BI896301 -0.041 0.453 0.4 0.072 -0.517 0.063 -0.132 -0.453 0.299 0.193 1.019 0.067

BI982117 -1.596 -1.039 -0.707 -0.044 -0.099 -0.255 -0.648 -0.977 0.426 -0.397 0.661 0.576

BI982877 0.718 0.142 0.147 0.316 -0.103 0.168 0.114 0.544 -0.035 -0.054 1.038 0.06

BM024326 -0.33 0.224 0.359 0.343 0.412 0.2 0.211 0.61 0.319 0.167 0.916 0.537

BM026491 -0.836 -0.599 -0.53 -0.134 0.832 -0.044 -0.411 -0.411 -0.091 -0.231 0.777 0.575

BM102223 -0.385 -0.25 0.431 0.075 -0.049 -0.136 0.124 -0.03 0.455 0.093 0.617 0.484

BM156086 -0.746 -0.413 -0.608 -0.318 0.086 -0.145 -0.273 -0.264 -0.023 -0.007 0.72 0.277

BM156937 -0.349 0.079 0.298 -0.598 -0.048 0.052 0.002 0.181 0.076 0.319 1.45 0.077

D32214 -0.111 -0.152 -0.016 0.203 0.091 0.131 -0.169 0.655 0.409 0.152 0.563 0.177

U43658 -0.259 0.254 0.344 0.34 0.377 0.065 -0.043 0.021 0.216 -0.27 0.987 0.036

U93478 -0.025 0.146 0.291 0.471 0.529 0.455 -0.1 -0.072 0.301 0.144 0.905 0.184

Y13948 -0.383 0.021 -0.107 -0.369 -0.325 -0.009 -0.075 0.071 0.276 0.128 1.097 0.553

AF071496 -0.786 -0.035 -0.135 -0.628 0.091 -0.221 -0.181 0.069 -0.019 -0.204 0.966 1.528

AF114262 -1.583 -1.935 -1.168 -0.925 -1.669 -0.838 -0.821 -0.889 -0.348 -0.797 -0.31 0.649

AF430840 -0.819 -0.481 0.028 0.266 0.057 0.05 -0.044 0.143 -0.21 -0.587 -0.029 0.913

AI444433 0.263 0.045 0.322 0.243 0.228 0.022 0.328 -0.113 0.181 0.143 0.484 0.892

AI477020 -0.543 -0.211 -0.702 -0.88 -0.565 0.149 -0.243 0.84 0.548 0.428 0.446 1.106

AI545002 -0.728 0.07 0.001 -0.012 0.12 0.018 -0.294 0.264 -0.253 -0.445 0.079 0.694

AI641460 -0.482 -0.355 0.391 0.474 0.71 0.197 0.291 0.544 0.411 -0.105 0.68 0.707

AI658249 -0.525 -0.047 0.149 -0.51 -1.217 -0.349 0.073 -0.742 -0.407 -0.375 0.31 1.46

AI667241 -0.614 -0.087 -0.483 -0.448 -0.193 -0.499 -0.308 -0.667 -0.26 -0.289 0.585 1.009

AI974174 -0.753 -0.031 -0.555 0.341 -1.775 -0.228 -0.379 -0.965 -0.494 -0.763 -0.059 1.16

AI974205 -0.396 -0.224 -0.26 -0.646 0.161 0.056 -0.21 -0.174 0.156 -0.19 0.462 0.587

AJ245964 -0.812 -0.402 -0.403 -0.893 -0.244 -0.147 -0.451 -0.013 0.141 -0.728 0.425 1.917

AW154075 -0.155 -0.652 -0.842 -1.192 -1.06 -0.234 -0.248 -0.893 -1.195 -0.608 -0.198 1.188

AW233556 -0.737 -0.039 0.017 0.097 -0.296 -0.403 -0.43 -0.285 -0.021 -0.225 0.043 0.807

AW282035 0.377 -0.11 -0.097 -0.109 -0.349 0.184 0.499 -0.244 0.109 0.191 0.372 0.922

AW419638 -0.539 -0.446 -0.264 -0.202 0.038 -0.018 0.053 -0.281 0.373 -0.17 0.34 0.384

AW510270 -0.052 -0.371 -0.433 -0.383 -0.395 -0.115 -0.338 -0.402 0.01 -0.119 0.237 0.697

AW567517 -0.749 -0.53 -1.515 -0.462 0.14 0.264 0.136 0.788 0.319 0.167 0.183 0.876

AW595094 -0.055 -0.22 -0.715 -1.119 -0.556 0.063 -0.391 -0.507 -0.307 -0.253 0.253 1.34

BF157011 -0.728 -0.037 0.277 -0.24 0.05 -0.332 -0.219 0.193 -0.182 -0.749 -0.125 0.942

BG305533 -0.885 0.091 -0.468 -0.192 -0.645 -0.199 -0.48 -0.708 -0.689 -0.606 -0.02 1.254

BG305857 -0.118 -0.062 -0.031 0.128 0.113 0.368 -0.059 0.083 0.378 0.128 0.384 0.65

BG308557 -0.345 -0.143 0.306 0.335 0.328 0.222 0.184 0.227 0.24 -0.013 0.409 0.757

BG884401 -0.278 -0.612 -0.505 -0.627 -0.266 -0.268 -0.125 -0.734 -0.212 -0.022 0.176 0.979

BI350696 -0.239 0.062 0.04 0.252 -0.668 -0.392 0.024 -0.057 0.112 -0.613 0.078 1.37

BI429006 -0.542 0.214 0.32 -0.367 -0.119 -0.042 -0.424 -0.131 -0.468 -0.708 -0.039 0.745

BI534295 -0.302 -0.16 0.223 -0.355 -0.043 0.146 -0.087 -0.47 -0.143 -0.268 0.555 0.56

BI710508 0.044 0.492 -0.192 -0.095 -0.21 -0.245 0.115 -0.069 0.138 -0.024 0.293 0.587

BI840762 -1.311 0.002 -0.776 -0.45 -0.265 -0.282 -0.613 -0.856 -0.746 -0.251 0.275 2.068

BI841667 -0.607 -0.063 0.016 -0.243 0.103 -0.396 -0.216 -0.166 -0.398 -0.36 0.246 0.812

BI865609 -1.249 -0.931 -0.229 -0.817 -0.646 -0.108 -0.153 -0.203 -0.24 -0.527 0.081 1.259

BI866326 -1.072 -1.154 -0.76 -1.47 -2.553 -1.016 -0.852 -2.017 -1.135 -1.357 0.19 1.484

BI866335 -0.701 -0.154 -0.025 -0.621 -0.763 -0.016 -0.555 -0.55 -0.13 -0.692 0.666 0.729

BI879533 -0.541 -0.068 0.729 -0.562 0.829 0.627 0.044 1.092 0.327 0.182 0.366 1.044

BI880170 -0.349 -0.054 -0.231 0.03 -0.252 -0.102 0.029 -0.421 -0.213 -0.25 0.109 0.823

BI880201 -0.353 -0.24 0.21 0.463 0.042 0.184 -0.097 -0.192 -0.066 -0.453 -0.015 0.968

BI888755 -1.44 -1.054 0.551 0.444 0.276 0.614 0.472 0.447 0.43 0.34 0.164 0.875

BM102022 -0.728 0.439 0.143 -0.535 -0.029 -0.161 -0.395 -0.114 -0.237 -0.545 0.402 1.501

BM154370 -0.63 -0.668 -0.843 -0.447 0.092 -0.026 0.029 0.191 -0.185 -0.359 0.333 0.644

BM182680 -0.587 0.545 -0.02 0.341 0.097 -0.045 -0.003 -0.207 0.394 -0.632 0.423 0.913

H56788 -1.223 -1.502 -1.697 -0.61 -1.168 -0.418 -0.971 -2.071 -0.922 -1.369 -0.32 1.883

AA542593 0.025 -0.185 0.313 -0.335 -0.032 -0.229 -0.043 -0.127 -0.192 -0.112 0.095 0.828

AA605655 -0.728 -0.005 0.143 -0.51 -0.244 -0.416 -0.111 -0.33 -0.406 -0.236 0.008 1.482

AF036148 -0.192 -0.239 -0.24 -0.039 -0.375 -0.167 -0.177 -0.42 -0.463 -0.101 0.063 1.317

AF173984 -2.697 -2.181 -3.052 -1.907 -2.252 -1.324 -1.223 -1.795 -1.332 0.041 -0.576 1.144

AI331606 -0.26 -1.349 -0.509 -0.978 -1.974 0.076 -0.714 -1.826 -0.757 -0.416 -0.337 1.483

AI353581 -0.529 -0.212 -0.448 -0.527 -0.435 -0.091 -0.588 -0.592 -0.122 -0.102 0.039 1.413

AI444432 -0.728 0.545 0.124 -0.339 -0.055 -0.125 -0.428 0.193 -0.332 0.005 0.05 1.006

AI626604 -0.051 -0.282 0.118 0.525 0.611 0.504 0.403 0.275 0.063 0.2 0.052 0.739

AI877924 -0.373 0.03 -0.176 0.038 0.486 -0.21 0.298 0.143 0.042 -0.159 -0.015 0.572

AI942990 -0.119 -0.542 -0.384 -0.432 -0.891 -0.189 0.046 -0.898 -0.38 -0.144 -0.28 1.279

AI957812 -0.627 -1.427 -0.245 -0.173 -1.454 -0.484 -0.255 -1.491 -0.641 -0.043 -0.064 1.004

AJ317957 -2.203 -2.176 -1.523 -1.589 -2.157 -1.172 -1.53 -2.458 -1.631 -0.743 -0.589 1.57

AW115841 -0.354 -0.589 0.284 0.956 0.847 1.033 0.96 1.085 0.658 0.7 0.346 1.139

AW116326 -0.573 -0.367 -0.313 -0.325 -0.426 -0.54 -0.152 -0.831 -0.1 -0.1 -0.04 0.742

AW171505 -0.723 -0.158 0.297 -0.701 -0.146 -0.134 -0.556 -0.285 -0.155 -0.422 -0.298 0.812

AW232264 0.443 -0.58 -0.003 -0.223 -0.559 -0.255 0.023 -0.476 -0.22 0.017 -0.023 0.851

AW233059 -0.728 -0.245 -0.831 -0.522 -0.579 -0.517 -0.375 -0.565 -0.322 0.241 0.096 1.3

AW233684 -0.2 0.016 0.098 -0.351 -0.607 -0.335 -0.233 -0.323 -0.092 -0.478 -0.269 1.442

AW281650 0.395 -0.017 0.485 -0.173 0.206 0.245 0.398 0.257 0.312 0.321 0.208 1.137

AW826304 -1.031 -0.077 -1.014 -0.413 -1.015 -0.183 -0.518 -0.906 -0.616 -0.684 -0.261 1.5

AW826726 -0.395 -0.4 0.189 1.233 0.056 -0.444 0.256 -0.672 -0.643 -0.406 -0.823 1.03

BE693194 -0.486 0.044 -0.373 0.538 0.416 0.777 0.297 1.228 0.48 0.959 0.416 1.229

BF717510 0.094 0.139 -0.286 -0.342 -0.775 0.046 -0.441 0.081 0.309 0.45 0.341 1.199

BF938407 0.2 -0.041 -0.115 0.341 -0.108 0.442 0.432 -0.245 0.295 0.049 0.16 1.651

BG305568 -0.649 -1.432 -0.958 -1.096 -1.292 -0.365 -0.196 -1.338 -0.662 -0.262 -0.27 0.898

BG738534 -0.876 -1.376 -1.552 -1.342 -0.396 -0.284 -0.322 -0.664 -0.949 -0.244 -0.024 1.461

BI475215 0.073 -0.05 -0.24 -0.019 -0.095 0.119 -0.127 -0.163 0.079 0.1 0.134 1.345

BI673470 -0.578 -0.147 0.521 -0.063 -0.252 -0.032 0.345 -0.344 0.094 -0.129 0.063 1.458

BI840999 -0.503 -0.803 -0.342 -1.094 -1.692 -0.519 -0.076 -0.556 -0.559 0.136 0.074 1.717

BI863932 -1.902 -1.064 -1.738 -0.537 -1.776 -0.427 -1.045 -1.593 -1.029 -0.317 -0.176 1.566

BI880342 -0.179 0.19 0.185 0.228 0.162 0.147 0.309 -0.158 0.164 -0.419 0.086 1.778

BI886435 -0.26 0.005 -0.052 0.54 0.788 0.514 0.205 0.51 -0.026 0.477 0.36 1.364

BI888934 -0.783 -0.551 -1.144 -0.24 0.533 0.361 0.283 1.302 0.769 0.944 0.612 1.509

BM035348 0.116 -1.108 0.077 -0.329 -1.14 -0.031 -0.115 -1.005 -0.209 -0.023 0.057 1.258

BM154199 -4.446 -4.19 -4.237 -4.168 -4.457 -2.868 -2.792 -4.815 -3.254 -3.974 -2.759 2.525

BM155853 -2.209 -1.565 -2.752 -1.168 -1.723 -2.121 -1.227 -2.024 -1.665 -2.593 -1.368 2.272

BM156079 -0.702 -0.431 -0.73 -0.212 -0.927 -0.595 -0.442 -1.094 -0.42 -0.665 -0.335 1.745

BM185901 0.05 -0.114 -0.085 0.066 -0.146 0.386 -0.313 -0.312 -0.166 -0.041 0.095 1.595

Mean -0.547 -0.326 -0.257 -0.273 -0.266 -0.094 -0.147 -0.213 -0.07 -0.171 0.345 0.841
