# Supplementary material for: Transcriptome Analysis of Zebrafish Embryogenesis Using Microarrays
Source: PLoS Genet. 2005 Aug 26;1(2):e29. doi: 10.1371/journal.pgen.0010029 (PMC1193535; doi:10.1371/journal.pgen.0010029)
Supplement: Dataset S13 — (137 KB DOC) [file pgen.0010029.sd013.doc]

Dataset S13. Genes exhibiting peak of expression at the unfertilized egg (maternally loaded RNA.

Genbank IDUF egg 3hpf 4.5hpf 6hpf 7.7hpf 9hpf 10.7hpf 12hpf 15hpf 24hpf 30hpf 48hpf

BM081091 2.827 0.293 -1.323 -0.651 -1.403 -0.535 -0.272 -1.384 -0.942 -1.909 -1.218 -1.157

BM181944 2.815 -0.694 -2.599 -0.429 -1.221 -0.721 -1.215 -1.553 -1.69 -2.598 -1.645 -1.903

BI673483 2.623 0.277 -2.229 -2.813 -2.008 -1.696 -2.105 -2.001 -3.12 -3.62 -3.256 -3.394

BM104005 2.588 -1.245 -1.378 -1.131 -1.885 -0.822 -0.933 -2.395 -0.908 -2.577 -1.87 -2.093

BI980436 2.555 -2.223 -3.135 -2.456 -3.163 -1.873 -2.613 -4.349 -2.714 -3.946 -4.395 -3.267

BI672829 2.547 0.527 -0.903 -1.725 -1.4 -1.287 -1.131 -1.923 -2.474 -1.877 -4.203 -3.296

BG883703 2.524 -1.172 -1.223 -0.883 -0.962 -0.401 -0.515 -0.986 -0.994 -0.703 -1.134 -0.364

BM095607 2.51 -3.655 -4.336 -4.056 -3.689 -2.586 -4.015 -4.684 -3.918 -5.755 -3.732 -4.504

BI708320 2.478 0.515 -0.776 -0.754 -1.52 -1.034 -1.279 -1.834 -1.67 -1.795 -1.709 -1.215

BI867066 2.461 1.229 0.932 1.043 0.177 0.817 0.618 0.272 -0.129 -0.187 -0.988 -1.367

AJ011789 2.436 0.195 -2.58 -3.21 -3.959 -3.696 -4.103 -4.251 -4.599 -5.055 -4.566 -5.09

BM037178 2.43 0.07 -1.643 -0.831 -2.107 -1.446 -1.065 -2.789 -1.413 -2.629 -2.144 -2.326

BM071805 2.427 0.296 -2.048 -3.703 -4.472 -0.848 -1.633 -4.037 -1.943 -2.593 -3.908 -2.017

AW116023 2.409 0.207 -0.941 0.075 -1.074 -0.385 -0.204 -0.933 -0.739 -1.949 -0.75 -1.119

BM035598 2.406 1.217 -0.192 -0.935 -1.439 -0.536 -0.677 -1.384 -0.632 -0.288 -1.305 -0.818

BM035368 2.385 0.61 -0.55 -2.382 -2.723 -1.072 -1.127 -2.802 -1.128 -1.942 -2.409 -2.345

BI673452 2.379 -0.165 -0.093 -0.248 -1.924 -1.703 -1.938 -3.082 -1.782 -2.238 -2.128 -2.426

BI979975 2.343 -2.442 -1.813 -1.49 -1.68 -0.991 -0.545 -1.417 -1.164 -0.867 -1.937 -2.565

BM104515 2.322 1.2 1.154 0.776 0.538 0.693 -0.183 -0.586 -1.334 -2.04 -2.601 -2.214

BI709863 2.316 0.367 -0.115 0.817 0.283 0.077 -0.373 0.039 -0.261 -0.673 -1.012 -0.903

AI974137 2.313 -2.212 -2.718 -2.913 -2.6 -1.181 -2.179 -1.366 -2.621 -2.786 -1.62 -2.028

BM101541 2.3 0.991 -0.337 -1.032 -1.346 -0.554 -0.865 -1.584 -1.161 -1.343 -1.559 -1.437

BI980382 2.253 2.013 0.884 -0.582 -1.17 -0.77 -0.566 -1.708 -0.784 -1.789 -1.242 -1.611

BI866963 2.231 -2.741 -2.877 -2.768 -2.901 -1.046 -2.767 -2.836 -2.166 -3.318 -3.14 -2.11

BM104000 2.197 -2.156 -1.677 -2.405 -3.089 -1.079 -1.529 -4.164 -2.167 -3.145 -3.527 -3.405

BI979286 2.197 -1.095 -1.643 -0.716 -0.504 -0.391 -0.732 -0.751 -0.786 -1.752 -0.744 -1.55

U00931 2.166 1.372 0.777 0.047 -1.133 -1.523 -2.244 -1.924 -2.301 -3.19 -2.85 -3.045

BI888350 2.148 1.536 0.863 0.877 0.35 0.466 0.282 0.266 -0.118 -0.257 -0.522 -0.675

BI878195 2.144 -2.395 -3.302 -2.216 -3.259 -3.066 -4.083 -4.539 -4.078 -5.2 -5.376 -5.064

BI843286 2.118 -1.165 -1.262 -2.332 -2.77 -1.576 -2.302 -3.616 -2.02 -1.951 -3.558 -2.277

AW128246 2.113 -1.006 -2.169 -2.199 -1.964 -0.922 -0.401 -1.425 -0.922 -1.679 -1.187 -1.656

AI497232 2.107 -2.202 -1.519 -1.5 -2.699 -1.905 -1.516 -3.002 -3.018 -2.578 -3.75 -2.593

AF149720 2.107 -1.396 -1.147 -1.066 -1.496 -0.845 -0.921 -1.455 -0.778 -1.451 -1.122 -1.356

BM101527 2.104 -2.532 -3.325 -3.098 -3.153 -2.984 -3.368 -3.428 -3.074 -4.723 -4.244 -3.918

AI964116 2.099 -3.467 -2.332 -2.284 -3.585 -2.832 -2.932 -4.446 -4.13 -3.956 -6.182 -5.295

AJ011790 2.088 0.415 -1.765 -0.938 -3.901 -1.892 -1.495 -3.68 -1.577 -2.617 -3.243 -3.236

BM036885 2.085 -0.061 -1.04 -0.072 -1.353 -0.358 -0.943 -0.564 -0.917 -1.82 -0.748 -0.862

BM184007 2.08 -3.212 -3.123 -2.097 -2.584 -2.071 -1.975 -3.349 -3.033 -2.774 -3.499 -3.265

BG302504 2.067 -4.968 -4.639 -4.352 -5.23 -4.092 -4.583 -5.691 -5.38 -5.537 -5.57 -6.063

BI884788 2.06 0.251 -0.329 -0.087 -0.6 0.014 -0.264 -0.154 -0.311 -0.427 -0.324 -0.587

BM095242 2.041 -2.393 -1.749 -1.447 -3.338 -1.81 -0.938 -3.798 -1.378 -2.945 -3.005 -3.586

BI885503 2.031 -0.301 -1.706 -0.795 -1.945 -0.385 -0.189 -1.856 -0.714 -1.651 -1.519 -1.094

U57390 2.024 -3.715 -3.445 -3.736 -3.603 -2.936 -4.124 -4.222 -3.421 -5.514 -3.691 -5.232

BM095845 2.021 0.705 -1.569 -1.068 -1.902 -0.949 -1.824 -1.418 -1.417 -2.774 -2.028 -1.939

AF288409 2.002 -0.708 -1.194 -0.813 -0.018 -0.431 -0.948 -0.336 -0.74 -1.505 -0.865 -0.964

AI667323 1.995 -0.778 -0.642 -0.7 -0.31 0.442 -0.109 0.334 -0.08 0.727 0.251 0.019

AW826487 1.992 0.014 -1.667 -1.659 -2.124 -0.611 -0.701 -2.197 -1.354 -2.326 -1.403 -1.156

BM182238 1.988 1.253 -0.441 -0.544 -2.303 -1.088 -0.497 -2.209 -1.117 -2.457 -1.286 -1.482

BM103840 1.987 -3.33 -3.321 -2.826 -3.824 -3.224 -3.424 -4.271 -3.633 -3.647 -4.548 -4.319

BM182782 1.983 -0.854 -1.131 -1.34 -1.648 -1.27 -2.014 -1.667 -1.625 -1.334 -2.039 -1.133

AW421941 1.955 -0.689 -1.343 -0.921 -0.96 -0.216 -0.806 -1.365 -1.052 -1.471 -1.637 -1.156

BI888131 1.944 0.378 0.512 0.566 0.25 0.489 0.087 0.47 -0.166 -0.216 -0.681 -1.159

AW116414 1.933 1.471 0.758 -0.084 -1.78 -2.103 -2.741 -3.332 -2.848 -2.794 -2.393 -2.236

AW117015 1.925 -1.043 -1.454 -0.865 -1.215 -0.517 -0.527 -1.445 -0.961 -1.169 -1.091 -0.89

BI708455 1.921 -0.034 -2.471 -0.983 -1.432 -1.185 -0.889 -1.566 -1.36 -2.74 -2.636 -2.164

BI878842 1.92 -1.273 -1.207 -1.088 -0.849 -0.355 -0.419 -0.988 -0.933 -1.854 -1.01 -0.915

BI878184 1.909 0.576 -0.756 -1.595 -2.638 -0.905 -1.678 -2.749 -1.701 -1.636 -2.416 -2.149

BI889925 1.904 -1.509 -1.511 -1.852 -2.242 -1.102 -1.552 -2.284 -1.875 -1.043 -1.95 -1.002

BM181792 1.903 0.429 -1.442 -1.394 -2.733 -2.416 -3.231 -3.422 -2.991 -2.4 -2.041 -2.725

BG303935 1.903 0.346 -0.101 0.571 0.239 0.228 -0.429 -1.102 -1.105 -2.289 -2.04 -2.077

AI667622 1.891 1.044 -0.791 -0.091 -0.537 -1.192 -0.739 -1.111 -1.715 -1.513 -1.977 -2.14

BG303377 1.89 1.564 -0.514 -0.296 -0.231 0.634 0.366 0.884 0.271 0.237 -0.429 -0.675

BI705594 1.886 0.066 -1.1 -1.604 -1.423 -0.48 -1.489 -0.951 -0.804 -0.878 -0.797 -0.467

AW134000 1.869 -3.232 -2.957 -3.488 -3.722 -2.825 -4.167 -5.218 -4.834 -5.32 -7.339 -5.879

BI890056 1.867 -0.331 0.554 0.994 0.513 0.619 -0.001 -0.506 -0.485 -1.176 -1.098 -1.316

BI980747 1.866 0.554 -0.143 0.386 0.682 1.054 0.498 0.917 0.325 0.183 -0.054 -0.393

BM184105 1.863 -3.329 -3.261 -2.873 -3.313 -2.25 -3.042 -4.128 -2.319 -3.802 -3.808 -3.464

AW116567 1.859 0.877 -0.153 -0.337 -1.023 -0.115 -0.513 -0.708 -1.15 -1.072 -0.972 -1.428

AB032727 1.857 -0.302 -1.492 -1.571 -3.025 -1.173 -0.679 -2.807 -0.758 -2.328 -1.868 -2.263

BI475648 1.855 0.641 -0.452 -0.044 0.217 0.479 0.011 0.131 -0.218 0.018 -0.078 -0.34

AW116573 1.854 -0.201 -2.067 -2.401 -1.422 -0.899 -1.579 -1.339 -1.817 -1.704 -1.46 -1.436

BI325685 1.853 1.552 0.338 0.256 0.034 0.266 -0.342 -0.018 -0.405 -0.571 -0.787 -0.621

BM103127 1.85 -0.53 -0.66 -0.796 -1.866 -0.903 -1.345 -2.272 -1.575 -0.732 -2.171 -1.894

BM181708 1.848 -0.241 -0.72 -0.896 -2.613 -2.091 -2.234 -3.924 -2.403 -2.319 -3.433 -2.627

BM071837 1.843 -0.709 -1.339 -0.549 -1.267 -0.661 -0.694 -1.425 -1.151 -1.484 -1.039 -1.029

BI980644 1.837 0.896 2.001 0.889 0.66 0.612 0.411 0.571 -0.091 -0.731 -0.628 -1.054

AW077854 1.834 0.508 -1.713 -1.325 -3.11 -2.077 -2.814 -3.555 -3.362 -2.32 -2.268 -2.525

BE557106 1.834 -0.668 -0.134 -0.944 -1.026 -0.384 -0.554 -0.75 -0.529 -1.095 -1.168 -0.941

AW171204 1.829 -0.057 -0.544 -0.195 -1.712 -0.703 -0.395 -1.05 -0.884 -0.878 -1.242 -1.261

AW173869 1.828 1.359 1.466 0.412 -0.882 -0.886 -1.453 -1.676 -1.255 -1.13 -0.905 -1.002

BM185291 1.825 0.297 -1.947 -1.845 -4.31 -1.753 -1.864 -4.659 -2.307 -3.591 -4.063 -3.301

BM095657 1.823 -2.53 -2.688 -1.408 -2.738 -2.2 -2.089 -3.419 -2.033 -2.199 -2.313 -2.133

AF302805 1.821 -0.08 -0.639 -0.524 -1.126 -0.395 -1.195 -1.136 -1.623 -1.82 -1.693 -1.706

BM095390 1.815 -1.135 -1.669 -0.91 -0.334 -0.546 -0.63 -0.822 -0.752 -1.429 -0.538 -1.078

BI892353 1.811 -2.305 -2.942 -2.859 -3.706 -2.955 -3.06 -3.945 -3.735 -3.958 -4.792 -4.513

AI384860 1.806 0.742 -0.612 -0.065 0.251 0.832 0.114 0.018 -0.389 -0.719 -0.762 -0.891

BM095897 1.801 -0.725 -1.563 -1.432 -3.851 -1.629 -1.147 -3.404 -1.282 -2.887 -3.118 -3.259

AW422010 1.794 1.917 0.438 -0.188 -1.51 -1.231 -1.644 -2.43 -2.169 -2.328 -2.958 -2.831

AW154587 1.792 0.269 -0.58 -0.202 -0.273 -0.045 -0.524 -0.563 -0.515 -1.046 -0.648 -0.679

AI882824 1.786 0.943 -0.267 -0.413 -1.726 -0.571 -0.415 -1.532 -0.66 -0.357 -0.834 -2.305

BE017795 1.779 -1.516 -0.513 -2.2 -2.122 0.03 -1.015 -1.44 -0.559 -0.314 -0.427 -0.46

AA494842 1.776 1.231 -1.928 -1.956 -1.46 -0.562 -1.724 -1.113 -1.675 -2.426 -2.345 -2.101

BG303781 1.776 1.794 0.651 0.088 -1.185 -0.466 -0.841 -1.053 -0.629 -0.975 -1.447 -1.003

BM036938 1.763 1.075 0.201 0.46 0.179 0.148 -0.287 -0.309 -0.504 -0.77 -0.513 -0.763

BM071732 1.755 0.041 0.683 0.286 0.181 0.873 0.344 0.409 0.171 0.128 -0.02 -0.109

AI558398 1.751 0.584 -0.479 -0.748 -0.722 0.031 -0.11 -0.423 -0.225 -0.192 -0.523 -0.502

AF331967 1.749 -3.198 -2.422 -2.993 -3.42 -2.84 -3.009 -3.908 -3.619 -4.677 -4.381 -4.744

BI878480 1.737 0.964 -1.527 -1.73 -2.973 -0.797 -1.838 -3.785 -2.046 -3.77 -3.31 -2.247

BM182720 1.721 1.553 0.166 -0.077 -0.855 -0.419 -1.185 -1.52 -1.158 -2.395 -1.831 -1.489

AW154176 1.711 0.948 -0.213 -0.455 -1.206 -0.534 -0.977 -1.441 -0.857 -0.953 -1.339 -1.037

BM184046 1.71 1.329 0.293 -0.118 -0.62 -0.375 -1.044 -1.032 -2.037 -2.516 -1.971 -2.401

BG884507 1.699 -0.863 -1.373 -1.433 -1.086 -0.062 -0.953 -0.568 -1.132 -1.665 -0.413 -1.148

BI979865 1.697 1.652 0.287 0.353 -0.658 -0.43 -0.53 -0.587 -0.578 -0.951 -0.981 -0.682

AW154153 1.695 0.377 -1.018 -0.291 -0.087 0.312 0.188 0.265 -0.198 -0.333 -0.441 -0.674

BI891821 1.695 0.27 -0.176 -0.463 -1.209 -0.806 -1.325 -2.47 -1.858 -2.457 -1.788 -2.243

BM181653 1.692 1.169 -1.147 -1.191 -1.975 -1.303 -2.028 -2.454 -2.436 -2.278 -3.029 -3.227

BM024634 1.69 0.61 0.759 0.533 0.077 0.728 -0.066 0.093 -0.329 -0.294 -0.446 -0.242

BM026879 1.688 -0.811 -0.595 -0.936 -0.767 0.01 -0.175 -0.672 -0.48 -0.315 -0.621 -0.358

AW116284 1.688 -0.206 -0.422 -0.291 -0.975 -0.954 -1.167 -1.626 -1.205 -1.255 -1.011 -0.786

BI705282 1.687 1.074 -0.022 0.135 0.004 0.45 0.207 0.517 0.255 0.151 0.173 -0.393

BM096012 1.686 1.358 -0.272 0.341 -0.233 -0.229 -0.231 0.07 -0.271 -0.687 -0.213 -0.409

BI983894 1.685 1.184 0.172 0.077 0.215 0.279 -0.029 0.123 0.032 0.458 0.01 -0.371

BM005437 1.682 -1.012 -1.051 -1.244 -1.034 -0.516 -0.917 -0.567 -0.622 -0.602 -0.728 -0.291

BI889409 1.681 -0.141 -0.417 -0.023 0.277 1.071 0.437 0.979 0.332 0.376 0.058 -0.354

AW421436 1.679 0.731 -0.409 -0.955 -1.93 -1.966 -1.806 -3.551 -2.246 -3.567 -4.003 -3.458

AW171271 1.664 1.07 0.343 -0.052 -0.402 -0.243 -0.687 -0.567 -1.024 -0.674 -0.969 -0.797

BM096428 1.663 -0.106 -0.15 0.369 0.187 0.411 0.225 0.713 0.12 0.39 -0.177 -0.287

BM183654 1.663 0.375 0.678 0.341 -0.103 0.013 -0.124 -0.24 -0.289 -1.018 -0.48 -0.671

BI889101 1.659 1.234 1.209 1.308 1.215 0.594 0.293 0.671 -0.054 -0.595 -0.57 -0.333

BM154014 1.654 0.824 -1.168 -0.12 -0.566 -0.351 -0.686 -0.922 -0.979 -1.713 -1.699 -2.088

AI667676 1.65 0.668 -0.701 -0.531 -0.73 -0.68 -0.395 -1.042 -1.179 -1.687 -1.613 -1.855

BM095404 1.65 -2.744 -2.535 -2.842 -3.273 -2.764 -3.46 -4.215 -4.317 -5.652 -5.952 -5.761

BM037539 1.647 0.312 -0.54 0.438 -0.291 -0.135 -0.275 -0.73 -0.626 -0.684 -1.085 -0.88

BM101604 1.647 -0.051 0.031 -0.019 -0.477 -0.126 -0.356 -1.171 -0.381 -0.98 -0.467 -1.157

AW115859 1.645 0.666 -0.914 -0.53 -0.261 -0.343 -0.834 -0.6 -0.724 -0.977 -0.959 -1.381

AW422929 1.644 -0.883 -1.754 -1.58 -1.564 -0.59 -1.229 -1.146 -1.477 -1.924 -2.023 -1.726

BM095484 1.631 -1.449 -2.099 -0.921 -2.802 -1.826 -1.071 -3.305 -1.783 -3.723 -2.218 -2.922

BM005412 1.63 1.116 -0.649 -0.522 0.022 0.276 -0.551 -0.089 -0.296 -0.241 -0.574 -0.584

BM070949 1.63 1.204 -0.386 -0.246 0.011 -0.179 -0.327 -0.443 -0.546 -0.523 -0.554 -0.562

BI673509 1.629 0.492 0.081 0.501 0.305 0.317 -0.061 -0.095 0.044 -0.754 -0.045 -0.369

BM184757 1.62 1.236 0.734 0.619 0.326 0.243 -0.424 -0.487 -0.62 -0.938 -1.328 -1.145

AW171049 1.619 -2.866 -1.865 -2.807 -3.899 -1.919 -1.944 -4.515 -3.745 -3.747 -5.879 -3.391

AI721398 1.611 -0.299 -3.023 -1.153 -2.283 -1.413 -1.155 -2.316 -1.451 -2.221 -1.308 -1.124

BG892077 1.609 0.095 0.409 0.701 0.962 0.71 0.869 0.444 0.095 0.176 -0.252 -0.527

BE016164 1.607 1.31 -0.3 -0.383 -0.42 -0.169 -0.106 0.608 -0.002 -0.451 -0.525 -0.439

BI843250 1.607 1.163 1.105 0.888 -0.183 0.037 -0.568 -0.396 -0.341 -0.232 -0.554 -0.528

BM082789 1.604 0.555 -0.173 -0.079 -1.14 -0.584 -0.727 -2.146 -1.103 -2.367 -1.677 -1.621

BM104526 1.596 -3.1 -3.537 -2.565 -3.504 -3.155 -2.908 -4.425 -3.431 -4.981 -3.16 -4.345

BM183795 1.595 0.601 -0.282 -0.342 -1.09 -0.357 -0.743 -1.472 -0.817 -1.783 -1.746 -1.28

AW170860 1.591 -3.303 -3.424 -2.933 -3.497 -1.938 -2.953 -4.659 -3.741 -5.338 -4.994 -4.085

BM095417 1.59 1.44 0.048 0.473 0.168 0.242 -0.151 -0.094 -0.353 -0.111 0.199 -0.563

BM037305 1.587 -0.089 0.256 0.269 0.452 0.476 0.162 0.584 0.257 0.499 0.272 -0.013

AW232464 1.587 0.399 -0.349 0.011 0.296 0.954 0.103 0.041 -0.594 -0.834 -1.607 -1.477

BG727413 1.582 0.28 -0.401 0.019 0.218 0.581 0.159 0.63 -0.264 -0.426 -0.207 -0.394

AW115660 1.579 0.154 -1.622 -0.503 -0.817 -0.59 -0.384 -1.055 -0.48 -1.513 -0.474 -0.808

BI979961 1.574 0.613 -0.487 -0.685 -0.075 -0.045 -0.37 -0.494 -0.29 0.006 -0.507 -0.308

BM184281 1.568 0.863 -1.31 -1.657 -1.503 -1.042 -1.194 -1.397 -1.998 -2.06 -2.549 -2.474

BM036958 1.564 1.427 0.243 0.102 -0.265 0.144 -0.232 -0.346 -0.454 -0.222 -0.985 -0.699

AI959659 1.564 0.438 0.078 0.374 0.414 0.629 0.135 0.396 0.071 -0.112 -0.524 -0.659

BI672616 1.559 0.042 -0.228 -0.011 -1.415 -0.724 -1.478 -2.638 -1.262 -1.386 -1.721 -0.943

AW115821 1.557 0.763 -0.552 -0.754 -0.739 0.042 -0.431 0.028 -0.523 -0.659 -0.611 -0.705

BI709146 1.556 0.341 -0.024 0.17 0.381 1.13 0.013 1.059 0.47 0.49 0.232 -0.223

BM182742 1.555 -0.768 -1.027 -0.799 -0.853 -0.293 -0.062 -0.834 -0.127 -0.621 -1.154 -0.896

BI867089 1.554 0.931 -0.418 0.05 -2.49 -1.309 -0.972 -2.543 -2.112 -1.616 -3.469 -2.513

BI843130 1.551 1.101 0.266 0.242 -0.313 -0.207 -0.467 -0.374 -0.531 -0.653 -1.025 -0.656

BM095815 1.551 -0.483 -2.051 -1.333 -1.737 -1.281 -1.167 -2.007 -2.196 -3.061 -2.458 -2.331

BG304232 1.547 -0.823 -0.57 -0.698 -2.327 -1.269 -1.655 -2.199 -1.904 -1.568 -2.441 -1.71

AW344043 1.545 1.541 0.894 0.82 0.832 1.091 0.412 0.715 0.071 0.102 -0.204 -0.563

BE201771 1.544 0.786 -0.991 -0.963 -0.675 -1.069 -1.526 -1.314 -1.67 -0.595 -0.448 -1.417

BG305492 1.541 1.562 1.072 0.726 0.65 0.61 0.34 0.248 -0.272 0.141 -0.226 0.124

BM181897 1.535 -0.067 -2.066 -3.148 -2.698 -1.025 -1.697 -1.682 -2.225 -2.663 -2.114 -2.25

AW116994 1.53 0.934 0.34 1.073 0.471 0.341 0.128 0.099 -0.19 -0.755 -0.781 -0.933

AW116681 1.529 0.742 0.269 0.265 0.231 0.512 -0.048 0.115 -0.373 -0.211 -0.604 -1.166

BI892254 1.518 -0.237 -1.226 -1.026 -1.213 -0.7 -0.777 -1.399 -1.383 -1.871 -1.389 -1.663

AW019723 1.518 0.81 0.182 0.215 0.294 0.507 0.335 0.668 -0.009 0.158 -0.011 -0.085

BG308713 1.515 0.399 -0.526 -0.15 0.333 0.182 0.09 0.365 0.129 -0.217 -0.261 -0.193

BG303457 1.513 1.158 0.949 0.057 -0.933 -0.145 0.04 -1.041 -0.421 -0.812 -1.004 -0.23

BM095156 1.508 0.515 -0.459 -0.079 0.117 -0.039 -0.456 -0.531 -0.631 -1.408 -0.72 -0.992

BM101590 1.507 -2.58 -1.698 -1.758 -2.787 -1.392 -1.451 -3.585 -2.01 -2.302 -3.691 -3.217

AI641069 1.506 1.293 0.691 0.133 0.316 0.196 0.049 0.789 0.038 -0.138 -0.08 -0.38

BM035394 1.498 0.055 -0.212 -0.726 -0.173 0.041 -0.357 -0.166 -0.284 -0.585 -0.443 -0.286

BI867264 1.498 0.429 -0.878 -0.609 -1.718 -1.304 -0.9 -2.424 -1.789 -1.439 -1.574 -1.503

BI878269 1.497 0.389 0.075 0.354 0.134 -0.018 -0.312 0.171 -0.067 -0.72 -0.417 -0.717

BI709743 1.497 0.407 -0.121 -0.195 0.056 0.361 -0.001 0.316 -0.01 0.191 0.074 -0.369

BI843480 1.493 -0.026 -0.108 -0.187 -0.166 -0.125 -0.414 -0.405 -0.626 -1.171 -0.591 -0.637

AW116159 1.488 1.578 0.779 0.535 0.19 0.538 0.499 0.98 0.446 0.249 -0.429 -0.713

BI887500 1.487 1.142 -1.149 -0.761 -0.946 0.08 -0.736 -0.703 -0.491 -1.492 -0.415 -0.309

BI878018 1.485 0.662 -1.518 -0.268 -1.178 -0.453 -0.698 -1.072 -0.831 -1.354 -1.006 -1.608

AI883911 1.482 -0.406 0.501 -0.124 -0.571 -0.269 -0.462 -1.218 -1.004 -1.892 -1.223 -1.281

BI888943 1.481 0.606 0.323 0.403 -0.357 -0.634 -1.182 -1.478 -1.297 -1.547 -1.683 -2.113

BM095259 1.481 1.427 0.047 -0.125 -0.249 0.168 0.048 0.959 0.358 0.059 0.155 -0.698

AW174275 1.48 -1.004 -0.824 0.967 0.171 -0.543 0.557 -0.923 -0.811 -2.882 -2.11 -2.221

BI878609 1.478 1.189 -0.064 -0.433 -1.371 -0.445 -1.174 -1.83 -0.873 -1.011 -1.253 -0.952

AI957831 1.475 1.006 -0.264 -0.299 -0.408 0.056 -0.183 -0.108 -0.253 -0.257 -0.337 -0.676

BM185226 1.466 0.626 -0.27 -0.635 -0.804 0.04 -0.485 -0.558 -0.41 -0.516 -0.307 -0.391

BE605880 1.461 0.71 0.952 0.299 -0.406 0.441 0.075 0.116 -0.562 -0.299 -0.958 -0.984

BI979556 1.458 -0.941 -0.303 0.789 0.95 0.12 -0.079 -0.214 -0.294 -0.544 -0.658 -0.748

AI444200 1.457 0.583 0.551 0.869 0.095 -0.151 -0.037 -0.166 -0.193 -0.585 -0.122 -0.654

AW116420 1.455 1.152 -0.605 -0.822 -0.585 -0.442 -0.575 -0.622 -1.138 -1.092 -1.181 -1.063

AY057058 1.451 -0.277 -1.309 -0.527 -1.699 -0.864 -0.23 -1.978 -0.616 -1.085 -1.244 -0.57

AW076546 1.451 1.47 0.702 0.14 -0.213 0.437 -0.097 -0.236 -0.667 -0.789 -1.396 -1.537

BG303079 1.451 0.132 -0.304 -0.283 -0.2 -0.344 0.169 -0.447 -0.291 -0.379 -0.432 -0.422

AF387820 1.449 1.061 -0.839 -0.632 -0.901 -0.232 -0.724 -0.561 -1.061 -1.166 -0.812 -1.02

AW116206 1.449 -3.773 -3.24 -2.841 -3.433 -2.796 -3.238 -4.262 -4.089 -5.011 -4.462 -5.078

AW116490 1.432 1.407 -0.549 -0.79 -0.695 -0.141 -0.374 -0.423 -0.602 -1.04 -0.747 -1.05

BI890158 1.432 -0.082 -1.082 -1.156 -1.313 -0.408 -0.786 -1.409 -1.237 -0.946 -1.734 -1.891

AW076964 1.428 -2.313 -3.401 -3.23 -3.732 -1.666 -2.508 -3.539 -3.797 -4.05 -3.9 -3.539

AW344075 1.428 -2.213 -2.493 -1.675 -2.414 -1.312 -1.389 -2.697 -1.922 -3.31 -2.504 -2.583

AW154231 1.428 1.243 0.222 0.537 0.152 0.701 0.189 0.348 -0.029 0.108 -0.347 -0.233

BM181720 1.427 -1.269 -0.747 -0.241 -0.987 -0.471 -0.76 -2.045 -1.17 -1.455 -1.973 -1.183

BG307539 1.425 0.326 1.024 1.475 1.177 0.592 0.892 0.811 0.131 0.112 -0.297 -0.428

AI584590 1.42 -0.394 -0.833 -0.745 -0.227 0.122 -0.213 -0.127 -0.088 0.026 -0.062 -0.197

BM182245 1.419 1.422 1.112 0.622 0.003 -0.331 -0.21 -1.164 -0.94 -0.984 -1.492 -1.459

AW128332 1.415 -0.534 -0.297 0.083 0.315 0.786 0.029 0.273 -0.104 -0.07 -0.259 -0.418

AF164477 1.413 1.219 0.458 0.341 0.651 0.351 0.221 0.551 0.243 0.195 -0.049 -0.639

BI474952 1.413 1.022 -0.627 -0.905 -1.733 -0.858 -1.057 -1.117 -1.67 -1.91 -0.923 -1.522

BI979228 1.411 -0.003 -0.13 0.045 -0.379 -0.326 -0.09 -0.698 -0.198 -0.962 -0.248 -0.574

BI897419 1.41 0.658 -1.211 -1.051 -1.615 -0.601 -1.016 -0.902 -0.962 -0.872 -0.097 -0.79

BI879686 1.409 0.874 0.826 1.221 0.912 1.52 0.617 0.815 -0.009 0.457 -0.157 -0.264

BM185294 1.408 1.023 -0.126 -0.159 -0.133 0.047 0.133 0.523 0.181 0.272 0.249 -0.139

BI979438 1.405 -0.673 -0.988 -0.898 -0.959 -0.348 -0.849 -1.193 -0.584 -1.163 -1.127 -0.981

BM070561 1.404 -1.209 -0.984 -0.914 -0.989 -0.174 -0.536 -1.155 -1.098 -0.639 -0.884 -0.118

BI839625 1.403 0.177 -1.091 -0.235 -0.799 -0.886 -0.59 -0.954 -0.6 -1.145 -0.786 -0.943

BI867248 1.401 -1.282 -1.543 -0.915 -1.181 -0.56 -1.162 -1.374 -1.005 -1.512 -1.009 -1.492

BI877328 1.4 1.403 1.243 1.146 0.507 -0.001 0.304 0.614 0.024 0.699 0.173 -0.589

AJ249490 1.399 1.02 -0.788 -1.466 -3.217 -2.073 -3.416 -3.605 -3.504 -3.908 -3.409 -3.471

AW154269 1.398 0.611 1.195 0.958 1.186 1.401 0.508 0.686 0.409 0.151 -0.036 -0.256

BI885851 1.397 0.958 -0.445 0.062 -0.132 0.217 0.372 -0.302 -0.176 -0.247 -0.208 -0.199

BI867642 1.397 0.773 0.699 0.394 0.289 0.494 0.209 0.967 0.213 0.256 -0.045 -0.055

BI984734 1.395 -0.794 -0.827 -0.576 -1.421 -0.517 -0.911 -1.439 -0.483 -1.081 -0.857 -0.721

BM104033 1.39 1.531 -0.563 -0.536 -0.418 -0.093 -0.195 -0.779 -0.599 -0.593 -0.55 -0.86

BI866724 1.389 1.367 0.559 0.235 -0.348 0.337 -0.099 0.118 -0.277 -0.685 -0.861 -0.739

BI983582 1.387 0.333 0.101 0.085 -0.004 0.112 -0.192 0.652 0.015 0.846 0.516 0.379

BI474957 1.387 0.698 -0.497 0.015 -0.211 -0.231 0.015 -0.453 -0.525 -0.572 -0.64 -0.572

AW154517 1.385 0.479 -0.996 -0.472 -1.375 -0.303 -0.611 -1.297 -0.949 -1.202 -0.958 -0.943

BM101600 1.385 1.222 0.363 0.667 0.584 0.217 0.263 0.312 -0.151 -0.196 -0.54 -0.204

BI890592 1.382 0.223 0.294 0.176 0.465 0.424 0.137 0.877 0.204 -0.016 0.246 -0.177

BM185239 1.381 0.244 0.236 0.099 0.884 1.303 1.032 1.351 0.593 0.523 0.119 -0.256

AI878452 1.379 1.165 0.538 0.081 -0.065 0.184 -0.133 -0.377 -0.604 -0.421 -0.686 -1.008

AW078163 1.376 0.135 -0.126 0.175 0.567 0.452 0.427 0.688 -0.125 0.042 -0.359 -0.21

BM182440 1.367 0.785 -0.757 -0.531 -0.361 -0.062 0.492 -0.131 -0.396 -0.103 -0.329 -0.666

AW305598 1.364 0.137 -0.001 -0.248 -0.219 0.115 -0.202 0.149 -0.216 -0.211 -0.11 -0.507

BI981133 1.357 0.695 -1.484 -0.479 -0.896 -0.171 -0.47 -0.936 -0.467 -0.535 -0.78 -0.757

AI544976 1.357 -0.236 0.392 0.318 -0.324 -0.133 -0.225 -0.905 -1.081 -0.779 -0.933 -0.613

BI673488 1.353 1.427 0.244 0.221 0.223 0.774 0.194 0.144 -0.381 -0.167 -0.3 -0.367

AW116489 1.35 -0.636 -0.185 -1.132 -3.283 -1.373 -1.47 -2.895 -1.823 -0.946 -3.801 -3.894

BM095989 1.346 1.088 0.119 0.621 0.395 1.02 0.646 0.763 0.435 0.216 0.033 -0.222

BM183873 1.345 1.011 0.43 0.097 -0.913 -0.762 -0.561 -1.652 -1.323 -1.921 -1.736 -2.218

AW115606 1.344 1.274 -0.467 0.341 0.086 -0.111 -0.313 0.174 -0.411 -0.683 -0.506 -0.369

AW077337 1.341 0.587 -0.82 -0.475 -0.489 0.067 -0.271 -0.258 -0.427 -0.683 -0.76 -0.452

AW115973 1.339 0.88 -0.879 -0.589 -1.264 -0.712 -0.754 -1.233 -0.455 -0.894 -1.323 -1.188

BI983855 1.338 0.214 -1.19 -0.601 -0.205 -0.182 -0.5 -0.547 -0.939 -0.894 -0.71 -1.228

AI964212 1.338 0.64 -0.055 -0.051 0.489 0.345 -0.01 0.323 0.071 -0.012 -0.165 0.058

BI877898 1.337 1.099 -0.564 -0.701 -0.735 -0.164 -0.733 -0.769 -0.69 -1.268 -0.952 -0.867

BI886119 1.333 1.009 -0.132 0.026 -0.106 0.203 -0.207 -0.459 -0.399 -0.717 -0.865 -0.788

BI879576 1.33 -0.051 -0.634 -0.676 -0.801 -0.39 -0.562 -1.157 -0.943 -0.91 -1.701 -0.939

BI980396 1.328 -0.851 -0.998 -0.453 -1.056 -0.845 -0.171 -1.374 -0.31 -1.038 -0.479 -0.437

AW018939 1.326 0.11 -1.478 -1.138 -0.635 -0.527 -0.444 -1.038 -0.69 -1.255 -0.477 -0.833

AW116271 1.325 0.535 0.116 1.121 -0.11 -0.113 -0.387 -0.295 -0.208 -0.273 -0.13 -0.339

AW154457 1.319 1.001 -0.087 -0.245 -0.236 0.229 -0.062 0.48 0.125 -0.106 0.137 -0.379

BI672630 1.316 0.721 0.035 0.044 -0.526 0.307 0.115 -0.289 -0.227 0.004 -0.26 -0.29

AW171467 1.311 0.937 -1.581 -1.835 -2.169 -0.61 -1.341 -1.494 -0.83 -1.456 -1.773 -1.317

BI891936 1.309 0.49 0.515 -0.145 -0.266 -0.111 -0.672 -1.057 -1.035 -0.098 0.051 -0.861

AW344056 1.308 1.104 -0.298 -0.561 -0.7 -0.394 -0.882 -1.052 -0.839 -1.23 -1.038 -0.797

BI866770 1.308 1.313 0.356 0.219 0.283 0.233 -0.025 0.298 -0.118 -0.009 -0.113 -0.275

BI706176 1.306 0.974 0.416 0.283 0.195 0.546 0.235 0.712 0.209 0.414 0.109 -0.244

BM082504 1.306 1.248 -0.539 -0.99 -0.905 -0.119 -0.551 -0.968 -0.516 -1.017 -0.687 -0.69

BG307383 1.305 -0.699 -0.659 -0.465 -0.572 -0.136 -0.137 -0.139 -0.352 0.212 -0.155 0.063

AW171085 1.304 0.445 0.225 0.238 0.512 0.61 0.372 0.662 0.236 0.01 -0.392 -0.626

BI889959 1.303 -1.154 -0.915 -0.964 -1.9 -0.604 -1.089 -2.161 -1.307 -0.875 -1.636 -0.975

BI887770 1.303 0.148 -0.837 -0.403 -0.648 -0.9 -0.36 -1.077 -1.286 -1.244 -2.087 -1.571

BM095365 1.303 0.251 -0.163 -0.389 -1.419 -0.022 -0.187 -0.909 -0.636 0.023 -0.224 -0.036

BI885564 1.303 0.082 -0.11 -0.28 -0.393 -0.28 -0.594 -0.534 -0.458 -1.129 -0.2 -0.486

BG305537 1.302 0.918 -0.099 0.136 -0.179 -0.028 -0.019 -0.06 -0.241 -0.675 -0.678 -0.408

BG727595 1.3 0.468 0.71 0.626 0.562 1.117 0.672 0.293 0.492 0.469 0.32 -0.093

BM185350 1.299 -0.792 -0.462 -0.329 -0.3 0.616 0.406 0.406 -0.071 0.886 0.451 0.037

BM183770 1.297 1.094 -0.433 -0.961 -1.126 -0.138 -0.753 -1.359 -0.924 -0.665 -1.248 -0.656

BI673579 1.293 1.008 -0.777 -0.432 -0.539 0.052 -0.619 -0.22 -0.475 -0.3 -0.539 -0.567

AW281416 1.293 0.085 0.329 0.566 0.412 0.192 -0.211 -0.18 -0.316 0.234 -0.306 -0.308

BI877878 1.289 -1.86 -1.365 -1.085 -1.594 -1.061 -0.794 -2.322 -0.952 -1.433 -2.008 -2.033

BI980026 1.287 -2.131 -2.184 -2.514 -3.578 -2.029 -2.416 -4.168 -3.162 -3.11 -4.399 -3.453

AI793622 1.287 1.022 -0.552 -0.413 -0.504 -0.133 -0.3 -0.225 -0.562 -0.976 -0.627 -0.913

AI641779 1.285 0.512 0.058 0.414 0.694 0.916 0.331 0.771 0.518 0.497 0.304 0.256

BI476292 1.284 0.631 -0.449 -0.39 -0.141 -0.149 -0.564 -0.14 -0.312 -0.579 -0.503 -0.354

BM103396 1.283 -0.743 -1.615 -0.851 -1.172 -0.452 -0.767 -0.8 -0.738 -0.36 -0.51 -0.081

AW116474 1.282 -0.968 -1.094 -0.857 -1.632 -0.531 -0.826 -1.72 -1.166 -1.616 -1.419 -1.74

BI888265 1.281 0.802 -0.819 -0.359 -0.627 -0.423 -0.717 -0.718 -0.433 -1.092 -0.757 -1.067

AI588468 1.281 0.736 -0.186 -0.474 -0.071 -0.059 -0.138 -0.375 0.225 -0.552 -0.134 -0.216

AW171595 1.281 0.594 0.761 0.442 0.754 1.172 0.575 0.807 0.383 0.138 0.339 0.056

AW171522 1.28 1.091 0.7 0.663 0.44 0.3 0.092 0.137 -0.206 -0.308 -0.285 -0.879

BM184003 1.279 0.656 -0.309 -0.472 -0.516 -0.191 -0.255 -0.509 -0.246 -0.549 -0.651 -0.699

BG303695 1.277 0.948 -0.099 0.144 -0.031 0.236 -0.422 -0.547 -0.902 -0.686 -0.488 -0.301

BM095386 1.276 -0.616 -2.389 -1.217 -1.503 -0.652 -1.426 -1.984 -1.812 -2.887 -2.301 -2.227

AW174595 1.272 -1.568 -1.208 -1.05 -0.746 0.028 -0.299 -0.103 -0.397 0.239 -0.168 0.219

BI709417 1.272 -1.539 -0.584 -1.482 -1.516 0.243 -0.398 -1.047 -0.546 -0.056 -0.219 0.072

AW344022 1.271 0.287 -0.499 -0.647 -0.285 0.01 -0.114 -0.486 -0.384 -0.192 -0.629 -0.336

BI878123 1.271 0.863 0.149 -0.003 -1.054 -0.352 -0.319 -0.488 -0.325 -0.246 -0.706 -0.533

BI673276 1.27 0.148 -1.438 -0.774 -0.769 0.159 0.158 0.634 -0.096 0.027 -0.466 -0.756

BE017551 1.268 -2.541 -2.35 -2.217 -2.396 -1.927 -2.569 -2.748 -1.702 -2.977 -2.953 -2.908

AI667141 1.264 0.117 0.466 0.026 -0.06 0.565 0.388 0.005 0.229 0.338 0.336 0.53

AW116727 1.263 0.576 -0.38 -0.248 -0.373 -0.043 -0.377 -0.467 -0.275 -0.813 -0.645 -0.474

AW116228 1.262 -1.761 -2.119 -1.705 -2.334 -2.987 -3.65 -3.64 -4.47 -5.033 -5.052 -4.477

BI979549 1.259 -0.418 -0.356 -0.525 -0.183 -0.375 -0.029 0.462 0.078 -0.1 0.063 -0.251

BM103974 1.257 0.9 -1.044 -1.302 -0.601 0.132 -0.154 -0.47 -0.763 -0.068 -0.279 -0.534

AI721420 1.257 0.872 1.082 0.817 0.063 0.007 0.375 -0.285 0.145 0.274 -0.225 -0.173

BM096095 1.254 -0.568 -0.169 -0.211 -0.018 0.268 0.191 0.553 0.273 0.46 0.152 -0.431

BI878459 1.253 0.58 -0.488 -0.582 -1.302 -0.363 -1.204 -1.011 -1.376 -1.319 -1.473 -0.963

AW420717 1.25 1.2 1.056 1.301 0.287 0.397 0.228 0.172 0.058 -0.424 -0.866 -0.685

BG799157 1.249 -0.824 -0.565 -0.401 0.04 0.294 0.02 0.182 -0.228 -0.238 -0.171 -0.138

BI877725 1.249 0.651 -0.789 -1.121 -0.229 -0.323 -0.449 -0.494 -0.585 -0.353 -0.316 0.032

AW171350 1.248 1.013 0.57 0.506 0.028 0.403 -0.153 -0.214 -0.144 -0.16 -0.602 -0.568

BI704370 1.248 0.597 0.979 1.223 0.677 0.732 0.574 0.119 -0.05 -0.299 -0.838 -0.621

BI891643 1.248 0.456 -0.609 -0.271 -0.207 0.426 0.166 -0.193 -0.534 -0.862 -1.31 -2.089

BE200811 1.244 1.355 0.051 -0.396 -1.075 -0.163 -0.715 -0.216 -0.532 -0.969 -1.025 -0.872

BI979304 1.243 1.111 0.957 1.055 0.715 0.484 0.235 0.77 0.15 0.232 -0.186 -0.215

AW128192 1.242 0.501 -1.287 -0.321 -0.44 0.08 -0.236 -0.274 -0.034 -0.548 -0.333 -0.653

BI429706 1.237 -0.997 -0.921 -0.722 -0.802 -0.358 -0.193 -0.61 -0.556 -0.546 -0.517 -0.168

BF157346 1.237 0.327 -0.802 -0.334 -0.543 0.206 0.148 -0.177 -0.159 -0.318 -0.502 -0.404

BM036445 1.236 0.187 -1.429 -0.687 -0.757 -0.161 -0.706 -0.469 -0.989 -0.617 -0.952 -1.336

AW777691 1.231 0.159 0.326 -0.046 0.253 0.883 0.285 0.991 0.431 0.972 0.573 0.061

BG306096 1.229 -0.06 -0.559 0.29 -0.562 -0.162 -0.231 -0.546 -0.276 -0.682 -0.476 -0.614

BI672555 1.229 0.013 -0.428 0.017 0.15 0.6 0.249 -0.084 -0.158 0.039 0.068 0.328

BM104357 1.225 1.297 0.688 0.342 0.189 -0.025 -0.108 -0.129 -0.165 -0.412 -0.404 -0.388

BG304274 1.224 0.883 0.255 -0.153 -0.538 -0.022 -0.494 -0.336 -0.462 -0.269 -0.651 -0.594

AF001299 1.224 -1.043 -1.387 -0.922 -0.98 -0.687 -0.607 -0.766 -1.082 -1.304 -1.17 -0.25

BI877645 1.224 1.214 -0.086 -0.352 -0.771 -0.209 -0.649 -1.154 -0.782 -0.395 -0.935 -0.632

BM184239 1.222 0.473 -0.083 -0.315 -0.187 0.253 0.231 0.454 -0.036 0.395 -0.155 -0.632

BM103922 1.221 -0.124 -0.014 -0.478 -0.883 -0.732 -1.229 -0.983 -1.033 0.42 0.17 0.521

AF095457 1.221 -2.998 -2.489 -3.206 -4.066 -3.554 -4.045 -5.04 -3.945 -4.241 -6.51 -5.517

BI891978 1.221 0.845 0.38 0.241 0.11 0.28 -0.204 -0.165 -0.284 -0.536 -0.849 -0.824

BM071664 1.219 -0.711 -0.3 -0.062 -0.018 0.763 0.333 0.808 0.287 0.709 0.259 -0.172

AI964174 1.216 -0.219 -0.56 -0.287 -0.35 -0.269 -0.329 -0.717 -0.496 -0.682 -0.565 -0.473

AI545442 1.216 -0.788 -0.52 -0.865 -0.807 -0.226 -0.822 -0.762 -0.574 -0.092 -0.134 0.313

BM104100 1.213 -0.102 -0.221 -0.297 -0.526 -0.051 -0.423 -0.689 -0.081 -0.142 -0.255 -0.169

BI983596 1.209 -0.214 0.044 0.184 0.138 0.381 -0.137 -0.03 -0.045 -0.163 -0.228 -0.299

BM155459 1.208 0.432 -0.038 0.485 0.819 1.113 0.583 0.594 -0.257 -0.344 -0.788 -1.277

AW282065 1.206 -1.398 -2.554 -2.628 -2.351 -1.725 -2.649 -2.895 -2.678 -3.401 -3.268 -2.962

AI878687 1.206 0.298 0.352 0.27 1.091 0.508 0.359 0.296 0.412 0.34 0.13 0.344

BM071697 1.203 -0.592 -0.256 -0.185 0.196 0.501 -0.009 -0.028 -0.081 -0.385 -0.114 -0.368

AW076692 1.202 1.157 0.038 -0.015 -1.312 -0.671 -0.926 -0.785 -0.88 -1.108 -1.44 -1.304

BG305800 1.202 0.155 0.404 0.038 -0.26 0.047 -0.249 0.072 -0.113 0.259 -0.023 -1.261

BI892293 1.201 -3.872 -3.16 -2.827 -4.032 -2.986 -2.674 -4.719 -3.465 -4.145 -4.836 -5.033

AW116628 1.2 0.397 -0.749 -0.745 -1.591 -0.707 -0.858 -1.647 -1.073 -1.173 -1.751 -2.269

AW154516 1.194 1.318 0.046 0.03 -0.267 -0.012 0.149 -0.107 0.021 -0.134 -0.105 -0.305

BI877887 1.191 0.367 0.154 0.5 0.286 0.514 -0.054 0.46 0.051 0.11 0.125 0.05

BI885932 1.19 0.812 1.26 0.612 -0.24 -0.411 -0.307 -1.046 -0.455 -0.863 -1.182 -1.145

AW116214 1.19 0.268 -0.624 -0.099 -0.478 -0.118 -0.237 -0.288 -0.267 -0.065 -0.33 0.003

AW154647 1.185 1.14 0.501 -0.021 -0.476 -0.125 -0.429 -0.355 -0.2 -0.015 -0.234 -0.405

AI667671 1.183 1.232 0.721 0.68 0.547 0.379 0.529 0.827 0.101 0.087 -0.065 -0.215

BM096092 1.183 -0.95 -1.172 -1.49 -1.063 -0.299 -0.83 -1.005 -0.859 -0.751 -0.614 -0.636

BM026053 1.182 -0.528 -1.49 -1.045 -0.603 -0.526 -0.765 -1.58 -1.021 -1.254 -1.334 -1.487

AJ245492 1.179 0.721 -0.139 -0.565 -1.051 -0.923 -1.24 -1.464 -1.131 -1.083 -1.054 -0.907

AI957426 1.179 0.865 1.032 0.998 0.961 0.727 0.165 -0.115 -0.208 0.01 -0.533 -0.366

AW171480 1.179 0.433 -0.128 -0.329 -0.287 0.068 -0.117 -0.167 -0.088 0.058 -0.116 -0.26

AI722482 1.179 0.576 -0.232 -0.237 -0.339 0.026 -0.005 -0.466 -0.17 -0.316 -0.077 -0.383

AW019444 1.178 0.296 -0.093 0.527 0.216 0.817 0.562 0.62 0.317 0.436 -0.006 0.025

BG303246 1.177 0.711 -0.114 -1.185 -0.762 -0.678 -0.912 -1.133 -0.828 -1.039 -0.635 -0.639

BI880563 1.175 0.947 0.267 0.183 0.682 0.976 0.32 0.682 0.25 0.393 0.181 0.265

BI705720 1.174 0.015 0.078 -0.426 0.11 0.276 -0.408 0.04 0.262 0.447 0.622 0.502

AW058902 1.172 0.241 0.639 0.704 0.437 1.285 0.666 1.256 1.021 1.039 0.401 -0.436

BI983762 1.172 0.551 -0.422 -0.479 -0.084 0.061 -0.326 0.53 -0.178 0.088 -0.116 0

BM184129 1.172 1.088 0.396 -0.374 -0.497 0.114 -0.311 -0.474 -0.267 -0.488 -0.482 -0.425

BI980084 1.171 0.766 -0.344 -0.072 0.09 0.474 -0.002 0.132 -0.293 0.002 -0.35 -0.898

BM184227 1.168 0.895 -0.798 -0.007 -0.975 -0.463 -0.346 -1.532 -0.414 -0.773 -1.159 -1.13

AW128153 1.168 1.028 0.75 1.014 0.746 1.113 0.461 0.406 0.008 0.193 -0.029 -0.559

BM155251 1.163 -0.217 -0.373 -0.071 -0.191 -0.296 0.282 -0.217 -0.312 -0.143 -0.454 -0.181

AF176316 1.163 0.289 -0.082 0.253 0.082 -0.356 -0.439 -0.709 -0.541 -1.129 -1.096 -0.92

BM082387 1.16 0.723 -0.095 0.181 0.42 -0.105 0.295 -0.015 -0.04 -0.369 -0.186 -0.469

AW133873 1.159 -0.566 -0.843 -0.503 -2.152 -0.755 -0.415 -1.82 -0.562 -0.688 -1.255 -0.78

AI477049 1.156 1.11 0.066 -0.2 -1.625 -0.487 -0.529 -0.626 -0.669 -0.818 -0.966 -1.244

BG729054 1.156 -1.105 -0.747 -0.933 -1.147 -0.78 -1.798 -1.343 -0.962 -1.052 -1.207 -1.229

AW343922 1.156 -1.703 -1.979 -1.476 -1.214 -0.594 -1.065 -0.931 -0.878 -0.449 -0.759 -0.508

AW171484 1.155 0.056 -0.178 -0.797 -1.04 0.52 -0.201 0.268 -0.014 -0.303 -0.543 -0.349

BM101584 1.151 -0.177 -0.146 0.184 0.186 -0.058 0.133 -0.083 -0.191 -0.308 0.055 -0.369

BI846235 1.147 0.449 -0.409 -0.154 -0.693 -0.286 -0.745 -0.817 -0.348 -0.334 -0.291 -0.315

BG303890 1.146 1.225 -0.304 -0.368 -1.054 -0.749 -0.556 -1.412 -1.004 -0.688 -1.091 -0.974

AW019758 1.146 -1.189 -0.63 -0.49 -0.137 -0.093 -0.26 -0.313 -0.227 -0.629 -0.842 -0.534

AW154324 1.144 0.506 0.111 0.113 0.177 0.715 0.447 0.97 -0.071 0.16 0.522 0.077

BI672095 1.143 0.546 0.615 0.413 0.273 0.266 0.184 0.135 0.089 -0.037 0.164 -0.252

AW154496 1.143 0.71 -0.615 -0.55 -0.602 -0.582 -0.744 -0.655 -0.841 -1.259 -0.464 -1.182

BI879969 1.143 0.9 0.269 0.012 -0.233 -0.249 -0.249 -0.053 -0.129 -0.153 -0.221 -0.396

AI882829 1.136 1.169 -0.2 -0.203 -0.451 0.02 -0.105 0.024 -0.294 0.157 -0.126 -0.232

BG304233 1.136 0.49 -0.442 -0.158 -0.86 -0.349 -0.07 -0.012 -0.237 -0.369 -0.719 -0.367

BI983850 1.134 0.815 0.121 0.695 0.6 1.244 0.303 0.323 0.403 0.249 0.202 0.357

BI980160 1.132 0.971 0.026 -0.382 -0.848 -0.236 -0.409 -0.34 -0.393 0.324 -0.146 -0.402

BM104379 1.13 0.64 0.111 -0.368 -1.189 -0.148 -0.576 -1.185 -0.499 -0.487 -0.864 -0.666

AW116246 1.129 0.992 0.856 0.432 0.022 -0.206 -0.124 -0.239 -0.397 -0.95 -0.647 -0.967

BM184284 1.128 0.231 0.501 0.662 0.171 0.787 0.548 0.81 0.521 0.6 0.244 0.238

BI865356 1.128 1.204 -0.144 0.115 0.162 0.123 0.129 -0.038 -0.045 -0.238 -0.155 -0.448

AW232589 1.127 -0.885 -0.917 -0.851 -0.865 -0.419 -0.473 -0.676 -1.088 -0.888 -1.277 -0.915

AW117161 1.127 0.774 0.597 0.32 0.622 0.434 0.446 0.731 0.127 -0.149 -0.316 -0.303

BG729177 1.127 1.144 0.153 -0.177 -0.166 0.302 -0.021 0.608 0.441 -0.047 -0.356 -0.285

AB055681 1.126 0.02 -0.07 0.495 0.035 0.188 0.448 0.524 0.158 0.594 0.267 0.035

BI710046 1.126 -0.3 0.149 0.049 0.245 0.423 0.048 0.419 0.013 0.31 0.075 -0.179

BI885399 1.125 0.377 -0.079 -0.171 -0.432 0.069 -0.207 -0.069 -0.06 0.069 -0.269 -0.237

BM181627 1.124 1.28 -0.289 -0.611 -1.28 -0.455 -0.926 -0.996 -0.861 -0.854 -1.024 -0.734

BM184122 1.122 0.332 0.165 -0.11 0.029 0.881 0.262 0.156 -0.029 0.409 0.267 0.281

AW171396 1.116 0.564 0.37 0.324 0.166 0.259 -0.079 -0.26 -0.1 -0.146 -0.033 0.213

BM095268 1.114 0.207 -0.001 0.175 -0.405 0.317 -0.117 0.546 0.31 0.591 0.26 -0.174

BG302931 1.113 0.993 -0.198 -0.157 -0.079 -0.205 0.009 -0.169 -0.312 -0.204 0.055 -0.238

AW077480 1.112 -1.164 -2.006 -1.794 -1.841 -1.243 -1.727 -1.471 -3.097 -2.986 -2.648 -2.122

AW018514 1.112 1.204 -0.301 -0.336 -0.365 -0.185 -0.368 -0.364 -0.237 -0.616 0.118 -0.241

BI888899 1.112 0.462 -0.578 -0.7 -0.177 0.03 0.004 -0.216 -0.165 -0.39 -0.362 -0.098

BI866874 1.109 -0.22 -0.653 -0.521 0.407 0.169 -0.324 -0.467 -0.429 -0.005 -0.188 -0.564

AF170069 1.109 -0.667 -1.106 -1.293 -2.115 -1.924 -1.105 -3.109 -2.77 -2.591 -2.384 -1.833

AI397333 1.109 0.61 0.607 0.501 0.011 0.307 0.04 -0.062 -0.204 -0.036 -0.261 -0.342

AW342745 1.109 0.724 0.163 0.303 0.834 1.161 0.392 0.599 0.164 0.292 -0.009 0.133

AI588173 1.106 0.632 -0.496 -0.203 0.366 0.343 -0.003 0.398 -0.106 0.339 0.163 -0.059

AI721290 1.106 0.677 0.457 0.154 0.115 0.6 0.309 0.751 0.166 0.345 0.116 -0.022

BG306308 1.104 -1.842 -1.875 -1.296 -1.045 0.128 -0.423 -0.104 -0.41 0.41 0.759 0.158

BI979957 1.103 0.937 -0.257 -0.242 -0.151 0.451 0.362 0.827 0.042 0.166 -0.437 -0.27

BM185181 1.101 1.021 0.293 -0.484 -2.334 -0.481 -0.637 -2.931 -1.535 -0.703 -2.689 -1.968

BM184017 1.101 0.143 0.075 -0.051 -0.745 -0.074 0.134 0.233 -0.04 -0.044 -0.219 -0.429

AI942944 1.099 0.025 -0.503 -0.119 0.17 0.228 -0.445 -0.134 0.135 -0.294 -0.469 -0.367

BI673308 1.098 0.669 0.132 0.394 0.31 0.488 0.139 0.591 0.186 -0.012 -0.323 -0.655

BI867235 1.097 0.62 -0.222 -0.299 -0.33 0.116 -0.256 -0.309 -0.321 -0.051 -0.309 0.144

AW171363 1.096 0.017 -0.221 0.298 0.173 0.018 -0.156 -0.396 -0.569 -0.203 -0.261 -0.123

AW154137 1.096 1.221 -1.177 -0.207 -0.341 -0.237 -0.367 -0.256 -0.384 -0.364 -0.53 -0.435

AF097875 1.095 0.213 -1.754 -1.652 -2.092 -1.451 -1.613 -1.79 -1.245 -0.967 -1.191 -1.683

BI878642 1.092 0.82 -0.491 -0.327 -0.84 0.253 -0.324 0.11 -0.177 0.073 -0.042 0.03

BM182575 1.091 -0.162 -0.259 0.306 0.387 0.152 0.006 -0.078 0.05 -0.684 -0.465 -0.476

AW344260 1.089 0.706 -0.525 -0.217 -0.29 0.218 0.256 0.584 0.324 0.265 0.271 -0.409

BI877691 1.088 0.736 -0.25 -0.294 -0.532 -0.047 -0.265 -0.075 0.348 0.616 0.458 0.077

BI672464 1.088 -0.034 -1.072 -0.582 -0.596 -0.45 -0.764 -1.128 -0.762 -1.046 -1.188 -0.731

BI980199 1.088 -0.02 -0.277 -0.002 0.032 0.042 0.224 0.874 0.194 0.586 0.07 0.153

AW115523 1.088 0.534 -0.01 -0.053 -0.389 -0.21 -0.878 -0.58 -0.639 -0.778 -1.035 -1.285

AW171527 1.087 0.596 0.162 -0.191 -0.872 -0.524 -0.283 -1.113 -0.945 -1.678 -0.891 -0.938

AW165310 1.087 0.328 0.017 -0.446 -1.489 0.082 -0.503 -1.317 -0.851 -1.073 -1.548 -1.22

AW115804 1.087 0.63 -0.508 -0.594 -0.546 -0.335 -0.417 -0.596 -0.232 -0.488 -0.566 -0.567

AB038320 1.086 -0.008 0.222 0.173 -0.107 0.358 -0.247 -0.446 -0.172 -0.175 -0.475 -0.622

AI942929 1.086 0.56 -0.916 -0.494 -0.306 0.101 -0.145 -0.088 -0.061 -0.083 -0.06 0.104

AW116237 1.084 0.276 -0.074 0.056 -0.535 -0.232 -0.379 -0.638 -0.694 0.079 -0.28 -0.543

BM103996 1.084 0.635 -0.119 0.214 0.174 0.234 0.099 -0.149 -0.406 -0.474 -0.447 -0.924

BM104048 1.083 1.044 0.581 0.284 0.596 0.309 -0.057 0.111 -0.664 -0.49 -0.678 -1.118

BI980223 1.081 0.771 -0.267 0.057 -1.244 -0.174 -0.213 -0.973 -0.673 -0.379 -1.099 -0.473

BG303824 1.079 0.185 0.236 0.091 -0.199 0.037 -0.079 -0.154 -0.226 -0.284 -0.07 -0.239

BG303641 1.079 0.945 -0.62 -0.05 -0.278 -0.074 -0.5 -0.488 -0.455 -1.022 -0.49 -0.252

BM183382 1.078 -0.785 -1.002 -1.209 -1.782 -0.937 -0.487 -2.098 -1.086 -1.537 -1.511 -1.298

AI721358 1.077 -0.315 -0.308 -0.451 -1.379 -0.502 -0.39 -1.494 -0.575 -0.125 -0.97 -0.339

BI844059 1.075 0.423 0.204 0.057 -0.251 0.514 -0.1 -0.126 -0.041 -0.245 -0.703 -0.775

AW076961 1.075 0.522 0.16 0.213 -0.226 -0.523 -0.506 -1.517 -1.53 -1.377 -1.658 -0.503

BI983027 1.071 0.487 0.071 -0.152 0.026 -0.063 -0.14 0.719 0.173 0.525 0.433 -0.267

AW232642 1.07 0.213 0.535 0.572 0.597 0.663 0.405 0.49 -0.013 -0.093 -0.342 -0.429

AI666911 1.069 0.898 -0.216 -0.129 -0.15 0.387 -0.371 -0.244 -0.053 0.096 -0.139 0.266

BG302802 1.067 -0.086 -0.944 -0.162 -0.196 0.407 -0.197 -0.039 -0.137 -0.487 -0.29 -0.417

AW116402 1.066 0.444 0.065 -0.069 -1.057 -0.803 -0.762 -2.496 -1.213 -1.497 -1.206 -1.198

AI641473 1.063 0.762 0.759 0.827 0.605 0.904 0.406 0.505 -0.025 -0.104 -0.451 -0.785

AW115598 1.063 1.09 0.165 -0.035 0.123 0.669 0.136 0.334 0.176 0.035 -0.123 0.075

AF364084 1.062 -0.065 -0.386 -0.193 -0.412 0.158 0.382 0.507 0.271 0.529 0.146 -0.428

AW175553 1.061 0.694 0.602 0.879 0.533 0.743 -0.022 -0.054 0.213 0.183 -0.218 0.046

BI867449 1.058 0.427 -0.421 -0.167 -0.332 -0.115 -0.233 -0.728 -0.15 -1.368 -0.275 -0.558

AI641585 1.057 -0.297 -0.188 0.023 -0.006 0.16 0.355 0.661 0.053 0.245 0.126 -0.002

BG303472 1.056 0.571 -1.243 -1.14 -0.925 -0.167 -0.391 -0.56 -0.707 -0.738 -0.773 -0.302

AW344030 1.055 0.334 -0.406 -0.003 0.097 0.652 -0.055 0.488 0.058 0.092 -0.232 -0.089

AW202620 1.051 -0.048 0.121 0.362 0.474 0.286 0.284 0.671 0.248 0.667 0.384 0.23

BM035613 1.047 0.674 0.238 -0.357 -0.087 0.058 -0.345 -0.044 0.099 -0.827 -0.645 -0.139

BI428520 1.043 0.002 -0.326 -0.258 -0.922 -0.175 -0.45 -0.766 -0.505 0.249 -0.034 -0.335

BG728382 1.042 0.349 -1.281 -0.641 -0.764 -0.402 -0.407 -0.199 -0.688 -0.735 -0.653 -0.981

BI886921 1.041 0.636 0.399 0.472 0.501 0.409 0.321 0.213 0.3 0.126 0.044 -0.13

AW154701 1.04 -0.012 0.411 0.414 -0.011 0.179 0.125 -0.405 -0.334 -0.4 -0.504 -0.391

AI959558 1.039 0.931 -0.212 -0.298 -1.983 -0.598 -0.162 -1.755 -0.545 -0.678 -1.556 -1.448

AI667289 1.039 -1.263 -0.838 -1.613 -0.966 -0.011 -0.625 -0.657 -1.122 -1.124 -1.648 -1.65

BG303177 1.036 1.072 0.352 -0.219 -0.262 0.158 -0.503 -0.678 -0.674 -0.716 -0.973 -0.807

BI891332 1.035 -0.436 -0.261 -0.212 0.092 -0.123 0.085 0.134 -0.38 0.18 0.235 0.164

BI709862 1.034 0.046 -0.458 0.024 -0.276 -0.386 -0.302 -0.601 -0.212 -0.366 -0.881 -0.498

BI867240 1.03 0.68 0.306 0.422 0.241 0.178 0.343 0.761 0.144 0.031 0.106 -0.008

BM181733 1.029 0.385 -0.312 0.041 -0.377 0.4 -0.01 -0.082 -0.045 -0.045 -0.504 -0.263

AI878397 1.028 1.153 -0.206 0.255 -0.115 -0.131 -0.263 -0.12 -0.563 -0.417 -0.587 -0.544

BI983753 1.028 0.47 1.03 0.53 0.09 0.127 -0.233 -0.351 -0.255 -0.268 -0.236 -0.368

BM035055 1.027 0.839 -0.566 -0.225 -0.46 -0.043 -0.043 -0.062 0.03 -0.083 -0.462 -0.281

BG308652 1.027 0.622 -1.17 -0.822 -1.329 -0.175 -0.58 -0.298 -0.345 -0.169 -0.672 -0.377

BI885469 1.024 0.804 -0.305 -0.021 -0.985 -0.608 -0.042 -0.935 0.109 -0.371 -0.663 -0.612

AI964178 1.023 0.223 -0.817 -0.044 -0.354 -0.182 -0.266 -0.239 -0.373 -0.214 -0.284 0.361

BI877999 1.023 1.094 0.349 0.703 0.366 0.001 0.097 -0.29 -0.3 -0.249 -0.722 -0.776

BM154327 1.02 -0.068 -0.104 0.253 -0.04 0.488 -0.303 0.133 -0.538 -1.246 -1.188 -1.295

BI877523 1.02 0.786 0.839 0.448 0.347 0.521 -0.083 0.038 0.358 -0.003 0.119 0.062

AW777332 1.016 0.832 0.545 0.641 0.034 0.457 0.007 0.423 -0.043 0.173 -0.087 -0.298

BI886717 1.01 0.515 1.119 0.634 0.676 0.434 0.067 0.116 -0.027 -0.076 -0.458 -0.204

BM182761 1.01 0.879 -0.482 -0.028 0.401 -0.115 0.048 -0.143 -0.384 -0.65 -0.73 -0.44

AW165251 1.006 -0.65 -0.471 0.19 0.599 0.474 0.35 0.667 0.137 -0.447 -0.354 -0.339

BI878416 1.005 0.662 -0.301 -0.234 -1.042 0.019 -0.472 -0.827 -0.415 -0.506 -0.617 -0.717

BM101666 1.005 0.751 -0.972 -0.462 -0.201 -0.419 -0.267 -0.756 -0.836 -1.141 -0.419 -0.707

AW777876 1.003 0.842 0.983 0.806 0.403 0.547 0.241 0.682 0.255 0.644 0.173 -0.164

BM101507 1.001 0.286 -0.512 0.066 -0.434 -0.289 -0.482 -0.278 0.025 0.04 -0.078 -0.106

AA658804 1.001 0.081 -0.566 -0.156 -0.958 0.19 0.452 0.54 0.431 0.75 0.171 0.111

BI709770 0.998 -0.123 -0.119 0.08 0.237 0.416 -0.08 -0.077 -0.159 -0.152 -0.425 -0.499

AI667659 0.997 -1.111 -1.388 -1.616 -1.386 -0.137 -0.477 -0.17 -0.357 -0.235 -0.246 0.089

AW077011 0.997 0.131 -0.048 -0.006 -0.025 -0.106 -0.256 -0.188 -0.127 -0.249 -0.212 -0.23

AW171388 0.997 0.937 -0.221 -0.69 -0.308 -0.032 -0.383 -0.445 -0.523 -0.818 -0.374 -0.115

AW019011 0.996 0.168 0.484 0.346 0.103 0.414 0.517 0.134 0.12 0.123 -0.335 -0.327

AW018983 0.996 0.135 -0.616 -1.432 -1.763 -0.094 -0.783 -0.985 -0.968 -0.501 -1.357 -0.675

BG304294 0.996 0.419 -0.036 0.021 -0.182 0.179 0.105 0.646 0.387 0.76 0.472 0.415

BM185202 0.992 0.74 0.623 0.35 0.404 1.003 0.191 0.225 0.062 0.154 -0.023 0.068

AI667214 0.991 0.459 -0.978 -1.179 -0.859 -0.102 -0.545 -0.647 -0.308 -0.409 -0.497 -0.498

BG883692 0.988 0.356 0.005 -0.154 0.32 0.694 -0.01 0.665 0.306 0.359 0.132 0.246

BI877478 0.987 -0.154 -0.161 -0.421 -0.789 -0.562 -0.564 -0.91 -0.67 -0.733 -1.029 -0.966

AI558559 0.987 0.519 -0.936 -0.401 -0.429 -0.055 -0.212 -0.099 -0.137 -0.995 -0.271 -0.721

BI896959 0.985 -0.166 -0.147 -0.329 0.171 0.2 0.134 0.68 0.297 0.448 0.598 0.387

BF717971 0.984 0.323 0.021 0.159 -0.782 -0.452 -1.076 -1.578 -0.769 -1.273 -1.054 -0.526

BI890907 0.984 0.878 0.485 0.094 -0.591 -0.08 -0.224 -0.317 -0.38 -0.46 -0.567 -0.366

BI885228 0.984 1.037 -1.269 -1.23 -1.536 -0.47 -1.038 -1.459 -1.155 -0.802 -0.793 -0.22

BI878817 0.983 0.986 -0.182 -0.274 -0.404 -0.137 -0.015 -0.44 -0.256 -0.464 -0.775 -0.483

BI879566 0.982 0.453 0.48 -0.024 0.146 0.767 0.267 0.22 0.343 0.044 -0.07 -0.064

BG305622 0.981 0.262 -0.213 -0.011 -0.772 -0.167 -0.411 -0.337 -0.342 -0.018 -0.214 -0.437

BI877608 0.977 0.017 0.058 -0.038 -0.168 0.102 0.19 -0.224 -0.33 0.087 -0.488 0.163

AY029577 0.977 -0.366 0.613 0.622 0.359 0.308 0.094 -0.228 -0.391 -0.086 -0.67 -0.748

BM185255 0.974 0.407 0.178 0.025 -0.16 0.101 0.03 -0.005 0.08 -0.01 0.025 0.113

AW116685 0.972 0.716 -0.982 -0.33 -0.993 -0.331 0.013 -1.21 -0.48 -0.704 -0.554 -0.515

BM071213 0.968 0.658 0.014 0.08 -0.03 0.114 -0.054 -0.266 -0.353 -0.494 -0.544 0.054

BI879616 0.967 -0.501 -0.03 0.569 -0.285 0.397 -0.073 0.102 -0.396 0.172 -0.017 0.063

BI885896 0.967 -0.681 -0.805 -0.356 -0.591 0.084 -0.653 -0.783 -0.827 -0.99 -1.213 -0.538

BI877285 0.966 0.493 0.022 -0.034 -0.152 -0.016 -0.213 -0.145 -0.229 -0.377 -0.335 -0.56

AF387341 0.965 0.333 -0.478 -0.31 -0.384 -0.186 -0.053 -0.055 -0.202 0.003 0.106 0.119

BI867169 0.964 -0.489 -0.236 -0.392 -0.307 -0.111 -0.467 -0.419 -0.775 -0.141 -0.297 0.087

AW170941 0.961 0.321 -0.961 -0.817 -0.575 -0.359 -0.434 -0.691 -0.638 -1.117 -1.162 -1.218

BI880518 0.96 1.131 -0.688 -0.996 -0.494 -0.196 -0.444 -0.678 -0.573 -0.66 -0.731 -0.544

BE017363 0.96 -0.295 -0.053 0.634 0.925 -0.187 -0.127 0.363 -0.019 -0.011 0.227 -0.311

BI877132 0.959 -3.513 -3.198 -3.268 -4.126 -3.181 -4.511 -5.157 -4.499 -6.549 -5.749 -6.206

BI983442 0.958 0.372 -0.113 0.165 -0.143 -0.078 -0.313 -0.066 -0.142 -0.305 -0.203 -0.245

BI840365 0.955 -0.92 -0.672 -0.228 -0.218 0.258 0.04 0.043 -0.425 0.008 -0.18 -0.134

BM156973 0.954 0.69 0.607 0.429 -1.245 -0.258 -0.408 -0.48 -0.455 -0.185 -0.228 -0.28

AW154320 0.952 0.134 -0.518 -0.031 -0.399 0.173 0.075 0.11 -0.011 -0.2 -0.576 -0.793

BI889465 0.95 0.277 -0.067 -0.038 -0.341 0.211 -0.523 -0.126 -0.36 0.324 0.125 0.087

BG305705 0.949 0.204 0.495 0.174 0.267 0.554 0.636 -0.184 0.429 0.271 0.512 0.523

BM024812 0.947 -0.091 -0.823 -0.698 -0.627 -0.244 -0.709 -0.673 -0.527 -0.388 -0.179 0.873

BI673444 0.943 1.002 0.207 0.268 0.012 -0.086 0.058 -0.224 -0.281 -0.241 -0.759 -0.892

AW165160 0.941 -2.896 -1.247 -2.895 -0.329 0.183 0.063 -0.081 -0.552 -0.412 -0.574 -0.396

AI545168 0.937 0.541 0.466 0.31 -0.092 -0.243 -0.519 -0.385 -0.601 -0.119 -0.389 -0.08

BG727181 0.935 0.325 0.173 -0.461 -0.714 0.096 -0.273 -0.717 -0.254 -0.71 -0.318 -0.46

BM182849 0.933 0.427 -0.285 -0.298 -0.87 -0.223 -0.27 -0.464 -0.494 -0.209 -0.658 -0.233

AW116504 0.933 0.393 0.234 0.407 0.43 0.961 0.521 0.63 0.37 0.194 -0.359 -0.438

BI843185 0.931 -0.005 -0.052 -0.719 0.026 -0.192 -0.034 -0.417 -0.192 -0.685 -0.274 -0.124

BG304211 0.93 0.215 -0.243 0.082 -0.29 0.201 0.083 -0.134 0.014 -0.429 -0.315 -0.569

BM095366 0.922 0.83 0.77 -0.083 0.101 0.207 -0.121 0.264 -0.278 -0.265 -0.475 -0.456

BI880780 0.921 0.921 0.089 0.079 -0.166 0.356 0.317 -0.129 -0.11 0.008 -0.316 -0.283

BG985703 0.921 -0.067 0.225 0.309 -0.374 0.09 0.085 -0.07 -0.124 0.401 -0.231 -0.695

AB040435 0.917 0.251 -0.593 -1.18 -2.03 -1.511 -2.238 -2.456 -3.02 -3.079 -3.34 -3.966

AW280158 0.916 0.204 -0.382 -0.345 -0.14 -0.04 -0.094 0.388 0.294 0.389 0.001 -0.256

BG302989 0.915 0.752 -0.909 -0.304 -0.821 -0.317 -0.345 -0.734 -0.543 -0.958 -0.298 -0.586

BG305441 0.915 0.361 -0.677 -0.291 0.119 -0.009 -0.982 -0.365 -1.198 -0.445 -0.344 -0.178

BI983566 0.912 0.675 -0.152 -0.093 -0.204 -0.244 -0.241 -0.09 -0.256 -0.014 -0.164 -0.282

BI845363 0.911 0.056 0.01 0.144 0.078 -0.135 0.108 0.28 -0.349 -0.031 -0.189 0.028

BE605975 0.907 0.582 0.67 0.275 -0.479 0.072 -0.463 -0.58 -0.289 -0.16 -0.398 -0.466

BI896532 0.907 -0.978 -0.959 -0.592 -1.232 -0.706 -0.743 -1.317 -0.789 -0.818 -0.737 -0.389

BM183399 0.906 0.573 -0.156 0.126 0.136 0.289 0.155 -0.071 -0.196 -0.273 -0.372 -0.327

BI891327 0.905 0.598 0.675 0.161 0.088 0.756 0.144 0.231 -0.043 -0.224 -0.65 -0.506

AI957837 0.904 0.405 0.023 0.486 -0.209 0.715 0.305 0.324 0.179 0.058 -0.411 -0.291

AI558273 0.903 0.356 0.337 0.377 0.359 0.829 0.062 0.929 0.474 0.349 0.273 -0.046

AI793794 0.903 -2.113 -2.251 -2.077 -1.759 -0.609 -1.292 -1.631 -1.825 -2.031 -2.409 -2.292

AW116302 0.901 0.588 0.197 0.67 -0.341 -0.258 -0.527 -1.119 -0.751 -0.897 -1.146 -1.234

BM185211 0.901 0.278 -1.278 -1.089 -0.353 -0.079 0.131 -0.259 -0.38 -0.336 -0.442 -0.856

BI891827 0.898 0.768 -1.067 -1.019 -0.5 0.111 -0.361 -0.488 -0.962 -0.929 -0.778 -0.124

AI667581 0.894 0.927 0.458 -0.276 -0.69 -0.149 -0.436 -1.147 -0.888 -0.393 -0.511 -0.428

BG729144 0.893 0.835 -0.359 -0.318 -0.401 -0.049 0.085 -0.449 -0.386 -0.66 -0.498 -0.718

AW154454 0.893 0.899 0.401 0.344 0.31 0.152 -0.201 -0.009 0.054 0.01 0.033 -0.244

AW117034 0.892 0.851 0.3 -0.015 0.122 0.204 -0.168 0.153 -0.292 -0.115 -0.406 -0.004

AF191561 0.892 0.25 0.319 0.337 0.068 0.524 0.141 0.611 0.226 0.519 -0.133 -0.092

BI980640 0.891 -0.66 -1.584 -1.172 -1.825 -1.098 -0.914 -1.637 -1.174 -2.28 -2.366 -1.978

AW077025 0.89 -0.077 0.522 0.279 0.416 0.745 0.647 0.09 0.478 0.257 0.31 0.546

BM095334 0.884 1.033 -0.351 -0.224 -0.117 0.026 -0.021 0.506 0.134 -0.311 -0.323 -0.349

BM181762 0.883 0.168 -0.007 0.188 -0.31 0.252 0.048 0.545 -0.28 0.182 -0.226 -0.224

U57964 0.878 -0.245 -0.017 0.493 0.026 0.417 0.15 0.17 -0.118 0.507 0.16 -0.448

BM102116 0.877 0.83 0.798 0.588 0.242 0.187 0.117 0.531 -0.06 -0.189 -0.125 -0.199

AW344046 0.873 0.133 -0.311 -0.636 -0.747 -0.226 -0.738 -1.097 -1.121 -0.229 -0.4 0.214

BM024816 0.873 -0.395 -0.282 -0.484 -0.141 0.15 -0.105 0.016 -0.152 0.054 0.224 0.166

BI672273 0.872 -0.147 -1.459 -0.593 -0.147 -0.57 -0.614 -0.622 -0.659 -0.302 -0.42 -0.507

BG729372 0.87 0.593 -0.231 -0.147 0.212 0.188 -0.297 0.616 0.128 -0.007 0.311 0.038

AI793558 0.87 0.349 -1.034 -0.618 -0.591 -0.193 -0.36 -0.769 -0.83 -1.25 -0.893 -0.68

BI983233 0.869 0.101 -1.359 -0.739 -0.343 -0.33 -0.272 -0.271 -0.368 -0.063 -0.307 0.136

BM181759 0.865 0.14 0.681 0.522 0.321 0.845 0.486 0.557 0.376 0.385 0.001 -0.153

AW059389 0.865 -0.556 -0.368 0.033 0.041 0.781 0.49 0.744 0.57 0.722 0.288 -0.513

AW116649 0.865 0.105 -1.39 -0.912 -1.147 -0.394 -0.646 -0.539 -0.381 -0.357 -0.151 0.05

BI880658 0.864 -0.897 -0.45 -0.358 -0.455 0.244 -0.327 0.004 0.067 0.412 0.153 0.17

AF397015 0.863 0.741 -0.132 -0.122 -0.33 -0.263 -0.677 -0.463 -0.457 -0.632 -0.612 -0.698

AW171576 0.862 0.887 0.644 0.048 -0.2 0.491 0.061 -0.104 -0.117 0.015 -0.222 -0.374

BM104302 0.861 0.725 -0.393 -0.432 -0.775 -0.077 -0.339 -0.521 -0.208 -0.119 -0.257 -0.33

AI666879 0.858 0.603 -0.342 -0.485 -0.247 -0.258 -0.448 -0.532 -0.784 -0.331 -0.405 -0.304

AW233564 0.852 -3.294 -3.229 -3.365 -2.646 -2.309 -2.979 -2.878 -3.898 -4.108 -3.754 -3.53

AI626681 0.847 0.235 -0.815 -0.482 -0.202 -0.066 0.042 0.032 -0.474 -0.406 -0.491 -0.495

AI883922 0.847 0.799 0.3 0.258 0.106 0.101 -0.093 0.31 0.141 0.832 0.497 0.142

AI942983 0.846 0.802 -0.384 -0.157 -0.159 -0.056 0.389 0.714 0.069 0.179 0.064 -0.11

AI793353 0.843 0.181 -0.168 -0.007 -0.776 -0.007 -0.356 -0.742 -0.52 -0.166 -0.051 -0.18

AI626336 0.843 0.172 -0.569 -0.205 -0.528 0.269 -0.191 0.064 -0.03 0.204 0.015 0.3

AW232088 0.841 0.341 -0.611 -0.374 -0.792 -0.018 -0.189 -0.586 -0.567 -0.163 -0.295 0.275

AI641491 0.839 0.841 0.761 -0.142 0.494 0.66 0.404 -0.005 0.196 0.261 0.252 0.557

AI617012 0.837 -0.386 -0.877 -0.816 -0.061 0.18 -0.206 0.218 -0.052 0.557 -0.018 -0.002

BG303725 0.835 0.957 -0.289 -0.107 -0.642 0.069 -0.486 -0.267 -0.244 -0.426 -0.252 -0.247

BI983378 0.832 0.685 0.463 0.348 -0.026 -0.009 0.041 -0.348 -0.343 -0.041 -0.616 -0.137

BM185242 0.828 -1.934 -3.851 -3.261 -3.715 -3.058 -3.714 -3.418 -3.859 -4.908 -3.251 -3.132

BI882464 0.828 0.735 0.208 0.082 -0.414 0.286 -0.135 -0.148 -0.261 -0.234 -0.329 -0.366

BI891871 0.828 0.401 -0.383 -0.227 0.364 0.415 -0.194 -0.043 -0.659 -0.762 -0.75 -0.56

AI965182 0.826 -0.049 0.467 0.266 0.173 0.157 0.168 0.668 0.005 0.195 -0.039 0.042

BM036431 0.826 -0.097 0.425 0.509 0.622 0.783 0.59 0.455 0.345 0.511 0.249 0.147

BM101574 0.822 -0.104 -1.582 -1.365 -0.929 -0.202 -0.73 -0.384 -0.348 -0.099 -0.133 -0.526

BI880357 0.822 -0.027 -0.364 0.062 0.405 0.398 0.107 -1.164 -0.805 -0.896 -0.84 -0.831

BM103911 0.819 0.465 -0.428 -0.587 -0.752 -0.038 -0.384 -0.863 -0.487 -0.155 -0.506 -0.535

BM036347 0.817 -0.151 -0.072 0.742 0.196 0.062 -0.087 0.327 0.067 -0.233 0.003 -0.243

BI891043 0.816 0.083 -0.195 -0.392 -0.346 -0.083 -0.154 -0.238 -0.197 0.145 0.045 0.039

AW777467 0.815 0.361 0.312 0.39 0.555 0.759 0.559 0.284 0.047 -0.044 -0.192 -0.074

BI888742 0.814 0.583 0.608 0.519 0.187 0.041 0.116 -0.115 -0.204 -0.376 -0.719 -0.635

AF262047 0.807 -0.201 -0.234 -0.248 -0.158 0.231 0.065 0.27 0.128 -0.263 0.09 -0.422

AW019847 0.804 -1.903 -1.283 -1.385 -1.313 -0.793 -1.041 -1.684 -1.312 -1.547 -1.094 -0.316

AI667411 0.802 0.79 -0.212 0.033 -0.122 0.015 -0.41 -0.099 -0.194 -0.524 -0.433 -0.636

AF277172 0.802 0.433 0.164 0.264 0.188 0.836 0.387 0.146 0.528 0.437 0.458 0.405

BI876830 0.795 0.666 0.735 0.684 -0.407 -0.326 -0.802 -1.161 -0.855 -1.196 -1.478 -1.345

BG303979 0.789 -1.53 -2.205 -2.106 -2.349 -1.738 -1.208 -2.789 -1.585 -2.387 -1.834 -2.223

BI888569 0.782 0.097 -0.415 -0.232 -0.575 -0.032 -0.043 -0.289 -0.112 0.112 -0.221 -0.17

BM071872 0.781 0.279 -0.563 -0.158 -0.91 -0.289 -0.611 -0.535 -0.591 -0.188 -0.198 -0.435

AI666886 0.778 0.36 -0.201 -0.142 -0.289 0.314 0.254 0.173 0.034 -0.255 -0.269 -0.216

BI980715 0.776 -0.191 -0.155 -0.845 -0.413 -0.052 -0.063 0.18 -0.198 -0.354 -0.38 -0.005

BI845763 0.767 0.324 -0.576 -0.119 -0.711 -0.372 -0.084 -0.433 -0.322 -0.352 -0.279 -0.472

BI867396 0.764 0.498 0.132 0.03 -0.24 0.268 -0.2 -0.588 -0.374 -0.515 -0.814 -0.469

AI416043 0.756 0.493 0.026 0.069 -0.016 0.21 0.294 0.277 0.049 -0.082 -0.275 0.052

BM183338 0.752 -1.383 -2.007 -1.548 -2.026 -0.962 -1.411 -2.394 -1.651 -1.135 -1.02 0.558

BI672468 0.751 -0.519 0.038 -0.274 -0.745 -0.132 -0.255 -1.037 -0.677 -0.222 -1.157 -0.932

AW115638 0.745 0.585 0.262 -0.263 -1.417 -0.388 -0.35 -1.33 -0.996 -0.49 -1.111 -1.234

AI601556 0.745 0.475 0.136 0.293 0.081 0.734 0.083 0.119 0.234 0.019 -0.319 -0.058

BI672378 0.741 -0.952 0.516 0.615 0.235 0.259 0.224 0.001 -0.25 0.56 0.177 -0.188

AW203026 0.741 -0.768 -1.06 -0.546 -1.031 -0.172 -0.164 -0.84 -0.449 0.136 -0.032 0.053

BI896491 0.739 0.452 -0.195 0.376 0.056 0.699 0.789 0.08 -0.16 -0.439 -0.877 -1.314

BM071061 0.736 -0.437 -0.517 -0.418 -0.621 0.208 -0.226 -0.134 -0.285 0.31 0.065 0.118

BI534308 0.736 0.363 -0.25 -0.004 -0.205 0.303 -0.383 -0.051 -0.214 0.263 0.238 0.122

BI866527 0.729 0.424 0.127 0.178 0.309 0.523 -0.042 0.104 0.095 -0.014 0.023 -0.04

AI884085 0.725 0.28 -0.807 -0.379 -0.578 0.084 -0.282 -0.445 -0.408 -0.519 -0.869 -0.79

BI879615 0.708 -2.781 -2.476 -2.08 -3.584 -2.575 -2.187 -3.87 -2.607 -3.835 -4.309 -4.2

BG305364 0.688 -0.196 -0.593 -0.88 -1.094 -0.133 -0.065 0.291 0.258 0.354 0.498 -0.144

BM155353 0.68 0.262 -0.449 -0.269 -0.541 0.166 -0.164 -0.598 -0.111 -0.21 -0.312 -0.412

Y12236 0.66 0.248 -0.083 -0.349 -0.664 -0.148 -0.557 -0.625 -0.313 -0.162 -0.503 -0.373

BI888359 0.647 0.467 0.066 0.602 0.469 0.61 0.202 -0.151 0.111 -0.095 -0.332 0.021

BI864067 0.606 -0.813 -0.41 -0.222 -0.563 -0.282 -0.398 -0.699 -0.573 0.204 0.169 0.115

AW134106 -0.512 -3.369 -3.237 -3.602 -4.37 -4.024 -4.196 -5.231 -4.961 -7.317 -6.735 -7.685

BI876262 -0.662 -3.149 -3.22 -3.553 -4.23 -4.031 -4.485 -5.109 -5.114 -7.37 -7.359 -8.24

Mean 1.313 0.129 -0.47 -0.41 -0.688 -0.241 -0.504 -0.733 -0.687 -0.825 -0.924 -0.923
