# Supplementary material for: Transcriptome Analysis of Zebrafish Embryogenesis Using Microarrays
Source: PLoS Genet. 2005 Aug 26;1(2):e29. doi: 10.1371/journal.pgen.0010029 (PMC1193535; doi:10.1371/journal.pgen.0010029)
Supplement: Dataset S14 — (130 KB DOC) [file pgen.0010029.sd014.doc]

Dataset S14. Genes exhibiting peak of expression at the blastula stage.

Genbank IDUF egg 3hpf 4.5hpf 6hpf 7.7hpf 9hpf 10.7hpf 12hpf 15hpf 24hpf 30hpf 48hpf

AA494728 0.905 1.376 1.165 0.795 0.872 0.814 0.468 0.475 0.212 -0.146 -0.509 -0.597

AA658720 0.137 0.936 0.821 0.012 -0.733 -0.332 -0.568 -0.189 -0.628 -0.729 -0.624 -0.844

AB055671 1.011 1.176 -0.136 0.145 -0.51 -0.15 0.044 0.203 -0.021 -0.249 0.163 -0.28

AF060499 0.643 1.249 1.105 0.866 0.787 0.341 0.162 0.811 -0.439 -0.103 0.074 -0.39

AF064830 0.399 1.211 0.842 0.69 -0.231 -0.448 -0.093 -0.306 -0.126 -0.548 -0.767 -1.147

AF097478 -0.025 0.727 0.721 0.679 0.298 -0.42 0.015 0.217 -0.098 0.04 -0.31 -0.311

AF097483 -0.384 1.324 1.059 1.136 -0.466 -0.171 -0.297 -0.008 -0.26 -0.662 -0.522 -0.44

AF124435 0.869 1.072 0.046 -0.31 -0.811 -0.108 -0.436 -0.414 -0.913 -0.625 -0.417 -0.507

AF127920 0.788 1.662 1.632 1.342 0.499 0.893 -0.042 0.46 -0.549 -0.313 -0.883 -1.224

AF195050 0.33 0.929 0.93 0.579 0.403 0.088 0.438 0.365 -0.034 -0.059 -0.527 -0.631

AF204241 -0.27 0.966 0.228 0.109 0.135 -0.215 0.129 0.623 0.242 -0.124 0.64 -0.123

AF231015 -0.31 0.895 0.459 0.552 0.208 0.326 0.056 0.866 0.206 0.049 0.178 -0.182

AI396688 0.536 1.344 1.175 1.173 0.422 0.086 0.129 0.228 0.242 -0.085 -0.16 -0.643

AI415871 0.282 0.921 0.922 0.662 0.063 -0.216 0.033 0.008 -0.212 0.015 -0.081 -0.203

AI436892 1.185 1.345 1.037 1.056 0.13 0.09 -0.16 -0.641 -0.551 -0.398 -1.064 -0.507

AI437156 0.241 1.11 1.123 0.715 0.416 0.806 0.066 0.232 -0.235 0.094 -0.211 -0.225

AI444335 -0.528 0.96 0.194 0.439 0.535 0.09 -0.022 0.335 -0.182 -0.059 -0.182 -0.109

AI545141 0.982 1.541 0.809 0.946 0.075 0.175 -0.115 0.387 -0.153 -0.233 -0.647 -0.889

AI584349 0.493 0.878 0.516 0.687 0.646 0.325 0.134 0.762 0.169 -0.005 0.19 -0.149

AI584415 0.934 1.18 -0.673 -0.737 -0.232 -0.123 0.004 -0.096 -0.212 0.067 -0.3 0.167

AI584429 0.567 0.574 0.576 0.352 0.212 0.024 -0.08 -0.094 -0.112 -0.29 -0.261 -0.546

AI584532 0.304 1.364 1.227 1.395 0.764 -0.28 -0.163 1.055 -0.022 0.084 0.038 -0.295

AI584541 0.293 1.216 0.6 1.118 0.843 0.59 0.44 0.994 0.097 -0.102 0.136 -0.291

AI585084 0.522 1.271 1.215 0.896 -0.205 -0.137 -0.285 0.447 -0.365 0.137 -0.391 -0.22

AI588114 0.516 1.384 0.958 0.223 0.39 0.374 0.027 0.578 -0.073 -0.21 -0.482 -0.325

AI588171 -0.184 1.299 0.44 0.592 0.761 0.115 0.124 0.532 0.068 -0.646 -0.077 -0.251

AI588306 0.836 1.115 0.767 0.327 0.154 0.501 0.488 0.592 0.741 -0.166 0.384 -0.191

AI588447 0.306 1.383 1.037 0.678 0.932 0.046 0.112 0.625 -0.174 -0.25 -0.216 -0.257

AI601514 -0.476 0.548 0.519 0.332 0.123 0.106 -0.191 0.108 0.128 -0.514 0.496 -0.1

AI641036 1.026 1.511 0.746 1.006 0.988 0.845 0.344 0.514 0.027 -0.007 -0.128 -0.526

AI657598 0.453 1.31 1.029 0.937 -0.38 -0.452 -0.456 0.031 -0.472 -0.066 -0.235 -0.347

AI658223 0.744 1.552 1.589 1.29 0.405 0.043 -0.028 -0.021 -0.013 -0.857 -0.449 -0.818

AI666873 0.674 1.331 1.17 0.901 0.662 -0.098 0.06 0.464 0.228 0.022 -0.02 -0.28

AI666881 -0.071 0.767 0.65 0.647 -0.301 0.005 0.029 0.182 -0.215 0.158 -0.236 -0.209

AI666897 -0.281 1.675 0.623 0.978 1.02 0.032 0.351 0.347 -0.084 -0.524 -0.304 -0.244

AI667252 0.168 1.377 0.815 0.948 0.604 -0.027 0.068 0.193 -0.113 -0.733 -0.47 -0.384

AI667279 0.844 1.161 0.77 0.299 -0.02 -0.137 -0.143 0.204 -0.039 0.164 0.095 -0.488

AI667526 0.307 0.986 0.644 0.706 0.42 0.135 0.213 0.714 0.012 0.129 0.089 -0.219

AI667626 1.113 1.152 1.181 0.907 0.256 0.17 0.007 -0.2 -0.401 -0.412 -0.673 -0.742

AI667682 0.836 1.832 0.835 0.363 0.557 -0.088 0.112 0.561 -0.059 -0.644 -0.264 -0.104

AI721654 0.128 1.127 0.585 1.04 0.697 0.505 0.335 1.177 0.109 0.014 0.309 -0.097

AI721869 -0.129 1.07 0.806 0.52 0.507 0.706 0.182 0.327 0.808 0.214 0.421 -0.067

AI722485 0.386 0.819 0.383 0.65 0.593 -0.235 0.086 0.376 0.115 -0.095 0.248 -0.064

AI722493 0.616 1.444 1.059 0.723 1.018 0.724 0.319 0.677 -0.073 -0.753 -0.31 -0.418

AI722566 0.999 1.258 0.202 0.073 -0.034 0.154 0.303 0.425 -0.213 -0.282 -0.462 -0.408

AI722733 -0.106 0.903 0.723 0.043 -0.064 -0.189 0.07 -0.191 0.045 -0.282 0.059 -0.411

AI793391 0.709 1.509 0.629 0.478 0.571 0.23 0.399 0.37 0.223 -0.159 -0.594 -0.423

AI793728 0.877 1.196 0.6 0.703 0.545 0.422 0.147 0.403 -0.209 -0.246 -0.428 -0.676

AI793802 0.768 1.114 0.897 0.498 -0.195 -0.214 0.036 0.281 -0.384 -0.397 -0.11 -0.727

AI793812 0.025 0.862 0.594 0.401 -0.039 0.319 0.364 0.879 0.244 -0.245 -0.358 -0.23

AI793838 0.412 0.933 0.779 0.781 0.921 0.269 0.382 1.032 0.084 0.329 0.063 -0.091

AI793850 0.579 1.202 0.767 0.003 -0.133 -0.285 0.266 -0.009 -0.184 -0.477 -0.415 -0.093

AI878437 0.036 0.769 0.151 0.259 0.422 -0.15 0.275 0.61 0.381 -0.046 0.465 -0.179

AI878608 -0.227 0.793 0.34 0.742 0.825 0.065 -0.217 0.399 0.093 -0.063 0.127 0.011

AI878632 0.403 1.073 0.516 0.777 0.679 0.337 0.012 0.443 -0.297 -0.066 0.023 -0.226

AI883356 0.544 1.301 1.268 0.853 0.186 0.601 0.024 0.142 -0.189 -0.112 -0.475 -0.411

AI942818 -0.031 1.327 0.329 0.662 0.924 0.595 0.354 1.212 0.079 -0.21 -0.155 -0.065

AI957464 0.752 1.585 1.036 0.628 -0.552 -0.075 -0.111 0.381 0.159 -0.229 0.403 0.196

AI957752 0.07 1.2 0.654 0.841 0.377 0.239 0.201 0.82 0.249 0.3 0.339 0.285

AI957821 0.43 0.677 0.225 0.565 0.522 0.376 0.123 0.378 0.327 0.1 0.069 0.104

AI957865 0.738 1.227 0.833 0.182 0.288 0.31 0.275 0.262 -0.251 -0.097 -0.47 -0.941

AI958147 0.45 0.798 0.476 0.577 0.253 -0.088 0.163 0.48 -0.026 -0.186 -0.317 -0.314

AI958665 0.066 1.398 0.203 -0.049 0.496 0.276 0.509 0.939 0.027 0.026 0.306 -0.269

AI964421 0.367 1.023 0.95 1.078 0.204 -0.3 -0.085 -0.407 -0.47 -0.107 -0.426 0.011

AI965037 0.371 1.138 1.113 0.658 0.328 0.242 0.154 -0.161 -0.27 0.257 -0.013 -0.117

AJ243959 0.616 1.45 0.76 0.62 0.063 0.283 -0.269 -0.172 -0.385 -0.596 -0.902 -0.607

AW019629 0.552 1.003 0.951 0.653 -0.485 -0.455 -0.044 -0.804 -0.602 -0.966 -1.066 -0.81

AW058706 -0.473 1.331 1.138 0.922 0.508 0.207 -0.298 -0.106 -0.554 0.097 -0.173 -1.137

AW058802 -0.62 0.929 0.639 0.638 0.951 -0.043 -0.223 0.153 -0.166 -0.038 0.002 -0.004

AW058803 0.16 1.284 0.897 0.253 0.225 0.447 0.15 0.588 0.183 0.002 -0.066 -0.514

AW078222 0.119 1.179 0.953 0.446 0.678 0.269 0.203 0.864 -0.008 -0.011 -0.178 -0.307

AW078404 -0.288 1.457 1.089 0.8 0.591 0.049 0.026 1.003 0.13 0.211 -0.074 -0.414

AW115906 0.708 1.252 0.985 0.565 -0.262 -0.253 -0.426 -0.368 -0.523 -0.599 -0.398 -0.666

AW115947 0.378 0.819 0.109 0.652 0.733 0.105 0.07 0.291 -0.082 -0.216 -0.628 -0.516

AW115964 -0.019 0.878 0.487 0.878 0.676 0.162 0.211 0.729 -0.003 -0.08 -0.039 -0.071

AW116199 0.814 1.747 1.383 0.467 -0.075 0.063 -0.46 -0.632 -0.567 -0.856 -0.766 -0.65

AW116335 0.275 1.49 0.448 0.784 0.719 0.18 0.111 0.504 -0.135 -0.104 -0.282 -0.171

AW116347 0.761 1.234 1.281 0.733 0.639 0.759 0.361 0.583 0.238 0.03 0.087 0.002

AW116392 0.729 0.985 0.716 0.259 -0.448 -0.326 -0.468 -0.526 -0.248 -0.373 0.012 0.003

AW116431 0.683 1.004 0.947 0.98 0.637 0.449 0.288 0.407 -0.165 0.003 -0.072 -0.471

AW116597 0.856 1.257 1.225 0.748 -0.2 -0.11 -0.205 0.046 -0.102 -0.491 -0.451 -0.643

AW116809 1.244 1.555 0.081 -0.554 -0.574 0.029 -0.208 0.396 -0.005 0.12 -0.09 -0.773

AW116874 0.26 1.203 0.89 0.105 -0.179 -0.102 0.012 -0.321 -0.53 -0.22 -0.675 -0.509

AW117104 1.161 1.396 -0.141 -0.434 -0.464 -0.388 -0.283 -0.585 -0.833 -0.849 -1.308 -0.768

AW117140 0.48 0.797 0.683 0.669 0.741 0.556 0.183 0.48 -0.089 -0.235 -0.428 -0.648

AW127926 0.003 1.013 0.873 0.574 -0.316 -0.32 0.004 -0.396 -0.046 -0.787 -0.061 -0.219

AW133729 0.401 0.685 0.655 0.481 0.331 0.471 -0.093 0.538 0.125 0.051 -0.233 -0.363

AW154162 -0.196 1.067 0.372 0.935 0.654 -0.236 -0.219 -0.021 -0.195 -0.672 -0.306 -0.389

AW154286 0.921 1.14 0.913 0.269 0.126 0.28 -0.026 0.46 0.33 0.231 0.037 -0.045

AW154302 0.175 1.214 0.817 0.174 0.167 -0.04 -0.034 0.666 0.074 -0.17 -0.221 -0.379

AW154399 1.185 1.504 0.974 0.307 -1.874 -0.669 -0.233 -2.166 -0.646 -1.495 -1.532 -2.07

AW154402 0.362 1.142 0.838 0.698 0.78 -0.089 -0.09 0.18 -0.119 -0.495 -0.272 -0.427

AW154475 0.265 1.451 0.938 0.853 0.755 0.114 -0.039 0.026 -0.14 -0.563 -0.249 -0.546

AW154476 0.452 1.243 1.149 1.014 0.389 0.789 0.435 0.528 0.542 0.305 0.328 0.48

AW165313 0.211 1.173 0.711 0.702 0.604 -0.09 0.089 0.57 -0.355 -0.06 -0.61 -0.444

AW170991 0.414 0.933 0.658 0.872 0.389 0.39 0.053 0.06 -0.344 -0.464 -0.747 -0.449

AW171143 0.458 0.757 0.761 0.674 0.676 0.37 0.324 0.523 -0.009 0.303 -0.138 -0.036

AW171194 0.852 1.014 0.941 0.331 -0.192 0.168 -0.008 0.37 -0.051 -0.173 -0.376 -0.271

AW171290 1.199 1.645 1.675 1.097 0.251 0.338 0.086 0.663 0.216 0.002 -0.348 -0.192

AW171303 0.494 1.253 1.126 1.175 0.608 -0.193 0.205 0.349 -0.096 0.213 0.04 -0.417

AW171529 -0.112 1.018 0.655 0.55 0.508 -0.162 0.444 0.561 0.164 -0.219 -0.338 -0.307

AW171610 0.648 1.157 0.717 0.865 0.995 0.76 0.391 0.733 0.118 -0.498 -1.472 -1.665

AW174486 -0.087 1.139 0.834 1.085 0.775 -0.043 0.278 0.353 0.252 -0.568 -1.792 -1.291

AW175150 -0.145 0.731 0.608 0.698 0.311 -0.326 0.07 0.363 -0.054 -0.042 -0.032 -0.333

AW184672 0.086 1.248 1.289 0.945 0.827 -0.024 0.165 0.605 -0.04 0.094 -0.158 -0.144

AW203156 0.495 2.18 1.15 0.953 0.1 -0.298 -0.353 0.201 -0.54 -1.056 -1.188 -0.982

AW232489 0.933 1.208 0.651 0.714 0.884 0.607 0.559 0.737 0.368 0.363 -0.13 0.04

AW232853 -0.71 0.545 0.407 -0.404 0.12 0.004 -0.161 -0.002 -0.029 -0.697 0.425 -0.062

AW233726 0.106 1.328 0.791 0.194 -0.037 -0.274 -0.389 -0.066 -0.045 -0.389 -0.039 -0.347

AW279908 0.672 1.295 0.741 -0.269 -1.085 0.024 -0.351 0.093 0.054 -0.023 -0.459 -0.014

AW280659 -0.503 1.147 1.155 0.693 0.525 -0.231 -0.043 0.659 -0.147 -0.426 -0.533 -0.182

AW282001 -0.074 0.996 0.622 0.959 0.842 0.143 -0.316 0.004 -0.163 0.256 0.227 0.652

AW306102 0.74 1.437 0.892 0.385 0.102 0.104 -0.078 0.437 -0.044 -0.191 0.03 -0.07

AW342850 0.844 1.331 1.112 0.808 0.552 0.714 0.569 1.262 0.275 0.295 -0.059 0.033

AW344134 1.057 1.184 0.578 -0.034 -0.308 -0.152 0.372 0.267 -0.135 -0.009 -0.36 -0.067

AW344202 0.972 1.505 1.089 0.725 -0.046 0.367 -0.389 -0.475 -0.6 -0.632 -0.517 -1.921

AW420722 0.435 1.226 0.829 0.418 -0.139 -0.295 -0.38 -0.363 -0.352 -0.043 -0.113 -0.226

AW421050 0.621 2.134 0.453 0.601 0.623 0.553 -0.206 -0.01 -0.65 -1.242 -1.216 -0.864

AW466547 0 0.844 0.585 0.399 0.388 0.523 0.13 0.355 0.133 -0.032 -0.045 0.149

AW466858 1.178 1.585 1.46 1.015 -0.308 -0.377 -0.48 -0.672 -1.026 -0.656 -0.969 -1.381

AW566530 -0.34 1.288 0.814 0.503 0.042 -0.141 -0.218 -0.001 -0.359 -0.816 -0.628 -0.914

AW594981 -1.293 1.091 1.113 0.773 0.379 -0.096 -1.062 -0.226 -0.082 0.236 0 -0.507

AW777445 0.893 1.011 0.417 0.803 0.299 0.522 0.495 0.322 -0.187 -0.231 -0.553 -0.922

AW777843 1.079 1.597 0.454 0.074 -0.843 0.024 -0.426 -0.597 -0.495 -0.801 -0.979 -0.774

AY029529 0.687 1.573 1.064 0.867 0.031 0.512 -0.089 0.891 0.673 0.317 -0.421 -1.255

BE016354 -0.281 0.824 0.741 0.73 0.835 -0.01 -0.049 0.944 0.295 0.328 -0.048 -0.483

BE605606 0.276 1.497 1.379 1.407 1.026 0.933 0.439 0.973 0.135 0.144 0.124 -0.05

BE605707 0.613 0.707 0.642 0.029 -0.631 -0.793 -0.346 -0.976 -1.318 -0.673 -0.651 -0.218

BE605810 0.634 0.683 0.696 0.387 0.316 0.451 0.111 0.321 0.192 0.058 -0.108 -0.231

BF156074 0.736 1.244 0.723 0.788 -0.43 -0.025 -0.289 -0.654 -0.653 -0.487 -1.046 -0.549

BF156260 0.618 1.642 1.423 1.133 0.058 0.173 -0.548 -0.188 -0.424 -0.424 -0.692 -0.688

BG302610 -0.093 1.203 0.808 0.796 -0.078 -0.254 -0.065 0.103 -0.223 -0.315 -0.464 -0.6

BG302813 0.888 1.348 1.144 0.562 0.081 0.42 0.123 0.321 -0.106 -0.078 -0.317 -0.706

BG302910 0.326 1.726 1.644 1.624 1.256 0.615 0.215 0.702 -0.241 -0.687 -0.628 -0.703

BG303491 0.157 1.119 1.183 0.674 0.061 0.035 -0.037 -0.072 -0.156 -0.449 -0.618 -0.417

BG303518 0.513 0.98 0.97 0.984 0.067 -0.409 -1.253 -0.966 -0.535 -0.281 -0.755 -0.224

BG304082 0.927 1.462 0.771 0.257 -0.462 -0.145 -0.518 0.158 0.023 -0.219 0.102 -0.572

BG304135 0.873 1.727 1.304 0.634 -0.977 -0.543 -0.462 -1.498 -1.05 -0.867 -1.482 -1.358

BG304162 0.108 1.173 1.14 1.117 -0.213 -0.043 -0.307 -0.834 -0.521 -0.628 -0.659 -0.366

BG305654 0.902 1.754 1.566 0.455 0.298 0.496 0.073 0.035 -0.164 -0.308 -0.291 -0.378

BG306148 0.321 1.378 0.969 1.107 1.166 0.904 0.39 1.26 0.489 0.198 -0.374 -0.692

BG307533 0.852 1.217 1.086 -0.058 -0.572 -0.038 -0.147 -0.791 -0.603 -0.61 -0.964 -0.539

BG727402 0.952 1.341 0.504 0.152 -0.465 -0.216 -0.745 -1.006 -0.743 -0.81 -0.942 -0.882

BG727480 0.071 1.015 0.825 0.171 0.176 -0.222 -0.098 -0.189 0.021 -0.201 0.043 -0.245

BG728726 -0.137 0.742 0.724 0.481 0.394 0.199 -0.062 -0.087 0.174 -0.003 -0.238 -0.348

BG729232 0.163 1.026 0.501 0.653 0.231 -0.039 0.042 0.846 -0.173 0.04 -0.149 -0.14

BG738899 0.523 1.205 0.837 0.627 -0.152 -0.265 -0.114 0.076 -0.193 -0.265 -0.228 -0.372

BG799622 0.351 1.413 0.998 1.025 0.549 -0.043 -0.037 -0.067 -0.184 -0.297 -0.565 -0.654

BG883210 0.995 1.26 0.206 0.631 -0.133 0.405 0.199 0.51 -0.079 0.228 -0.046 -0.289

BG985512 0.745 1.387 1.522 0.518 0.423 0.711 0.376 0.762 0.14 0.15 -0.095 -0.274

BG985833 0.033 1.04 0.049 0.022 0.531 0.566 0.271 0.889 0.179 0.197 -0.003 -0.115

BI325513 -0.091 1.452 0.759 0.826 0.153 -0.178 0.093 -0.04 -0.095 -0.04 -0.328 -0.219

BI430207 1.052 1.775 1.071 -0.03 0.13 0.495 0.192 0.65 0.095 -0.424 -0.473 -0.833

BI430332 0.57 1.856 1.886 1.5 1.138 0.01 -0.471 -0.5 -0.364 -0.948 -0.607 -0.001

BI473712 0.827 0.918 0.382 0.501 -0.195 0.355 0.233 0.156 -0.205 0.021 -0.282 -0.338

BI475104 0.664 0.749 0.097 0.236 0.101 0.355 0.327 0.426 -0.069 -0.203 -0.147 -0.197

BI533326 0.613 0.902 0.66 0.477 0.132 0.165 -0.208 0.491 0.069 0.306 0.177 0.05

BI534261 0.322 0.599 0.563 0.331 -0.068 0.22 0.171 0.243 -0.165 0.276 -0.202 -0.345

BI563035 0.012 1.03 0.775 0.162 0.003 -0.074 -0.24 -0.052 0.212 -0.255 0.043 -0.148

BI670841 0.672 1.113 0.774 0.849 -0.207 0.027 -0.254 0.022 -0.064 0.105 0.16 -0.305

BI671845 1.553 1.658 1.55 0.31 0.295 0.444 0.245 0.507 0.332 0.257 0.058 0.031

BI672025 1.221 1.519 1.11 1.252 0.744 1.022 0.599 0.918 0.149 0.187 -0.2 -0.306

BI672089 0.881 1.08 -0.11 0.126 0.26 0.096 0.133 0.334 0.088 -0.407 0.183 -0.41

BI672216 -0.045 1.33 0.642 1.018 0.624 -0.203 0.235 0.527 -0.114 -0.397 -0.742 -0.106

BI672450 0.428 1.467 0.897 0.866 0.429 -0.325 -0.219 0.198 -0.058 -0.221 -0.211 -0.3

BI672475 -0.308 1.059 0.67 0.706 -0.155 -0.314 0.009 0.206 0.036 -0.14 -0.106 -0.172

BI673408 0.042 0.574 0.613 0.072 -0.179 0.075 0.052 -0.248 -0.023 0.202 -0.239 -0.049

BI673511 0.491 1.015 0.671 0.949 0.362 -0.158 -0.11 -0.945 -0.842 -0.72 -1.533 -0.89

BI673527 0.227 1.081 0.595 0.709 0.983 0.602 0.299 0.944 0.335 0.41 0.475 0.027

BI673663 0.599 1.362 1.025 0.838 0.373 0.054 0.156 0.839 0.786 0.08 0.416 -0.413

BI673727 0.895 1.288 1.008 0.978 0.59 0.466 0.177 0.758 -0.088 -0.368 -0.479 -0.768

BI704181 0.044 0.77 0.39 0.605 0.324 -0.226 -0.049 0.842 -0.02 -0.041 -0.433 -0.452

BI704240 0.701 1.191 1.142 1.114 1.191 0.801 0.948 0.971 0.007 0.175 -0.387 -0.856

BI704353 0.195 0.77 0.576 0.695 0.664 0.052 0.13 0.133 -0.04 -0.005 0.111 0.301

BI704401 0.369 0.973 0.851 0.954 0.276 0.068 0.3 -0.106 -0.351 -0.268 -1.035 -0.819

BI705512 -0.279 1.584 1.209 0.988 0.115 -0.431 -0.232 -0.161 -0.43 -0.749 -1.225 -0.843

BI705519 1.161 1.77 1.023 0.858 0.341 0.405 0.027 0.178 0.154 -0.261 0.317 -0.246

BI705646 -0.174 1.013 0.355 0.439 0.149 -0.225 0.006 0.625 0.032 -0.252 -0.111 0.004

BI706873 0.174 0.771 0.55 0.745 0.323 0.275 0.243 0.802 0.11 0.42 -0.024 -0.039

BI706999 -0.365 0.989 0.135 0.789 0.906 0.159 0.296 0.841 -0.04 0.104 0.392 -0.182

BI708483 -0.421 2.093 1.921 1.611 0.45 0.286 -0.407 -0.455 0.044 -0.296 -0.033 -0.83

BI709411 0.918 1.315 1.12 0.687 0.221 0.014 -0.143 0.03 -0.07 -0.068 -0.006 0.085

BI710035 0.837 1.271 0.675 0.1 -0.476 -0.174 -0.2 -0.413 -0.364 -0.406 -0.235 -0.549

BI840279 -0.202 1.538 1.277 1.279 0.686 0.192 0.012 0.373 -0.125 -0.371 -0.234 -0.348

BI843105 0.52 1.179 0.885 0.737 0.522 0.425 0.296 0.822 0.068 0.245 0.109 -0.201

BI843291 0.457 1.247 0.854 0.549 -0.49 -0.346 -0.399 -0.614 -0.641 -0.334 -0.793 -1.003

BI843298 0.861 1.404 1.052 0.612 -0.001 -0.148 -0.277 0.02 -0.176 -0.37 -0.473 -0.553

BI844046 0.564 1.457 1.152 0.778 0.651 1.195 0.776 1.011 0.308 0.335 0.051 -0.1

BI845927 0.803 0.913 0.868 0.394 -0.153 0.232 -0.094 0.241 -0.061 -0.225 -0.416 -0.332

BI864033 0.205 0.897 0.792 0.574 -0.042 -0.224 -0.342 -0.022 0.102 -0.199 -0.059 -0.348

BI867308 0.302 1.156 0.323 0.782 0.769 -0.087 0.081 0.45 0.299 -0.246 -0.357 -0.411

BI867407 1.485 2.125 1.904 1.13 0.111 0.305 0.019 -0.021 -0.328 -0.584 -0.572 -0.713

BI867483 0.802 1.196 0.731 0.589 -0.863 -0.264 0.247 -0.807 -0.372 -0.36 -1.038 -1.039

BI876694 0.454 1.229 0.492 0.363 -0.882 -0.775 -1.486 -1.907 -1.89 -1.892 -2.598 -3.032

BI877500 1.468 1.803 -0.483 -0.277 -0.508 -0.201 -0.393 -0.203 -0.699 -0.828 -0.63 -1.058

BI877620 1.054 1.548 0.789 0.166 -0.004 0.482 0.274 0.524 0.207 0.195 -0.253 -0.141

BI877634 0.485 1.149 1.15 1.01 0.049 0.03 -0.356 -0.122 -0.182 -0.839 -0.416 -0.408

BI877644 0.315 1.161 0.948 0.75 0.51 -0.24 -0.245 -0.056 -0.274 -0.884 -0.52 -0.609

BI877751 0.64 1.163 0.751 0.765 -0.375 -0.102 -0.315 0.05 0.055 0.594 0.16 -0.481

BI877974 0.446 1.149 0.908 0.372 -0.457 -0.365 0.017 -0.28 -0.439 -0.581 -0.877 -0.623

BI878013 0.457 1.604 1.021 0.812 0.795 0.325 0.266 0.587 0.517 -0.256 0.311 0.045

BI878280 1.446 1.704 0.4 -0.341 -2.037 -0.408 -1.154 -1.416 -0.872 -1.841 -2.539 -1.641

BI878611 1.154 1.838 1.662 1.131 0.078 0.278 -0.547 -0.208 -0.615 -0.91 -1.139 -1.231

BI878714 -0.337 0.867 0.639 0.643 0.181 0.053 -0.08 -0.364 -0.565 -0.784 -0.478 -0.756

BI878849 -0.106 1.278 1.121 0.985 0.12 -0.319 -0.075 -0.419 0.123 -0.474 -0.41 -0.413

BI878876 0.16 0.81 0.722 0.818 0.596 0.29 0.338 0.715 0.328 0.182 -0.077 -0.057

BI878928 0.829 1.19 0.789 0.632 -0.151 -0.078 -0.758 -1.287 -1.112 -0.756 -1.003 -1.079

BI879432 0.94 1.574 0.178 0.341 0.129 0.209 0.506 0.986 0.693 0.742 0.514 0.205

BI880767 0.995 1.284 0.645 0.452 0.091 0.401 0.137 0.465 -0.037 0.346 0.381 0.368

BI883215 0.839 1.63 0.609 0.559 0.412 0.144 -0.06 0.121 -0.071 -0.736 -0.564 -0.665

BI884413 -0.552 0.689 0.753 0.098 -0.059 -0.11 0.064 -0.03 -0.128 -0.055 -0.045 0.15

BI885257 0.317 1.114 0.837 0.746 1.016 1.001 0.52 0.908 0.143 0.009 -0.066 -0.598

BI885759 0.323 1.415 -0.552 -0.584 -0.899 -0.057 0.104 1.002 0.483 0.523 0.053 -0.811

BI885890 -0.815 0.844 0.274 0.54 0.669 0.651 0.212 0.819 -0.07 0.194 0.325 0.406

BI885914 -0.16 0.843 0.778 0.367 -0.141 -0.027 0.26 0.633 0.149 0.777 0.301 -0.085

BI886249 0.853 1.247 1 1.227 0.995 0.901 0.742 0.975 -0.098 0.114 -0.054 -0.274

BI886268 0.298 1.464 0.925 0.817 0.375 -0.342 0.011 -0.139 -0.304 0.185 -0.156 -0.667

BI886468 0.184 1.203 0.593 0.573 0.232 0.027 -0.049 0.223 -0.207 -0.08 -0.118 -0.692

BI886473 0.026 1.105 0.665 0.647 -0.01 -0.29 -0.032 0.571 0.089 0.361 0.238 -0.213

BI886552 -0.289 0.728 0.666 0.52 0.693 0.072 0.261 0.546 0.122 -0.072 -0.118 -0.293

BI886664 0.833 1 0.686 0.654 -0.214 -0.29 -0.09 -0.672 -0.673 -1.103 -1.022 -1.205

BI886699 1.327 1.71 0.138 0.237 -0.291 -0.06 -0.367 -0.827 -0.419 -1.118 -1.372 -1.72

BI886728 -0.14 0.992 0.781 0.796 0.411 0.277 0.011 0.661 0.24 0.286 0.158 -0.001

BI886736 0.753 1.382 0.423 0.145 0.192 0.188 -0.188 -0.291 -0.589 -1.112 -0.844 -0.859

BI887138 0.77 0.896 0.734 0.496 0.536 0.227 0.133 -0.329 -0.505 -0.048 -0.258 -0.271

BI887141 -0.247 0.57 0.318 0.327 0.354 0.368 -0.157 0.501 -0.223 0.406 0.103 0.141

BI887157 0.172 1.249 0.998 1.117 0.266 -0.208 -0.496 0.015 -0.199 -0.33 -0.148 -0.444

BI887534 0.352 1.231 0.862 0.256 0.132 0.057 -0.31 -0.067 -0.24 -0.111 -0.169 -0.13

BI887658 0.211 1.057 0.978 0.54 0.273 0.223 0.043 0.193 0.263 -0.362 -0.374 -0.889

BI887791 0.738 1.293 1.156 1.081 0.836 0.71 0.63 0.767 -0.072 0.001 -0.011 -0.246

BI887958 0.464 1.133 0.872 0.211 -0.447 -0.21 -0.37 0.231 -0.064 -0.189 -0.211 -0.42

BI888372 0.725 1.168 1.007 0.511 -0.158 -0.295 -0.046 -0.368 -0.158 -0.236 -0.314 -0.55

BI888449 0.026 1.131 1.103 0.729 0.821 0.593 0.282 0.397 -0.184 -0.046 -0.428 -0.615

BI888634 0.106 1.083 0.656 0.915 0.425 0.456 0.118 0.59 0.062 0.055 -0.159 -0.111

BI888683 -0.632 0.797 0.037 0.421 0.149 -0.322 -0.142 0.831 0.103 -0.39 -0.365 -0.323

BI888703 0.684 1.38 1.363 0.573 0.692 0.864 0.564 0.782 -0.027 -0.156 -0.171 -0.188

BI888760 0.672 1.167 0.563 0.433 0.043 0.322 -0.382 -0.638 -0.443 -0.346 -0.706 -0.358

BI888884 -0.18 0.823 0.584 -0.025 -0.046 -0.143 0.001 -0.181 -0.327 -0.068 -0.136 -0.417

BI888982 0.147 1.702 1.479 0.853 0.32 0.529 -0.012 0.06 -0.379 -0.337 0.084 -0.989

BI889009 1.34 1.756 0.641 0.268 -0.145 -0.155 -0.339 -0.34 -0.597 -0.845 -0.85 -1.089

BI889686 0.057 1.039 0.966 0.785 0.26 -0.118 0.136 0.375 -0.104 -0.234 -0.601 -0.657

BI889705 0.052 1.062 0.61 0.708 0.016 -0.176 0.046 0.124 -0.422 0.199 0.012 -0.153

BI889943 1.191 1.845 1.753 0.194 -2.17 -1.242 -1.987 -2.778 -2.313 -3.369 -2.553 -2.505

BI889958 1.049 1.784 1.048 1.518 0.719 0.31 0.376 0.186 -0.524 -0.163 -1.109 -1.515

BI890317 0.907 1.334 0.661 0.684 0.887 0.319 -0.008 0.726 0.108 -0.027 0.191 -0.091

BI890693 0.424 1.442 0.663 0.326 0.037 0.448 -0.22 0.309 -0.411 -0.177 0.318 -0.251

BI890854 0.371 1.144 0.514 0.741 0.824 0.367 0.688 0.528 -0.058 0.093 -0.129 0.053

BI890925 -0.205 1.093 0.887 0.727 0.203 -0.215 -0.207 0.28 -0.155 -0.507 -0.381 -0.322

BI891293 0.318 1.373 0.3 0.389 0.516 0.134 0.364 0.996 0.263 -0.115 -0.015 0.116

BI891592 -0.541 0.615 0.074 0.469 0.594 0.098 -0.19 0.222 -0.068 -0.203 -0.352 0.084

BI892022 0.249 1.378 0.928 0.99 0.237 0.599 0.047 -0.022 0.009 -0.298 -0.148 -0.509

BI979995 -0.058 1.458 0.824 0.292 0.302 -0.05 -0.293 0.046 -0.375 -0.32 -0.47 -0.592

BI980047 0.476 1.218 0.803 0.856 0.721 -0.168 0.24 0.581 -0.108 0.235 -0.371 -0.312

BI980311 0.263 1.871 1.294 0.481 0.13 -0.209 -0.392 -0.045 0.032 -0.494 -0.14 -0.497

BI980457 0.65 1.106 0.657 -0.158 -0.941 0.068 0.236 -0.876 -0.279 -0.306 -0.834 -0.506

BI983256 -0.301 1.148 0.74 1.059 0.034 -0.249 -0.324 -0.597 -0.124 -0.315 -0.601 -0.454

BI983375 0.644 1.354 1.386 0.665 0.79 0.12 0.316 1.031 0.216 0.305 0.432 -0.162

BI983651 1.396 1.544 1.019 0.72 0.348 -0.089 -0.302 0.143 -0.147 -0.334 -0.395 -0.823

BI984775 0.018 1.323 0.712 0.508 -0.903 -0.548 -0.922 -1.145 -0.459 -0.642 -1.024 -0.775

BM034949 0.873 1.219 0.564 0.576 0.562 0.032 -0.179 0.077 -0.201 -0.177 -0.249 -0.373

BM036098 -0.328 0.981 0.712 0.495 -0.475 -0.324 -0.324 -0.399 -0.472 -0.731 -0.686 -0.621

BM036121 0.889 1.196 0.639 0.802 0.167 0.13 -0.109 -0.308 -0.345 -0.628 -0.326 -0.236

BM036281 1.025 1.646 0.694 0.33 0.148 0.183 0.035 0.286 -0.323 -0.357 -0.312 -0.374

BM036413 0.545 1.391 1.094 0.395 0.601 0.753 0.745 1.372 0.845 0.579 0.5 -0.044

BM036903 0.787 1.148 1.16 0.691 -0.365 -0.076 -0.177 -0.114 -0.264 0.1 -0.149 -0.672

BM071784 0.269 1.345 0.706 0.336 -0.131 -0.115 0.073 0.035 -0.178 -0.157 -0.422 -0.355

BM071795 1.468 1.794 0.222 -0.353 -0.881 0.116 -0.711 -1.126 -0.948 -1.227 -2.103 -1.228

BM072375 1.085 1.561 0 -0.003 -0.675 -0.276 -0.785 -0.536 -0.549 -0.604 -0.782 -0.626

BM095814 0.308 1.275 1.199 0.801 -0.11 -0.233 0.264 0.039 -0.162 -0.254 -0.44 -0.493

BM096050 1.195 1.243 1.35 0.426 0.405 0.469 0.205 0.777 0.305 0.371 0.031 -0.245

BM096076 -0.099 0.897 0.234 0.828 0.669 -0.269 -0.253 0.285 0.26 0.344 0.434 -0.119

BM101576 0.215 1.364 1.021 0.846 0.56 -0.304 0.269 0.372 0.233 -0.376 -0.17 0.008

BM101621 1.379 1.582 1.018 0.03 -0.247 0.095 -0.171 -0.274 -0.155 -0.522 -0.36 -0.396

BM101681 1.161 1.613 1.433 0.71 0.327 0.549 0.335 0.639 0.459 0.397 0.146 -0.319

BM101834 0.213 1 0.637 0.75 0.959 0.286 -0.143 0.359 0.459 -0.218 -0.204 -0.47

BM102093 -0.623 0.783 0.696 0.179 -0.452 -0.219 -0.473 -1.031 -0.08 0.353 0.721 0.5

BM102580 1.581 1.936 1.209 0.605 0.016 0.087 -0.022 0.012 -0.3 -0.197 -0.931 -1.174

BM103793 0.58 0.907 0.65 0.887 0.559 0.533 0.451 0.491 -0.09 -0.115 -0.592 -0.883

BM103866 0.382 0.981 0.9 0.375 0.377 -0.145 0.22 -0.334 -0.035 -0.948 -0.25 -0.352

BM103889 0.791 1.039 0.57 1.029 0.742 0.855 0.667 0.948 0.073 0.206 -0.077 -0.07

BM103906 0.845 1.867 0.809 0.495 0.151 -0.023 -0.089 0.38 0.213 -0.634 -0.259 -0.361

BM103941 -0.032 1.017 0.364 0.602 0.522 -0.315 0.281 0.862 0.117 0.175 0.063 0.188

BM104076 0.621 1.25 0.655 0.787 0.839 0.508 0.386 1.001 0.34 0.292 0.227 -0.02

BM104103 0.365 1.387 0.996 0.847 0.078 -0.245 -0.181 -0.594 -0.543 -0.498 -1.069 -0.702

BM104112 0.011 0.637 0.42 0.421 0.435 0.05 0.095 0.206 0.001 0.05 0.021 0.121

BM104267 -0.293 0.947 0.345 0.691 0.832 -0.483 0.09 0.505 -0.096 -0.504 -0.076 -0.295

BM104296 1.094 1.434 1.586 0.389 -0.952 -0.637 -0.767 -1.236 -1.305 -1.353 -1.537 -1.398

BM104369 0.492 1.089 1.022 0.394 0.308 0.304 0.041 0.026 -0.125 -0.199 -0.282 -0.609

BM104753 0.88 1.93 1.629 1.228 0.164 0.483 -0.399 -0.526 -0.381 -0.913 -0.882 -0.736

BM154193 0.602 0.75 0.008 0.315 0.187 0.22 -0.263 0.829 -0.177 -0.06 0.078 -0.173

BM156550 0.04 1.385 1.374 0.444 0.013 0.008 -0.153 0.032 -0.096 -0.445 -0.103 -0.316

BM156904 1.519 1.754 1.823 1.037 0.593 0.343 0.315 0.029 -0.749 -0.686 -1.323 -1.466

BM157299 0.174 0.724 0.195 0.197 0.596 0.066 0.153 0.762 0.043 -0.24 -0.138 0.03

BM171814 0.738 1.744 1.43 1.095 -0.119 0.05 -0.143 -0.261 -0.643 -0.602 -0.81 -0.571

BM182031 1.182 1.341 1.14 -0.144 -0.286 -0.12 -0.103 -0.228 -0.372 -0.696 -0.715 -1.094

BM182332 0.832 1.071 -0.498 -0.978 -1.239 -0.735 -0.555 -0.551 -0.588 -0.627 -0.683 -0.672

BM182430 -0.031 1.432 1.176 0.875 0.267 0.261 -0.219 0.353 -0.296 -0.29 0.198 -0.207

BM182738 0.263 0.735 0.72 0.561 0.698 0.61 0.147 0.489 0.141 -0.154 -0.158 -0.207

BM183234 0.662 1.152 1.105 0.824 0.726 0.346 0.25 0.649 0.09 0.308 -0.163 -0.077

BM183276 0.88 1.012 0.628 0.216 -0.168 0.196 -0.034 0.362 0.021 0.26 0.186 0.242

BM183288 0.019 1.423 0.911 0.264 0.518 0.08 0.029 0.303 -0.083 -0.232 -0.093 -0.34

BM183764 1.021 1.289 0.838 0.21 -0.035 0.475 -0.058 0.004 -0.175 -0.14 -0.499 -0.808

BM183908 0.667 0.867 0.604 0.601 -0.31 -0.14 0.01 -0.062 -0.042 -0.038 -0.358 -0.362

BM184075 1.329 1.528 0.479 -0.295 -0.763 -0.463 -1.033 -1.399 -1.231 -2.388 -2.028 -2.502

BM184104 0.21 1.465 0.746 0.44 -0.75 -0.1 -0.279 -1.407 -0.786 -0.713 -1.656 -0.97

BM184209 0.312 1.747 0.753 0.697 0.222 0.287 0.072 0.194 -0.029 -0.262 -0.873 -0.711

BM184447 1.721 1.887 1.047 0.476 -0.509 -0.273 -0.312 -0.947 -0.896 -1.472 -1.26 -1.557

BM185338 -0.028 1.311 0.723 0.405 -0.441 -0.397 -0.24 -0.564 -0.562 -0.535 -0.359 -0.567

L03399 0.281 1.193 0.377 0.678 0.944 0.306 -0.039 1.158 0.231 -0.021 -0.492 -0.395

U62619 0.205 0.983 0.749 0.285 -0.198 0.441 -0.195 0.074 -0.548 -0.425 -0.619 -0.217

Y14534 -0.728 0.545 0.407 0.05 0.071 -0.125 -0.012 0.603 -0.31 -0.34 0.527 -0.274

AA497153 0.898 1.285 1.849 1.78 1.427 1.846 0.946 1.186 0.549 0.465 -0.015 -0.013

AA605842 0.952 1.336 2.382 1.403 1.262 0.922 0.479 0.003 0.408 0.044 0.14 -0.034

AB034245 -0.279 0.263 2.344 1.875 0.737 -0.218 0.546 0.743 0.302 -0.137 0.447 -0.088

AB046866 0.68 1.272 1.588 1.545 1.532 1.395 0.652 1.077 0.266 0.22 0.171 0.586

AF030519 0.123 0.83 1.112 0.655 0.152 0.208 -0.124 0.333 0.2 0.314 0.121 0.009

AF038425 -0.638 0.587 1.993 1.417 1.369 0.893 -0.376 0.952 0.489 0.445 0.027 -0.079

AF041440 0.472 0.361 2.184 1.274 0.959 0.785 0.036 0.352 -0.076 -0.072 0.003 -0.223

AF064838 0.643 0.903 1.055 0.823 0.39 0.21 0.12 -0.068 -0.079 -0.229 -0.464 -0.483

AF130459 0.289 0.842 1.003 0.424 0.263 0.788 0.144 0.03 0.001 -0.242 -0.248 -0.013

AF137535 0.459 0.735 1.14 0.771 0.545 0.089 -0.298 0.223 -0.055 -0.398 -0.352 -0.747

AF157109 -1.102 1.007 2.918 1.854 2.338 0.978 0.095 1.098 -0.088 0.816 0.294 0.49

AF161270 0.001 0.522 0.667 0.309 0.305 0.057 0.093 0.477 0.541 -0.004 -0.006 -0.468

AF184244 -0.061 0.885 2.183 1.788 1.437 1.317 1.156 1.633 0.8 0.815 0.223 0.068

AF193837 -0.796 1.757 4.28 3.725 3.714 2.698 2.596 2.427 1.815 -0.419 0.163 -0.243

AF196346 -0.133 0.74 0.778 0.712 0.175 0.224 -0.353 0.334 0.057 0.001 -0.172 -0.187

AF201379 -0.728 0.249 2.5 1.948 1.345 0.289 -0.219 0.772 -0.314 -0.66 -0.286 -0.666

AF212941 0.145 1.433 1.728 1.306 0.464 -0.28 -0.282 -0.132 -0.343 -0.436 -0.464 -0.881

AF234784 0.81 1.692 2.084 1.562 0.464 0.17 -0.465 -0.333 -0.806 -0.907 -1.084 -1.411

AF239925 -1.014 -0.124 2.182 1.543 1.62 1.576 0.569 0.975 0.234 0.629 0.412 0.579

AF259079 0.545 0.932 1.834 1.395 1.757 1.042 1.132 1.541 0.489 0.889 0.62 -0.202

AF260240 -0.447 0.631 1.263 0.615 -0.477 -0.543 -0.138 -0.135 -0.077 -0.698 -0.651 -0.884

AF262978 -0.293 0.998 2.602 2.105 1.805 0.57 0.658 1.481 0.2 0.314 0.186 -0.193

AF263000 0.112 1.685 2.116 2.205 0.315 -0.371 -0.151 0.49 -0.277 -0.59 0.13 -0.201

AF359242 0.709 0.588 1.74 0.887 0.648 1.013 0.309 0.346 -0.073 -0.134 -0.174 -0.126

AI384221 -0.222 -0.472 1.555 0.331 0.7 0.25 0.13 0.828 -0.031 0.082 -0.067 0.24

AI396886 -0.605 0.756 0.957 0.94 0.726 -0.098 -0.164 0.544 0.081 0.034 -0.198 -0.367

AI477285 0.628 0.641 1.044 0.961 0.821 0.41 0.789 0.53 0.612 0.025 -0.661 -0.588

AI497075 -0.088 0.586 0.724 0.535 0.26 0.087 0.019 0.274 -0.072 -0.101 -0.299 -0.511

AI558578 0.226 0.556 1.976 1.461 1.677 0.812 0.577 0.898 0.458 -0.123 0.091 -0.098

AI584659 -0.743 0.455 1.246 0.759 1.286 -0.028 -0.023 0.183 0.183 -0.132 0.281 0.14

AI588214 -1.46 -0.374 2.555 2.093 1.883 1.455 1.76 1.256 1.42 1.021 0.888 0.227

AI588286 0.133 0.279 2.754 2.045 1.857 0.516 0.404 1.659 0.485 0.696 0.235 -0.125

AI588305 0.832 0.944 1.161 0.551 0.677 0.444 0.146 0.465 0.083 -0.158 -0.39 -0.77

AI588384 0.745 0.789 1.707 1.099 1.384 0.936 0.505 0.649 0.076 0.073 -0.164 -0.475

AI588480 0.727 0.917 1.964 1.464 1.467 1.137 1.192 1.179 0.775 0.765 0.32 -0.01

AI601325 -0.327 0.791 1.425 1.251 1.055 -0.006 0.25 0.323 -0.077 0.261 0.116 -0.282

AI601396 -0.23 0.406 0.524 0.341 0.175 0.176 0.26 0.193 0.117 -0.324 0.486 0.186

AI601527 -0.085 0.633 0.897 0.732 0.478 0.135 0.222 0.801 0.188 0.198 -0.05 -0.044

AI601691 -0.186 0.459 0.586 0.521 0.445 -0.308 0.147 0.135 -0.017 0.114 0.554 0.204

AI626420 1.582 1.488 1.703 0.916 0.988 0.973 0.694 1.035 0.251 0.243 0.015 -0.01

AI641708 0.365 0.545 0.748 0.344 0.619 0.135 0.036 0.212 -0.012 -0.112 0.201 -0.307

AI667165 0.534 0.388 1.383 0.915 0.941 1.114 0.683 0.614 0.251 0.279 0.014 -0.179

AI667202 0.468 0.044 0.816 0.466 0.418 0.256 0.336 0.709 0.435 0.681 -0.054 0.096

AI667275 -2.328 0.627 3.249 2.456 1.78 1.655 1.485 1.596 1.414 1.142 0.612 0.355

AI667625 0.769 0.677 1.273 1.029 1.067 0.934 0.639 1.062 0.404 0.446 -0.33 -0.482

AI667677 0.596 1.117 1.655 1.416 0.896 0.19 0.335 0.726 0.542 0.318 0.246 -0.066

AI721423 0.92 1.255 1.642 1.212 0.644 1.226 0.4 0.439 0.202 0.078 -0.187 -0.113

AI721511 0.333 0.605 0.804 0.736 0.406 0.837 0.256 0.794 0.453 0.675 0.234 -0.151

AI721701 -0.609 0.226 2.295 1.464 1.557 1.793 1.413 1.64 0.911 0.79 0.274 0.192

AI721710 -0.463 0.089 1.633 1.161 1.462 0.609 0.517 1.037 0.547 0.386 0.367 0.123

AI722499 1.196 1.737 2.443 0.999 0.448 0.499 -0.107 -0.099 -0.04 -0.372 -0.597 -0.942

AI723212 -0.539 0.104 2.374 1.695 1.807 0.869 1.168 1.458 0.72 0.742 0.47 0.293

AI793540 0.485 0.636 0.7 0.38 0.223 0.231 0.045 0.241 0.083 0.222 0.061 -0.107

AI793714 0.553 1.052 1.352 0.917 0.528 0.093 0.099 0.843 -0.128 0.234 0.134 -0.087

AI793793 0.507 0.592 0.87 0.266 0.418 0.385 0.169 0.645 0.284 0.063 0.006 -0.457

AI816701 -0.073 0.766 0.798 0.771 -0.881 -0.454 -0.688 -1.313 -0.605 -0.299 -0.106 0.452

AI878204 0.568 1.196 1.234 1.047 0.988 0.785 0.292 0.781 0.313 0.236 -0.124 -0.291

AI878772 1.016 1.282 1.535 1.406 0.877 1.055 0.345 0.265 -0.29 -0.558 -0.646 -0.771

AI878793 0.123 0.627 0.815 0.532 -0.078 -0.226 -0.576 0.121 0.15 -0.001 -0.078 -0.019

AI882827 0.673 1.117 1.17 1.181 0.977 0.445 0.318 0.693 0.092 0.071 -0.057 -0.12

AI883327 0.04 1.476 1.581 0.821 0.531 0.568 -0.086 0.609 0.537 0.142 -0.053 -0.402

AI942594 0.072 0.816 1.031 0.466 0.589 0.605 -0.045 0.57 0.202 0.146 -0.293 -0.247

AI943044 0.472 1.073 1.229 1.11 0.778 0.289 0.271 0.858 0.177 0.009 -0.107 -0.251

AI957572 0.906 1.041 1.139 0.797 -0.13 0.405 -0.149 -0.001 -0.566 0.059 -0.302 -0.32

AI957777 0.857 1.104 1.306 1.211 0.884 0.487 0.395 0.591 -0.057 -0.605 -0.571 -0.535

AI957915 0.164 1.527 2.586 1.4 1.227 0.909 0.357 0.107 0.249 0.014 0.116 -0.187

AI958084 -0.777 0.517 1.364 0.935 0.905 0.754 -0.134 0.939 0.535 0.686 0.476 0.392

AI959637 0.624 0.113 1.226 0.719 0.877 0.761 0.639 0.432 -0.179 -0.17 -0.24 -0.14

AI964328 1.112 3.178 4.891 2.989 0.824 -0.252 -0.121 -0.286 -0.435 -0.831 -0.734 -0.426

AI965120 0.153 1.223 1.455 1.373 1.001 0.581 0.282 0.843 0.025 0.048 -0.008 -0.246

AI974149 -0.031 -0.04 1.084 0.61 0.767 -0.095 0.322 0.448 0.166 0.441 0.157 0.069

AJ011788 0.619 1.141 1.292 0.869 -0.37 -0.287 -0.066 -0.183 -0.485 -0.679 -1.158 -1.508

AJ243250 0.046 0.866 1.561 1.149 1.143 0.88 0.381 0.223 -0.523 -0.44 -0.716 -0.957

AJ249795 0.231 0.867 1.278 0.793 0.97 1.013 0.245 1.386 0.646 0.805 0.503 0.624

AJ250201 -0.465 0.997 1.175 0.866 -0.002 -0.156 -0.47 0.115 -0.253 0.246 -0.141 -0.323

AW018718 0.013 1.052 1.283 1.191 -0.186 -0.227 -0.701 -0.599 -0.388 -0.254 -0.366 -0.507

AW018770 0.309 1.425 1.461 1.457 0.993 1.033 0.741 0.727 0.288 0.214 0.184 -0.129

AW018979 1.316 1.397 1.538 1.077 0.986 1.217 0.358 0.425 -0.201 -0.103 -0.565 -0.503

AW019436 -1.357 -0.408 2.255 1.612 0.906 0.977 0.436 0.189 -0.404 -0.342 -0.48 -0.512

AW058848 -1.052 0.186 3.297 2.22 2.094 1.557 1.237 1.749 0.962 1.064 0.499 0.263

AW059478 -0.246 -0.09 0.507 0.402 0.485 -0.222 0.056 0.4 0.134 0.1 -0.138 -0.256

AW077778 0.372 1.039 1.834 1.258 0.908 0.016 -0.302 0.494 0.044 0.284 0.026 -0.031

AW078193 -0.438 0.926 1.48 0.658 0.052 -0.17 -1.025 0.036 -0.247 -0.447 0.041 -0.742

AW078302 -0.465 0.127 1.287 1.056 0.895 0.3 0.306 1.026 0.363 -0.601 0.806 0.141

AW115718 0.813 0.414 0.976 0.468 0.673 0.611 0.202 0.169 -0.049 -0.185 -0.412 -0.486

AW116032 0.548 0.744 1.127 0.645 -0.145 0.144 -0.366 0.008 0.413 0.042 -0.122 0.009

AW116090 0.746 1.291 1.488 1.336 0.826 1.496 1.09 1.48 0.806 0.486 -0.019 -0.023

AW116521 0.679 1.152 1.518 1.212 0.109 0.304 -0.144 0.29 0.167 -0.271 0.01 0.013

AW116650 1.008 1.289 2.383 1.871 1.733 2.319 1.484 1.497 1.007 0.734 0.153 -0.116

AW116767 0.963 0.787 1.125 0.896 -0.053 0.344 0.164 -0.216 -0.173 -0.418 -0.775 -0.327

AW116777 0.483 0.564 0.783 0.465 0.718 0.643 0.46 0.704 0.443 0.209 -0.155 -0.311

AW116894 -0.225 0.64 1.084 0.928 1.076 -0.261 0.228 0.055 -0.089 -0.21 -0.461 -0.579

AW116961 -0.429 0.252 3.512 1.77 2.755 0.844 0.361 0.941 -0.002 -0.316 0.176 -0.174

AW128619 0.543 0.606 1.385 1.183 1.088 1.038 0.815 1.049 0.201 0.303 -0.34 -0.783

AW153364 0.194 1.017 1.793 1.419 0.771 0.601 0.061 0.09 -0.212 -0.285 -0.181 -0.359

AW154108 1.053 0.927 1.547 0.639 0.404 -0.142 -0.853 -0.406 -0.395 0.044 -0.054 -0.037

AW154514 -0.395 0.101 1.26 0.7 1.042 0.418 0.337 0.624 0.178 -0.524 -0.034 -0.23

AW170915 0.542 1.247 1.481 0.801 0.56 0.447 0.03 0.038 -0.388 -0.534 -0.634 -0.889

AW171207 0.371 0.868 1.165 0.654 0.235 -0.288 -0.328 0.218 0.026 -0.188 -0.463 -0.389

AW171211 0.778 1.126 1.244 1.056 0.574 0.792 0.369 0.675 0.047 -0.119 -0.669 -0.612

AW171224 0.312 0.739 0.796 0.543 0.163 0.238 0.332 0.284 -0.191 -0.181 -0.542 -0.277

AW171255 -0.01 0.855 1.727 1.58 1.637 -0.06 0.169 -0.129 -0.179 -0.628 -0.674 -0.362

AW171553 0.482 0.639 0.954 0.763 0.75 0.478 0.117 0.528 -0.143 -0.024 -0.33 -0.256

AW174328 -0.615 0.976 1.027 0.676 -0.363 -0.612 -0.658 -0.895 -0.616 -1.02 -0.849 -0.973

AW174421 -0.562 0.348 1.362 0.922 0.607 0.061 -0.033 0.14 -0.229 -0.146 -0.15 0.484

AW174733 1.082 0.901 1.574 1.111 0.473 0.167 -0.074 -0.244 -0.323 -0.803 -0.456 -0.008

AW174734 0.651 1.306 2.28 1.029 0.474 0.051 -0.012 -0.138 -0.155 -0.572 -0.39 -1.09

AW175518 0.196 1.263 1.285 1.23 1.027 0.29 0.262 1.011 0.028 0.112 0.026 0.043

AW202926 -0.028 0.784 1.333 1.064 1.092 0.494 0.392 0.487 0.19 0.019 -0.283 -0.564

AW232047 0.162 0.368 1.299 0.968 0.63 -0.195 0.116 -0.195 -0.147 -0.225 -0.261 -0.238

AW232262 0.244 0.868 1.114 0.746 0.138 -0.289 0.461 0.101 0.109 -0.107 -0.425 -0.531

AW233729 0.635 0.924 1.224 0.826 0.592 0.689 0.217 0.669 0.443 0.682 0.468 0.344

AW280046 0.294 0.76 0.787 0.698 -0.069 -0.109 -0.003 -0.354 -0.288 0.06 -0.389 -0.067

AW281340 0.434 1.061 1.596 1.22 0.675 0.527 0.231 0.478 0.318 -0.051 -0.157 -0.299

AW282022 -0.345 0.448 0.881 0.756 0.711 -0.211 0.061 0.04 0.195 0.286 0.099 -0.236

AW343655 0.768 0.707 1.229 0.911 0.739 1.115 0.482 0.397 0.096 0.011 -0.133 0.002

AW343865 0.456 0.599 0.999 0.622 0.511 0.838 0.116 0.639 0.137 0.202 -0.164 -0.233

AW343882 0.664 0.685 1.047 1.037 0.717 0.438 0.363 0.509 -0.128 0.064 -0.449 -0.467

AW344188 0.894 1.143 1.469 1.2 0.399 0.758 0.238 0.51 -0.226 -0.103 -0.242 -0.533

AW420509 0.611 1.387 1.783 1.157 0.963 0.988 0.545 0.148 0.066 -0.548 -0.167 -0.199

AW422278 -0.024 0.724 1.243 0.39 0.071 -0.097 -0.523 0 0.135 -0.254 -0.149 -0.652

AW466488 0.751 1 1.801 1.346 1.138 0.967 0.84 0.983 0.187 0.19 0.099 -0.305

AW567528 0.356 1.032 2.052 1.919 1.707 1.959 1.026 1.204 0.782 0.636 -0.105 -0.307

AW826500 -0.471 0.19 1.579 1.403 0.996 1.471 0.904 1.046 0.549 0.847 0.475 0.573

BE016510 0.394 0.231 1.453 0.653 0.719 1.141 0.573 0.59 0.002 0.517 -0.087 0.095

BE017477 1.274 1.173 1.633 1.043 0.793 1.059 0.866 0.967 0.727 1.326 0.815 0.075

BE201677 -0.662 1.211 3.703 2.578 0.621 0.317 0.03 -0.09 -0.047 0.186 0.675 0.159

BE201992 0.373 0.877 1.143 0.556 -0.1 -0.183 -0.275 -0.781 -0.407 -0.724 -0.661 -0.563

BE693122 1.142 0.854 1.336 1.318 0.73 0.97 0.412 0.819 0.545 0.824 0.347 -0.22

BF156211 -1.505 -1.153 2.498 1.714 1.457 0.687 1.094 0.794 1.45 0.947 0.843 0.094

BF718003 0.266 1.065 1.568 0.967 -0.125 -0.266 -0.172 -0.391 -0.601 -0.756 -0.455 -0.537

BG302584 0.225 0.565 0.689 0.461 -0.393 -0.469 -0.628 -0.448 -0.483 -0.078 -0.239 -0.258

BG302674 1.074 1.094 1.272 0.939 0.838 1.098 0.326 0.411 -0.021 0.023 -0.195 -0.316

BG302974 0.167 0.846 1.555 1.451 1.208 -0.137 0.014 0.313 -0.095 0.143 -0.057 -0.495

BG303243 -0.457 0.57 1.462 1.313 0.822 0.859 0.719 0.475 0.284 0.089 -0.004 -0.023

BG303445 0.419 1.221 1.287 1.125 0.572 0.673 0.484 0.957 0.131 0.013 -0.4 -0.467

BG303835 1.474 1.151 1.873 1.368 1.147 0.512 0.089 0.311 -0.337 -0.704 -0.797 -0.567

BG728569 -0.114 0.877 0.963 1.032 -0.237 -0.493 -1.027 -1.652 -0.96 -0.011 -0.03 -0.144

BG883878 0.555 1.441 1.97 1.813 1.187 0.53 0.081 0.576 0.296 -0.326 -0.326 -0.101

BG884388 -0.723 -0.117 2.244 1.73 1.501 0.554 0.479 0.513 0.402 0.381 0.476 0.558

BG985472 -0.181 0.283 2.434 1.823 1.871 1.142 0.834 0.845 1.207 0.27 0.454 0.165

BG985475 -0.031 1.133 1.756 1.674 1.365 0.812 0.632 0.845 0.1 0.063 -0.092 0.515

BI326788 -0.119 0.556 0.843 0.787 0.688 0.244 0.284 0.587 0.376 -0.201 -0.495 -1.002

BI350885 0.28 1.382 1.62 1.457 1.615 0.407 0.726 1.427 0.193 -0.434 0.035 -0.303

BI430095 0.891 0.511 1.464 0.963 0.994 0.552 0.045 0.457 -0.202 -0.246 -0.464 -0.503

BI430221 0.115 0.723 0.828 0.789 0.476 -0.02 -0.164 -0.255 -0.039 -0.456 -0.711 -0.872

BI474897 0.498 0.828 0.965 0.952 0.15 0.344 0.368 0.845 0.094 0.356 -0.101 0.021

BI672781 0.394 0.915 1.893 0.84 1.125 0.726 0.389 0.795 0.427 0.396 0.268 0.276

BI704306 0.651 0.732 1.348 0.204 0.337 0.384 -0.095 0.007 0.092 -0.205 -0.412 -0.536

BI704311 0.132 0.78 1.303 1.045 0.71 0.186 -0.725 -0.163 0.388 -0.338 0.088 -0.469

BI704416 0.519 0.628 1.282 1.21 1.071 0.25 0.443 0.904 0.105 0.3 0.008 0.143

BI704420 -0.745 0.815 3.803 2.866 1.771 1.245 1.156 1.671 1.334 0.652 0.593 0.217

BI705495 0.542 0.451 0.935 0.459 0.38 0.703 0.184 0.313 0.036 0.184 -0.08 -0.037

BI707547 -0.244 0.363 1.932 1.169 0.996 0.469 0.229 0.353 0.051 -0.055 -0.442 -0.228

BI707695 0.19 0.603 0.692 0.423 0.45 -0.166 -0.337 0.05 -0.088 0.326 0.11 -0.003

BI709715 0.317 0.536 0.873 0.52 0.635 0.295 0.323 0.717 0.04 0.362 0.157 0.014

BI710499 -0.685 0.793 1.127 0.78 0.957 -0.202 -0.232 0.294 0.062 -0.165 -0.027 -0.076

BI840738 1.678 1.31 2.136 1.531 1.004 1.276 0.808 0.763 0.403 0.23 0.007 -0.502

BI842428 1.108 1.221 1.687 1.107 0.482 0.888 0.57 1.164 0.268 0.736 0.544 0.065

BI845254 -0.043 0.062 0.657 0.481 0.547 0.322 0.324 0.238 0.41 -0.118 -0.263 -0.038

BI845638 -0.136 0.287 1.001 0.478 0.798 0.45 0.07 0.436 0.189 0.168 0.062 -0.149

BI846219 -0.828 0.556 1.608 0.45 1.403 0.097 0.279 0.744 0.729 0.36 0.645 -0.455

BI850015 -0.511 -0.138 2.214 1.239 1.222 0.389 0.339 1.019 0.236 1.155 0.701 0.261

BI864018 0.576 0.546 1.606 1.329 0.796 0.934 0.57 0.862 0.35 0.018 0.081 0.004

BI865765 -0.074 0.963 1.134 0.364 0.134 -0.041 -0.401 -0.391 -0.389 -0.642 -0.538 -0.568

BI866264 0.513 1.244 1.441 0.723 0.437 0.48 0.361 0.871 0.004 0.207 -0.072 -0.031

BI876181 1.701 1.078 3.005 2.817 2.091 2.166 1.413 1.558 0.437 0.058 -0.408 -0.907

BI876503 -0.316 0.557 1.475 1.189 1.421 0.106 -0.032 0.812 0.375 0.379 -0.014 -0.209

BI876589 -0.502 0.18 0.686 0.427 0.503 0.261 0.254 0.029 0.217 -0.004 -0.393 -0.958

BI877686 0.543 0.566 0.951 0.539 0.427 0.373 0.095 0.113 0.269 -0.071 0.014 -0.117

BI878039 0.97 0.931 1.915 1.486 1.435 1.6 0.525 0.667 0.446 0.372 0.176 0.17

BI878403 0.068 0.411 1.062 0.902 0.763 0.626 0.399 0.418 0.249 0.454 0.514 0.635

BI878578 0.208 0.671 1.271 0.793 0.675 1.223 0.673 0.731 0.79 0.446 0.072 -0.51

BI878741 0.682 0.791 1.219 0.85 0.856 0.365 0.033 0.414 -0.141 -0.295 -0.466 -0.336

BI882529 -0.27 0.573 1.164 0.863 0.944 0.107 0.255 0.188 0.202 0.065 0.201 0.136

BI883229 0.041 0.819 1.021 0.497 -0.079 -0.103 -0.219 0.012 0.236 -0.28 0.297 0.1

BI883421 -0.406 1.008 2.374 2.075 1.027 0.457 0.204 0.221 0.151 0.05 0.053 -0.336

BI883928 0.262 1.22 4.936 2.901 2.733 0.544 0.439 0.549 0.36 -0.082 0.242 -0.103

BI885983 -0.191 0.308 0.552 0.516 0.512 -0.218 0.059 0.49 0.466 -0.079 0.383 -0.2

BI885984 0.925 1.511 2.227 1.52 1.497 1.244 0.75 1.031 0.33 0.22 -0.048 -0.43

BI886089 -0.177 0.793 1.158 0.865 0.539 -0.064 0.052 -0.192 -0.007 0.048 -0.642 -0.33

BI886106 -0.253 0.481 2.597 0.911 1.567 0.313 0.327 0.961 0.616 0.517 0.525 -0.014

BI886271 -0.491 1.412 4.23 3.43 3.297 1.383 0.952 1.988 0.327 -0.141 0.873 -0.03

BI886276 1.328 1.234 1.582 1.32 1.54 1.056 0.602 1.624 0.492 0.483 0.083 -0.102

BI886303 0.009 0.631 2.1 1.063 1.253 0.871 0.588 0.796 0.299 0.312 -0.202 0.271

BI886316 -0.135 -0.195 2.099 1.21 1.556 1.077 1.115 0.818 0.435 0.921 1.179 0.837

BI886511 0.476 0.444 0.739 0.547 0.473 0.574 -0.198 0.138 -0.266 -0.063 -0.418 -0.05

BI886513 0.89 0.971 1.078 0.808 0.842 0.735 0.247 0.614 -0.311 0.197 -0.343 0.067

BI886597 -0.023 0.552 0.737 0.601 0.473 0.301 0.449 0.334 0.234 0.058 -0.435 -0.453

BI886711 -0.356 1.031 1.28 0.687 0.413 -0.125 0.111 0.184 -0.015 -0.462 -0.012 0.134

BI886755 -0.254 -0.202 1.927 1.059 1.618 1.11 0.431 1.159 0.175 0.069 -0.066 -0.056

BI886819 0.74 1.782 3.097 1.887 1.388 0.604 0.322 0.156 0.086 0.076 0.058 -0.246

BI887326 0.236 0.58 1.552 1.209 0.682 0.12 -0.22 0.207 0.243 -0.121 -0.314 -0.548

BI887570 -0.205 0.791 1.502 1.342 1.096 0.689 0.564 0.339 -0.255 -0.242 -0.816 -0.913

BI887628 0.023 0.859 0.995 0.643 0.016 0.654 0.308 0.293 0.279 -0.389 -0.182 -0.302

BI887742 -1.164 -0.028 3.072 2.684 1.29 1.018 1.191 1.77 1.082 1.139 0.474 0.31

BI887847 0.61 0.965 1.268 1.177 0.788 0.474 0.541 0.807 0.043 0.088 -0.388 -0.576

BI887957 -0.319 1.279 1.837 1.362 0.52 0.039 0.191 0.589 0.113 0.094 -0.003 -0.114

BI888179 -0.168 1.003 2.581 2.108 1.087 -0.176 0.143 0.165 -0.032 -0.319 0.321 0.211

BI888206 1.461 1.396 2.219 1.609 1.414 1.25 1.101 0.779 0.194 0.034 -0.116 -0.013

BI888210 -0.991 -0.05 1.499 1.138 1.236 0.979 0.842 1.017 1.206 0.714 0.384 0.015

BI888263 -0.033 1.661 2.115 1.385 -0.152 -0.173 -0.463 -1.141 -0.82 -1.362 -1.642 -1.449

BI888534 0.469 1.019 1.081 0.698 0.916 0.602 0.338 1.171 0.151 0.342 0.074 -0.112

BI888571 -0.493 1.216 1.862 1.432 1.118 0.348 0.206 0.317 0.379 -0.085 -0.108 -0.324

BI888816 0.368 0.368 1.371 1.172 1.188 0.97 0.997 0.938 0.889 0.516 0.109 -0.597

BI888818 -0.666 0.335 1.273 1.023 0.845 0.774 -0.621 0.361 0.431 0.262 0.013 0.188

BI888822 0.077 1.03 1.05 1.025 0.409 -0.277 0.234 0.19 -0.291 -0.095 -0.565 -0.638

BI888826 0.564 1.014 1.78 1.049 1.154 0.216 -0.05 0.13 -0.128 -0.378 -0.47 -0.525

BI888888 0.045 0.742 1.514 1.41 1.144 0.886 0.166 0.394 0.024 -0.195 -0.286 -0.242

BI888891 -0.279 -0.312 2.211 1.804 1.677 0.503 0.306 1.008 0.27 -0.243 0.248 -0.088

BI889005 0.607 1.205 1.788 1.673 1.66 1.733 0.775 1.253 0.53 0.265 -0.535 -0.968

BI889335 0.207 0.789 1.229 1.095 0.593 0.04 0.038 -0.088 0.004 -0.413 -0.048 -0.346

BI889402 0.723 1.149 1.236 0.916 0.551 0.062 -0.026 0.532 -0.219 0.337 0.303 -0.187

BI889437 1.23 1.628 2.012 1.855 0.916 1.126 0.843 0.626 0.221 0.06 -0.038 -0.731

BI889458 0.105 1.181 1.389 1.255 1.171 0.306 0.09 0.293 -0.124 -0.038 -0.035 -0.127

BI889627 -0.039 0.941 1.118 0.996 0.469 0.126 0.096 -0.092 -0.151 -0.09 -0.459 -0.041

BI889650 0.438 1.067 1.281 0.763 0.803 0.438 0.238 0.582 0.162 0.323 0.054 0.07

BI889676 -0.771 1.396 1.979 1.773 1.542 1.467 1.292 0.314 0.546 -0.379 -0.011 -0.824

BI889720 0.607 0.707 1.975 1.545 1.711 1.667 1.362 1.708 0.544 0.681 0.159 0.032

BI889835 0.873 0.175 1.498 1.008 0.741 0.017 -0.131 -0.728 -0.733 -1.715 -1.066 -1.089

BI889995 0.019 1.405 1.454 1.386 0.922 0.001 -0.053 0.021 -0.254 -0.756 -0.389 -0.927

BI890193 0.552 1.677 2.238 1.779 1.911 1.577 0.694 0.75 -0.155 -0.609 -0.415 -0.67

BI890214 -0.682 0.938 2.635 1.725 1.109 0.925 -0.2 0.332 0.255 -0.229 -0.164 -0.526

BI890323 -0.93 0.55 2.61 2.294 1.78 1.489 0.673 0.948 1.209 0.698 0.121 -0.114

BI890453 -0.405 1.09 2.373 1.776 1.967 1.465 0.729 1.175 0.975 0.795 0.33 0.16

BI890491 1.196 1.385 2.264 2.165 1.656 2.236 1.508 1.57 0.692 0.555 -0.196 -0.43

BI890519 0.387 0.479 1.25 1.042 1.153 0.319 0.2 0.694 0.08 0.005 -0.356 -0.542

BI890612 0.882 1.243 2.185 1.494 1.417 1.814 1.16 0.908 0.319 0.473 0.16 0.787

BI890616 -0.683 0.169 1.564 1.324 1.028 0.294 -0.358 0.549 0.601 0.422 0.407 0.192

BI890830 1.213 0.815 1.713 1.184 1.153 0.908 0.319 0.227 -0.472 -0.43 -0.377 -0.767

BI890871 0.533 0.783 1.165 0.696 0.783 0.988 0.318 0.706 0.342 0.199 -0.254 -0.375

BI890894 -0.014 0.729 0.873 0.761 0.552 -0.064 0.219 0.811 0.071 -0.115 -0.042 -0.342

BI890940 -0.477 0.503 3.17 1.253 1.054 0.067 0.369 0.45 0.307 -0.539 0.341 -0.005

BI891054 -0.189 0.929 1.88 1.628 1.095 -0.023 0.943 0.516 0.177 -0.081 0.095 -0.26

BI891112 0.9 0.906 1.087 0.958 0.055 -0.17 -0.394 0.098 -0.244 -0.056 -0.244 -0.38

BI891329 0.151 0.973 1.346 1.332 0.983 0.745 0.545 0.757 0.33 0.217 -0.373 -0.441

BI891349 0.915 1.122 1.248 0.596 0.443 0.446 0.045 0.191 -0.052 -0.714 -0.454 -0.534

BI891411 0.446 0.646 0.753 0.547 0.646 0.515 0.382 0.295 0.054 -0.218 -0.245 -0.296

BI891474 -0.476 0.126 1.735 0.384 1.426 0.708 0.321 1.024 0.427 0.358 0.085 -0.124

BI891613 0.668 0.117 1.86 0.887 1.113 1.343 0.571 1.131 0.586 0.333 0.101 -0.096

BI891699 0.075 0.496 1.583 1.297 1.078 1.085 0.883 0.29 0.295 0.342 0.044 0.396

BI891752 -0.503 0.089 1.557 1.099 0.615 0.683 -0.149 -0.307 -0.536 -0.482 -0.378 0.322

BI891913 0.947 0.334 1.316 0.761 0.966 1.184 0.791 1.014 0.328 0.406 -0.556 -0.818

BI892054 -0.039 0.818 1.396 1.119 0.747 0.339 -0.016 0.745 0.134 0.479 0.371 0.578

BI892084 0.71 1.288 1.955 1.846 1.246 1.206 0.762 0.843 0.27 0.466 0.12 0.079

BI892136 -0.429 1.513 4.304 2.872 2.969 1.261 0.449 0.97 -0.184 -0.333 0.03 -0.542

BI892197 0.278 0.613 0.72 0.518 0.117 -0.137 -0.066 -0.056 0.085 -0.022 -0.383 -0.513

BI897147 -0.668 0.222 1.372 1.041 0.628 0.21 0.296 -0.368 0.868 0.151 0.477 -0.879

BI897366 0.309 1.495 1.65 1.644 1.004 0.332 0.296 0.971 0.309 0.426 -0.05 -0.125

BI979759 1.308 1.806 2.13 1.881 1.702 1.409 1.129 1.755 0.542 0.785 0.287 -0.199

BI983014 0.041 0.509 1.625 1.424 1.195 0.767 1.079 0.691 0.039 0.53 0.065 0.009

BI983343 1.452 1.414 2.103 1.472 0.111 -0.271 -1.308 -1.384 -1.904 -2.021 -2.775 -2.796

BI983488 0.226 1.02 1.233 0.908 0.801 0.687 0.156 0.807 0.111 -0.197 -0.209 -0.223

BI984291 0.522 0.821 1.08 1.053 0.955 0.724 0.656 0.919 0.212 0.367 -0.221 -0.528

BI984492 0.101 0.831 1.606 1.407 0.746 0.945 0.546 0.591 0.112 -0.313 -0.364 -0.55

BI984759 0.009 0.177 1.007 0.867 0.806 0.65 0.257 0.317 0.259 0.253 0.069 0.12

BM023676 0.178 1.272 1.636 1.504 1.584 0.229 0.367 0.833 0.066 0.089 -0.107 -0.045

BM024193 0.444 0.525 0.596 0.558 0.637 0.147 0.214 0.254 -0.121 0.145 -0.212 -0.007

BM025872 0.401 0.677 0.816 0.557 0.67 0.339 0.062 0.843 -0.133 0.221 -0.081 -0.067

BM026057 0.237 0.232 0.688 0.415 0.461 0.258 0.263 0.55 -0.209 -0.097 -0.291 0.042

BM026462 0.725 1.134 1.362 1.084 0.783 0.764 0.214 0.712 0.349 -0.148 -0.062 -0.662

BM035545 1.044 1.33 1.427 1.049 0.285 0.425 0.043 0.491 -0.087 -0.826 -0.336 -0.474

BM036471 0.053 0.175 1.121 0.406 0.437 0.356 0.291 0.768 0.113 -0.333 -0.129 -0.089

BM037469 1.409 1.968 2.456 1.735 1.479 1.078 0.706 0.696 -0.095 -0.237 -0.577 -0.98

BM080877 0.566 1.184 1.327 1.168 0.501 -0.22 0.378 0.086 -0.236 -0.665 -0.876 -0.668

BM095865 -0.281 0.525 3.001 1.331 0.999 0.209 0.606 0.222 -0.225 -0.328 0.032 -0.155

BM095877 1.301 2.068 2.347 1.552 0.302 -0.641 -0.914 -1.311 -1.177 -2.19 -1.225 -1.367

BM095884 -0.163 0.068 1.236 1.132 0.592 0.81 0.689 0.528 0.159 0.476 0.464 -0.018

BM096116 0.039 0.766 0.817 0.509 -0.707 -0.366 -0.167 -0.481 -0.091 -0.394 -0.326 -0.382

BM103051 0.571 1.334 1.552 0.538 -0.805 -0.495 -0.388 0.262 -0.162 -0.167 -0.031 -0.656

BM103785 0.72 1.241 1.618 0.363 0.935 1.205 0.479 1.173 0.605 -0.015 -0.042 -0.262

BM104124 1.508 1.031 2.577 1.373 0.732 -0.067 -0.024 -0.504 -0.55 -1.014 -0.348 -0.489

BM104313 0.464 0.981 1.419 1.209 0.845 0.242 0.46 0.32 -0.402 -0.415 -0.321 -0.396

BM154897 1.317 1.16 1.704 1.689 0.704 0.488 0.115 0.3 0.231 -0.342 -0.38 -0.765

BM156822 0.62 0.758 0.904 0.486 0.893 0.621 0.054 0.558 0.03 -0.436 -0.148 -0.064

BM156854 0.497 1.025 1.353 0.592 0.404 0.436 0.342 0.769 0.12 0.185 -0.018 -0.059

BM156878 0.476 1.001 2.003 1.591 1.392 0.984 0.556 0.252 0.74 -0.014 0.186 -0.061

BM181650 1.313 1.287 1.875 1.825 1.332 1.162 0.354 0.532 -0.12 -0.026 -0.615 -0.973

BM181685 0.927 1.05 1.375 0.875 0.685 0.52 0.389 1.035 0.439 0.19 -0.154 -0.166

BM181726 0.572 1.063 1.125 0.822 0.899 0.676 0.159 0.758 0.422 0.151 0.359 0.179

BM182386 1.214 1.141 1.787 1.62 0.95 1.159 0.293 0.258 0.095 0.273 -0.205 -0.229

BM183463 -0.43 1.337 1.725 1.397 0.758 -0.136 -0.032 0.284 0.166 0.232 0.136 -0.263

BM184161 -0.492 0.147 2.595 2.185 1.085 0.126 0.616 0.97 0.65 0.18 0.36 -0.013

BM185145 0.608 0.993 1.629 1.291 1.26 0.869 0.732 0.872 0.007 -0.043 -0.508 -0.28

BM185263 0.388 0.893 1.376 1.016 0.286 0.821 0.467 0.512 0.073 0.261 0.025 -0.217

BM185382 0.36 0.904 1.23 0.615 0.976 0.927 0.768 0.93 0.367 0.137 -0.029 0.053

L03395 0.157 0.961 1.465 1.267 0.916 0.385 0.02 0.76 -0.145 -0.02 -0.157 -0.142

L11711 0.308 0.8 0.914 0.738 0.563 0.475 0.093 0.358 0.382 0.052 -0.212 -0.515

L48017 -0.663 0.613 2.432 1.413 1.983 0.003 0.132 1.409 0.154 -0.116 0.493 -0.27

S68799 -0.221 0.37 1.468 0.803 1.194 1.231 0.403 0.965 -0.091 -0.024 -0.2 -0.096

U27121 -0.338 0.099 3.223 1.031 1.12 0.289 0.106 0.544 0.114 0.063 -0.148 -0.386

U57965 0.779 1.136 1.253 1.004 0.342 0.234 -0.378 -0.452 -0.465 -0.486 -0.528 -1.331

U60804 0.164 0.584 1.205 0.629 0.585 0.206 0.392 0.877 0.047 0.287 -0.026 0.004

U85091 0.308 1.041 1.604 1.169 0.639 -0.014 -0.209 0.011 -0.417 0.375 0.455 0.939

X83594 1.515 1.564 2.156 1.635 -0.206 -0.108 -0.701 -0.762 -1.16 -1.984 -1.912 -1.837

X84224 1.632 1.718 3.393 3.219 2.202 1.232 -0.26 -1.81 -1.918 -2.715 -2.928 -2.551

Y12819 -0.767 0.864 1.209 1.133 0.514 -0.065 -0.619 0.024 -0.082 -0.209 -0.888 -0.586

Y16043 0.579 0.601 1.043 0.84 -0.021 -0.046 -0.762 -0.818 -1.057 -0.672 -0.412 -0.478

Mean 0.341 1.023 1.195 0.868 0.527 0.311 0.129 0.328 -0.009 -0.123 -0.221 -0.353
