# Supplementary material for: Transcriptome Analysis of Zebrafish Embryogenesis Using Microarrays
Source: PLoS Genet. 2005 Aug 26;1(2):e29. doi: 10.1371/journal.pgen.0010029 (PMC1193535; doi:10.1371/journal.pgen.0010029)
Supplement: Dataset S15 — (199 KB DOC) [file pgen.0010029.sd015.doc]

Dataset S15. Genes exhibiting peak of expression at the gastrula stage.

Genbank IDUF egg 3hpf 4.5hpf 6hpf 7.7hpf 9hpf 10.7hpf 12hpf 15hpf 24hpf 30hpf 48hpf

BI704310 -0.505 0 4.183 4.459 5.947 4.662 2.738 3.76 1.964 0.446 0.184 0.359

BI887446 -0.728 0.843 4.999 4.723 5.164 3.541 2.85 2.104 0.391 -0.38 -0.059 -0.19

BI888483 -0.438 0.993 5.111 4.773 5.126 2.01 1.304 2.75 1.361 -0.117 -0.144 0.092

AF077225 -0.605 0.807 4.683 4.967 4.68 2.241 1.615 3.264 0.636 -0.037 -0.017 -0.295

BI890505 -0.285 -0.09 4.319 3.893 4.671 3.049 1.938 1.746 0.989 -0.311 0.149 -0.077

BI886431 -0.691 -0.474 1.99 3.04 4.379 3.376 2.483 3.455 2.321 -0.149 0.078 -0.148

BM036509 -0.832 -1.089 1.921 3.063 4.217 3.439 3.199 3.112 2.503 0.124 0.31 -0.36

AI959644 -0.881 0.013 2.18 3.173 4.202 3.96 3.578 3.8 2.558 -0.271 0.566 -0.431

AB055677 -1.444 -2.002 2.149 3.533 4.187 4.457 3.943 3.753 3.494 1.27 0.464 -0.705

BG799399 -0.777 -0.694 1.608 3 4.15 4.333 3.419 3.418 2.052 0.008 0.025 -0.241

BI889130 0.02 -0.615 0.622 1.87 4.055 2.217 1.535 2.181 1.275 0.022 -0.163 -0.363

BI892244 -0.513 -0.056 2.232 3.232 3.987 2.062 1.974 1.669 1.208 0.137 0.572 0.044

BI891773 -0.828 -0.697 1.44 1.516 3.962 3.15 1.954 2.415 1.74 0.11 -0.042 -0.386

BI890587 -0.126 0.229 3.047 3.361 3.914 2.207 3.129 3.702 3.213 0.388 0.772 -0.033

AF207751 -0.462 0.804 4.331 4.013 3.902 2.737 1.996 3.268 1.29 1.233 1.19 0.352

AF052251 -0.728 0.214 2.368 2.087 3.867 1.415 0.804 1.812 -0.021 -0.416 -0.083 -0.266

AF246162 -0.685 -0.707 1.11 2.543 3.85 0.879 1.507 1.991 1.235 -0.041 -0.063 -0.295

BI886791 -0.772 -0.611 3.814 3.471 3.747 2.49 2.088 2.781 1.013 0.485 0.309 0.923

AW777769 -0.243 -0.136 1.301 2.744 3.642 1.661 1.352 1.63 1.275 -0.515 -0.495 -0.193

BM026830 -0.583 0.145 0.397 2.439 3.626 0.064 1.179 0.172 0.121 -0.674 0.009 -0.156

BE200802 -0.085 -0.108 0.348 3.046 3.583 0.269 1 1.22 0.131 -0.595 -0.369 -0.231

AW059366 -0.03 0.142 1.531 1.689 3.508 3.088 2.077 1.357 1.046 0.042 0.932 0.157

BI673712 -0.449 0.344 0.783 2.888 3.442 0.682 0.488 0.994 0.043 -0.2 0.474 0.084

BI892068 -0.234 0.46 1.047 2.446 3.409 0.9 1.05 0.813 0.134 -0.122 0.064 0.12

AI588792 -0.958 0.621 3.218 3.383 3.336 1.811 1.388 2.132 2.479 1.48 0.833 0.44

AW115956 0.119 -0.422 0.763 1.929 3.313 1.68 1.617 1.777 1.345 -0.159 -0.373 -0.527

AI959735 -0.678 0.385 2.405 2.732 3.303 1.507 1.295 2.131 0.26 -0.104 0.133 -0.36

BM185158 -0.132 0.013 1.945 3.112 3.293 2.218 1.281 1.514 0.715 -0.062 0.191 0.333

BG985460 -1.109 -1.649 1.801 2.578 3.241 2.613 1.623 1.109 1.668 0.504 0.229 -0.073

BI888158 -0.567 0.055 2.182 2.398 3.208 1.226 0.67 2.981 1.848 0.477 0.048 0.042

BM072263 -0.124 -1.727 0.92 0.719 3.206 3.052 2.56 2.146 1.799 -0.001 -0.074 0.119

AI877596 0.027 0.181 1.012 2.523 3.165 1.089 1.485 0.711 0.657 -0.586 -0.282 0.002

BG728817 0.377 -0.05 0.755 1.343 3.093 1.549 1.395 1.308 0.925 -0.066 -0.224 -0.427

BG985498 -1.013 -1.923 2.883 2.542 3.052 1.85 1.369 2.023 0.953 0.744 0.504 0.282

BI888232 -0.195 0.697 1.866 3.261 3.045 1.671 1.983 2.777 1.522 -0.301 0.721 -0.392

AW343985 0.676 0.906 1.24 2.366 3.043 2.724 2.107 1.825 1.317 -0.153 -0.26 -0.711

BI868116 -1.267 -3.251 -0.071 1.089 3.043 2.44 2.587 1.719 1.335 -0.465 0.072 0.078

U68234 -0.236 0.873 0.433 1.208 2.995 1.007 1.277 1.841 0.342 -0.21 0.368 -0.324

BI879444 1.036 0.868 2.755 3.127 2.955 1.676 2.028 1.635 0.746 1.122 0.848 0.509

L46801 0.534 0.449 1.877 2.076 2.922 2.188 1.918 2.095 1.126 0.414 0.188 -0.149

BI890279 -0.199 -0.483 1.692 2.054 2.906 1.847 1.443 1.88 0.563 0.387 0.147 0.222

BI892349 -0.493 0.065 3.322 3.139 2.871 2.644 2.198 1.907 0.968 0.944 0.976 0.056

BI877872 0.082 0.756 2.55 2.114 2.863 2.766 1.911 1.865 0.263 0.296 -0.062 0.131

BM025184 -0.295 -1.02 0.009 1.676 2.839 0.877 1.28 1.111 0.781 -0.187 0.102 0.009

U10869 0.055 0.572 0.685 0.983 2.823 0.173 0.76 1.707 0.163 -0.011 -0.134 -0.074

AY057095 -0.437 0.676 2.997 2.712 2.818 1.017 1.3 2.482 0.545 0.658 0.487 0.149

BG883207 0.706 0.484 2.427 1.969 2.813 1.712 1.247 1.881 0.663 0.963 0.383 0.135

BI885653 -3.548 -1.131 3.457 3.722 2.8 1.754 1.059 1.023 0.56 0.958 0.877 1.316

AW567130 -0.533 -0.575 1.898 2.066 2.706 1.28 2.222 2.362 1.631 0.057 0.043 -0.127

AF191578 -0.572 0.032 1.052 2.112 2.705 0.244 0.468 1.513 0.46 0.187 0.22 0.015

BI888620 1.143 1.28 2.635 2.463 2.685 1.633 0.894 -0.73 -1.085 -0.832 -1.243 -0.986

BI888241 -0.728 0.04 0.618 1.503 2.68 1.586 1.825 1.617 0.395 -0.762 -0.274 -0.505

BI888338 -1.058 -0.729 1.79 1.534 2.663 2.163 1.734 2.502 1.936 0.469 1.079 0.39

AI965249 -0.916 0.059 1.887 2.029 2.632 2.428 1.793 1.964 1.421 1.408 1.236 1.455

BG727557 -0.434 0.252 1.633 1.795 2.593 2.382 1.807 2.117 1.253 1.097 0.471 0.078

BI704288 -0.894 -0.719 1.727 2.316 2.584 3.313 2.896 2.643 1.91 1.66 1.137 0.856

AF157110 -0.522 -0.377 0.855 1.995 2.574 1.429 1.305 2.254 0.628 -0.123 -0.133 -0.387

AF052245 -0.116 -0.212 1.348 1.297 2.57 0.785 0.514 1.204 0.119 0.163 0.294 0.106

BI885944 -0.85 0.45 1.191 1.649 2.567 1.012 0.739 1.695 1.016 -0.504 -0.684 -0.65

U42392 -0.102 -0.654 0.545 2.58 2.552 0.339 1.061 0.924 0.872 -0.34 0.174 -0.068

BI886789 -2.881 -1.165 3.378 3.291 2.527 1.698 0.991 0.476 0.471 0.962 0.918 1.344

BI891793 -0.728 -0.335 2.14 1.682 2.526 2.648 2.007 2.011 1.134 0.097 0.566 0.387

BI979581 -0.817 -1.189 1.126 1.87 2.524 0.791 1.075 2.093 1.796 -0.808 -0.988 -1.127

AF034606 -0.728 0.076 2.492 3.041 2.51 1.905 0.929 2.251 0.723 0.101 0.023 -0.316

BM083966 -0.477 0.181 0.802 2.011 2.5 1.293 1.129 1.728 0.337 -0.074 0.132 0.9

AW058811 -0.754 -0.511 1.928 2.388 2.487 0.996 0.579 0.318 -0.344 0.012 -0.306 -0.693

BI892110 -0.496 -0.892 1.503 2.679 2.476 0.098 1.275 1.143 1.483 0.757 -0.012 -0.173

AI588196 -0.441 -0.957 0.16 2.314 2.446 -0.124 1.381 1.012 0.812 0.189 0.355 -0.019

BF157620 0.231 0.475 1.886 1.999 2.439 0.894 0.118 1.468 0.726 0.478 -0.218 -0.266

BI888090 -0.024 -0.167 0.765 0.549 2.432 2.316 1.942 2.327 1.465 0.27 0.057 -0.109

AI964310 -0.405 -0.15 2.21 1.926 2.429 0.911 0.881 1.219 0.53 0.481 0.578 0.355

BI887548 -0.725 0.523 2.469 2.135 2.426 3.137 2.288 2.011 1.243 1.528 0.989 0.844

BI673416 0.415 0.891 2.481 2.38 2.425 2.695 2.027 1.668 0.991 0.813 0.404 -0.074

AW019725 0.578 -0.764 2.338 2.888 2.418 2.453 1.632 1.454 0.519 1.512 1.026 0.657

BI886653 -0.245 0.381 1.379 1.607 2.417 1.66 1.173 1.97 0.486 1.143 0.718 0.622

BI891338 -0.493 -0.665 1.322 2.059 2.403 0.907 0.367 0.462 0.418 -0.137 -0.194 0.224

AI957698 -0.786 -0.26 1.664 1.773 2.383 1.099 0.856 1.987 0.641 0.375 -0.041 -0.912

AF336123 -1.57 -0.396 0.263 1.712 2.381 1.641 1.859 2.302 1.093 1.564 1.231 0.662

BI890262 -0.728 0.17 1.09 2.848 2.359 0.268 -0.219 0.193 -0.378 -0.68 -0.117 -0.384

AF219949 -0.195 0.389 0.407 0.433 2.351 1.238 1.071 2.132 0.41 -0.469 1.171 0.451

BI475794 1.108 0.485 1.92 1.892 2.347 2.82 2.145 2.367 1.589 1.619 1.017 0.375

BI428973 -0.406 -0.284 1.891 2.453 2.338 2.51 1.666 1.07 0.827 0.821 0.44 0.616

BE201475 0.179 0.423 2.574 2.595 2.318 0.907 0.997 1.851 0.906 0.184 -0.171 -0.336

AI477963 -0.728 -0.059 0.342 1.852 2.316 0.605 0.823 0.786 0.205 0.459 1.126 0.408

AA494997 0.093 1.166 1.246 1.116 2.289 1.522 0.841 1.659 0.223 -0.069 -0.325 -0.485

BI891984 -0.299 -0.03 2.326 2.165 2.287 0.248 0.46 1.084 0.32 0.164 0.053 -0.208

AI974191 -0.413 -0.907 1.633 1.901 2.286 2.464 2.089 2.321 0.974 0.744 0.218 -0.394

AF168007 -0.304 0.263 1.977 2.021 2.285 1.291 0.849 2.239 0.503 0.632 0.527 0.299

AI626435 0.045 1.336 1.606 2.652 2.284 0.922 0.828 1.492 0.242 0.02 0.464 -0.196

BM104683 0.807 1.779 2.227 2.012 2.262 1.8 1.856 2.086 0.958 1.073 0.376 0.265

BI890294 -0.144 -0.11 0.704 1.974 2.251 0.889 0.575 1.213 0.487 0.208 0.155 0.125

BI888267 -0.026 -0.34 0.953 1.409 2.243 0.337 0.844 1.119 0.524 0.489 0.753 -0.474

BI892155 -0.319 -0.307 1.614 2.161 2.241 2.704 2.01 2.167 1.038 0.79 0.355 0.414

BI888493 -0.11 -0.057 1.792 2.345 2.239 0.066 0.389 1.186 0.032 0.181 0.311 -0.199

BI890191 -0.438 -0.363 1.143 1.333 2.239 0.83 0.461 1.514 0.662 0.347 0.456 0.151

AI641480 -0.682 0.079 0.893 2.363 2.239 0.221 0.022 0.248 -0.235 -0.799 0.107 0.018

AI477962 0.623 1.065 1.93 1.953 2.226 1.983 1.718 1.384 0.266 0.754 0.195 0.338

BI886272 -0.195 0.334 1.563 2.079 2.216 1.158 1.427 1.986 0.671 0.598 0.336 0.148

AF211852 0.535 0.542 2.347 2.444 2.201 1.979 1.774 1.813 0.951 0.925 0.525 0.287

BI883252 0.018 -0.384 0.256 1.169 2.185 2.142 1.616 2.184 0.891 0.477 0.538 -0.261

BI980148 0.523 0.29 0.583 1.658 2.178 1.664 1.765 1.579 0.88 -0.028 0.109 -0.191

BM184367 -1.181 0.405 1.523 2.016 2.175 0.921 -0.064 1.287 0.516 0.565 0.258 0.215

X87581 -1.892 -1.153 2.857 2.754 2.173 1.424 1 1.17 0.804 1.252 1.018 0.398

AI794024 -0.839 -0.043 2.469 2.163 2.17 1.952 1.496 1.687 0.706 1.009 1.093 0.425

AI958945 -0.364 -0.189 1.392 1.866 2.146 0.542 0.536 1.185 0.249 0.449 0.321 0.322

BI983579 -0.048 -0.111 1.708 1.68 2.141 2.049 1.264 1.447 0.999 0.845 0.337 -0.126

BI880061 0.262 0.555 1.904 1.459 2.139 2.208 1.57 2.18 1.066 1.246 0.71 0.75

BI892410 -0.12 1.219 0.658 1.727 2.12 0.401 0.43 1.208 -0.043 -0.321 0.133 -0.039

BM183279 -0.225 1.068 1.525 2.298 2.119 0.371 0.698 1.401 0.039 -0.237 0.086 0.3

BI888721 -0.525 0.77 1.886 1.942 2.106 2.55 1.879 2.022 1.506 0.724 -0.212 -1.139

U49417 0.27 1.065 2.324 1.579 2.096 2.487 1.62 1.71 0.906 0.25 0.294 0.013

AI437428 -0.247 0.539 1.858 1.632 2.081 1.957 1.738 1.096 0.661 0.466 -0.105 -0.208

AI964130 0.869 1.036 2.211 2.089 2.08 2.145 1.808 1.322 0.708 0.57 0.145 0.097

AW058763 -0.035 -0.601 0.757 1.392 2.078 1.945 1.238 1.241 0.892 0.653 0.722 0.989

BI889129 1.37 1.333 2.155 2.24 2.077 2.091 1.058 1.625 0.767 0.399 -0.686 -0.411

BI892128 -0.5 0.16 1.673 1.781 2.075 1.717 0.788 2.028 0.449 -0.276 0.102 0.198

AW165150 -1.089 -0.023 1.427 1.321 2.062 2.211 1.078 1.628 0.703 0.591 0.312 0.285

BI890669 -0.011 0.059 1.836 0.835 2.062 2.269 1.703 1.855 1.04 0.883 0.553 0.23

AI942952 0.683 0.694 1.03 1.305 2.06 2.016 2.097 1.57 1.59 0.455 -0.444 -0.519

BI704344 -0.438 -0.08 2.02 2.297 2.045 2.031 1.203 1.416 0.997 0.779 0.709 0.558

AI544512 -0.264 0.091 1.096 1.692 2.037 0.167 0.746 1.734 0.205 0.631 0.259 0.263

BI980843 -0.107 0.185 2.232 1.995 2.032 1.509 0.835 1.257 0.229 0.532 0.493 0.504

BI888564 0.077 0.476 1.388 1.506 2.022 0.556 0.356 1.225 -0.114 -0.185 -0.11 0.016

AJ236882 -5.67 -3.087 1.093 1.801 2.022 2.735 2.118 1.986 1.444 1.6 1.121 0.061

AW165417 0.12 0.884 1.787 2.045 2.015 1.164 0.775 1.768 0.595 0.505 -0.232 -0.578

AI722328 0.381 0.015 1.576 1.122 2.013 1.245 1.017 1.432 0.778 0.383 0.162 -0.086

BI887656 -0.191 0.343 0.481 1.538 2.004 0.676 0.747 1.808 0.42 0.01 0.271 0.553

U23839 -0.393 -0.134 0.838 1.611 2.002 0.903 1.071 1.917 0.833 0.502 0.71 0.194

AI477969 -0.728 1.013 2.31 2.843 1.995 0.108 -0.083 0.553 -0.378 -0.851 -0.652 -0.636

AW059007 -0.846 -0.118 1.549 1.923 1.99 0.899 0.536 1.01 0.102 0.318 0.281 -0.107

BI879454 -0.515 0.545 0.751 1.136 1.98 1.181 0.722 2.033 0.617 0.686 0.073 0.408

D26174 -0.284 0.052 -0.004 1.143 1.975 0.321 0.575 1.242 0.297 -0.121 1 0.015

BG883304 -0.547 0.453 1.935 1.845 1.97 0.81 0.118 0.299 -0.127 -0.022 -0.641 -0.355

AI721361 -0.202 -0.509 1.223 1.74 1.965 1.81 0.826 0.961 0.726 0.29 0.067 0.244

AI667403 0.116 0.165 2.419 2.743 1.961 1.197 0.527 0.779 0.42 0.85 0.166 0.691

BM104310 0.246 0.068 1.656 1.399 1.958 1.705 0.66 1.532 1.546 1.585 0.985 0.111

BI888360 -0.07 -1.288 1.24 2.033 1.955 0.744 0.721 1.108 0.372 0.75 0.753 0.319

BG304158 -0.024 0.384 1.589 1.826 1.952 1.364 1.475 1.67 0.969 0.929 0.227 -0.325

AI943105 -0.236 0.113 1.302 1.596 1.952 0.19 0.788 1.089 0.434 0.455 0.246 -0.079

AF398433 -0.455 0.236 0.643 0.601 1.95 2.425 1.344 1.659 1.19 0.167 0.637 0.096

AW059137 0.038 -0.282 0.947 1.012 1.947 1.203 0.678 1.243 0.535 0.249 0.289 0.012

BI877730 -0.589 0.447 1.153 1.37 1.943 0.872 0.52 0.739 0.477 0.344 0.381 0.025

BI888977 0.578 1.082 2.224 2.134 1.942 1.339 1.073 1.517 0.761 0.789 0.519 0.16

BI892060 0.16 -0.133 0.757 1.039 1.941 0.74 0.677 1.356 0.398 0.897 0.392 -0.254

BI890739 -0.044 0.123 1.074 1.599 1.94 0.867 0.742 1.875 0.39 0.417 0.688 -0.076

BI672391 -0.006 0.094 1.156 1.734 1.935 0.353 0.341 0.781 0.358 0.445 0.093 0.042

AF354754 -0.329 0.69 1.428 1.844 1.929 0.808 0.183 1.064 0.376 0.205 0.769 0.076

BI888172 -0.069 0.004 0.057 1.149 1.929 0.219 0.186 0.406 0.436 -0.029 -0.132 -0.204

AI964189 0.267 1.099 1.988 1.98 1.928 1.729 1.553 1.695 0.454 0.816 0.278 -0.108

BM070558 0.149 0.833 1.028 1.453 1.928 1.144 1.037 1.28 0.415 0.242 0.402 0.339

BI890334 -0.503 -0.096 1.285 1.668 1.927 0.674 1 0.976 0.919 0.241 -0.208 -0.512

BI890618 -0.728 0.138 0.238 1.14 1.921 0.883 0.79 1.711 0.755 0.868 0.824 -0.057

BI704278 0.235 0.826 1.924 2.043 1.917 1.005 1.357 0.819 0.016 0.76 0.369 -0.452

AI544535 0.64 1.491 2.202 2.273 1.915 0.284 0.465 0.467 -0.113 0.054 0.084 -0.036

AI722745 -1.013 0.196 0.92 0.761 1.912 1.332 0.137 1.489 1.304 0.77 0.493 1.446

AI958627 0.025 0.469 1.697 1.959 1.904 1.67 1.284 1.064 0.465 0.561 0.327 0.581

AW115560 0.891 1.376 1.756 2.224 1.903 1.805 0.949 1.514 0.489 0.359 -0.054 -0.395

BI533952 1.515 1.093 1.14 1.623 1.902 2.304 1.646 1.891 0.953 0.857 0.419 -0.161

AF388363 -0.588 -0.037 0.536 1.212 1.899 0.826 0.534 1.494 0.15 0.044 0.729 -0.073

AW019294 -0.155 0.132 1.911 1.669 1.898 1.171 1.136 1.087 0.865 0.413 0.521 0.331

BI892036 -0.808 -0.22 0.73 1.577 1.897 1.264 0.113 -0.091 -0.274 0.572 0.902 -0.134

AW566851 1.424 1.352 2.148 2.218 1.897 2.049 1.666 1.407 0.71 0.566 0.012 -0.275

BI890367 -0.771 0.247 0.862 1.822 1.886 1.07 0.339 1.694 1.73 1.5 0.567 0.091

BI891492 -0.321 -1.729 0.917 1.394 1.885 1.036 1.3 1.67 0.634 0.804 0.708 0.639

AI497265 -0.109 0.2 1.042 1.416 1.874 0.296 0.801 1.496 0.234 0.474 0.151 0.056

AW019482 -0.72 0.137 0.945 1.542 1.862 2.158 1.461 1.758 0.798 0.659 0.443 0.291

BI474299 -0.368 -0.823 1.011 1.127 1.853 2.506 2.282 2.019 1.33 0.804 0.309 -0.186

BI891877 0.443 0.341 1.468 1.753 1.852 1.587 1.372 1.382 0.841 0.683 -0.064 -0.819

BI672347 -0.501 -1.061 0.898 1.444 1.845 1.542 0.77 0.734 -0.149 0.027 -0.178 -0.484

AW059146 -0.048 0.489 1.663 1.671 1.844 1.612 1.307 1.608 0.424 0.593 0.344 0.388

BM101748 -0.418 0.236 0.359 1.428 1.839 0.325 0.58 1.186 0.205 -0.256 0.444 -0.262

BI886353 -0.033 1.17 0.83 2.112 1.837 0.88 0.609 1.248 0.004 0.094 -0.214 -0.289

BI887861 0.136 0.37 1.073 0.819 1.835 0.96 0.277 0.014 -0.527 -0.237 -1.15 -1.157

AW171604 0.608 1.401 1.186 1.595 1.835 0.656 0.699 0.496 -0.433 -0.71 -0.69 -0.316

BI885973 -0.077 1.298 1.376 1.529 1.834 1.341 0.743 1.56 0.503 0.187 0.126 -0.082

BI885798 -0.064 0.696 0.89 1.303 1.831 0.559 0.346 1.304 0.395 0.157 0.272 -0.182

AI477419 -0.495 -0.332 0.882 1.233 1.83 0.344 0.614 1.522 0.73 0.902 0.466 0.056

BI886470 -0.728 -0.501 0.201 0.707 1.821 0.121 0.208 0.672 0.272 -0.045 -0.025 -0.278

BI704281 -4.509 -4.245 0.937 1.408 1.815 2.267 1.964 1.85 1.03 0.217 0.049 -0.251

AW116075 1.269 1.205 1.827 1.985 1.811 1.349 0.586 -0.015 -0.933 -1.598 -1.821 -2.886

BI888748 -0.018 0.078 1.517 1.524 1.81 1.091 1.067 1.429 0.711 0.487 -0.333 -0.774

AW128377 -0.088 -0.053 0.602 1.234 1.809 0.524 0.898 1.65 0.318 0.378 0.153 0.345

BG985698 -0.739 -0.431 0.482 0.859 1.807 1.733 0.706 1.382 0.308 0.398 0.828 0.355

BI879550 -0.275 0.606 0.918 1.501 1.805 0.612 0.879 1.233 -0.004 0.396 0.037 -0.037

BI318080 -0.443 -0.284 0.142 1.68 1.805 0.36 0.372 0.227 -0.029 -0.271 -0.444 -0.239

BI888431 0.308 0.018 1.028 0.665 1.805 0.693 0.305 1.212 0.172 -0.17 -0.02 -0.049

BI886936 -1.375 -0.076 2.026 1.845 1.802 1.209 0.3 0.923 1.111 0.66 0.508 0.539

BI886677 -0.5 0.454 1.876 2.443 1.798 0.792 0.009 1.232 0.844 0.558 0.482 0.224

AI616791 -0.498 -0.311 0.902 1.224 1.794 1.573 0.828 1.025 0.399 0.892 1.055 1.106

BI888545 -0.535 0.102 1.261 1.244 1.79 0.441 0.632 1.7 1.06 0.096 0.597 0.087

AW232627 1.073 0.833 2.196 2.255 1.786 2.237 1.956 1.376 0.474 0.525 0.195 0.267

BM101524 0.961 0.937 1.8 1.434 1.779 2.168 1.556 1.693 0.887 0.791 -0.197 -0.616

BM071679 0.378 1.01 1.705 1.556 1.776 0.869 0.902 1.281 0.464 0.71 0.131 0.122

AW420284 -0.707 0.086 1.145 1.406 1.771 0.826 0.378 0.515 -0.194 -0.102 0.206 -0.077

AI584322 -0.728 -0.129 0.191 0.191 1.77 0.432 0.263 1.276 0.308 -0.171 -0.056 0.134

BM156110 -0.319 0.211 0.928 1.469 1.767 1.709 1.163 1.454 0.577 0.456 0.411 0.462

AI584501 -0.134 -0.118 0.923 0.971 1.757 1.225 0.958 1.632 0.756 0.453 0.714 0.16

AF364085 0.055 0.302 0.883 1.221 1.755 1.378 0.816 1.584 0.556 0.82 0.21 -0.512

AW306107 1.057 1.458 2.09 2.065 1.75 1.318 0.388 0.803 -0.001 -0.345 -0.923 -1.164

BI892135 -0.199 0.747 2.318 1.786 1.74 2.348 1.313 1.145 0.584 0.461 -0.082 -0.135

AF426384 -0.75 0.026 0.392 1.604 1.737 1.203 0.569 1.317 0.163 0.606 0.979 0.166

BG305790 -0.653 0.033 1.14 1.154 1.736 1.791 1.188 1.785 0.532 0.399 0.296 0.083

U41081 -0.488 -0.143 1.492 1.729 1.731 1.694 1.771 1.534 1.014 0.996 0.69 0.283

AI721655 -0.68 0.012 0.889 1.683 1.731 0.89 0.362 1.019 0.822 0.66 0.475 0.509

BI891684 0.64 0.509 1.25 1.796 1.729 1.798 1.338 1.521 1.137 0.57 0.149 -0.447

BG985503 -0.612 -0.164 1.419 1.396 1.722 1.968 1.606 1.962 1.2 1.045 0.958 -0.26

BI708386 0.701 0.832 1.589 1.486 1.719 1.861 1.322 1.448 0.979 0.428 0.281 -0.1

BI890073 -0.275 0.415 1.398 1.833 1.712 0.781 1.057 1.716 0.153 0.481 0.419 0.573

AW116461 0.129 0.9 1.408 1.805 1.712 1.554 0.77 1.263 0.443 0.256 0.028 -0.315

BI704199 0.322 -0.087 0.861 1.916 1.706 -0.142 0.45 1.02 0.116 0.357 0.209 -0.02

BM101651 1.225 1.091 1.942 1.807 1.705 2.256 1.988 2.148 1.337 1.087 0.439 -0.15

BI887226 0.631 0.774 1.577 1.514 1.704 2.587 2.379 2.332 1.737 1.325 0.847 0.672

BI885991 0.748 0.839 0.752 1.797 1.704 0.996 0.776 0.998 0.124 0.141 -0.125 -0.179

AA605765 0.749 1.146 2.046 1.648 1.7 2.385 1.314 1.738 0.828 0.704 0.182 0.011

BI886702 -0.107 0.2 0.151 2.293 1.7 0.482 0.074 -0.028 -0.148 -0.805 -1.051 -1.286

BI878854 -0.1 0.766 1.531 1.757 1.699 1.258 0.966 1.081 0.572 0.723 0.392 -0.175

BI889637 -0.998 -0.213 0.805 1.805 1.697 -0.11 0.185 -1.339 -1.211 -0.444 -0.659 0.034

BI980224 1.625 1.308 2.334 1.236 1.695 2.59 2.08 2.035 1.458 0.827 0.402 -0.5

BI672395 -1.685 -0.208 1.617 1.404 1.689 1.504 0.723 0.741 0.986 0.604 0.78 -0.446

BI887764 -0.166 -0.148 1.247 1.069 1.686 0.61 0.671 1.161 0.635 0.345 0.567 0.02

AI884177 -0.723 0.173 1.481 1.658 1.686 0.137 -0.12 0.193 -0.299 -0.609 -0.086 -0.222

AI641092 -0.824 -1.091 0.25 0.571 1.685 2.137 1.515 0.862 1.178 0.875 1.039 0.792

BI710730 -0.712 0.204 1.652 1.848 1.684 0.801 0.598 1.066 0.859 0.378 -0.424 -1.049

BI892406 -0.149 0.355 1.499 0.908 1.684 1.464 0.716 1.096 0.401 0.362 -0.048 0.014

BI884839 -0.697 0.391 0.826 1.374 1.683 1.598 0.711 0.905 0.607 0.594 0.281 0.221

X79821 -2.071 -0.96 2.076 2.461 1.681 2.096 1.317 1.66 1.403 1.531 1.042 0.382

AJ315468 -0.367 -0.446 0.193 1.11 1.673 0.82 0.069 0.42 -0.145 -0.31 0.701 0.272

BI892200 -0.823 -0.459 0.243 1.066 1.667 1.094 1.022 1.034 0.141 0.534 0.644 0.035

BG307572 -0.468 -0.085 0.294 0.809 1.666 -0.494 0.437 1.08 0.424 0.043 -0.081 -0.267

BI982141 -0.121 -1.062 1.356 1.03 1.666 1.508 0.761 -0.324 0.066 0.517 0.629 -0.309

BI671227 -0.691 -0.282 1.073 0.852 1.665 1.088 0.985 0.875 0.376 0.108 0.038 0.274

BM096465 0.034 0.111 2.124 1.906 1.663 1.33 0.691 0.911 0.251 0.343 0.075 -0.037

AW232003 -0.38 -0.255 1.567 1.959 1.653 2.21 2.064 1.948 1.125 0.661 0.161 0.267

BM155225 1.698 0.842 1.388 1.632 1.652 1.903 1.532 1.095 0.275 -0.065 -0.472 -0.704

BI475856 0.36 0.483 1.043 1.407 1.648 0.711 1.143 1.31 0.41 0.055 0.037 -0.124

AW077980 -0.685 0.621 0.55 0.739 1.647 -0.13 0.43 1.327 -0.28 -0.422 0.008 -0.117

AI884112 1.236 0.976 1.369 1.842 1.645 2.304 1.567 1.26 0.598 0.487 0.071 -0.335

BI881679 -0.39 -0.395 1.395 1.655 1.644 2.129 1.82 1.645 1.028 0.607 0.174 0.125

AW154540 0.896 0.853 1.53 1.265 1.643 1.562 0.912 1.645 0.873 1.022 0.293 0.163

AI641052 0.185 0.342 1.664 1.511 1.641 2.115 1.561 1.352 1.171 1.08 0.581 0.538

AI476925 0.247 0.27 0.639 1.368 1.639 0.291 0.774 0.845 0.045 0.135 -0.19 -0.17

BI890314 -0.248 -0.196 0.655 2.21 1.637 0.228 1.112 1.317 0.234 -0.021 0.39 0.84

AI641022 -0.327 -0.016 1.234 0.821 1.636 1.909 1.426 1.858 1.155 1.089 0.366 0.319

AW420304 -0.403 0.384 0.391 1.028 1.635 -0.048 0.159 0.749 -0.017 -0.076 -0.226 -0.206

AW059069 0.311 1.188 1.583 1.983 1.633 2.62 2.126 2.5 1.704 0.55 -0.009 0.039

AI943062 1.088 1.142 1.202 1.477 1.633 1.909 0.91 1.149 0.18 0.328 0.066 -0.134

AI666882 0.26 0.045 1.103 1.61 1.632 1.736 1.657 1.429 0.796 0.682 0.01 -0.07

AW059316 -0.006 -0.363 0.395 0.734 1.632 0.782 0.638 1.336 0.562 0.174 -0.001 -0.154

BI889194 -0.577 0.037 0.587 1.239 1.632 -0.116 0.264 0.754 0.322 0.275 0.392 -0.039

AI793839 -1.442 -0.725 0.824 0.78 1.624 2.048 1.371 1.703 1.114 0.755 0.401 1.15

BI883935 0.336 0.332 1.274 1.116 1.622 1.538 1.239 0.803 0.795 0.759 0.529 0.481

AF359425 -0.992 0.095 1.926 1.36 1.619 1.774 1.296 1.245 0.265 0.406 0.362 -0.302

BM186051 0.612 1.491 1.718 1.152 1.618 1.923 1.76 1.734 1.531 1.431 0.688 0.517

BE557115 -0.035 1.025 2.097 2.263 1.617 0.521 0.387 0.596 0.108 -0.347 -0.253 -0.564

BI889298 -0.963 -0.727 1.212 1.632 1.616 1.92 1.521 1.855 1.465 1.092 0.472 0.53

BI704422 -0.373 -0.027 1.213 1.078 1.615 1.338 0.931 1.354 0.694 0.973 0.218 -0.146

BI533160 0.686 -0.172 0.495 0.916 1.614 2.26 1.59 2.254 1.444 1.623 1.002 0.405

AW165108 0.566 0.342 1.388 1.127 1.614 2.253 1.434 1.999 1.012 1.015 0.523 0.814

AB045624 -1.486 -1.495 0.571 1.069 1.612 0.87 0.508 0.171 -0.063 -0.449 -0.531 -0.726

AI793574 0.573 0.999 1.116 1.66 1.612 1.636 0.847 1.562 0.293 0.25 -0.118 -0.415

AW059156 -0.661 -0.711 -0.209 0.756 1.612 1.994 1.079 1.573 0.915 0.75 0.686 0.608

BG883236 -0.138 -0.138 0.509 0.198 1.61 0.805 0.639 1.027 0.563 0.32 0.273 0.516

BI887199 0.07 0.481 1.145 1.263 1.608 0.569 0.438 1.467 0.188 0.509 0.215 0.371

BG985763 -0.728 -0.149 0.465 0.903 1.606 -0.125 0.132 0.929 -0.378 -0.766 -0.543 -0.913

AF139990 1.639 1.578 1.918 1.939 1.601 1.011 0.602 0.876 0.101 -0.02 -0.364 -0.807

BI890823 -0.244 -0.159 0.691 1.14 1.6 1.535 0.912 1.284 0.972 0.822 0.837 0.175

BI887535 -0.001 0.101 1.259 1.209 1.598 0.166 0.326 0.967 0.188 0.489 0.099 0.012

BI890477 -0.378 -0.06 1.048 0.248 1.597 1.577 0.376 1.105 0.574 0.249 0.156 -0.009

AW342762 -0.174 0.54 1.771 1.505 1.597 2.172 1.592 1.808 1.451 0.82 0.543 0.356

BI883233 -0.728 0.203 0.467 0.841 1.59 0.116 0.378 0.717 -0.414 -0.453 -0.178 -0.287

AI877518 -0.497 0.024 0.383 0.727 1.587 1.335 0.651 1.519 0.862 0.411 0.47 0.357

AI601443 -0.528 -0.162 0.577 1.422 1.582 0.275 0.553 1.269 0.512 0.703 0.754 0.551

BF938808 -0.731 -0.028 1.161 1.773 1.582 1.521 0.905 1.267 0.223 0.566 0.226 -0.574

BI886020 0.569 1.643 1.901 1.967 1.581 1.595 1.053 0.74 0.327 0.175 0.308 0.078

BI880833 0.039 0.038 1.447 1.085 1.579 0.222 0.145 0.506 0.072 -0.035 0.368 0.422

AY050507 -0.589 0.13 1.223 1.089 1.576 1.483 0.492 1.223 0.505 0.334 0.325 0.202

AY026507 1.134 1.436 1.375 0.976 1.575 1.622 0.943 1.301 0.496 0.569 0.2 -0.434

BG985846 -0.622 -0.493 1.243 1.426 1.57 2.372 2.141 1.812 1.375 0.889 0.503 0.223

AI883253 -0.135 0.175 0.806 0.79 1.567 0.171 0.339 1.262 0.518 0.308 0.183 -0.259

BE016292 -0.647 0.336 0.408 0.931 1.567 0.287 -0.142 1.182 0.769 0.137 0.056 -0.326

AI942987 0.299 -0.455 0.096 -0.044 1.566 1.277 0.66 1.213 0.678 0.352 0.823 0.414

AA497205 -0.508 -0.123 0.87 1.577 1.563 0.152 0.435 1.465 0.39 0.438 0.601 0.215

AW058759 -0.133 -0.173 0.809 1.078 1.562 0.345 0.234 0.663 0.397 0.104 0.373 0.014

BI980805 0.181 -0.02 0.519 0.747 1.561 0.442 0.294 1.039 0.064 0.033 -0.231 0.03

BI892041 0.439 0.116 0.777 1.193 1.561 1.592 0.479 0.72 0.077 0.495 0.274 0.565

AW420314 0.385 0.698 1.157 1.016 1.558 1.498 1.127 1.542 0.539 0.385 0.235 0.004

BI887935 0.21 1.13 0.655 1.352 1.556 0.186 0.66 1.079 0.325 0.075 -0.176 -0.291

AI477417 -0.728 0.173 1.274 1.391 1.552 0.749 0.812 1.279 0.198 -0.272 0.142 -0.143

AF290981 -0.434 0.509 1.624 1.355 1.547 1.303 0.73 0.827 0.36 0.706 -0.262 -0.43

BG305892 1.007 1.177 1.098 1.248 1.54 1.95 1.4 0.77 0.532 -0.09 0.422 -0.189

BM184035 0.587 0.685 1.359 1.391 1.538 1.314 1.225 1.163 0.467 0.168 -0.384 -0.183

AF072456 -0.561 0.019 0.927 0.681 1.537 -0.189 0.353 1.302 -0.153 0.304 0.188 0.199

BM026121 -0.106 0.414 1.115 1.333 1.537 0.359 0.605 1.261 0.315 0.177 0.198 -0.005

BG303872 0.514 0.455 0.622 1.08 1.536 0.19 0.185 0.528 0.206 0.019 -0.432 -0.167

AI544688 -0.728 0.757 1.734 2.33 1.529 0.394 0.62 0.543 0.217 -0.471 0.453 -0.173

BI983370 1.076 0.901 1.388 1.398 1.529 1.391 0.869 0.903 0.07 0.323 -0.203 -0.235

AW058804 -0.376 -0.412 0.82 0.917 1.528 0.88 0.502 0.927 0.398 0.406 0.861 0.736

AW078306 0.154 0.721 1.511 1.672 1.524 1.603 1.117 1.374 0.991 1.262 0.454 0.458

BI887377 -0.723 0.469 0.808 1.198 1.521 0.287 0.151 0.862 -0.156 -0.522 0.568 -0.501

BI867819 -0.281 -0.249 1.511 1.684 1.521 1.821 1.231 1.866 0.62 0.598 0.141 0.291

AI584401 0.668 0.665 1.474 1.55 1.52 1.443 1.105 1.153 0.259 0.261 -0.294 -0.32

BM183794 0.222 0.859 1.593 1.649 1.52 1.251 0.959 0.681 0.443 0.118 0.341 0.359

AI618715 -0.966 -1.265 0.052 0.887 1.519 0.915 0.659 1.409 0.644 0.487 0.298 0.486

AF240772 -0.382 0.015 0.807 1.661 1.515 0.753 0.684 1.179 0.527 -0.004 0.594 0.138

AF172089 -0.155 0.202 0.631 1.057 1.514 -0.151 0.447 1.453 -0.137 -0.169 0.66 -0.259

AF151535 -1.002 -0.486 0.271 0.802 1.512 2.165 1.236 1.618 0.843 0.878 1.336 0.856

BI877985 0.758 1.496 1.147 1.963 1.511 0.629 0.279 0.806 -0.129 -0.47 -0.379 -0.399

AW117083 -0.975 0.212 0.938 1.226 1.508 0.754 1.013 1.41 0.439 0.558 0.499 -0.201

BG304149 0.807 1.426 1.581 1.624 1.508 1.442 0.85 1.103 0.028 0.215 -0.289 -0.133

AI877506 1.136 1.232 1.428 1.545 1.507 1.297 1.195 1.471 0.714 0.654 -0.292 -0.84

AW059234 -1.056 -0.709 -0.091 0.332 1.507 2.547 2.236 2.037 1.279 0.884 -0.048 -0.151

BG306390 1.019 0.343 1.777 1.25 1.505 2.005 1.641 1.624 0.915 0.879 0.434 0.201

AF168008 -0.308 0.116 0.876 1.313 1.504 1.804 0.889 1.415 0.377 0.404 0.515 0.261

AW595163 -0.281 0.604 1.283 1.406 1.503 2.125 1.462 1.608 0.654 1.041 0.477 0.301

BI980764 -0.155 0.177 0.512 1.124 1.499 0.547 0.487 1.113 0.153 -0.273 0.054 -0.031

AI584407 -0.537 -0.92 1.275 0.508 1.497 1.178 0.732 0.919 0.626 0.308 0.232 0.119

BI888567 0.283 0.689 1.137 1.271 1.497 0.968 0.598 1.318 0.559 0.247 0.251 0.333

AW174685 0.107 0.579 1.26 1.003 1.497 1.407 0.709 0.962 0.541 0.565 0.205 0.07

BI889290 0.365 0.665 1.253 1.263 1.493 1.047 0.491 1.075 0.129 0.248 -0.115 -0.234

AI601583 0.744 -0.652 1.139 0.976 1.491 1.387 0.721 0.909 0.538 0.269 0.163 -0.135

AW134011 -0.693 0.281 0.709 1.327 1.489 0.36 0.183 0.813 0.07 0.333 0.302 -0.091

BI428991 -0.483 0.232 1.546 1.819 1.482 0.424 0.317 0.747 -0.049 0.313 0.469 0.58

BI890446 -5.187 -4.116 0.084 0.595 1.476 2.73 2.164 2.261 1.733 1.813 1.288 1.067

BI475873 -0.375 0.95 2.319 2.254 1.475 0.507 0.473 0.399 0.077 -0.174 -0.522 -0.365

BI866879 0.177 -0.048 1.255 1.347 1.475 0.306 0.033 0.297 -0.343 -0.067 0.019 -0.558

AW280171 0.079 0.878 1.108 1.232 1.475 1.04 0.945 1.458 0.332 0.711 0.375 -0.171

BI890512 -0.619 0.788 1.553 1.003 1.474 1.431 0.711 1.432 0.675 0.506 0.367 0.215

BI888000 -0.728 -0.05 0.9 1.579 1.472 0.508 0.378 1.501 0.62 0.144 1.035 0.675

BI890444 -0.087 -0.021 0.71 0.652 1.47 1.837 0.662 1.028 0.674 0.644 0.37 0.228

BI887789 0.319 -0.817 0.325 0.712 1.465 0.392 0.683 0.855 0.818 0.644 1.033 0.303

AI884048 0.556 0.316 1.127 1.467 1.463 1.244 1.074 1.301 0.544 0.085 0.003 0.68

BI878078 -0.082 0.041 0.61 1.364 1.461 2.308 1.46 1.825 1.186 1.38 0.699 0.535

AI723269 0.601 1.203 1.872 1.617 1.461 1.903 1.756 1.564 0.847 0.7 0.159 -0.096

BG799259 0.268 0.853 1.432 1.605 1.458 1.78 0.648 1.409 0.451 0.701 0.3 -0.144

BI878929 0.402 0.814 0.957 1.401 1.457 1.156 1.063 1.216 0.202 0.43 0.285 0.244

BG303391 0.065 1.113 1.439 1.436 1.456 -0.087 0.349 0.716 -0.202 -0.574 -0.476 -0.442

BI883674 -0.248 -0.459 0.97 1.757 1.453 1.901 1.769 1.394 0.92 0.538 0.154 0.076

AW116371 0.388 1.274 1.625 1.818 1.451 1.261 0.844 1.121 0.235 0.08 -0.079 -0.26

AI793673 0.273 -0.065 1.228 1.295 1.448 1.053 0.67 0.708 0.406 0.382 0.169 -0.354

BI889190 -0.643 -1.184 0.411 1.186 1.448 1.606 0.88 1.167 0.5 0.29 0.528 0.24

BI879928 0.615 0.789 0.906 1.547 1.447 0.64 0.681 0.998 -0.275 -0.249 -0.383 -0.363

BI866992 0.81 0.445 0.665 1.102 1.446 0.983 0.903 1.332 0.517 0.672 0.047 -0.166

BM023680 -0.011 -0.236 0.462 1.358 1.445 0.885 0.87 0.526 -0.024 -0.026 -0.283 0.058

AW154725 0.945 1.027 1.404 1.534 1.442 1.922 1.227 0.995 0.371 -0.098 -0.397 -0.116

BI846965 -0.173 0.548 0.688 0.639 1.441 -0.004 0.252 0.862 0.036 0.144 0.164 -0.004

BI889160 0.123 0.816 1.683 1.64 1.441 0.211 0.838 0.637 0.364 -0.649 -0.199 -0.107

AI884185 -0.668 0.38 0.407 1.306 1.437 0.179 0.086 0.451 -0.293 -0.61 0.223 -0.232

BI865459 -0.46 0.092 -0.209 0.762 1.432 0.648 0.419 0.758 -0.128 0.157 0.128 0.875

AW154413 0.862 0.47 1.101 1.396 1.43 1.529 1.239 1.41 0.399 0.271 -0.08 0.057

BI889609 -0.372 -0.97 0.969 0.685 1.429 1.069 0.879 0.86 0.868 1.146 1.01 0.412

AW423211 -1.426 -1.134 1.503 1.609 1.428 1.211 0.814 0.129 0.303 0.299 -0.244 -0.272

BI886376 1.216 0.636 1.462 1.908 1.428 0.963 0.373 0.512 0.126 0.223 -0.225 -0.918

BI885886 -0.008 -0.184 0.859 1.769 1.423 0.701 1.15 0.785 0.503 0.353 0.162 0.115

AW421191 -1.226 -0.49 -0.224 0.924 1.42 1.404 1.054 0.995 0.458 -0.003 -0.19 -0.238

BI886934 -0.01 0.714 1.342 1.359 1.415 1.014 0.758 0.875 0.581 0.559 0.255 0.024

AI958820 -0.209 0.294 1.041 1.007 1.413 0.8 0.515 0.997 0.339 0.396 0.333 0.255

BG891955 0.241 -0.182 0.119 0.842 1.412 1.218 0.752 1.013 0.366 0.218 0.276 0.388

BI891754 -0.405 -0.111 0.374 0.708 1.411 -0.042 0.046 0.409 0.044 -0.133 0.297 0.275

AF164483 0.265 0.294 1.3 0.84 1.41 1.083 0.877 1.17 0.339 0.345 -0.269 -0.122

BI887133 -0.089 0.527 1.49 1.59 1.409 1.602 1.022 0.723 0.755 0.582 0.162 -0.018

AI584406 0.025 0.305 0.401 0.837 1.408 0.032 0.294 0.923 0.027 0.074 0.141 -0.123

AI626599 -0.023 -0.314 1.163 1.64 1.403 1.238 0.924 1.662 0.66 0.414 0.029 0.048

AF222996 -1.021 -0.438 0.325 0.806 1.402 1.757 1.288 1.089 0.955 0.837 0.542 0.704

BM072353 0.473 0.54 0.742 0.924 1.4 0.864 0.862 1.348 0.505 0.48 -0.197 -0.567

BI887338 0.599 0.324 1.356 2.578 1.4 1.321 1.149 1.225 0.969 0.494 0.025 -0.086

AI522514 -0.312 -0.032 0.782 0.659 1.398 0.212 0.444 0.849 0.334 0.479 0.67 0.509

BI842162 -0.211 -0.151 1.002 1.192 1.394 0.868 0.376 0.775 0.995 -0.123 0.512 0.356

BI888349 -0.675 0.23 0.969 1.43 1.394 0.231 0.09 0.247 -0.36 -0.622 0.019 -0.297

BI890821 0.599 0.75 1.667 1.795 1.393 1.971 1.662 1.526 0.871 1.079 0.876 0.525

BG305572 -0.611 -0.194 1.141 1.774 1.391 0.352 0.622 1.448 0.955 0.751 0.348 -0.521

BI887709 -1.484 -0.61 0.814 1.017 1.389 2.209 1.917 1.841 1.374 1.642 0.772 1.117

AW059048 0.216 0.168 0.698 1.075 1.389 0.868 0.804 1.318 0.402 0.833 0.403 0.068

AI477017 1.506 -0.515 0.621 1.004 1.389 1.994 1.16 1.452 0.888 0.757 -0.007 -0.564

BI891905 0.152 0.667 0.387 0.552 1.389 0.656 0.551 1.309 0.268 0.125 -0.352 -0.454

AI588319 -0.127 0.464 0.595 0.909 1.387 0.643 0.384 0.929 0.097 0.099 0.056 0.077

D38454 0.876 1.359 2.007 2.087 1.386 1.501 1.006 1.561 0.718 0.588 -0.164 -0.882

BI878820 0.534 0.874 1.336 1.253 1.384 1.809 0.923 1.11 0.333 0.481 0.045 0.521

BI891245 -1.868 -2.408 0.065 1.288 1.382 1.273 0.992 1.096 0.201 0.466 0.709 0.641

BG985787 0.125 1.156 1.189 1.268 1.38 0.354 0.424 0.699 0.187 -0.337 -0.627 -1.127

BG727431 -0.814 0.123 0.488 0.704 1.38 1.51 0.744 0.93 0.526 0.27 0.277 0.253

AI878755 0.516 0.607 0.799 0.811 1.377 1.699 0.833 1.014 0.508 0.453 0.307 0.312

AW117141 -0.08 -0.119 1.599 1.511 1.376 1.157 0.727 1.056 1.265 0.589 0.735 0.823

BI889119 0.746 1.614 1.708 1.8 1.376 1.006 0.559 1.102 0.4 -0.222 0.108 -0.465

BI886184 0.353 0.196 0.826 1.147 1.372 1.249 0.804 0.868 0.672 0.228 -0.035 -0.316

BI889113 0.98 1.194 1.865 2.179 1.371 1.229 1.238 1.15 0.53 0.475 -0.01 -0.448

BG985470 0.483 0.016 0.212 0.576 1.37 1.464 0.449 1.033 0.217 0.473 0.062 0.408

AW342711 0.508 -0.05 -0.042 1.87 1.368 -0.159 -0.377 -0.285 -0.515 -0.595 -0.612 -0.251

AI558301 0.074 0.849 0.845 0.861 1.366 0.63 0.907 0.975 0.515 -0.046 -0.196 0.013

AW165240 0.257 1.152 1.488 1.474 1.365 0.905 0.371 0.847 0.003 -0.287 -0.314 -0.108

BF713867 -0.4 0.541 1.484 1.659 1.362 0.546 0.344 0.536 0.561 0.003 -0.298 -0.571

BM181840 0.45 0.729 0.807 1.245 1.359 0.631 0.805 1.087 0.079 0.245 0.001 0.08

BI887540 -0.95 -0.795 1.422 1.292 1.359 2.048 1.594 1.583 1.454 1.34 0.943 0.261

BG727249 0.443 0.606 2.247 2.163 1.354 0.72 0.979 0.502 0.3 -0.084 -0.675 -0.313

BI704177 -0.32 0.568 0.185 1.051 1.353 0.698 0.587 0.816 0.419 -0.114 -0.058 0.144

AL591442 -1.908 -0.526 1.339 1.685 1.353 1.079 0.71 0.85 0.871 0.333 0.797 0.273

BI980217 -0.122 0.711 1.383 1.513 1.35 1.022 1.272 1.236 0.595 0.919 0.372 0.591

BI877821 -0.747 -0.558 0.37 0.813 1.342 1.011 0.867 1.189 0.414 0.694 0.048 0.135

BG306405 0.144 0.368 1.268 1.287 1.34 2.351 1.345 1.481 0.771 0.889 0.638 0.563

BM036823 0.737 1.155 -0.268 1.286 1.339 0.465 0.654 1.258 0.492 -0.003 -0.326 -0.453

BM072333 -0.131 0.357 0.284 1.288 1.328 0.864 0.616 0.968 0.402 0.056 0.334 0.332

BI867261 0.561 0.41 0.716 0.75 1.328 0.767 0.466 0.976 0.279 0.435 0.345 0.069

AF135438 -0.29 0.726 1.255 1.48 1.326 0.28 -0.144 1.137 0.438 0.387 -0.051 -0.181

BI882056 0.126 1.122 1.111 1.06 1.326 0.623 0.503 0.688 0.378 0.175 0.076 -0.48

AW153863 0.015 1.062 0.888 1.001 1.326 -0.055 0.098 1.013 0.141 0.155 -0.007 -0.238

BI888920 -0.018 -0.318 0.553 0.721 1.323 0.897 0.612 0.937 0.519 0.467 0.201 -0.105

BI889170 0.095 -0.055 0.51 0.822 1.318 0.965 0.926 1.127 0.155 0.232 -0.204 -0.169

BG883685 -0.128 0.513 0.885 1.296 1.307 0.222 0.424 1.052 0.23 0.001 0.096 -0.074

AI959620 0.036 0.096 0.341 0.928 1.303 -0.135 0.088 0.67 0.05 -0.074 0.667 -0.074

BI886095 -1.066 -0.774 0.889 0.964 1.302 2.394 1.851 1.67 1.44 1.299 1.194 1.203

AI793637 -1.564 -1.937 0.593 1.126 1.301 1.884 1.595 1.335 0.873 1.228 0.708 0.565

AW343764 1.17 0.703 0.983 1.197 1.298 1.631 1.137 1.283 0.636 0.37 -0.515 -1.036

AI416338 0.308 0.37 1.191 1.312 1.297 1.639 1.251 1.06 0.708 0.346 0.058 -0.289

AW058967 -0.916 -0.32 -0.062 1.133 1.297 0.985 1.037 0.835 0.727 0.616 1.1 0.576

AI641129 0.016 -0.176 1.639 1.64 1.296 0.248 0.447 0.561 -0.086 -0.352 -0.555 -0.583

AF332983 0.046 0.352 1.238 1.049 1.295 1.073 0.448 0.412 -0.261 0.192 -0.049 -0.624

AW232641 0.629 -0.723 0.217 0.707 1.293 1.914 1.536 1.577 1.116 1.052 0.429 -0.164

BM103978 1.052 0.92 1.456 1.856 1.293 0.258 0.286 0.503 -0.511 -0.329 -0.781 -0.947

AF160646 -0.299 -0.131 0.385 0.697 1.293 1.662 0.914 1.302 1.022 0.924 0.603 0.087

BI885813 -0.035 -0.048 0.906 1.149 1.29 0.617 0.358 0.491 0.33 -0.055 0.123 -0.013

BI889241 -0.661 -0.451 0.839 1.163 1.29 1.395 0.633 0.888 0.379 -0.068 0.596 -0.063

BI886745 0.003 0.053 1.196 1.123 1.289 0.521 0.499 0.742 0.202 0.045 -0.31 -0.376

AW165272 0.336 0.06 0.401 0.887 1.288 0.791 1.006 1.17 0.401 0.304 -0.103 -0.241

AW154283 0.729 1.14 1.173 1.22 1.287 0.854 0.571 1.29 -0.049 -0.021 -0.31 -0.657

AW019523 -0.573 -0.073 0.747 0.856 1.286 0.354 0.241 1.115 0.44 0.269 0.261 0.17

BI704267 0.288 1.392 1.552 1.613 1.286 0.773 0.23 1.092 0.071 0.522 0.147 0.173

AI657632 -0.458 0.206 0.665 1.115 1.281 0.464 0.086 0.526 -0.232 0.041 0.138 -0.321

BI883251 0.101 -0.434 0.163 0.344 1.281 1.334 0.744 0.944 0.563 0.687 0.49 0.421

AW175474 0.663 0.741 0.367 0.421 1.28 0.87 0.419 0.896 0.12 -0.376 -0.352 -0.251

BG303239 0.086 0.414 1.064 1.213 1.279 0.449 0.126 0.698 -0.045 -0.039 -0.035 -0.279

AF039410 -0.695 0.411 0.385 0.84 1.276 0.016 0.636 1.182 0.158 -0.113 0.276 -0.315

BI476854 -1.031 -0.864 0.567 1.176 1.276 1.893 1.312 1.323 0.636 0.836 0.459 0.749

BE605983 -0.36 -0.136 0.467 1.178 1.275 0.096 0.412 1.169 0.213 0.249 0.26 -0.065

BI890492 0.124 0.734 0.752 0.897 1.272 0.159 0.704 0.868 0.303 -0.058 0.185 -0.142

AW466751 0.084 0.827 1.088 0.973 1.271 0.781 0.704 0.886 0.336 0.657 0.268 0.079

BI704340 0.287 0.393 0.872 0.953 1.269 1.434 1.216 1.16 0.764 0.705 0.359 0.344

BI890755 -0.269 -0.589 0.586 0.912 1.268 0.861 0.645 0.329 0.759 0.494 0.265 0.033

BI889064 -0.087 1.037 1.942 1.998 1.267 0.843 0.462 0.96 0.012 0.172 -0.035 0.143

BI877594 -0.296 -0.075 1.134 0.982 1.267 0.518 0.349 0.675 0.615 0.541 0.477 0.414

BG985501 -0.387 0.668 1.113 1.281 1.266 1.107 0.647 1.369 0.022 0.801 0.656 0.337

AI588095 -0.27 0.29 1.117 1.12 1.265 -0.232 0.538 0.639 0.24 0.111 0.461 0.043

AY034614 0.129 0.971 0.334 1.19 1.263 0.68 0.912 1.229 0.561 -0.025 -1.001 -1

BI880060 -0.347 -0.03 1.283 1.413 1.261 1.186 0.2 0.803 0.172 0.399 0.175 0.138

AI883323 0.079 -0.096 0.409 0.909 1.259 0.696 0.144 0.613 0.082 0.244 0.338 0.149

AJ293862 -0.734 -0.999 -0.188 0.916 1.259 -0.079 0.066 0.086 0.055 -0.247 0.682 0.344

BI885993 0.458 0.852 1.015 1.554 1.256 -0.038 0.449 1.062 0.038 0.385 0.235 -0.062

AW128002 -0.302 0.68 1.494 1.649 1.253 -0.149 0.084 0.553 -0.099 -0.015 0.238 -0.187

BI891521 -0.555 -0.554 0.597 1.466 1.253 -0.131 0.318 0.694 0.135 0.155 0.6 0.481

BI886374 0.256 0.559 1.199 0.986 1.248 1.173 0.978 0.967 0.623 0.448 0.072 -0.367

AI964232 -0.299 -0.054 0.428 0.958 1.247 1.649 0.884 1.518 0.848 0.471 0.094 -0.369

BI889810 0.369 0.846 1.635 1.631 1.244 1.05 0.737 1.276 0.636 0.377 -0.12 -0.12

BI885898 -0.052 0.316 0.661 0.626 1.241 0.28 0.326 0.7 0.224 0.14 -0.209 -0.18

BG799357 -0.163 0.138 0.89 1.221 1.239 -0.105 0.059 0.31 -0.052 -0.222 -0.117 -0.171

BI888986 0.279 1.188 1.047 1.284 1.238 0.321 0.606 0.688 0.031 -0.044 -0.487 -0.671

BI888784 -0.681 -0.498 0.363 1.113 1.238 0.373 0.304 0.306 0.474 -0.017 0.118 0.618

BI888425 0.52 0.694 1.459 1.477 1.235 1.428 1.205 1.043 0.423 0.747 0.575 0.342

BI889901 0.255 0.192 1.275 1.641 1.234 0.66 0.757 0.506 0.138 0.122 -0.097 0.048

BI704373 0.171 0.492 0.906 1.354 1.233 0.689 0.306 0.779 0.173 0.215 0.042 -0.147

BM036795 0.201 0.782 1.001 1.14 1.233 0.437 0.368 0.76 0.021 0.178 0.082 0.236

BI883953 0.114 0.064 0.262 0.655 1.233 2.162 1.285 2.023 1.472 1.516 0.964 0.473

BE017652 -1.536 -0.098 0.239 0.418 1.231 1.86 1.504 1.459 0.999 0.557 0.271 -0.957

BM104315 0.805 1.23 1.685 1.591 1.23 1.872 0.578 0.888 0.277 -0.07 -0.424 -0.574

AF286364 -0.596 -0.075 0.967 1.782 1.228 0.245 0.227 0.516 0.605 -0.278 0.827 -0.003

U61395 0.595 -0.562 0.804 1.183 1.228 2.068 1.489 1.456 0.899 1.16 0.815 0.148

BI887057 -0.33 -0.483 0.191 0.546 1.222 0.337 0.423 0.596 0.3 0.667 0.509 0.109

AW116947 1.236 0.505 0.956 1.217 1.221 1.419 0.829 1.037 0.177 0.471 -0.094 -0.018

BI889937 -0.892 0.267 0.861 0.938 1.22 0.35 -0.08 -0.734 0.148 0.076 0.003 -1.026

BI880092 -0.443 0.051 0.572 0.84 1.219 0.056 0.147 0.62 0.612 0.133 0.566 0.615

BI892237 0.553 0.465 1.344 0.675 1.218 1.726 0.913 0.896 0.359 0.355 -0.2 -0.032

BI885889 -0.728 0.21 1.711 1.772 1.218 0.307 -0.219 0.34 -0.255 -0.143 0.55 -0.112

BI886811 -0.502 -0.102 0.448 1.216 1.217 -0.204 0.306 0.264 -0.254 -0.61 0.036 -0.293

BI888150 1.062 -2.111 0.455 1.152 1.217 1.738 1.336 0.92 0.655 1.051 0.639 0.2

BI891858 0.236 0.094 0.466 0.746 1.215 0.408 0.331 0.499 0.147 0.291 -0.044 -0.101

AI964204 0.243 -0.413 0.616 0.969 1.214 1.337 0.509 1.149 0.682 0.282 0.141 0.238

BI890287 -1.467 -2.301 -0.023 0.517 1.214 1.119 0.978 0.835 0.073 -0.503 -0.828 -1.662

BI880302 -0.238 0.096 0.725 1.278 1.214 0.003 0.119 0.406 0.273 0.174 -0.395 -0.596

AI964300 0.537 0.311 1.499 1.12 1.213 1.572 0.928 0.812 0.672 0.209 0.062 -0.117

BI886006 -0.164 -0.351 1.008 1.247 1.213 -0.029 0.205 0.741 0.111 0.221 0.325 -0.037

AI883908 -0.4 0.346 0.176 0.363 1.212 0.103 0.551 1.099 0.227 -0.148 0.396 -0.112

Z22762 -0.869 -0.077 1.045 0.736 1.212 1.485 0.813 0.983 0.378 0.556 0.534 0.034

AW019116 0.571 0.185 0.621 0.97 1.212 1.627 0.977 0.958 0.407 0.364 0.003 0.004

BM185759 -0.645 0.099 0.461 1.394 1.207 -0.134 0.597 1.076 0.426 0.657 0.772 0.353

AI385123 -0.453 -0.376 0.432 0.406 1.204 1.823 1.231 1.413 1.259 0.395 0.439 0.247

BG985620 -0.42 -0.472 -0.314 0.207 1.201 0.569 0.465 1.132 0.333 0.189 0.155 0.207

AW019187 -0.874 -0.461 0.833 1.219 1.198 1.379 1.178 0.686 0.438 0.06 -0.141 -0.054

BM183574 -0.293 0.406 1.344 2.067 1.196 0.141 0.256 0.17 0.021 -0.268 0.128 -0.194

AI416261 -1.02 -0.431 0.521 1.226 1.196 0.436 0.383 0.878 0.338 -0.039 0.046 -0.145

BM104287 0.505 0.404 1.326 0.726 1.196 1.249 0.66 1.156 0.394 0.824 0.086 0.115

BG883671 0.005 0.215 0.765 0.679 1.193 1.241 0.565 0.953 0.681 0.489 0.242 0.349

BI890643 -0.72 0.323 0.494 0.966 1.192 -0.085 0.256 0.991 -0.128 -0.061 -0.128 0.124

AW077193 -0.081 0.14 1.186 1.099 1.192 1.396 1.008 1.37 0.666 0.424 0.108 0.137

BG985598 0.142 -0.126 0.182 0.685 1.191 0.686 0.513 1.026 0.272 0.732 0.469 0.286

BI890315 -0.462 0.29 0.766 0.725 1.19 0.098 -0.153 0.807 0.305 0.681 0.843 0.203

BE016756 0.305 0.23 0.875 1.128 1.19 0.695 0.763 0.628 0.536 0.434 0.148 -0.11

AF159135 -0.161 0.308 0.812 0.545 1.188 -0.241 0.101 0.851 -0.073 -0.039 -0.018 -0.006

BI709110 -0.53 0.104 0.649 0.521 1.185 0.827 0.238 1.217 0.463 0.623 0.325 0.623

AI794634 -0.361 0.948 0.696 1.214 1.183 0.241 0.386 0.883 0.14 -0.056 -0.31 -0.107

AW078150 -0.798 0.206 0.541 0.831 1.182 -0.206 0.074 0.979 0.205 0.588 0.569 0.82

AI721615 -0.543 -0.642 0.421 0.496 1.182 0.597 0.455 1.008 0.23 0.301 -0.059 0.112

BI886461 0.232 0.019 0.634 0.432 1.182 0.974 0.677 1.114 0.361 0.605 0.141 0.306

BM154625 -0.344 -0.156 0.402 1.139 1.182 1.408 0.515 1.313 0.265 0.059 0.091 -0.106

AW058828 -0.596 -0.16 0.431 1.085 1.181 0.457 0.275 0.518 -0.083 -0.478 0.537 0.131

BI672139 0.087 0.39 0.466 1.169 1.179 0.633 0.694 0.772 0.179 0.516 0.228 0.259

BI888432 -0.199 -0.176 0.34 0.769 1.178 0.894 0.579 1.215 1.048 0.645 0.172 -0.297

AW117160 0.388 0.004 0.836 0.906 1.178 0.912 0.593 0.46 0.574 0.142 0.705 0.535

AI558351 -0.774 -0.705 0.49 0.338 1.178 1.902 1.068 1.295 1.039 0.704 1.115 0.679

BE605308 0.811 0.531 1.466 1.511 1.174 1.37 0.579 0.402 0.015 -0.711 -1.254 -1.466

AI793772 -0.158 -0.506 0.017 0.336 1.171 0.833 0.443 0.745 0.195 0.145 0.889 0.421

BI672394 0.8 0.316 0.466 0.734 1.169 1.874 1.096 0.768 0.222 0.236 0.065 -0.173

BI840104 -0.486 0.069 0.581 0.927 1.169 -0.032 0.011 0.363 0.209 -0.034 0.059 -0.008

BI886373 -0.546 0.568 0.741 0.912 1.168 0.261 0.354 0.906 0.84 0.65 0.218 -0.218

AI601596 -0.367 0.031 0.253 0.313 1.167 -0.029 0.014 0.644 0.003 0.116 0.174 0.299

AF414110 1.6 1.424 1.844 1.89 1.164 1.489 1.107 1.006 0.102 0.158 -0.195 -0.099

BI891527 0.57 -0.035 0.509 0.757 1.159 0.208 0.544 0.963 0.325 -0.003 0.006 -0.434

AI641041 -0.561 -0.429 0.645 0.737 1.158 0.984 0.489 0.562 0.468 0.393 0.662 0.601

BM154123 -0.416 0.073 0.541 0.764 1.158 -0.164 0.048 0.531 -0.044 -0.241 0.01 -0.224

S76875 -0.04 0.633 0.589 0.869 1.157 0.376 0.227 0.69 0.086 -0.241 0.077 -0.353

BM155767 0.593 0.913 0.7 1.344 1.155 0.212 0.291 0.435 -0.044 -0.03 -0.059 -0.092

BF157841 -0.09 -1.141 0.017 0.652 1.155 0.007 0.338 1.013 0.101 0.148 0.062 0.118

BG305367 0.283 0.13 0.038 0.648 1.153 0.534 0.63 1.037 0.078 0.1 0.002 0.404

BI878952 -0.015 0.674 0.466 1.036 1.152 0.949 0.259 0.428 -0.048 0.084 0.107 -0.262

AI979356 -0.382 -0.561 0.783 0.569 1.152 1.043 0.456 1.207 0.648 0.57 0.167 -0.054

BI673487 0.207 0.846 1.328 1.305 1.15 0.35 0.874 1.165 0.426 0.401 -0.047 -0.586

BI891176 0.195 0.258 0.646 0.741 1.15 0.669 0.816 1.073 0.309 0.395 -0.032 0.092

AW116617 1.164 0.874 1.055 1.172 1.148 2.02 0.9 1.451 0.382 0.559 0.27 -0.002

AW203151 0.578 0.55 1.365 1.15 1.147 2.004 1.441 1.395 0.912 0.887 0.548 0.191

BI892210 -2.439 -2.367 -0.129 0.23 1.147 1.659 1.434 1.532 1.332 1.308 0.993 1.009

BI888520 0.902 0.755 1.51 1.736 1.142 0.16 -0.063 0.257 0.298 -0.006 0.104 -0.062

AI793830 -0.348 -0.007 0.559 1.02 1.138 1.832 0.884 1.421 0.943 1.125 0.448 0.123

BI890848 1.3 -0.596 1.444 1.07 1.136 1.949 0.975 1.323 1.069 0.578 -0.068 -0.389

AI601641 0.384 1.425 -0.648 -0.115 1.136 1.503 0.612 1.368 0.765 -0.259 0.074 0.041

AW170898 0.34 0.706 0.471 1.125 1.136 0.249 0.446 0.729 0.321 0.162 -0.147 0.127

BM103179 -0.068 0.604 1.426 1.578 1.132 0.69 -0.049 0.704 0.251 0.21 0.044 -0.12

BI886133 -0.728 0.545 0.407 1.341 1.131 0.022 -0.089 0.193 -0.328 -0.749 -0.129 -0.358

BI879735 0.526 0.463 0.792 0.759 1.127 1.658 0.705 1.407 0.481 0.899 0.308 0.166

BM183759 0.401 1.259 1.157 1.472 1.126 0.743 0.496 1.392 0.421 0.396 0.295 0.032

AI957472 0.583 0.498 0.649 0.819 1.125 1.292 0.762 0.624 0.48 0.306 -0.025 -0.078

BI841477 -0.549 0.388 0.506 0.828 1.125 0.551 0.293 1.081 0.034 0.184 0.296 0.043

AI878713 -0.205 -0.058 0.224 0.667 1.124 0.782 0.633 0.831 0.122 -0.048 -0.457 -0.234

BG985522 -0.791 -0.142 1.535 1.639 1.122 0.92 0.337 0.759 0.492 0.211 0.549 0.071

BI883231 -0.227 0.812 0.461 0.657 1.12 0.465 0.493 1.022 0.075 0.067 0.061 -0.263

BM096052 0.338 0.893 0.545 0.781 1.119 0.756 0.268 1.033 0.254 0.441 -0.164 -0.131

BG737927 -0.728 0.545 0.666 0.969 1.115 -0.29 -0.053 0.618 0.146 -0.717 0.122 -0.25

BM023720 -0.237 -0.23 0.489 0.625 1.111 0.593 0.643 0.786 0.472 0.056 -0.344 -0.077

BI887480 -0.005 0.618 0.788 1.018 1.11 0.193 0.346 0.912 0.019 0.28 0.385 -0.104

BI880524 -3.095 -1.639 1.033 1.548 1.109 0.924 0.341 0.306 -0.034 0.784 0.765 0.84

AF168614 -0.314 0.874 0.981 1.126 1.105 0.882 0.512 0.686 0.375 0.042 -0.362 -0.728

BI886872 -0.53 0.192 0.258 0.196 1.105 -0.065 0.565 0.918 0.25 0.115 0.302 0.882

BI890893 0.874 0.005 0.708 0.413 1.103 0.537 0.375 1.064 -0.033 -0.3 -0.119 -0.031

BI891666 -0.728 -0.147 0.656 1.377 1.101 -0.147 0.31 0.848 0.298 -0.251 -0.038 0.133

BI888828 0.773 1.088 0.918 1.233 1.101 1.198 0.506 0.807 0.087 0.554 0.185 -0.007

BI891429 -0.367 0.558 0.659 0.98 1.101 0.506 0.479 0.83 0.23 -0.048 -0.066 -0.223

AW422167 0.731 -0.04 0.759 1.17 1.098 1.125 0.506 0.501 -0.146 -0.48 -0.834 -0.624

BM083945 -0.678 0.306 0.34 0.776 1.097 0.217 0.422 1.076 0.525 -0.129 -0.106 -0.469

BI884752 -0.201 0.837 1.809 1.89 1.095 -0.149 0.152 0.159 0.067 0.326 -0.21 -0.341

BI887101 0.42 0.935 0.738 0.545 1.094 0.201 0.032 0.862 -0.054 -0.096 -0.116 -0.37

AF132445 -0.272 0.398 0.602 0.77 1.093 0.46 0.076 0.642 -0.003 0.186 0.342 -0.019

BI877917 0.211 0.446 0.36 0.563 1.09 0.332 0.28 0.396 0.063 -0.022 -0.497 -0.595

BI708259 0.791 0.14 0.616 1.035 1.09 1.226 0.64 0.976 0.436 0.828 0.341 0.321

BM184248 0.067 -0.07 -0.375 0.895 1.088 -0.219 -0.367 -0.422 -0.579 -1.084 -0.88 -0.899

AF359424 -0.764 -0.125 1.203 1.285 1.086 0.495 0.352 0.713 0.075 0.048 0.386 0.002

BI889700 1.168 0.491 0.378 0.794 1.082 1.364 0.895 0.817 0.051 -0.233 -0.257 -0.623

BM083940 0.243 -0.894 0.175 0.614 1.082 1.178 0.693 0.842 0.969 0.103 0.334 0.181

BI671843 0.981 0.709 0.699 0.495 1.081 1.446 0.977 1.355 0.8 -0.191 -0.577 -0.349

AW171011 0.32 -0.569 0.953 1.037 1.08 1.518 1.208 0.945 0.948 0.336 -0.114 -0.163

AW171228 -0.034 -0.208 1.343 1.244 1.08 1.389 0.999 1.205 0.906 0.933 0.543 0.409

L47669 -2.241 -1.942 0.378 0.997 1.079 1.453 1.127 0.92 0.463 0.72 0.449 0.124

AW232471 -0.509 -0.271 0.135 1.05 1.079 0.311 0.073 0.323 0.045 -0.114 -0.287 -0.218

BI890305 -0.728 -0.028 0.201 1.267 1.079 -0.03 0.245 0.844 0.264 0.262 0.872 0.652

BI888914 1 0.771 0.739 1.068 1.076 0.646 0.608 0.696 -0.097 0.043 -0.485 -0.677

AF031378 -0.268 -0.348 -0.122 0.849 1.075 -0.02 0.253 0.442 0.161 -0.056 0.767 0.447

BI891279 -1.118 -1.771 0.474 0.973 1.074 0.88 0.705 0.954 0.789 0.743 0.262 -0.45

AI958208 -0.514 0.139 0.939 0.894 1.072 1.263 0.683 0.912 0.67 0.711 0.442 0.594

BI878102 0.5 0.884 1.149 1.288 1.07 1.511 0.962 1.088 0.688 0.711 0.114 -0.147

BF157344 -0.779 -0.292 -0.093 0.931 1.069 -0.161 0.16 0.912 0.065 0.588 0.479 0.293

BI887931 -0.629 -0.056 0.427 0.792 1.069 1.367 0.726 1.058 0.374 0.412 0.642 0.431

AI641716 0.317 0.322 0.714 0.954 1.067 0.457 0.371 0.875 0.204 0.291 0.529 0.078

BI979955 1.272 1.184 0.859 1.2 1.062 1.67 0.316 1.025 0.172 0.38 0.043 0.215

BI891993 -0.097 0.711 1.056 1.39 1.062 0.819 0.453 0.938 0.231 0.142 -0.414 -0.255

BI891136 0.025 0.361 0.758 0.955 1.059 0.578 0.688 0.847 0.435 0.313 -0.035 0.226

AW019421 -0.211 0.709 1.595 1.739 1.058 0.417 0.061 0.046 0.095 -0.43 -0.882 -1.063

BI888750 -0.189 -0.684 1.319 1.747 1.055 0.381 0.067 0.759 0.025 -0.231 0.036 0.097

AJ245965 0.024 0.446 0.157 0.569 1.049 1.102 0.557 1.021 0.386 0.698 0.639 0.072

AW165221 0.961 0.715 0.915 1.065 1.049 0.346 0.69 0.683 0.102 0.169 -0.199 -0.028

BI888859 -0.029 0.081 0.701 0.675 1.043 1.688 1.078 1.331 0.88 0.758 0.141 0.417

BI709805 -0.728 0.639 0.421 0.89 1.042 1.081 0.116 1.154 -0.378 -0.468 -0.543 -0.913

AI545284 -0.187 -0.769 0.34 0.057 1.041 1.21 0.552 0.771 0.562 0.41 0.504 0.377

AA495032 -0.181 -0.012 0.363 0.807 1.041 1.164 0.697 0.495 0.142 0.202 -0.017 0.121

BI886766 -0.098 0.955 1.181 1.193 1.04 0.896 0.096 0.531 0.359 -0.058 -0.329 -0.505

AI877869 -0.097 -0.094 0.089 0.54 1.04 0.286 0.058 0.962 0.017 0.312 0.337 0.186

BI878961 0.401 0.722 0.766 1.204 1.039 0.418 0.324 0.807 0.396 -0.111 -0.337 -0.117

AI723236 -0.343 0.006 0.612 0.858 1.039 0.361 -0.031 0.469 -0.096 0.06 0.393 -0.191

BI887477 -0.438 0.623 0.467 0.566 1.039 -0.04 0.248 0.833 0.102 0.354 -0.022 0.143

AF375226 -0.185 0.188 0.395 1.056 1.037 -0.039 0.364 0.808 0.131 0.035 -0.108 0.005

BM095301 -0.154 0.759 0.557 0.237 1.036 0.136 0.004 0.493 0.182 0.344 0.137 -0.012

AI641409 -0.197 -0.576 1.189 0.473 1.036 1.642 0.824 1.416 0.585 0.159 0.072 0.021

BI891608 0.458 1.304 1.193 1.447 1.035 0.793 0.284 0.46 -0.135 -0.102 -0.063 -0.526

BG985688 1.008 1.091 0.733 0.953 1.034 1.173 0.695 0.847 0.215 0.169 -0.382 -0.827

BI877866 0.366 0.664 1.018 1.156 1.034 0.352 0.335 0.594 0.302 0.265 -0.122 0.041

AW116486 -0.473 0.045 0.73 0.476 1.028 -0.438 0.178 0.858 0.436 0.202 0.072 0.196

AW466697 0.163 -0.143 -0.06 0.627 1.025 0.778 0.86 0.591 0.428 -0.105 -0.251 -0.303

BG727207 0.322 0.214 0.745 1.111 1.024 1.162 0.746 1.093 0.447 0.529 0 -0.036

BM182450 -0.395 -0.015 0.683 0.778 1.024 1.195 0.83 1.046 0.152 0.287 -0.085 -0.129

AI883714 0.06 0.446 0.3 0.724 1.024 0.295 0.269 0.558 -0.238 -0.49 -0.202 0.076

BM183973 1.164 1.241 1.479 1.526 1.023 1.063 0.601 1.079 0.582 0.651 0.275 -0.466

BM083944 0.127 0.415 0.645 0.595 1.022 0.341 0.286 0.556 0.076 0.378 0.251 0.158

BG306468 -0.159 -0.385 0.407 0.904 1.022 0.464 0.596 0.978 0.163 -0.26 0.111 0.195

AF428249 -0.922 0.003 0.306 0.538 1.021 -0.032 0.067 0.877 0.416 0.348 0.045 0.181

AI641454 0.093 0.655 1.033 1.221 1.018 0.028 0.224 0.41 -0.091 -0.049 -0.455 -0.218

BI706321 -0.484 0.142 0.338 0.991 1.014 0.312 0.059 0.287 -0.069 -0.554 -0.128 -0.341

AW344020 -0.509 1.088 0.972 1.342 1.013 -0.309 0.379 0.502 -0.125 -0.528 0.064 -0.242

AI331812 -0.575 -0.348 1.008 0.453 1.013 0.733 0.209 0.254 0.187 0.126 -0.036 0.227

AF140608 -0.054 0.063 0.275 1.049 1.012 0.39 0.447 0.915 0.135 0.612 0.499 0.233

AI657697 0.163 0.479 0.868 1.062 1.009 1.234 0.817 1.075 0.605 0.449 0.072 -0.113

BM036484 -0.542 0.214 0.61 0.705 1.008 -0.159 0.198 0.847 0.126 0.236 0.328 -0.111

AI723092 -0.676 0.067 0.391 0.868 1.008 0.306 0.499 0.884 0.216 -0.055 0.73 0.317

AW127725 -0.249 0.588 0.489 0.724 1.008 0.096 0.025 0.78 0.048 -0.173 -0.21 -0.073

AW076882 0.499 0.884 0.72 0.945 1.007 0.535 0.291 0.62 -0.026 0.031 -0.557 -0.383

BI890420 -0.058 0.13 0.475 0.769 1.007 0.828 0.715 0.763 0.446 0.192 0.262 -0.161

AY057057 0.411 0.189 0.399 0.644 1.005 0.528 0.108 0.682 0.09 -0.074 -0.144 -0.402

BE556841 -0.368 0.253 0.912 0.831 1.004 0.228 0.107 0.662 0.213 -0.258 0.647 0.161

AW233578 0.066 -0.61 0.567 -0.08 1.002 1.086 0.199 -0.178 -0.092 -0.188 -0.551 -0.135

AY045753 -0.49 -0.086 -0.084 0.658 1.002 0.45 0.158 0.502 -0.258 -0.101 -0.254 -0.024

AW454617 -0.549 0.479 1.151 1.328 1.002 0.716 0.574 0.596 0.144 0.368 -0.085 -0.039

AI816663 -0.45 0.383 0.801 0.659 0.999 0.49 0.347 0.361 0.393 -0.105 -0.104 -0.463

BI984810 -0.074 0.201 0.082 0.499 0.998 0.011 0.125 0.966 0.239 0.298 0.209 0.187

AJ005936 -1.251 -0.65 0.418 0.774 0.996 0.108 0.469 0.665 0.688 0.731 0.434 0.266

AB030899 -0.131 -1.333 0.113 0.429 0.994 1.176 0.857 0.967 0.45 0.391 0.494 0.16

BI889410 -0.529 0.073 1.206 1.115 0.994 1.008 0.815 0.351 0.332 0.453 -0.005 0.043

AW077190 0.074 -0.456 0.536 1.077 0.994 1.385 0.868 1.307 0.559 0.486 0.045 0.071

BI890038 0.182 1.28 0.64 1.501 0.992 0.426 -0.061 0.244 -0.221 -0.24 -0.535 -0.035

BM172681 -0.187 0.546 0.896 1.198 0.992 0.397 0.05 0.331 -0.07 -0.114 -0.321 -0.305

AI588395 -0.6 0.093 0.028 0.635 0.989 0.549 0.12 0.754 0.137 0.42 0.621 0.385

AI584575 -0.271 0.764 0.417 0.899 0.988 0.239 0.327 0.869 -0.142 -0.026 -0.068 0.292

AF258786 0.62 0.941 0.474 0.551 0.983 1.272 0.298 0.896 0.239 -0.318 -0.409 -0.388

BG304285 0.438 0.763 0.892 0.795 0.981 1.086 0.514 0.64 0.223 0.083 -0.139 -0.085

AW454605 0.279 -0.635 0.1 0.587 0.981 0.837 0.464 0.239 0.509 0.579 0.7 0.553

BG308220 -0.434 0.425 0.687 1.041 0.981 -0.019 0.489 0.61 0.129 -0.038 -0.235 -0.276

AI476945 -0.153 -0.091 0.317 0.17 0.98 -0.018 -0.117 0.544 -0.005 -0.103 0.598 -0.21

BM096064 0.957 0.912 1.315 1.776 0.976 0.866 0.77 0.945 0.435 0.23 0.035 -0.578

BI891423 -0.526 -0.081 0.793 0.609 0.976 1.246 0.653 1.158 0.406 0.7 0.264 0.424

BI878536 0.402 0.452 0.466 0.59 0.975 0.426 0.221 0.509 0.167 -0.242 -0.229 -0.183

BI890375 1.333 1.22 1.52 1.497 0.974 1.578 1.288 0.649 0.621 0.55 0.182 0.037

BI878475 0.2 0.725 1.388 1.372 0.973 0.497 0.327 0.758 0.358 0.094 0.006 0.421

AI588119 0.559 0.213 0.123 0.379 0.972 1.73 0.689 1.441 1.033 0.977 0.541 0.036

AA494837 0.897 0.492 0.584 1.001 0.972 0.092 0.701 1.004 -0.08 0.271 0.165 0.194

AI793701 0.068 0.575 0.466 1.301 0.962 0.516 0.435 0.978 0.321 0.376 0.286 0.09

BE693134 -0.123 0.248 0.159 0.495 0.962 0.351 0.447 0.74 0.327 0.171 0.392 -0.009

BG303721 0.628 0.26 0.85 0.344 0.959 1.681 0.808 0.823 0.573 0.586 0.183 -0.196

BM095922 0.032 1.012 0.948 1.141 0.959 -0.115 0.101 0.668 -0.03 -0.191 -0.319 0.179

BI891090 -0.208 0.555 0.246 0.929 0.957 0.626 0.304 0.409 -0.005 -0.439 -0.367 -0.173

BI888149 -0.111 -0.326 0.14 0.55 0.956 0.967 0.753 0.723 0.279 0.106 -0.016 0.11

AW344023 -0.728 -0.036 0.097 1.118 0.956 -0.451 -0.139 -0.636 -0.105 -1.127 -1.415 -1.233

BI864988 0.02 0.033 0.816 1.309 0.955 0.46 0.297 0.755 0.245 -0.151 0.057 -0.095

AW116601 0.186 0.207 1.063 1.001 0.955 0.32 0.297 0.793 0.121 0.583 0.617 0.508

AI793666 0.02 0.489 0.931 1.336 0.951 0.277 0.546 0.361 0.269 -0.137 -0.309 -0.304

BI867531 0.341 0.565 0.41 0.94 0.951 0.204 0.402 0.781 0.256 0.512 0.358 0.259

AI588743 -1.066 -0.039 0.733 1.169 0.949 0.389 0.062 0.22 0.937 0.386 0.074 -0.843

AI415831 -0.826 0.079 0.066 0.566 0.945 0.294 0.144 0.885 0.293 0.123 0.076 -0.243

BG304114 0.293 0.605 1.356 0.916 0.944 1.453 0.592 0.857 0.711 0.695 0.1 0.293

AW116232 0.592 0.462 0.205 0.64 0.944 0.457 0.336 0.769 -0.024 0.119 -0.222 -0.01

U16310 -0.92 0.663 1.142 1.317 0.939 -0.315 -0.03 0.432 -0.096 -0.327 -0.469 -0.194

BI890682 -0.009 0.164 0.389 0.747 0.938 0.005 0.177 -0.021 0.182 0.033 -0.18 -0.266

AI959157 0.003 -0.26 0.567 0.817 0.936 0.266 0.611 0.3 -0.123 0.033 -0.186 -0.068

AW076798 -0.235 0.403 0.88 0.938 0.935 0.673 0.1 0.756 0.402 0.116 -0.485 -0.856

BI891472 -0.293 -0.688 0.925 0.692 0.935 1.083 0.425 0.733 0.658 0.261 0.471 0.146

BE693173 0.389 0.159 1.046 1.356 0.934 1.353 0.592 0.566 0.136 -0.153 -0.532 -0.696

AI942839 -0.522 0.018 0.094 -0.188 0.934 0.067 0.11 0.738 0.456 0.153 0.543 -0.027

BI885768 0.066 -0.015 1.031 0.788 0.932 1.66 0.815 0.939 0.3 0.218 0.184 0.096

AI601856 0.392 0.307 0.127 0.694 0.928 1.055 0.77 0.237 0.212 0.058 0.034 0.285

BF157279 -0.142 0.679 0.397 1.203 0.927 -0.017 0.039 0.474 -0.151 -0.223 0.243 -0.343

AI794059 -0.196 -0.539 -0.123 0.075 0.925 1.626 1.021 1.183 0.916 0.755 0.559 0.532

BI887324 -0.18 0.3 0.749 0.54 0.922 0.868 0.448 0.641 0.309 0.131 -0.244 -0.486

BE016113 -0.567 -0.727 0.356 0.707 0.922 0.386 0.314 0.631 0.093 0.257 0.161 -0.05

AI964318 0.019 -0.1 0.453 0.368 0.921 1.184 0.796 1.044 0.18 0.108 -0.351 -0.859

AW171190 0.132 0.125 0.215 0.671 0.921 0.488 0.495 0.787 0.046 0.377 0.034 0.166

AW128293 0.898 1.158 1.219 0.862 0.92 1.376 0.501 0.876 0.483 0.6 0.213 -0.585

AI958373 -0.53 0.308 0.559 0.889 0.912 0.221 0.122 0.998 0.228 0.022 -0.37 -0.95

BG303647 0.794 1.098 1.247 1.363 0.91 1.352 0.62 0.589 0.348 0.344 -0.077 -0.175

AI794204 0.202 0.395 0.867 0.801 0.909 0.978 0.899 0.76 0.123 0.603 0.258 0.015

BI878962 0.344 0.698 0.808 0.805 0.909 0.359 0.282 0.562 0.02 -0.034 -0.201 -0.128

BE201470 -3.167 -1.166 0.12 0.73 0.907 1.468 0.849 0.831 0.873 0.891 0.373 0.364

AI877743 -0.093 -0.196 -0.001 -0.246 0.906 0.173 0.273 0.575 0.23 0.309 0.471 0.103

AI974195 0.266 0.98 0.663 1.144 0.905 0.396 0.456 0.809 -0.223 0.42 0.598 0.389

AW184334 -0.319 -0.02 -0.087 0.633 0.903 -0.025 0.138 0.522 0.323 0.419 0.268 0.171

BI888165 0.092 -0.167 0.22 -0.05 0.901 0.478 0.216 0.6 0.18 0.207 0.103 0.04

AW116453 -0.075 -0.592 -0.241 0.553 0.9 1.251 1.161 1.112 1.093 0.557 0.041 -0.288

BI889396 -0.505 -0.015 0.201 0.612 0.896 -0.013 0.181 0.681 0.179 0.03 0.289 -0.01

AF124332 -0.139 0.113 0.599 0.453 0.896 0.038 0.249 0.739 0.04 0.088 -0.151 -0.161

BI887512 -0.01 -0.297 0.309 0.401 0.896 1.086 0.374 1.037 0.313 0.662 0.113 0.246

AW115873 1.082 1.112 1.131 1 0.896 1.318 1.024 0.44 0.244 0.394 0.136 0.08

AI722650 -0.292 0.327 -0.009 0.211 0.895 1.289 0.768 1.201 0.816 0.342 0.206 0.164

BI892066 -0.363 -0.414 0.556 0.342 0.895 0.883 0.554 0.57 0.332 0.306 0.023 0.023

AI322210 0.442 -0.646 1.248 0.241 0.894 1.7 1.392 0.795 0.879 0.557 0.334 -0.499

BM005035 0.437 0.544 0.524 0.834 0.893 -0.123 0.279 0.555 -0.015 0.059 -0.14 -0.296

BI865976 -1.248 -0.042 0.828 1.11 0.892 0.673 0.411 -0.5 0.548 -0.104 0.274 -0.466

BI886522 -0.132 0.632 0.835 0.948 0.892 -0.14 0.048 0.332 0.006 0.041 -0.199 -0.183

BI671110 0.693 0.865 1.258 1.018 0.889 1.353 0.389 0.852 0.213 0.235 0.119 -0.031

AI723170 0.006 0.093 1.063 0.931 0.887 1.412 1 1.195 1.034 0.901 0.516 0.273

BI887627 -0.163 0.397 0.635 0.786 0.887 0.068 0.09 0.536 0.076 -0.163 -0.627 -0.594

AW116425 0.321 -0.637 0.278 0.85 0.885 1.145 0.691 0.932 0.193 0.474 0.027 -0.093

AI641272 0.257 -0.569 0.765 0.707 0.884 0.081 -0.03 0.524 -0.356 -0.046 -0.053 -0.259

BI887651 0.047 -0.561 0.071 0.169 0.882 1.121 0.733 0.842 0.241 -0.02 -0.135 -0.136

BE202229 0.522 -0.773 0.196 0.605 0.882 1.213 1.009 1.1 0.681 0.48 -0.081 -0.179

U84616 0.033 -0.002 0.935 1.018 0.881 0.137 0.408 0.671 0.101 0.241 0.118 -0.067

BM034969 0.851 1.274 1.297 1.516 0.881 1.472 0.591 0.867 0.534 0.253 0.044 -0.088

BM101689 0.514 -0.129 0.086 0.194 0.879 1.226 0.403 0.597 0.725 0.483 0.677 0.466

BG303999 0.185 0.386 1.173 1.145 0.879 0.708 0.519 0.838 0.81 0.809 0.41 0.726

AI722567 -0.036 -0.045 1.117 1.003 0.878 0.75 0.565 0.507 0.532 0.076 0.232 -0.438

BG728404 0.409 0.156 0.225 0.591 0.877 0.25 0.508 0.736 -0.124 0.163 0.137 0.06

AW305388 0.553 0.168 0.443 0.643 0.877 1.888 1.101 1.388 0.946 1.128 0.938 0.51

AW116226 0.287 0.543 0.858 0.704 0.877 1.48 0.697 0.744 0.338 0.342 -0.107 0.018

BM182574 -0.728 0.153 0.303 0.514 0.875 0.116 -0.087 0.629 -0.099 -0.203 0.076 -0.218

AI957850 0.06 0.696 0.927 0.997 0.872 0.221 -0.097 0.402 0.122 -0.399 0.011 -0.42

AI667326 0.028 0.772 1.289 1.623 0.868 1.361 0.476 0.787 0.16 0.148 0.043 -0.155

BI880125 0.321 -0.05 0.512 0.455 0.866 1.326 0.593 0.526 0.448 0.692 0.312 0.338

BI875704 -0.124 0.598 0.496 0.893 0.865 0.517 0.335 0.647 -0.175 0.01 -0.064 -0.166

AB011826 0.083 0.58 0.583 0.899 0.865 0.125 0.202 0.154 -0.064 0.099 0.182 -0.196

BI428543 0.045 -0.195 0.882 1.007 0.864 0.855 0.739 0.559 0.127 0.461 0.017 0.457

BI888863 -0.06 0.227 0.663 0.729 0.864 0.746 0.249 0.931 0.276 0.352 0.047 -0.012

BM070534 -0.495 0.627 0.749 1.081 0.86 -0.033 -0.123 0.473 0.198 0.16 0.089 0.069

BI984272 0.076 0.185 0.275 0.575 0.859 0.136 -0.309 0.849 0.715 0.339 -0.075 0.034

AW344255 0.409 0.639 0.828 0.945 0.859 0.828 0.619 0.692 0.465 0.237 -0.002 -0.322

BE605766 -0.173 0.309 0.738 1.079 0.856 0.166 0.349 0.623 0.224 0.666 0.222 0.121

AW019740 -0.814 -0.001 0.605 1.051 0.851 0.495 0.202 0.344 -0.231 -0.289 -0.208 -0.379

BE693152 -0.338 0.173 0.705 0.821 0.851 1.33 0.865 1.176 0.501 0.69 0.416 0.357

AI957604 -0.095 -0.072 0.101 0.142 0.85 1.088 0.627 0.896 0.596 0.455 0.671 0.669

AI667530 -0.08 0.15 1.032 0.963 0.85 0.448 0.312 0.498 0.03 -0.121 -0.711 -0.293

AI667167 -0.613 -0.133 1.09 0.248 0.846 1.755 0.972 1.149 1.245 0.806 0.397 0.227

BI883903 0.092 -0.412 0.496 0.613 0.845 0.185 -0.085 -0.255 -0.084 0.036 -0.061 0.38

BI888550 -0.756 -0.802 -0.356 0.293 0.844 1.217 0.629 0.812 -0.286 -0.878 -1.165 -1.088

AW281646 0.03 0.884 1.149 1.438 0.841 0.748 0.273 0.726 0.004 0.233 0.127 0.164

AI641717 0.464 0.648 1.092 0.632 0.841 1.251 0.993 0.793 0.319 0.6 0.119 0.056

AI957711 -0.701 -0.494 -0.405 0.512 0.84 1.262 0.538 0.731 0.537 0.295 0.158 -0.161

AW233689 0.36 0.274 0.995 1.173 0.84 0.594 0.389 0.708 0.256 0.293 -0.177 -0.289

BI889456 -0.566 -0.272 0.348 0.576 0.838 1.186 0.624 1.047 0.575 0.367 0.239 -0.019

BM103255 -0.553 -0.795 0.761 0.706 0.836 0.138 0.404 -0.153 0.458 0.168 -0.255 -0.722

BM185124 0.687 -0.161 0.633 0.808 0.835 0.966 0.773 0.667 -0.006 -0.212 -0.475 -0.644

AI957847 -0.228 0.331 0.561 1.037 0.834 0.433 0.431 0.776 0.008 0.103 -0.09 -0.066

AI722764 -0.272 0.001 0.826 0.677 0.834 0.166 0.153 0.782 0.222 0.041 0.152 -0.086

AF093129 -1.54 -1.446 0.378 0.752 0.834 1.562 1.1 0.85 0.377 0.503 0.231 0.039

BM082684 -0.287 0.758 0.818 0.888 0.833 0.068 0.288 0.193 -0.191 -0.092 -0.069 0.045

BI888732 1.092 0.083 0.784 0.188 0.832 1.41 0.606 0.289 0.584 0.479 0.388 0.356

BI705734 -0.614 -0.092 0.379 0.511 0.832 0.082 0.036 0.305 -0.171 -0.707 0.05 0.033

AI601529 -0.672 -0.08 0.451 0.677 0.831 -0.089 -0.27 0.284 -0.122 -0.539 0.453 0.031

BI843117 0.359 0.803 0.531 1.109 0.83 0.267 0.244 0.644 -0.102 0.063 -0.24 -0.069

AW077743 0.49 0.492 -0.105 0.647 0.83 0.485 0.301 0.704 0.445 -0.082 0.031 -0.179

AW116374 0.124 0.481 0.866 0.714 0.828 0.871 0.782 0.763 -0.148 0.353 -0.116 -0.233

AW115702 0.163 0.232 0.522 0.899 0.828 0.165 0.161 -0.161 -0.216 -0.292 -0.356 -0.463

BM181821 0.83 1.029 1.35 1.496 0.827 0.816 0.258 0.533 0.122 0.099 -0.058 -0.401

AI964207 0.285 0.825 0.812 0.869 0.825 -0.043 0.347 0.461 -0.125 0.009 -0.226 -0.162

AF164726 0.022 0.02 0.534 0.502 0.825 0.049 0.251 0.177 -0.179 -0.262 -0.622 -0.651

BM095746 -0.173 0.343 0.492 1.085 0.822 0.258 0.015 0.23 0.418 -0.009 0.918 0.043

BI983593 0.341 0.295 0.603 0.994 0.822 0.859 0.729 0.56 0.073 0.398 -0.06 0.489

BI885777 -0.784 -0.029 0.569 0.662 0.821 1.282 0.758 0.736 0.451 0.552 0.249 0.475

AW233144 -0.01 0.402 0.551 0.852 0.818 0.541 0.637 0.773 0.181 0.319 0.356 0.094

AW078394 0.28 0.389 0.75 1.017 0.814 0.722 0.18 0.624 0.426 0.583 0.836 0.781

AW018998 -0.728 0.148 0.766 0.85 0.813 0.046 -0.219 0.193 -0.167 -0.36 0.147 -0.194

BI889553 0.197 0.476 0.267 0.678 0.813 0.172 0.18 0.381 -0.37 -0.026 -0.292 -0.117

AI883443 -0.609 -0.381 0.454 0.59 0.807 0.921 0.411 0.795 0.354 0.664 0.392 0.433

BM103822 -0.228 0.162 0.826 0.553 0.806 1.076 0.597 0.976 0.529 0.659 0.236 -0.036

AI437388 -0.494 -0.04 0.003 0.336 0.806 0.104 0.17 0.668 0.127 0.204 0.178 -0.033

AW422582 -0.076 0.744 0.563 1.024 0.804 0.204 -0.127 -0.137 0.069 -0.332 -0.581 -0.557

AI667083 0.266 0.081 0.14 0.262 0.801 0.441 0.135 0.761 0.142 0.157 -0.005 0.037

BI880444 0.459 -0.006 0.499 0.756 0.799 0.305 0.546 0.177 0.024 -0.128 -0.197 -0.035

BI980132 0.124 0.716 0.717 1.007 0.796 -0.056 -0.122 0.154 0.12 -0.187 -0.348 -0.708

AI878344 0.25 0.321 0.613 0.718 0.794 0.435 0.437 0.418 0.093 0.315 -0.174 -0.539

BI882568 -0.029 0.307 0.641 0.672 0.793 1.09 0.373 0.719 0.303 0.151 -0.184 -0.406

BI886016 -0.365 0.292 0.635 1.121 0.791 0.647 0.738 0.923 0.476 0.376 0.054 0.002

BI892038 -0.43 0.435 0.69 0.799 0.791 0.562 0.175 0.695 0.193 0.045 -0.05 -0.187

AI545320 0.348 0.042 0.842 0.593 0.79 1.042 0.978 0.831 0.605 0.494 0.524 0.264

BI325086 -0.137 0.481 0.156 0.675 0.786 0.25 0.436 0.084 0.413 0.386 -0.283 -0.374

BI889944 -0.395 0.587 0.875 0.856 0.786 0.111 0.099 -0.353 0.295 -0.425 -0.366 -0.463

BI887704 0.016 0.222 0.854 0.523 0.784 1.005 0.552 0.881 0.441 0.495 0.257 0.411

BI892201 0.341 0.247 0.338 0.293 0.783 1.176 0.894 1.006 0.663 0.099 0.161 -0.127

AI722421 -0.653 0.171 0.763 0.981 0.783 0.17 -0.085 0.665 0.125 0.346 -0.166 0.213

BG985493 0.379 -0.001 0.841 0.602 0.78 1.435 1.206 0.955 0.589 0.43 0.071 -0.034

AI477432 0.338 0.127 0.755 0.766 0.774 1.349 0.993 1.105 0.715 0.406 0.307 0.257

BI891108 0.618 0.51 1.673 -0.115 0.771 1.507 0.535 0.738 0.229 0.12 -0.073 -0.144

AW059158 -0.081 0.08 0.469 0.673 0.771 -0.128 0.414 0.705 0.228 0.159 -0.186 -0.087

BI887350 -0.22 0.716 0.636 0.243 0.771 0.839 0.511 0.631 0.119 0.082 -0.056 -0.379

BI887309 -0.035 -0.251 0.865 0.795 0.768 0.277 0.087 0.769 0.231 0.333 -0.219 -0.197

BI878903 0.185 0.704 0.537 1.096 0.767 0.332 0.123 0.04 -0.197 -0.152 -0.45 -0.526

AI793733 0.126 0.386 0.381 0.51 0.765 0.367 0.154 0.595 0.352 0.303 0.085 -0.078

BI888024 0.143 -0.936 0.281 0.619 0.761 0.774 0.684 0.575 0.476 0.383 -0.002 0.245

BI878059 -0.728 0.579 0.735 1.232 0.758 -0.045 0.161 0.699 0.149 -0.099 0.369 -0.178

AI721340 0.433 -0.438 0.269 0.028 0.757 1.373 0.738 1.051 0.793 0.768 0.457 0.267

BI891235 -0.06 0.346 0.655 1.159 0.755 0.002 0.027 -0.096 -0.048 0.118 0.036 -0.129

BI890749 -0.107 0.06 0.61 0.57 0.754 0.105 0.328 0.714 0.561 0.256 0.059 -0.092

BI889332 0.355 0.464 0.409 0.466 0.754 0.317 0.243 0.145 0.009 0.091 -0.199 -0.126

AF160659 -1.165 -0.489 0.023 0.785 0.753 0.326 0.069 0.624 0.318 0.147 0.352 0.161

AW128354 0.458 0.223 0.798 0.66 0.751 1.138 0.383 0.799 0.625 0.669 0.174 0.147

AF124396 -0.369 -0.12 0.494 0.799 0.75 0.38 0.329 0.678 0.139 0.258 0.455 0.172

AW567345 0.624 0.916 1.29 1.332 0.75 0.073 0.493 0.541 -0.015 -0.075 -0.27 -0.27

BF718182 -0.725 -0.545 -0.939 0.616 0.749 0.866 0.312 0.676 0.215 0.777 0.423 0.815

BF713861 0.139 0.824 1.033 1.192 0.748 -0.046 0.013 -0.355 0.024 -0.061 -0.231 -0.51

BI890257 0.049 0.658 0.491 0.495 0.747 0.835 0.401 0.806 0.181 0.194 -0.003 0.026

AB017117 0.082 0.723 1.193 1.264 0.747 1.196 0.673 0.649 0.267 0.523 0.127 -0.002

BI886779 -0.012 -0.02 0.709 1.092 0.747 0.35 0.34 0.279 0.009 -0.055 -0.199 -0.026

BM005197 0.677 0.44 0.449 0.79 0.746 -0.02 0.187 0.588 -0.066 0.021 -0.436 -0.322

AI641066 -0.024 0.629 1.026 1.216 0.744 0.113 0.125 0.613 0.116 0.402 0.32 -0.095

AI943086 -0.645 0.012 0.158 0.652 0.742 0.24 0.328 0.563 -0.21 -0.081 0.632 0.37

AF210637 -0.817 -0.619 0.045 0.632 0.741 0.103 0.219 -0.06 0.481 0.328 -0.015 -0.148

AI964285 -0.015 0.487 0.512 0.578 0.741 0.184 0.069 0.117 0.467 0.07 0.503 0.083

AI959694 0.345 -0.045 1.047 1.182 0.737 0.699 0.446 0.258 0.091 0.604 0.368 -0.139

BI980800 -0.433 -0.441 -0.095 1.71 0.735 0.844 0.375 0.905 0.537 0.931 0.911 0.806

BM102647 -0.504 0.538 0.496 0.894 0.735 -0.175 0.137 0.848 0.131 0.199 0.062 0.147

BM183157 0.641 0.921 1.471 1.574 0.735 1.038 0.197 1.293 0.655 0.975 0.283 0.306

BI705525 0.913 0.826 0.36 0.629 0.734 1.295 0.191 0.768 0.267 0.271 -0.195 -0.177

BE201596 -0.281 0.592 0.524 0.277 0.73 1.772 1.511 1.459 0.765 0.759 0.341 0.42

BI888908 0.105 0.683 0.673 0.622 0.727 0.433 0.245 0.346 -0.056 -0.186 -0.391 -0.403

BE017549 -0.464 0.275 0.391 0.543 0.726 -0.141 -0.215 0.495 0.232 0.046 0.099 -0.224

BI885994 0.18 0.544 0.592 0.542 0.726 0.318 0.095 0.315 0.108 0.11 -0.095 -0.174

BM103291 0.314 0.581 0.257 0.677 0.721 -0.127 0.151 0.644 0.142 0.09 -0.262 -0.221

BI476846 -0.546 -1.171 -1.035 0.201 0.715 1.199 0.479 0.38 0.234 0.752 0.489 1.006

BI705506 -0.592 -0.513 0.105 0.828 0.714 0.566 0.508 0.633 0.291 0.431 0.032 0.169

BG303768 0.868 0.85 0.981 1.233 0.713 0.203 0.541 0.267 -0.02 -0.057 -0.838 -0.632

AW203061 -1.304 -0.998 -0.442 0.148 0.713 1.429 0.707 1.075 0.895 1.033 0.507 0.258

AW280152 -0.138 -0.035 -0.291 1.18 0.712 0.174 -0.05 0.237 0.054 0.027 -0.049 0.533

AW165301 -0.464 0.498 0.736 0.873 0.711 -0.261 0.379 0.268 -0.18 -0.267 -0.249 0.042

AW175486 0.154 -0.374 -0.066 0.469 0.707 0.462 0.211 0.439 -0.101 0.203 -0.188 -0.299

BI984060 0.317 0.207 0.532 0.329 0.704 0.337 0.24 0.606 0.07 0.349 0.107 0.023

BM185057 0 0.699 0.79 1.342 0.701 0.223 0.233 0.235 -0.245 -0.404 -0.382 0.231

AI558282 -0.201 0.225 0.372 0.829 0.701 -0.136 0.126 0.435 0.166 -0.009 0.4 0.559

BI876201 -0.562 0.025 0.774 0.737 0.7 0.026 0.41 0.216 0.72 -0.006 -0.442 -1.233

BI705891 0.076 -0.753 0.636 0.627 0.696 1.263 0.992 1.09 0.645 0.533 0.268 0.382

BI981134 0.03 0.417 0.278 0.54 0.694 -0.077 0.225 0.14 0.037 0.077 -0.111 -0.037

BM181828 0.774 0.223 0.413 1.023 0.693 0.821 0.752 0.358 0.316 0.231 -0.125 -0.165

AW058867 -0.019 0.25 0.637 1.037 0.693 0.169 0.39 0.537 0.218 0.375 0.227 0.029

BI888576 -0.269 0.899 0.889 1.035 0.693 -0.039 0.349 0.999 0.281 0.397 0.075 -0.436

AI584348 -0.017 0.242 0.541 0.778 0.689 -0.188 0.175 0.752 -0.031 -0.2 -0.164 0.086

BM184696 -0.635 0.218 0.733 0.782 0.688 -0.061 -0.077 0.299 0.36 0.158 0.26 -0.303

BI325924 0.53 1.193 1.161 1.4 0.687 0.088 -0.207 -0.025 -0.154 -0.924 -0.464 -0.766

AI884211 0.299 1.061 1.446 1.511 0.686 0.695 0.184 0.675 0.208 0.447 -0.169 -0.439

BI886025 -2.881 -1.279 -0.295 -0.28 0.686 1.605 1.052 1.385 0.65 1.423 1.05 0.933

AI793925 -0.348 0.651 0.648 0.857 0.685 0.131 -0.152 0.096 -0.384 -0.216 0.318 -0.309

BM102339 0.093 0.778 0.866 1.235 0.683 0.285 -0.138 0.22 -0.323 -0.345 -0.437 0.27

AI667325 -0.598 -0.28 1.121 0.333 0.682 1.574 0.927 1.124 0.786 0.921 0.682 1.106

BG985504 -0.057 0.376 0.675 0.424 0.681 0.794 0.101 0.587 0.292 0.338 0.325 0.074

AW777355 0.753 0.203 0.736 0.664 0.679 1.237 0.919 0.885 0.721 0.543 0.231 -0.008

AW171454 -0.283 -0.063 0.018 0.295 0.679 1.254 0.515 1.25 0.621 0.857 0.376 0.249

BI980138 0.342 -0.22 0.086 0.407 0.679 1.142 0.413 0.762 0.211 0.268 -0.013 -0.171

AF274877 0.095 0.558 0.122 0.587 0.677 1.137 0.273 0.804 0.228 0.401 0.338 0.127

BI877921 0.281 -0.439 0.729 0.519 0.675 1.277 1.193 0.899 0.417 0.414 -0.02 0.047

BI883242 0.488 0.994 0.915 0.85 0.673 1.098 0.285 0.758 0.185 0.22 0.175 0.342

BI704334 -0.37 -1.092 0.676 1.06 0.673 -0.031 0.064 -0.185 -0.454 -0.508 -0.564 -0.576

BI980725 0.123 -0.522 0.042 0.58 0.671 0.29 0.204 0.63 -0.117 0.308 0.025 0.45

BG308318 -0.728 0.366 0.439 0.524 0.67 -0.058 -0.331 0.229 -0.306 -0.589 -0.18 -0.265

BI891331 -0.696 -0.05 0.418 0.563 0.666 -0.15 -0.051 0.304 -0.048 0.321 0.174 0.217

AI957869 0.875 0.925 1.238 0.883 0.665 1.637 0.751 1.474 0.948 0.696 0.059 -0.429

AI354177 -0.336 0.27 0.211 0.358 0.664 1.093 0.646 0.947 0.325 0.216 -0.043 0.038

AW281574 -0.364 -0.491 0.221 0.648 0.66 -0.027 0.135 0.515 -0.128 0.514 0.255 0.479

AI878421 0.928 0.298 0.001 0.359 0.66 1.096 1.008 0.962 0.632 0.727 0.55 0.736

AW170850 0.012 0.141 0.224 0.473 0.655 0.091 0.146 0.207 -0.029 -0.296 -0.531 -0.269

BI891493 -0.326 0.14 0.183 0.539 0.653 0.157 0.159 0.54 0.319 0.132 -0.248 -0.185

BI886477 0.255 0.374 0.554 1.213 0.652 -0.071 0.353 0.833 0.133 0.267 0.194 0.087

AW171172 -0.189 0.162 0.197 0.306 0.651 0.221 0.075 0.558 -0.161 0.09 0.531 0.169

BI867929 0.542 0.533 0.161 0.643 0.646 -0.096 0.283 0.364 -0.045 -0.081 -0.071 -0.08

BI843229 -0.197 -0.159 0.272 0.295 0.645 1.666 0.914 1.167 0.626 1.01 0.149 0.162

AW077976 -0.703 -0.171 -0.006 0.488 0.64 0.46 -0.123 -0.024 -0.216 -0.065 -0.383 -0.408

BI888926 -0.608 -1.851 -0.112 0.11 0.638 1.155 0.613 0.55 -0.029 0.704 0.383 0.495

AW777430 0.613 -0.148 0.717 0.675 0.637 1.325 0.884 0.479 0.697 0.712 0.467 0.014

AF062643 -0.284 0.202 0.658 0.743 0.637 0.505 0.166 0.346 -0.022 0.087 0.51 0.013

BI878743 0.242 0.682 0.711 0.989 0.636 0.184 0.506 0.77 0.238 -0.29 -0.141 -0.012

AF169146 0.215 0.419 0.692 0.782 0.635 0.85 0.182 0.211 0.034 0.058 0.223 0.002

BM182673 0.092 0.47 0.523 0.623 0.634 -0.026 -0.045 0.125 0.066 -0.168 -0.295 -0.327

AI667069 0.171 0.513 0.453 0.338 0.632 0.161 0.214 0.095 0.025 0.136 -0.058 0.065

BI890609 0.567 -0.045 0.172 0.224 0.632 1.762 1.62 0.936 0.869 0.138 -0.419 -0.864

AW232866 -1.023 -0.546 -0.786 -0.074 0.631 2.158 1.541 1.99 1.424 0.46 -0.095 -0.226

BI890789 -0.617 0.786 0.794 1.114 0.631 0.513 0.004 0.503 0.134 -0.03 -0.038 -0.164

BI886564 -0.411 -0.067 0.52 0.568 0.631 -0.11 0.306 0.395 0.259 0.09 -0.117 -0.405

BI878904 0.698 0.553 0.861 0.661 0.627 1.082 0.368 0.459 0.357 0.222 0.231 0.306

AA494762 0.505 -0.047 0.173 0.56 0.624 0.742 0.684 0.446 0.475 0.064 -0.043 -0.186

BI878043 -0.369 -0.754 0.427 0.8 0.621 0.772 0.387 0.374 0.361 0.452 0.319 0.258

BG302933 -0.13 0.263 0.743 0.865 0.62 -0.284 0.01 0.291 0.278 0.192 0.105 0.3

BI672214 -0.075 0.335 0.66 0.786 0.62 0.046 0.149 0.499 0.06 0.154 0.19 0.183

BI885824 0.109 -0.025 0.496 0.772 0.62 0.893 0.551 0.797 0.218 0.394 0.223 -0.137

BI672045 0.49 -0.19 0.056 0.397 0.617 0.367 0.343 0.595 0.088 0.091 0.087 0.105

AF177868 -0.136 0.466 0.868 1.318 0.615 1.058 0.538 0.727 0.537 0.238 0.179 0.272

AW171254 0.464 -0.553 0.21 0.504 0.612 0.955 0.476 0.665 0.413 0.486 0.221 0.226

BI891276 0.109 -0.73 0.448 0.585 0.612 0.694 0.348 0.725 0.214 0.344 0.417 -0.147

AI558661 -0.107 -0.48 0.162 0.061 0.61 -0.053 0.27 -0.127 -0.333 -0.094 -0.605 -0.836

BE693119 -1.16 -0.795 0.469 0.5 0.61 0.464 -0.272 -0.563 -0.082 0.502 0.33 0.078

AI883980 -0.404 0.422 0.543 0.65 0.609 0.128 0.452 0.363 0.254 -0.305 0.261 -0.311

BI886691 -0.079 0.215 0.428 0.943 0.605 0.185 0.293 0.32 0.099 0.086 -0.16 -0.301

AW280174 0.87 -0.103 0.08 0.687 0.604 1.356 1.242 1.233 0.643 0.884 0.415 0.257

BI878907 -0.15 0.253 0.353 0.5 0.598 -0.046 -0.136 0.049 0.035 -0.18 -0.089 -0.135

BE605721 1.023 1 0.816 0.814 0.596 1.134 0.562 0.986 0.575 0.396 0.148 0.009

AF387342 -0.103 0.646 0.609 0.746 0.591 0.147 0.347 0.341 0.181 -0.206 -0.221 -0.887

AW116726 -0.351 -0.017 0.796 0.813 0.59 -0.145 -0.177 0.122 0.005 -0.451 -0.668 -0.484

AI497192 -0.397 0.109 0.183 0.478 0.585 -0.141 -0.116 0.277 0.055 0.214 0.152 -0.2

AI878080 0.046 0.113 0.796 0.813 0.584 0.409 0.225 0.889 0.133 0.341 0.168 0.293

AW018941 -0.728 0.121 0.11 0.437 0.581 -0.125 -0.029 0.215 -0.378 -0.557 -0.543 -0.913

BI670969 0.465 0.996 0.739 1.126 0.581 0.955 0.51 0.999 0.635 0.901 0.381 0.071

AI965130 -0.282 0.467 0.761 0.863 0.579 0.127 -0.046 0.271 0.01 0.397 0.201 0.107

BI887270 -0.852 -0.655 0.027 0.392 0.578 0.678 0.374 0.395 0.221 0.161 0.25 0.603

BI891069 0.147 0.413 0.608 0.691 0.576 0.129 0.181 0.765 0.004 0.14 -0.093 -0.365

AW115633 0.286 0.317 0.44 0.746 0.576 0.451 0.227 0.792 0.258 0.583 0.078 0.153

BI891859 -0.106 0.101 0.33 0.542 0.575 0.437 0.065 0.011 0.306 -0.574 -0.437 -0.347

BM181598 -0.078 0.544 0.669 0.675 0.568 -0.173 0.295 0.674 0.158 0.203 -0.15 -0.429

BI879262 -0.437 -0.354 0.563 0.638 0.566 1.416 0.814 0.716 0.233 0.507 0.405 0.487

AF210646 -0.043 0.54 -0.018 0.754 0.563 -0.3 0.069 0.333 -0.092 0.036 -0.026 -0.144

BI474928 -0.704 -0.286 0.407 0.571 0.562 -0.065 0.371 0.294 -0.225 -0.502 0.161 -0.139

L77146 -0.894 -0.78 0.531 0.89 0.56 0.832 0.434 0.456 0.289 0.684 0.369 0.071

AW116915 0.468 0.606 0.639 0.707 0.557 0.248 0.419 0.443 0.041 0.092 -0.134 -0.255

BM036406 0.86 0.689 0.485 0.64 0.556 1.038 0.526 1.01 0.436 0.146 -0.294 -0.736

BI980448 -0.396 0.227 0.521 0.71 0.554 1.321 0.647 1.247 0.77 1.1 0.713 0.427

AB030897 0.787 0.594 0.344 0.056 0.553 0.926 0.276 0.644 0.045 -0.187 -0.358 -0.378

AW115799 0.94 0.937 0.824 0.933 0.551 1.09 1.025 0.933 0.419 0.363 0.018 0.005

AB032415 -0.434 0.201 0.524 0.563 0.55 0.183 -0.264 -0.184 0.234 0.203 0.128 0.109

AI883929 -0.099 -0.297 0.153 0.398 0.549 0.643 0.133 0.184 0.088 0.001 -0.073 0.079

BI890195 0.522 0.652 0.794 0.979 0.543 0.289 0.103 0.547 0.177 0.092 -0.282 -0.349

AW154483 -0.008 0.592 0.621 0.996 0.542 0.797 0.594 0.295 -0.143 0.018 -0.268 0.262

BG307024 0.822 1.055 1.072 1.249 0.53 0.555 -0.024 0.024 0.024 -0.044 -0.158 -0.304

AW077755 -0.041 -0.315 -0.148 0.497 0.526 1.095 0.565 0.665 0.183 0.324 0.034 0.404

AF286375 -1.659 -2.38 0.139 0.622 0.526 0.043 -0.247 -0.318 -0.421 -0.229 -0.353 -0.213

BI673772 0.224 0.062 0.624 0.665 0.526 0.323 0.399 0.605 0.165 0.634 0.272 -0.05

BM185394 0.23 -0.364 0.004 -0.002 0.525 1.176 0.482 0.857 0.403 0.551 0.666 0.159

AW171012 0.84 0.668 0.155 0.405 0.521 1.242 0.457 0.98 0.344 0.205 -0.083 -0.264

BI979115 -0.03 0.334 0.46 0.33 0.519 0.616 0.227 0.613 0.145 -0.044 -0.233 -0.049

BI840839 0.129 -0.408 0.438 0.397 0.514 1.425 0.553 0.831 0.749 0.567 0.566 0.323

AW344143 0.27 0.507 0.371 0.702 0.512 0.155 0.4 0.572 0.111 0.242 0.142 -0.029

BI892250 -0.427 0.369 0.261 0.554 0.512 -0.113 -0.075 -0.083 0.185 0.044 -0.147 -0.282

AF295373 0.546 -0.135 0.76 0.722 0.509 1.227 0.575 0.477 0.186 0.288 -0.117 -0.515

AW116556 -0.129 0.516 0.423 0.458 0.499 0.636 0.239 0.317 -0.037 -0.086 -0.192 -0.404

BG304168 -0.146 -0.082 0.527 0.36 0.496 0.909 0.403 0.829 0.291 0.401 0.088 0.051

AI641561 -0.145 -0.866 -0.151 0.448 0.492 0.302 -0.19 -0.212 0.35 -0.318 0.316 0.02

AW305456 -0.233 -0.214 -0.336 0.472 0.491 0.742 0.653 0.266 0.646 0.303 0.325 0.104

AW116620 -0.701 -0.673 0.002 0.486 0.49 0.1 -0.162 0.179 0.049 -0.088 -0.088 -0.337

AW170975 0.296 0.239 0.463 0.615 0.485 0.596 0.405 0.28 0.154 0.018 0.087 0.004

BM082431 0.336 0.063 0.503 0.535 0.481 -0.187 0.107 -0.123 0.079 0.096 -0.375 -0.016

BM186588 -0.012 0.043 -0.04 0.494 0.478 0.087 0.179 0.258 -0.09 -0.083 0.092 0.016

BI891158 -1.998 -2.154 -0.18 -0.886 0.478 3.195 1.919 1.072 1.235 -1.221 -0.852 -0.985

AF007414 0.524 -1.877 1.262 0.47 0.477 1.456 0.91 0.807 0.949 0.972 0.879 0.077

AW171308 0.045 -0.321 0.018 -0.013 0.474 0.715 0.408 0.345 0.274 0.258 0.158 -0.086

AI794547 -0.113 0.352 0.066 0.584 0.47 0.178 0.255 0.087 0.022 0.047 -0.093 -0.139

AW154285 0.283 0.755 0.772 0.995 0.463 0.346 0.399 0.391 -0.088 0.259 -0.431 -0.233

BI980786 0.329 0.453 0.882 0.881 0.463 0.468 0.202 0.52 0.189 0.243 0.001 -0.178

AW128316 0.533 0.757 0.634 0.456 0.461 0.841 0.351 0.817 0.291 0.084 -0.097 -0.417

AF246184 -0.459 -0.408 -0.091 0.486 0.46 -0.09 0.235 0.061 0.036 -0.157 -0.106 -0.118

BI429605 -0.416 -0.616 0.092 0.241 0.459 0.947 0.506 0.703 0.344 0.109 0.327 0.006

AW171268 0.31 0.526 1.07 0.747 0.454 1.221 0.524 0.88 0.478 0.744 0.351 0.1

BI708766 -1.55 -0.564 0.724 0.98 0.452 0.329 -0.011 0.701 0.229 0.753 0.055 0.106

AF337035 -0.722 -0.503 0.686 0.739 0.448 1.055 0.587 0.151 0.274 0.449 0.274 0.834

BM104403 0.159 0.683 1.03 1.567 0.446 -0.061 0.128 -0.076 -0.068 -0.601 -0.296 -0.533

AI444338 0.784 0.757 0.782 0.902 0.445 1.413 0.53 1.087 0.92 1.205 0.696 0.236

BI840935 0.209 -0.514 0.441 0.929 0.445 1.432 0.696 0.232 0.261 0.718 0.275 -0.303

AI477247 0.545 0.955 0.938 1.03 0.438 0.031 0.137 0.404 0.128 0.049 -0.452 -0.538

BI983044 0.213 0.09 0.031 0.264 0.432 1.123 0.591 0.591 0.466 0.271 0.013 -0.191

BM005077 0.548 0.077 -0.136 0.011 0.43 0.759 0.226 0.135 0.439 0.344 0.374 0.441

AF075384 -1.18 0.235 0.404 0.464 0.425 0.839 0.444 0.389 0.634 0.617 0.503 0.253

AI964367 0.249 -0.308 0.948 1.216 0.423 0.927 0.368 0.679 0.749 0.902 0.618 0.411

AI883925 0.534 0.306 0.09 0.48 0.422 0.824 0.191 0.835 0.559 0.68 0.343 -0.08

BG985468 -1.635 -1.49 -0.657 -0.15 0.419 1.35 0.973 1.159 0.906 0.68 0.195 -0.218

BE016123 0.806 0.394 0.197 0.285 0.404 0.955 0.567 0.504 0.155 -0.318 -0.791 -0.664

BI865412 0.194 -0.362 0.465 0.368 0.402 1.24 0.365 0.484 0.31 0.609 0.398 0.415

BG303594 -0.103 0.89 0.926 1.015 0.401 0.814 0.463 0.441 -0.046 -0.418 -0.094 -0.303

AW342840 0.739 -0.073 0.092 0.139 0.384 1.054 0.764 0.81 0.294 0.063 -0.137 -0.572

BI877718 0.434 -1.025 -0.108 -0.218 0.383 1.094 0.271 0.619 0.491 0.435 0.581 0.49

BI886248 -0.283 -0.412 0.353 0.181 0.364 0.826 0.359 0.442 0.298 0.611 0.342 0.234

AF210638 -0.371 0.095 0.697 0.318 0.362 0.792 0.313 0.554 0.287 0.691 0.33 0.086

AI584379 0.305 0.545 0.513 1.477 0.34 0.42 0.693 0.49 0.33 -0.158 0.567 0.773

BE557308 0.017 -0.636 0.212 0.433 0.339 1.201 0.781 0.964 0.625 1.055 0.615 0.198

AI793769 -0.569 -0.448 -0.277 0.011 0.334 0.862 0.444 0.736 0.27 0.771 0.218 0.231

AI793490 0.348 0.044 0.244 0.586 0.333 0.752 0.537 0.206 0.156 -0.007 -0.123 -0.17

BE558184 0.257 -0.282 0.299 -0.122 0.33 0.72 0.446 0.675 0.458 0.463 0.055 -0.018

BI885492 -1.278 -0.993 -0.203 0.33 0.324 1.172 0.583 0.626 0.414 0.852 0.67 0.465

BM103957 0.216 0.664 0.663 0.461 0.316 0.853 0.241 0.593 0 0.08 -0.25 -0.096

BI892229 0.4 0.507 0.376 0.239 0.312 0.934 0.358 0.741 0.351 0.312 -0.172 -0.462

BG305296 0 -0.569 -0.178 0.062 0.31 1.359 0.498 1.102 0.566 0.596 0.488 0.068

AW154506 0.911 0.729 0.818 1.033 0.305 -0.072 -0.388 -0.177 -0.137 0.204 0.005 -0.171

AW115793 0.134 0.517 0.827 0.234 0.305 0.887 0.3 0.422 0.213 0.02 -0.22 -0.379

AW018972 -1.257 0.026 -0.563 0.341 0.305 -0.061 -0.407 -0.535 -1.048 -1.696 -1.781 -2.074

BM104348 0.427 0.841 0.692 0.965 0.271 0.324 0.372 0.308 -0.038 0.177 -0.245 -0.537

AI585168 -0.114 0.263 0.484 0.714 0.254 0.052 0.151 0.436 0.164 0.548 0.229 -0.24

BI890235 -0.289 -0.044 0.036 0.616 0.252 0.778 0.265 0.721 0.236 0.623 0.148 0.385

AI943108 0.094 -0.52 0.018 0.035 0.251 0.975 0.464 0.526 0.725 0.696 0.817 0.23

BI891591 0.216 0.04 0.051 0.33 0.239 1.002 0.41 0.968 0.413 0.35 0.725 0.198

AI793485 0.155 -0.238 -0.144 -0.184 0.233 0.506 0.2 0.494 -0.076 0.199 0.01 0.096

BI887522 0.688 -0.261 0.566 -1.41 0.226 1.058 0.419 0.797 -0.78 -0.078 -0.32 -0.85

BI887468 0.077 -0.517 0.377 0.148 0.222 0.756 0.239 0.708 0.655 0.582 0.138 0.187

AI584395 0.621 0.697 0.736 0.986 0.218 0.173 -0.126 0.014 -0.01 0.19 0.158 0.417

BI980758 0.345 -0.078 -0.066 0.271 0.216 0.69 0.392 0.048 0.427 -0.018 0.351 0.606

BE017542 0.198 0.658 1.045 1.212 0.215 0.092 0.608 0.938 0.095 0.397 -0.065 -0.581

BM036771 1.151 1.204 1.008 1.409 0.206 -0.229 -0.516 -0.709 -0.598 -0.519 -0.562 -0.728

AA658756 0.667 -0.161 0.094 -0.023 0.183 0.755 0.449 0.226 0.372 0.317 0.132 0.105

X68324 -0.717 0.343 0.063 0.962 0.149 0.38 -0.128 0.269 0.559 0.412 0.658 0.48

AI584569 0.67 0.386 0.318 0.372 0.148 0.933 0.234 0.756 0.465 0.609 0.349 -0.106

BI888169 -0.002 -1.276 -0.706 -0.244 0.144 1.06 0.839 0.81 0.32 0.892 0.418 0.145

AW116409 -0.907 -0.61 -0.04 -0.153 0.134 0.655 0.251 0.482 0.36 0.512 0.101 -0.014

AW115809 -0.118 -0.698 -0.013 -0.078 0.132 0.904 0.536 0.751 0.46 0.608 0.349 0.205

BI840953 -0.082 -0.409 0.192 0.251 0.13 0.851 0.409 0.148 0.179 0.174 0.301 0.65

BI891355 -0.346 -0.222 -0.061 0.035 0.123 0.947 0.515 0.775 0.616 0.633 0.151 0.421

BI886935 0.432 -0.64 -0.077 0.103 0.119 0.882 0.33 0.565 0.288 0.341 0.085 -0.052

BI879523 0.861 0.475 0.677 0.997 0.076 -0.157 0.222 0.134 0.091 0.461 -0.132 -0.18

BG303575 0.919 -0.894 -0.521 -0.212 0.017 1.337 0.968 0.801 0.187 0.225 -0.309 -0.509

BI878819 0.824 0.696 0.724 0.957 0.015 0.426 0.027 0.157 -0.369 -0.176 -0.423 -0.262

BI888008 0.323 0.553 0.041 0.039 -0.011 0.901 0.124 0.589 0.138 0.512 0.238 0.684

BI890607 -0.68 -1.293 -0.427 -0.061 -0.044 0.549 0.192 0.444 0.158 0.333 0.051 -0.329

AI641534 -1.09 -1.603 -0.709 -0.182 -0.053 0.813 0.509 0.667 0.37 0.645 0.348 0.135

BI896231 -1.151 -1.418 -0.402 -0.082 -0.078 1.014 0.593 0.812 0.682 0.824 0.501 0.029

AW116327 0.577 -0.348 -0.368 -0.407 -0.079 0.832 0.432 0.605 0.189 0.403 0.328 -0.458

BI867171 -0.005 -0.894 -0.899 -0.407 -0.091 0.917 0.689 0.719 0.394 0.643 0.307 0.051

AW777906 0.28 -0.091 -0.353 -0.119 -0.147 0.704 -0.072 0.399 0.319 0.425 0.375 0.065

AW171471 -0.482 -1.335 -0.506 -0.657 -0.258 0.694 0.218 0.416 -0.065 0.03 -0.32 -0.548

AI721419 -0.464 -1.437 -1.735 -1.2 -0.273 1.44 0.916 1.195 0.501 0.619 -0.011 -0.364

AI943216 0.146 -2.595 -1.038 -1.915 -0.375 1.056 -0.103 0.646 -0.881 0.596 -1.298 -0.064

Mean -0.105 0.13 0.854 1.118 1.302 0.936 0.675 0.943 0.404 0.279 0.14 -0.023
