# Supplementary material for: Transcriptome Analysis of Zebrafish Embryogenesis Using Microarrays
Source: PLoS Genet. 2005 Aug 26;1(2):e29. doi: 10.1371/journal.pgen.0010029 (PMC1193535; doi:10.1371/journal.pgen.0010029)
Supplement: Dataset S16 — (142 KB DOC) [file pgen.0010029.sd016.doc]

Dataset S16. Genes exhibiting peak of expression at the segmentation stage.

Genbank IDUF egg 3hpf 4.5hpf 6hpf 7.7hpf 9hpf 10.7hpf 12hpf 15hpf 24hpf 30hpf 48hpf

BI887718 -0.791 -0.525 2.215 3.674 4.74 4.448 5.293 4.54 3.835 0.612 0.468 -0.196

AI544649 -0.454 0.248 0.878 1.482 3.674 4.326 4.566 3.167 3.601 1.261 0.632 -0.616

AI353412 -1.573 -0.006 3.87 3.542 3.997 4.521 4.403 4.471 3.266 1.757 1.5 0.192

AI477935 -0.542 -0.004 1.268 2.229 3.722 4.366 4.045 4.581 3.747 1.287 -0.149 -0.687

BI890439 -0.514 0.081 1.452 2.519 3.822 3.403 2.873 3.828 1.495 0.191 0.148 -0.304

BI889922 -2.288 -1.316 1.667 2.018 2.774 2.812 2.82 2.879 2.829 -0.87 -1.154 -1.457

BI887368 -0.627 0.484 4.191 4.239 4.366 2.479 2.782 4.767 3.248 0.972 1.059 -0.289

BI704249 -1.19 -2.038 1.18 2.333 3.317 3.151 2.768 3.828 2.173 1.371 1.122 0.344

BI891948 -0.474 -0.088 3.268 3.516 3.885 2.822 2.563 4.233 2.387 1.651 0.537 -0.247

AI545065 -0.663 -0.527 2.102 2.511 2.801 2.428 2.265 2.853 1.872 0.184 0.039 -0.54

BI889166 -0.93 -0.964 1.667 2.222 2.232 2.397 2.12 2.434 1.542 1.662 1.003 0.224

BI891709 -0.956 -0.348 0.556 1.394 1.888 2.097 2.11 2.502 1.283 1.252 0.511 0.421

BI889398 0.024 0.807 1.253 1.286 2.089 1.138 2.005 3.676 1.857 0.508 -0.139 -0.246

BM184694 0.908 0.46 0.925 1.089 1.531 1.81 1.978 2.494 1.003 1.383 0.796 0.409

AW281931 0.195 1.091 1.751 0.99 1.3 1.683 1.808 2.73 1.257 0.765 0.301 -0.103

BI886160 0.253 -0.349 0.761 1.303 1.205 2.184 1.784 3.178 1.374 0.71 -0.338 -0.093

AI477949 0.08 1.131 1.806 1.987 2.348 1.944 1.782 2.498 0.84 0.783 0.082 -0.156

AJ309314 -0.588 0.005 0.308 0.974 2.329 2.346 1.78 2.696 1.5 0.096 0.168 -0.141

BI673573 -0.009 -0.327 0.074 0.113 0.82 1.942 1.719 1.875 1.404 1.17 0.307 0.115

AI965251 -0.702 -0.455 -0.115 0.356 1.284 1.235 1.701 2.703 0.916 0.719 0.669 -0.582

AI396677 0.165 -0.381 0.503 0.469 1.603 1.706 1.653 2.001 0.752 0.435 0.279 0.081

X66958 -0.423 0.138 0.256 2.309 3.55 1.587 1.615 4.469 1.539 0.262 0.251 0.201

BI892172 0.962 0.185 0.573 0.837 1.536 1.242 1.606 2.258 0.978 0.816 0.33 -0.142

BI891320 0.594 0.645 1.328 1.705 1.746 0.994 1.596 1.706 0.751 1.04 0.457 0.173

BI892263 -0.754 -0.429 1.008 1.416 1.222 1.511 1.547 1.361 1.575 1.399 0.875 0.259

AW232296 0.447 1.06 1.453 1.484 1.437 1.446 1.522 1.477 0.718 1.064 0.46 -0.095

AF423762 -0.142 -0.795 0.677 0.921 1.334 1.573 1.501 1.662 1.391 1.102 0.797 0.828

AI877955 -0.401 -0.152 1.463 1.831 1.44 1.581 1.491 1.916 0.626 0.595 0.17 0.067

AW077184 -0.201 -0.029 1.361 1.523 1.613 1.728 1.488 1.846 0.959 0.593 0.192 0.22

BI892384 0.18 0.329 1.129 1.039 1.26 1.013 1.474 1.665 1.04 0.749 0.208 0.06

BI850028 -1.04 -1.585 0.542 1.324 2.291 2.079 1.427 2.39 1.744 0.82 0.474 0.098

AW117050 0.237 -0.371 0.559 0.731 1.188 1.392 1.424 1.456 0.683 0.767 0.072 -0.531

AW128428 -1.238 -2.825 0.046 0.605 1.181 1.452 1.416 1.442 0.173 -1.017 -1.803 -1.647

BI888166 1.014 -0.557 0.099 0.848 1.435 1.862 1.414 1.931 0.426 0.984 0.608 0.344

BE201182 0.008 0.333 1.443 1.519 0.831 1.214 1.411 1.714 0.522 0.295 0.474 0.436

BG306295 0.177 0.583 1.178 1.241 1.784 1.326 1.411 1.961 0.76 0.748 0.282 0.231

AW421022 -1.389 -1.786 -1.083 -0.061 0.489 1.613 1.407 1.551 1.189 0.905 0.751 0.069

BI891860 0.245 -0.126 1.259 1.444 1.464 0.812 1.385 1.597 1.146 0.707 0.751 0.587

AW058839 -0.698 -0.825 0.386 0.664 1.145 1.679 1.379 2.02 1.258 0.405 0.173 -0.151

BI427758 0.092 -0.435 1.28 1.538 1.374 1.614 1.376 1.878 0.726 0.639 -0.108 0.028

AW280006 0.09 0.319 0.487 0.602 1.234 1.633 1.37 1.66 0.867 1.026 0.626 0.284

AI878677 -0.852 -1.265 0.82 1.121 1.023 1.064 1.344 2.301 1.447 1.729 0.933 -0.264

BG306779 -0.147 -0.472 0.314 0.221 0.985 1.349 1.342 1.555 0.949 0.759 0.727 0.716

BM182327 0.91 0.888 1.003 1.504 1.698 1.609 1.339 1.971 1.017 0.705 0.128 0.256

BI846833 -2.254 -2.063 -0.124 0.504 0.823 1.352 1.334 0.954 1.512 1.114 1.139 0.442

BI891278 -0.916 -2.831 -0.923 -0.106 0.79 1.201 1.326 1.401 0.528 0.875 0.707 0.733

BE201733 0.476 0.19 0.174 0.612 1.345 2.044 1.315 2.221 1.258 1.26 0.594 -0.144

AI497234 -0.419 -0.043 0.332 0.753 1.343 1.136 1.301 2.252 0.944 0.02 -0.356 0.086

AW019691 0.071 -0.249 1.142 1.576 1.157 1.004 1.297 1.874 0.555 0.291 0.019 0.123

BI891704 -0.049 -0.406 -0.1 0.906 1.283 1.188 1.284 1.609 1.022 1.259 0.302 0.145

BE201769 -0.324 -1.017 -0.783 0.033 1.123 1.183 1.279 1.592 0.877 0.607 0.656 0.942

AI884227 0.196 0.985 1.325 1.286 1.446 1.292 1.278 1.883 1.39 0.926 -0.087 -0.546

BM036802 -0.049 0.341 1.187 1.578 1.378 1.27 1.273 1.853 0.782 0.559 -0.021 0.222

AF030560 -0.237 0.088 1.435 1.235 1.537 0.666 1.262 2.145 0.688 0.231 0.204 -0.04

AI721479 -0.112 -0.631 -0.518 0.092 1.091 0.905 1.256 1.958 1.054 1.017 1.155 0.074

X97333 -1.144 -0.011 0.164 -0.138 0.672 1.826 1.256 2.191 1.625 1.272 1.44 0.741

AI957519 -0.695 -0.203 1.35 1.334 1.455 1.772 1.255 1.657 1.828 1.182 0.752 0.673

AW777535 -0.408 -0.081 0.782 0.864 1.022 1.426 1.239 1.671 1.012 1.29 0.861 0.564

BM095389 1.332 0.206 0.067 0.586 0.618 1.414 1.229 1.707 0.79 0.644 0.609 0.184

BG303611 -1.053 0.619 0.949 -2.695 -3.259 1.368 1.218 -0.13 1.09 0.652 -1.626 -1.486

BI889140 0.176 0.384 0.309 1.308 1.748 1.262 1.216 1.84 0.403 0.463 0.435 0.317

BI886200 0.606 0.698 1.303 1.248 1.493 1.745 1.215 1.865 0.96 0.653 0.367 -0.164

AI477286 -0.158 -0.096 0.154 0.555 0.687 1.258 1.211 1.411 1.169 1.064 0.472 0.312

BI888791 -0.38 0.148 0.243 1.236 1.632 0.735 1.209 1.865 0.663 -0.543 -1.019 -0.862

S80425 -0.432 -0.018 1.244 1.182 1.073 0.885 1.207 1.964 0.254 0.059 0.42 0.804

BI887812 -0.491 0.115 1.565 1.179 1.569 1.326 1.203 1.885 1.133 0.684 0.174 0.361

BI840456 0.264 0.572 1.064 0.536 1.259 1.61 1.193 1.848 0.682 0.792 0.294 0.161

AW344187 0.185 -0.179 -0.122 0.272 1.148 1.294 1.173 1.682 0.792 1.046 0.255 0.113

AW777460 -0.406 -0.023 0.729 1.145 1.211 1.027 1.171 1.208 0.383 0.053 -0.333 -0.038

BG728568 -0.065 0.141 0.596 0.895 1.13 0.366 1.155 1.498 0.35 -0.132 -0.068 -0.095

AI964231 -0.279 -0.069 0.737 0.893 1.368 1.505 1.154 1.719 0.749 0.598 0.188 -0.337

BI850032 0.378 -0.529 -0.862 -0.542 0.142 0.891 1.139 1.014 0.839 0.64 0.142 0.3

AW154574 0.949 -0.009 -0.064 0.174 0.312 1.366 1.139 1.302 0.353 -0.188 -0.874 -0.985

BI890196 -0.791 0.19 -0.295 0.365 0.663 1.497 1.113 1.521 1.318 0.584 0.33 0.128

BM070584 0.472 0.084 0.808 1.137 1.148 0.909 1.101 1.174 0.728 0.618 0.28 0.301

BI891455 0.758 0.232 0.409 0.789 0.978 0.951 1.101 1.28 0.727 1.173 0.598 0.392

U18312 -0.744 0.451 1.602 1.94 1.952 1.665 1.098 2.151 0.744 -0.18 0.224 0.742

BM080950 0.821 -0.045 0.31 0.987 1.3 1.168 1.092 1.536 0.418 0.954 0.699 0.347

BI891674 0.229 -0.449 -0.141 0.33 1.577 1.452 1.086 2.633 1.606 0.478 0.247 0.044

BI887620 0.319 -0.033 0.124 0.502 0.854 0.872 1.086 1.372 0.723 0.845 0.638 0.722

BM095658 0.051 0.468 0.681 1.006 1.262 0.988 1.074 1.444 0.576 1.005 0.601 -0.289

AW420476 -0.207 0.017 1.64 1.499 1.477 1.001 1.065 1.827 0.769 0.771 0.652 0.532

AW420829 -0.25 -0.035 -0.435 -0.482 1.248 0.651 1.046 0.975 0.34 0.058 0.331 0.207

AW019690 0.431 -0.371 1.104 1.592 1.316 1.465 1.042 1.728 0.764 0.509 -0.001 0.073

BI891116 0.296 0.932 1.414 1.07 1.516 1.417 1.039 1.569 0.944 0.432 0.218 -0.052

AI793555 0.5 0.112 -0.263 -0.146 0.56 1.143 1.034 2.216 1.185 0.7 0.21 0.005

BI891596 -0.679 0.306 0.506 1.402 1.64 0.831 1.028 2.391 0.936 0.054 -0.149 -0.387

BI672337 -1.251 -1.507 -1.458 -0.47 0.104 1.041 1.026 1.491 0.245 0.77 -0.285 -0.201

X67648 -1.484 -0.865 -0.497 0.518 1.062 0.712 1.023 1.535 0.88 0.98 0.555 0.628

BI887172 0.36 0.238 0.86 0.975 1.743 0.948 1.018 1.916 0.273 0.395 0.093 0.011

BI879231 1.259 0.694 -0.082 0.337 0.88 1.045 1.014 1.425 0.535 0.027 -0.03 -0.558

AI942866 -0.505 -1.596 -1.538 -1.226 0.022 0.716 1.01 0.835 1.822 0.661 0.255 -0.874

AI477305 -0.535 -2.694 -0.681 -0.215 0.28 0.859 1.005 1.103 0.507 0.807 0.362 0.08

AF160635 -0.537 -0.251 0.316 0.554 0.621 0.788 1.004 0.862 0.694 0.609 0.365 0.266

BE606048 -0.153 -0.514 0.6 0.466 0.701 0.515 0.998 0.58 0.779 0.838 0.155 0.385

BI839727 0.324 -0.324 0.422 0.321 0.342 0.881 0.988 0.524 0.239 0.253 -0.064 -0.138

BI867717 0.822 0.522 0.389 0.738 1.247 1.36 0.987 1.463 0.307 0.276 0.334 0.046

AI641775 0.52 -0.523 -0.217 0.085 0.244 0.851 0.986 0.91 0.598 0.813 0.477 0.325

AW282142 0.317 -0.822 1.506 1.124 2.397 1.256 0.986 2.554 0.568 0.586 -0.098 -0.414

U40995 -0.741 -0.14 -0.448 -0.49 1.136 1.203 0.984 2.288 0.767 0.683 0.995 -0.093

AF030281 -0.668 -0.228 -0.204 -0.567 -0.091 1.184 0.982 2.012 0.803 0.797 0.888 0.781

BI878322 -0.023 0.069 0.994 1.21 1.378 0.954 0.976 1.657 0.455 0.788 0.478 0.377

AI545142 -0.853 0.334 -0.436 0.341 1.054 0.905 0.97 2.212 0.996 1.545 0.791 -0.38

AW019528 0.035 -0.242 -0.086 -0.026 0.45 0.944 0.965 0.978 0.559 0.383 0.23 0.085

AI545424 -0.092 -0.308 0.425 0.758 1.135 0.753 0.961 1.091 0.771 0.771 1.032 -0.053

AJ242515 -0.633 -0.261 1.215 1.369 1.127 0.905 0.96 1.837 0.972 0.157 0.575 0.199

AW154642 -0.033 0.08 0.401 0.609 1.588 0.957 0.955 1.947 0.855 0.468 0.174 -0.414

BI841627 0.641 0.732 1.335 1.354 1.279 1.143 0.951 1.659 0.752 0.76 0.279 -0.092

BI704293 -0.728 -0.233 0.216 0.152 0.847 1.076 0.945 1.281 0.536 0.69 0.641 0.112

AW116127 0.687 0.337 0.601 1.006 1.419 0.685 0.944 1.657 0.472 0.37 0.274 0.099

BI892167 -0.592 0.33 1.084 2 1.776 1.112 0.94 3.149 2.268 1.455 0.915 -0.497

BI889074 0.307 0.591 0.797 0.406 0.934 0.802 0.935 1.107 1.11 0.749 0.181 -0.666

AW019321 -0.917 -0.62 -0.537 -0.049 0.294 0.836 0.931 1.233 0.546 -0.448 -0.683 -0.729

BI885240 -0.594 -0.621 -0.103 0.693 0.916 0.956 0.923 1.376 0.338 0.622 0.247 -0.002

AI793745 0.352 -0.605 -0.104 0.502 1.037 0.949 0.922 1.215 0.649 0.322 0.05 -0.325

BI704236 0.017 0.672 1.448 1.486 1.461 1.704 0.918 2.217 0.828 0.741 0.236 -0.293

BI887401 0.286 0.613 0.693 0.672 1.098 0.47 0.913 1.565 0.245 0.458 0.157 0.179

BM026308 -0.463 0.508 0.31 1.229 1.628 1.304 0.913 1.87 0.154 0.33 0.135 -0.153

BI709791 0.401 0.147 0.542 0.974 0.961 0.605 0.91 1.311 0.118 0.368 0.124 -0.011

AI584307 -0.236 -0.373 -0.223 -0.095 0.772 0.641 0.91 0.734 0.419 0.776 0.734 0.566

BI705930 -0.413 0.053 0.891 1.043 1.799 0.857 0.903 2.107 0.961 0.614 0.353 -0.268

BI891665 0.384 0.727 0.759 1.22 1.061 0.744 0.895 1.434 0.198 0.234 -0.023 -0.017

BI891768 -0.24 0.184 1.556 1.377 1.567 0.938 0.89 1.853 0.48 0.635 0.29 -0.294

BM102635 0.275 -0.176 0.534 0.587 0.553 0.442 0.88 0.576 0.635 0.552 0.47 0.118

BI889946 0.555 0.334 0.59 0.121 0.48 1.18 0.877 1.201 0.699 0.919 0.444 0.017

BI886549 -0.524 -0.475 -0.366 0.237 0.645 0.918 0.877 1.172 0.287 0.782 0.176 0.13

AF042191 -0.184 -0.353 0.534 1.385 1.698 0.571 0.871 2.281 0.729 -0.179 -0.105 -0.226

AW059073 0.966 0.634 0.312 0.25 0.454 1.015 0.87 1.402 0.45 0.118 -0.152 -0.638

AW231998 0.369 0.955 1.376 1.182 1.357 1.014 0.866 1.464 0.295 0.183 -0.341 -0.268

BM182319 -0.338 0.264 -0.049 0.629 1.057 0.616 0.858 1.489 0.309 -0.358 -0.722 -1.107

BI890034 -0.645 -0.75 1.688 1.398 1.372 0.551 0.855 1.619 1.225 0.972 0.716 -0.164

AI629101 0.284 -0.129 0.553 1.176 1.561 0.353 0.855 1.596 0.29 0.095 0.299 -0.119

BG306038 0.708 -0.509 -0.081 0.377 0.645 1.127 0.853 1.424 0.731 0.974 0.33 0.143

U62134 -0.479 -0.42 0.806 0.673 0.789 0.725 0.85 1.002 0.598 0.461 0.05 0.009

BM156012 0.639 0.484 0.461 0.389 0.779 1.541 0.849 1.777 0.917 0.615 0.465 -0.028

AI601765 -0.175 -0.603 -0.322 0.188 0.907 0.45 0.841 1.248 -0.378 0.586 -0.543 -0.525

AW171554 0.818 0.41 0.443 0.859 0.981 0.872 0.841 1.563 0.585 0.477 -0.311 -0.518

AI723219 -1.339 -1.305 0.957 0.726 0.466 0.607 0.838 0.602 0.502 0.778 0.449 0.174

AW115770 0.128 0.243 0.713 0.754 0.716 0.547 0.835 1.097 0.439 0.937 0.302 -0.027

AW232317 0.78 0.297 -0.24 0.194 0.682 0.687 0.832 1.388 0.678 0.631 0.29 0.14

BI882727 1.069 -0.892 -0.251 0.019 0.249 1.391 0.829 1.396 1.114 1.204 0.613 0.004

AW116147 0.117 -0.114 0.345 0.843 0.92 0.395 0.829 1.245 0.365 0.85 0.252 -0.072

AW116281 0.3 0.737 0.558 0.762 0.916 1.116 0.826 1.739 0.978 0.305 0.012 -0.902

BI892323 -0.688 0.023 -0.257 0.037 0.455 0.16 0.82 1.502 0.553 0.138 -0.229 -0.086

BG306139 -0.728 -0.321 -0.53 0.092 0.905 0.195 0.82 1.87 0.962 0.423 0.449 0.021

BI888606 0.139 0.639 1.428 0.292 -0.006 0.468 0.819 2.067 0.641 0.975 1.109 0.157

AI965321 0.176 0.248 -0.321 -0.089 0.751 0.921 0.814 1.139 0.081 0.532 0.399 -0.422

AI584354 0.156 -0.621 0.073 0.146 0.107 0.304 0.809 1.042 0.467 0.575 0.557 -0.04

BM103927 0.584 0.02 0.592 0.322 0.489 1.07 0.808 1.485 0.46 0.296 -0.061 -0.129

AI958191 -0.528 -1.156 -0.045 0.112 0.461 1.236 0.808 1.392 1.153 0.898 0.231 -0.143

AI957527 0.53 0.673 0.477 0.273 0.556 0.64 0.805 0.794 0.2 0.059 0.153 0.107

BI877938 0.425 0.528 0.486 0.398 0.229 0.773 0.803 0.843 0.427 0.407 0.028 0.035

BI885924 0.742 -0.077 0.319 0.41 0.544 1.176 0.799 1.157 0.458 0.409 0.171 -0.184

BI892082 0.211 0.072 0.36 0.99 0.697 0.558 0.798 1.212 0.3 0.407 -0.164 -0.109

AI723286 0.779 0.534 0.436 0.42 0.723 0.844 0.796 0.943 0.478 0.8 0.099 -0.285

AW165362 0.304 -1.184 0.459 1.312 1.702 0.84 0.794 1.936 0.357 0.654 -0.258 -0.57

BI889917 0.131 0.098 -0.6 -0.28 1.228 1.231 0.794 1.542 -0.07 0.32 0.222 -0.158

AW777539 0.462 0.017 0.315 0.164 0.389 0.693 0.783 1.47 0.955 1.126 1.004 0.234

BM185237 -0.285 -0.45 0.693 0.618 0.411 0.812 0.782 0.796 0.323 0.165 -0.137 -0.248

BI706908 -0.16 -1.223 0.45 1.101 0.917 0.536 0.776 1.256 0.046 0.171 0.276 -0.005

BM183903 0.596 -1.367 -0.441 0.295 0.508 0.692 0.773 1.294 0.622 0.942 0.541 0.124

BG884096 -0.008 -0.169 0.677 0.468 0.454 0.852 0.77 0.894 0.532 0.602 0.348 0.094

BI705721 0.513 0.615 -0.446 0.172 0.805 0.625 0.769 0.905 0.443 0.188 -0.196 -0.405

BI839826 -0.27 -0.1 0.991 -0.667 0.257 0.081 0.769 1.683 0.311 -0.042 0.515 0.173

BI866976 0.714 -0.115 0.081 0.285 0.572 0.336 0.768 1.078 0.118 0.656 0.839 0.791

BI878979 0.9 0.04 0.851 0.496 0.951 0.962 0.767 1.062 0.34 0.417 -0.4 -0.658

AI601541 0.11 0.089 0.064 0.372 0.69 0.569 0.766 1.31 0.282 0.331 0.032 -0.286

BE606074 0.053 0.259 0.728 0.368 0.538 0.454 0.765 1.043 0.531 0.125 -0.146 -0.346

BI890050 -0.134 -0.668 -0.111 0.347 0.955 0.453 0.761 1.217 0.542 0.512 0.45 0.276

AW343567 -0.282 0.234 0.786 0.616 1.126 0.024 0.758 1.342 0.09 -0.096 0.237 -0.222

BI891689 0.903 0.318 0.182 0.491 0.362 1.008 0.757 1.416 0.441 0.385 0.053 -0.408

AI584394 0.083 0.4 1.163 0.993 1.231 0.412 0.756 1.165 1.409 0.743 0.703 -0.931

BI889280 0.42 -0.441 0.591 1.001 1.383 0.305 0.755 1.604 0.627 0.528 0.329 -0.446

BI890250 0.058 1.066 1.703 1.222 1.595 0.58 0.751 1.809 1.011 0.619 0.259 0.273

AF371368 -0.696 -0.669 0.619 0.478 0.593 0.421 0.748 1.447 0.733 0.497 0.91 0.167

BI886329 -0.325 0.242 1.073 1.125 1.314 1.193 0.745 1.851 0.857 1.47 0.753 0.31

AW115729 -0.093 0.141 0.011 0.38 0.662 0.779 0.735 1.331 0.41 0.561 0.726 0.167

BI892444 0.454 0.01 0.712 0.792 1.718 0.442 0.731 1.949 0.543 0.718 -0.148 -0.313

AI943227 -0.093 -0.64 -0.638 -0.264 0.303 0.759 0.731 1.59 1.07 0.737 0.384 -0.072

BI886251 -0.942 0.35 1.821 1.613 1.83 1.545 0.726 2.09 1.646 0.922 0.061 -0.45

BG306318 -0.519 -0.806 0.173 -0.688 0.186 0.344 0.725 1.374 0.494 0.71 0.621 -0.02

BI840845 -0.559 -0.773 0.458 0.821 0.14 0.708 0.722 0.705 0.223 0.158 -0.147 -0.093

AI964306 0.949 0.638 0.411 0.347 0.223 0.166 0.72 1.223 0.563 0.415 -0.091 -0.231

AF252546 -0.053 -0.333 -0.309 -0.183 0.212 0.56 0.714 1.074 0.621 0.233 0.198 -0.191

AY007990 -0.608 -0.548 0.527 1.177 1.086 0.759 0.708 1.865 0.567 1.075 1.531 0.413

BE556846 0.061 -1.876 0.188 0.374 0.121 0.574 0.708 1.133 0.569 0.447 0.391 -0.112

AW232289 0.433 -0.016 0.114 0.429 0.775 0.566 0.705 0.886 0.349 0.242 0.091 -0.012

AW826449 -0.167 -0.481 0.095 0.485 0.748 0.221 0.705 1.194 0.337 0.515 0.751 -0.065

BI427744 0.083 -0.766 -0.593 -0.754 -0.166 0.09 0.7 1.512 0.578 1.069 1.138 0.023

S76877 -0.351 -0.322 0.419 0.56 0.98 0.838 0.691 1.088 0.602 0.22 0.535 0.04

AB032726 0.896 0.238 0.501 0.47 0.7 1.306 0.689 1.327 0.915 0.619 0.162 -0.406

AW777903 0.152 0.285 0.528 0.388 0.451 0.658 0.689 1.402 0.47 0.273 0.748 0.449

AW154707 0.88 -0.493 -0.297 0.474 0.264 0.764 0.688 1.051 0.408 0.708 0.055 0.091

AW154792 0.565 -0.707 -0.073 0.311 0.759 0.616 0.688 1.214 0.469 0.314 0.007 -0.307

AB055662 -1.395 -1.305 -0.421 0.387 0.599 1.262 0.688 1.629 0.793 1.006 0.85 -0.02

BM186050 0.493 0.712 0.711 0.554 0.836 0.758 0.688 0.863 0.456 0.41 -0.112 -0.533

AY029808 -0.242 -0.338 0.414 1.08 1.201 0.212 0.686 1.673 0.498 0.414 0.818 0.4

BI888928 0.855 -0.448 -0.357 0.299 0.5 0.69 0.685 1.439 0.458 0.467 0.585 0.283

BI704244 0.209 -0.035 0.77 0.402 0.5 1.056 0.685 1.569 1.168 0.698 0.738 -0.351

BI704393 0.291 -0.534 -0.097 0.648 0.603 0.552 0.684 1.307 0.47 0.112 -0.117 -0.67

BM071353 0.631 0.304 -0.085 -0.112 0.182 0.73 0.684 0.874 0.422 0.583 0.286 0.026

BI670852 0.539 -0.255 0.195 0.538 0.909 0.766 0.682 1.346 0.534 1.099 0.76 0.529

BI473383 -0.328 -0.371 0.195 0.444 0.471 0.353 0.681 0.189 0.213 0.266 -0.29 -0.145

AI444340 0.335 0.841 -0.512 -0.384 0.819 1.035 0.68 1.139 0.347 -0.348 -0.06 -0.146

AI957575 -0.666 -2.018 -1.048 -1.215 -0.306 0.448 0.679 1.135 0.512 0.698 0.369 -0.538

AI330980 0.04 -0.956 0.079 0.091 0.229 0.475 0.678 1.175 0.362 0.939 0.873 0.564

AI641018 0.337 0.065 -0.137 0.267 0.476 0.91 0.678 1.033 0.409 0.528 0.424 0.129

BM102865 0.15 -0.587 0.36 0.586 1.163 0.423 0.676 1.277 0.374 0.305 -0.055 0.017

BI473004 1.165 -0.66 -0.799 -0.479 -0.048 0.898 0.675 1.362 0.411 0.856 0.725 -0.004

BI896347 -0.793 -0.996 -0.574 -0.292 -0.132 1.003 0.675 1.373 1.15 0.863 0.847 0.248

AI497414 0.699 0.869 0.698 0.746 1.026 1.083 0.675 1.145 0.543 0.079 0.345 -0.185

AW115824 -0.079 0.695 0.123 0.202 0.553 0.388 0.674 1.318 0.504 0.293 0.695 0.429

AI957786 0.09 0.306 0.756 0.555 0.519 0.159 0.672 1.005 0.211 0.858 0.381 0.241

BI878967 0.797 0.543 0.289 0.531 0.754 0.724 0.668 1.211 0.129 0.124 0.402 0.027

AW019720 -0.091 -0.326 -0.009 0.19 0.806 0.838 0.667 1.236 0.766 0.27 0.156 0.006

BI887817 0.118 -0.177 0.402 0.546 1.46 0.666 0.661 1.592 0.38 0.214 -0.029 -0.165

AB055666 -1.046 0.105 -0.64 0.31 -0.316 0.81 0.659 1.662 0.732 0.991 0.796 1.208

BM083952 0.007 0.279 0.091 0.712 0.771 0.315 0.659 0.954 0.399 0.31 0.298 -0.062

AW154375 -2.034 -1.766 -0.663 -0.291 0.245 1.085 0.658 1.755 0.705 0.023 -0.35 -0.686

BI888458 0.149 0.751 0.733 0.911 0.834 0.156 0.657 0.946 -0.241 -0.076 -0.076 -0.346

AW466584 0.658 -0.589 -0.562 -0.169 -0.126 0.56 0.654 0.906 0.203 0.193 -0.023 -0.104

AW826550 -0.447 -0.205 -0.089 -0.308 0.31 0.615 0.654 1.052 0.672 0.244 0.636 0.817

AI964258 -0.804 -1.302 -0.598 -0.063 0.155 0.718 0.651 0.896 0.61 0.683 0.138 -0.006

AI877538 0.349 0.379 0.269 1.25 1.241 -0.143 0.647 1.385 0.304 0.303 0.281 -0.362

AI878617 -0.017 0.094 -0.005 0.749 0.763 0.925 0.644 1.087 0.151 0.502 0.051 0.004

AI883716 0.416 0.213 0.588 0.6 0.986 0.698 0.643 1.108 0.218 0.344 0.192 0.114

BG737957 -0.869 0.267 -0.12 -0.52 0.285 0.691 0.642 1.6 0.856 0.988 0.801 1.199

X60095 -0.572 -0.111 0.277 -0.178 -0.074 0.016 0.639 1.558 0.587 0.221 0.639 0.196

AF254955 -0.186 0.453 0.48 0.347 1.183 -0.05 0.637 1.061 0.239 -0.682 -0.514 -0.608

BI476025 0.38 0.717 0.928 0.912 1.282 0.61 0.637 1.305 0.391 0.263 0.068 0.364

AW076647 -0.447 0.496 0.695 0.942 1.116 -0.095 0.636 1.256 -0.05 -0.183 0.158 -0.28

BI885968 0.785 -0.222 -0.224 -0.131 -0.018 0.596 0.634 0.926 0.656 0.791 0.447 -0.173

BI891290 0.356 -0.442 0.277 0.339 0.95 0.191 0.632 1.737 0.417 0.578 0.559 0.384

BM182325 0.303 0.52 -0.04 0.252 0.254 0.508 0.629 0.743 0.374 0.311 0.003 -0.015

AI943154 0.451 -0.596 0.032 0.268 0.563 0.622 0.625 1.268 0.635 0.812 0.564 0.295

BI472540 0.047 -0.037 0.244 0.57 0.483 0.067 0.624 1.821 0.393 0.061 0.056 -0.179

AF222995 -0.711 -0.268 0.179 -0.978 -0.193 0.351 0.619 1.84 0.337 0.551 0.739 0.861

BI672201 -0.34 -0.173 0.293 0.353 0.562 1.222 0.619 1.451 0.698 0.91 0.307 0.035

BI879932 -0.023 0.003 0.152 -0.029 0.11 -0.317 0.619 2.253 0.598 0.552 0.177 -0.129

BG985671 0.174 0.674 1.022 0.646 0.603 1.245 0.619 1.324 0.561 0.888 0.371 -0.011

AI666922 -0.128 -0.953 -0.889 -0.499 -0.16 0.379 0.619 1.418 0.867 0.702 0.608 0.758

U31079 -0.428 -0.692 -0.58 -0.093 0.363 0.471 0.617 1.884 0.669 0.94 0.956 0.639

U16311 -0.739 0.472 0.042 0.341 1.947 0.759 0.612 2.266 0.562 0.657 0.574 0.197

AI626587 0.697 0.594 0.294 0.758 0.27 0.517 0.607 1 0.308 0.552 0.458 0.021

AW117146 -0.692 -0.108 -0.788 0.008 0.531 0.898 0.607 1.055 0.269 0.712 0.194 -0.069

BG305445 -0.541 -0.382 -0.352 0.079 0.808 1.121 0.606 1.303 0.84 0.728 0.477 0.562

AW059102 -0.023 -0.968 0.309 -0.216 1.174 0.282 0.602 1.757 0.383 0.283 0.178 -0.06

BI709409 0.544 0.386 0.13 0.31 0.498 0.805 0.602 1.003 0.812 0.477 0.515 0.138

BI890141 0.218 0.137 0.246 0.792 1.365 0.386 0.601 1.469 0.275 0.257 0.174 0.295

AI384140 -0.669 -0.801 -0.083 0.388 0.933 0.919 0.6 1.437 0.506 0.587 0.49 0.15

AI588696 0.306 -0.119 0.624 0.771 0.964 0.264 0.599 1.087 0.228 0.103 0.045 -0.072

AF039411 -0.705 -0.496 -0.617 -0.148 0.25 -0.008 0.596 1.573 0.25 0.922 0.662 0.158

AA495427 -0.788 -0.757 -0.145 0.004 0.325 1.278 0.596 1.715 0.429 -0.017 -0.293 0.206

AI793969 -0.451 -0.395 0.004 0.268 0.757 0.045 0.596 0.738 0.192 0.422 0.551 0.25

AW184237 -2.052 -1.684 0.479 0.079 0.66 0.798 0.595 0.363 1.142 0.284 0.738 0.428

AI721428 0.46 -0.218 -0.226 0.381 0.788 0.166 0.595 1.062 0.383 0.305 -0.027 -0.413

AI588190 -0.728 -0.001 0.259 0.339 0.604 1.124 0.593 1.295 0.832 0.802 0.267 -0.169

BI672765 0.059 0.622 0.077 -0.276 1.064 0.033 0.593 1.155 -0.073 -0.205 0.002 -0.093

BI866308 0.261 -0.022 0.261 0.563 1.093 0.858 0.592 1.13 0.412 0.121 0.797 0.847

AI331043 -1.565 -1.236 -0.708 -0.075 -0.076 0.568 0.586 0.307 0.324 0.26 0.335 -0.232

AW344113 0.091 0.172 1 0.771 0.897 0.909 0.586 1.296 0.317 0.281 -0.148 -0.02

BM024640 0.374 -0.03 0.378 0.634 0.651 0.312 0.585 0.874 0.09 0.459 0.351 0.272

AA658743 -0.777 -0.882 -0.138 0.241 0.702 0.328 0.579 1.535 0.358 0.785 0.581 0.128

AI545274 0.249 0.629 0.637 0.432 0.592 0.058 0.579 0.752 0.014 0.21 0.074 0.214

AI959657 0.55 0.686 0.626 0.72 0.729 0.374 0.577 0.821 0.202 0.402 -0.09 -0.25

AI437364 0.286 0.159 0.361 0.833 0.938 0.625 0.576 1.125 0.41 0.32 0.606 0.1

BI886127 0.255 -0.085 0.734 1.066 1.133 0.715 0.573 1.338 0.404 0.211 -0.053 -0.321

BI891681 -1.3 -1.649 -1.081 -0.119 0.28 -0.09 0.571 0.844 0.594 0.77 0.299 0.542

BG738252 -1.155 -0.118 0.654 0.679 0.991 1.174 0.57 1.256 0.719 0.749 0.604 0.506

BE201398 -0.619 0.545 0.508 0.58 0.563 0.361 0.566 1.091 0.799 0.096 0.286 -0.376

AI721587 -0.033 -0.06 0.542 1.116 1.282 0.449 0.566 1.408 0.635 0.466 0.139 0.169

AI964375 0.125 0.381 0.552 0.69 0.988 0.228 0.564 1.078 0.452 0.2 -0.005 -0.485

BI889526 -0.542 -0.235 0.916 1.028 1.015 1.218 0.563 1.338 0.373 0.122 -0.048 0.2

BI890108 -0.502 -1.355 0.259 0.369 0.902 0.873 0.558 1.819 0.902 1.173 0.543 0.044

BI891570 0.407 0.303 0.967 0.997 0.924 0.221 0.556 1.14 0.415 0.553 0.134 -0.183

BI846592 -0.52 -0.291 -0.575 0.186 1.094 1.081 0.554 1.892 0.678 1.301 0.118 -0.436

AW116722 0.525 0.542 0.007 0.417 0.614 0.377 0.554 1.037 0.342 -0.026 -0.199 -0.31

AI588475 -0.433 0.267 0.245 0.072 0.889 0.409 0.553 1.35 -0.078 -0.604 0.486 0.234

BM182248 0.2 0.884 0.332 0.627 0.838 0.006 0.552 1.2 0.205 0.412 0.443 -0.129

AF369382 -0.763 -0.006 -0.131 0.629 0.595 0.736 0.552 1.467 -0.304 0.657 0.708 0.574

AI793934 -0.071 0.136 -0.165 0.114 1.356 0.772 0.551 2.449 1.206 0.85 0.444 -0.18

AI793690 -0.058 0.081 -0.27 -0.071 0.038 0.456 0.551 1.453 0.487 0.102 0.722 0.704

AF210645 0.457 0.801 0.59 0.761 1.076 0.24 0.548 1.339 0.29 0.429 0.346 -0.131

BI878477 0.022 -0.058 0.234 0.272 0.519 0.699 0.542 0.952 0.331 0.742 0.306 0.314

AI957816 -0.729 -1.567 -1.601 -1.015 0.029 0.34 0.542 0.886 0.552 0.466 -0.205 -0.112

AI793880 0.294 0.613 0.98 0.732 1.227 0.492 0.541 1.367 0.465 0.118 -0.092 -0.511

AI721611 0.311 0.496 0.9 1.112 1.47 0.691 0.54 1.651 0.301 0.529 0.026 -0.248

AW171089 -0.18 -0.155 0.499 0.466 0.679 0.668 0.54 0.699 0.298 0.511 0.168 0.035

BE200552 -0.674 -1.039 0.077 -0.394 0.096 0.382 0.538 1.188 0.455 0.794 0.822 0.709

BI705182 -0.728 -0.185 0.155 0.106 0.832 0.507 0.536 1.471 0.908 0.234 0.708 0.082

U49412 -0.546 0.095 -0.166 0.391 0.748 -0.258 0.534 1.412 0.258 0.578 0.592 0.171

AI974209 -0.775 -0.388 -0.578 -0.255 0.382 0.071 0.53 1.333 0.019 0.258 0.194 -0.291

BI704359 -0.304 -1.06 0.096 0.645 1.735 0.303 0.528 1.749 0.437 0.283 0.529 0.44

BI673606 -0.078 0.373 0.325 0.767 0.994 0.313 0.526 1.359 0.067 0.529 0.105 -0.048

BI887659 -0.701 -0.062 0.214 0.668 1.419 0.944 0.525 1.618 0.846 0.473 0.175 0.002

AI588476 0.272 0.142 0.253 0.585 0.849 0.213 0.525 0.972 -0.053 0.44 0.232 0.263

BI984380 0.336 0.393 0.042 0.314 0.728 0.079 0.524 0.916 0.076 0.334 -0.021 -0.151

BI704280 -0.279 -0.691 0.123 0.992 0.841 0.25 0.523 1.311 0.568 0.54 0.595 0.417

AI601692 0.341 0.179 0.308 0.105 -0.163 0.039 0.522 0.564 0.155 0.241 0.217 0.294

AW117105 0.204 -0.309 0.113 0.221 0.367 0.624 0.522 0.667 0.272 0.16 0.261 0.011

AI974139 -0.093 -1.046 0.212 0.858 0.2 0.406 0.516 0.949 0.342 0.337 0.576 0.043

AW279985 1.203 -0.098 0.258 0.185 0.795 0.619 0.512 1.388 0.367 0.61 0.473 0.196

AW019238 -0.216 -0.052 0.308 0.259 0.529 0.395 0.511 1.461 0.615 0.015 0.247 -0.15

X65060 -0.646 -0.467 -0.024 0.006 0.701 0.742 0.508 1.287 0.144 -0.121 -0.148 -0.276

BG305366 0.322 0.513 0.245 0.638 0.53 0.491 0.506 0.911 0.162 0.815 0.398 0.204

BI883638 -0.728 0.378 0.775 1.612 1.456 0.707 0.503 1.887 -0.378 -0.449 -0.543 -0.913

BI863967 0.131 0.228 0.98 0.564 0.477 0.53 0.502 0.953 0.076 0.077 -0.141 -0.179

AI721616 0.691 0.11 0.198 -0.016 -0.117 0.319 0.501 0.952 0.286 0.653 0.175 -0.063

BM184237 0.094 0.504 0.321 0.699 0.965 -0.177 0.499 1.073 -0.014 0.031 -0.002 -0.173

AW171260 0.381 0.077 -0.427 -0.154 0.043 0.557 0.499 0.498 0.136 -0.414 -0.58 -0.524

BI979451 0.071 0.36 0.395 0.587 0.775 0.232 0.498 0.795 0.091 0.041 -0.14 -0.056

BI890704 0.302 0.594 0.268 0.378 0.358 0.844 0.498 0.95 0.372 0.328 0.004 -0.126

AI601467 0.295 0.45 -0.291 0.007 0.289 0.643 0.498 1.266 0.734 0.573 0.454 0.084

AW232435 0.644 0.616 0.426 0.362 0.253 0.831 0.497 0.966 0.474 0.834 0.413 0.335

BG303289 -0.632 0.013 0.411 0.523 0.544 0.1 0.496 1.044 0.188 0.146 0.331 0.149

BM034983 0.245 0.51 0.16 0.251 0.304 0.635 0.495 1.312 0.202 0.273 -0.363 -0.348

AA658750 0.041 0.208 0.33 0.477 -0.055 0.158 0.495 0.662 0.061 0.534 0.217 0.036

BG892019 0.159 0.083 0.669 0.665 0.672 0.278 0.495 0.822 0.211 0.453 -0.01 0.037

AW305462 0.421 0 0.585 0.565 0.322 0.704 0.492 0.879 0.265 0.389 -0.042 -0.058

BI879889 0.705 0.291 0.82 0.328 0.393 0.187 0.491 1.122 0.447 0.905 0.363 0.28

BG302807 0.549 0.227 0.123 0.95 0.915 0.701 0.488 1.128 0.562 0.239 0.2 -0.414

BG306178 0.08 0.793 0.318 0.59 0.873 0.06 0.486 1 0.686 -0.001 -0.122 0.064

AI793372 0.432 0.756 0.352 0.117 0.601 -0.04 0.484 1.118 0.3 0.272 0.27 0.282

AW019266 -0.253 0.726 0.354 0.75 1.036 -0.332 0.482 1.434 0.373 -0.17 -0.141 -0.236

AI931096 0.099 0.244 0.837 0.742 1.172 0.295 0.481 1.511 0.302 0.834 0.223 0.106

AW343883 0.373 0.24 0.551 0.566 0.89 1.023 0.477 1.217 0.45 0.045 -0.234 -0.461

AF184245 -0.307 -0.157 -0.004 0.143 0.742 0.353 0.475 1.435 0.402 0.764 0.143 0.369

BI890247 0.321 0.482 0.614 0.81 1.18 0.528 0.473 1.237 0.183 0.411 0.32 0.043

BI878923 0.3 0.254 1.012 0.878 1.016 0.996 0.472 1.171 0.444 0.739 0.144 0.102

Y14531 -0.339 0.288 0.407 0.237 -0.081 0.1 0.471 1.725 0.067 -0.194 0.5 0.227

BI890508 0.304 -0.413 0.111 0.245 0.847 0.552 0.47 0.83 0.356 0.268 0.406 0.471

AW078266 -0.078 -0.497 0.216 0.744 1.043 -0.03 0.47 1.069 0.415 0.355 0.564 0.132

AY017308 -0.808 0.235 0.33 0.476 1.175 0.352 0.469 1.762 0.836 0.169 0.921 0.336

AF162696 -0.76 -0.417 0.218 0.299 0.412 0.356 0.468 0.214 0.738 0.699 0.679 0.363

BE015668 -0.182 1.074 1.08 0.947 0.827 0.062 0.468 1.818 0.313 0.286 -0.556 0.062

AI545947 0.164 -0.293 -0.11 0.073 0.438 0.215 0.468 0.779 0.231 0.233 0.068 -0.42

AW058992 -0.039 -0.188 -0.093 0.244 0.086 -0.093 0.467 0.636 -0.128 0.402 -0.015 -0.666

BI889202 -0.289 -0.363 0.182 0.284 0.772 0.344 0.466 1.021 -0.036 0.026 0.164 0.177

AI722402 0.497 0.816 0.284 0.611 0.811 0.596 0.465 1.401 0.527 0.218 -0.107 -0.516

AI883326 -0.163 -1.796 -0.008 -0.222 0.779 0.658 0.464 1.484 0.917 0.872 0.61 0.264

BM154004 0.531 0.71 0.359 0.425 0.795 0.171 0.464 0.92 -0.072 0.22 0.05 0.197

AW116386 -0.049 0.603 0.558 0.815 0.705 0.072 0.464 0.967 0.141 0.109 0.195 -0.153

U77595 0.054 -0.069 0.181 0.283 0.299 0.545 0.463 1.15 0.403 0.323 0.356 0.031

BG891893 -0.21 0.27 -0.006 0.373 0.649 0.117 0.462 0.588 0.186 0.069 0.149 0.247

AI666989 -0.332 -0.525 -0.538 0.242 0.673 1.069 0.462 1.38 0.822 1.018 1.017 0.511

BM026316 0.367 0.518 0.407 -0.014 0.108 0.602 0.459 0.746 0.102 0.154 -0.219 0.032

AI667324 0.467 -0.606 0.223 0.693 0.376 0.021 0.458 0.789 0.113 0.431 0.04 -0.264

AW154414 -0.098 0.387 0.774 0.743 1.154 0.213 0.457 1.152 0.089 0.296 0.058 0.102

BI890013 0.559 -0.731 0.232 -0.039 0.617 0.482 0.456 0.94 0.489 0.809 0.704 -0.197

BM025866 0.014 0.147 0.154 0.431 0.999 0.036 0.455 1.55 0.297 0.729 0.231 0.056

BI318628 0.088 0.256 0.334 0.489 0.756 0.102 0.454 1.29 0.105 0.162 0.433 0.088

AI793487 -0.114 -0.2 -0.067 -0.477 0.332 -0.035 0.451 1.478 0.196 0.884 0.973 0.308

AW128413 0.744 0.198 0.701 0.257 0.598 0.514 0.451 1.35 0.585 0.37 0.263 -0.412

AW076659 0.034 -0.111 0.243 0.034 0.511 0.546 0.451 0.871 0.656 0.781 0.076 0.414

BI887166 0.133 0.242 0.172 0.361 0.636 0.122 0.45 0.833 0.156 0.035 -0.074 -0.18

BM102623 0.191 0.548 0.08 0.494 0.914 0.222 0.45 1.166 0.294 0.126 0.137 0.102

BI877263 0.119 0.345 0.164 0.219 0.266 0.125 0.448 0.684 0.17 0.009 -0.103 -0.219

AI957815 0.153 0.465 0.694 0.433 0.691 0.478 0.448 1.005 0.234 0.203 0.013 0.066

AI964971 -0.306 -1.124 -0.387 -0.436 -0.022 0.591 0.447 0.853 0.598 0.703 0.323 -0.139

AF143493 -0.013 -0.285 0.104 -0.093 0.029 0.255 0.447 1.058 0.43 0.858 0.295 0.039

AW077940 -0.856 -0.226 0.373 0.554 0.555 0.273 0.446 1.243 0.733 0.174 0.514 0.69

AI884026 0.565 0.023 -0.148 0.177 0.318 0.5 0.446 1.012 0.384 0.222 0.032 -0.619

AI641523 -0.172 0.17 -0.164 0.213 1.067 -0.067 0.444 1.602 0.26 0.11 0.033 -0.017

AW420705 -0.372 -0.078 0.27 0.464 0.984 -0.113 0.442 0.959 0.266 0.249 0.412 0.311

AW232241 -0.089 0.129 0.047 0.222 0.233 1.098 0.441 1.182 0.638 0.768 0.663 0.428

BI889933 0.021 0.406 0.306 0.636 0.852 0.102 0.439 1.003 0.256 0.303 0.443 -0.062

AW059104 -0.452 -0.557 -0.719 -0.07 -0.082 0.753 0.438 1.768 0.401 0.533 0.671 0.06

BG302868 0.489 -0.089 0.111 0.492 0.677 0.755 0.438 1.127 0.455 0.336 0.142 -0.371

BG985777 0.309 0.374 0.744 0.386 0.72 0.534 0.437 1.592 0.551 0.72 -0.118 -0.579

BM103943 -0.252 -0.35 0.106 0.388 1.142 0.929 0.436 1.385 0.36 0.752 0.646 0.303

AI657922 -0.116 -0.578 -0.066 0.346 0.471 0.231 0.436 0.797 0.129 0.511 0.483 0.49

AW059217 -0.014 -0.236 0.313 -0.342 0.351 -0.25 0.435 1.328 0.246 0.191 0.87 -0.109

BI890730 0.135 -0.08 0.171 0.591 1.052 0.243 0.432 1.53 0.565 0.062 -0.264 -0.052

BI707482 -0.38 -1.249 -0.961 -0.459 0.531 0.528 0.432 0.733 0.787 0.623 0.403 0.525

BI889310 -0.124 0.214 0.238 0.664 0.692 0.334 0.432 0.748 0.197 0.206 -0.427 -0.472

AI878386 -0.662 0.18 0.463 0.782 1.305 0.18 0.431 1.531 0.258 0.763 0.271 0.937

AW117076 -0.788 -0.032 0.368 1.124 1.434 0.635 0.431 1.525 0.822 0.522 0.35 0.111

BM182750 0.234 0.431 0.012 0.411 0.521 0.563 0.43 0.877 0.183 0.157 -0.044 -0.157

AI722829 -0.224 -0.395 -0.176 0.306 0.288 0.022 0.43 1.018 0.1 0.542 0.578 0.324

BI878085 -0.526 0.415 0.407 0.288 0.202 -0.151 0.43 1.141 0.264 -0.131 0.569 0.335

BI889786 -0.592 -0.224 0.329 -0.058 0.719 0.242 0.43 1.88 0.831 0.626 0.618 -0.513

AI666923 0.55 0.252 -0.219 0.375 0.395 0.063 0.429 0.674 0.13 0.324 -0.05 -0.087

BI889302 -0.309 0.105 0.437 0.073 0.165 0.014 0.428 1.442 0.082 0.122 -0.083 0.527

BG303613 0.671 0.767 0.7 0.937 0.708 0.574 0.426 1.408 0 0.325 -0.119 -0.285

BI866342 0.452 0.068 -0.178 0.055 0.278 0.625 0.425 0.881 0.291 0.397 0.016 0.007

BI850036 -0.381 0.056 0.543 -0.309 -0.03 0.063 0.425 0.655 0.134 -0.065 0.434 0.083

AF030283 -0.49 -0.014 0.074 0.132 0.328 0.359 0.424 1.649 0.341 0.292 1.011 0.076

AI588708 -0.728 -0.544 0.996 0.962 1.027 0.593 0.424 0.634 1.445 0.812 0.558 0.162

AW343324 -0.004 0.199 0.592 0.629 0.609 0.435 0.42 1.155 0.283 0.495 0.512 0.554

AI877678 0.233 0.399 0.186 0.598 0.928 -0.074 0.416 1.391 0.687 0.393 0.459 -0.097

AI959106 -0.626 -0.271 -0.568 -0.182 0.529 1.267 0.413 1.391 0.362 0.571 0.146 -0.026

AF257519 -0.154 -0.169 -0.168 0.507 0.914 0.658 0.413 0.961 0.053 0.316 -0.035 -0.274

AI416128 -0.248 -0.285 0.115 0.198 0.524 0.178 0.413 0.819 0.445 0.495 0.036 -0.106

AF395113 -0.6 -0.524 -0.397 0.002 0.366 0.461 0.413 0.778 0.283 0.033 0.597 0.003

AF061252 -0.637 0.223 -0.483 -0.334 -0.429 0.25 0.412 1.095 0.387 0.447 0.773 0.787

BI891001 -1.158 -1.384 -0.627 0.24 0.119 0.592 0.412 1.011 0.16 0.531 0.659 0.802

BI891068 -0.046 0.847 0.558 0.753 1.104 0.043 0.411 1.189 0.138 0.193 0.119 0.113

U77627 0.101 0.147 0.3 0.64 0.963 1.236 0.407 1.358 0.766 0.434 0.17 -0.376

AI722496 -0.011 0.319 0.407 0.644 0.704 0.181 0.406 0.834 -0.024 0.324 -0.195 -0.03

AI477424 0.691 -0.221 -0.071 0.057 0.64 0.92 0.401 1.207 0.367 0.707 0.506 0.251

BF937404 0.212 0.156 0.651 0.435 0.607 0.324 0.4 0.773 0.24 0.602 -0.049 0.081

AW343911 -0.114 -1.189 -0.055 0.133 0.529 0.566 0.399 1.256 0.611 0.682 0.188 -0.321

BG891864 -0.132 -1.478 -0.474 -0.068 0.363 0.3 0.399 0.859 0.068 0.103 -0.12 0.195

AI588225 -0.567 0.041 0.126 -0.254 -0.134 -0.255 0.399 0.78 0.111 0.432 0.646 0.074

BM102070 -0.146 -0.157 0.071 0.101 0.68 0.341 0.395 0.958 0.272 0.225 0.264 0.127

BI430251 -0.274 -0.238 -0.341 -0.236 0.341 0.221 0.393 1.176 0.249 0.38 0.78 0.069

BM036521 0.598 -0.516 -0.007 0.168 0.566 0.322 0.39 1.266 0.366 0.566 0.331 0.037

AI641051 0.208 -0.129 0.006 0.333 0.011 0.176 0.389 0.722 0.391 0.411 0.488 0.355

AW058875 0.214 0.637 0.516 0.708 0.6 0.046 0.389 1.093 0.217 -0.139 0.384 -0.029

BI710051 -0.037 0.579 0.505 0.256 -0.097 0.487 0.388 0.65 0.534 0.469 -0.082 -0.392

BM184045 0.084 0.626 0.251 0.688 0.601 0.427 0.387 0.698 0.085 0.216 0.081 0.009

BI883910 0.607 0.336 -0.259 -0.077 0.421 0.596 0.387 0.75 0.407 0.354 0.28 -0.016

AI943082 -0.317 -0.636 -0.741 -0.255 0.879 0.382 0.386 1.002 0.374 0.663 0.355 0.272

BI888505 0.288 0.726 0.419 0.644 0.852 -0.256 0.384 1.143 -0.022 0.092 0.002 -0.229

BI878279 0.3 0.394 0.667 0.684 1.069 0.373 0.383 1.175 0.197 0.486 0.1 -0.11

BI672058 -0.224 -0.816 0.346 0.071 0.371 0.61 0.381 0.825 0.307 0.334 0.672 0.194

BI706099 -0.055 0.244 -0.038 0.179 0.294 0.177 0.38 0.61 0.124 0.205 -0.161 0.187

AW281219 0.341 -0.868 -0.066 0.243 0.54 0.726 0.378 1.288 0.465 0.608 0.631 0.27

BG727890 -0.012 -0.366 0.39 0.278 0.043 0.527 0.378 0.565 0.079 0.365 -0.012 -0.1

BM182302 -0.191 0.224 0.419 0.228 0.811 0.059 0.376 0.998 -0.039 0.183 0.541 0.186

BI843129 0.032 0.708 0.535 0.885 0.934 -0.018 0.376 1.124 0.078 0.23 0.099 -0.296

AF177869 -0.261 0.375 0.536 0.329 0.056 0.543 0.376 0.783 0.577 0.051 0.253 -0.04

BI673379 0.753 0.688 0.925 0.464 0.553 0.563 0.375 1.037 0.089 0.173 -0.032 -0.124

AI722515 -0.114 -0.675 0.045 0.037 0.398 0.461 0.374 1.338 0.517 0.515 -0.171 -0.885

BI878464 -0.075 0.635 0.266 0.656 0.628 -0.246 0.374 1.176 0.277 -0.514 -0.365 -0.245

AF354750 -0.023 -0.284 0.184 0.206 0.451 0.198 0.372 0.968 0.506 0.8 0.341 0.12

AI793428 -0.228 -0.384 -0.578 -0.249 0.072 0.3 0.371 0.732 0.447 0.628 0.208 0.355

BI842822 -1.016 -0.271 -0.224 0.258 1.266 0.882 0.371 1.271 1.214 0.726 -0.008 -0.18

L25273 -0.733 -0.728 -0.523 -0.321 -0.617 -0.451 0.37 0.866 0.162 0.341 0.249 0.65

BM184100 0.398 -0.112 0.133 0.409 1.143 0.212 0.37 1.15 0.223 0.379 0.104 0.201

AF270789 -0.768 0.025 0.009 0.201 0.555 0.715 0.369 2.49 0.706 -0.086 0.024 -0.31

BM184886 0.512 0.378 0.148 0.219 0.709 0.051 0.368 0.842 -0.089 0.038 0.019 -0.12

BM004967 0.139 0.479 0.187 0.056 0.033 0.512 0.366 1.108 0.384 0.724 0.381 0.963

AI964352 -0.118 -0.94 -0.222 0.035 0.062 0.362 0.365 0.761 0.318 0.668 0.401 0.209

AI793927 -0.529 0.067 -0.017 0.454 0.945 0.033 0.364 1.141 0.392 0.461 0.037 -0.36

BI562940 -0.06 -0.044 0.106 0.389 0.862 1.158 0.364 1.418 0.699 1.054 0.773 0.669

BM023784 0.268 0.468 -0.161 0.068 0.262 0.487 0.363 1.108 0.378 0.4 0.293 0.112

AI558431 -0.044 -0.079 0.01 0.708 0.633 0.555 0.363 1.151 0.241 0.472 0.246 -0.199

BI878520 0.1 0.287 0.144 0.035 0.19 0.463 0.361 1.139 0.462 0.548 0.454 0.218

AW174857 0.29 -0.631 -0.05 -0.074 0.383 0.505 0.36 1.174 0.454 0.642 0.457 0.329

AW777479 0.049 -0.186 0.12 0.715 0.884 -0.048 0.359 1.185 0.444 0.262 0.262 -0.189

AJ132931 0.169 0.456 0.171 0.132 0.26 0.227 0.358 1.171 0.025 0.341 0.032 0.226

AF067532 -0.332 -0.29 -0.013 -0.33 -0.07 -0.09 0.358 1.309 0.122 0.128 0.457 0.812

BI850039 0.1 0.126 0.228 0.623 0.88 0.045 0.356 1.09 0.158 0.709 0.145 0.304

AI884050 -0.403 -0.444 0.494 0.806 1.05 0.714 0.356 1.399 0.134 0.716 0.025 0.258

AI584440 -0.704 -0.12 -0.491 0.252 0.611 0.684 0.356 0.99 0.288 0.438 0.377 0.237

BI886229 0.375 -0.317 -0.203 -0.213 0.042 0.496 0.352 0.69 0.702 0.435 0.591 0.527

BM026429 0.237 0.112 0.195 0.334 0.646 0.694 0.352 1.212 0.319 0.463 0.054 0.085

AW133803 0.254 -0.058 0.032 0.345 0.447 0.014 0.35 0.624 0.378 0.373 0.186 0.211

AI942585 -0.843 -0.683 -0.746 -0.875 -0.652 -0.014 0.347 1.265 0.563 0.823 0.447 0.11

AW078116 0.282 0.095 0.469 0.446 0.505 0.278 0.346 0.749 0.143 0.447 0.009 0.162

AI331515 -1.071 -1.318 -1.511 -0.609 -0.23 0.267 0.346 0.899 0.579 0.513 -0.479 -0.35

BE606087 -0.052 -0.401 -0.179 0.094 0.115 0.046 0.345 1.013 0.465 0.889 0.18 -0.148

BI476729 0.063 -0.106 0.142 0.163 0.568 0.101 0.344 0.921 -0.099 0.38 0.234 0.358

BE017831 0.538 0.104 0.372 0.222 0.359 0.432 0.344 0.809 0.055 0.45 0.032 -0.251

BM185367 0.436 0.298 0.508 0.38 0.577 0.591 0.344 0.805 0.36 0.485 0.212 0.158

BI887574 -0.626 -0.374 0.745 -0.085 0.26 0.537 0.343 0.787 0.467 0.654 -0.021 0.28

AY029527 -0.174 -0.863 0.398 0.28 -0.097 0.795 0.341 -0.083 1.029 0.829 0.22 -0.052

AY008836 -0.811 -0.735 -1.049 -0.212 0.15 0.323 0.34 1.645 0.148 0.727 0.314 0.86

AI545040 -0.029 0.338 -0.164 0.209 0.508 -0.019 0.34 0.66 -0.159 0.143 0.153 0.511

AW203038 -0.059 0.351 -0.203 -0.148 0.441 0.361 0.34 0.717 -0.241 -0.36 -0.183 -0.433

AW174887 -0.282 0.635 0.886 0.909 1.184 0.015 0.339 1.237 0.293 0.34 0.276 -0.102

BM103340 -1.107 -0.902 0.261 0.425 0.277 0.638 0.337 0.481 0.69 0.539 0.115 -0.329

AJ290391 -0.311 0.368 0.397 0.625 0.558 -0.267 0.337 0.764 -0.007 -0.37 0.082 -0.231

BM095868 -0.75 -0.797 -0.393 -0.154 0.532 -0.016 0.337 1.427 0.743 0.591 1.118 0.455

AW058816 0.159 0.187 0.357 0.631 0.419 0.019 0.333 0.955 -0.057 0.549 0.158 -0.125

AW420405 -0.071 -0.023 0.186 0.047 0.649 0.501 0.328 0.902 0.125 0.178 -0.003 0.148

AI437466 -0.387 -0.621 -0.058 -0.887 0.112 0.216 0.328 1.015 0.692 0.34 0.449 0.426

AI721333 0.565 0.614 0.097 0.184 0.683 0.681 0.327 0.74 0.198 0.36 -0.057 -0.101

AI883967 0.084 0.349 0.644 0.591 1.085 0.535 0.326 1.413 0.054 0.163 -0.2 -0.493

BI891122 -0.222 -0.152 0.366 0.649 0.468 0.617 0.326 0.919 0.295 0.652 0.158 0.151

BI892151 -0.482 0.739 0.814 1.05 1.093 0.584 0.325 1.247 0.461 0.128 0.047 -0.341

BI886163 0.361 -0.104 -0.048 -0.308 0.044 0.455 0.325 0.939 0.197 0.443 -0.016 -0.08

BI843230 0.316 -0.747 0.132 0.234 0.402 0.536 0.324 0.727 0.139 0.246 -0.305 -0.682

Y14533 -0.755 -0.185 -0.048 -0.962 -0.1 0.132 0.324 1.793 0.594 -0.331 0.967 -0.072

AW076666 0.053 -1.078 0.156 0.276 0.48 0.37 0.324 1.105 0.231 0.925 0.675 0.261

BG728956 -0.929 -0.46 -1.681 -0.726 -1.024 -0.008 0.322 1.004 0.57 0.774 -0.277 -0.505

BG985449 0.908 -0.539 -0.955 -0.986 -1.351 0.252 0.322 0.346 1.054 0.678 0.291 0.228

BI883018 -0.337 -0.472 0.065 -0.359 0.905 0.68 0.321 0.851 0.161 0.512 0.402 -0.028

AF071255 -0.905 0.093 -0.778 0.244 -0.325 0.26 0.318 1.383 0.516 0.658 0.965 0.502

AW154507 0.028 0.573 0.343 0.265 0.538 -0.254 0.317 0.818 -0.045 -0.506 -0.399 -0.431

BI888344 -1.041 -1.027 0.084 0.256 0.891 0.619 0.313 0.673 1.001 0.263 0.657 0.388

AI722538 -0.034 0.225 0.152 0.562 0.77 0.131 0.309 1.052 0.611 0.717 0.239 -0.322

AF032392 0.235 -0.672 -0.855 0.019 0.458 0.342 0.308 1.282 0.319 0.938 0.257 0.16

BF938837 -0.15 0.631 0.229 0.472 0.262 0.044 0.308 0.665 -0.017 0.075 0.017 0.087

BM157248 0.826 0.935 0.283 0.433 0.388 -0.127 0.306 1.135 0.211 0.498 0.277 0.144

BG729245 -0.412 -0.172 -0.486 -0.946 -0.063 0.099 0.305 0.898 0.999 0.289 0.1 0.362

AW154468 -0.342 -0.932 -0.562 -0.633 -0.581 -0.276 0.304 1.138 0.262 0.285 0.593 0.406

AI331953 0.488 0.324 -0.25 -0.25 0.06 0.346 0.303 1.086 0.233 0.88 0.162 0.246

BI888801 0.314 0.814 0.626 0.715 0.243 -0.121 0.302 0.97 0.136 0.63 0.515 -0.061

AI793853 -0.231 0.266 0.239 0.595 1.231 0.052 0.301 1.25 0.584 0.53 0.156 0.758

BE558061 -0.099 0.286 0.105 -0.236 0.339 -0.172 0.295 0.679 0.012 -0.174 -0.116 0.425

AF071248 -0.341 0.06 -0.074 0.346 0.185 -0.23 0.293 1.46 0.299 0.384 0.365 0.006

BM185168 0.14 -0.359 -0.911 -0.641 -0.545 0.387 0.293 1.006 0.334 0.439 0.159 0.371

BM036297 -0.107 -0.63 0.247 0.194 0.572 0.37 0.29 0.74 0.261 0.232 -0.027 0.016

BG728626 -0.497 0.217 0.21 -0.233 -0.117 0.035 0.289 0.951 -0.057 -0.26 -0.216 -0.117

BI885253 0.145 -0.137 0.177 0.218 0.499 0.154 0.289 0.887 0.481 0.391 0.375 0.019

BE201102 -0.578 -0.002 0.328 -0.584 -0.775 -0.235 0.285 1.231 0.15 0.147 -0.153 -0.326

AF202054 0.13 -0.536 -0.569 -0.264 -0.092 0.16 0.284 1.028 0.177 0.464 0.083 -0.08

AW019579 0.906 0.853 0.742 0.673 0.84 0.675 0.283 1.079 0.481 0.085 0.233 0.453

BI708781 0.073 0.201 0.265 0.527 0.675 0.398 0.281 0.786 0.106 0.247 -0.006 -0.206

AI882884 -0.177 -0.613 -0.234 -0.156 0.273 0.05 0.281 1.255 0.692 0.836 0.55 -0.114

BI885907 0.416 0.36 0.02 -0.174 0.313 0.857 0.281 0.893 0.136 0.273 -0.049 -0.2

AF255044 -0.371 0.262 0.783 1.146 1.548 -0.188 0.28 1.75 0.25 -0.007 0.072 -0.027

AI793350 -0.234 -0.906 -0.018 -0.168 0.372 -0.106 0.28 1.278 0.1 0.523 0.352 0.264

BI882169 0.11 -0.045 0.026 0.393 0.693 0.66 0.272 1.09 0.126 0.275 -0.025 -0.012

BI877622 0.153 -0.125 0.64 0.42 0.789 0.275 0.272 0.846 0.019 0.412 0.136 0.265

AI666975 -0.728 -0.309 0.323 1.066 1.581 0.041 0.272 1.619 1.134 0.562 0.345 -0.237

AI641124 -0.24 -0.89 -0.023 -0.238 0.248 0.41 0.271 0.988 0.134 0.485 0.265 0.01

AW154716 -1.197 -0.184 -1.041 -0.576 -0.364 0.245 0.27 1.144 1.325 1.127 0.896 0.699

BI476547 0.147 0.468 0.546 0.176 0.55 0.333 0.265 1.093 0.116 0.204 -0.104 -0.039

AI397023 -0.387 -0.111 -0.141 0.058 0.484 0.303 0.265 0.886 0.336 0.445 0.629 0.44

AI957409 -0.502 -1.417 -0.925 -0.707 -0.655 -0.092 0.264 1.075 0.989 0.915 0.962 0.066

AW059220 0.235 -0.046 0.575 0.652 1.364 0.493 0.261 1.408 0.062 0.507 -0.006 -0.279

BM157095 -0.138 0.607 0.684 0.72 0.69 0.491 0.261 1.014 0.605 0.342 0.09 -0.38

AI964108 -0.001 0.685 0.155 0.717 0.533 0.354 0.258 0.732 0.236 -0.167 0.394 -0.194

AW116978 -0.312 -0.771 -0.522 -0.709 -0.091 0.037 0.257 0.961 0.14 0.23 0.677 0.208

BI892431 0.001 0.348 0.383 0.528 0.428 -0.117 0.255 0.784 0.191 -0.035 -0.11 0.045

BI704180 0.319 0.358 0.727 0.517 0.876 0.657 0.255 1.283 0.477 0.025 -0.332 -0.762

BM184838 0.555 0.055 0.702 0.275 0.941 0.633 0.254 1.262 0.75 0.825 0.183 -0.06

AI884148 -0.074 -0.159 0.254 0.351 0.335 0.363 0.253 0.715 0.446 -0.091 0.54 0.226

AI584986 -0.36 0.029 -0.226 0.105 1.225 -0.155 0.253 1.454 0.486 0.322 0.519 0.264

BM156717 0.249 0.103 -0.327 -0.045 0.042 -0.217 0.252 0.733 0.051 0.586 0.119 0.344

AW466509 0.628 0.29 -0.333 0.205 0.295 0.679 0.251 0.769 0.262 0.518 0.539 -0.117

AW233586 -0.114 0.677 0.161 0.532 0.429 -0.106 0.25 0.911 -0.139 0.129 -0.032 -0.018

AI942627 -0.07 0.135 -0.116 0.096 0.152 -0.001 0.248 0.638 0.116 0.154 0.271 0.1

AF361478 -0.037 0.524 0.601 0.764 0.631 0.857 0.246 0.954 0.331 0.758 0.567 0.04

BM184509 0.564 0.656 0.638 0.63 0.418 0.275 0.244 0.901 0.298 0.514 0.666 0.433

AW077961 -2.047 -1.221 -1.763 -0.814 -0.336 0.25 0.242 1.024 0.522 0.434 -0.41 -0.603

AI558845 -0.504 0.514 0.444 0.392 0.199 -0.248 0.242 0.741 -0.195 0.168 -0.217 -0.234

AW059266 0.158 0.215 0.188 0.286 0.696 0.945 0.241 1.054 0.253 0.621 0.474 0.113

BG304219 -0.361 -0.025 -0.153 -0.098 -0.032 0.003 0.239 0.952 0.087 -0.122 0.053 -0.176

BI879489 0.118 -0.063 -0.06 0.04 0.258 0.617 0.239 0.842 0.35 0.73 0.333 0.425

AJ006310 -0.664 0.042 -0.077 0.124 0.057 -0.003 0.237 2.107 0.619 0.539 0.451 -0.207

BG985738 0.078 -0.628 0.151 -0.304 0.21 0.055 0.237 1.621 0.634 0.365 0.529 0.32

BM185198 -0.061 -0.147 0.116 -0.103 0.653 0.341 0.236 0.891 0.133 0.162 -0.018 0.275

BI889131 -0.601 -0.014 0.006 0.433 0.771 -0.04 0.234 0.902 0.287 0.335 0.091 -0.054

BI886187 -0.127 0.156 0.777 0.348 0.603 0.188 0.232 0.835 0.332 0.304 0.002 -0.029

BE605273 -0.283 -0.062 -0.172 0.329 0.25 -0.038 0.23 0.66 0.254 0.527 0.28 0.449

AI793974 -0.055 -0.485 0.167 -0.184 0.429 0.185 0.228 1.131 0.153 0.149 0.022 0.038

BE606152 -0.55 -0.017 -0.217 0.158 1.158 0.562 0.227 1.464 0.395 0.658 0.113 0.532

BI887346 -0.167 -0.595 -0.09 0.205 0.414 0.462 0.227 1.161 0.579 0.595 0.144 -0.045

BI864451 0.26 0.605 0.67 0.508 0.044 0.342 0.226 0.95 0.162 0.759 0.497 0.026

BI887559 -0.43 -0.904 0.027 0.295 0.577 0.494 0.226 0.983 0.265 0.658 0.243 0.282

BI888033 -0.728 0.119 -0.124 0.55 1.038 -0.063 0.225 1.296 0.491 0.525 0.516 0.404

AI882781 0.351 0.068 0.312 0.157 0.244 0.141 0.224 0.528 0.112 0.237 0.376 0.064

AI878021 -0.333 -0.343 -0.879 -0.108 0.35 0.374 0.223 1.184 0.342 0.948 0.392 0.385

AI601297 -0.613 0.279 -0.266 -0.337 0.239 0.318 0.221 1.004 0.45 0.447 0.833 0.567

AI877609 -0.145 0.023 0.261 0.489 0.494 0.029 0.22 0.615 0.173 0.307 -0.154 0.025

AW171367 0.452 0.272 0.154 0.507 0.808 0.712 0.217 1.368 0.436 0.289 -0.271 -0.79

AJ005026 -0.342 0.254 -0.072 0.343 0.378 0.355 0.217 0.708 0.468 0.094 0.385 0.193

AW059030 0.104 -0.347 0.134 0.258 0.526 0.516 0.216 1.421 -0.122 -0.024 0.64 0.033

AW343508 0.249 0.318 0.471 0.047 0.663 0.561 0.214 0.925 0.137 0.332 -0.055 0.053

BG305992 -0.157 0.144 0.072 0.335 0.219 -0.068 0.211 0.688 0.035 0.378 0.111 0.128

AI330707 0.118 -0.108 -0.728 -0.276 0.377 0.156 0.211 1.068 0.26 0.647 0.376 0.078

BM182275 0.121 0.366 0.235 0.671 0.456 -0.113 0.207 0.762 0.153 0.356 0.512 -0.089

AI584421 0.379 -0.258 -0.486 0.06 0.091 0.279 0.204 0.574 0.31 0.306 -0.139 -0.251

AW128211 -0.266 -0.234 0.154 -0.416 0.363 -0.163 0.204 1.017 0.087 0.403 -0.314 -0.426

AI878758 -0.266 0.514 0.209 0.341 0.214 0.219 0.203 0.953 0.368 -0.117 -0.32 -0.079

BI892100 -0.454 -0.31 -0.4 -1.317 -0.335 0.121 0.202 1.495 0.386 0.402 0.552 0.297

AI657601 -0.706 -1.033 -1.125 -0.355 0.11 0.124 0.199 1.333 0.738 0.992 0.306 -0.36

AI959670 0.186 0.643 0.592 0.517 -0.191 0.204 0.199 1.025 0.221 -0.353 0.053 0.037

BI892176 0.513 0.527 0.17 0.424 0.305 0.068 0.198 0.792 0.181 0.567 0.08 -0.256

BE016083 -0.035 -0.098 0.035 0.456 0.474 0.431 0.197 0.631 0.356 0.278 0.189 0.181

AW171447 0.667 0.164 0.611 0.125 0.573 0.439 0.197 1.374 0.402 0.225 0.222 -0.33

BI888258 -0.51 0.062 0.597 0.844 1.021 -0.054 0.195 1.114 0.218 0.218 0.296 0.178

AI794483 -0.643 -0.774 -0.534 -0.417 -0.028 0.101 0.194 0.935 0.181 0.428 0.182 0.611

BM026032 0.384 0.164 0.846 0.284 0.554 0.438 0.193 0.89 0.207 0.249 -0.031 -0.132

AA497159 -0.012 0.621 0.133 0.342 0.563 0.228 0.192 1.263 0.121 0.358 0.413 -0.124

BF718175 -0.286 -0.042 0.059 0.483 -0.026 0.257 0.192 0.661 0.306 0.286 0.363 0.357

AW421309 -0.122 0.398 0.321 0.5 0.544 0.231 0.19 1.307 0.613 0.281 0.215 -0.162

BI672219 0.247 -0.101 -0.38 0.034 0.498 0.312 0.19 0.671 0.086 -0.432 0.067 -0.231

BI843519 -0.63 -0.289 -0.402 -0.171 0.878 -0.011 0.19 1.519 0.458 -0.708 -0.575 -0.416

BM036954 -0.079 0.525 0.89 0.22 1.117 0.466 0.19 1.613 0.165 0.167 0.228 0.004

AF200950 -0.381 -0.19 0.329 0.669 0.266 0.25 0.189 0.783 0.699 0.353 0.556 -0.118

BG308784 0.444 0.503 0.804 0.768 0.886 0.339 0.189 1.159 0.033 0.518 0.085 0.168

BI888177 -0.01 0.348 -0.054 0.445 0.422 0.422 0.189 0.886 0.49 0.258 0.348 0.211

AI461367 -0.352 -0.114 0.283 0.108 1.076 0.493 0.188 1.131 0.361 0.796 0.867 0.709

AB032263 -0.416 0.361 0.039 0.596 0.933 0.617 0.188 1.09 -0.117 0.689 0.233 0.008

AW116039 -0.055 -0.295 0.189 0.181 0.877 0.581 0.187 1.28 0.467 -0.106 -0.044 -0.391

BI979064 -0.218 -0.288 -0.129 -0.046 -0.31 0.021 0.185 0.941 0.209 0.108 0.337 0.199

BI892134 -0.265 0.443 0.555 0.226 0.768 0.073 0.184 1.085 -0.059 0.162 -0.417 -0.216

BI845781 -0.008 0.347 0.11 0.182 0.395 -0.03 0.183 0.903 0.18 0.137 0.487 0.039

BM186124 -0.343 -0.094 0.164 -0.309 0.013 -0.168 0.181 0.864 0.348 0.497 0.628 0.32

AW019613 -0.08 -0.118 -0.361 0.092 0.006 0.044 0.174 0.594 0.079 0.024 -0.144 -0.242

BI867489 -0.16 -0.564 -0.125 0.387 0.646 0.258 0.174 0.87 0.14 0.63 0.463 -0.099

AW128372 -0.413 -0.823 -0.677 -1.099 -0.88 0.196 0.171 1.339 0.938 0.645 0.184 -0.491

AI584556 -0.399 0.289 0.283 0.759 -0.031 -0.01 0.17 0.987 0.238 0.344 0.3 0.338

AI588304 -1 0.029 0.025 -0.225 -0.148 0.099 0.169 1.064 -0.005 0.687 0.515 -0.214

AW115897 0.181 -0.533 -0.133 -0.214 -0.004 -0.083 0.168 0.65 -0.03 0.017 0.118 0.078

AW421172 -0.081 0.036 -0.268 0.755 0.219 0.32 0.166 1.056 0.125 0.761 0.275 0.124

BG891888 0.237 0.63 0.372 0.591 -0.076 0.396 0.163 0.737 0.082 0.35 0.254 -0.278

BG304234 0.568 0.008 0.163 0.06 0.227 0.673 0.158 1.032 0.392 0.569 0.254 0.199

BI429020 -0.22 -0.742 -0.084 0.032 0.357 0.415 0.154 1.037 0.637 0.835 0.106 -0.164

BM183007 0.444 0.612 0.397 0.323 0.502 0.597 0.151 1.044 0.517 0.875 0.719 0.593

AW778179 0.308 -0.115 -0.143 -0.24 -0.255 -0.109 0.15 1.203 0.796 0.798 0.824 0.035

BI982770 -0.661 -0.359 -0.402 -0.829 -0.266 0.203 0.145 1.147 0.423 0.501 0.769 0.463

AI657832 0.158 0.189 0.207 0.398 0.443 0.094 0.144 0.691 0.185 -0.221 -0.206 -0.444

AW078445 -0.426 0.271 0.407 0.896 1.286 -0.01 0.14 1.529 -0.233 -0.064 0.999 0.102

BI878583 -0.481 0.053 0.104 0.547 0.369 -0.154 0.139 0.851 0.031 -0.047 0.409 0.215

AI437239 -0.021 -1.183 -0.31 -0.336 0.16 0.495 0.137 0.794 0.148 0.536 0.326 0.187

AI942960 -1.507 -0.377 -1.227 -0.116 -0.445 0.181 0.129 1.352 1.273 0.969 0.552 -0.224

AI958505 -0.562 -1.321 -1.543 -0.584 -0.434 -0.16 0.123 0.973 0.444 0.536 -0.071 0.151

BF156220 -0.23 0.1 0.252 0.703 0.807 -0.046 0.121 0.845 0.22 0.081 0.073 0.195

AI878627 0.013 0.331 0.228 0.187 0.59 0.194 0.116 1.024 0.251 0.168 -0.156 -0.45

AI588156 -0.327 -0.326 -0.248 0.008 -0.062 -0.252 0.116 1.492 0.267 0.761 0.222 -0.304

AF160683 -0.938 -0.639 -0.579 0.327 0.29 0.437 0.116 0.877 0.4 0.205 -0.148 -0.155

AI437402 -0.165 -0.364 0.127 0.385 0.528 0.418 0.115 0.732 0.042 0.15 0.12 -0.016

AI878787 0.332 0.429 0.124 0.468 0.447 0.014 0.114 0.669 -0.163 0.005 0.107 -0.207

AI641028 0.111 0.253 0.23 0.248 0.103 0.205 0.108 0.848 -0.047 0.141 -0.01 -0.058

AA497147 -0.129 0.013 0.253 0.51 0.286 0.097 0.106 0.586 0.115 0.175 0.448 0.024

AI943392 0.068 -0.144 0.122 0.099 0.474 0.345 0.1 0.991 0.258 0.024 -0.168 -0.295

BI880694 -0.27 -0.141 0.065 -0.013 -0.093 -0.083 0.098 0.623 -0.017 -0.078 0.239 0.178

AI959222 -0.022 0.029 0.034 0.083 0.471 0.182 0.096 0.798 0.064 0.065 0.074 0.374

AW116868 0.237 0.591 0.304 0.268 0.542 0.48 0.096 0.829 0.232 0.368 -0.072 0.055

BM102872 0.207 0.21 0.893 0.293 0.483 0.693 0.095 1.446 0.681 0.428 0.236 0.04

BM095178 -0.112 0.016 -0.015 -0.14 0.352 0.171 0.092 0.885 0.154 -0.005 -0.271 -0.003

AI957914 -0.311 0.217 -0.501 0.353 0.68 0.081 0.092 0.778 -0.034 -0.317 0.002 0.043

BI867946 0.179 -0.387 0.061 0.059 0.296 -0.118 0.092 1.01 0.206 0.468 0.4 -0.2

AI641146 0.398 -0.079 0.539 0.171 -0.065 0.086 0.092 0.738 0.16 0.295 0.112 0.075

AW422922 -0.19 0.227 0.55 0.56 0.542 0.724 0.091 0.686 0.929 0.86 0.764 0.417

BG304255 -0.066 0.101 0.286 0.641 1.051 0.373 0.082 1.34 0.684 0.338 0.093 -0.46

BI890771 0.276 0.736 0.531 0.582 0.446 0.285 0.081 0.893 0.244 0.626 0.545 0.164

BI884186 0.749 -0.589 -0.155 -0.244 0.011 0.492 0.077 0.901 -0.09 0.164 0.316 -0.217

AI641401 -0.728 0.141 0.218 0.819 1.336 0.255 0.075 1.694 -0.378 0.006 0.349 0.119

AI415997 0.073 -0.259 -0.303 0.179 0.277 0.174 0.069 0.648 0.116 0.228 0.081 0.238

AW175480 0.345 -0.109 0.185 0.187 -0.407 0.096 0.067 0.76 0.487 0.601 0.414 0.037

AI721573 -0.708 0 0.34 0.464 0.534 -0.276 0.066 0.809 0.279 0.438 0.588 -0.01

BI866448 0.095 0.187 0.309 0.309 0.682 0.279 0.065 0.91 0.125 0.223 0.033 0.225

AJ286835 -0.492 0.227 0.486 0.832 0.48 0.209 0.063 1.143 0.247 0.35 0.796 -0.004

AW128384 0.108 0.352 0.364 -0.174 0.091 -0.059 0.06 0.767 -0.057 0.067 -0.231 0.071

BG799577 -0.631 0.045 0.034 0.119 0.914 0.084 0.059 1.057 0.763 0.388 0.565 0.135

BI841076 -0.681 0.197 0.33 0.745 0.871 0.254 0.059 1.281 0.29 0.453 -0.033 -0.511

AI667333 0.392 -0.088 0.227 -0.058 0.493 0.402 0.058 0.707 0.183 0.133 -0.005 0.018

BI845814 0.069 0.095 -0.177 0.134 -0.256 0.089 0.047 0.549 0.251 0.205 0.046 0.269

BM101531 -0.318 0.397 0.127 0.201 0.949 -0.039 0.046 1.014 0.273 0.331 0.236 0.204

BM183518 0.1 0.243 -0.014 -0.125 0.167 -0.146 0.046 0.563 -0.139 0.129 0.01 -0.021

BI876163 -2.743 -1.634 0.339 0.339 0.379 0.164 0.043 -0.046 0.84 0.796 0.493 0.366

AI943195 -0.468 -0.635 0 0.199 0.517 0.359 0.043 0.679 0.126 0.256 0.17 0.102

U49408 -0.328 -0.358 -0.316 -0.29 -0.057 0.499 0.038 1.437 1.11 0.717 1.1 0.23

AF348959 -0.604 0.237 -0.677 -0.256 -0.354 0.338 0.029 0.537 0.491 0.317 -0.113 -0.066

BI886387 0.233 0.051 0.434 0.114 0.265 0.056 0.027 0.89 0.439 0.296 0.077 -0.426

AI496761 -0.621 -0.01 -0.239 -0.082 0.45 0.052 0.022 0.919 0.08 0.036 0.055 -0.229

AI878796 -0.451 0.013 -0.399 -0.194 0.083 -0.049 0.017 0.745 0.162 0.188 0.034 -0.057

L27585 -0.148 -0.173 -0.071 -0.033 -0.116 -0.32 0.016 0.667 -0.069 0.26 0.183 0.359

AI721501 -0.452 0.545 -0.058 0.341 0.569 0.14 -0.001 0.836 -0.254 -0.357 -0.059 -0.503

BM186095 0.177 0.138 -0.56 -0.163 -0.294 -0.133 -0.008 0.603 0.135 0.063 -0.064 -0.058

AB055679 -0.016 0.107 0.131 0.493 0.366 0.515 -0.018 0.903 0.448 0.038 0.14 0.085

BI891076 0.3 0.27 -0.037 0.211 0.031 0.105 -0.019 0.816 -0.143 -0.192 -0.315 -0.166

AI584223 -0.486 0.151 0.326 0.339 0.614 0.346 -0.02 0.933 0.381 0.524 0.328 0.23

BI889533 0.048 -0.127 0.298 0.074 -0.053 -0.279 -0.022 0.926 -0.041 0.295 -0.405 -0.383

AW184197 -0.121 0.059 0.254 0.378 0.313 -0.003 -0.033 0.53 -0.219 -0.272 -0.368 -0.109

AI601696 0.227 0.631 0.089 -0.002 0.318 -0.127 -0.037 0.706 0.08 -0.462 -0.14 -0.307

AA606013 -0.728 -0.187 0.145 0.5 0.594 0.433 -0.04 1.119 -0.378 -0.552 -0.543 -0.913

BM156045 -0.618 0.02 -0.055 0.239 0.589 0.056 -0.056 0.624 -0.119 0.085 0.118 0.126

BI709620 0.032 -0.252 -0.603 -0.133 0.059 0.526 -0.067 0.751 0.044 0.553 0.28 0.403

BI981135 -0.642 0.545 0.362 0.61 0.599 0.225 -0.07 1.06 -0.157 0.07 -0.329 0.32

AW777378 0.036 -0.307 -1.174 -0.382 -0.065 0.42 -0.079 0.796 0.208 0.238 0.037 0.365

AI957893 -0.668 0.142 0.229 0.22 0.243 -0.022 -0.082 1.21 0.063 -0.186 0.148 0.129

AF052249 -0.818 -0.338 0.077 -0.194 0.561 0.13 -0.094 0.814 0.194 0.541 0.081 0.074

BI880304 -0.323 0.093 -0.191 0.341 0.123 -0.264 -0.1 0.91 -0.118 0.113 -0.039 -0.355

U89380 -0.604 0.194 -0.116 0.148 -0.026 -0.007 -0.121 0.629 0.317 0.261 0.328 0.011

AI793509 -0.395 0.147 -0.373 -0.013 0.204 0.007 -0.127 1.169 0.592 0.453 0.704 -0.18

BM183249 -0.239 -0.69 -0.482 -0.151 0.087 0.486 -0.127 0.966 -0.378 0.361 -0.543 -0.913

AI641680 -0.825 0 -0.263 0.341 -0.199 -0.148 -0.144 0.789 1.02 0.682 0.456 0.044

X70322 -0.733 -0.057 -0.061 -0.137 -0.059 -0.259 -0.16 1.412 0.16 -0.317 -0.045 -0.371

AI878235 -1.376 -2.631 -0.993 -0.448 -0.506 -0.381 -0.162 -1.336 0.286 -0.643 0.145 -0.122

AF295804 -0.341 0.332 -0.702 0 -0.059 0.071 -0.166 0.728 -0.108 0.454 0.236 0.048

AW116654 0.295 -0.367 -0.503 -0.621 0.42 -0.053 -0.171 0.67 -0.059 0.173 -0.543 -0.408

AI721548 -1.777 -1.616 -0.273 -0.415 0.22 0.126 -0.172 -0.319 0.709 0.074 0.554 0.312

BE556860 0.1 0.477 -0.408 -0.266 -0.365 -0.281 -0.19 0.71 0.141 0.227 0.003 0.032

AF084948 -0.226 -0.469 -0.34 -0.118 -0.031 -0.113 -0.198 0.694 -0.035 0.194 -0.126 -0.093

AW306050 0.124 -0.909 -0.997 -1.829 0.367 0.405 -0.212 0.804 0.226 0.141 -0.63 -0.598

AI522349 -0.728 -0.428 -0.969 -1.548 -0.881 -0.25 -0.274 0.85 0.518 0.233 -0.098 -0.006

BI891804 -2.112 -1.02 -0.426 -0.228 -0.004 -0.159 -0.345 -0.585 0.409 -0.315 0.343 0.283

AI522706 -0.503 -0.335 -0.576 0.09 -0.618 -0.441 -0.364 -0.156 0.76 0.744 0.408 -0.411

X12802 -0.67 -2.849 -2.012 -2.563 -0.37 -0.278 -0.387 -0.018 -1.55 -1.091 -1.142 -0.236

BM185251 -2.041 -1.986 -0.391 -0.447 0.317 -0.132 -0.396 -1.013 0.494 -0.344 0.437 0.42

AA495267 -2.947 -2.885 -0.284 -0.332 0.144 -0.21 -0.444 -0.942 0.653 -0.326 0.395 0.116

BI710295 -2.211 -3.349 -0.592 -0.752 -0.206 -0.391 -0.467 -0.939 0.43 -0.376 0.427 0.18

AI658234 -1.346 -1.949 -1.498 -0.678 -1.042 -0.726 -0.529 0.2 0.974 0.74 0.588 -0.372

AI588340 -2.249 -1.739 -2.15 -0.657 -1.332 -0.6 -0.582 0.446 1.137 1.017 0.799 0.13

AI626451 -0.32 -0.687 0.08 -0.842 0.26 -0.195 -0.607 -0.378 0.51 -0.343 0.468 0.429

AI461372 -2.624 -1.519 -0.695 -0.453 0.061 -0.085 -0.633 -0.366 0.678 -0.228 0.483 0.375

BM184381 -0.688 -1.837 -1.252 -0.738 -1.163 -0.957 -0.642 -0.323 1.195 0.975 1.079 -0.445

BI880007 -2.635 -2.384 -0.794 -0.805 -0.067 -0.291 -0.774 -1.134 0.475 -0.469 0.472 0.251

AI584734 -3.322 -3.041 -0.694 -0.67 0.139 -0.288 -0.813 -1.15 0.762 -0.179 0.518 0.339

AI667396 -3.384 -3.319 -3.531 -2.728 -2.813 -1.5 -1.265 -0.582 0.796 0.417 0.672 0.143

Mean -0.145 -0.138 0.185 0.359 0.642 0.543 0.562 1.215 0.477 0.426 0.25 0.015
