# Supplementary material for: Transcriptome Analysis of Zebrafish Embryogenesis Using Microarrays
Source: PLoS Genet. 2005 Aug 26;1(2):e29. doi: 10.1371/journal.pgen.0010029 (PMC1193535; doi:10.1371/journal.pgen.0010029)
Supplement: Dataset S17 — (156 KB DOC) [file pgen.0010029.sd017.doc]

Dataset S17. Genes exhibiting peak of expression at the pharyngula.

Genbank IDUF egg 3hpf 4.5hpf 6hpf 7.7hpf 9hpf 10.7hpf 12hpf 15hpf 24hpf 30hpf 48hpf

AI883430 -1.335 -0.905 -1.353 -2.157 -1.205 -0.307 -0.838 -0.856 -0.694 2.693 1.963 -0.741

BM181246 -5.052 -4.362 -1.702 -0.922 0.042 1.376 1.551 1.722 1.819 2.433 1.635 0.163

BI896418 -1.978 -1.482 -2.546 -1.598 -1.638 0.279 0.624 1.209 1.117 1.881 0.983 1.089

AF202639 -0.613 -0.003 -0.32 -0.664 -0.238 0.185 -0.287 0.175 0.472 1.872 1.163 0.51

Y08321 -6.291 -6.731 -4.656 -4.12 -4.311 -1.57 -0.998 -0.069 0.394 1.843 1.12 0.034

AF257743 -0.082 -0.921 -1.051 -0.775 -0.778 0.705 0.394 1.372 0.901 1.828 0.974 0.173

AI877922 -4.777 -4.532 -4.199 -3.744 -4.242 -3.224 -3.122 -1.581 -0.243 1.774 1.229 0.918

AI601781 -2.101 -1.46 -2.149 -0.736 -2.274 -0.679 -1.625 -1.358 -0.714 1.746 1.367 0.135

AF149802 -2.361 -2.012 -2.327 -1.351 -2.433 -0.921 -1.253 -1.481 -0.171 1.679 1.269 0.639

AI477274 -2.032 -1.834 -2.202 -0.937 -2.806 -0.883 -0.898 -1.154 -0.095 1.615 0.624 0.823

BI892272 -1.568 -1.744 0.544 1.253 1.566 1.288 1.045 0.142 1.384 1.615 0.99 1.425

AI478002 -0.677 -1.323 -1.58 -0.586 -0.107 -0.392 -0.357 1.118 0.836 1.606 0.535 -0.04

BM183152 -0.646 -0.811 0.948 1.471 1.276 1.907 1.164 1.278 0.508 1.604 1.859 0.663

BI864190 -1.084 -0.7 -1.045 -0.167 -0.089 1.079 0.966 1.528 1.245 1.569 1.186 -0.003

AF155581 0.429 -0.426 0.606 -0.316 1.309 1.134 0.563 1.773 0.732 1.564 0.632 0.468

AW019142 -0.724 0.159 -0.25 0.046 -0.109 0.053 0.466 1.115 0.87 1.555 0.56 -0.099

BM157063 -0.563 -1.812 -1.162 -0.545 -0.27 0.346 0.277 0.886 0.282 1.551 1.254 0.958

AW078048 -0.932 -1.798 -0.404 -0.089 0.267 0.92 0.835 1.043 0.721 1.55 1.148 1.269

L35586 -1.849 -1.375 -2.687 -1.058 -1.502 -1.008 -1.039 -0.424 0.173 1.539 0.226 -0.745

AI397462 -0.728 -0.201 -0.552 -0.216 -0.161 -0.137 -0.366 0.039 0.646 1.536 0.641 0.034

U66872 -0.714 0.442 -0.494 0.358 1.101 0.399 0.536 0.965 0.444 1.535 0.5 -0.017

AA605677 -3.724 -3.317 -0.595 -0.783 -0.731 1.127 0.383 0.401 0.608 1.526 1.438 1.089

AF197880 -4.428 -3.512 -0.999 0.126 0.276 0.993 0.961 0.606 0.849 1.512 0.519 -0.012

AA658796 0.349 -0.742 -0.667 0.168 0.518 1.107 1.077 1.529 0.863 1.476 0.724 0.412

AW777320 -1.1 -1.251 -2.176 -1.766 -2.285 -0.829 -1.21 -1.728 -0.461 1.47 0.849 1.216

AI601365 -2.036 -1.302 -1.991 -2.172 -1.735 -0.879 -1.403 -1.276 -0.216 1.467 1.012 0.26

AI884029 -0.462 -0.524 -1.05 -0.45 -0.463 0.08 -0.152 0.969 0.272 1.447 0.631 -0.498

AF155578 0.313 -0.514 0.39 0.13 0.558 0.931 0.461 1.235 0.989 1.446 0.664 0.47

AY007304 0.939 0.191 0.161 0.524 0.421 0.979 0.664 1.472 1 1.444 0.823 -0.052

BI886392 -0.413 0.217 0.457 0.627 0.378 0.555 0.448 1.431 1.164 1.438 0.746 0.246

AI522421 -0.979 -0.025 0.021 -0.384 0.01 -0.129 -0.526 -0.562 -0.337 1.421 0.496 -0.591

AI957746 -0.868 0.135 -0.267 0.302 0.006 -0.158 -0.472 -0.381 -0.08 1.375 0.733 -0.41

BM155568 -0.257 -1.979 -0.899 -0.654 -0.319 0.668 0.379 0.63 0.512 1.372 1.123 0.449

BG727615 -2.472 -2.218 -2.066 -1.095 -1.503 -0.435 0.193 0.359 0.435 1.371 0.848 0.289

BI982718 -0.198 -1.867 -0.564 -0.64 -0.364 0.475 0.295 0.742 0.324 1.369 1.053 0.211

AI106421 -0.405 -2.679 -0.973 -0.895 -0.423 0.048 0.4 0.622 0.317 1.361 0.739 0.218

AW595789 -0.21 -1.286 -0.297 -0.382 -0.204 0.914 0.48 0.762 0.761 1.357 0.84 -0.115

AW343989 -1.334 -1.42 -0.581 -0.238 0.284 0.058 0.042 0.834 1.027 1.354 0.637 0.215

BI889566 -0.419 -0.85 -0.027 -0.362 -0.602 -0.247 0.121 1.031 0.616 1.344 1.312 0.118

BI889370 -1.505 -1.791 -1.555 -0.444 0.061 0.712 0.594 1.074 0.752 1.344 0.869 -0.617

BM182911 -2.587 -1.889 -2.749 -1.759 -1.38 -0.522 -0.083 0.379 0.235 1.343 0.634 0.19

AF195881 -0.784 -0.034 0.315 0.422 0.145 -0.023 0.055 0.351 0.652 1.341 0.448 0.214

BG884107 -1.586 -0.292 -0.458 -0.057 0.501 1.295 0.719 0.735 0.428 1.33 0.827 1.131

BE693153 0.499 -0.348 0.179 0.296 0.453 0.958 0.557 1.242 0.772 1.309 0.608 0.031

U93458 -1.236 -1.746 -1.256 -1.114 -0.746 -0.326 -0.024 0.816 1.083 1.308 0.914 0.596

AW777561 -0.751 0.344 -0.508 0.027 -0.076 -0.055 -0.357 0.476 0.626 1.305 0.412 -0.147

BI325825 -0.91 -2.913 -1.058 -0.825 -0.396 0.65 0.253 0.413 0.465 1.3 0.914 0.265

AA606010 1.15 -0.39 -0.253 -0.035 -0.155 0.535 0.69 0.879 0.934 1.298 0.572 0.18

AI353394 0.508 -0.983 -0.048 0.133 0.391 1.198 0.557 1.057 0.416 1.295 0.863 1.017

BM104738 -0.511 -2.579 -1.188 -1.129 -0.491 0.62 0.2 0.667 0.375 1.291 0.891 0.217

AW077063 -0.056 -1.141 -1.24 -0.326 -0.3 0.329 0.276 0.482 0.571 1.29 0.937 0.332

BI887231 -0.545 -2.001 -0.8 -0.67 -0.408 0.642 0.297 0.504 0.377 1.288 1.08 0.462

BI882972 -1.1 -2.183 -1.072 -0.797 -0.575 0.589 0.221 0.361 0.406 1.287 1.086 0.645

BI839952 -0.362 -0.75 -0.205 0.339 -0.353 -0.163 0.399 0.406 0.558 1.287 0.729 0.287

AI477493 0.18 -0.213 0.028 0.063 0.196 0.419 0.802 1.331 0.691 1.281 0.609 0.005

BM181817 -0.262 -0.242 0.426 0.53 0.386 1.329 0.468 0.735 0.642 1.276 0.831 0.189

AF134850 -6.382 -5.38 -1.241 -0.434 -0.066 0.822 0.451 0.66 0.58 1.266 0.871 0.136

AF029250 -0.548 -0.766 -1.42 -1.819 -2.053 -0.817 -1.346 -0.756 -0.544 1.261 1.026 1.119

BI896504 -0.896 -2.379 -1.221 -0.962 -0.551 0.671 0.328 0.516 0.381 1.259 0.912 0.405

AW058797 0.257 -0.004 0.647 0.722 1.008 0.826 0.423 1.33 0.672 1.259 0.33 0.363

AI522447 -1.414 -0.532 -1.15 -0.611 -0.559 0.454 0.529 1.046 0.344 1.242 1.29 1.055

BG728552 -1.205 -0.451 -1.385 -0.15 -0.187 -0.316 -0.047 0.299 1.104 1.242 0.601 0.369

AW117056 -1.271 -1.706 -1.154 -1.412 -1.451 0.151 0.257 0.997 1.223 1.241 0.872 -1.13

BG985680 -0.446 -0.434 1.023 1.189 0.533 0.861 0.561 0.786 0.649 1.24 1.329 0.321

BI879038 -0.813 -0.971 -0.863 -0.06 0.584 1.263 0.756 1.253 0.583 1.237 0.858 0.725

AW567098 -0.6 -0.207 -0.045 0.895 1.161 1.016 0.767 1.139 0.346 1.237 0.84 0.235

AI601793 -1.177 -1.514 -1.009 -1.502 -2.075 -0.573 -0.092 -0.039 0.422 1.235 0.755 -0.048

BI710147 -0.552 -2.654 -0.952 -1.001 -0.428 0.616 0.315 0.443 0.249 1.234 0.854 0.227

BI886388 -1.219 -0.599 -0.253 0.177 0.745 1.142 1.019 1.107 0.36 1.232 0.98 1.37

BI890906 -0.854 -2.499 -0.93 -0.976 -0.607 -0.024 0.213 0.32 0.139 1.214 0.734 0.286

BE201151 -0.659 -0.26 -0.538 -0.086 0.038 0.066 -0.003 0.817 0.371 1.208 0.578 -0.458

BI891434 -0.854 -1.878 -0.893 -0.836 -0.581 0.393 0.187 0.493 0.308 1.204 0.915 0.388

BM159128 -0.54 -2.462 -0.958 -1.048 -0.505 0.555 0.163 0.451 0.153 1.202 0.83 0.313

BI982030 0.113 0.211 -0.6 -0.241 0.034 0.877 0.399 0.846 0.739 1.2 0.643 0.558

BI672508 -0.783 -3.033 -1.339 -1.012 -0.677 0.484 0.127 0.473 0.401 1.198 0.868 0.25

Y13653 -5.37 -5.897 -4.748 -3.724 -2.815 -0.626 -0.576 -0.466 -0.078 1.198 1.081 0.949

AW203148 -0.973 -2.026 -1.075 -0.416 -0.234 0.492 0.46 0.634 0.523 1.189 0.95 0.528

AI106133 -0.516 -1.723 -0.777 -0.373 -0.788 0.497 0.273 0.7 0.296 1.187 0.581 -0.44

BI842921 -0.811 -2.677 -1.475 -1.214 -0.899 0.445 0.084 0.342 0.212 1.18 0.723 0.355

BI886029 -1.442 -1.297 -0.919 -0.611 -0.257 0.461 0.146 0.532 0.394 1.176 0.762 0.686

AI883262 -0.925 -0.179 -0.666 -0.121 -0.646 -0.25 -0.426 -0.169 0.01 1.172 0.89 0.371

AI558451 -0.775 -2.631 -1.15 -0.979 -0.572 0.505 0.165 0.356 0.32 1.172 0.919 0.344

AW115602 0.466 -2.087 -0.839 0.005 0.057 0.807 0.364 0.572 0.579 1.171 0.673 0.673

AI964264 -1.151 -2.091 -1.282 -1.84 -1.095 0.798 0.824 0.897 0.45 1.169 0.719 0.311

AF068773 -1.865 -1.741 -1.225 -0.055 -0.325 -0.073 0.072 0.165 0.198 1.167 0.202 -0.258

AJ245490 -0.638 -0.866 -1.139 -1.351 -0.244 -0.328 -0.398 0.7 0.443 1.163 0.828 0.197

AI959750 -0.863 -2.434 -1.026 -0.776 -0.33 0.681 0.351 0.673 0.311 1.163 0.796 0.462

BI979883 0.493 0.208 0.8 0.961 0.405 0.757 0.58 1.203 0.784 1.162 0.668 0.254

BM070699 -0.087 -2.361 -1.068 -0.649 -0.569 0.561 0.153 0.48 0.383 1.152 0.897 0.251

BM183474 -1.163 -2.775 -1.341 -0.84 -0.581 0.496 0.215 0.389 0.337 1.15 0.855 0.296

AI384355 -1.279 -3.67 -1.778 -1.335 -0.982 0.264 -0.028 0.217 0.234 1.146 0.997 0.597

X70300 -0.97 -0.821 -0.583 -0.154 -0.295 -0.247 0.227 1.08 0.979 1.143 0.954 0.102

BI886290 0.084 -0.391 -0.381 0.344 0.325 0.611 0.379 0.925 0.429 1.142 0.638 0.25

AW154782 -1.778 -0.775 -1.734 -0.781 -0.494 -0.023 0.258 0.696 0.657 1.141 0.584 0.313

AI722592 -0.538 -1.219 -0.539 -0.498 -0.266 0.573 0.348 0.444 0.251 1.137 0.826 0.239

AI957628 -1.834 -1.738 -1.437 -1.382 -1.926 -0.757 -0.716 0.819 0.934 1.133 0.968 0.351

AI884082 -0.675 -0.73 -1.096 -0.71 -0.271 1.196 0.913 1.137 0.977 1.132 0.594 0.125

BI325085 -1.339 -1.656 -0.812 -0.741 -1.008 -0.257 -0.282 -0.012 0.552 1.131 1.106 0.503

BI896233 -1.099 -1.381 -1.669 -1.194 -1.558 -0.638 -1.261 -1.456 -0.61 1.129 1.034 0.809

AI496943 -1.23 -0.709 -0.039 0.199 0.472 1.339 1.013 1.592 1.061 1.124 1.151 1.58

AI793467 -0.764 -1.463 -1.138 -1.435 -2.131 -0.337 -1.379 -1.269 -0.665 1.122 0.881 0.275

AW116780 -1.142 -0.48 -0.991 -0.811 -0.835 -0.716 -1.04 -1.141 -0.994 1.119 0.401 -0.205

BG985722 -1.335 -0.4 -1.024 -1.283 -1.091 -0.341 -0.809 -0.505 -0.018 1.117 1.059 1.44

AI964216 -1.265 -2.28 -1.221 -0.904 -0.57 0.389 0.046 0.422 0.469 1.115 0.904 0.395

AW455045 -2.069 -1.071 -2.143 0.05 -1.339 -0.448 -0.834 0.347 0.521 1.114 0.45 0.282

AI657670 -0.458 -0.325 -0.061 -0.169 -0.727 -0.039 -0.808 -0.158 -0.169 1.113 0.607 -0.049

BI842900 -0.559 -3.581 -1.423 -1.184 -0.556 0.15 0.087 0.471 0.181 1.112 0.699 0.002

AI477041 -0.327 -2.059 -0.292 0.216 0.284 0.507 0.696 1.021 0.699 1.109 0.578 -0.317

AA606173 -0.869 -3.327 -1.503 -1.121 -0.847 0.487 0.23 0.29 0.372 1.104 0.84 0.393

BM186976 -1.623 -1.194 -1.556 -1.064 -1.519 -0.459 -0.539 0.105 0.376 1.103 0.567 0.221

AW077286 -0.713 -2.383 -1.037 -0.819 -0.578 0.427 0.207 0.088 0.387 1.103 0.776 0.126

BE202178 -0.455 -0.141 0.193 0.763 0.568 0.544 0.551 0.972 0.549 1.096 0.624 0.693

X97330 -0.077 0.171 -0.234 -0.088 -0.21 -0.334 0.121 1.176 0.519 1.096 0.642 0.21

BE017895 -1.018 -2.981 -1.196 -1.049 -0.586 0.427 0.113 0.434 0.204 1.092 0.592 0.25

BI891040 -0.575 -2.54 -1.146 -0.869 -0.684 -0.021 -0.001 0.187 0.044 1.09 0.722 0.339

AW232020 -2.569 -2.335 -3.261 -2.127 -2.903 -0.649 -1.642 -2.548 -0.871 1.088 0.764 0.95

BI891138 -0.667 -3.238 -1.973 -0.918 -0.846 0.381 0.072 0.294 0.392 1.085 0.723 0.102

AF102865 -2.487 -2.252 -1.72 -1.622 -1.635 -1.096 -0.397 -0.975 -1.315 1.083 0.289 0.515

AI558833 -0.853 -3.19 -1.354 -1.176 -0.748 0.48 0.001 0.246 0.239 1.075 0.661 0.196

U93459 -1.054 -1.831 -1.001 -0.746 -0.356 -0.204 0.327 0.336 0.917 1.073 0.784 0.513

BG302711 0.091 -0.646 -1.555 -0.311 -1.013 0.072 -0.163 0.217 0.36 1.07 0.67 0.596

AF134852 -1.041 -4.135 -2.07 -1.404 -1.052 0.269 -0.205 0.105 0.178 1.069 0.826 0.299

BI887366 -0.872 -2.986 -1.682 -1.209 -0.679 0.346 0.225 0.54 0.379 1.067 0.699 0.098

BI881430 -0.305 -1.265 -0.28 -0.387 -0.357 0.654 0.221 0.364 0.285 1.066 0.611 0.208

BI890861 -1.181 -1.99 -0.937 -1.081 -0.49 0.335 0.101 0.423 0.198 1.064 0.826 0.374

BG303602 0.27 -0.154 0.072 -0.094 -0.309 0.718 0.213 0.65 0.471 1.063 -0.012 -0.461

BI707410 -0.587 -3.041 -1.196 -0.901 -0.492 0.486 0.33 0.498 0.231 1.061 0.627 0.072

AI721634 0.177 -0.407 0.097 0.219 -0.094 0.312 0.459 0.857 0.812 1.055 0.212 -0.623

X97332 -1.003 -1.133 -0.652 -0.4 -1.48 -0.429 -0.069 0.154 -0.08 1.053 1.263 1.022

BI888186 0.454 -0.156 0.032 0.179 0.473 0.856 0.506 1.096 0.781 1.052 0.528 0.468

AI958585 0.108 -0.186 -0.773 -0.413 -0.211 0.974 0.61 0.998 0.358 1.049 0.399 0.229

BI885026 -0.948 -0.04 -0.415 -0.659 -0.003 0.263 0.433 1.042 0.447 1.046 -0.079 -0.682

AI722464 -0.906 -1.086 -1.397 -1.71 -1.401 -0.259 -0.757 -1.252 -0.156 1.04 0.971 0.994

AI667290 -1.257 0.384 -0.379 0.072 0.206 0.906 0.276 0.588 0.71 1.038 1.216 0.817

BI897492 0.265 0.189 0.567 0.529 0.333 0.648 0.396 0.855 0.599 1.038 0.562 0.416

BI886825 -0.096 -1.845 -0.856 -0.698 -0.475 0.628 0.142 0.326 0.307 1.037 0.726 0.2

BI983379 0.159 -1.579 -0.742 -0.456 -0.52 0.342 0.193 0.212 0.116 1.037 0.615 0.161

AF025330 -1.404 -1.682 -0.85 -0.588 -0.842 -0.235 -0.487 0.207 0.375 1.035 0.821 0.383

BM153976 -0.481 -3.354 -1.545 -0.985 -0.782 0.432 0.108 0.438 0.26 1.035 0.651 0.172

AI878068 -1.035 -2.87 -1.539 -1.093 -0.572 0.381 0.111 0.427 0.27 1.034 0.561 0.176

AW019428 0.858 -0.408 -0.085 -0.179 0.143 0.441 0.384 1.001 0.571 1.033 0.444 0.054

BM183623 -0.533 -3.251 -1.514 -1.026 -0.687 0.337 0.142 0.162 0.273 1.032 0.632 -0.118

AW115626 0.923 -0.746 -0.58 -0.225 -0.494 0.863 0.555 0.985 0.835 1.029 0.351 -0.409

AI658011 -2.105 -2.831 -2.338 -2.415 -2.821 -1.4 -1.731 -2.48 -1.406 1.029 0.832 1.501

AW128382 -0.528 -0.464 -1.765 -0.396 -0.415 -0.084 0.178 0.665 0.51 1.028 0.294 0.28

AW128744 -1.007 -2.859 -1.569 -1.22 -0.937 0.313 0.035 0.282 0.202 1.024 0.813 0.298

AF127981 -0.958 -0.046 -0.805 -0.918 -0.366 0.211 -0.069 0.373 -0.399 1.024 1.016 0.868

AI878761 -0.626 -0.932 -0.926 -0.819 -0.266 -0.094 -0.332 -0.207 -0.195 1.017 0.692 0.806

AI588172 0.325 -0.006 -0.097 0.163 -0.616 0.172 -0.283 0.27 0.291 1.015 0.589 0.091

BF718229 -1.24 -2.836 -1.569 -1.154 -0.781 0.263 0.076 0.29 0.402 1.01 0.687 0.174

BM181739 -0.461 -0.193 0.016 -0.78 -0.472 -0.525 -0.172 0.968 0.418 1.007 0.62 -0.323

BI888812 -0.992 -2.583 -1.804 -1.041 -0.646 0.337 0.228 0.293 0.263 1.001 0.661 0.341

BE693133 -0.053 -0.372 0.235 0.366 0.509 0.623 0.589 1.078 0.597 0.997 0.23 0.319

AA606085 -0.434 -0.477 0.323 0.384 0.229 0.079 0.359 0.872 0.364 0.994 0.275 0.351

AI588758 0.45 -0.119 -0.517 -0.112 -0.197 0.256 0.173 0.919 0.538 0.994 0.268 -0.119

BI890218 -0.725 -2.578 -1.236 -1.145 -0.79 0.059 0.127 0.301 0.015 0.992 0.613 0.291

AF036149 -0.543 -0.131 -0.273 -0.669 -0.066 0.044 0.297 1.182 0.359 0.991 1.196 0.855

BI892430 -0.446 -2.368 -0.921 -0.77 -0.351 0.508 0.28 0.356 0.181 0.988 0.587 0.065

AI957415 -2.72 -2.669 -2.664 -2.652 -0.691 0.493 0.659 0.985 0.942 0.983 0.551 0.254

AY007434 -3.23 -1.089 0.313 0.389 0.479 1.025 0.255 0.584 0.6 0.98 0.986 0.608

BG304333 -0.224 -1.104 -0.552 -0.631 -0.127 0.743 0.279 0.902 0.659 0.979 0.675 0.276

BM081047 -0.507 -1.418 -1.065 -0.7 -0.595 -0.076 0.04 0.114 0.274 0.978 0.551 -0.143

BI880392 -1.024 -1.346 -0.976 -0.708 -0.872 0.022 0.498 0.34 0.667 0.97 0.838 0.348

AI957504 -0.998 -1.175 -1.491 -0.812 -1.036 -0.75 -0.903 -1.276 -0.798 0.965 0.435 -0.245

BI878456 -0.193 -0.718 -0.733 -0.473 -0.744 -0.133 0.192 0.166 0.158 0.964 0.56 0.556

AF175294 -1.885 -0.783 -1.672 -0.669 -0.841 -0.191 0.083 -0.462 0.345 0.961 0.941 -0.081

BE693194 -0.486 0.044 -0.373 0.538 0.416 0.777 0.297 1.228 0.48 0.959 0.416 1.229

AI964218 -1.063 -2.966 -1.49 -1.1 -0.802 0.384 0.038 0.24 0.18 0.958 0.635 0.085

AI545576 0.576 -0.05 -0.632 0.102 0.507 0.163 0.454 0.953 0.767 0.956 0.692 -0.193

BI886167 0.108 -2.879 -1.573 -1.301 -1.091 0.126 -0.17 0.157 0.12 0.956 0.595 0.143

BI318094 -0.654 -1.773 -1.007 -0.343 -0.466 0.407 0.197 0.292 0.131 0.953 0.497 0.204

AI558502 -0.434 -1.186 -0.728 0.067 0.023 0.696 0.934 0.884 0.698 0.948 0.424 0.6

BM024216 -0.087 -0.775 -0.118 0.3 0.266 0.288 0.618 0.901 0.804 0.946 0.452 0.033

BI896246 -1.362 -2.14 -2.257 -1.651 -1.44 -0.442 0.131 0.4 0.193 0.946 0.858 0.117

BI888934 -0.783 -0.551 -1.144 -0.24 0.533 0.361 0.283 1.302 0.769 0.944 0.612 1.509

BI672301 -0.881 -0.728 0.197 0.229 0.258 0.355 0.448 0.755 0.374 0.944 0.543 0.096

BM182744 -0.595 -1.724 -0.008 -0.143 -1.112 -0.347 -0.046 -0.802 -0.112 0.943 0.97 0.147

AW077142 -1.016 -2.732 -0.928 -0.065 -0.137 0.746 0.33 0.531 0.387 0.943 0.497 -0.203

BM071290 -0.511 0.27 -0.122 -0.244 -0.725 -0.299 -0.344 0.321 0.262 0.939 0.862 0.359

BI672150 -0.368 -0.524 0.397 0.384 0.26 0.432 0.402 0.652 0.362 0.936 0.291 0.416

BI889621 -0.953 -0.862 0.165 0.352 0.615 0.428 0.005 0.555 0.081 0.935 0.438 0.832

AI964322 -0.514 -1.284 -1.037 -0.286 -0.249 0.4 0.268 0.878 0.348 0.928 0.213 0.253

AW202947 -1.019 -0.323 -1.246 -0.985 -1.012 -0.166 -0.531 -0.875 -0.295 0.927 0.532 0.569

BM183980 -0.762 -1.606 -0.741 -0.245 -0.617 0.327 0.201 0.274 0.219 0.927 0.759 0.144

AA658759 -2.666 -4.094 -2.319 -1.724 -1.067 -0.272 -0.21 0.079 0.109 0.924 0.728 0.332

AF095639 -0.623 -0.384 -0.403 -0.285 -0.165 -0.238 -0.213 0.233 -0.09 0.92 1.242 -0.005

BI877740 0.669 -0.11 -0.387 -0.156 -0.955 -0.327 -0.236 -0.097 0.079 0.92 0.146 -0.792

BM156982 0.166 -0.269 -0.822 -0.454 -0.753 -0.33 -0.241 0.559 0.399 0.92 0.891 0.36

AF150107 0.675 0.144 0.212 0.564 0.589 0.839 0.138 0.926 0.075 0.919 0.297 -0.073

AF315945 -0.717 -1.069 -1.43 -1.13 -0.521 -0.314 0.202 0.504 0.169 0.919 0.605 0.569

BG799326 -1.094 -0.934 -0.305 0.084 -0.084 0.686 0.655 0.419 0.634 0.918 0.918 0.623

BI889445 -0.734 -2.851 -1.29 -0.81 -0.641 0.539 0.088 0.326 0.15 0.918 0.621 0.236

AW777836 -1.107 -1.465 -1.334 -0.982 -1.213 -0.543 -0.044 0.416 0.685 0.914 1.129 0.5

BG303560 0.085 -2.986 -0.314 -0.492 0.083 0.825 0.377 0.9 0.29 0.912 0.545 0.263

AI965225 0.385 0.1 0.366 0.443 0.177 0.728 0.128 0.679 0.557 0.91 0.227 0.078

AI331055 -0.388 -0.482 -0.354 -0.336 -0.383 0.581 -0.022 0.659 0.334 0.907 0.43 0.36

AI883679 -0.337 -0.382 -0.552 -0.278 -0.355 0.422 -0.127 0.393 0.132 0.905 0.493 0.389

BG303497 0.129 -0.702 -0.418 0.001 0.112 -0.077 0.426 0.797 0.452 0.905 0.359 -0.293

BI890023 -0.393 -0.456 -0.051 -0.108 0.025 0.613 0.505 0.776 0.54 0.901 0.515 -0.18

AI545318 -0.295 -0.544 -0.651 0.093 0.443 0.915 0.425 0.979 0.319 0.901 0.372 0.478

BI981429 -0.165 -1.342 -1.079 0.012 -0.126 0.05 0.472 0.7 0.524 0.901 0.585 0.451

BI890954 -0.337 -0.396 -0.422 -0.401 -0.88 -0.252 -0.229 -0.076 0.085 0.9 0.815 0.653

AI584446 -0.878 -1.711 -0.404 -0.379 0.365 0.351 0.291 0.907 0.283 0.9 0.152 0.439

AJ299411 -0.186 0.183 0.007 0.103 -0.685 -0.563 -0.478 -0.525 -0.5 0.896 0.641 -0.304

AW127801 0.314 -0.316 -0.121 0.056 -0.103 0.978 0.378 0.669 0.864 0.894 0.486 -0.162

AI793901 -2.554 -1.255 -2.495 -1.142 -2.193 -1.29 -1.81 -1.537 -0.729 0.894 0.707 0.66

BI430340 -0.091 -0.961 -0.802 -0.365 -0.002 0.605 0.326 0.69 0.242 0.893 0.566 0.626

BM156154 -1.247 -2.881 -1.119 -0.826 -0.929 0.237 -0.092 0.007 0.132 0.892 0.55 0.054

BG985673 -1.717 -3.699 -2.59 -1.413 -2.097 -1.158 -0.793 -1.653 -0.601 0.891 0.598 0.537

AA497144 -0.845 -0.103 -0.057 0.032 -0.285 -0.131 -0.579 -0.558 -0.31 0.891 0.921 0.352

AW128244 -0.854 -0.754 -0.244 0.072 -0.318 0.052 0.358 0.277 0.225 0.888 0.697 0.756

BI472743 -0.839 -3.492 -1.865 -1.411 -1.021 0.326 0.016 0.27 0.204 0.885 0.611 -0.089

BM071941 0.111 0.051 -0.476 0.014 0.091 1.016 0.491 0.75 0.388 0.882 0.348 -0.128

AI353168 -1.17 -3.123 -1.685 -0.944 -0.667 0.354 0.063 0.253 0.299 0.877 0.585 -0.313

AI965042 -0.077 -0.682 -0.696 -0.065 0.064 0.84 0.516 0.696 0.432 0.873 0.289 0.191

BI890113 -1.149 -2.935 -1.558 -1.169 -0.93 0.223 0.063 0.176 0.192 0.871 0.486 -0.044

BI865754 -0.415 -1.431 0.346 0.38 0.672 0.692 0.566 0.398 0.439 0.871 0.826 1.202

BG306138 -0.777 -0.608 -0.966 -0.466 0.111 0.454 0.42 0.834 0.532 0.871 0.952 1.011

AF157560 -0.912 -0.376 -0.576 -0.04 -0.673 -0.25 0.118 -0.272 -0.12 0.869 0.852 0.419

BF717296 -0.574 -2.459 -1.426 -1.099 -0.962 0.169 -0.069 0.034 0.165 0.868 0.488 -0.02

AW019450 -0.462 -0.461 0.982 0.437 0.053 0.283 0.307 0.902 0.588 0.868 0.439 0.425

BI889529 -4.057 -4.534 -2.887 -2.368 -2.081 -0.716 -1.026 -0.761 -0.06 0.867 0.685 0.452

AI588301 -0.394 -0.098 -0.49 -0.059 -0.337 -0.236 0.004 0.458 0.122 0.867 0.261 -0.124

AI722349 -0.764 -0.031 -0.62 -0.738 -0.003 -0.013 0.06 0.554 0.57 0.867 1.085 0.237

AI658017 0.117 -0.431 -0.108 -0.129 0.069 0.093 -0.129 0.543 0.42 0.86 0.51 0.251

BF157490 -1.959 -0.387 0.678 0.703 0.25 0.005 -0.188 0.268 0.789 0.857 0.313 0.217

BE557659 0.146 0.119 0.209 0.521 0.239 0.274 0.172 0.952 0.346 0.857 0.353 0.145

AI878458 -0.804 -0.282 -0.936 -0.193 -0.797 -0.23 -0.015 0.145 0.269 0.854 -0.033 0.251

BM181646 0.413 0.477 0.041 0.066 -0.753 -0.066 -0.375 -0.337 0.003 0.853 0.697 -0.422

BI883698 -0.382 -0.403 0.133 0.325 0.695 0.815 0.401 0.892 0.622 0.853 -0.11 0.124

AI965224 -0.778 0.545 0.045 0.341 0.173 -0.046 -0.563 -0.008 0.011 0.853 1.139 1.169

AF210641 -0.601 -1.358 -0.484 -0.13 -0.566 0.468 0.042 0.312 0.07 0.852 0.697 0.087

AI957875 -0.454 -0.784 -0.343 -0.241 -0.533 0.186 0.434 0.898 0.81 0.852 0.611 -0.295

BG305988 -0.94 -3.359 -1.396 -1.333 -0.816 0.51 0.114 0.176 0.032 0.85 0.589 0.252

AI641664 -1.452 -1.617 -2.646 -2.268 -2.03 -1.118 -1.655 -1.529 -1.812 0.849 0.719 0.356

AW128379 -1.503 -0.336 -1.991 -0.74 -2.033 -0.576 -0.909 -1.605 -1.566 0.848 0.675 0.909

AF101266 0.902 -0.047 -0.45 -0.333 -0.682 0.249 -0.224 0.984 0.732 0.848 1.17 0.109

BG306111 -0.962 -2.195 -1.69 -1.341 -0.632 -0.114 0.142 0.428 -0.117 0.846 0.483 0.263

AW777364 -0.072 -0.774 -0.934 -0.29 -0.193 0.462 0.318 0.754 0.335 0.843 0.082 0.333

AI958567 -2.103 -1.031 -2.17 -0.444 -1.285 -0.708 -1.107 -1.642 -1.779 0.84 0.382 1.396

AI964239 -1.077 -3.921 -2.01 -1.342 -0.951 0.276 0.098 0.198 0.217 0.835 0.545 -0.094

AW059046 -0.538 -0.237 0.072 0.478 0.647 0.491 0.194 0.961 0.279 0.83 0.348 0.21

AI721504 -0.653 -0.005 -0.296 -0.503 -0.058 0.011 -0.45 -0.221 0.107 0.829 1.237 0.601

BI886648 -0.401 -0.331 0.122 0.294 0.713 -0.234 0.319 0.946 0.28 0.825 1.742 0.166

BI886794 -0.224 -0.301 -0.043 0.329 0.084 0.351 -0.012 0.964 0.67 0.821 0.625 0.304

S80986 -2.376 -1.161 -1.912 -1.309 -2.06 -0.93 -1.115 -0.798 -0.318 0.82 0.636 0.982

BG985441 0.1 -1.247 -0.382 -0.574 -0.825 0.298 0.332 0.345 0.725 0.82 0.969 0.606

BG303237 -2.013 -2.195 -2.393 -1.643 -1.457 -1.21 -1.649 -1.447 -1.254 0.818 0.75 0.479

BM182739 0.157 -0.057 0.003 -0.12 -0.103 0.009 0.251 0.442 0.288 0.817 -0.2 -0.078

BI534059 -0.754 -1.263 -0.584 -0.022 0.18 0.589 0.284 0.751 0.354 0.814 0.498 0.194

AF448057 -0.219 -0.907 -0.76 -0.383 -2.167 -0.98 -1.017 -2.255 -0.785 0.807 0.832 0.182

AW174544 0.217 -0.882 -0.416 -0.291 -0.411 0.522 0.033 0.571 0.138 0.804 0.596 0.276

AI667546 -2.11 -0.978 -1.102 -0.883 -0.985 -0.513 -0.573 -0.122 0.356 0.803 0.768 0.873

BM181750 -1.133 -2.179 -0.998 -0.665 -1.136 -0.425 -0.232 -0.612 -0.346 0.801 0.876 0.184

AI584292 0.196 -0.045 0.188 0.406 0.206 0.356 0.495 0.752 0.131 0.798 0.459 0.165

AI545321 -0.405 -0.149 -0.362 -0.273 -0.473 0.142 0.261 1.085 0.862 0.797 0.94 -0.03

BG728452 -0.464 -2.34 -1.934 -1.535 -0.892 0.171 -0.212 0.31 0.022 0.797 0.316 -0.067

AW567292 -0.627 0.157 -0.008 0.259 0.294 0.015 -0.209 0.375 0.101 0.795 0.96 0.636

BM024150 -0.728 -0.193 -0.153 -0.574 -0.83 -0.137 -0.193 0.637 0.338 0.794 1.079 0.533

BM025541 -0.387 -1.141 0.066 0.303 -1.97 -0.468 0.01 0.63 0.599 0.793 0.499 0.075

AF250368 -0.451 -1.192 -1.136 -0.614 -0.451 0.162 -0.055 0.562 0 0.793 0.522 0.24

AI722432 -1.451 -0.382 -1.165 -0.655 -0.826 -0.443 -0.396 -0.681 -0.318 0.792 0.012 -0.404

AW117039 0.662 0.307 0.668 0.286 0.739 0.316 0.406 -0.015 0.353 0.789 0.791 0.787

AW305460 0.065 -0.668 -0.675 -0.483 -0.637 0.302 0.467 0.294 0.448 0.788 0.699 0.468

BI706468 -0.031 -0.679 -0.415 -0.615 0.198 0.437 0.464 0.791 0.357 0.786 0.29 0.084

AJ278244 0.02 -0.043 0.438 0.458 0.774 0.423 -0.084 0.201 0.756 0.786 0.87 0.863

BI891769 -0.88 -2.729 -1.832 -1.288 -0.963 0.421 0.146 0.206 0.299 0.784 0.576 0.042

AF180921 -0.519 -2.559 -1.147 -1.693 -2.319 0.183 0.319 0.716 0.576 0.779 -0.157 -1.305

BI882313 -0.917 -1.832 -0.762 -1.084 -2.069 -0.466 -0.489 -1.611 -0.627 0.776 0.757 0.428

BI896473 0.104 -0.597 0.284 0.22 0.101 -0.224 0.19 0.461 0.32 0.775 0.596 -0.011

BI889982 -0.03 -0.499 -0.452 -0.136 0.357 0.113 0.151 0.884 0.027 0.77 0.34 0.448

AF124095 -1.012 -0.48 -0.755 -0.016 -0.305 -0.028 -0.306 0.271 0.352 0.77 0.74 0.359

BI891041 -0.203 -0.189 0.415 0.27 0.586 0.249 0.218 0.673 0.345 0.768 -0.054 -0.001

AI721534 -0.236 -0.683 -1.504 -1.349 -1.148 -0.39 -0.268 0.692 0.392 0.767 0.299 0.284

BI877998 0.245 -0.314 -0.33 0.244 -0.039 0.256 0.258 0.645 0.31 0.766 0.316 0.381

BM005469 -0.258 -0.587 -1.047 -0.043 0.128 0.295 0.038 0.637 0.098 0.766 0.496 0.333

BI887737 0.197 -0.312 -0.937 -0.397 -0.238 0.681 0.148 0.548 0.421 0.765 0.179 0.198

BM095392 0.094 0.029 -0.896 -0.481 -0.764 -0.064 -0.424 -0.148 -0.296 0.764 0.453 0.3

AW281444 -0.849 -0.423 -0.301 -0.229 -0.044 0.496 0.035 0.745 0.381 0.76 0.22 -0.334

AJ245491 -5.149 -5.056 -3.874 -3.209 -2.408 -0.59 -0.413 -0.294 0.033 0.759 0.643 0.604

AI793600 -0.057 -1.383 -1.53 -0.068 -0.102 0.679 0.144 0.543 0.674 0.759 0.545 0.256

AW233688 -0.357 -1.439 -1.201 -1.372 -1.137 -0.106 -0.315 -0.264 -0.241 0.754 0.344 0.581

AI601848 -0.914 -1.341 -0.284 -0.322 0.17 0.262 0.102 0.542 0.578 0.753 0.338 0.101

AI657777 -0.662 -0.891 -0.183 -0.73 -0.205 0.076 0.02 0.622 0.124 0.746 1.055 0.396

BI892074 -1.096 -1.388 -1.078 -0.567 -0.569 -0.198 -0.292 -0.064 0.108 0.743 0.574 0.799

AI444293 -0.397 -0.351 0.39 0.397 -0.069 0.651 0.29 0.393 0.312 0.742 0.345 0.453

AI588515 -0.859 -0.582 -0.687 -0.003 -0.214 0.456 0.087 0.188 0.084 0.741 0.391 0.265

AI545450 -0.645 -0.367 -0.76 0.09 0.233 0.523 0.243 0.647 0.533 0.741 0.492 0.019

BI866952 0.454 0.427 0.565 0.435 -0.452 -0.457 -0.39 -0.476 -0.127 0.738 0.87 -0.239

AW116980 -0.046 -0.416 -0.388 -0.107 0.398 0.269 0.369 0.736 0.075 0.735 0.108 0.187

BI888897 -1.229 -2.824 -1.785 -1.181 -0.974 0.193 -0.091 0.092 0.039 0.735 0.348 -0.092

AW420720 -1.617 -1.541 -1.361 -1.208 -1.811 -0.647 -0.423 -0.63 -0.126 0.734 0.584 -0.002

AW232975 -0.373 -2.874 -0.874 -0.479 -0.155 0.745 0.639 0.615 0.359 0.734 0.424 -0.085

AF295377 -1.073 -3.867 -1.78 -2.067 -1.968 -0.506 -0.701 -0.981 -0.004 0.731 0.807 0.134

AI722334 -1.989 -3.035 -1.474 -2.454 -3.394 -1.038 -1.001 -1.328 -0.558 0.73 0.718 0.134

AW202603 -1.511 -1.898 -0.93 -0.636 -0.409 0.233 -0.064 0.081 0.152 0.726 0.583 0.455

AI884045 -0.728 -0.616 -0.806 -0.849 -0.985 -0.137 0.065 0.805 0.496 0.724 0.735 0.453

AF115774 -0.801 -0.447 -0.72 -0.429 0.379 -0.146 0.224 0.524 0.27 0.723 0.705 0.117

BG985455 -2.468 -2.178 -2.284 -1.861 -1.904 0.224 0.138 -0.069 0.355 0.721 1.045 1.76

U85090 -1.479 -1.014 1.227 0.659 0.539 -0.125 -0.049 0.738 0.21 0.719 0.742 1.204

AI793533 -0.659 -1.727 -0.722 -1.231 -1.88 -0.436 -0.498 -1.366 -0.506 0.715 0.805 -0.056

AW018949 -4.499 -3.927 -4.361 -3.394 -4.318 -1.436 -0.688 -0.633 0.188 0.714 0.229 -0.113

BI427792 -0.625 -3.292 -1.079 -1.225 -1.044 0.025 -0.026 0.018 -0.017 0.713 0.472 -0.033

BI878117 -0.586 -1.903 -0.987 -1.094 -1.077 -0.233 -0.14 0.299 -0.074 0.713 0.413 0.474

BI842851 -0.886 -1.071 -1.46 -0.259 -0.862 -0.323 0.065 0.128 -0.004 0.713 0.211 0.311

AW133635 -0.73 -0.255 0.201 0.544 0.39 0.478 -0.221 0.878 0.458 0.709 0.123 -0.013

AI601390 -1.251 -1.719 -0.529 0.223 0.208 0.239 0.559 0.558 0.138 0.709 0.501 0.545

BE016522 -0.557 -1.183 -1.225 -0.75 -0.324 0.292 0.181 0.683 0.274 0.702 0.552 -0.021

AW115841 -0.354 -0.589 0.284 0.956 0.847 1.033 0.96 1.085 0.658 0.7 0.346 1.139

AI793807 -0.603 -0.248 -0.274 0.38 0.596 -0.077 0.128 0.391 -0.148 0.696 0.817 0.62

AW059098 -0.006 -0.227 0.079 -0.481 -0.439 0.027 0.281 0.421 0.079 0.687 0.256 0.362

Y14530 -0.653 -0.433 -0.675 -0.807 -0.245 0.079 0.036 0.759 0.387 0.687 0.876 0.851

BI474953 0.452 0.064 -0.158 -0.053 -0.034 0.154 -0.036 -0.068 0.262 0.683 0.532 0.699

AI964274 -1.162 -0.725 -1.032 -0.951 -0.804 -0.254 -0.058 -0.741 -0.329 0.677 0.475 0.684

AW175139 -0.872 -0.355 -0.974 -0.231 -0.24 -0.104 -0.144 0.478 0.343 0.677 0.19 0.196

AF286374 -0.968 0.166 -0.691 -0.765 -0.405 -0.017 -0.389 -0.167 -0.868 0.674 0.845 0.986

BI887526 0.21 -0.275 -0.29 -0.067 -0.098 0.431 -0.089 0.197 0.166 0.671 0.265 0.398

BI672289 -0.225 -0.651 -1.036 -0.329 -0.207 0.385 0.344 0.714 0.346 0.67 0.566 0.753

BM157381 -0.33 -1.806 -0.878 -0.646 -0.581 0.332 0.145 0.288 0.073 0.668 0.68 0.167

BI473591 -0.17 -1.132 -1.181 -1.591 -0.562 -0.276 -0.413 0.286 0.584 0.666 0.364 -0.243

BG985507 -1.117 -1.324 -1.357 -0.779 -2.544 -0.533 -0.769 -1.615 -0.253 0.663 0.774 1.403

BF717548 -2.75 -1.849 -2.551 -0.542 -0.94 -0.673 -1.024 -1.216 -0.918 0.662 0.408 0.872

AW420883 -0.084 0.051 0.154 0.313 -0.145 0.392 -0.194 0.254 0.283 0.661 0.289 -0.389

AI964289 -0.623 -1.55 -0.759 -0.474 -0.499 -0.02 0.035 0.196 0.07 0.655 0.337 0.107

BI886767 -0.125 -0.486 -0.32 -0.277 -0.25 0.138 -0.091 0.181 0.127 0.648 0.232 0.32

AI641624 -0.047 -0.483 -0.436 -0.194 0.009 0.179 0.435 0.536 0.209 0.647 0.122 0.405

AI793454 0.05 -0.188 -0.35 0.145 -0.07 -0.274 -0.045 0.15 0.174 0.647 0.337 -0.202

AF012747 -0.886 -2.264 -1.705 -2.215 -0.854 -0.339 -0.772 -0.733 -0.48 0.647 0.486 0.219

AI878489 -0.756 -1.637 -2.01 -2.019 -0.727 -0.525 -0.8 -0.341 -1.445 0.644 0.245 -0.564

BE693123 0.246 -0.376 -0.212 -0.541 -0.829 0.232 0.067 -0.412 -0.151 0.642 0.647 0.791

AF359429 -0.643 0.096 -0.074 0.006 -0.031 0.259 -0.257 0.716 0.699 0.642 0.552 0.787

AI545291 -0.977 -2.79 -0.623 -1.127 -2.039 -0.943 -0.852 -2.154 -1.263 0.641 0.65 0.223

BM071225 -5.191 -4.481 -4.423 -3.382 -3.575 -3.213 -4.224 -4.817 -3.741 0.638 0.744 0.749

BE605522 0.614 0.081 -0.54 -0.227 -0.479 0.131 -0.091 -0.086 -0.272 0.638 0.193 0.04

AI522700 -0.056 -0.832 -0.606 -0.434 -0.675 0.069 0.099 -0.085 0.2 0.637 -0.195 -0.006

AF375871 -0.508 -1.287 -1.201 -0.967 -1.404 0.093 0.171 0.264 0.427 0.633 0.754 0.457

BI890462 -0.09 -0.339 0.336 0.513 0.607 0.16 0.243 0.814 0.206 0.632 0.614 1.065

AI882728 -0.213 -0.435 -0.348 -0.24 0.387 0.466 0.478 0.625 0.45 0.631 0.081 0.371

AI601714 -2.571 -1.432 -1.49 -0.935 -1.685 -0.957 -0.92 -1.683 -0.705 0.631 0.65 0.957

AI601488 -0.921 -1.14 -0.198 0.256 0.441 0.266 -0.006 0.479 0.122 0.613 0.471 0.957

AI588357 -1.767 -1.984 -1.2 -0.954 -0.742 0.548 -0.315 0.22 -0.266 0.611 0.085 0.65

AI793560 -0.718 -0.403 -0.25 -0.418 -0.504 -0.255 -0.192 -0.246 0.027 0.608 0.514 0.546

AA494787 -0.261 -0.292 -0.099 -0.305 0.354 0.132 0.673 0.8 0.255 0.604 0.833 0.586

BI865978 -1.666 -2.161 -0.524 -0.348 -0.393 -0.478 -0.242 0.078 0.241 0.604 0.163 -0.147

AW826482 -0.755 0.545 -0.445 0.312 -0.027 0.115 0.054 0.6 0.385 0.604 0.757 0.937

AI878403 -1.743 -1.781 -0.48 -1.104 -1.099 -0.43 -0.448 -1.237 -0.066 0.603 0.814 0.576

BG306459 -0.931 -2.668 -0.832 -0.844 -0.338 0.485 0.303 0.473 0.265 0.598 0.283 -0.037

BI879035 -0.256 -0.929 -0.814 -1.53 -1.314 -0.235 -0.583 -0.26 -0.465 0.596 0.47 -0.035

AW019276 -1.098 -0.992 -1.846 -1.07 -1.045 -0.521 -0.504 -0.084 -0.277 0.594 0.084 0.02

BM035036 -0.873 -1.719 -1.64 -0.908 -0.768 -0.007 -0.236 0.114 0.056 0.59 0.245 -0.382

BE200900 -0.459 0.059 -0.275 -0.145 0.133 -0.177 -0.02 0.661 0.33 0.589 0.56 -0.103

AI444184 -0.169 -0.222 -0.361 0.482 0.13 -0.029 -0.183 0.654 0.085 0.587 0.325 0.246

BI890772 0.065 -1.307 -0.353 -0.191 0.144 0.522 -0.002 0.096 0.398 0.587 -0.093 0.038

BI847093 -1.038 -1.035 -1.448 -0.391 -0.717 -0.215 -0.219 0.086 -0.162 0.584 0.681 0.28

AW115580 0.062 -0.657 -0.085 -0.53 -0.497 0.517 -0.027 0.157 0.026 0.581 0.514 0.591

BI880006 0.132 -0.488 -0.067 0.19 0.263 0.493 0.309 0.357 0.184 0.578 0.305 0.293

AI461323 -0.974 -0.223 -0.573 -0.678 -0.395 -0.034 -0.307 -0.07 0.076 0.576 0.698 0.728

AB032265 -1.131 -0.903 -1.384 -1.116 -0.593 -0.015 -0.367 0.571 0.364 0.573 0.419 0.51

AW232071 -0.086 -1.143 -0.733 -0.705 -0.77 -0.405 -0.111 0.256 -0.212 0.572 0.274 -0.082

AF191559 -1.181 -2.258 -1.867 -1.843 -0.516 -0.426 -0.489 -0.469 -0.786 0.572 0.508 0.358

BI879661 -0.879 -3.098 -1.445 -1.712 -3.558 -0.932 -0.982 -2.772 -0.837 0.568 0.61 1.063

AB017118 -0.545 -1.512 -0.861 -0.704 -0.642 0.443 0.135 0.316 0.294 0.566 0.429 0.616

AI626686 -0.387 -0.136 -0.525 0.087 0.303 0.121 -0.039 0.424 0.236 0.565 -0.05 0.033

BM036392 0.016 -0.904 -0.603 -0.225 -0.23 0.338 -0.104 0.197 -0.07 0.565 0.267 0.228

AF288217 -1.691 -2.012 -0.517 -0.286 -0.333 0.553 0.171 0.485 0.175 0.563 0.244 -0.125

BI672656 -0.605 -1.062 -1.097 -0.743 -1.02 -0.012 -0.72 0.01 0.204 0.562 0.487 0.55

BM023958 -0.612 -1.373 -1.264 -1.406 -0.673 -0.091 -0.37 -0.08 -0.425 0.561 0.106 0.318

BM153935 -0.464 -0.304 0.108 -0.332 -0.877 -0.406 -0.656 -0.335 -0.042 0.558 0.516 0.811

AF014370 -0.64 0.102 -0.449 0.187 -0.097 -0.209 -0.259 0.01 0.055 0.556 0.741 0.083

U62018 -1.289 -1.084 -1.565 -1.417 -1.375 -0.435 -0.259 -1.036 -0.228 0.554 0.629 1.284

X65061 -0.674 0.211 -0.605 -0.467 -0.133 -0.001 0.161 0.573 0.296 0.552 0.747 0.085

AI721335 -0.232 -0.286 -0.267 0.164 0.387 0.568 0.379 0.529 0.173 0.546 0.132 -0.223

AW826723 -0.195 -1.229 -0.851 -0.38 -0.188 0.518 0.107 0.264 -0.079 0.545 0.275 0.48

BI705843 -1.068 -0.873 -1.079 -0.68 -0.974 -0.614 -0.302 -1.079 -0.707 0.545 0.944 0.482

AI959257 -0.859 -1.429 -1.687 -0.912 -0.976 -0.477 -0.514 -0.062 0.001 0.544 0.187 0.513

BM026470 -0.16 -0.107 0.154 0.305 0.001 0.227 0.061 0.657 0.405 0.54 0.594 0.256

AI397274 -0.373 -0.746 -1.164 -0.981 -0.778 -0.371 -0.683 -0.687 -0.447 0.54 0.617 1.327

BG306420 -0.449 -0.369 -0.812 -0.335 -0.064 0.092 0.17 0.465 0.047 0.54 0.34 0.164

AI965312 -0.653 -0.311 0.166 -1.399 -0.769 -0.381 -0.562 -0.425 -0.181 0.539 0.838 0.764

AI793866 -1.399 -0.942 -1.183 -1.287 -0.542 0.175 -0.075 0.146 0.265 0.538 0.964 1.008

AI667513 -0.629 -1.308 -1.288 -0.48 -0.708 -0.255 -0.211 0.224 -0.08 0.537 0.757 0.367

AF064835 -3.391 -2.993 -0.805 -0.603 -0.7 -1.293 -1.726 -0.682 0.318 0.534 0.119 -0.17

AI884099 -0.216 -1.39 -1.02 -0.618 -0.681 -0.266 -0.312 -0.125 -0.004 0.531 0.062 -0.107

AW281249 -0.963 -0.636 -0.621 -1.172 -1.456 -0.465 -0.548 -1.574 -0.562 0.528 0.576 0.804

BG985575 -0.436 -0.403 0.252 0.179 0.04 0.305 -0.019 0.687 0.395 0.525 0.853 0.462

AF389401 -1.942 -0.713 -1.278 -0.59 -0.489 -0.051 -0.602 0.246 0.154 0.519 0.208 0.69

AW419481 -0.558 0.018 -0.305 0.14 0.531 0.517 0.27 0.809 0.191 0.518 0.573 0.774

BM026841 -0.407 -0.573 -0.384 -0.572 0.089 -0.034 -0.002 -0.297 0.392 0.518 0.694 0.324

BI897414 -0.304 0.048 -1.529 -0.56 -1.089 -0.538 -0.372 -0.447 -0.335 0.513 0.123 0.238

AW170954 -0.644 -0.246 -0.246 0.059 0.283 0.68 0.297 0.515 0.468 0.51 0.678 0.487

BM182563 -2.762 -5.406 -1.611 -0.382 -0.439 0.344 -0.06 -0.263 -0.409 0.509 -0.033 -0.381

AI959722 0.539 0.234 -0.142 0.134 0.114 0.409 -0.036 0.148 0.35 0.508 0.67 0.71

BI887990 -0.482 -0.195 0.108 0.092 -0.027 0.436 0.393 0.522 0.342 0.505 0.41 0.139

BE693186 -2.7 -4.376 -4.107 -3.132 -4.54 -2.778 -3.48 -5.365 -3.512 0.504 1.16 2.194

BM184148 0.245 0.076 -0.151 -0.005 -0.435 0.494 -0.119 0.462 -0.244 0.502 -0.037 0.405

AF108819 -0.383 -0.261 0.243 0.665 0.508 0.7 0.375 0.695 0.281 0.493 0.818 0.623

AI384393 -1.225 0.136 -0.716 -0.388 -1.029 0.07 -0.496 -0.771 -0.101 0.493 0.426 0.739

AI878244 -0.515 -0.507 0.333 0.497 0.373 0.126 0.204 0.459 0.406 0.492 0.003 0.051

AF068772 -1.603 -1.025 -0.605 0.007 -0.206 -0.05 -0.123 0.162 0.047 0.486 0.004 -0.163

AF210320 -0.766 -0.533 -0.787 -1.15 -1.077 -0.153 -0.352 -0.318 0.103 0.486 0.903 0.702

AI943183 -0.816 -0.564 -0.752 -0.732 -0.457 -0.094 -0.342 0.091 0.558 0.486 1.172 0.844

BI880002 -1.299 -1.334 -1.435 -1.556 -1.263 -0.527 -0.236 0.019 0.093 0.483 0.676 0.476

AI626645 -1.82 -1.646 -2.22 -1.588 -1.941 -0.231 -1.139 -1.811 -0.93 0.483 0.18 -0.159

BI476031 -0.211 0.045 -0.309 0.466 0.539 0.025 -0.057 0.504 0.281 0.479 0.121 0.072

AI641141 -0.559 -0.699 -0.4 -0.305 -0.516 -0.172 -0.133 -0.03 0.351 0.478 0.419 0.265

AI641626 -0.248 -0.233 -0.185 -0.638 -0.208 -0.238 0.251 0.668 0.133 0.478 0.807 0.214

BI886435 -0.26 0.005 -0.052 0.54 0.788 0.514 0.205 0.51 -0.026 0.477 0.36 1.364

AI878053 -0.255 -0.547 -0.895 -0.449 -0.651 0.165 -0.67 0.302 0.253 0.475 -0.17 0.126

AI588589 -0.379 -0.404 -0.413 -1.15 -1.002 -0.033 -0.06 -0.853 -0.094 0.473 0.913 0.602

AI584555 -1.235 -1.715 -1.292 -2.098 -2.281 -0.677 -1.338 -2.143 -0.862 0.47 0.826 -0.032

AI878520 -0.436 -0.275 0.469 -0.804 -1.431 -0.219 -0.138 -0.155 -0.108 0.469 0.384 0.74

AW077448 0.099 -0.101 0.247 0.454 0.277 0.451 0.318 0.55 -0.153 0.469 0.641 0.472

AI793761 -0.488 -0.489 -1.011 -0.203 -0.361 -0.341 -0.191 -0.448 -0.184 0.469 -0.059 0.175

BI892407 -0.244 -0.259 -0.318 0.121 0.103 0.125 -0.058 0.435 0.101 0.469 0.588 0.103

BI880074 -2.049 -1.205 -1.944 -0.5 -1.529 -0.49 -1.399 -1.693 -1.043 0.456 0.495 1.076

U14590 0.002 -0.289 0.077 -0.093 -0.087 -0.197 -0.069 -0.101 -0.167 0.452 0.902 0.797

BF717510 0.094 0.139 -0.286 -0.342 -0.775 0.046 -0.441 0.081 0.309 0.45 0.341 1.199

U66570 -0.401 -0.682 -0.692 -1.034 -0.838 -0.392 0.065 -0.039 0.065 0.45 0.457 -0.045

BI878720 -0.785 -1.941 -0.526 -0.582 -0.483 0.006 -0.061 -0.322 0.189 0.449 0.251 0.059

BE605276 -0.11 0.188 0.016 0.019 -0.366 -0.212 0.014 0.648 -0.062 0.448 0.734 0.191

AI601782 0.255 0.129 -0.327 -0.542 0.077 0.166 0.243 0.311 0.126 0.44 0.869 0.615

BI672308 -0.728 0.037 -0.058 0.184 0.32 -0.059 -0.229 0.147 0.141 0.434 0.518 -0.261

AF071246 -0.837 -0.615 -1.049 -0.62 -0.727 -0.323 -0.558 0.051 0.525 0.432 0.788 0.489

AI957401 -1.283 -1.883 -1.559 -1.393 -1.536 -0.303 -0.016 -0.081 -0.097 0.431 0.799 1.195

BI892299 -0.595 0.144 0.455 0.485 0.705 0.341 -0.026 0.397 0.506 0.429 0.67 0.686

AI477020 -0.543 -0.211 -0.702 -0.88 -0.565 0.149 -0.243 0.84 0.548 0.428 0.446 1.106

AJ278268 -0.13 -0.951 -0.309 -1.138 -0.687 0.184 -0.251 0.066 -0.223 0.425 -0.219 -0.561

AI558352 -0.453 0.095 -0.352 -0.623 0.407 0.066 0.149 0.172 -0.026 0.425 0.511 -0.126

AJ311846 -3.017 -1.827 -2.235 -1.521 -1.92 -1.408 -2.209 -1.976 -2.409 0.424 1.34 1.516

AF375227 -1.038 -0.328 -0.643 -0.715 -0.69 -0.152 -0.372 -0.496 0.257 0.406 0.661 0.59

BI705531 0.451 -0.448 -0.185 0.026 -0.271 -0.12 -0.293 -0.11 -0.002 0.402 0.639 -0.018

BM026015 -1.111 -0.52 0.31 0.312 0.711 -0.183 -0.431 -0.171 0.773 0.401 1.282 0.924

AA606080 -0.476 -0.352 -0.474 -0.089 -0.325 -0.001 -0.225 -0.467 -0.452 0.398 0.713 1.42

AF332623 -0.353 0.164 -0.041 0.224 0.169 0.177 0.117 0.456 0.21 0.397 0.19 0.071

AI721531 -0.209 -1.324 -0.461 -0.812 -1.284 -0.48 -0.043 -0.98 -0.255 0.389 0.465 1.205

AI793424 -1.891 -1.113 -1.885 0.01 -1.501 -0.571 -0.502 -1.886 -1.307 0.388 0.528 1.185

BI672022 -1.198 -1.693 -2.077 -2.443 -2.278 -1.256 -1.318 -1.925 -1.458 0.387 0.402 1.391

BI888729 -1.749 -1.295 -1.547 -0.988 -0.625 -0.261 -0.795 -0.395 0.143 0.386 0.058 -0.013

BG308628 -1.186 -1.349 -1.625 -0.659 -1.519 -0.454 -0.863 -1.605 -0.731 0.386 -0.061 -0.218

AI965247 0.582 -0.198 -0.252 -0.683 -1.504 0.115 -0.041 0.044 -0.2 0.384 0.695 -0.128

AI943036 -0.842 -0.064 0.05 -0.098 0.133 0.113 0.072 0.662 0.558 0.382 1.113 0.706

AW420849 -0.32 -0.158 -0.423 0.024 0.067 0.432 0.146 0.048 0.444 0.381 0.334 0.595

AW076688 -0.571 -0.687 -0.539 -1.223 -1.573 -0.367 -0.2 -1.827 -0.571 0.379 0.994 0.633

Y07905 -0.702 -0.446 -0.365 -0.273 -0.697 -0.017 -0.574 -0.409 0.247 0.377 0.922 0.652

U23822 -3.984 -3.574 -3.624 -2.803 -3.981 -2.283 -2.654 -3.118 -1.063 0.375 0.669 0.858

X70299 -0.468 -0.75 -0.195 -0.379 -0.561 -0.024 -0.424 -0.514 0.069 0.37 0.697 0.937

BG308501 -0.356 -0.736 -0.215 -0.078 -0.298 0.516 0.567 0.398 0.5 0.369 0.434 0.875

BI883230 -0.817 0.011 -0.315 -0.783 -0.326 -0.12 -0.079 0.128 -0.102 0.368 0.704 -0.102

AI584977 -0.858 -0.978 -0.466 -1.324 -1.717 -0.394 -0.399 -1.434 -0.84 0.367 0.714 0.421

BI886259 -0.472 -0.698 0.159 0.519 0.573 -0.188 0.259 0.554 0.059 0.363 0.775 0.847

AJ011112 -0.569 0.11 -0.195 -0.071 0.098 0.01 0.079 0.49 -0.141 0.357 0.723 -0.1

AI353083 -4.956 -4.135 -4.296 -2.727 -4.102 -3.193 -3.663 -4.553 -3.654 0.35 1.008 2.248

X87750 -0.43 0.015 0.059 0.059 0.348 -0.012 -0.17 0.551 0.128 0.349 0.559 0.397

BI888755 -1.44 -1.054 0.551 0.444 0.276 0.614 0.472 0.447 0.43 0.34 0.164 0.875

BI886847 -0.112 -0.872 -0.194 0.081 0.248 0.046 0.333 0.473 0.368 0.339 0.526 0.324

AF321194 -0.879 -0.611 -0.648 -0.711 -0.925 -0.125 -0.312 -0.412 -0.204 0.332 0.861 1.31

AF030031 -1.495 -1.641 -1.553 -1.041 -0.655 -0.159 0.379 0.113 -0.139 0.331 0.749 0.613

BI980180 -0.167 0.156 0.041 -0.825 -0.706 -0.247 -0.331 -0.407 -0.323 0.325 0.845 0.402

AW281650 0.395 -0.017 0.485 -0.173 0.206 0.245 0.398 0.257 0.312 0.321 0.208 1.137

AF083557 -0.778 -2.324 -0.694 -1.19 -1.518 -0.846 -0.526 -1.55 -0.692 0.32 0.35 -0.026

BM156937 -0.349 0.079 0.298 -0.598 -0.048 0.052 0.002 0.181 0.076 0.319 1.45 0.077

AW279630 0.099 -0.042 -0.632 0.081 0.128 -0.113 0.085 0.482 0.172 0.317 0.544 0.203

AW421939 -5.597 -6.185 -5.674 -4.972 -4.629 -3.232 -2.978 -2.079 -0.6 0.315 0.385 -0.22

BI325077 -1.956 -1.575 -2.353 -1.153 -1.71 -1.106 -1.498 -1.321 -0.062 0.313 0.604 1.321

BI845510 -0.728 -0.12 0.041 0.182 0.888 0.869 0.996 0.957 1.059 0.31 1.139 0.931

AW232425 -0.013 0.166 0.371 0.071 0.097 -0.448 -0.176 -0.23 -0.195 0.307 0.496 0.069

AI667071 -0.681 -0.216 -0.191 -1.076 -0.968 -0.206 -0.061 -0.242 -0.248 0.303 0.625 1.088

BI982778 -1.391 -0.567 0.017 -1.212 -1.817 0.09 -1.219 -1.575 -0.847 0.299 0.768 1.895

AW777326 -0.408 0.122 -0.085 0.353 0.157 0.304 -0.126 0.36 0.004 0.298 0.443 0.049

Y13944 -0.95 -1.046 -0.46 -0.402 -1.318 -0.378 -0.214 0.11 0.289 0.292 0.601 0.804

X85977 -0.448 -0.042 0.179 -0.156 -0.138 -0.182 0.04 0.281 0.021 0.29 0.46 0.583

AW018967 -0.576 -1.951 -0.202 -0.545 -0.219 0.28 0.529 0.381 0.485 0.29 0.612 0.248

AA497336 -0.276 -0.342 -0.592 -0.083 -1.184 -0.383 -0.12 -0.661 -0.015 0.288 0.33 0.528

BG985485 -0.443 -0.841 -1.389 -0.255 -0.177 -0.452 -0.386 -0.073 -0.252 0.287 -0.165 -0.094

BG305033 -0.458 0.132 -0.08 0.328 -0.083 0.015 -0.397 -0.14 0.039 0.284 0.601 0.997

AI641772 -0.666 -0.093 -0.096 0.135 0.015 -0.378 0.026 -0.379 -0.113 0.283 0.348 1.126

AY029402 -0.471 -0.761 0.617 0.669 -0.418 -0.265 0.076 -0.945 -0.242 0.281 0.747 0.537

AW117094 -1.936 -2.566 -1.102 -0.582 -0.472 -0.16 -0.121 -0.03 -0.515 0.28 0.393 -0.334

AI884046 -0.793 -0.679 -0.35 -1.676 -0.971 -0.436 -0.219 -0.209 -0.368 0.279 0.854 0.686

AI957739 -0.583 -0.103 0.01 -0.311 0.009 0.114 -0.054 0.299 -0.168 0.278 0.778 0.128

BI886069 -0.589 -0.668 -1.333 -0.536 -0.784 -0.517 0.308 -0.237 0.129 0.271 0.069 -0.366

BI878304 -0.728 0.321 0.001 -0.122 -0.107 -0.135 -0.243 0.193 -0.336 0.27 0.107 0.707

AF190144 -0.978 -0.948 -1.346 -0.545 -1.109 -0.306 -0.448 -0.474 -0.263 0.269 1.169 0.445

AI942982 -0.587 0.545 0.407 0.341 -0.048 0.161 0.179 0.571 0.304 0.268 0.582 0.237

BI429638 -0.186 -0.525 0.122 -0.168 0.107 -0.289 -0.305 0.611 0.509 0.259 0.619 0.119

AW826646 -0.423 -1.077 -0.193 -0.166 0.365 -0.258 -0.061 -0.347 0.277 0.253 0.522 0.262

AF006831 -0.796 -0.238 -0.671 -0.2 -0.276 -0.376 -0.572 -0.094 -0.303 0.251 0.416 0.668

AW018635 -2.344 -2.123 -1.911 -2.042 -2.15 -1.245 -1.531 -0.845 -0.3 0.248 0.501 1.415

BM102195 -0.505 0.222 0.397 0.105 -0.343 0.336 -0.151 -0.203 -0.138 0.246 0.318 0.789

BF717503 -2.046 -1.241 -1.596 -0.88 -1.301 -0.747 -1.135 -0.731 -0.539 0.243 -0.099 -0.587

AW233059 -0.728 -0.245 -0.831 -0.522 -0.579 -0.517 -0.375 -0.565 -0.322 0.241 0.096 1.3

AF030284 -0.087 -0.162 0.244 0.055 0.547 -0.212 0.109 0.9 0.052 0.24 0.989 0.358

AF280090 -0.907 -0.473 -0.864 -0.522 -0.757 -0.287 -0.747 -0.727 -0.202 0.24 1.04 1.187

AW077420 -0.42 -0.177 -0.273 -0.683 -0.373 -0.149 -0.491 -0.506 -0.078 0.239 0.413 0.808

BI865477 -0.766 -0.882 -1.301 -1.439 -0.642 -1.073 -0.96 -1.381 -0.722 0.236 0.8 1.444

AI585020 -0.046 -0.362 -0.165 -0.035 0.212 0.324 0.453 0.303 0.227 0.232 0.523 0.114

Y14548 -0.667 -0.321 0.461 0.024 -0.234 -0.369 -0.098 0.428 0.244 0.224 0.572 0.203

AW175546 -0.601 -0.1 0.308 0.322 0.043 0.238 -0.071 0.579 0.133 0.223 0.705 0.747

AI794518 -1.509 -1.481 -3.206 -1.149 -2.079 -0.902 -1.031 -1.601 -0.638 0.222 0.241 1.5

AW171479 -1.391 -1.518 -1.069 -0.516 -1.096 -0.507 -1.242 -0.989 -0.469 0.222 0.383 0.646

BI563231 -3.401 -3.266 -2.836 -1.927 -2.733 -0.643 -0.943 -0.335 -0.309 0.219 0.32 1.05

AI957592 -0.202 -1.759 -0.142 -0.501 -0.423 0.089 0.23 0.605 0.365 0.217 0.494 0.581

AF272962 -0.451 -0.033 -0.153 -0.166 0.03 0.159 -0.578 0.657 0.541 0.215 0.862 0.415

AI943112 -0.436 0.018 0.181 -0.314 0.187 -0.013 -0.357 -0.306 0.096 0.21 0.847 0.784

AF082662 -4.932 -5.147 -4.876 -4.098 -4.939 -3.778 -3.013 -4.62 -4.127 0.208 0.905 2.008

AI641414 0.025 -0.377 -0.269 -0.221 -0.269 0.144 -0.03 -0.048 0.388 0.2 0.423 0.905

AI626604 -0.051 -0.282 0.118 0.525 0.611 0.504 0.403 0.275 0.063 0.2 0.052 0.739

BM026607 -0.296 -0.288 -0.976 -1.154 -1.704 -0.211 0.151 -0.756 -0.819 0.199 0.097 1.465

AI545455 -0.45 -0.795 -0.018 -0.136 -0.437 -0.158 0.02 -0.447 -0.057 0.195 0.351 0.126

AA495157 0.133 -0.022 -0.092 0.069 -0.001 0.063 0.162 -0.271 -0.23 0.195 0.533 0.274

BI879868 0.52 -0.239 0.246 0.186 -0.077 0.27 0.467 -0.127 0.363 0.193 0.479 0.746

BI896301 -0.041 0.453 0.4 0.072 -0.517 0.063 -0.132 -0.453 0.299 0.193 1.019 0.067

AW282035 0.377 -0.11 -0.097 -0.109 -0.349 0.184 0.499 -0.244 0.109 0.191 0.372 0.922

BE693132 -0.792 -0.118 -0.14 -0.855 -0.627 -0.163 -0.362 -0.469 -0.434 0.19 0.884 0.174

AW827044 -0.213 -0.031 0.221 0.013 -0.746 -0.221 -0.145 -0.707 -0.084 0.186 0.261 0.754

D13045 -0.895 -1.574 -0.982 -0.75 -1.018 -0.474 -0.353 -1.007 -0.421 0.185 0.882 0.48

U49407 -0.582 -0.107 0.132 -0.301 -0.272 0.045 -0.1 -0.114 0.117 0.185 0.522 0.23

AI793374 -0.007 -0.394 -0.129 -0.012 0.594 0.085 0.383 0.131 0.191 0.183 0.542 0.484

BI879533 -0.541 -0.068 0.729 -0.562 0.829 0.627 0.044 1.092 0.327 0.182 0.366 1.044

AW203020 -0.119 0.073 0.2 0.359 0.478 0.52 0.101 0.371 0.217 0.18 0.603 0.296

BI863998 -0.506 -0.04 -0.259 -0.149 -0.883 -0.452 -0.477 -0.69 -0.065 0.18 0.163 -0.643

AW420737 0.303 0.139 0.179 0.448 0.063 0.297 0.425 0.282 0.482 0.174 0.499 0.482

BI708877 -0.344 -0.764 -0.158 0.165 0.076 -0.091 -0.005 -0.069 0.111 0.174 0.388 0.273

BG302583 0.4 0.208 0.14 0.019 -0.576 -0.224 -0.613 -0.456 -0.083 0.17 0.4 0.658

AI957909 -0.01 -0.919 0.124 -0.129 -1.073 -0.205 -0.171 -0.804 -0.431 0.169 0.423 0.232

BM024326 -0.33 0.224 0.359 0.343 0.412 0.2 0.211 0.61 0.319 0.167 0.916 0.537

AW567517 -0.749 -0.53 -1.515 -0.462 0.14 0.264 0.136 0.788 0.319 0.167 0.183 0.876

BG985742 -0.728 0.42 0.27 -0.369 -0.645 -0.317 -0.478 -0.054 -0.406 0.157 0.724 0.08

BI708092 -0.788 -0.604 -0.383 -0.128 0.335 0.018 -0.092 0.278 0.515 0.155 0.813 0.763

BG985516 -0.567 0.222 0.467 0.401 0.104 -0.334 0.148 0.04 0.321 0.154 0.464 0.482

D32214 -0.111 -0.152 -0.016 0.203 0.091 0.131 -0.169 0.655 0.409 0.152 0.563 0.177

AI959515 -0.553 -0.111 -0.309 -0.501 -0.131 0.301 -0.197 -0.734 -0.382 0.148 0.675 -0.098

AA495154 0.016 -0.528 0.061 0.713 0.944 0.488 0.231 0.589 0.071 0.145 0.559 1.024

AW115640 -0.658 -0.291 0.162 -0.037 0.458 0.309 -0.187 -0.117 0.224 0.144 0.418 -0.09

U93478 -0.025 0.146 0.291 0.471 0.529 0.455 -0.1 -0.072 0.301 0.144 0.905 0.184

AI444433 0.263 0.045 0.322 0.243 0.228 0.022 0.328 -0.113 0.181 0.143 0.484 0.892

BM155230 -0.102 -0.069 -0.161 -0.579 -0.022 -0.031 0.076 -0.401 0.083 0.139 0.542 0.137

BI840999 -0.503 -0.803 -0.342 -1.094 -1.692 -0.519 -0.076 -0.556 -0.559 0.136 0.074 1.717

Y13948 -0.383 0.021 -0.107 -0.369 -0.325 -0.009 -0.075 0.071 0.276 0.128 1.097 0.553

BG305857 -0.118 -0.062 -0.031 0.128 0.113 0.368 -0.059 0.083 0.378 0.128 0.384 0.65

BE693143 -0.197 -0.392 0.544 0.01 0.49 0.246 0.29 0.071 -0.237 0.127 0.492 0.277

AB055663 -0.925 -0.955 -1.396 -1.318 -1.08 -0.399 -0.805 -0.881 -0.579 0.124 0.827 0.529

AI721741 -0.271 -0.258 -0.276 -0.824 -0.76 0.033 -0.221 0.458 0.36 0.122 1.137 0.62

AA494746 -0.547 -0.364 -0.35 0.21 -0.024 0.01 -0.004 -0.209 -0.105 0.118 0.506 0.689

AA495418 -0.524 -0.54 -0.283 -1.292 -1.088 -0.06 -0.467 -0.934 -0.543 0.116 0.698 0.689

AF071245 -0.798 -0.426 -0.383 -0.262 -0.305 -0.04 -0.512 0.077 0.075 0.114 1.072 0.666

AI943132 -0.19 -0.16 -0.053 -0.101 -0.547 -0.228 -0.233 -0.734 -0.286 0.11 0.31 0.873

AJ404970 -0.461 0.545 0.15 0.341 -0.173 0.253 -0.098 0.604 -0.008 0.108 0.582 0.946

AB006104 -0.392 -0.055 -0.35 -0.068 -0.099 0.188 -0.234 -0.017 0.366 0.107 1.15 0.477

AI957596 0.25 -0.803 -1.084 -1.247 -1.395 -0.251 -0.16 -0.226 -0.268 0.104 0.209 0.808

AI667319 -0.359 -0.349 -0.775 -0.18 -0.332 -0.091 0.024 -0.306 -0.087 0.102 1.13 0.728

BI475215 0.073 -0.05 -0.24 -0.019 -0.095 0.119 -0.127 -0.163 0.079 0.1 0.134 1.345

AI722307 -0.287 0.545 0.407 -0.438 -0.025 -0.163 -0.193 -0.121 0.158 0.097 0.705 0.044

BM102223 -0.385 -0.25 0.431 0.075 -0.049 -0.136 0.124 -0.03 0.455 0.093 0.617 0.484

AI354033 -0.406 -0.046 -0.066 0.025 -0.083 -0.252 0.121 0.233 0.192 0.09 0.428 0.112

AW203129 0.196 -0.695 -0.284 -0.071 -0.154 0.054 0.226 0.279 0.314 0.088 0.675 0.444

U14587 0.126 -0.451 -0.485 -0.458 -0.64 -0.269 0.183 -0.499 -0.163 0.086 0.982 0.524

BF717383 -0.933 -1.16 -0.635 -0.832 -2.045 -0.734 -0.438 -1.136 -0.477 0.084 0.285 1.197

AF277097 -0.608 -0.04 -0.143 -0.226 0.158 0.191 0.145 0.117 0.111 0.083 0.727 0.551

BG303217 -0.767 -0.118 -0.081 -0.202 -0.221 0.123 -0.049 -0.236 -0.015 0.081 0.727 -0.099

BI430050 -0.865 -0.895 -0.432 -0.234 -1.125 -0.483 -0.184 -1.048 -0.102 0.079 0.253 0.535

AF395831 -0.942 -0.211 -0.385 -0.621 -0.059 -0.037 -0.402 -0.283 -0.291 0.076 0.238 0.848

X96422 -0.427 -1.564 -0.962 -1.61 -1.825 -0.285 -0.648 -1.446 -0.39 0.075 0.886 0.743

AI584515 -0.679 -1.22 -0.3 -0.396 -0.491 0.068 -0.242 -0.349 -0.108 0.07 0.086 0.433

AF237712 -1.784 -1.984 -1.481 -0.544 -1.188 -0.671 -0.61 -1.588 -1.102 0.066 0.255 1.869

AI477343 -0.045 0.01 -0.442 -1.177 -1.693 -0.553 -0.404 -0.747 -0.651 0.065 0.082 -0.226

AI878392 -0.689 -0.979 -0.56 -0.257 -0.584 -0.101 -0.597 -0.446 -0.289 0.065 0.252 0.787

AI722645 -0.351 -1.571 -0.358 -0.759 -0.684 -0.154 -0.295 -0.463 -0.036 0.063 0.524 0.778

BI534277 0.08 -0.005 0.001 0.202 -0.018 0.09 -0.01 -0.325 -0.048 0.061 0.449 0.306

BI891552 -0.658 -0.202 -0.013 -0.591 0.062 0.095 0.034 0.153 0.638 0.058 1.013 0.678

AW077237 -0.75 -0.075 -0.461 -0.083 -0.205 0.045 -0.234 0.189 0.167 0.051 0.636 0.364

BF938407 0.2 -0.041 -0.115 0.341 -0.108 0.442 0.432 -0.245 0.295 0.049 0.16 1.651

AI584487 -1 -1.245 -0.437 -0.42 0.349 0.114 -0.035 0.329 0.571 0.046 0.602 0.421

BG305295 0.508 0.013 -0.603 -0.323 -0.219 -0.084 -0.264 -0.278 -0.321 0.045 0.124 0.721

AI330805 -1.018 -1.527 -1.655 -1.051 -1.536 -0.675 -0.455 -0.947 -0.037 0.043 1.056 0.06

AF173984 -2.697 -2.181 -3.052 -1.907 -2.252 -1.324 -1.223 -1.795 -1.332 0.041 -0.576 1.144

BM070919 -0.585 -0.933 -0.588 -0.311 -0.646 -0.466 -0.338 -0.795 -0.292 0.041 0.098 0.752

AW305840 -0.491 -0.356 -0.932 -1.162 -0.869 -0.409 -0.393 -0.64 -0.391 0.036 0.335 1.135

BI842047 -0.425 0.053 -0.061 0.36 0.344 -0.238 -0.184 0.412 -0.038 0.036 0.576 0.146

AW421096 -0.622 -0.134 0.036 -0.583 -1.01 -0.265 -0.247 -0.267 -0.284 0.034 0.753 0.321

AI558547 -0.651 -1.56 -0.297 -0.069 0.232 -0.157 -0.193 -0.387 0.216 0.03 0.336 0.156

AI793897 -0.2 -0.537 -0.48 0.064 -0.934 -0.058 -0.043 0.046 0.15 0.026 0.281 0.757

AI722818 -0.093 -0.373 -0.099 -0.085 0.614 0.004 -0.028 -0.114 0.148 0.018 0.491 0.43

AW232264 0.443 -0.58 -0.003 -0.223 -0.559 -0.255 0.023 -0.476 -0.22 0.017 -0.023 0.851

AF204240 -0.779 -0.236 -0.154 -0.964 -0.678 -0.392 -0.489 -0.447 -0.36 0.017 0.903 1.621

U89710 -0.728 -0.019 -0.578 -0.259 -0.268 -0.125 -0.484 0.042 -0.785 0.013 0.625 1.005

BG305301 -1.243 -1.257 -0.695 -0.462 0.228 0.177 -0.279 -0.201 0.526 0.008 0.61 0.39

BG883345 -0.691 0.545 -0.168 0.341 0.441 0.398 0.25 -0.057 0.046 0.005 0.794 0.512

AI444432 -0.728 0.545 0.124 -0.339 -0.055 -0.125 -0.428 0.193 -0.332 0.005 0.05 1.006

BM156086 -0.746 -0.413 -0.608 -0.318 0.086 -0.145 -0.273 -0.264 -0.023 -0.007 0.72 0.277

BG308557 -0.345 -0.143 0.306 0.335 0.328 0.222 0.184 0.227 0.24 -0.013 0.409 0.757

AI437329 -0.952 -1.467 -1.101 -0.624 -0.446 -0.484 -0.239 -1.054 -0.121 -0.015 0.469 0.3

U57973 -0.84 -0.793 -0.944 -0.276 -0.396 -0.097 -0.458 -0.596 0.532 -0.018 0.611 0.074

BM186246 -1.733 -2.543 -1.219 -1.008 -0.969 -0.102 -1.35 -0.932 -0.876 -0.02 -0.173 0.497

BG884401 -0.278 -0.612 -0.505 -0.627 -0.266 -0.268 -0.125 -0.734 -0.212 -0.022 0.176 0.979

BM035348 0.116 -1.108 0.077 -0.329 -1.14 -0.031 -0.115 -1.005 -0.209 -0.023 0.057 1.258

BI710508 0.044 0.492 -0.192 -0.095 -0.21 -0.245 0.115 -0.069 0.138 -0.024 0.293 0.587

BM186665 -1.055 -0.089 -0.201 -0.421 -0.421 -0.178 -0.489 -0.323 -0.705 -0.025 0.356 1.458

AI558311 -0.445 -0.226 -0.538 -0.23 0.206 0.221 0.047 0.532 0.148 -0.038 0.681 0.137

AI558512 -0.54 0.545 0.407 -0.128 0.105 0.056 -0.212 0.215 -0.097 -0.04 0.701 -0.153

BM185901 0.05 -0.114 -0.085 0.066 -0.146 0.386 -0.313 -0.312 -0.166 -0.041 0.095 1.595

AI957812 -0.627 -1.427 -0.245 -0.173 -1.454 -0.484 -0.255 -1.491 -0.641 -0.043 -0.064 1.004

AI883799 -0.621 0.076 0.167 0.296 0.132 -0.19 0.019 -0.251 0.356 -0.045 0.406 0.234

AF071267 -0.355 0.309 0.512 -0.422 -0.138 0.04 0.257 0.86 0.29 -0.05 1.139 0.185

BI982877 0.718 0.142 0.147 0.316 -0.103 0.168 0.114 0.544 -0.035 -0.054 1.038 0.06

AI884279 -0.233 -0.38 -0.17 -0.291 -0.034 -0.278 -0.008 -0.608 0.064 -0.054 0.528 -0.049

AF121796 -2.395 -1.666 -2.489 -2.316 -1.773 -1.2 -1.064 -1.928 -2.096 -0.057 0.326 1.466

BI428149 -0.685 -0.785 -0.169 -0.271 -0.255 -0.187 -0.02 -0.893 -0.486 -0.058 0.475 0.393

AW154726 -0.988 -0.38 -0.295 0.149 -0.383 -0.174 -0.205 -0.497 -0.316 -0.058 0.01 0.584

BG308524 -0.524 -0.841 -0.08 -0.221 -1.358 -0.907 -0.189 -0.763 -0.325 -0.058 0.348 1.366

AF384863 -0.487 0.225 -0.084 0.125 0.457 0.09 0.264 0.336 -0.137 -0.064 0.729 -0.092

AW171072 -0.984 -0.475 0.038 -0.319 -0.201 -0.067 0.097 -0.46 0.334 -0.065 0.411 0.312

BI533161 -0.951 -2.53 -1.153 -1.991 -2.07 -0.193 -1.051 -2.653 -1.072 -0.066 0.536 1.081

BI865912 -0.728 -0.334 -0.036 -0.89 -0.972 -0.522 -0.312 -0.932 -0.284 -0.075 0.796 1.537

BG302934 -0.955 -1.085 -1.254 -0.422 -1.665 -0.474 -0.545 -1.982 -0.751 -0.079 0.684 0.728

AI584554 -0.494 -0.907 -0.138 -0.466 0.161 -0.489 -0.052 -0.31 0.014 -0.079 0.365 0.339

AI601355 -0.728 -0.045 0.277 -0.754 -0.902 -0.532 0.118 0.815 0.399 -0.08 0.932 0.559

AJ245962 -0.519 -0.315 -0.206 -0.361 -0.723 -0.113 -0.543 -0.681 -0.25 -0.084 0.272 1.063

AW282014 -1.081 -0.44 -0.941 0.066 -0.202 -0.052 -0.596 -0.253 -0.868 -0.098 0.113 1.25

AW116326 -0.573 -0.367 -0.313 -0.325 -0.426 -0.54 -0.152 -0.831 -0.1 -0.1 -0.04 0.742

AF036148 -0.192 -0.239 -0.24 -0.039 -0.375 -0.167 -0.177 -0.42 -0.463 -0.101 0.063 1.317

AI353581 -0.529 -0.212 -0.448 -0.527 -0.435 -0.091 -0.588 -0.592 -0.122 -0.102 0.039 1.413

BM154820 -0.595 -0.851 -0.908 -0.556 0.055 -0.347 -0.008 -0.676 0.069 -0.104 0.518 0.206

AI641460 -0.482 -0.355 0.391 0.474 0.71 0.197 0.291 0.544 0.411 -0.105 0.68 0.707

AA542593 0.025 -0.185 0.313 -0.335 -0.032 -0.229 -0.043 -0.127 -0.192 -0.112 0.095 0.828

BI882649 -0.302 -0.942 0.173 -1.098 -0.869 -0.356 0.048 -1.126 -0.669 -0.114 0.618 1.476

AW510270 -0.052 -0.371 -0.433 -0.383 -0.395 -0.115 -0.338 -0.402 0.01 -0.119 0.237 0.697

AJ245493 -1.13 -1.228 -0.674 -0.241 -0.605 -0.424 -0.373 -0.311 -0.157 -0.119 0.355 0.446

AF281003 -2.203 -2.458 -2.203 -1.488 -2.283 -1.568 -1.738 -1.873 -1.876 -0.125 0.271 0.602

BI673470 -0.578 -0.147 0.521 -0.063 -0.252 -0.032 0.345 -0.344 0.094 -0.129 0.063 1.458

BI430334 0.361 -0.492 -0.661 -0.096 -0.719 -0.109 -0.02 -0.694 -0.443 -0.131 0.05 0.753

BI670932 -1.039 -1.085 -1.016 -0.629 -0.621 -0.544 -0.571 -0.696 -0.721 -0.136 -0.13 0.518

BI845367 -0.734 -0.238 -0.375 -0.398 -0.243 -0.157 -0.183 -0.676 -0.332 -0.137 0.32 1.005

AF364083 -0.811 -0.549 -0.023 -0.339 -0.923 -0.125 -0.656 -0.812 -0.121 -0.138 0.515 0.927

AI626799 -0.679 0.35 0.212 -0.308 -1.107 -0.284 -0.286 -0.373 -0.331 -0.14 1.113 0.552

AI942990 -0.119 -0.542 -0.384 -0.432 -0.891 -0.189 0.046 -0.898 -0.38 -0.144 -0.28 1.279

BG727484 -1.565 -0.908 -0.623 -0.482 0.297 -0.261 -0.487 -0.618 0.426 -0.158 0.582 0.571

AI877924 -0.373 0.03 -0.176 0.038 0.486 -0.21 0.298 0.143 0.042 -0.159 -0.015 0.572

AW419638 -0.539 -0.446 -0.264 -0.202 0.038 -0.018 0.053 -0.281 0.373 -0.17 0.34 0.384

AA606054 -2.172 -2.391 -1.001 -1.194 -0.848 -0.495 -0.896 -0.977 -0.583 -0.175 -0.007 0.401

AI626598 -0.879 -0.234 0.05 0.065 0.115 -0.039 -0.164 -0.062 -0.096 -0.175 0.595 0.384

BI879589 -0.925 -0.847 -1.692 -1.046 -1.503 -0.277 -0.797 -0.765 0.367 -0.177 0.659 1.18

AY017309 -1.071 -1.343 -0.731 -0.465 0.184 -0.169 -0.201 -0.096 0.057 -0.183 0.656 0.583

BM187382 -1.106 -0.907 -1.01 -0.681 -0.467 -0.343 -0.513 -0.617 0.03 -0.186 0.445 0.311

AI974205 -0.396 -0.224 -0.26 -0.646 0.161 0.056 -0.21 -0.174 0.156 -0.19 0.462 0.587

AW171390 -0.351 0.054 -0.449 -0.241 -0.264 -0.116 -0.16 -0.477 -0.418 -0.196 0.462 0.403

AF180889 -2.938 -2.542 -1.676 -0.694 -3.392 -2.131 -1.994 -2.811 -2.393 -0.201 0.778 1.283

AF071496 -0.786 -0.035 -0.135 -0.628 0.091 -0.221 -0.181 0.069 -0.019 -0.204 0.966 1.528

AW281293 -0.893 -1.451 -0.856 -0.712 -0.959 -0.325 -0.183 -1.134 -0.451 -0.204 -0.102 0.77

BI882791 -2.34 -3.06 -0.442 -0.742 -0.084 -0.461 -0.358 -0.776 0.336 -0.218 0.432 0.079

AI626348 -0.35 -0.718 -0.735 -0.837 -1.027 -0.29 -0.182 -1.088 -0.446 -0.223 0.291 0.518

AW233556 -0.737 -0.039 0.017 0.097 -0.296 -0.403 -0.43 -0.285 -0.021 -0.225 0.043 0.807

BM026491 -0.836 -0.599 -0.53 -0.134 0.832 -0.044 -0.411 -0.411 -0.091 -0.231 0.777 0.575

AA605655 -0.728 -0.005 0.143 -0.51 -0.244 -0.416 -0.111 -0.33 -0.406 -0.236 0.008 1.482

AF060118 -0.761 -0.797 -0.555 -0.972 -0.633 0.103 -0.464 -0.201 0.066 -0.24 0.834 0.256

BG738534 -0.876 -1.376 -1.552 -1.342 -0.396 -0.284 -0.322 -0.664 -0.949 -0.244 -0.024 1.461

BI880136 -0.5 -1.189 -0.529 -0.753 -0.095 -0.173 -0.258 -0.479 0.155 -0.247 0.406 0.312

BI981066 -0.838 -0.802 -0.963 -0.398 0.004 -0.106 -0.461 -0.511 -0.206 -0.247 0.519 0.597

BI880170 -0.349 -0.054 -0.231 0.03 -0.252 -0.102 0.029 -0.421 -0.213 -0.25 0.109 0.823

BI840762 -1.311 0.002 -0.776 -0.45 -0.265 -0.282 -0.613 -0.856 -0.746 -0.251 0.275 2.068

BI891855 -1.777 -2.561 -0.856 -0.943 -0.315 -0.441 -0.043 -1.211 0.222 -0.253 0.396 0.13

AW595094 -0.055 -0.22 -0.715 -1.119 -0.556 0.063 -0.391 -0.507 -0.307 -0.253 0.253 1.34

AW826653 -0.593 -0.604 -0.399 -0.24 -0.109 -0.415 0.02 -0.455 0.282 -0.254 0.367 0.351

BG305568 -0.649 -1.432 -0.958 -1.096 -1.292 -0.365 -0.196 -1.338 -0.662 -0.262 -0.27 0.898

BI534295 -0.302 -0.16 0.223 -0.355 -0.043 0.146 -0.087 -0.47 -0.143 -0.268 0.555 0.56

BI982951 -0.739 -1.111 -0.897 -0.904 -1.172 -0.62 -0.674 -1.239 -0.474 -0.269 -0.019 0.623

U43658 -0.259 0.254 0.344 0.34 0.377 0.065 -0.043 0.021 0.216 -0.27 0.987 0.036

BI326643 -0.901 -1.378 -1.043 -0.745 -0.008 -0.345 -0.328 -0.76 0.061 -0.278 0.357 0.379

AI667241 -0.614 -0.087 -0.483 -0.448 -0.193 -0.499 -0.308 -0.667 -0.26 -0.289 0.585 1.009

AW019275 -1.221 -2.211 -0.596 -0.737 -0.188 -0.437 -0.242 -0.974 0.297 -0.293 0.013 0.428

AI601567 -3.082 -2.648 -0.939 -1.165 -0.426 -0.621 -0.905 -1.129 0.364 -0.312 0.473 0.26

BG307330 -0.184 0.053 0.096 -0.829 0.086 0.314 0.233 -0.219 -0.061 -0.314 0.655 0.207

BI863932 -1.902 -1.064 -1.738 -0.537 -1.776 -0.427 -1.045 -1.593 -1.029 -0.317 -0.176 1.566

AI584352 -1.853 -2.896 -1.132 -0.809 -0.347 -0.2 -0.326 -1.229 0.245 -0.334 0.315 0.532

AI641634 -1.56 -2.449 -0.737 -0.679 -0.167 -0.28 -0.223 -0.933 0.154 -0.354 0.311 0.292

BM154370 -0.63 -0.668 -0.843 -0.447 0.092 -0.026 0.029 0.191 -0.185 -0.359 0.333 0.644

BI841667 -0.607 -0.063 0.016 -0.243 0.103 -0.396 -0.216 -0.166 -0.398 -0.36 0.246 0.812

BE201395 -2.225 -4.034 -2.193 -3.84 -4.285 -1.064 -1.929 -4.284 -2.203 -0.365 0.497 2.327

AI658249 -0.525 -0.047 0.149 -0.51 -1.217 -0.349 0.073 -0.742 -0.407 -0.375 0.31 1.46

AI722354 -0.247 0.545 0.504 0.341 0.064 -0.205 0.051 0.193 0.048 -0.378 0.606 -0.082

AW279837 -2.401 -2.601 -0.686 -0.867 -0.009 -0.09 -0.67 -1.003 0.346 -0.393 0.46 0.3

BI982117 -1.596 -1.039 -0.707 -0.044 -0.099 -0.255 -0.648 -0.977 0.426 -0.397 0.661 0.576

AF180890 -2.656 -6.312 -1.701 -4.795 -5.838 -2.243 -2.459 -5.819 -2.443 -0.401 -0.283 0.891

AW826726 -0.395 -0.4 0.189 1.233 0.056 -0.444 0.256 -0.672 -0.643 -0.406 -0.823 1.03

BI839927 -1.594 -1.453 -2.144 -1.338 -1.4 -0.594 -1.089 -1.931 -1.114 -0.407 0.065 1.837

AW777717 -0.26 0.367 0.522 -0.229 -0.13 0.487 0.178 0.824 -0.37 -0.414 0.754 0.271

AI331606 -0.26 -1.349 -0.509 -0.978 -1.974 0.076 -0.714 -1.826 -0.757 -0.416 -0.337 1.483

BI880342 -0.179 0.19 0.185 0.228 0.162 0.147 0.309 -0.158 0.164 -0.419 0.086 1.778

AW171505 -0.723 -0.158 0.297 -0.701 -0.146 -0.134 -0.556 -0.285 -0.155 -0.422 -0.298 0.812

BG306150 -1.028 -1.311 -0.669 -0.556 -0.985 -0.761 -0.453 -1.686 -1.026 -0.434 0.147 1.506

AI545002 -0.728 0.07 0.001 -0.012 0.12 0.018 -0.294 0.264 -0.253 -0.445 0.079 0.694

AW826907 -0.448 -1.272 -0.307 -0.741 -0.22 -0.241 -0.183 -1.127 0.158 -0.453 0.422 0.22

BI880201 -0.353 -0.24 0.21 0.463 0.042 0.184 -0.097 -0.192 -0.066 -0.453 -0.015 0.968

BE693204 -1.552 -2.278 -2.064 -1.637 -2.103 -1.823 -1.123 -2.236 -1.454 -0.46 0.754 1.422

AW233684 -0.2 0.016 0.098 -0.351 -0.607 -0.335 -0.233 -0.323 -0.092 -0.478 -0.269 1.442

BG883325 -1.649 -0.661 -1.583 -1.699 -1.948 -0.215 -1.175 -1.512 -1.097 -0.482 0.275 1.626

BI865609 -1.249 -0.931 -0.229 -0.817 -0.646 -0.108 -0.153 -0.203 -0.24 -0.527 0.081 1.259

AW116863 -3.941 -2.372 -2.632 -1.828 -3.077 -2.975 -2.703 -2.872 -1.808 -0.528 -0.293 0.644

AI384655 -1.691 -1.564 -1.034 -0.488 -0.433 -0.418 -0.532 -1.088 0.038 -0.538 0.253 0.463

BM102022 -0.728 0.439 0.143 -0.535 -0.029 -0.161 -0.395 -0.114 -0.237 -0.545 0.402 1.501

AI965047 -2.765 -3.779 -2.306 -1.728 -3.807 -2.588 -1.639 -3.865 -1.881 -0.582 0.743 1.421

AF430840 -0.819 -0.481 0.028 0.266 0.057 0.05 -0.044 0.143 -0.21 -0.587 -0.029 0.913

BG305533 -0.885 0.091 -0.468 -0.192 -0.645 -0.199 -0.48 -0.708 -0.689 -0.606 -0.02 1.254

AW154075 -0.155 -0.652 -0.842 -1.192 -1.06 -0.234 -0.248 -0.893 -1.195 -0.608 -0.198 1.188

BI350696 -0.239 0.062 0.04 0.252 -0.668 -0.392 0.024 -0.057 0.112 -0.613 0.078 1.37

BM182680 -0.587 0.545 -0.02 0.341 0.097 -0.045 -0.003 -0.207 0.394 -0.632 0.423 0.913

BM156079 -0.702 -0.431 -0.73 -0.212 -0.927 -0.595 -0.442 -1.094 -0.42 -0.665 -0.335 1.745

AW826304 -1.031 -0.077 -1.014 -0.413 -1.015 -0.183 -0.518 -0.906 -0.616 -0.684 -0.261 1.5

BI866335 -0.701 -0.154 -0.025 -0.621 -0.763 -0.016 -0.555 -0.55 -0.13 -0.692 0.666 0.729

BI429006 -0.542 0.214 0.32 -0.367 -0.119 -0.042 -0.424 -0.131 -0.468 -0.708 -0.039 0.745

AJ245964 -0.812 -0.402 -0.403 -0.893 -0.244 -0.147 -0.451 -0.013 0.141 -0.728 0.425 1.917

AJ317957 -2.203 -2.176 -1.523 -1.589 -2.157 -1.172 -1.53 -2.458 -1.631 -0.743 -0.589 1.57

BI864920 -0.704 -1.545 -1.307 -0.933 -1.6 -0.64 -0.762 -1.528 -0.211 -0.748 -0.089 0.896

BF157011 -0.728 -0.037 0.277 -0.24 0.05 -0.332 -0.219 0.193 -0.182 -0.749 -0.125 0.942

BI704324 -2.499 -3.043 -2.506 -2.699 -2.57 -1.229 -1.429 -3.134 -1.655 -0.749 0.615 1.315

AI974174 -0.753 -0.031 -0.555 0.341 -1.775 -0.228 -0.379 -0.965 -0.494 -0.763 -0.059 1.16

AI626603 -3.014 -3.394 -1.296 -1.237 -0.6 -0.834 -0.941 -1.553 0.368 -0.767 0.39 0.412

AW232844 -3.529 -2.511 -1.069 -1.098 -0.558 -0.873 -1.008 -1.582 0.108 -0.785 0.187 -0.003

BM183857 -1.962 -2.772 -2.052 -1.539 -1.674 -1.625 -1.748 -1.647 -1.685 -0.79 -0.158 1.103

AF114262 -1.583 -1.935 -1.168 -0.925 -1.669 -0.838 -0.821 -0.889 -0.348 -0.797 -0.31 0.649

AI477656 -2.837 -2.541 -2.726 -2.208 -2.339 -1.241 -1.199 -2.91 -1.675 -0.838 0.67 1.67

AI722369 -1.634 -4.058 -1.963 -2.107 -2.634 -1.155 -1.471 -3.924 -1.597 -0.916 0.582 1.915

BI846231 -1.586 -2.519 -1.313 -2.333 -2.094 -0.479 -1.142 -2.751 -1.55 -0.96 -0.386 0.686

AI964276 -4.704 -4.884 -3.941 -3.54 -3.911 -2.741 -3.768 -5.221 -3.738 -1.064 0.367 2.267

AI601664 -3.851 -5.142 -4.7 -4.147 -4.55 -2.514 -2.505 -3.943 -3.246 -1.096 0.785 1.543

AF180892 -6.37 -6.683 -5.006 -5.412 -6.568 -5.087 -6.354 -6.553 -4.62 -1.118 -0.464 0.707

AF047837 -4.534 -3.707 -3.381 -3.489 -3.716 -2.618 -3.104 -3.535 -2.718 -1.352 -0.351 0.577

BI866326 -1.072 -1.154 -0.76 -1.47 -2.553 -1.016 -0.852 -2.017 -1.135 -1.357 0.19 1.484

H56788 -1.223 -1.502 -1.697 -0.61 -1.168 -0.418 -0.971 -2.071 -0.922 -1.369 -0.32 1.883

BM155853 -2.209 -1.565 -2.752 -1.168 -1.723 -2.121 -1.227 -2.024 -1.665 -2.593 -1.368 2.272

AI618133 -4.183 -6.661 -3.612 -4.65 -5.34 -3.359 -3.884 -5.997 -3.872 -3.063 -2.339 0.703

BM154199 -4.446 -4.19 -4.237 -4.168 -4.457 -2.868 -2.792 -4.815 -3.254 -3.974 -2.759 2.525

Mean -0.836 -1.054 -0.742 -0.57 -0.597 -0.085 -0.197 -0.148 -0.017 0.501 0.529 0.501
