# Supplementary material for: Transcriptome Analysis of Zebrafish Embryogenesis Using Microarrays
Source: PLoS Genet. 2005 Aug 26;1(2):e29. doi: 10.1371/journal.pgen.0010029 (PMC1193535; doi:10.1371/journal.pgen.0010029)
Supplement: Dataset S18 — (21 KB DOC) [file pgen.0010029.sd018.doc]

Dataset S18. Cell cycle related genes.

Genbank IDUF egg 3hpf 4.5hpf 6hpf 7.7hpf 9hpf 10.7hpf 12hpf 15hpf 24hpf 30hpf 48hpf

AF234784 0.81 1.692 2.084 1.562 0.464 0.17 -0.465 -0.333 -0.806 -0.907 -1.084 -1.411

AB040435 0.917 0.251 -0.593 -1.18 -2.03 -1.511 -2.238 -2.456 -3.02 -3.079 -3.34 -3.966

AW422010 1.794 1.917 0.438 -0.188 -1.51 -1.231 -1.644 -2.43 -2.169 -2.329 -2.958 -2.831

X87581 -1.892 -1.153 2.857 2.754 2.173 1.424 1 1.17 0.804 1.252 1.018 0.398

X83594 1.515 1.564 2.156 1.635 -0.206 -0.109 -0.701 -0.762 -1.16 -1.984 -1.912 -1.837

BI672128 -0.116 -1.089 0.076 0.661 1.062 0.083 0.349 0.444 0.234 -0.103 0.17 -0.32

AI959372 0.181 0.363 1.247 0.715 -0.182 0.047 -0.453 -0.365 -0.548 0.118 -0.376 -0.072

BE556826 -0.889 -0.21 -0.379 0.057 0.161 -0.007 -0.424 -0.074 -0.394 0.174 0.533 0.398

BE017206 -0.544 -1.539 -1.274 -0.933 -1.175 -0.334 -0.447 -0.814 -0.484 0.071 -0.083 0.429

BI878600 0.482 1.28 0.36 0.25 0.447 -0.053 0.158 0.862 0.168 0.249 0.088 -0.03

BG303647 0.795 1.098 1.247 1.363 0.91 1.352 0.62 0.589 0.348 0.344 -0.077 -0.175

AF398516 0.352 0.759 0.251 0.161 -0.465 -0.339 -0.74 -0.367 -0.09 -0.651 0.197 0.422

BI887574 -0.626 -0.374 0.745 -0.085 0.26 0.537 0.343 0.787 0.467 0.654 -0.021 0.28

AF203736 -1.08 -1.184 -1.121 -0.989 -0.739 -0.282 -0.598 -0.5 -0.797 -0.008 -0.202 0.341
