# Supplementary material for: Transcriptome Analysis of Zebrafish Embryogenesis Using Microarrays
Source: PLoS Genet. 2005 Aug 26;1(2):e29. doi: 10.1371/journal.pgen.0010029 (PMC1193535; doi:10.1371/journal.pgen.0010029)
Supplement: Dataset S19 — (24 KB DOC) [file pgen.0010029.sd019.doc]

"Dataset S19. Proteasomes, Ubiquitins."

Proteasome

Genbank IDUF egg 3hpf 4.5hpf 6hpf 7.7hpf 9hpf 10.7hpf 12hpf 15hpf 24hpf 30hpf 48hpf

AW174857 0.290 -0.631 -0.050 -0.074 0.383 0.506 0.360 1.174 0.454 0.642 0.457 0.329

AW305657 0.555 -0.608 -1.108 -0.496 -0.487 0.260 0.193 0.539 0.184 0.615 0.484 0.179

BI705544 0.317 -0.465 -0.094 0.183 0.449 0.833 0.434 0.731 0.321 0.570 0.341 0.445

BI670969 0.465 0.996 0.739 1.126 0.581 0.955 0.510 0.999 0.635 0.901 0.381 0.071

BI839952 -0.362 -0.750 -0.205 0.339 -0.353 -0.163 0.399 0.406 0.558 1.287 0.729 0.287

AI415997 0.073 -0.259 -0.303 0.179 0.277 0.174 0.069 0.648 0.116 0.228 0.081 0.239

BM183903 0.596 -1.367 -0.441 0.295 0.508 0.692 0.773 1.294 0.622 0.942 0.541 0.124

AF354750 -0.023 -0.284 0.184 0.206 0.451 0.198 0.372 0.968 0.506 0.800 0.341 0.120

AF354751 -0.421 0.036 -0.027 0.178 -0.013 -0.362 -0.122 0.356 -0.227 -0.541 -0.151 -0.180

AI330980 0.040 -0.956 0.079 0.091 0.229 0.476 0.678 1.175 0.363 0.939 0.873 0.564

AF155578 0.313 -0.514 0.390 0.130 0.558 0.931 0.461 1.235 0.989 1.446 0.664 0.471

AF155581 0.429 -0.426 0.606 -0.316 1.309 1.134 0.563 1.773 0.732 1.564 0.632 0.468

AF032390 0.068 -0.176 -0.144 0.403 0.290 0.101 0.243 0.716 0.168 0.529 0.040 0.108

AF155580 0.004 -0.319 -0.171 -0.016 0.181 0.325 0.177 0.416 0.120 0.264 0.191 -0.057

AW019161 0.842 0.819 -0.468 -0.726 -0.070 -0.107 -0.163 -0.007 -0.228 -0.296 0.057 -0.158

AF195050 0.330 0.929 0.930 0.580 0.403 0.088 0.438 0.365 -0.034 -0.059 -0.527 -0.631

Ubiquitins

Genbank IDUF egg 3hpf 4.5hpf 6hpf 7.7hpf 9hpf 10.7hpf 12hpf 15hpf 24hpf 30hpf 48hpf

BM185181 1.101 1.021 0.293 -0.484 -2.334 -0.481 -0.637 -2.931 -1.535 -0.703 -2.689 -1.968

BI878085 -0.526 0.415 0.407 0.288 0.202 -0.151 0.430 1.141 0.264 -0.131 0.569 0.335

AI437156 0.241 1.110 1.123 0.715 0.416 0.806 0.066 0.232 -0.235 0.094 -0.211 -0.225

BI868107 0.509 1.048 0.215 0.426 -0.042 -0.325 0.102 -0.069 -0.030 -0.251 -0.006 -0.153

BI984826 0.083 0.618 -0.166 0.178 -0.093 -0.144 0.057 -0.195 -0.197 -0.057 -0.181 0.084

BI878098 0.726 0.111 0.773 0.498 0.272 0.860 0.179 0.201 0.222 0.088 -0.167 -0.111

BI888920 -0.018 -0.318 0.553 0.721 1.323 0.898 0.612 0.937 0.519 0.467 0.201 -0.105

AW566976 0.188 0.745 0.519 0.082 -0.224 0.292 0.021 0.349 -0.022 0.385 0.160 0.398

AW154232 -0.296 0.659 0.611 0.590 0.107 0.036 0.302 0.461 -0.152 0.203 -0.139 -0.010

BM071353 0.631 0.304 -0.085 -0.112 0.182 0.730 0.684 0.874 0.422 0.583 0.286 0.026

AI444365 0.354 0.478 0.745 0.537 0.472 0.822 0.168 0.495 0.165 0.345 -0.111 -0.116

BI877866 0.366 0.664 1.018 1.156 1.034 0.352 0.335 0.594 0.302 0.265 -0.122 0.041

AW116617 1.164 0.874 1.055 1.172 1.148 2.020 0.900 1.451 0.382 0.559 0.270 -0.002

AW058706 -0.473 1.331 1.138 0.922 0.508 0.207 -0.299 -0.106 -0.554 0.097 -0.173 -1.137
