# Supplementary material for: Transcriptome Analysis of Zebrafish Embryogenesis Using Microarrays
Source: PLoS Genet. 2005 Aug 26;1(2):e29. doi: 10.1371/journal.pgen.0010029 (PMC1193535; doi:10.1371/journal.pgen.0010029)
Supplement: Dataset S20 — (24 KB DOC) [file pgen.0010029.sd020.doc]

Dataset S20 _ somitogenesis related genes

MSP genes

U F Egg 3hpf 4.5hpf 6hpf 7.7hpf 9hpf 10.7hpf 12hpf 15hpf 24hpf 30hpf 48hpf

AF180887 -2.082 -2.112 -1.135 -0.644 -1.186 -1.129 -1.242 -1.133 -1.035 0.026 -0.512 -0.335

BM024218 -1.947 -1.651 -1.583 -2.396 -2.695 -0.965 -2.058 -2.444 -0.922 -1.626 -2.833 -1.854

AF116824 -4.524 -3.421 -2.129 -1.307 -2.16 -2.301 -2.812 -2.49 -1.934 -0.393 -0.314 0.447

AF180892 -6.37 -6.683 -5.006 -5.412 -6.568 -5.087 -6.354 -6.553 -4.62 -1.118 -0.464 0.707

AY036972 -5.525 -5.003 -5.39 -3.697 -5.175 -4.103 -3.322 -5.857 -4.37 -1.882 -0.957 0.359

AI964276 -4.704 -4.884 -3.941 -3.54 -3.911 -2.741 -3.768 -5.221 -3.738 -1.064 0.367 2.267

BI983747 -1.206 -1.816 -2.165 -1.999 -2.578 -1.215 -1.311 -0.807 -0.727 0.237 -0.075 -0.18

AF180893 -4.328 -3.977 -3.292 -2.994 -3.241 -3.263 -3.476 -4.021 -3.658 -0.725 0.259 0.087

AF165817 -7.915 -7.061 -7.715 -6.179 -6.916 -6.409 -6.121 -7.305 -6.302 -4.811 -3.544 -2.009

AF081462 -5.224 -6.799 -5.081 -4.517 -6.043 -4.551 -4.107 -6.647 -4.078 -1.126 -0.595 0.265

AF180891 -7.556 -5.814 -6.298 -5.327 -6.805 -5.933 -5.671 -6.762 -5.449 -1.903 -1.116 0.493

AF180890 -0.328 -0.328 -4.071 -4.071 -4.997 -2.44 -2.314 -5.149 -2.45 -0.582 -0.271 0.912

AF180889 -2.191 -0.92 -1.02 -1.037 -1.788 -1.249 -1.192 -2.144 -1.81 0.065 0.742 1.188

Muscle specific transcription factors

Genbank iUF egg 3hpf 4.5hpf 6hpf 7.7hpf 9hpf 10.7hpf 12hpf 15hpf 24hpf 30hpf 48hpf

AB037939 -0.042 -0.056 0.62 0.536 0.633 0.168 0.309 0.368 0.06 0.012 -0.072 -0.104

AB037940 0.045 0.282 0.419 0.534 0.155 0.168 0.115 0.295 0.122 -0.071 0.067 0.12

AF219949 0.077 0.544 0.725 1.177 1.368 1.145 0.971 0.961 -0.017 0.532 0.324 0.582

BI880399 -0.187 -0.481 -0.233 -0.263 -0.095 -0.294 -0.17 -0.094 0.025 -0.112 0.167 0.069

U66569 -0.543 -0.687 -0.723 -0.25 -0.509 -0.259 -0.359 -0.779 0.021 -0.028 0.212 0.082

U66570 -0.401 -0.682 -0.692 -1.034 -0.838 -0.392 0.065 -0.039 0.065 0.45 0.457 -0.045

AF270789 -0.768 0.025 0.009 0.201 0.555 0.715 0.369 2.49 0.706 -0.086 0.024 -0.31

AF270789 -0.613 -0.003 -0.32 -0.664 -0.238 0.185 -0.287 0.175 0.472 1.872 1.163 0.51

AF240772 -0.202 0.399 0.768 1.508 1.093 0.81 0.371 0.595 0.345 0.005 0.411 0.108

X97333 0.005 -0.073 -0.19 -0.135 0.464 0.915 1.063 1.944 1.572 1.209 1.066 0.538

AF301264 0.201 -0.291 0.14 -0.001 0.398 0.204 0.41 1.173 0.461 0.382 0.801 0.251

AI794276 -0.143 -0.236 -0.23 -0.192 -0.092 -0.143 0.01 0.197 -0.051 1.285 1.26 1.13

X97330 -0.077 0.171 -0.234 -0.088 -0.21 -0.334 0.121 1.176 0.519 1.096 0.642 0.21
