# Supplementary material for: Transcriptome Analysis of Zebrafish Embryogenesis Using Microarrays
Source: PLoS Genet. 2005 Aug 26;1(2):e29. doi: 10.1371/journal.pgen.0010029 (PMC1193535; doi:10.1371/journal.pgen.0010029)
Supplement: Dataset S21 — (25 KB DOC) [file pgen.0010029.sd021.doc]

Dataset S21. Ribosomal protein genes.

Genbank IDUF egg 3hpf 4.5hpf 6hpf 7.7hpf 9hpf 10.7hpf 12hpf 15hpf 24hpf 30hpf 48hpf

BI891769 -0.880 -2.729 -1.832 -1.289 -0.963 0.421 0.146 0.206 0.299 0.784 0.577 0.042

AW077286 -0.713 -2.383 -1.037 -0.819 -0.578 0.427 0.208 0.088 0.387 1.103 0.776 0.126

BI881430 -0.305 -1.265 -0.280 -0.387 -0.358 0.654 0.221 0.364 0.285 1.066 0.611 0.208

AF385081 -0.914 -2.984 -0.501 -1.013 -0.722 0.082 0.173 0.302 0.075 0.859 0.660 0.183

BM153976 -0.481 -3.354 -1.545 -0.985 -0.782 0.432 0.108 0.438 0.260 1.035 0.651 0.173

AI878068 -1.035 -2.870 -1.539 -1.093 -0.573 0.381 0.111 0.427 0.270 1.034 0.561 0.176

BI886167 0.108 -2.879 -1.573 -1.302 -1.091 0.126 -0.170 0.157 0.120 0.956 0.595 0.143

BI707410 -0.587 -3.041 -1.196 -0.901 -0.492 0.487 0.330 0.498 0.231 1.061 0.627 0.072

BI673760 -0.001 1.029 0.336 0.106 0.153 0.010 -0.169 -0.122 0.426 -0.379 0.403 0.483

BI890906 -0.854 -2.499 -0.930 -0.976 -0.607 -0.024 0.213 0.320 0.139 1.214 0.734 0.286

BM096077 -1.651 -2.577 -1.345 -1.175 -1.015 -0.314 -0.757 -0.433 -0.847 0.280 0.311 0.382

AI106421 -0.405 -2.679 -0.973 -0.895 -0.423 0.048 0.400 0.622 0.317 1.361 0.739 0.218

AI353168 -1.170 -3.123 -1.685 -0.944 -0.667 0.354 0.063 0.253 0.300 0.877 0.585 -0.313

BI888897 -1.229 -2.824 -1.785 -1.181 -0.974 0.193 -0.091 0.092 0.039 0.735 0.348 -0.092

BG305988 -0.940 -3.359 -1.396 -1.333 -0.816 0.510 0.114 0.176 0.032 0.850 0.590 0.252

AI964218 -1.063 -2.966 -1.490 -1.100 -0.802 0.384 0.038 0.240 0.180 0.958 0.635 0.085

AW128744 -1.007 -2.859 -1.569 -1.220 -0.937 0.313 0.035 0.282 0.202 1.024 0.813 0.298

BI890218 -0.725 -2.578 -1.236 -1.145 -0.790 0.059 0.127 0.301 0.015 0.992 0.613 0.291

BI842921 -0.811 -2.677 -1.475 -1.214 -0.899 0.445 0.084 0.342 0.212 1.180 0.723 0.355

BI892430 -0.446 -2.368 -0.921 -0.770 -0.351 0.508 0.280 0.356 0.181 0.988 0.587 0.065

BM070699 -0.087 -2.361 -1.068 -0.649 -0.569 0.561 0.153 0.480 0.383 1.152 0.897 0.251

AI722592 -0.539 -1.219 -0.539 -0.498 -0.266 0.573 0.348 0.444 0.251 1.137 0.826 0.239

BM183474 -1.163 -2.775 -1.341 -0.840 -0.581 0.496 0.215 0.389 0.337 1.150 0.855 0.296

BM104738 -0.511 -2.579 -1.188 -1.129 -0.491 0.621 0.200 0.667 0.375 1.291 0.891 0.217

AF210641 -0.601 -1.359 -0.484 -0.131 -0.566 0.469 0.042 0.312 0.071 0.852 0.697 0.087

BM183623 -0.533 -3.251 -1.514 -1.026 -0.687 0.337 0.142 0.162 0.273 1.032 0.632 -0.118

BI318094 -0.654 -1.773 -1.007 -0.343 -0.466 0.407 0.197 0.292 0.131 0.953 0.497 0.204

BF717296 -0.574 -2.459 -1.426 -1.099 -0.962 0.169 -0.069 0.034 0.165 0.868 0.488 -0.020

BM156154 -1.247 -2.881 -1.119 -0.826 -0.929 0.237 -0.092 0.007 0.132 0.892 0.550 0.054

BM155568 -0.257 -1.979 -0.899 -0.654 -0.319 0.668 0.379 0.630 0.512 1.372 1.123 0.449

AI558833 -0.853 -3.190 -1.354 -1.176 -0.748 0.480 0.001 0.246 0.239 1.075 0.661 0.196

BE017895 -1.018 -2.981 -1.196 -1.049 -0.587 0.427 0.113 0.434 0.204 1.093 0.593 0.250

BI889445 -0.734 -2.851 -1.290 -0.810 -0.641 0.539 0.088 0.326 0.150 0.918 0.621 0.236

AI964216 -1.265 -2.280 -1.221 -0.904 -0.570 0.389 0.046 0.422 0.469 1.115 0.904 0.395

AF134852 -1.041 -4.135 -2.070 -1.404 -1.052 0.269 -0.205 0.105 0.178 1.069 0.826 0.299
