# Supplementary material for: Transcriptome Analysis of Zebrafish Embryogenesis Using Microarrays
Source: PLoS Genet. 2005 Aug 26;1(2):e29. doi: 10.1371/journal.pgen.0010029 (PMC1193535; doi:10.1371/journal.pgen.0010029)
Supplement: Dataset S22 — (22 KB DOC) [file pgen.0010029.sd022.doc]

Dataset S22. Gene expression dataset of pre-MBT and post-MBT stages_ maternal dominant gene.

Genbank ID1-4cell11-4cell2 1-4cell3 64cell1 64cell2 64cell3 4hpf1 4hpf2 6hpf1 6hpf2 6hpf3

BI880613 0.675 0.659 0.712 -1.143 0.439 -0.721 -0.058 -0.123 -0.512 -0.839 -0.661

AI397304 0.442 0.601 0.554 -0.355 -0.338 -0.417 -0.380 -0.580 -0.439 -0.652 -0.368

BI980138 0.394 0.491 0.424 -0.313 -0.335 -0.328 -1.123 -1.364 -0.733 -0.776 -0.993

BI864995 0.605 0.508 0.546 -0.359 -0.155 -0.310 0.593 -0.123 -0.428 -0.211 -0.087

AW344232 0.582 0.710 0.510 0.059 -0.123 -0.078 -0.898 -1.085 -0.535 -0.166 -0.447

AA497153 0.488 0.734 0.497 0.012 0.089 -0.169 -0.090 -0.515 -0.156 -0.240 -0.786

BI886968 0.474 0.666 0.500 0.055 0.034 -0.138 -0.575 -0.514 -0.933 -0.733 -0.916

AI959042 0.344 0.342 0.338 0.048 -0.007 -0.078 -0.424 -0.513 -1.983 -1.647 -2.260

BE016868 0.694 0.765 0.532 -0.011 0.091 0.008 -0.037 -0.453 -2.043 -1.841 -2.012

AW170975 0.668 0.692 0.482 -0.026 0.214 -0.075 -0.445 -0.370 -0.150 -0.160 0.025

AI722489 0.466 0.488 0.390 0.215 -0.051 0.123 0.086 -0.071 -2.474 -2.925 -2.652

BI887548 0.550 0.344 0.525 0.137 0.105 0.075 0.337 0.025 0.216 -0.250 -1.682

AW174136 0.920 0.537 0.814 0.161 0.009 0.160 -0.001 0.018 -0.096 -0.087 0.128

BI879867 0.739 0.685 0.794 0.118 0.114 0.126 -0.058 0.173 -0.053 0.154 0.488

BI891318 0.470 0.638 0.526 0.203 0.237 0.093 0.036 -0.490 0.285 0.258 0.134

AI942509 0.487 0.702 0.803 0.335 -0.114 0.389 -0.301 0.002 -0.744 -1.082 -0.985

AF025305 -0.420 -0.813 -0.024 0.197 0.198 0.225 0.983 0.622 0.636 0.509 0.851

AW019428 -0.009 -0.383 -0.109 0.957 0.869 0.878 1.323 1.120 1.157 0.985 0.985

AI883963 0.368 -1.717 -0.260 0.470 1.197 1.041 1.904 1.098 1.030 0.790 0.962
