# Supplementary material for: Transcriptome Analysis of Zebrafish Embryogenesis Using Microarrays
Source: PLoS Genet. 2005 Aug 26;1(2):e29. doi: 10.1371/journal.pgen.0010029 (PMC1193535; doi:10.1371/journal.pgen.0010029)
Supplement: Dataset S23 — (39 KB DOC) [file pgen.0010029.sd023.doc]

Dataset S23. Gene expression dataset of pre-MBT and post-MBT stages_ onset of expression from 64 cells onwards.

Genbank ID1-4cell11-4cell2 1-4cell3 64cell1 64cell2 64cell3 4hpf1 4hpf2 6hpf1 6hpf2 6hpf3

AA494839 -0.107 -0.343 -0.306 1.045 1.018 1.305 1.726 1.797 1.833 2.1 1.901

AA495420 0.548 -0.398 1.132 2.607 2.249 2.442 2.105 2.88 2.339 2.717 3.318

AA658756 -0.674 -0.85 -0.316 1.628 0.942 1.757 1.718 2.129 2.204 2.292 2.051

AB040044 -0.699 -0.549 -0.335 1.23 1.31 1.257 1.688 1.925 1.602 1.605 1.837

AF412832 0.17 0.176 0.488 1.732 1.531 1.69 1.889 2.133 2.215 1.985 1.825

AI416207 -0.145 0.113 0.749 1.86 1.01 1.852 1.11 1.356 1.578 1.484 1.356

AI437239 -0.297 -0.69 0.472 1.22 0.929 1.438 2.673 2.423 1.954 2.021 2.555

AI477962 0.167 0.008 1.155 1.408 1.328 1.37 1.718 1.697 1.904 1.934 2.128

AI545320 -0.035 0.162 -0.001 1.43 0.98 1.372 2.084 1.785 2.139 2.426 2.441

AI558322 0.069 -0.098 0.121 2.03 1.578 1.969 2.38 2.175 3.152 3.332 3.457

AI558431 -0.312 -0.414 0.353 1.24 1.206 1.058 1.263 1.643 2.375 2.24 2.071

AI584292 -0.306 -0.692 0.031 1.118 1.044 1.33 1.95 2.107 1.979 1.894 1.702

AI588361 -0.324 -0.26 -0.703 0.923 0.8 1.491 0.733 1.914 1.567 2.184 2.062

AI601527 0.411 0.526 0.709 1.351 1.5 1.398 1.382 1.464 2.058 2.252 2.083

AI601836 0.337 -1.24 -0.563 1.144 0.506 1.168 2.21 1.548 1.498 1.726 1.858

AI626820 -0.573 -1.074 -0.641 1.396 0.718 1.379 1.215 1.241 1.605 2.047 1.682

AI641775 0.009 -0.083 0.439 1.85 1.691 1.783 1.858 2.086 2.88 2.662 2.86

AI657670 0.243 0.213 0.475 1.205 1.216 1.01 1.765 1.595 1.625 1.691 1.711

AI721468 -0.489 -0.668 0.35 3 1.656 2.743 2.412 2.932 2.975 3.149 2.807

AI723191 -0.411 -0.518 0.131 0.945 1.049 1.202 0.835 0.893 1.681 1.326 1.689

AI793372 0.677 0.894 0.963 1.464 1.072 2.091 2.065 2.091 1.994 2.064 1.875

AI793498 -0.496 -0.488 0.762 2.149 2.007 2.399 2.172 2.503 2.705 2.98 2.835

AI793574 0.198 -0.952 0.023 1.531 1.557 1.585 2.308 2.516 3.393 3.502 3.523

AI878344 0.178 0.436 0.849 1.279 1.022 1.406 1.679 1.444 1.61 1.828 1.338

AI878755 0.756 0.687 1.687 2.015 1.816 1.936 2.537 2.344 2.307 2.345 2.638

AI878758 0.029 0.035 0.448 1.166 1.397 1.202 1.313 1.55 1.492 1.676 1.406

AI878774 0.073 -0.137 -0.442 1.178 0.619 1.274 0.711 1.099 0.895 1.069 1.029

AI959677 -0.694 -0.448 0.39 1.118 0.818 1.149 1.332 1.391 1.796 1.712 1.683

AW019011 0.005 0.243 -0.036 1.605 0.919 1.629 1.861 1.616 2.102 1.706 1.867

AW019116 0.21 -0.116 -0.05 1.354 0.757 1.139 1.825 1.793 2.001 2.09 2.39

AW019444 -0.826 -0.79 -0.171 2.153 1.585 2.059 2.414 2.255 1.787 1.977 2.24

AW076882 0.282 0.374 0.978 1.772 1.604 1.881 1.679 1.795 2.024 1.99 2.107

AW077428 0.366 0.49 0.576 1.83 0.879 1.829 1.201 1.725 1.698 1.77 1.577

AW115594 0.121 -0.194 0.6 1.474 0.555 1.649 1.781 2.256 1.788 1.985 1.715

AW115602 -0.988 -0.868 0.176 1.035 1.249 1.32 1.452 1.446 2.656 2.624 2.768

AW115732 -0.487 -0.589 0.226 1.406 0.796 1.178 1.498 1.704 2.129 1.957 1.84

AW115770 -0.132 -0.177 0.054 1.386 1.385 1.476 2.403 2.45 2.708 2.714 2.85

AW116396 0.249 0.254 0.228 0.916 1.029 1.252 1.624 1.666 1.552 1.679 1.722

AW116838 -1.872 -2.311 -0.665 0.875 1.057 0.832 1.114 1.35 1.515 1.186 1.505

AW117141 -0.685 -0.605 -0.724 1.609 1.367 1.216 1.924 2.429 3.865 4.523 4.186

AW128619 0.413 0.387 0.287 1.099 1.195 1.155 1.335 1.489 2.231 1.957 2.121

AW154620 0.306 0.138 1.194 1.732 1.606 2.128 3.03 3.076 2.926 2.4 2.759

AW154623 0.418 0.531 1.086 1.438 1.253 1.567 1.945 1.712 2.187 2.24 2.077

AW154770 0.446 0.249 0.519 1.205 1.38 1.463 1.345 1.647 1.282 1.649 1.585

AW165130 -0.609 -0.516 0.41 2.824 2.264 3.183 3.239 3.249 3.012 3.438 3.31

AW165251 -0.196 -0.24 0.18 2.425 1.728 2.077 1.822 1.962 2.949 2.983 2.662

AW170898 0.605 0.722 1.412 1.78 1.785 1.693 2.026 1.866 2.357 2.422 2.214

AW171290 0.556 0.343 0.832 1.429 1.464 1.4 1.427 1.602 1.427 1.668 1.496

AW171367 -0.531 -0.159 -0.797 1.488 1.14 1.53 1.354 2.428 2.268 2.602 2.404

AW171454 0.157 0.104 0.072 1.167 1.419 1.432 1.362 1.3 1.783 2.045 1.812

AW232166 0.075 -0.544 -0.045 1.175 0.786 1.55 1.746 1.523 1.499 1.559 1.503

AI626587 0.14 0.11 -0.16 0.98 0.83 0.7 0.78 1.9 1.16 1.31 1.33

AW232317 -0.236 -0.189 0.453 1.526 0.999 1.509 2.063 2.06 2.073 2.54 2.567

AW232489 0.097 0.092 0.233 1.491 1.486 1.481 2.151 2.201 2.172 2.647 2.38

AW232692 0.099 -0.197 0.781 2.139 1.942 2.139 2.947 3.196 3.361 3.398 0.918

AW281831 0.047 -0.371 0.365 1.626 1.468 1.255 1.305 1.467 1.852 1.443 1.737

AW305657 -0.851 -1.048 -1.026 1.539 0.821 1.482 1.994 2.235 2.734 2.684 2.751

AW343764 -0.021 0.093 0.875 2.353 2.426 2.338 3.264 2.997 3.489 3.587 -0.726

AW466488 0.746 0.483 0.845 1.898 1.777 2.054 2.542 2.532 2.402 2.334 2.628

AW777876 0.157 -0.006 0.445 1.632 1.443 1.581 2.206 1.813 1.74 1.896 1.95

AY029577 -0.241 -0.265 1.006 2.063 1.505 2.351 2.929 3.019 2.655 2.393 3.095

AY052752 -0.519 -0.284 0.054 1.697 1.627 1.334 2.143 1.931 2.08 2.159 1.777

BE017922 0.106 0.151 -0.048 1.303 1.35 1.07 1.577 2.131 1.753 1.853 1.732

BE200673 0.083 -0.088 0.639 0.928 1.134 0.953 1.252 1.377 1.137 1.395 1.209

BE201533 -0.037 0.386 0.686 1.755 1.683 2.023 2.066 2.432 2.447 2.835 2.535

BE202194 0.355 -0.178 0.147 1.723 1.256 1.373 1.483 1.745 0.828 1.555 1.467

BE557308 -0.102 -0.456 0.593 1.475 1.611 1.401 2.112 2.05 2.298 2.164 1.991

BE558184 -0.009 0.339 0.148 1.253 0.681 1.59 1.904 1.627 1.826 1.817 1.411

BE605436 -0.636 -0.505 0.263 2.526 1.855 2.387 2.359 1.842 2.376 1.924 2.387

BE605606 0.127 0.108 0.155 1.824 1.832 1.849 2.07 2.059 2.254 2.544 2.318

BE693153 0.048 0.241 1.021 1.63 0.875 1.914 1.538 1.568 2.237 1.544 1.924

BG302674 1.293 0.875 2.011 2.14 1.519 2.193 2.073 1.786 1.835 1.545 0.841

BG303462 0.479 -0.07 0.435 1.712 1.809 1.645 1.769 1.906 2.273 2.401 2.408

BG303497 0.128 0.04 -0.082 1.599 0.997 1.568 1.697 1.761 2.258 2.48 2.527

BG303647 0.152 -0.115 0.81 1.735 1.839 1.679 2.273 2.425 1.705 2.173 1.692

BG303764 0.784 0.849 1.326 2.043 1.668 2.039 2.045 1.497 2.356 2.551 2.621

BG304149 0.335 0.442 0.439 1.028 1.201 1.064 1.337 1.372 1.931 1.661 1.973

BG884085 -1.716 -1.717 -1.27 1.314 1.233 1.222 -0.058 1.352 1.774 3.361 2.725

BG884477 -0.149 0.122 0.434 1.316 1.241 1.501 1.755 1.572 1.781 2.173 1.992

BI428543 0.116 0.199 0.99 1.382 1.246 1.237 2.1 1.977 1.561 2.014 1.651

BI429526 0.422 -0.791 -0.71 1.212 0.813 1.461 0.953 1.272 1.801 1.816 1.766

BI533270 0.008 -0.513 -0.071 1.337 1.101 1.373 1.947 2.082 2.167 2.165 1.851

BI672345 0.265 0.359 0.836 1.262 0.94 1.606 1.431 1.531 1.56 1.968 1.875

BI673358 -0.31 -0.62 0.04 1.66 1.68 2.84 2.13 3.38 1.71 1.48 2.81

BI673573 -0.513 -0.953 0.467 2.206 1.702 2.108 2.254 2.765 4.317 4.401 3.935

BI710394 -0.132 -1.717 -1.458 1.481 0.62 1.427 2.042 1.138 2.052 2.011 1.928

BI844046 0.258 -0.212 0.39 1.287 1.211 1.229 1.716 1.596 1.598 1.368 1.675

BI867099 0.25 0.183 1.477 2.752 1.554 2.652 3.111 3.438 2.858 3.702 3.122

BI877552 -0.215 -0.55 -0.032 1.824 1.201 1.811 2.107 2.409 3.181 3.669 3.285

BI877740 0.301 0.352 -0.01 1.664 1.133 1.824 2.328 2.542 2.117 2.333 2.053

BI877866 0.087 0.366 1.137 1.816 1.734 1.823 2.174 2.316 2.344 2.369 2.125

BI877917 0.738 0.241 0.99 1.478 0.924 1.62 1.914 1.55 1.785 1.279 1.674

BI877938 0.011 0.05 0.55 1.266 0.886 1.296 1.344 1.306 1.531 1.291 1.538

BI878078 -0.136 0.06 0.204 1.352 1.64 1.171 1.492 2.098 2.813 3.151 3.077

BI878826 -0.368 -0.559 -0.13 1.661 0.595 1.383 0.951 1.837 1.618 2.091 1.614

BI878979 0.744 0.787 1.505 1.657 1.433 1.877 1.891 2.165 2.192 2.459 1.879

BI882131 -0.056 -0.6 -0.04 1.216 0.736 1.771 1.766 1.729 2.158 2.148 1.953

BI887935 0.066 -0.192 0.666 1.435 1.342 1.578 2.111 2.247 2.917 2.657 2.611

BI888169 -0.143 -0.211 0.019 1.258 0.632 1.486 1.952 2.327 2.368 1.557 2.106

BI888253 0.056 0.281 0.443 1.621 0.611 1.616 1.695 1.793 1.459 1.246 1.605

BI888458 -0.09 -0.176 0.217 1.345 0.748 1.478 1.948 1.922 1.514 1.899 1.753

BI888748 0.257 0.153 1.03 1.505 1.568 1.447 1.788 1.906 2.054 2.216 2.448

BI889463 -0.384 0.032 0.048 1.697 1.331 1.431 1.628 1.707 1.936 1.898 2.054

BI887620 0.28 0.24 0.8 1.7 0.71 2.19 1.89 2.03 3.24 3.9 3.47

BI890375 0.618 0.568 1.162 2.088 2.018 1.995 2.385 2.189 2.098 1.766 2.063

BI891065 -0.914 -1.055 -0.402 1.431 1.39 1.411 1.292 1.662 1.993 1.766 1.945

BI891591 0.561 0.101 -0.464 0.885 1.372 0.956 1.341 1.391 2.074 1.862 1.635

BI891643 -0.38 -0.212 0.348 1.593 1.528 1.571 1.827 1.618 1.36 1.341 1.234

BI896231 -0.901 -0.828 0.463 1.595 1.5 1.451 2.32 2.192 3.466 3.244 3.337

BI979955 -0.734 0.374 0.572 1.839 1.737 2.259 2.195 2.133 2.749 2.599 -0.275

BI980472 0.23 0.218 0.232 1.396 1.389 1.418 1.452 1.74 1.541 1.696 1.498

BI980644 0.52 0.13 0.07 1.5 1.27 1.15 2.26 2.54 2.38 2.53 2.69

BM005062 0.003 -0.353 0.705 2.29 2.52 2.079 2.274 2.287 1.938 2.009 1.98

BM005100 -0.23 -0.05 0.56 1.26 1.05 1.42 1.65 1.72 1.57 1.58 2.32

BM023680 0.65 0.809 1.79 2.901 2.442 2.584 2.591 2.522 3.952 3.631 3.351

BM071885 0.649 0.424 1.325 1.866 1.147 2.246 1.792 1.625 2.507 2.546 2.693

BM096095 -0.028 -0.021 0.524 1.252 0.823 1.148 1.625 1.562 1.619 1.693 1.432

BM101651 0.281 0.149 0.643 2.02 1.557 1.863 3.035 3.238 3.512 3.769 3.617

BM104313 -0.13 0.041 0.162 1.217 1.214 1.115 1.558 1.49 1.712 1.291 1.475

BM172681 -0.399 -0.224 -0.206 1.171 1.042 0.859 1.071 1.418 1.606 1.602 1.636

BM183919 -0.636 -0.863 -0.045 1.25 1.298 1.522 0.642 1.582 1.406 2.065 1.786

BM184030 0.092 0.156 0.687 1.981 1.527 1.689 1.455 1.778 1.538 1.353 1.622

BM184694 -0.007 -0.001 0.041 1.191 1.143 1.114 1.663 1.695 2.52 2.636 2.378

D38454 0.533 0.634 1.499 2.376 2.33 2.404 2.876 2.664 2.315 2.236 2.65

U85091 0.187 0.492 1.334 2.015 1.925 2.092 2.072 2.198 0.849 0.624 1.039
