# Supplementary material for: Transcriptome Analysis of Zebrafish Embryogenesis Using Microarrays
Source: PLoS Genet. 2005 Aug 26;1(2):e29. doi: 10.1371/journal.pgen.0010029 (PMC1193535; doi:10.1371/journal.pgen.0010029)
Supplement: Dataset S24 — (40 KB DOC) [file pgen.0010029.sd024.doc]

Dataset S24. Gene expression dataset of pre-MBT and post-MBT stages_ onset of expression from 4hpf onwards.

Genbank ID1-4cell11-4cell2 1-4cell3 64cell1 64cell2 64cell3 4hpf1 4hpf2 6hpf1 6hpf2 6hpf3

BI474299 -1.06 -1.717 -0.312 -0.5 -0.573 -0.021 2.149 1.859 3.738 4.087 4.073

BM187315 -0.207 -1.621 -1.458 -0.419 -0.726 -0.758 1.873 2.199 2.685 3.222 2.946

AW174857 0.078 0.14 0.28 0.854 0.762 1.173 2.027 2.066 1.988 1.898 2.279

AB055664 0.159 -0.797 -1.458 0.583 0.053 0.511 2.171 1.961 2.354 2.393 2.311

BI318628 -0.131 -0.404 -0.049 0.892 0.085 1.317 2.152 2.018 1.254 1.84 1.931

BM103861 0.028 -0.235 0.365 1.249 0.711 1.264 1.875 2.315 2.764 2.778 2.534

U18312 0.075 -1.019 0.602 -0.393 -0.311 -0.524 1.938 2.285 3.001 3.378 3.564

BI672045 0.265 -0.092 0.131 1.191 0.353 1.206 2.006 2.232 2.574 2.52 2.644

AI943149 -0.106 -0.976 -0.479 -0.047 -0.115 -0.346 2.099 2.15 2.749 2.827 3.001

BG303312 -0.022 0.103 0.392 0.981 0.786 0.925 1.86 2.396 3.157 3.422 3.156

BI704293 0.285 0.506 0.178 0.082 -0.275 0.157 2.219 2.073 2.868 3.226 2.972

AA495267 -1.675 -1.717 -1.458 -0.728 -0.758 -0.968 2.197 2.095 3.977 4.3 4.14

BI878085 -0.086 0.4 0.139 0.919 0.516 0.999 2.246 2.05 1.908 1.991 2.025

AW154707 -0.774 -0.564 0.033 0.835 0.583 0.808 2.207 2.101 2.762 2.479 1.954

AI444184 -0.104 -0.24 0.581 0.488 -0.009 0.568 1.902 2.41 1.72 2.159 1.892

BG727421 -1.837 -3.329 -2.603 -1.512 -0.635 -1.56 2.346 2.014 3.988 4.164 3.787

BI980148 0.329 -0.53 0.791 0.604 0.213 0.381 2.47 1.897 3.377 3.788 3.875

BG305930 -1.716 -1.717 -1.458 -0.709 0.274 0.013 2.317 2.094 4.246 4.497 4.313

BE016419 0.053 0.018 0.737 -0.237 0.113 -0.313 1.969 2.505 2.101 1.933 2.473

BI325955 0.028 -0.668 0.115 1.007 0.501 1.237 2.008 2.481 2.597 2.676 2.475

AW282142 -0.055 -0.159 -0.797 -0.012 -0.189 -0.199 2.127 2.398 4.197 4.326 4.273

AA497205 0.053 0.543 0.865 0.28 0.225 0.692 2.01 2.527 4.059 4.436 3.955

BG985522 -0.184 -1.032 -0.608 -0.127 0.291 -0.31 2.336 2.211 2.457 2.306 2.29

AI793839 -0.131 -0.037 -0.27 0.649 0.465 0.3 2.255 2.298 3.197 4.062 3.428

BI670852 0.154 -0.976 0.436 0.712 0.598 0.941 2.178 2.383 2.817 2.971 2.984

AI331812 -0.073 -0.461 -1.075 -0.207 0.049 0.083 2.344 2.258 2.874 2.622 2.811

AI477017 -0.032 0.075 -0.174 0.892 0.946 0.987 2.383 2.225 2.472 2.721 1.933

BI890858 -0.859 -0.978 -1.219 -0.064 -0.217 -0.161 2.082 2.608 1.779 2.145 1.891

BI476025 0.207 0.385 0.982 1.099 0.661 1.408 2.274 2.425 2.216 2.52 2.299

AI666982 -0.692 -0.757 -0.959 0.04 0.197 -0.007 2.273 2.532 3.198 3.296 3.383

BI878279 0.189 -0.093 0.618 0.869 0.386 1.127 2.356 2.473 1.605 2.308 2.23

BI887717 -0.241 -0.104 0.749 0.917 0.115 1.026 2.143 2.725 2.522 2.682 2.297

BI980644 0.24 0.126 0.129 1.461 1.209 1.156 2.479 2.405 2.105 2.509 2.666

AW076666 -0.157 -0.194 0.62 1.177 0.286 1.332 2.182 2.704 2.341 2.066 2.361

BI887656 -0.004 -1.717 -0.141 -0.524 -0.768 -0.613 2.455 2.435 2.967 3.322 3.144

AF168007 0.247 0.036 0.119 0.799 0.222 1.273 2.454 2.449 3.676 3.696 3.597

BI671227 0.306 -0.845 0.496 0.073 0.112 0.017 2.39 2.521 2.34 3.169 2.852

BI880447 -0.062 -1.717 -1.458 -0.514 -0.145 -0.629 2.257 2.665 3.255 3.721 3.248

BI867531 -0.173 0.078 0.87 1.348 0.934 1.531 2.375 2.632 2.487 2.885 2.72

AW019523 0.046 0.263 0.808 0.22 0.109 0.423 2.21 2.807 3.436 3.271 3.209

BI888891 0.02 -0.34 0.943 -0.103 -0.049 -0.01 2.846 2.197 2.647 3.036 2.925

BI839904 -1.716 -1.717 -1.458 0.001 -0.998 0.826 2.422 2.737 2.254 0.833 2.319

BI886755 -0.204 -0.972 -0.614 0.343 0.095 0.189 2.29 2.903 2.924 3.299 2.87

BI891474 -0.249 0.184 0.792 0.022 -0.094 -0.032 2.596 2.598 2.336 2.782 2.374

X65062 -0.586 -0.391 0.091 -0.27 -0.096 -0.242 2.321 2.875 2.596 3.32 2.509

AI601443 -1.104 -1.717 -1.458 -0.017 -0.183 0.115 2.368 2.886 4.767 4.168 4.821

BG727339 0.159 -0.387 0.032 0.656 0.28 0.389 2.786 2.546 4.292 4.284 4.379

AW019450 -0.472 -1.717 1.109 -0.201 0.219 -0.03 2.647 2.714 3.168 3.045 3.046

BI850015 -0.345 -0.833 -0.614 0.012 -0.283 0.105 2.306 3.065 2.568 3.492 2.695

BI889280 0.076 -0.907 -1.458 0.149 -0.418 0.118 2.521 2.894 3.885 3.955 3.909

BI891768 0.202 0.033 -0.328 -0.258 -0.508 -0.486 2.419 3.008 3.587 3.214 3.647

BI890439 0.493 -1.717 0.098 -0.123 -0.225 0.185 2.403 3.115 5.223 5.491 5.42

AI721701 0.154 -0.222 -0.073 -0.227 -0.241 -0.162 2.665 2.899 2.532 2.536 2.219

AF072456 -0.474 -0.085 -0.174 -0.047 -0.085 -0.277 2.641 2.929 2.39 2.653 2.306

BM096343 0.482 0.59 1.32 1.814 0.485 1.624 2.484 3.153 2.877 3.007 3.087

AW019725 -0.419 -0.354 0.022 0.391 0.499 0.345 2.941 2.746 3.584 3.294 4.044

BI878962 0.332 0.392 1.559 1.375 1.467 1.617 2.626 3.066 2.765 2.654 2.802

BI891666 0.098 -1.418 -1.458 -0.121 -0.797 -0.529 2.669 3.053 4.09 4.681 4.435

BI890462 -0.603 0.083 0.414 0.092 0.179 -0.12 2.571 3.164 2.801 3.323 3.113

AF255044 -0.18 0.265 -0.09 -0.289 -0.157 -0.332 2.657 3.098 3.113 3.617 3.635

AF201451 -0.709 -1.064 -1.215 -0.526 0.059 -0.518 2.87 2.947 3.065 3.239 3.228

AL591442 0.78 0.428 0.675 -0.426 -0.615 -0.3 2.902 2.954 4.79 4.859 5.133

AF052245 -1.716 -0.882 -1.458 -0.542 0.296 -0.025 2.862 3.022 5.617 6.094 5.684

BI889166 0.182 -1.717 -0.897 -0.202 -0.205 -0.096 2.795 3.104 5.593 5.616 5.646

AF359425 0.501 0.296 0.761 -0.234 -0.468 -0.327 2.736 3.215 4.374 4.194 4.24

BG884388 0.458 -0.992 0.824 0.275 -0.098 0.137 2.862 3.12 4.199 4.569 4.381

AJ315468 0.382 -1.132 0.027 -0.333 -0.293 -0.141 2.852 3.174 3.476 3.468 3.418

AI544607 0.052 -1.706 -1.4 -0.174 0.071 -0.556 2.937 3.178 4.057 4.238 4.326

AI883283 0.199 -0.663 -0.032 0.136 -0.075 -0.111 2.782 3.338 1.71 2.475 2.726

BI672412 -0.144 -1.717 -1.458 -1.143 -0.998 -0.721 2.849 3.359 4.322 4.707 4.503

AW019294 -1.716 -1.717 -1.458 0.122 0.002 0.51 2.916 3.294 4.424 4.895 4.503

AF392995 -1.314 -1.118 -1.04 -0.137 0.067 0.275 2.776 3.457 3.672 3.448 3.562

AF262978 -0.368 -0.789 -1.458 -0.654 -0.34 -0.101 3.374 2.916 2.672 3.205 2.872

BI982141 -1.716 -1.717 -1.458 0.007 -0.2 0.412 2.935 3.355 4.676 5.198 5.041

BM101651 0.333 0.115 0.742 2.007 1.507 1.908 3.087 3.206 3.645 3.549 3.5

BM184161 -1.356 -1.717 -1.458 0.146 -0.415 -0.42 2.791 3.504 4.747 4.692 4.734

BI886316 -0.836 -1.24 -1.458 -0.144 0.116 0.367 3.422 2.916 4.263 4.647 4.923

BI886271 0.016 -0.285 -0.907 -0.16 -0.001 0.395 3.256 3.087 2.952 3.396 3.267

BI888750 -0.007 -1.717 -1.458 -0.138 -0.381 -0.287 3.165 3.189 3.351 4.68 5.001

AF336123 0.108 -0.251 0.635 -0.269 -0.415 0.041 3.016 3.368 3.845 5.184 5.111

BI890191 0.073 -1.717 -1.458 -0.134 -0.312 0.044 2.937 3.449 4.517 6.182 5.865

U23839 0.108 -0.201 0.237 0.042 -0.257 -0.3 3.088 3.299 3.566 3.237 4.127

AW184237 -0.757 -1.717 -1.458 -0.446 -0.745 0.254 3.09 3.344 4.87 5.432 5.402

L48017 -0.001 -0.396 0.913 -0.204 -0.161 -0.391 3.044 3.414 3.052 3.78 3.625

AF052251 -1.716 -1.717 -0.132 -0.153 0.011 0.049 3.211 3.309 5.43 5.711 5.333

U27121 -0.424 -0.646 -0.814 -0.209 -0.238 0.09 3.097 3.538 4.37 3.964 3.971

BI886791 -1.716 -1.717 0.309 -0.208 -0.196 -0.359 3.356 3.297 5.092 5.472 5.936

BI886789 0.019 -0.049 0.217 -0.502 -0.425 -0.543 3.402 3.254 2.491 2.312 3.271

BI886431 0.254 -1.717 -1.458 -0.234 -0.176 -0.01 3.316 3.429 5.047 5.691 6.09

AJ245492 0.217 -0.913 -0.774 -0.167 -0.361 -0.299 3.258 3.538 4.377 4.307 4.438

BI880524 -0.202 -0.596 -0.065 -0.668 -0.514 -0.849 3.557 3.356 3.938 4.128 4.305

BI892210 -1.716 -0.425 -1.16 -1.306 -0.088 -0.248 3.199 3.802 6.065 6.628 6.042

BI888344 0.318 -0.05 0.434 -0.192 -0.326 -0.214 3.217 3.809 5.567 5.547 5.466

AI477969 0.161 -1.717 0.095 0.093 0.064 0.246 3.435 3.832 3.747 3.955 3.867

BI885653 -0.453 0.126 -0.584 -0.347 -0.448 -0.448 4.091 3.424 4.725 3.954 4.862

AI353412 0.501 -0.297 0.876 0.079 -0.04 -0.079 4.149 3.412 6.058 6.75 6.433

U85090 -0.203 -0.017 -0.214 -0.44 -0.28 -0.378 3.396 4.318 3.484 3.424 3.702

BI880833 -1.716 -1.717 -1.458 -0.051 0.013 0.059 3.752 4.06 4.56 4.978 4.742

AI959735 0.442 0.126 0.965 0.09 0.196 0.113 3.721 4.175 4.407 4.782 5.17

BI865754 -0.315 -1.717 -1.458 0.884 0.397 0.781 3.762 4.23 4.781 5.486 5.268

BI704420 -0.606 -0.228 0.549 0.372 0.534 0.405 3.985 4.12 3.403 3.99 4.007

AL590147 -0.498 -1.053 -0.724 -1.094 -0.086 -0.978 3.885 4.227 4.674 5.066 5.211

AW422093 -0.003 -0.198 0.647 0.15 0.082 0.277 3.666 4.461 3.511 3.139 3.805

AW421066 0.472 -1.717 0.937 -0.773 -0.218 -0.699 3.861 4.373 4.592 4.855 4.286

AF007414 0.023 -0.096 -0.01 1.254 1.041 1.118 4.025 4.292 4.221 4.457 4.398

BI888338 -1.716 -1.717 -1.26 0.048 -0.384 0.426 3.949 4.39 5.273 5.543 5.924

AF398433 0.291 -0.268 -0.782 -0.288 0.545 -0.343 3.755 4.72 3.945 3.426 3.769

BE201475 -0.625 -0.646 -1.284 -0.222 0.202 -0.036 4.255 4.255 4.609 4.522 4.45

AF077225 0.311 -0.139 0.113 -0.693 -0.433 -0.705 4.436 4.135 4.967 4.531 5.072

X79821 -0.561 -1.717 -1.458 0.542 -0.034 0.326 4.001 4.856 5.479 5.489 6.132

BI673712 -0.57 -1.717 -1.458 -0.162 -0.015 -0.154 4.497 4.763 6.191 6.26 6.732

BG985460 0.293 -0.069 -0.224 0.676 0.795 0.65 4.424 5.09 4.294 4.299 4.336

BM024762 0.117 -0.685 -0.099 1.053 0.636 0.802 4.182 5.399 4.097 4.56 4.686

AF207751 0.338 -0.009 0.777 0.278 -0.296 0.565 4.504 5.087 5.397 4.974 5.807

BG985498 0.187 -0.978 -0.648 -0.042 -0.054 -0.043 4.891 4.778 5.255 5.291 5.75

BI887718 0.217 -1.717 -1.458 -0.291 0.312 -0.037 4.825 4.996 4.044 3.944 4.89

BG985680 -0.866 -0.768 0.194 0.318 0.277 0.59 4.506 5.351 5.45 4.884 5.077

AF193837 -0.258 -1.717 0.428 -0.654 -0.172 -0.721 5.164 5.143 5.107 5.035 5.516

BI890505 0.219 -0.06 0.566 -0.1 0.197 0.087 4.95 5.37 4.108 3.957 4.813

BI888360 0.22 -1.717 -1.458 0.018 0.257 0.206 5.118 5.358 4.737 4.722 5.116

AI667275 -1.017 -1.026 -0.036 0.183 -0.425 0.097 4.88 5.606 4.714 3.648 5.184

AW058848 0.221 0.004 1.189 -0.106 -0.085 -0.164 5.665 5.031 4.955 5.069 5.228

X87581 0.235 -0.228 0.717 -0.592 -0.163 -0.33 5.453 5.852 5.23 5.246 5.548

BI892136 0.136 -0.478 0.091 -0.085 -0.149 -0.163 5.4 5.914 6.215 6.882 7.276
