# Supplementary material for: Transcriptome Analysis of Zebrafish Embryogenesis Using Microarrays
Source: PLoS Genet. 2005 Aug 26;1(2):e29. doi: 10.1371/journal.pgen.0010029 (PMC1193535; doi:10.1371/journal.pgen.0010029)
Supplement: Dataset S25 — (100 KB DOC) [file pgen.0010029.sd025.doc]

Dataset S25. Gene expression dataset of pre-MBT and post-MBT stages_ onset of expression from 6hpf onwards.

Genbank ID1-4cell11-4cell2 1-4cell3 64cell1 64cell2 64cell3 4hpf1 4hpf2 6hpf1 6hpf2 6hpf3

AW421080 0.030 0.002 0.818 -0.354 0.020 -0.087 -0.058 -0.123 2.061 2.056 1.889

BI891791 0.191 -0.031 -0.195 0.450 0.393 0.579 0.967 1.040 2.024 2.024 1.976

BI475971 -1.095 -1.717 -1.458 -0.084 -0.074 -0.020 -0.058 -0.117 2.343 1.900 1.790

BI867819 -0.109 0.088 -0.226 0.083 -0.032 0.057 0.616 0.731 2.245 2.194 1.598

AI878386 0.234 0.544 0.302 0.263 0.201 0.249 0.791 0.802 2.021 2.033 1.999

AI942574 -0.976 -1.535 -1.087 -0.512 -0.213 -0.483 -1.032 -0.643 2.026 1.874 2.162

AI722366 0.533 0.429 -0.412 -0.271 -0.175 -0.090 0.889 0.770 2.327 1.740 1.995

BG883236 0.204 0.040 -0.747 -0.047 0.565 0.053 -0.590 -0.250 2.134 1.945 1.995

AI793909 -0.284 -1.717 -0.453 0.182 0.381 0.853 0.576 -0.123 2.049 2.067 1.960

BM101667 -0.544 -0.544 -0.552 0.832 0.720 0.728 0.712 0.589 2.014 1.999 2.063

BI886127 -0.012 -0.058 -0.565 -0.221 -0.083 -0.341 -0.177 -0.036 2.121 1.880 2.084

AI793487 -0.649 -0.454 -0.400 -0.565 -0.339 -0.075 0.391 -0.123 2.168 1.876 2.042

AW115956 0.398 -0.121 0.065 0.516 0.623 0.722 -0.022 0.337 2.016 1.709 2.372

AA497337 -0.294 -0.920 -0.621 -0.021 0.189 -0.046 0.174 0.649 2.103 2.084 1.911

BG303391 -0.289 -0.740 -0.175 0.868 0.695 0.923 0.900 -0.123 2.150 1.958 1.996

BI890730 -0.136 -0.465 -0.961 0.163 0.069 -0.002 -1.094 -0.421 2.098 1.912 2.133

BI886106 -0.379 -0.629 -1.444 -0.262 -0.450 -0.369 0.455 0.645 2.314 1.971 1.863

AI544512 0.011 0.142 0.846 0.034 -0.092 -0.167 -0.873 1.092 2.189 2.049 1.924

BM036954 -0.350 -0.085 -0.483 0.040 0.091 0.094 0.159 0.432 2.127 1.942 2.122

AI476945 -0.127 -0.390 -0.181 0.446 0.517 0.488 0.474 0.379 2.113 1.898 2.181

AI601339 0.244 0.161 -0.417 -0.259 -0.113 -0.109 0.201 -0.297 2.022 2.068 2.103

AI793880 0.114 0.031 0.248 0.485 0.160 1.178 1.042 1.014 2.229 2.029 1.940

AB046866 0.050 0.190 -0.023 0.876 0.809 0.893 0.769 0.994 2.004 2.102 2.127

AI584379 -0.213 -0.559 -0.562 0.144 0.175 0.138 0.242 -0.216 2.157 2.051 2.029

AI793355 -1.716 -1.717 -1.458 -0.328 -0.264 0.207 0.301 -0.123 2.112 2.307 1.820

BG303586 -1.177 -1.162 -1.458 0.173 0.115 0.045 0.148 0.607 2.002 2.078 2.162

BI840456 0.428 -0.096 0.176 0.332 0.318 0.397 0.647 0.944 2.150 1.834 2.273

AI397334 -0.705 -0.495 -0.633 -0.363 -0.191 -0.148 -0.189 0.413 2.036 2.112 2.131

BI892155 0.049 0.280 0.015 -0.115 0.148 0.000 0.798 0.652 2.033 1.690 2.581

BI886735 0.878 1.168 1.350 1.660 1.469 1.565 1.441 1.418 2.063 2.372 1.870

BI890361 0.126 0.055 0.088 -0.176 -0.033 0.117 0.648 0.147 2.278 1.775 2.262

AI793853 -0.179 -0.306 -0.620 0.053 0.279 0.064 0.223 0.653 2.013 2.374 1.933

BI878031 -0.668 -0.767 -0.961 0.154 0.061 -0.372 0.046 0.147 2.010 2.279 2.035

BI710046 -0.212 -0.593 -0.248 0.379 0.044 0.630 0.406 0.570 2.179 2.165 1.997

AF030560 0.195 -0.144 -0.067 0.019 -0.231 0.009 -0.062 0.685 2.003 1.989 2.388

BI889130 0.581 0.572 0.370 -0.100 0.176 0.164 0.162 0.326 2.139 2.309 1.932

BI840867 0.183 -0.985 -0.229 0.000 -0.099 0.076 1.329 0.448 2.106 2.123 2.156

BM155067 -0.937 -1.323 -1.826 -0.293 -0.022 -0.298 0.618 0.432 2.137 1.954 2.299

AJ005936 0.222 0.368 0.785 0.281 0.309 0.335 0.697 0.419 2.267 2.106 2.033

AI721660 0.448 0.296 0.805 0.026 0.021 0.109 0.428 -0.123 2.228 2.351 1.832

BG307354 -1.716 -1.717 -1.458 -0.958 -0.249 -0.721 0.108 -0.123 2.413 1.895 2.110

AI794243 -1.332 -1.209 -1.448 -0.806 -0.672 -0.866 -0.434 -0.675 2.123 2.178 2.150

BG727233 -0.177 -1.717 0.094 -0.582 -0.673 -0.458 0.315 0.375 2.102 2.288 2.070

BI888810 -0.066 -0.195 -0.273 0.570 0.240 0.495 -0.081 0.063 2.051 2.167 2.257

AF146429 0.176 0.048 -0.127 -0.235 0.057 -0.191 -0.009 0.019 2.008 2.518 1.957

BI890050 -0.136 -0.102 -0.054 0.495 0.871 0.440 0.667 0.230 2.087 2.202 2.215

AI497342 -1.716 -1.717 -1.458 -0.010 -0.093 0.354 -0.021 -0.123 2.037 2.310 2.163

BI709111 -1.716 -1.717 -1.458 -0.019 -0.030 0.393 -0.058 -0.031 2.072 2.439 1.999

BI891155 -0.811 -0.113 -0.333 0.136 0.047 0.191 0.844 0.969 2.221 2.446 1.853

AI397023 -0.259 -1.717 -1.458 0.122 -0.052 0.352 -0.151 -0.123 2.174 2.200 2.157

BM154337 0.011 0.102 -0.378 -0.302 -0.324 -0.300 1.265 0.380 2.235 2.243 2.058

AF246162 -0.252 -0.205 0.465 -0.163 -0.106 0.011 -0.058 -0.123 2.056 2.269 2.217

AW059316 -0.234 -0.204 -0.278 -0.377 -0.161 -0.374 -0.572 -0.295 2.291 2.036 2.225

AW171471 -0.111 -0.301 0.363 0.196 0.459 0.486 1.223 0.819 2.190 2.240 2.140

AF096509 0.298 -0.978 -1.023 0.459 0.279 0.625 -0.301 0.570 2.152 2.459 1.965

BG891955 0.070 -0.099 -0.301 0.874 0.792 0.803 0.340 0.981 2.056 2.271 2.258

BM024216 -0.499 -0.597 -0.854 0.262 0.601 0.111 0.154 -0.198 2.369 2.086 2.130

AA658759 -1.032 -1.368 -1.559 -0.289 -0.342 -0.214 -0.077 -0.155 2.387 2.258 1.948

BI888934 0.137 0.233 1.085 -0.052 -0.095 -0.169 1.180 0.147 2.191 2.318 2.088

AW117094 -3.012 -2.805 -2.785 -2.061 -2.155 -2.063 -2.281 -1.712 2.087 2.311 2.216

BG738252 -0.199 -0.050 0.157 -0.049 -0.187 -0.200 0.327 0.769 2.146 2.336 2.143

BI865007 -1.078 -1.698 0.669 -0.558 -0.543 -0.636 0.802 0.103 2.063 2.186 2.379

AI878005 0.033 -0.119 0.521 0.021 0.108 0.317 0.329 -0.123 2.135 2.357 2.138

AI958945 0.271 0.142 -0.337 -0.272 -0.267 -0.298 -0.020 -0.060 2.393 1.867 2.376

BM025842 -0.086 -1.717 -1.458 -0.076 0.182 0.010 0.083 -0.123 2.571 2.073 1.999

BM095174 0.599 -1.717 -1.458 0.007 0.212 0.385 0.445 -0.123 2.115 2.440 2.140

BI888432 0.127 -0.232 0.290 0.127 -0.082 -0.088 0.232 0.723 2.084 2.354 2.258

AI522502 0.222 0.081 0.739 0.139 -0.017 0.068 -0.399 0.126 2.199 2.321 2.181

BI882594 -1.443 -1.717 -0.873 -1.871 -0.998 -1.351 -0.528 -0.276 2.199 2.530 1.974

AI666947 0.307 0.383 0.554 1.177 1.026 1.354 1.556 1.293 2.258 2.276 2.183

AW171254 0.101 -0.217 -0.400 0.884 -0.127 0.961 1.284 0.829 2.189 2.335 2.207

AW422269 -0.889 0.296 -1.458 -0.124 0.269 -0.544 -0.021 -0.444 2.568 2.160 2.016

BI881679 0.056 0.299 -0.228 -0.090 0.085 -0.077 0.655 0.739 2.320 2.223 2.202

AW077184 -0.025 -0.015 0.112 -0.018 -0.164 -0.001 0.823 0.969 2.313 2.197 2.239

AF169639 -1.716 -1.717 -1.458 0.315 0.102 -0.108 -0.219 0.412 2.236 2.361 2.159

BI886251 -0.069 -0.080 0.254 0.527 0.454 0.447 0.484 0.437 2.159 2.293 2.312

AI793969 0.136 -1.717 1.025 -0.103 -0.003 -0.575 1.062 0.165 2.049 2.208 2.517

BI430413 -0.399 -1.717 -1.458 -0.550 0.045 -0.337 1.275 0.028 2.250 2.307 2.228

BG303052 -0.798 -0.481 -0.209 0.324 0.100 0.254 0.313 0.817 2.098 2.182 2.528

BI891674 0.148 -1.717 -1.458 0.060 -0.153 0.290 -0.245 0.120 2.106 2.476 2.236

BE017030 0.304 -1.717 -1.458 -0.422 -0.541 -0.198 0.157 -0.123 2.242 2.468 2.110

BI704244 -0.287 -0.035 -0.511 -0.248 -0.096 -0.343 0.432 0.413 2.348 2.254 2.220

AW059104 -0.402 0.063 -1.458 -0.122 -0.126 -0.111 -0.058 0.404 2.203 2.675 1.954

BI704438 -0.003 0.264 0.645 0.812 0.511 0.705 0.832 1.064 2.157 2.494 2.185

AI883443 -0.554 -0.456 -0.374 0.359 0.527 0.214 0.443 0.693 2.321 2.358 2.158

AI588395 -0.044 -0.301 -0.641 -0.208 -1.783 0.080 -0.058 -0.323 2.380 2.278 2.181

AW184048 0.568 0.541 0.404 0.512 0.429 0.772 0.905 0.997 2.532 2.139 2.187

BI891290 -0.483 -1.040 -1.307 0.040 0.159 0.286 0.543 0.460 2.183 2.334 2.341

BM026700 -0.325 -0.230 -0.970 -0.321 0.076 -0.374 -0.690 -0.532 2.222 2.277 2.360

BI865832 -1.000 -1.305 0.428 0.111 0.108 -0.014 0.378 0.595 2.212 2.345 2.319

AI657601 -0.577 -1.717 -1.602 -0.395 -0.182 -0.505 -1.781 -1.644 2.196 2.452 2.231

BI880133 0.258 0.367 0.779 -0.282 -0.088 -0.285 0.690 1.182 2.149 2.545 2.190

BI430340 -0.737 -0.507 -0.511 0.656 0.470 0.414 0.820 0.932 2.417 2.418 2.051

AI964264 -0.345 -1.674 -0.199 0.048 0.034 -0.037 0.581 0.129 2.244 2.387 2.264

AW826969 -0.070 -0.158 -0.476 -0.236 -0.227 -0.316 0.385 0.955 2.159 2.498 2.247

AI793807 0.484 -0.197 0.601 0.354 0.766 0.584 1.257 1.297 2.158 2.385 2.369

BI880051 0.771 0.498 0.980 -1.045 -0.696 -0.721 -0.058 -0.123 2.129 1.908 2.884

BM036754 0.220 0.277 0.092 -0.299 -0.322 -0.148 0.638 0.601 2.492 2.306 2.146

BI704334 -1.192 -3.385 -2.957 -1.272 -0.233 -2.842 -2.251 -1.417 2.199 2.104 2.642

L42547 -0.814 -0.335 -0.507 -0.111 -0.210 -0.372 -0.021 -0.123 2.108 2.680 2.169

BI886872 -0.519 -1.652 0.557 0.156 0.037 0.043 0.628 -0.123 2.281 2.309 2.384

BI889170 -0.389 -0.609 -0.451 0.774 0.584 0.610 1.079 1.138 2.396 2.361 2.226

AI793924 -1.716 -1.717 -1.458 -1.143 -0.998 -0.721 -0.058 -0.123 2.210 2.456 2.334

AF257519 -0.249 -0.107 -0.425 0.112 -0.255 0.035 -0.197 0.646 2.117 2.529 2.360

BI867327 -1.857 -2.055 -2.125 -1.072 -0.795 -1.064 -0.099 -0.084 2.399 2.234 2.374

BI885186 0.340 0.052 0.840 0.741 0.923 0.663 0.882 1.100 2.274 2.230 2.507

BI877622 -0.285 0.124 0.205 0.184 -0.057 0.149 0.802 1.366 2.222 2.499 2.290

BI885905 -0.235 -0.023 0.139 -0.155 -0.127 -0.261 0.049 -0.187 2.322 2.494 2.214

AI793849 -0.239 -0.105 0.014 0.096 -0.028 0.278 0.350 -0.123 2.081 2.576 2.379

AA495305 0.160 -1.717 0.675 -0.037 0.202 0.161 0.188 0.199 2.412 2.292 2.332

BI707482 -1.504 -1.252 -0.793 -0.409 -0.019 -0.171 -0.446 -0.568 2.408 2.238 2.391

AI601495 -0.154 0.212 -0.350 0.174 0.276 0.328 0.155 0.196 2.601 2.454 1.995

AW420284 0.237 0.308 0.767 -0.019 0.082 -0.163 0.638 0.816 2.221 2.418 2.412

X89722 -0.573 -1.391 -0.571 -0.029 0.189 0.404 0.764 0.060 2.193 2.670 2.189

AW420476 -0.358 -0.180 -0.492 -0.037 0.016 -0.016 0.700 1.285 2.125 2.671 2.258

BM026491 0.334 -1.717 -1.458 -0.236 -0.844 0.108 0.234 -0.123 2.215 2.811 2.031

AI496838 -0.662 -0.932 0.669 0.181 0.197 0.170 0.357 -0.123 2.206 2.618 2.240

BI841768 -0.071 -1.717 0.446 -0.816 -0.998 -0.721 -0.058 -0.123 2.215 2.595 2.267

BM172666 -1.530 -1.878 -2.252 -0.972 -1.042 -0.863 0.342 0.143 2.249 2.389 2.451

BI704253 -0.448 -2.644 -2.683 -1.301 -1.705 -1.671 -1.027 -2.338 2.263 2.234 2.592

BI841542 -0.586 -0.556 -0.025 0.334 0.479 0.692 0.404 0.883 2.188 2.491 2.412

AF359424 -1.925 0.335 -0.090 -0.742 -0.905 -0.325 0.992 0.497 2.182 2.554 2.358

AI957894 -1.716 -1.717 0.711 -0.406 -0.249 -0.400 1.361 -0.123 2.071 2.144 2.881

BG305301 0.407 0.367 0.848 0.199 -0.260 0.126 0.271 0.441 2.277 2.633 2.213

AI959620 0.586 -1.717 0.522 -0.428 -0.246 -0.022 0.483 -0.123 2.145 2.447 2.540

BI879531 -2.145 -1.016 -1.822 -1.023 -0.176 -0.451 0.005 0.443 2.013 2.506 2.619

BG306692 -1.213 -1.980 -1.580 -0.509 -1.028 -0.897 0.303 -0.058 2.472 2.129 2.539

AI723236 0.154 -1.717 1.091 0.056 0.347 -0.042 0.149 0.784 2.270 2.354 2.520

BM181246 -0.128 0.346 -0.186 0.332 0.378 0.225 0.497 0.460 2.231 2.584 2.340

BI706506 -0.630 -0.685 -0.889 0.186 0.038 0.013 0.447 -0.123 2.260 2.627 2.281

AA605677 -0.553 -0.172 0.295 -0.229 -0.159 -0.287 0.800 0.798 2.549 2.194 2.455

AI964258 0.353 0.230 0.171 0.587 0.389 0.636 1.130 1.073 2.518 2.517 2.194

AF175294 -0.036 -0.326 -0.017 -0.172 -0.468 -0.379 0.905 -0.123 2.334 2.505 2.402

BG891864 0.275 -0.418 -0.703 -0.040 0.048 -0.204 0.755 -0.854 2.197 2.725 2.323

BI881918 -0.525 -0.201 0.203 -0.172 0.071 -0.201 -0.307 -0.123 2.056 2.699 2.498

BI887651 -0.057 0.113 0.433 0.134 0.072 0.230 0.462 0.818 2.510 2.557 2.194

BI887758 -0.013 0.041 -0.180 -0.253 0.168 -0.140 0.038 0.023 2.608 2.170 2.500

BI886160 0.236 0.094 0.217 -0.114 -0.035 0.045 0.113 0.241 2.339 1.934 3.021

BE016173 -0.083 0.104 0.755 -0.187 -0.662 -0.471 1.425 0.504 2.266 2.714 2.336

BG985836 0.324 0.293 0.039 0.218 -0.134 0.402 1.025 0.918 2.319 2.406 2.595

BI704280 -0.007 -0.463 -0.448 0.053 0.203 0.313 0.572 0.870 2.500 2.425 2.395

BI865578 -0.616 -0.454 -1.051 -0.217 -0.083 -0.461 0.968 0.405 2.387 2.410 2.535

AI588475 0.265 -1.717 -1.458 0.028 -0.459 -0.658 0.842 -0.123 2.467 2.484 2.388

BI979581 0.352 -0.273 0.076 0.386 -0.026 0.403 0.685 0.476 2.330 2.556 2.462

AI883929 -0.475 -1.717 -0.218 -0.009 0.353 0.310 0.904 0.699 2.654 2.421 2.274

BM005106 -0.561 -0.996 -0.942 -0.318 -0.329 -0.434 0.575 0.039 2.367 2.385 2.597

AB006084 -0.925 -0.817 -0.882 -0.309 -1.013 -0.157 -0.170 -0.286 3.138 2.793 1.421

AW117146 -0.136 0.049 0.927 -0.119 -0.229 0.230 -0.058 -0.031 2.197 2.742 2.416

BI890823 0.369 0.128 -0.593 -0.214 0.422 -0.252 -0.497 -0.359 2.451 2.539 2.378

AW058967 0.263 -1.717 1.073 -0.174 -0.219 -0.193 -0.058 -0.198 2.148 2.770 2.468

AI544617 0.262 -0.690 0.460 0.217 0.165 -0.234 0.132 -0.123 2.424 2.178 2.790

BI888493 -0.034 -0.280 -0.054 -0.084 -0.239 -0.212 0.651 1.042 2.126 2.495 2.772

AI544649 -0.297 -0.322 -0.810 -0.322 -0.181 -0.078 -1.061 -0.898 2.395 2.378 2.636

BE605983 0.134 -1.717 -0.234 -0.080 -0.217 0.067 1.515 0.102 2.244 2.599 2.569

AI384140 0.323 -1.464 -0.873 -0.212 -0.616 -0.308 -0.058 0.065 2.476 2.222 2.729

BI428973 0.382 0.347 0.446 0.489 0.605 0.458 1.115 0.658 2.480 2.316 2.642

AW826633 0.017 -1.717 0.252 -0.159 -0.050 -0.126 0.856 -0.123 2.472 2.562 2.410

BM026550 -1.100 -1.425 -1.532 -0.036 -0.685 -0.233 0.085 0.380 2.413 2.596 2.439

AW826907 0.023 -1.717 -1.458 -0.331 -0.998 -0.605 0.496 -0.123 2.410 2.478 2.562

BM083952 0.235 -0.378 0.020 0.063 0.653 0.816 1.297 1.241 2.330 2.648 2.489

AW232855 -0.427 -1.717 -1.458 0.109 0.340 0.324 -0.058 -0.123 2.504 2.575 2.390

BE605493 0.123 -0.114 -0.875 -0.301 -0.541 -0.376 0.079 0.087 2.538 2.496 2.460

BI889190 0.576 -0.751 -0.595 0.201 -0.108 0.319 0.765 0.458 2.667 2.496 2.334

BI843214 -0.623 -0.740 -1.501 0.280 0.138 0.447 0.869 1.020 2.270 2.680 2.548

AW232003 -0.132 -0.173 -0.479 -0.108 -0.050 0.169 0.903 0.741 2.593 2.405 2.511

AW077761 -0.372 -0.582 -0.903 -0.223 -0.476 -0.582 -0.753 -0.281 2.439 2.363 2.710

BI885889 0.156 -0.343 0.588 -0.186 -0.027 -0.466 0.227 0.517 2.421 2.832 2.263

AI641698 -0.174 -0.012 -0.841 0.292 0.235 0.519 0.571 0.920 2.421 2.387 2.709

BI886464 0.647 0.402 -0.782 -0.197 0.120 -0.081 0.558 -0.123 2.522 2.480 2.521

AI723092 -0.240 -1.631 -1.378 0.386 0.142 0.436 0.533 0.784 2.534 2.534 2.459

AW019740 -0.466 -0.992 -0.812 -0.008 -0.112 -0.312 -0.982 0.688 2.372 2.665 2.491

BE202131 -0.543 -0.189 -1.076 0.410 0.799 0.514 -0.022 0.234 2.113 2.764 2.657

AW594957 0.079 -1.717 -1.458 0.382 0.093 -0.178 -1.075 0.066 2.183 2.994 2.363

BI980224 -0.367 -0.414 -0.026 0.962 0.899 0.800 1.167 1.309 2.349 2.464 2.738

AW154501 -0.129 -0.176 0.483 -0.467 -0.144 0.009 -1.080 -0.123 2.265 2.958 2.363

AI722973 -0.479 0.319 0.403 0.295 -0.344 0.446 1.799 -0.041 2.592 2.483 2.515

AF030281 -1.716 -1.717 -1.458 0.182 0.028 0.225 -0.914 -0.123 2.381 2.902 2.320

AF160683 -1.716 -1.717 -1.458 -0.630 -1.672 -0.707 -1.253 -0.993 2.437 2.753 2.422

BI883638 0.078 0.192 0.023 0.378 0.289 0.339 1.210 0.868 2.469 2.550 2.597

BG985846 -0.173 -0.117 -0.512 0.324 0.019 0.153 1.058 0.876 2.491 2.554 2.588

AI384221 -0.153 -0.575 -1.170 -0.153 0.064 0.105 -0.230 0.311 2.325 2.808 2.520

AF292032 -1.254 -0.939 -0.531 -0.028 -0.159 0.209 -0.535 -0.123 2.432 2.592 2.631

AI353786 -0.036 -0.281 -0.349 0.405 0.255 0.380 1.144 1.116 2.462 2.632 2.566

BM072263 -0.432 -0.151 -1.043 -0.236 0.025 -0.423 -1.094 -1.051 2.517 2.750 2.409

BI888165 -0.249 -0.439 -0.500 -0.177 -0.004 -0.075 -0.300 -0.425 2.565 2.632 2.481

BI427758 -0.152 -0.201 -1.026 0.054 0.185 -0.252 0.778 0.571 2.580 2.479 2.647

AI397380 -1.409 -1.644 -1.409 -0.646 -0.245 -0.703 -0.388 -0.134 2.619 2.563 2.569

BI886259 -0.051 -0.605 -0.988 0.407 0.217 0.347 0.135 0.961 2.409 2.690 2.672

BI888812 -1.256 -1.260 -1.105 -0.190 0.042 -0.020 0.478 0.830 2.595 2.781 2.403

AW117083 0.364 0.495 -0.185 0.057 -0.227 0.034 0.485 0.488 2.485 2.519 2.780

AI330339 0.273 -1.717 -1.458 0.522 -0.112 0.640 0.679 1.182 2.528 2.958 2.314

BI889526 -0.778 -0.917 0.567 -0.176 -0.178 -0.263 0.315 0.441 2.354 3.223 2.266

AW567130 -0.197 0.324 0.640 0.017 -0.020 0.238 0.308 0.100 2.493 2.753 2.597

AI793769 -0.077 -0.262 -0.541 -0.145 0.063 0.076 -0.599 -0.113 2.617 2.873 2.362

BI886549 -1.078 -1.331 -1.077 -0.087 0.182 0.199 0.022 0.183 2.455 2.637 2.775

AW279916 -0.222 -0.301 0.447 -0.340 -0.142 -0.575 0.188 0.018 2.866 2.591 2.412

BI887817 -0.057 -0.212 0.654 0.013 0.155 -0.098 -0.096 0.313 2.439 2.957 2.484

AI721606 -0.545 -0.803 -0.441 -0.047 -0.085 -0.072 0.649 -0.123 2.522 2.467 2.897

AI618715 0.234 -0.201 0.624 0.143 -0.125 0.192 -1.404 -0.123 2.398 2.595 2.906

AI793934 -0.438 -0.236 -0.211 -0.321 -0.121 -0.070 -0.183 -0.123 2.583 2.710 2.617

U31079 -1.167 -1.501 -1.458 0.133 -0.178 -0.223 -0.541 -0.179 2.391 2.973 2.563

BM185759 0.149 -0.525 -1.458 -0.068 0.014 0.012 0.116 0.544 2.286 2.990 2.654

AW077931 -0.126 -0.560 -0.492 1.171 0.675 0.786 1.095 1.342 2.749 2.820 2.361

AW154514 -0.098 -0.661 0.381 0.138 0.179 0.432 1.054 1.281 2.304 2.659 2.973

AW078266 -0.092 -1.717 0.613 -0.208 0.117 -0.319 0.188 -0.123 2.398 2.849 2.700

BG799070 0.171 -1.717 0.827 -0.627 -0.630 -0.339 0.712 0.597 2.436 3.054 2.489

X67648 -0.212 0.098 -0.433 -0.242 -0.346 -0.406 -0.293 -0.703 2.596 2.928 2.489

BI671399 -0.153 -0.530 -1.458 0.083 0.039 0.251 0.089 -0.123 2.693 2.551 2.779

BI888550 0.198 -0.247 -0.621 -0.044 0.181 -0.092 -0.084 -0.395 2.617 2.796 2.617

AF157110 -1.974 -2.050 -2.466 -0.861 -0.450 -1.034 -1.201 -1.937 2.584 2.857 2.596

BI672301 -0.833 -1.717 -1.499 -0.370 -0.551 -0.407 0.636 0.370 2.626 2.764 2.655

BI846265 -1.716 -1.717 -1.458 -0.016 0.266 0.694 0.301 0.524 2.641 3.028 2.377

AI793772 -0.097 -1.717 -1.458 -0.228 0.192 0.101 -0.058 0.845 2.458 3.026 2.580

BI880127 0.187 -1.717 -1.458 0.139 -0.048 -0.171 0.188 -0.123 2.712 2.704 2.648

AI477949 0.508 0.841 0.645 0.669 0.611 0.681 1.457 1.392 2.866 2.589 2.660

AB006323 0.099 -1.717 -0.753 -0.889 -0.649 -0.721 0.116 -0.123 2.602 3.050 2.465

AW058804 0.123 -1.717 0.200 0.048 -0.133 0.102 0.728 0.297 2.524 2.904 2.689

BI706217 -1.716 -0.121 -0.675 -0.277 -0.031 -0.221 -0.058 -0.061 2.893 2.561 2.678

BG985714 -0.065 -0.565 -0.190 -0.541 -0.275 -0.496 -0.482 -0.445 2.526 2.791 2.828

BI846489 0.354 -0.233 0.369 0.011 0.357 -0.186 0.174 0.484 2.762 2.842 2.549

AI588708 -0.321 -0.714 -0.895 0.335 0.215 0.212 0.618 0.638 2.712 2.591 2.856

AI545142 -0.217 0.580 0.118 -0.052 -0.225 0.342 1.418 -0.123 2.530 2.963 2.677

BI672347 -1.553 -1.163 -1.202 -0.679 -0.907 -0.738 -0.158 -0.398 2.887 2.711 2.592

BM026608 -1.152 -0.803 -0.580 0.103 0.001 -0.071 0.366 0.858 2.579 2.971 2.662

AI793745 0.253 -0.183 0.231 0.652 0.352 0.787 1.048 1.400 2.780 2.641 2.791

BI980800 0.111 0.355 0.023 -0.113 -0.054 -0.186 -0.414 -0.123 2.633 2.675 2.909

AI641272 0.171 -0.132 -0.781 -0.029 -0.224 0.105 -0.094 0.248 2.720 2.817 2.697

BM103369 0.237 -1.046 -1.458 -0.422 -0.120 -0.562 1.045 -0.123 2.737 2.655 2.853

AI721615 0.032 0.449 0.042 -0.086 0.024 0.031 0.553 0.967 2.923 2.962 2.363

AI584659 -0.743 -1.674 0.506 -0.518 -0.577 -0.526 0.326 0.741 2.520 3.126 2.606

AI641648 -0.498 -1.717 -1.458 -0.209 -0.123 -0.206 -0.159 -0.442 2.497 2.765 3.006

AW019720 0.104 0.325 -0.176 0.075 -0.033 0.119 0.750 -0.909 2.576 2.976 2.752

BI845510 0.159 -1.717 0.632 -0.075 -0.079 0.118 -0.058 -0.123 2.637 2.641 3.033

BI891596 -0.089 -0.146 -0.651 0.040 -0.212 -0.026 -0.505 -0.270 2.766 2.680 2.879

BI891860 0.192 0.340 -0.391 -0.032 0.422 0.142 0.960 1.106 2.708 2.999 2.638

BG305445 -0.035 -0.560 -0.992 -0.049 0.109 0.517 0.195 0.448 2.825 3.067 2.514

AW019646 0.217 -0.931 -1.458 -0.089 0.005 0.359 -0.214 -0.123 2.540 2.945 2.923

BI888041 0.222 -0.926 0.610 0.058 -0.042 -0.240 0.640 0.829 2.621 2.952 2.859

AI397375 -0.760 -0.911 -1.130 -0.443 -0.308 -0.353 1.035 0.577 2.869 2.611 3.010

BM026830 0.126 -1.717 -1.458 0.224 -0.612 -0.281 0.570 0.047 2.764 3.003 2.734

AI601488 0.348 0.258 -0.018 -0.061 0.022 0.011 -0.626 0.163 2.657 3.019 2.854

AI558275 -0.249 -0.972 -1.543 -0.642 -0.612 -1.205 -1.360 -0.385 2.718 2.738 3.077

AI545576 -0.033 0.038 -0.740 0.140 0.463 0.249 0.201 -0.137 2.856 2.650 3.028

BI888232 0.413 -0.305 -1.458 0.243 0.336 0.587 0.733 0.018 2.527 2.819 3.193

AW154197 0.490 0.434 0.273 1.228 1.223 1.117 1.507 1.635 2.864 2.983 2.712

AW059322 -1.341 -1.068 -0.978 -0.372 -0.169 -0.172 -0.150 0.172 2.663 3.307 2.610

AI584501 0.375 -1.082 0.887 -0.075 -0.116 -0.037 0.796 1.000 2.506 3.112 2.992

AI721745 -0.399 -1.717 -1.458 -0.194 0.198 0.238 -0.058 -0.123 2.489 3.113 3.013

AI641768 0.083 -0.945 -0.550 -0.309 -0.606 -0.831 -0.058 0.773 2.869 2.655 3.103

BI880307 -1.140 -0.786 -1.458 -0.138 -0.127 0.040 0.236 0.404 2.792 3.071 2.769

AI322210 -0.338 -0.645 -0.712 -0.029 -0.052 -0.094 -1.795 -0.105 2.344 3.141 3.148

AW420768 -2.107 -2.927 -2.625 -1.096 -0.436 -0.928 -0.028 0.015 2.796 2.941 2.909

AI641634 -0.027 -0.839 -1.458 0.045 -0.464 -0.005 0.739 0.934 2.607 3.135 2.919

BM095979 0.263 -1.717 -1.458 -0.093 0.324 0.086 0.645 0.732 2.688 2.844 3.142

BI890314 0.198 -1.717 -1.458 -0.113 -0.098 -0.246 -0.058 -0.123 2.205 2.829 3.641

AI384268 0.088 0.090 0.424 -0.018 0.098 0.056 0.712 0.508 3.133 2.861 2.684

U80951 -0.210 -0.706 -1.458 0.138 -0.025 0.185 -0.192 -0.312 2.822 2.962 2.930

AW078150 -0.200 -0.073 0.187 -0.108 -0.509 -0.550 0.451 -0.123 2.837 3.120 2.759

AI878099 -0.234 -0.502 -1.102 0.085 -0.084 0.028 -0.432 -0.348 3.092 2.831 2.804

U14592 -1.716 -0.939 -1.458 0.018 -0.121 0.222 -0.833 -0.123 2.969 2.991 2.771

AI588196 -0.027 -0.427 -1.458 0.154 0.064 0.533 -0.058 -0.123 2.039 3.406 3.321

BI846484 -1.716 -1.717 -1.458 -1.143 0.021 -0.721 0.371 -0.123 2.977 2.725 3.084

AF151535 -0.695 -0.803 1.183 -0.015 -0.110 -0.280 0.296 0.405 2.444 3.357 2.989

BE201769 0.060 -0.391 -1.458 -0.420 0.053 -0.627 -1.058 -0.144 2.423 3.085 3.284

BI476854 0.161 0.275 0.532 0.692 0.324 0.674 0.941 0.828 2.808 2.984 3.003

BM095962 -0.165 -0.565 -0.634 0.462 0.426 0.497 0.025 0.207 2.814 3.095 2.886

AW344044 -1.716 -1.717 -1.458 -0.159 -0.060 0.106 0.120 0.182 2.687 3.450 2.677

AF354754 1.039 -1.521 0.113 -0.006 -0.147 0.106 1.172 -0.123 2.720 2.994 3.115

U49417 0.677 0.390 0.682 1.389 1.332 1.115 1.516 1.388 2.870 3.058 2.915

BI891984 0.343 0.293 0.844 0.115 0.037 -0.013 1.193 0.858 2.609 3.462 2.773

BI845265 -3.102 -3.107 -2.781 -1.092 -1.477 -1.173 0.096 0.185 3.008 2.720 3.117

AI444494 -1.716 -1.717 -1.458 1.006 -0.998 0.578 -0.058 1.408 2.833 3.332 2.704

AW232365 0.080 -1.717 -1.458 0.150 0.433 0.535 0.234 -0.123 2.763 3.253 2.886

BI476752 0.258 -0.803 0.051 -0.406 -0.423 -0.276 0.279 0.839 2.920 3.227 2.786

BG727974 -1.716 0.051 -1.458 -0.211 0.085 -0.356 1.219 0.845 2.749 3.322 2.880

BE017652 0.612 0.465 0.371 0.543 0.087 0.047 1.209 0.656 2.673 2.746 3.537

AI478015 -0.630 -1.206 -1.067 -0.197 -0.081 -0.230 0.847 0.839 3.076 3.011 2.924

BE693134 0.211 -0.079 0.674 0.072 -0.110 -0.069 0.539 -0.132 3.102 2.779 3.136

AW567292 -0.274 -1.717 0.290 -0.179 -0.940 -0.216 0.329 -0.123 2.962 3.238 2.822

AF075384 -0.271 -0.014 -0.273 -0.511 -0.209 -0.021 0.847 1.325 2.846 3.218 2.964

BM103277 0.514 -0.252 0.519 -0.391 -0.623 -0.452 -0.040 -0.123 2.807 3.349 2.883

BM103943 -0.085 0.115 -0.647 0.397 0.053 0.561 -0.147 0.678 3.037 3.070 2.936

BI887789 0.338 0.334 0.156 0.558 0.218 0.684 0.855 0.921 2.999 2.860 3.207

AI544688 0.486 -1.717 0.811 -0.910 -0.176 -0.683 1.128 0.965 3.055 3.343 2.680

AL592495 -0.391 -0.718 -0.891 -0.598 -0.498 -0.573 1.056 0.941 2.876 2.930 3.298

AI793637 0.305 0.699 0.252 -0.069 0.102 -0.079 0.367 0.059 2.946 3.142 3.065

BI887540 -0.217 -0.123 -0.287 -0.068 -0.098 -0.045 1.160 0.973 2.753 3.286 3.138

BI672128 0.288 0.211 0.105 -0.096 -0.058 -0.022 0.908 0.809 2.706 3.440 3.048

Y08321 -1.716 -1.717 -1.458 -0.211 -0.780 0.354 -0.675 -0.123 3.130 3.380 2.688

AA658743 -0.335 -1.717 0.760 -0.295 -0.036 -0.154 0.527 -0.123 2.893 3.222 3.085

BM083966 0.121 0.207 -0.347 0.580 0.921 0.637 1.206 1.144 3.128 3.157 2.942

AW231990 -1.716 -0.786 0.282 0.224 0.052 0.457 1.159 0.524 2.545 3.592 3.094

AY029808 0.197 -0.590 -0.963 -0.198 -0.541 -0.309 0.242 -0.081 3.043 2.847 3.342

BG799399 0.417 0.354 -0.157 0.030 -0.040 -0.204 -0.368 -0.502 3.288 3.030 2.937

AI957669 -1.675 -1.717 -1.458 0.347 0.104 0.706 -0.416 0.136 2.973 3.560 2.772

AI959766 -0.031 0.203 0.012 -0.079 -0.610 0.049 1.112 -0.123 2.883 3.233 3.200

BI867416 -1.716 -1.717 -1.458 -0.124 -0.998 0.086 -0.058 -0.123 3.089 3.223 3.005

AI477490 -1.716 -1.717 -2.662 0.174 0.352 -0.077 -0.058 0.093 3.041 3.312 2.974

BI890848 -0.331 -0.466 0.442 0.632 0.869 0.771 1.620 1.536 2.919 3.541 2.884

BI844240 -0.153 -0.487 -0.384 0.656 0.390 0.504 1.731 0.914 3.102 2.859 3.398

AI545424 -0.062 -1.717 -1.458 -0.147 -0.073 0.587 0.728 0.529 2.823 3.438 3.122

AI588190 0.963 0.562 0.854 -0.027 -0.352 0.304 0.685 0.190 2.165 2.467 4.759

AW826859 -0.596 -1.590 -1.458 -0.509 -0.391 -0.531 -0.058 -0.123 3.074 3.315 3.011

BI891245 0.036 -0.016 -0.065 -0.057 -0.315 -0.464 1.396 1.071 2.918 2.971 3.513

AW018998 0.343 -1.717 -1.458 0.612 0.380 0.898 0.876 1.374 3.180 3.325 2.903

AW077429 0.454 -1.717 -0.395 1.257 -0.569 1.030 0.100 1.084 3.004 3.578 2.830

AI558351 -0.623 -1.053 -0.067 0.467 0.069 0.387 -0.120 0.399 2.751 3.714 2.965

AI477041 -0.117 -0.505 -0.456 0.473 0.249 0.681 1.129 1.126 2.912 3.205 3.321

BG884107 0.139 0.285 1.241 0.044 0.068 -0.023 -0.058 0.628 2.933 3.333 3.212

AW059137 -0.137 -0.212 0.027 0.708 0.234 0.943 1.346 1.340 3.121 3.245 3.118

BM026015 0.428 0.540 1.117 -0.146 -0.151 -0.302 1.340 -0.123 2.461 3.010 4.046

AF359430 -0.851 -1.032 -0.585 -0.263 -0.452 -0.591 1.416 0.790 3.225 3.216 3.085

BI890305 0.289 -1.717 -1.458 0.004 -0.470 -0.019 -0.058 -0.123 3.073 3.325 3.146

BI879038 0.071 -0.289 -0.160 0.555 0.788 0.856 1.509 1.579 3.143 3.219 3.199

BI891338 0.514 0.126 0.411 -0.113 -0.231 -0.250 0.805 1.011 3.072 2.923 3.577

BI670950 -0.874 -1.155 -1.458 1.635 -0.171 0.951 0.706 0.969 2.886 3.578 3.140

BI705182 -1.716 -1.717 -1.458 -0.279 -0.817 -0.286 -0.058 -0.123 3.138 3.579 2.944

AF301264 -1.388 -1.717 -1.458 0.541 0.435 0.809 -0.349 -0.131 3.250 3.628 2.832

AF388363 0.132 -0.530 0.658 -0.096 -0.124 -0.040 0.242 0.120 3.159 3.323 3.251

BI879005 -1.716 -1.717 -1.458 -0.517 -0.965 -0.721 0.928 0.009 3.091 3.362 3.291

BI886272 0.135 0.320 0.670 1.242 1.482 1.546 1.824 1.891 3.340 3.316 3.117

AI657922 0.261 -0.053 -1.458 0.116 0.203 0.281 -0.283 -0.123 3.415 3.073 3.287

AI496943 -0.184 -1.717 0.891 0.068 0.111 0.003 1.505 1.359 2.979 3.516 3.281

BI843080 -1.716 -1.717 0.094 0.235 -0.012 0.097 -0.590 0.241 2.661 3.191 3.934

AF197880 -0.653 -0.158 -0.294 -0.480 -0.558 -0.723 0.517 0.680 2.974 2.984 3.830

BE201470 -0.511 -0.293 -1.664 -0.760 -1.569 -1.117 -0.120 0.986 4.148 4.029 1.651

BG883207 -0.022 -0.110 -1.137 -0.022 0.006 -0.099 0.563 1.356 3.379 3.170 3.306

BG729043 -0.662 -1.717 -1.367 0.586 0.357 0.573 1.027 -0.123 3.201 3.712 2.952

BI881145 -0.215 -0.972 0.012 0.091 -0.301 -0.087 -0.058 -0.123 3.157 3.416 3.293

BI839233 -1.716 -1.717 -1.458 -0.051 -0.824 0.199 -0.058 0.538 3.213 3.468 3.190

AI958983 -1.716 -1.717 -1.458 -0.283 -0.159 -0.119 -0.058 -0.123 3.305 3.452 3.135

BM182563 -4.011 -4.514 -4.124 -3.048 -2.091 -2.839 -1.567 -1.785 3.208 2.941 3.758

BI887764 0.346 -1.717 -1.458 -0.290 0.274 0.138 0.980 1.273 2.888 3.320 3.707

BI887620 0.370 0.237 0.853 1.656 0.714 2.243 2.104 1.900 3.208 3.561 3.187

AA658586 0.837 -1.717 -1.458 -0.596 -0.492 -0.800 0.900 0.056 3.600 4.264 2.125

AI626478 -0.063 0.000 -0.027 0.044 -0.017 0.336 0.987 0.156 3.177 3.624 3.200

AW165108 0.145 0.029 -0.474 0.124 -0.010 0.001 0.439 0.669 3.366 3.278 3.375

BI890216 -0.267 -1.305 -1.458 0.002 -0.280 0.077 0.149 -0.042 3.329 3.740 2.966

AW019162 -1.716 -1.717 -1.458 0.478 -0.413 0.820 -0.057 0.483 2.986 3.703 3.364

AI584322 -0.055 -1.717 1.344 -0.113 -0.401 -0.568 0.322 -0.123 3.066 3.380 3.622

BG307572 0.301 -1.717 -0.122 -0.262 -0.236 -0.376 0.628 -0.123 3.401 3.533 3.137

AB055662 -0.244 -0.309 -0.767 -0.050 0.064 0.162 0.058 -0.054 3.114 3.526 3.444

AI641660 -1.716 -1.717 -0.634 1.086 0.202 1.219 0.558 0.708 3.307 3.609 3.230

AI883326 -1.294 -1.717 -1.458 -0.086 -0.093 0.356 1.054 0.944 3.406 3.429 3.352

AW175575 0.420 -1.347 -0.525 -0.011 0.232 0.069 0.617 -0.061 3.507 3.313 3.386

AI884185 -0.079 -1.717 0.383 -0.087 -0.062 -0.441 0.871 0.964 2.796 3.944 3.485

BG727557 -0.022 0.018 -0.223 0.152 0.127 0.085 -0.093 0.233 3.414 3.409 3.415

BI888241 0.308 -0.303 1.001 -0.408 -0.534 -0.342 0.032 0.376 3.158 3.584 3.511

BE606011 -0.434 -0.474 -1.311 0.048 0.161 0.040 0.296 0.685 3.351 3.512 3.401

AW421072 -1.000 -1.717 -1.458 -0.099 -0.204 -0.867 -0.058 -0.123 3.601 3.757 2.950

AI477305 -0.732 -0.658 -0.290 0.676 0.654 0.781 1.379 1.251 3.111 3.628 3.575

BI890294 -0.219 -0.625 -0.727 -0.187 -0.107 0.117 0.380 0.457 3.516 3.400 3.401

BI980805 0.617 -0.188 -0.670 0.149 0.001 0.445 0.689 0.940 2.952 3.570 3.800

AI477419 -0.269 0.428 0.684 0.785 0.331 0.999 1.452 1.304 3.249 3.666 3.407

BI879454 0.096 0.282 -0.560 -0.551 -0.321 -0.510 -0.528 -0.266 3.397 3.616 3.314

AI942987 -0.195 -1.339 -0.980 0.439 0.033 0.469 0.915 1.445 2.825 4.021 3.518

AI666975 0.222 0.247 -0.420 -0.388 -0.252 -0.064 0.351 -0.291 3.259 3.670 3.446

U55177 -0.682 -0.913 -0.614 -0.231 -0.173 0.265 0.077 -0.123 3.238 3.922 3.258

AI959496 0.763 -1.717 -1.458 0.478 0.312 0.211 0.091 -0.123 3.101 3.586 3.735

BI887434 -1.716 -1.717 -1.458 -0.016 -0.998 -0.721 -0.058 -0.123 3.229 3.703 3.499

AJ309314 -0.130 -0.540 -0.041 -0.456 -0.497 -0.454 -0.597 -0.785 3.566 3.498 3.377

AF039412 0.257 0.150 0.710 0.057 -0.604 0.604 1.160 1.922 3.182 3.735 3.526

AW058839 -0.456 -1.054 -0.166 0.616 0.191 0.576 0.499 1.201 3.391 3.383 3.690

BE556846 -0.149 -0.305 -0.228 0.873 0.743 1.084 1.775 1.679 3.395 3.572 3.530

BI885240 -0.771 -0.985 -1.458 -0.070 -0.292 -0.160 -0.072 -0.001 3.082 3.628 3.795

BI892167 -0.030 0.648 0.972 -0.158 -0.004 -0.151 0.210 0.645 3.237 3.577 3.695

BI891492 -0.070 -0.175 -0.897 -0.227 -0.107 -0.380 1.514 0.723 3.546 3.410 3.568

AI965251 0.010 -1.717 -1.281 0.016 0.012 -0.335 -0.560 -0.340 3.237 3.799 3.516

AI878677 0.481 -1.717 -1.458 -0.399 -0.176 -0.097 0.271 0.084 3.333 3.718 3.515

Y13653 0.240 -0.022 0.342 0.224 0.060 0.229 0.066 0.584 2.936 3.991 3.772

AI641460 -1.716 -1.717 -1.458 0.347 -0.893 -0.721 0.701 0.359 3.317 3.798 3.643

U57975 -0.167 -1.717 -1.458 0.057 -0.131 -0.157 -0.237 0.357 3.386 3.805 3.567

AW059193 -0.461 0.296 -0.493 0.053 0.189 0.463 0.795 0.974 3.522 3.768 3.526

AI522514 -0.501 -0.920 -0.946 0.862 0.243 1.341 1.407 1.905 3.583 3.565 3.676

BI890917 0.404 0.245 0.436 0.003 -0.153 0.293 1.371 2.023 3.313 3.678 3.852

BI888545 0.321 -1.717 0.276 -0.549 -0.215 0.144 1.783 -0.123 3.540 3.998 3.311

AI385123 -1.716 -1.717 0.261 0.635 0.036 0.682 -0.683 0.888 3.305 3.850 3.746

AJ242515 -0.181 -1.717 -1.458 -0.311 -0.045 -0.077 -0.058 0.524 3.395 3.776 3.744

BI892151 0.393 0.664 -0.264 0.261 -0.121 0.156 1.214 1.020 3.691 3.472 3.760

BI887911 -0.211 -0.403 0.040 0.563 0.657 0.640 0.981 1.181 3.423 3.696 3.807

BI887709 0.200 0.836 -0.026 0.405 0.094 0.130 1.022 1.005 3.500 3.801 3.643

AI601390 0.161 -0.690 0.716 -0.063 -0.505 0.220 -0.058 -0.123 3.631 3.870 3.460

BM185169 0.472 0.440 -0.152 0.032 0.445 0.060 0.740 0.606 3.609 3.451 3.904

BI883483 0.102 0.026 -0.014 0.032 0.208 0.384 -0.255 0.406 3.716 3.866 3.397

BI882203 -1.716 -1.717 -1.458 0.155 -0.129 0.021 -0.158 0.767 2.771 4.065 4.196

AI641480 0.635 -0.821 0.947 0.268 -0.069 0.409 -0.058 0.199 3.620 3.758 3.736

AI545309 -0.785 -1.717 -1.458 -0.087 -0.272 -0.272 0.431 -0.123 3.281 3.902 3.934

BI846314 -0.105 -1.717 -1.458 0.152 0.015 -0.721 -0.058 -0.123 3.314 4.043 3.777

BI888956 0.053 0.315 -0.601 -0.226 -0.525 -0.551 1.411 1.072 3.313 3.815 4.018

BI890034 -0.670 -0.358 -1.041 -0.411 -0.593 -0.537 0.521 0.971 3.857 3.426 3.917

BI891709 0.098 0.371 -0.365 0.152 0.288 0.184 0.784 1.064 3.750 3.794 3.662

AW826550 -1.716 -1.717 -1.458 0.071 -0.139 0.874 -0.021 -0.123 3.362 4.166 3.704

BI888210 -0.586 -1.149 -1.616 -0.696 0.100 -0.285 0.343 -0.207 4.115 3.866 3.443

AI641401 0.176 -0.028 -1.458 -0.644 -0.885 -0.123 0.180 -0.123 3.832 4.210 3.417

AW058828 0.518 -1.717 -0.724 -0.006 -0.155 -0.436 1.493 0.517 3.883 3.583 3.998

BG728568 -0.736 -1.717 -1.458 -0.085 0.750 0.076 -0.058 0.708 3.622 3.674 4.170

BE200552 -1.716 -1.717 -1.458 -0.627 -0.703 -0.598 0.832 -0.123 3.782 3.833 3.883

BI326746 0.021 -1.717 0.542 -0.021 -0.178 0.336 -0.058 -0.123 3.984 3.690 3.827

AW420304 -1.104 -1.089 -0.760 0.670 -0.229 1.064 -0.058 0.112 3.651 3.964 3.979

AW128428 -0.857 -1.239 -1.963 -0.842 -0.500 -0.857 0.304 -0.225 3.873 4.079 3.659

BI865459 -0.190 -0.302 -1.097 0.019 0.009 -0.050 0.706 0.516 3.863 4.059 3.711

AW421191 -2.012 -2.300 -0.946 -1.872 -1.573 -1.881 -1.541 -0.868 3.619 4.151 3.885

BM102179 -0.082 -1.717 -1.458 0.162 -0.318 -0.335 -0.058 0.784 3.529 4.283 3.863

AI657858 0.409 -0.145 0.056 -0.052 -0.025 0.142 0.307 0.750 3.931 3.737 4.028

AW466697 -1.284 -1.322 -1.334 0.105 -0.269 0.566 1.168 1.802 3.504 4.278 3.943

BI892068 -0.392 -1.717 -0.897 0.039 0.094 -0.001 -0.297 0.003 3.949 3.867 3.919

BI890446 -1.045 -1.095 -0.911 -0.110 -0.194 -0.233 1.348 1.547 3.861 3.921 4.064

AI722328 -0.259 -0.271 0.705 1.053 0.859 1.063 1.710 1.614 4.217 3.811 3.856

BI704359 -0.244 -0.459 -0.255 -0.141 0.018 0.000 1.230 -0.123 3.898 3.624 4.377

AF197909 -0.735 -1.632 -1.293 -1.070 -0.635 -0.881 -1.221 -0.796 4.158 3.758 3.988

AF042191 0.310 -1.717 0.187 -0.512 -0.354 -0.217 0.679 -0.241 3.917 4.062 3.949

BI889241 0.021 -1.717 -1.458 -0.150 -0.340 0.034 0.515 0.352 4.016 3.919 4.026

BI891948 0.055 -0.187 -1.458 -0.035 -0.149 -0.064 0.028 2.225 3.527 4.234 4.251

AF426384 0.150 0.393 0.776 -0.517 -1.140 -0.721 0.398 -0.123 3.870 4.381 3.781

BF717548 0.301 0.791 -0.339 -0.043 -0.295 0.076 2.083 0.120 3.964 4.014 4.090

BI892272 -0.041 -0.251 -0.421 0.447 0.135 0.187 1.507 1.291 3.637 4.250 4.189

BI890279 -0.924 -2.189 -0.874 -0.834 -0.210 -1.152 0.395 0.345 3.765 4.145 4.193

AF201379 -0.284 -1.717 -0.696 -0.216 -1.236 0.210 0.690 2.229 3.919 4.329 3.994

BI883233 -0.474 -1.717 -1.458 -0.182 -0.752 -0.300 -0.058 -0.123 4.129 4.164 4.024

AI641092 -0.084 -1.717 0.340 0.153 0.712 0.325 -0.167 0.253 3.792 4.450 4.090

BI892444 0.163 -0.642 0.348 0.606 0.167 1.005 0.862 1.467 4.074 4.292 4.113

BI892200 -0.126 -1.717 0.886 0.277 0.087 -0.014 1.772 1.760 3.778 4.386 4.354

AW078445 -0.331 -0.821 0.878 -0.182 -0.153 -0.389 0.149 -0.123 4.154 4.209 4.247

BI890262 -0.297 -1.717 1.915 -0.767 -0.998 -0.721 0.040 -0.123 4.068 4.543 4.002

BI704249 0.149 -0.550 -1.458 -0.319 -0.015 -0.089 1.461 1.666 4.002 4.203 4.417

AB071895 -0.050 0.067 -0.366 -0.232 -0.267 -0.307 0.637 1.136 4.249 4.100 4.331

BI886470 -0.960 -1.297 -0.525 0.665 0.155 0.424 0.283 0.281 4.006 4.622 4.162

BI890045 -1.281 -1.787 -1.044 -0.774 0.036 -0.531 0.913 0.644 4.327 4.303 4.208

BI704281 -0.536 -3.100 -1.803 -1.848 -1.798 -2.124 0.296 0.126 4.263 4.397 4.298

AW117076 0.590 -1.717 -1.458 0.057 -0.295 -0.329 1.242 0.773 3.850 4.803 4.310

AI957698 0.264 -0.197 -1.458 -0.129 0.501 0.037 0.851 0.219 3.757 4.645 4.587

BI883252 0.118 -1.717 -1.458 0.019 0.191 0.590 -0.058 -0.123 4.441 4.413 4.182

AW344023 -0.208 -1.717 -1.458 -1.143 -0.998 -0.721 -0.058 -0.123 4.335 4.429 4.298

AI461367 -1.716 -1.717 -1.458 0.111 -0.150 0.059 -0.058 1.023 4.119 4.484 4.501

BG985503 0.080 -1.717 0.556 -0.165 0.023 -0.180 1.533 2.229 4.139 4.498 4.484

BI889922 -1.716 -1.717 -1.458 0.163 -0.102 -0.050 -0.136 0.067 4.376 4.633 4.189

X66958 0.068 -1.717 -1.458 -0.010 -0.597 -0.386 0.364 -0.123 4.244 4.066 4.935

AF222996 0.570 -1.717 -1.458 0.119 0.151 0.410 0.755 0.866 4.476 4.598 4.260

BM185251 -0.579 -1.606 -0.914 0.103 0.649 -0.368 1.148 0.313 4.321 4.765 4.256

AI641409 -0.555 -1.717 -1.458 0.112 0.317 0.041 0.152 0.941 4.099 4.833 4.480

AW059366 -0.568 -1.012 -0.190 0.152 0.173 0.187 0.705 0.330 4.353 4.394 4.747

AF191578 -0.261 0.101 0.515 -0.367 -0.265 -0.569 0.279 -0.123 4.589 4.805 4.271

AJ245491 0.215 -1.717 0.808 -0.421 -0.998 -0.328 -0.058 -0.123 4.332 4.465 4.887

BI671260 -1.716 -1.717 -1.239 -0.663 0.477 0.118 0.068 0.199 4.389 4.845 4.535

BG985518 0.164 0.085 -1.104 -0.280 -0.128 -0.068 1.497 1.107 4.609 4.562 4.629

BI318080 0.019 -1.717 -1.458 -0.254 0.231 -0.114 0.545 0.497 4.346 4.862 4.677

BM035358 -1.716 -1.717 -1.458 -0.209 0.000 -0.058 0.515 0.190 4.795 4.612 4.853

AW058763 0.357 -1.717 0.407 0.271 0.135 0.405 0.527 -0.041 4.430 4.898 5.033

AI477963 -1.466 -1.289 -0.988 -0.826 -0.809 -0.650 0.425 -0.123 4.511 5.030 4.886

BI886811 -0.371 -1.717 0.426 -0.045 -0.153 -0.290 -0.058 -0.123 4.834 5.107 4.609

BI891773 -0.669 -1.224 -0.988 -0.112 -0.098 0.148 -2.208 -0.404 4.406 5.526 4.643

BI891278 -1.716 -1.717 -1.458 0.103 0.389 0.163 0.271 0.419 4.602 5.250 4.724

BI850028 -1.716 -1.717 0.142 -0.501 0.088 0.079 -0.306 -0.123 4.949 5.057 4.732

AF134850 -0.875 -1.082 -1.020 -1.100 -1.039 -1.669 1.215 1.180 4.693 4.928 5.211

BM036509 0.719 -0.199 0.748 0.070 -0.058 0.232 1.882 0.812 4.701 5.114 5.114

BI890287 -0.030 0.159 0.532 1.896 -0.541 1.823 0.992 1.447 5.170 4.926 4.968

AB045624 -0.474 -1.717 0.062 0.351 -0.098 -0.224 2.778 1.465 4.761 5.437 4.896

AI959644 0.686 0.741 0.886 -0.109 0.303 -0.329 -0.058 -0.123 4.454 5.256 5.419

BI892036 0.605 -1.717 1.118 -0.131 -0.332 -0.494 -0.058 -0.123 5.212 4.758 5.160

BM083940 0.016 -1.717 -1.458 -0.040 0.209 0.607 0.733 0.558 4.951 5.203 5.125

BI892128 -0.118 -1.717 -1.458 -0.138 -0.120 -0.290 0.271 -0.031 5.007 5.082 5.381

BI704310 -0.005 0.278 0.074 -0.491 -0.382 -0.221 1.154 3.020 4.868 5.767 4.958

AI974191 -0.143 -1.717 -1.458 -0.596 -0.128 0.035 0.817 0.224 5.429 5.931 5.820

BI868116 0.292 -1.717 -1.458 -0.092 -0.110 -0.224 0.545 -0.123 6.498 6.685 6.630

AB055677 -1.140 -1.717 -1.458 -0.279 -0.201 -0.013 0.960 2.294 7.446 7.437 6.270

BI887368 0.140 -0.292 0.708 -0.396 -0.239 -0.529 2.863 3.667 6.840 7.389 7.747
