# Supplementary figures and images for: Transcriptome Analysis of Zebrafish Embryogenesis Using Microarrays
Source: PLoS Genet. 2005 Aug 26;1(2):e29. doi: 10.1371/journal.pgen.0010029 (PMC1193535; doi:10.1371/journal.pgen.0010029)

Figure S1

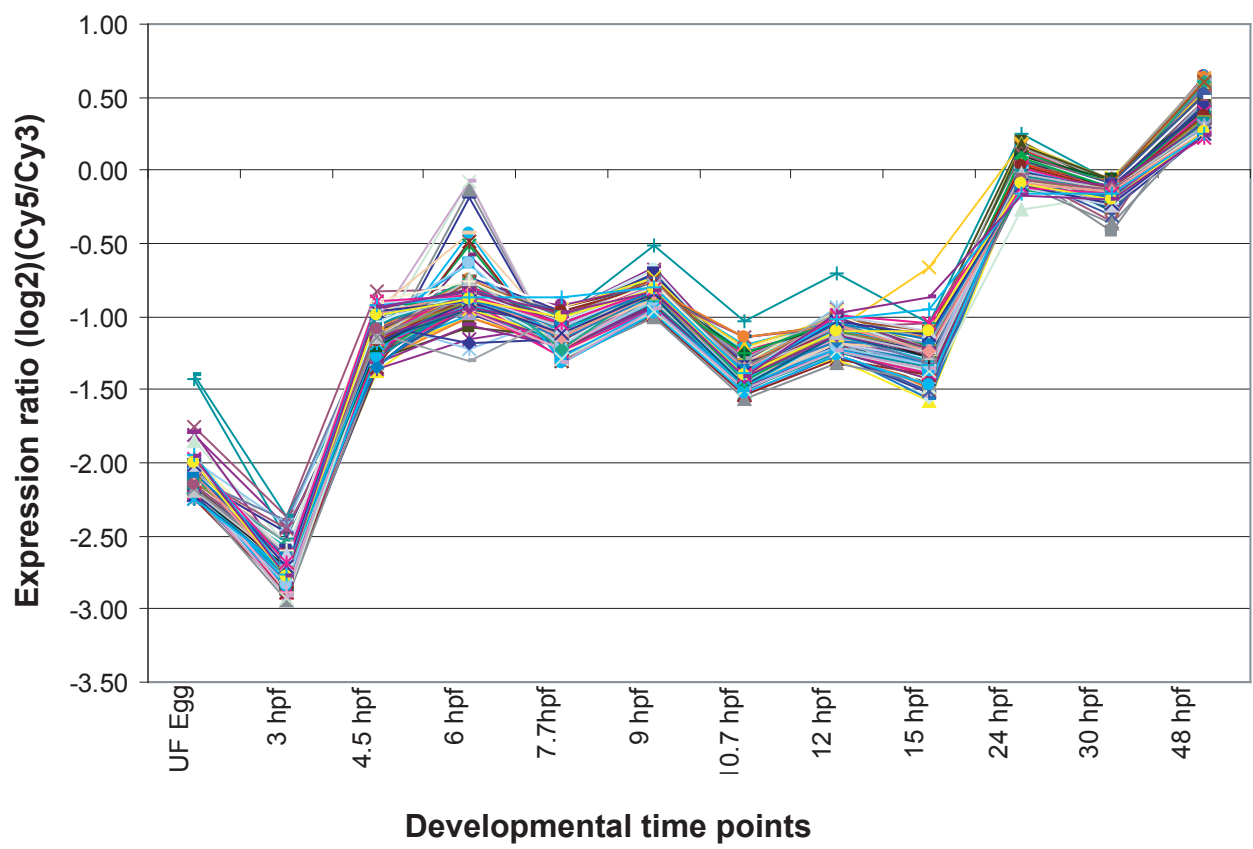

Supplement: Figure S1 — All the copies of the oligos in the array showed an almost identical expression pattern, indicating the reproducibility and homogeneity of the array data. (410 KB DOC) [file pgen.0010029.sg001.doc]

Figure S2

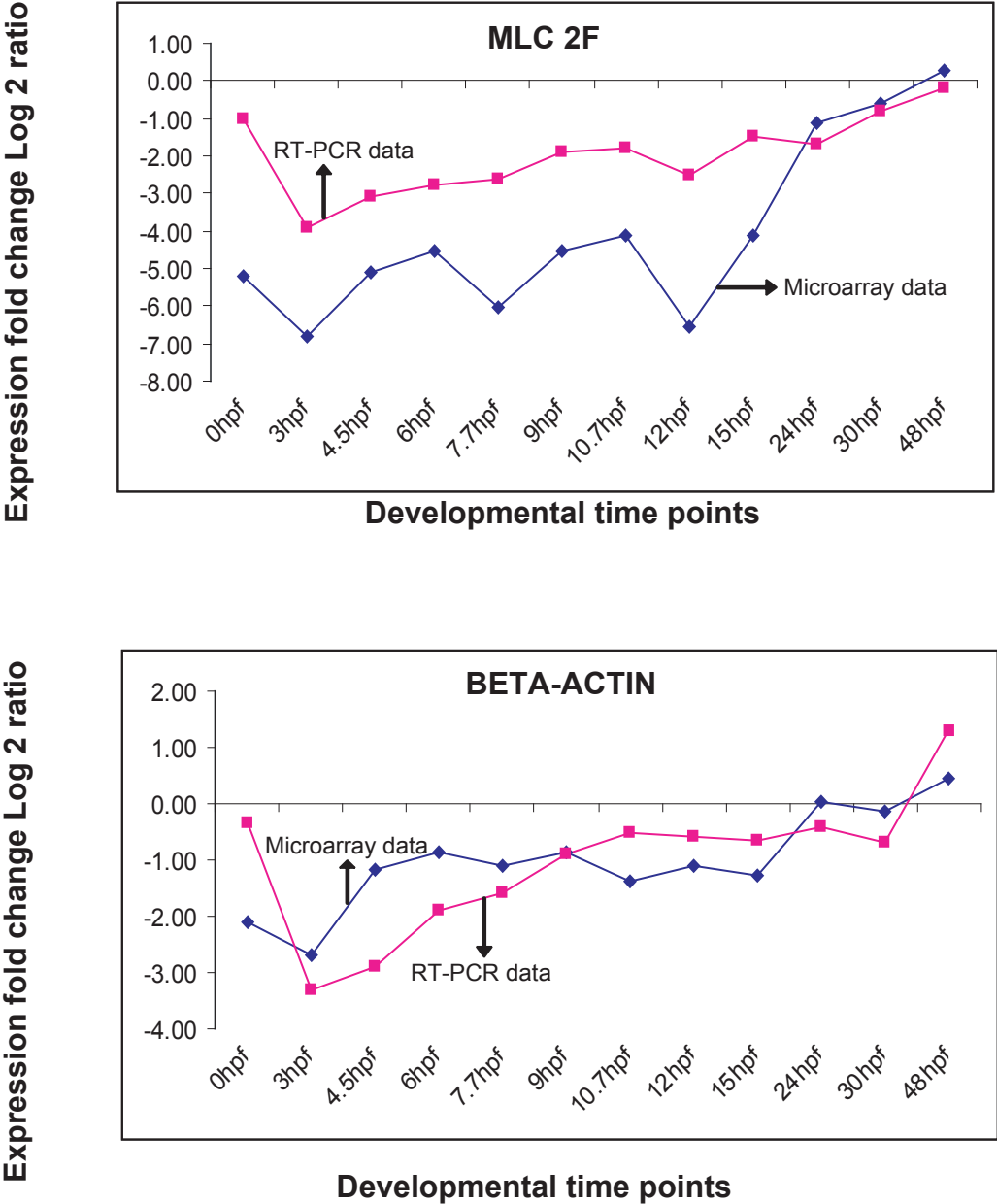

Supplement: Figure S2 — The patterns of expression detected in both methods are almost identical. (204 KB DOC) [file pgen.0010029.sg002.doc]

Figure S3

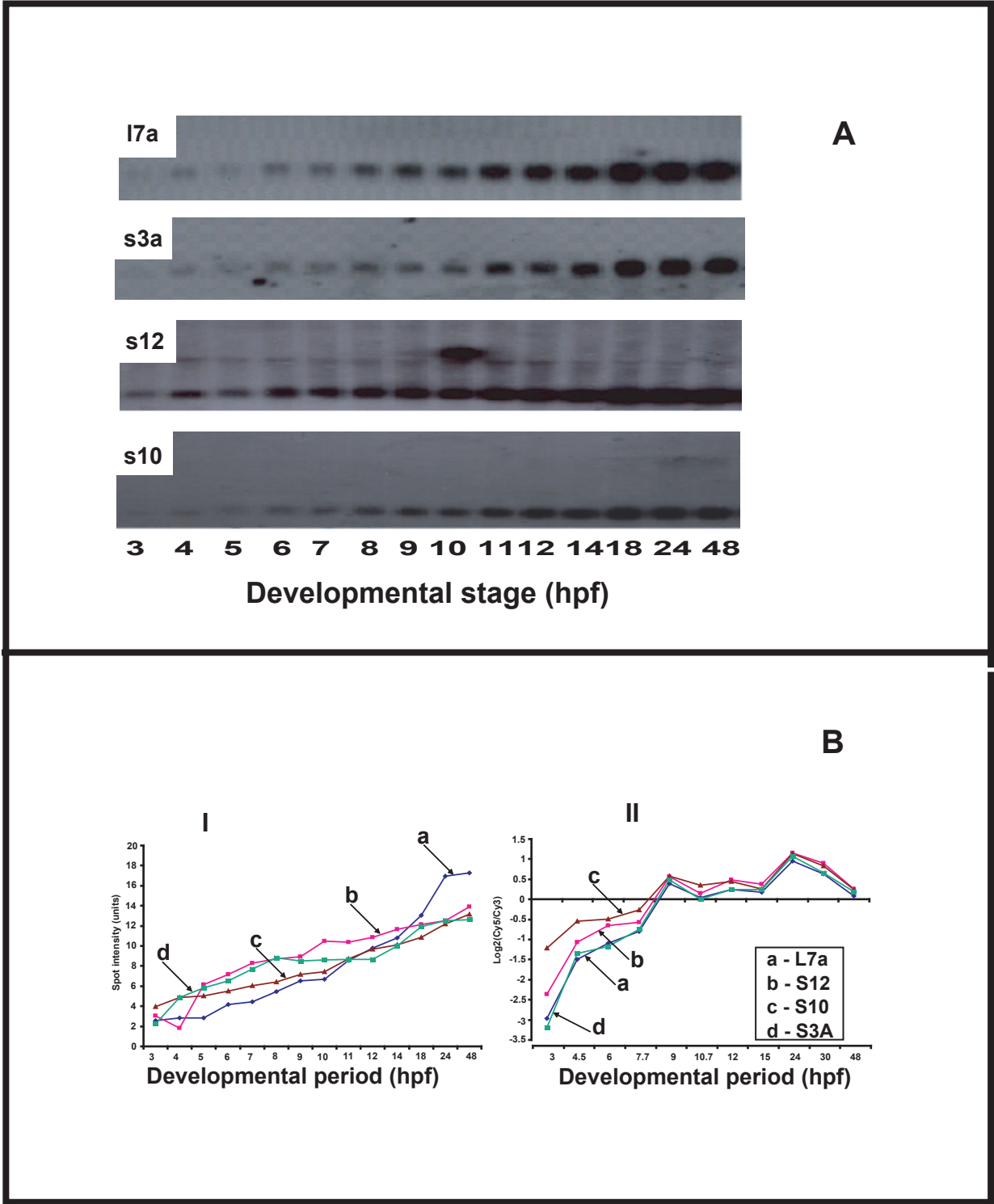

Supplement: Figure S3 — (A) Northern blot analysis to detect the transcript abundance for selected RP genes (l7a, s3a, s12, and s10) during embryogenesis. The expression pattern obtained by Northern blot analysis is similar to the data obtained from the array analysis. (B) (I). Intensity of hybridization in the Northern blots for the selected RP gene was digitized and plotted. (II). Expression data obtained from the arrays for the selected RP genes used in Northern blotting are presented. Both analyses show similarity in the pattern of transcript accumulation. (463 KB DOC) [file pgen.0010029.sg003.doc]

**Figure S4**

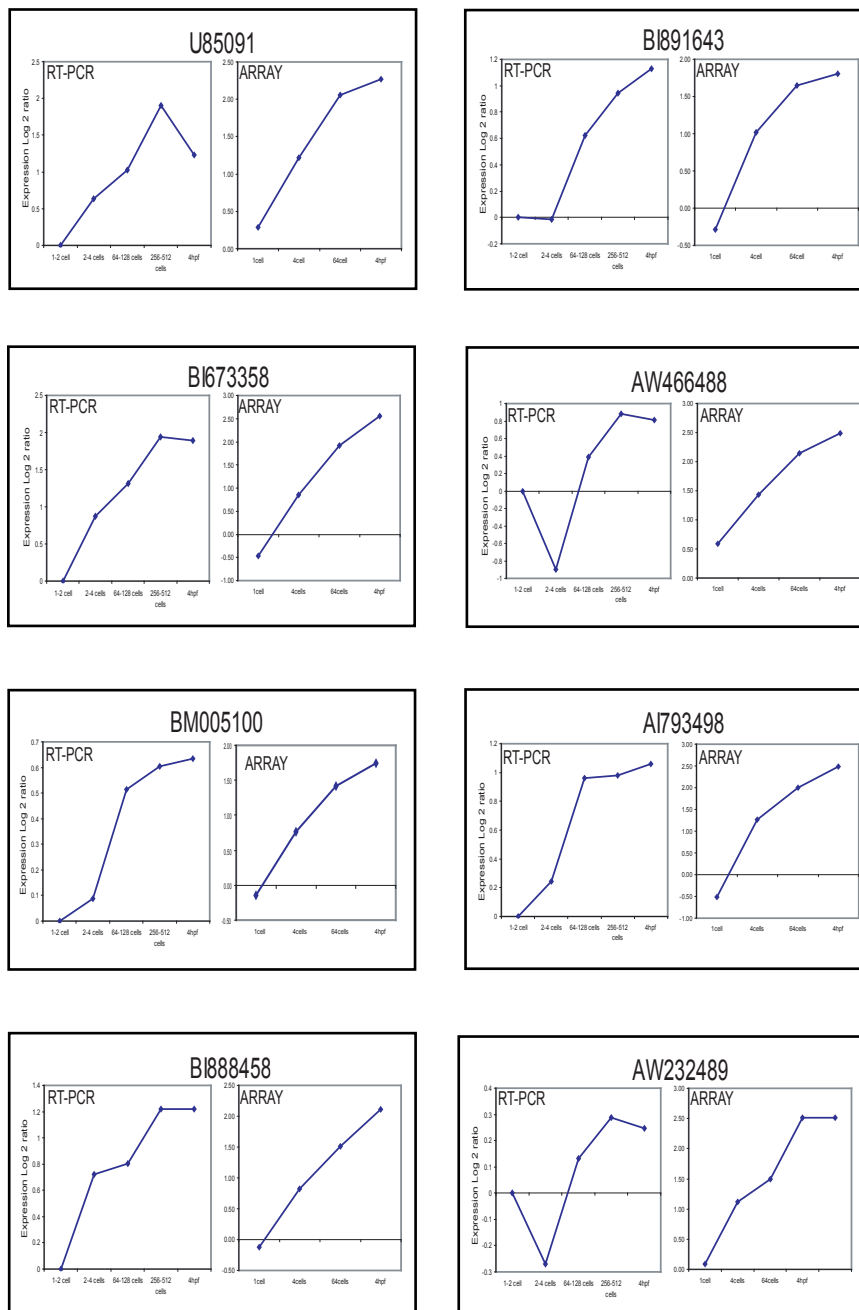

Supplement: Figure S4 — (227 KB PDF) [file pgen.0010029.sg004.pdf]
