# Supplementary material for: Transcriptome Analysis of Zebrafish Embryogenesis Using Microarrays
Source: PLoS Genet. 2005 Aug 26;1(2):e29. doi: 10.1371/journal.pgen.0010029 (PMC1193535; doi:10.1371/journal.pgen.0010029)
Supplement: Figure S5 — Real-time PCR was done using total RNA extracted from early stages of embryonic development (one-cell, two-/four-cell, 64-/128-cell, 256-/512-cell, and 4-hpf) for eight genes from Cluster 2 (pre-MBT transcribed genes). The expression pattern of these genes by real-time PCR and microarray are compared. The expression pattern is similar in both types of analysis. (182 KB PDF) [file pgen.0010029.sg005.pdf]

**Figure S5**

## Expression of early genes at other stages of development

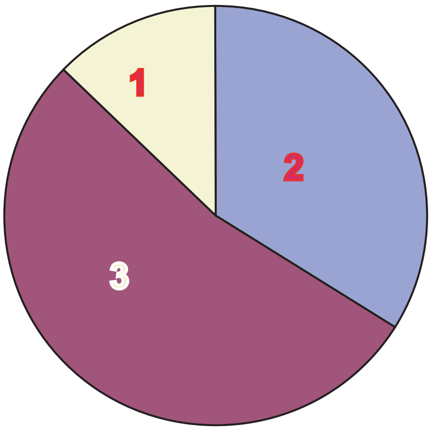


1. Peak at maternal stage (13%)
2. Peak at blastula stage (34%)
3. Peak at gastrula stage (53%)

## Classification of early genes based on functions

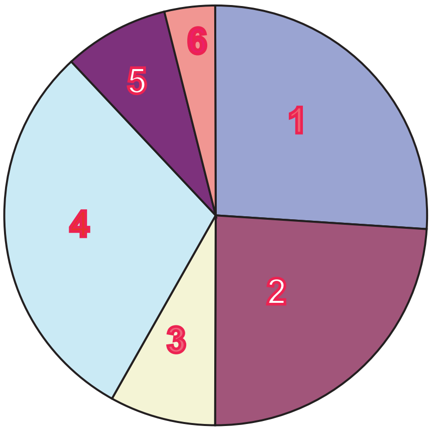


1. ATP binding/protein folding/ biosynthesis (26%).
2. DNA/RNA binding/ developmennt (24%).
3. Cell cycle/regulation (8%)
4. Metabolism/transport (30%)
5. GTP binding activity (8%)
6. Others (4%)

Distribution of early gene function at different developmental stages. Values are in percent.
