# Supplementary material for: Transcriptome Analysis of Zebrafish Embryogenesis Using Microarrays
Source: PLoS Genet. 2005 Aug 26;1(2):e29. doi: 10.1371/journal.pgen.0010029 (PMC1193535; doi:10.1371/journal.pgen.0010029)
Supplement: Table S1 — (11 KB PDF) [file pgen.0010029.st001.pdf]

Table S1. Comparison of expression profile of selected genes in microarray analysis and other methods of transcript detection (Northern/ in situ).

Gene Name	Microarray Results	Supporting Data	
		Northern blot	in situ
Egg envelope protein ZP2	Expressed highly during Oocyte stage only	Transcripts detected only at oocyte stage. (Wang H and Gong Z.2000)	
Sox31	Expressed highly at blastula stage and continues till early gastrula.		Maternally expressed and maximum expression extended to blastula stage(Girard et al. 2001)
Tumor suppressor P53	Expressed from zygote to early segmentation stage after which it declines	Transcript is most abundant in zygotes and early cleavage embryos thereafter declining to barely detectable levels at 48 hpf. (Cheng et al. 1997)	
Cyclin D1	Highly expressed all stages except at oocyte and cleavage stage.	Transcripts are first detected at epiboly and continues till somitogenesis. (Yarden et al. 1995)	
Pax6	Expressed at late gastrula stage and continues till 48 hours		Transcripts are first observed in neural plate(10 hpf) and are detected at later stages in hindbrain (24 hpf). (Amirthalingam et al. 1995)
Beta-thymosin	Transcripts detected from 2 somite stage (10.7 hpf) and the maximum at 48 hpf	Transcripts are first seen at 12 hpf and maximum at hatching (Roth et al. 1999)	
Connexin (cx43.4)	Expressed from 3 hpf with a maximum at 70 % epiboly(7.7 hpf) and continued till hatching	Transcripts are abundant at shield stage (6 hpf) and expressed till hatching. (Essner et al. 1996)	
