# Supplementary material for: Transcriptome Analysis of Zebrafish Embryogenesis Using Microarrays
Source: PLoS Genet. 2005 Aug 26;1(2):e29. doi: 10.1371/journal.pgen.0010029 (PMC1193535; doi:10.1371/journal.pgen.0010029)
Supplement: Table S2 — (9 KB PDF) [file pgen.0010029.st002.pdf]

**Table S2: Characteristics of developmental stages of embryos that were collected for the expression profile analysis in this work.**

| | **Developmental stages** | **Characteristics** |
|---|---|---|
| 1 | Unfertilized egg | Unfertilized eggs, maternal mRNA |
| 2 | High stage (3 hpf) | Early post-MBT embryo, transcripts that could be linked to activation of transcription, early zygotic transcripts, yolk syncytial layer |
| 3 | 30% epiboly (4.5 hpf) | Early post-MBT embryo, early zygotic transcripts |
| 4 | Shield stage (6 hpf) | Determinants of germ layers, axis determinants |
| 5 | 70% epiboly (7.7 hpf) | Determinants of germ layers and embryonic axis |
| 6 | 90% epiboly (9 hpf) | Determinants of determination of primary neurons |
| 7 | 2 somite stage (10.7 hpf) | Determinants of neural determination and differentiation |
| 8 | 5-6 somite stage (12 hpf) | Determinants of neural determination and differentiation<br>Determinants of organogenesis |
| 9 | 12-14 somites stage (15 hpf) | Determinants of neural determination, differentiation, axonal outgrowth, liver, pancreas |
| 10 | Prim 5 stage (24 hpf) | Determinants of secondary neurogenesis, endoderm, heart, limbs, fins |
| 11 | Prim 15 stage (30 hpf) | Determinants of endoderm, foregut, gliogenesis |
| 12 | Late organogenesis (48 hpf) | Determinants of late differentiation, hatching |
