# Supplementary material for: Transcriptome Analysis of Zebrafish Embryogenesis Using Microarrays
Source: PLoS Genet. 2005 Aug 26;1(2):e29. doi: 10.1371/journal.pgen.0010029 (PMC1193535; doi:10.1371/journal.pgen.0010029)
Supplement: Table S3 — (24 KB PDF) [file pgen.0010029.st003.pdf]

**Table S3: Subset of Zebrafish early genes with  human and mouse orthologs**

| Genbank ID(ZF | Zebrafish ID | Entrez Ger | Unigene Name (zebrafish) | Gene symbo | Human  ID | Humann ortholog | Human Gene description |
|---|---|---|---|---|---|---|---|
| AW116838 | Dr.19225 | 336425 | Aldolase a, fructose-bisphosphate | aldoa | Hs.155247 | ALDOA | Fructose-bisphosphate aldola |
| BM005100 | Dr.5438 | 327026 | ADP-ribosylation factor 1 like | arf1l | Hs.119177\|\|Hs | ARF1_HUMAN | ADP-ribosylation factor 1 |
| AW076882 | Dr.6582 | 403025 | Cancer susceptibility candidate 3 | casc3 | Hs.350229 | CASC3 | Cancer susceptibility candidat |
| AI437239 | Dr.6928 | 116994 | Chaperonin containing TCP1, subun | cct6a | Hs.73072\|\|Hs. | CCT6A | T-complex protein 1, zeta sub |
| BE557308 | Dr.134 | 192324 | Chaperonin containing TCP1, subun | cct7 | Hs.368149 | CCT7 | T-complex protein 1, eta subu |
| BG303647 | Dr.26326 | 321602 | Cyclin-dependent kinase 9 (CDC2-re | cdk9 | Hs.150423 | CDK9 | Cell division protein kinase 9 |
| AB040044 | Dr.8161 | 57970 | Coatomer protein complex, subunit z | copz1 | Hs.37482\|\|Hs. | Copz2 | Coatomer zeta-2 subunit |
| BI888253 | Dr.20911 | 30436 | Eyes absent homolog 1 | eya1 | Hs.491997 | EYA4 | Eyes absent homolog 4 |
| AI878758 | Dr.3225 | 317737 | Glutamate dehydrogenase 1a | glud1a | Hs.368538\|\|Hs | GLUD1 | Glutamate dehydrogenase 1, |
| AW128619 | Dr.1388 | 325284 | G1 to S phase transition 1 | gspt1 | Hs.59523\|\|Hs. | GSPT1 | G1 to S phase transition prote |
| AF412832 | Dr.12595 | 140427 | Heat shock factor 2 | hsf2 | Hs.158195 | HSF2 | Heat shock factor protein 2 |
| D38454 | Dr.20916 | 30151 | Insulin gene enhancer protein Islet3 | isl3 | Hs.444677 | ISL2 | Insulin gene enhancer protein |
| AY052752 | Dr.7485 | 170444 | Pbx/knotted 1 homeobox 1.1 | pknox1.1 | Hs.431043 | PKNOX1 | Homeobox protein PKNOX1 |
| AW305657 | Dr.1375 | 336786 | Proteasome (prosome, macropain) 2 | psmc1 | Hs.131528\|\|Hs | PRS4_HUMAN | 26S protease regulatory subu |
| AW116396 | Dr.11425 | 327512 | Phosphoserine aminotransferase 1 | psat1 | Hs.494261 | PSAT1 | Phosphoserine aminotransfer |
| BE558184 | Dr.1119 | 322129 | Retinoblastoma binding protein 4, lik | rbb4l | Hs.16003 | RBBP7 | Histone acetyltransferase type |
| AA495420 | Dr.11588 | 334757 | Sideroflexin 2 | sfxn2 | Hs.44070 | SFXN2 | Sideroflexin 2 |
| AI545320 | Dr.3018 | 323071 | Transcription elongation regulator 1 ( | tcerg1 | Hs.443465 | TCERG1 | Transcription elongation regul |
| AY029577 | Dr.12594 | 140614 | X-box binding protein 1 | xbp1 | Hs.437638 | XBP-1 | X box binding protein-1 |
| AW777876 | Dr.9798 | 368235 | Tyrosyl-tRNA synthetase | yars | Hs.213264 | YARS | Tyrosyl-tRNA synthetase, cyto |
| U85091 | Dr.5112 | 30603 | SRY-box containing gene 11b | sox11b | | | |
| BM023680 | Dr.26724 | 321659 | Zinc finger RNA binding protein | zfr | | | |

| Mouse ID | Mouse ortholo | Mouse gene descri | Gene Ontology |
|---|---|---|---|
| Mm.7729 | Aldoa | Fructose-bisphospha | Not avaiable |
| Mm.5061\|\|M | Arf1 | ADP-ribosylation fac | molecular function\|GTP binding\|IEA\|GO:0005525\|ZFIN\|na//biological process\|small GTPase mediated signal transduc |
| Mm.40120 | Casc3 | Cancer susceptibility | Not avaiable |
| unit | | | molecular function\|ATP binding\|IEA\|GO:0005524\|ZFIN\|na//biological process\|protein folding\|IEA\|GO:0006457\|ZFIN\|na |
| Mm.28990( | Cct7 | T-complex protein 1 | molecular function\|ATP binding\|IEA\|GO:0005524\|ZFIN\|na//biological process\|protein folding\|IEA\|GO:0006457\|ZFIN\|na |
| Mm.27557\| | Cdk9 | cyclin-dependent kir | molecular function\|ATP binding\|IEA\|GO:0005524\|ZFIN\|na//molecular function\|kinase activity\|IEA\|GO:0016301\|ZFIN\|na |
| Mm.22144\| | Copz2 | Coatomer zeta-2 sul | cellular component\|clathrin vesicle coat\|IEA\|GO:0030125\|ZFIN\|na//biological process\|intracellular protein transport\|IEA |
| Mm.25018 | Eya4 | Eyes absent homolo | molecular function\|catalytic activity\|IEA\|GO:0003824\|ZFIN\|na//biological process\|development\|IEA\|GO:0007275\|ZFIN\| |
| Mm.10600\| | Glud1 | Glutamate dehydrog | biological process\|amino acid metabolism\|IEA\|GO:0006520\|ZFIN\|na//molecular function\|oxidoreductase activity\|IEA\|G |
| Mm.20826\| | Gspt2 | G1 to S phase trans | molecular function\|GTP binding\|IEA\|GO:0005525\|ZFIN\|na//biological process\|protein biosynthesis\|IEA\|GO:0006412\|Z |
| Mm.26139 | Hsf2 | heat shock factor 2 | molecular function\|DNA binding\|IEA\|GO:0003677\|ZFIN\|na//cellular component\|nucleus\|IEA\|GO:0005634\|ZFIN\|na//biol |
| Mm.27399 | Isl2 | Insulin gene enhanc | molecular function\|DNA binding\|IEA\|GO:0003677\|ZFIN\|na//biological process\|development\|IEA\|GO:0007275\|ZFIN\|na/ |
| Mm.25929 | Pknox1 | Homeobox protein F | molecular function\|DNA binding\|IEA\|GO:0003677\|ZFIN\|na//biological process\|cell differentiation\|IMP\|GO:0030154\|ZFI |
| Mm.15710 | Psmc1 | 26S protease regula | molecular function\|ATP binding\|IEA\|GO:0005524\|ZFIN\|na//cellular component\|cytoplasm\|IEA\|GO:0005737\|ZFIN\|na//c |
| Mm.28993 | Psat1 | Phosphoserine amir | biological process\|L-serine biosynthesis\|IEA\|GO:0006564\|ZFIN\|na//biological process\|metabolism\|IEA\|GO:0008152\|Z |
| e B subunit 2 | | | Not avaiable |
| Mm.29683 | Sfxn2 | Sideroflexin 2 | biological process\|cation transport\|IEA\|GO:0006812\|ZFIN\|na//molecular function\|cation transporter activity\|IEA\|GO:000 |
| Mm.27051 | Tcerg1 | transcription elongat | Not avaiable |
| Mm.22718 | Xbp1 | X-box binding protei | molecular function\|DNA binding\|IEA\|GO:0003677\|ZFIN\|na//cellular component\|nucleus\|IEA\|GO:0005634\|ZFIN\|na |
| Mm.14548 | Yars | tyrosyl-tRNA synthe | molecular function\|ATP binding\|IEA\|GO:0005524\|ZFIN\|na//molecular function\|nucleic acid binding\|IEA\|GO:0003676\|Z |
| Mm.39088\| | Sox19 | Transcription factor | molecular function\|DNA binding\|IEA\|GO:0003677\|ZFIN\|na//biological process\|regulation of transcription, DNA-depend |
| Mm.27349 | Zfr | zinc finger RNA binc | molecular function\|nucleic acid binding\|IEA\|GO:0003676\|ZFIN\|na//cellular component\|nucleus\|IEA\|GO:0005634\|ZFIN\| |

| | | | | | | | | | | | | |
|---|---|---|---|---|---|---|---|---|---|---|---|---|
| | | | | | | | | | | | | |
| | | | | | | | | | | | | |
| | | | | | | | | | | | | |
| tion\|IEA\|GO:0007264\|ZFIN\|na | | | | | | | | | | | | |
| | | | | | | | | | | | | |
| //molecular function\|unfolded protein binding\|IEA\|GO:0051082\|ZFIN\|na | | | | | | | | | | | | |
| //molecular function\|unfolded protein binding\|IEA\|GO:0051082\|ZFIN\|na | | | | | | | | | | | | |
| a//biological process\|protein amino acid phosphorylation\|IEA\|GO:0006468\|ZFIN\|na//molecular function\|protein kinase activity\|IEA\|GO:0004672\|ZFIN\| | | | | | | | | | | | | |
| A\|GO:0006886\|ZFIN\|na//molecular function\|structural molecule activity\|IEA\|GO:0005198\|ZFIN\|na//biological process\|transport\|IEA\|GO:0006810 | | | | | | | | | | | | |
| na//biological process\|metabolism\|IEA\|GO:0008152\|ZFIN\|na | | | | | | | | | | | | |
| O:0016491\|ZFIN\|na | | | | | | | | | | | | |
| FIN\|na | | | | | | | | | | | | |
| ogical process\|protein folding\|IEA\|GO:0006457\|ZFIN\|na//biological process\|regulation of transcription, DNA-dependent\|IEA\|GO:0006355\|ZFIN\|na//biol | | | | | | | | | | | | |
| /cellular component\|nucleus\|IEA\|GO:0005634\|ZFIN\|na//biological process\|regulation of transcription, DNA-dependent\|IEA\|GO:0006355\|ZFIN\|na//molecula | | | | | | | | | | | | |
| N\|na//cellular component\|cytoplasm\|IDA\|GO:0005737\|ZFIN\|na//biological process\|hindbrain development\|IMP\|GO:0030902\|ZFIN\|na//cellular component | | | | | | | | | | | | |
| ellular component\|cytosol\|IEA\|GO:0005829\|ZFIN\|na//molecular function\|hydrolase activity\|IEA\|GO:0016787\|ZFIN\|na//molecular function\|nucleotide bind | | | | | | | | | | | | |
| FIN\|na//molecular function\|transaminase activity\|IEA\|GO:0008483\|ZFIN\|na//molecular function\|transferase activity\|IEA\|GO:0016740\|ZFIN\|na | | | | | | | | | | | | |
| | | | | | | | | | | | | |
| 08324\|ZFIN\|na//cellular component\|membrane\|IEA\|GO:0016020\|ZFIN\|na | | | | | | | | | | | | |
| | | | | | | | | | | | | |
| | | | | | | | | | | | | |
| FIN\|na//biological process\|tRNA aminoacylation for protein translation\|IEA\|GO:0006418\|ZFIN\|na//molecular function\|tRNA binding\|IEA\|GO:0000049\|ZF | | | | | | | | | | | | |
| ent\|IEA\|GO:0006355\|ZFIN\|na | | | | | | | | | | | | |
| na//molecular function\|zinc ion binding\|IEA\|GO:0008270\|ZFIN\|na | | | | | | | | | | | | |
