# Supplementary material for: Transcriptome Analysis of Zebrafish Embryogenesis Using Microarrays
Source: PLoS Genet. 2005 Aug 26;1(2):e29. doi: 10.1371/journal.pgen.0010029 (PMC1193535; doi:10.1371/journal.pgen.0010029)
Supplement: Protocol S1 — (71 KB PDF) [file pgen.0010029.sd028.pdf]

Supplementary Protocol: 1.

Onset and Offset Finding Algorithm

The objective of the algorithm is to detect the onset time (beginning) and offset time (ending) of accumulation of the mRNA for a given gene using its time-course expression profile. Peak of the expression is defined as the time at which the mRNA abundance attains its highest level. The onset (offset) time of expression is defined as the earliest (latest) time of the peak in the profile. All points spanned from onset to offset are called peak points. This implies that the onset (offset) of the peak is same as the earliest (farthest) "peak point" in the profile.

All peak points in the given profile are detected using the following procedure:

1. The profile F_i of gene G_i is normalized such that its dynamic range i.e. the difference between the highest expression value and the lowest expression value in F_i.
2. The profile F_i is then smoothened by using Gaussian function as follows:

$$F_{it} = \sum_x F_{ix} * e^{-(t-x)^2/(2*\sigma^2)}$$

Where F_{it} is log of the relative expression of gene G_i at time point t and _ is the scaling factor and was set to 0.75. This step has been adopted to reduce the influence of random fluctuations on onset and offset time determination.

3. Let p be the time at which the above processed profile attains maximum value F_{ip}.
4. For each time point $x < p$, whose expression value is F_{ix}, find the number of time points T _ $\{t|x>t<p\}$ for which the perpendicular distance of (T,F_{iT}) from the line joining the points (x,F_{ix}) and (p,F_{ip}) (whose formula is given below) from below is less than certain threshold(Thr_d). If this number is below certain threshold (Thr_n)then consider this point as *peak point*. Thr_d and Thr_n were set to 0.2 and 1 respectively

$$m = (F_{ix} - F_{ip})/(x-p)$$
$$c = F_{ip} - m*p$$
$$D(T,x,p) = (|F_{ix} - F_{iT} - m*(x - T)|)/\sqrt{(1+m*m)}$$

Where m is the slope of the line joining the points (x,F_{ix}) and (p,F_{ip}) and c is the intercept of this line on expression axis. $D(T,x,p)$ is the perpendicular distance of (T,F_{iT}) from the line joining the points (x,F_{ix}) and (p,F_{ip}).

5. After having completed the above step, noise and deviations are corrected for by considering the non-peak points as peak points if their both left and right nearest points are peak points according to the above step i.e. for any time t if $t-1$ and $t+1$ are peak points according to step 2, then t is also considered to be peak point.

Then, starting from the time of peak (p), find the earliest time T_{on} such that (T_{on} -1) is not a peak point but (T_{on} $+1$) is a peak point. Then T_{on} is the onset of the expression. Repeat the above procedure on the right hand side of the peak and the offset is the nearest time point T_{off} such that (T_{off} $+1$)is not a peak point but (T_{off} -1)is a peak point.
